# Yttrium Doping of Perovskite Oxide La_2_Ti_2_O_7_ Nanosheets for Enhanced Proton Conduction and Gas Sensing Under HighHumidity Levels

**DOI:** 10.3390/s25030901

**Published:** 2025-02-02

**Authors:** Jian Wang, Caicai Sun, Jusheng Bao, Zhiwei Yang, Jian Zhang, Xiao Huang

**Affiliations:** Institute of Advanced Materials (IAM), School of Flexible Electronics (SoFE), Nanjing Tech University (NanjingTech), 30 South Puzhu Road, Nanjing 211816, China

**Keywords:** perovskite oxide, proton transport, yttrium doping, gas sensing, high humidity level

## Abstract

Water molecules from the environment or human breath are one of the main factors affecting the accuracy, efficiency, and long-term stability of electronic gas sensors. In this contribution, yttrium (Y)-doped La_2_Ti_2_O_7_ (LTO) nanosheets were synthesized by a hydrothermal reaction, demonstrating improved proton conductivity compared to their non-doped counterparts. The response of Y-doped LTO with the optimal doping concentration to 100 ppm NO_2_ at 43% relative humidity (RH) was −21%, which is four times higher than that of bare La_2_Ti_2_O_7_. As the humidity level increased to 75%, the response of Y-doped LTO further increased to −64%. Unlike the gas doping effect observed in previous studies of semiconducting metal oxides, the sensing mechanism of Y-doped LTO nanosheets is based on the enhanced dissociation of H_2_O in the presence of target NO_2_ molecules, leading to the generation of more protons for ion conduction. This also resulted in a greater resistance drop and thus a larger sensing response at elevated humidity levels. Our work demonstrates that proton-conductive oxide materials are promising gas-sensing materials under humid conditions.

## Simple Summary

In this work, Y-doped LTO nanosheets were synthesized for NO_2_ gas sensing under different humidity conditions. These materials showed increased responses at higher humidity levels due to enhanced proton conduction and NO_2_-assisted proton generation. Notably, Y-doped LTO nanosheets exhibited higher responses than pure LTO, as Y-doping created more oxygen vacancies, improving water adsorption and proton dissociation.

## 1. Introduction

Nitrogen dioxide (NO_2_) is a type of atmospheric pollutant released from fuel combustion and industry exhausts [1,2] which can cause environmental issues such as dense fog and acid rain [3,4]. Long-term exposure to parts per billion (ppb) levels and above of NO_2_ can cause diseases such as pulmonary edema, tracheitis, and pneumothorax [5,6]. Exhaled NO_2_ in one’s breath is also a gas-phase biomarker related to diseases such as nasal polyposis and chronic obstructive pulmonary disease [7,8]. Therefore, there is a great need to develop NO_2_ sensing materials and devices [6,9]. Under ambient conditions, the detection of NO_2_ is often interfered by H_2_O molecules in humid air or from human breath [10,11,12]. Many gas-sensing materials show decreased responses at high humidity levels due to competitive adsorption between NO_2_ and H_2_O molecules [13,14]. Various methods have thus been developed to address this issue, including increasing the surface roughness of materials to decrease surface energies and thus reduce hydrophilicity [15,16] and coating the sensing materials with hydrophobic layers [17,18] or metal nanoparticles [19]. For instance, Pd/TiO_2_ NTs coated with a hydrophobic polydimethylsiloxane (PDMS) layer was prepared by a thermal evaporating method, which can enhance the humidity resistance and improve the long-term stability of Pd/TiO_2_ NT-based sensors [20]. In another example, combining electrospinning with a thermal sulfidation approach, a hydrophobic all-inorganic self-supporting SnO_2_-SnS_2_-SiO_2_/SiO_2_ sensor was fabricated and enhanced moisture resistance was achieved due to the formation of Sn-S bonds during the sulfidation of SnO_2_ nanofibers [21]. However, the complicated preparation process and poor conductivity of sensing materials in low-humidity environments still hinder the application of these materials. Therefore, other material systems capable of delivering a high gas-sensing performance at high humidity levels require continuous exploration.

La_2_Ti_2_O_7_ (LTO) is an oxide with a layered perovskite structure. It has shown good catalytic activity, stability, and proton transport properties and has been widely applied in electronics, catalysis, and energy storage [22,23,24]. This type of oxide has a general formula of A_n_B_n_O_3n+2_, characterized by corner-shared BO_6_ octahedra and A cations filling in the dodecahedral holes formed by the BO_6_ octahedra [25]. Using elements with a lower valence, such as Y^3+^, Yb^3+^, and Bi^3+^, to replace Ti^4+^ can make LTO lose electrical neutrality and generate oxygen vacancies [26,27,28,29], which can further improve its surface activity and proton transport ability [30].

In this study, Y-doped LTO nanosheets (with doping concentrations of 0.3%, 0.7%, and 1.2% in weight) were synthesized using a hydrothermal method. Among them, 0.7% Y-doped LTO nanosheets exhibited superior proton conductivity over a wide humidity range and demonstrated the best gas-sensing performance. The sensors also exhibited enhanced performance with increasing humidity levels, which is different from conventional gas sensors. The Y-doping-induced formation of oxygen vacancies likely enhances water adsorption, which then reacts with the exposed NO_2_ to generate more protons, leading to an improved sensing response of the Y-doped LTO nanosheets. Additionally, these doped nanosheets displayed remarkable selectivity towards NO_2_, underscoring their potential in practical gas-sensing applications such as breath analysis.

## 2. Materials and Methods

Materials: La(NO_3_)_3_·5H_2_O and Y(NO_3_)_3_·6H_2_O were purchased from Shanghai Alfa Aesar Chemical Co., Ltd., Shanghai, China. Ti(SO_4_)_2_ was purchased from Shanghai Aladdin Biochemical Technology Co., Ltd., Shanghai, China. Absolute ethanol (99.7%) was purchased from Wuxi Yasheng Chemical Co., Ltd., Wuxi, China. NaOH and HCl were purchased from Sinopharm Chemical Reagent Co., Ltd., Shanghai, China. All gasses were purchased from Nanjing Special Gas Co., Ltd., Nanjing, China. Interdigitated Au electrodes (0.1 mm spacing over a 1 × 2 cm^2^ area, substrate: Al_2_O_3_) were purchased from Huizhou Xinwenxiong Commerce & Trade Co., Ltd., Huizhou, China. 

Preparation of pure La_2_Ti_2_O_7_: Typically, 0.4 mmol La(NO_3_)_3_·5H_2_O and 0.4 mmol Ti(SO_4_)_2_ were dispersed in 2 mL deionized water and ultra-sonicated for 5 min to make the solution uniform. Then, 2 mL NaOH (2 mol/L) was slowly added into the mixture. After stirring for 4 h, the whole mixture was added to a 5 mL Teflon-lined stainless steel autoclave and then heated at 240 °C for 10 h. After being naturally cooled to room temperature, the solution was centrifuged and washed with HCl (2 mol/L), deionized water (two times), and ethanol, respectively, and finally re-dispersed in 10 mL ethanol for further use. By following the same procedure, except for partially replacing Ti(SO_4_)_2_ with Y(NO_3_)_3_·6H_2_O, La_2_Ti_2_O_7_ doped with different concentrations of Y was prepared.

Characterizations: Transmission electron microscope (TEM, JEOL, 2100Plus, Tokyo, Japan) and high resolution transmission electron microscope (HRTEM, JEOL, 2100F, Tokyo, Japan) were used to investigate the morphologies and microstructures of the samples. X-ray diffraction (XRD, Rigaku, XtaLAB mini II, Tokyo, Japan) was performed with Cu Ka radiation (λ = 1.54 Å). X-ray photoelectron spectroscopy (XPS, Versa Probe, PHI 5000, Chigasaki, Japan) was utilized to analyze the bonding environment of each element and all the binding energies were calibrated to the C 1s band at 284.6 eV. The sample powder was pressed into a pellet and then fixed between two copper electrodes to measure its impedance spectra at an electrochemical station (Autolab 86,567, Englewood, CO, USA). Inductively coupled plasma-atomic emission spectrometry (ICP-AES, Perkin Elmer, Avio200, Waltham, MA, USA) was applied to measure the Y-doped contents of YLTO nanosheets. Dynamic vapor sorption (DVS, SMS-DVS, Intrisic, London, UK) was used to test the water adsorption of the nanosheets.

Gas sensing measurement: Fabrication of the gas sensor typically began with 50 μL ethanoic solution of the sensing material (30 mg/mL) being dropped onto a Au interdigitated electrode (with 0.1 mm spacing over a 2 × 1 cm^2^ area, Changchun Mega Borui Technology Co., Ltd., Changchun, China). Then, the whole electrode was dried in air at room temperature (RT). Gas sensing tests were conducted at RT in a sealed chamber. Constant currents were applied to the sensor electrodes and the variation in resistance was recorded using a data acquisition system (Agilent 34972A, Santa Clara, CA, USA). A gas diluter (Nanjing Dibaisi Technology Co., Ltd., Nanjing, China) was employed to aerate different concentration target gasses within the test chamber.

Measurement of proton conductivities: The as-prepared samples were cold-pressed into pellets with a diameter of 10 mm under a pressure of 6 MPa. The thickness of the pellets was measured using a Vernier caliper. Then, the pellet was fixed between two copper electrodes to measure its impedance spectra in a home-made chamber, where temperature and humidity were controlled by an oven and saturated salt solutions, respectively. The ionic conductivity (σ) was calculated using σ = L/(R × A), where L is the thickness of the measured pellet, A is the contact area between the sample and the electrode, and R is the resistance obtained by fitting the semicircle.

## 3. Results

**Figure 1:**
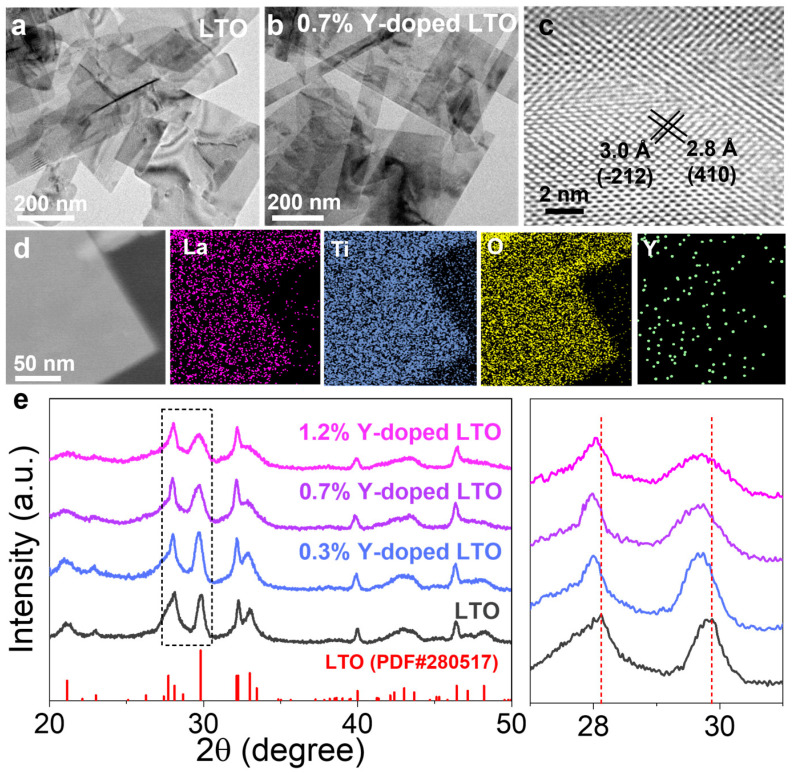
Transmission electron microscope images of (**a**) LTO and (**b**) 0.7% Y-doped LTO. (**c**) High resolution transmission electron microscope image and (**d**) energy-dispersive X-ray mapping of 0.7% Y-doped LTO nanosheets. (**e**) X-ray diffraction patterns of Y-doped LTO nanosheets compared to non-doped LTO.

La_2_Ti_2_O_7_ (LTO) nanosheets were prepared by reacting La(NO_3_)_3_·5H_2_O and Ti(SO_4_)_2_ hydrothermally [31], and Y-doped LTO (YLTO) nanosheets were synthesized by partially replacing Ti(SO_4_)_2_ with Y(NO_3_)_3_·6H_2_O. The Y doping concentrations were 0.3%, 0.7%, and 1.2% in weight, respectively, as measured by ICP-AES. Both LTO and YLTO were 200~800 nm in lateral size, as shown in the TEM images in Figure 1a,b and Figure S1. Therefore, Y doping did not alter the sheet-like morphology of the product. As shown in Figure 1c, lattice spacings of 2.8 and 3.0 Å can be observed in a typical 0.7% Y-doped LTO, assignable to the (410) and (−212) planes of LTO. As shown Figure S2, the selected area electron diffraction (SAED) pattern revealed a pattern along the [02-1] zone axis and exhibited diffraction plots belonging to the (−112) and (212) crystal faces of LTO. Energy-dispersive X-ray (EDX) mapping of a typical nanosheet (Figure 1d) revealed a uniform distribution of La, Ti, O, and Y elements, indicating successful Y doping. The XRD patterns of the Y-doped LTO nanosheets with different doping concentrations (Figure 1e) were compared with the non-doped one (PDF#280517) and showed a slight shift to lower angles. This might be because Ti atoms were partially replaced by Y atoms, which have a larger ionic radius, resulting in lattice expansion and thus a shift in the XRD peaks to smaller angles. 

**Figure 2:**
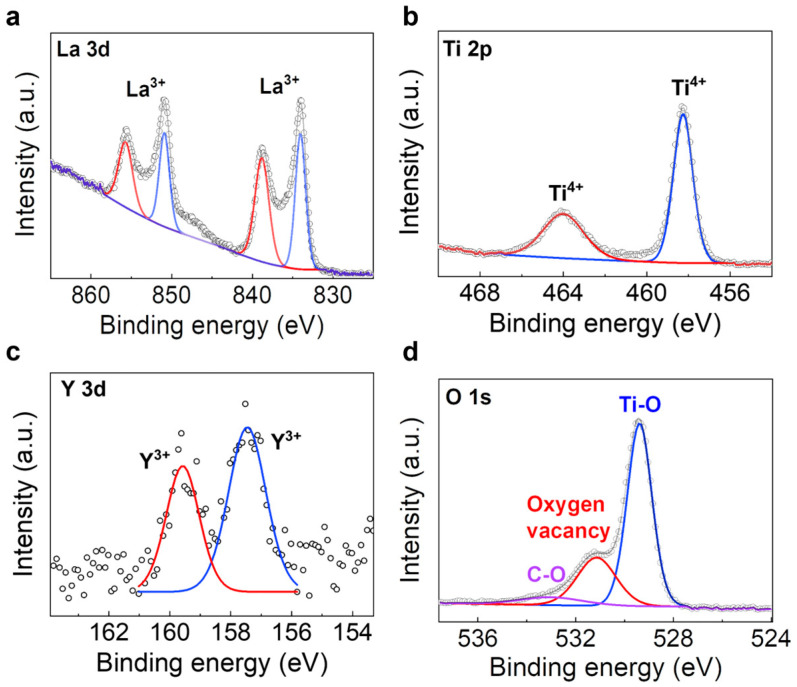
High-resolution (**a**) La 3d, (**b**) Ti 2p, (**c**) Y 3d, and (**d**) O 1s X-ray photoelectron spectroscopy spectra of 0.7% Y-doped LTO nanosheets.

An XPS analysis was performed to investigate the valence states of the elements in the LTO and YLTO nanosheets (Figure 2 and Figures S3–S6). For 0.7% doped LTO, peaks for La^3+^ and Ti^4+^ species were clearly observed in the La 3d and Ti 2p spectra, respectively (Figure 2a,b). The high-resolution Y 3d spectrum shows a doublet at 157.4 and 159.5 eV, corresponding to the 3d_3/2_ and 3d_5/2_ bands of Y^3+^, respectively (Figure 2c), further proving the successful doping of Y. In the high-resolution O 1s spectrum, peaks at 529.7, 531.5 eV, and 532.6 eV were observed, corresponding to Ti-O, oxygen vacancies, and C-O, respectively [32]. By calculating the areas under the fitted peaks, we also found that the concentration of oxygen vacancies increased with increasing Y-doping concentrations, from 0 to 0.7% (Figure S4). This is likely because replacing Ti^4+^ with Y^3+^ disrupted the charge neutrality, and thus a higher Y doping level favored the loss of more oxygen atoms [33]. However, further increasing the doping concentration from 0.7% to 1.2% did not create more oxygen vacancies, but generated more defects, as evidenced by the increased full-width-at-half-maximum (FWHM) of the XRD peaks (Figure 1e) [34,35].

**Figure 3:**
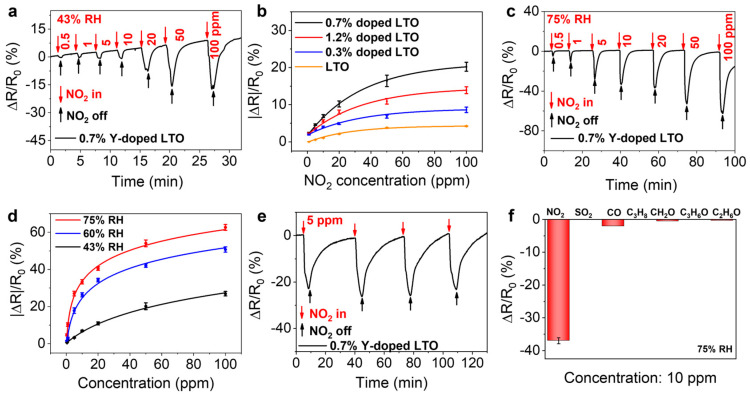
(**a**) Response–recovery curves of 0.7% Y-doped LTO nanosheets to NO_2_ of increasing concentrations under 43% RH. (**b**) Comparison of responses of Y-doped LTO (doping levels of 0.3%, 0.7%, and 1.2%) toward NO_2_ of different concentrations at 43% RH. (**c**) Response–recovery curves of 0.7% Y-doped LTO nanosheets to NO_2_ of increasing concentrations under 75% RH. (**d**) Comparison of responses of 0.7% Y-doped LTO nanosheets toward NO_2_ of different concentrations under 43% RH, 60% RH, and 75% RH. (**e**) Cyclic stability test of 0.7% Y-doped LTO nanosheets in response to 5 ppm NO_2_ gas under 75% RH. (**f**) Sensing responses of 0.7% Y-doped LTO nanosheets towards NO_2_ and other gasses at a concentration of 10 ppm under 75% RH.

The sensing performance of the LTO and YLTO nanosheets towards NO_2_ was first conducted under 43% RH and RT. Their sensing response is defined by ΔR/R_0_, where ΔR= R_g_ − R_0_ and R_0_ and R_g_ are the resistances of the sensor before and after exposure to NO_2_, respectively. As shown in Figure 3a and Figure S7, both LTO and YLTO nanosheets showed decreased resistance upon NO_2_ exposure. Among the YLTO with different doping levels, 0.7% Y-doped LTO showed the highest sensing responses (Figure 3b). Next, the sensing performance of YLTO nanosheets towards NO_2_ under different RH levels at RT was further investigated. All of the Y-doped LTO nanosheets exhibited increased sensing responses with increasing RH levels and outperformed non-doped LTO nanosheets (Figure 3c,d, Figures S7 and S8). In addition, the 0.7% Y-doped LTO nanosheets demonstrated a fast response–recovery rate, with a response time of 30 s and a recovery time of 204 s when detecting 10 ppm NO_2_ at 75% RH, as shown in Figure S9. The 0.7% Y-doped LTO nanosheets had an outstanding performance under high-humidity conditions compared to previously reported single-component metal oxide sensors (Table S1). The sensing response of the 0.7% Y-doped LTO nanosheets toward 5 ppm NO_2_ under 75% RH was repeatedly tested (Figure 3e), showing their good stability. The sensing response of the 0.7% Y-doped LTO nanosheet remained nearly unchanged even after one week, further demonstrating its excellent durability. (Figure S10). In addition, its sensing response towards NO_2_ was found to be much higher than that towards other gasses, including SO_2_, CO, C_3_H_8_, CH_2_O, C_3_H_6_O, and C_2_H_6_O (Figure 3f), suggesting outstanding selectivity. 

## 4. Discussion

**Figure 4:**
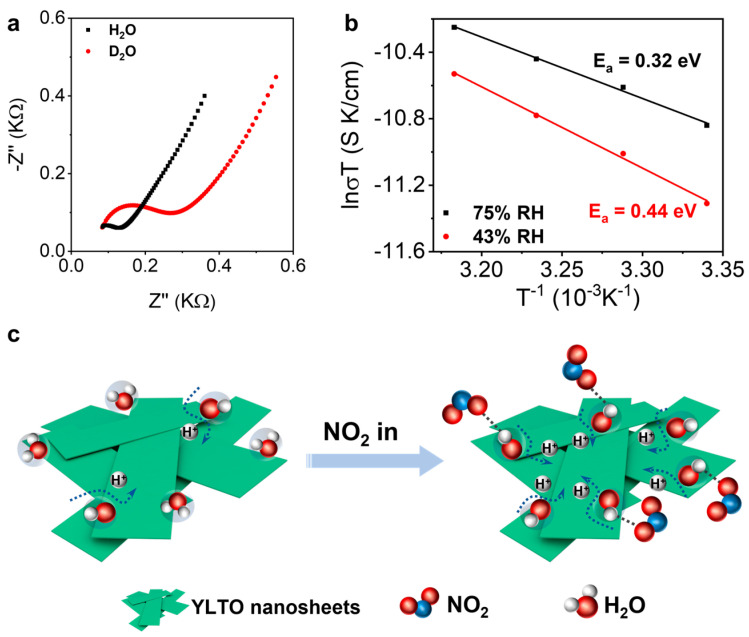
(**a**) Nyquist plots of 0.7% Y-doped LTO in D_2_O and H_2_O atmospheres. (**b**) Estimation of the proton conduction activation energy of 0.7% Y-doped LTO under 43% and 75% relative humidity. (**c**) Schematic illustration of the NO_2_-sensing mechanism of YLTO.

Note that LTO and Y-doped LTO showed a poor/no sensing response under dry or low-humidity (23%) conditions (Figure S11); LTO and YLTO are not typical *p*-type semiconductors. This suggests that the sensing response observed in Figure 3 was not likely due to the *p*-doping effect of NO_2_. Therefore, we suspected that the increased gas sensing performance of LTO and YLTO under higher humidity conditions was related to their ionic conductive nature. We thus measured the impedance spectra of LTO and YLTO nanosheets in H_2_O and D_2_O vapor environments to determine their ionic carriers (Figure 4a and Figure S12). The results showed that their ionic conductivities were at least three times higher in an H_2_O environment than in D_2_O, suggesting that the main charge carriers were protons rather than hydroxyl ions [36]. We also measured the activation energy (E_a_) required for proton conduction in the 0.7% Y-doped nanosheets and found that E_a_ decreased from above 0.4 eV (0.44 eV) to below 0.4 eV (0.32 eV) as the environment humidity increased from 43% to 75% (Figure 4b and Figure S13), suggesting a transition from the vehicle mechanism to a more effective hopping mechanism for proton conduction [37]. This further agrees with the enhanced sensing performance seen under higher humidity levels.

Based on the above observations, we propose the following sensing mechanism: NO_2_ gas molecules can interact with H_2_O molecules adsorbed on the surface of YLTO nanosheets and promote the dissociation of H_2_O molecules to generate protons [38]. The protons thus increase the ionic conduction of the YLTO nanosheets, resulting in a resistance drop for the sensor (Figure 4c).

The reason why 0.7% Y-doped LTO showed the best gas-sensing performance can be attributed to its superior water adsorption and proton conduction ability. These abilities mainly come from its high concentration of surface oxygen vacancies, based on the above XPS analysis (Figure 2 and Figure S4). These oxygen vacancies can assist in water adsorption (Figure S14) and promote the dissociation of water molecules to yield protons [30].

**Figure 5:**
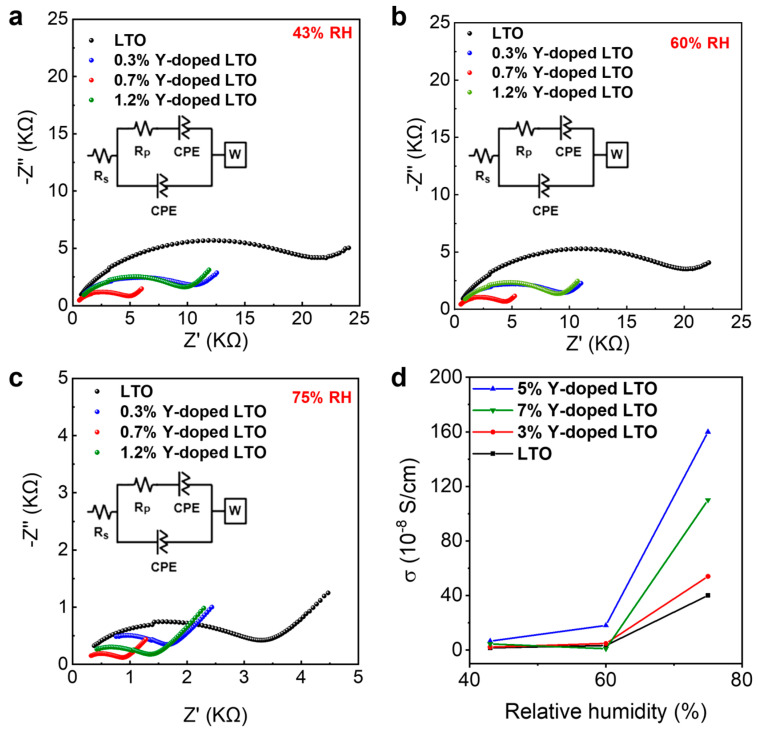
Nyquist plots of LTO and YLTO nanosheets with different Y doping concentrations under (**a**) 43% relative humidity, (**b**) 60% relative humidity, and (**c**) 75% relative humidity. (**d**) Comparison of conductivity of various Y-doped LTO nanosheets under 43% relative humidity, 60% relative humidity, and 75% relative humidity conditions.

To further prove our conjecture, the proton conductivities of YLTO nanosheets with different doping concentrations were obtained, based on their Nyquist plots, under 43% RH, 60% RH, and 75% RH (Figure 5). Their proton conductivities were calculated and are summarized in Figure 5d. All the nanosheets exhibited increasing proton conductivities with increasing RH levels. The 0.7% Y-doped LTO nanosheets showed the highest proton conductivity compared with the others, which is in line with their superior sensing performance. 

Note that despite the similar oxygen vacancy content in 0.7% Y-doped and 1.2% Y-doped LTO, the proton conductivity of the 1.2% Y-doped LTO was lower than that of the 0.7% Y-doped one. This can be attributed to the interplay between the effects of oxygen vacancies and crystallinity on the proton conductivity of these materials. At relatively low Y contents (≤0.7%), the number of oxygen vacancies increased with the Y content (Figure S4), resulting in improved proton conductivity. At higher Y contents, e.g., 1.2%, an increased defect level or reduced crystallinity was observed, as mentioned above (Figure 1e), which might block the proton transport pathways, resulting in a poorer sensing performance.

## 5. Conclusions

In summary, Y-doped LTO nanosheets were synthesized and utilized for NO_2_ gas sensing under different humidity conditions. These sensing materials all showed an increased response under higher humidity levels due to increased proton conduction and NO_2_-assisted proton generation. In particular, Y-doped LTO nanosheets showed higher responses compared to those based on pure LTO. This is because the Y doping induced the formation of oxygen vacancies, which helped improve the adsorption of water and its dissociation into protons. Our work demonstrates that proton-conductive oxide materials are promising gas-sensing materials for practical use under high humidity levels.

## Data Availability

The data presented in this study are available on request from the corresponding author.

## Supplementary Materials

The following supporting information can be downloaded at: https://www.mdpi.com/article/10.3390/s25030901/s1, Figure S1. TEM images of (a) 0.3%, (b) 0.7% and (c) 1.2% Y-doped LTO nanosheets; Figure S2. SAED pattern of 0.7% Y doped LTO nanosheet; Figure S3. High-resolution Y 3d spectra of (a) 0.3% and (b) 1.2% Y-doped LTO nanosheets; Figure S4. High-resolution O 1s spectra of (a) pure LTO, (b) 0.3% and (c) 1.2% Y-doped LTO nanosheets. (d) Oxygen vacancy ratio table of YLTO nanosheets; Figure S5. High-resolution La 3d spectra of (a) pure LTO, (b) 0.3% and (c) 1.2% Y-doped LTO nanosheets; Figure S6. High-resolution Ti 2p spectra of (a) pure LTO, (b) 0.3% and (c) 1.2% Y-doped LTO nanosheets; Figure S7. Sensing responses of LTO nanosheets to NO_2_ under (a) 43% RH, (b) 60% RH and (c) 75% RH; Figure S8. Sensing responses of 0.7% Y-doped LTO nanosheets to NO_2_ under 60% RH; Figure S9. Dynamic response-recovery curves of 0.7% Y doped LTO to 10 ppm NO_2_ at 75% RH; Figure S10. Dynamic response-recovery curves of 0.7% Y doped LTO after one week standing; Figure S11. Sensing responses of LTO and 0.7% Y-doped LTO nanosheets to 100 ppm NO_2_ under 23 % RH; Figure S12. Nyquist plot of LTO nanosheet in H_2_O and D_2_O environments under 100% RH; Figure S13. Nyquist plots of 0.7% Y-doped LTO nanosheets under (a) 43% RH and (b) 75% RH; Figure S14. Water adsorption curves of LTO and 0.7% Y-doped LTO nanosheets; Table S1. Comparison of the sensing performance of the recently reported NO_2_ sensors based single-component metal oxides. References [38,39,40,41,42,43,44,45] are cited in Supplementary Materials.
