# Supplementary material for: Yttrium Doping of Perovskite Oxide La2Ti2O7 Nanosheets for Enhanced Proton Conduction and Gas Sensing Under HighHumidity Levels
Source: Sensors (Basel). 2025 Feb 2;25(3):901. doi: 10.3390/s25030901 (PMC11819912; doi:10.3390/s25030901)
Supplement: Supplementary file 1 [file sensors-25-00901-s001.zip › references.pdf]

Sensors & Actuators: B. Chemical 337 (2021) 129783

Contents lists available at ScienceDirect

Sensors and Actuators: B. Chemical

journal homepage: www.elsevier.com/locate/snb

A room-temperature NO_2 gas sensor based on CuO nanoflakes modified with rGO nanosheets

Haineng Bai [a], Hui Guo [a], Jin Wang [b], Yan Dong [a], Bin Liu [a], Zili Xie [a], Fuqiang Guo [c,*], Dunjun Chen [a,*], Rong Zhang [a], Youdou Zheng [a]

[a] Key Laboratory of Advanced Photonic and Electronic Materials, School of Electronic Science and Engineering, Nanjing University, Nanjing, 210023, China
[b] College of Electronic and Optical Engineering & College of Microelectronics, Nanjing University of Posts and Telecommunications, Nanjing, 210023, China
[c] Department of Physics, Changji University, Xinjiang, Changji, 831100, China

ARTICLE INFO

Keywords:
CuO/rGO
Hydrothermal
Nanoflakes
Nanosheets
Gas-sensing mechanism

ABSTRACT

Conventional gas-sensing materials for detecting trace NO_2 gas are suffering from limitations such as low selectivity, high operation temperature and an agglomerated tendency during synthesis. In this work, uniform CuO nanoflakes modified with rGO nanosheets (CuO/rGO) were synthesized via an ultra-low-cost hydrothermal process with a thermal treatment. The gas sensor based on the as-synthesized CuO/rGO was fabricated by spin-coated method and applied to detect ppb-level NO_2 gas at room temperature (23℃). Remarkably, the CuO/rGO-based sensor maintained an ultrahigh response behavior of approximately 400.8 % towards 5 ppm NO_2 gas with ultrafast response time of 6.8 s at 23℃, and the limit of detection was down to 50 ppb (S = 20.6 %). In addition, the gas sensor had outstanding consistency, reliable repeatability, and long-term stability (30 days). The improved gas-sensing response was attributed to the synergetic effects of CuO and rGO. Specifically, the porous flake-like nanostructures of CuO and high surface areas of CuO/rGO may provide additional active adsorption sites for NO_2 gas and accelerate the redox reaction on the surface of CuO/rGO, leading to the ultrahigh response. On the other hand, the rGO nanosheets with excellent transport capability may serve as highly conductive channels to accelerate carrier transfer, which dramatically reduced the response and recovery time. Furthermore, abundant surface defects of CuO/rGO were also conducive to surface reactions in the gas-sensing process. Undoubtedly, the scientific study of the present work will promote potential applications of ppb-level NO_2 detection at room temperature.

1. Introduction

Nitrogen oxide (NO_2), as one of the most hazardous and toxic gases, is a great threat to both environmental security and human health [1,2]. Notably, low concentrations of NO_2 (~1 ppm) can damage our lungs and cause severe respiratory diseases with symptoms similar to those of asthma [3]. In particular, NO_2 is one of the main factors that can exasperate chronic obstructive pulmonary disease (COPD). Thus, the development of long-term and highly efficient NO_2 gas sensors for real-time monitoring is increasingly important and urgent. Semiconductor-based gas sensors, particularly gas-sensing nanomaterials with high performance, such as WO_3 [4,5], In_2O_3 [6,7], SnO_2 [8,9], Fe_2O_3 [10], ZnO [11], and CeO_2 [12,13], have been widely explored. However, the gas sensors based on metal-oxide nanomaterials lack sufficient selectivity and need a high operation temperature, resulting in device complexity and high power consumption [14,15]. Therefore, there is a growing demand to develop an excellent gas-sensing nanomaterials with room-temperature (RT) operation, high sensitivity and selectivity, fast response, and high limit of detection (LOD).

Copper (II) oxide (CuO, 1.5 eV) is one of the promising and outstanding p-type semiconductors, and it is non-poisonous, environmentally friendly and easy-to-get, which has been extensively studied in various fields, especially as an effective and affordable sensing material for gas sensors owing to two types of sensing behaviors: adsorption-induced surface depletion and chemical conversion by changing the electrical potential [16–18]. Volanti et al. studied the influences of the morphology of CuO on its gas-sensing performances, and observed good long-term stability in air with a higher hydrogen sensor signal due to the urchin-like morphology [19]. Ramgir et al. proposed CuO thin films for

* Corresponding authors.
E-mail addresses: cjxyedu@163.com (F. Guo), djchen@nju.edu.cn (D. Chen).

https://doi.org/10.1016/j.snb.2021.129783
Received 23 November 2020; Received in revised form 22 January 2021; Accepted 9 March 2021
Available online 15 March 2021
0925-4005/© 2021 Elsevier B.V. All rights reserved.

sensing sub-ppm H_2S at room temperature, and the sensor was found to be highly reversible with quick response and recovery time of 60 s and 90 s [20]. Nevertheless, pure CuO materials have difficulty meeting the requirements for detecting gases with state-of-the-art performance. Therefore, the development of appropriate materials that can be applied to decorate CuO is urgently needed to further enhance the gas-sensing property. As is known, functionalized graphene-based composites offer innumerable opportunities to further improve the sensing performance of semiconductors [21–24]. Thus, the metal-oxide nanomaterials decorated with reduced graphene oxide (rGO) may be of interest for gas-sensing applications [25,26], because rGO displays ideal physical and chemical properties, including high carrier mobility, superior specific surface area and abundant surface defects, which are beneficial to gas adsorption and redox reaction on the surface of materials [8,27,28]. Also, the hybrid heterojunctions between metal oxides and rGO can provide numerous carrier transport channels, resulting in more active sites for gas adsorption [29], which is a very important factor to improve the sensing performance of gas sensors. Sun et al. successfully synthesized novel 4.0 wt% rGO-decorated CdS/CdO heterojunction materials for the first time by a facile one-step hydrothermal method, which not only exhibited a 9-fold higher response and a 4.75-fold low detection limit than those of pristine CdS, but also possessed the response and recovery time of 76 and 82 s towards 2 ppm NO_2 gas at 125℃ [30]. Ren et al. prepared a NiO@rGO nanocomposite by a freeze-drying method combined with heat treatment, and this material showed a gas-sensing response of 0.64, with the response/recovery time of 28 and 142 s towards 1% of H_2 gas at 50℃ [31]. Furthermore, Ramgir et al. reported the successful detection of NO_2 gas via hydrothermally synthesized SnO_2/rGO nanohybrids, which demonstrated the response/recovery time of 10 and 40 s for 1 ppm NO_2 gas at 200℃ with the limit of detection of 0.5 ppm [8]. Although a lot of studies were done on the applications of metal-oxide nanomaterials modified with rGO in NO_2 gas sensors, the response speed and the operating temperature of gas sensor are still not ideal. According to abundant surveys, it is quite plausible that the nanostructured CuO decorated with rGO can substantially improve the gas-sensing properties to realize fast response and room-temperature operation for detecting ppb-level NO_2 gas. Moreover, the hydrothermal method is one of the most facile and ultra-low-cost methods to obtain the required superior nanomaterials, owing to its low operating temperature, one-step process, and high reproducibility [32,33].

In this regard, we presented a green and facile hydrothermal process to synthesize uniform CuO/rGO nanocomposites combined with a thermal treatment. The structural and functional analysis of the products was performed using various characterization means. With our approach, the gas sensor based on CuO nanoflakes modified with rGO

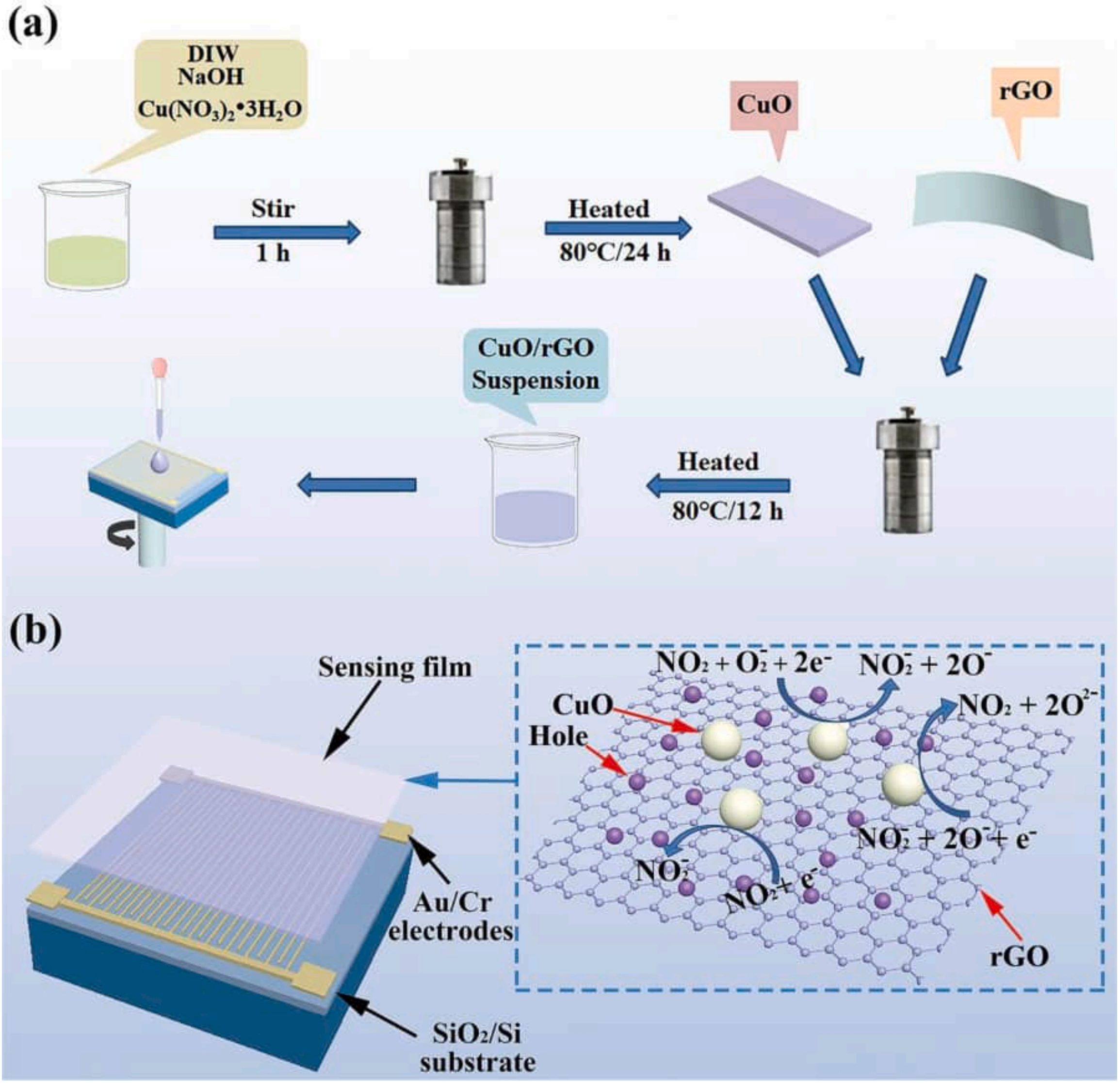

Fig. 1. (a) Experimental setup for the sensor. (b) Schematic diagram of the gas sensor.

nanosheets provided an efficient strategy for more excellent gas-sensing performances than those of pure CuO based sensor for detecting NO_2 gas at 23℃. A plausible sensing mechanism of CuO/rGO gas sensor was also investigated in detail. The outstanding performances of porous CuO/rGO were likely ascribed to the synergistic effect of unique architectures, formation of hybrid heterojunctions, and abundant active adsorption/desorption sites.

2. Experimental sections

2.1. Materials and synthesis of CuO

Urea, copper nitrate trihydrate ($Cu(NO_3)_2 \cdot 3H_2O$), and sodium hydroxide (NaOH) were obtained from Sigma Aldrich, and rGO powders (surface area: 84.63 m^2/g) were purchased from Nanjing XFNANO Materials Tech. Deionized water (DIW) was obtained from local sources. All the above chemical reagents were used without further treatment. The CuO nanomaterials were synthesized via a green and low-temperature hydrothermal route, as depicted in Fig. 1a. Typically, 1.4 g of $Cu(NO_3)_2 \cdot 3H_2O$ and 2 mL of NaOH solution (1.0 M) were respectively added into 40 mL of DIW. Thereafter, the above mixture was stirred for 1 h to form a homogeneous solution, and the speed of the magnetic stirrer was 2000 rpm. Then, the above solution was transferred to the stainless steel Teflon-lined autoclave with 50 mL capacity. The reaction was maintained at 80℃ for 24 h in a vacuum oven. After cooling to room temperature naturally, the precipitates were collected by filtering and rinsing several times with ethanol and DIW. Afterwards, the precipitates were dried in a vacuum oven at 60℃ for 6 h. Finally, the products were obtained and further used after thermal treatment at 200℃ for 2 h.

2.2. Synthesis of CuO/rGO nanocomposites

The CuO/rGO nanohybrids were synthesized by a similar hydrothermal process, as described in Fig. 1a. In a typical synthesis process, 10 mg of single-layer rGO was dispersed in 10 mL of DIW with ultrasonic cleaning for 2 h to form a homogeneous solution with the concentration of 1 g/L. 32 mg of prepared CuO was dispersed in 30 mL of DIW and vigorously stirred for 2 h to form a well-distributed solution with the concentration of ~1.07 g/L. Then, the above two suspensions were mixed together and magnetically stirred for 1 h with the stirring speed of 2000 rpm. Subsequently, the mixture was heated in the 50 mL of Teflon-lined autoclave at 80℃ for 12 h. After cooling to RT, the synthesized product was dried in an oven at 60 °C for 6 h. Finally, the CuO/rGO nanocomposites were obtained by heat treatment at 200℃ for 2 h, and then dispersed in ethanol for further use.

2.3. Characterization

The crystalline phase of the products was identified by X-ray diffraction (XRD, X'TRA) using Cu Kα radiation (40 kV and 40 mA, 0.02°/step from 15° to 65°). Field-emission scanning electron microscopy (FE-SEM, JSM-7000 F) in conjunction with energy dispersive X-ray spectroscopy (EDX) was performed to observe the morphological features. Furthermore, high-resolution transmission electron microscopy (HR-TEM, JEM-200 CX) was conducted to confirm the crystallinity and microstructure, and Raman spectra were recorded on a Priston CCD Raman Microprobe (DL-2 Instruments) using a 514 nm DPGL-2200 laser at room temperature (DPGL-2200, Shanghai Gaoyi Laser Technology, China). The UV–vis diffuse reflectance spectra (DRS) of the products were measured by a double beam UV–vis spectrophotometer (TU-1901, PERSEE). In addition, X-ray photoelectron spectroscopy on the surface of the products was performed (XPS, AMICUS ESCA3200). The Brunauer-Emmett-Teller (BET) surface area of the as-prepared products was determined using a nitrogen adsorption analyzer (Quadrasorb-S1, Quantachrome, USA), and the pore-size distribution was estimated by the Barrett-Joyner-Halenda (BJH) method.

2.4. Gas sensor fabrication and measurement

Fig. 1b shows the interdigitated electrodes with Au (thickness ~120 nm) and Cr (thickness ~50 nm) layers on a SiO_2/Si wafer. After cleaning treatment, 2.0 μL of CuO/rGO ethanol solution (50 mg/mL) was deposited on the interdigitated electrodes by a spin-coated method. Typically, the substrate was spin coated at 1000 rpm for 30 s to fabricate gas-sensing films. Then, the CuO/rGO films were dried in an oven at 100 °C for 2 h. Thus, the CuO/rGO-based gas sensor was successfully fabricated. In the same way, the CuO-based gas sensor was prepared by the same routes as mentioned above. Finally, the fabricated interdigitated electrodes were connected into the gas sensing test system, as shown in Fig. S1 (in the Supplementary Material). Afterwards, the gas sensitivity of the CuO/rGO sensor was determined by a sensing system at 25 % relative humidity (RH). Additionally, the tested gases were standard gases, and they were diluted with nitrogen. The tested gases were connected to MF2600, which can control the desired gas concentration in test. The electrical resistance signal was recorded using a semiconductor parameter analyzer (Keithley 4200) with a bias voltage of 0.1 V. The sensor response was evaluated by the change in the resistance values with respect to the variation in NO_2 gas concentrations. The sensing response (S) can be defined as $S(\%) = |R_a - R_g|/R_g \times 100$ for the p-type response, while $S(\%) = |R_a - R_g|/R_a \times 100$ for the n-type response, where, R_a is the initial resistance value in ambient air, and R_g is the resistance value of sensors exposed to NO_2 gas. The response and recovery time (t_{res} and t_{rec}) are defined to be the time taken by the sensors to 90 % of the total resistance value change under target gas and air environment.

3. Results and discussion

3.1. Structural and morphological characterization

The morphological characteristics of CuO and CuO/rGO were obtained by low- and high-magnification SEM, as shown in Fig. 2a–d. It was concluded that the existing products exhibit flake-like nanostructures with 20~40 nm in thickness. In addition, the results confirm that the as-prepared flake-like CuO is uniformly well mixed with rGO nanosheets in CuO/rGO nanocomposites (see Fig. 2c and d). Subsequently, the XRD patterns of the as-prepared products were obtained to determine the phase compositions, indicating that the products have good crystallinity (see Fig. 2e). Overall, rGO shows a weak and broad diffraction peak corresponding to (002) at $2\theta = 24.2°$, which is in good agreement with the standard card PDF#41-1487, confirming the successful synthesis of rGO. In addition, the characteristic peaks centered at approximately $2\theta = 32.8°$, 35.9°, 39.1°, 49.1°, 53.7°, 58.5°, and 61.8° correspond to (110), (11−1), (111), (20−2), (020), (202), and (11−3) planes of monoclinic tenorite CuO (PDF#48−1548), respectively, with no other impurity peaks, indicating the successful synthesis of pure CuO after hydrothermal reaction. However, the diffraction peaks of rGO (002) emerge for the CuO/rGO nanocomposites, which may be ascribed to the lower content of rGO. As shown in Fig. 2f, the EDX spectrum of CuO exhibits the strong peaks of Cu and O elements, and the EDX spectrum of CuO/rGO clearly confirms the presence of Cu, O and C elements, with no other elements introduced, suggesting that our desired products are successfully synthesized. In order to further confirm the the composition of the products, the SEM mapping images of CuO and CuO/rGO are also analyzed in Fig. S2, and the results further indicate the existence of Cu, O and C elements in CuO/rGO.

The microstructure of the formed products was further studied using TEM and HRTEM, as shown in Fig. 3. Fig. 3a and b shows curled rGO thin layers with an area of a few square micrometers and the lattice distance of 0.33 nm for rGO corresponding to the (002) plane. Moreover,

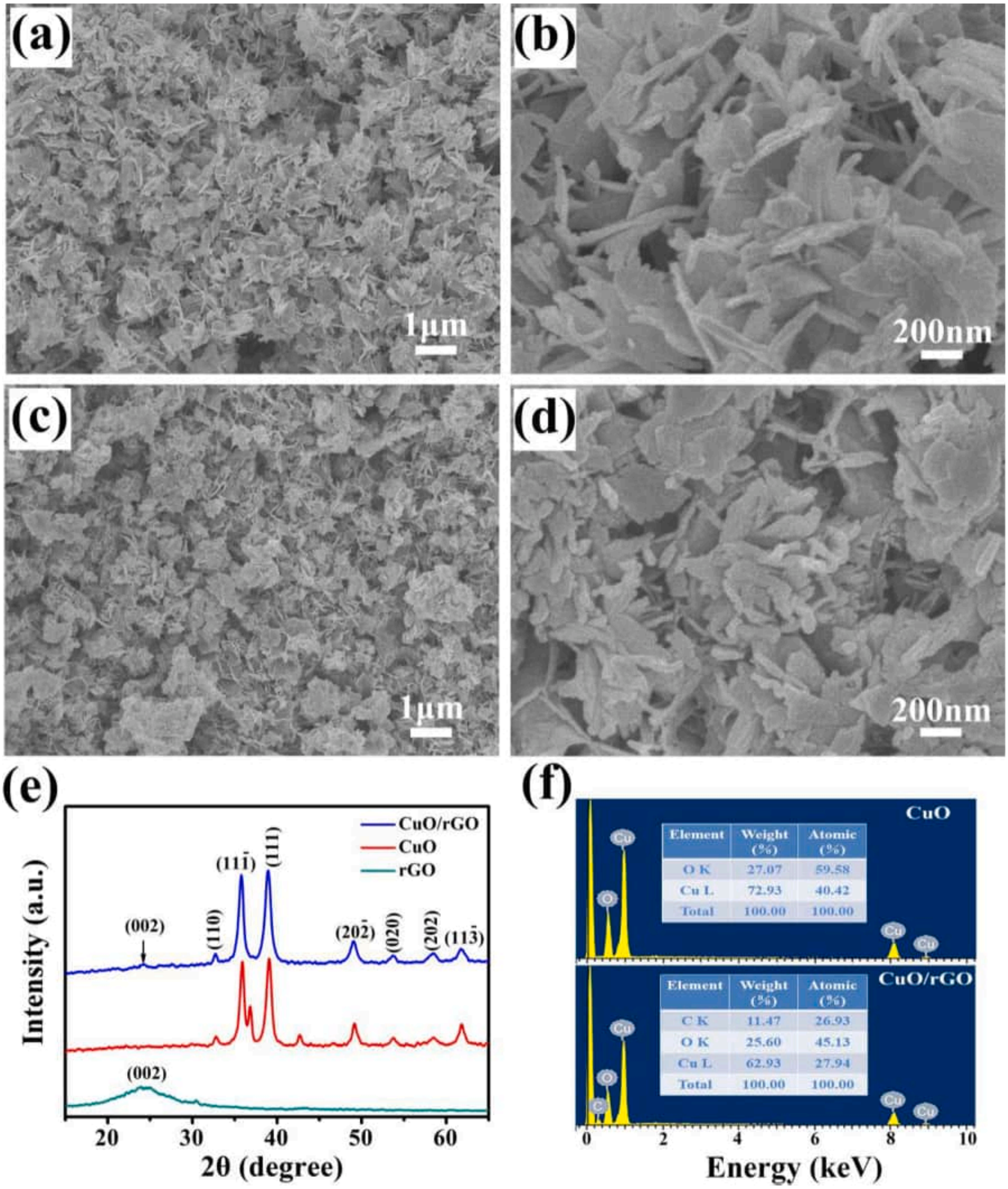

Fig. 2. Low- and high-magnification SEM images of (a) and (b) CuO, (c) and (d) CuO/rGO. (e) XRD patterns of CuO/rGO, CuO and rGO, respectively. (f) EDX spectra of CuO and CuO/rGO.

it can be observed from the low-magnification TEM images of CuO and CuO/rGO that the existing products exhibit flake-like nanostructures, which matches well with the SEM images in Fig. 2. In addition, the selected area electronic diffraction (SAED) pattern (inset in Fig. 3e) shows ring-like profiles, and the diffraction rings confirm that the CuO/rGO composites are successfully synthesized. In Fig. 3d and f, the lattice distance of 0.26 nm for CuO corresponds to the (111) plane, which is in good accordance with the XRD analysis. Also, it can be signified from Fig. 3d and f that the wrinkled rGO thin layers attach well to the surface of CuO to form a stable hybrid heterojunction between CuO and rGO, such well intact interfaces may be beneficial to the carrier transfer.

3.2. XPS, Raman and BET analysis

Gas sensing is known to be a surface phenomenon, and the surface composition and chemical state of materials play a significant role in governing the sensing mechanism [34]. Thus, XPS of CuO/rGO was performed. The overall XPS survey spectrum confirms the main constituent elements of C, O, and Cu in the corresponding composites, as shown in Fig. 4a. Additionally, Fig. 4b shows the high-resolution spectrum of C 1s, which is fitted to four peaks with binding energies at 284.6, 284.9, 286.5 and 288.5 eV, attributed to C—C, C—OH, C—O—C and C—OOH in CuO/rGO. Notably, compared to that in GO, the content of C—O—C bonds in CuO/rGO decreased upon introduction of CuO (see Fig. S3). In contrast, the hydroxyl groups (C—OH) appeared suddenly

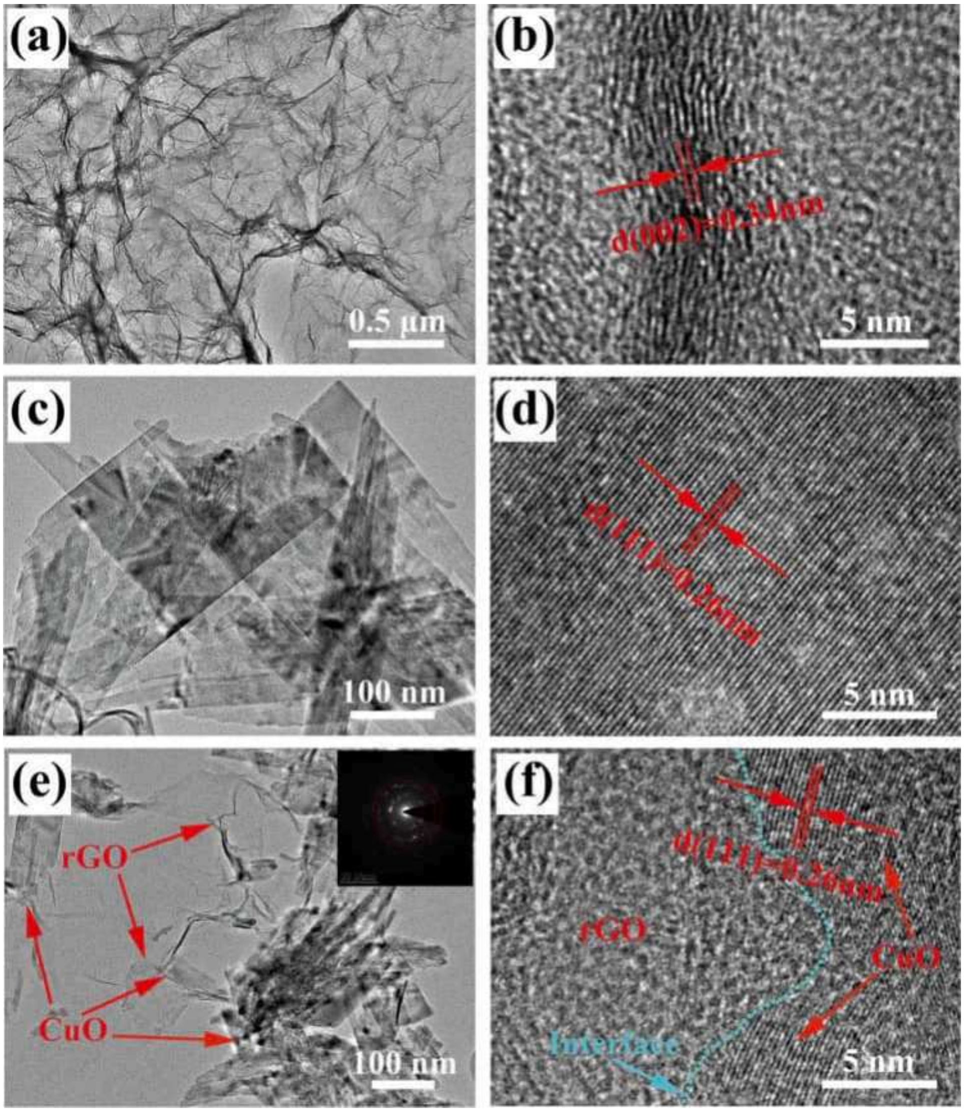

Fig. 3. TEM and HRTEM images of the as-prepared products: (a) and (b) rGO, (c) and (d) CuO, (e) and (f) CuO/rGO (SAED pattern inset (f)).

among the oxygen-based functional groups. This phenomenon may indicate that GO has been successfully reduced to rGO during the hydrothermal process, and pristine CuO is embellished by rGO [35]. Furthermore, the fitted peaks of O 1s at 529.6, 531.1, and 533.2 eV well correspond to lattice oxygen (O_L), oxygen-vacancy regions (O_V), and surficial-absorbed oxygen species (O_C), respectively. The chemisorbed oxygen species on the surface of the existing products exert a significant promoter action on the gas-sensing performance of materials. In particular, the O_V on the material surfaces may increase the selective adsorption and electron transfer for NO_2 to improve the selectivity and sensitivity [36]. Meantime, two resolved peaks of Cu 2p are observed in Fig. 4d, one peak is attributed to Cu $2p_{3/2}$ and centered at approximately 933.8 eV, and the other peak of Cu $2p_{1/2}$ at 953.9 eV. In addition, two satellite lines are observed, confirming the paramagnetic chemical state of Cu^{2+} in CuO/rGO [37].

The GO and CuO/rGO were also evaluated by Raman spectroscopy, and the typical peaks of the formed products were revealed (see Fig. 5a). As shown in Fig. 5a, both GO and CuO/rGO composites exhibit two dominant Raman peaks, in good agreement with the D and G bands of graphene, respectively. Generally, the relative intensity ratio of D to G (I_D/I_G) is an indication of the graphene quality. The value of I_D/I_G for the CuO/rGO composites ($I_D/I_G = 1.01$) is smaller than that for GO (1.13), indicating the recovery of the aromatic structure in the compound during the reaction process [38]. After introducing CuO, the G band of the CuO/rGO composites shifts to a lower wavenumber than that of GO, indicating that GO has been reduced [39]. Moreover, the D band is associated with a large number of defects, which may provide more active sites for gas adsorption, and it is conducive to surface reactions in the process of gas-sensing detection. In addition, the 2D band in GO is illustrated, which representes the graphene formulation induced by the photothermal laser reduction [40]. However, the 2D band is not exhibited in CuO/rGO composites. Two extra weak peaks of CuO/rGO centered at around 295 and 630 cm^{-1} can be obtained in the low-frequency Raman range and are indexed to the A_g and B_g modes of vibration of CuO. This implies that rGO may cover the CuO to form a stable hybrid heterojunction between CuO and rGO, and establish an excellent charge transfer pathway, thus endowing the potential superior gas-sensing performance of the CuO/rGO nanocomposites. In order to further prove that the CuO/rGO composites were successfully synthesized, UV–vis DRS of the products were performed in Fig. 5b. As is observed, the two products have high absorption intensity and wide absorption range in the visible and near-infrared region. With the modification of rGO, the light absorption of CuO/rGO is stronger than that of pure CuO in the near-infrared region, which is probably because rGO has good near-infrared effect to enhance the absorption of near-infrared light. In addition, the band gap energy of CuO/rGO is a

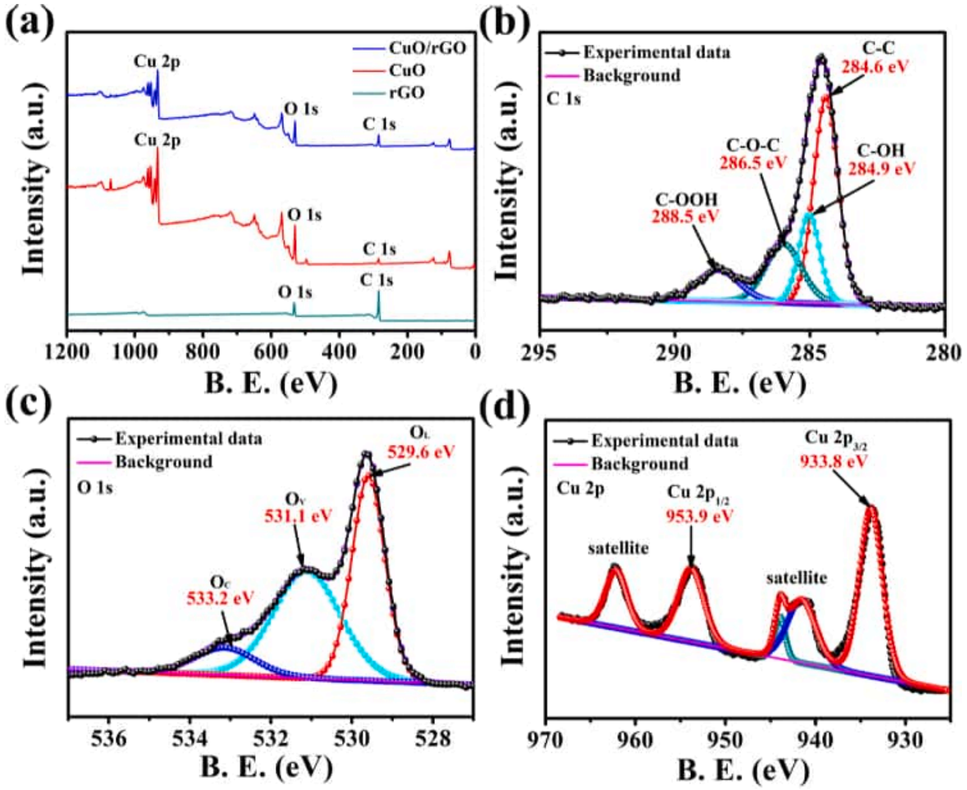

Fig. 4. (a) XPS spectra of CuO/rGO, CuO and rGO. XPS spectra of (b) C 1s, (c) O 1s, (d) Cu 2p for CuO/rGO.

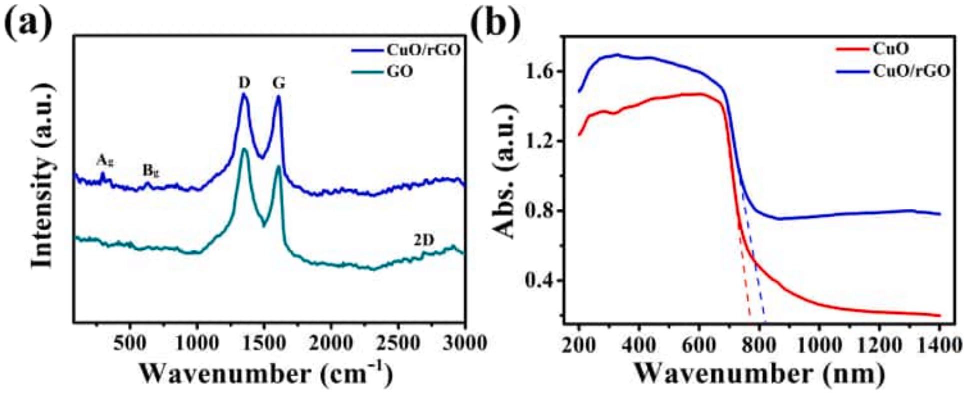

Fig. 5. (a) Raman spectra of GO and CuO/rGO. (b) UV–vis DRS of CuO and CuO/rGO.

little smaller than that of CuO, a slight red shift of absorption edge, which may be attributed to the formation of O—C chemical bonds in CuO/rGO [41].

Fig. 6a–c depicts the N_2 adsorption-desorption isotherms, BET surface areas, and the corresponding BJH pore-size distribution plots of the rGO, CuO, and the CuO/rGO composites, respectively. The isotherms of the formed products are almost the same and can be classified into type IV with clear hysteresis loops, indicating the relatively uniform distribution of pores. The rGO, CuO and CuO/rGO exhibit a uniform pore-size distribution of 2.19, 2.39, and 2.18 nm, indicative of mesoporous structures. Furthermore, the CuO/rGO show an increased pore volume compared to that of CuO. The larger pore volume is conducive to more gas diffusion into the materials and reactions on the surface of the materials. In addition, the BET surface areas of rGO, CuO and CuO/rGO were also determined to be 84.63, 47.97, and 76.07 m^2/g, respectively, revealing that with the introduction of rGO, the CuO/rGO composites have a higher specific surface area than pure CuO nanoflakes. This profits from the higher BET surface area of rGO and good pore interconnectivity upon deployment of CuO/rGO [42]. The uniform mesoporous structures and high BET surface area may provide more active sites for gas adsorption, which favors surface reactions in the gas-sensing process, resulting in rapid response/recovery kinetics and high response for gas sensors. Fig. 6d depicts the current-versus-voltage (I–V) curves of the as-synthesized products at 23℃. It makes clear that the linear characteristics of the I–V curves demonstrate Ohmic contacts between the formed products and substrate electrodes. Moreover, CuO/rGO exhibits better conductivity than pure CuO, which may be attributed to two factors [43]: (1) the overall surface area of the formed hybrid heterojunction between CuO and rGO and (2) the high carrier mobility of rGO.

3.3. Gas sensing performance

Fig. 7a and b shows the relevant resistance change of these two gas

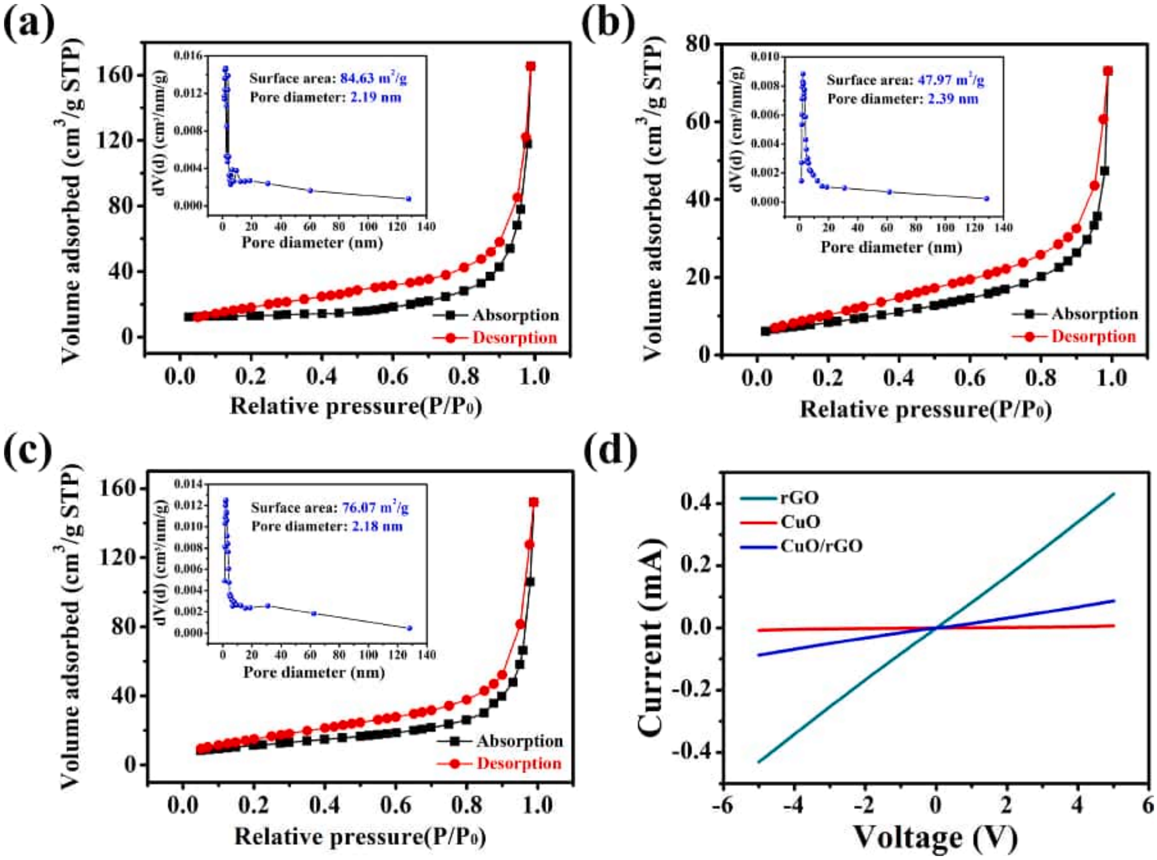

Fig. 6. N_2 adsorption-desorption isotherms (inset: pore-size distribution) of the as-prepared products: (a) rGO, (b) CuO, and (c) CuO/rGO. (d) IV curves of the as-prepared products.

sensors towards various NO_2 concentrations at 23℃, and it is found that the response performance is positively correlated with NO_2 gas. Fig. 7a and b show that, with the increase of concentration, the resistance change rate of CuO/rGO gas sensor is larger than that of CuO gas sensor, indicating that CuO/rGO gas sensor has a better response performance. Fig.7c depicts the time-dependent response/recovery curves of CuO and CuO/rGO sensors exposed to NO_2 gas with greatly varied concentrations ranging from 50 ppb to 100 ppm at 23℃. As is seen, the response values of CuO and CuO/rGO sensors are positively correlated with the gas concentrations of NO_2. For comparison, the CuO/rGO composite gas sensor exhibits a better gas-sensing performance than pristine CuO gas sensor, which can be ascribed to the formation of hybrid heterojunctions between CuO and rGO, as well as a superior specific surface area of CuO/rGO composite. Additionally, in the inset of Fig. 7c, we can see that even the NO_2 concentration is as low as 50 ppb, the gas response of CuO/rGO gas sensor is still over 20.6 %, which is about 6 times higher than that of CuO gas sensor (only 3.5 %). Upon further increasing the NO_2 concentration from 100 ppb to 100 ppm, the gas response of CuO/rGO gas sensor dramatically increases from 42.3%–3612.8% at 23℃, while the gas response of CuO gas sensor increases from 20.5%–1221.3%. The gas-sensing response values of these two sensors as a function of NO_2 gas concentration are demonstrated in Table S1. This phenomenon shows no difference with the reported works [44,45], which originates from the gas-sensing response following the Langmuir isotherm for gas molecule adsorption on the material surface, leading to an enhanced sensing response of the sensor with increasing the NO_2 gas concentration. Furthermore, the CuO/rGO gas sensor can respond more quickly than pure CuO with NO_2 gas intake. The results reveal that the CuO/rGO gas sensor has an outstanding reversibility towards NO_2 gas. Thereafter, Fig. 7d illustrates the normalized response curves of CuO and CuO/rGO sensors as a function of NO_2 gas concentration at 23℃, which shows that the gas responses of these two sensors tend to rise with increasing the concentrations of NO_2 gas, but the CuO/rGO sensor exhibits much higher increasing speed. From the insert of Fig. 7d, these two sensors exhibit better linear relationships between the response property and NO_2 at relatively low NO_2 gas. It is clear that the two fitted equations of the response Y for the CuO and CuO/rGO sensors and the NO_2 gas concentration X are respectively represented as $Y_1 = 130.0537X_1 + 3.3528$ and $Y_2 = 246.4963X_2 + 8.3203$, with regression coefficients $R_1^2 = 0.9872$ and $R_2^2 = 0.9842$. Such a good data fit can confirm that our sensors can be used to identify the source of leakage, and are very useful in a variety of safety-required applications [46].

It is universally known that the response and recovery time of gas-sensing materials to the target gas are key indicators to evaluate the sensitivity of gas sensors. Thus, the dynamic transient response curves of CuO and CuO/rGO gas sensors towards 5 ppm NO_2 gas at 23℃ were further explored (see Fig. 8). As can be seen, the response/recovery time of CuO and CuO/rGO gas sensors towards 5 ppm NO_2 gas are 40.5/66.7 s and 6.8/55.1 s, respectively. Interestingly, upon modification with rGO, the CuO/rGO gas sensor displays faster response/recovery characteristics than CuO gas sensor, which indicates a drastic improvement in the gas-sensing characteristics of CuO/rGO gas sensor. Moreover, Fig. S5 demonstrates the response and recovery time curves of these two sensors towards different concentrations of NO_2 gas at 23℃, indicating that the CuO/rGO gas sensor has a faster response time from 50 ppb to 100 ppm than that of CuO gas sensor. The faster response time for CuO/rGO composite gas sensors may be ascribed to the mesoporous structure and uniform combination between rGO and CuO, facilitating the redox reaction on the surface with an enlarged specific surface area [36]. Besides, the fast carrier transfer by the hybrid heterojunctions at the interface of rGO and CuO also accelerates the reaction process [42].

Fig. 9 depicts the repeatability of CuO and CuO/rGO sensors towards

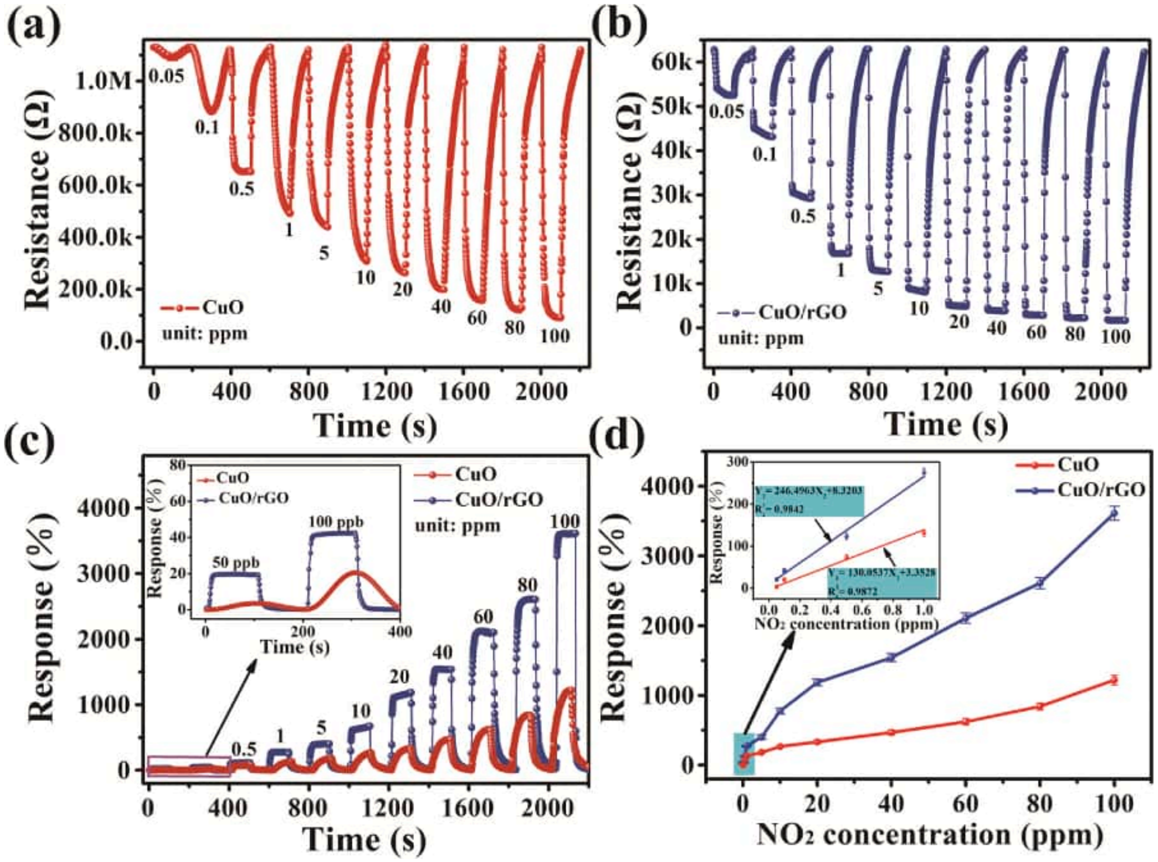

Fig. 7. Resistance change curves of (a) CuO and (b) CuO/rGO sensors towards various NO_2 concentrations at 23℃. (c) Dynamic response curves of CuO and CuO/rGO sensors exposed to NO_2 gas with various concentrations ranging from 50 ppb to 100 ppm at 23℃. (d) Normalized response curves of CuO and CuO/rGO sensors as a function of NO_2 gas concentration at 23℃.

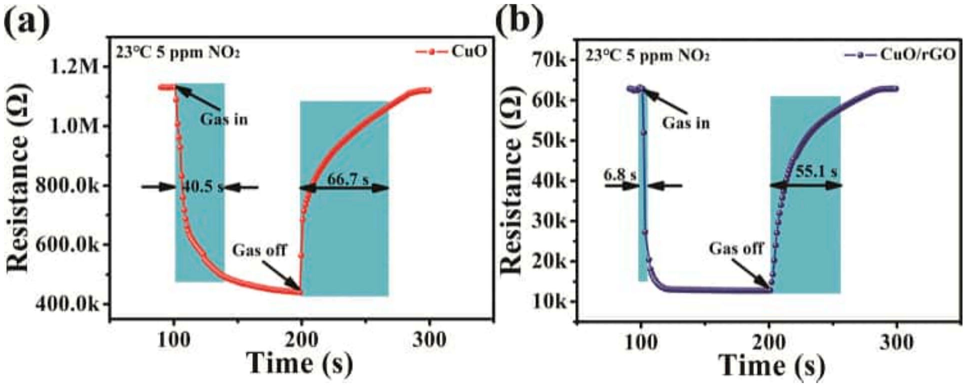

Fig. 8. The response and recovery time of (a) CuO and (b) CuO/rGO sensors towards 5 ppm NO_2 gas at 23℃.

different concentrations of NO_2 gas. The measurement was carried out for five cycles at 23℃. It is obvious that not only pure CuO sensor but also CuO/rGO sensor maintain excellent consistency and repeatability over the repeated experiments. As is known, the long-term stability of sensor is another indispensable factor for practical applications, so a comparative study on the long-term stability of CuO and CuO/rGO sensors was conducted, as plotted in Fig. 10a. The measurements were performed every 5 days for 30 days. It is found that although CuO and CuO/rGO sensors have negligibly decreased, they present rather stable responses with time, verifying the present sensors have a distinctive long-term stability. The corresponding dynamic resistance changes along with the long-term stability of sensors are also demonstrated in Figs. S6–S7. In addition, an outstanding selectivity is also one of the vital factors for gas sensors in practical applications. Therefore, the selectivity estimates for the sensors based on CuO and CuO/rGO sensors towards 100 ppm NO_2, H_2, SO_2, CO and NH_3 at 23℃ were carried out. Obviously, it can be seen from Fig. 10b that the CuO and CuO/rGO sensors show high gas-sensing responses of ~1221.3 and ~3612.8 % towards 100 ppm NO_2 gas, respectively. However, these two sensors show weak gas-sensing responses to other gas (including 100 ppm H_2, SO_2, CO and NH_3 gases), indicating that the CuO/rGO sensor exhibits an excellent selectivity. There may be two main reasons for the excellent selectivity: (1) At RT, target gases such as H_2, SO_2, CO and NH_3 can not fully react with the surface adsorbed oxygen species (O_2^-, O^-, or O^{2-}) due to their low oxidation ability and the high required reaction energy; (2) NO_2 is a highly electrophilic gas and can directly capture the electrons from the

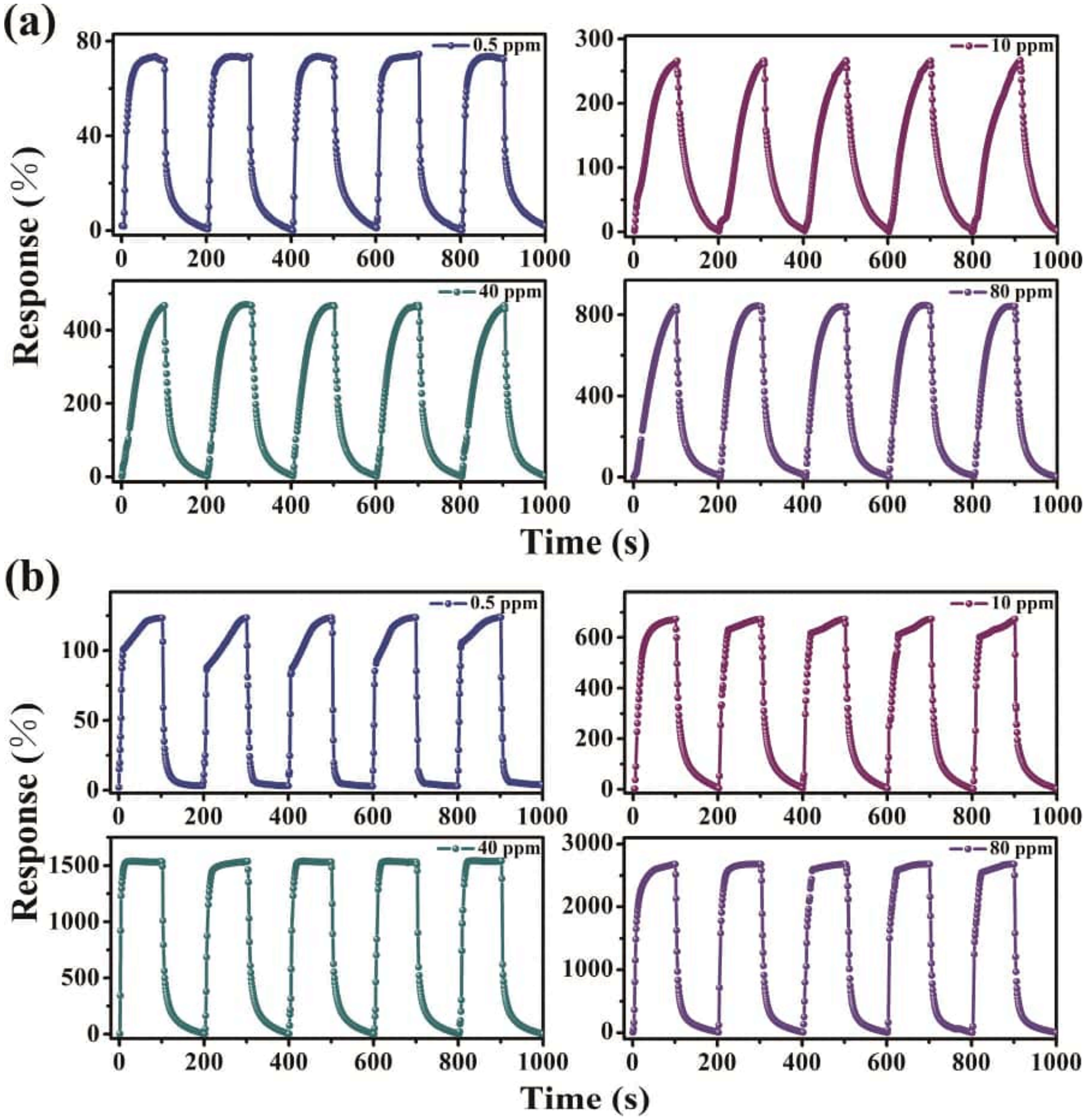

Fig. 9. Repeatability of (a) CuO and (b) CuO/rGO sensors towards different NO_2 concentrations: 0.5 ppm, 10 ppm, 40 ppm, and 80 ppm at 23℃.

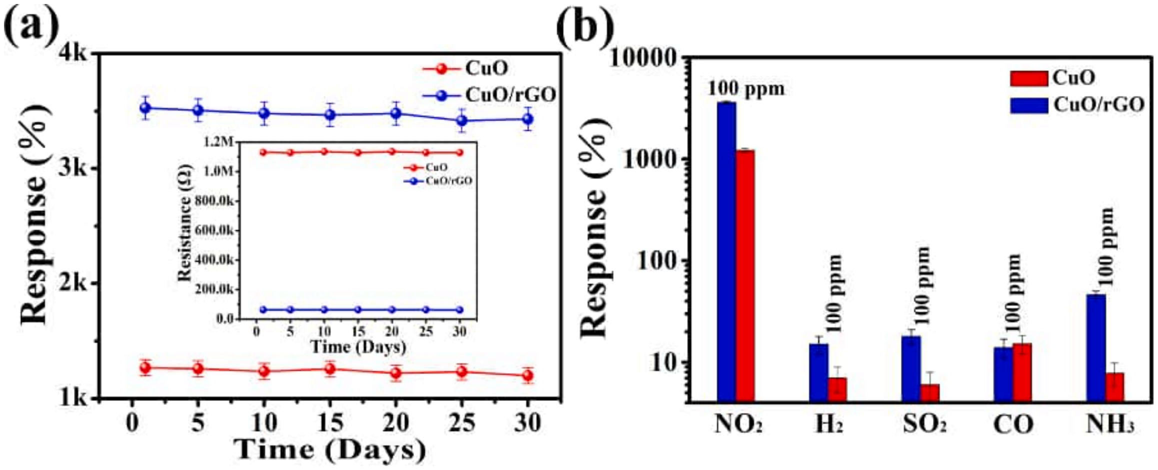

Fig. 10. (a) Long-term stability of CuO and CuO/rGO sensors towards 100 ppm NO_2 gas at 23℃. (b) Selectivity of CuO and CuO/rGO sensors exposed to various interfering gases at 23℃.

surface of the formed material to react with the adsorbed oxygen species, which may contribute to the improved selectivity for gas sensors. Moreover, the adsorption energy of NO_2 on the oxygen-vacancy site (~0.98 eV) is about three times larger than that on the perfect site (~0.3 eV) [47]. Thus, the oxygen vacancies on the surface of CuO/rGO may also enhance the selective adsorption and electron transfer towards NO_2 gas. In summary, it is believed that the present gas sensors may have tremendous and considerable potential for detecting trace NO_2 gas in practical applications.

The relative humidity (RH) is also another important condition for practical applications. Therefore, the relationships between the RH and the average response performance of these two sensors were further studied towards 100 ppm NO_2 gas at 23 °C, as shown in Fig. 11. As can be clearly seen, the response performance of these two sensors dramatically decrease with the increase of environment RH. Even so, when the environment RH reaches 85 %, the CuO/rGO sensor still has higher response performance than that of CuO sensor. Meantime, the corresponding dynamic resistance changes of these two sensors under different RH conditions are respectively presented in Figs. S8–S9. The response values of CuO sensor under 25, 36, 48, 60, 73 and 85 % of environment RH are 1221.3, 998.6, 680.6, 437.5, 221.6 and 97.1 %, respectively, whereas the CuO/rGO sensor are 3612.8, 3211.3, 2459.3, 1685.3, 1089.4 and 511.8 %, respectively. The results reveal that the adsorption of water molecules on the surface of the materials under high environment RH makes the chemisorption of oxygen more difficult, which is not conducive to the redox reaction, and leads to the decreasing response performance under high environment RH [48]. In addition, water molecules also block the adsorption of NO_2 gas. At high environment RH, the chemisorbed oxygen ions can react with water molecules on the surface of the materials and form the surface hydroxyls (OH^-) and a proton (H^+), which reduces the baseline resistance of the sensors and significantly deteriorates the response performance of the sensors [49].

A comparison of the various gas-sensing ability towards NO_2 between the current sensors and other reported nanomaterial-based sensors in previous literature is displayed in Table 1 [50–56]. Comparatively, this realistic comparison highlights that the present gas sensor here possesses high response and is particularly attractive because of its shorter response/recovery time and high response towards NO_2 at RT.

3.4. Gas sensing mechanism

The present CuO/rGO-based sensor exhibits gas-sensing performance towards NO_2 gas superior to that of CuO nanoflakes at 23℃. The excellent sensing performance may be ascribed to the synergistic effect of the mesoporous structures, extremely specific surface area and high

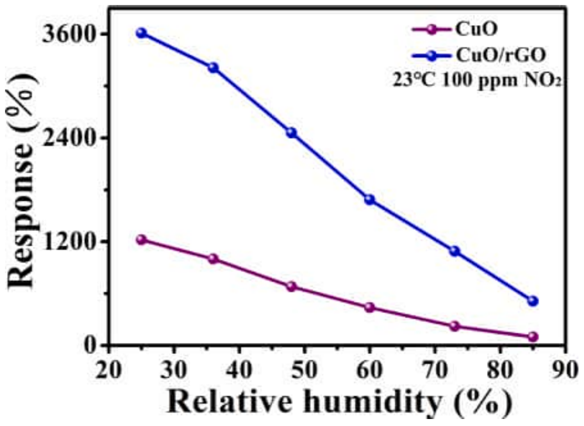

Fig. 11. Effect of relative humidity on the gas-sensing responses of CuO and CuO/rGO sensors towards 100 ppm NO_2 gas at 23℃.

carrier mobility of rGO. In general, the gas-sensing response mainly involves gas chemisorption, charge transfer and gas desorption procedures, as postulated in Fig. 12a. Notably, oxygen adsorption plays a significant role in the charge transport process of CuO/rGO composite. As shown in Fig. 12a, in air, due to good pore interconnectivity upon deployment of the present materials, adequate O_2 molecules can be quickly spread and easily adsorbed on the surface of the materials to form adsorbed oxygen ($O_{2(ads)}$) (in Eq. (1)). Then, the adsorbed oxygen will rapidly capture electrons from the conduction band of p-type CuO to form active O_2^- ions (in Eq. (2)). In addition, adequate oxygen vacancies also enable the adsorbed oxygen to form O^- ions (in Eq. (3)) [18]. Thus, the above conversion processes result in a significant increase in the hole concentration in the flake-like CuO. Since the work functions of CuO (5.3 eV) and rGO (4.8 eV) are different and the valence band level of CuO is lower than that of rGO [57], the holes in CuO will rapidly transfer to rGO until the Fermi level in the nanocomposite reaches equilibrium, as plotted in Fig. 12b. As a result, the hybrid heterojunctions are formed between the CuO and rGO interface, and the hybrid heterojunctions will provide numerous carrier transport channels, which can lead to more active sites for gas adsorption, which can not only improve the gas-sensing response, but also accelerate the redox reaction on the material surface [58]. Additionally, the fast carrier transport channels of rGO induce intensive hole transfer to the substrate electrodes. Hence, the existing sensor shows a dramatic resistance change. The reactions can be presented as follows [52].

$$O_{2(gas)} \rightarrow O_{2(ads)} \tag{1}$$

$$O_{2(ads)} + e^- \rightarrow O^-_{2(ads)} \tag{2}$$

$$O_{2(ads)} + e^- \rightarrow 2O^-_{(ads)} \tag{3}$$

Once the existing sensor is exposed to NO_2 gas, the adsorbed NO_2 molecules on the surface of CuO/rGO participate in redox reactions (in Eq. (4)). On the one hand, the adsorbed NO_2 molecules can capture electrons from the conduction band of the materials to form NO_2^- (in Eq. (5)) [59]. On the other hand, because NO_2 molecules have higher electronegativity than oxygen, the adsorbed NO_2 molecules will react with the surficial-absorbed oxygen species (O_2^-, and O^-) (in Eq. (6) and (7)) [60]. The reaction processes are as follows:

$$NO_{2(gas)} \rightarrow NO_{2(ads)} \tag{4}$$

$$NO_{2(ads)} + e^- \rightarrow NO^-_{2(ads)} \tag{5}$$

$$NO_{2(ads)} + O^-_{2(ads)} + 2e^- \rightarrow NO^-_{2(ads)} + 2O^-_{(ads)} \tag{6}$$

$$NO^-_{2(ads)} + 2O^-_{(ads)} + e^- \rightarrow NO_{2(gas)} + 2O^{2-}_{(ads)} \tag{7}$$

This phenomenon leads to further depletion of electrons and, conversely, a sharp increase in hole concentration, and the conductivity of the gas sensor is dramatically enhanced, leading to a drastic change in the sensor current of CuO/rGO. Another reason for the highly enhanced gas-sensing performance is the large specific surface area and abundance of defects of CuO/rGO composites, resulting in more active sites for gas adsorption/desorption and greatly accelerating carrier transfer during gas-sensing reactions. Besides, the present gas sensor is operated at RT, the water molecules in ambient air have an important effect on the gas-sensing properties of the sensor [61]. Therefore, the role of water molecules in gas-sensing mechanism should be seriously considered. At low environment RH, the chemisorbed oxygen ions will react with water molecules and form the surface hydroxyls (OH^-) [62]. Through the hydroxylation effect of humidity, the baseline resistance of the present sensor is decreased. With increasing RH, the proton (H^+) of water is reduced with the decrease of surface electron of CuO [63]. At high environment RH, the physical adsorbed water can cover the material surface, and then be reduced on the oxygen vacancy site of CuO. The H^+

Table 1
Comparison of various gas-sensing materials towards NO_2 gas, including the current sensor and other reported nanomaterial-based sensors in previous literature.

Products	Methods	NO_2 (ppm)	Temp. (°C)	S (%)	t_{res}/t_{rec} (s)	Ref.
p-CuO nanowires	thermal oxidation growth technique	10	300	55	~100/~200	[50]
Cu_xO/Graphene	vacuum-assisted reflux method	97	RT	95.1	9.6/ca. 200	[51]
MoS_2/G	chemical vapor deposition method	10	200	69	0.7/0.9	[52]
ZnO/Graphene aerogel	solvothermal method	100	RT	8.9	ca. 200/400	[53]
Cu_2O nanowire/rGO	hydrothermal conditions	2	RT	67.8	ca. 300/480	[54]
ZnO thin film	sol-gel method	100	200	37.2	6.72/52.62	[55]
In_2O_3 cubes/rGO	microwave-assisted hydrothermal method	5	RT	60.8	180/240	[56]
CuO nanoflakes/rGO	hydrothermal method	0.05	RT	20.6	31.8/60.6	This work
		5		400.8	6.8/55.1	

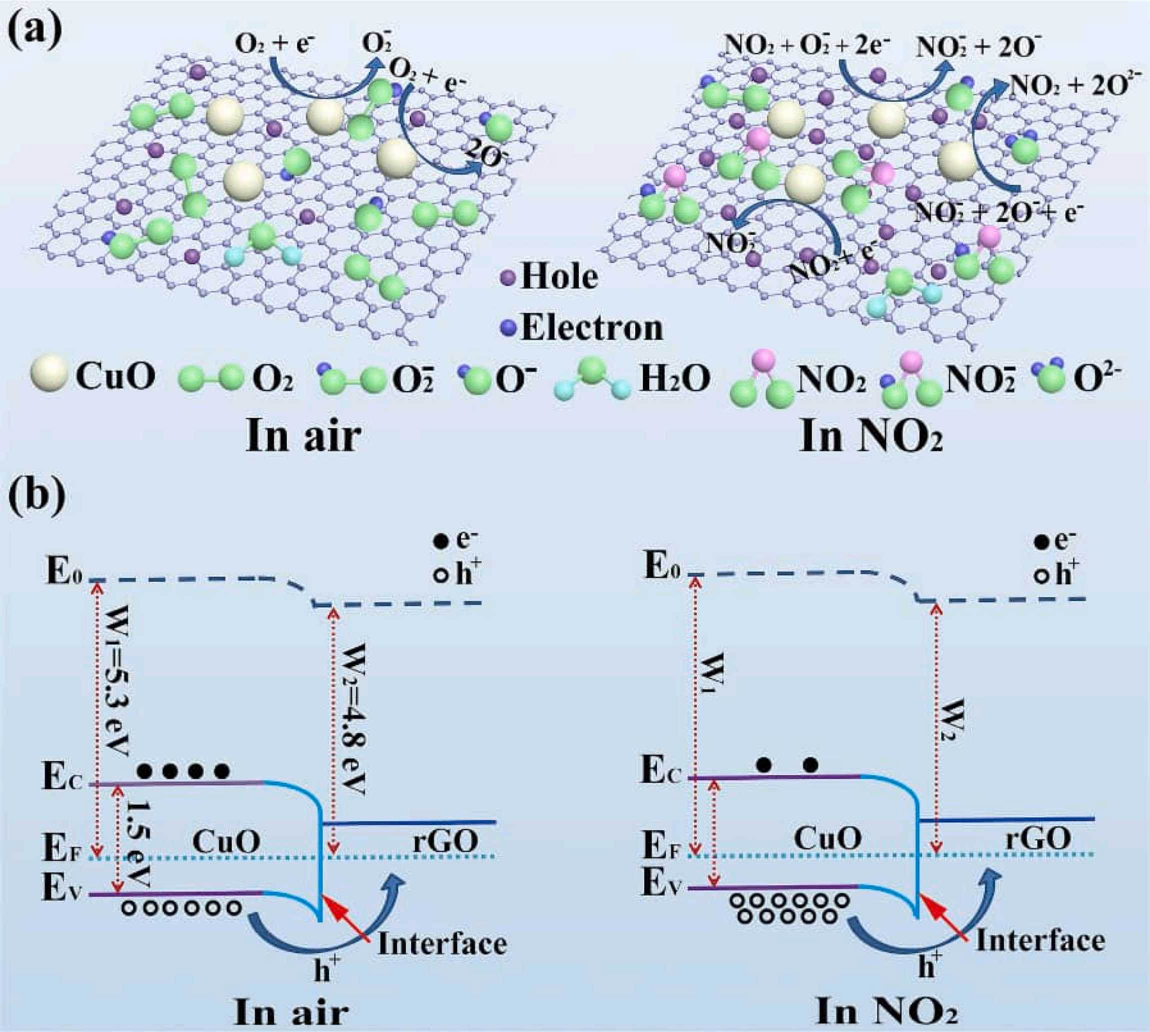

Fig. 12. (a) Schematic diagram of the gas-sensing mechanism for CuO/rGO-based sensor towards NO_2 gas at 23℃. (b) Energy band diagram of CuO/rGO and the hole transfer between CuO and rGO.

ions can move freely in the physisorbed water according to the Grotthuss's chain reaction. These give the reason why the sensor signals decrease towards NO_2 gas in high humidity. Therefore, vast works will be carried out to reduce the impact of humidity on the gas-sensing properties of the sensor in the future.

4. Conclusions

The gas-sensing materials of CuO/rGO composites were successfully synthesized by a cost-effective hydrothermal route followed by thermal treatment, and the morphology, microstructure and compositional characteristics were successively studied. The results show that the CuO/rGO composites had mesoporous structures, abundant defects and high specific surface area. Notably, the fabricated gas sensor based on CuO/rGO exhibited excellent performance towards ppb-level NO_2 gas at RT. In addition, the present sensor demonstrated a response to 5 ppm NO_2 gas equal to 400.8 % as well as fast response time of 6.8 s at RT. The LOD of the present sensor was down to 50 ppb with a response of 20.6 %. Furthermore, the proposed gas sensor also possessed superior consistency and repeatability, long-term stability (30 days) and superior selectivity towards NO_2 gas. Finally, a plausible gas-sensing mechanism of the present gas sensor towards NO_2 gas was explored in detail.

Obviously, our work will shine light on a promising candidate for ppb-level NO_2 gas-sensing applications.

CRediT authorship contribution statement

Haineng Bai: Conceptualization, Writing - original draft, Writing - review & editing. **Hui Guo:** Methodology, Data curation. **Jin Wang:** Software. **Yan Dong:** Investigation. **Bin Liu:** Investigation. **Zili Xie:** Formal analysis. **Fuqiang Guo:** Supervision, Formal analysis. **Dunjun Chen:** Supervision, Writing - review & editing, Resources, Funding acquisition. **Rong Zhang:** Validation. **Youdou Zheng:** Validation.

Declaration of Competing Interest

The authors report no declarations of interest.

Acknowledgements

This work was funded by the NSFC (No. 61634002); the NSAF (U1830109); and the Jiangsu Provincial Department of Water Resources, China (2019048).

Appendix A. Supplementary data

Supplementary material related to this article can be found, in the online version, at doi:https://doi.org/10.1016/j.snb.2021.129783.

References

[1] S. Jain, A. Paliwal, V. Gupta, M. Tomar, Long range surface plasmons assisted highly sensitive and room temperature operated NO_2 gas sensor, Sens. Actuators B: Chem. 311 (2020), 127897.
[2] R.K. Sonker, B.C. Yadav, V. Gupta, M. Tomar, Fabrication and characterization of ZnO-TiO_2-PANI (ZTP) micro/nanoballs for the detection of flammable and toxic gases, J. Hazard. Mater. 370 (2019) 126–137.
[3] M. Guarnieri, J.R. Balmes, Outdoor air pollution and asthma, Lancet 9928 (2014) 1581–1592.
[4] S.S. Shendage, V.L. Patil, S.A. Vanalakar, S.P. Patil, N.S. Harale, J.L. Bhosale, J. H. Kim, P.S. Patil, Sensitive and selective NO_2 gas sensor based on WO_3 nanoplates, Sens. Actuators B: Chem. 240 (2017) 426–433.
[5] J. Zhao, M.Q. Hu, Y. Liang, Q.L. Li, X.Y. Zhang, Z.Y. Wang, A room temperature sub-ppm NO_2 gas sensor based on WO_3 hollow spheres, New J. Chem. 44 (2020) 5064–5070.
[6] W.J. Du, L.L. Wu, J.B. Zhao, W.X. Si, F.L. Wang, J.R. Liu, W. Liu, Engineering the surface structure of porous indium oxide hexagonal nanotubes with antimony trioxide for highly-efficient nitrogen dioxide detection at low temperature, Appl. Surf. Sci. 484 (2019) 853–863.
[7] C.W. Na, J.H. Kim, H.J. Kim, H.S. Woo, A. Gupta, H.K. Kim, J.H. Lee, Highly selective and sensitive detection of NO_2 using rGO-In_2O_3 structure on flexible substrate at low temperature, Sens. Actuators B: Chem. 255 (2018) 1671–1679.
[8] B. Bhangare, N.S. Ramgir, S. Jagtap, A.K. Debnath, K.P. Mutheb, C. Terashima, D. K. Aswal, S.W. Gosavi, A. Fujishima, XPS and kelvin probe studies of SnO_2/RGO nanohybrids based NO_2 sensors, Appl. Surf. Sci. 487 (2019) 918–929.
[9] A. Katoch, J.H. Kim, Y.J. Kwon, H.W. Kim, S.S. Kim, Bifunctional sensing mechanism of SnO_2-ZnO composite nanofibers for drastically enhancing the sensing behavior in H_2 gas, ACS Appl. Mater. Interfaces 7 (2015) 11351–11358.
[10] J.N. Mao, B. Hong, H.D. Chen, M.H. Gao, J.C. Xu, Y.B. Han, Y.T. Yang, H.X. Jin, D. F. Jin, X.L. Peng, J. Li, H.L. Ge, X.Q. Wang, Highly improved ethanol gas response of n-type α-Fe_2O_3 bunched nanowires sensor with high-valence donor-doping, J. Alloys Compd. 827 (2020), 154248.
[11] Y.F. Luo, A. Ly, D. Lahem, C. Zhang, M. Debliquy, A novel low-concentration isopropanol gas sensor based on Fe-doped ZnO nanoneedles and its gas sensing mechanism, J. Mater. Sci. 56 (2021) 3230–3245.
[12] D.E. Motaung, G.H. Mhlongo, P.R. Makgwane, B.P. Dhonge, F.R. Cummings, H. C. Swart, S.S. Ray, Ultra-high sensitive and selective H_2 gas sensor manifested by interface of n-n heterostructure of CeO_2-SnO_2 nanoparticles, Sens. Actuators B: Chem. 254 (2018) 984–995.
[13] D. Wang, Y. Yin, P.C. Xu, F. Wang, P. Wang, J.C. Xu, X.Y. Wang, X.X. Li, The catalytic-induced sensing effect of triangular CeO_2 nanoflakes for enhanced BTEX vapor detection with conventional ZnO gas sensors, J. Mater. Chem. A 8 (2020) 11188–11194.
[14] H. Guo, X.L. Jia, Y. Dong, J.D. Ye, D.J. Chen, R. Zhang, Y.D. Zheng, Applications of AlGaN/GaN high electron mobility transistor-based sensors in water quality monitoring, Semicond. Sci. Technol. 35 (2020), 123001.
[15] Y.J. Wang, D. Liu, J.B. Yin, Y.X. Shang, J. Du, Z.X. Kang, R.M. Wang, Y.L. Chen, D. F. Sun, J.Z. Jiang, An ultrafast responsive NO_2 gas sensor based on a hydrogen-bonded organic framework material, Chem. Commun. 56 (2020) 703–706.
[16] P. Rai, S.-H. Jeon, C.-H. Lee, J.-H. Lee, Y.-T. Yu, Functionalization of ZnO nanorods by CuO nanospikes for gas sensor applications, RSC Adv. 4 (2014) 23604–23609.
[17] Z.J. Li, Y.Y. Liu, D.F. Guo, J.J. Guo, Y.L. Su, Room-temperature synthesis of CuO/reduced graphene oxide nanohybrids for high-performance NO_2 gas sensor, Sens. Actuators B: Chem. 271 (2018) 306–310.
[18] N. Zhang, X.H. Ma, Y.Y. Yin, Y. Chen, C.N. Li, J.Z. Yin, S.P. Ruan, Synthesis of CuO-CdS composite nanowires and their ultrasensitive ethanol sensing properties, Inorg. Chem. Front. 6 (2019) 238–247.
[19] D.P. Volanti, A.A. Felix, M.O. Orlandi, G. Whitfield, D.J. Yang, E. Longo, H. L. Tuller, J.A. Varela, The role of hierarchical morphologies in the superior gas sensing performance of CuO-based chemiresistors, Adv. Funct. Mater. 23 (2013) 1759–1766.
[20] N.S. Ramgir, S.K. Ganapathi, M. Kaur, N. Datta, K.P. Muthe, D.K. Aswal, S. K. Gupta, J.V. Yakhmi, Sub-ppm H_2S sensing at room temperature using CuO thin films, Sens. Actuators B: Chem. 151 (2010) 90–96.
[21] E. Singh, M. Meyyappan, H.S. Nalwa, Flexible graphene-based wearable gas and chemical sensors, ACS Appl. Mater. Interfaces 9 (2017) 3454–34586.
[22] C.Z. Zhu, S.J. Guo, Y.X. Fang, S.J. Dong, Reducing sugar: new functional molecules for the green synthesis of graphene nanosheets, ACS Nano 4 (2010) 2429–2437.
[23] K.S. Kim, Y. Zhao, H. Jang, S.Y. Lee, J.M. Kim, K.S. Kim, J.H. Ahn, P. Kim, J. Y. Choi, B.H. Hong, Large-scale pattern growth of graphene films for stretchable transparent electrodes, Nature 457 (2009) 706–710.
[24] L. Banszerus, M. Schmitz, S. Engels, J. Dauber, M. Oellers, F. Haupt, K. Watanabe, T. Taniguchi, B. Beschoten, C. Stampfer, Ultrahigh-mobility graphene devices from chemical vapor deposition on reusable copper, Sci. Adv. 1 (2015) e1500222.
[25] J. Hu, C. Zou, Y.J. Su, M. Li, Y.T. Han, E.S.-W. Kong, Z. Yang, Y.F. Zhang, An ultrasensitive NO_2 gas sensor based on a hierarchical Cu_2O/CuO mesocrystal nanoflower, J. Mater. Chem. A 6 (2018) 17120–17131.
[26] D. Konios, C. Petridis, G. Kakavelakis, M. Sygletou, K. Savva, E. Stratakis, E. Kymakis, Reduced graphene oxide micromesh electrodes for large area, flexible, organic photovoltaic devices, Adv. Funct. Mater. 25 (2015) 2213–2221.
[27] P.H. Zhu, S.S. Li, C.R. Zhao, Y. Zhang, J.H. Yu, 3D synergistical rGO/Eu(TPyP)(Pc) hybrid aerogel for high-performance NO_2 gas sensor with enhanced immunity to humidity, J. Hazard. Mater. 384 (2020), 121426.
[28] Y.W. Luo, D.Z. Zhang, X. Fan, Hydrothermal fabrication of Ag-decorated $MoSe_2$/reduced graphene oxide ternary hybrid for H_2S gas sensing, IEEE Sens. J. 20 (2020) 13262–13268.
[29] F.F. Yin, Y. Li, W.J. Yue, S. Gao, C.W. Zhang, Z.X. Chen, Sn_3O_4/rGO heterostructure as a material for formaldehyde gas sensor with a wide detecting range and low operating temperature, Sens. Actuators B: Chem. 312 (2020), 127954.
[30] J.H. Sun, L.X. Sun, N. Han, H.M. Chu, S.L. Bai, X. Shu, R.X. Luo, A.F. Chen, rGO decorated CdS/CdO composite for detection of low concentration NO_2, Sens. Actuators B: Chem. 299 (2019), 126832.
[31] H.B. Ren, C.P. Gu, S.W. Joo, J.J. Zhao, Y.F. Sun, J.R. Huang, Effective hydrogen gas sensor based on NiO@rGO nanocomposite, Sens. Actuators B: Chem. 266 (2018) 506–513.
[32] H.N. Bai, F.Q. Guo, B.H. Zhang, L.T. Gai, R.Q. Guo, One-step synthesis of high pure CdS nanofilms via hydrothermal method, J. Mater. Sci.-Mater. EL 29 (2018) 9193–9199.
[33] F.Q. Guo, H.N. Bai, B.H. Zhang, X. Li, Q. Yang, L.T. Gai, R.Q. Guo, Y.N. Huang, Controlled growth of highly pure TiO_2 nanorod arrays/nanoflower clusters via one-step hydrothermal route, J. Mater. Sci.-Mater. EL 29 (2018) 12169–12177.
[34] T.T. Wang, J.Y. Hao, S.L. Zheng, Q. Sun, D. Zhang, Y. Wang, Highly sensitive and rapidly responding room-temperature NO_2 gas sensors based on WO_3 nanorods/sulfonated graphene nanocomposites, Nano Res. 11 (2018) 791–803.
[35] Y.R. Choi, Y.G. Yoon, K.S. Choi, J.H. Kang, Y.S. Shim, Y.H. Kim, H.J. Chang, J. H. Lee, C.R. Park, S.Y. Kim, Role of oxygen functional groups in graphene oxide for reversible room-temperature NO_2 sensing, Carbon 91 (2015) 178–187.
[36] Y. Xia, J. Wang, J.L. Xu, X. Li, D. Xie, L. Xiang, S. Komarneni, Confined formation of ultrathin ZnO nanorods/reduced graphene oxide mesoporous nanocomposites for high-performance room-temperature NO_2 sensors, ACS Appl. Mater. Interfaces 8 (2016) 35454–35463.
[37] J. Kim, W. Kim, K. Yong, CuO/ZnO heterostructured nanorods: photochemical synthesis and the mechanism of H_2S gas sensing, J. Phys. Chem. C 116 (2012) 15682–15691.
[38] X.L. Zheng, J.B. Xu, K.Y. Yan, H. Wang, Z.L. Wang, S.H. Yang, Space-confined growth of MoS_2 nanosheets within graphite: the layered hybrid of MoS_2 and graphene as an active catalyst for hydrogen evolution reaction, Chem. Mater. 26 (2014) 2344–2353.
[39] Y. Cao, J.Y. Zhu, J. Xu, J.H. He, J.L. Sun, Y.X. Wang, Z.R. Zhao, Ultra-broadband photodetector for the visible to terahertz range by self-assembling reduced graphene oxide-silicon nanowire array heterojunctions, Small 10 (2014) 2345–2351.
[40] J.-E. Park, N. Oh, H. Nam, J.-H. Park, S. Kim, J.S. Jeon, M.Y. Yang, Efficient capture and raman analysis of circulating tumor cells by nano-undulated AgNPs-rGO composite SERS substrates, Sensors 20 (2020) 5089.
[41] X. Lin, D. Xu, Z. Lin, S.S. Jiang, L.M. Chang, Construction of heterostructured TiO_2/$InVO_4$/RGO microspheres with dual-channels for photogenerated charge separation, RSC Adv. 5 (2015) 84372–84380.
[42] S.F. Shao, L.S. Che, Y.Y. Chen, M. Lai, S.B. Huang, R.A. Koehn, Novel RGO-MoS_2-CdS nanocomposite film for application in the ultrasensitive NO_2 detection, J. Alloys Compd. 774 (2019) 1–10.
[43] J. Hu, C. Zou, Y.J. Su, M. Li, N.T. Hu, H. Ni, Z. Yang, Y.F. Zhang, Enhanced NO_2 sensing performance of reduced graphene oxide by *in situ* anchoring carbon dots, J. Mater. Chem. C 5 (2017) 6862–6871.

[44] Q. Zhu, H. Wang, J. Yang, C.S. Xie, D.W. Zeng, N. Zhao, Red phosphorus: an elementary semiconductor for room-temperature NO_2 gas sensing, ACS Sens. 3 (2018) 2629–2636.
[45] A.N. Abbas, B.L. Liu, L. Chen, Y.Q. Ma, S. Cong, N. Aroonyadet, M. Kopf, T. Nilges, C.W. Zhou, Black phosphorus gas sensors, ACS Nano 9 (2015) 5618–5624.
[46] X.J. Dong, K.L. Wu, W.F. Zhu, P.C. Wu, J. Hou, Z.X. Wang, R. Li, J.N. Wu, Z.Y. Liu, X.H. Guo, TiO_2 nanotubes/g-C_3N_4 quantum dots/rGO schottky heterojunction nanocomposites as sensors for ppb-level detection of NO_2, J. Mater. Sci. 54 (2019) 7834–7849.
[47] W. An, X. Wu, X.C. Zeng, Adsorption of O_2, H_2, CO, NH_3, and NO_2 on ZnO nanotube: a density functional theory study, J. Phys. Chem. C 112 (2008) 5747–5755.
[48] X.P. Song, L. Li, X. Chen, Q. Xu, B. Song, Z.Y. Pan, Y.N. Liu, F.Y. Juan, F. Xu, B. Q. Cao, Enhanced triethylamine sensing performance of α-Fe_2O_3 nanoparticle/ZnO nanorod heterostructures, Sens. Actuators B: Chem. 298 (2019), 126917.
[49] J.W. Yoon, J.S. Kim, T.H. Kim, Y.J. Hong, Y.C. Kang, J.H. Lee, A new strategy for humidity independent oxide chemiresistors: dynamic self-refreshing of In_2O_3 sensing surface assisted by layer-by-layer coated CeO_2 nanoclusters, Small 12 (2016) 4229–4240.
[50] J.H. Kim, A. Katoch, S.W. Choi, S.S. Kim, Growth and sensing properties of networked p-CuO nanowires, Sens. Actuators B: Chem. 212 (2015) 190–195.
[51] Y. Yang, C.G. Tian, J.C. Wang, L. Sun, K.Y. Shi, W. Zhou, H.G. Fu, Facile synthesis of novel 3D nanoflower-like Cu_xO/multilayer graphene composites for room temperature NO_x gas sensor application, Nanoscale 6 (2014) 7369–7378.
[52] H.S. Hong, N.H. Phuong, N.T. Huong, N.H. Nam, N.T. Hue, Highly sensitive and low detection limit of resistive NO_2 gas sensor based on a MoS_2/graphene Two-Dimensional Heterostructures, Appl. Surf. Sci. 30 (2019) 449–454.
[53] X. Liu, J.B. Sun, X.T. Zhang, Novel 3D graphene aerogel-ZnO composites as efficient detection for NO_2 at room temperature, Sens. Actuators B: Chem. 211 (2015) 220–226.
[54] S. Deng, V. Tjoa, H.M. Fan, H.R. Tan, D.C. Sayle, M. Olivo, S. Mhaisalkar, J. Wei, C. H. Sow, Reduced graphene oxide conjugated Cu_2O nanowire mesocrystals for high-performance NO_2 gas sensor, J. Am. Chem. Soc. 134 (2012) 4905–4917.
[55] M.A. Chougule, S. Sen, V.B. Patil, Fabrication of nanostructured ZnO thin film sensor for NO_2 monitoring, Interceram.-Int. Ceram. Rev. 38 (2012) 2685–2692.
[56] W. Yang, P. Wan, X.D. Zhou, J.M. Hu, Y.F. Guan, L. Feng, Additive-free synthesis of In_2O_3 cubes embedded into graphene sheets and their enhanced NO_2 sensing performance at room temperature, ACS Appl. Mater. Interfaces 6 (2014) 21093–21100.
[57] Y. Xu, M.A.A. Schoonen, The absolute energy positions of conduction and valence bands of selected semiconducting minerals, Am. Mineral. 85 (2000) 543–556.
[58] R. Kalidoss, S. Umapathy, R. Anandan, V. Ganesh, Y. Sivalingam, Comparative study on the preparation and gas sensing properties of reduced graphene oxide/ SnO_2 binary nanocomposite for detection of acetone in exhaled breath, Anal. Chem. 91 (2019) 5116–5124.
[59] Y.J. Zhong, W.W. Li, X.L. Zhao, X. Jiang, S.Y. Lin, Z. Zhen, W.D. Chen, D. Xie, H. W. Zhu, High-response room-temperature NO_2 sensor and ultrafast humidity sensor based on SnO_2 with rich oxygen vacancy, ACS Appl. Mater. Interfaces 11 (2019) 13441–13449.
[60] V.L. Patil, S.S. Kumbhar, S.A. Vanalakar, N.L. Tarwal, S.S. Mali, J.H. Kim, P.S. Patil, Gas sensing properties of 3D mesoporous nanostructured ZnO thin films, New J. Chem. 42 (2018) 13573–13580.
[61] H.N. Bai, H. Guo, J. Wang, Y. Dong, B. Liu, F.Q. Guo, D.J. Chen, R. Zhang, Y. D. Zheng, Hydrogen gas sensor based on SnO_2 nanospheres modified with Sb_2O_3 prepared by one-step solvothermal route, Sens. Actuators B: Chem. (2021), https://doi.org/10.1016/j.snb.2021.129441.
[62] H. Li, B. Liu, D.P. Cai, Y.R. Wang, Y. Liu, L. Mei, L.L. Wang, D.D. Wang, Q.H. Li, T. H. Wang, High-temperature humidity sensors based on WO_3-SnO_2 composite hollow nanospheres, J. Mater. Chem. A 2 (2014) 6854–6862.
[63] S. Pokhrel, K.S. Nagaraja, Electrical and humidity sensing properties of Chromium (III) oxide-tungsten(VI) oxide composites, Sens. Actuators B: Chem. 92 (2003) 144–150.

Haineng Bai received the master degree in Department of Physics, Changji University, in 2016. He is currently studying for his Ph.D. degree in School of Electronic Science and Engineering, Nanjing University, China.

Hui Guo received her master degree in 2015 from Department of Physics, Lanzhou University in China. Currently, she is studying for her Ph.D. degree in School of Electronic Science and Engineering, Nanjing University, China.

Jin Wang received the Ph.D. degree from School of Electronic Science and Engineering, Nanjing University, China in 2019. He is currently working in School of Electronic Science and Engineering, Nanjing University of Posts and Telecommunications in China.

Yan Dong is engaged in her post-doctoral research in School of Electronic Science and Engineering, Nanjing University, China.

Bin Liu is a professor in Nanjing University, China. His current research is preparation and application of semiconductor oxide, especial in gas sensor and solar cell. As a member of the academic team, he won the first prize of natural science and technological invention in Colleges and universities of the Ministry of education. He has visited the III–V semiconductor National Research Center of the University of Sheffield, the Royal Swedish Institute of Technology (KTH) and the Chinese University of Hong Kong.

Zili Xie is mainly engaged in the growth of nitrides semiconductor by MOCVD, device structure design, GaN based UV photodetectors and solar cells. During the Ninth Five Year Plan period, he was once an expert in the evaluation of GaAs single crystal materials in the field of 863 semiconductor materials. He has successively presided over national 863, National Natural Science Foundation, national defense 973 program and key projects of Natural Science Foundation of Jiangsu Province.

Fuqiang Guo is a professor in Changji University, China. Now, he is engaged in the preparation and application of gas sensors and photocatalysis of metal oxides and metal sulfides.

Dunjun Chen was funded by GSAS of Harvard University and was invited as a visiting scholar in its school of engineering and applied sciences from 2006 to 2007. In 2005, he was selected into the Ministry of education's new century excellent talent program, and in 2008, he was rated as the outstanding academic leader of young and middle-aged students of Nanjing University. His current research is nano-sensing materials and ultraviolet detector.

Rong Zhang has been committed to the growth of optoelectronic information functional semiconductor materials, new semiconductor materials, devices, and physical research. Now, he is a member of the national major special project of semiconductor lighting engineering, a member of the academic committee of the national key basic research planning (973) project, a member of the information science department of the science and Technology Commission of the Ministry of education, and an expert recommended and peer reviewed by Chen Jiageng Science Award of the Chinese Academy of Sciences,

Youdou Zheng graduated from the Physics Department of Nanjing University in 1957 and was elected academician of Chinese Academy of Sciences in 2003. He is engaged in the research of novel semiconductor heterostructure materials and devices. From 1988 to 1992, he was a member of the National Natural Science Foundation of China. From 1992 to 2001, he was a member of the National Natural Science Foundation of China (NSFC), a member of the national "863 Program" optoelectronic subject expert group, and a member of the national "climbing program" project expert committee.

Chem Soc Rev

ROYAL SOCIETY OF CHEMISTRY

REVIEW ARTICLE

View Article Online
View Journal | View Issue

Published on 17 February 2020. Downloaded by NANJING TECH UNIVERSITY on 2/1/2025 6:08:03 AM.

Check for updates

Cite this: *Chem. Soc. Rev.*, 2020, **49**, 1756

Printed gas sensors

Jie Dai,†[a] Osarenkhoe Ogbeide,†[b] Nasiruddin Macadam,†[b] Qian Sun,[ac] Wenbei Yu,[bd] Yu Li,[d] Bao-Lian Su,[d] Tawfique Hasan,*[b] Xiao Huang*[a] and Wei Huang*[ac]

The rapid development of the Internet of Things (IoT)-enabled applications and connected automation are increasingly making sensing technologies the heart of future intelligent systems. The potential applications have wide-ranging implications, from industrial manufacturing and chemical process control to agriculture and nature conservation, and even to personal health monitoring, smart cities, and national defence. Devices that can detect trace amounts of analyte gases represent the most ubiquitous of these sensor platforms. In particular, the advent of nanostructured organic and inorganic materials has significantly transformed this field. Highly sensitive, selective, and portable sensing devices are now possible due to the large surface to volume ratios, favorable transport properties and tunable surface chemistry of the sensing materials. Here, we present a review on the recent development of printed gas sensors. We first introduce the state-of-the-art printing techniques, and then describe a variety of gas sensing materials including metal oxides, conducting polymers, carbon nanotubes and two-dimensional (2D) materials. Particular emphases are given to the working principles of the printing techniques and sensing mechanisms of the different material systems. Strategies that can improve sensor performance *via* materials design and device fabrication are discussed. Finally, we summarize the current challenges and present our perspectives in opportunities in the future development of printed gas sensors.

Received 26th June 2019

DOI: 10.1039/c9cs00459a

rsc.li/chem-soc-rev

[a] *Institute of Advanced Materials (IAM), Nanjing Tech University (NanjingTech), 30 South Puzhu Road, Nanjing 211816, P. R. China. E-mail: iamxhuang@njtech.edu.cn*

[b] *Cambridge Graphene Centre, University of Cambridge, Cambridge CB3 0FA, UK. E-mail: th270@cam.ac.uk*

[c] *Shaanxi Institute of Flexible Electronics (SIFE), Northwestern Polytechnical University (NPU), 127 West Youyi Road, Xi'an 710072, P. R. China. E-mail: iamwhuang@nwpu.edu.cn*

[d] *State Key Laboratory of Advanced Technology for Materials Synthesis and Processing, Wuhan University of Technology, Wuhan 430070, China*

† These authors contributed equally to this work.

1. Introduction

The need for gas detection became apparent after the effects of harmful gases on human health were experienced by workers during the industrial age. As a result, industries where humans could be exposed to such gases required a method to alert them to the presence of the gas. This was the case for coal miners, and as a safety measure, gas detection methods were created for use in the mines. The first gas detector of the Industrial Age,

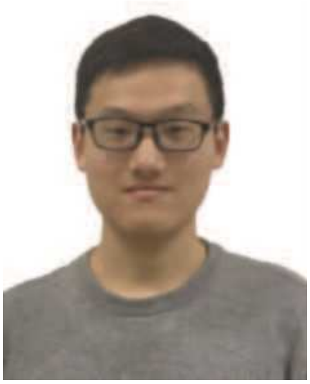

Jie Dai

Jie Dai received his bachelor's degree in 2017 from Nanjing Tech University. After that, he worked as a graduate student in the Institute of Advanced Materials (IAM), Nanjing Tech University, under the supervision of Professor Xiao Huang. His current research interest is on the synthesis and application of two-dimensional nanomaterial composites for gas sensing.

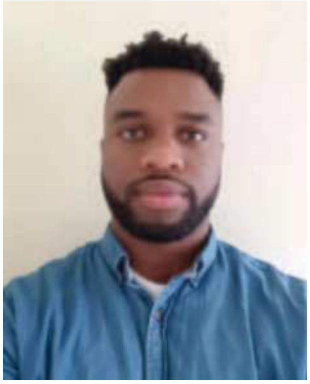

Osarenkhoe Ogbeide

Osarenkhoe Ogbeide received his Integrated Master's Degree in Interdisciplinary Science from the University of Leicester, UK (2016). He joined the Cambridge Graphene Centre as a PhD student in the Hybrid Nanomaterials Engineering (HNE) group in 2018, under the supervision of Dr Tawfique Hasan. His research is currently focused on addressing the limitations of traditional metal oxide gas sensors by nanoengineering. He is also a member of Churchill College.

This journal is © The Royal Society of Chemistry 2020

Published on 17 February 2020. Downloaded by NANJING TECH UNIVERSITY on 2/1/2025 6:08:03 AM.

the Davy lamp, created in 1816, consisted of an oil flame enclosed in a mesh screen.[1] The mesh would act as a flame arrestor, preventing flame propagation through it but still allowing for the flow of gas. In the presence of firedamp (*i.e.* methane and other flammable gases), the flame would burn higher with a coloured spire; with prolonged exposure the flame would increase in height reaching the top of the lamp, expand and cause the lamp to become insufferably hot and glow with a cherry-red heat,[2] providing a clear signal that the conditions in the mine were unsafe and miners should evacuate. However, the legacy of the Davy Lamp is marred by several cases of its use which led to an increase in the number of deaths from explosions and mine accidents. This was due to device mishandling and a false sense of security which resulted in miners working in previously closed off areas of the mines.[3]

In the early 20th century, the use of the Davy Lamp was eclipsed by the safer, more economical and reliable canary bird. As a method of early detection for carbon dioxide (CO_2), carbon monoxide (CO) and methane (CH_4), coal miners would bring the songful bird down into the tunnels in cages; a British mining tradition that dates to 1911. When exposed to the harmful gases even in small quantities, the canary would stop singing, display other signs of distress or die; signalling the need for evacuation. The use of canaries in Britain's coal mines persisted until 1986 before it was phased out by new, cheaper, quantifiable and more effective electronic detectors. In today's mines, sensors ranging from chemiresistive to electrochemical types can be found. By analysing the adoption of the canary bird and subsequent technologies in coal mines as a gas safety system, it was clear to see and appreciate the need for extensive sensor research for other industrial environments.

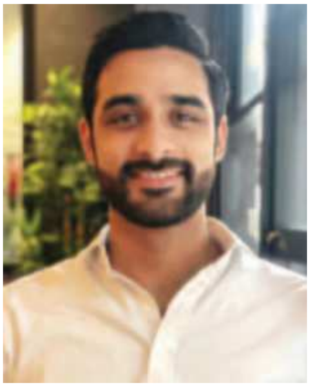

Nasiruddin Macadam

Nasiruddin Macadam received his MSci (integrated masters) degree in Chemistry from Queen Mary University of London in 2017. He received his second Masters in Graphene Technology from the University of Cambridge in 2018, where he is currently, completing a PhD under the supervision of Dr Tawfique Hasan. His current work is based on the exfoliation of two-dimensional materials and their formulation into a range of functional inks that are suitable for a variety of printing processes.

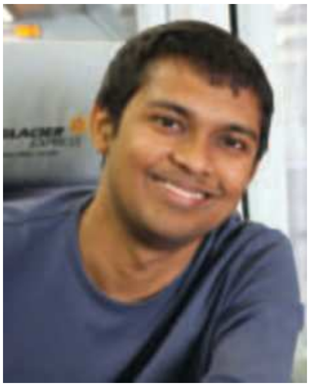

Tawfique Hasan

Tawfique Hasan is a Reader (Associate Professor) in Nanomaterials Engineering at the Cambridge Graphene Centre (CGC), Cambridge University Engineering Department. He received his PhD from Cambridge University in 2009. He currently leads the Hybrid Nanomaterials Engineering research group at CGC, with active research interests in 1D, 2D, 2D–2D and 0D–2D material systems for (opto)electronics, photonics and sensing. He has published more than 100 journal articles, including in Science, Science Advances, Nature Photonics, Nature Communications, Physical Review Letters and Advanced Materials, and has spun out two start-up companies, one of which was acquired by Versarien plc in 2017.

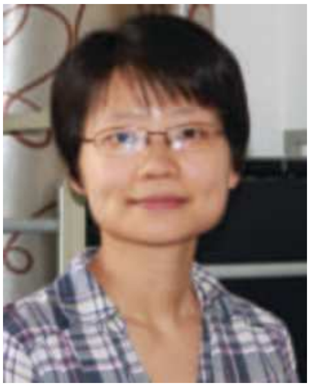

Xiao Huang

Xiao Huang received her bachelor's degree from the School of Materials Science and Engineering at Nanyang Technological University in Singapore in 2006 and completed her PhD in 2011 under the supervision of Prof. Hua Zhang and Prof. Freddy Boey. She is currently a professor at the Institute of Advanced Materials (IAM), Nanjing Tech University. Her research interest includes two-dimensional materials, their heterostructures, and related applications in sensing and energy.

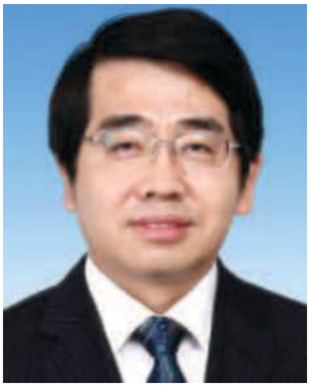

Wei Huang

Wei Huang is an academician of the Chinese Academy of Sciences, foreign academician of the Russian Academy of Sciences, foreign member of the ASEAN Academy of Engineering and Technology, fellow of the Royal Society of Chemistry and fellow of the Optical Society of America. He received his BSc, MSc and PhD from Peking University, and then carried out his postdoctoral research at the National University of Singapore, where he co-founded the Institute of Materials Research and Engineering. He is currently the deputy president and provost of Northwestern Polytechnical University, China. His research interests include polymer sciences, organic/plastic/flexible electronics, nanoelectronics and bioelectronics.

View Article Online

Published on 17 February 2020. Downloaded by NANJING TECH UNIVERSITY on 2/1/2025 6:08:03 AM.

In addition to their traditional use in coal mines, gas sensors have come a long way from the humble Davy lamp, and are now needed in a wider range of applications, from indoor/outdoor air quality control and chemical process control to industrial manufacturing and national defense. Sensors which are more responsive, safer to use, easier to operate, portable, economical and more sensitive to specific target analytes than existing technologies are highly desirable for industrial use. Consequently, much of the research in the field has largely focused on optimising sensing parameters which can be linked to the aforementioned qualities.[4–7] Beyond these performance metrics, modern applications additionally demand miniaturization for potentially portable and Internet of Things (IoT) enabled applications, with manufacturing processes supporting low material consumption and cost. Recent advancements in functional printing of nanostructured materials can adequately address all these challenges, and offer new opportunities and flexibility in the manufacturing of gas sensors.[8–11]

The unique strength and versatility in this family of additive manufacturing process are rooted in the traditional graphics printing technologies, developed over one hundred years. In 1903, Albert Hanson first prepared conductive copper traces laminated on to an insulating board.[12] This marked the beginning of the history of printed electronics. The first demonstration of printed circuits in 1936 by Paul Eisler facilitated the large-scale radio production during the World War II. This invention was later released for commercial use in the USA in 1948.[13] Since the mid-1950s, printed circuits have become key components in most commercial electronics. Over the years, the technology has diversified, and printed electronics have evolved and advanced in techniques, applications and materials. State-of-the-art printing techniques now offer customizable devices, nanoscale features, and even low-temperature integration of multiple materials and functions on a single substrate, paving the way for printed sensors.[14,15] Depending on the nature of the functional materials and the type of sensor designs, rheology of ink formulations, and ink drying dynamics require careful optimization for most suitable printing processes.[15,16] As for the sensor structures and working principles, the majority of printed gas sensors are chemiresistive[17,18] and field-effect transistor (FET) type devices that respond to the analyte gas *via* the change in channel conductance.[19–22] Their sensing performance such as sensitivity, selectivity, detection limit is closely related to the intrinsic properties of the nanomaterials (*e.g.*, electrical conductivities, doping levels, crystal defects, and surface chemistry).[23,24] The physical structure of the deposited nanomaterials, such as thickness, porosity, and interface with substrates and electrodes also play a vital role in the device performance.[25–27]

1.1 Electronic approaches to gas sensing and common sensing architectures

Gas sensors can be differentiated according to their operating principle of signal transduction into electrochemical,[28] optical,[29] acoustical,[30] thermometric[31] or gravimetric[32] sensors.[33] Among these, electrically-transduced gas sensors are one of the most investigated, primarily due to their simplicity, portability, compatibility with standard electronics, non-line-of-sight detection, capability of continuous monitoring and potential for wireless transmission.[34–36]

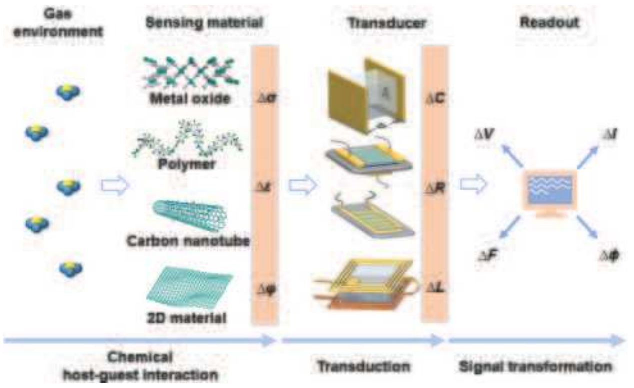

Fig. 1 Schematic illustration of electrical gas sensing process. Gas molecules interact with the sensing material changes some of its physical properties such as conductivity (σ), work function (φ) and permittivity (ε). The transducer converts one of these physical quantities into the variation of its electric parameters such as capacitance C, inductance L and resistance R. Finally, the circuit to which the sensor is connected gives rise to the sensing electrical signal which will be in either current (I) or voltage (V) and for each we can measure magnitude, frequency (F) and phase (φ).

An electrically-transduced gas sensing device generally contains two main components: the sensing material and the transducer (Fig. 1).[37] The sensing material, exposed to the ambient atmosphere, is responsible for covalent or noncovalent interaction with the target gas; undergoing a change in one or more of its physical properties (*e.g.* change in electrical conductivity ($\Delta\sigma$), work function ($\Delta\varphi$), and permittivity ($\Delta\varepsilon$)).[37] Using standard electronic components such as a capacitors, diodes, resistors, field-effect transistors (FETs), the changes in their physical properties are converted into variations in their electrical parameters such as capacitance (ΔC), resistance (ΔR) and inductance (ΔL), and finally outputted in either change of device current (ΔI) or voltage (ΔV) with measurable magnitude, frequency (ΔF) and phase ($\Delta \Phi$).[38–40] Printing-enabled gas sensors that convert gas–solid interaction into these electrical signals mainly take the following four device architectures, namely, chemiresistor, FET, capacitor and inductor (Fig. 2).[41–43] Before discussing these four kinds of sensors, we introduce a list of typical parameters that are used to evaluate a gas sensor's performance such as sensitivity, selectivity, limit of detection and drift in Table 1.

Chemiresistive gas sensors are one of the most explored sensor types for printed devices due to its relatively simple configuration and working principle.[44,45] A typical device consists of one to several pairs of electrodes, on top of which a layer of sensing material is deposited. To enlarge the area of sensing material in between the electrodes while keeping the electrode gaps narrow, interdigitated electrodes are widely used (Fig. 2a). A constant current or potential is applied across the device, and upon adsorption or desorption of a gaseous analyte, the electrical resistance or conductance of the sensing material

 This journal is © The Royal Society of Chemistry 2020

Published on 17 February 2020. Downloaded by NANJING TECH UNIVERSITY on 2/1/2025 6:08:03 AM.

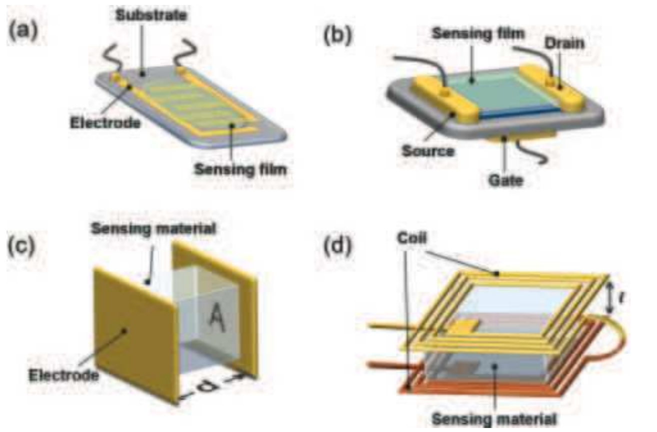

Fig. 2 Schematic illustration of (a) chemiresistive, (b) FET, (c) capacitive and (d) inductive gas sensors. In (c), A represents the interface area between the dielectric sensing material and electrodes while d represents the distance between the parallel electrodes. In (d), l represents the gap spacing between the coils.

changes, measured as the output. Despite the simple setup, chemiresistors are limited by their single type of output, that is, the resistance, which is easily influenced by environmental perturbations.

FET based gas sensors were demonstrated as early as in 1975 by Lundstrom.[49] In its simplest form, an FET consists of two electrodes (the source and the drain), connected by a semiconductor sensing layer as the channel, and a gate electrode located typically at the back of the substrate (Fig. 2b). When a source–drain voltage is applied, a current passes through the channel, whose charge carriers can be modulated by applying a gate voltage. This provides additional means to control the current response in the sensing layer upon interacting with a target gas. Outputs other than changes in channel current, such as the threshold voltage and sub-threshold swing, can also be used to reflect the sensing process.[20,50] In spite of additional complexity, these advantages make FETs more stable and reliable for gas sensing compared to chemiresistors.

Capacitive structures are widely used transducers for molecular detection, including DNA,[51] biomolecules[52] and gases.[53] Generally, a capacitive sensor consists of a layer of dielectric sensing material sandwiched between two parallel electrodes (Fig. 2c). The capacitance of the sensor can be expressed as $C = \varepsilon_0\varepsilon_r A/d$, where ε_0 is the permittivity in vacuum, ε_r is the relative permittivity of the sensing material, A is the capacitor area, and d is the distance between the electrodes.[54] Gas adsorption on the sensing material changes ε_r and thus alters the sensor capacitance. For the case of polymer based sensing materials, gas induced swelling and the subsequent change of d or A between the electrodes can also lead to a capacitance change.[55] The capacitor set up also allows for the measurement of material impedance, which correlates to both the resistive and capacitive changes in response to gas adsorption, usually in the range of zero to several megaHertz.[56] Therefore, it potentially provides additional measurement capabilities than chemiresistors, allowing for better selectivity and reliability for chemical detection.

Inductor-based gas sensors are less common as compared with the resistive or capacitive sensors, most likely due to their relatively complex circuit configuration. An example of such sensor is based on an L–C circuit that consists of a fixed capacitor and a variable inductor.[57] The inductor, as illustrated in Fig. 2d, is constructed by two spiral coils that are connected and aligned in parallel with a gap (d) between them. The sensing material, usually a polymeric material that swells/de-swells upon gas adsorption, is sandwiched in between the two coils. The total inductance of the system, $L_{(\text{total})}$, can be expressed as $L_{(\text{total})} = L + M_v(d)$, where L is the total self-inductance of the dual-coil and $M_v(d)$ is the mutual-inductance between the coils. Since $M_v(d)$ is a function of d, the change in $L_{(\text{total})}$ can be used to indicate the gas induced dimensional change of the sensing material in between the coils. One advantage of

Table 1 Gas sensing parameters and definitions[34,46–48]

Performance parameter	Definition
Sensitivity	The amount of change in a sensor's output in response to a target gas interaction, over the entire range of the sensor.
Limit of detection	The lowest concentration of target gas that can be reliably distinguished from its absence in a sample, with specified precision and reproducibility (typically 99% confidence interval).
Limit of quantitation	The lowest concentration of target gas that can be determined with acceptable precision and accuracy under the stated conditions of test.
Selectivity	The ability of a sensor to measure only one parameter, in the case of a chemical sensor, to measure only one target gas.
Response time	The time required for a stable output reading after exposure to the target gas.
Recovery time	The time at which the output signal reaches a certain percentage (*e.g.* 90–95%) due to incomplete recovery.
Signal-to-noise-ratio	The ratio of the power of a signal (meaningful information) to the power of background noise (unwanted signal).
Drift	Gradual departure of the instrument output from the calibrated output.
Reproducibility	The ability of the gas sensor to produce the same output signal after the experimental conditions have been altered.
Stability	The ability of the gas sensor to produce the same output signal when performing the same analytical measurement over a period of time.
Blank determination	A procedure which follows all steps of analysis but in the absence of target gas. It is used for detection and compensation of systematic analysis mistakes. The blank determination is applied when the blank analysis gives results with a nonzero standard deviation.
Linear regression	A linear approach to model the relationship between response (dependent variable) and the standard concentration for a limited range of concentration (independent variables).
Limit of blank	The highest apparent analyte concentration expected to be found when replicates of a blank sample containing no analyte are tested.

Published on 17 February 2020. Downloaded by NANJING TECH UNIVERSITY on 2/1/2025 6:08:03 AM.

such an inductive sensor is that it can be magnetically coupled with an external coil for wireless detection.

1.2 Data processing

Gas sensing devices which can distinguish between gas-phase chemical compounds with high confidence are useful for a range of applications (*e.g.* non-invasive early diagnosis of disease and monitoring of glucose control in patients with diabetes).[58,59] In light of this, the sensor must demonstrate high selectivity (ability to discriminate between target analyte and other interfering chemicals and environmental perturbations) in addition to high sensitivity.[60] In practice, the majority of the sensing materials show limited selectivity. To address this, the metrics from gas sensing data such as baseline, device voltage, response and recovery time, and analyte concentration can be collected and processed *via* dimension reduction methods (*i.e.* compressing large amounts of data into the essence of the original data) such as principal component analysis (PCA) for the discrimination of measured analytes.[60]

PCA is a widely used statistical approach which uses an orthogonal transformation to convert a set of observations of potentially correlated variables into a set of values of linearly uncorrelated variables called principal components (PC).[61,62] These observations can be features from the normalised gas response curve like: the maximum resistance change; in addition to, the area of the response and recovery sections of the curve.[60] It should be noted that normalisation is done to highlight the shape and direction of the curve for this analysis.[60] PCs describe the variation of the data and can be plotted on 2 or 3 axis for comparison; analytes/gases which are highly correlated, are grouped together on the plot.[58,60] It is important to note that the PC ranking (*e.g.* PC1 – the first principal component, PC2 – the second principal component, *etc.*) indicate the order of importance such that differences along the PC1 axis are more important than differences along the PC2 axis.[61] For example, PC1 is the axis that spans the most variation in the gas response. Similarly, PC2 and PC3 are the axes that span the 2nd and 3rd most variation in the gas response, respectively. There are a few diagnostic plots which can be used to assess the quality of the PCs. An example of this is a scree plot, which displays how much variation each PC can account for; an indication of a good group of PCs is when the majority of the variation is accounted for by PC1 and PC2.[63,64]

Fig. 3a shows normalized responses from a single unmodified-graphene chemiresistive sensor toward a set of volatile organic compounds (VOCs) and Fig. 3b shows the corresponding PCA transform, exhibiting excellent separability.[60] Further explanation for the percentages next to the PC1, PC2 and PC3 labels can be provided as these indicate the percentage of the variation that the respective PC accounts for (*e.g.* 88.23% of the variation in the data is accounted for by PC1). The inputs for this particular PCA plot were based on the following features from a gas response curve (Fig. 3c): the maximum change in resistance (ΔR), area of the response signal (A_{Resp}), area of the recovery signal (A_{Recov}), and a ratio of the response to recovery area ($A_{\mathrm{Resp/Recov}}$). The ΔR, A_{Resp}, A_{Recov} and $A_{\mathrm{Resp/Recov}}$ features were calculated for each measurement (11 compounds, 20 repetitions) to create a 4×220 feature vector as the input to PCA. In addition to the broad selectivity of the sensor, the ability of the sensor to correctly classify unseen gas response data can also be evaluated by a combination of cross-validation and linear discrimination analysis (LDA), *k*-nearest neighbour (kNN), random forest (RF), and support vector machine (SVM) classifiers.[60]

1.3 Conventional gas sensing materials

Electronic gas sensing materials are generally conducting or semiconducting in nature, and undergo changes of electrical properties upon exposure to gas. A diverse range of such materials has been explored over the past years, with different compositions, dimensionalities, and chemical/electrical properties investigated. In the following sections, typical gas sensing materials including metal–oxide semiconductors, conducting polymers, carbon nanotubes and 2D materials are briefly introduced.[65–67] Most of these materials are applied in chemiresitive gas sensors, and some of them in FET sensors.

1.3.1 Metal oxides. Metal oxides are among the most intensively studied gas sensing materials. Indeed, the majority of the commercialized gas sensors are based on them. Typical metal

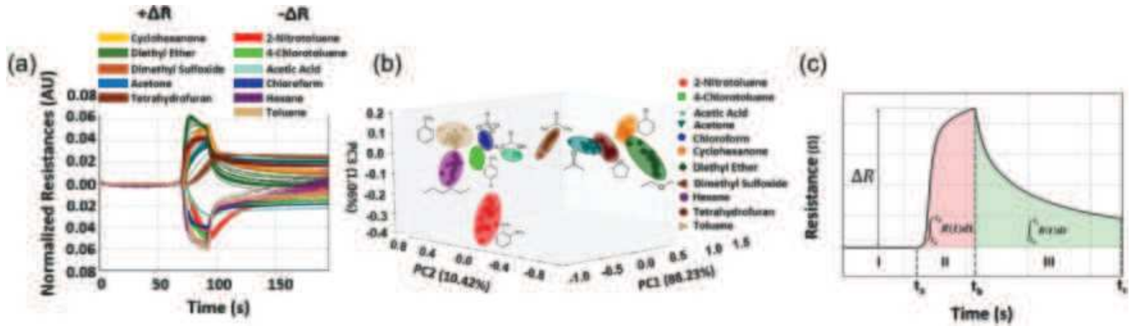

Fig. 3 (a) Normalized sensor response from a single unmodified-graphene chemiresistive sensor, to a chemically diverse set of compounds; (b) the corresponding PCA transform, the percentages next to the principal components indicate the percentage of variation the PCs and (c) typical sensor response curve with regions I, baseline; II, sample; and III, recovery. Potential features that can be extracted include the maximum change in resistance (ΔR), area of the response (A_{Resp}, red shaded area), and area of the recovery (A_{Recov}, green shaded area).[60] Reproduced from ref. 60. Copyright 2015, American Chemical Society.

Published on 17 February 2020. Downloaded by NANJING TECH UNIVERSITY on 2/1/2025 6:08:03 AM.

oxides for gas sensing include NiO, SnO_2, Fe_2O_3 and ZnO.[43,68,69] Their sensing mechanism is primarily based on the gas induced charge transfer and doping, with surface reactions sometimes also involved.[68,70,71] Therefore, they are particularly sensitive to polar or reducing/oxidizing gases. Because of the low conductivity and poor surface activity of common metal oxides under ambient conditions, chemiresistive gas sensors based on metal oxides typically work at elevated temperatures (*e.g.* 150–400 °C) to promote conductivity and surface reactions.[72] Making nano-sized metal oxide crystals and hybridizing them with other functional materials are effective strategies to lower their working temperatures in order for them to suit better with printed devices on arbitrary substrates.[73,74]

1.3.2 Conducting polymers. Conducting polymers are particularly attractive for detecting organic vapors.[24,75] Their tailorable molecular backbones, modifiable end groups and side chains make their electrical properties and surface chemistry highly tuneable toward specific gas response.[76,77] This allows for the rational design and selection of polymers for the development of sensor arrays.[78,79] Most importantly, polymeric materials are flexible and their solutions are compatible with various printing processes.[80] They are frequently utilized as matrix materials to be hybridized with functional fillers such as metal oxides, graphene and CNTs for additional gas sensing pathways which may enhance sensitivity, selectivity or/and shorten response time.[81,82]

1.3.3 Carbon nanotubes (CNTs). CNTs possess good chemical and mechanical stability, excellent electronic properties, and ultrahigh surface to volume ratios.[83] Their electronic transport properties can be easily modulated by surface absorbents. CNT based gas sensors are therefore extremely sensitive toward strong electron withdrawing or donating gases, such as NH_3 and NO_2, respectively, even at room temperature.[84,85] For detecting less reactive gases at room temperature, such as H_2 and H_2S, CNTs are usually modified with functional groups, polymers and noble metals to enhance their sensor performance.[86,87] The advantages of CNTs in these hybrid systems also lie largely in their ability to form continuous and electrically conductive networks,[88] as well as forming metal/semiconductor heterojunctions for modulation of barrier heights.[89]

1.3.4 2D materials. In recent years, graphene and its analogues such as metal chalcogenides, hexagonal boron nitride (h-BN), black phosphorus, and the MXene material family have demonstrated significant potential for gas sensing.[19,90–92] They are generally capable of providing abundant active sites, especially on edges and surface defects for gas–solid interaction, functionalisable surfaces for specific gas binding, and most importantly, multiple pathways for modulation of their electrical properties.[93–95] Depending on their chemical, physiochemical and electronic properties, different sensing mechanisms have been proposed, such as surface reaction, charge transfer and charge scattering.[96,97] The performance of 2D material-based sensors can be enhanced by doping, formation of heterostructures[98] and tuning surface/edge electronic structure,[99] and defect engineering.[93,100] Although a great number of works have explored the printed gas sensors based on graphene-related materials,[8,101–103] those with other 2D materials have been much less explored, leaving a scope for further, wide-ranging investigations.

After a brief introduction to gas sensing history, common transducing pathways and data processing methods, and gas sensing materials, in the following section, we discuss common printing technologies and related ink formulation strategies. We then divide the various printed electrical gas sensors into four groups, sensors based on: metal oxides, conducting polymers, carbon nanotubes, and graphene and related 2D materials. In each group, we discuss the relevant literature on sensor structures, typical ink formulation, and device fabrication strategies, sensing mechanisms, and potential strategies for performance enhancement. In the concluding section, we present our views on the current trends, future materials, device designs and manufacturability, and technology convergence.

2. Printing

By incorporating functional materials into ink systems, printing can be used as an additive manufacturing process for electronic device fabrication.[104] Printed electronics is an area that merges electronic device manufacturing and graphics printing to yield high-quality electronic devices that are thin, lightweight, flexible, low-cost and environmentally friendly. It is much simpler than modern silicon electronics manufacturing techniques which consist of upwards of one hundred steps including repeated deposition, lithography and etching steps.[105] Printed devices have two distinctive differences compared to traditional Si based electronics: firstly, printing is an additive process, where materials are added layer by layer to make the device, producing less waste and consuming less energy. Secondly, in most cases, the function of the device is independent of the substrate, widening the scope of substrates these devices can be fabricated on. Moreover, printing may enable large-scale targeted deposition with surface features that could not be achieved with other means. With printing however, the accuracy and resolution are compromised. For high performance electronic systems where very fine features are required, this can be problematic. Besides, printing may not always be able to replicate properties and microstructures of deposited materials. For example, the surface chemistry of functional materials may not be preserved with 3D printing if polymer binders cannot be avoided in ink formulation. Inkjet printing may not be able to produce flatly deposited, self-assembled 2D materials as Langmuir Blodgett coating. Nevertheless, for many applications, the resolution of various printing techniques is already adequate and are continually improving. Provided that the functional materials required can be incorporated into an ink system, printing is suitable for manufacturing devices for a wide range of applications including optoelectronics, photonics, energy storage, and most relevant to this review, sensing.[14]

2.1 Printing methods

There are a range of printing methods with varying capabilities in terms of resolution and print speed. Their selection is

Published on 17 February 2020. Downloaded by NANJING TECH UNIVERSITY on 2/1/2025 6:08:03 AM.

primarily based on feature size, throughput and the substrate to be printed on,[106] along with the target application of the printed material. For example, inkjet printing is the most commonly utilised method for research and development of functional material-based devices due to the ease of prototyping and the requirement for only very small amounts (1–2 mL) of low loading ink (<0.1 wt%). In comparison, flexographic printing has a much higher throughput, allowing inexpensive, large scale device manufacturing, but requires large amounts (>1 L) of high loading (>10 wt%) ink, making it impractical for small-scale device prototyping.

The target application also dictates the most suitable printing method. For example, for typical battery electrodes, a thick film is desired, rendering inkjet printing inefficient with low loading ink and small droplet size. In this case, screen printing is a more suitable technique. Therefore, many factors should be considered when determining the most suitable printing method for device manufacture. Printing processes can be divided into two groups, contact and non-contact methods, outlined in the remainder of this section.

2.1.1 Non-contact printing methods. Non-contact printing methods are based on the use of a nozzle or a print-head to deposit a material on a substrate directly. The print-head or substrate can be moved precisely with respect to the other to create the desired pattern without the need for a mask or template, making it very attractive for prototyping. Fig. 4 shows three different non-contact printing techniques.

In inkjet printing, drops are ejected from an ink reservoir onto a substrate to directly pattern a desired image.[107] There are two main methods by which drops can be ejected; continuous inkjet (CIJ) and drop on demand (DOD) (Fig. 4a).[108] CIJ involves continuous jetting of drops passing through an electrostatic field which imparts a charge on selected drops. The charge deflectors selectively deflect the charged droplets to the substrate and uncharged droplets to the gutter collect, where the ink can be recycled. CIJ allows high jetting speed and enables the use of volatile inks with low probability of the nozzles clogging. In DOD, drops are only generated and jetted where required. There are a few ways in which drops can be generated. One example is with a voltage pulse in a piezoelectric DOD (P-DOD) system. The pulse applied to the piezoelectric actuator induces a change in its shape, exerting pressure on the ink in the reservoir to eject a drop. The ejected droplets have a volume of ~1–100 pL leading to a typical feature size of between 30–100 μm.[108,109] Electro-hydrodynamic (EHD) printing can be considered as a form of inkjet printing, where the droplets are pulled-out from the nozzle by an external electric field, rather than forced out as with P-DOD. EHD is capable of achieving extremely high resolution, down to ~1 μm.[110] To achieve this however, a much smaller nozzle is required. Naturally, this causes the nozzles to clog very frequently. Therefore, very dilute inks are usually used as a preventative measure. This has adverse effects on the quality of the print.[111] Ongoing developments in the technique are based on the design of dual-channel nozzles, where early work has shown a great reduction in the probability of the nozzle-clogging.[111] Inkjet printing is one of the slowest printing techniques due to the requirement of the print-head to scan the substrate. The printing speed can be increased by employing a greater number of nozzles, reducing the amount of movement required to build the pattern, potentially reaching speeds of up to ~10 m min^{-1}.[112]

Aerosol jet printing (AJP) is a method that has emerged more recently for use with functional material printing.[113,114] The process starts with the atomisation of a liquid ink into droplets with a diameter of 1–5 μm *via* either an ultrasonic or pneumatic (depicted in Fig. 4b) atomiser, which are transported by a carrier gas toward the print-head. A single or multi-stage coaxial sheath gas is added to focus the aerosol, by occupying the outer regions of the flow.[115] This results in a high density deposit, with feature size a fraction of the size of the nozzle opening. Patterning is achieved by the movement of the stage and the delivery of the aerosol is continuous. Therefore a shutter arm is used to block deposition in areas that are not to be patterned. Commercially available systems are capable of achieving minimum feature size of ~10 μm at translation speeds of up to 12 m min^{-1}.[116] AJP is ideally suited to the deposition of continuous features, such as interconnects or electrodes.

3D printing can be divided into various types. The most common one depends on material extrusion and represents an additive manufacturing process where polymeric structures are fabricated through their selective dispensing using a single or

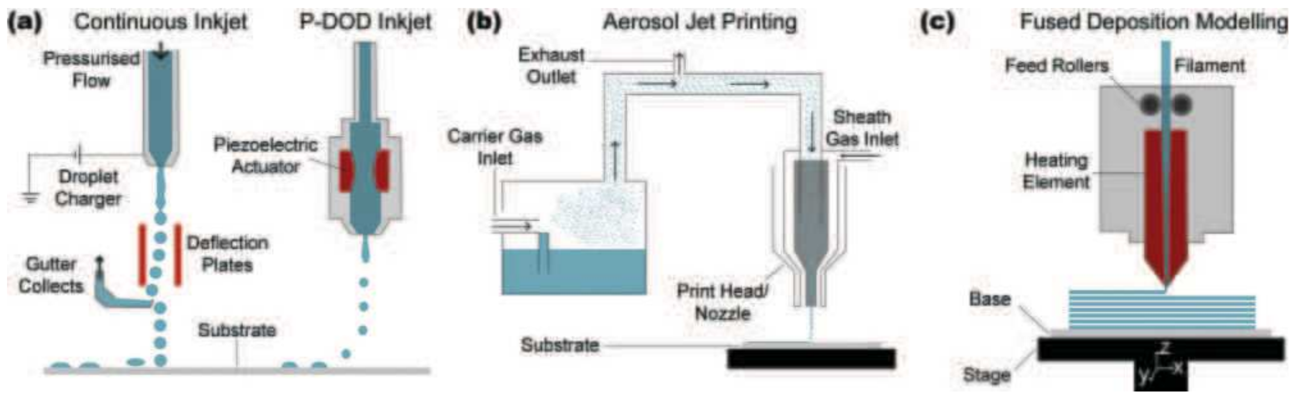

Fig. 4 Schematic representation of common non-contact printing methods. (a) Inkjet printing, continuous and drop on demand (DOD), (b) aerosol jet printing, (c) fused deposition modelling.

 This journal is © The Royal Society of Chemistry 2020

Published on 17 February 2020. Downloaded by NANJING TECH UNIVERSITY on 2/1/2025 6:08:03 AM.

multiple nozzles.[117] Techniques such as fused deposition modelling (FDM), 3D dispensing, 3D microextrusion, 3D fibre extrusion among others are all classed as material extrusion.[118] Typically, the desired structure is manufactured by the deposition of material, layer by layer, with the synchronous movement of the nozzle/extrusion head and the building platform.

3D printing has so far primarily been used to produce prototypes; the incorporation of functional materials in 3D structures has not yet been extensively explored. Of the techniques mentioned above, FDM is the most commonly utilised for functional material incorporation (Fig. 4c). In FDM, a thermoplastic filament is fed into a heated chamber where it melts and moves to a print head. The melted plastic extrudes from the nozzle and produces individual layers with a typical thickness of $\sim$100–250 μm.[112] Layer by layer deposition of both the 'build material' and a water soluble 'support material' is used to create complex and intricate 3D geometries. Incorporation of sensing materials into the build material filament for such FDM systems can be achieved by melt mixing of the material and a host thermoplastic such as polylactic acid (PLA), followed by melt extrusion.[119–121]

The advantages of material extrusion over other 3D printing techniques such as selective laser sintering (SLS) include lower input energy, lower material costs and reduced waste with greater consistency in accuracy.[118] Among material extrusion techniques, FDM is the most popular. With this said, 3D dispensing is an interesting technique which consists of printing based on the solidification of polymer achieved through a range of chemical or physical processes. It differs from FDM in that it does not use a solid filament, but a fluid precursor. An example of a physical process for 3D dispensing would be to use a shear thinning ink such as a paste or hydrogel, with the material regaining rigidity after extrusion.[122] Chemical 3D dispensing utilises reversible and irreversible crosslinking of polymers. Unlike FDM, the polymer solidification process is attained by crystallisation and chain entanglement. It is simpler to integrate functional materials in a liquid. Therefore, 3D dispensing is an attractive technique for the incorporation of functional materials in 3D structures.

2.1.2 Contact printing methods. Contact printing techniques are based on a component of the printing set up, for example a screen or a roller, contacting the substrate when the ink is deposited. These methods allow the scaling of printing processes to a much larger level. They are capable of a much higher throughput at a higher speed compared to the previously mentioned non-contact printing methods, as they can usually be adapted for roll-to-roll (R2R) processing.

Screen printing is a stencil process where ink is deposited on a substrate through a mesh made from silk, synthetic fibres or metal thread.[123] The non-imaging areas of the mesh are blocked with a resin, leaving the pores in the image areas open, allowing ink to pass through. The screen printing process starts with the flooding of ink on the screen followed by a squeegee being drawn across. The applied shear force pushes the ink through the open pores directly onto the underlying substrate (Fig. 5a). Screen printing can be semi/fully automated to

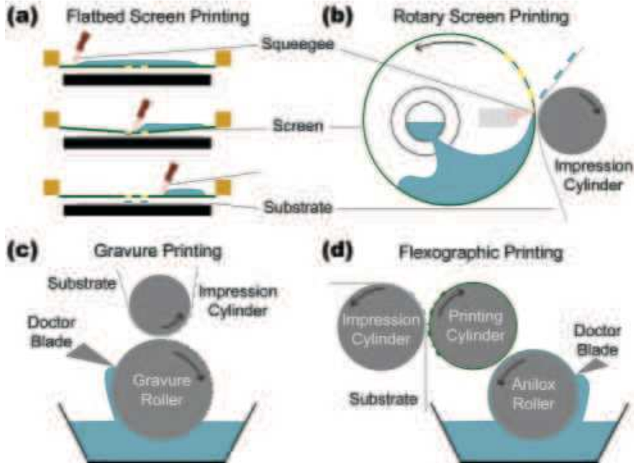

Fig. 5 Schematic representation of contact printing methods, (a) flatbed screen printing, and methods amenable to R2R processing: (b) rotary screen printing, (c) gravure printing and (d) flexographic printing.

increase the rate of printing. Fully automated flatbed presses can reach speeds of up to $\sim$70 m min^{-1}, and reduce inconsistencies between prints.[112] The process can be further adapted to incorporate R2R printing, as represented in Fig. 5b, with rotary screen printing. This permits faster operation of up to $\sim$100 m min^{-1} as the substrate does not need to be replaced after every run.

Gravure and flexographic printing are amenable to R2R processing and are widely used in large scale printing applications such as packaging.[112] For printing of functional materials, these methods have not been explored extensively due to a considerably higher initial cost and ink volume required for prototyping.

In gravure printing (Fig. 5c), the desired pattern is engraved into a metal roll, the gravure roller, which directly transfers the ink on to the substrate.[123,124] The roller is flooded with ink, and a doctor blade ensures ink only remains in the recessed image areas on the roll. The ink in the cells of the gravure roller are then transferred to the substrate with use of an impression roller. Gravure printing is capable of high resolution (10–50 μm) while achieving print speeds of up to 1000 m min^{-1}.

Flexographic printing (Fig. 5d), is a relief printing process, with the desired pattern protruding from the printing cylinder, compared to being flat for rotary screen and recessed in gravure printing.[123,124] Ink firstly fills the cells of the anilox roller, and the thickness is controlled using a doctor blade. The ink is transferred from the anilox roller to the printing cylinder, from which the desired pattern is printed onto the substrate. Flexographic printing is capable of a resolution of 45–100 μm at print speeds of up to $>$500 m min^{-1}, which can drop to $\sim$100 m min^{-1} for functional material printing.

Each of these above printing techniques have their drawbacks and specific advantages. It is therefore crucial to select the most appropriate printing method for application-specific process requirements and print precision. At the same time, the corresponding ink formulation is also an indispensable factor to be considered. This is discussed in the next section.

Published on 17 February 2020. Downloaded by NANJING TECH UNIVERSITY on 2/1/2025 6:08:03 AM.

2.2 Ink formulation

The differences in printing processes necessitates the formulation of inks with properties tailored to a specific printing technique. The fundamental components that constitute an ink are the pigment, the binder system, solvent(s) and additives.[123,124] The composition of these components is adjusted to yield inks with the required fluidic properties for specific printing technologies (Table 3).

The pigment traditionally is the colourant component in graphics inks, and can be replaced by functional materials such as metallic nanoparticles,[125] polymers,[126] carbon materials,[127] 2D materials[128] and more, to make the pigment "active". Any material that possesses the desired functionality, such as conductivity or response to a particular analyte can be incorporated into the functional ink, as long as they can be dispersed within a matrix of the other ink components.

The binder usually consists of polymers and act to bind the pigment particles together as well as providing adhesion to the substrate. Although binders are essential for graphics inks, they can limit the performances of printed devices. For example, in gas sensors, interaction between sensing materials and analytes is key to the sensing performance, which can be affected by the presence of commonly insulating polymer binders. For optimal gas sensing, post print removal of binders are therefore pursued.[129] Another approach to ink formulation for gas sensing is binder-free inks. This, however adds constraints to available printing techniques and typically, non-uniformity in the printed materials.[106]

The solvent acts as the carrier of the other components during the printing process, until it is deposited on to the substrate where it evaporates. The solvent can be aqueous or an organic and coupled with the binder, and significantly affects the viscosity and rheological properties of the ink. The properties of the solvent, especially the boiling point (bp) determine the applicability of the ink to different printing methods. For example, for gravure printing, rapid drying is required, dictating the use of solvents with low bp such as isopropanol. On the other hand, for screen printing, rapid drying is an issue that may lead to the blockage of the screen opening. Hence, solvents with a higher bp such as terpineol are typically employed to ensure sufficient dwell time on the screen.

Additives alter the properties of inks and the printed film. They are added in small proportions but can have a significant effect on the printability of the ink and the final printed film.

Table 3 Ink composition ranges for various printing methods; 'AJ' corresponds to aerosol jet[112,114,115,123,124,131]

Printing method	Typical ink composition (wt%)				Viscosity (mPa s)
	Pigment	Binder	Solvent	Additive	
Inkjet	5–10	0–20	65–95	0–5	4–30
AJ ultrasonic	$\leq$55	n/a	$\geq$45	n/a	1–10
AJ pneumatic	$\leq$75	n/a	$\geq$25	n/a	1–1000
Screen	12–20	40–65	20–30	1–5	1000–10 000
Gravure	12–17	20–35	60–65	1–2	100–1000
Flexographic	12–17	40–45	25–45	1–5	1000–2000

An example of a type of additive is a defoamer which limits the formation of unwanted bubbles during printing.[123]

For inkjet printing, especially DOD, stable generation and jetting of droplets is essential while avoiding the formation of secondary (satellite) droplets to ensure accurate deposition of the material. A dimensionless figure of merit (FOM) that can be used to predict the stable jetting of droplets is the Ohnesorge (Oh) number which can be derived from other FOMs; the Reynolds (Re) and the Weber (We) numbers;[128] $\mathrm{Oh} = \sqrt{\mathrm{We}}/\mathrm{Re}$. The inverse Ohnesorge number, $Z = 1/\mathrm{Oh} = \sqrt{\gamma\rho\alpha}/\eta$, where γ, ρ, α and η are the surface tension (mN m^{-1}), density (g cm^{-3}), nozzle diameter (μm) and viscosity (mPa s), respectively, has been shown to be more convenient for predicting jetting stability.[132] As a rule of thumb, a Z-value between 1–14 is considered as the range within which inks may exhibit stable jetting; >1 to prevent the formation of long fluid ligaments and <14 to prevent the ejection of secondary or "satellite" drops.[128] The surface tension of the ink also has to be sufficiently low to achieve good wetting of the substrate, facilitating uniform deposition. If the surface tension is too high, the wettability of the ink on the substrate decreases and the droplet beads, leading to discontinuous deposition.[108]

For AJP, no such FOM to directly link the properties of the ink with the quality of aerosol generation and deposition currently exists as there is insufficient understanding of the process. A key challenge for AJP is developing the ability to control the evaporation of the ink during atomisation, transport and deposition. Low bp solvents evaporate during the transport phase, using them alone can lead to the deposition of discrete, dry particles leading to a large surface roughness.[115] The loss of mass during transportation can also lead to other unwanted factors which make the process less efficient. To achieve flat features, a thin liquid layer needs to be deposited, which effectively dries by a combination of the sheath air flow

Table 2 Properties of the common solvents[130]

Solvents	Density (g cm^{-3})	Boiling point (°C)	Evap. rate (Bu Ac = 10)	Surface tension (mN m^{-1} at 25 °C)	Viscosity (mPa s)
Ethanol	0.789	78.0	33.0	21.97	1.074 (20 °C)
Ethyl acetate	0.902	77.0	62.0	26.29	0.426 (25 °C)
Ethoxy propanol	0.900	132.0	4.9	n/a	2.00 (20 °C)
Isopropyl alcohol	0.786	82.6	29	22.90	2.86 (15 °C)
Ethylene glycol	1.113	197.3	n/a	50.21	n/a
Butanol	0.810	117.7	0.46 (Bu Ac = 1.0)	27.18	2.95 (20 °C)
2-Butanol	0.806	99.0	n/a	n/a	4.20
N-Methylpyrrolidone	1.028	202.0	n/a	33.70	1.67 (20 °C)
N,*N*-Dimethylformamide	0.944	153.0	n/a	36.42	0.802 (20 °C)

 This journal is © The Royal Society of Chemistry 2020

Published on 17 February 2020. Downloaded by NANJING TECH UNIVERSITY on 2/1/2025 6:08:03 AM.

and substrate heating.[133] This can be achieved in part by using a combination of low and high bp solvents.[113] The low bp solvent evaporates relatively quickly after atomisation, saturating the carrier gas which stabilises droplet volume during transport to the nozzle. Interaction with the sheath gas causes the high bp solvent to begin to evaporate by which point, the liquid film can form on the substrate. A proportion of 10% of high bp solvent is often sufficient to prevent the total drying of the aerosol droplets before deposition.[115]

In theory, any material that can be suspended within an aerosol is compatible with AJP. Depending on the method of atomisation, the viscosity of the parent ink is limited. The advantage of ultrasonic atomisation is that it forms highly uniform aerosols. However, it can only be used for inks with viscosities of up to 10 mPa s. Pneumatic atomisation enables the aerosolization of a wider range of inks, viscosities of up to 1000 mPa s, but with inferior uniformity between the aerosol droplets formed.

Screen printing inks must be shear thinning. The ink flows when shear forces are exerted from the squeegee, and ceases to flow when the squeegee and accompanying shear forces are removed; the deposited pattern then holds the shape it is deposited in. To achieve the high initial viscosity, a much higher proportion of binder is required, with use of solvents that have a sufficiently high bp (Table 2) to enable sufficient dwell time on the screen. Ethyl cellulose in terpineol has emerged as a staple binder-solvent system for functional screen printable inks.[121,134]

Gravure and flexographic inks have similar requirements in that they need to be mobile, have lower viscosities and dry rapidly. This allows the ink to fill the cells of the gravure and anilox roller respectively, whilst maintaining the fluidity to transfer from the roller to the substrate effectively.[123,124]

Adjustments to the ink surface tension and viscosity can be implemented in both these two printing methods to minimise the possibility of cohesive–adhesive failure between the roller/print cylinder and substrate, maximising the amount of ink printed on the substrate.[135]

In this section, we have discussed different printing techniques that can be utilised for the fabrication of functional devices with the ink requirements for each of the techniques outlined. The following sections will give examples of gas sensors fabricated using these printing methods.

3. Printed metal oxide nanomaterial gas sensors

The earliest demonstration of the use of metal oxides for gas sensing dates back to the 1960s, when Dimbat *et al.* found that the adsorption or desorption of ethanol molecules on the surface of a ZnO thin film changed its electrical conductivity.[136] Since then, a wide variety of metal oxides including SnO_2,[137,138] TiO_2[139,140] and WO_3[141,142] have been explored in gas sensing, particularly based on the chemiresistive configuration.

3.1 Sensing mechanisms for metal oxide gas sensors

The sensing mechanism of semiconductor-based electrical gas sensors is mostly attributed to gas adsorption induced charge transfer and doping.[68] The conductivity behaviour of various metal oxides is presented in Table 4 with Table 5 listing the metal oxides most suited to detection of specific gases.[143,144]

The majority charge carriers in an n-type semiconductor are electrons. Upon interaction with a reducing (electron donating) gas, its electrical conductivity increases.[145] Conversely, an oxidizing (electron withdrawing) gas tends to deplete the surface layer of electrons of the n-type semiconductor, resulting in a decrease in the film conductivity.[146] A p-type semiconductor, on the other hand, conducts with holes as the majority charge carriers, and thus shows an increased conductivity in the presence of an oxidizing gas and a decreased conductivity in the presence of a reducing gas.[147]

Under ambient conditions, most semiconducting metal oxides interact with gas molecules physically, and their conductivity changes due to the gas-induced charge doping as mentioned above. At elevated temperatures (typically >150 °C), metal oxides become more conductive due to the increased carrier concentration;[148,149] and in the presence of air, highly reactive oxygen molecules can exchange electrons with the metal oxide (reactions (1)–(3)) and stay adsorbed in the form of oxygen anions, such as O_2^-, O^- and O^{2-}.[150,151]

$$O_2\ (\text{adsorbed}) + e^- \Leftrightarrow O_2^-\ (<100\ ^\circ\text{C}) \tag{1}$$

$$O_2^- + e^- \Leftrightarrow 2O^-\ (100\text{–}300\ ^\circ\text{C}) \tag{2}$$

$$O^- + e^- \Leftrightarrow O^{2-}\ (>300\ ^\circ\text{C}) \tag{3}$$

Taking an n-type metal oxide as an example (Fig. 6), the oxygen anions take away electrons, resulting in the formation of an electron depletion layer. When exposed to reducing gas molecules (Fig. 6a), the surface adsorbed oxygen anions react with the oxidizing gas molecules and release electrons back to the metal oxide core, resulting in a thinner depletion layer and an increased film conductance. On the other hand, an oxidizing gas, acting in a similar way, can take away additional electrons, resulting in a wider depletion layer and an increased film resistance (Fig. 6b).

The thickness of the aforementioned charge depletion layer (L) is proportional to the material Debye length (L_D) by

$$L = L_D(2eV_s/kT)^{1/2} \tag{4}$$

where eV_s and kT are surface potential and thermal energy, respectively.[152] The Debye length is a material property used to

Table 4 Conductivity behaviour of selected metal oxides[143,144]

n-Type	p-Type	n,p-Type
SnO_2, ZnO, TiO_2, In_2O_3, MoO_3, MgO, Al_2O_3, Ga_2O_3, Nb_2O_5, ZrO_2, CaO, V_2O_5, Ta_2O_5, WO_3, $SrTiO_3$, $SrTiFeO_3$	NiO, CeO_2, Mn_2O_3, Co_3O_4, La_2O_3, Y_2O_3, PdO, Ag_2O, Bi_2O_3, Sb_2O_3, TeO_2, CuO, Cr_2O_3, $Cr_{2-x}Ti_xO_{3+z}$	Fe_2O_3, HgO_2

Published on 17 February 2020. Downloaded by NANJING TECH UNIVERSITY on 2/1/2025 6:08:03 AM.

Table 5 Selected list of preferred metal oxides for applications in specific gases[144]

Detected gas	Metal oxides preferable for application
Reducing gases (CO, H_2, CH_4)	SnO_2; $Cr_{2-x}Ti_xO_{3+z}$; Ga_2O_3; In_2O_3
Oxidizing gases (O_3, NO_x, Cl_2)	In_2O_3; WO_3; ZnO; TiO_2
H_2S, SO_2	SnO_2/CuO; SnO_2/Ag_2O
NH_3	WO_3; MoO_3; In_2O_3
CO_2	SnO_2/La_2O_3; Al_2O_3/V_2O_5
Alcohol	La_2O_3/In_2O_3; La_2O_3/SnO_2; In_2O_3/Fe_2O_3
Oxygen	Ga_2O_3, $SrTiO_3$, $SrTiFeO_3$; TiO_2; Nb_2O_5; ZnO

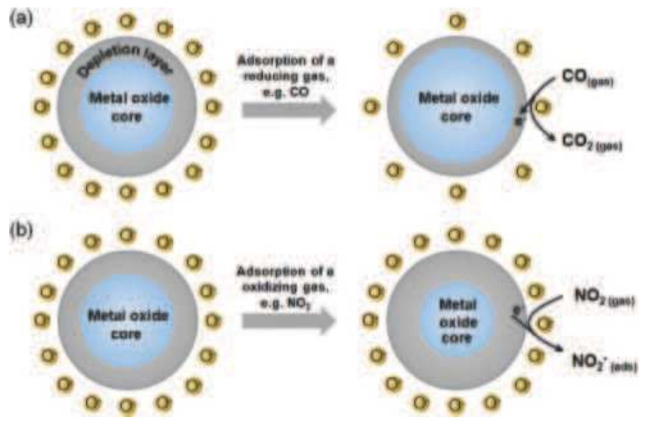

Fig. 6 Schematic illustration of the change of depletion layer of an n-type semiconducting metal oxide with surface adsorbed oxygen anions in response to (a) a reducing gas and (b) an oxidizing gas.

indicate the scale over which mobile charge carriers screen out electric fields in semiconductors and can be calculated by the following equation:

$$L_D = (kT\varepsilon/q^2N_D)^{1/2} \quad (5)$$

where k, T, q, ε and N_D are the Boltzmann constant, absolute temperature, electron charge, dielectric constant ($\varepsilon_0 \cdot \varepsilon_r$) and the net density of dopants.[153] These two equations indicate that the Debye length depends mainly on the net density of dopants. For a typical metal oxide semiconductor with a dopant density ranging from 10^{15} to 10^{17} cm^{-3}, L_D is in the range of a few to tens of nanometers.[152,154] Therefore, nanostructured metal oxides are particularly attractive since a gas-adsorption induced depletion layer can develop in a considerably large portion of the whole crystal to provide maximized electrical response. Further examples will be provided in Section 3.3.

3.2 Types of printing and ink formulation

For FET sensors, the source and drain electrodes are usually pre-fabricated by conventional photolithography in combination with physical or chemical vapor deposition.[155–157] Chemiresistive sensors are less demanding in device complexity and feature resolution. Therefore, in addition to the conventional nano/micro-fabrication processes, interdigitated electrodes for these sensors can be directly printed, for example, with Ag or Au inks.[158–160] The sensing materials can then be deposited through inkjet,[161,162] EHD,[163] aerosol jet,[164] screen,[165] or gravure[74] printing between the source and drain electrodes for FET sensors and across the interdigitated electrodes for chemiresistive sensors (Table 6).

A metal oxide ink is a mixture of metal oxide nanocrystals, solvent and sometimes, additives to adjust the printability and stability of the ink against aggregation/sedimentation. Metal oxide nanocrystals are commonly synthesized by sol–gel and solvothermal methods in polar solvents by using precursors such as metal halides, metal nitrates, metal acetates and metal alkoxides.[173] In most cases, as-prepared metal oxide nanocrystals carry negative surface charges in the form of OH^-.[174,175] The resulting product solutions can be directly used as inks without further modification. For example, Shen *et al.* prepared an SnO_2 based ink for inkjet-printed H_2S sensor through direct hydrolysis of an $SnCl_2$ precursor in anhydrous ethanol.[168] Alternatively, pre-synthesized metal oxide nanocrystals are collected and re-dispersed in a target solvent depending on the printing type and specific ink requirement.[176,177] Treatments such as surface

Table 6 Selected examples of printing methods and sensing performances of various printed metal oxide gas sensors

Target gases	Materials	Printing techniques	Substrates	Electrodes/printing techniques	Sensitivity	Temperature (°C)	Flexibility	Sensor type	Ref.
NO_2	ZnO	Inkjet	Si/SiO_2	Ag/Au	44% at 1 ppm	RT	No	Chemiresistive	166
	ZnO	Inkjet	Al_2O_3	Pt	95% at 1 ppm	150	No	Chemiresistive	167
	WO_3/PEDOT:PSS	Gravure	PI	Ag ink/gravure	14% at 50 ppb	RT	Yes	Chemiresistive	74
	SnO_2	Inkjet	PI	Au ink/inkjet	20% at 0.6 ppm	200	Yes	Chemiresistive	161
	SnO_2	EHD	Si/SiO_2	Au	60 at 2 ppm	300	No	Chemiresistive	163
	In_2O_3	EHD	Si/SiO_2	Au	55 at 2 ppm	300	No	Chemiresistive	163
	WO_3	EHD	Si/SiO_2	Au	125 at 2 ppm	300	No	Chemiresistive	163
	NiO	EHD	Si/SiO_2	Au	45% at 2 ppm	300	No	Chemiresistive	163
	SnO_2	Inkjet	Si/SiO_2	Au	95% at 50 ppm	375	NO	Chemiresistive	168
EtOH	TiO_2	Screen	PET	Ag ink/screen	97% at 100 ppm	150	Yes	Chemiresistive	165
	SnO_2	Screen	Si/Al	Au ink/screen	83% at 10 ppm	400	No	Chemiresistive	169
	SnO_2	Inkjet	PET	Ag	20% at 7.7%	RT	Yes	Chemiresistive	170
	SnO_2	Inkjet	Polycarbonate	Au	75% at 1000 ppm	200	Yes	Chemiresistive	171
NH_3	Zn_2GeO_4	Electrospinning	PI	Ag	189% at 10 ppm	RT	Yes	FET	10
O_2	Pt/In_2O_3	Inkjet	Si/SiO_2	Cr/Au	10% at 30%	200	No	Chemiresistive	156
HCHO	In_2O_3	Screen + Micro-injection	Al_2O_3	Au ink/screen	83% at 1 ppm	300	No	Chemiresistive	172

 This journal is © The Royal Society of Chemistry 2020

Published on 17 February 2020. Downloaded by NANJING TECH UNIVERSITY on 2/1/2025 6:08:03 AM.

modification,[178] ultrasonication,[179] and ball milling[180] can be employed to further improve their dispersion and consistency without the use of binders or additives. As mentioned before, binder free inks (or post deposition binder decomposition) for gas sensors are desirable for a number of reasons, including increased accessible surface available for analyte–material interaction, and improved conductivity due to the absence of insulating polymers.[181] However, if pure solvent based inks are not optimally formulated, performance reliability of the printed devices could be affected. For example, Kukkola *et al.* used metal-decorated WO_3 active material for gas sensing, which exhibited significant coffee-ring effect.[182] With additional layers, a continuous, yet non-uniform print was achieved. Such lack of consistently between prints could affect the device-to-device performance variation, and hence, the manufacturability. To address this for pure solvent based inks, formulations with mixed solvent systems could be used.[183] For example, Hagen *et al.* dispersed hollow SnO_2 spheres in water with added EG.[162] EG can also be used to reduce rapid evaporation of volatile solvents, which otherwise, may cause nozzle blockage.[161]

Polymers and surfactants such as polyvinyl pyrrolidone (PVP) can be added to reduce the surface energy of metal oxide nanomaterials for enhanced ink consistency and stability.[161,162,184] For example, Perez *et al.* prepared an ink by mixing CuO colloidal suspension and a ZnO sol with added PVP.[184]

As mentioned in Section 2, while fluids with low viscosity are desired by inkjet printing, highly viscous, shear thinning inks are needed for screen printing. The sol–gel method, as compared with other wet chemical synthesis methods, is capable of producing sols with relatively high particle concentration and high viscosity with shear thinning behavior.[185] Alternatively, polymers can be added to inks to raise their viscosities. For example, Georges *et al.* added PVP to the terpineol dispersion of TiO_2 nanopowder to make it viscous enough to fabricate a screen printed ethanol sensor.[165]

3.3 Improving sensor performance through device and materials design

3.3.1 Device design. Most chemiresistive metal oxide sensors operate at relatively high temperatures (*e.g.* 150–450 °C) for optimizing their sensitivity and selectivity.[68] This requires integration of a Joule heating element. There are four predominant heater configurations. In the first configuration (Fig. 7a and b), while the electrodes and sensing film are printed on the top of the substrate, the heater is printed on the back of the substrate. Commercial Au[161] and Pt[186] inks/pastes are commonly utilized to print heaters. SiO_2[187] and Si_3N_4[188] are commonly used substrate materials that are electrically insulating and thermally conductive. However, this configuration can result in relatively high power consumption due to the heat loss across the thick substrate. This drawback can be addressed using complementary metal oxide semiconductor (CMOS) compatible micro-electro-mechanical system (MEMs) fabrication processes to thin the substrate supporting the interdigitated electrode structures (Fig. 7c and d).[129,189] Guha *et al.* illustrated this concept on a Si_3N_4 membrane, with a buried tungsten microheater and an integrated heat spreader for good thermal uniformity across the exposed gold interdigitated electrodes.[189] This design allows for highly controlled temperature modulation with greatly reduced power consumption for inkjet printed NH_3, acetone[129] and humidity[191] sensors on 1 mm × 1 mm chips.

In the third configuration (Fig. 7e and f), the heater is printed on the same side with the electrodes, and close to the sensing material such that heat can be transferred through both air and the substrate.[172] However, having the heater and electrodes on the same side of the substrate increases the planar size of the device and typically results in non-uniform heating of the sensing layer. As an alternative, Ramíreze *et al.* came up with an interesting planar configuration involving only two printed electrodes (Fig. 7g and h). One of the electrodes are designed

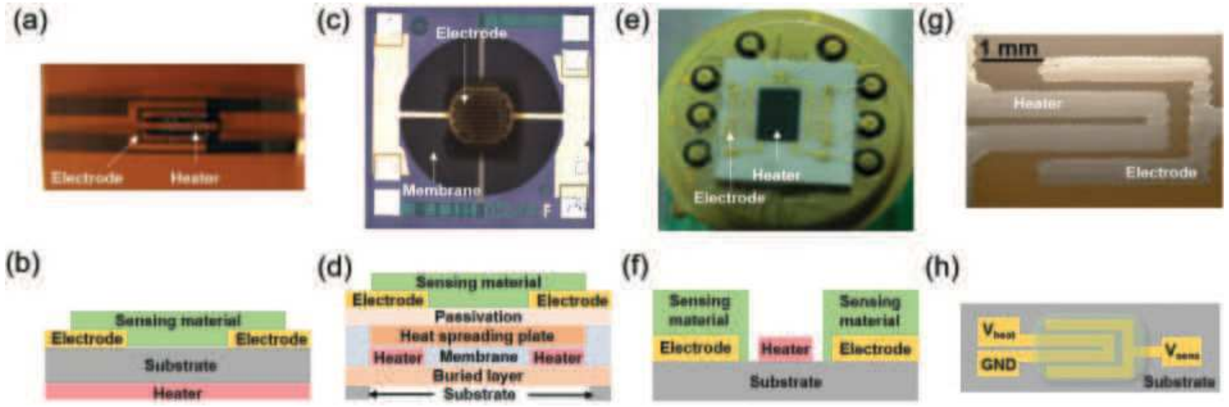

Fig. 7 (a) Top-view photograph and (b) side-view schematic illustration of an inkjet-printed CO sensor, in which the heater and electrodes on the opposite sides of the substrate.[161] Reproduced from ref. 161. Copyright 2017, Elsevier Ltd. (c) Top-view photograph and (d) side-view schematic illustration of an inkjet-printed NH_3 sensor, in which the heater is embedded within a membrane.[129] Reproduced from ref. 129. Copyright 2019, Nature Publishing Group. (e) Top-view photograph and (f) side-view schematic illustration of a screen-printed HCHO sensor, in which the heater and the electrodes are on the same side of the substrate.[172] Reproduced from ref. 172. Copyright 2013, Elsevier Ltd. (g) Top-view photograph and (h) Top-view schematic illustration of an inkjet-printed H_2 sensor, based on a two-electrode configuration in which one of the electrodes also acts as the heater.[192] Reproduced from ref. 192. Copyright 2017, Elsevier Ltd. Schemes in (a), (c), (e), and (g) are not drawn to scale.

Published on 17 February 2020. Downloaded by NANJING TECH UNIVERSITY on 2/1/2025 6:08:03 AM.

to have two contacts; one contact is grounded (GND) and the other is biased (V_{heat}) to generate heat. Simultaneously, this electrode also assists in draining the current flowing through the sensing material under the applied potential difference between the two electrodes (V_{sens}).[192] This method avoids using a dielectric layer between the heater and the electrodes, and reduces the amount of expensive metallic inks required for patterning heater and electrodes.

The challenges facing many gas sensors, including low precision and poor cross-selectivity, can potentially be overcome by using sensor arrays based on different sensing materials. The basic principle is that in response to a target gas, a sensor array can output a set of signals that form a unique fingerprint for that particular gas. For example, Kang *et al.* produced several types of metal oxide (SnO_2, In_2O_3, WO_3 and NiO) nanofibers by electrospinning, and then fragmented them into smaller pieces by ultrasonication. These were dispersed in alpha-terpineol or EG solvents as inks for further EHD printing (Fig. 8a–c).[163] Since each sensing material responded differently toward different target gases, it is possible to discriminate between them by using PCA as briefly discussed in Section 1.3.

Combining gas sensing capability with other functions in a single device can be realized through appropriate device design and layer-by-layer integration. Recently, a self-powered wearable device with printed interconnects, gas sensor, and supercapacitors along with embedded solar cells was demonstrated by Lin *et al.* with inkjet printing.[170] As shown in Fig. 9, Ag interconnects and SnO_2 sensor for ethanol/acetone detection were printed on PET substrate as the first layer. On top of this, MnO_2-based planar supercapacitors were then printed. This example demonstrates that a wide variety of wearable and portable devices with multiple functional components can be achieved with additive printing techniques, especially for personalized healthcare and biomedical applications.

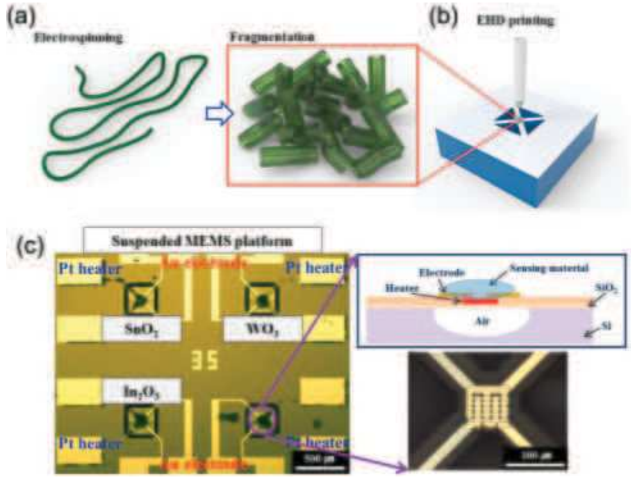

Fig. 8 (a) Metal oxide nanofiber fragments are prepared by electrospinning and fragmentation process (*e.g.* ultrasonication). (b) highly integrated gas sensor array is fabricated by micro-patterning of heterogeneous metal oxide nanofibers *via* sequential or parallel EHD printing process. (c) MEMS gas sensor array fabricated by EHD printing of SnO_2, WO_3 and In_2O_3 nanofibers for low power consumption. The platform size is 3.5 mm × 3.5 mm.[163] Reproduced from ref. 163. Copyright 2017, Elsevier Ltd.

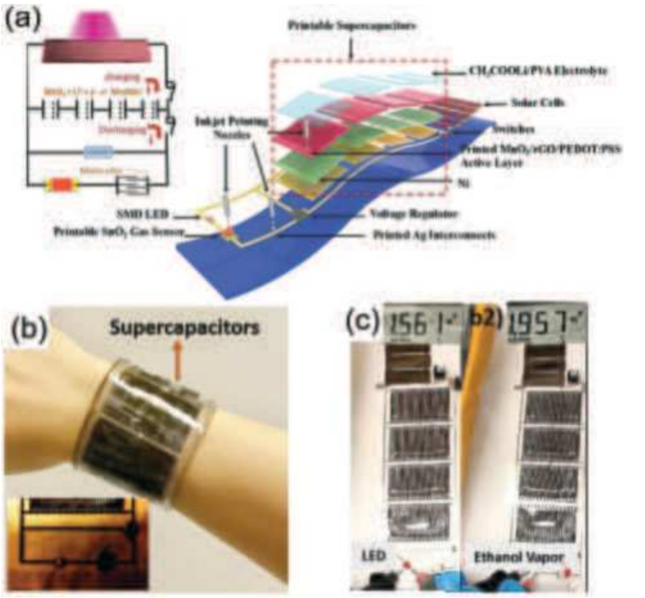

Fig. 9 (a) Schematic images of a flexible and wearable monolithically integrated self-powered smart sensor system on plastic substrate. The printing techniques and operational mechanism are also shown (b) Photo of a wearable wristband. Inset: LED as the indicator of gas detection. (c) Photos of voltage rise on LED without (left) and with (right) detection of ethanol in ambient. Insets: Voltage values read on multimeter.[170] Reproduced from ref. 170. Copyright 2019, John Wiley & Sons, Inc.

3.3.2 Materials design. The sensing performance of a metal oxide material can be influenced by several factors. These can be categorized into two groups. The first group of factors includes their microstructural properties, such as shape, size, and porosity.[163,193,194] The second group of factors relates to materials chemistry, such as surface decoration, type of doping, and formation of heterostructures.[72,195,196]

Increasing the gas–solid interaction surface is one of the most straightforward strategies to improve the sensor performance, and can be achieved by reducing the particle size of the sensing material.[197,198] The size effect was systematically investigated by Ansari *et al.*, who found that an H_2 sensor based on 20 nm SnO_2 particles was about 10 times more sensitive than that based on 25–40 nm particles.[199] A reduced particle size can also lead to an increased relative size of the charge depletion layer upon gas adsorption.[200] As discussed in Section 3.1, when the particle/grain size becomes comparable to the Debye length of that particular material, the charge depletion region can cover almost in the entire crystal, allowing maximization of the sensing response.[197]

Similarly, one-dimensional materials with diameters comparable to the Debye length (a few to tens of nanometers) are also potentially capable of achieving a high sensor response.[201] Various synthesis techniques including the solvothermal method,[202] vapor–liquid–solid method,[203] thermal evaporation[204] and electrospinning[163] have been employed to prepare 1D metal oxide nanostructures for gas sensing. However, direct printing can only generate a film of randomly distributed nanowires as indicated in the schematic in Fig. 8. In an effort to control the

Published on 17 February 2020. Downloaded by NANJING TECH UNIVERSITY on 2/1/2025 6:08:03 AM.

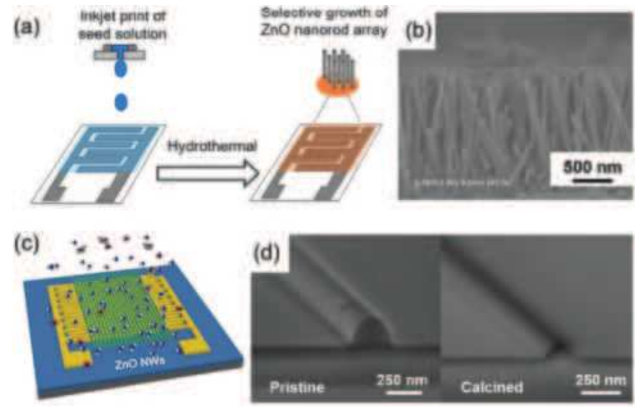

Fig. 10 (a) Schematic illustration for the selective growth of vertically aligned ZnO nanorod arrays on a sensor substrate by ink-jet printing of seed solutions and hydrothermal process. (b) FESEM images of the cross-section of ZnO nanorods. Scale bar, 500 nm.[167] Reproduced from ref. 167. Copyright 2010, Elsevier Ltd. (c) Schematic illustration of highly-aligned ZnO wires gas sensor. (d) SEM of pristine ZnO nanowire and calcined ZnO nanowire. Scale bar, 250 nm.[166] Reproduced from ref. 166. Copyright 2019, Elsevier Ltd.

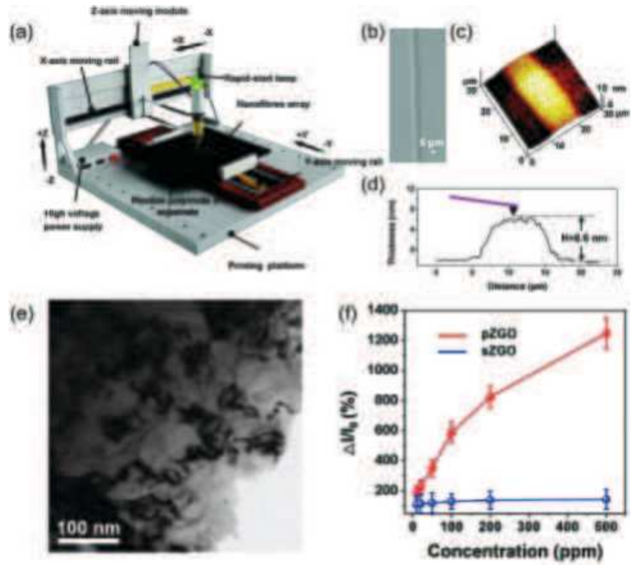

Fig. 11 (a) Schematic diagram of printing setup. Inset image shows the process of electrospinning technique. (b) SEM images of polycrystalline Zn_2GeO_4 (pZGO) wires on flexible PI substrate. (c) AFM topography and (d) height profle analysis of pZGO and (e) TEM image of pZGO. Scale bar, 100 nm. (f) Sensitivity plots of pZGO and single-crystalline Zn_2GeO_4 (sZGO) wires sensors to 10–500 ppm ammonia gas molecules (n = 5 measurements).[10] Reproduced from ref. 10. Copyright 2018, John Wiley & Sons,Inc.

orientation of printed nanowires, Chang *et al.* first ink-jet printed a ZnO seed ethanolic solution onto an interdigitated Pt electrode. The authors then placed the modified electrode in an aqueous solution containing $Zn(NO_3)_2$ and hexamethylenetetramine to allow the seeds to grow hydrothermally into vertically aligned ZnO nanorods (Fig. 10a and b).[167] Such a nanorod-array structure with largely exposed surfaces favored the adsorption and desorption of NO_2 molecules, enabling rapid response and recovery kinetics. In addition, the exposed active facets appear to be more conducive to reduce the surface activation barrier, promoting charge transfer. Besides the vertically oriented nanowire arrays, horizontally aligned nanowires were prepared by Kim *et al.* by using a home-built EHD printer. The authors printed a blended solution of PVP and $Zn(CH_3COO)_2{\cdot}2H_2O$ into arbitrarily-long and continuous solid-state wires with computer-controlled position and alignment (Fig. 10c).[166] The polymer components were then removed by high temperature decomposition, during which aligned and porous ZnO nanowires were obtained (Fig. 10d). The thus-prepared ZnO wire-based NO_2 sensor exhibited 40 times higher response and faster recovery time compared to a densely packed film of ZnO nanoparticles produced from the same printing solution. This can be attributed to the largely exposed surfaces of the well separated and porous wire structures.

For bulk or polycrystalline materials, when the grain size is much larger than the Debye length, most of the volume of the grain is unaffected by surface interaction with the gas molecules. In this case, the effect of gas adsorption on the charge transport across the grain boundaries (GBs) becomes the dominant factor for the conductivity modulation of the sensor. In a model proposed by Xu *et al.*,[194] abundant trap states could exist at GBs between large aggregates of crystallites. In addition, dangling bonds at GBs could also form deep defect states. Both of these could markedly impact gas adsorption and charge transfer kinetics in the sensing film. This was experimentally demonstrated by Wang *et al.* who printed a precursor solution containing $Zn(CH_3COO)_2{\cdot}2H_2O$, $[Ge(OEt)_4]$ and PVP on a flexible PI substrate by combining electrospinning and 3D printing, followed by calcination to produce polycrystalline Zn_2GeO_4 wires (pZGO) with abundant GBs (Fig. 11a–e).[10] The pZGO sensor exhibited an ultrahigh sensitivity toward NH_3 (598 at% 100 ppm) at room temperature, over $\sim$6.5 times higher than that of a single-crystal Zn_2GeO_4 wire (sZGO) sensor (Fig. 11f). Furthermore, the pZGO wires also showed better mechanical flexibility than the sZGO wires. The authors suggested that this was due to the reduced slip and dislocation motion at GBs which reduced the Young's Modulus, enhancing the flexibility of the wires.

Apart from reducing the particle size, creation of porous structures also provides large specific surface areas to enable enhanced gas adsorption and diffusion. Creating porous metal oxides is usually achieved by template-assisted methods. Two kinds of templates are commonly used, namely, soft templates such as surfactants and block copolymers,[205,206] and hard templates such as nanoscale or mesoporous silica or carbon based materials.[207] For instance, Yi *et al.* combined screen printing and sacrificial template-assisted calcination to prepare In_2O_3 nanowire networks for gas sensing.[172] In a typical process, a CNT paste was first screen-printed onto the surface of an Au electrode, followed by micro-injecting InN_3O_9 solution onto the CNT template film. Further calcination of the film at 650 °C resulted in the removal of the CNT template and conversion of the InN_3O_9 to In_2O_3 nanowires (Fig. 12a and b).

Published on 17 February 2020. Downloaded by NANJING TECH UNIVERSITY on 2/1/2025 6:08:03 AM.

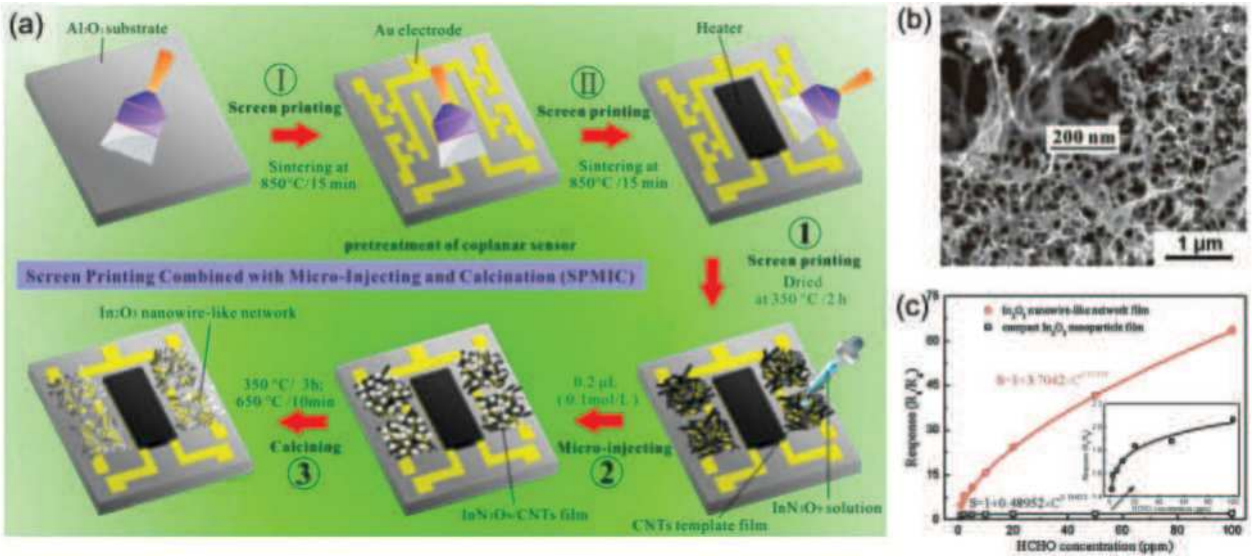

Fig. 12 (a) Schematic diagram depicting the procedure to prepare the porous In_2O_3 nanowire-like network. (b) SEM image showing the In_2O_3 nanowire-like network. (c) the response as a function of HCHO concentration at 300 °C.[172] Reproduced from ref. 172. Copyright 2013, Elsevier Ltd.

This porous network of nanowires allowed gas molecules to diffuse through the pores to be in full contact with the surfaces of the sensing material. This sensor exhibited a 30 times greater response toward formaldehyde compared to the sensor based on a compact In_2O_3 nanoparticle film; Fig. 12c.

For hollow materials with large inherent porosities, gas–solid interaction could take place on both their interior and exterior surfaces. In addition, the charge depletion layer could be built in almost the entire structure of a hollow material upon gas adsorption if the shell thickness is comparable to the Debye length, enabling an optimized sensing response.[162] McAleer *et al.* synthesized hollow SnO_2 nanospheres by coating a sol of partially hydrolyzed $[Sn(OBu^t)_4]$ onto carbon nanospheres and subsequent removal of the carbon nanospheres *via* calcination.[208] They then ink-jet-printed these SnO_2 hollow nanospheres to fabricate an ethanol sensor which showed a sensitivity of 3300% at 50 ppm, superior to a densely printed SnO_2 quantum dots film sensor with ~390% response to 1000 ppm ethanol.

The development of H_2 sensors with pure metal oxides has been challenging because H_2 is neither a strong electron donating nor withdrawing gas at ambient conditions. To address this, metal oxides have been doped with noble metal ions or nanoparticles to enhance their response towards H_2.[209–211] Printed H_2 sensors based on metal-modified metal oxides have been fabricated recently by screen printing[212] and inkjet printing.[182] The sensing mechanisms of these hybrid materials are dependent on the type of metal decoration. For example, Wen *et al.* prepared nanosized TiO_2 doped with Ag^+ ions by the sol–gel method, and screen printed the resulting paste on an aluminum substrate.[212] They achieved a significantly enhanced H_2 sensing response compared to the undoped TiO_2. Theoretical calculations revealed that new DOS peaks emerged near the Fermi level of the Ag^+-doped TiO_2 after H_2 adsorption, suggesting that the Ag-doping enhanced the electron exchange between H_2 and TiO_2. Kukkola *et al.* decorated WO_3 nanoparticles *via* wet impregnation with Pd and Pt, and directly inkjet-printed the supernatant solution without large precipitates to fabricate H_2 gas sensors.[182] The Pd–WO_3 gas sensor exhibited an ultrahigh response of 1000% per ppm H_2. This was attributed to the fact that Pd is capable of catalyzing the dissociation of adsorbed oxygen molecules into ionized oxygen species (*e.g.* O^- and O^{2-}) at elevated temperatures,[195,213] which in turn interacts selectively with H_2 through redox reactions.

4. Printed polymer and related composite gas sensors

Since the first demonstration of using polypyrrole (PPy) for NH_3 sensing in the early 1980s, other conducting polymers such as polyaniline (PANI), poly(3,4-ethylene-dioxythiophene):poly(styrene-sulfonate) (PEDOT:PSS), polythiophene (PTh), polyacetylene (PA) and their derivatives have been used as the active element in various gas sensing devices.[214–216] Compared to other gas sensing materials, conducting polymers have several advantages such as room temperature operation, ease of chemical modification, and enhanced mechanical flexibility.[217–219] Therefore, a great number of current generation gas sensors are based on conducting polymers and related composites (Fig. 13) (Table 7).

4.1 Sensing mechanisms of gas sensors based on conducting polymers

Depending on their chemical activities, gaseous analytes can either react chemically with polymers or interact with them *via* weak inter-molecular forces such as π–π interaction, H-bonding, and dipole–dipole interaction.[220,221] Upon gas adsorption, the electrical properties of conducting polymers can be modified *via* gas induced charge doping even at room temperature (Fig. 14a).[222] For example, when p-type PPy is exposed to a reducing gas like NH_3, electrons can be removed from NH_3 and transferred

 This journal is © The Royal Society of Chemistry 2020

Published on 17 February 2020. Downloaded by NANJING TECH UNIVERSITY on 2/1/2025 6:08:03 AM.

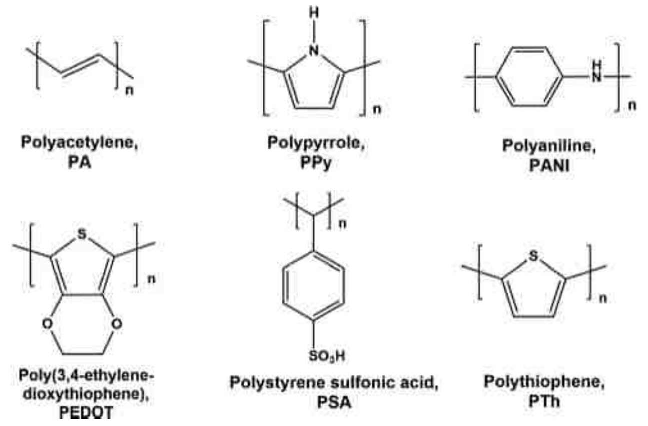

Fig. 13 Examples of conducting polymers used for gas sensors.

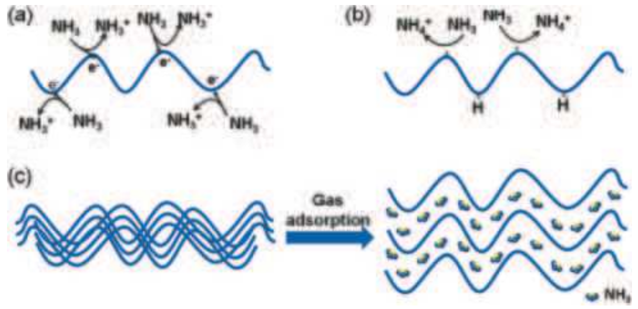

Fig. 14 Schematic illustration of sensing mechanisms for conducting polymers *via* (a) charge doping, (b) de-protonation and (c) swelling.

to the aromatic rings of PPy, increasing its resistance.[223] In addition, proton doping/de-doping *via* acid/base reactions have also been proposed to explain certain gas sensing phenomena with conducting polymers (Fig. 14b).[224–226] For instance, p-type PANI can be proton-doped *via* reactions with acidic gases, such as HCl and H_2S.[226,227] This converts its surface –NH– groups to $–NH_2^+–$, where the doped protons act as charge carriers to increase the film conductivity. Conversely, de-protonation of PANI by exposing it to NH_3 can turn its –NH– groups into $–N^-–$, and the resulting negative charges could further recombine with the holes in PANI; reducing the conductivity.[228]

Given the strong affinity of most VOCs towards polymers *via* various inter-molecular interactions and the abundant adsorption sites on polymeric chains, considerable uptake of VOC molecules in polymer films can be expected to cause structural changes. This, in addition to charge/proton doping, can lead to the modification of their electrical properties. For example, swelling of polymers induced by gas adsorption can enlarge their inter chain distance, which restrict the electron hopping between the chains, reducing the sensing film conductance (Fig. 14c).[229] In some cases, certain alcohols and ketones can strongly interact with the nitrogen atoms on a conducting polymer like PANI, causing its molecular chains to expand to a more stretched conformation, leading to an improved electron transport.[230]

The large uptake of VOC molecules in a polymer film can also cause its mass increase. For example, by recording the mass change of a poly(etherurethane) film with a quartz microbalance, different kinds of VOCs including ethanol and acetone can be detected.[231] Besides, this kind of mass measurement can be combined with resistance measurement to provide additional output signals. Examples include ethanol[232] and methanol[233] sensors based on both resistance and mass changes of PPy films.

4.2 Solution processability and ink formulation

Conventionally, conducting polymers have mostly been synthesized in the form of powders or films by using chemical or electrochemical polymerization methods.[237,238] Some of them can be dissolved in organic solvents to form homogeneous inks. In dilute inks, polymeric molecules are isolated from one another with weak intermolecular interactions, while in concentrated inks/pastes, molecular chains are entangled with each other.[239] In this regard, polymer based solutions with a wide range of rheological properties are suitable for various kinds of printing methods, such as inkjet,[11,79,240] screen[217] and gravure.[74,235]

Polymer inks can also be prepared from dispersions of polymer nanostructures synthesized by template-assisted method,[241] interfacial polymerization,[242] micellar synthesis[235,243] and electropolymerization method,[244] which possess good colloidal stability and solution-phase processability. Further improved solubility has been observed in nanostructured polymers with side chains.[245–248] For example, Park *et al.* found that decylthiophenyl side chains can improve the solubility of polythiophene derivatives in chloroform.[248] Most of these polymer nanomaterial solutions can be directly used as inks, with some exceptions where solvent exchange may be required depending on the printing method.[235] Examples of printable polymer nanostructure solutions for gas sensing are aqueous dispersions of PANI nanoparticles for NH_3 sensing[234] and poly(3-hexylthiophene) nanoparticles and nanofibers dispersed in trichlorobenzene for detection of various VOCs.[236] Sometimes, the as-synthesized polymer structures show large size distributions, which may

Table 7 Selected examples of printing methods and sensing performances of printed conducting polymer gas sensors

Target gases	Materials	Printing techniques	Substrates	Electrodes/printing techniques	Sensitivity	Temperature (°C)	Flexibility	Sensor type	Ref.
NO_2	Polyaniline	Inkjet	PET	Ag ink/screen	11% at 10 ppm	80	Yes	Chemiresistive	234
NH_3	Polyaniline	Gravure	PET	Carbon	13% at 20 ppm	RT	Yes	Chemiresistive	235
	Polyaniline	Inkjet	NFC tag	Ag	225% at 5 ppm	RT	Yes	Capacitive	11
	PEDOT:PSS	Inkjet	Plastic	Ag paste	19.42% at 5 ppm	RT	Yes	Chemiresistive	236
CO	Polyaniline/SnO_2	Screen	Al_2O_3	Pt	53% at 25 ppm	30	No	Chemiresistive	185
VOCs	PDDT	Inkjet	—	—	—	—	No	Chemiresistive	79

Published on 17 February 2020. Downloaded by NANJING TECH UNIVERSITY on 2/1/2025 6:08:03 AM.

Table 8 Selected examples of printing methods and sensing performances of printed carbon nanotube gas sensors

Target gases	Materials	Print techniques	Substrates	Electrodes/pinting techniques	Sensitivity	Temperature (°C)	Flexibility	Sensor type	Ref.
NH_3	PABS-SWCNT	Inkjet	Paper	Ag ink/inkjet	50 ppm	RT	Yes	Capacitive	274
	SWCNT	Inkjet	Paper	Ag ink/inkjet	—	RT	Yes	Capacitive	275
NO_2	Functionalized CNT	Inkjet	Paper	Ag ink/inkjet	30% at 10 ppm	RT	Yes	Chemiresistive	276
	SWCNT	Aerosol jet	Si/SiO_2	HfO_2	96% at 60 ppm	RT	No	Chemiresistive	277
	$SWCNT/WO_3$	Screen	—	Au	400% at 100 ppb	250	No	Chemiresistive	278
	SWCNT	Inkjet	Si/SiO_2	Pt	5.7% at 100 ppb	RT	No	Chemiresistive	9
	Plasma treated SWCNT	Screen	Si/SiO_2	Au	19% at 1 ppm	130	No	Chemiresistive	279
H_2	Pt/SWCNT	Aerosol jet	Si/SiO_2	Cr/Au	0.5% at 20 ppm	80	No	Chemiresistive	164
EtOH	COOH/PEDOT:PSS-MWCNT	Inkjet	PET	Ag ink/inkjet	0.7% at 200 ppm	RT	Yes	Chemiresistive	280
HCHO	MWCNTs/PEDOT:PSS	Inkjet	—	Ag ink/inkjet	30% at 10 ppm	RT	Yes	Chemiresistive	281

cause non-uniformity in film printing. In such cases, the polymer solution may be filtered through a membrane to remove relatively large particles before ink formulation.[79] Additives/binders such as EG and Triton x-100 are commonly used to decrease the viscosity and surface tension of the ink to improve the print uniformity. Additionally, high boiling point EG is typically used to prevent rapid drying, and nozzle clogging.[249]

4.3 Strategies for improving sensor performance

To date, several strategies have been developed to improve the sensitivity, response time and stability of conducting polymer based gas sensors. These include designing specific surface structures,[236] modifying the polymer molecular structures,[79] and hybridizing them with other functional materials.[185]

4.3.1 Printing enabled control of film topology. One of the most important advantages of polymer printing lies in the fact that a homogeneous polymer solution, rather than dispersion of particles, can be deterministically printed into continuous, smooth structures which then solidifies as the solvent evaporates. This allows for the precise control over the surface topologies of a printed film.

For the case of inkjet printing, Li *et al.* demonstrated that by printing a PEDOT:PSS aqueous solution with controllable droplet spacings (D_s), adjacent droplets could be connected *via* narrow "neck" regions into line-like structures as illustrated in Fig. 15a and b.[236] The "neck" regions became more evident with increasing D_s values from 25 to 40 μm, and were correlated to an increased sensing response toward NH_3 (Fig. 15c). The authors proposed that given the p-type nature of PEDOT:PSS, a hole depletion layer was formed upon NH_3 adsorption. The volume ratio of the depleted layer was much larger in the thinner "neck" regions than in the wider droplet regions, leading to a more profound effect on modulation of the charge transport property of the film.

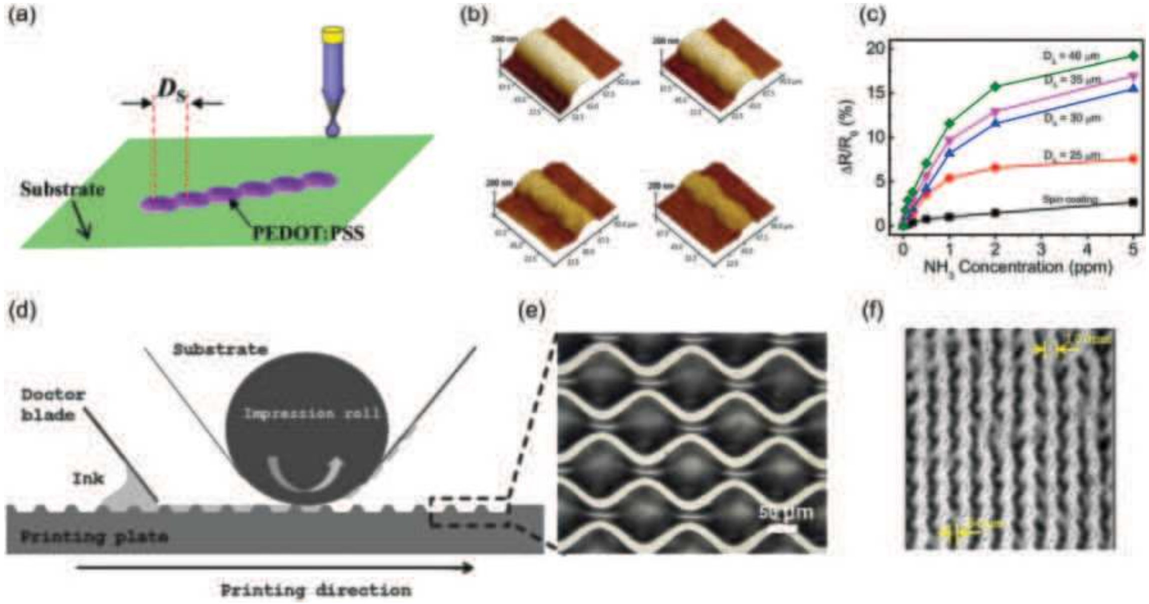

Fig. 15 (a) Inkjet printing PEDOT:PSS line structures with control of drop spacing (D_S). (b) Measured surface atomic force microscopy (AFM) images of the inkjet-printed PEDOT:PSS line structures with different drop spacing (25 μm, 30 μm, 35 μm, 40 μm). (c) Measured $\Delta R/R_0$ as functions of the NH_3 concentration for the different devices.[236] Reproduced from ref. 236. Copyright 2013, IEEE. (d) Schematic representation of the gravure printing process. (e) Representative engraved surface patterns. (f) The optical image of the printed strip-patterned WO_3-PEDOT:PSS layer.[74] Reproduced from ref. 74. Copyright 2015, Elsevier Ltd.

This journal is © The Royal Society of Chemistry 2020

Published on 17 February 2020. Downloaded by NANJING TECH UNIVERSITY on 2/1/2025 6:08:03 AM.

For gravure printing, creating surface patterns is feasible by using a printing plate with pre-designed surface features. For example, Lin *et al.* first gravure printed Ag electrodes and then dispersed WO_3 powders in a PEDOT:PSS solution to formulate a composite ink, and gravure-printed it on a PI substrate into well separated micro-scale strips for sufficient gas–solid interaction (Fig. 15d–f).[74] The PEDOT:PSS not only acted as a conductive matrix but also a binder to enhance the adhesion of the strips on the substrate and prevent them from cracking. The as-fabricated gas sensor exhibited an ultra-high sensitivity toward NO_2 gas, *i.e.* a responsivity of $\sim$16.1% at 50 ppb at room temperature.

4.3.2 Chemical modification. The rich chemistry of polymeric materials enables their rational design, synthesis and chemical modification for selective and sensitive gas sensing.[79,218,250–252] Through fabrication of sensor arrays, polymers with different chemical properties could be compared in parallel for investigation of their surface chemistry dependent sensing behaviors. For example, Li *et al.* synthesized various regioregular polythiophene (rr-PT) based conducting polymers/copolymers with different side chains, end groups or secondary side chains. They were deposited on concentric spiral electrodes with diameters of $\sim$200 μm.[79] By controlling the deposition sequence, position, and feature size, a sensor array consisting of 24 sensor elements in a 4 × 6 matrix was produced (Fig. 16a–c). It was found that among the various poly(3-hexylthiophene) (P3HT)-based polymers, P3HT-benzyl was particularly sensitive toward toluene and benzene but showed no response to hexane and cyclohexane (Fig. 16d and e). This was attributed to the strong van der Waals interaction between the benzene rings of the analyte and the benzyl end groups on P3HT. Such interaction could bring the polymer chains closer together, and reduce the charge hopping distance between them, increasing the electrical conductance.

4.3.3 Hybridization. Besides the modification of the surface functional groups, the combination of conducting polymers with other conductive or semiconducting materials has also been attempted to enhance their performance. By combining suitable semiconducting polymers and metal oxides, p–n junctions can be created. For example, Jian *et al.* deposited p-type PANI on screen-printed n-type SnO_2 nanoparticles to realize CO detection.[185] The electron depletion layer in SnO_2 due to adsorption of oxygen from air widened at the p–n junction. This wider depletion layer caused a larger resistance change upon switching the gas environment from air to the electron donating gas CO, leading to a largely improved sensitivity. The beneficial effect of the formation of p–n junctions has also been demonstrated in other hybrid systems such as the one combining n-type WO_3 and p-type PEDOT:PSS for enhanced NO_2 sensing.[74]

Due to their molecular nature, the conductivity of these polymers is still orders of magnitude lower compared to low dimensional crystalline carbon materials such as CNTs and graphene. Therefore, hybridization of polymers with them has been explored to improve the sensing film conductance, which can bring benefits such as a higher signal to noise ratio and faster response time.[253] Seekaew *et al.* fabricated an NH_3 gas sensor by ink-jet printing a mixture of graphene dispersion and PEDOT:PSS solution on a transparent substrate with pre-fabricated

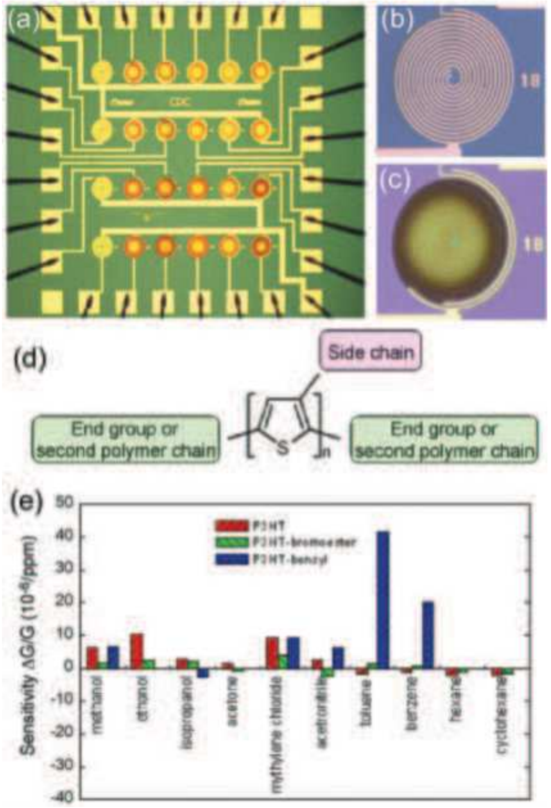

Fig. 16 Optical micrograph of sensor arrays. (a) Completed, wire bonded, test chip showing 24 electrode patterns with different inkjet printed polymers. (b) View of the gold spiral electrodes with no polymer. (c) Spiral electrodes with jetted poly(3-hexylthiophene) polymer formed from 10 drops of 5 mg mL^{-1} polymer concentration dissolved in trichlorobenzene. (d) Schematic diagram of regioregular polythiophene based polymers. (e) End group effects on response pattern.[79] Reproduced from ref. 79. Copyright 2006, Elsevier Ltd.

Ag electrode with screen printing.[249] The obtained sensor exhibited three times larger response and higher selectivity toward NH_3 compared to a sensor based on PEDOT:PSS alone. The graphene nanosheets embedded in the polymer matrix provided conductive pathways that favored electron hopping, enabling a low resistance at equilibrium. In addition, the NH_3-absorption induced swelling of PEDOT:PSS could not only increase its intermolecular chain distance but also disrupt the graphene conductive network. This in turn increased the sensor resistance to enhance the sensing response. Importantly, both polymers and graphene are ideal candidates for flexible devices. The obtained sensor could function under bending angles of up to 70°, and exhibited a trend of increased response at higher bending angles, suggesting potential use for wearable devices.

5. Printed carbon nanotube gas sensors

CNTs are known for their excellent electron transport properties, functionalizable surfaces based on the versatile carbon chemistry, and ultrahigh specific surface areas. They have been

Published on 17 February 2020. Downloaded by NANJING TECH UNIVERSITY on 2/1/2025 6:08:03 AM.

exploited in gas sensing as early as in the 1990s for practical applications and as a prototype for the fundamental investigation of gas–solid interaction on low dimensional sensing platforms.[254–257] CNTs are chiral structures that consist of either one single wall (single-wall carbon nanotube, SWCNT), or multiple concentrically nestled walls (multiwall carbon nanotube, MWCNT). SWCNTs can be either metallic or semiconducting, depending on their diameter and chirality.[258] Various CNT-based sensors have demonstrated high sensitivity toward gases like NO_2,[259,260] NH_3,[261,262] NO[263,264] and H_2,[265,266] as well as VOCs like ethanol[267,268] and formaldehyde.[269] The detection limit of a SWCNT-based NO_2 chemiresistive sensors has even reached the parts per trillion (ppt)-level.[270]

5.1 Sensing mechanisms

Gas adsorption induced charge transfer is the mostly accepted mechanism for CNT-based sensors. For example, on the basis of density functional theory (DFT) calculations, Chang and co-workers reported that every NO_2 molecule adsorbed on an SWCNT can withdraw approximately 0.1 electrons, whereas every adsorbed NH_3 molecule can donate 0.04 electrons.[271] Simulation results on gas induced charge transfer obtained by Zhao *et al.* suggested that gas molecules can diffuse through SWCNT bundles and adsorb on the interstitial sites for effective charge transfer.[272] We note that different proposals have also been put forward. For example, Kong *et al.* used the (10, 0) SWCNT as a model for first-principle calculations and found that there was no binding affinity between NH_3 and SWCNT.[254] They suggested that the commonly observed NH_3 sensing ability of a SWCNT device might arise from the interaction between NH_3 molecules and the hydroxyl groups on the SiO_2 substrate. This could partially neutralize the negative charges on SiO_2, indirectly influencing the electronic structure of the SWCNT. To experimentally investigate the working principle of CNT FETs, Zhang *et al.* covered the interfaces between the CNT and the Pd electrodes with a passivation layer in a way that only the middle section of the CNT was exposed.[273] This resulted in a much smaller and slower response of the device toward NO_2, indicating that the gas induced modulation of the potential barrier at the CNT channel/Pd junction could play a dominant role in the device performance. Another proposed mechanism is based on the fact that pre-adsorbed oxygen species on a SWCNT could interact with a target gas like NH_3 and in turn alter its electrical response.[254]

5.2 Types of printing and ink formulation

Different printing techniques including inkjet,[9,280,281] aerosol jet,[164] screen[278,279] and 3D printing[282] have been utilized to fabricate CNT-based gas sensors. Preparation of CNT dispersions is challenging due to the strong van der Waals interaction between CNTs and their tendency to orient themselves in parallel to each other to tightly assemble into bundles.[283] This can easily lead to agglomeration and sedimentation of the nanotubes. Such an unstable dispersion, when used as an ink for inkjet printing, may cause un-wanted nozzle clogging. Organic solvents can interact with CNTs *via* hydrophobic interaction and counter the attractive van der Waals forces between the nanotubes.[9,284] For example, dimethylformamide (DMF) and *N*-methylpyrrolidone (NMP), have well matched Hansen solubility parameters (HSPs) with the nanotubes, therefore these solvents are suitable to form a stable dispersion. However, these solvents have a high boiling point and are toxic which can make them challenging to print,[285] as the evaporation of the solvent and control of non-uniform deposition becomes non-trivial. It is also undesirable for large scale manufacturing as they bring considerable environmental and safety concerns. The nanotubes can be transferred to lower boiling point and less toxic solvents such as isopropyl alcohol, by sedimentation *via* centrifugation or filtration followed by re-dispersion. Although this reduces the duration of ink drying, it increases the chances of the nozzle clogging.

In this regard, aqueous CNT inks are desirable for ''green'' printing technology. High energy sonication can help disperse CNTs in water but exhibits poor stability and fast sedimentation over time.[286] Also, prolonged strong sonication can result in chemical and structural defects and even scission of the tubes.[287] Therefore a stabilising agent such as a polymer or surfactant is typically required to achieve a stable aqueous dispersion of CNTs. Alternatively, CNTs can be covalently or noncovalently modified before ink formulation. The covalent methods are usually based on treating the CNTs with strong oxidizing acids, such as H_2SO_4 and HNO_3. This creates hydrophilic oxygen-containing functional groups such as –OH and –COOH on their sidewalls.[276] Such acid treatments inevitably introduce surface defects, altering the electrical properties.[288] This may also impart desirable surface reactivity for gas sensing. Beside covalent surface modification, noncovalent functionalization of CNTs has also been attempted with polymers and surfactants, mostly *via* van der Waals forces or π–π interaction, thus avoiding significant degradation of the intrinsic properties of the CNTs.[289,290] For the case of polymer based functionalization, conducting polymers such as PEDOT:PSS and poly(*m*-aminobenzene sulfonic acid) (PABS) not only help disperse the CNTs but also contribute to the added sensing capability.[253,280] Similarly, surfactants such as Triton X-100 (polyethylene glycol-*tert*-octylphenyl ether) and poly(4-styrenesulfonic acid) solution (PSS, 18 wt% in H_2O), can act as wetting agents to reduce the surface tension of solvents like water.[291] This can also be achieved by mixing water with a solvent with lower surface tension, such as ethanol.[280] When an ink with high viscosity is required (for example, for screen printing), SWCNTs dispersed in organic solvent can be mixed with polymeric binders such as ethyl cellulose to produce high viscosity, shear thinning paste.[279]

3D printing and in particular, FDM has emerged as a powerful technique that is capable of producing complex architectures with a wide range of materials. As described in Section 2, it commonly works with a thermoplastic filament forced to extrude through a heated nozzle. The nozzle is capable of 3-axis movement. Thus, the filament coming out of it can be deposited layer-by-layer with controlled positioning to render a 3D object. CNT based sensing materials can be pre-mixed with a thermoplastic such as polyvinylidene fluoride

 This journal is © The Royal Society of Chemistry 2020

Published on 17 February 2020. Downloaded by NANJING TECH UNIVERSITY on 2/1/2025 6:08:03 AM.

(PVDF) and polylactic acid (PAA) in polar solvents and subsequently dried before extrusion at elevated temperatures. It was found by Kennedy *et al.* that the incorporation of relatively large amounts of MWCNTs in PVDF (up to 15 wt%) could reduce the extruded filament diameter without sacrificing the uniformity of the CNTs in the polymer matrix.[282] This indicates that deposition of finely patterned structures could be achieved while simultaneously incorporating the sensing capabilities of MWCNTs.

5.3 Improving sensor performance through materials design and optimization of fabrication processes

5.3.1 Materials design. Modification of bare CNTs through surface functionalization,[292,293] adatom doping,[294,295] and hybridization[296,297] can impart them with additional or enhanced sensing capabilities. As shown in Fig. 17, oxygen containing groups such as –COOH and –OH can be created on the side-walls and terminations of CNTs *via* acid or plasma treatment.[283,298–300] Other functional groups such as $-NH_3$ can also be created by reaction between chlorinated-MWCNTs and ethylene diamine.[301] These surface groups usually interact with a target gas more strongly as compared to bare CNTs, and can act as linkers to facilitate the charge transfer between the gas and CNTs. For example, good sensor performances have been achieved with COOH-SWCNTs/MWCNTs toward CO[302] and ethanol,[280] OH-CNTs toward NO_2,[276] and NH_2–MWCNTs toward formaldehyde.[301] However, the enhanced gas–solid interaction was usually accompanied with a slow gas desorption.[276] Nitrogen doped MWCNTs or boron-doped SWCNTs have exhibited significantly different physical and chemical properties compared to bare CNTs, notably with modified electronic structures and increased surface reactivities.[303,304] For instance, B-doping could create holes in SWCNTs, rendering them to p-type semiconductors for enhanced sensitivity toward electron-rich HCN molecules.[303] Besides, B-doped MWCNTs also exhibited a specifically strong affinity toward NO_2 due to the strong interaction between NO_2 and B dopants at carbon vacancies.[305]

Noble metal NPs (Pt,[86,306] Pd,[307,308] Au[309,310] and Ag[311,312]) have shown beneficial effects on the performance of CNT-based sensors. For example, Cui *et al.* found that MWCNTs decorated

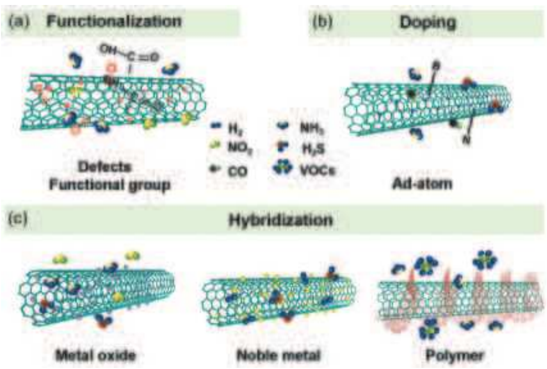

Fig. 17 Schematic illustration of strategies to promote sensing performance of CNTs *via* doping, surface functionalization and hybridization.

with Ag NPs showed not only a higher sensitivity but also a faster response to NO_2 compared to bare MWCNTs.[311] Based on DFT calculations, the authors attributed this to the formation of an NO_3 complex between NO_2 and oxygen on the Ag surface which mediated electron transfer from the Ag–MWCNT hybrid to NO_2. Also, Liu *et al.* found that Pt nanoparticles deposited on SWCNTs could bond to H_2 to form a PtH_x intermediate that bridged the electron transfer from H_2 to SWCNTs.[164] Au NP/SWCNT nanohybrids, on the other hand, were found to be particularly sensitive to H_2S. This was due to the Au–S covalent interaction which modified the Au electronic structure and thus its transport properties.[309]

Hybridization of CNTs with polymers[81,313] and metal oxides[314,315] have also been attempted for performance enhancement. In these hybrid systems, CNTs primarily act as conductive charge shuttles; while the metal oxides or polymers provide their respective sensing capabilities. Their related sensing mechanisms are discussed in Sections 3.1 and 4.1.

5.3.2 Device fabrication. Screen printing can be used to fabricate patterned films over large areas with a high throughput.[278,279,316–318] Without surface modification, thick CNT pastes can be used directly for screen printing, and post-printing treatments can be applied to improve the sensing film functionality. For example, Dong *et al.* screen printed mechanically dispersed SWCNTs onto Ti/Au electrodes to fabricate an FET-type gas sensor. The authors then plasma treated the devices to introduce surface defects and dopants using Ar or O_2.[279] The Ar plasma-treated SWCNTs showed an increase in defect sites due to ion bombardment during the treatment. The O_2 plasma-treated SWCNTs, on the other hand, exhibited not only increased defect sites but also carried abundant oxygen containing groups. Similarly, Evans *et al.* screen printed SWCNT–metal oxide (SnO_2 and WO_3) to fabricate highly sensitive NO_2 sensors.[278] Post-printing annealing at 400 °C helped to remove the surfactants and additives in the film and improve the crystallinity of the metal oxide particles.

With ink-jet printing, gas sensors can be fabricated on a wide variety of substrates, particularly those with high flexibility. For example, Alshammari and colleagues fabricated flexible ethanol sensors on PET substrates with inks containing either acid-treated MWCNTs or PEDOT:PSS-MWCNT mixtures.[280] On a Kodak photo paper, Lin *et al.* successfully fabricated an NO_2 sensor based on acid-treated MWCNTs.[276] High tolerance for substrates with inkjet printing was demonstrated by Huang *et al.*, who deterministically printed SWCNT/PABS on a radio frequency identification (RFID) tag to develop a wireless NH_3 sensor.[275] Recently, in an effort to reduce printing cost, Timsorn *et al.* fabricated an MWCNT-based formaldehyde sensor with a commercial inkjet printer (HP deskjet 2000 j210).[281] They printed a composite ink containing PEDOT:PSS and nitrogen-functionalized MWCNTs over a pre-screen printed Ag interdigitated electrode. If resolution is not a concern, home printers are attractive for quick evaluation of ink formulation and demonstration of device prototypes.

As mentioned in Section 2, AJP is a relatively new printing technology for electronics,[319] capable of producing ultra-small droplets with volumes of 0.0001–0.0005 pL, significantly lower

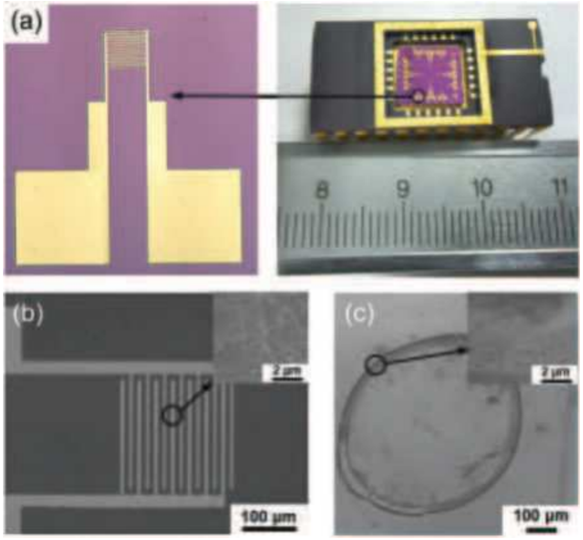

Fig. 18 (a) Optical image of electrode and test chip. Graph of SWCNT ink on the Si substrate (b) aerosol jet printing; (c) drop-casting.[164] Reproduced from ref. 164. Copyright 2019, IOP.

than that of common inkjet printers (1–80 pL).[164] On an array of interdigitated electrodes (Fig. 18a), Liu *et al.* AJP printed an ink of Pt-decorated SWCNTs on individual electrodes to prepare an H_2 sensor.[164] Because of the small droplet size and rapid evaporation of the small amount of water as solvent, non-uniform deposition due to coffee ring effect was suppressed considerably (Fig. 18b) compared to the film deposited with drop casting (Fig. 18c). The resulting homogeneous network of Pt-SWCNTs between electrodes enabled a good H_2 sensing performance with 1.5% response at 40 ppm.

6. Printed 2D material-based gas sensors

6.1 Introduction of 2D materials for gas sensing

2D materials, with thicknesses ranging from few to tens of nanometres and lateral dimensions reaching up to centimetres possess attractive physical and chemical properties with promising potential for electrically transduced gas sensing (Fig. 19).[34] The majority of these layered materials are held together by interlayer van der Waals forces in their bulk form. They cover a wide range of electronic properties, which can also dictate the most appropriate sensing architecture. Furthermore, the tuneable band gap and ability of 2D materials to act as a molecular scaffold for the immobilization of additional recognition components (*e.g.* metal/metal oxide nanoparticles) creates an avenue to further improve and tune their sensing properties.[320]

Graphene is one of the most widely studied 2D materials for gas sensing. It is an allotrope of carbon with a two-dimensional atomic layer arrangement of sp^2-hybridized carbon atoms covalently bound together in a honeycomb lattice. It is also the basic building block of several other carbon based allotropes such as graphite, CNTs and fullerenes.[34,321] The extraordinary electrical, physiochemical and structural properties of graphene such as a very high carrier mobility and carrier density, in addition to, a substantial theoretical specific surface area of $\sim$2600 $m^2\ g^{-1}$ and intrinsically low electrical noise, support the claim that graphene is one of the most promising materials for gas sensing applications.[322–326] However, despite of its high sensitivity, selectivity has always been an issue for graphene, which can be addressed by surface functionalization and hybridization.

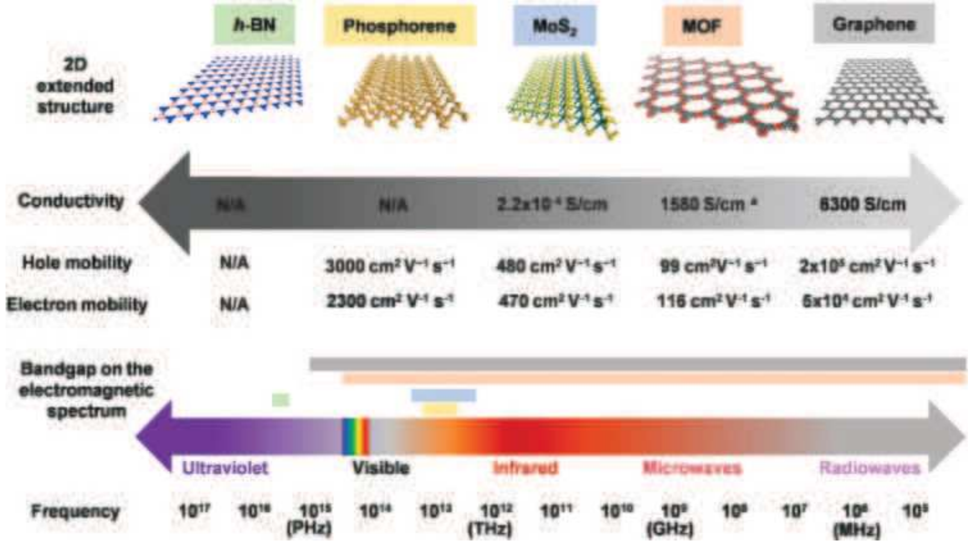

Fig. 19 Atomically thin 2D materials span a wide range of chemical structures, conductivities, carrier motilities, and bandgaps. Chemical structures of 2D materials have been discovered with both mixed (h-BN, 2D-MOFs, and MoS_2) and pure (black phosphorus and graphene) elemental structures. The electronic structure of 2D materials covers a wide range from insulating h-BN to the metallic conductivity of graphene. This wide range of properties exhibited by diverse 2D materials is the key to their success in sensing applications.[34] Reproduced from ref. 34. Copyright 2019 American Chemical Society.

Published on 17 February 2020. Downloaded by NANJING TECH UNIVERSITY on 2/1/2025 6:08:03 AM.

Published on 17 February 2020. Downloaded by NANJING TECH UNIVERSITY on 2/1/2025 6:08:03 AM.

Black phosphorus (BP) exists as a single-elemental layered crystalline material composed of sp^3 hybridized phosphorus atoms. Arranged in a layered orthorhombic crystal structure with the *Cmca* space group; it forms a honeycomb lattice structure with notable non-planarity in the shape of structural ridges.[34] The resulting 2D material exhibits unique properties, such as anisotropy in structural and charge transport behaviours, quasi-one-dimensional excitonic nature, and an intrinsic direct band gap covering from 0.3–2 eV.[34,327] A potential challenge that must be overcome for BP in gas sensing is its limited stability against light and oxygen, and the possible formation of phosphoric acid species on its surface, in the presence of moisture.[34]

Hexagonal boron nitride, also known as ''white graphene'', has a similar periodic structure in plane to graphene, where instead of hexagonally bonded carbon atoms, alternating boron and nitrogen pairs compose the hexagonal lattice structure. h-BN is an inorganic analogue of, and is isoelectronic to, graphene; but exhibits different stacking order.[328] However, due to the electronegativity differences between the boron and nitrogen atoms, the π electrons tend to localise around the nitrogen atomic centres, resulting in an electrically insulating material with high-temperature resistance against oxidation.[34,329] Pristine h-BN nanosheets have been rarely explored in gas sensing, but doped or defective ones show response to several gaseous analytes as suggested by theoretical calculations.[330,331]

Graphitic carbon nitride (g-C_3N_4) is an analogue of graphite with a layered structure where each layer is formed through the sp^2 hybridization of carbon and nitrogen atoms.[332] The semiconducting bandgaps of g-C_3N_4 lead to poor electronic conductivity and due to incomplete condensation during synthesis, g-C_3N_4 contains a small amount of hydrogen in the form of primary and/or secondary amine groups on the terminating edges.[34] Terminal hydrogen together with high electron affinity of nitrogen to many gaseous analytes results in the rich surface properties of g-C_3N_4. Including basic surface functionalities, electron-rich character, and H-bonding motifs; which can be exploited for gas sensing.[333]

Layered metal dichalcogenides (MDs) are a group of inorganic materials with a chemical formula of MX_2, where M is typically a hexavalent transition (Mo and W) or post-transition metal (Cd, Ga, In and Sn) ion and X is a divalent chalcogen (S, Se, and Te).[34,334] For example, in MoS_2, each monolayer contains covalently bonded atoms with the form of S–Mo–S; a layer of Mo atoms sandwiched between S atoms.[335] As a result, the surface chemistry of the basal plane of MDs is dominated by the perpendicularly projected chalcogenide lone pairs and on the prismatic edges, where either metal or chalcogenide atoms can be present, depending on the growth environment.[34,336] In particular, transition metal dichalcogenides (TMDs) such as MoS_2, $MoSe_2$, WS_2 and WSe_2 have sizeable bandgaps that change from indirect in the bulk to direct in single layers; and can be found as insulators, semiconductors, or metallic conductors depending on factors such as their crystal structure, intercalation species, and externally applied potential bias.[337,338] This allows for MDs to be incorporated into a broad range of analytical devices in which charge transport can be easily modulated by surface adsorbents.[34,335]

Metal–organic frameworks (MOFs) are a class of crystalline and porous hybrid materials formed *via* molecular self-assembly of metal-containing nodes with organic linkers (*e.g.*, carboxylate, hydroxyl, thiol, and amino) to form bulk crystals. Most reported MOFs are topological insulators. However, due to their structural and functional tunability, MOFs have garnered much interest in the field of chemical sensing.[339] Furthermore, introducing planar and fully conjugated ligands have given rise to MOFs that are semiconductive or conductive in nature.[339] Recently, some research groups reported new electrically conductive 2D MOFs such as $Cu_3(HITP)_2$ (HITP = 2,3,6,7,10,11-hex-aiminotriphenylene) and 2D MOF $Ni_3(HITP)_2$.[340] The authors attributed the excellent chemiresistive sensing performance of $Cu_3(HITP)_2$ for NH_3 to the enhanced electrical conductivity and the active copper sites.

MXenes are a class of 2D layered transition metal carbides and/or nitrides produced by selectively etching a certain element from $M_{n+1}AX_n$ phases (n = 1, 2, or 3), where M is an early transition metal, A is related to a main group (mostly group III A and IV A) element, X is carbon and/or nitrogen.[341] The surfaces of exfoliated MXenes typically contain –O, –F, –OH functional group terminations, introduced during the chemical etching process. These terminal groups allow for both charge transfer and interactions with surface analytes.[34] Besides, terminal groups such as O^- can modify the electronic structures of MXenes, causing their transition from metallic to semiconducting.[342] This enables using MXenes in electrical sensors on the basis of both charge transfer and doping.

Many of the 2D materials discussed above have newly emerged and show great potential for gas sensing. However, for many of them, their printed devices for gas sensing have not yet been demonstrated in literature. Consequently, this creates a very important direction for future research. To the best of our knowledge, thus far there have been several printed graphene-based gas sensors; in addition to a limited number of MD-based gas sensors (such as MoS_2, WS_2 and SnS_2) reported in the literature (Table 9).[103,191,343–350]

6.2 Working mechanisms of 2D materials in gas sensing

The structure of graphene and other 2D materials allows for individual atoms in the surface layer to be exposed to the ambient environment, and therefore, surface adsorbates. This offers a high surface area per unit volume to maximise the possibility of material-analyte interactions.[34] Consequently, charge transport in these materials is highly sensitive to its chemical environment. Theoretical studies have suggested that the interaction of an adsorbed gaseous analyte on the graphene surface can modulate the doping level and induce a step-like change in electrical conductivity of the material.[352] The active site for this interaction is the large, electron rich π system of the material which allows for van der Waals and π–π interactions, and charge transfer.[34] Additionally, structural defects and the edges of graphene-based materials, are regions where electron density can be localised, giving rise to much faster electron transfer rates at these sites compared to its basal plane.[353] Based on the work of Wehling *et al.* and Leenaerts *et al.*, two

Published on 17 February 2020. Downloaded by NANJING TECH UNIVERSITY on 2/1/2025 6:08:03 AM.

Table 9 Selected examples of printing methods and sensing performances of printed 2D material based gas sensors

Target gases	Materials	Printing techniques	Substrates	Electrodes/printing techniques	Sensitivity	Temperature (°C)	Flexibility	Ref.
NO_2	rGO/GO	Inkjet	PET			RT	Yes	101
	MoS_2	EHD	SiO_2	Cr/Au	35% at 10 ppm	100	No	345
	Ag–s-rGO	Gravure	PI	Ag ink/gravure	3.4% at 50 ppb	25	Yes	379
NH_3	Graphene–PEDOT:PSS	Inkjet	Flexible substrate	Ag ink/inkjet	9.6% at 500 ppm	RT	Yes	249
	GO nanoflake	Electrospray	Si/SiO_2	Cr/Au	500 ppm	RT	No	102
	SnS_2	Inkjet	Paper	—	5 ppm	—	Yes	344
	Graphene/ZnO	Inkjet	Si_3N_4 membrane	Au	1500% at 10 ppm	325/25	No	129
Acetone	$Pt/WO_3/rGO$	Gravure	Polyimide	Ag ink/gravure	12.2% at 10 ppm	200	Yes	73
	Graphene/WO_3	Inkjet	Si_3N_4 membrane	Au	9.12% at 2 ppm	300/150	No	129
HCHO	rGO/PMMA	—	Si/SiO_2	Cr/Au	1.51% at 100 ppm	RT	No	351

primary mechanisms for charge transfer between small molecules and graphene surfaces have been proposed.[354,355] The first of which is applicable to all molecules and operates through orbital hybridization of the analyte molecule bands and the graphene p_z bands, resulting in a small charge transfer, as in the case of physisorption.[354] On the other hand, the second mechanism is only applicable to open shell molecules and is dependent on the position of the HOMO and LUMO of the analyte molecule with respect to the Dirac point of graphene. This typically leads to larger charge transfer between adsorbed gas molecules and graphene.[355]

A similar charge transfer interaction with an adsorbed gaseous species described for graphene above, occurs in other conducting and semiconducting 2D materials at their respective active sites; in some cases, additional and/or different interactions arise due to the specific structure, surface chemistry and electronic properties of the 2D material (Table 10).[34,95,356–360] For instance, charge transfer based sensing mechanism has been reported in semiconducting MDs such as MoS_2[361] and SnS_2,[95] and metallic MDs such as positive ion-intercalated MoS_2[97] and alloyed $Sn_{0.5}W_{0.5}S_2$ nanosheets.[98] The extent of charge transfer and binding affinity between the target gas and 2D nanosheets are dependent highly on the composition of the nanosheets. For example, Wang *et al.* recently synthesized $Sn_{0.5}W_{0.5}S_2$ nanosheets on SnS_2 nanoplates for acetone sensing, which showed superior performance than SnS_2 nanoplates alone.[98] Part of the reason is the five times increased adsorption energy of acetone on $Sn_{0.5}W_{0.5}S_2$ than on SnS_2 which allows for more effective charge transfer (Fig. 20a and b). Unlike in 3D particles, charge transportation in a 2D atomically thin layer may be more easily effected by surface perturbations. As a consequence, gas adsorption may disrupt the original charge transport in the 2D layer, creating less oriented transporting pathways, and usually resulting in reduced electrical conductance.[362,363] This, in addition to charge transfer and doping, has also been regarded as a possible gas sensing mechanism for 2D gas sensing materials. For example, Kim and co-workers reported that an increased channel resistance was always observed when different VOCs

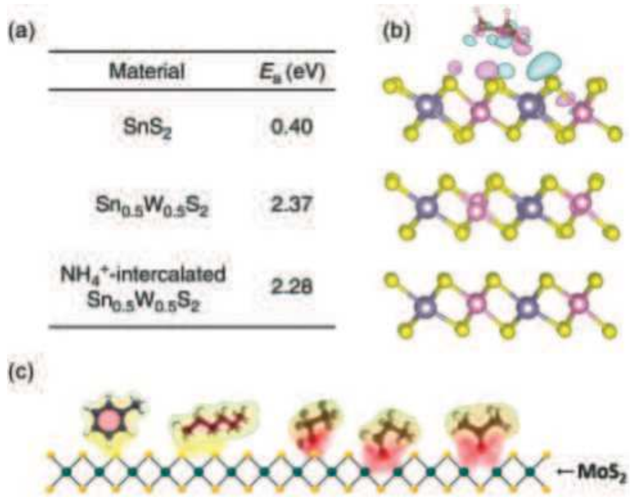

Fig. 20 (a) Calculated adsorption energy, E_a (eV), of acetone on different sensing materials. (b) Side view of the fully relaxed structural model of $Sn_{0.5}W_{0.5}S_2$ with surface adsorption of an acetone molecule. Cyan regions indicate charge accumulation, while pink regions represent charge depletion.[98] Reproduced from ref. 98. Copyright 2018, Nature Publishing Group. (c) Schematic description of sensing mechanism for MoS_2 sensing channels with target VOCs molecules (the VOCs molecules directly interact with the primitive MoS_2 surface and oxygen functionalized VOCs strongly interact with the sulfur vacancies sites).[365] Reproduced from ref. 365. Copyright 2014, American Chemical Society.

Table 10 Analyte–material interactions of 2D materials used in electrically-transduced gas sensing[34]

Material	Active sites	Primary resulting interactions
Graphene	sp^2 orbital of carbon (large π system), defects	π–π interaction, van der Waals, charge transfer
Black phosphorus (BP)	p_z orbital of P atom	Charge transfer
Transition metal dichalcogenides (TMDCs)	p orbital of chalcogenide atoms, defects	Charge transfer
Hexagonal boron nitride (h-BN), graphitic carbon nitride (g-C_3N_4)	Electron deficient π system, defects, adsorbed O_2^-	π–π interaction, van der Waals, oxidation reaction
2D metal organic frameworks (MOFs)	d orbital of metal node, adsorbed water/oxygen	Coordination, H-bond, charger transfer
Two dimensional transition metal carbides (MXene)	–O, –OH, and –F functional groups on surface	van der Waals, H-bond, charge transfer

 This journal is © The Royal Society of Chemistry 2020

Published on 17 February 2020. Downloaded by NANJING TECH UNIVERSITY on 2/1/2025 6:08:03 AM.

including toluene, hexane, ethanol, propanol, and acetone, regardless of their electron withdrawing or donating property, were respectively adsorbed on the surface of MoS_2 nanosheets.[364] The authors proposed that hexane and toluene interacted with MoS_2 only physically, while those VOCs with oxygen functional groups could interact with MoS_2 more strongly *via* the sulfur vacancies. The stronger interaction resulted in stronger dipole induced scattering of the charge carriers in the sensing channel (Fig. 20c), leading to a larger increase in the channel resistance.

Although the insulating nature of h-BN prevents charge transfer, it resembles graphene to allow for π–π interaction with gas molecules which can change its electrical conductance.[366] Moreover, H-bonding motifs which can be found on the surface of MOFs and MXene allow for H-bond interactions with gaseous analytes.[34] On the other hand, in g-C_3N_4, these motifs enable oxidation reactions.[333] For MOF materials, MOFs with an absent axial ligand will result in the presence of open coordination sites on the MOF surface, available for molecular interaction.[367]

6.3 Solution processability for sensor ink formulation

Graphene and related 2D materials such as (reduced graphene oxide (rGO), BP, h-BN, MoS_2, *etc.*) can be deposited using different printing techniques and the ink formulation is dependent on the chosen technique. Regardless of the printing technique, stable dispersions of the graphene and related 2D materials is essential in realising high throughput, low-cost and commercially viable device fabrication *via* printing.[135]

Prior to ink formulation, the production of mono- and few-layer graphene flakes is highly sought-after for devices.[368,369] This is true for other 2D materials with respect to their bulk form. In addition, significant challenges such as large scale production of high quality flakes and the subsequent stable dispersion must be addressed.[368]

Solution processing techniques employing high shear forces such as ultrasonic assisted liquid phase exfoliation (UALPE) is a relatively inexpensive and up-scalable method of producing graphene and other 2D materials. UALPE can also be used to directly create 2D material inks in suitable solvents (dictated by Hansen solubility parameters) and stabilisers, such as polymers including PVP, to prevent re-stacking; with appropriate post processing (*e.g.* centrifugation or filtration) to remove large particles.[135,191,370] Some of the most suitable solvents for graphene dispersions (*e.g.* NMP, DMF) are toxic and have high boiling points.[135,371] Zhang *et al.* has reported a simple solvent exchange method to prepare a meta-stable, mono- and few-layer graphene dispersion in ethanol, at concentrations up to 0.04 mg mL^{-1}.[368] Key observations from this work are: an initial stable dispersion in the suitable NMP solvent; then a solvent exchange and multiple sonication–filtering cycles in the low boiling point ethanol solvent, followed by the collection of the supernatant after centrifugation. The final stability of the graphene–ethanol dispersion could partly be attributed to the thorough sonication-based processing which would produce relatively smaller flakes as the crystallite size decreases with increased sonication time.[372] Presence of trace amounts of NMP and edge functionalisation during sonication could also be the determining factors. Other studies have replicated such solvent exchange processes to formulate stable, surfactant free 2D materials in single or mixed solvent systems.[183,344,373,374] Although printable, single solvent inks are usually unable to suppress the non-uniform deposition due to coffee ring effect.[16] Hu *et al.* has recently demonstrated a binder-free ink formulation for BP composed of isopropyl alcohol (IPA) and 2-butanol.[183] This inkjet ink formulation demonstrated natural suppression of coffee rings on to untreated substrates through Marangoni enhanced spreading and droplet deformation.[183]

6.4 Approaches to improve sensing performance through materials design and device fabrication

The remarkable properties of 2D materials result in excellent sensing performances in terms of sensitivity, detection limit and low power operation. For practical applications of 2D material gas sensors, the issue of selectivity persists. This was illustrated by the pristine graphene sensor in the work by Chen *et al.*, which showed high sensitivity but poor selectivity to a range of different gases.[375] To address this issue, functionalisation of these materials can be explored to improve sensor performance.[320]

The surface chemistry of 2D materials can be altered *via* chemical modification. One such approach is the doping of 2D materials with heteroatoms. This can endow the resultant material with new physiochemical and electronic properties which can be tuned towards better gas sensing performance.[376–378] Huang *et al.* demonstrated a fully gravure-printed chemiresistor for NO_2 detection with the sulfonated reduced graphene oxide (S-rGO).[379] The sensing material was further functionalized with silver nanoparticles forming Ag–S-rGO.[379] The sensor displayed good performance such as room temperature operation, high sensitivity (74.6%) to 50 ppm NO_2, and fast response and recovery times of 12 s and 20 s, respectively.[379] The strong electronegativity of sulfur atoms cause the S-doped rGO to become more resistive because of the free carrier trapping. However, this also increases the number of active sites for material–analyte interaction. This, coupled with the demonstrated ability of catalytic Ag NPs to promote sensitivity to NO_2 and reduced response time explains the improved performance.[378,379]

In addition to metal nanoparticles, functional elements such as metal oxides and polymers can be added to the surface of 2D materials to improve sensing performance.[190,380–385] Similar to CNTs mentioned in previous section, the response of 2D materials to VOCs, as well as oxidising and reducing gases can be significantly improved through the incorporation of metal oxides using synthetic approaches such as solvothermal and hydrothermal method, thermal hydrolysis, sol–gel method, electrodeposition, *etc.*[386–390] For instance, Chen *et al.* demonstrated a fully gravure printed chemiresistive gas sensor using WO_3/Pt-decorated rGO nanosheets.[73] The sensor showed good selectivity to acetone and a high response of 12.2 to 10 ppm concentrations. Fast response and recovery times of 14.1 s and 16.8 s, at a 200 °C operating temperature were also reported.[73] The superior performance of the WO_3/Pt-decorated rGO compared to pure rGO was attributed to the introduction of active

Published on 17 February 2020. Downloaded by NANJING TECH UNIVERSITY on 2/1/2025 6:08:03 AM.

sites in the form of hetero p–n junctions formed by the n-type semiconducting metal oxides like WO_3 and the p-type material, rGO.[380] In addition, the morphology of the WO_3 nanoparticles which exposed the (002) active facets was also highlighted by Chen *et al.* as a contributing factor to the good sensing performance.[35,73,391] The sensitivity to analytes in such hybrid systems could be enhanced with reduced recovery time by using an operating temperature above the ambient.[68] The adverse effect of using high operating temperatures is the high power required for the heating platform. A method to alleviate this has been recently demonstrated by Wu *et al.* in an inkjet printed graphene–metal oxide-based sensor system, integrated onto a 1 mm × 1 mm CMOS compatible microhotplate chip.[189] Instead of constant temperature (commonly known as the isothermal (ISO) mode), the authors used a controlled, periodic heating (temperature-pulsed modulation (TM) mode) strategy.[129] Along with shorter response and recovery and reduced power consumption (∼6 mW, 78% more energy efficient), the sensor also showed significantly reduced baseline drift (0.67% per cycle, 95% improvement), repeatability of responsivity (1.4% ± 0.08% of average cycle variation, a 70% improvement) over traditional ISO mode. In addition to the highly repeatable device performance due to ink formulation, a contributing factor to the sensor performance was the porosity induced by the selective post-decomposition of the ink binder. The authors suggested that this improved gas diffusion at the graphene–metal oxide heterojunctions at which the bandgap alteration occurs during the sensing event.

Another example of optimization of sensing performance through device fabrication process was presented by Lim *et al.* The authors demonstrated an MoS_2 chemiresistive gas sensor, with the active layer deposited *via* electrohydrodynamic (EHD) printing.[345] The device is capable of NO_2 and NH_3 detection down to few ppm, with sensitivities of approximately 25% and 10%, respectively at an operating temperature of 100 °C. A key observation on Lim *et al.*'s work is the high deposition uniformity of the MoS_2 flakes. This resulted in gas sensing performance on par with that of a CVD-grown MoS_2 gas sensor.[345] In addition, optimising the available surface area for gaseous analyte and 2D material interactions can also improve gas sensing performance. This is evident in Ma *et al.*'s work, where an inkjet printed NH_3 gas sensor was created with a binder-free ink consisting only of SnS_2 nanosheets and ethanol.[344] The SnS_2 sensor exhibited a good response time of ∼60 s; however, the sensitivity of the device was poor.[344]

7. Conclusion and outlook

Recent advancements in manufacturing gas sensors have been significantly propelled by new and increasingly mature printing technologies. Once suitable inks are formulated with well-controlled ink drying dynamics, printing can be used as a powerful yet adaptable additive manufacturing tool. A wide range of state-of-the-art equipment allow for large-scale, high-speed, cost-effective fabrication of devices on both rigid and flexible substrates. This enables development of device configurations, incorporation of materials with the required functionalities, such as conductivity for electrodes or selectivity for specific analytes for sensing layers with high device to device performance uniformity for large scale device manufacturing. Some emerging techniques are beginning to implement the use of traditional printing techniques to fabricate multilayer devices on a sacrificial layer, followed by the transfer of the device onto arbitrarily shaped objects.[392] This shows that despite being a mature, established technique, printing technologies are continually evolving in light of functional material deposition and device fabrication.

Thus far, printing has been utilized to fabricate gas sensors with metal oxides, conducting polymers, carbon nanotubes, graphene and certain metal chalcogenide nanosheets. These materials differ in ink formulation, suitable printing method, and gas sensing behaviour.

Metal oxides are among the most promising sensing materials for electron doping/withdrawing gases such as NO_2, NH_3 and acetone. However, elevated working temperature requires the printed devices to be integrated with a heating element. This adds complexity to the fabrication process, making it less suitable for flexible sensors on plastic substrates. Room temperature metal oxide sensors with good performance have been achieved by, for example, hybridizing metal oxides with graphene for improved charge transfer, and conducting polymers for creating p–n junctions.

The sensing pathways in conducting polymers are versatile, including gas adsorption induced charge doping, proton doping, and orientation/conformation change of molecular chains. They are also compatible with various solution processing techniques and ink formulations. Most importantly, compared to inorganic nanomaterials, polymers can be more easily functionalized with desired surface groups to realize specific gas recognition. The negative side of polymers is their relatively low conductivity, which can be overcome by forming composites with CNTs or graphene.

CNTs and graphene nanosheets, in addition to their intrinsic sensing capabilities, have been recognized as important conductive platforms to construct various hybrids/composites with meals, oxides, chalcogenides and polymers. Many reported sensor prototypes have demonstrated performances that meet the requirements for practical applications (Tables 8, 10 and 11), although detection of toxic VOCs such as formaldehyde is still challenging with printed sensors.

New materials or material systems that combine sensitive electronic properties, surface activities, and structural stability are continuously being explored, such as the recently emerged MXene family and versatile MOF materials.

A key consideration when designing the sensing materials for electronic gas sensors is the trade-off between high conductivity and sensitivity, the surface energy and sensor recovery, and the reactivity and stability of the material. Moreover, for practical applications, efforts in developing these functional sensing materials should be particularly devoted to improving their stability and durability, especially under conditions of changing humidity, temperature and mechanical stress/strain.

 This journal is © The Royal Society of Chemistry 2020

Published on 17 February 2020. Downloaded by NANJING TECH UNIVERSITY on 2/1/2025 6:08:03 AM.

Table 11 Requirements for practical gas sensing applications

Gases	Sensing application	Concentration (ppm)	Ref.
NO_2	Air quality	0.11	393
NH_3	Indoor air quality	0.29	393
	Food spoilage detection	5	11
C_2H_6O	Alcohol detection	47	394
HCHO	Indoor air quality	0.0815	393
H_2	Explosive detection	40 000	48

The majority of printed gas sensor prototypes that have recently been developed in research laboratories are single-output sensors. A key issue for their commercialisation from research to industrial scale is their poor selectivity to various gases. Although over the past years this has been addressed by using sensor arrays, sensor drift and unstable output in presence of unknown interferences still pose formidable challenges.

A newly emerging generation of gas sensors, the multivariable sensors, may potentially be able to counter the above challenges the conventional sensors face. A multivariable sensor involves a single sensing material with multiresponse mechanisms to different gases, coupled with a multivariable transducer to produce independent, for instance, electrical, optical, and electrochemical outputs corresponding to the different sensing responses. With high response dispersion, most multivariable gas sensors can discriminate individual gases,[396,397] with some sensors reported to be able to discriminate up to four different gases in mixtures.[398] Multivariable gas sensors are therefore ideally poised to replace conventional gas sensors in the future (Fig. 21).[395] However, it is challenging to manufacture multivariable gas sensors with printing techniques, which demand strongly coordinated developments in sensing materials, ink formulation, transducer designs, and most importantly, printing resolution. For instance, high quality semiconductor crystals that are able to respond to different gases both electrically and optically may show poor solution processability for ink formulation; while optical sensing transducers and readout devices are difficult to fabricate with existing printing methods due to high device complexity and precision requirement.

Although sensor arrays and multivariable sensors combined with computational approaches to data processing have enabled enhanced sensor performance, gas detection scenarios with complex and unknown gas environment may require further

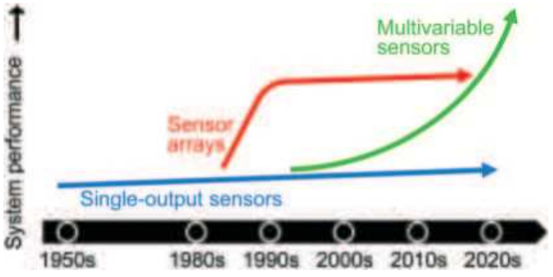

Fig. 21 Disruptive multivariable sensor technologies to complement and ultimately to replace conventional single output sensors and sensor arrays.[395] Reproduced from ref. 395. Copyright 2016 American Chemical Society.

data collection, modelling and machine learning. Traditional learning algorithms such as the neural network with the non-linear approximation ability is able to perform both qualitative and quantitative analysis. This has been demonstrated in gas sensor arrays for alcohol analysis,[399] and identification of food, oil and liquor samples.[400] Improvement in machine learning to enable generalization of knowledge based on existing data could allow predicting unseen situations, offering new opportunities to compensate the limitations of materials systems through powerful computational techniques. Therefore, a combination of machine learning for enhanced performance and printing for the large scale sensor fabrication is expected in future for a wide range of IoT and other automated sensor networks and systems.

Conflicts of interest

There are no conflicts to declare.

Acknowledgements

This work was supported by the National Natural Science Foundation of China (grant no. 5182001), the Fundamental Research Funds for the Central Universities of China, and the Young 1000 Talents Global Recruitment Program of China. It was also supported by the Engineering and Physical Sciences Research Council (EP/L016087/1) and Alphasense Limited in United Kingdom.

Notes and references

1 R. Rennie and J. Law, *A Dictionary of Chemistry*, Oxford University Press, 2016.
2 Franklin Institute, State Pennsylvania, Promot. Mech. Arts; Devoted to Mech. Phys. Sci. Civ. Eng. Arts Manuf. Rec. Am. Other Pat. Invent, vol. 22, no. 2, 1838.
3 O. Wood, *Kendal: Cumberland & Westmorland Antiquarian & Archaeological Society*, 1988.
4 X. Zhou, S. Lee, Z. Xu and J. Yoon, *Chem. Rev.*, 2015, **115**, 7944–8000.
5 M. Donarelli and L. Ottaviano, *Sensors*, 2018, **18**, 3638.
6 S. Mao, G. Lu and J. Chen, *J. Mater. Chem. A*, 2014, **2**, 5573–5579.
7 Y. Shimizu and M. Egashira, *MRS Bull.*, 1999, **24**, 18–24.
8 J. H. Kim, W. S. Chang, D. Kim, J. R. Yang, J. T. Han, G. W. Lee, J. T. Kim and S. K. Seol, *Adv. Mater.*, 2015, **27**, 157–161.
9 J. Kim, J. Yun, J. Song and C. Han, *Sens. Actuators, B*, 2009, **135**, 587–591.
10 L. Wang, S. Chen, W. Li, K. Wang, Z. Lou and G. Shen, *Adv. Mater.*, 2019, **31**, 1804583.
11 Z. Ma, P. Chen, W. Cheng, K. Yan, L. Pan, Y. Shi and G. Yu, *Nano Lett.*, 2018, **18**, 4570–4575.
12 Printed wires, *Br. Pat.*, 4681, 1903.
13 C. F. Coombs and H. T. Holden, *Printed circuits handbook*, McGraw-Hill, New York, 2001.

Published on 17 February 2020. Downloaded by NANJING TECH UNIVERSITY on 2/1/2025 6:08:03 AM.

14 A. Kamyshny and S. Magdassi, *Chem. Soc. Rev.*, 2019, **48**, 1712–1740.
15 W. Wu, *Nanoscale*, 2017, **9**, 7342–7372.
16 G. Hu, J. Kang, L. W. T. Ng, X. Zhu, R. C. T. Howe, C. G. Jones, M. C. Hersam and T. Hasan, *Chem. Soc. Rev.*, 2018, **47**, 3265–3300.
17 M. G. Campbell, S. F. Liu, T. M. Swager and M. Dinca, *J. Am. Chem. Soc.*, 2015, **137**, 13780–13783.
18 C. Anichini, W. Czepa, D. Pakulski, A. Aliprandi, A. Ciesielski and P. Samorì, *Chem. Soc. Rev.*, 2018, **47**, 4860–4908.
19 S. Cui, H. Pu, S. A. Wells, Z. Wen, S. Mao, J. Chang, M. C. Hersam and J. Chen, *Nat. Commun.*, 2015, **6**, 8632.
20 S. Mao, J. Chang, H. Pu, G. Lu, Q. He, H. Zhang and J. Chen, *Chem. Soc. Rev.*, 2017, **46**, 6872–6904.
21 M. Hartwig, R. Zichner and Y. Joseph, *Chemosensors*, 2018, **6**, 66.
22 A. Alreshaid, J. Hester, W. Su, Y. Fang and M. Tentzeris, *J. Electrochem. Soc.*, 2018, **165**, B407–B413.
23 A. Kaushik, R. Kumar, S. K. Arya, M. Nair, B. Malhotra and S. Bhansali, *Chem. Rev.*, 2015, **115**, 4571–4606.
24 M. Xue, F. Li, D. Chen, Z. Yang, X. Wang and J. Ji, *Adv. Mater.*, 2016, **28**, 8265–8270.
25 W. Luo, Q. Fu, D. Zhou, J. Deng, H. Liu and G. Yan, *Sens. Actuators, B*, 2013, **176**, 746–752.
26 Y. Yang, S. Li, W. Yang, W. Yuan, J. Xu and Y. Jiang, *ACS Appl. Mater. Interfaces*, 2014, **6**, 13807–13814.
27 J. Zhang, A. Boyd, A. Tselev, M. Paranjape and P. Barbara, *Appl. Phys. Lett.*, 2006, **88**, 123112.
28 P. Rai, R. Khan, S. Raj, S. M. Majhi, K. K. Park, Y. T. Yu, I. H. Lee and P. K. Sekhar, *Nanoscale*, 2014, **6**, 581–588.
29 Z. Jin, Y. Su and Y. Duan, *Sens. Actuators, B*, 2001, **72**, 75–79.
30 W. P. Jakubik, *Thin Solid Films*, 2011, **520**, 986–993.
31 P. Tardy, J. R. Coulon, C. Lucat and F. Menil, *Sens. Actuators, B*, 2004, **98**, 63–68.
32 A. Mujahid, H. Stathopulos, P. A. Lieberzeit and F. L. Dickert, *Sensors*, 2010, **10**, 4887–4897.
33 P. K. Sekhar, E. L. Brosha, R. Mukundan and F. Garzon, *Electrochem. Soc. Interface*, 2010, **19**, 35–40.
34 Z. Meng, R. M. Stolz, L. Mendecki and K. A. Mirica, *Chem. Rev.*, 2019, **119**, 478–598.
35 A. Gurlo, *Nanoscale*, 2011, **3**, 154–165.
36 Global gas sensor, detector and analyzer market (2019–2024), Modor Intelligence, 2018.
37 R. Paolesse, S. Nardis, D. Monti, M. Stefanelli and C. Di Natale, *Chem. Rev.*, 2016, **117**, 2517–2583.
38 E. Bakker and M. Telting-Diaz, *Anal. Chem.*, 2002, **74**, 2781–2800.
39 R. Deshpande, *Capacitors: technology and trends*, Tata McGraw-Hill Education, 2012.
40 I. Sinclair, *Passive components for circuit design*, Elsevier, 2000.
41 C. Zhang, P. Chen and W. Hu, *Chem. Soc. Rev.*, 2015, **44**, 2087–2107.
42 N. L. Teradal, S. Marx, A. Morag and R. Jelinek, *J. Mater. Chem. C*, 2017, **5**, 1128–1135.
43 M. S. Yao, W. X. Tang, G. E. Wang, B. Nath and G. Xu, *Adv. Mater.*, 2016, **28**, 5229–5234.
44 S. Kanaparthi and S. G. Singh, *ACS Appl. Nano Mater.*, 2019, **2**, 700–706.
45 S. Pandey and K. K. Nanda, *ACS Sens.*, 2015, **1**, 55–62.
46 F. G. Banica, *Chemical sensors and biosensors: fundamentals and applications*, John Wiley & Sons, 2012.
47 B. C. Sisk and N. S. Lewis, *Sens. Actuators, B*, 2005, **104**, 249–268.
48 O. K. Varghese, D. Gong, M. Paulose, K. G. Ong and C. A. Grimes, *Sens. Actuators, B*, 2003, **93**, 338–344.
49 K. Lundström, M. Shivaraman and C. Svensson, *J. Appl. Phys.*, 1975, **46**, 3876–3881.
50 L. Torsi, A. Dodabalapur, L. Sabbatini and P. Zambonin, *Sens. Actuators, B*, 2000, **67**, 312–316.
51 G. T. Feliciano, C. Sanz-Navarro, M. D. Coutinho-Neto, P. Ordejón, R. H. Scheicher and A. R. Rocha, *Phys. Rev. Appl.*, 2015, **3**, 034003.
52 H. Cui, C. Cheng, X. Lin, J. Wu, J. Chen, S. Eda and Q. Yuan, *Sens. Actuators, B*, 2016, **226**, 245–253.
53 O. Yassine, O. Shekhah, A. H. Assen, Y. Belmabkhout, K. N. Salama and M. Eddaoudi, *Angew. Chem., Int. Ed.*, 2016, **55**, 15879–15883.
54 Z. Lou, L. Li, L. Wang and G. Shen, *Small*, 2017, **13**, 1701791.
55 U. Altenberend, A. Oprea, N. Barsan and U. Weimar, *Anal. Bioanal. Chem.*, 2013, **405**, 6445–6452.
56 S. Pokhrel, C. Simion, V. Quemener, N. Barsan and U. Weimar, *Sens. Actuators, B*, 2008, **133**, 78–83.
57 V. Sridhar and K. Takahata, *Sens. Actuators, A*, 2009, **155**, 58–65.
58 H. G. Moon, Y. Jung, S. D. Han, Y. S. Shim, B. Shin, T. Lee, J. S. Kim, S. Lee, S. C. Jun and H. H. Park, *ACS Appl. Mater. Interfaces*, 2016, **8**, 20969–20976.
59 A. P. Turner and N. Magan, *Nat. Rev. Microbiol.*, 2004, **2**, 161–166.
60 E. C. Nallon, V. P. Schnee, C. Bright, M. P. Polcha and Q. Li, *ACS Sens.*, 2015, **1**, 26–31.
61 M. Ringnér, *Nat. Biotechnol.*, 2008, **26**, 303–304.
62 K. J. Porter, *College Composition and Communication*, 2001, pp. 574–611.
63 V. Shukla, J. Wärnå, N. K. Jena, A. Grigoriev and R. Ahuja, *J. Phys. Chem. C*, 2017, **121**, 26869–26876.
64 S. B. Franklin, D. J. Gibson, P. A. Robertson, J. T. Pohlmann and J. S. Fralish, *J. Veg. Sci.*, 1995, **6**, 99–106.
65 J. Van den Broek, S. Abegg, S. E. Pratsinis and A. Güntner, *Nat. Commun.*, 2019, **10**, 1–8.
66 F. A. Nugroho, I. Darmadi, L. Cusinato, A. Susarrey-Arce, H. Schreuders, L. J. Bannenberg, A. B. da Silva Fanta, S. Kadkhodazadeh, J. B. Wagner and T. J. Antosiewicz, *Nat. Mater.*, 2019, **18**, 489–495.
67 J. Zhang, X. Liu, G. Neri and N. Pinna, *Adv. Mater.*, 2016, **28**, 795–831.
68 C. Wang, L. Yin, L. Zhang, D. Xiang and R. Gao, *Sensors*, 2010, **10**, 2088–2106.
69 M. W. Hoffmann, L. Mayrhofer, O. Casals, L. Caccamo, F. Hernandez Ramirez, G. Lilienkamp, W. Daum,

 This journal is © The Royal Society of Chemistry 2020

Published on 17 February 2020. Downloaded by NANJING TECH UNIVERSITY on 2/1/2025 6:08:03 AM.

M. Moseler, A. Waag and H. Shen, *Adv. Mater.*, 2014, **26**, 8017–8022.
70 Y. F. Sun, S. B. Liu, F. L. Meng, J. Y. Liu, Z. Jin, L. T. Kong and J. H. Liu, *Sensors*, 2012, **12**, 2610–2631.
71 K. Wetchakun, T. Samerjai, N. Tamaekong, C. Liewhiran, C. Siriwong, V. Kruefu, A. Wisitsoraat, A. Tuantranont and S. Phanichphant, *Sens. Actuators, B*, 2011, **160**, 580–591.
72 A. Dey, *Mater. Sci. Eng., B*, 2018, **229**, 206–217.
73 L. Chen, L. Huang, Y. Lin, L. Sai, Q. Chang, W. Shi and Q. Chen, *Sens. Actuators, B*, 2018, **255**, 1482–1490.
74 Y. Lin, L. Huang, L. Chen, J. Zhang, L. Shen, Q. Chen and W. Shi, *Sens. Actuators, B*, 2015, **216**, 176–183.
75 H. Yoon and J. Jang, *Adv. Funct. Mater.*, 2009, **19**, 1567–1576.
76 M. Ates, *Mater. Sci. Eng.*, 2013, **33**, 1853–1859.
77 H. Yoon, *Nanomaterials*, 2013, **3**, 524–549.
78 M. Babaei and N. Alizadeh, *Sens. Actuators, B*, 2013, **183**, 617–626.
79 B. Li, S. Santhanam, L. Schultz, M. Jeffries-El, M. C. Iovu, G. Sauvé, J. Cooper, R. Zhang, J. C. Revelli, A. G. Kusne, J. L. Snyder, T. Kowalewski, L. E. Weiss, R. D. McCullough, G. K. Fedder and D. N. Lambeth, *Sens. Actuators, B*, 2007, **123**, 651–660.
80 B. Weng, R. L. Shepherd, K. Crowley, A. Killard and G. G. Wallace, *Analyst*, 2010, **135**, 2779–2789.
81 S. Abdulla, T. L. Mathew and B. Pullithadathil, *Sens. Actuators, B*, 2015, **221**, 1523–1534.
82 V. Talwar, O. Singh and R. C. Singh, *Sens. Actuators, B*, 2014, **191**, 276–282.
83 J. Wang, *Electroanalysis*, 2005, **17**, 7–14.
84 T. Zhang, S. Mubeen, N. V. Myung and M. A. Deshusses, *Nanotechnology*, 2008, **19**, 332001.
85 P. Bondavalli, P. Legagneux and D. Pribat, *Sens. Actuators, B*, 2009, **140**, 304–318.
86 A. Kaniyoor, R. I. Jafri, T. Arockiadoss and S. Ramaprabhu, *Nanoscale*, 2009, **1**, 382–386.
87 M. Asad and M. H. Sheikhi, *Sens. Actuators, B*, 2014, **198**, 134–141.
88 A. N. Volkov and L. V. Zhigilei, *Phys. Rev. Lett.*, 2010, **104**, 215902.
89 Z. K. Horastani, S. M. Sayedi and M. H. Sheikhi, *Sens. Actuators, B*, 2014, **202**, 461–468.
90 X. Liu, T. Ma, N. Pinna and J. Zhang, *Adv. Funct. Mater.*, 2017, **27**, 1702168.
91 A. N. Abbas, B. Liu, L. Chen, Y. Ma, S. Cong, N. Aroonyadet, M. Köpf, T. Nilges and C. Zhou, *ACS Nano*, 2015, **9**, 5618–5624.
92 G. Liu, S. Rumyantsev, C. Jiang, M. Shur and A. Balandin, *IEEE Electron Device Lett.*, 2015, **36**, 1202–1204.
93 W. Yuan and G. Shi, *J. Mater. Chem. A*, 2013, **1**, 10078–10091.
94 W. Yang, L. Gan, H. Li and T. Zhai, *Inorg. Chem. Front.*, 2016, **3**, 433–451.
95 J. Z. Ou, W. Ge, B. Carey, T. Daeneke, A. Rotbart, W. Shan, Y. Wang, Z. Fu, A. F. Chrimes and W. Wlodarski, *ACS Nano*, 2015, **9**, 10313–10323.
96 B. Cho, M. G. Hahm, M. Choi, J. Yoon, A. R. Kim, Y. J. Lee, S.-G. Park, J. D. Kwon, C. S. Kim and M. Song, *Sci. Rep.*, 2015, **5**, 8052.
97 K. Yang, X. Wang, H. Li, B. Chen, X. Zhang, S. Li, N. Wang, H. Zhang, X. Huang and W. Huang, *Nanoscale*, 2017, **9**, 5102–5109.
98 X. Wang, Z. Wang, J. Zhang, X. Wang, Z. Zhang, J. Wang, Z. Zhu, Z. Li, Y. Liu and X. Hu, *Nat. Commun.*, 2018, **9**, 3611.
99 J. H. Cha, S. J. Choi, S. Yu and I.-D. Kim, *J. Mater. Chem. A*, 2017, **5**, 8725–8732.
100 D.-H. Baek and J. Kim, *Sens. Actuators, B*, 2017, **250**, 686–691.
101 V. Dua, S. P. Surwade, S. Ammu, S. R. Agnihotra, S. Jain, K. E. Roberts, S. Park, R. S. Ruoff and S. K. Manohar, *Angew. Chem., Int. Ed.*, 2010, **49**, 2154–2157.
102 A. P. Taylor and L. F. Velasquez-Garcia, *Nanotechnology*, 2015, **26**, 505301.
103 K. K. Tadi, S. Pal and T. N. Narayanan, *Sci. Rep.*, 2016, **6**, 25221.
104 A. Nathan, A. Ahnood, M. T. Cole, S. Lee, Y. Suzuki, P. Hiralal, F. Bonaccorso, T. Hasan, L. Garcia-Gancedo and A. Dyadyusha, *Proc. IEEE*, 2012, **100**, 1486–1517.
105 Z. Cui, *Printed electronics: materials, technologies and applications*, JohnWiley & Sons Singapore Pte. Ltd, Singapore, 1st edn, 2016.
106 K. Suganuma, *Introduction to Printed Electronics*, Springer Science & Business Media, 2014.
107 M. Singh, H. M. Haverinen, P. Dhagat and G. E. Jabbour, *Adv. Mater.*, 2010, **22**, 673–685.
108 B. Derby, *Annu. Rev. Mater. Res.*, 2010, **40**, 395–414.
109 G. Hu, J. Kang, L. W. T. Ng, X. Zhu, R. C. T. Howe, C. G. Jones, M. C. Hersam and T. Hasan, *Chem. Soc. Rev.*, 2018, **47**, 3265–3300.
110 J.-U. Park, M. Hardy, S. J. Kang, K. Barton, K. Adair, D. Kishore Mukhopadhyay, C. Y. Lee, M. S. Strano, A. G. Alleyne and J. G. Georgiadis, *Nat. Mater.*, 2007, **6**, 782–789.
111 Z. Li, K. N. Al Milaji, H. Zhao and D. R. Chen, *J. Micromech. Microeng.*, 2019, **29**, 035013.
112 L. Ng, R. C. T. Howe, G. Hu, X. Zhu, Z. Yang, C. Jones and T. Hasan, *Functional Inks and Printing of Graphene and Related 2D Materials: Technology, Formulation and Applications*, Springer, 2018.
113 E. B. Secor, *Flexible Printed Electron.*, 2018, **3**, 035002.
114 A. Mahajan, C. Daniel Frisbie and L. F. Francis, *ACS Appl. Mater. Interfaces*, 2013, **5**, 4856–4864.
115 N. J. Wilkinson, M. A. A. Smith, R. W. Kay and R. A. Harris, *Int. J. Adv. Des. Manuf. Technol.*, 2019, 1–21.
116 J. M. Hoey, A. Lutfurakhmanov, D. L. Schulz and I. S. Akhatov, *J. Nanotechnol.*, 2012, 324380.
117 A. Yardimci, S. Guceri, S. Danforth, M. Agarwala and A. Safari, *Numerical modelling of fused deposition modelling*, San Francisco, USA, 1995.
118 K. Deshmukh, A. Muzaffar, T. Kovářík, T. Křenek, M. B. Ahamed and S. K. Pasha, *3D and 4D Printing of Polymer Nanocomposite Materials*, Elsevier, 2020, pp. 527–560.
119 D. Zhang, B. Chi, B. Li, Z. Gao, Y. Du, J. Guo and J. Wei, *Synth. Met.*, 2016, **217**, 79–86.
120 F. Daniel, N. H. Patoary, A. L. Moore, L. Weiss and A. D. Radadia, *Int. J. Adv. Des. Manuf. Technol.*, 2018, **99**, 1215–1224.

Published on 17 February 2020. Downloaded by NANJING TECH UNIVERSITY on 2/1/2025 6:08:03 AM.

121 M. Á. Caminero, J. M. Chacón, E. García-Plaza, P. J. Núñez, J. M. Reverte and J. P. Becar, *Polymers*, 2019, **11**, 799.
122 K. Pusch, T. J. Hinton and A. W. Feinberg, *HardwareX*, 2018, **3**, 49–61.
123 R. H. Leach and R. J. Pierce, *The Printing ink manual*, Springer, 1993.
124 A. Goldschmidt and H.-J. Streitberger, *BASF handbook on basics of coating technology*, Vincentz Network, 2003.
125 J. Yang, Y. Li, Y. Zheng, Y. Xu, Z. Zheng, X. Chen and W. Liu, *Small*, 2019, **15**, 1902826.
126 P. Wei, H. Leng, Q. Chen, R. C. Advincula and E. B. Pentzer, *ACS Appl. Polym. Mater.*, 2019, **1**, 885–892.
127 X. You, J. Wu and Y. Chi, *Anal. Chem.*, 2019, **91**, 5058–5066.
128 X. Tang, H. Zhou, Z. Cai, D. Cheng, P. He, P. Xie, D. Zhang and T. Fan, *ACS Nano*, 2018, **12**, 3502–3511.
129 T.-C. Wu, A. De Luca, Q. Zhong, X. Zhu, O. Ogbeide, D. S. Um, G. Hu, T. Albrow-Owen, F. Udrea and T. Hasan, *NPJ 2D Mater. Appl.*, 2019, **3**, 1–10.
130 O. Simseker, B. Kurt and E. Arman, *Asian J. Chem.*, 2012, **24**, 5253–5256.
131 H. Sirringhaus, T. Kawase, R. H. Friend, T. Shimoda, M. Inbasekaran, W. Wu and E. P. Woo, *Science*, 2000, **290**, 2123–2126.
132 P. Beecher, P. Servati, A. Rozhin, A. Colli, V. Scardaci, S. Pisana, T. Hasan, A. J. Flewitt, J. Robertson, G. W. Hsieh, F. M. Li, A. Nathan, A. C. Ferrari and W. I. Milne, *J. Appl. Phys.*, 2007, **102**, 43710.
133 F. Torrisi, T. Hasan, W. Wu, Z. Sun, A. Lombardo, T. S. Kulmala, G.-W. Hsieh, S. Jung, F. Bonaccorso, P. J. Paul, D. Chu and A. C. Ferrari, *ACS Nano*, 2012, **6**, 2992–3006.
134 D. McManus, S. Vranic, F. Withers, V. Sanchez-Romaguera, M. Macucci, H. Yang, R. Sorrentino, K. Parvez, S. K. Son, G. Iannaccone, K. Kostarelos, G. Fiori and C. Casiraghi, *Nat. Nanotechnol.*, 2017, **12**, 343–350.
135 L. W. Ng, G. Hu, R. C. Howe, X. Zhu, Z. Yang, C. G. Jones and T. Hasan, *Printing of Graphene and Related 2D Materials*, Springer, 2019.
136 T. Seiyama, A. Kato, K. Fujiishi and M. Nagatani, *Anal. Chem.*, 1962, **34**, 1502–1503.
137 N. Van Hieu, *Sens. Actuators, B*, 2010, **150**, 112–119.
138 S. Das and V. Jayaraman, *Prog. Mater. Sci.*, 2014, **66**, 112–255.
139 S. Lin, D. Li, J. Wu, X. Li and S. Akbar, *Sens. Actuators, B*, 2011, **156**, 505–509.
140 J. Gong, Y. Li, Z. Hu, Z. Zhou and Y. Deng, *J. Phys. Chem. C*, 2010, **114**, 9970–9974.
141 B. Urasinska-Wojcik, T. A. Vincent, M. F. Chowdhury and J. W. Gardner, *Sens. Actuators, B*, 2017, **239**, 1051–1059.
142 S. Shendage, V. Patil, S. Vanalakar, S. Patil, N. Harale, J. Bhosale, J. Kim and P. Patil, *Sens. Actuators, B*, 2017, **240**, 426–433.
143 D. R. Miller, S. A. Akbar and P. A. Morris, *Sens. Actuators, B*, 2014, **204**, 250–272.
144 G. Korotcenkov, *Mater. Sci. Eng., B*, 2007, **139**, 1–23.
145 Z. Zhang, M. haq, Z. Wen, Z. Ye and L. Zhu, *Appl. Surf. Sci.*, 2018, **434**, 891–897.
146 N. Barsan, D. Koziej and U. Weimar, *Sens. Actuators, B*, 2007, **121**, 18–35.
147 H. J. Kim and J. H. Lee, *Sens. Actuators, B*, 2014, **192**, 607–627.
148 Z. H. Jing and J. H. Zhan, *Adv. Mater.*, 2008, **20**, 4547–4551.
149 A. Oprea, E. Moretton, N. Barsan, W. J. Becker, J. Wollenstein and U. Weimar, *J. Appl. Phys.*, 2006, **100**, 033716.
150 A. Labidi, E. Gillet, R. Delamare, M. Maaref and K. Aguir, *Sens. Actuators, B*, 2006, **120**, 338–345.
151 P. P. Sahay, S. Tewari, S. Jha and M. Shamsuddin, *J. Mater. Sci.*, 2005, **40**, 4791–4793.
152 N. Yamazoe and N. Miura, *Chem. Sens. Technol.*, 1992, **4**, 19–42.
153 J. Mizsei, *Sens. Actuators, B*, 1995, **23**, 173–176.
154 V. Demarne, A. Grisel, R. Sanjines, D. Rosenfeld and F. Lévy, *Sens. Actuators, B*, 1992, **7**, 704–708.
155 L. Liao, Z. Zhang, B. Yan, Z. Zheng, Q. Bao, T. Wu, C. M. Li, Z. Shen, J. Zhang and H. Gong, *Nanotechnology*, 2009, **20**, 085203.
156 S. Hong, J. Shin, Y. Hong, M. Wu, D. Jang, Y. Jeong, G. Jung, J. H. Bae, H. W. Jang and J. H. Lee, *Nanoscale*, 2018, **10**, 18019–18027.
157 P. Andrei, L. Fields, J. Zheng, Y. Cheng and P. Xiong, *Sens. Actuators, B*, 2007, **128**, 226–234.
158 W. C. Tian, Y. H. Ho, C. H. Chen and C. Y. Kuo, *Sensors*, 2013, **13**, 865–874.
159 H. Pandya, S. Chandra and A. Vyas, *Sens. Actuators, B*, 2012, **161**, 923–928.
160 Y. Qin, X. Li, F. Wang and M. Hu, *J. Alloys Compd.*, 2011, **509**, 8401–8406.
161 M. Rieu, M. Camara, G. Tournier, J. P. Viricelle, C. Pijolat, N. F. de Rooij and D. Briand, *Sens. Actuators, B*, 2016, **236**, 1091–1097.
162 R. Von Hagen, M. Sneha, S. Mathur and B. Derby, *J. Am. Ceram. Soc.*, 2014, **97**, 1035–1040.
163 K. Kang, D. Yang, J. Park, S. Kim, I. Cho, H. H. Yang, M. Cho, S. Mousavi, K. H. Choi and I. Park, *Sens. Actuators, B*, 2017, **250**, 574–583.
164 R. Liu, H. Ding, J. Lin, F. Shen, Z. Cui and T. Zhang, *Nanotechnology*, 2012, **23**, 505301.
165 G. Dubourg and M. Radovic, *ACS Appl. Mater. Interfaces*, 2019, **11**, 6257–6266.
166 T.-S. Kim, Y. Lee, W. Xu, Y. H. Kim, M. Kim, S. Y. Min, T. H. Kim, H. W. Jang and T. W. Lee, *Nano Energy*, 2019, **58**, 437–446.
167 C.-J. Chang, S. T. Hung, C. K. Lin, C. Y. Chen and E. H. Kuo, *Thin Solid Films*, 2010, **519**, 1693–1698.
168 W. Shen, *Sens. Actuators, B*, 2012, **166–167**, 110–116.
169 N. Van Hieu, *Sens. Actuators, B*, 2010, **144**, 425–431.
170 Y. Lin, J. Chen, M. M. Tavakoli, Y. Gao, Y. Zhu, D. Zhang, M. Kam, Z. He and Z. Fan, *Adv. Mater.*, 2019, **31**, 1804285.
171 L. Xiao, H. Shen, R. von Hagen, J. Pan, L. Belkoura and S. Mathur, *Chem. Commun.*, 2010, **46**, 6509–6511.
172 S. Yi, S. Tian, D. Zeng, K. Xu, S. Zhang and C. Xie, *Sens. Actuators, B*, 2013, **185**, 345–353.

 This journal is © The Royal Society of Chemistry 2020

Published on 17 February 2020. Downloaded by NANJING TECH UNIVERSITY on 2/1/2025 6:08:03 AM.

173 X. Liu, T. J. Tarn, F. Huang and J. Fan, *Particuology*, 2015, **19**, 1–13.
174 M. Kang, S. Y. Lee, C. H. Chung, S. M. Cho, G. Y. Han, B. W. Kim and K. J. Yoon, *J. Photochem. Photobiol., A*, 2001, **144**, 185–191.
175 H. Y. Niu, J. M. Wang, Y. L. Shi, Y. Q. Cai and F. S. Wei, *Microporous Mesoporous Mater.*, 2009, **122**, 28–35.
176 R. A. John, A. C. Nguyen, Y. Chen, S. Shukla, S. Chen and N. Mathews, *ACS Appl. Mater. Interfaces*, 2016, **8**, 1139–1146.
177 S. Khan and D. Briand, *Flexible Printed Electron.*, 2019, **4**, 015002.
178 M. Ata, Y. Liu and I. Zhitomirsky, *RSC Adv.*, 2014, **4**, 22716–22732.
179 E. Ohayon and A. Gedanken, *Ultrason. Sonochem.*, 2010, **17**, 173–178.
180 N. Mandzy, E. Grulke and T. Druffel, *Powder Technol.*, 2005, **160**, 121–126.
181 C. Ban, Z. Wu, D. T. Gillaspie, L. Chen, Y. Yan, J. L. Blackburn and A. C. Dillon, *Adv. Mater.*, 2010, **22**, E145–E149.
182 J. Kukkola, M. Mohl, A. R. Leino, G. Tóth, M. C. Wu, A. Shchukarev, A. Popov, J. P. Mikkola, J. Lauri, M. Riihimäki, J. Lappalainen, H. Jantunen and K. Kordás, *J. Mater. Chem.*, 2012, **22**, 17878.
183 G. Hu, T. Albrow-Owen, X. Jin, A. Ali, Y. Hu, R. C. Howe, K. Shehzad, Z. Yang, X. Zhu and R. I. Woodward, *Nat. Commun.*, 2017, **8**, 278.
184 A. Ortiz Perez, H. Gao, X. Lyu, J. Wöllenstein, V. Kallfaß, J. Fonollosa and S. Palzer, *Appl. Nanosci.*, 2018, **8**, 1907–1914.
185 K. S. Jian, C. J. Chang, J. Wu, Y. C. Chang, C. Y. Tsay, J. H. Chen, T. L. Horng, G. J. Lee, L. Karuppasamy, S. Anandan and C. Y. Chen, *Polymers*, 2019, **11**, 184.
186 C. Bittencourt, E. Llobet, P. Ivanov, X. Correig, X. Vilanova, J. Brezmes, J. Hubalek, K. Malysz, J.-J. Pireaux and J. Calderer, *Sens. Actuators, B*, 2004, **97**, 67–73.
187 W. Hellmich, G. Müller, C. B. V. Braunmühl, T. Doll and I. Eisele, *Sens. Actuators, B*, 1997, **43**, 132–139.
188 P. Fau, M. Sauvan, S. Trautweiler, C. Nayral, L. Erades, A. Maisonnat and B. Chaudret, *Sens. Actuators, B*, 2001, **78**, 83–88.
189 P. K. Guha, S. Z. Ali, C. Lee, F. Udrea, W. Milne, T. Iwaki, J. A. Covington and J. Gardner, *Sens. Actuators, B*, 2007, **127**, 260–266.
190 E. Lee, Y. S. Yoon and D.-J. Kim, *ACS Sens.*, 2018, **3**, 2045–2060.
191 S. Santra, G. Hu, R. Howe, A. De Luca, S. Ali, F. Udrea, J. Gardner, S. Ray, P. Guha and T. Hasan, *Sci. Rep.*, 2015, **5**, 17374.
192 J. L. Ramírez, F. E. Annanouch, E. Llobet and D. Briand, *Sens. Actuators, B*, 2018, **258**, 952–960.
193 M. Tiemann, *Chemistry*, 2007, **13**, 8376–8388.
194 C. Xu, J. Tamaki, N. Miura and N. Yamazoe, *Sens. Actuators, B*, 1991, **3**, 147–155.
195 Y. S. Shim, L. Zhang, D. H. Kim, Y. H. Kim, Y. R. Choi, S. H. Nahm, C.-Y. Kang, W. Lee and H. W. Jang, *Sens. Actuators, B*, 2014, **198**, 294–301.
196 Z. Zhang, X. Zou, L. Xu, L. Liao, W. Liu, J. Ho, X. Xiao, C. Jiang and J. Li, *Nanoscale*, 2015, **7**, 10078–10084.
197 H. Ogawa, M. Nishikawa and A. Abe, *J. Appl. Phys.*, 1982, **53**, 4448–4455.
198 H. Ogawa, A. Abe, M. Nishikawa and S. Hayakawa, *J. Electrochem. Soc.*, 1981, **128**, 2020–2025.
199 S. Ansari, P. Boroojerdian, S. Sainkar, R. Karekar, R. Aiyer and S. Kulkarni, *Thin Solid Films*, 1997, **295**, 271–276.
200 A. Rothschild and Y. Komem, *J. Appl. Phys.*, 2004, **95**, 6374–6380.
201 B. Wang, L. F. Zhu, Y. H. Yang, N. S. Xu and G. W. Yang, *J. Phys. Chem. C*, 2008, **112**, 6643–6647.
202 Y. Qin, W. Shen, X. Li and M. Hu, *Sens. Actuators, B*, 2011, **155**, 646–652.
203 H. K. Yu and J. L. Lee, *Sci. Rep.*, 2014, **4**, 6589.
204 C. Cheng, K. Liu, B. Xiang, J. Suh and J. Wu, *Appl. Phys. Lett.*, 2012, **100**, 103111.
205 B. A. Helms, T. E. Williams, R. Buonsanti and D. J. Milliron, *Adv. Mater.*, 2015, **27**, 5820–5829.
206 Y. Wan, H. Yang and D. Zhao, *Acc. Chem. Res.*, 2006, **39**, 423–432.
207 W. Yue, C. Randorn, P. S. Attidekou, Z. Su, J. T. Irvine and W. Zhou, *Adv. Funct. Mater.*, 2009, **19**, 2826–2833.
208 J. McAleer, P. Moseley, J. Norris, D. Williams, P. Taylor and B. Tofield, *Mater. Chem. Phys.*, 1987, **17**, 577–583.
209 H. G. Moon, Y. S. Shim, D. H. Kim, H. Y. Jeong, M. Jeong, J. Y. Jung, S. M. Han, J. K. Kim, J. S. Kim, H. H. Park, J. H. Lee, H. L. Tuller, S. J. Yoon and H. W. Jang, *Sci. Rep.*, 2012, **2**, 588.
210 D. H. Kim, Y. S. Shim, H. G. Moon, H. J. Chang, D. Su, S. Y. Kim, J. S. Kim, B. K. Ju, S. J. Yoon and H. W. Jang, *J. Phys. Chem. C*, 2013, **117**, 17824–17831.
211 Y.-S. Shim, L. Zhang, D. H. Kim, Y. H. Kim, Y. R. Choi, S. H. Nahm, C.-Y. Kang, W. Lee and H. W. Jang, *Sens. Actuators, B*, 2014, **198**, 294–301.
212 Z. Wen and L. Tian-mo, *Phys. B*, 2010, **405**, 564–568.
213 J. Zhang, X. Liu, S. Wu, M. Xu, X. Guo and S. Wang, *J. Mater. Chem.*, 2010, **20**, 6453–6459.
214 I. Fratoddi, I. Venditti, C. Cametti and M. V. Russo, *Sens. Actuators, B*, 2015, **220**, 534–548.
215 C. Nylander, M. Armgarth and I. Lundström, *Anal. Chem. Symp. Ser.*, 1983, **17**, 203–207.
216 A. Lv, Y. Pan and L. Chi, *Sensors*, 2017, **17**, 213.
217 S. Waghuley, S. Yenorkar, S. Yawale and S. Yawale, *Sens. Actuators, B*, 2008, **128**, 366–373.
218 H. Bai and G. Shi, *Sensors*, 2007, **7**, 267–307.
219 O. S. Kwon, E. Park, O. Y. Kweon, S. J. Park and J. Jang, *Talanta*, 2010, **82**, 1338–1343.
220 L. Ruangchuay, A. Sirivat and J. Schwank, *Synth. Met.*, 2004, **140**, 15–21.
221 B. P. de Lacy Costello, N. M. Ratcliffe and P. S. Sivanand, *Synth. Met.*, 2003, **139**, 43–55.
222 L. Al-Mashat, H. D. Tran, W. Wlodarski, R. B. Kaner and K. Kalantar-Zadeh, *Sens. Actuators, B*, 2008, **134**, 826–831.
223 H. Yoon, M. Chang and J. Jang, *J. Phys. Chem. B*, 2006, **110**, 14074–14077.
224 S. Virji, J. Huang, R. B. Kaner and B. H. Weiller, *Nano Lett.*, 2004, **4**, 491–496.

Published on 17 February 2020. Downloaded by NANJING TECH UNIVERSITY on 2/1/2025 6:08:03 AM.

225 E. Kriván, C. Visy, R. Dobay, G. Harsányi and O. Berkesi, *Electroanalysis*, 2000, **12**, 1195–1200.
226 Q. Hao, X. Wang, L. Lu, X. Yang and V. M. Mirsky, *Macromol. Rapid Commun.*, 2005, **26**, 1099–1103.
227 S. Virji, J. D. Fowler, C. O. Baker, J. Huang, R. B. Kaner and B. H. Weiller, *Small*, 2005, **1**, 624–627.
228 H. Hu, M. Trejo, M. Nicho, J. Saniger and A. Garcıa-Valenzuela, *Sens. Actuators, B*, 2002, **82**, 14–23.
229 Y. S. Jung, W. Jung, H. L. Tuller and C. Ross, *Nano Lett.*, 2008, **8**, 3776–3780.
230 A. A. Athawale and M. V. Kulkarni, *Sens. Actuators, B*, 2000, **67**, 173–177.
231 C. Hagleitner, D. Lange, A. Hierlemann, O. Brand and H. Baltes, *IEEE J. Solid-State Circuits*, 2002, **37**, 1867–1878.
232 K. Nigorikawa, Y. Kunugi, Y. Harima and K. Yamashita, *J. Electroanal. Chem.*, 1995, **396**, 563–567.
233 B. Hwang, J. Yang and C. Lin, *Sens. Actuators, B*, 2001, **75**, 67–75.
234 K. Crowley, A. Morrin, A. Hernandez, E. Omalley, P. Whitten, G. Wallace, M. Smyth and A. Killard, *Talanta*, 2008, **77**, 710–717.
235 T. Syrový, P. Kuberský, I. Sapurina, S. Pretl, P. Bober, L. Syrová, A. Hamáček and J. Stejskal, *Sens. Actuators, B*, 2016, **225**, 510–516.
236 S. Li, Y. Li, S. Chen, W. Tang, Y. Huang, S. Peng, J. Qi and X. Guo, *IEEE Sens. Lett.*, 2018, **2**, 1–4.
237 R. Gangopadhyay and A. De, *Chem. Mater.*, 2000, **12**, 608–622.
238 D. Li and R. B. Kaner, *Chem. Commun.*, 2005, 3286–3288.
239 R. Koningsveld, W. Stockmayer, J. Kennedy and L. Kleintjens, *Macromolecules*, 1974, **7**, 73–79.
240 K. Crowley, E. O'Malley, A. Morrin, M. R. Smyth and A. J. Killard, *Analyst*, 2008, **133**, 391–399.
241 N. Haberkorn, J. S. Gutmann and P. Theato, *ACS Nano*, 2009, **3**, 1415–1422.
242 N. Nuraje, K. Su, N.-l. Yang and H. Matsui, *ACS Nano*, 2008, **2**, 502–506.
243 M. G. Han, S. K. Cho, S. G. Oh and S. S. Im, *Synth. Met.*, 2002, **126**, 53–60.
244 H. Peng, L. Zhang, C. Soeller and J. Travas-Sejdic, *Biomaterials*, 2009, **30**, 2132–2148.
245 R. D. McCullough, *Adv. Mater.*, 1998, **10**, 93–116.
246 H. Sirringhaus, N. Tessler and R. H. Friend, *Science*, 1998, **280**, 1741–1744.
247 N. Dubey and M. Leclerc, *J. Polym. Sci., Part B: Polym. Phys.*, 2011, **49**, 467–475.
248 J. W. Park, D. H. Lee, D. S. Chung, D. M. Kang, Y. H. Kim, C. E. Park and S. K. Kwon, *Macromolecules*, 2010, **43**, 2118–2123.
249 Y. Seekaew, S. Lokavee, D. Phokharatkul, A. Wisitsoraat, T. Kerdcharoen and C. Wongchoosuk, *Org. Electron.*, 2014, **15**, 2971–2981.
250 X. Chen, C. K. Wong, C. A. Yuan and G. Zhang, *Sens. Actuators, B*, 2012, **175**, 15–21.
251 C. M. Hangarter, N. Chartuprayoon, S. C. Hernández, Y. Choa and N. V. Myung, *Nano Today*, 2013, **8**, 39–55.
252 D. Nicolas-Debarnot and F. Poncin-Epaillard, *Anal. Chim. Acta*, 2003, **475**, 1–15.
253 L. Huang, P. Jiang, D. Wang, Y. Luo, M. Li, H. Lee and R. A. Gerhardt, *Sens. Actuators, B*, 2014, **197**, 308–313.
254 J. Kong, N. R. Franklin, C. Zhou, M. G. Chapline, S. Peng, K. Cho and H. Dai, *Science*, 2000, **287**, 622–625.
255 J. D. Shi, X. M. Li, H. Y. Cheng, Z. J. Liu, L. Y. Zhao, T. T. Yang, Z. H. Dai, Z. G. Cheng, E. Z. Shi, L. Yang, Z. Zhang, A. Y. Cao, H. W. Zhu and Y. Fang, *Adv. Funct. Mater.*, 2016, **26**, 2078–2084.
256 R. Kocache, *Sens. Rev.*, 1994, **14**, 8–12.
257 C. Marliere, P. Poncharal, L. Vaccarini and A. Zahab, *Mater. Res. Soc. Symp. Proc.*, 1999, **593**, 173.
258 E. Joselevich and C. M. Lieber, *Nano Lett.*, 2002, **2**, 1137–1141.
259 L. Valentini, I. Armentano, J. Kenny, C. Cantalini, L. Lozzi and S. Santucci, *Appl. Phys. Lett.*, 2003, **82**, 961–963.
260 Z. Zanolli, R. Leghrib, A. Felten, J. J. Pireaux, E. Llobet and J. C. Charlier, *ACS Nano*, 2011, **5**, 4592–4599.
261 N. Du, H. Zhang, B. Chen, X. Ma, Z. Liu, J. Wu and D. Yang, *Adv. Mater.*, 2007, **19**, 1641–1645.
262 S. Cui, H. Pu, G. Lu, Z. Wen, E. C. Mattson, C. Hirschmugl, M. Gajdardziska-Josifovska, M. Weinert and J. Chen, *ACS Appl. Mater. Interfaces*, 2012, **4**, 4898–4904.
263 T. Ueda, M. Bhuiyan, H. Norimatsu, S. Katsuki, T. Ikegami and F. Mitsugi, *Phys. E*, 2008, **40**, 2272–2277.
264 G. Chen, T. M. Paronyan, E. M. Pigos and A. R. Harutyunyan, *Sci. Rep.*, 2012, **2**, 343.
265 J. Kong, M. G. Chapline and H. Dai, *Adv. Mater.*, 2001, **13**, 1384–1386.
266 V. R. Khalap, T. Sheps, A. A. Kane and P. G. Collins, *Nano Lett.*, 2010, **10**, 896–901.
267 T. Someya, J. Small, P. Kim, C. Nuckolls and J. T. Yardley, *Nano Lett.*, 2003, **3**, 877–881.
268 N. Van Hieu, N. A. P. Duc, T. Trung, M. A. Tuan and N. D. Chien, *Sens. Actuators, B*, 2010, **144**, 450–456.
269 M. Asad, M. H. Sheikhi, M. Pourfath and M. Moradi, *Sens. Actuators, B*, 2015, **210**, 1–8.
270 P. Qi, O. Vermesh, M. Grecu, A. Javey, Q. Wang, H. Dai, S. Peng and K. Cho, *Nano Lett.*, 2003, **3**, 347–351.
271 H. Chang, J. D. Lee, S. M. Lee and Y. H. Lee, *Appl. Phys. Lett.*, 2001, **79**, 3863–3865.
272 J. Zhao, A. Buldum, J. Han and J. P. Lu, *Nanotechnology*, 2002, **13**, 195.
273 J. Zhang, A. Boyd, A. Tselev, M. Paranjape and P. Barbara, *Appl. Phys. Lett.*, 2006, **88**, 123112.
274 H. Lee, G. Shaker, K. Naishadham, X. Song, M. McKinley, B. Wagner and M. Tentzeris, *IEEE Trans. Microwave Theory Tech.*, 2011, **59**(10), 2665–2673.
275 L. Yang, R. Zhang, D. Staiculescu, C. P. Wong and M. M. Tentzeris, *IEEE Antenn. Wirel. Propag. Lett.*, 2009, **8**, 653–656.
276 Z. Lin, T. Le, X. Song, Y. Yao, Z. Li, K. S. Moon, M. M. Tentzeris and C.-p. Wong, *J. Electron. Packag.*, 2013, **135**, 011001.
277 C. Zhou, J. Zhao, J. Ye, M. Tange, X. Zhang, W. Xu, K. Zhang, T. Okazaki and Z. Cui, *Carbon*, 2016, **108**, 372–380.

 This journal is © The Royal Society of Chemistry 2020

Published on 17 February 2020. Downloaded by NANJING TECH UNIVERSITY on 2/1/2025 6:08:03 AM.

278 G. P. Evans, D. J. Buckley, N. T. Skipper and I. P. Parkin, *RSC Adv.*, 2014, **4**, 51395–51403.
279 K. Y. Dong, D. J. Ham, B. H. Kang, K. Lee, J. Choi, J. W. Lee, H. H. Choi and B. K. Ju, *Talanta*, 2012, **89**, 33–37.
280 A. S. Alshammari, M. R. Alenezi, K. T. Lai and S. R. P. Silva, *Mater. Lett.*, 2017, **189**, 299–302.
281 K. Timsorn and C. Wongchoosuk, *J. Mater. Sci.: Mater. Electron.*, 2019, **30**, 4782–4791.
282 Z. Kennedy, J. Christ, K. Evans, B. Arey, L. Sweet, M. Warner, R. Erikson and C. Barrett, *Nanoscale*, 2017, **9**, 5458–5466.
283 L. Girifalco, M. Hodak and R. S. Lee, *Phys. Rev. B: Condens. Matter Mater. Phys.*, 2000, **62**, 13104.
284 T. Hasan, V. Scardaci, P. Tan, A. G. Rozhin, W. I. Milne and A. C. Ferrari, *J. Phys. Chem. C*, 2007, **111**, 12594–12602.
285 P. Beecher, P. Servati, A. Rozhin, A. Colli, V. Scardaci, S. Pisana, T. Hasan, A. Flewitt, J. Robertson and G. Hsieh, *J. Appl. Phys.*, 2007, **102**, 043710.
286 K. Lu, R. Lago, Y. Chen, M. Green, P. Harris and S. Tsang, *Carbon*, 1996, **34**, 814–816.
287 D. S. Hecht, L. Hu and G. Irvin, *Adv. Mater.*, 2011, **23**, 1482–1513.
288 J. Hilding, E. A. Grulke, Z. George Zhang and F. Lockwood, *J. Dispersion Sci. Technol.*, 2003, **24**, 1–41.
289 A. Hirsch, *Angew. Chem., Int. Ed.*, 2002, **41**, 1853–1859.
290 S. H. Lee, J. S. Park, B. K. Lim and S. O. Kim, *J. Appl. Polym. Sci.*, 2008, **110**, 2345–2351.
291 X. Li, Y. J. Jeong, J. Jang, S. Lim and S. H. Kim, *Phys. Chem. Chem. Phys.*, 2018, **20**, 1210–1220.
292 J. A. Robinson, E. S. Snow, Ş. C. Bădescu, T. L. Reinecke and F. K. Perkins, *Nano Lett.*, 2006, **6**, 1747–1751.
293 S. Dhall, N. Jaggi and R. Nathawat, *Sens. Actuators, A*, 2013, **201**, 321–327.
294 J. J. Adjizian, R. Leghrib, A. A. Koos, I. Suarez-Martinez, A. Crossley, P. Wagner, N. Grobert, E. Llobet and C. P. Ewels, *Carbon*, 2014, **66**, 662–673.
295 L. Bai and Z. Zhou, *Carbon*, 2007, **45**, 2105–2110.
296 Y. Liao, C. Zhang, Y. Zhang, V. Strong, J. Tang, X.-G. Li, K. Kalantar Zadeh, E. M. Hoek, K. L. Wang and R. B. Kaner, *Nano Lett.*, 2011, **11**, 954–959.
297 H. Y. Jeong, D. S. Lee, H. K. Choi, D. H. Lee, J. E. Kim, J. Y. Lee, W. J. Lee, S. O. Kim and S. Y. Choi, *Appl. Phys. Lett.*, 2010, **96**, 213105.
298 D. Fu, H. Lim, Y. Shi, X. Dong, S. Mhaisalkar, Y. Chen, S. Moochhala and L.-J. Li, *J. Phys. Chem. C*, 2008, **112**, 650–653.
299 M. L. Y. Sin, G. C. T. Chow, G. M. K. Wong, W. J. Li, P. H. W. Leong and K. W. Wong, *IEEE Trans. Nanotechnol.*, 2007, **6**, 571–577.
300 N. P. Zschoerper, V. Katzenmaier, U. Vohrer, M. Haupt, C. Oehr and T. Hirth, *Carbon*, 2009, **47**, 2174–2185.
301 H. Mu, K. Wang, S. Zhang, K. Shi, S. Sun, Z. Li, J. Zhou and H. Xie, *IEEE Sens. J.*, 2014, **14**, 2362–2368.
302 K. Y. Dong, J. Choi, Y. D. Lee, B. H. Kang, Y.-Y. Yu, H. H. Choi and B.-K. Ju, *Nanoscale Res. Lett.*, 2013, **8**, 12.
303 Y. Zhang, Y. Zhang, D. Zhang and C. Liu, *J. Phys. Chem. B*, 2006, **110**, 4671–4674.
304 R. J. Nicholls, Z. Aslam, M. C. Sarahan, A. Koós, J. R. Yates, P. D. Nellist and N. Grobert, *ACS Nano*, 2012, **6**, 7800–7805.
305 J.-J. Adjizian, R. Leghrib, A. A. Koos, I. Suarez-Martinez, A. Crossley, P. Wagner, N. Grobert, E. Llobet and C. P. Ewels, *Carbon*, 2014, **66**, 662–673.
306 M. K. Kumar and S. Ramaprabhu, *J. Phys. Chem. B*, 2006, **110**, 11291–11298.
307 Y. Sun and H. H. Wang, *Appl. Phys. Lett.*, 2007, **90**, 213107.
308 S. Mubeen, T. Zhang, B. Yoo, M. A. Deshusses and N. V. Myung, *J. Phys. Chem. C*, 2007, **111**, 6321–6327.
309 S. Mubeen, T. Zhang, N. Chartuprayoon, Y. Rheem, A. Mulchandani, N. V. Myung and M. A. Deshusses, *Anal. Chem.*, 2009, **82**, 250–257.
310 Z. Zanolli, R. Leghrib, A. Felten, J.-J. Pireaux, E. Llobet and J.-C. Charlier, *ACS Nano*, 2011, **5**, 4592–4599.
311 S. Cui, H. Pu, E. C. Mattson, G. Lu, S. Mao, M. Weinert, C. J. Hirschmugl, M. Gajdardziska-Josifovska and J. Chen, *Nanoscale*, 2012, **4**, 5887–5894.
312 D. W. H. Fam, A. I. Y. Tok, A. Palaniappan, P. Nopphawan, A. Lohani and S. G. Mhaisalkar, *Sens. Actuators, B*, 2009, **138**, 189–192.
313 S. Sharma, S. Hussain, S. Singh and S. Islam, *Sens. Actuators, B*, 2014, **194**, 213–219.
314 C. Wongchoosuk, A. Wisitsoraat, A. Tuantranont and T. Kerdcharoen, *Sens. Actuators, B*, 2010, **147**, 392–399.
315 C. Wongchoosuk, A. Wisitsoraat, D. Phokharatkul, A. Tuantranont and T. Kerdcharoen, *Sensors*, 2010, **10**, 7705–7715.
316 Z. Bao, Y. Feng, A. Dodabalapur, V. Raju and A. J. Lovinger, *Chem. Mater.*, 1997, **9**, 1299–1301.
317 T. Sekitani, H. Nakajima, H. Maeda, T. Fukushima, T. Aida, K. Hata and T. Someya, *Nat. Mater.*, 2009, **8**, 494.
318 E. Menard, M. A. Meitl, Y. Sun, J.-U. Park, D. J. L. Shir, Y.-S. Nam, S. Jeon and J. A. Rogers, *Chem. Rev.*, 2007, **107**, 1117–1160.
319 A. Mahajan, C. D. Frisbie and L. F. Francis, *ACS Appl. Mater. Interfaces*, 2013, **5**, 4856–4864.
320 V. Georgakilas, M. Otyepka, A. B. Bourlinos, V. Chandra, N. Kim, K. C. Kemp, P. Hobza, R. Zboril and K. S. Kim, *Chem. Rev.*, 2012, **112**, 6156–6214.
321 E. Singh, M. Meyyappan and H. S. Nalwa, *ACS Appl. Mater. Interfaces*, 2017, **9**, 34544–34586.
322 F. Bonaccorso, L. Colombo, G. Yu, M. Stoller, V. Tozzini, A. C. Ferrari, R. S. Ruoff and V. Pellegrini, *Science*, 2015, **347**, 1246501.
323 J.-H. Chen, C. Jang, S. Xiao, M. Ishigami and M. S. Fuhrer, *Nat. Nanotechnol.*, 2008, **3**, 206.
324 A. Akturk and N. Goldsman, *J. Appl. Phys.*, 2008, **103**, 053702.
325 S. Rumyantsev, G. Liu, W. Stillman, M. Shur and A. Balandin, *J. Phys.: Condens. Matter*, 2010, **22**, 395302.
326 X. Tang, A. Du and L. Kou, *Wiley Interdiscip. Rev.: Comput. Mol. Sci.*, 2018, **8**, e1361.
327 P. Ou, P. Song, X. Liu and J. Song, *Adv. Theory Simul.*, 2019, **2**, 1800103.
328 K. Xu, C. Fu, Z. Gao, F. Wei, Y. Ying, C. Xu and G. Fu, *Instrum. Sci. Technol.*, 2018, **46**, 115–145.

Published on 17 February 2020. Downloaded by NANJING TECH UNIVERSITY on 2/1/2025 6:08:03 AM.

329 M. Topsakal, E. Aktürk and S. Ciraci, *Phys. Rev. B: Condens. Matter Mater. Phys.*, 2009, **79**, 115442.
330 L. H. Li and Y. Chen, *Adv. Funct. Mater.*, 2016, **26**, 2594–2608.
331 K. Zhang, Y. Feng, F. Wang, Z. Yang and J. Wang, *J. Mater. Chem. C*, 2017, **5**, 11992–12022.
332 T. Miller, A. B. Jorge, T. Suter, A. Sella, F. Cora and P. McMillan, *Phys. Chem. Chem. Phys.*, 2017, **19**, 15613–15638.
333 J. Zhu, P. Xiao, H. Li and S. A. Carabineiro, *ACS Appl. Mater. Interfaces*, 2014, **6**, 16449–16465.
334 M. Chhowalla, H. S. Shin, G. Eda, L.-J. Li, K. P. Loh and H. Zhang, *Nat. Chem.*, 2013, **5**, 263.
335 Q. H. Wang, K. Kalantar-Zadeh, A. Kis, J. N. Coleman and M. S. Strano, *Nat. Nanotechnol.*, 2012, **7**, 699.
336 H. Zhang, *ACS Nano*, 2015, **9**, 9451–9469.
337 S. Manzeli, D. Ovchinnikov, D. Pasquier, O. V. Yazyev and A. Kis, *Nat. Rev. Mater.*, 2017, **2**, 17033.
338 M. Chhowalla, Z. Liu and H. Zhang, *Chem. Soc. Rev.*, 2015, **44**, 2584–2586.
339 L. Sun, M. G. Campbell and M. Dincă, *Angew. Chem., Int. Ed.*, 2016, **55**, 3566–3579.
340 M. G. Campbell, D. Sheberla, S. F. Liu, T. M. Swager and M. Dincă, *Angew. Chem., Int. Ed.*, 2015, **54**, 4349–4352.
341 C. Tan, X. Cao, X. J. Wu, Q. He, J. Yang, X. Zhang, J. Chen, W. Zhao, S. Han and G. H. Nam, *Chem. Rev.*, 2017, **117**, 6225–6331.
342 M. Khazaei, M. Arai, T. Sasaki, C. Y. Chung, N. S. Venkataramanan, M. Estili, Y. Sakka and Y. Kawazoe, *Adv. Funct. Mater.*, 2013, **23**, 2185–2192.
343 Y. Jeong, J. Shin, Y. Hong, M. Wu, S. Hong, K. C. Kwon, S. Choi, T. Lee, H. W. Jang and J. H. Lee, *Solid State Electron.*, 2019, **153**, 27–32.
344 X. Ma, Z. Xie, Z. Yang, G. Zeng, M. Xue and X. Zhang, *Mater. Res. Express*, 2018, **6**, 015025.
345 S. Lim, B. Cho, J. Bae, A. R. Kim, K. H. Lee, S. H. Kim, M. G. Hahm and J. Nam, *Nanotechnology*, 2016, **27**, 435501.
346 V. Dua, S. P. Surwade, S. Ammu, S. R. Agnihotra, S. Jain, K. E. Roberts, S. Park, R. S. Ruoff and S. K. Manohar, *Angew. Chem., Int. Ed.*, 2010, **49**, 2154–2157.
347 T. Le, V. Lakafosis, S. Kim, B. Cook, M. M. Tentzeris, Z. Lin and C.-P. Wong, *42nd European Microwave Conference*, 2012, pp. 412–415.
348 A. P. Taylor and L. F. Velásquez-García, *Nanotechnology*, 2015, **26**, 505301.
349 J. H. Kim, W. S. Chang, D. Kim, J. R. Yang, J. T. Han, G. W. Lee, J. T. Kim and S. K. Seol, *Adv. Mater.*, 2015, **27**, 157–161.
350 Y. Seekaew, S. Lokavee, D. Phokharatkul, A. Wisitsoraat, T. Kerdcharoen and C. Wongchoosuk, *Org. Electron.*, 2014, **15**, 2971–2981.
351 W. Y. Chuang, S. Y. Yang, W. J. Wu and C. T. Lin, *Sensors*, 2015, **15**, 28842–28853.
352 S. Tang and Z. Cao, *J. Chem. Phys.*, 2011, **134**, 044710.
353 M. Zhou, Y. Zhai and S. Dong, *Anal. Chem.*, 2009, **81**, 5603–5613.
354 T. Wehling, K. Novoselov, S. Morozov, E. Vdovin, M. Katsnelson, A. Geim and A. Lichtenstein, *Nano Lett.*, 2008, **8**, 173–177.
355 O. Leenaerts, B. Partoens and F. M. Peeters, *Microelectron. J.*, 2009, **40**, 860–862.
356 J. Park, J. Mun, J. S. Shin and S. W. Kang, *R. Soc. Open Sci.*, 2018, **5**, 181462.
357 Y. Cai, Q. Ke, G. Zhang and Y. W. Zhang, *J. Phys. Chem. C*, 2015, **119**, 3102–3110.
358 J. Zhu, E. Ha, G. Zhao, Y. Zhou, D. Huang, G. Yue, L. Hu, N. Sun, Y. Wang and L. Y. S. Lee, *Coord. Chem. Rev.*, 2017, **352**, 306–327.
359 H. Shiozawa, B. C. Bayer, H. Peterlik, J. C. Meyer, W. Lang and T. Pichler, *Sci. Rep.*, 2017, **7**, 2439.
360 S. K. Gupta, D. Singh, K. Rajput and Y. Sonvane, *RSC Adv.*, 2016, **6**, 102264–102271.
361 D. J. Late, Y. K. Huang, B. Liu, J. Acharya, S. N. Shirodkar, J. Luo, A. Yan, D. Charles, U. V. Waghmare and V. P. Dravid, *ACS Nano*, 2013, **7**, 4879–4891.
362 I. Silvestre, E. A. de Morais, A. O. Melo, L. C. Campos, A.-M. B. Goncalves, A. R. Cadore, A. S. Ferlauto, H. Chacham, M. S. Mazzoni and R. G. Lacerda, *ACS Nano*, 2013, **7**, 6597–6604.
363 N. Fu, Y. Duan, W. Lu, M. Zhu, G. Zhang, D. Xie, Y. Lin, M. Wei and H. Huang, *J. Mater. Chem. A*, 2019, **7**, 11520–11529.
364 S. J. Kim, H.-J. Koh, C. E. Ren, O. Kwon, K. Maleski, S.-Y. Cho, B. Anasori, C.-K. Kim, Y.-K. Choi and J. Kim, *ACS Nano*, 2018, **12**, 986–993.
365 J.-S. Kim, H.-W. Yoo, H. O. Choi and H.-T. Jung, *Nano Lett.*, 2014, **14**, 5941–5947.
366 H. Vovusha, R. G. Amorim, R. H. Scheicher and B. Sanyal, *RSC Adv.*, 2018, **8**, 6527–6531.
367 Z. Hu and D. Zhao, *CrystEngComm*, 2017, **19**, 4066–4081.
368 X. Zhang, A. C. Coleman, N. Katsonis, W. R. Browne, B. J. Van Wees and B. L. Feringa, *Chem. Commun.*, 2010, **46**, 7539–7541.
369 A. C. Ferrari, F. Bonaccorso, V. Fal'Ko, K. S. Novoselov, S. Roche, P. Bøggild, S. Borini, F. H. Koppens, V. Palermo and N. Pugno, *Nanoscale*, 2015, **7**, 4598–4810.
370 E. B. Secor, P. L. Prabhumirashi, K. Puntambekar, M. L. Geier and M. C. Hersam, *J. Phys. Chem. Lett.*, 2013, **4**, 1347–1351.
371 G. Hu, J. Kang, L. W. Ng, X. Zhu, R. C. Howe, C. G. Jones, M. C. Hersam and T. Hasan, *Chem. Soc. Rev.*, 2018, **47**, 3265–3300.
372 Y. Xu, H. Cao, Y. Xue, B. Li and W. Cai, *Nanomaterials*, 2018, **8**, 942.
373 A. Ciesielski and P. Samorì, *Chem. Soc. Rev.*, 2014, **43**, 381–398.
374 X. Han, Y. Chen, H. Zhu, C. Preston, J. Wan, Z. Fang and L. Hu, *Nanotechnology*, 2013, **24**, 205304.
375 G. Chen, T. M. Paronyan and A. R. Harutyunyan, *Appl. Phys. Lett.*, 2012, **101**, 053119.
376 H. Chen, Y. Zhao, L. Shi, G. D. Li, L. Sun and X. Zou, *ACS Appl. Mater. Interfaces*, 2018, **10**, 29795–29804.
377 R. Lv, G. Chen, Q. Li, A. McCreary, A. Botello-Méndez, S. Morozov, L. Liang, X. Declerck, N. Perea-López and D. A. Cullen, *Proc. Natl. Acad. Sci. U. S. A.*, 2015, **112**, 14527–14532.
378 X. Wang, G. Sun, P. Routh, D.-H. Kim, W. Huang and P. Chen, *Chem. Soc. Rev.*, 2014, **43**, 7067–7098.

 This journal is © The Royal Society of Chemistry 2020

Published on 17 February 2020. Downloaded by NANJING TECH UNIVERSITY on 2/1/2025 6:08:03 AM.

379 L. Huang, Z. Wang, J. Zhang, J. Pu, Y. Lin, S. Xu, L. Shen, Q. Chen and W. Shi, *ACS Appl. Mater. Interfaces*, 2014, **6**, 7426–7433.
380 R. Ghosh, J. W. Gardner and P. K. Guha, *IEEE Trans. Electron Devices*, 2019, **66**, 3254–3264.
381 D. Zhang, Z. Wu and X. Zong, *Sens. Actuators, B*, 2019, **289**, 32–41.
382 D. Ponnamma, Q. Guo, I. Krupa, M. A. S. Al-Maadeed, K. Varughese, S. Thomas and K. K. Sadasivuni, *Phys. Chem. Chem. Phys.*, 2015, **17**, 3954–3981.
383 L. Zhang, Q. Tan, H. Kou, D. Wu, W. Zhang and J. Xiong, *Sci. Rep.*, 2019, **9**, 9942.
384 H. Tang, Y. t. Li, R. Sokolovskij, L. N. Sacco, H. Zheng, H. Ye, H. Yu, X. Fan, H. Tian and T. L. Ren, *ACS Appl. Mater. Interfaces*, 2019, **11**, 41850–41859.
385 W. Yuan, L. Huang, Q. Zhou and G. Shi, *ACS Appl. Mater. Interfaces*, 2014, **6**, 17003–17008.
386 D. Sun, Y. Luo, M. Debliquy and C. Zhang, *Beilstein J. Nanotechnol.*, 2018, **9**, 2832–2844.
387 H. Zheng, K. Feng, Y. Shang, Z. Kang, X. Sun and J. Zhong, *Inorg. Chem. Front.*, 2018, **5**, 1180–1187.
388 P. D. Tran, S. K. Batabyal, S. S. Pramana, J. Barber, L. H. Wong and S. C. J. Loo, *Nanoscale*, 2012, **4**, 3875–3878.
389 A. Al Nafiey, A. Addad, B. Sieber, G. Chastanet, A. Barras, S. Szunerits and R. Boukherroub, *Chem. Eng. J.*, 2017, **322**, 375–384.
390 M. Khan, M. N. Tahir, S. F. Adil, H. U. Khan, M. R. H. Siddiqui, A. A. Al-warthan and W. Tremel, *J. Mater. Chem. A*, 2015, **3**, 18753–18808.
391 Q. Q. Jia, H. m. Ji, D. H. Wang, X. Bai, X. H. Sun and Z. G. Jin, *J. Mater. Chem. A*, 2014, **2**, 13602–13611.
392 L. W. Ng, X. Zhu, G. Hu, N. Macadam, D. Um, T.-C. Wu, F. L. Moal, C. G. Jones and T. Hasan, 2018, arXiv preprint arXiv:1811.01073.
393 World Health Organization, WHO guidelines for indoor air quality: selected pollutants, 2010.
394 J.-K. Park, H.-J. Yee, K. S. Lee, W.-Y. Lee, M.-C. Shin, T.-H. Kim and S.-R. Kim, *Anal. Chim. Acta*, 1999, **390**, 83–91.
395 R. A. Potyrailo, *Chem. Rev.*, 2016, **116**, 11877–11923.
396 M. Amrani, K. Persaud and P. Payne, *Meas. Sci. Technol.*, 1995, **6**, 1500.
397 M. H. Amrani, P. A. Payne and K. C. Persaud, *Sens. Actuators, B*, 1996, **33**, 137–141.
398 M. H. Amrani, R. M. Dowdeswell, P. A. Payne and K. C. Persaud, *Sens. Actuators, B*, 1997, **44**, 512–516.
399 K. Shiba, R. Tamura, T. Sugiyama, Y. Kameyama, K. Koda, E. Sakon, K. Minami, H. T. Ngo, G. Imamura and K. Tsuda, *ACS Sens.*, 2018, **3**, 1592–1600.
400 V. Schroeder, E. D. Evans, Y.-C. M. Wu, C.-C. A. Voll, B. R. McDonald, S. Savagatrup and T. M. Swager, *ACS Sens.*, 2019, **4**, 2101–2108.

Science Bulletin 66 (2021) 2471–2478

Contents lists available at ScienceDirect

Science Bulletin

journal homepage: www.elsevier.com/locate/scib

Article

Suppression of Sn^{2+} and Lewis acidity in SnS_2/black phosphorus heterostructure for ppb-level room temperature NO_2 gas sensor

Tingting Liang [a], Zhengfei Dai [a,c,*], Yaoda Liu [a], Xu Zhang [a], Haibo Zeng [b,*]

[a] State Key Laboratory for Mechanical Behavior of Materials, Xi'an Jiaotong University, Xi'an 710049, China
[b] MIIT Key Laboratory of Advanced Display Materials and Devices, College of Materials Science and Engineering, Nanjing University of Science and Technology, Nanjing 210094, China
[c] Xi'an Jiaotong University Suzhou Academy, Suzhou 215123, China

ARTICLE INFO

Article history:
Received 10 February 2021
Received in revised form 15 April 2021
Accepted 25 May 2021
Available online 8 July 2021

Keywords:
Phosphorene
SnS_2
Interface engineering
Surface Lewis acidity
Nitrogen dioxide detection

ABSTRACT

The selective detection of harmful gases is of great significance to human health and air quality, triggering the need for special customizations of sensing material structure. In this study, we prepared a novel SnS_2/black phosphorus (BP) two-dimensional (2D)-2D heterostructure via the *in situ* hydrothermal growth of SnS_2 nanosheets on exfoliated BP lamellae for NO_2 sensing applications. In the SnS_2/BP composite, the holes with high oxidizability in p-type BP could oxidize Sn^{2+} into Sn^{4+}, thus inhibiting the formation of Lewis acidic S vacancies. This Sn^{2+}/Lewis acidity suppression of the composite was further confirmed by X-ray photoelectron spectroscopy and acidic double-layer capacitance analyses, and promoted the adsorption and detection of acidic NO_2. Owing to its valence and Lewis acidity engineering, the SnS_2/BP heterostructure sensor could detect trace levels of NO_2 as low as 100 ppb (parts per billion) with high response, fast response/recovery, good stability, and selectivity at room temperature. The high absorption energy of NO_2 (−0.74 eV), as indicated by the density functional theory calculations, suggests that NO_2 was chemically adsorbed on the SnS_2/BP surface, which was also evidenced by the *in situ* Raman spectroscopy results. This work opens up interesting opportunities for the rational design of highly efficient NO_2 gas sensors through Lewis acidity modification and interface engineering.

© 2021 Science China Press. Published by Elsevier B.V. and Science China Press. All rights reserved.

1. Introduction

As the major cause of acid rain and photochemical smog, nitrogen dioxide (NO_2) has become one of the greatest concerns in recent years [1,2]. National Institute of Occupational Safety and Health (NIOSH) has set 20 and 1 ppm (parts per million) as the immediately dangerous level and airborne exposure limits of NO_2, respectively [3,4]. The effective detection of sub-ppm NO_2 is of great significance to human health and air quality monitoring [5]. One promising technique to achieve this is to develop metal-oxide sensors, for which SnO_2 is extensively investigated owing to its favorable NO_2 sensing response, kinetics, and stability [6,7]. However, SnO_2 sensors usually operate at high temperature (greater than 300 °C) to achieve appreciable sensitivity and kinetics, which limits their wide-spread application [8]. To overcome this limitation, oxide-beyond sensing materials have been proposed for next-generation sensors [9,10]. Owing to their high edge-site activity, adjustable bandgap, and energetic gas absorption, two-dimensional (2D) layered metal disulfides are considered as promising materials for SnO_2 sensors [11,12]. Tin disulfide (SnS_2, n-type), which is analogous to SnO_2, is also promising candidate for gas sensor applications owing to its sensitivity to different gases [13]. However, SnS_2 shows sluggish reaction kinetics and poor sensitivity to NO_2, which limit its applications in NO_2 detection [14]. Hence, optimum surface and interface engineering should be taken into account to improve the NO_2 sensing properties of SnS_2 [15].

The charge transfer of SnS_2 can be accelerated by combining it with a conductive substrate (e.g., graphene or carbon nanotubes) [16]. For instance, Wu et al. [17] reported a SnS_2/graphene nanocomposite NO_2 sensor with shortened response/recovery times as the electrons could transfer through the interface. However, carbon substrates always show intrinsically poor sensitivity to gas molecules, which may present some difficulties in the full optimization of the sensing performance of the resulting composites. In addition, the formation of SnS_2 nanostructures (usually hydrothermal) is normally accompanied with the generation low-valence Sn^{2+} and incidental S vacancies (Vs″) from the reductive hydrothermal environment. As for NO_2 sensing, the Lewis acidic Vs″ are unfavorable for the adsorption of acidic NO_2 gas on the SnS_2 surface [18]. Hence, for the development of advanced

* Corresponding authors.
E-mail addresses: sensdai@mail.xjtu.edu.cn (Z. Dai), zeng.haibo@njust.edu.cn (H. Zen).

https://doi.org/10.1016/j.scib.2021.07.007
2095-9273/© 2021 Science China Press. Published by Elsevier B.V. and Science China Press. All rights reserved.

SnS_2 NO_2 sensors, it is imperative to suppress the generation of Sn^{2+} ions and Vs″ in SnS_2 as well as to develop novel carbon-beyond conductive/active host substrates. In terms of conductivity and activity, black phosphorus (BP), which is a 2D material with a narrow bandgap, is a potential candidate for carbon-beyond conductive/active substrates [19,20]. Moreover, holes with high oxidizability in the p-type BP can oxidize Sn^{2+} into Sn^{4+}, thus inhibiting the formation of Lewis acidic Vs″ in SnS_2 for better NO_2 sensing. However, the BP-SnS_2 synergy and hole-injection effect of the composites of BP and SnS_2 for NO_2 sensing have not been investigated till date.

In this study, a SnS_2/BP 2D-2D heterostructure was constructed through the *in situ* hydrothermal growth of SnS_2 nanosheets on exfoliated BP lamellae for application in NO_2 gas sensors. The SnS_2/BP composite showed significantly suppressed formation of low-valence Sn^{2+} ions and Lewis acidic S vacancies, as revealed by the X-ray photoelectron spectroscopy (XPS) and double-layer capacitance (C_{dl}) analyses. The SnS_2/BP material showed improved NO_2 sensing performances as compared to the bare SnS_2 and BP counterparts. The composite showed low detection limit (100 ppb, parts per billion), high response (6.08), short response/recovery time (18/280 s), and good stability. Density functional theory (DFT) calculations were performed to investigate the mechanism underlying the improved sensing performance of the composite. This indicates that the NO_2 adsorption ability of the SnS_2/BP heterostructure was stronger than that of SnS_2, and the p-n junction between SnS_2 and BP afforded interfacial electron transfer for efficient NO_2 sensing response and kinetics. The adsorption energy (ΔE_{ads}, −0.74 eV) of NO_2 on SnS_2/BP was more negative than that of the other gases. This value along with the *in situ* Raman spectroscopy results suggested the selective chemisorption of NO_2 on the substrate. This work paves the pathway for the rational design of highly efficient NO_2 gas sensors through Lewis acid modification and 2D-2D interface engineering.

2. Experimental

2.1. Chemicals

Bulk BP crystals (99%), tetrabutylammonium tetrafluoroborate (TBAB, 98%), tin tetrachloride pentahydrate ($SnCl_4 \cdot 5H_2O$, 99%), L-cysteine (99%), dimethyl sulfoxide (DMSO, 99.9%), ethanol (99.9%) were purchased from Sinopharm.

2.2. Exfoliation process of BP

BP nanosheets were obtained by using an electrochemical exfoliation method as shown in Fig. S1 (online). DMSO (50 mL) dissolved in 300 mg TBAB was used as the electrolyte. A two-electrode system (BP bulk as the cathode and Pt wire as the anode) was used for the expansion of bulk BP at −10 V in the electrolyte. Then, the sponge-like BP was further exfoliated and dispersed via ultrasonication using a biomixer for 7 min at power of 270 W, and a working and stop time of 5 s. The obtained BP suspension was first separated at 2000 r/min for 5 min to remove large BP particles. Then, the top suspension was thoroughly washed several times at 10,000 r/min for 10 min with absolute ethanol to remove the residual electrolyte. The precipitate obtained was then collected after drying in a vacuum oven at 60 ℃ for 6 h.

2.3. Synthesis of the SnS_2/BP heterostructure

In a typical synthesis procedure, 30 mg BP nanosheets, $SnCl_4 \cdot 5H_2O$ (195 mg), and L-cysteine (125 mg) were dissolved in 35 mL ethanol. After vigorous stirring for 30 min, the solution was transferred into a 50 mL Teflon-lined autoclave and heated at 180 °C for 16 h. Then the autoclave was allowed to cool down the solution to room temperature naturally. The resulting brown product was collected, rinsed with deionized water and ethanol repeatedly, and finally dried at 60 °C for 6 h. The synthesis process of pure SnS_2 was similar to that of SnS_2/BP, except that the BP nanosheets were not added.

2.4. Characterization

The morphologies of the samples were examined using field emission scanning electron microscopy (SEM, FEI Verios 460). Transmission electron microscopy (TEM, JEM-2100F) with energy dispersive X-ray spectroscopy (EDX) was utilized to investigate the morphologies, crystal structures, and elemental distributions of the samples. Atomic force microscopy (AFM, Dimension Icon) was used to determine the thickness of BP. X-ray diffraction (XRD, PANalytical X'Pert Pro) was used to determine the crystalline phase compositions of the materials. The Raman spectra of the samples were recorded on a Horiba HR800 spectrometer with a 532 nm laser as the excitation source. XPS (Thermo Fisher Scientific ESCALAB Xi+) was employed to investigate the surface valence states and compositions of the samples.

2.5. Gas sensing measurements

Au interdigital electrodes (200 μm spacing, 8 pairs with a microheater underneath) were used to investigate the gas-sensing performance of the samples. A Pt wire was linked to such an electrode to build a sensor. First, 30 mg of the sample was uniformly dispersed in 1 mL of ethanol under ultrasonication. Then, 2 μL of the dispersed sensing materials was dropped on the electrode with a pipette and dried naturally. Finally, the gas-sensing performance of the electrode was tested using a dynamic gas-sensing system. The gas concentrations were controlled by adjusting the flow rates of the target gas and dry air, while maintaining the flow rate of the gas mixture constant at 100 standard cubic centimeter per minute (sccm). A Keithley DMM6500 multimeter was employed to record the resistance variations of the sensors during the gas-sensing experiments.

2.6. Electrochemical test

The catalytic ink was prepared by dispersing 5 mg of the samples in a mixture of 0.9 mL ethanol, 0.08 mL ultrapure water, and 0.02 mL Nafion. Then, 5 μL of the ink was placed uniformly on a glass-carbon electrode (3 mm diameter) and dried slowly. The C_{dl} values of the electrodes were determined using an Autolab PGSTAT204 station with a conventional three-electrode setup in 0.5 mol/L H_2SO_4. The C_{dl} measurements within the potential range of 0.166–0.264 V vs. normal hydrogen electrode (RHE) were detected at the scan speed from 20 to 120 mV/s. The C_{dl} was calculated by plotting half of the current density difference between the positive and negative scans (Δj) at −0.216 V vs. RHE against the scan rate. The Mott-Schottky plots of the samples were also measured in 0.5 mol/L Na_2SO_4 electrolyte.

2.7. DFT calculations

All the electronic calculations were carried out using the DFT with the projector augmented wave method using the Vienna ab simulation package (VASP). The generalized gradient approximation of the Perdew-Burke-Ernzerhof electronic exchange–correlation functional was used for the interacting electrons [21]. The van der Waals interaction was considered by Grimme (DFT-D3) and the local density approximation for reference. A kinetic energy

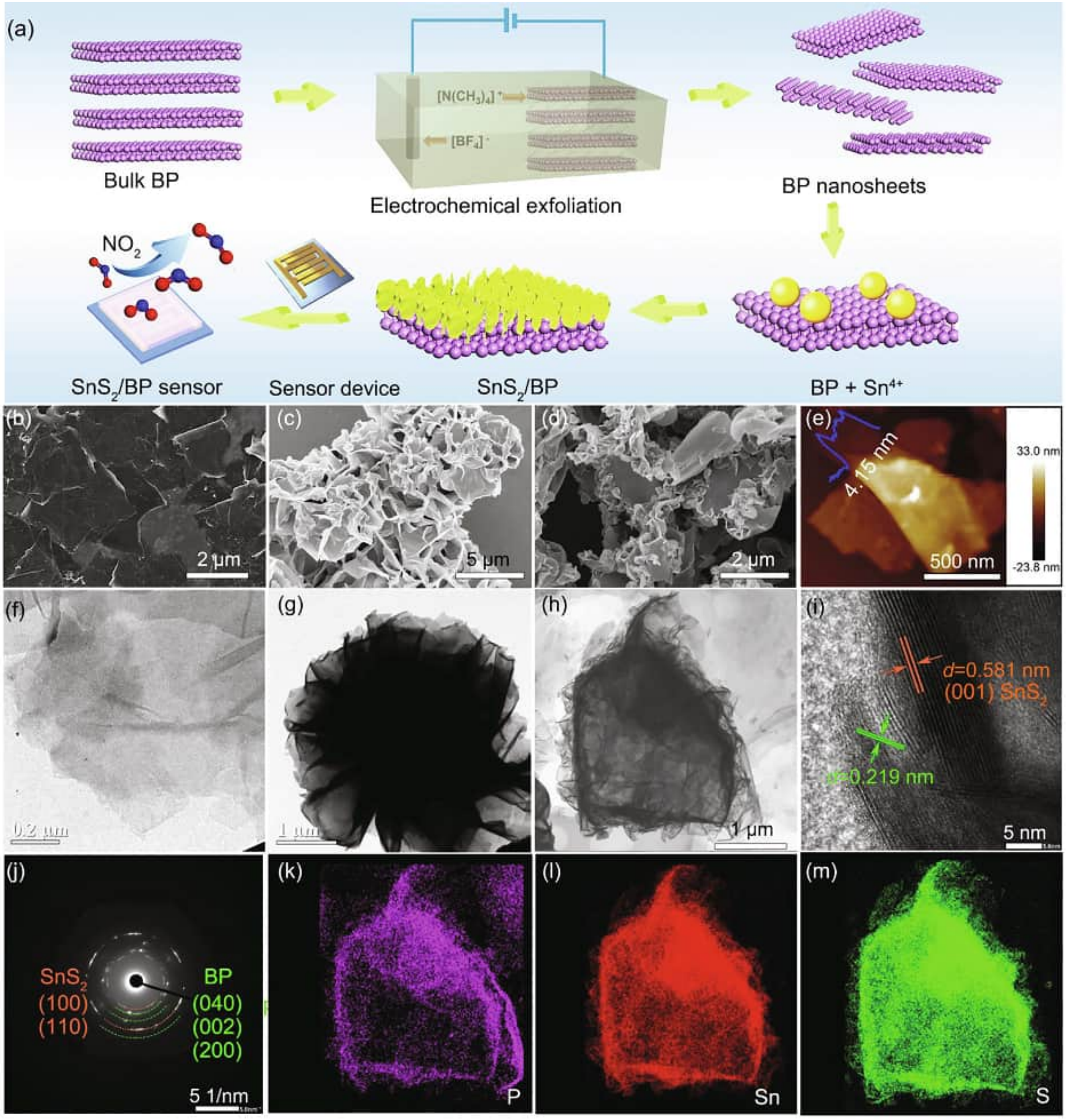

Fig. 1. Synthesis and microstructure of the SnS_2/BP heterostructure. (a) Synthesis process. (b–d) SEM images of the bare (b) BP, (c) SnS_2 nanosheets, and (d) SnS_2/BP composite. (e) AFM image of BP. (f–h) TEM images of the (f) bare BP, (g) SnS_2 nanosheets, and (h) SnS_2/BP composite, (i) HRTEM image and (j) SAED pattern of SnS_2/BP. (k–m) EDX mapping of SnS_2/BP.

cut-off of 450 eV was selected for plane-wave basis set expansion. The k-point sampling of the Brillouin zone was set as 1 × 1 × 1 Monkhorst-Pack grid. The spin-orbital coupling effect was included by employing spin polarized calculations (collinear, ISPIN = 2) during all the simulations. All the structures in this work were fully relaxed using the conjugate gradient method. The Hellmann-Feynman forces acting on each atom (EDIFF) were less than 0.01 eV/Å, and the convergence criterion for the energy difference (EDIFFG) between the two consecutive steps was less than 10^{-5} eV.

To evaluate the stability of gas absorption on the sensing materials, the absorption energy (E^*_{ads}) was calculated using Eq. (1)

$$E^*_{ads} = E^*_{sub+gas} - E^*_{sub} - E^*_{gas}, \quad (1)$$

where the E^*_{gas} is the energy of the bare gas, E^*_{sub} is the energy of the substrate (sensing materials), and $E^*_{sub+gas}$ is the total energy of the adsorbed gas molecule system [22].

3. Results and discussion

3.1. Morphological and structural characterizations

The schematic for the synthesis process of the SnS_2/BP heterostructure is shown in Fig. 1a. Bulk BP was first electrochemically exfoliated for high-yield preparation of thin BP microlamellae (Fig. S1 online) [23]. SnS_2 nanosheets were grown *in situ* on the surface of the BP lamellae using a hydrothermal method to prepare the final SnS_2/BP heterostructure (Fig. 1a). In addition, the bare SnS_2 control sample was also synthesized in the absence of BP via similar hydrothermal route. The NO_2 sensor device could be fabricated by loading the SnS_2/BP material onto an interdigital electrode (spacing of 3 μm, Fig. S2 online). Morphologies and structures of the samples were studied by SEM, AFM, and TEM. Fig. 1b–d show the SEM images of the as-prepared BP nanosheets, flower-

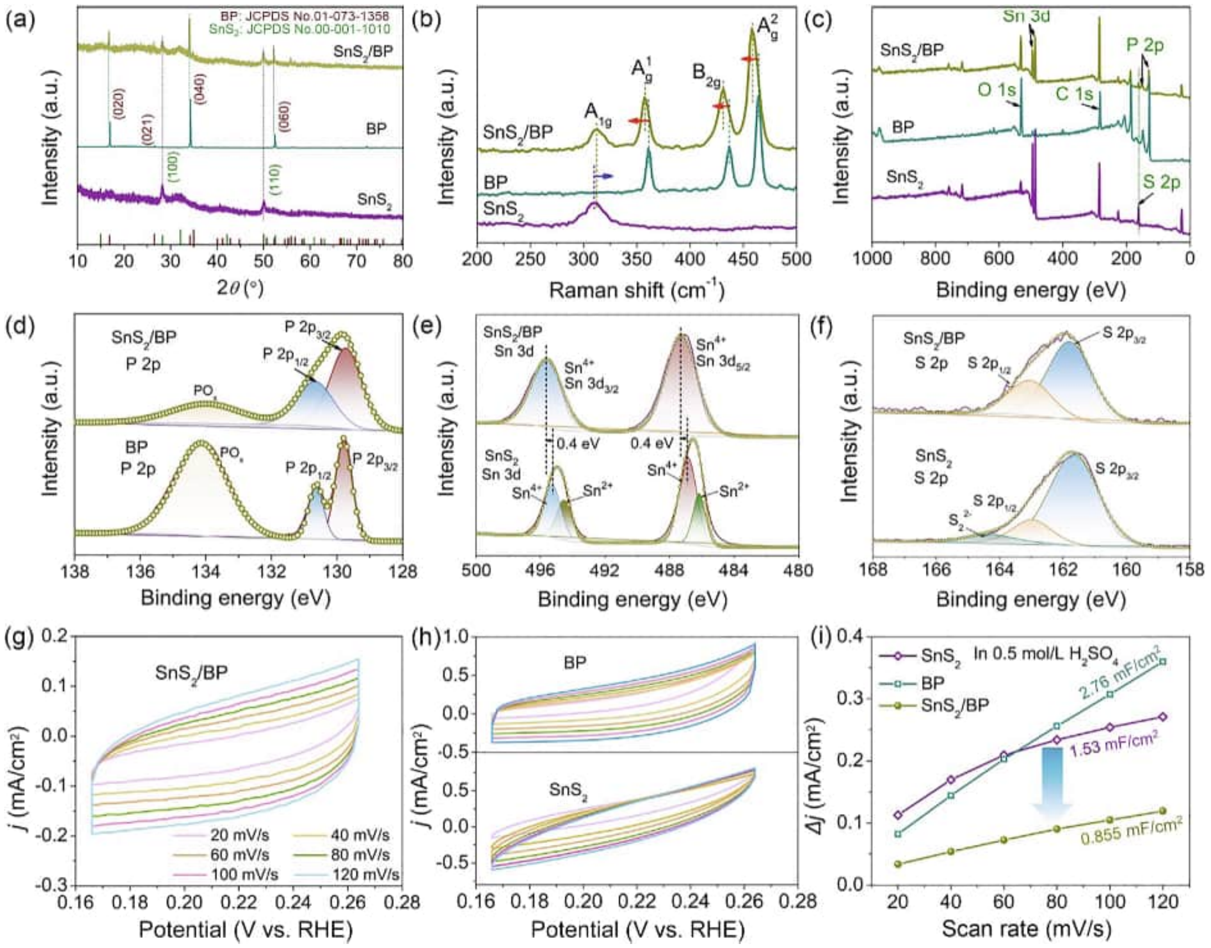

Fig. 2. XRD patterns (a), Raman spectra (b), and XPS survey profiles (c) of the bare BP, SnS_2, and SnS_2/BP samples. (d) P 2p XPS profiles of SnS_2/BP and BP. Sn 3d (e) andS 2p (f) XPS profiles of SnS_2/BP and SnS_2. (g, h) Cyclic voltammetry curves of BP, SnS_2, and SnS_2/BP in acid electrolyte at different scan rates in the non-Faradaic region. (i) Charging current density (at −0.216 V vs. RHE) of the samples as a function of the scan rate.

like SnS_2 nanosheets, and SnS_2/BP heterostructure, respectively. The electroexfoliated 2D BP nanosheets had a lateral size of approximately 2 μm (Fig. 1b) and a thickness of ca. 4.15 nm (Fig. 1e). The SnS_2/BP composite (Fig. 1d) consisted of thin SnS_2 nanosheets grown on the surface of the BP nanosheets.

The TEM images of the BP, SnS_2 nanosheet, and SnS_2/BP composite are shown in Fig. 1f–h. The structural characteristics observed from the TEM images of the samples were consistent with those observed from SEM images. Fig. 1i shows the high-resolution TEM (HRTEM) image of the SnS_2/BP heterostructure. The interplanar distances of 0.581 and 0.219 nm can be ascribed to the (001) plane of SnS_2 and the (002) plane of BP, respectively [24,25]. Moreover, the lattice fringes shown in Fig. 1i and Fig. S3 (online) were continuous and well-distinguished, indicating the presence of highly crystalline BP and SnS_2 phases in the composite. The lattice relationship between the BP and SnS_2 crystal domains indicated by the white dotted lines in Fig. S3 (online) suggested that the SnS_2/BP heterostructure was formed via a chemical growth process [26]. The selected-area electron diffraction (SAED, Fig. 1j) pattern of the composite also revealed the presence of both the SnS_2 ((100) and (110)) and BP ((040), (002), and (200)) crystal planes. In addition, the EDX mapping (Fig. 1k–m) results revealed that P (orange), Sn (red), and S (green) were uniformly distributed in the composite. The morphological images and EDS results of the control samples, BP, and SnS_2 are shown in Figs. S4–S6 (online). These results confirm the formation of the SnS_2/BP heterostructure.

The phase and chemical states of the samples were further investigated using XRD, Raman spectroscopy, and XPS, respectively. Fig. 2a shows the XRD pattern of SnS_2/BP, where the peaks located at 16.3°, 34.2°, 35.0°, and 52.5° correspond to the (020), (040), (111), and (060) crystal planes of BP (JCPDS No. 01-073-1358) [26], while the peaks located at 15.03°, 28.3°, 31.6°, and 50.2° correspond to the (001), (100), (101), and (110) crystal planes of SnS_2 (JCPDS No. 01-1010) [27]. The SnS_2/BP heterostructure showed the XRD peaks of both BP and SnS_2. Raman spectra of SnS_2/BP (Fig. 2b) showed the characteristic Raman peaks of both SnS_2 (A_{1g}, 312.66 cm^{-1}) and BP (A_g^1, 360.51 cm^{-1}, B_{2g}, 431.39 cm^{-1}, and A_g^2, 457.97 cm^{-1}) [28]. The red shift of the BP Raman peaks and the blue shift of the SnS_2 peaks indicated the covalent binding and charge transfer of BP-SnS_2 [29]. Fig. S7 (online) shows the Raman mapping of the SnS_2/BP heterostructure, indicating that SnS_2 was distributed along the edges of the BP nanosheets. This is consistent with the SEM result shown in Fig. 1d.

The XPS profiles of the BP, SnS_2, and SnS_2/BP samples (Fig. 2c) showed XPS peaks corresponding to P 2p, S 2p, Sn 3d, C 1s, and O 1s. The binding energies obtained from the XPS profiles were corrected for specimen charging by referencing the C 1s line to 284.8 eV. Fig. 2d–f show the P 2p, Sn 3d, and S 2p core-level XPS profiles of the samples, respectively. The P 2p XPS profile of the SnS_2/BP heterostructure (Fig. 2d) showed double peaks located at 129.6 and 130.5 eV, corresponding to P $2p_{3/2}$ and P $2p_{1/2}$, respectively [30]. The peak located at 134.0 eV corresponds to PO*x* species, which are commonly observed in phosphorus-based

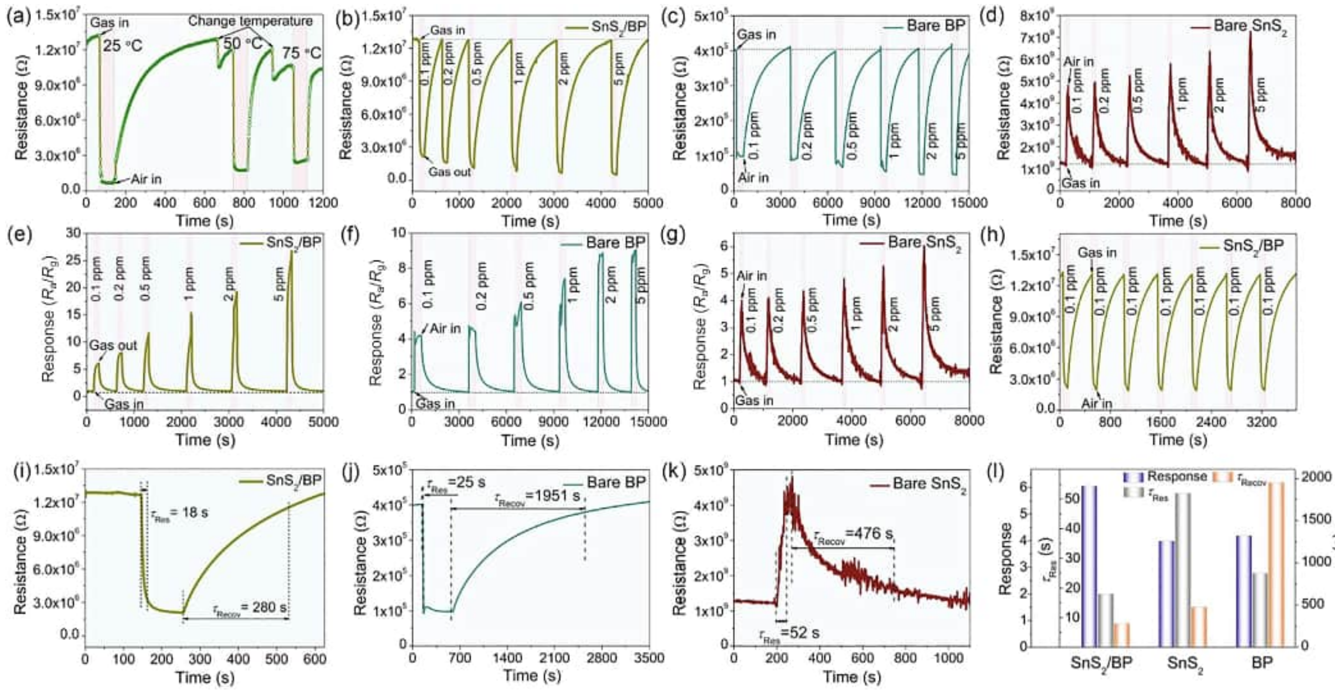

Fig. 3. NO_2 sensing properties of the SnS_2/BP, bare BP, and SnS_2 materials. (a) Resistance transients of SnS_2/BP to 5 ppm NO_2 at different temperatures. (b–d) Resistance and (e–g) R_a/R_g transients of SnS_2/BP, bare BP, and SnS_2 to 0.1–5 ppm NO_2 at 25 °C, respectively. (h) Sensing signals for SnS_2/BP to 100 ppb NO_2 over 6 cycles. (i–k) Response of SnS_2/BP, bare BP, and SnS_2 to 100 ppb NO_2 at 25 °C. (l) Summary of the sensing response, response/recovery time for different samples to 100 ppb NO_2 at 25 °C.

materials because of the surface oxidation in ambient air [31]. The Sn 3d profile of SnS_2/BP (Fig. 2e) showed a doublet with peaks located at 487.2 and 495.7 eV corresponding to Sn $3d_{5/2}$ and Sn $3d_{3/2}$, respectively, indicating the presence of Sn^{4+} [32]. The Sn 3d peaks of pure SnS_2 shifted towards lower binding energies, and another doublet could be deconvoluted into the peaks at 494.6 and 486.2 eV corresponding to Sn^{2+} [33]. This was mainly due to the use of a reductive sulfur source (L-cysteine) that could reduce some Sn^{4+} ions to Sn^{2+} ions. This positive peak shift of SnS_2/BP relative to the bare SnS_2 indicates the higher electron-donating ability of the heterostructure.

Moreover, the suppressed formation of Sn^{2+} in the heterostructure is attributed to the contribution from BP. The hole in the p-type semiconducting BP with high oxidizability facilitated the oxidation of Sn^{2+} to Sn^{4+} during the SnS_2 growth process. Without the hole-injection effect, Sn^{4+} could possibly be reduced to the low-valence Sn^{2+} ($Sn^{4+} \rightarrow Sn^{2+} + V_s''$), leading to the generation of S vacancies (V_s''). This was also evidenced by the S 2p spectrum of the SnS_2 sample (Fig. 2f). In addition to the S $2p_{3/2}$ (161.8 eV) and S $2p_{1/2}$ (163.1 eV) peaks, an additional peak located at 164.3 eV was observed, indicating the presence of S vacancies (S_2^{2-} species) in the SnS_2 sample [34]. For NO_2 sensing, the formation of the Lewis acidic Vs″ was unfavorable for the adsorption and sensing of acidic NO_2 gas. The electrochemical double-layer capacitance (C_{dl}) of the samples in an acidic electrolyte was determined to understand their surface acidity better (Fig. 2g, h). The C_{dl} of SnS_2/BP (0.855 mF/cm^2, Fig. 2i) was lower than those of BP (2.76 mF/cm^2) and SnS_2 (1.53 mF/cm^2). In the acidic electrolyte, a positive charge (H^+) was adsorbed onto the surfaces of the negatively charged SnS_2 or BP [35,36], forming a Stern electric double layer. In the Stern model, the material surface acidity is directly related to the interactions with the charges in the electrolyte. The more acidic surface of SnS_2/BP absorbed less positive charge and leaded to low C_{dl}, as illustrated in Fig. S8 (online). Thus, the proposed SnS_2/BP heterostructure with suppressed of Sn^{2+} and Vs″ formation is a promising candidate for NO_2 sensing applications.

3.2. Gas sensing performance

The gas-sensing behaviors of the samples are shown in Fig. 3. The sensing responses of the samples were calculated using the material resistance variation (R_a/R_g for p-type material, R_g/R_a for n-type material) in a dynamic gas-sensing system. Here, R_a and R_g are the sensor resistances in air and gas ambient, respectively. First, the resistance transient of SnS_2/BP was tested at 5 ppm NO_2 at different temperatures (Fig. 3a). It was obviously found that the resistance variation at room temperature (25 °C) was higher than those at 50 and 75 °C. This can be explained by the balance between gas adsorption and desorption rates [15]. At higher temperatures, the accelerated surface NO_2 desorption process resulted in a decrease in the sensing response. Thus, the gas sensing tests were performed at 25 °C. Fig. 3b–d show the resistance transients of the SnS_2/BP, bare BP, and SnS_2 samples exposed to 0.1–5 ppm NO_2 at 25 °C, respectively. With respect to SnS_2/BP (Fig. 3b), the decreased resistance of the samples upon NO_2 exposure featured a typical p-type material sensing behavior similar to that of the bare BP (Fig. 3c). On the other hand, the SnS_2 sample showed a typical n-type sensing behavior with increased resistance to NO_2 gas (Fig. 3d) [32]. The Mott-Schottky curves shown in Fig. S9 (online) also confirm the semiconducting behavior of the SnS_2/BP, bare BP, and SnS_2 [37].

Fig. 3e–g show the R_a/R_g response transients of the SnS_2/BP, bare BP, and SnS_2 sensors to 0.1–5 ppm NO_2 at 25 °C, respectively. The sensor responses increased with an increase of the NO_2 concentration (Fig. 3e), and the R_a/R_g responses transients for 100 ppb and 5 ppm NO_2 were 6.08 and 26.5, respectively. The corresponding response value for the SnS_2/BP sensor as a function of the NO_2 concentration is shown in Fig. S10 (online). Fig. S11 (online) shows the responses of the SnS_2/BP sensor to 10, 20, 50, 80, and 100 ppm NO_2. As can be observed, the response increased with an increase of the NO_2 concentration over the 10–100 ppm range. For 5 ppm NO_2, the bare BP and SnS_2 samples showed the response transients of 8.9 (Fig. 3f) and 6.0 (Fig. 3g), respectively, which were

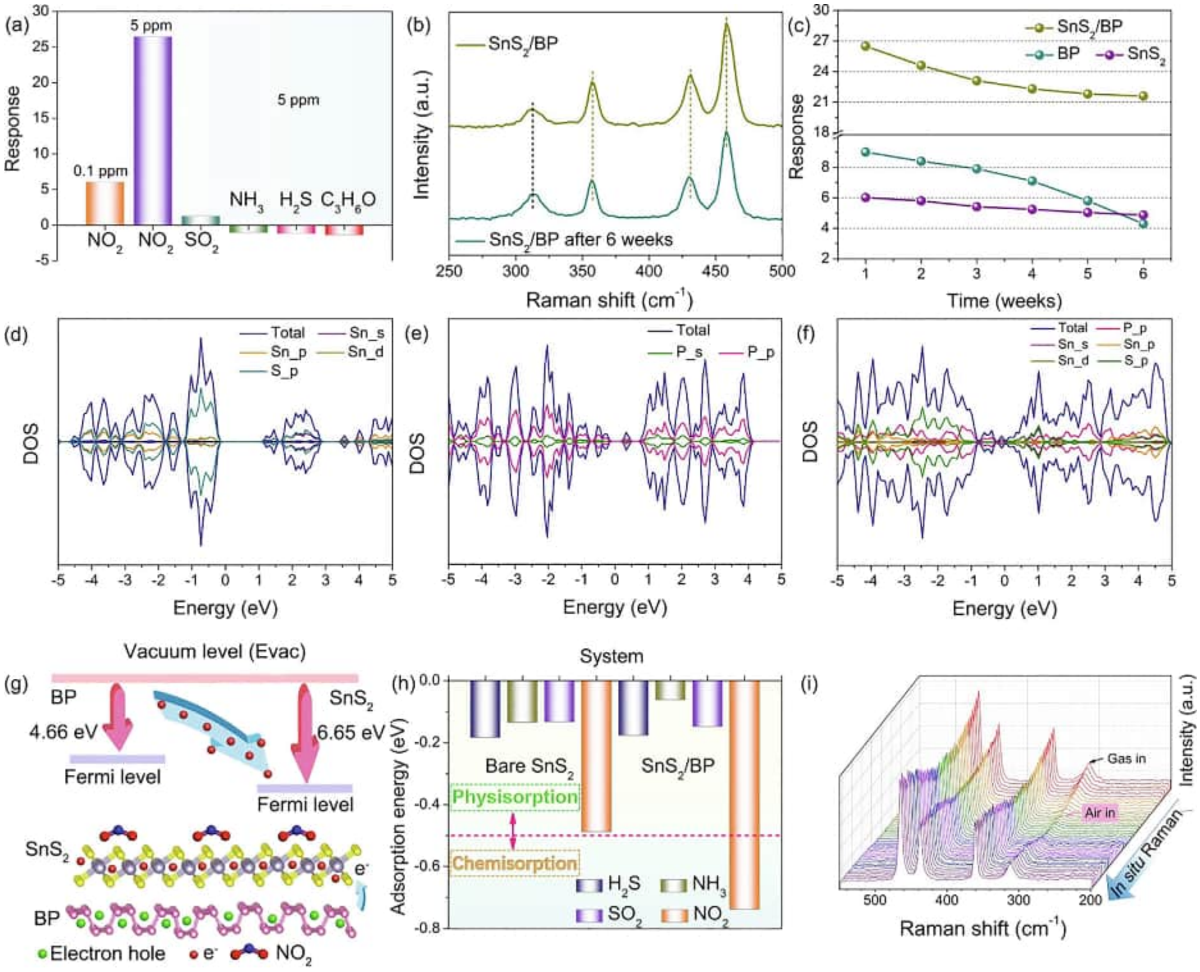

Fig. 4. Selectivity, stability, and mechanism studies for SnS_2, BP, and SnS_2/BP sensors. (a) Response of the SnS_2/BP heterostructure for different gases at 25 °C. (b) Raman spectra of the SnS_2/BP heterostructure before and after six weeks. (c) Response of SnS_2/BP and BP to 5 ppm NO_2 gas for six weeks at 25 °C. (d–f) DOS of (d) SnS_2, (e) BP, and (f) SnS_2/BP, respectively. (g) Sensing mechanism of the SnS_2/BP exposed to NO_2 gas. (h) Adsorption energy of different gases on SnS_2 and SnS_2/BP. (i) *In situ* Raman spectra during the NO_2 sensing process.

much lower than that of the SnS_2/BP heterostructure (26.5). This improvement in the NO_2 sensing performance of the heterostructure can be understood by using the Lewis acid-base principle [18]. Positively charged Vs″ can be classified as Lewis acid because of its electron-accepting characteristic. The SnS_2/BP heterostructure was characterized by suppressed Sn^{2+} and Vs″ formation, featuring high surface Lewis basicity, and hence enhanced adsorption of acidic NO_2 gas. Furthermore, the sensing signals of SnS_2/BP to 100 ppb NO_2 over six cycles are shown in Fig. 3h, demonstrating the outstanding recyclability of the heterostructure. Moreover, the response and recovery times of the SnS_2/BP, BP, and SnS_2 samples exposed to 100 ppb NO_2 are shown in Fig. 3i–k, respectively. The response and recovery time of SnS_2/BP were 18 and 280 s, respectively, which are much faster than those of the bare BP (25 and 1951 s) and SnS_2 (52 and 476 s) sensors, as shown in Fig. 3l.

The selectivity of the SnS_2/BP heterostructure was evaluated by comparing its responses to different gases (Fig. 4a). The response of the SnS_2/BP heterostructure to NO_2 was much higher than the absolute value of responses exposed to 5 ppm SO_2, NH_3, H_2S, and acetone. The changes in the resistances of the SnS_2/BP sensor exposed to various types of gases are shown in Fig. S12 (online). It can be observed that the SnS_2/BP sensor showed only slight resistance changes when exposed to 5 ppm SO_2, NH_3, H_2S, and C_3H_6O, demonstrating excellent selectivity to NO_2 gas. The SnS_2/BP heterostructure showed huge potential for practical applications owing to its excellent sensing performance, including high response, low detected limitation concentration, fast response/recovery speed, and apparent selectivity. Furthermore, Fig. S13 and Table S1 (online) compare the NO_2 gas-sensing performances of the SnS_2/BP sensor with those of the other SnS_2- and BP-based sensors, demonstrating the excellent sensing behavior of SnS_2/BP. In addition, we compared the performances of SnS_2/BP and other sensing materials, as shown in Fig. S14 (online). Owing to its high response and fast kinetics at 25 °C, the SnS_2/BP-based sensor exhibited superior NO_2 sensing performance to other sensors. The stability of the SnS_2/BP heterostructure used as the NO_2 sensor was also investigated as shown in Fig. 4b, c. The Raman spectrum of SnS_2/BP after six weeks is shown in Fig. 4b, in which the characteristic Raman peaks were well-maintained even after six weeks, indicating the good structural stability of the SnS_2/BP heterostructure [38]. In addition, the sensing performance of the SnS_2/BP sensor was reevaluated after six weeks of aging at 25 °C and 60% relative humidity (Fig. S15 online). The sensor exhibited good stability with high response retention (ca. 85%) after six weeks. Fig. 4c shows the response evolutions of SnS_2/BP, SnS_2, and BP to 5 ppm NO_2 gas over six weeks. The heterostructure showed more stable NO_2 sensing than BP and SnS_2.

To investigate the mechanism underlying the improved sensing performance of the heterostructure sensor, DFT calculations were performed using VASP software [39]. The supercells of bare BP

(7 × 3), SnS_2 (5 × 3), and heterostructure (Fig. S16 online) models were stably constructed. The mismatch between the BP and SnS_2 layers is less than 5%. All the thicknesses of the vacuum slab were set at more than 20 Å to effectively avoid the interlayer interactions between the adjacent periodic images. The density of states (DOS) of SnS_2, BP, and SnS_2/BP within the energy window of −5–5 eV are shown in Fig. 4d–f, repectively. As for SnS_2, the valence band maximum (VBM) is mainly dominated by S 3p states, whereas the conduction band minimum (CBM) predominantly consists of the hybrid orbitals of the S 3p and Sn 5s states. As for SnS_2/BP, the VBM was mainly governed by P 3p, whereas the CBM predominantly consisted of the hybridized orbital of the P 3p and S 3p states. In contrast to the DOS values of SnS_2 and BP, the DOS near the Fermi level of SnS_2/BP was not zero, indicating that the electrons near the Fermi level could easily move to the CBM. The corresponding band structures of SnS_2, BP, and SnS_2/BP were calculated and are shown in Fig. S17 (online). Interestingly, the SnS_2/BP p-n junction showed a type-Ⅱ band structure (Fig. 4g), which could effectively inhibit the electron-hole recombination and improve the sensing performance [40]. During the NO_2 sensing, the NO_2 gas molecules accepted electrons from the n-type SnS_2 and formed the NO_2-adsorber. In addition, BP with a lower work function (Fig. S18 online) transferred the electrons to SnS_2 to further sustain the surface NO_2 adsorption and sensing. Furthermore, the band diagrams of BP and SnS_2 are shown in Fig. S19 (online). It is clear that the CBM of BP was more negative than that of SnS_2, suggesting that the electron transfer occurred from BP to SnS_2. The calculated adsorption energies (ΔE_{ads}) of the SnS_2/BP and SnS_2 systems exposed to different gases are shown in Fig. 4h and Figs. S20, S21 (online). The adsorption energy of NO_2 molecules on SnS_2/BP is −0.74 eV, indicating that strong chemisorption of NO_2 molecules onto the SnS_2/BP heterostructure ($\Delta E_{ads} > -0.5$ eV). Thus, SnS_2/BP showed significantly improved sensing response as compared to bare SnS_2 [41]. The more negative ΔE_{ads} of SnS_2/BP for NO_2 than those for other gases also explains the NO_2 gas selectivity of the SnS_2/BP heterostructure (Fig. 4h).

To further understand the adsorption behavior of small gas molecules on the bare SnS_2 and SnS_2/BP sensors, their DOS within the energy window of −5–5 eV was analyzed (Figs. S22, S23 online). In the case of the NH_3- and NO_2-adsorbed SnS_2/BP systems, the spin-up and spin-down DOS were symmetric, indicating that SnS_2/BP was non-magnetic system [42]. For the H_2S-, and SO_2-adsorbed SnS_2/BP systems, the spin-up and spin-down DOS were anti-symmetric, showing magnetic characteristics. In addition, the valence band of SnS_2/BP was contributed by the P 3p orbitals, while the conduction band is contributed by the mixture of P 3p, S 3p, and Sn 5s orbitals. The structure of the SnS_2/BP heterostructure improved its conduction band, thus improving its sensing properties. Furthermore, the *in situ* Raman spectra of SnS_2/BP obtained during the NO_2 sensing process are shown in Fig. 4i. The vibration mode of SnS_2/BP did not change during the gas-sensing process because of its stable structure. Notably, the vibration intensity change suggested the occurrence of rapid electron exchange between the gas and the material during the sensing process (Fig. S24 online) [43]. The A_{1g} peaks shift to higher wavenumbers after the exposure to NO_2, indicating the chemical bonding between the gas and SnS_2/BP material (Fig. 4i), which is consistent with the DFT calculation results (Fig. 4h).

4. Conclusion

In summary, the SnS_2/BP heterostructure was constructed through the *in situ* growth of SnS_2 nanosheets on electrochemically exfoliated BP lamellae for application in room-temperature NO_2 sensing. Compared to pure SnS_2, the SnS_2/BP composite showed suppressed formation of low-valence Sn^{2+} and Lewis-acidic S vacancies, which improved its NO_2 sensing properties, as evidenced by the XPS and C_{dl} analyses. As a result, the NO_2 sensing capability of the SnS_2/BP heterostructure was superior to those of the SnS_2 and BP nanosheets in terms of the selectivity, response, and response/recovery rate. The heterostructure showed a NO_2 detection limit as low as 100 ppb with a response of 6.08, fast response (18 s) and recovery (280 s), and good sensor stability. The *in situ* Raman spectroscopy measurements carried out during the sensing process revealed that NO_2 was chemisorbed on the SnS_2/BP surface, which improved the sensing performance of the heterostructure. Furthermore, the DFT calculations revealed that the excellent sensing properties of the SnS_2/BP composite were attributed to the strong NO_2 chemisorption ability and improved electron transfer at the SnS_2-BP interface. This work provides a novel strategy for the design and fabrication of highly efficient gas sensors based on metal chalcogenide/BP heterostructures through Lewis acidity modification and 2D-2D interface engineering.

Conflict of interest

The authors declare that they have no conflict of interest.

Acknowledgments

This work was supported by the National Natural Science Foundation of China (51802252), Natural Science Foundation of Shaanxi Province (2020JM-032), Natural Science Foundation of Jiangsu Province (BK20180237), and 111 Project 2.0 (BP0618008). We thank Miss Liu at Instrument Analysis Center of Xi'an Jiaotong University for her assistance with XPS analysis. This research used the resources of the HPCC platform in Xi'an Jiaotong University.

Author contributions

Zhengfei Dai supervised this work. Tingting Liang performed the experiments and DFT calculation. Tingting Liang, Yaoda Liu, Xu Zhang, and Haibo Zeng contributed to the interpretation of the results and wrote the manuscript.

Appendix A. Supplementary materials

Supplementary materials to this article can be found online at https://doi.org/10.1016/j.scib.2021.07.007.

References

[1] Pham T, Li G, Bekyarova E, et al. MoS_2-based optoelectronic gas sensor with sub-parts-per-billion limit of NO_2 gas detection. ACS Nano 2019;13:3196–205.
[2] Zhang X, Wang B, Huang L, et al. Breath figure-derived porous semiconducting films for organic electronics. Sci Adv 2020;6:eaaz1042.
[3] Su T-Y, Chen Y-Z, Wang Y-C, et al. Highly sensitive, selective and stable NO_2 gas sensors with a ppb-level detection limit on 2D-platinum diselenide films. J Mater Chem C 2020;8:4851–8.
[4] Azizi A, Dogan M, Long Hu, et al. High-performance atomically-thin room-temperature NO_2 sensor. Nano Lett 2020;20:6120–7.
[5] Guo J, Wen R, Zhai J, et al. Enhanced NO_2 gas sensing of a single-layer MoS_2 by photogating and piezo-phototronic effects. Sci Bull 2019;64:128–35.
[6] Yuan T, Li Z, Zhang W, et al. Highly sensitive ethanol gas sensor based on ultrathin nanosheets assembled Bi_2WO_6 with composite phase. Sci Bull 2019;64:595–602.
[7] Liu W, Gu D, Li X. Detection of ppb-level NO_2 using mesoporous ZnSe/SnO_2 core-shell microspheres based chemical sensors. Sens Actuators B 2020;320:128365.
[8] Kim J-H, Mirzaei A, Kim HW, et al. Improving the hydrogen sensing properties of SnO_2 nanowire-based conductometric sensors by Pd-decoration. Sens Actuators B 2019;285:358–67.
[9] Xiong Ya, Xu W, Ding D, et al. Ultra-sensitive NH_3 sensor based on flower-shaped SnS_2 nanostructures with sub-ppm detection ability. J Hazard Mater 2018;341:159–67.

[10] Zhao D, Huang H, Chen S, et al. *In situ* growth of leakage-free direct-bridging gan nanowires: application to gas sensors for long-term stability, low power consumption, and sub-ppb detection limit. Nano Lett 2019;19:3448–56.
[11] Kwon KC, Suh JM, Lee TH, et al. SnS_2 nanograins on porous SiO_2 nanorods template for highly sensitive NO_2 sensor at room temperature with excellent recovery. ACS Sensors 2019;4:678–86.
[12] Ou JZ, Ge W, Carey B, et al. Physisorption-based charge transfer in two-dimensional SnS_2 for selective and reversible NO_2 gas sensing. ACS Nano 2015;9:10313–23.
[13] Patil SG, Tredgold RH. Electrical and photoconductive properties of SnS_2 crystals. J Phys D 1971;4:718–22.
[14] Sun Q, Wang J, Hao J, et al. SnS_2/SnS p-n heterojunctions with an accumulation layer for ultrasensitive room-temperature NO_2 detection. Nanoscale 2019;11:13741–9.
[15] Ping J, Fan Z, Sindoro M, et al. Recent advances in sensing applications of two-dimensional transition metal dichalcogenide nanosheets and their composites. Adv Funct Mater 2017;27:1605817.
[16] Huang Y, Jiao W, Chu Z, et al. Ultrasensitive room temperature ppb-level NO_2 gas sensors based on SnS_2/rGO nanohybrids with p-n transition and optoelectronic visible light enhancement performance. J Mater Chem C 2019;7:8616–25.
[17] Wu J, Wu Z, Ding H, et al. Flexible, 3D SnS_2/reduced graphene oxide heterostructured NO_2 sensor. Sens Actuators B 2020;305:127445.
[18] Choudhuri I, Sadhukhan D, Garg P, et al. Lewis acid-base adducts for improving the selectivity and sensitivity of graphene based gas sensors. ACS Sensors 2016;1:451–9.
[19] Yan S, Song H, Wan LF, et al. Hydroxyl-assisted phosphorene stabilization with robust device performances. Nano Lett 2020;20:81–7.
[20] Zhou W, Zhang S, Wang Y, et al. Anisotropic in-plane ballistic transport in monolayer black arsenic-phosphorus fets. Adv Electron Mater 2020;6:1901281.
[21] Perdew JP, Burke K, Ernzerhof M. Generalized gradient approximation made simple. Phys Rev Lett 1996;77:3865–8.
[22] Li G, Zhang H, Meng L, et al. Adjustment of oxygen vacancy states in ZnO and its application in ppb-level NO_2 gas sensor. Sci Bull 2020;65:1650–8.
[23] Li J, Chen C, Liu S, et al. Ultrafast electrochemical expansion of black phosphorus toward high-yield synthesis of few-layer phosphorene. Chem Mater 2018;30:2742–9.
[24] Zhou X, Hu X, Zhou S, et al. Tunneling diode based on WSe_2/SnS_2 heterostructure incorporating high detectivity and responsivity. Adv Mater 2018;30:1703286.
[25] Lin M-L, Leng Y-C, Cong X, et al. Understanding angle-resolved polarized Raman scattering from black phosphorus at normal and oblique laser incidences. Sci Bull 2020;65:1894–900.
[26] Liang T, Liu Y, Cheng Y, et al. Scalable synthesis of a MoS_2/black phosphorus heterostructure for pH-universal hydrogen evolution catalysis. ChemCatChem 2020;12:2840–8.
[27] Pyeon JJ, Baek I-H, Song YG, et al. Highly sensitive flexible NO_2 sensor composed of vertically aligned 2D SnS_2 operating at room temperature. J Mater Chem C 2020;8:11874–81.
[28] Chen H, Chen Y, Zhang H, et al. Suspended SnS_2 layers by light assistance for ultrasensitive ammonia detection at room temperature. Adv Funct Mater 2018;28:1801035.
[29] Shi F, Geng Z, Huang K, et al. Cobalt nanoparticles/black phosphorus nanosheets: an efficient catalyst for electrochemical oxygen evolution. Adv Sci 2018;5:1800575.
[30] Wang Z, Liu H, Ge R, et al. Phosphorus-doped Co_3O_4 nanowire array: a highly efficient bifunctional electrocatalyst for overall water splitting. ACS Catal 2018;8:2236–41.
[31] Liang T, Liu Y, Zhang P, et al. Interface and valence modulation on scalable phosphorene/phosphide lamellae for efficient water electrolysis. Chem Eng J 2020;395:124976.
[32] Huang Y, Jiao W, Chu Z, et al. SnS_2 quantum dot-based optoelectronic flexible sensors for ultrasensitive detection of NO_2 down to 1 ppb. ACS Appl Mater Interfaces 2020;12:25178–88.
[33] Baek I-H, Pyeon JJ, Lee G-Y, et al. Cation-regulated transformation for continuous two-dimensional tin monosulfide. Chem Mater 2020;32:2313–20.
[34] Kibsgaard J, Jaramillo TF, Besenbacher F. Building an appropriate active-site motif into a hydrogen-evolution catalyst with thiomolybdate $[Mo_3S_{13}]^{2-}$ clusters. Nat Chem 2014;6:248–53.
[35] Paul F, William RR, Richard L, et al. Chemistry. Texas: OpenStax; 2016.
[36] Yuan Z, Li J, Yang M, et al. Ultrathin black phosphorus-on-nitrogen doped graphene for efficient overall water splitting: dual modulation roles of directional interfacial charge transfer. J Am Chem Soc 2019;141:4972–9.
[37] Zhang P, Liu Y, Liang T, et al. Nitrogen-doped carbon wrapped Co-Mo_2C dual Mott-Schottky nanosheets with large porosity for efficient water electrolysis. Appl Catal B 2021;284:119738.
[38] Pyeon JJ, Baek I-H, Lee WC, et al. Wafer-scale, conformal, and low-temperature synthesis of layered tin disulfides for emerging nonplanar and flexible electronics. ACS Appl Mater Interfaces 2020;12:2679–86.
[39] Kresse G, Joubert D. From ultrasoft pseudopotentials to the projector augmented-wave method. Phys Rev B 1999;59:1758–75.
[40] Zheng W, Xu Y, Zheng L, et al. MoS_2 van der waals p-n junctions enabling highly selective room-temperature NO_2 sensor. Adv Funct Mater 2020;30:2000435.
[41] Zhao R, Wang T, Zhao M, et al. A theoretical simulation of small-molecules sensing on an S-vacancy SnS_2 monolayer. Phys Chem Chem Phys 2017;19:10470–80.
[42] Guo SY, Wang YY, Hu XM, et al. Ultrascaled double-gate monolayer SnS_2 mosfets for high-performance and low-power applications. Phys Rev Appl 2020;14:044031.
[43] He R, Hua J, Zhang A, et al. Molybdenum disulfide-black phosphorus hybrid nanosheets as a superior catalyst for electrochemical hydrogen evolution. Nano Lett 2017;17:4311–6.

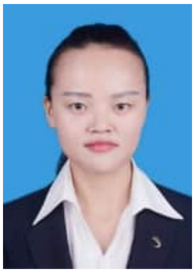

Tingting Liang received her B.S. and M.S. degrees from School of Materials Science and Engineering, Henan University of Science and Technology in 2014 and 2018, respectively. Now she is a doctoral candidate of Xi'an Jiaotong University, and she is conducting research on the fabrication and performance of gas sensor devices using nano-structured materials.

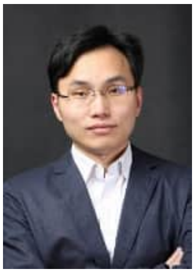

Zhengfei Dai is a research professor of Materials Science and Engineering, Xi'an Jiaotong University. He received his Ph.D. degree from University of Chinese Academy of Sciences in 2013. Then, he worked at Department of Materials Science & Engineering, Korea University. Since 2014, he had worked at Department of Chemistry, Kyoto University as a JSPS research fellow. From 2016, he has worked as a research fellow at Department of Materials Science & Engineering, Nanyang Technological University. He has been a faculty member at Xi'an Jiaotong University since 2017. His research interest focuses on gas-involved reactions in electrocatalysis and molecular sensing.

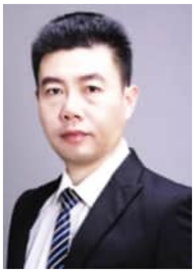

Haibo Zeng is a professor at Material Science and Optics, Nanjing University of Science and Technology (NJUST). He received his Ph.D. degree in Material Physics in 2006 from the Institute of Solid State Physics, Chinese Academy of Sciences, and then worked at University of Karlsruhe (Germany) in 2007 as a visiting scholarship and at the National Institute for Materials Science (Japan) as a JSPS researcher fellow from 2008 to 2011. In 2011, he joined NJUST and then founded the Institute of Optoelectronics & Nanomaterials and the MIIT Key Laboratory of Advanced Display Materials & Devices. His research group focuses on nano-optoelectronics, including quantum dots and two-dimensional materials, their theoretical design, controlled synthesis, light-emitting diodes and photodetectors.

ACS APPLIED MATERIALS & INTERFACES

www.acsami.org Research Article

Superior NO_2 Sensing of MOF-Derived Indium-Doped ZnO Porous Hollow Cages

Zhou Li, Yong Zhang, Hong Zhang, Yong Jiang, and Jianxin Yi*

Cite This: ACS Appl. Mater. Interfaces 2020, 12, 37489−37498 Read Online

ACCESS | Metrics & More | Article Recommendations | Supporting Information

ABSTRACT: Highly sensitive semiconductor gas sensors hold great potential for applications in trace gas detection. Reliable detection of ppb-level NO_2 is crucial for environmental monitoring, which however still remains a challenge. In this work, we demonstrated ultrahigh NO_2 sensitivity of indium-doped ZnO porous hollow cages. Doping of In into ZnO was accomplished via a facile one-pot MOF encapsulation—calcination route, which led to remarkably enhanced NO_2 sensing performance. In-doped ZnO exhibited a large response of 3.7 to 10 ppb NO_2, an ultrahigh sensitivity of 187.9 ppm^{-1}, and a limit of detection of 0.2 ppb, outperforming state-of-the-art ZnO-based NO_2 sensors. The superior NO_2 sensing properties were attributed to a synergy of excellent gas accessibility of the porous hollow structure, abundant adsorption sites, and electronic sensitization by In doping. Our findings could be extended to design other porous doped ZnO oxides for high performance gas sensors and other applications.

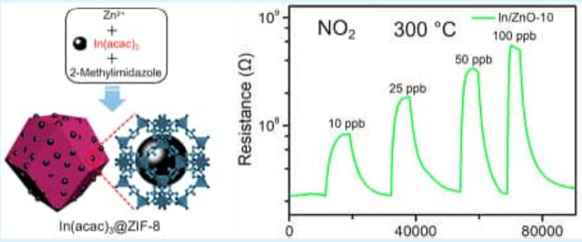

KEYWORDS: *gas sensor, NO_2 detection, MOF, ZnO, porous structure*

Downloaded via NANJING TECH UNIV on February 1, 2025 at 06:09:54 (UTC).
See https://pubs.acs.org/sharingguidelines for options on how to legitimately share published articles.

1. INTRODUCTION

As a prominent toxic pollutant gas with pungent odor, nitrogen dioxide (NO_2), which is mainly generated from the combustion of fossil fuels and industrial production activities, has received great attention due to its potential hazards to environmental safety and human health.[1] The excessive emission of NO_2 causes various environmental problems such as acid rain, photochemical smog, and PM 2.5 (particulate matter smaller than 2.5 μm). Exposure to NO_2 can put people at high risk for respiratory diseases and heart problems. The World Health Organization (WHO) requires the NO_2 level in cities to be less than 200 $\mu g/m^3$ (106 ppb) over 1 h average and 40 $\mu g/m^3$ (21 ppb) averaged over the whole year.[2] Meanwhile, NO_2 generated by human metabolic activity is a diagnostic biomarker. The assistant diagnosis of human diseases including chronic obstructive pulmonary diseases can be achieved efficiently, simply by detecting the NO_2 content in exhaled gases.[3] Therefore, achieving highly sensitive and selective detection of the trace level NO_2 is of great significance for environmental protection and personal safety.

Chemiresistive gas sensors, primarily based on metal oxide semiconductors (MOSs), such as In_2O_3, SnO_2, Fe_2O_3, WO_3, and ZnO, play a predominant role in gas detecting devices for domestic, lab, and commercial applications, owing to their high sensitivity, fast response speed, low cost, and excellent portability.[4] As a representative MOS sensing material, ZnO has been widely studied for the detection of various gases including NO_2. However, for most ZnO-based sensors, the limit of detection (LOD) is still at a high level and the sensitivity known as resolution ratio is not adequate to accurately monitor the variations of low-concentration NO_2.[5] To achieve high gas-sensing performance, the use of porous micro- and nanostructures has been proven to be one of the most effective ways. In particular, materials of porous hollow structure are of great interest owing to their large surface area and high gas accessibility.[6] Alternatively, high gas sensing performance can also be achieved via selective doping. Sensors based on ZnO doped with metal elements such as Sn, Al, and In had been found to exhibit higher sensitivity, better selectivity toward the target gas, or shorter response/recovery time.[7−9] Using successive ionic layer adsorption and reaction, Shishiyanu et al. obtained Sn-doped ZnO films with higher response to NO_2 at 150 °C.[7] Navale et al. fabricated Al-doped ZnO nanoparticles via a coprecipitation process, which exhibited enhanced response to NO_2 with less interference from other gases including ethanol.[8] Ferro et al. found that In-doped ZnO films, synthesized by a spray pyrolysis method, were more responsive to NO_2 compared with pure ZnO film.[9] Although different methods have been utilized to obtain doped ZnO, the accompanying dopant aggregation and particle agglomeration limit further enhancement of the sensing performance.[10] Difficulties in achieving uniform distribution

Received: June 9, 2020
Accepted: July 9, 2020
Published: July 9, 2020

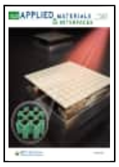

© 2020 American Chemical Society

Scheme 1. Synthesis Procedure for Pristine and in-Doped ZnO Cages[a]

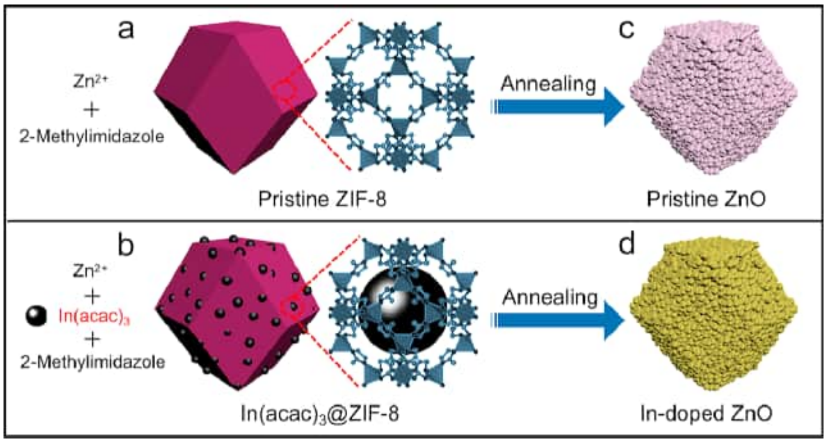

[a](a) Pure ZIF-8; (b) $In(acac)_3$@ZIF-8; (c) pristine ZnO cage; and (d) In-doped ZnO cage.

of doping elements while maintaining highly porous and gas-accessible structure still remain to be settled.

Zeolitic imidazolate frameworks (ZIFs), a subclass of widely known metal−organic frameworks (MOFs), are an emerging class of porous materials composed of metal nodes and imidazole-derivatives linkers.[11] Among all kinds of ZIFs, ZIF-8 (Zn), consisted of zinc and 2-methylimidazole forming sodalite topology, has been most extensively investigated mainly because of its robust synthesis protocols, excellent thermal/chemical stability, and well-defined porosity.[12,13] These unique advantages of ZIF-8 have greatly promoted its applications in various research fields including gas separation, gas storage, and catalysis.[14−16] A widely studied capacity of ZIF-8 is constraining noble metal nanoparticles, such as Pd and Pt, within its cavities.[17] When it comes to developing novelty MOS based sensing materials, ZIF-8 was usually used a sacrificial template to obtain highly porous noble metal loaded ZnO.[18,19] Koo et al. embedded ZIF-8-derived Pd-loaded ZnO nanocubes into WO_3 nanofibers and achieved excellent toluene sensitivity.[18] ZIF-8-derived Pt-loaded ZnO was loaded into In_2O_3 nanofibers and high response to acetone was obtained by Guo et al.[19] However, complex multistep synthesizing process and expensive noble metals are the major obstacles for practical application. Therefore, using selective doping strategy with a facile and noble-metal-free synthetic approach could be a promising alternative. Additionally, to our best knowledge, ZIF-8 derived doped ZnO materials with superior NO_2 sensing properties have not been reported yet.

In this work, we adopted a one-pot encapsulation strategy for synthesizing indium-doped ZnO porous hollow cages. $In(acac)_3$ was selected as the indium source due to its molecular diameter (9.9 Å) between the cavity size (11.6 Å) and the aperture size (3.4 Å) of ZIF-8, indicating that it can be effectively isolated in the cavities. Accordingly, during the crystallization progress of ZIF-8, it was successfully in situ trapped in the cavities and $In(acac)_3$@ZIF-8 composites were formed. During calcination, metal ion species, composed of $In(acac)_3$@ZIF-8, were oxidized, leading to the formation of In-doped ZnO porous hollow cages. In this case, ZIF-8 served as the host backbone for the uniform distribution of the indium source as well as the production of porous hollow structure. In doping led to significantly enhanced NO_2-sensing performance, achieving a response of 3.7 to 10 ppb NO_2, a high sensitivity of 187.9 ppm^{-1}, and a limit of detection as low as 0.2 ppb. The mechanism of enhanced NO_2-sensing properties was discussed in the terms of the intrinsic excellent gas accessibility of porous hollow structure and electronic sensitization by In doping. Moreover, our facile and effective synthetic strategy could be further extended to other doped-ZnO materials.

2. EXPERIMENTAL SECTION

2.1. Synthesis of Materials. Chemical reagents were purchased from Sinopharm Chemical Reagent Co., Ltd., China. As illustrated in Scheme 1, $In(acac)_3$@ZIF-8 composites were synthesized by a one-pot encapsulation method. Briefly, 2.7 mmol (810 mg) of zinc nitrate hexahydrate ($Zn(NO_3)_2{\cdot}6H_2O$) was dissolved in 40 mL of methanol. One of the prepared zinc nitrate methanol solutions was used for the preparation of pristine ZIF-8 (Scheme 1a). By adding 0.054 mmol (22.9 mg), 0.162 mmol (68.7 mg), and 0.27 mmol (114.5 mg) of Indium(III) acetylacetonate ($Inacac)_3$) into three other prepared zinc nitrate methanol solutions separately with stirring, precursor solutions with In/Zn of 2 at. %, 6 at. %, and 10 at. % were obtained (Scheme 1b). Then, prepared 2-methylinidazole methanol solutions containing 8.8 mmol (721.6 mg) of 2-methylinidazole in 40 mL methanol were separately added to the above four solutions. After stirring for 20 min, the resulting solutions were kept at room temperature (26 ± 1 °C) for 24 h. Precipitates in the beakers were collected by centrifugation, thoroughly washed with methanol, and further dried at 60 °C for 24 h. The obtained samples are hereafter denoted as $In(acac)_3$@ZIF-8-*X*, where *X* represents the nominal In/Zn atomic ratio (multiplied by 100) used during the synthesis, e.g., $In(acac)_3$@ZIF-8−2 for In/Zn of 2 at. % and $In(acac)_3$@ZIF-8−10 for In/Zn of 10 at. %. Note that while preparing precursors with higher In/Zn of 15%, 20%, and 30%, $In(acac)_3$ could not be dissolved completely into methanol, leading to formation of irregular particles at the micron level after calcination.

2.2. Characterization. Crystal structures of as-prepared samples were analyzed by powder X-ray diffraction (XRD, TTR III) using Cu Kα radiation. Microstructure of the samples was investigated by scanning electron microscopy (SEM, Gemini SEM 500) equipped with an energy-dispersive X-ray spectrometer (EDX), and transmission electron microscopy (TEM, Talos F200X). X-ray photoelectron spectroscopy (XPS) was performed on an ESCLAB 250 spectrometer using Al Kα as an excitation source. The specific surface area and the pore size distribution were obtained by using Brunauer−Emmett−Teller (BET) and Barrett−Joyner−Halenda (BJH) method based on the corresponding N_2 adsorption/desorption isotherms studied by Tristar II 3020M.

2.3. Fabrication and Measurement of the Gas Sensor. A 40-mg portion of as-prepared pristine ZIF-8 or $In(acac)_3$@ZIF-8 composites were ultrasonically dispersed in 1.6 mL of 1-butanol to conduct uniform suspensions with an concentration of 25 $g{\cdot}L^{-1}$

https://dx.doi.org/10.1021/acsami.0c10420
ACS Appl. Mater. Interfaces 2020, 12, 37489−37498

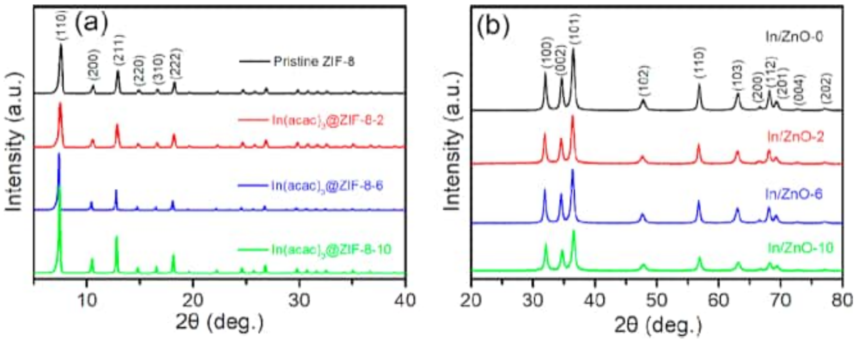

Figure 1. (a) XRD patterns of pristine ZIF-8 and $In(acac)_3$@ZIF-8 composites. (b) XRD patterns of pristine and In-doped ZnO porous cages.

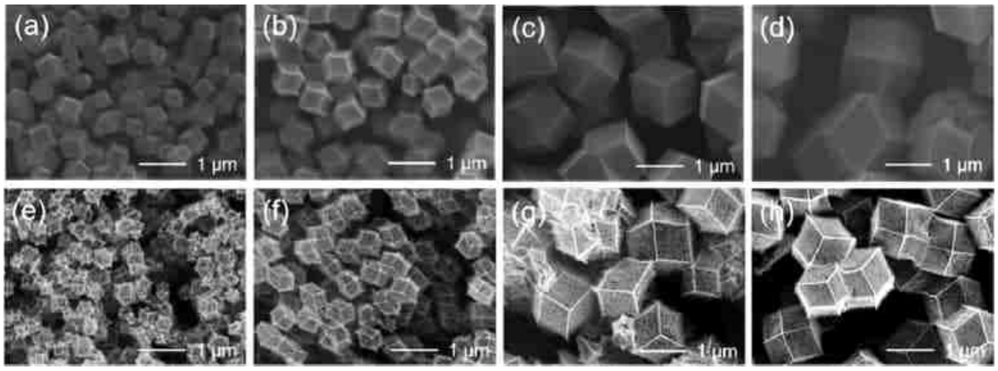

Figure 2. Typical SEM images of (a) pristine ZIF-8, (b) $In(acac)_3$@ZIF-8−2, (c) $In(acac)_3$@ZIF-8−6, (d) $In(acac)_3$@ZIF-8−10, (e) In/ZnO-0, (f) In/ZnO-2, (g) In/ZnO-6, and (h) In/ZnO-10.

separately. Subsequently, 40 μL of the prepared suspensions were dropped on an Al_2O_3 flat ceramic substrate (8 × 10 mm^2) with Au interdigital electrodes (electrode width of 200 μm and separation of 200 μm) on its top surface. After drying at room temperature (26 ± 1 °C) and 60 °C for 2 and 1 h, respectively, ceramic substrates coated with as-prepared samples were annealed at 500 °C for 2 h in air to remove the organic components (Scheme 1c,d). The resulting sensors, which were transformed from ceramic substrates coated with pristine ZIF-8, $In(acac)_3$@ZIF-8−2, −6, and −10, respectively, were hereafter denoted as In/ZnO-0, -2, -6, and -10. Before gas-sensing measurements, all the obtained sensors were aged at 400 °C for 5 days to achieve stable performance.

Sensor measurement was conducted on a homemade setup. Four sensors were fixed on a ceramic heating plate (Xinxin, Beijing) inside a sealed stainless steel container with a volume of ~170 mL. The working temperature of sensors was controlled by adjusting the voltage applied to the heating element with a DC power supply (Querli, Shanghai), and monitored with a thermocouple (Omega, U.S.A.). The direct current two-point probe resistance of the sensor was measured with a Keithley 6482 picoammeter controlled by Labview software. Certified analyte gases (Nanjing Specialty Gases Co., Ltd.) were introduced into the air stream for measurements of the sensing performance. The gas flow rates were controlled by MFC (CS200, Sevenstar Electronics, Beijing), and the total flow rate was kept at 200 sccm. For recovery, the flow of analyte gases was cut off so that the sensors were exposed to air again. At optimum sensing conditions, the effect of humidity was studied by varying the ambient humidity at 11% RH (saturated solution of LiCl), 43% RH (saturated solution of K_2CO_3) and 94% RH (deionized water). The response value was defined as Response = R_g/R_a (NO_2) or R_a/R_g (all the tested reducing gases), where R_a and R_g are the electrical resistance of the sensor in air and test gas, respectively. In addition, the sensitivity, which is defined as the slope of the response change (ΔR) of sensors versus the concentration change (ΔC) of gas molecules, can be also used as a parameter for evaluating gas-sensing capability: Sensitivity = $\Delta R/\Delta C$.[20] The limit of detection (LOD) is determined as the lowest concentration at which the response is 3-fold higher than the standard deviation (δ_{noise}) of the baseline: LOD = 3 × rms_{noise}/S, where rms_{noise} is the root-mean-square noise of the sensors, and S is the sensitivity ($\Delta R/\Delta C$).[20] The response (recovery) time was the time that the resistance variation reached 90% of the total value after introduction (removal) of the analyte gas.

3. RESULTS AND DISCUSSION

3.1. Materials Properties and Microstructure. Figure 1a shows the XRD patterns of the as-prepared pristine ZIF-8 and $In(acac)_3$@ZIF-8 composites. The strong diffraction peaks at 7.30, 10.35, 12.70, 14.80, 16.40, and 18.00°, which can be respectively indexed to planes (110), (200), (211), (220), (310), and (222), implied the high crystallinity of pristine ZIF-8. For composite samples, the phase structure of ZIF-8 remained unchanged and no extra peaks from any impurity phase were observed, indicating that the introduction of $In(acac)_3$ had no effect on forming crystal structure of ZIF-8. The XRD patterns of pristine and In-doped ZnO were shown in Figure 1b. All the diffraction peaks for pristine ZnO can be indexed to a hexagonal wurtzite structure (JCPDS No. 36−1451) The phase structure remained almost unchanged for the samples doped with In, and no extra peak from any impurity phase was observed. This observation suggested that In had been successfully doped into ZnO and did not significantly segregate. No evident shift of the peaks was observed with In doping mainly due to the low content of In.

Typical SEM images of the as-prepared pristine ZIF-8, $In(acac)_3$@ZIF-8 composites, and pristine and In-doped ZnO cages are presented in Figure 2. For pristine ZIF-8, nearly monodisperse rhombic dodecahedral particles were observed (Figure 2a). For the $In(acac)_3$@ZIF-8 composites, similar geometric structure with a significant increase in particle size

https://dx.doi.org/10.1021/acsami.0c10420
ACS Appl. Mater. Interfaces 2020, 12, 37489−37498

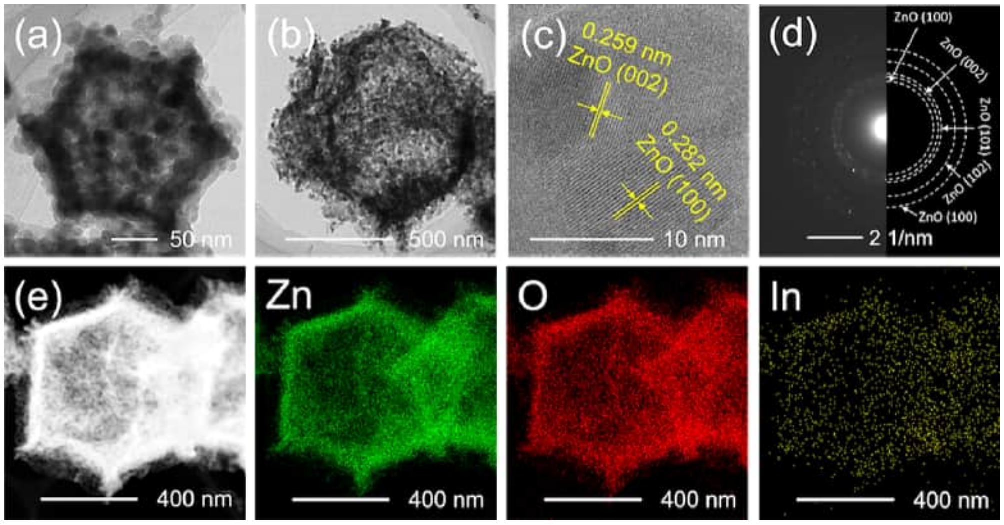

Figure 3. TEM analysis of pristine ZnO and In/ZnO-10. (a) Low-magnification TEM image of pristine ZnO; (b) low-magnification TEM, (c) HRTEM image, (d) SAED pattern, and (e) HADDF image and element mapping of In/ZnO-10.

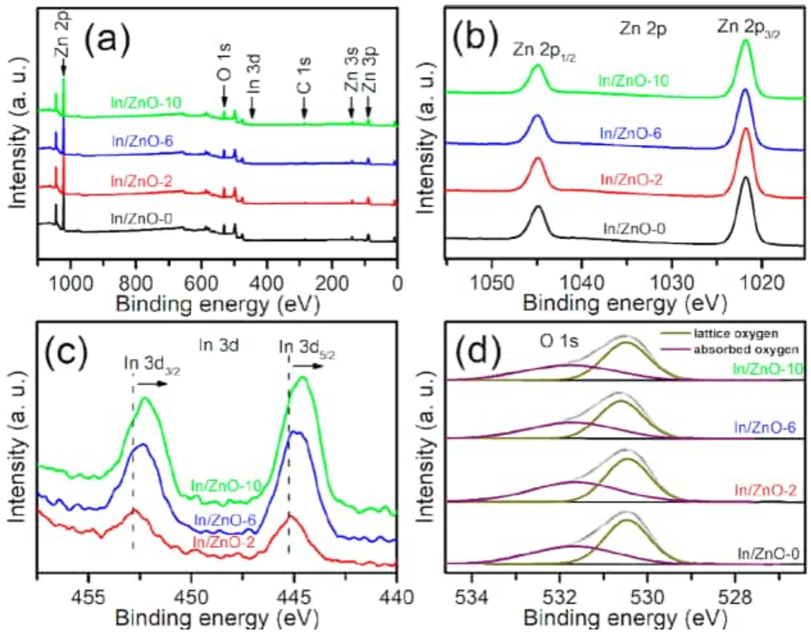

Figure 4. XPS spectra of pristine and In-doped ZnO samples. (a) Survey spectrum, and high resolution spectra for (b) Zn 2p, (c) In 3d, and (d) O 1s.

with the increasing In(acac)$_3$ concentration was identified (Figure 2b–d). After thermal treatment, whereas the overall sizes of the particles remained similar, the surface morphology changed significantly (Figure 2e–h). Specifically, the organic components were removed and porous cages were obtained by annealing. In addition, EDX analyses revealed that In-doped ZnO samples were mainly constituted of Zn and O containing extremely low content of In element (Figure S1 of the Supporting Information, SI). In/Zn atomic ratios for In/ZnO-2, In/ZnO-6, and ZnO-10 were 0.4%, 0.5%, and 0.8%, respectively. To evaluate the porosity, the BET surface areas and the BJH pore sizes were calculated based on the corresponding N_2 adsorption/desorption isotherms. As shown in Figure S2, the specific surface area increased monotonically from 23.1 m^2/g for pristine ZnO to 28.7 m^2/g for In/ZnO-10 while the pore size slightly fluctuated between 16.4 and 17.6 nm.

More in-depth microstructural analysis was performed on pristine ZnO and In/ZnO-10 samples with TEM, as shown in Figure 3. TEM images showed that the obtained samples presented very similar porous and hollow structures, despite the different sizes (Figure 3a, b). Each cage was a homogeneous assembly of nanometer-sized crystalline grains. A decrease in average crystalline size from 16.0 nm for pristine ZnO to 13.5 nm for In/ZnO-10 indicated In doping suppressed the grain growth during thermal treatment, which agreed well with previous reports.[21,22] HRTEM observation presented interplanar spacing of 0.282 and 0.259 nm (Figure 3c), corresponding to the (002) and (100) planes of ZnO, respectively. Accordingly, SAED analysis showed that all the diffraction rings or spots belonged to ZnO, and no evidence for

https://dx.doi.org/10.1021/acsami.0c10420
ACS Appl. Mater. Interfaces 2020, 12, 37489–37498

presence of In_2O_3 or any other impurity phase could be observed (Figure 3d). HADDF image also clearly exhibited the porous and hollow feature of In/ZnO-10 (Figure 3e). EDX mapping of In/ZnO-10 revealed homogeneous distribution of In within the cage framework of Zn and O elements.

To assess the composition and chemical states of the elements in the samples, XPS studies were carried out. Zn and O were found in all the samples, and In was also detected for In-doped samples (Figure 4a). No impurity element but carbon contamination was observed. The binding energy for the C 1s peak at 284.8 eV was used as a reference for energy calibration. For pristine ZnO sample, two strong peaks of Zn $2p_{1/2}$ and Zn $2p_{3/2}$ were found at the binding energy of 1044.9 and 1021.8 eV, respectively, indicating an oxidation state of +2 for Zn (Figure 4b). Within the experimental errors, the peak positions of Zn 2p remained almost unchanged for the In-doped ZnO samples (Table S1). As seen in Figure 4c, the two peaks of In 3d were shifted toward lower binding energy with increasing In content (Table 1). This may be attributed to the

Table 1. Deconvolution Data of In 3d and O 1s for Pristine and In-Doped ZnO Samples

	In $3d_{3/2}$ (eV)	In $3d_{5/2}$ (eV)	In/Zn (at.%)	O_{lat} (%)	O_{ads} (%)	O_{ads}/O_{lat}
In/Zn-0			0	57.2	42.8	0.75
In/Zn-2	452.8	445.2	0.3	50.8	49.2	0.97
In/Zn-6	452.4	445.0	0.7	54.8	45.2	0.82
In/Zn-10	452.3	444.6	0.9	55.7	44.3	0.80

larger electronegativity of In relative to Zn, which results in a larger screening effect in the oxygen atom.[23,24] The shift of the In 3d binding energy also suggests formation of In-doped ZnO solid solution rather than formation of In_2O_3/ZnO composite. The In/Zn atomic ratios for In-doped samples obtained by XPS were 0.3%, 0.7%, and 0.9%, respectively, which are close to the EDX results (Figure S1). The fitting curves of the O 1s spectra are shown in Figure 4d. Each O 1s peak consisted of two components at ~530.5 eV and ~531.7 eV, corresponding to lattice oxygen (O_{lat}) and adsorbed oxygen species (O_{ads}), respectively (Table S1). With increasing In content, the relative content of O_{ads}(O_{ads}/O_{lat}) first increased from 0.75 for pristine ZnO to 0.97 for In/ZnO-2, followed by a decrease of 0.17 to 0.80 for In/ZnO-10 (Table 2). It is noteworthy that there is no significant difference (0.05) between the adsorbed oxygen ratios for pristine ZnO and In/ZnO-10.

3.2. Gas-Sensing Performance. Figure 5a shows the electrical resistance for the fabricated gas sensors measured in air. Typical semiconductor behavior was observed, i.e., resistance decreased as temperature rose. Furthermore, the resistance remarkably decreased with the increase of In doping content. Figure 5b presents the temperature dependence of the gas response to 10 ppm of NO_2 for the sensors. For the pristine ZnO sensor, the response was very low, with a maximum value of only 15 at 300 °C. The In-doped ZnO sensors exhibited much larger response values, and the response significantly increased with the increase of In content. For the In/ZnO-10 sensor, a high response of 339 was obtained at 300 °C for 10 ppm of NO_2, 22 times higher than that of pristine ZnO sensor. Since the In/ZnO-10 sensor showed much faster response and recovery speed at 300 °C than at 270 °C, at which it exhibited the maximum response to NO_2, 300 °C was chosen as the optimum operating temperature for following study.

Figure 6a depicts dynamic electrical resistance changes of pristine and In-doped ZnO sensors in the presence of NO_2 from 0.2 to 10 ppm at 300 °C. The sensors showed typical n-type semiconductor response behavior, the increase of electrical resistance in the presence of NO_2. It can be clearly seen that the electrical resistance increased with the increase of NO_2 concentration for all the sensors, and at each concentration the increment of electrical resistance for In/ZnO-10 was always much higher than that those for other sensors. Further investigations on the performance of In/ZnO-10 in the NO_2 concentration below 0.2 ppm were conducted. Figure 6b shows the dynamic response and recovery curves for the In/ZnO-10 sensor, which were measured by alternately exposing the sensor to ambient air and air/NO_2 mixture at 300 °C. Clearly the In/ZnO-10 sensor was effective for the

Table 2. Comparison of Characteristics of Reported ZnO-Based NO_2 Sensors

material	Temp. (°C)	Conc. (ppm)	Response (R_a/R_g)	Sensitivity (ppm^{-1})	LOD[a] (ppb)	Refs.
ZnO needles	195	0.5	1.8	0.5	75.0	26
ZnO rods	200	0.1	1.3	2.1	17.9	27
ZnO fibers	150	0.03	1.6	6.1	6.1	28
Sn/ZnO particles	150	1.5	1.1	N/A	N/A	7
Ni/ZnO flakes	200	5	2.1	0.1	375	29
In/ZnO particles	275	5	90	N/A	N/A	9
Cr/ZnO rods	100	2	3.7	1.2	31.3	30
Pd/ZnO wires	100	1	9.4	9.5	3.9	31
Au/ZnO spheres	250	0.5	7.8	10.5	3.6	32
Y/ZnO particles	200	0.1	2.6	15.5	2.4	33
Pt/ZnO particles	200	0.02	1.3	16.9	2.2	34
BP/ZnO spheres[b]	160	0.1	20.0	87.2	0.4	35
Nb/ZnO particles	300	0.1	5.0	100.3	0.4	36
Al/ZnO narcissus	240	0.1	6.6	108.5	0.4	37
		0.01	3.7			
In/ZnO-10	300	0.1	24.6	187.9	0.2	this work
		1	185.8			

[a]As the LOD and rms noise were not provided in most publications cited here, we used the rms noise obtained in the present work to estimate the LOD for all the listed sensors, in order to make a rough comparison. [b]BP refers to black phosphorus.

https://dx.doi.org/10.1021/acsami.0c10420
ACS Appl. Mater. Interfaces 2020, 12, 37489−37498

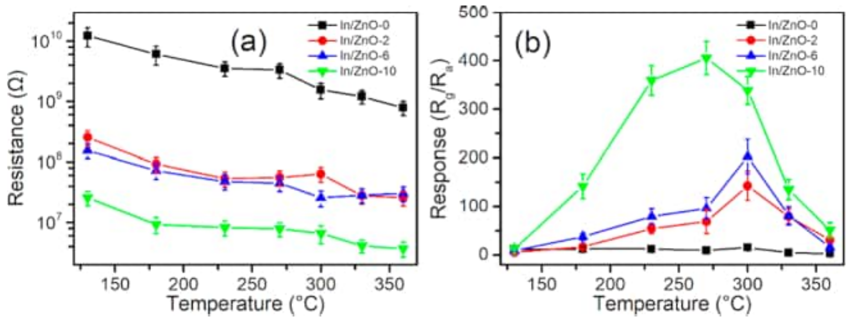

Figure 5. (a) Electrical resistance of pristine and In-doped ZnO sensors in air at various operating temperatures. (b) Response of pristine and In-doped ZnO sensors to 10 ppm of NO_2 at various operating temperatures.

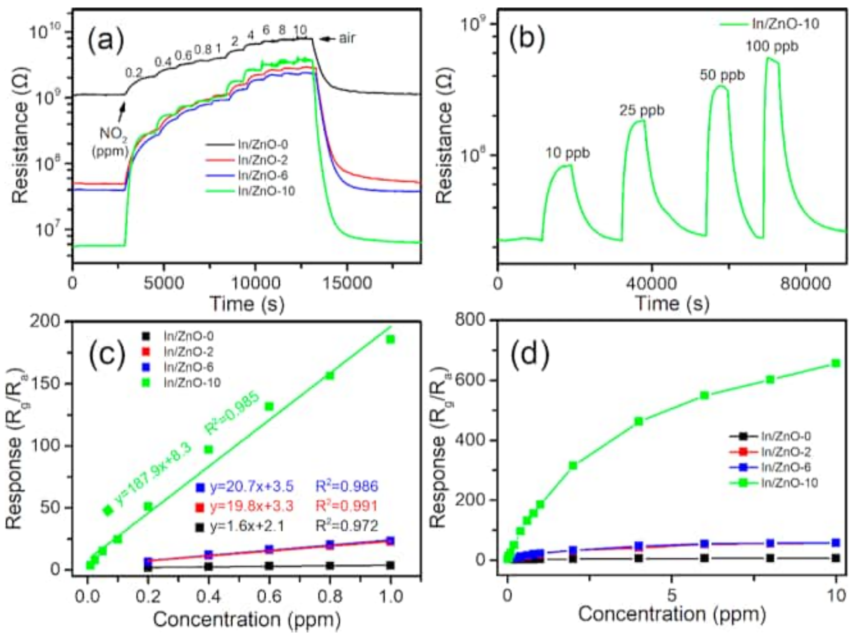

Figure 6. (a) Dynamic response curve of as a function of NO_2 concentration in the range from 0.2 to 10 ppm for pristine and In-doped ZnO samples at 300 °C. (b) Dynamic response curve of In/ZnO-10 as a function of NO_2 concentration in the range from 10 to 100 ppb at 300 °C. (c) Linear relationships between response value and NO_2 concentration for pristine and In-doped ZnO samples at 300 °C. (d) Response value as a function of NO_2 concentration in a wide range for pristine and In-doped ZnO samples at 300 °C.

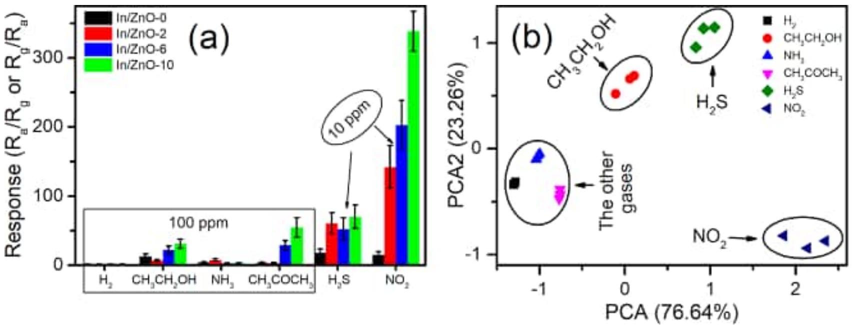

Figure 7. (a) Cross-sensitive responses of pristine and In-doped ZnO sensors to various gases at 300 °C. (b) Pattern recognition based on principal component analysis (PCA) using a sensor array composed of pristine ZnO and In/ZnO-10.

detection of NO_2 at 10, 25, 50, and 100 ppb, and a high response of 3.7 to 10 ppb of NO_2 gas was obtained. And the calculated limit of detection was as low as 0.2 ppb, 2 orders of magnitude lower than that (23.4 ppb) of pristine ZnO. Furthermore, the sensor response could restore to the initial baseline during the recovery process. Therefore, the In/ZnO-10 sensor could be suitable for the detection of ppb-level NO_2 put forward by the WHO for monitoring environmental pollution. Figure 6c exhibits the response of pristine and In-doped ZnO sensors as a function of NO_2 concentration in the range below 1 ppm at 300 °C. The response value increased linearly with the increasing NO_2 concentration, and the slope (i.e., sensitivity) also became larger with increasing In content. A large sensitivity of 187.9 ppm^{-1} was obtained for the In/

https://dx.doi.org/10.1021/acsami.0c10420
ACS Appl. Mater. Interfaces 2020, 12, 37489−37498

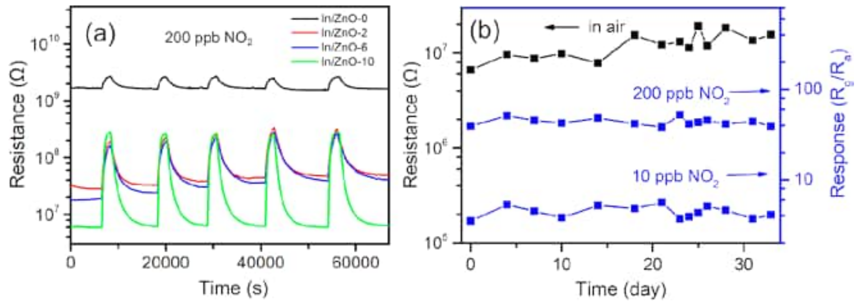

Figure 8. (a) Dynamic resistance changes of pristine and In-doped ZnO sensors in 200 ppb NO_2 at 300 °C. (b) Long-term test of the electrical resistance in air and the response to NO_2 for the In/ZnO-10 sensor at 300 °C.

ZnO-10 sensor, 117.4, 9.5, and 9.1 times larger than that for pristine ZnO (1.6 ppm^{-1}), In/ZnO-2 (19.8 ppm^{-1}) and In/ZnO-6 (20.7 ppm^{-1}), respectively. The wide linear dependence and high sensitivity of the In/ZnO-10 sensor are favorable for calibration and determination of the NO_2 concentration in practical applications. Further tests at higher concentrations above 1 ppm showed the slope decreased with the increasing NO_2 concentration, indicating the sensors were near saturation (Figure 6d). Nevertheless, the In/ZnO-10 sensor still possessed the highest response value and sensitivity among the sensors in the high-concentration range.

Figure 7a compares cross-sensitive responses of pristine and In-doped ZnO sensors to various gases at 300 °C. For the ZnO-0 sensor, the response to 10 ppm of NO_2 was higher than those to 100 ppm of common interfering gases, including CH_3CH_2OH and CH_3COCH_3. However, its response to NO_2 was lower than that to H_2S, indicating its poor selectivity to H_2S. In doping drastically increased the NO_2 response, but only slightly increased the response to C_2H_5OH, CH_3COCH_3, and H_2S. Negligible responses to the other gases (H_2 and NH_3) were also observed. These changes led to marked increase of the NO_2 selectivity, which was represented by the response ratio of NO_2 over the most interfering gases. The NO_2/H_2S, NO_2/C_2H_5OH, and NO_2/CH_3COCH_3 response ratios of the In/ZnO-10 sensor were 4.9, 10.9, and 6.2, respectively, much higher than 0.8, 1.2, and 4.2 of the pristine ZnO sensor. Despite the enhanced NO_2 selectivity for In/ZnO-10, it is unlikely to achieve absolute NO_2 selectivity and exclude interference from other gases with a single sensor. Fortunately, the differences between the pristine and In-doped ZnO sensors in the response to NO_2, H_2S, CH_3CH_2OH, and CH_3COCH_3 could be used to further improve the ability of NO_2 recognition by making a gas sensor array, which is favored by a combination of sensors with distinctly different response characteristics. To verify this idea, a sensor array composed of pristine ZnO and In/ZnO-10 sensor was assembled. Using principal component analysis (PCA) method, pattern recognition of NO_2 and the interfering gases was performed. The six different gases were clearly classified into four groups, NO_2, H_2S, CH_3CH_2OH, and the other gases, without overlap (Figure 7b), thereby successfully distinguishing NO_2 gas from other gases.

Figure 8a presents the dynamic response and recovery curves of pristine and In-doped ZnO sensors in 200 ppb NO_2 at 300 °C. During the five continuous cycles, not only the response and recovery transients but also the baselines were excellently repeated. Consequently, the response and recovery time were calculated for pristine and In-doped ZnO sensors, as shown Figure S3. The response time did not change significantly with the increasing In doping content. A response time of 1055 s was obtained for In/ZnO-10, pretty close to that (1036 s) for pristine ZnO. In contrast, the recovery time decreased from 2712 s for pristine ZnO to 1221s for In/ZnO-10 with the increase of In content. The response and recovery time of the ZnO sensors are relatively long, which may be ascribed to the very low NO_2 concentration (200 ppb). Significant increase of response and recovery time with decreasing analyte concentration has been reported for other semiconductor gas sensors.[25] Long-term stability is another key factor for practical applications of a gas sensor. To illustrate this aspect, one In/ZnO sensor was subject to a long-term measurement. Both the electrical resistance in air and the NO_2 response fluctuated only slightly (Figure 8b). Note that within this period, the sensor was also subjected to other measurements, such as selectivity and repeatability tests, which showed no significant performance degradation with increasing test number. These results suggest good long-term stability of the In/ZnO-10 sensor.

It is well-known that humidity in the environment often affects the sensing performance of semiconductor oxide sensors. As can be seen from Figure S4, the response to 10 ppm of NO_2 for the In/ZnO sensor was still much higher than that for pristine ZnO sensor under different humidity conditions at 300 °C, while the response for pristine ZnO and In/ZnO-10 sensors both decreased as the relative humidity (RH) increased. For the In/ZnO sensor, the response decreased significantly by 68% at 11% RH when compared with that in dry air. As the RH was further raised to 94%, only a slight decrease by 22% was observed. Apparently, the response of the In/ZnO-10 sensor varied only slightly with humidity change within the RH range of 11%−94%, which covers an ambient RH range frequently encountered. Moreover, a high response of 109 and 35 for 10 ppm of NO_2 still remained at 11% and 94% RH, respectively. These results implied the potential applications of the In/ZnO sensor in environments of relatively high humidity. For practical use, possible strategies, e.g., calibrating sensors at different RH before use or dehumidifying the analyte gases, could be adopted to reduce the influence of humidity on the sensing performance.

The above results demonstrate that the In/ZnO-10 sensor in this present work possessed excellent overall sensing performance. When compared with state-of-the-art ZnO-based NO_2 sensors, the In/ZnO-10 sensor exhibited markedly higher response and sensitivity and lower limit of detection (Table 2).

3.3. Sensing Mechanism. For gas sensors based on n-type MOSs such as ZnO, a widely accepted gas sensing mechanism lies in the resistance change of the material due to gas

https://dx.doi.org/10.1021/acsami.0c10420
ACS Appl. Mater. Interfaces 2020, 12, 37489−37498

adsorption and reaction.[38−40] Oxygen molecules in ambient air can be adsorbed on the material surface and transformed into surface-adsorbed ionized oxygen species by capturing free electrons from the conduction band of the material. In this process, an electron depletion layer is formed on the material's surface, resulting in a decrease in the charge carrier concentration and hence higher sensor resistance. When exposed to an NO_2-containing atmosphere, based on previous studies,[1] charge exchanges on the surface of n-type MOSs follow a process like the following:

$$NO_2(ads) + e^- \rightarrow NO_2^-(ads) \quad (1)$$

$$NO_2^-(ads) + O^-(ads) + 2e^- \rightarrow NO(gas) + 2O^{2-}(ads) \quad (2)$$

where e^- indicates a free electron in the semiconductor material. The adsorbed NO_2 molecules will draw out the electrons from ZnO to generate NO_2^- (reaction 1). In addition, electrons could be further captured via the reaction between the formed NO_2^- and the surface-adsorbed O^- (reaction 2). Consequently, the thickness of the depletion layer was thickened and the electrical resistance was increased.

In this work, single phase In-doped ZnO cages were obtained via a one-pot encapsulation strategy. Similar porous and hollow structures were confirmed, suggesting their intrinsic excellent gas accessibility (Figure 2e−h and 3a,b). The increase in surface area (Figure S2) and the decline in crystal size (Figure 3a,b) with the increasing In content would generate more adsorption sites for NO_2 molecules. Moreover, electronic sensitization, which has been well recognized in related studies,[41−43] could occur as a result of In doping according to the following defect reaction,

$$In_2O_3 \xrightarrow{ZnO} 2In_{Zn}^{\bullet} + 3O_O^{\times} + 2e^- \quad (3)$$

This accounts for the observed decrease in electrical resistance by 2 orders of magnitude by In-doping in Figure 5a. Synergy of excellent gas accessibility and abundant adsorption sites and free electrons for the In-doped ZnO cages may greatly promote reaction 1, favoring enhanced response and sensitivity to NO_2. Similarly, these may also account for the reduced optimum working temperature for the In/ZnO-10 sensor in Figure 5b.

Besides, more available free electrons might also favor formation of more surface-adsorbed oxygen, and thus promote the reaction 2.[44−46] However, there was no significant difference in the content of adsorbed oxygen among pristine and In-doped ZnO cages, as verified by XPS analysis (Figure 4d and Table 1), indicating that adsorbed oxygen might not have a significant effect on the NO_2-sensing performance.

Overall, the excellent NO_2-sensing performance of the In/ZnO-10 cages was mainly ascribed to the intrinsic excellent gas accessibility of porous hollow structure, abundant adsorption sites, and electronic sensitization by In doping.

4. CONCLUSIONS

A one-pot encapsulation strategy was used to fabricate In-doped ZnO porous hollow cages. The In doping led to significant enhancement in the NO_2-sensing performance of ZnO porous hollow cages. The In/ZnO-10 sensor was effective for ppb-level NO_2 detection with a response of 3.7 to 10 ppb of NO_2. The calculated limit of detection was as low as 0.2 ppb, 2 orders of magnitude lower than that (23.4 ppb) of pristine ZnO. The In/ZnO-10 sensor showed only minor sensitivity to a number of common interfering gases, and NO_2 was clearly classified using PCA with a sensor array composed of pristine ZnO and In/ZnO-10. The response to NO_2 of the In/ZnO-10 sensor fluctuated only slightly within 33 days. The greatly enhanced NO_2-sensing performance of In-doped ZnO cages was mainly related to the excellent gas accessibility of the porous hollow structure and electronic sensitization by In doping. These findings could possibly be extended to other porous doped ZnO oxides, which may be prepared using other metal acetylacetonates of appropriate molecular diameters, such as $Cr(acac)_3$, $Fe(acac)_3$, and $Mn(acac)_3$.

■ ASSOCIATED CONTENT

Supporting Information

The Supporting Information is available free of charge at https://pubs.acs.org/doi/10.1021/acsami.0c10420.

EDX spectra; surface area and pore size; XPS peak positions of Zn 2p and O 1s; response and recovery time to 200 ppb NO_2; and influence of the humidity on the response (PDF)

■ AUTHOR INFORMATION

Corresponding Author

Jianxin Yi − *State Key Laboratory of Fire Science, Department of Safety Science and Engineering, University of Science and Technology of China, Hefei, Anhui 230026, P. R. China;* orcid.org/0000-0002-1025-865X; Email: yjx@ustc.edu.cn

Authors

Zhou Li − *State Key Laboratory of Fire Science, Department of Safety Science and Engineering, University of Science and Technology of China, Hefei, Anhui 230026, P. R. China;* orcid.org/0000-0003-4225-8985

Yong Zhang − *State Key Laboratory of Fire Science, Department of Safety Science and Engineering, University of Science and Technology of China, Hefei, Anhui 230026, P. R. China*

Hong Zhang − *State Key Laboratory of Fire Science, Department of Safety Science and Engineering, University of Science and Technology of China, Hefei, Anhui 230026, P. R. China*

Yong Jiang − *State Key Laboratory of Fire Science, Department of Safety Science and Engineering, University of Science and Technology of China, Hefei, Anhui 230026, P. R. China;* orcid.org/0000-0001-5535-4293

Complete contact information is available at: https://pubs.acs.org/10.1021/acsami.0c10420

Notes

The authors declare no competing financial interest.

■ ACKNOWLEDGMENTS

Finance support by the National Natural Science Foundation of China (grant Nos. 61871359, U1432108) and Fundamental Research Funds for the Central Universities (grant No. WK2320000044) is gratefully acknowledged.

■ REFERENCES

(1) Casals, O.; Markiewicz, N.; Fabrega, C.; Gracia, I.; Cane, C.; Wasisto, H. S.; Waag, A.; Prades, J. D. A Parts Per Billion (ppb) Sensor for NO_2 with Microwatt (μW) Power Requirements Based on Micro Light Plates. *ACS Sens.* **2019**, *4*, 822−826.

(2) World Health Organization, Nitrogen Dioxide. In *Air Quality Guidelines for Europe*, 2nd ed.; WHO Regional Office for Europe: Copenhagen, Denmark, 2000; Vol. 3, pp 175−180.
(3) Li, Z.; Yi, J.-X. Synthesis and Enhanced NO_2-sensing Properties of ZnO-decorated SnO_2 Microspheres. *Mater. Lett.* **2019**, *236*, 570−573.
(4) Fahad, H. M.; Shiraki, H.; Amani, M.; Zhang, C.; Hebbar, V. S.; Gao, W.; Ota, H.; Hettick, M.; Kiriya, D.; Chen, Y.-Z.; Chueh, Y.-L.; Javey, A. Room Temperature Multiplexed Gas Sensing Using Chemical-Sensitive 3.5-nm-Thin Silicon Transistors. *Sci. Adv.* **2017**, *3*, e1602557.
(5) Ahn, M. W.; Park, K. S.; Heo, J. H.; Park, J. G.; Kim, D. W.; Choi, K. J.; Lee, J. H.; Hong, S. H. Gas Sensing Properties of Defect-Controlled ZnO-Nanowire Gas Sensor. *Appl. Phys. Lett.* **2008**, *93*, 263103.
(6) Li, Z.; Yi, J.-X. Enhanced Ethanol Sensing of Ni-Doped SnO_2 Hollow Spheres Synthesized by a One-Pot Hydrothermal Method. *Sens. Actuators, B* **2017**, *243*, 96−103.
(7) Shishiyanu, S. T.; Shishiyanu, T. S.; Lupan, O. I. Sensing Characteristics of Tin-Doped ZnO Thin Films as NO_2 Gas Sensor. *Sens. Actuators, B* **2005**, *107*, 379−386.
(8) Navale, S. C.; Ravi, V.; Srinivas, D.; Mulla, I.S.; Gosavi, S.W.; Kulkarni, S.K. EPR and DRS Evidence for NO_2 Sensing in Al-Doped ZnO. *Sens. Actuators, B* **2008**, *130*, 668−673.
(9) Ferro, R.; Rodríguez, J. A.; Bertrand, P. Peculiarities of Nitrogen Dioxide Detection with Sprayed Undoped and Indium-Doped Zinc Oxide Thin Films. *Thin Solid Films* **2008**, *516*, 2225−2230.
(10) Lee, J.-H. Gas Sensors using Hierarchical and Hollow Oxide Nanostructures: Overview. *Sens. Actuators, B* **2009**, *140*, 319−336.
(11) Banerjee, R.; Phan, A.; Wang, B.; Knobler, C.; Furukawa, H.; O'Keeffe, M.; Yaghi, O. M. High-Throughput Synthesis of Zeolitic Imidazolate Frameworks and Application to CO_2 Capture. *Science* **2008**, *319*, 939−943.
(12) Park, K. S.; Ni, Z.; Cote, A. P.; Choi, J. Y.; Huang, R. D.; Uribe-Romo, F. J.; Chae, H. K.; O'Keeffe, M.; Yaghi, O. M. Exceptional Chemical and Thermal Stability of Zeolitic Imidazolate Frameworks. *Proc. Natl. Acad. Sci. U. S. A.* **2006**, *103*, 10186−10191.
(13) Ji, S.-F.; Chen, Y.-J.; Zhao, S.; Chen, W.-X.; Shi, L.-J.; Wang, Y.; Dong, J.-C.; Li, Z.; Li, F.-W.; Chen, C.; Peng, Q.; Li, J.; Wang, D.-S.; Li, Y.-D. Atomically Dispersed Ruthenium Species Inside Metal-Organic Frameworks: Combining the High Activity of Atomic Sites and the Molecular Sieving Effect of MOFs. *Angew. Chem., Int. Ed.* **2019**, *58*, 4271−4275.
(14) Sun, J.; Semenchenko, L.; Lim, W. T.; Ballesteros Rivas, M. F.; Varela-Guerrero, V.; Jeong, H.-K. Facile Synthesis of Cd-Substituted Zeolitic-Imidazolate Framework Cd-ZIF-8 and Mixed-Metal CdZn-ZIF-8. *Microporous Mesoporous Mater.* **2018**, *264*, 35−42.
(15) Shi, Q.; Chen, Z.; Song, Z.; Li, J.; Dong, J. Synthesis of ZIF-8 and ZIF-67 by Steam-Assisted Conversion and an Investigation of their Tribological Behaviors. *Angew. Chem., Int. Ed.* **2011**, *50*, 672−675.
(16) Bae, T. H.; Lee, J. S.; Qiu, W.; Koros, W. J.; Jones, C. W.; Nair, S. A High-Performance Gas- Separation Membrane Containing Submicrometer-Sized Metal-Organic Framework Crystals. *Angew. Chem., Int. Ed.* **2010**, *49*, 9863−9866.
(17) Hermes, S.; Schroter, M. K.; Schmid, R.; Khodeir, L.; Muhler, M.; Tissler, A.; Fischer, R. W.; Fischer, R. A. Metal@MOF: Loading of Highly Porous Coordination Polymers Host Lattices by Metal Organic Chemical Vapor Deposition. *Angew. Chem., Int. Ed.* **2005**, *44*, 6237−6341.
(18) Koo, W.-T.; Choi, S.-J.; Kim, S.-J.; Jang, J.-S.; Tuller, H. L.; Kim, I.-D. Heterogeneous Sensitization of Metal-Organic Framework Driven Metal@Metal Oxide Complex Catalysts on an Oxide Nanofiber Scaffold Toward Superior Gas Sensors. *J. Am. Chem. Soc.* **2016**, *138*, 13431−13437.
(19) Guo, L.-L.; Chen, F.; Xie, N.; Kou, X.-Y.; Wang, C.; Sun, Y.-F.; Liu, F.-M.; Liang, X.-S.; Gao, Y.; Yan, X.; Zhang, T.; Lu, G.-Y. Ultra-Sensitive Sensing Platform Based on Pt-ZnO-In_2O_3 Nanofibers for Detection of Acetone. *Sens. Actuators, B* **2018**, *272*, 185−194.
(20) Koo, W.-T.; Jang, J.-S.; Kim, I.-D. Metal-Organic Frameworks for Chemiresistive Sensors. *Chem.* **2019**, *5*, 1938−1963.
(21) Zheng, Y.; Wang, J.; Yao, P. Formaldehyde Sensing Properties of Electrospun NiO-Doped SnO_2 Nanofibers. *Sens. Actuators, B* **2011**, *156*, 723−730.
(22) Cheng, J.-P.; Wang, B.-B.; Zhao, M.-G.; Liu, F.; Zhang, X.-B. Nickel-Doped Tin Oxide Hollow Nanofibers Prepared by Electrospinning for Acetone Sensing. *Sens. Actuators, B* **2014**, *190*, 78−85.
(23) Chen, W.; Qiu, Y.-C.; Zhong, Y.-C.; Wong, K.-S.; Yang, S.-H. High-Efficiency Dye-Sensitized Solar Cells Based on the Composite Photoanocles of SnO_2 Nanoparticles/ZnO Nanotetrapods. *J. Phys. Chem. A* **2010**, *114*, 3127−3138.
(24) Zhang, Z.; Shao, C.; Li, X.; Wang, C.; Zhang, M.; Liu, Y. Electrospun Nanofibers of P-Type NiO/N-Type ZnO Heterojunctions with Enhanced Photocatalytic Activity. *ACS Appl. Mater. Interfaces* **2010**, *2*, 2915−2923.
(25) Xun, H.-T.; Zhang, Z.-Z.; Yu, A.-H.; Yi, J.-X. Remarkably Enhanced Hydrogen Sensing of Highly-Ordered SnO_2-Decorated TiO_2 Nanotubes. *Sens. Actuators, B* **2018**, *273*, 983−990.
(26) Gonzalez-Chavarri, J.; Parellada-Monreal, L.; Castro-Hurtado, I.; Castaño, E.; Mandayo, G. G. ZnO Nanoneedles Grown on Chip for Selective NO_2 Detection Indoors. *Sens. Actuators, B* **2018**, *255*, 1244−1253.
(27) Öztürk, S.; Kılınç, N.; Öztürk, Z. Z. Fabrication of ZnO Nanorods for NO_2 Sensor Applications: Effect of Dimensions and Electrode Position. *J. Alloys Compd.* **2013**, *581*, 196−201.
(28) Lee, H.-U.; Ahn, K.; Lee, S.-J.; Kim, J.-P.; Kim, H.-G.; Jeong, S.-Y.; Cho, C.-R. ZnO Nanobarbed Fibers: Fabrication, Sensing NO_2 Gas, and Their Sensing Mechanism. *Appl. Phys. Lett.* **2011**, *98*, 193114.
(29) Ganbavle, V. V.; Inamdar, S. I.; Agawane, G. L.; Kim, J. H.; Rajpure, K. Y. Synthesis of Fast Response, Highly Sensitive and Selective Ni:ZnO Based NO_2 Sensor. *Chem. Eng. J.* **2016**, *286*, 36−47.
(30) Chang, C.-J.; Chen, J.-K.; Yang, T.-L. Cr-Doped ZnO Based NO_2 Sensors with High Sensitivity at Low Operating Temperature. *J. Taiwan Inst. Chem. Eng.* **2014**, *45*, 1876−1882.
(31) Chen, X.-X.; Shen, Y.-B.; Zhou, P.-F.; Zhao, S.-K.; Zhong, X.-X.; Li, T.-T.; Han, C.; Wei, D.-Z.; Meng, D. NO_2 Sensing Properties of One-Pot-Synthesized ZnO nanowires with Pd Functionalization. *Sens. Actuators, B* **2019**, *280*, 151−161.
(32) Veeran Ponnuvelu, D.; Abdulla, S.; Pullithadathil, B. Highly Monodispersed Mesoporous, Heterojunction ZnO@Au Microspheres for Trace-level Detection of NO_2 Gas. *Microporous Mesoporous Mater.* **2018**, *255*, 156−165.
(33) Kılınc, N.; Ozturk, S.; Arda, L.; Altındal, A.; Ozturk, Z. Z. Structural, Electrical Transport and NO_2 Sensing Properties of Y-Doped ZnO Thin Films. *J. Alloys Compd.* **2012**, *536*, 138−144.
(34) Giancaterini, L.; Cantalini, C.; Cittadini, M.; Sturaro, M.; Guglielmi, M.; Martucci, A.; Resmini, A.; Anselmi-Tamburini, U. Au and Pt Nanoparticles Effects on the Optical and Electrical Gas Sensing Properties of Sol-Gel-Based ZnO Thin-Film Sensors. *IEEE Sens. J.* **2015**, *15*, 1068−1076.
(35) Li, Q.; Cen, Y.; Huang, J.-Y.; Li, X.-J.; Zhang, H.; Geng, Y.-F.; Yakobson, B. I.; Du, Y.; Tian, X.-Q. Zinc Oxide-Black Phosphorus Composites for Ultrasensitive Nitrogen Dioxide Sensing. *Nanoscale Horiz.* **2018**, *3*, 525−531.
(36) Kruefu, V.; Liewhiran, C.; Wisitsoraat, A.; Phanichphant, S. Selectivity of Flame-Spray-Made Nb/ZnO Thick Films towards NO_2 Gas. *Sens. Actuators, B* **2011**, *156*, 360−367.
(37) Zhang, Y.-H.; Li, Y.-L.; Gong, F.-L.; Xie, K.-F.; Liu, M.; Zhang, H.-L.; Fang, S.-M. Al Doped Narcissus-like ZnO for Enhanced NO_2 Sensing Performance: An Experimental and DFT Investigation. *Sens. Actuators, B* **2020**, *305*, 127489.
(38) Chen, D.-D.; Yi, J.-X. One-Pot Electrospinning and Gas-Sensing Properties of $LaMnO_3$ Perovskite/SnO_2 Heterojunction Nanofibers. *J. Nanopart. Res.* **2018**, *20*, 65.
(39) Yi, J.-X.; Chen, W.-J.; Han, J.; Chen, D.-D. Sensitive and Selective Detection of Plasticizer Vapors with Modified-SnO_2 Hollow

https://dx.doi.org/10.1021/acsami.0c10420
ACS Appl. Mater. Interfaces 2020, 12, 37489−37498

Nanofibers for Electrical Fire Warning. *Sens. Actuators, B* **2019**, *287*, 364−370.
(40) Wang, Z.-H.; Xue, J.; Han, D.-M.; Gu, F.-B. Controllable Defect Redistribution of ZnO Nanopyramids with Exposed {1010} Facets for Enhanced Gas Sensing Performance. *ACS Appl. Mater. Interfaces* **2015**, *7*, 308−317.
(41) Choi, S.-W.; Katoch, A.; Kim, J.-H.; Kim, S.-S. Striking Sensing Improvement of N-Type Oxide Nanowires by Electronic Sensitization Based on Work Function Difference. *J. Mater. Chem. C* **2015**, *3*, 1521−1527.
(42) Fu, J.-C.; Zhao, C.-H.; Zhang, J.-L.; Peng, Y.; Xie, E.-Q. Enhanced Gas Sensing Performance of Electrospun Pt-Functionalized NiO Nanotubes with Chemical and Electronic Sensitization. *ACS Appl. Mater. Interfaces* **2013**, *5*, 7410−7416.
(43) Barbosa, M. S.; Suman, P. H.; Kim, J. J.; Tuller, H. L.; Orlandi, M. O. Investigation of Electronic and Chemical Sensitization Effects Promoted by Pt and Pd Nanoparticles on Single-Crystalline SnO Nanobelt-Based Gas Sensors. *Sens. Actuators, B* **2019**, *301*, 127055.
(44) Wang, Z.-H.; Tian, Z.-W.; Han, D.-M.; Gu, F.-B. Highly Sensitive and Selective Ethanol Sensor Fabricated with In-Doped 3DOM ZnO. *ACS Appl. Mater. Interfaces* **2016**, *8*, 5466−5474.
(45) Phan, D. T.; Chung, G. S. Effects of Defects in Ga-doped ZnO Nanorods Formed by a Hydrothermal Method on CO Sensing Properties. *Sens. Actuators, B* **2013**, *187*, 191−197.
(46) Bai, S. L.; Guo, T.; Zhao, Y. B.; Sun, J. H.; Li, D. Q.; Chen, A. F.; Liu, C. C. Sensing Performance and Mechanism of Fe-doped ZnO Microflowers. *Sens. Actuators, B* **2014**, *195*, 657−666.

■ NOTE ADDED AFTER ASAP PUBLICATION

Due to a production error, this paper was published on the Web August 3, 2020, with errors in the bottom two rows or Table 2. The corrected version was reposted on August 4, 2020.

https://dx.doi.org/10.1021/acsami.0c10420
ACS Appl. Mater. Interfaces 2020, 12, 37489−37498

Nanoscale Advances

ROYAL SOCIETY OF CHEMISTRY

PAPER

View Article Online
View Journal | View Issue

Open Access Article. Published on 02 February 2022. Downloaded on 2/1/2025 6:14:34 AM. This article is licensed under a Creative Commons Attribution-NonCommercial 3.0 Unported Licence.

Check for updates

Cite this: *Nanoscale Adv.*, 2022, **4**, 1345

Ligand-assisted deposition of ultra-small Au nanodots on Fe_2O_3/reduced graphene oxide for flexible gas sensors†

Jian Wang,[a] Essalhi Fatima-Ezzahra,[a] Jie Dai,[a] Yanlei Liu,[a] Chengjie Pei,[a] Hai Li,[a] Zhiwei Wang*[ab] and Xiao Huang*[a]

The development of flexible room-temperature gas sensors is important in environmental monitoring and protection. In this contribution, by using 1-octadecanethiol (ODT) as a surface ligand, Au nanodots (NDs) with ultra-small size of ~1.7 nm were deposited on the surface of α-Fe_2O_3/reduced graphene oxide (rGO). The Au ND–ODT/α-Fe_2O_3/rGO composite was fabricated into flexible gas sensors, which could detect NO_2 gas down to 200 ppb at room temperature. Compared with α-Fe_2O_3/rGO, Au ND–ODT/α-Fe_2O_3/rGO showed enhanced sensing performance because of the beneficial effects of Au NDs, including facilitating the adsorption of NO_2 molecules and forming ohmic-like contact with rGO and α-Fe_2O_3. In addition, the sensing performance of the composite was also influenced by the surface ligands of the Au NDs. Ligands with less polar terminal groups were found to be beneficial to charge transfer in the sensing film. Moreover, Au ND–ODT/α-Fe_2O_3/rGO-based flexible sensors showed negligible performance deterioration under moderately bent conditions, suggesting their potential to be used in portable and wearable devices.

Received 9th October 2021
Accepted 17th January 2022

DOI: 10.1039/d1na00734c

rsc.li/nanoscale-advances

1. Introduction

The fast technological and industrial development has led to increasingly serious air pollution. Among the several gaseous pollutants in air, nitrogen dioxide (NO_2), which is usually produced by fuel burning and vehicle exhausts,[1] can cause several pulmonary diseases such as pulmonary edema, tracheitis and pneumothorax, and is also the main cause for acid rain. Therefore, the development of highly sensitive and selective NO_2 gas sensors is important to environmental monitoring and protection.[2,3] In addition, making gas sensors flexible can enable their integration into portable and wearable devices.[4,5]

Metal oxides (MOXs), such as ZnO,[6–8] NiO,[9–11] Fe_2O_3,[12–14] and In_2O_3 (ref. 15–17) have been widely used for detection of hazardous gases over the past few decades. For example, alpha-iron oxide (α-Fe_2O_3) is a commonly used sensing material for detection of acetone,[18] H_2S[19] and NO_2 (ref. 14) because of its low cost and simple production.[20] However, gas sensors based on MOXs normally require high operating temperatures due to their poor conductivity and moderate surface activity at room temperature (RT), making it difficult to fabricate RT gas sensors with simple configurations towards flexible devices.

Hybridization of MOXs with conductive materials such as graphene derivatives[21,22] and carbon nanotubes[23,24] can improve their gas sensing performances. For instance, reduced graphene oxide (rGO), which possesses excellent electrical conductivity, large specific surface area, and good surface activity, has been wildly utilized to composite with MOXs for gas sensing.[25–27] Examples of MOX/rGO-based sensing materials include ZnO/rGO,[28] Fe_2O_3/rGO,[29] and CuO/rGO[30] for enhanced H_2S, NO_2, and acetone detection, respectively. Introducing noble metal nanostructures in sensing materials is another strategy to improve the gas sensing performance of MOX-based gas sensors. This is because, in addition to their good conductivity, noble metal nanomaterials can provide lower energy required for adsorption of gas molecules based on both experimental and theoretical investigations.[31] For example, noble metals such as platinum (Pt),[32] gold (Au),[33] silver (Ag)[34] and palladium (Pd)[35] have been used to improve the gas sensing response of MOX-based sensors. Besides, previous reports have suggested that the type of surface ligands can influence the surface activity of noble metal nanostructures.[2,36] However, few studies have investigated the ligand effects of metal nanomaterials in gas sensing.

In this work, a composite material, *i.e.* Au ND–ODT/α-Fe_2O_3/rGO, containing ultra-small Au nanodots (NDs) functionalized with 1-octadecanethiol (ODT), α-Fe_2O_3 and rGO was prepared

[a]*Institute of Advanced Materials (IAM), Nanjing Tech University (Nanjing Tech), 30 South Puzhu Road, Nanjing 211816, China. E-mail: iamxhuang@njtech.edu.cn*

[b]*Frontiers Science Center for Flexible Electronics, Xi'an Institute of Flexible Electronics (IFE), Xi'an Institute of Biomedical Materials & Engineering, Northwestern Polytechnical University, 127 West Youyi Road, Xi'an 710072, China. E-mail: iamzwwang@nwpu.edu.cn*

† Electronic supplementary information (ESI) available: Fig. S1–S12. See DOI: 10.1039/d1na00734c

© 2022 The Author(s). Published by the Royal Society of Chemistry

Open Access Article. Published on 02 February 2022. Downloaded on 2/1/2025 6:14:34 AM.
This article is licensed under a Creative Commons Attribution-NonCommercial 3.0 Unported Licence.

and applied in NO_2 gas sensing at RT, showing enhanced performance as compared with α-Fe_2O_3/rGO-based sensor. The improved sensing performance was attributed to the Au NDs, which facilitated the adsorption of NO_2 gas and formed the ohmic-like contact with rGO and α-Fe_2O_3. The surface ligands of Au NDs were also found to influence the sensing performance. Flexible gas sensors based on Au ND–ODT/α-Fe_2O_3/rGO exhibited well-maintained responses under bent conditions due to the good mechanical strength, flexibility and film-forming ability of rGO.

2. Materials and methods

2.1. Materials

Graphene oxide (GO) aqueous solution (0.5 g L^{-1}) was commercially purchased from Shanghai Carbon Source Huigu New Material Technology Co., Ltd, China. $FeCl_3\cdot 6H_2O$ was purchased from Shanghai Adamas Reagents Co., Ltd, China. $HAuCl_4\cdot H_2O$ was purchased from Beijing Huawei Ruike Chemical Co., Ltd, China. 1-Octadecanethiol (ODT) and 16-mercaptohexadecanoic acid (MHA) were purchased from Shanghai Aladdin Biochemical Technology Co., Ltd, China. Ethanol (99.7%) was purchased from Sinopharm Chemical Reagent Co., Ltd, China. Printable Ag ink was purchased from Shanghai Mifang Electronic Technology Co., Ltd, China. All chemicals were used without further purification.

2.2. Preparation of α-Fe_2O_3/rGO hybrids

Typically, 0.25 mmol $FeCl_3\cdot 6H_2O$ was mixed with 40 mL GO aqueous solution (0.5 g L^{-1}) under stirring for 10 min. This mixture was further ultra-sonicated for 20 min before being added into a 50 mL Teflon-lined stainless-steel autoclave and then heated at 160 °C for 18 h. After being naturally cooled to room temperature (RT), the solution was centrifuged and washed with deionized water for 3 times and re-dispersed in 10 mL ethanol for further use.

2.3. Preparation of Au/α-Fe_2O_3/rGO hybrids

To decorate Au nanodots (NDs) on α-Fe_2O_3/rGO, 2 mL of the above-mentioned α-Fe_2O_3/rGO dispersion, 1 mL $HAuCl_4\cdot$ethanol solution (1 mg mL^{-1}), 2 mL ODT (or MHA) ethanolic solution (1 mg mL^{-1}) and 8 mL ethanol were mixed in a 20 mL glass bottle, which was irradiated with a 150 W xenon lamp for 2 min. The solution was then centrifuged at 7000 rpm and washed twice with ethanol. Depending on the ligand used, the products were named as Au ND–ODT/α-Fe_2O_3/rGO hybrids or Au ND–MHA/α-Fe_2O_3/rGO hybrids.

2.4. Materials characterization

Transmission electron microscopy (TEM, JEOL, 2100Plus and 2100F, Japan) was used to investigate the morphologies and microstructures of the samples. X-ray diffraction (XRD, Rigaku, XtaLAB mini II, Japan) was performed with Cu Kα radiation (λ = 1.54 Å). An X-ray photoelectron spectrometer (XPS, VersaProbe, PHI 5000, Japan) was utilized to analyze the oxidation state of the GO, and all the binding energies were calibrated to the C 1s band at 284.6 eV. An ultraviolet photoelectron spectrometer (UPS, VersaProbe, PHI 5000, Japan) coupled with an ultraviolet and visible spectrophotometer (UV, Shimadzu, UV-1780, Japan) was used to measure the electronic work functions and band levels of different materials.

2.5. Fabrication of flexible gas sensors and gas sensing tests

By employing a microelectronic printer (Shanghai Mifang Electronic Technology Co., Ltd, Scientific 3, China) with commercial Ag ink, arrays of interdigitated Ag electrodes were printed on polyethylene terephthalate (PET) substrates. To fabricate the flexible gas sensors, 20 μL of the sensing material solution was spin-coated on the as-prepared interdigitated Ag electrodes and then dried at RT for 2–3 h.

The gas sensing tests were conducted at RT in a sealed chamber. Constant currents were applied to the sensor electrodes and the variation of resistance was recorded using a data acquisition system (34972A, Agilent). The response of the sensor was defined as $\Delta R/R_0$ (%) ($\Delta R = R_g - R_0$, in which R_0 and R_g are the resistances of the sample in simulated air environment (20.9% O_2, 79.1% N_2, and 19% RH) and the target gas, respectively). The sensor response and recovery times refer to the times when the sensor reaches 90% of the total resistance change during the response and recovery process, respectively.

3. Results and discussion

The preparation process of Au nanodot (ND)–1-octadecanethiol (ODT)/α-Fe_2O_3/reduced graphene oxide (rGO) hybrid is schematically illustrated in Fig. 1a. Typically, α-Fe_2O_3 nanoparticles (NPs) with a size of 30–40 nm were first deposited on GO nanosheets *via* a hydrothermal reaction, during which GO was also reduced to rGO because of the reducibility of H_2O under high temperature and pressure[29,37] (see Fig. S1 and S2 in the ESI†). After that, by using ODT as a surface ligand, Au NDs were decorated on the as-prepared composite through a photochemical reaction.[38] As shown in Fig. 1b and S3a (see in the ESI†), Au NDs with an average size of ~1.7 nm were well dispersed on the surface of α-Fe_2O_3/rGO. The ultra-small size of the Au NDs was achieved due to the strong binding of the ODT molecules to Au surfaces, which prevented the Au nanoparticles from growing large. Energy dispersive X-ray (EDX) mapping of a typical composite reveals the distribution of Au, Fe and C elements (Fig. 1c). The structure properties of Au ND–ODT/α-Fe_2O_3/rGO were investigated with X-ray diffraction (XRD), high resolution transmission electron microscope (HRTEM) analysis and selected area electron diffraction (SAED). The XRD pattern of the composite (see Fig. S3b in the ESI†) is similar to that of α-Fe_2O_3/rGO without showing the characteristic peaks for Au, which is due to the ultra-small size of the Au NDs based on Scherrer's theory.[39,40] As shown in the HRTEM image in Fig. 1d, lattice spacings of 0.17 and 0.23 nm can be observed, which can be assigned to the (116) planes of α-Fe_2O_3 and (111) planes of Au, respectively. Besides, the SAED pattern of the product also reveals rings for rGO and α-Fe_2O_3 (Fig. 1e).

 © 2022 The Author(s). Published by the Royal Society of Chemistry

Open Access Article. Published on 02 February 2022. Downloaded on 2/1/2025 6:14:34 AM.
This article is licensed under a Creative Commons Attribution-NonCommercial 3.0 Unported Licence.

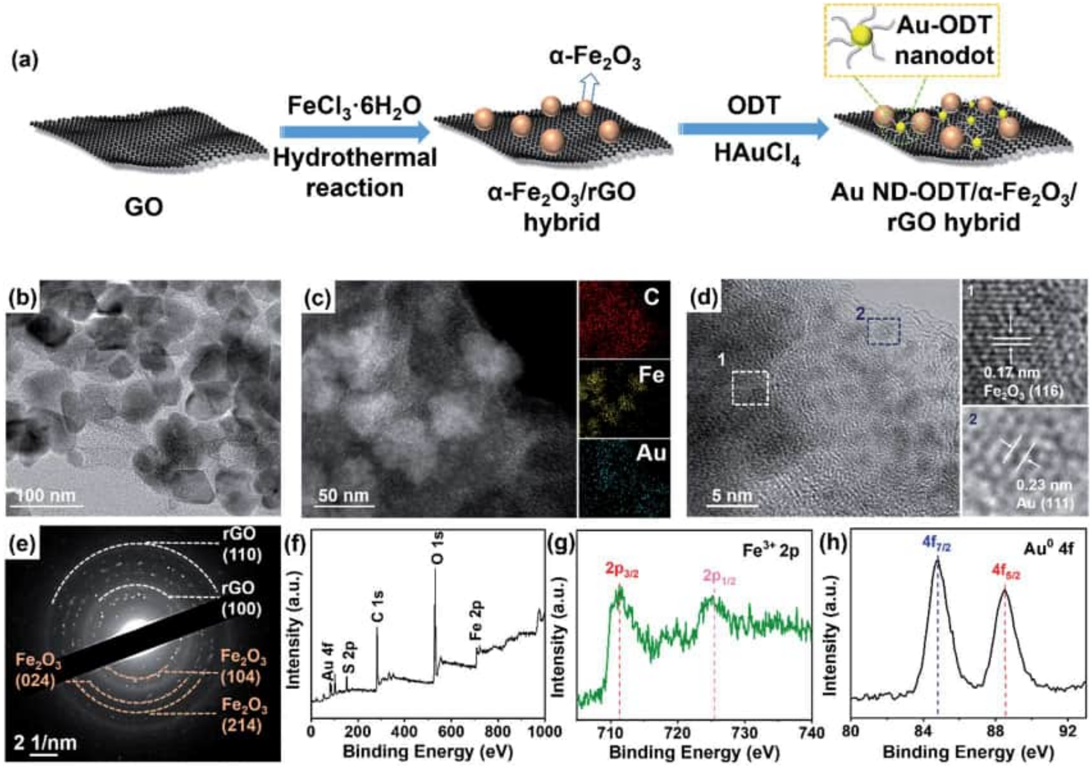

Fig. 1 (a) Schematic illustration of the preparation of Au ND–ODT/α-Fe_2O_3/rGO hybrids. (b) TEM image, (c) STEM image and EDX mapping, (d) HRTEM image, (e) SAED pattern, (f) XPS full scan spectrum, (g) high-resolution Fe 2p spectrum and (h) Au 4f spectrum of the Au ND–ODT/α-Fe_2O_3/rGO composite.

The oxidation states of the as-prepared Au ND–ODT/α-Fe_2O_3/rGO were investigated by X-ray photoelectron spectroscopy (XPS). The survey spectrum of the sample shows the presence of Au, Fe and C elements (Fig. 1f). The high-resolution Fe 2p spectrum shows a doublet at 711.7 and 725.6 eV, corresponding to the $2p_{3/2}$ and $2p_{1/2}$ bands of Fe^{3+}, respectively (Fig. 1g).[41] Besides, in the Au 4f spectrum, two peaks at binding energies of 84.8 and 88.5 eV can be assigned to the Au $4f_{7/2}$ and $4f_{5/2}$ bands of Au^0, respectively (Fig. 1h), indicating the successful photochemical reduction of Au^{3+}.[42]

The as-prepared composites, namely, α-Fe_2O_3/rGO and Au ND–ODT/α-Fe_2O_3/rGO were spin-coated onto interdigitated Ag electrodes which were inkjet-printed on flexible polyethylene terephthalate (PET) substrates (Fig. 2a) to prepare flexible gas sensors. The response–recovery curves of these gas sensors toward NO_2 were studied at RT in simulated air (20.9% O_2, 79.1% N_2, and 19% RH) with target gas concentrations increasing from 200 to 1000 ppb (Fig. 2b). All of the sensors showed decreased resistance upon NO_2 exposure, and the reduction in resistance (ΔR) increased with increasing NO_2 concentrations (Fig. 2b). The α-Fe_2O_3/rGO and Au ND–ODT/α-Fe_2O_3/rGO sensors both exhibited fast response times (*e.g.*, about tens of seconds to 800 ppb NO_2) but slow recovery times (*e.g.*, more than 20 minutes to 800 ppb NO_2) (see Fig. S4 in the ESI†). This points to the strong interaction between the NO_2 molecules and the sensing material, leading to a slow desorption process.[43–45] In our sensing materials, the rGO nanosheets and α-Fe_2O_3 showed p-type and n-type semiconducting properties, respectively, based on their ultraviolet photoelectron spectroscopy (UPS) and ultraviolet-visible (UV-vis) spectroscopy analysis results (see Fig. S5 and S6 in the ESI†).

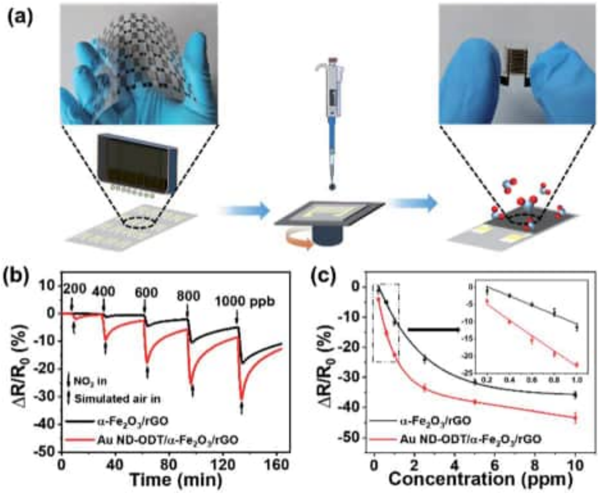

Fig. 2 (a) Schematic illustration of the preparation of the flexible NO_2 sensor, with photographs of the printed Ag electrode array and flexible gas sensor coated with Au ND–ODT/α-Fe_2O_3/rGO. (b) Dynamic response–recovery curves of α-Fe_2O_3/rGO and Au ND–ODT/α-Fe_2O_3/rGO in response to NO_2 gas with different concentrations at RT. (c) Responses of α-Fe_2O_3/rGO and Au ND–ODT/α-Fe_2O_3/rGO sensors for NO_2 with different concentrations.

© 2022 The Author(s). Published by the Royal Society of Chemistry

Open Access Article. Published on 02 February 2022. Downloaded on 2/1/2025 6:14:34 AM.
This article is licensed under a Creative Commons Attribution-NonCommercial 3.0 Unported Licence.

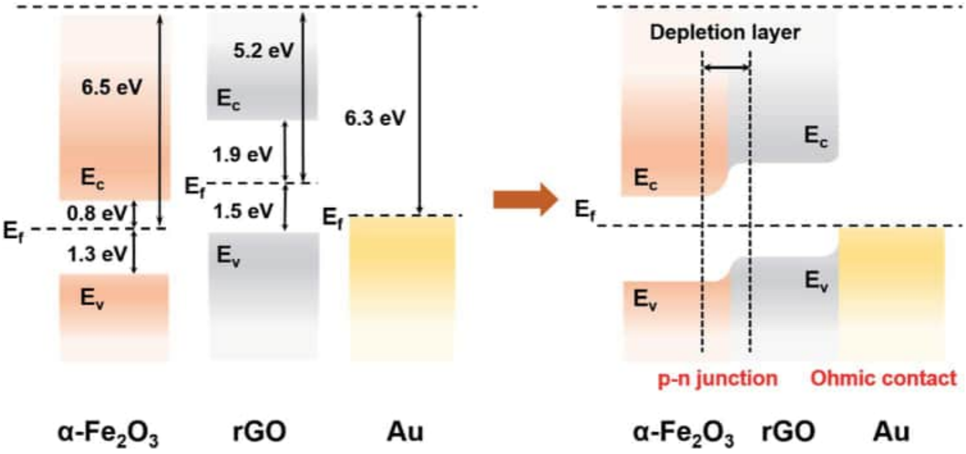

Fig. 3 Schematic of the band level diagram of the Au ND–ODT/α-Fe_2O_3/rGO composite.

Generally, NO_2, as an oxidizing gas, could extract electrons from sensing materials. This increased the concentration of holes in the p-type rGO, which was the main conducting matrix in the hybrid sensing material. The n-type α-Fe_2O_3 also lost electrons to NO_2, and because of its lower Fermi level than that of rGO (Fig. 3), its electron vacancies were compensated by extracting electrons from rGO. This further increased the hole concentration in rGO, thus decreasing the resistance of the sensing film.[46] Moreover, with the work functions, bandgaps and band level positions determined with UPS and UV-vis spectrometers (see Fig. S5 and S6 in the ESI†), we also studied the band alignment in the composite material as shown in Fig. 3. It can be seen that a p–n junction might form between rGO and α-Fe_2O_3, resulting in a charge neutral interface (the depletion layer). The adsorption of NO_2 molecules could narrow the width of the depletion layer and facilitate the charge transfer at the heterojunction, thus resulting in further decreased resistance of the sensing film.[47–50]

The Au ND–ODT/α-Fe_2O_3/rGO-based sensor showed an enhanced sensing performance as compared with the α-Fe_2O_3/rGO sensor, for example, with at least three times higher response at 200 ppb (Fig. 2c). Such an improvement might be attributed, in part, to the increased gas adsorption sites on the ultra-small Au NDs. Besides, due to the lower work function of Au NDs as compared to that of rGO, an ohmic contact might be established between them (Fig. 3), which favoured the charge transfer from rGO to Au NDs and finally to the NO_2 molecules, thus improving the sensitivity of the hybrid sensing material.[51,52] Moreover, ohmic like contact might also form between α-Fe_2O_3 and Au NDs (see Fig. S7 in the ESI†), which enabled facile electron transfer across the Au/α-Fe_2O_3 interface, leading to the formation of additional electron vacancies in α-Fe_2O_3 and thus an enhanced sensitivity. In addition, our Au ND–ODT/α-Fe_2O_3/rGO-based sensor exhibits performance comparable to that of many previously reported RT NO_2 gas sensors (see Table S1 in the ESI†)[53–61] and outperforms the recently reported rGO nanofiber,[54] Au–CuO[59] and MoS_2 (ref. 60) based sensors.

Since ODT molecules were used as the capping agent to prepare the ultra-small Au NDs, we also investigated the effect of surface ligands on the gas sensing performance by using another type of thiol, *i.e.* 16-mercaptohexadecanoic acid (MHA), to prepare Au NDs (see Fig. S8 and S9 in the ESI†), and the resulting material was denoted as Au ND–MHA/α-Fe_2O_3/rGO. Our results showed that the Au ND–ODT/α-Fe_2O_3/rGO-based sensor outperformed the Au ND–MHA/α-Fe_2O_3/rGO sensor (see Fig. S10 in the ESI†). This was mainly attributed to the different terminal groups of the two types of thiols (*i.e.* carboxylic acid groups in MHA and methyl groups in ODT), considering their similar alkane chain lengths. Compared to the less polar methyl groups of ODT molecules, the polar carboxylic acid groups of MHA possess higher electron withdrawing ability which could extract electrons from Au NDs,[62] and hinder the charge transfer in the sensing film (Fig. 4). This was proved by the lower baseline resistance of the Au ND–ODT/α-Fe_2O_3/rGO sensor compared to that of the Au ND–MHA/α-Fe_2O_3/rGO sensor (see Fig. S10 in the ESI†).

The selectivity of Au ND–ODT/α-Fe_2O_3/rGO-based sensors was studied by comparing their responses to NO_2 with other

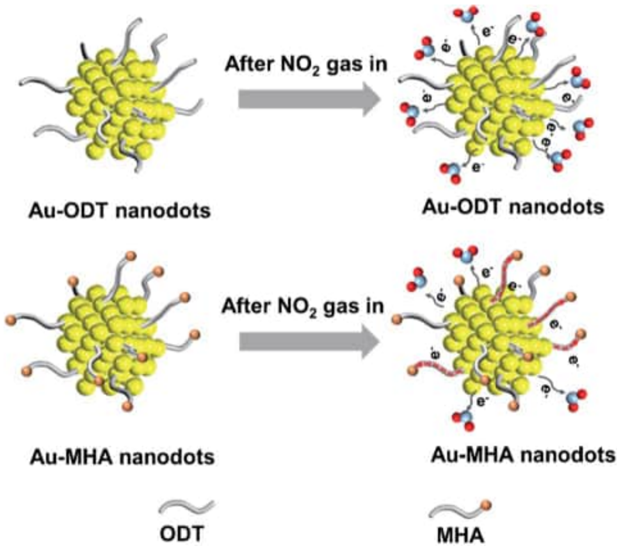

Fig. 4 Schematic illustration of the effect of surface ligands of Au NDs on gas sensing.

 © 2022 The Author(s). Published by the Royal Society of Chemistry

Open Access Article. Published on 02 February 2022. Downloaded on 2/1/2025 6:14:34 AM.
This article is licensed under a Creative Commons Attribution-NonCommercial 3.0 Unported Licence.

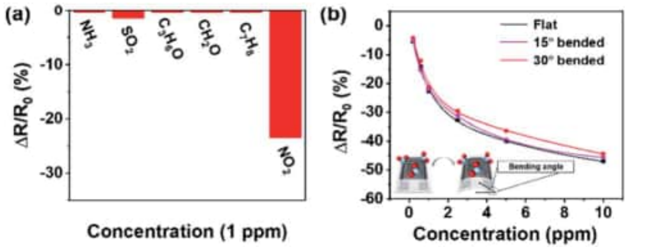

Fig. 5 (a) Responses of the Au ND–ODT/α-Fe_2O_3/rGO sensor to 1 ppm of NH_3, SO_2, C_3H_6O, CH_2O, C_7H_8 and NO_2. (b) Comparison of the responses of a typical Au ND–ODT/α-Fe_2O_3/rGO sensor to NO_2 under flat and different bent conditions.

gases including NH_3, SO_2, acetone, formaldehyde and methylbenzene. The responses to the other gases were at least 8 times lower than that to NO_2, indicating the good selectivity of the hybrid sensing material (Fig. 5a). Lastly, we demonstrated that our sensors on flexible substrates can work under bent conditions. As shown in Fig. 5b, a typical gas sensor showed similar sensing responses under the flat and bent conditions at bending angles of 15° and 30°, and negligible performance change even after the device was bent 300 times (see Fig. S11 in the ESI†). This can be due to the good mechanical strength, flexibility and film-forming ability of rGO sheets, which could help to avoid the loss of contact points under moderately bent conditions. However, at larger bending angles, *e.g.* 45° and 60°, the loss of contact points in the sensing film might occur, causing performance deterioration (see Fig. S12 in the ESI†).

4. Conclusion

In summary, the Au ND–ODT/α-Fe_2O_3/rGO composite was successfully prepared, which was utilized for NO_2 sensing. The sensor could detect NO_2 with concentrations down to 200 ppb at RT in simulated air, and showed improved sensing performance as compared with α-Fe_2O_3/rGO because of the advantageous effects of Au NDs. Besides, the surface ligands on Au NDs also influenced the sensing performance, that is, less polar terminal groups of the ligands favored charge transfer in the sensing film. Flexible gas sensors based on Au ND–ODT/α-Fe_2O_3/rGO exhibited well-maintained sensing responses under moderately bent conditions, indicating their potential applications in portable and wearable electronic devices.

Conflicts of interest

The authors declare no competing interests.

Acknowledgements

This work was supported by the National Natural Science Foundation of China (grant no.: 51832001), the Fundamental Research Funds for the Central Universities of China, the Young 1000 Talents Global Recruitment Program of China, the Joint Research Funds of Department of Science & Technology of Shaanxi Province and Northwestern Polytechnical University (grant no.: 2020GXLH-Z-026 and 2020GXLH-Z-027), and the China Postdoctoral Science Foundation (grant no.: 2021M692618).

References

1 W. Huang, X. Zhuang, F. S. Melkonyan, B. Wang, L. Zeng, G. Wang, S. Han, M. J. Bedzyk, J. Yu, T. J. Marks and A. Facchetti, *Adv. Mater.*, 2017, **29**, 1701706.
2 Q. Sun, J. Wang, X. Wang, J. Dai, X. Wang, H. Fan, Z. Wang, H. Li, X. Huang and W. Huang, *Nanoscale*, 2020, **12**, 16987–16994.
3 H. Wang, W. P. Lustig and J. Li, *Chem. Soc. Rev.*, 2018, **47**, 4729–4756.
4 J. Dai, O. Ogbeide, N. Macadam, Q. Sun, W. Yu, Y. Li, B. L. Su, T. Hasan, X. Huang and W. Huang, *Chem. Soc. Rev.*, 2020, **49**, 1756–1789.
5 S. Ota, A. Ando and D. Chiba, *Nat. Electron.*, 2018, **1**, 124–129.
6 H. Kim, Y. Pak, Y. Jeong, W. Kim, J. Kim and G. Y. Jung, *Sens. Actuators, B*, 2018, **262**, 460–468.
7 G. Namgung, Q. T. H. Ta, W. Yang and J. S. Noh, *ACS Appl. Mater. Interfaces*, 2019, **11**, 1411–1419.
8 H. Yuan, S. Aljneibi, J. Yuan, Y. Wang, H. Liu, J. Fang, C. Tang, X. Yan, H. Cai, Y. Gu, S. J. Pennycook, J. Tao and D. Zhao, *Adv. Mater.*, 2019, **31**, e1807161.
9 P. Li, C. Cao, Q. Shen, B. Bai, H. Jin, J. Yu, W. Chen and W. Song, *Sens. Actuators, B*, 2021, **339**, 129886.
10 S. Y. Yi, Y. G. Song, J. Y. Park, J. M. Suh, G. S. Kim, Y. S. Shim, J. M. Yuk, S. Kim, H. W. Jang, B. K. Ju and C. Y. Kang, *ACS Appl. Mater. Interfaces*, 2019, **11**, 7529–7538.
11 L. Sui, T. Yu, D. Zhao, X. Cheng, X. Zhang, P. Wang, Y. Xu, S. Gao, H. Zhao, Y. Gao and L. Huo, *J. Hazard. Mater.*, 2020, **385**, 121570.
12 H. Fu, Q. Wang, J. Ding, Y. Zhu, M. Zhang, C. Yang and S. Wang, *Sens. Actuators, B*, 2020, **303**, 127186.
13 R. Ahmad, M. S. Ahn and Y. B. Hahn, *Adv. Mater. Interfaces*, 2017, **4**, 1700691.
14 C. Hua, Y. Shang, Y. Wang, J. Xu, Y. Zhang, X. Li and A. Cao, *Appl. Surf. Sci.*, 2017, **405**, 405–411.
15 Z. Wang, C. Hou, Q. De, F. Gu and D. Han, *ACS Sens.*, 2018, **3**, 468–475.
16 L. Jun, Q. Chen, W. Fu, Y. Yang, W. Zhu and J. Zhang, *ACS Appl. Mater. Interfaces*, 2020, **12**, 38425–38434.
17 X. Liu, K. Zhao, X. Sun, C. Zhang, X. Duan, P. Hou, G. Zhao, S. Zhang, H. Yang, R. Cao and X. Xu, *Sens. Actuators, B*, 2019, **285**, 1–10.
18 S. Zhang, M. Yang, K. Liang, A. Turak, B. Zhang, D. Meng, C. Wang, F. Qu, W. Cheng and M. Yang, *Sens. Actuators, B*, 2019, **290**, 59–67.
19 Y. Guo, X. Tian, X. Wang and J. Sun, *Sens. Actuators, B*, 2019, **293**, 136–143.
20 B. Zhang, J. Liu, X. Cui, Y. Wang, Y. Gao, P. Sun, F. Liu, K. Shimanoe, N. Yamazoe and G. Lu, *Sens. Actuators, B*, 2017, **241**, 904–914.
21 G. Bae, I. S. Jeon, M. Jang, W. Song, S. Myung, J. Lim, S. S. Lee, H. K. Jung, C. Y. Park and K. S. An, *ACS Appl. Mater. Interfaces*, 2019, **11**, 16830–16837.

© 2022 The Author(s). Published by the Royal Society of Chemistry

Open Access Article. Published on 02 February 2022. Downloaded on 2/1/2025 6:14:34 AM. This article is licensed under a Creative Commons Attribution-NonCommercial 3.0 Unported Licence.

22 S. S. Niavol, M. Budde, A. Papadogianni, M. Heilmann, H. M. Moghaddam, C. M. Aldao, G. Ligorio, E. J. W. List Kratochvil, J. M. J. Lopes, N. Barsan, O. Bierwagen and F. Schipani, *Sens. Actuators, B*, 2020, **325**, 128797.
23 M. Sinha, S. Neogi, R. Mahapatra, S. Krishnamurthy and R. Ghosh, *Sens. Actuators, B*, 2021, **336**, 129729.
24 V. S. Bhati, M. Kumar and R. Banerjee, *J. Mater. Chem. C*, 2021, **9**, 8776–8808.
25 O. C. Compton and S. T. Nguyen, *Small*, 2010, **6**, 711–723.
26 C. Chang, W. Chen, Y. Chen, Y. Chen, Y. Chen, F. Ding, C. Fan, H. Jin Fan, Z. Fan, C. Gong, Y. Gong, Q. He, X. Hong, S. Hu, W. Hu, W. Huang, Y. Huang, W. Ji, D. Li, L.-J. Li, Q. Li, L. Lin, C. Ling, M. Liu, N. Liu, Z. Liu, K. Ping Loh, J. Ma, F. Miao, H. Peng, M. Shao, L. Song, S. Su, S. Sun, C. Tan, Z. Tang, D. Wang, H. Wang, J. Wang, X. Wang, X. Wang, A. T. S. Wee, Z. Wei, Y. Wu, Z.-S. Wu, J. Xiong, Q. Xiong, W. Xu, P. Yin, H. Zeng, Z. Zeng, T. Zhai, H. Zhang, H. Zhang, Q. Zhang, T. Zhang, X. Zhang, L.-D. Zhao, M. Zhao, W. Zhao, Y. Zhao, K.-G. Zhou, X. Zhou, Y. Zhou, H. Zhu, H. Zhang and Z. Liu, *Acta Phys.-Chim. Sin.*, 2021, **37**, 2108017.
27 K. Yang, X. Wang, H. Li, B. Chen, X. Zhang, S. Li, N. Wang, H. Zhang, X. Huang and W. Huang, *Nanoscale*, 2017, **9**, 5102–5109.
28 T. K. Dang, N. T. Son, N. T. Lanh, P. H. Phuoc, N. N. Viet, L. V. Thong, C. M. Hung, N. V. Duy, N. D. Hoa and N. V. Hieu, *J. Alloys Compd.*, 2021, **879**, 160457.
29 H. Zhang, L. Yu, Q. Li, Y. Du and S. Ruan, *Sens. Actuators, B*, 2017, **241**, 109–115.
30 M. Liu, Z. Wang, P. Song, Z. Yang and Q. Wang, *Sens. Actuators, B*, 2021, **340**, 129946.
31 V. Montes Garcia, M. A. Squillaci, M. Diez Castellnou, Q. K. Ong, F. Stellacci and P. Samori, *Chem. Soc. Rev.*, 2021, **50**, 1269–1304.
32 R. Jolly Bose, N. Illyasukutty, K. S. Tan, R. S. Rawat, M. Vadakke Matham, H. Kohler and V. P. Mahadevan Pillai, *Appl. Surf. Sci.*, 2018, **440**, 320–330.
33 J. Ma, X. Xiao, Y. Zou, Y. Ren, X. Zhou, X. Yang, X. Cheng and Y. Deng, *Small*, 2019, **15**, e1904240.
34 Q. A. Drmosh, Y. A. Al Wajih, I. O. Alade, A. K. Mohamedkhair, M. Qamar, A. S. Hakeem and Z. H. Yamani, *Sens. Actuators, B*, 2021, **338**, 129851.
35 Y. Wang, X. Meng, M. Yao, G. Sun and Z. Zhang, *Ceram. Int.*, 2019, **45**, 13150–13157.
36 J. S. Kim, H. W. Yoo, H. O. Choi and H. T. Jung, *Nano Lett.*, 2014, **14**, 5941–5947.
37 Y. Zhou, Q. Bao, L. A. L. Tang, Y. Zhong and K. P. Loh, *Chem. Mater.*, 2009, **21**, 2950–2956.
38 X. Huang, X. Zhou, S. Wu, Y. Wei, X. Qi, J. Zhang, F. Boey and H. Zhang, *Small*, 2010, **6**, 513–516.
39 K. O'Connell and J. R. Regalbuto, *Catal. Lett.*, 2015, **145**, 777–783.
40 A. Weibel, R. Bouchet, F. Boulc and P. Knauth, *Chem. Mater.*, 2005, **17**, 2378–2385.
41 F. Qu, X. Zhou, B. Zhang, S. Zhang, C. Jiang, S. Ruan and M. Yang, *J. Alloys Compd.*, 2019, **782**, 672–678.
42 P. Li, H. Zhan, S. Tian, J. Wang, X. Wang, Z. Zhu, J. Dai, Y. Dai, Z. Wang, C. Zhang, X. Huang and W. Huang, *ACS Appl. Mater. Interfaces*, 2019, **11**, 13624–13631.
43 H. Y. Lee, Y. C. Heish and C. T. Lee, *J. Alloys Compd.*, 2019, **773**, 950–954.
44 J. Hu, C. Zou, Y. Su, M. Li, X. Ye, B. Cai, E. S. W. Kong, Z. Yang and Y. Zhang, *Sens. Actuators, B*, 2018, **270**, 119–129.
45 B. I. Adamu, A. Falak, Y. Tian, X. Tan, X. Meng, P. Chen, H. Wang and W. Chu, *ACS Appl. Mater. Interfaces*, 2020, **12**, 8411–8421.
46 B. Zhang, G. Liu, M. Cheng, Y. Gao, L. Zhao, S. Li, F. Liu, X. Yan, T. Zhang, P. Sun and G. Lu, *Sens. Actuators, B*, 2018, **261**, 252–263.
47 S. Zhao, Y. Shen, F. Hao, C. Kang, B. Cui, D. Wei and F. Meng, *Appl. Surf. Sci.*, 2021, **538**, 148140.
48 M. Shafiei, J. Bradford, H. Khan, C. Piloto, W. Wlodarski, Y. Li and N. Motta, *Appl. Surf. Sci.*, 2018, **462**, 330–336.
49 X. Jiang, H. Tai, Z. Ye, Z. Yuan, C. Liu, Y. Su and Y. Jiang, *Mater. Lett.*, 2017, **186**, 49–52.
50 X. Wang, Y. Liu, J. Dai, Q. Chen, X. Huang and W. Huang, *Chem.–Eur. J.*, 2020, **26**, 3870–3876.
51 X. Wang, Z. Wang, J. Zhang, X. Wang, Z. Zhang, J. Wang, Z. Zhu, Z. Li, Y. Liu, X. Hu, J. Qiu, G. Hu, B. Chen, N. Wang, Q. He, J. Chen, J. Yan, W. Zhang, T. Hasan, S. Li, H. Li, H. Zhang, Q. Wang, X. Huang and W. Huang, *Nat. Commun.*, 2018, **9**, 3611.
52 J. Bao, S. Zeng, J. Dai, X. Wang, Q. Liu, H. Li, X. Huang and W. Huang, *Chem. Commun.*, 2021, **57**, 5590–5593.
53 H. J. Park, W. J. Kim, H. K. Lee, D. S. Lee, J. H. Shin, Y. Jun and Y. J. Yun, *Sens. Actuators, B*, 2018, **257**, 846–852.
54 J. Zha, Z. Yuan, Z. Zhou, Y. Li, J. Zhao, Z. Zeng, L. Zhen, H. Tai, C. Tan and H. Zhang, *Small Struct.*, 2021, **2**, 2100067.
55 Jyoti and G. D. Varma, *J. Alloys Compd.*, 2019, **806**, 1469–1480.
56 S. Li, Z. Wang, X. Wang, F. Sun, K. Gao, N. Hao, Z. Zhang, Z. Ma, H. Li, X. Huang and W. Huang, *Nano Res.*, 2017, **10**, 1710–1719.
57 M. Reddeppa, N. T. KimPhung, G. Murali, K. S. Pasupuleti, B. G. Park, I. In and M. D. Kim, *Sens. Actuators, B*, 2021, **329**, 129175.
58 M. S. Choi, H. G. Na, J. H. Bang, A. Mirzaei, S. Han, H. Y. Lee, S. S. Kim, H. W. Kim and C. Jin, *Sens. Actuators, B*, 2021, **326**, 128801.
59 X. Chen, S. Zhao, P. Zhou, B. Cui, W. Liu, D. Wei and Y. Shen, *Sens. Actuators, B*, 2021, **328**, 129070.
60 R. Kumar, N. Goel and M. Kumar, *ACS Sens.*, 2017, **2**, 1744–1752.
61 B. Liu, X. Liu, Z. Yuan, Y. Jiang, Y. Su, J. Ma and H. Tai, *Sens. Actuators, B*, 2019, **295**, 86–92.
62 N. D. K. Tu, J. Choi, C. R. Park and H. Kim, *Chem. Mater.*, 2015, **27**, 7362–7369.

 © 2022 The Author(s). Published by the Royal Society of Chemistry

ADVANCED MATERIALS
www.advmat.de

Check for updates

A Self-Powered Portable Nanowire Array Gas Sensor for Dynamic NO_2 Monitoring at Room Temperature

Shiyu Wei, Zhe Li, Krishnan Murugappan, Ziyuan Li, Fanlu Zhang, Aswani Gopakumar Saraswathyvilasam, Mykhaylo Lysevych, Hark Hoe Tan, Chennupati Jagadish, Antonio Tricoli,* and Lan Fu**

The fast development of the Internet of Things (IoT) has driven an increasing consumer demand for self-powered gas sensors for real-time data collection and autonomous responses in industries such as environmental monitoring, workplace safety, smart cities, and personal healthcare. Despite intensive research and rapid progress in the field, most reported self-powered devices, specifically NO_2 sensors for air pollution monitoring, have limited sensitivity, selectivity, and scalability. Here, a novel photovoltaic self-powered NO_2 sensor is demonstrated based on axial p–i–n homojunction InP nanowire (NW) arrays, that overcome these limitations. The optimized innovative InP NW array device is designed by numerical simulation for insights into sensing mechanisms and performance enhancement. Without a power source, this InP NW sensor achieves an 84% sensing response to 1 ppm NO_2 and records a limit of detection down to the sub-ppb level, with little dependence on the incident light intensity, even under <5% of 1 sun illumination. Based on this great environmental fidelity, the sensor is integrated into a commercial microchip interface to evaluate its performance in the context of dynamic environmental monitoring of motor vehicle exhaust. The results show that compound semiconductor nanowires can form promising self-powered sensing platforms suitable for future mega-scale IoT systems.

1. Introduction

The large-scale implementation of Internet of Things (IoT) technology over the recent decade has increased consumer demand for reliable and accurate gas sensor networks for real-time data collection and autonomous response in applications such as air pollution monitoring, industrial chemical hazard detection, smart cities, and personal healthcare.[1,2] Given the immense number of sensors required to feed into such a network, there is an urgent need to obtain sensors with characteristics such as being micro- or nanosized, ever superior sensitivity and detectivity, shorter response times, and critically require orders of magnitude less power consumption than current commercial devices.[3,4] Conventional battery-powered sensors not only require continuous power supply during operation but are difficult to miniaturize, limiting their use in highly integrated

S. Wei, Z. Li, F. Zhang, H. H. Tan, C. Jagadish, L. Fu
Australian Research Council Centre of Excellence for Transformative Meta-Optical Systems
Department of Electronic Materials Engineering
Research School of Physics
The Australian National University
Canberra, ACT 2601, Australia
E-mail: lan.fu@anu.edu.au

Z. Li, A. G. Saraswathyvilasam
Department of Electronic Materials Engineering
Research School of Physics
The Australian National University
Canberra, ACT 2601, Australia
E-mail: zhe.li@anu.edu.au

K. Murugappan, A. Tricoli
Nanotechnology Research Laboratory
Research School of Chemistry
College of Science
The Australian National University
Canberra, ACT 2601, Australia

K. Murugappan
Commonwealth Scientific and Industrial Research Organisation (CSIRO)
Mineral Resources
Private Bag 10, Clayton South, Victoria 3169, Australia

M. Lysevych
Australian National Fabrication Facility
The Australian National University
Canberra, ACT 2601, Australia

A. Tricoli
Nanotechnology Research Laboratory
School of Biomedical Engineering, Faculty of Engineering
The University of Sydney
NSW 2006, Australia
E-mail: antonio.tricoli@anu.edu.au

The ORCID identification number(s) for the author(s) of this article can be found under https://doi.org/10.1002/adma.202207199.

DOI: 10.1002/adma.202207199

 © 2023 Wiley-VCH GmbH

15214095, 2023, 12, Downloaded from https://advanced.onlinelibrary.wiley.com/doi/10.1002/adma.202207199 by Nanjing Tech University, Wiley Online Library on [31/01/2025]. See the Terms and Conditions (https://onlinelibrary.wiley.com/terms-and-conditions) on Wiley Online Library for rules of use; OA articles are governed by the applicable Creative Commons License

systems.[5,6] Self-powered sensing systems are one of the most promising solutions to overcome both the power supply issue and achieve miniaturization.[7–9] Such systems can harvest energy from the environment such as sunlight, body motion, and heat, to realize zero-power consumption.[6,10] Among these different types of self-powered gas sensors, photovoltaic (PV) effect based sensors are being explored, as the PV effect and gas sensing function can be achieved simultaneously within the same junction or semiconductor active layer.[11] This simplified structure is low-cost and has a simpler fabrication process, which are desirable characteristics for their possible integration into next generation IoT systems.[12]

Over the past decade, self-powered PV gas sensors have been demonstrated by forming p–n or Schottky junctions based on a variety of bulk or low-dimensional materials, including 2D materials (e.g., graphene, WS_2, and WSe_2),[13,14] metal oxide,[15] perovskite,[16] carbon nanotube,[17] and hyper-doped Si.[18,19] However, most of the reported self-powered gas sensors show issues such as limited sensitivity and selectivity at room temperature,[20] signal drifts and lack of stability over time,[21,22] as well as challenges in integration with complementary metal-oxide-semiconductor (CMOS) based technology.[16] Also, in these sensors, the junction regions are often buried within the devices and do not interact with gas molecules due to the limitation of device architectures.[11]

III–V compound semiconductors are the key materials for record-efficiency single- and multijunction solar cells[23] due to their direct and tunable bandgap, superior optical and electronic properties. III–V compound semiconductor nanowires (NWs) have also been widely investigated for solar cell applications.[24,25] Compared with other III–V NWs, InP NWs show lower surface recombination velocity and higher carrier mobility, which is ideal for solar cell applications. So far, most of the high-efficiency NW solar cells have been demonstrated in InP-NW-based systems.[26,27] Recently, it has been reported that InP NW array based chemiresistive sensors can achieve high sensitivity, selectivity, and long-term stability to NO_2 at room temperature.[28] Combining their superior PV properties and NO_2 sensitivity, InP NW array is a promising candidate for self-powered sensing applications. To date, despite that a few different III–V nanowire materials were investigated for gas and biomolecule sensing,[29–31] they have rarely been demonstrated as self-powered gas sensors.

In this work, we report a novel InP NW array based gas sensor architecture that combines a PV junction and an active sensing region to achieve highly sensitive, selective, and reproducible self-powered NO_2 sensing at room temperature. Prior to NW growth, the NW and device structure were designed via numerical simulation that models PV NW sensing mechanisms and optimizes performance. Then axial junction based InP NW arrays were grown according to the simulation optimized structures by selective-area metal–organic vapor-phase epitaxy (SA-MOVPE).[32] The NW crystal structure and optical properties were characterized by transmission electron microscopy (TEM), photoluminescence (PL), and time-resolved photoluminescence (TRPL). The NW array sensor devices were designed and fabricated to enable robust contacts for signal extraction, while maintaining a large, exposed NW active area for gas sensing. Without an external power source, the device operates as PV gas sensor and responds to NO_2 with a high sensitivity, even under a much weaker light intensity than a standard one sun illumination. This device also presents a high selectivity, and a sub-ppb level limit of detection (LOD). Finally, we demonstrate that our NW sensor can be readily integrated onto a microchip board for on-field dynamic NO_2 concentration measurements from vehicle exhaust pollutant. Our results pave the way for a miniaturized, power source-free nanoscale sensor platform for future IoT system applications.

2. Results and Discussion

2.1. Device Design and Self-Powered Sensing Mechanism

When applied to gas sensing, the 1D nanoscale NW geometry provides an abundant and engineerable surface area for adsorbing and interacting with gas molecules. For an InP NW array, it is found that once the NO_2 molecules are adsorbed onto the NW surface, the resultant acceptor-like surface states capture electrons from the NW surface,[28] turning it into a high resistance state and leading to a measurable signal of the adsorbed NO_2. Based on this fundamental mechanism and PV effect attainable by the formation of a p–n junction, we have conceptualized an InP NW array PV sensor to achieve gas sensing and self-powered carrier collection simultaneously. Under light illumination, a photocurrent is generated in the p–n junction embedded NWs, i.e., a short-circuit current (I_{SC}) without an external power supply. NO_2 exposure effectively modulates the photocurrent flowing through the p–n junction due to the charge (electrons) transfer from the NWs to the adsorbed NO_2 molecules, causing a notable change to the photocurrent. Therefore, the p–n junction-based sensing response (R) can be defined as

$$R = \frac{\left(I_0 - I_{\text{gas}}\right)}{I_0} \times 100\% \quad (1)$$

where I_0 and I_{gas} denote the initial I_{SC} in air and the final I_{SC} in response to gas adsorption, respectively. I_{gas} can be either greater or smaller than I_0 as will be discussed later, leading to either a positive or negative value for the measured R.

To better understand the PV NW sensing mechanism, we investigated and compared two common axial p–n junction structures based on material growth, which has been extensively studied and optimized in our previous work: [25,31–33] NW array with i-(intrinsic) and n-doped segments grown on a p-doped InP substrate, referred as p–i–n hereafter (**Figure 1**a), and NW array with i-(intrinsic) and p-doped segments grown on an n-doped InP substrate, referred as n–i–p hereafter (Figure 1b). To allow effective gas sensing and NW top contact for signal extraction, a light illumination window is created at the top of the NW for gas exposure (Figure 1a). Considering the mechanical stability of the NW array, the size of the exposure window cannot be too large. The length of the whole exposed NW is set to be 700 nm (500 nm of the doping segment and 200 nm of the exposure window depth) in the simulation, which is a realistic size that can be achieved reliably by device fabrication. By varying the top i–n or i–p junction depth of these two types of NW structures

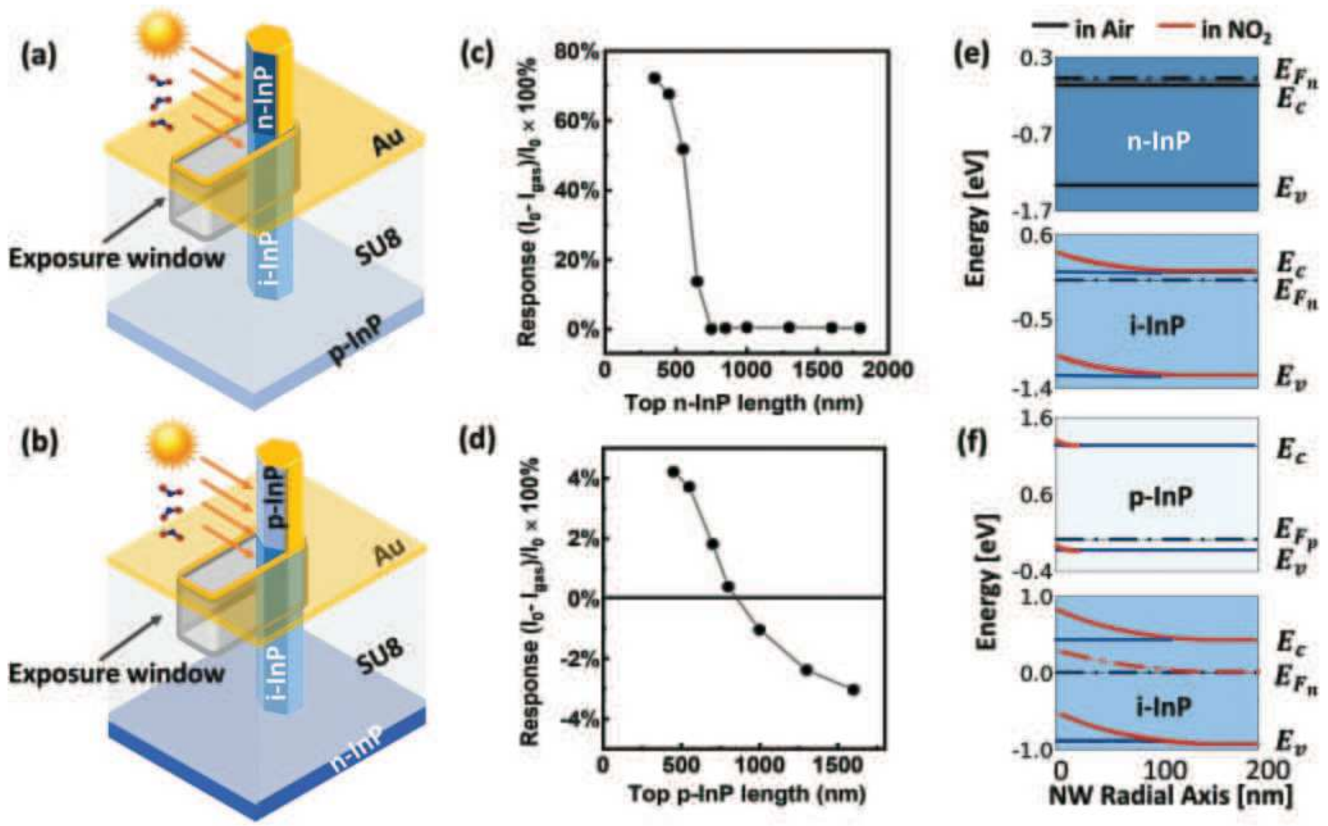

Figure 1. a,b) 3D schematic of a unit cell of a p–i–n and an n–i–p NW based sensor, respectively. c,d) Simulated gas sensing response versus NW top segment length. e,f) Band diagram of the respective structures before (i.e., in air) and after gas exposure (i.e., in NO_2).

shown in Figure 1a,b, we simulated I_{SC} before and after gas exposure to calculate sensing response by Equation (1) using COMSOL Multiphysics (see Table S1 and Figure S1 in the Supporting Information for details of model setup).

It was found that the relative position of i–n or p junction with respect to the exposure window played a critical role in sensing performance, as plotted in Figure 1c,d, respectively. For the p–i–n structure, large positive responses are obtained as shown in Figure 1c, whereas for the n–i–p structure, the response changes from positive to negative within a small range of values with the junction depth increasing as shown in Figure 1d. The polarity of the sensing response for different structures can be explained with the aid of a band diagram of the NW top junction (Figure 1e,f), where the case of n-/p-doped top segment length is set to 450 nm. For the case of a p–i–n structure under illumination, NO_2 gas molecules adsorbed on the top n-doped segment and i-region of the NW (lightly n-doped due to background impurity doping during SA-MOVPE growth, based on experimental data[33]) will form surface states that act as electron acceptors, giving rise to an upward surface band bending as shown in Figure 1e.[34,35] As a result, the majority carrier (i.e., electron) concentration in both the top n-doped segment and the i-region is reduced, leading to a decreased photocurrent and hence a positive sensing response as calculated by Equation (1). Since the top n-segment is highly doped to 3×10^{18} cm^{-3}, the corresponding band bending (and carrier depletion) is much less evident than that of the i-segment when subjected to the same amount of gas adsorption. Therefore, the sensing response of the p–i–n structure decreases sharply as the top n-segment length increases and becomes longer than the exposure window such that no i-segment is exposed for sensing, since most carrier depletion occurs within the i-layer.

When an n–i–p NW sensor is exposed to NO_2, the resultant upward band bending (Figure 1f) in the top p-doped segment increases the majority carrier (i.e., hole) concentration and thus the photocurrent shows an enhancement, whereas in the i-region the band bending depletes the majority carriers (i.e., electron), leading to a decreased photocurrent in contrast to the p-segment. For a shallow i–p junction as in Figure 1f, due to the large band bending in the i-segment, electron depletion plays a dominant role compared to the hole enhancement in the top p-doped segment. The net effect is a decreased photocurrent and positive sensing response. As the junction depth increases, the hole concentration enhancement becomes increasingly larger, which eventually leads to increased photocurrent and thus a negative sensing response. By comparing the response curves for the two structures, the p–i–n-based device is a better candidate for high-performance oxidative gas sensing. The length of the top n-segment plays a critical role, which should be kept as short as possible just for the electrical contact, so that the i-region can be sufficiently exposed for a better sensing performance. For the n–i–p structure, varying the junction depth plays limited role due to its bipolar sensing response in the top

© 2023 Wiley-VCH GmbH

15214095, 2023, 12, Downloaded from https://advanced.onlinelibrary.wiley.com/doi/10.1002/adma.202207199 by Nanjing Tech University, Wiley Online Library on [31/01/2025]. See the Terms and Conditions (https://onlinelibrary.wiley.com/terms-and-conditions) on Wiley Online Library for rules of use; OA articles are governed by the applicable Creative Commons License

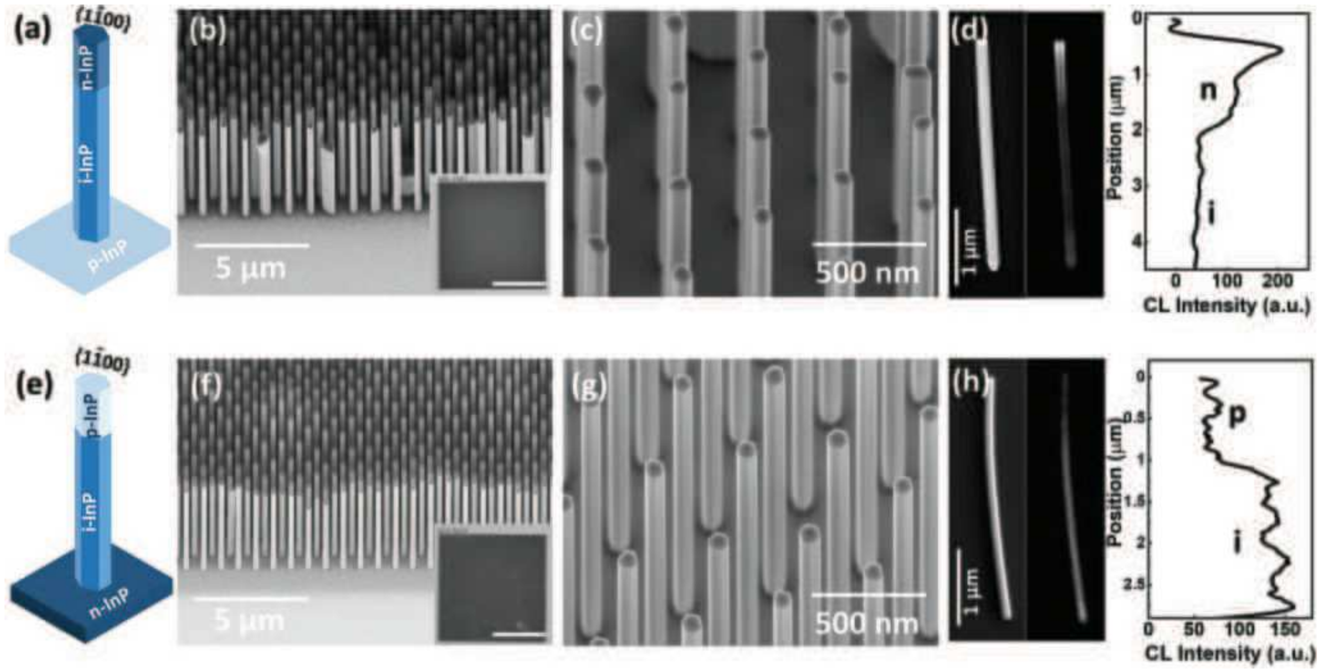

Figure 2. a–c) Schematic and SEM images of i–n InP NW on p-doped InP substrate (p–i–n). e–g) Schematic and SEM images of i–p InP NW on n-doped InP substrate (n–i–p). d,h) SEM and corresponding cathodoluminescence (CL) image with CL intensity profile of p–i–n and n–i–p InP NW, respectively.

p-segment and i-region. As a result, the absolute response value is less than 5% with all junction depths considered.

2.2. Experimental Results and Discussion

To confirm the simulation results, two types of axial homojunction InP NW structures, p–i–n and n–i–p, illustrated in **Figure 2**a,e, were grown by SA-MOVPE (see details in the Experimental Section and Figure S2, Supporting Information) under the same conditions as reported previously.[34] The scanning electron microscope (SEM) images are illustrated in Figure 2b,c,f,g, showing well-ordered InP NWs with a taper-free hexagonal cylinder shape. The two structures display a similar configuration due to the same growth time and substrate pattern design, with a diameter of around 110 nm, a length of 3–4 μm, and an inter-spacing of 600 nm. The crystal structure is determined by TEM analysis, confirming a pure wurtzite crystalline structure (Figure S3 in the Supporting Information).

The doping profiles of the two NW structures were characterized by cathodoluminescence (CL) of single NWs,[32] transferred from NW arrays to a Si substrate, as shown in Figure 2d,h, clearly indicating the two different doping segments within these two structures. The bright-and-dark contrast of the CL panchromatic image[36] arises from the different doping type and level of NW along the axial direction. Figure 2d shows that the n-segment has a much stronger luminescence than the i-segment, due to a higher electron concentration, which increases the transition probability of a donor-valence band transition, in comparison to an intrinsic-valence band transition. While for the p-doped top segment (Figure 2f), the transition probability of an acceptor-valence band transition is lower than that of an intrinsic-valence band transition, leading to a lower luminescence in the p-segment than that of the i-segment. The SEM-CL characterization indicates that both n–i–p and p–i–n structures grown by the SA-MOVPE method present well-defined junctions. The NWs were also transferred to a SiO_2/Si substrate for PL and time-resolved photoluminescence (TRPL) measurements (Figure S4, Supporting Information). It is found that the lifetimes of i–p and i–n nanowire segments are ≈0.7–0.4 and ≈0.3–0.1 ns, respectively. Although these values are lower than the best lifetime values reported previously by our group,[33] it is adequate to realize thePV effect (**Figure 3**) for the self-powered sensing application.

For the PV and sensing characterization, the as-grown NW arrays were then fabricated into an innovative NW PV sensor structure as illustrated in Figure 3a, by planarization (with polymer SU8-5), tilt-angle metal contact deposition[28] and gas exposure window opening steps (detailed in the Experimental Section and Figure S5, Supporting Information). It is worth noting that based on the insight from our simulation, we found that the length of the window structure is critical for high sensing performance. A long exposed i-region will maximize the NW surface interaction with the target gas molecules and thus the sensing response insight from the simulation result. Figure 3b presents the SEM images of a fabricated sensor, showing that this novel design has been successfully implemented with the top NW side wall partially covered with metal for electrical contact and the other part exposed to air for gas sensing. The exposure window at the top of NW was successfully obtained through carefully tailoring the Inductively Coupled Plasma (ICP) etching process (details in the Experimental Section), to achieve an average exposure length of ≈700 ± 50 nm. The 50 nm variation is estimated by measuring over 50 NWs by

 © 2023 Wiley-VCH GmbH

15214095, 2023, 12, Downloaded from https://advanced.onlinelibrary.wiley.com/doi/10.1002/adma.202207199 by Nanjing Tech University, Wiley Online Library on [31/01/2025]. See the Terms and Conditions (https://onlinelibrary.wiley.com/terms-and-conditions) on Wiley Online Library for rules of use; OA articles are governed by the applicable Creative Commons License

15214095, 2023, 12, Downloaded from https://advanced.onlinelibrary.wiley.com/doi/10.1002/adma.202207199 by Nanjing Tech University, Wiley Online Library on [31/01/2025]. See the Terms and Conditions (https://onlinelibrary.wiley.com/terms-and-conditions) on Wiley Online Library for rules of use; OA articles are governed by the applicable Creative Commons License

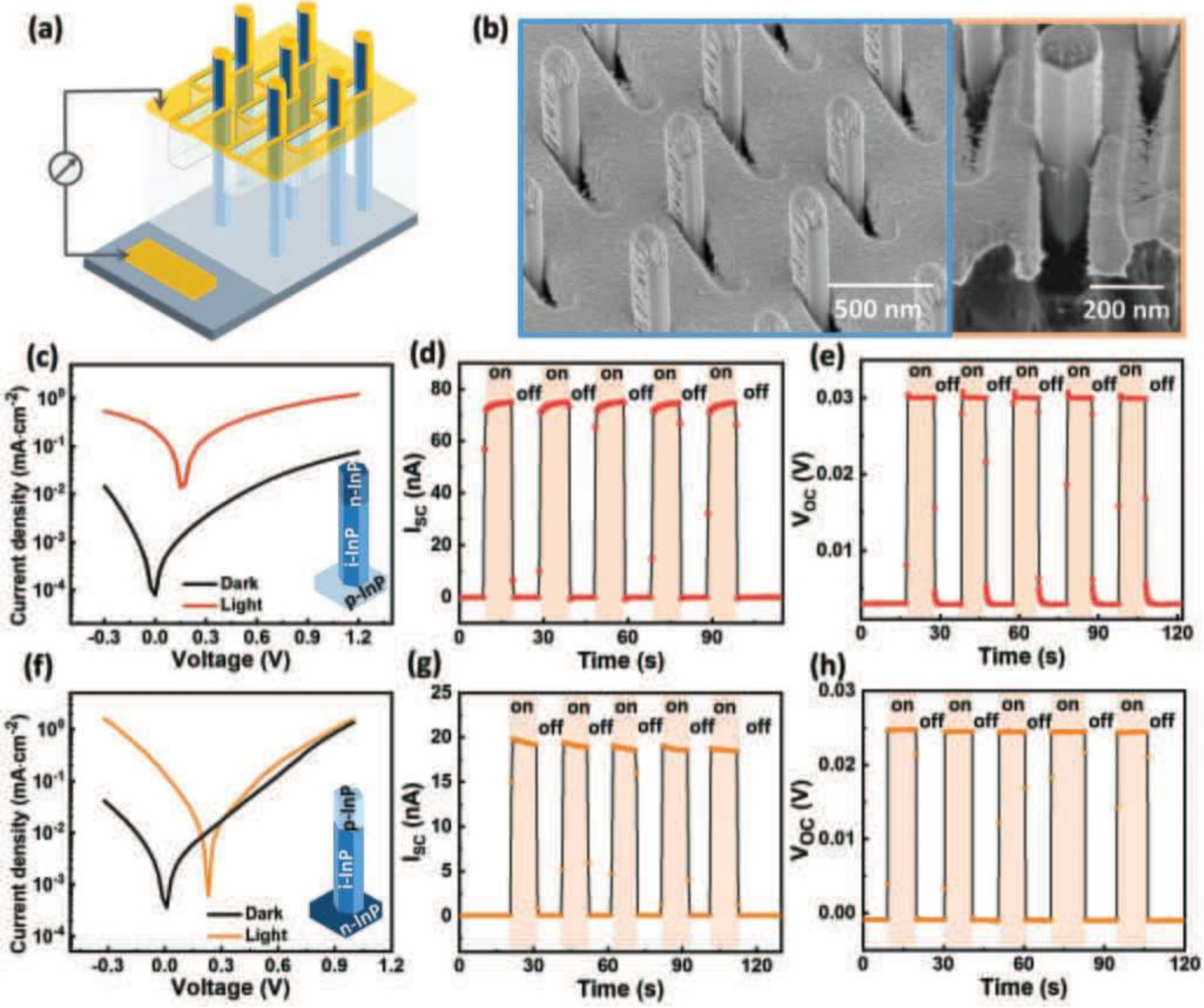

Figure 3. a) Schematic of as-fabricated InP NW array sensor. b) SEM image from the top of the device, with a cross-sectional view highlighting the window at the top junction region opened for gas sensing (right panel). c,f) Dark/light *I*–*V* characteristics of the p–i–n and n–i–p InP NW arrays (light source: solar simulator @AM1.5, 42.3 mW cm^{-2}). d,g) Time-dependent short-circuit current (I_{SC}) of p–i–n and n–i–p InP NW device, respectively; "on" and "off" correspond to the states with and without illumination. e,h) Time-dependent open-circuit voltage (V_{OC}) of p–i–n and n–i–p InP NW device, respectively.

SEM from the fabricated NW array device, which is difficult to avoid due to fabrication induced nonuniformity.

The electrical property of two NW device structures (p–i–n and n–i–p) was characterized by standard dark/light current–voltage (*I*–*V*) measurements as shown in Figure 3c,f. The dark *I*–*V* curves of both devices show the typical diode characteristic. When illuminated under a solar simulator @AM1.5 at a power of 42.3 mW cm^{-2}, both devices show PV behavior, indicating successful device fabrication. The p–i–n and n–i–p structures have an I_{SC} density of 0.19 and 0.13 mA cm^{-2} (device area of 200 × 200 µm^2), and an open-circuit voltage (V_{OC}) of 0.16 and 0.25 V, respectively. The time-dependent photoresponses are shown in Figure 3d,e,g,h for I_{SC} and V_{OC}, where distinct "ON" and "OFF" PV states can be well manipulated to fit into the subsequent self-powered gas sensing measurements.

2.3. Laboratory Self-Powered Gas Sensing Performance

Sensing performance was characterized by detecting NO_2 in a gas sensing setup schematically illustrated in **Figure 4**a. The gas sensing chamber was configured to be illuminated by a solar simulator (with conditions equivalent to where the light *I*–*V* characteristics were measured) at zero bias to characterize self-powered sensing performance. Prior to gas injection, the device was placed in the gas sensing chamber with illumination and constant air flow to generate steady-state I_{SC}. As the NO_2 gas injection started, I_{SC} either decreased or increased, depending on the device structure as discussed earlier, and then reached a saturation. When NO_2 gas was switched off and air flow was resumed, I_{SC} recovered back to the initial base level. Utilizing the variation of I_{SC} as a sensing

 © 2023 Wiley-VCH GmbH

ADVANCED MATERIALS
www.advmat.de

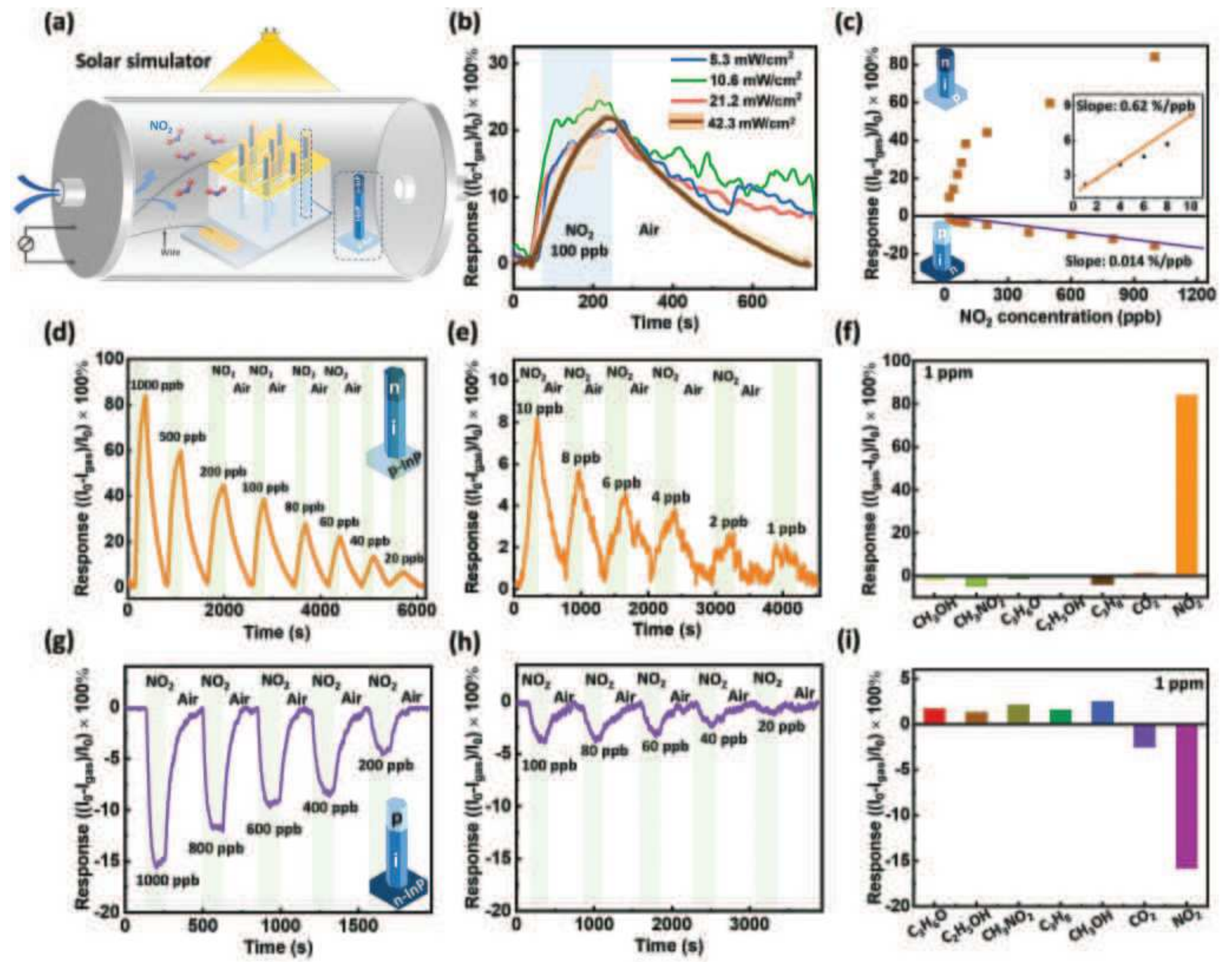

Figure 4. a) Schematic of self-powered sensing measurement setup under illumination of a solar simulator @AM1.5. b) Time-dependent response measured from a p–i–n device under different illumination intensities. The averaged time-dependent sensing response and standard deviation range under a light intensity of 42.3 mW cm^{-2} is shown as the shaded area. c) Summarized NO_2 concentration versus calculated response for p–i–n and n–i–p NW structures. The inset shows an enlarged graph of p–i–n sensing responses to 1–10 ppb NO_2. d,e,g,h) Sensing response measured from p–i–n (d,e), and n–i–p (g,h) NW structures for different range of NO_2 concentrations. f,i) Target gas selectivity measurement of p–i–n (f) and n–i–p (i) NW structure, respectively, with a gas concentration of 1 ppm for methanol, methyl nitrides, acetone, ethanol, propane, CO_2, and NO_2. The sensing measurements in (d–i) were performed under light intensity of 42.3 mW cm^{-2}.

signal, the NO_2 sensing R was then calculated according to Equation (1).

To find out the threshold light intensity for our self-powered NW sensor operation and the capacity of the device working under various illumination conditions, the p-i-n device was measured under a series of attenuated light intensities tuned by a neutral density filter. Figure 4b shows that with 100 ppb NO_2, the R value was consistent within the standard deviation range of R (22 ± 4.9%, for 10 cycles of sensing measurement) under a series of decreased light intensities from the original level 42.3 mW cm^{-2} (shaded area), even when the light intensity was decreased to 10% of its original level (or <5% 1-sun condition). Under the reduced light intensity, the baseline I_{SC} (i.e., I_0) and the gas adsorption induced I_{SC} (i.e., I_{gas}) decreases simultaneously, such that the R calculated by Equation (1) remains at a similar value. Such light-intensity independent performance is highly desirable for sensing in a realistic environment since atmospheric solar irradiation usually varies drastically during the day. Figure 4b also indicates minor variation of sensing response time with the light intensity, which is discussed in Figure S6, Supporting Information.

Figure 4d,e shows the time-dependent response of the p–i–n device in the concentration range 1–1000 ppb. The results from the n–i–p sample are illustrated in Figure 4g,h, with a concentration range of 20–1000 ppb. Figure 4c summarizes the corresponding concentration-dependent sensing response for both p–i–n and n–i–p samples with their linear-fitting slope (L) which is defined as device sensitivity, marking the sensor output signal changes with unit analytes input. It is noticeable that the absolute value of response at 1000 ppb and the device sensitivity for n–i–p structure (15.9%, 0.014%/ppb) is much smaller than those of p–i–n structure (84.1%, 0.62%/ppb),

 © 2023 Wiley-VCH GmbH

15214095, 2023, 12, Downloaded from https://advanced.onlinelibrary.wiley.com/doi/10.1002/adma.202207199 by Nanjing Tech University, Wiley Online Library on [31/01/2025]. See the Terms and Conditions (https://onlinelibrary.wiley.com/terms-and-conditions) on Wiley Online Library for rules of use; OA articles are governed by the applicable Creative Commons License

verifying the results of the numerical simulation that predicts the NW with unipolar sensing response from i–n top junction is superior to the bipolar one from i–p junction. According to the aforementioned sensing mechanism in Section 2.1, I_{SC} of the n–i–p structure increases with NO_2 injection, thus giving a negative sensing response, in contrast to the decreasing I_{SC} and a positive sensing response from the p–i–n structure.[39,40]

From Figure 4d,g, it is noted the extracted response time of the n–i–p device (≈50 s) is shorter than that of the p–i–n device (≈140 s). This difference mainly results from two factors: i) the transient photocurrent change is intrinsically limited by the drift of photo-generated carriers within the exposure window, which is directly proportional to the built-in electric field formed near the top i–n or i–p junction as determined by their doping profiles. The electric field distribution for the two junction regions can be found in Figure S7 in the Supporting Information, which implies that the carrier drift velocity in n–i–p structure is larger than that in the p–i–n structure, leading to a faster transient photocurrent change and thus a shorter response time; ii) different doping type (Si and Zn) in the top n/p segment of two NW structures may also result in different surface activation energy, thereby changing the reaction rate constant as gas particles interact with the NW surface and affecting the response time.[41,42]

For the more sensitive p–i–n structure, a linear correlation of response versus concentration can be observed at two separate concentration regimes, with a smaller slope at the high concentration range (>100 ppb). This phenomenon also exists in previously reported work, which indicates that the sensor saturates gradually due to the limited number of surface adsorption sites.[22,37] The superior sensitivity performance of the p–i–n structure is also indicated by the measurement of an extremely low NO_2 concentration (1 ppb), where a distinguishable sensing response of 2% can still be obtained as shown in Figure 4e. Based on the classification of International Union of Pure and Applied Chemistry,[38] limit of detection (LOD) is defined as a concentration of the analyte which causes a response 3 times higher than the noise level of the device (i.e., in the absence of the analyte), i.e., LOD = $3 \times (RMS_{noise}/L)$. Gas sensor noise was calculated via root-mean-square deviation (RMS_{noise}) processing from 100 consecutive baseline data points taken under the exposure to air.[39] From the baseline in Figure 4e, the $RMS_{noise} = 0.10\%$ and $L = 0.62\%/\text{ppb}$ (obtained from the inset of Figure 4c), so the calculated LOD is 0.49 ppb, which is the lowest record achieved by a self-powered gas sensor to the best of our knowledge. Also, the effect of humidity on this p–i–n sensor was investigated under various relative humidity (RH) levels (Figure S8, Supporting Information). It was found that even with a high concentration of water vapor (RH 65%), the NW sensor maintains its high sensitivity despite a small degradation in sensing response (< 30%) compared to the dry condition (RH 0%), demonstrating excellent humidity tolerance.

To investigate the device reproducibility, the better performing p–i–n device was repeatedly exposed to NO_2 (at the same concentration) and we observed a consistent sensing response for an extended period of time (Figure S9c,d in the Supporting Information). Furthermore, both the n-i-p and p–i–n structured devices demonstrated a high selectivity to NO_2 as shown in Figure 4f,i. At 1 ppm level, the sensing response of these two devices to other organic vapors and common atmosphere gases (methanol (CH_3OH), ethanol (C_2H_5OH), methyl nitrite (CH_3NO_2), carbon dioxide (CO_2), and propane (C_3H_8)) are negligible compared to NO_2. Besides I_{SC}, the time-dependent V_{OC} can be also measured as an alternative readout signal that can be useful for integration with a digital microchip circuit board, as demonstrated in Figures S9 and S10 in the Supporting Information.[13] The V_{OC} sensing response measured from both the p–i–n and n-i-p NW sensors presents similar characteristics to what was observed with I_{SC}, i.e., the magnitude of the sensing response is concentration dependent, and V_{OC} decreases (increases) similarly when exposed to NO_2 for the p–i–n (n-i-p) structure.

Compared with different types of recently reported self-powered gas-sensing devices as summarized in **Table 1**, our p–i–n InP NW NO_2 sensor shows an overall outstanding performance with a high response (84%/ppm), the lowest LOD (≈0.49 ppb), and an ability to work stably under a natural light source with varied intensity at room temperature. The NW sensor's performance reported here is also comparable to the best reported nonself-powered sensors[43,44] taking into consideration of the operation conditions, which are important figures of merit for practical applications. For example, as summarized in Table S2 (Supporting Information), a well-designed layer-like WO_3 sensor can achieve lower LOD of 0.243 ppb, but required a high operation temperature of 160 °C.[43] A single-layer MoS_2 based device has realized a sensitivity up to 4.9%/ppb and LOD of 0.1 ppb,[44] but required intense red LED illumination (60.9 mW cm^{-2}) to enhance the conductivity of the MoS_2 channel and thus the NO_2 sensing response.

2.4. On-Field Gas Sensing

Based on the laboratory sensing measurement results, the p–i–n NW sensor was chosen to confirm its applicability in a realistic environment. A real-time on-field measurement was enabled by connecting our NW device to a portable microprocessor with a USB interface (JLM Innovation with supplementary software JLMlogSP), where the electrical signal generated by the NW sensor is transmitted and processed. A prototype workflow from the on-field sensing measurement to the data analysis and output result is illustrated in **Figure 5**. Prior to on-field operation, a sensor calibration in self-powering mode was demonstrated, as shown in Figure 5a, which correlates the sensing response value with the NO_2 concentration. This calibration was performed in a laboratory gas sensing setup where the injected gas concentration was known and can be precisely controlled (Figure S11, Supporting Information). However, when the NW sensor was connected to the portable USB microprocessor, it could no longer be fitted into the same sensing setup. In this case, the calibration was then performed in a home-made 3D-printing enclosure (with an LED light source) that can accommodate the NW sensor integrated USB system. In this case, the enclosure was not completely sealed and isolated from the ambient atmosphere as in a standard gas sensing chamber. Hence, the measured sensing response tends to overestimate the injected gas concentration (i.e., consider a small amount of gas leakage). The calibration performed with

© 2023 Wiley-VCH GmbH

15214095, 2023, 12, Downloaded from https://advanced.onlinelibrary.wiley.com/doi/10.1002/adma.202207199 by Nanjing Tech University, Wiley Online Library on [31/01/2025]. See the Terms and Conditions (https://onlinelibrary.wiley.com/terms-and-conditions) on Wiley Online Library for rules of use; OA articles are governed by the applicable Creative Commons License

15214095, 2023, 12, Downloaded from https://advanced.onlinelibrary.wiley.com/doi/10.1002/adma.202207199 by Nanjing Tech University, Wiley Online Library on [31/01/2025]. See the Terms and Conditions (https://onlinelibrary.wiley.com/terms-and-conditions) on Wiley Online Library for rules of use; OA articles are governed by the applicable Creative Commons License

Table 1. Comparison of typical sensing performance parameters from self-powered gas sensors reported in the literature (room temperature operation).

Device structure	Light source	Measured signal	Target gas (LOD)	Response 100% (gas concentration)	Response/recovery time [s]	Refs.
p–i–n InP NW	Solar simulator (AM 1.5)	I_{SC} [nA] V_{OC} [mV]	NO_2 (0.49 ppb)	84% (1 ppm)	200/400	This work
p-Si/n-ZnO (amine-/thiol-functionalization)	Solar simulator (AM 1.5)	V_{OC} [mV]	NO_2 (170 ppb)	Amine: 7.5% Thiol: 9.8% (250 ppb)	≈1000	[15]
CNT-SiNW	Solar simulator (AM 1.5)	V_{OC} [V]	NO_2 (10 ppm)	46% (10 ppm)	4–6	[17]
SWNTs/Si	λ = 600 nm, P = 1.8 mW cm^{-2}	V_{OC} [mV]	NO_2 (100 ppb)	2.23% (400 ppb)	≈50	[45]
NiO/ZnO/ITO	Solar simulator 100 mW cm^{-2} AM1.5	V_{OC} [V]	CO_2	0.1%	150/100	[46]
$CsPbBr_3$	Solar simulator (AM 1.5)	I_{SC} [nA]	Acetone (1 ppm)	3% (1 ppm)	10/5	[16]
$CsPbBr_2I$	Solar simulator (AM 1.5)	I_{SC} [nA]	Acetone (1 ppm)	16% (1 ppm)	150	[47]
Gr/WS_2/Gr	White light P = 100 mW cm^{-2}	I_{SC} [µA] or V_{OC} [mV]	NO_2 (5 ppm), H_2 (50 ppm)	NO_2: 70% (5 ppm), H_2: 17% (50 ppm)	≈500	[14]
SiNWs/ITO	Red LED λ = 576 nm, P = 20 mW cm^{-2}	I_{SC} [µA]	NO_2 (5ppb)	90% (1 ppm)	80/850	[41]
Au@rGO/GaN nanorods	UV LED λ = 382 nm, P = 1.71 mW cm^{-2}	I_{SC} [µA]	CO (5 ppm)	38% (20 ppm)	400/1000	[48]
FMCPIB	Solar simulator (AM 1.5)	I_{SC} [nA]	NO_2 (1 ppm)	8% (8 ppm)	17/126	[49]

the portable USB microprocessor locates the boundary of such an overestimation. Therefore, it is expected that a real measurement result from a vehicle exhaust should fall in between the two calibration lines as indicated by the pink shaded area.

The portable NW sensor system was then brought to the realistic environment for on-field measurement with the exposure of a 4-cylinder gasoline car exhaust (Figure 5b). An example time-dependent response curve shows that the value and shape of response pulse vary in different accelerator pedal pressing duration and revolutions per minute (RPM) of the engine, as NO_2 concentration in the exhaust gas depends on these parameters. The measured response curve, together with the calibration results, was transmitted to a data evaluation unit (e.g., a laptop, or an embedded system with USB or Bluetooth interface) for processing. Here we demonstrate a simple pipeline of data acquisition and processing (Figure 5c) for a vehicle exhaust at 2500 RPM where the gas pedal was pressed four times, each of which lasted ≈25 s. The raw data from the measurement was then evaluated with a logistic filter to remove noise (a double-logistic filter in this demonstration) to extract sensing response value.

It is worth mentioning that in addition to NO_2, there are also small amount of carbon monoxide (CO), nitrogen monoxide (NO), sulfur dioxide (SO_2), formaldehyde (HCHO), and benzene (C_6H_6) contained in the car exhaust.[50] However, they may have negligible effects on the NO_2 sensing results due to their weak or nonoxidizing nature. As indicated in Figure 4f, the InP NW sensor only produces a large positive sensing response to the highly oxidizing NO_2 due to its high electron affinity, with negligible responses to CO_2 (weak oxidizing gas) and other reducing gases including acetone, propane, and ethanol. For the aforementioned gases in the car exhaust, CO and other hydrocarbons are reducing gases, while NO and SO_2 have a much smaller electron affinity (NO: 0.025 eV; SO_2 1.10 eV) than that of NO_2 (2.27 eV). Moreover, the effects of those weak or nonoxidizing gases on crystalline InP structure have been investigated previously. Wencai et al. constructed a model of 2D InP monolayer to calculate the electron charge transfer (Q) and absorption energy (E_a) of CO, NO, and NO_2 by ab initio density functional theory (DFT),[51] which found that the Q and E_a of NO_2 (0.588 e, 1.299 eV) on the InP surface are much larger than those of CO and NO (CO: 0.035 e, 0.17 eV; NO: 0.312 e, 0.641 eV). The larger Q and E_a of NO_2 indicate that it has a significantly stronger chemical adsorption and charge transfer ability with InP than those of CO and NO. The experimental studies from Kostryukov et al.[52] and Garcia et al.[53] also demonstrated that InP thin film has a much smaller sensing response to CO and NO than to NO_2. Furthermore, according to the study from Christian et al.,[54] and Parthasarathi et al.,[55] the concentration of hydrocarbons and SO_2 in vehicle exhaust is much smaller than that of NO_2 (NO_2 is ≈0.4 g km^{-1}; hydrocarbons in total is ≈0.1 g km^{-1}; SO_2 only shows possible trace from vehicle emission). Therefore, it is reasonable to conclude that the presence of a mixture of other interfering gases has a negligible impact on the NO_2 monitoring results in our on-field vehicle exhaust measurements.

The calculated NO_2 concentration from this p–i–n NW sensor on-field measurement is ≈15.2–16.6 ppm, which is in good agreement with the result measured from a commercial NO_2 air quality sensor (≈8.9–13.0 ppm, Figure S12, Supporting Information), as well as previously reported studies (10.0–50.0 ppm from optical fiber sensors[56] and 15.0–16.9 ppm from chemiluminescence[57]), indicating the practical functionality and accuracy of our self-powered NW sensor system.

To improve the device performance and functionalities, the p–i–n structure can be further optimized by reducing n-segment length and increasing i-segment length to enhance its sensitivity. Apart from the axial p–n junction, it may be worth

 © 2023 Wiley-VCH GmbH

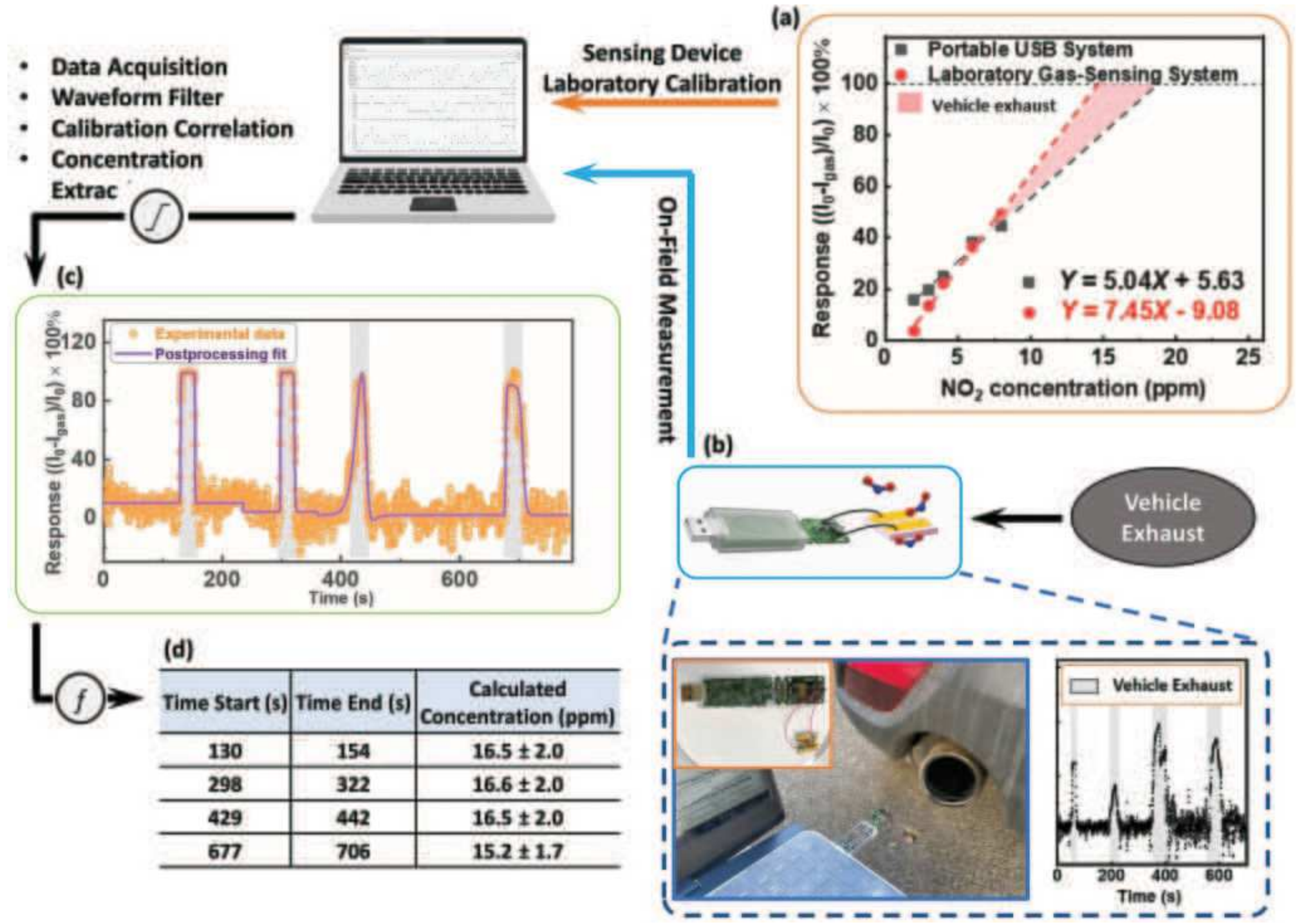

Figure 5. A prototype workflow for practical on-field gas sensing measurement. a) The NW sensor concentration calibration in a laboratory environment (portable USB microprocessor system and laboratory gas sensing system). b) The schematic and on-site photos of the on-field vehicle exhaust measurement based on the NW sensor integrated with the USB interface and an example of acquired data. c) Data measured by this USB interface and the corresponding post-processed fitting result by user-specified data filters to remove noise and extract sensing response values. d) By correlating with the laboratory calibration, the NO_2 concentration can be dynamically monitored.

investigating a radial-junction p–n NW structure, which could provide a larger junction region and simplify the device fabrication process. Furthermore, multiple NW arrays with varied pitch sizes and diameters can be grown during the same epitaxial growth sequence to provide multichannel detection, which could further enhance the dynamic range of the NO_2 detection.

3. Conclusion

A novel self-powered sensor architecture was successfully designed and demonstrated based on bottom-up grown p–n homojunction InP NW arrays. We designed the p–n-junction-based NW sensor structure and explored the sensing mechanism via numerical simulation. The fabricated sensors based on the p–i–n junction InP NW array show excellent sensitivity with a limit of detection down to the sub-ppb level and a high selectivity to NO_2 in self-powered operation mode. These results promise a new family of self-powered sensing platforms to achieve high-performance gas sensors with a strong environmental fidelity enabled by the PV effect. The integration of the NW sensor with a portable USB interface for practical dynamic on-field measurement is further demonstrated, precisely quantifying NO_2 concentration from a motor vehicle exhaust. Our prototype InP PV NW sensor design paves the way toward a battery-free, highly integrable and compatible sensing platform for applications such as environmental monitoring, industrial safety and hazard alarming. It is a promising step forward to creating a next-generation sensing network for the Internet of Things.

4. Experimental Section

Numerical Simulation: The gas sensing simulation was performed using the semiconductor module of COMSOL Multiphysics. To ensure timely convergence of simulated processes, only a 2D equivalent unit cell of nanowire array device, consisting of a single NW, was constructed in simulation. The 3D view, together with the 2D simulation geometry of the unit cell is illustrated in Figure S1 in the Supporting Information. The exposure window is fixed at 700 nm measured from the top of NWs, while the junction depth is varied from 350 to 1800 nm. The left surface

© 2023 Wiley-VCH GmbH

15214095, 2023, 12, Downloaded from https://advanced.onlinelibrary.wiley.com/doi/10.1002/adma.202207199 by Nanjing Tech University, Wiley Online Library on [31/01/2025]. See the Terms and Conditions (https://onlinelibrary.wiley.com/terms-and-conditions) on Wiley Online Library for rules of use; OA articles are governed by the applicable Creative Commons License

within this window is subject to gas exposure, for which a negative surface charge boundary condition is applied to model oxidative NO_2 adsorption. The light generation is only enabled within the window, below which is considered negligible since light is largely blocked by metal contact (Figure 1). The top horizontal boundary, together with right side boundary (300 nm from the top), is set as Ohmic contact to replicate realistic device structure. All simulation related parameters are summarized in Table S1 in the Supporting Information.

p–i–n/n–i–p Junction InP NW Growth: 1) Substrate processing: Figure S2 (Supporting Information) illustrates the processes of preparing InP (111)A substrate (both p-doped and n-doped with a concentration of (0.8–8) × 10^{18} cm^{-3}) for NW growth. First, a 30 nm SiO_2 layer was deposited on (111)A InP substrate by plasma enhanced chemical vapor deposition (PECVD) at 300 °C, and the thickness was measured by ellipsometry. A negative photoresist AR6200.09 was spin-coated (step 1: 500 rpm 5 s; step 2: 2000 rpm for 60 s) on SiO_2 layer and baked at 150 °C for 1 min on hotplate. Then, the resist was exposed by Raith 150 electron beam lithography (EBL) system based on a designed pattern consisting of 200 × 200 μm^2 hexagonal dot array with a size of 80 nm and pitch of 600 nm. The exposed pattern area in photoresist layer was removed by the corresponding resist developer. After the development of resist, oxygen plasma (RF power: 300 W; 300 sccm O_2 flow; 2 min,) was used to remove the footage residues in the patterned area. The exposed pattern was then etched by RIE to remove the SiO_2 layer on InP substrate (RF power: 20 W; 20 sccm CHF_3 flow; 4.5 min). To remove the possible damage on the exposed InP surface during SiO_2 deposition, 10% H_2O_2 was first used to oxidize the InP layer for 2 min followed by etching off the oxide layer with 10% H_3PO_4 solution for 2 min. These steps were repeated sequentially for 5 times and then, the sample was immediately transferred into the MOVPE reactor for NW growth.

(2) p–i–n/n-i-p Junction InP NW Growth: The InP NW arrays were grown in AIXTRON 200/4 MOVPE reactor, operating at a base pressure of 100 mbar, using H_2 as a carrier gas with a total flow of 14.5 l min^{-1}. Trimethylindium (TMIn) and phosphine (PH_3) were used as precursors for the group III (In) and group V (P) elements, respectively. Molar fractions of TMIn and PH_3 were set at 9.38 × 10^{-6} and 7.59 × 10^{-4} mol min^{-1}, respectively, corresponding to a V/III ratio of 80. All samples were hot baked at 750 °C for 10 min under the PH_3 flow and grown for 4 min at 730 °C for undoped segment (i-InP). Then, Silane (SiH_4) and diethylzinc (DEZn) were introduced during the growth of n-doped segment and p-doped segment for 2 min with all the other parameters kept the same as those used for the growth of undoped InP segment. With this two-step growth process, the i–n and i–p InP NW were grown on p-doped and n-doped substrate respectively, to form n-i-p and p–i–n junctions.

InP NW characterization: The morphology of the as-grown InP NW arrays were characterized by scanning electron microscopy (SEM, FEI VERIOS 460) with electron beam voltage of 2 kV and current of 13 pA. The crystal structure is determined by transmission electron microscopy (TEM, JEOL 2100F) analysis of NWs that were directly transferred from the original array to a copper mesh. Cathodoluminescence (CL) technique was applied on single NWs which were transferred from the array to a Si substrate to characterize its optical property. The CL images were acquired from SEM equipped with a Gatan MONO CL4 components under an electron excitation voltage of 10 kV and current of 0.8 nA at room temperature. The NWs were also transferred to a SiO_2/Si substrate for PL and TRPL at room temperature. The PL system is composed of Horiba LabRAM system equipped with confocal optics, a diode pumped solid-state (DPSS) 532 nm laser, and a liquid-nitrogen-cooled array InGaAs detector. The laser beam was focused through a 100x microscope objective lens, resulting in a spot size of 0.36 μm (in radius) estimated by vector diffraction calculation. For TRPL, the emission was collected by the same objective lens and detected by a single photon avalanche diode, which was connected to the Picoharp 300 time-correlated single photon counting (TCSPC) system. PL spectra were acquired along single NW from top to bottom position.[3] The minority carrier lifetime in the NWs was extracted from the single exponential fitting of TRPL decay curve.

Fabrication of Self-Powered InP NW NO_2 Sensor: To fabricate a chemiresistive sensor based on InP axial junction NW array, photoresist SU8-5 (Kirsten Hackenbroich) was spin-coated on the NW arrays to fully cover the entire NW arrays. Then SU8-5 was etched back by barrel-etcher (PVA Tepla Gigabatch 310M) with O_2 flow rate of 300 sccm and power of 500 W to expose ≈500 nm of the top of InP NWs for subsequent electrical contact. Then the sample was flood exposed under UV illumination and baked at 200 °C to solidify SU8-5. Since the top junction is deeper than 500 nm, the i-segment of NW is embedded in SU8-5, which prevents shorting of the junction and provides necessary mechanical support to the NWs. A 200 nm Au layer was deposited on nanowires using e-beam evaporator for top contact. The samples were mounted on a special holder to enable the tile-angle deposition to provide not only electrical contact, but also to partially expose the nanowires to allow gas adsorption and sensing.[28] The tilt-angle deposition leaves a shadow area behind each NW covered by SU8-5, which can be further etched to extend the exposure window below the top junction. The etching rate of inductively coupled plasma (ICP) etching has been well calibrated such that another ≈500 nm of SU8-5 was removed precisely as shown in Figure S5g (Supporting Information). The SU8-5 layer shadowed by NW during deposition was exposed and subsequently etched by ICP-F, which formed a hole besides each NW. Then, a 200 nm Au layer was deposited on the back of InP substrate as bottom contact.

Electrical Characterization: The electrical and photovoltaic properties of InP nanowire array were characterized by current–voltage (*I–V*) measurements by Keysight system B2902A source/measurement unit, incorporated with a solar simulator.

NO_2 Sensing Measurement: The gas sensing performance was measured by home-made sensing setup which consists of a Linkam chamber with a sample stage and Au probes for electrical contact, mass flow controllers (MFCs Bronkhorst), a solar simulator and gas cylinders. For the gas sensing measurements, the carrier gas was simulated air with volume ratio of N_2 to O_2 at 4 ($V_{N2}/V_{O2} = 4$, N_2 and O_2, BOC gas). The gas flow rate was controlled by MFCs while the total gas flow rate was kept at 1 L min^{-1} for ppm level concentration test and 0.5 l min^{-1} for sub-ppm level concentration test. For the measurement of analyte gas, volatile organic compounds (VOCs) (ethanol, 9.91 ppm in N_2, Coregas; acetone, 10.1 ppm in N_2, Coregas; methanol, 10 ppm in N_2, BOC gas) or NO_2 gas was diluted to desired concentration before being purged into the chamber.

Statistical Analysis: For PL and TRPL, more than 5 single NWs from p–i–n and n–i–p arrays were measured, respectively. The lifetime data were analyzed by MATLAB. Dark/light *I–V* characteristics of the p–i–n and n–i–p InP NW arrays in Figure 3 were obtained from more than 3 samples which were tested under the same dark condition and light intensity. The sensing response of p–i–n sample in Figure 4b was tested more than 10 times of sensing measurement cycles and are given in the form of mean ± standard deviation by Microsoft Excel. The data in Figure 4c were analyzed using the linear fitting function of Origin 2018 (OriginLab Co.) to obtain the slope of concentration dependent sensing response curve, which gives a measure of sensitivity. The fitted linear slope is $Y = 0.62X + 1.36$ ($r^2 = 0.98$) for p–i–n sample and $Y = -0.014X + 1.98$ ($r^2 = 0.95$) for n–i–p sample, both with 95% confidence level. For the on-field vehicle exhaust data, more than 5 times of pedaling were measured by the p–i–n InP NW sensor and commercial NO_2 air quality sensor.

Supporting Information

Supporting Information is available from the Wiley Online Library or from the author.

Acknowledgements

The authors acknowledge the financial support from the Australian Research Council. The authors also acknowledge the Australian

 © 2023 Wiley-VCH GmbH

15214095, 2023, 12, Downloaded from https://advanced.onlinelibrary.wiley.com/doi/10.1002/adma.202207199 by Nanjing Tech University, Wiley Online Library on [31/01/2025]. See the Terms and Conditions (https://onlinelibrary.wiley.com/terms-and-conditions) on Wiley Online Library for rules of use; OA articles are governed by the applicable Creative Commons License

National Fabrication Facility (ACT node) for facility support. This research was also funded by and has been delivered in partnership with Our Health in Our Hands (OHIOH), a strategic initiative of the Australian National University, which aims at transforming healthcare by developing new personalized health technologies and solutions in collaboration with patients, clinicians, and healthcare providers. A.T. gratefully acknowledges the support of the Australian Research Council for a Future Fellowship (No. FT200100939) and Discovery Grant No. DP190101864. A.T. also acknowledges financial support from the North Atlantic Treaty Organization Science for Peace and Security Programme project AMOXES (No. G5634). S.W. thanks the China Scholarship Council and the Australian National University for scholarship support. The authors also thank Dr. Mary Gray for proofreading/editing the manuscript.

Conflict of Interest

The authors declare no conflict of interest.

Data Availability Statement

The data that support the findings of this study are available from the corresponding author upon reasonable request.

Keywords

gas sensors, InP nanowires, p–n homojunction, self-powered devices

Received: August 8, 2022
Revised: November 29, 2022
Published online: January 1, 2023

[1] Z. Y. Qian, S. H. Kang, V. Rajaram, C. Cassella, N. E. McGruer, M. Rinaldi, *Nat. Nanotechnol.* **2017**, *12*, 969.
[2] M. Shirvanimoghaddam, K. Shirvanimoghaddam, M. M. Abolhasani, M. Farhangi, V. Z. Barsari, H. Y. Liu, M. Dohler, M. Naebe, *IEEE Access* **2019**, *7*, 94533.
[3] X. Li, C. Q. Luo, H. Ji, Y. D. Zhuang, H. L. Zhang, V. C. M. Leung, *Int. J. Commun. Syst.* **2020**, *33*, 4171.
[4] A. Ahmed, I. Hassan, M. F. El-Kady, A. Radhi, C. K. Jeong, P. R. Selvaganapathy, J. Zu, S. Ren, Q. Wang, R. B. Kaner, *Adv. Sci.* **2019**, *6*, 1802230.
[5] V. A. Aksyuk, *Nat. Nanotechnol.* **2017**, *12*, 940.
[6] H. Askari, E. Hashemi, A. Khajepour, M. B. Khamesee, Z. L. Wang, *Nano Energy* **2018**, *53*, 1003.
[7] Z. Y. Wu, T. H. Cheng, Z. L. Wang, *Sensors* **2020**, *20*, 2925.
[8] Z. L. Song, W. H. Ye, Z. Chen, Z. S. Chen, M. T. Li, W. Y. Tang, C. Wang, Z. A. Wan, S. Poddar, X. L. Wen, X. F. Pan, Y. J. Lin, Q. F. Zhou, Z. Y. Fan, *ACS Nano* **2021**, *15*, 7659.
[9] E. Lee, H. Yoo, *Molecules* **2021**, *26*, 1802230.
[10] Z. Wen, Q. Q. Shen, X. H. Sun, *Nanomicro Lett.* **2017**, *9*, 45.
[11] X. L. Liu, Y. Zhao, W. J. Wang, S. X. Ma, X. J. Ning, L. Zhao, J. Zhuang, *IEEE Sens. J.* **2021**, *21*, 5628.
[12] P. A. Laplante, M. Kassab, N. L. Laplante, J. M. Voas, *IEEE Intell. Syst.* **2018**, *12*, 3030.
[13] Y. Kim, S. Lee, J.-G. Song, K. Y. Ko, W. J. Woo, S. W. Lee, M. Park, H. Lee, Z. Lee, H. Choi, W.-H. Kim, J. Park, H. Kim, *Adv. Funct. Mater.* **2020**, *30*, 2003360.
[14] D. Lee, H. Park, S. D. Han, S. H. Kim, W. Huh, J. Y. Lee, Y. S. Kim, M. J. Park, W. I. Park, C.-Y. Kang, C.-H. Lee, *Small* **2019**, *15*, 1804303.
[15] M. W. G. Hoffmann, L. Mayrhofer, O. Casals, L. Caccamo, F. Hernandez-Ramirez, G. Lilienkamp, W. Daum, M. Moseler, A. Waag, H. Shen, J. D. Prades, *Adv. Mater.* **2014**, *26*, 8017.
[16] H. Chen, M. Zhang, R. Bo, C. Barugkin, J. Zheng, Q. Ma, S. Huang, A. W. Y. Ho-Baillie, K. R. Catchpole, A. Tricoli, *Small* **2018**, *14*, 1702571.
[17] Y. Jia, Z. Zhang, L. Xiao, R. Lv, *Nanoscale Res. Lett.* **2016**, *11*, 299.
[18] X. L. Liu, S. X. Ma, S. W. Zhu, Y. Zhao, X. J. Ning, L. Zhao, J. Zhuang, *Sens. Actuators, B* **2019**, *291*, 345.
[19] X. L. Liu, Y. Zhao, S. X. Ma, S. W. Zhu, X. J. Ning, L. Zhao, J. Zhuang, *ACS Sens.* **2019**, *4*, 3056.
[20] M. W. G. Hoffmann, O. Casals, A. E. Gad, L. Mayrhofer, C. Fabrega, L. Caccamo, F. Hernandez-Ramirez, G. Lilienkamp, W. Daum, M. Moseler, H. Shen, A. Waag, J. D. Prades, *Procedia Eng.* **2015**, *120*, 623.
[21] E. Modaresinezhad, S. Darbari, *Sens. Actuators, B* **2016**, *237*, 358.
[22] X.-L. Liu, Y. Zhao, S.-X. Ma, S.-W. Zhu, X.-J. Ning, L. Zhao, J. Zhuang, *ACS Sens.* **2019**, *4*, 3056.
[23] M. A. Green, E. D. Dunlop, J. Hohl-Ebinger, M. Yoshita, N. Kopidakis, K. Bothe, D. Hinken, M. Rauer, X. Hao, *Prog. Photovolt.: Res. Appl.* **2022**, *30*, 687.
[24] Z. Y. Li, H. H. Tan, C. Jagadish, L. Fu, *Adv. Mater. Technol.* **2018**, *3*, 1800005.
[25] M. C. Beard, J. M. Luther, A. J. Nozik, *Nat. Nanotechnol.* **2014**, *9*, 951.
[26] D. van Dam, N. J. J. van Hoof, Y. Cui, P. J. van Veldhoven, E. P. A. M. Bakkers, J. Gómez Rivas, J. E. M. Haverkort, *ACS Nano* **2016**, *10*, 11414.
[27] J. Wallentin, N. Anttu, D. Asoli, M. Huffman, I. Åberg, M. H. Magnusson, G. Siefer, P. Fuss-Kailuweit, F. Dimroth, B. Witzigmann, H. Q. Xu, L. Samuelson, K. Deppert, M. T. Borgström, *Science* **2013**, *339*, 1057.
[28] S. Wei, Z. Li, A. John, B. I. Karawdeniya, Z. Li, F. Zhang, K. Vora, H. H. Tan, C. Jagadish, K. Murugappan, A. Tricoli, L. Fu, *Adv. Funct. Mater.* **2022**, *32*, 2107596.
[29] M. F. Schneidereit, F. Scholz, F. Huber, H. Schieferdecker, K. Thonke, N. Naskar, T. Weil, A. Pasquarelli, *Sens. Actuators, B* **2020**, *305*, 127189.
[30] J. Du, D. Liang, H. Tang, X. P. A. Gao, *Nano Lett.* **2009**, *9*, 4348.
[31] M. Bai, H. Huang, Z. Liu, T. T. Zhan, S. F. Xia, X. G. Li, N. Sibirev, A. Bouravleuv, V. G. Dubrovskii, G. Cirlin, *Appl. Surf. Sci.* **2019**, *498*, 143756.
[32] Z. Y. Li, I. Yang, L. Li, Q. Gao, J. S. Chong, Z. Li, M. N. Lockrey, H. H. Tan, C. Jagadish, L. Fu, *Prog. Nat. Sci.: Mater. Int.* **2018**, *28*, 178.
[33] Q. Gao, Z. Li, L. Li, K. Vora, Z. Li, A. Alabadla, F. Wang, Y. Guo, K. Peng, Y. C. Wenas, S. Mokkapati, F. Karouta, H. H. Tan, C. Jagadish, L. Fu, *Prog. Photovolt.: Res. Appl.* **2019**, *27*, 237.
[34] Q. Gao, D. Saxena, F. Wang, L. Fu, S. Mokkapati, Y. A. Guo, L. Li, J. Wong-Leung, P. Caroff, H. H. Tan, C. Jagadish, *Nano Lett.* **2014**, *14*, 5206.
[35] Y. Kim, S. Lee, J. G. Song, K. Y. Ko, W. J. Woo, S. W. Lee, M. Park, H. Lee, Z. Lee, H. Choi, W. H. Kim, J. Park, H. Kim, *Adv. Funct. Mater.* **2020**, *30*, 2003360.
[36] I. Yang, X. Zhang, C. L. Zheng, Q. Gao, Z. Y. Li, L. Li, M. N. Lockrey, H. Nguyen, P. Caroff, J. Etheridge, H. H. Tan, C. Jagadish, J. Wong-Leung, L. Fu, *ACS Nano* **2018**, *12*, 10374.
[37] X. Zhou, J. Y. Liu, C. Wang, P. Sun, X. L. Hu, X. W. Li, K. Shimanoe, N. Yamazoe, G. Y. Lu, *Sens. Actuators, B* **2015**, *206*, 577.
[38] T. Pham, G. Li, E. Bekyarova, M. E. Itkis, A. Mulchandani, *ACS Nano* **2019**, *13*, 3196.
[39] K. D. Wu, M. Debliquy, C. Zhang, *Chem. Eng. J.* **2022**, *444*, 136449.
[40] H. Long, A. Harley-Trochimczyk, T. Pham, Z. R. Tang, T. L. Shi, A. Zettl, C. Carraro, M. A. Worsley, R. Maboudian, *Adv. Funct. Mater.* **2016**, *26*, 5158.
[41] J. K. Choi, I. S. Hwang, S. J. Kim, J. S. Park, S. S. Park, U. Jeong, Y. C. Kang, J. H. Lee, *Sens. Actuators, B* **2010**, *150*, 191.

© 2023 Wiley-VCH GmbH

15214095, 2023, 12, Downloaded from https://advanced.onlinelibrary.wiley.com/doi/10.1002/adma.202207199 by Nanjing Tech University, Wiley Online Library on [31/01/2025]. See the Terms and Conditions (https://onlinelibrary.wiley.com/terms-and-conditions) on Wiley Online Library for rules of use; OA articles are governed by the applicable Creative Commons License

[42] K. Y. Dong, J. K. Choi, I. S. Hwang, J. W. Lee, B. H. Kang, D. J. Ham, J. H. Lee, B. K. Ju, *Sens. Actuators, B* **2011**, *157*, 154.
[43] X. Z. Wang, D. Ansari, *J. En. Chem. Eng.* **2022**, *10*, 107786.
[44] T. Pham, G. Li, E. Bekyarova, M. E. Itkis, A. Mulchandani, *ACS Nano* **2019**, *13*, 3196.
[45] D. Liu, Q. Chen, A. Chen, J. Wu, *RSC Adv.* **2019**, *9*, 23554.
[46] L. Liu, G. H. Li, Y. Wang, Y. Y. Wang, T. Li, T. Zhang, S. J. Qin, *Nanoscale* **2017**, *9*, 18579.
[47] T. Hamada, M. Sugiyama, *Jpn. J. Appl. Phys.* **2022**, *61*, 054002.
[48] H. Chen, M. Zhang, X. Fu, Z. Fusco, R. Bo, B. Xing, H. T. Nguyen, C. Barugkin, J. Zheng, C. F. J. Lau, S. Huang, A. W. Y. Ho-Baillie, K. R. Catchpole, A. Tricoli, *Phys. Chem. Chem. Phys.* **2019**, *21*, 24187.
[49] M. Reddeppa, S. B. Mitta, T. Chandrakalavathi, B. G. Park, G. Murali, R. Jeyalakshmi, S. G. Kim, S. H. Park, M. D. Kim, *Curr. Appl. Phys.* **2019**, *19*, 938.
[50] H. Chen, M. Zhang, B. Xing, X. Fu, R. Bo, H. K. Mulmudi, S. Huang, A. W. Y. Ho-Baillie, K. R. Catchpole, A. Tricoli, *Adv. Opt. Mater.* **2020**, *8*, 1901863.
[51] W. C. Yi, X. Chen, Z. X. Wang, Y. C. Ding, B. C. Yang, X. B. Liu, *J. Mater. Chem.* **2019**, *7*, 7352.
[52] V. F. Kostryukov, I. Y. Mittova, S. Ali, *Inorg. Mater.* **2020**, *56*, 66.
[53] M. A. Garcia, M. Losurdo, S. D. Wolter, W. V. Lampert, J. Bonaventura, G. Bruno, C. Yi, A. S. Brown, *Sens. Lett.* **2008**, *6*, 627.
[54] C. Weber, I. Sundvor, E. Figenbaum, *Atmos. Environ.* **2019**, *206*, 208.
[55] P. Bera, M. Hegde, *J. Indian Inst. Sci.* **2010**, *90*, 299.
[56] G. Dooly, E. Lewis, C. Fitzpatrick, P. Chambers, *IEEE Sens. J.* **2007**, *7*, 685.
[57] S. Karthikeyan, E. M. Thomson, P. Kumarathasan, J. Guénette, D. Rosenblatt, T. Chan, G. Rideout, R. Vincent, *Toxicol. Sci.* **2013**, *135*, 437.

© 2023 Wiley-VCH GmbH

15214095, 2023, 12, Downloaded from https://advanced.onlinelibrary.wiley.com/doi/10.1002/adma.202207199 by Nanjing Tech University, Wiley Online Library on [31/01/2025]. See the Terms and Conditions (https://onlinelibrary.wiley.com/terms-and-conditions) on Wiley Online Library for rules of use; OA articles are governed by the applicable Creative Commons License

analytical chemistry

pubs.acs.org/ac Article

Optoelectronic Gas Sensor Based on Few-Layered InSe Nanosheets for NO_2 Detection with Ultrahigh Antihumidity Ability

Lu Zhang,[▽] Zhongjun Li,[▽] Jiang Liu, Zhengchun Peng, Jia Zhou,* Han Zhang,* and Yingchun Li*

Cite This: *Anal. Chem.* 2020, 92, 11277−11287 Read Online

ACCESS | Metrics & More | Article Recommendations | SI Supporting Information

ABSTRACT: Two-dimensional (2D) transition-metal/metal chalcogenides including MoS_2, $MoSe_2$, WS_2, SnS_2, etc. have shown considerable potential for the fabrication of gas sensors for NO_2 detection. However, these sensors usually suffer from sluggish and incomplete recovery at room temperature, and their sensitivities are limited by presorbed O_2. In this work, a novel optoelectronic gas sensor based on direct-bandgap InSe nanosheets was demonstrated. Because of the excellent photoelectric and sensing properties in few-layer InSe, detection of NO_2 at room temperature was realized. Ultrahigh and reversible responses were obtained under ultraviolet (UV) light illumination, and the limit of detection (0.98 ppb) was ~40 times lower than that observed without UV light. Furthermore, the effects of O_2 and H_2O molecules on sensor performance were fully studied through experiments and density functional theory. Some new mechanisms of NO_2 detection in high relative humidity conditions under UV illumination were proposed, including regulation of proton transfer and induction of H_2O_2 reduction. In all, this work not only broadens the application field of 2D InSe, but also demonstrates the potential prospect of detecting ppb-level NO_2 in complex circumstances such as human breath by using 2D material-based sensors with light activation.

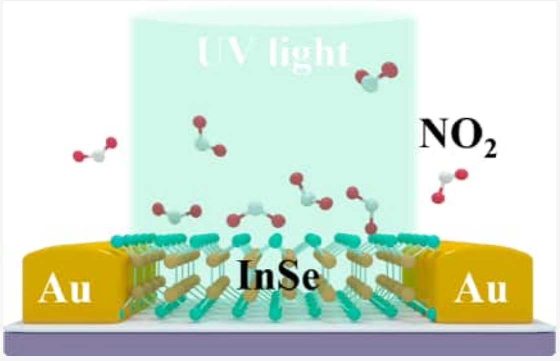

Downloaded via NANJING TECH UNIV on February 1, 2025 at 06:19:45 (UTC).
See https://pubs.acs.org/sharingguidelines for options on how to legitimately share published articles.

Nitrogen dioxide (NO_2) is one of the atmospheric pollutants released from fuel combustion and automotive engines, which can cause numerous environmental issues, such as dense fog and acid rain.[1] In addition, long-term exposure to only trace (ppb) levels of NO_2 gas can have adverse effects on human respiratory system and skin.[2,3] The concentration of NO_2, furthermore, can reflect the level of nitrogen oxides (NO_x, a species of NO, NO_2, etc.) in exhaled breath, where NO_x can be considered as a biomarker to diagnose diseases such as nasal polyposis, chronic obstructive pulmonary disease, etc.[4,5] Therefore, detection of NO_2 gas is of the great importance for protecting the environment and human beings, as well as assessing health status.

Chemiresistive gas sensors are one of the most common and effective instruments for detecting trace gas molecules.[6−8] In recent years, two-dimensional (2D) materials are gaining great attention for use in designing gas sensors, because of their excellent semiconductor properties and high surface-area-to-volume ratio.[9] Compared with conventional gas sensors based on metal oxides, 2D-material-based gas sensors can fully interact with target gas and can work in the absence of oxygen ions, resulting in decent gas-sensing performance, even at room temperature (RT).[10] 2D transition-metal dichalcogenides (TMDs) and metal chalcogenides (MCs), such as MoS_2,[11] WS_2,[12] and SnS_2,[13] have recently been verified as ideal candidates for sensing NO_2 by virtue of their high binding energy and large charge transfer number for adsorbing NO_2 molecules. Unfortunately, these sensors usually display a lengthy response to NO_2 and incomplete recovery at RT.[14] Besides, sensing behavior could be impaired by the preadsorbed oxygen species that are dwelling on the surface of sensing materials and occupying active sites.[15] For this, light irradiation has been used as an effective tactic that not only shortens response/recovery time, but also helps to release oxygen species. However, a prerequisite of this method is that the sensing materials should possess good photoelectric properties.[16,17]

Indium selenide (InSe), as a nova in the class of 2D MCs, is a van der Waals layered material with an electron effective mass of ~0.143 m_0, and band gaps of 1.26 eV for the bulk and 2.11 eV for the single-layer at RT.[18,19] InSe has been widely used to fabricate optoelectronic devices, because of their high on/off ratios (~10^8),[20] high carrier mobility (~1000 cm^2 V^{-1} s^{-1} at RT),[21] good stability in air (compared with phosphene), and excellent photoresponsivity with broad spectral range from UV to near-infrared.[21,22] In addition, density functional theory

Received: May 5, 2020
Accepted: July 20, 2020
Published: July 20, 2020

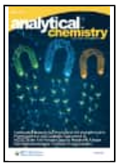

© 2020 American Chemical Society

https://dx.doi.org/10.1021/acs.analchem.0c01941
Anal. Chem. 2020, 92, 11277−11287

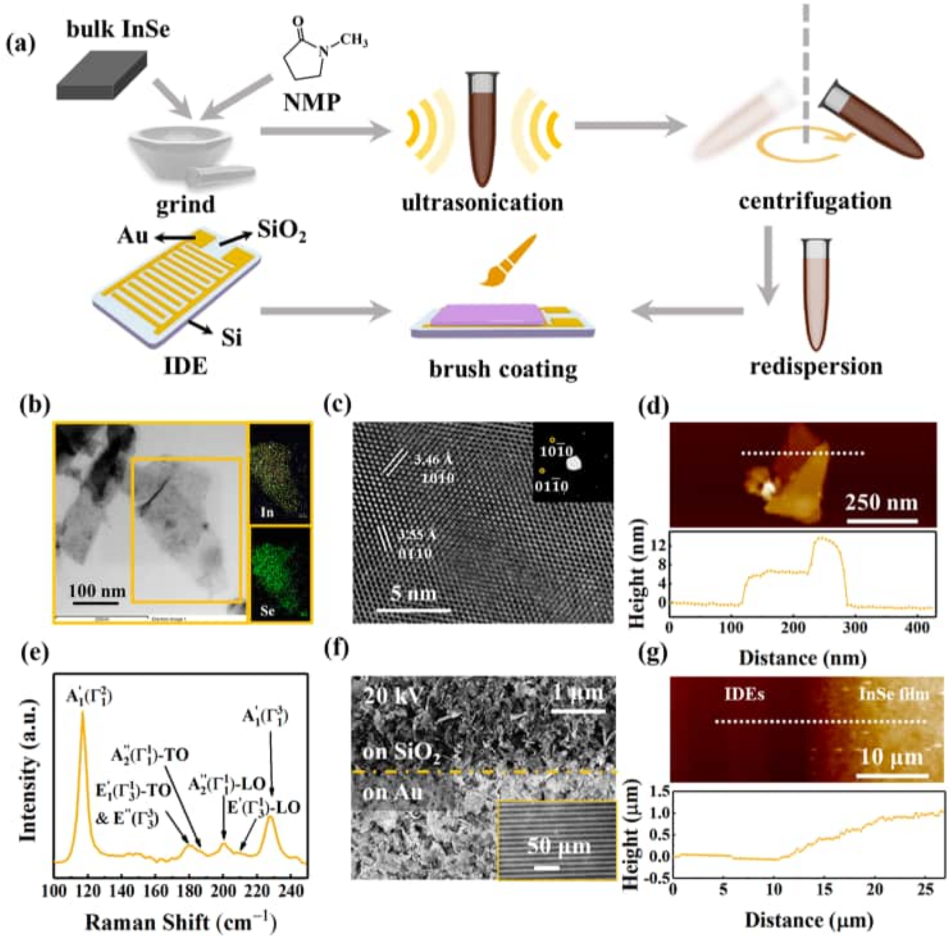

Figure 1. (a) Preparation of InSe by LPE method and fabrication of InSe/IDEs. (b) TEM and EDS mapping of prepared InSe nanosheets. (c) High-resolution transmission electron microscopy (HRTEM) image of the InSe (inset shows the selected-area electron diffraction (SAED) pattern). (d) Atomic force microscopy (AFM) of representative InSe nanosheets and height profile of the indicated section (white dashed lines). (e) Raman spectra for InSe samples on IDEs. (f) SEM image of the surface of InSe/IDEs; the inset shows an SEM image of enlarged view with SiO_2 and Au stripes. (g) AFM of the edge of InSe film on IDEs and height profile of the indicated section (white dashed lines).

(DFT) calculations have already noted that 2D InSe has a superior gas-sensing ability for detecting open-shell molecules (NO and NO_2) based on the charge-transfer mechanism.[23,24] In addition, few-layer InSe is endowed with a direct bandgap characteristic when there are more than six layers present, which is beneficial to light absorption and thus enhances the photoelectric properties.[25] The excellent photoelectric performance of randomly stacked few-layer InSe nanosheets has been confirmed in our previous work.[26] Compared with TMDs that exhibit direct-bandgap properties only in a single layer, fewer-layer InSe nanosheets are easier to prepare and have a greater possibility of mass production by using the liquid-phase exfoliation (LPE) method.[27] Besides, according to the literature, the photoelectric and gas-sensing properties of randomly stacked 2D-material nanosheets are comparable to those of their single-crystal counterpart.[28,29] Altogether, it can be concluded that few-layer InSe nanosheets has great promise to build a photoelectric gas sensor for determining NO_2.

In this work, an optoelectronic gas sensor based on few-layered InSe nanosheets was obtained, where InSe nanosheets were prepared via the LPE method. Besides, one-chip InSe devices were also fabricated by micromechanical exfoliation method for comparison. The prepared InSe nanosheets had high crystallization with favorable thickness. The adsorption behaviors of O_2, H_2O, and NO_2 on the 2D InSe monolayer was analyzed by DFT calculation. The excellent gas-sensing performance of InSe-based sensor toward ppb-levels NO_2 under UV illumination has been proven by abundant experiments. In addition, the feasibility to detect NO_2 in human breath samples by using the InSe-based sensor was verified, and a possible sensing mechanism and the effect of UV illumination were deeply discussed.

■ EXPERIMENTAL SECTION

Reagents and Instruments. Bulk InSe (99.9999%) was obtained from 2D Semiconductors, Inc. (Scottsdale, AZ, USA). *N*-Methyl-2-pyrrolidone (NMP, 99.5%) was purchased from Shanghai Aladdin Co. Ltd. (Shanghai, China). Methanol, ethanol, and acetone were purchased from Titan Scientific Co., Ltd. (Shanghai, China). NO_2, H_2S, and NH_3 at 1000 ppm were obtained from Dalian Haide Tech. Co., Ltd. (Dalian, China). Synthetic air as well as pure N_2 were obtained by Guangzhou Xiangyuan Gas Co., Ltd. (Guangzhou, China). All chemicals were of analytical grade and used without further purification.

Interdigital electrodes (IDEs) were obtained from Huizhou Xinwenxiong Trade Co., Ltd. (Huizhou, China). It has a monocrystalline Si substrate with 300 nm SiO_2 on the top, and Cr/Au (10 nm/100 nm) electrodes were deposited on the

https://dx.doi.org/10.1021/acs.analchem.0c01941
Anal. Chem. 2020, 92, 11277−11287

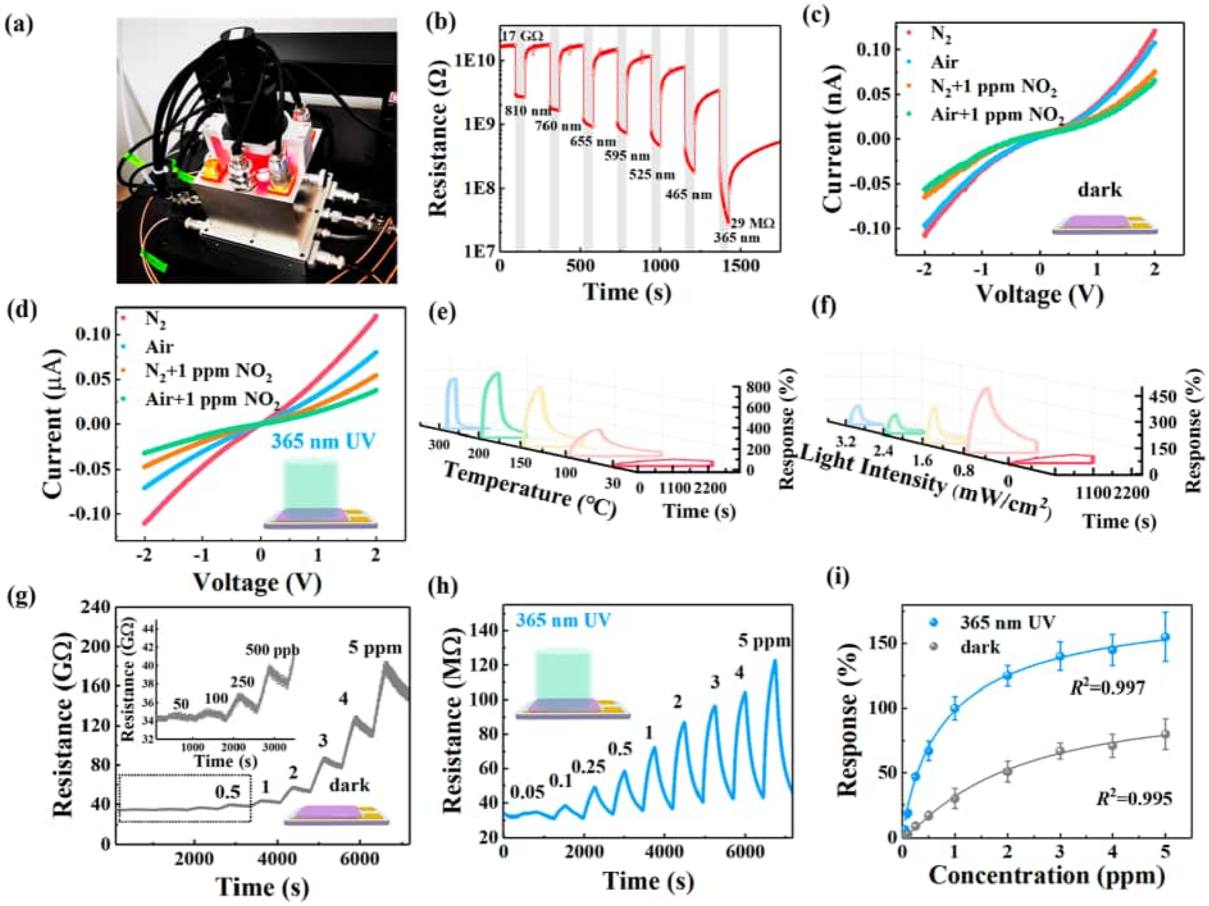

Figure 2. (a) Photograph of the gas chamber used for testing sensing performance. (b) Dynamic response curve of InSe/IDEs to irradiation of different wavelengths. *I*−*V* curves of InSe/IDEs under different gas atmospheres (c) in darkness conditions and (d) under 365-nm light. Response curves of InSe/IDEs to 1 ppm of NO_2 gas (e) at different temperatures in a dark environment and (f) under different light intensities of 365-nm UV light. Dynamic response curves of InSe/IDE for 0.05−5 ppm of NO_2 gas (g) in a dark environment and (h) under 365-nm UV light (the inset shows the amplified curve at low concentrations). (i) Response intensity curves of InSe/IDEs for 0.05−5 ppm of NO_2 (n = 3). All operating temperatures are 30 °C, with a total gas flow rate of 1000 sccm; all background gases are air, except the test of *I*−*V* curves, and all light intensities are 3.2 mW/cm^2, unless noted otherwise.

surface of SiO_2. In this experiment, the width of the Au strip and strip space is 5 μm for loading InSe-nanosheets film, and 30 μm electrode strips with 20 μm strip space were designed to fabricate the one-chip InSe device.

The morphology was characterized by using a scanning electron microscopy (SEM) system (Zeiss, Model SIGMA) that was operating at 20 kV and a high-resolution transmission electron microscopy (TEM) system (JEOL, Model JEM 2100F). The structure of the prepared InSe was investigated using a Raman microscope (Horiba LabRAM HR800) with a 532 nm laser source. The absorption spectra of InSe nanosheets was obtained using a spectrophotometer (Model UH4150, Hitachi, Tokyo, Japan). The thickness distribution of the InSe film/layer was acquired using an atomic force microscopy (AFM) system (Bruker Dimension Ico). Electrical signals of the sensors were collected using a CGS-MT Micro Analysis System (Beijing Elite Tech. Co., Ltd., Beijing, China). A dynamic gas distribution device (Model DGL-III) was also purchased from Beijing Elite Tech. Co., Ltd. (Beijing, China). Light-emitting diodes (LEDs) with different wavelengths that were used to illuminate the device were purchased from EPILEDS Tech. Co., Ltd. (Taiwan, China).

Preparation of InSe Nanosheets. InSe nanosheets were obtained through the LPE method. Briefly, 200 mg of bulk InSe crystal was added in 20 mL of NMP, and then the mixture was ground in an agate mortar for 60 min, followed by further ultrasonication for 8 h at a power of 300 W at temperatures of <20 °C (to decrease possible degradation at elevated temperature). Subsequently, the obtained liquid was centrifuged at a speed of 2500 rpm for 5 min. The supernatant was sucked out and further centrifuged at 18 000 rpm for 10 min; the final sediment was collected and redispersed in 5 mL of NMP.

Preparation of InSe Gas Sensors. First, the IDEs were soaked in acetone for 15 min, followed by washing with ethanol and deionized water successively, and drying with nitrogen at 50 °C. After that, InSe nanosheets (3.2 mg/mL) were painted on the surface of IDEs by using ultrafine brush. Compared with spin coating and screen-printing methods, brush coating has minimum requirements toward the coating material, the coated substrate and the equipment. In addition, it is relatively easy to control the modified area compared with drop coating. The prepared sensors (InSe/IDEs) were then dried in a vacuum oven at 90 °C for 2 h.

For comparison, one-chip InSe sensors were prepared by a micromechanical exfoliation method using a hand-built 2D-material transfer platform.

https://dx.doi.org/10.1021/acs.analchem.0c01941
Anal. Chem. 2020, 92, 11277−11287

Gas-Sensing Experiments. The gas sensing performances of InSe/IDEs were investigated through a commercial gas-sensing measurement system. An InSe/IDEs was placed into a mini probe station consisting of a stainless-steel chamber with the size of 1 L and a source and measurement meter. A certain amount of target gases with a dynamic humidity were independently injected into the chamber, which were controlled by a gas distribution device. The device contains 5 mass flow controllers and a humidity control unit, whose schematic diagram can be seen in Figure S1 in the Supporting Information. All the measurements were performed at a bias voltage of 1 V at RT. The total gas flow rate was fixed at 1000 sccm, and the diluting gas (background gas) was N_2 or dry air. Sensor response is defined (expressed as a percentage) as $((R_g - R_0)/R_0) \times 100$, where R_g and R_0 is sensor resistance when exposed to gas analyte or background gas, respectively. Herein, the response/recovery time is set as the time to reach 90% response/recovery equilibrium upon exposure to target gas or background gas.

Prior to use, the devices were stabilized under UV irradiation in N_2 for 30 min.

■ RESULTS AND DISCUSSION

Characterizations of Materials and Devices. InSe nanosheets were obtained via the LPE method, and the preparation process is briefly described in Figure 1a. TEM analysis was performed to investigate the morphology of the as-prepared InSe nanosheets. As shown in Figure 1b, the nanosheets have intact nanoscale structure with wide lateral dimensions (~200 nm). Energy-dispersive X-ray spectroscopy (EDS) mapping of selective area (Figure 1b) reflects that In and Se elements are uniformly distributed over a piece of sheet, and their atomic ratio is ~1:1, according to the EDS spectra (see Figure S2a in the Supporting Information). Clear lattice fringes in the HRTEM image in Figure 1c suggest high crystallinity of the nanosheets. An interplanar spacing of 3.46 or 3.55 Å corresponds to lattice surface $(10\bar{1}0)$ or $(01\bar{1}0)$ of pristine InSe (JCPDS File No. 34-1431). SAED pattern (insert of Figure 1c) taken from the HRTEM image exhibits clear diffraction spots, which can be also matched with the structural information on pristine InSe crystal. AFM image (Figure 1d) shows typical InSe nanosheets on silicon wafer, and average thickness value is 5.75 ± 0.20 nm (n = 40). The thickness distribution of InSe can be seen in Figures S2b and S2c in the Supporting Information. According to the previous reports, the interlayer distance of InSe nanosheets is ~0.83 nm,[30,31] indicating that most of InSe nanosheets have a seven-layer structure. Besides, InSe can change from indirect bandgap to direct bandgap as the number of layers increases up to six. Thus, it is reasonable to deduce that the as-prepared InSe nanosheets have a direct bandgap, which is favorable for the generation of photogenerated carriers.[25] Since the productivity of nanosheets by LPE method has a positive correlation with the thickness within a certain range, fewer layers of InSe nanosheets are easier to prepare, in comparison with TMDs that only exhibit direct band gaps in a monolayer. Figure 1e exhibits the Raman spectrum of InSe on IDEs. Seven characteristic modes of InSe can be observed at 532 nm laser excitation. $A_1'(\Gamma_1^2)$ at 117 cm^{-1}, $E_1'(\Gamma_3^1)$-TO″ and $E''(\Gamma_3^3)$ at 179 cm^{-1}, $A_2''(\Gamma_1^1)$ at 200 cm^{-1} and $A_1''(\Gamma_1^3)$ at 227 cm^{-1} can be accredited to the modes of bulk InSe. In addition, the presence of $A_2''(\Gamma_1^1)$ at 187 cm^{-1} and $E''(\Gamma_1^3)$-LO at 211 cm^{-1} implies that most InSe are few-layer nanosheets.[31] The morphology of InSe/IDE in the SEM image (Figure 1f) shows that the InSe nanosheets are randomly and uniformly distributed on the electrode surface. Importantly, the resulting InSe membrane is fluffy and porous, which is beneficial to molecular and Knudsen diffusion. The high-resolution SEM image of InSe film can be seen in Figure S2d in the Supporting Information. The AFM graph (Figure 1g) illustrates that the thickness of InSe film on IDE is ~1 μm. The related characterizations of the one-chip InSe device are shown in Figure S3 in the Supporting Information.

Electrical Characteristics and Gas-Sensing Performance of the Sensors. All the prepared devices were tested in a small probe station (gas chamber), as displayed in Figure 2a, where parallel light rays can enter from the quartz glass above the chamber and illuminate all of the sensors vertically. First, the photoresponse intensity of InSe/IDEs was investigated. As shown in Figure 2b, InSe/IDEs demonstrated a broad photoresponse to the radiation from UV to near-infrared (NIR). The resistance of InSe/IDEs decreased as the wavelength decreased, which is in good agreement with previous reports.[19,32] The resistance especially decreased by 3 orders of magnitude in the case of UV irradiation (from ~17 GΩ to ~29 MΩ), which might be attributed to multiple exciton generation.[33] Some details of the multiple exciton generation are described in Figure S4 in the Supporting Information. In this experiment, an excitation wavelength of 365 nm was used to study gas-sensing characteristics, since more photogenerated electrons can be generated under 365-nm irradiation than under visible light. Note that the photoresponse/decay time is relatively slow, compared with reported 2D-material-based photodetectors, which may be related to interface defects, dangling bonds, and oxygen adsorbed on the surface of InSe nanosheets.[34,35] Apart from photoresponse, stability and repeatability of InSe/IDEs toward 365-nm irradiation were also tested, and the results are given in Figure S5 in the Supporting Information.

The I–V characteristics of InSe/IDEs were studied as displayed in Figures 2c and 2d. Consistent with the above results, the current values were increased by 3 orders of magnitude with the illumination of UV light. Besides, it is noticed that there is an obvious nonlinear relationship between current and voltage, indicating the existence of Schottky contact between the InSe nanosheets and the Au electrode.[36] According to previous studies, the Schottky contact device might possess higher sensitivity to the target gas, compared with an ohmic contact device.[37] In addition, the currents of the device under nitrogen and air atmospheres were different. This is because O_2 molecules in air can capture electrons on the InSe surface, resulting in a decrease in current. When the sensor was exposed to NO_2, which is a strong electron acceptor, the current further decreased significantly. These results prove the possibility of the sensor to detect NO_2 both in nitrogen and air atmospheres.

Figure 2e presents the responses of InSe/IDEs toward 1 ppm of NO_2 at different operating temperatures, ranging from 30 °C to 300 °C. In this experiment, the sensor was first exposed to NO_2 with a concentration of 1 ppm. Air was injected into the gas chamber to replace the target gas when the resistance became stable. It can be observed that the sensing response increased gradually with temperature until 200 °C (from 35% to 688%) and decreased with further temperature elevation. Such a phenomenon is similar to the trend of traditional NO_2 sensors, based on metal oxide.[38,39]

Table 1. 2D-Material-Based Gas Sensors for Detecting NO_2

sensing material	detection range (ppm)	response (ppm^{-1})	working state	response/recovery times (s)	ref
p-type graphene layers	1−10	12%	100 °C, air	1000/>3600	42
n-type SnS_2 flaks	0.6−10	360%	120 °C, air	170/140	43
p-type $PtSe_2$ layers	0.1−1	0.25%	RT, N_2	30/10	44
p-type $MoSe_2$ nanosheets	5−100	3%	RT, N_2	300/600	29
p-type $MnPS_3$ nanosheets	0.1−80	400%	RT, N_2	96/220	45
p-type NbS_2 nanosheets	0.5−10	3%	RT, air	3000/9000	46
p-type SnS flakes	0.15−3.75	18%	60 °C and 0.3 mW/cm^2, white light, air	40/1040	47
n-type SnS_2/rGO hybrids	0.125−1	650%	1 mW/cm^2, red light, air	75/242	48
p-type $MoTe_2$ layers	0.5−100	900%	2.5 mW/cm^2, UV, N_2	300/120	41
n-type MoS_2 nanosheets	5−100	3.6%	1.2 mW/cm^2, UV, Ar	29/350	36
n-type InSe nanosheets	0.05−5	190%	3.2 mW/cm^2, UV, air	233/350	this work
		402%	0.8 mW/cm^2, UV, air	470/1400	this work

Briefly, when a sensor works at low temperature, electrons are insufficient for adequate adsorption of NO_2 in the environment, leading to low sensing response. As the temperature increases, increases in the thermal energy generates more thermally excited electrons, thereby enhancing the sensitivity. However, with further increases in temperature, the accelerated desorption of molecules becomes the dominant factor, which weakens the interaction between NO_2 and InSe nanosheets. In this stage, the response is restrained by quick desorption of NO_2. Apart from response intensity, temperature also has a large effect on response/recovery time. The reduction of response/recovery time with increases in temperature is shown in Figure 2e, and the resistance can be fully restored to the initial state after re-exposure in air under high temperature. Note that the prepared device can still maintain a good response to NO_2 with temperatures increasing up to 300 °C. The good stability in air at high temperature may be due to the formation of a dense oxide layer on the surface of InSe, where the sensing principle might be based on the interaction between In_2O_3 and NO_2. The response data and the relative recovery rate under different temperatures can be clearly seen in Table S1 in the Supporting Information.

The above results suggest that increasing the temperature could significantly boost sensor performance. However, long-term work at high temperature causes high energy consumption and safety issues. At this point, light irradiation, as an alternative method, can play an important role in enhancing sensing behavior. Some literature has that light illumination could reduce the binding energy of materials toward gas molecules, which can further shorten response/recovery time, compared with the heating method.[40] Figure 2f displays the responses of InSe/IDEs toward 1 ppm of NO_2 at different UV intensities in the range of 0−3.2 mW/cm^2. Similar to that observed with increasing temperature, illumination can significantly improve the sensing ability for NO_2. When the power was set at 0.8 mW/cm^2, the sensing performance could compare favorably with that at 150 °C. In addition, one can observe that response as well as response/recovery time decreased and recovery rate increased with increases in light intensity. This phenomenon could be explained as described below. A certain intensity of UV illumination can produce sufficient photogenerated electron−hole pairs. The photogenerated electrons could induce adsorption of NO_2 and elevate sensing response. Besides, the reaction of photogenerated holes with negatively charged NO_2 promotes desorption of NO_2. However, further increased light intensity results in high-speed desorption of NO_2, which accelerates recovery but weakens sensing response.[41] Response and recovery rate under different light intensities are presented in Table S1. Herein, a working intensity of 3.2 mW/cm^2 was chosen in further investigations, since a rapid and recoverable response is essential in practical applications. Figures 2g and 2h present the dynamic response curves of InSe/IDEs for 0.05−5 ppm of NO_2 gas in a dark environment and under 365-nm UV irradiation, where responses had a positive correlation with NO_2 concentrations. It can be seen that the device recoveries are slow and incomplete under darkness conditions, which could be due to the high binding energy of InSe toward NO_2. In comparison, under UV irradiation, the recovery speed is greatly improved. Also, the lower noise level of the sensor under light irradiation, in comparison with that observed under darkness conditions, is another advantage of the sensor.

Figure 2i shows the corresponding relationship between sensor response and NO_2 concentration. The as-prepared device under UV excitation exhibited an ~3 times higher response than that observed under darkness conditions. The data points fit well with the Langmuir isotherm. The equations are

$$\text{response (\%)} = \frac{209.23}{(1/C_{0.88}) + 1.12} \qquad \text{(under UV conditions)}$$

and

$$\text{response (\%)} = \frac{44.52}{(1/C_{1.20}) + 0.43} \qquad \text{(under darkness conditions)}$$

where C is the concentration of NO_2 (with units of ppm). The limit of detection (LOD) under 365-nm irradiation is estimated to be 0.98 ppb (signal-to-noise ratio (S/N) = 3), which is 40 times lower than that under darkness conditions (~40 ppb). The performances of some recent NO_2 sensors based on 2D material are summarized in Table 1. It can be observed that the UV-illuminated InSe/IDEs exhibits a decent response for trace NO_2 with an acceptable response/recovery time, even under an air environment. Note that materials with *p*-type features have higher sensitivity to oxidizing gases (such as NO_2).[41] Further improvement in sensing NO_2 is expected when InSe is converted from *n*-type to *p*-type by ingenious methods. More-extensive comparison with other recently reported NO_2 sensors can be seen in Table S2 in the Supporting Information.

The performance of the one-chip InSe device was also tested (see Figure S6 in the Supporting Information). Compared with InSe nanosheets, the one-chip InSe showed responses similar to those of NO_2 with shorter response/recovery time (~310 s/

https://dx.doi.org/10.1021/acs.analchem.0c01941
Anal. Chem. 2020, 92, 11277−11287

~300 s) under darkness conditions. However, under UV illumination (0.8 mW/cm^2), the sensor showed depressed responses, compared with that under dark conditions. This phenomenon can be attributed to the compact structure of a single chip of InSe. Gas molecules have difficulty in diffusing into the interior of the chip. Thus, the diffusion effect is negligible, while the desorption effect is more likely to occupy a dominant role under UV light illumination, so that the response is decreased. In addition, the poor stability of the one-chip device is another drawback. In our test, only ~60% response was retained after 15 days of testing.

Oxygen is one of the composition gases of air. The theoretical calculation results show that the interaction between O_2 and the InSe surface is very weak, belonging to physisorption. Figure 3a presents the most stable configuration

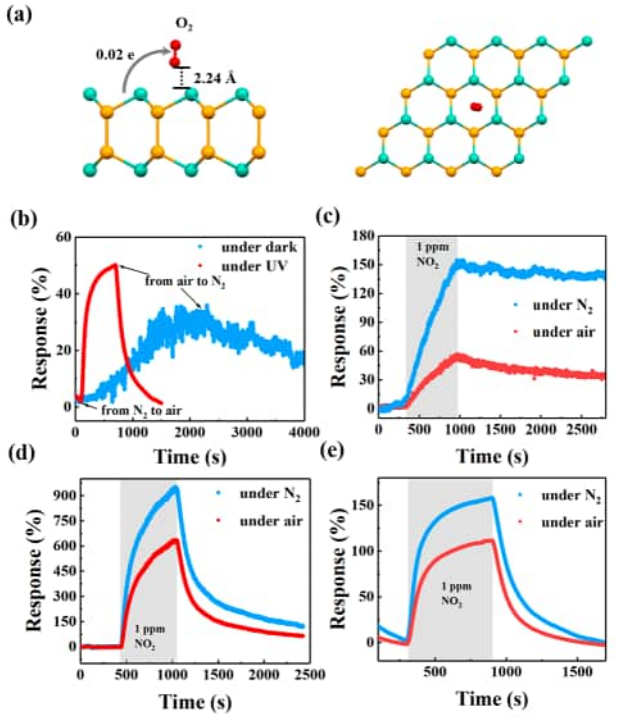

Figure 3. (a) Top and side views of the most stable configuration of O_2 on InSe, where In and Se are illustrated by orange and green balls, respectively. (b) Dynamic response of InSe/IDEs to air (210 000 ppm of O_2) and N_2 at 365 nm UV and under darkness conditions. Dynamic response of InSe/IDEs to 1 ppm of NO_2 (c) at RT and (d) at 150 °C under different vapor atmospheres. (e) Dynamic response of InSe/IDEs to 1 ppm of NO_2 under different vapor atmospheres at 365 nm UV.

of O_2 on InSe, where O_2 is almost vertical above the hexagon center, with the vertical distance of 2.24 Å. InSe has a weaker adsorption energy (−76 meV) for O_2 adsorption, in comparison with phosphene (−270 meV),[49] indicating the difficulty in oxidation of InSe under dry air conditions. As depicted in Figure 3b, the response was unrecoverable after the background was switched from air to N_2, implying the hardness of O_2 desorption from the surface of InSe without extra energy. Besides, under darkness conditions, exposure to air (containing ~21% O_2) only yielded ~30% response. The low sensitivity is consistent with the results of theoretical calculations (low charge transfer number). In comparison, with the help of UV illumination, the sensing response to O_2 was enhanced to ~50%; a much faster responding speed was obtained and the device fully recovered to its initial resistance when air was replaced with N_2. The related equations are shown below:

$$hv \leftrightarrow h^+_{(hv)} + e^-_{(hv)} \quad (1)$$

$$O_{2(gas)} \leftrightarrow O_{2(ads)} \quad (2)$$

$$O_{2(ads)} + e^-_{(hv)} \leftrightarrow O_2{}^-{}_{(ads)} \quad (3)$$

$$O_2{}^-{}_{(ads)} + h^+_{(hv)} \leftrightarrow O_{2(gas)} \quad (4)$$

Similar to the effect toward NO_2, photogenerated electrons could induce the adsorption of O_2 molecules, and photogenerated holes could react with adsorbed O_2^-, which could increase the desorption of O_2. The effect of background gas on NO_2 sensing performance is revealed in Figures 3c−e. It can be seen that the presence of O_2 attenuated the sensing response to NO_2 under darkness conditions at RT. This phenomenon can be attributed to the fact that O_2 can also be considered as an electron acceptor and compete with NO_2 for combining electrons on InSe. In addition, when the working state was set at RT, the preadsorbed O_2 is stable on the surface of InSe, resulting in a limited number of free electrons (a high depletion region in InSe) and a higher energy level of surface state, compared with the Fermi level. These elements might hinder NO_2 from further adsorption on InSe. When the temperature increased to 150 °C, the oxygen species, most of which are O_2^- at this temperature,[50] could absorb thermal energy and began to desorb. Under these conditions, NO_2 molecules compete and interact with oxygen species on the InSe surface. According to previous reports, the possible reactions are as follows:[51]

$$NO_{2(gas)} \leftrightarrow NO_{2(ads)} \quad (5)$$

$$NO_{2(ads)} + e^- \leftrightarrow NO_2{}^-{}_{(ads)} \quad (6)$$

$$NO_{2(gas)} + O_2{}^-{}_{(ads)} + 2e^- \leftrightarrow NO_2{}^-{}_{(ads)} + 2O^-{}_{(ads)} \quad (7)$$

Experimental results showed that the response in air was slightly lower than that in N_2, implying that eqs 5 and 6 are dominant reactions under this circumstance. In other words, the presence of O_2 hinders the sensing response to NO_2, rather than helping NO_2 to further capture the electrons from the conduction band of InSe. The similar phenomenon can also be observed under UV irradiation, as displayed in Figure 3e. The above analyses suggest that the influence of oxygen is negligible in measuring NO_2 under UV irradiation.[52]

Humidity is another factor that must be considered, especially in exhaled gas detection. Similar to O_2, H_2O molecule can be physically adsorbed on InSe, and their most stable configuration is displayed in Figure 4a. H_2O molecule is located above the hexagon center (2.32 Å), where H atoms point to the top Se atoms. The calculated adsorption energy is −189 meV, and the number of charge transfer is around 0.08 e from InSe to H_2O. Figure 4b presents the sensing response to RH from 4% to 90% at dark condition. It can be seen that with the continuous increase of RH, the value of the response negatively increased (the resistance was decreased), indicating that water molecules seem to play a traditional n-type doping effect (as electron donor) in this process. This effect is contrary to theoretical calculations and might be explained by the proton transfer mechanism (Figure 4c).[53] When the sensor

https://dx.doi.org/10.1021/acs.analchem.0c01941
Anal. Chem. 2020, 92, 11277−11287

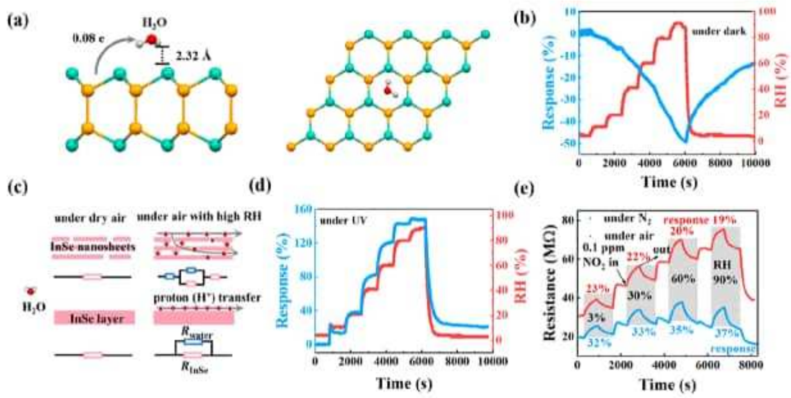

Figure 4. (a) The top and side views of the most stable configuration of H_2O on InSe. (b) Dynamic response of InSe/IDEs to different humidity under darkness conditions, where the background is air. (c) The scheme of InSe nanosheets and one-chip InSe layer and the related equivalent circuit diagram under dry air and under air with high RH. (d) Dynamic response of InSe/IDEs to different humidity levels under 365-nm UV irradiation. (e) Dynamic response of InSe/IDEs to 0.1 ppm of NO_2 at different humidity levels under 365-nm UV irradiation.

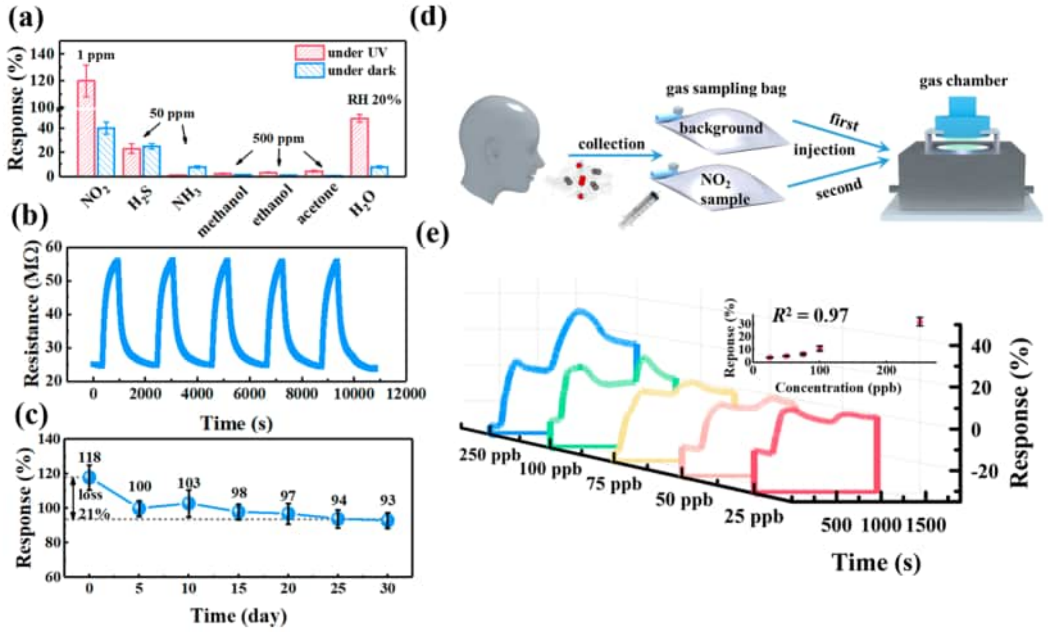

Figure 5. (a) Responses of InSe/IDEs to 1 ppm of NO_2 gas and six other gases, where the response is taken as an absolute value (n = 3). (b) Repeated dynamic responses of InSe/IDEs for 1 ppm of NO_2. (c) Long-term stability of InSe/IDEs to 1 ppm of NO_2 (n = 3). (d) Schematic process regarding breath analysis using an InSe-based sensor. (e) Response curves of InSe/IDEs to simulated NO_2 breath samples at different concentrations; inset shows calibration curves correlating the response with the concentration of NO_2 (n = 3).

was placed in dry air, the resistance mainly depends on the conductive behavior of InSe and the contact barrier. With increasing humidity, physically adsorbed H_2O molecules would aggregate on InSe to form a proton transfer layer, and H_3O^+ or H^+ would be transferred between the surface water droplets, thus reducing the resistance of the sensor. The related reaction equations are

$$H_2O_{(ads)} \leftrightarrow H^+ + OH^- \quad (8)$$

$$2H_2O_{(ads)} \leftrightarrow H_3O^+ + OH^- \quad (9)$$

Similar experimental results were obtained when the background vapor was N_2 (see Figure S7a in the Supporting Information). In addition, one-chip InSe was also tested under the same condition, and the results were shown in Figure S7b in the Supporting Information. When RH was below 60%, the device demonstrated a positive response (resistance increased). At this time, the role of H_2O in capturing electrons was mainly exhibited. When humidity continued to rise, the surface of InSe was mostly occupied by water. Proton transfer effect was dominant, and the device exhibited a negative response. Compared with a one-chip InSe layer, the InSe film stacked with nanosheets has porous structure. H_2O molecules could be more likely to diffuse into the film, so that proton-transfer effect is dominant on the change of resistance.

Interestingly, InSe/IDEs displayed different response to humidity under UV illumination (see Figure 4d, as well as Figure S7c in the Supporting Information); similar results have been reported in humidity sensors based on metal oxides.[54] Apart from direct electron capture by H_2O molecules, the increased resistance might also be contributed by the following reactions:[55]

$$h^+_{(h\nu)} + H_2O_{(ads)} \leftrightarrow H^+ + \bullet OH \quad (10)$$

$$h^+_{(h\nu)} + OH^- \leftrightarrow \bullet OH \quad (11)$$

$$2h^+_{(h\nu)} + 2H_2O_{(ads)} \leftrightarrow 2H^+ + H_2O_2 \quad (12)$$

$$H_2O_2 + e^-_{(h\nu)} \leftrightarrow \bullet OH + OH^- \quad (13)$$

H_2O molecules could react with photogenerated holes and electrons, while the mobility of the electrons (~1000 cm^2 V^{-1} s^{-1}) was much larger than that of holes (~40 cm^2 V^{-1} s^{-1}) at RT, which caused an increase in InSe resistance under low RH. However, as RH continued to rise (~80%), proton transfer effect was remarkably enhanced, and thus the resistance began to show a downward trend at N_2 atmosphere. This phenomenon was not obvious in air, because more oxygen species were generated under the light excitation, which could compete with H_2O for adsorption on InSe. Briefly, O_2 might hinder the formation of H_2O membrane and weaken the proton transfer effect.

The effect of humidity on NO_2 sensing under UV irradiation is reflected in Figure 4e. In this experiment, NO_2 was injected into the gas chamber when the resistance stabilized at different RH. One can see that the sensor maintained a relatively stable response (~21% for 0.1 ppm of NO_2 in air with a relative standard deviation (RSD) of 8.69%) with increasing RH from 3% to 90%. The similar result was found under darkness conditions (Figure S7d in the Supporting Information) and for sensing 1 ppm of NO_2 (Figure S8 in the Supporting Information). The presence of H_2O seems to have no significant effect on the response toward NO_2, which has been also reported in other TMD-based gas sensors. The sensing behavior for NO_2 was relatively complicated, because there were diverse oxygen species and water derivatives existing on the interface. Besides capturing electrons directly on the InSe surface, the obtained NO_2^- might further react with H^+, which not only attenuates the proton transfer effect, but also accelerates H_2O_2 to capture electrons on InSe. The related reactions are shown in eqs 14 and 15:[53,56]

$$OH^-_{(ads)} + H^+_{(ads)} + NO_{2(gas)} + e^- \leftrightarrow NO_2^-{}_{(ads)} + H_2O_{(gas)} \quad (14)$$

$$NO_2^-{}_{(ads)} + H^+ \leftrightarrow HNO_2 \quad (15)$$

Selectivity toward NO_2 against other common biomarkers in breathing gas was examined. As presented in Figure 5a, the sensor exhibited good selectivity for NO_2, under both dark and light conditions, because of the high binding energy and charge transfer of InSe for the NO_2 molecule. The related dynamic responses of InSe/IDEs for others gases are displayed in Figure S9 in the Supporting Information. To investigate repeatability, the sensor was exposed to 1 ppm of NO_2 for five cycles (Figure 5b). Clearly, a stable and recoverable response was obtained, demonstrating decent repeatability of the fabricated sensor. After testing, the devices were stored under dark air condition. Compared with the initial response, ~80% of response intensity was retained after one month, implying acceptable stability of long-term utilization (Figure 5c). Note that the surface of InSe was oxidized after a long period of use, and the gas-sensitive interface might become an *n*−*n* type InSe/In_2O_3 heterojunction. The EDS-mapping images demonstrate the oxidation of InSe on IDEs after long-term use (see Figure S10 in the Supporting Information). Apart from that, a mild oxygen plasma treatment was used to improve the stability of sensor. The result is shown in Figure S11 in the Supporting Information.

Practicability of the sensor was investigated by analyzing simulated NO_2 breath samples. In fact, direct detection of trace NO_2 in breath samples is difficult, because of high humidity and the presence of volatile organic compounds (VOCs), which could mask sensing signals from NO_2. Herein, a blank breath sample was used as a background atmosphere. The sample containing NO_2 was injected into a gas chamber when the resistance was stabilized in a blank background. The operation process is illustrated in Figures 5d and 5e. It was found that, when the blank background was injected in, the resistance initially increased and then decreased to a stable level, which has much to do with the change in RH (from ~30% to ~85%).[57] After NO_2 samples were injected, the response had a decent positive linear relationship with the concentration of NO_2, implying good practicability in detecting ppb-level nitrogen oxides (NO_x) in exhaled gas for disease diagnosis. In real applications, the dead space gas, an exhaled gas without NO_2 molecules, could be collected and injected as the background gas.

Gas Sensing Mechanism. The gas sensing mechanism of InSe/IDEs can be attributed to the charge carrier transfer and Schottky barrier modulation, which was the same as other reported 2D-material gas sensors.[58] According to the DFT calculation, the NO_2 molecule is located ~2.69 Å above the hexagon center, and the plane of NO_2 is almost perpendicular to InSe (see Figure 6a). The binding energy and the amount of charge transfer are calculated to be −272 meV and −0.12 e, respectively, which are larger than that of other gas molecules and might be related to the coexistence of a large dipole moment of NO_2 and resonant molecular levels with the InSe states.[23] The results implied that InSe can easily and selectively adsorb NO_2 molecules, thus bringing about a big change in the number of carriers, which is comparable to the calculated results of MoS_2 (see Table 2). Although the binding energy of InSe for NO_2 is lower than that of phosphene, they show the same amount of electron transfer, which means that InSe not only possesses high sensitivity to NO_2, but also has a faster desorption rate than phosphene. As an *n*-type semiconductor, when InSe was exposed to NO_2 (oxidizing gas), a certain number of electrons flowed from the InSe conduction band to the adsorbed NO_2, leading to a shift of the Fermi level toward the valence band. As a result, the Schottky barrier (V_s) was enhanced and the built-in potential was decreased. In other words, the resistance of the device was increased after adsorbing NO_2, which can be illustrated by the following equation, which is suitable for *n*-type semiconductors.[59]

$$R = R_0 \exp\left(\frac{qV_s}{kT}\right) \quad (16)$$

Herein, R and R_0 represent the sensor resistance before and after contacting with target gas, respectively; q is the elementary charge.

V_s is the Schottky barrier from the Au/InSe and InSe/InSe interfaces, k the Boltzmann constant, and T the working temperature. In addition, the sensing film consists of randomly connected InSe nanosheets, which possess more edge sites, compared to the one-chip InSe. These sites might serve as active sites and participate in adsorption of NO_2. Besides, the actual concentration (C_I) of the target gas in the sensing film is

https://dx.doi.org/10.1021/acs.analchem.0c01941
Anal. Chem. 2020, 92, 11277−11287

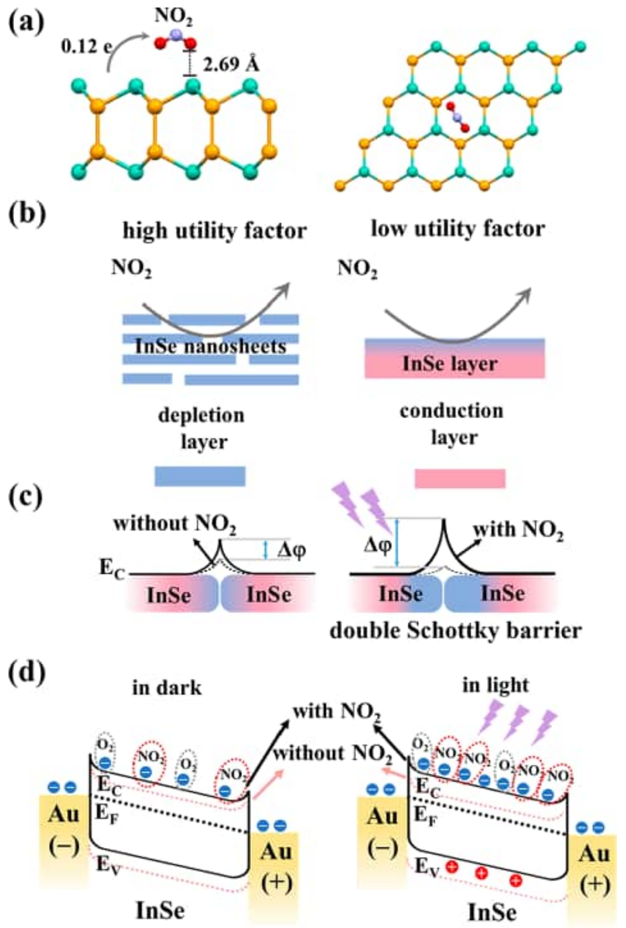

Figure 6. (a) Top and side views of the most stable configuration of NO_2 on InSe. (b) Diffusion model of NO_2 molecules at InSe-nanosheet film and at the one-chip InSe layer. (c) Potential barrier model of the InSe interface under darkness conditions and under 365 nm UV irradiation. (d) Band diagram of the sensor depicting interaction between InSe and NO_2 molecules under two conditions.

Table 2. DFT-Calculated Adsorption Energy (E_{ads}) and Charge Transfer (Q) from the Monolayer to the Adsorbed Molecule

material	molecule	E_{ads} (meV)	Q (e)	ref
InSe	O_2	−76	−0.02	this work
InSe	H_2O	−189	−0.08	this work
InSe	NO_2	−272	−0.12	this work
graphene	NO_2	−67	−0.10	60
MoS_2	NO_2	−276	−0.10	61
SnS_2	NO_2	−150	−0.05	43
phosphene	NO_2	−680	−0.12	49

less than that outside the sensing film (C_O), where the relationship between them can be probably expressed by the following equations:[62]

$$C_I = C_O \frac{\cosh\left(1 \frac{x}{L}\right) L \sqrt{\frac{k}{D_k}}}{\cosh\left(L \sqrt{\frac{k}{D_k}}\right)} \quad (17)$$

$$D_K = \frac{4r}{3}\sqrt{\frac{2RT}{\pi M}} \quad (18)$$

Here, x is the depth of gas diffusion into the sensing film, L is the film thickness, D_K is the Knudsen diffusion coefficient, k is the surface reaction rate, T is the temperature, r is the pore radius of the sensing film, R is the gas constant, and M is the molecular mass of target gas. Stacked films have more molecular diffusion channels (large average pore radius), which means that InSe/IDEs has a higher "utility factor" than that of the one-chip InSe device that suffers from a high gas-dilution effect (Figure 6b).[63] Apart from that, electron capture effect of InSe/SiO_2 interface might negatively affect the carrier number of the device and, hence, reduce sensing performance, which could be relieved by sensing film with a certain thickness.[64,65]

Apart from the excellent gas-sensing properties of pristine InSe, 365 nm UV illumination acts as an essential contributor to sensing performance. First, light illumination can refresh the surface of InSe. Normally, in a dark environment, oxygen molecules adsorbed on the edge sites of InSe and the Se defects may not be easily removed, because of low thermal energy. In this case, the number of free electrons is limited and the energy level of surface state is higher than the Fermi level, impeding NO_2 adsorption on InSe. According to the calculation, the bandgap of prepared InSe is 1.44 eV (see Figure S12 in the Supporting Information). Thanks to the narrow and direct bandgap, electron−hole pairs can be easily generated under 365 nm UV illumination, and the preadsorbed oxygen can be desorbed by reacting with the photoexcited holes (eq 4). As a result, the surface state returns to the initial position (below the Fermi level) and thus more active sites are refreshed to adsorb NO_2.[66] Especially in an exhaled gas test, a large number of preadsorbed water and VOCs are also activated under 365-nm UV illumination. Therefore, NO_2 can easily adsorb on the active sites, because of the high binding energy, and increase the resistance with the assistance of water derivatives (see eqs 14 and 15). This could explain the good sensitivity to NO_2 in breath samples. Apart from that, light irradiation can produce sufficient photoexcited electrons. Accordingly, more NO_2 can be adsorbed and capture electrons from the conduction band, and thus the Schottky barriers are elevated (see Figures 6c and 6d),[67,68] which could greatly increase the resistance. Ultimately, the recovery time in NO_2 detection was obviously reduced under 365-nm UV illumination, because photoexcited holes can react with the adsorbed NO_2^- and accelerate the desorption rate. The related reaction is shown as follows:

$$NO_{2}^{-}{}_{(ads)} + h^+ \leftrightarrow NO_{2(gas)} \quad (19)$$

In addition to the help from photoexcited holes, photoexcited plasmons in InSe might be another promoting factor for NO_2 desorption.[41] According to the reports, the molecular desorption efficiency is inversely proportional to the excitation wavelength of light. Compared to the wavelength of visible light, 365 nm is more favorable for NO_2 desorption.[69] The comparison of using visible light and UV light is displayed in Figures S13 and S14 in the Supporting Information.

■ CONCLUSION

This work has described an optoelectronic gas sensor based on direct-bandgap InSe nanosheets prepared via the LPE method. Theoretical results proved that InSe has excellent sensitivity and selectivity to NO_2. Thanks to the outstanding photo-sensitivity and the superior gas-sensing property of InSe, the

sensor showed a reversible and extremely high response to ppb-level NO_2 gas under UV illumination. During the tests, UV irradiation can refresh active sites of InSe and promote the adsorption and desorption of NO_2 molecules. As a result, the prepared sensor exhibits good gas-sensing performance, even facing a complexed sample such as human breath. Compared with one-chip InSe devices, the sensors stacked by InSe nanosheets are not only easier to prepare, but also have better long-term stability. It can be considered that light-activated platform based on InSe is a very promising candidate for practical applications in detecting trace NO_2.

■ ASSOCIATED CONTENT

Supporting Information

The Supporting Information is available free of charge at https://pubs.acs.org/doi/10.1021/acs.analchem.0c01941.

Methods of NO_2 detection in human breath and theoretical calculation, the schematic diagram of the gas-sensing measurement system, more details of characterization and sensing performance of InSe/IDEs and one-chip InSe device and the details of O_2 plasma treatment for InSe/IDEs (PDF)

■ AUTHOR INFORMATION

Corresponding Authors

Jia Zhou − *School of Science, Harbin Institute of Technology, Shenzhen 518055, China*; orcid.org/0000-0001-5418-5141; Email: jiazhou@hit.edu.cn

Han Zhang − *Collaborative Innovation Center for Optoelectronic Science & Technology, International Collaborative Laboratory of 2D Materials for Optoelectronics Science and Technology of Ministry of Education, College of Optoelectronic Engineering, Shenzhen University, Shenzhen 518060, China*; orcid.org/0000-0002-2197-7270; Email: hzhang@szu.edu.cn

Yingchun Li − *School of Science, Harbin Institute of Technology, Shenzhen 518055, China*; orcid.org/0000-0002-5371-3587; Email: liyingchun@hit.edu.cn

Authors

Lu Zhang − *School of Science, Harbin Institute of Technology, Shenzhen 518055, China*

Zhongjun Li − *Collaborative Innovation Center for Optoelectronic Science & Technology, International Collaborative Laboratory of 2D Materials for Optoelectronics Science and Technology of Ministry of Education, College of Optoelectronic Engineering, Shenzhen University, Shenzhen 518060, China*

Jiang Liu − *School of Science, Harbin Institute of Technology, Shenzhen 518055, China*

Zhengchun Peng − *Collaborative Innovation Center for Optoelectronic Science & Technology, International Collaborative Laboratory of 2D Materials for Optoelectronics Science and Technology of Ministry of Education, College of Optoelectronic Engineering, Shenzhen University, Shenzhen 518060, China*; orcid.org/0000-0002-7114-1797

Complete contact information is available at: https://pubs.acs.org/10.1021/acs.analchem.0c01941

Author Contributions

$^{\nabla}$These authors contributed equally.

Notes

The authors declare no competing financial interest.

■ ACKNOWLEDGMENTS

The work financially supported by Innovation and Entrepreneurship Projects for Overseas High-Level Talents of Shenzhen (Nos. KQJSCX20180328165437711 and KQTD20170810105439418), National Natural Science Foundation of China (Nos. 81973280 and 81773680). Computer time that was made available by the National Supercomputing Center of China in Shenzhen (Shenzhen Cloud Computing Center) is gratefully acknowledged.

■ REFERENCES

(1) Guarnieri, M.; Balmes, J. R. *Lancet* **2014**, *383*, 1581−1592.
(2) Alving, K.; Weitzberg, E.; Lundberg, J. *Eur. Respir. J.* **1993**, *6*, 1368−1370.
(3) Schwela, D. *Rev. Environ. Health* **2000**, *15*, 13−42.
(4) Barnes, P. J.; Dweik, R. A.; Gelb, A. F.; Gibson, P. G.; George, S. C.; Grasemann, H.; Pavord, I. D.; Ratjen, F.; Silkoff, P. E.; Taylor, D. R.; Zamel, N. *Chest* **2010**, *138*, 682−692.
(5) Broza, Y. Y.; Vishinkin, R.; Barash, O.; Nakhleh, M. K.; Haick, H. *Chem. Soc. Rev.* **2018**, *47*, 4781−4859.
(6) Yao, M. S.; Xiu, J. W.; Huang, Q. Q.; Li, W. H.; Wu, W. W.; Wu, A. Q.; Cao, L. A.; Deng, W. H.; Wang, G. E.; Xu, G. *Angew. Chem., Int. Ed.* **2019**, *58*, 14915−14919.
(7) Campbell, M. G.; Sheberla, D.; Liu, S. F.; Swager, T. M.; Dincă, M. *Angew. Chem., Int. Ed.* **2015**, *54*, 4349−4352.
(8) Campbell, M. G.; Liu, S. F.; Swager, T. M.; Dincă, M. *J. Am. Chem. Soc.* **2015**, *137*, 13780−13783.
(9) Liu, X.; Ma, T.; Pinna, N.; Zhang, J. *Adv. Funct. Mater.* **2017**, *27*, 1702168.
(10) Zhang, L.; Khan, K.; Zou, J.; Zhang, H.; Li, Y. *Adv. Mater. Interfaces* **2019**, *6*, 1901329.
(11) Kumar, R.; Zheng, W.; Liu, X.; Zhang, J.; Kumar, M. *Adv. Mater. Technol.* **2020**, *5*, 1901062.
(12) Xu, T.; Liu, Y.; Pei, Y.; Chen, Y.; Jiang, Z.; Shi, Z.; Xu, J.; Wu, D.; Tian, Y.; Li, X. *Sens. Actuators, B* **2018**, *259*, 789−796.
(13) Kim, Y.-H.; Phan, D.-T.; Ahn, S.; Nam, K.-H.; Park, C.-M.; Jeon, K.-J. *Sens. Actuators, B* **2018**, *255*, 616−621.
(14) Zhou, Y.; Zou, C.; Lin, X.; Guo, Y. *Appl. Phys. Lett.* **2018**, *113*, 082103.
(15) Pham, T.; Li, G.; Bekyarova, E.; Itkis, M. E.; Mulchandani, A. *ACS Nano* **2019**, *13*, 3196−3205.
(16) Espid, E.; Taghipour, F. *Crit. Rev. Solid State Mater. Sci.* **2017**, *42*, 416−432.
(17) Chen, Y.-Z.; Wang, S.-W.; Yang, C.-C.; Chung, C.-H.; Wang, Y.-C.; Chen, S.-W. H.; Chen, C.-W.; Su, T.-Y.; Lin, H.-N.; Kuo, H.-C.; Chueh, Y.-L. *Nanoscale* **2019**, *11*, 10410−10419.
(18) Li, M.; Yang, F. S.; Hsiao, Y. C.; Lin, C. Y.; Wu, H. M.; Yang, S. H.; Li, H. R.; Lien, C. H.; Ho, C. H.; Liu, H. J.; et al. *Adv. Funct. Mater.* **2019**, *29*, 1809119.
(19) Yang, Z.; Jie, W.; Mak, C.-H.; Lin, S.; Lin, H.; Yang, X.; Yan, F.; Lau, S. P.; Hao, J. *ACS Nano* **2017**, *11*, 4225−4236.
(20) Feng, W.; Zheng, W.; Cao, W.; Hu, P. *Adv. Mater.* **2014**, *26*, 6587−6593.
(21) Bandurin, D. A.; Tyurnina, A. V.; Yu, G. L.; Mishchenko, A.; Zólyomi, V.; Morozov, S. V.; Kumar, R. K.; Gorbachev, R. V.; Kudrynskyi, Z. R.; Pezzini, S.; et al. *Nat. Nanotechnol.* **2017**, *12*, 223.
(22) Feng, W.; Wu, J.-B.; Li, X.; Zheng, W.; Zhou, X.; Xiao, K.; Cao, W.; Yang, B.; Idrobo, J.-C.; Basile, L. *J. Mater. Chem. C* **2015**, *3*, 7022−7028.
(23) Cai, Y.; Zhang, G.; Zhang, Y.-W. *J. Phys. Chem. C* **2017**, *121*, 10182−10193.
(24) Ma, D.; Ju, W.; Tang, Y.; Chen, Y. *Appl. Surf. Sci.* **2017**, *426*, 244−252.

(25) Hamer, M. J.; Zultak, J.; Tyurnina, A. V.; Zólyomi, V.; Terry, D.; Barinov, A.; Garner, A.; Donoghue, J.; Rooney, A. P.; Kandyba, V.; et al. *ACS Nano* **2019**, *13*, 2136−2142.
(26) Li, Z.; Qiao, H.; Guo, Z.; Ren, X.; Huang, Z.; Qi, X.; Dhanabalan, S. C.; Ponraj, J. S.; Zhang, D.; Li, J.; et al. *Adv. Funct. Mater.* **2018**, *28*, 1705237.
(27) Curreli, N.; Serri, M.; Spirito, D.; Lago, E.; Petroni, E.; Martín-García, B.; Politano, A.; Gürbulak, B.; Duman, S.; Krahne, R.; et al. *Adv. Funct. Mater.* **2020**, *30*, 1908427.
(28) Yu, Y.; Hu, S.; Su, L.; Huang, L.; Liu, Y.; Jin, Z.; Purezky, A. A.; Geohegan, D. B.; Kim, K. W.; Zhang, Y.; Cao, L. *Nano Lett.* **2015**, *15*, 486−491.
(29) Zhang, S.; Nguyen, T. H.; Zhang, W.; Park, Y.; Yang, W. *Appl. Phys. Lett.* **2017**, *111*, 161603.
(30) Lei, S.; Wen, F.; Ge, L.; Najmaei, S.; George, A.; Gong, Y.; Gao, W.; Jin, Z.; Li, B.; Lou, J.; et al. *Nano Lett.* **2015**, *15*, 3048−3055.
(31) Lei, S.; Ge, L.; Najmaei, S.; George, A.; Kappera, R.; Lou, J.; Chhowalla, M.; Yamaguchi, H.; Gupta, G.; Vajtai, R.; et al. *ACS Nano* **2014**, *8*, 1263−1272.
(32) Kumar, R.; Jenjeti, R. N.; Austeria, M. P.; Sampath, S. *J. Mater. Chem. C* **2019**, *7*, 324−329.
(33) Eshet, H.; Baer, R.; Neuhauser, D.; Rabani, E. *Nat. Commun.* **2016**, *7*, 13178.
(34) Fan, C.; Li, Y.; Lu, F.; Deng, H.-X.; Wei, Z.; Li, J. *RSC Adv.* **2016**, *6*, 422−427.
(35) Hu, P.; Wen, Z.; Wang, L.; Tan, P.; Xiao, K. *ACS Nano* **2012**, *6*, 5988−5994.
(36) Kumar, R.; Goel, N.; Kumar, M. *ACS Sens.* **2017**, *2*, 1744−1752.
(37) Liu, B.; Chen, L.; Liu, G.; Abbas, A. N.; Fathi, M.; Zhou, C. *ACS Nano* **2014**, *8*, 5304−5314.
(38) Sun, J.; Sun, L.; Han, N.; Pan, J.; Liu, W.; Bai, S.; Feng, Y.; Luo, R.; Li, D.; Chen, A. *Sens. Actuators, B* **2019**, *285*, 68−75.
(39) Dang, V. T.; Nguyen, T. T. O.; Truong, T. H.; Le, A. T.; Nguyen, T. D. *Mater. Today Commun.* **2020**, *22*, 100826.
(40) Vijjapu, M. T.; Surya, S. G.; Yuvaraja, S.; Zhang, X.; Alshareef, H. N.; Salama, K. N. *ACS Sens.* **2020**, *5*, 984−993.
(41) Wu, E.; Xie, Y.; Yuan, B.; Zhang, H.; Hu, X.; Liu, J.; Zhang, D. *ACS Sens.* **2018**, *3*, 1719−1726.
(42) Kim, Y. H.; Kim, S. J.; Kim, Y.-J.; Shim, Y.-S.; Kim, S. Y.; Hong, B. H.; Jang, H. W. *ACS Nano* **2015**, *9*, 10453−10460.
(43) Ou, J. Z.; Ge, W.; Carey, B.; Daeneke, T.; Rotbart, A.; Shan, W.; Wang, Y.; Fu, Z.; Chrimes, A. F.; Wlodarski, W.; et al. *ACS Nano* **2015**, *9*, 10313−10323.
(44) Yim, C.; Lee, K.; McEvoy, N.; O'Brien, M.; Riazimehr, S.; Berner, N. C.; Cullen, C. P.; Kotakoski, J.; Meyer, J. C.; Lemme, M. C.; Duesberg, G. S. *ACS Nano* **2016**, *10*, 9550−9558.
(45) Kumar, R.; Jenjeti, R. N.; Sampath, S. *ACS Sens.* **2020**, *5*, 404−411.
(46) Kim, Y.; Kwon, K. C.; Kang, S.; Kim, C.; Kim, T. H.; Hong, S.-P.; Park, S. Y.; Suh, J. M.; Choi, M.-J.; Han, S.; Jang, H. W. *ACS Sens.* **2019**, *4*, 2395−2402.
(47) Jannat, A.; Haque, F.; Xu, K.; Zhou, C.; Zhang, B. Y.; Syed, N.; Mohiuddin, M.; Messalea, K. A.; Li, X.; Gras, S. L.; et al. *ACS Appl. Mater. Interfaces* **2019**, *11*, 42462−42468.
(48) Huang, Y.; Jiao, W.; Chu, Z.; Ding, G.; Yan, M.; Zhong, X.; Wang, R. *J. Mater. Chem. C* **2019**, *7*, 8616−8625.
(49) Ray, S. *Sens. Actuators, B* **2016**, *222*, 492−498.
(50) Barsan, N.; Weimar, U. *J. Electroceram.* **2001**, *7*, 143−167.
(51) Kumar, R.; Al-Dossary, O.; Kumar, G.; Umar, A. *Nano-Micro Lett.* **2015**, *7*, 97−120.
(52) Liu, D.; Tang, Z.; Zhang, Z. *Sens. Actuators, B* **2020**, *303*, 127114.
(53) Song, Y. G.; Shim, Y.-S.; Suh, J. M.; Noh, M.-S.; Kim, G. S.; Choi, K. S.; Jeong, B.; Kim, S.; Jang, H. W.; Ju, B.-K.; Kang, C.-Y. *Small* **2019**, *15*, 1902065.
(54) Hsu, C.-L.; Chang, L.-F.; Hsueh, T.-J. *Sens. Actuators, B* **2017**, *249*, 265−277.
(55) Tan, Y.; Yu, K.; Yang, T.; Zhang, Q.; Cong, W.; Yin, H.; Zhang, Z.; Chen, Y.; Zhu, Z. *J. Mater. Chem. C* **2014**, *2*, 5422−5430.
(56) Yan, W.; Worsley, M. A.; Pham, T.; Zettl, A.; Carraro, C.; Maboudian, R. *Appl. Surf. Sci.* **2018**, *450*, 372−379.
(57) Mansour, E.; Vishinkin, R.; Rihet, S.; Saliba, W.; Fish, F.; Sarfati, P.; Haick, H. *Sens. Actuators, B* **2020**, *304*, 127371.
(58) Bag, A.; Lee, N.-E. *J. Mater. Chem. C* **2019**, *7*, 13367−13383.
(59) Hua, Z.; Li, Y.; Zeng, Y.; Wu, Y. *Sens. Actuators, B* **2018**, *255*, 1911−1919.
(60) Leenaerts, O.; Partoens, B.; Peeters, F. *Phys. Rev. B: Condens. Matter Mater. Phys.* **2008**, *77*, 125416.
(61) Yue, Q.; Shao, Z.; Chang, S.; Li, J. *Nanoscale Res. Lett.* **2013**, *8*, 425.
(62) Sakai, G.; Matsunaga, N.; Shimanoe, K.; Yamazoe, N. *Sens. Actuators, B* **2001**, *80*, 125−131.
(63) Zhang, J.; Qin, Z.; Zeng, D.; Xie, C. *Phys. Chem. Chem. Phys.* **2017**, *19*, 6313−6329.
(64) Chen, I.-C.; Holland, S.; Hu, C. *J. Appl. Phys.* **1987**, *61*, 4544−4548.
(65) Chen, H.; Chen, Y.; Zhang, H.; Zhang, D. W.; Zhou, P.; Huang, J. *Adv. Funct. Mater.* **2018**, *28*, 1801035.
(66) Li, G.; Sun, Z.; Zhang, D.; Xu, Q.; Meng, L.; Qin, Y. *ACS Sens.* **2019**, *4*, 1577−1585.
(67) Kathiravan, D.; Huang, B.-R.; Saravanan, A.; Prasannan, A.; Hong, P.-D. *Sens. Actuators, B* **2019**, *279*, 138−147.
(68) Wang, X.; Liu, Y.; Dai, J.; Chen, Q.; Huang, X.; Huang, W. *Chem. - Eur. J.* **2020**, *26*, 3870−3876.
(69) Chen, R. J.; Franklin, N. R.; Kong, J.; Cao, J.; Tombler, T. W.; Zhang, Y.; Dai, H. *Appl. Phys. Lett.* **2001**, *79*, 2258−2260.

Chem Soc Rev

REVIEW ARTICLE

View Article Online
View Journal | View Issue

Check for updates

Cite this: *Chem. Soc. Rev.*, 2018, **47**, 4781

Published on 11 June 2018. Downloaded on 2/1/2025 6:21:23 AM.

Synergy between nanomaterials and volatile organic compounds for non-invasive medical evaluation†

Yoav Y. Broza, ‡[a] Rotem Vishinkin,‡[a] Orna Barash,[a] Morad K. Nakhleh [bcd] and Hossam Haick *[a]

This article is an overview of the present and ongoing developments in the field of nanomaterial-based sensors for enabling fast, relatively inexpensive and minimally (or non-) invasive diagnostics of health conditions with follow-up by detecting volatile organic compounds (VOCs) excreted from one or combination of human body fluids and tissues (*e.g.*, blood, urine, breath, skin). Part of the review provides a didactic examination of the concepts and approaches related to emerging sensing materials and transduction techniques linked with the VOC-based non-invasive medical evaluations. We also present and discuss diverse characteristics of these innovative sensors, such as their mode of operation, sensitivity, selectivity and response time, as well as the major approaches proposed for enhancing their ability as hybrid sensors to afford multidimensional sensing and information-based sensing. The other parts of the review give an updated compilation of the past and currently available VOC-based sensors for disease diagnostics. This compilation summarizes all VOCs identified in relation to sickness and sampling origin that links these data with advanced nanomaterial-based sensing technologies. Both strength and pitfalls are discussed and criticized, particularly from the perspective of the information and communication era. Further ideas regarding improvement of sensors, sensor arrays, sensing devices and the proposed workflow are also included.

Received 21st April 2018

DOI: 10.1039/c8cs00317c

rsc.li/chem-soc-rev

1. Introduction

Medical diagnostics, or disease diagnosis, is the identification of the presence of a specific disease, based on symptoms manifested in an individual. In order to save lives and provide patients with the optimum treatment, data classification is particularly important for comprehending the etiology and pathogenesis of a disease.[1,2] Therefore, disease classification is based on the following steps: first, identifying as many candidate conditions as possible that can explain the expressed symptoms. Second, an elimination process to rule out the less probable conditions by additional medical tests and observations. And last, fine-tuning, *i.e.*, allowing the physician to reach the stage where only one of the most probable disease candidates remains.[1,2] Modern diagnostics relates to two main categories of diseases: non-infectious (non-transmissible) and infectious diseases.[3,4] More than 60% of the worldwide disease-related deaths are due to non-infectious diseases; they could be slow developing chronic conditions, or more acute ones that result in rapid death.[3] The likelihood of certain non-infectious diseases is influenced by different risk factors, such as genetic background, environment and lifestyle. Thus, related deaths in the next 12 to 15 years are expected to increase by more than 40%.[3] The second most frequent cause of death worldwide is infectious diseases, which leads to >15 million deaths annually.[5]

For the medical world to cope with these growing numbers, novel diagnostic approaches and improved technologies are needed. A whole range of diagnostic methodologies and technologies are being used for diverse infectious and non-infectious diseases.[3–5] It is gradually being accepted that better-quality diagnostic tools should be developed to give a personalized approach. Such an approach should take into full consideration detection of individuals at risk of developing diseases, data interpretation of the diagnostic tests, delivering prognostic data, and foreseeing and following the efficacy of therapies.[6,7] Nowadays, diagnosis is based on a

[a] *Department of Chemical Engineering and Russell Berrie Nanotechnology Institute, Technion – Israel Institute of Technology, Haifa 3200003, Israel. E-mail: hhossam@technion.ac.il; Fax: +972-77-8871880; Tel: +972-4-8293087*

[b] *Univ Paris-Sud, Faculté de Médecine, Université Paris-Saclay, Le Kremlin Bicêtre, France*

[c] *AP-HP, DHU TORINO, Service de Pneumologie, Hôpital Bicêtre, Le Kremlin Bicêtre, France*

[d] *Inserm UMR_S 999, LabExLERMIT, Hôpital Marie Lannelongue, Le Plessis Robinson, France*

† Electronic supplementary information (ESI) available. See DOI: 10.1039/c8cs00317c

‡ These authors contributed equally to this study.

View Article Online

physical examination followed by a range of tests that include chemical, imaging, endoscopic, immunological, genomic and pathological procedures among others. Each has pros and cons, invasive *vs.* non-invasive; life threatening *vs.* safe; expensive *vs.* low-cost; complicated *vs.* simple; point of care *vs.* offline lab; fast *vs.* slow.

One rapidly developing strategy in the diagnostic field is based on volatolomic analysis, *i.e.* the combined analysis of volatile organic compounds (VOCs) emitted from different bodily sources (see below and ref. 8). Real-world implementation of this approach will benefit from a true point-of-care, non-invasive, easy to use and relatively low-cost system that can be an optimal screening tool for different diseases. VOC sampling, in general, and breath sampling, in particular, have high compliance because they are patient-friendly. The general concept addresses diseases in different organs by sampling VOCs from different bodily fluids or tissue (*e.g.* breath, skin, urine, and blood) and measuring them by different sensor techniques for point-of-care (POC) diagnostics (Fig. 1).

For the detection of disease-related VOCs, 2 main sensing strategies can be used. The first is based on a selective sensing mechanism, in which a chemical sensor is designated to

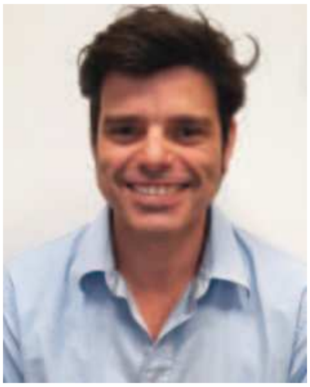

Yoav Y. Broza

Yoav Y. Broza received his PhD in Biotechnology & Food Engineering in 2009 from the Technion – Israel Institute of Technology. He is a senior researcher, head of the biological and analytical team in the Laboratory for Nanomaterial-Based Devices, headed by Prof. Hossam Haick. Yoav served as a researcher and consultant in several biomed and food-tech startups in the field of nanosensors. Additionally, Yoav holds an external lecturer position in the Technion. His current research interests include volatolomics studies, single cell analysis, identification of disease and infectious-agents' biomarkers through volatile gas analysis by analytical methods and nano-arrays, and the development of novel technologies for gas sampling applications.

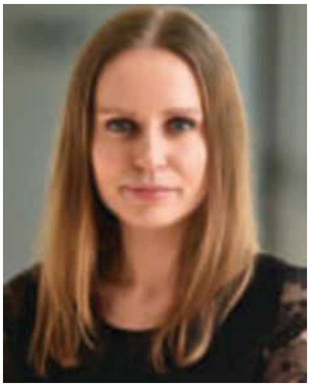

Rotem Vishinkin

Rotem Vishinkin received her Bachelor's (2012) degree in Biochemical Engineering from the Technion – Israel Institute of Technology, Israel. Rotem joined the group of Professor Hossam Haick during her BSc research project on development and characterization of chemical sensors based on field effect transistors of Si nanowires. In August 2014, Rotem obtained her MSc degree in Chemical Engineering and currently continues her direct PhD studies on non-invasive tuberculosis detection based on volatile organic biomarker patterns from skin and breath. Her research interests include development of field effect transistors and gold nanoparticle-based sensors for sensing applications and detection of disease-related volatile biomarkers, especially those emitted from the skin, using mass spectrometry methods and nano-arrays of chemical sensors.

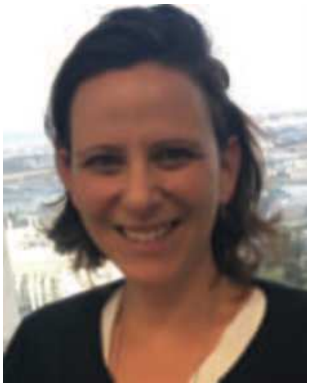

Orna Barash

Orna Barash has finished her PhD in the Laboratory for Nanomaterial-based Devices (LNBD) under the supervision of Prof. Hossam Haick in 2010. Her research focus was on "Detection and Characterization of Volatile Organic Compounds Exhaled by In Vitro Lung Cancer Cells". Orna has been working as a Researcher with the LNBD group for 2 years and held responsibilities of research management. Orna has recently joined a spin-off company named NanoScent as the Director of Research. NanoScent uses the core technology developed by prof. Hossam Haick at the Technion for scent detection in various fields.

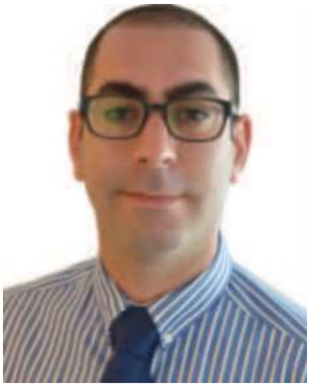

Morad K. Nakhleh

Morad K. Nakhleh holds a BSc in Emergency Medicine (2006) and MSc in Medical Sciences (2011). In 2014, he received his PhD in Nanoscience and Nanotechnology, under the supervision of Prof. Hossam Haick, at the Russell Berrie Nanotechnology Institute, Technion-IIT, Haifa, Israel. Currently, Dr Nakhleh is performing his postdoctoral studies at INSERM UMR_S999, Le Plessis Robinson, France. In his current research, Dr Nakhleh focuses on the volatolomics associated with pulmonary arterial hypertension (PAH). His goal is to explore the underlying biological and molecular mechanisms of the disease by profiling the volatolome both on the vascular level and in exhaled breath, searching for early biomarkers associated with the diseases.

Published on 11 June 2018. Downloaded on 2/1/2025 6:21:23 AM.

 This journal is © The Royal Society of Chemistry 2018

Published on 11 June 2018. Downloaded on 2/1/2025 6:21:23 AM.

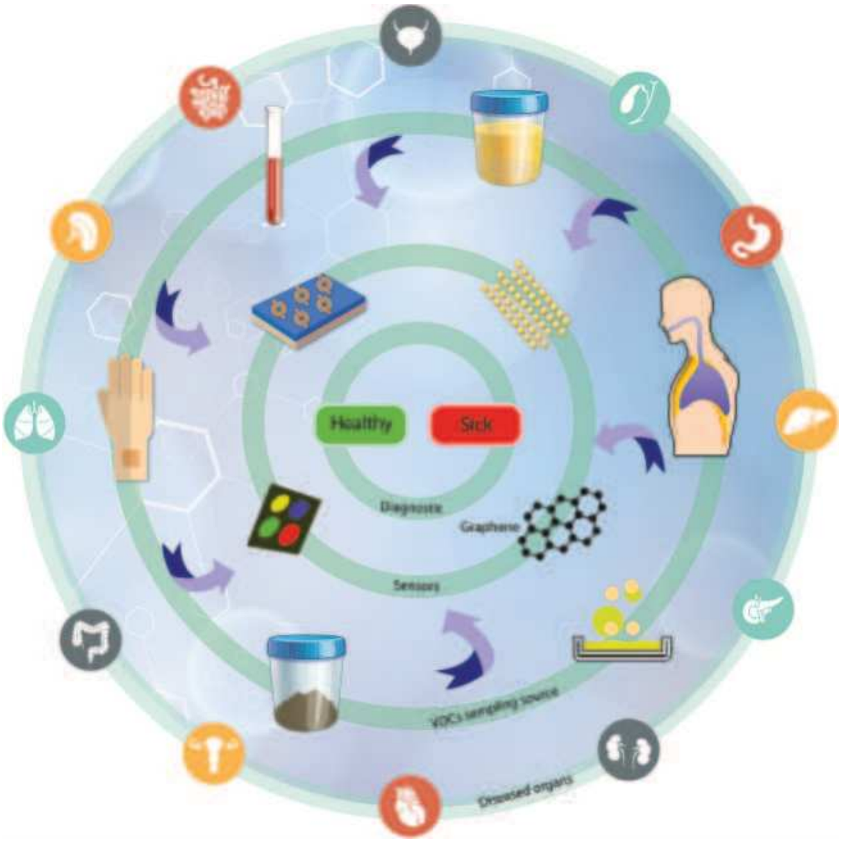

Fig. 1 VOC-based diagnostics approach is divided into several levels. Addressing diseases in different organs; VOC sampling from different bodily fluids/places (such as breath, skin, urine, blood *etc.*); sensor measurement using different sensor techniques; and POC diagnostics based on classification algorithms.

interact and measure the presence of a single compound in a mixture (lock-and-key like). For this approach to be applied, one needs to have specific biomarker information on the medical condition being targeted. The second strategy is bio-inspired by the sense of smell and is based on semi-selective/cross-reactive sensors combined with pattern recognition methods. Since it is hard to find a single and unique VOC that could perform as a stand-alone diagnostic for a specific disease in the clinical world, a cross-reactive sensing approach is more favorable. Nevertheless, for this approach to mature, further progress in sensor technology – increased sensitivity, data-mining and pattern recognition power – is needed. We herein provide an updated overview of current advances in emerging technologies in the field of nanomaterial-based sensors, which are used, or have the potential, for disease and biomarker detection using VOCs. We also discuss new approaches in the field, such as hybrid sensors and future perspectives.

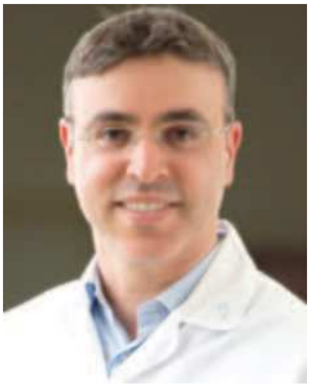

Hossam Haick

Hossam Haick is a Full Professor in the Technion – Israel Institute of Technology and head of three major European consortia, has received numerous prestigious Prizes and Awards and was included in several important lists, including the list of the world's 35 leading young scientists published by MIT's Technology Review, the Nominet Trust 100 list (London), which includes the world's 100 most influential inventors and digital developments, and the Los Angeles-based GOOD Magazine's list of the 100 most influential people in the world. His research interests include nanomaterial-based chemical (flexible) sensors, electronic skin, nanoarray devices for screening, diagnosis, and monitoring of disease, breath analysis, volatile biomarkers, and molecular electronic devices.

2. Origin and emission of volatile organic compounds from human body sources

During each cell's life span, extensive and continuous metabolic processes take place to sustain life, and adapt and react according to local and systemic stimuli.[9] Among the main purposes of these chemical changes are energy production, anabolism (building up of cellular components), catabolism

Published on 11 June 2018. Downloaded on 2/1/2025 6:21:23 AM.

(breaking down of organic matter) and elimination of cellular waste.[9] Under healthy and normal conditions, these processes ensure the well-being of the cells in maintaining vital functions. Metabolic reactions are facilitated mainly by enzymes that in turn interact with different biochemical compounds, including amino acids, proteins, lipids, carbohydrates, minerals and others.[9]

During metabolism, a wide range of byproducts are formed, including a family of organic chemicals characterized by high vapor pressure at both room and body temperatures, thus known as VOCs.[10,11] For example, ketones are produced by cells in the glycolytic pathway of energy production, whereas acetone is synthesized by the decarboxylation of ketone bodies.[12] Isoprene, on the other hand, is formed in the mevalonic pathway of cholesterol synthesis in the cytosol.[11] Many molecular pathways and enzymes constantly produce dozens of other VOCs. Interestingly, due to their relatively small size, low molecular weight and their volatility, these compounds spread from their original microenvironment through blood circulation to distal organs, from which they may be excreted in different body fluids. VOCs are detectable at low concentrations in the peripheral blood, sweat (skin secretion), urine (kidney), feces (gastrointestinal tract) and exhaled breath (lungs). Therefore, VOCs can be highly informative regarding metabolic activity.[12–14]

When subjected to stress under systemic or local stimuli, cells usually respond by activation of different intracellular cascades involving multiple signaling pathways and enzymatic activity. These rapid metabolic reactions change baseline activities and/or the functions of the cells (*e.g.*, accumulation of reactive oxygen species or hypoxia), adapting them to changing micro-environmental requirements.[13,15,16] A wide spectrum of pathophysiological processes occur during disease states that are followed by adaptive and altered metabolism, ranging from the single cell level and up to whole organ response such as the liver and the kidney to maintain homeostasis. During these responses, extensive alterations in VOC production are reflected in the blood composition and excreted body fluids. Therefore, VOCs are informative non-invasive biomarkers, reflecting alterations in metabolism during disease.[17]

Hundreds of different VOC metabolites have been identified in human samples from different sources. VOCs in feces, for example, could be due to bacterial activity in the colon, as well as due to diffusion throughout the colon from both cellular activity and blood stream.[18] In the kidney, on the other hand, VOCs diffuse throughout the glomerular filtrate and end up in the urine. Acids, alcohols, ketones, aldehydes, amines, N-heterocycles, O-heterocycles, VSCs and other VOCs have been detected and quantified in urine.[19,20] A range of compound classes appear in skin VOC profiles, including aldehydes, carboxylic acids, alcohols, ketones, esters and amines.[21] Skin VOCs are derived mainly from gland secretions and skin microbiota activity; therefore, skin VOC diversity and concentrations are directly correlated with the distribution of glands on the skin as well as cutaneous microorganisms.[22,23]

Aside from the systemic alterations in the rate of production of VOCs, their exhaled concentrations are influenced by the difference between the alveolar concentration ($C_{alveolar}$) and ambient air concentration of a given compound. As summarized in the Farhi equation, $C_{alveolar}$ is positively correlated with the concentration in mixed venous blood.[24] On the other hand, it is negatively correlated with the ratio between the alveolar ventilation and cardiac output. Thus, during stress/effort, for example, increase in alveolar ventilation leads to a decrease in $C_{alveolar}$. The second factor negatively affecting the $C_{alveolar}$ is the 'blood:air' partition coefficient, reflecting the solubility of a given compound in the blood. Higher solubility in the blood results with higher binding of the VOC to blood proteins, thus negatively affecting $C_{alveolar}$.[17,24] The third factor is the 'fat:blood' partition coefficient, since a wide range of VOCs can be stored in the fat compartments. Therefore, the total fat tissue and the total blood volume of an individual should be taken into account as factors affecting the blood concentrations and, as a result, the final alveolar concentrations.[17,24]

3. Harnessing VOC data from analytical approaches towards sensor design

Two main approaches have been developed that are used to assess VOCs in biological samples. The goal of the first is to quantitatively profile each of the compounds in each mixture. Identification of the VOC spectrum allows one to study each compound separately and estimate the likelihood that it can serve as an individual biomarker of disease. This approach is based on variations of chromatography w/wo spectrometry, including, but not limited to, Gas Chromatography linked with Mass Spectrometry (GC-MS), proton transfer reaction mass spectrometry, and selected ion flow tube mass spectrometry. Due to these analytical variations and different sampling methods, it is difficult to reach a singular VOC pattern for each disease. On the other end of the spectrum, the second approach is based on different types of chemically-sensitive gas sensors that give a total assessment of the mixture/profile of VOCs in a sample.

Semi-selective/cross-reactive sensors combined with artificial intelligence algorithms have been adopted, which allows the identification of patterns of VOCs as conjoined markers of a disease. While this lacks the ability to identify each VOC present, the overall pattern can be detected and used in future to classify similar patterns. Therefore, in cases of multiple VOC alterations, such as in systemic diseases, this approach should be more compatible with assessment of any disease-associated patterns.[14,23,25] In general, these 2 approaches are independent. However, to gain advantage when designing and developing novel sensing approaches, the knowledge and connection of specific molecules and their physiochemical properties are important. Towards this end, we updated a compilation of the current specific disease-related VOCs (patterns, as obtained from different studies) and their source of sampling, as summarized in Fig. 2, 3 and Table S1 (ESI†). This compilation shows the fraction and relation among the chemical family, disease and source; it allows researchers to focus and tune their potential

This journal is © The Royal Society of Chemistry 2018

Published on 11 June 2018. Downloaded on 2/1/2025 6:21:23 AM.

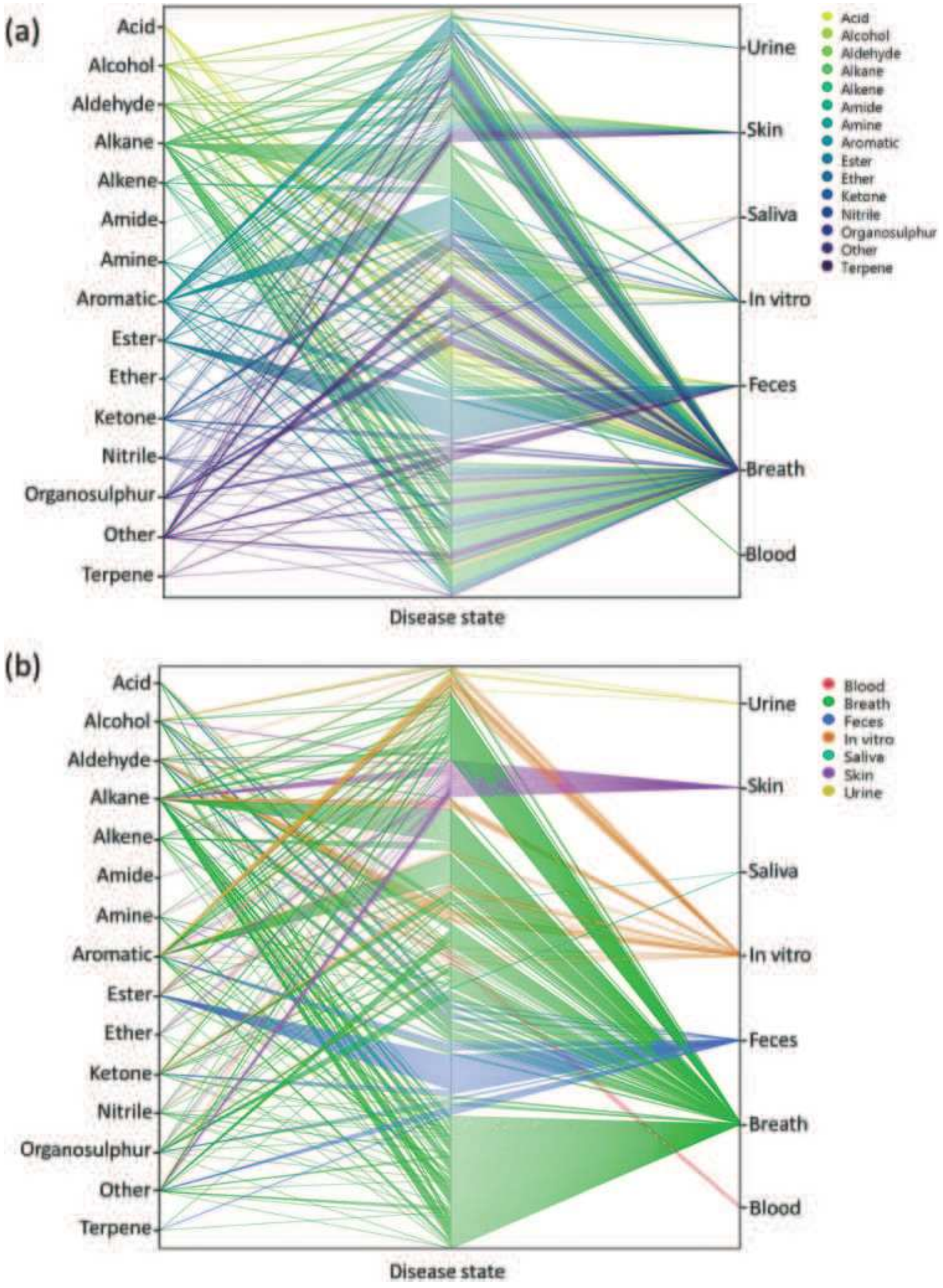

Fig. 2 Relationships between chemical families (on the left side of each graph), disease states (the different diseases addressed, middle part of each graph) and sample source (on the right side of each graph) obtained by spectrometry methods. A specific color palette has been selected for each graph, according to (a) chemical families of VOCs and (b) sample source. Each figure is constructed from all the identified VOCs, while each line represents a single VOC. The more the lines the wider the occurrence of a specific VOC, which could also suggest that it was more thoroughly researched in the studies.

gas-sensing system, based on currently available data. These figures can serve as a map that allows the reader to decipher the ''disease-VOC-source'' connection. This complete information can aid in choosing the correct (existing) sensing device for a clinical condition of interest, tuning the sensor development methodology based on chemical data of potential biomarkers, and developing suitable sampling methods based on the biomarkers' source that fit real world clinical use.

Here are some examples of insights that can be deducted from the figures to improve sensing development for clinical use.

(1) Ester groups are frequently connected with fecal samples in gastrointestinal disease (Fig. 2a and 3b). Thus, in aiming to develop a sensing system for these diseases, it is preferable to focus on a semi-polar sensing layer that can react more strongly with esters that enhance sensitivity. Until recently, all studies that involved diagnosis of gastrointestinal diseases focused on

Published on 11 June 2018. Downloaded on 2/1/2025 6:21:23 AM.

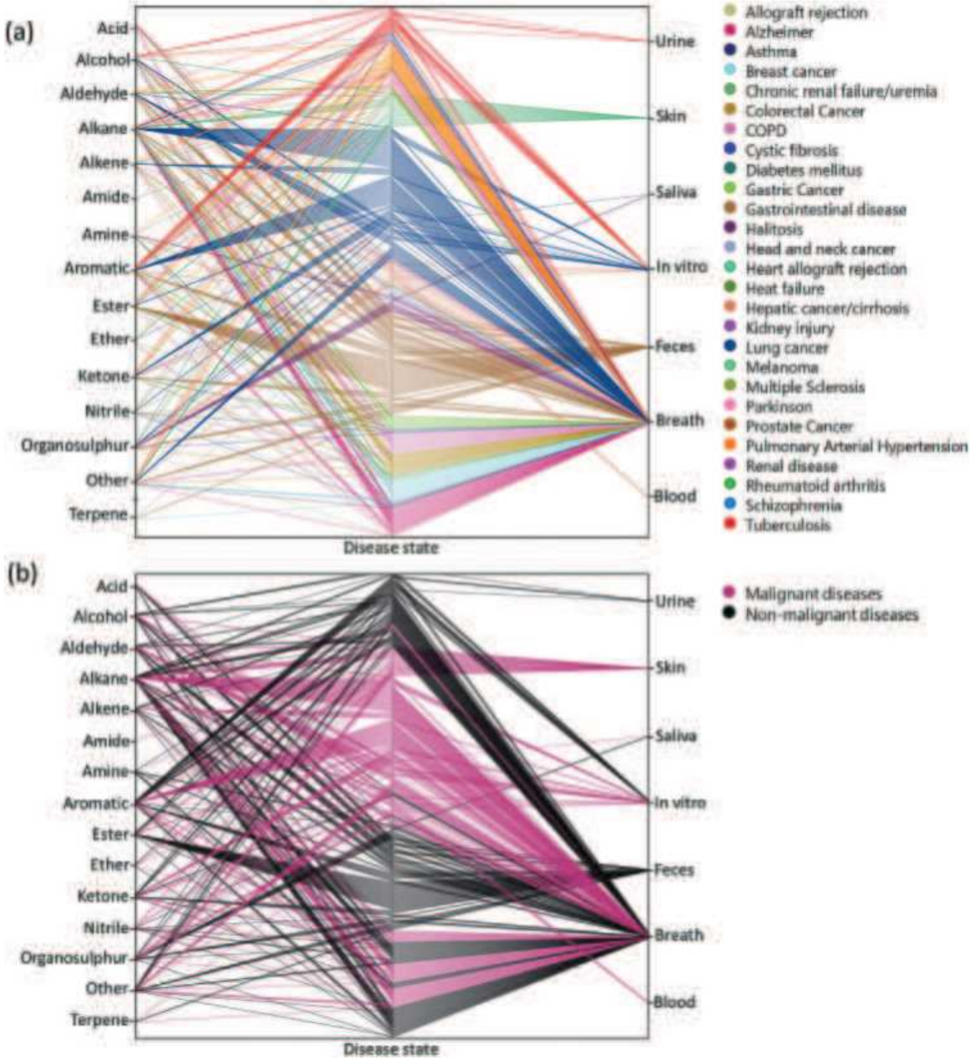

Fig. 3 Relationships between chemical families (on the left side of each graph), disease states (the different diseases addressed, middle part of each graph) and sample source (on the right side of each graph) obtained by spectrometry methods. A specific color palette has been selected for each graph, according to (a) all disease states and (b) malignant and non-malignant diseases. Each figure is constructed from all the identified VOCs, while each line represents a single VOC. The more the lines the wider the occurrence of a specific VOC, which could also suggest that it was more thoroughly researched in the studies.

fecal samples (Fig. 3a). Therefore, if such diseases are of interest, it would be preferable to design a sensor that is suitable for fecal samples, which needs to cope with the noisy VOC background interference that they are likely to possess.

(2) It would appear that malignant diseases (Fig. 3b) have been sampled from three main sources, namely breath, skin and *in vitro* culture. Therefore, to evaluate a proposed sensor system for detection of malignant disease, one should choose tentative biomarkers from either breath or skin.

(3) Halitosis is diagnosed only from breath, and most of the compounds are organosulfur, which means that a potential sensor system will require selective or semi-selective sensing layers for volatile sulfur compounds.

Development of disease diagnostic tools based on nanomaterials must be correlated with the potential pattern of VOCs reported in Fig. 2, 3 and Table S1 (ESI†). The following sections will introduce the VOC sensing abilities of many nanomaterial-based sensors, in both laboratory conditions and real clinical studies to determine those that are best in improving diagnostic accuracy.

4. Nanomaterial-based sensors for VOC detection

4.1. Metal oxide sensors

Semiconducting metal oxides (MOs) are broadly used in chemiresistors as a sensing layer for oxidizing or reducing gases; they usually operate at high temperatures (150–500 °C) compared to other semiconducting sensors.[26–28] MOs also are used as sensing layers in field-effect transistor (FET) devices.[29–31] MOs layers have a high affinity for negatively charged oxygen

 This journal is © The Royal Society of Chemistry 2018

Published on 11 June 2018. Downloaded on 2/1/2025 6:21:23 AM.

moieties (*e.g.* O_2^-, O^-), which leads to the creation of either an electron-depletion or a hole-accumulation surface charge layer, according to the type of semiconductor, n-type and p-type, respectively.[32] The gaseous phase reacts with the adsorbed negatively charged oxygen on the metal-oxide surface, altering the surface-trapped charge density, resulting in changes in resistance.[32]

MOs can be divided according to their electronic structures into 2 groups: transition-MOs (*e.g.* NiO, Cr_2O_3) and non-transition-MOs (*e.g.* ZnO, SnO_2).[33] Transition-MOs that have more than one preferred oxidation state are better suited for gas sensing applications.[33] For sensitive detection at ppb levels, binary, ternary, quaternary or noble metal-decorated MOs are usually used.[33,34] By controlling MO parameters – composition, size, shape, morphology, surface area, fabrication methods, doping levels, microstructures of sensing layers, humidity and operating temperature – their sensitivity and selectivity performance can be tuned to detect gases of interest under real-world conditions.[27,34–49] Experimentally it has been found that as the particle size decreased for impurity-doped SnO_2 and WO_3 nanoparticles, the sensor's response towards H_2, CO, and i-C_4H_{10} or NO_2 and NO improved, respectively.[27] Furthermore, sensors with grain sizes less than twice the thickness of the surface charge layer were significantly more sensitive to the gases tested.[44,45] Shape, morphology and surface area are also critical parameters for sensitivity enhancement of MO-based gas sensors, since they determine the number of atoms located at the edges of the nanocrystal structure.[45,46] Pristine WO_3 nanotubes (NTs) have a high and selective response to 5 ppm NO at 90% RH and 350 °C, and a small response towards toluene at the same concentration, whereas Pt- and Pd-loaded WO_3 NT sensors have the opposite trend of responses to NO and toluene at 400 °C (Fig. 4).[47] In order to increase the surface area to increase reaction sites, methods such as electrospinning are used for the generation of thin films of porous structures.[45,50–52] Jin *et al.*[46] compared the influence of various morphologies, synthesis routes, surface area and operating temperature on the performance of SnO_2 MO-based sensors to ethanol exposure.[46] In brief, SnO_2 MO-based sensors with a large surface area gave stronger responses, whereas the operating temperature and morphology had less influence on their sensing performance.

The use of MO-based gas sensors is limited by the ability to sense the gaseous phase of oxidizing or reducing species that can be both polar and nonpolar compounds. Such sensors are usually restricted for selective detection of a single gas of interest. The humidity dependence of the sensing properties emphasizes the biggest challenge for integration of sensors into real-world diagnostics, especially for exhaled breath as it contains at least 80% relative humidity (RH) under ambient conditions. Water adsorption reduces the sensitivity of MOs due to the prevention of electron donation to the surface charge layer, mostly for n-type MOs.[46,48,53] The dependence mechanism might be explained by 2 models that describe the interaction between water vapor and oxygen found on the MO surface, as detailed elsewhere.[54,55] Further investigation of these mechanisms will be critical for obtaining the underlying knowledge of how to generate humidity independence. Continuous exposure to humidity leads to the gradual formation of surface hydroxyls, leading to a progressive degradation that can be reversed by heating to temperatures >400 °C.[45,56] Addition of surface agents with high affinity to water assists in trapping the water vapor and reduces the interaction with the sensing layer. CuO,[48] NiO,[57] Pd,[58] Sb[59] and CeO_2[60] are potential absorbents to be integrated into MO sensors. Fig. 5 presents responses of MOs based on Ce–In_2O_3 to acetone at different RH levels.[60] Optimization of the operating temperature must be balanced between the elimination of the humidity

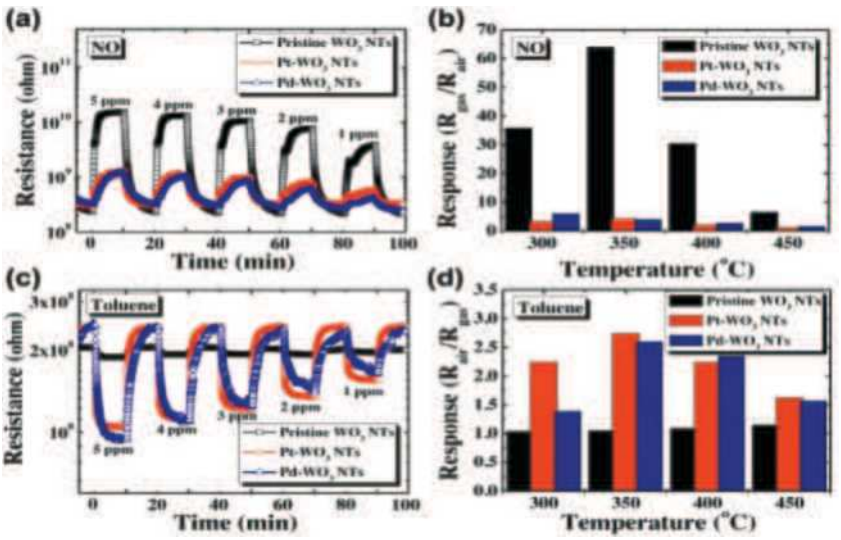

Fig. 4 (a) Dynamic response of pristine WO_3 NTs, Pt–WO_3 NTs, and Pd–WO_3NTs to NO in the range of 1–5 ppm at 350 °C, (b) temperature dependent NO response characteristic at 5 ppm in the range of 300–450 °C, (c) dynamic resistance transients of pristine WO_3 NTs, Pt–WO_3 NTs, and Pd–WO_3 NTs to toluene in the range of 1–5 ppm at 400 °C, (d) temperature dependent toluene response characteristic at 5 ppm in the range of 300–450 °C. Reprinted from ref. 47 with permission from Elsevier, copyright 2016.

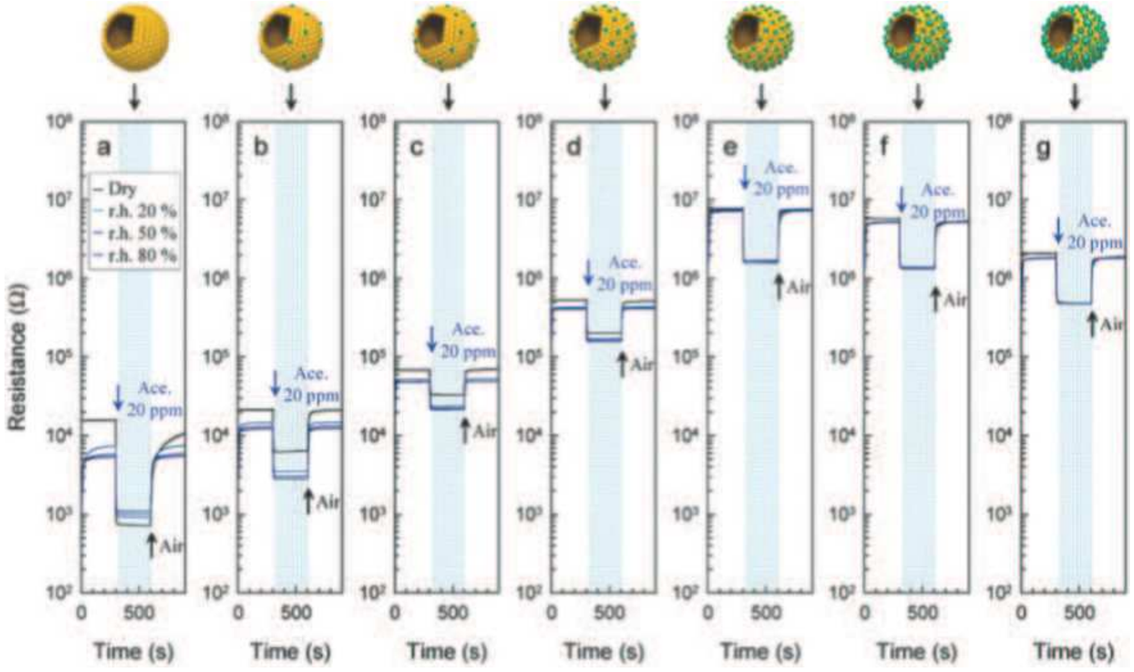

Fig. 5 Gas sensing transients of (a) pure, (b) 1.04, (c) 2.33, (d) 4.97, (e) 11.7, (f), 22.4, and (g) 39.9 Ce–In_2O_3 hollow spheres to 20 ppm acetone at 450 °C under dry and humid conditions (RH = 20, 50, and 80%). Reprinted from ref. 60 with permission from Wiley-VCH, copyright 2016.

Published on 11 June 2018. Downloaded on 2/1/2025 6:21:23 AM.

effects, magnitude of response, and recovery time, to the gas being investigated. Due to elevated operation temperatures, MOs – in comparison to other gas sensors – suffer from an inability to attach organic molecules or receptors to the sensor's surface in order to increase the cross-reactive selectivity to the gaseous phase.[36] High operation temperature is a serious obstacle for room-temperature applications and for portable applications, although it has the advantage of rapid reversible recovery.[27,36] However, detection of different gases has been possible by shifting the operation temperature.[43,47,61,62] Even though some detrimental performance issues of MO sensors remain, due to wide investment of effort in tailoring the sensing behavior of MO sensors, they have been reported in the literature for successful detection and quantification of several single gases associated with potential diagnostic applications. It should be noted, however, that most of these studies were conducted in a dry atmosphere that does not mimic real-world conditions in which detection of health associated gaseous biomarkers is required. Furthermore, almost all the reports on sensors based on MOs for gas detection refer to a rigid substrate and only a few studies have presented flexible sensors for gas sensing purposes.[62,63] To provide more specific details on the application of MO sensors for detection of VOC biomarkers, a general summary follows:

4.1.1. Nitric oxide detection. Nitric oxide (NO) is associated with asthmatic disorders, and is now being considered a potential diagnostic biomarker in exhaled breath.[64,65] According to the literature, asthma patients exhale above 30 ppb NO, whereas healthy population exhales lower concentrations.[65] Several studies have evaluated the performance of MO gas sensors regarding either NO or NO_2, their response to both gases being similar. Moon *et al.*[66] found high responses to 200–1000 ppb NO at 80% RH with villi-like WO_3 nanostructures at 200 °C. This sensor had high selectivity for the NO_x of interest by its high sensitivity to NO and NO_2 with a detection limit of 88 ppt, whereas it responds negligibly to ethanol, ammonia, acetone and carbon monoxide.

4.1.2. Ammonia detection. Ammonia is a potential biomarker in exhaled breath for renal, liver diseases and *Helicobacter pylori* infection.[67–70] For end-stage renal disease patients, the concentration of NH_3 ranges from 820 to 14 700 ppb, being lower for healthy volunteers.[68] The majority of the studies reported high and selective responses under dry atmospheric conditions to ammonia at from 50 ppb to 4000 ppm compared to other gases at 200–500 °C operating temperatures.[62,71–77] The majority of these sensors are based on MoO_3[62,71–73,77] and WO_3 with different doping,[74–76] which are acidic oxides. Thus, acid–base interactions may explain the high NH_3 responses as this is a basic gas. Güntner *et al.*[78] reported on the operation of Si-doped α-MoO_3 at 90% relative humidity that gave a selective response to 400 ppb NH_3 at 400 °C.

4.1.3. Acetone detection. Acetone is considered a potential biomarker for monitoring diabetes,[79–82] with 1.8 ppm given as the lower concentration of acetone for diabetes patients, whereas the concentration <0.8 ppm was reported for healthy people.[82] MOs have been investigated, to a large degree, for the selective detection of acetone in high humid atmospheres (>80% RH).[52,60,83–97] The majority were based on catalyst-loaded WO_3[83–88,91,98] and SnO_2[52,89] for 0.1–5 ppm acetone concentrations at 250–450 °C. Righettoni *et al.*[86] reported at least a 40% difference in 10 mol% Si-doped WO_3-based sensor response between correlated acetone concentrations of healthy

Published on 11 June 2018. Downloaded on 2/1/2025 6:21:23 AM.

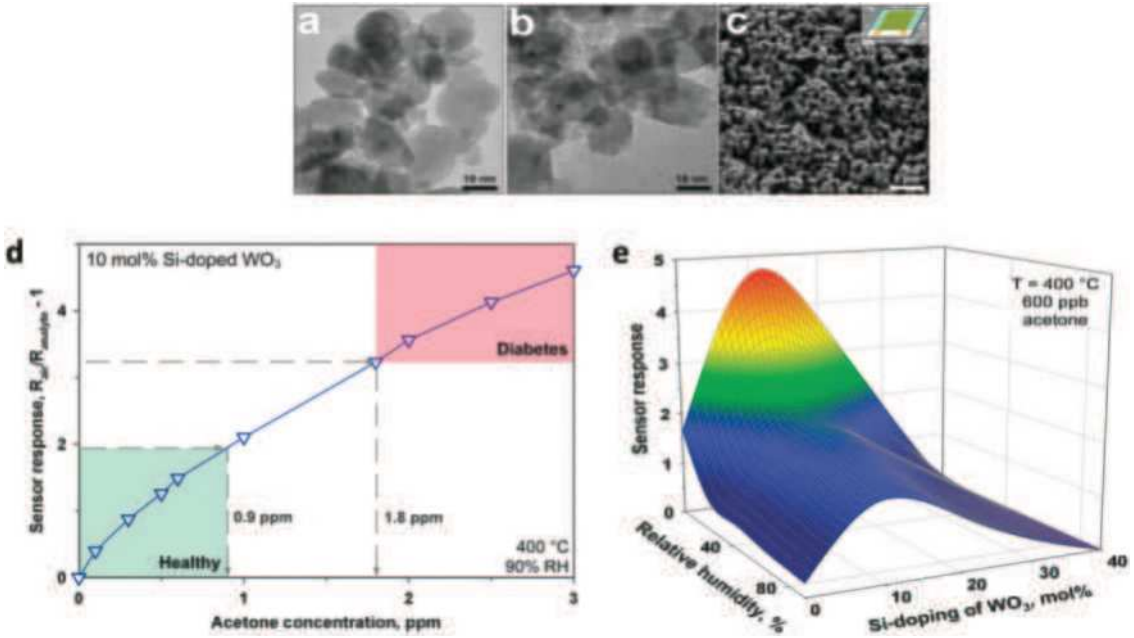

Fig. 6 TEM images of flame-made (a) pure and (b) 10 mol% Si-doped WO_3 nanoparticles collected downstream of the sensor substrate. (c) SEM image of a pure WO_3 film directly deposited onto the sensor substrate after *in situ* annealing. (d) Sensor response for acetone detection (0–3 ppm) of a 10 mol% Si-doped WO_3 sensor at 90% RH and 400 °C. Diabetic patients (1.8 ppm) can be clearly distinguished from healthy humans (0.9 ppm) by at least a 40% difference in sensor response. (e) WO_3-Based sensor response to 600 ppb acetone at different RHs and doping levels as a function of Si-content (400 °C). Reprinted with permission from ref. 86. Copyright (2010) American Chemical Society.

and diabetes subjects (Fig. 6). In a follow-up study, they assessed the performance of the sensors on 5 healthy volunteers, in whom they were able to detect acetone levels of 880–970 ppb.[93] Choi *et al.*[83] explored long-term stability tests of Pt-functionalized WO_3 hemitubes, which, after 7 months in air, showed a decrease of 6% of the response to 2 ppm acetone at 300 °C. Functionalized WO_3 hemitubes were used to sense acetone down to a detection limit of 120 ppb at 300 °C operating temperature.[94] In 2 newer studies, the same group used the same semiconductor material to prepare metal functionalized mesoporous WO_3 nanofibers.[84,85] A catalytic nanoparticle was encapsulated into apoferritin. The functionalized proteins were then incorporated into the nanofibers to achieve a sensing layer. They estimated that the complex detection limit of acetone was 0.54 ppb by linear approximation of the data.[85] The same concept was also investigated using SnO_2 nanotubes that achieved a 10 ppb detection limit.[52] Yet the latter sensor studies were only evaluated on pure standards, not in real-world breath samples, and their efficiency must be proven in the clinical scene. The most recent studies have explored Pd-loaded ZnO/$ZnCo_2O_4$ hollow spheres, giving 400 ppb detection;[95] RuO_2-loaded WO_3 nanofibers with 100 ppb acetone detection;[91] Rh_2O_3-loaded WO_3 nanofibers giving 1–5 ppm,[96] and PdO-loaded Co_3O_4 hollow nanocages with a calculated detection limit of 100 ppb.[92] Sensors based on a single Fe_2O_3 nanowire evaluated for acetone detection achieved a 20 ppb detection limit for a nanowire of 25 nm diameter.[97] However, it is noteworthy that many MO devices fabricated for acetone sensing are similarly sensitive to ethanol, which can hamper clinical breath applications.[99] Recently, hollow In_2O_3 spheres decorated with CeO_2-based sensors showed excellent very similar responses to 20 ppm of acetone under both dry and 80% RH conditions.[60] Nevertheless, humidity remains a huge challenge for sensing acetone concentrations under real-life conditions.

4.1.4. Ethanol detection. Similar responses were obtained from most of the MO sensors after exposure to acetone and ethanol,[100,101] due to a similar intermediate product during oxidative sensing reactions of both compounds.[102] Selectivity enhancement towards ethanol can be reached by adding a basic metal oxide, such as La_2O_3, to the sensor structure.[102–104] On the other hand, acidic oxides are advantageous for increasing sensor selectivity to acetone. Selective detection of 10–500 ppm ethanol, mainly by α-Fe_2O_3-based sensors, has been explored[103,105–107] under dry conditions and at temperatures from 240 to 350 °C. Shifting the operational temperature is another possibility for increasing the sensitivity to both acetone and ethanol, even in the presence of high humidity.[53] A typical example to detect ethanol is by using ZnO nanorods and graphene hybrid architectures on a flexible substrate of stainless steel foils.[63] Besides a desirable sensing response to 10–50 ppm ethanol at 300 °C in dry air, stability was particularly emphasized for this sensor. For instance, multiple (up to 100 times) bending tests showed that this hybrid architecture allowed deformation without electrical or mechanical failure for bending radius of $<$0.8 cm.

4.1.5. Hydrogen sulfide detection. Detection of H_2S is potentially important for diagnosis of halitosis.[108,109] CuO-,[43,110–121] MoO_3-[62,122–124] and Ag-[125–128] based oxide sensors are most commonly used for such sensing application under dry or moderately humid conditions. Many of these sensors can be operated at low operating temperatures, even down to

Published on 11 June 2018. Downloaded on 2/1/2025 6:21:23 AM.

room temperature.[110,111,114,122] CuO hollow spheres with hierarchical pores[112] and CuO nanosheets[113] were investigated for selective detection of 30–1200 ppb H_2S at higher operating temperatures (190–240 °C) with a short response time and strong recovery abilities. A detection limit of 2 ppb was quoted in both studies.[112,113] However, it was reported that a 3-month stability test of gas sensors based on CuO nanosheets yielded a constant response.[113] Continuous exposure to H_2S at low temperature might lead to the problem of modifying CuO to CuS; hence CuO-based sensor's recovery at low operating temperatures was obtained by pulse heating[114] to reverse the oxidation of CuO to CuS, whereas this action was unnecessary at high temperature.[114,115] In addition to the high sensitivity and low detection limit, Choi *et al.*[48] reported that CuO-loaded hollow SnO_2 spheres had excellent selective responses to 0.2–1 ppm H_2S, with negligible dependence on the high humidity level (80% RH) at 300 °C. In contrast to CuO-based sensors, MoO_3 is a nonreactive adsorption compound, such as $MoO_3{\cdot}SH_2$, which has high reversibility at 70–300 °C.[62,122,129] Li *et al.*[62] reported on a flexible MoO_3 nanopaper-based sensor for the detection of several gases, including H_2S. When operated under dry conditions and at 250 °C, the sensor had a high selective response to 0.25–5 ppm H_2S. The sensor became more selective towards NH_3 after altering the operational temperature.

4.1.6. Detection of benzene ring-based (aromatic) VOCs. Detection of less-reactive long chains (C_4–C_{10}) or benzene ring-based VOCs, related to different diseases, by a single selective MO-based sensor continues to pose many challenges. Some progress towards detection of benzene ring VOCs was achieved with p-type MOs of high catalytic activities; however, this was only under dry conditions.[61,130–141] High selectivity was found with a Pd-loaded SnO_2 yolk–shell sensing film coated with a thin catalytic Co_3O_4 overlayer by exposing to benzene and other indoor pollutants, such as toluene, xylene and ethanol.[130] The sensor responded quickly to 5 ppm benzene and had negligibly low cross-responses to other indoor pollutants at 375–475 °C and dry air. Uncoated and coated (with a thin zeolitic imidazolate framework; ZIF-8) molecular sieve membrane ZnO nanowires were examined as gas sensors for detecting 10–30 ppm H_2, toluene and benzene. The uncoated ZnO nanowires were markedly sensitive to toluene and benzene, whereas the coated sensors showed the highest selectivity to H_2.[142]

4.1.7. Metal oxide-based nanoarrays. MO-Based nanoarrays are useful for detecting gaseous mixtures with different properties, like real VOC patterns, which are associated with a variety of diseases.[143–145] For example, 4 Pt-, Si-, Pd-, and Ti-doped SnO_2 films were used for detecting formaldehyde in a gaseous mixture of acetone, ethanol and NH_3 with 90% RH, mimicking real human breath. The detection limit of the array was 3 ppb formaldehyde at 400 °C, with low response times. A single sensor cannot detect and quantify formaldehyde at low concentration (0–180 ppb) in a humid gas mixture. Thus, the formaldehyde range of the 4 sensors was estimated accurately by multivariate linear regression, with an average error of ≤9 ppb in up to 4-analyte mixtures (Fig. 7).[143] Moon *et al.*[145] described a nanoarray of 9 MO-based sensors, including thin films, Au-functionalized thin films and villi-like nanostructures. The nanoarray was exposed to different gases at 80% RH. The array had enhanced sensitivity in the detection of H_2S, NH_3 and NO, with detection ranges of 534 ppt–2.87 ppb, 44542.29 ppb, and 206 ppt–2.06 ppb, respectively. Principal component analysis (PCA) strengthened the discriminatory abilities of the nanoarray.[145]

4.2. Chemiresistors based on monolayer-capped metal nanoparticles

Chemiresistors based on monolayer-coated metal nanoparticles (MCMNPs) are widely used for gas sensing due to their valuable advantages, including: large surface-to-volume ratio, low-output impedance, room temperature operation, (sub-)ppb detection

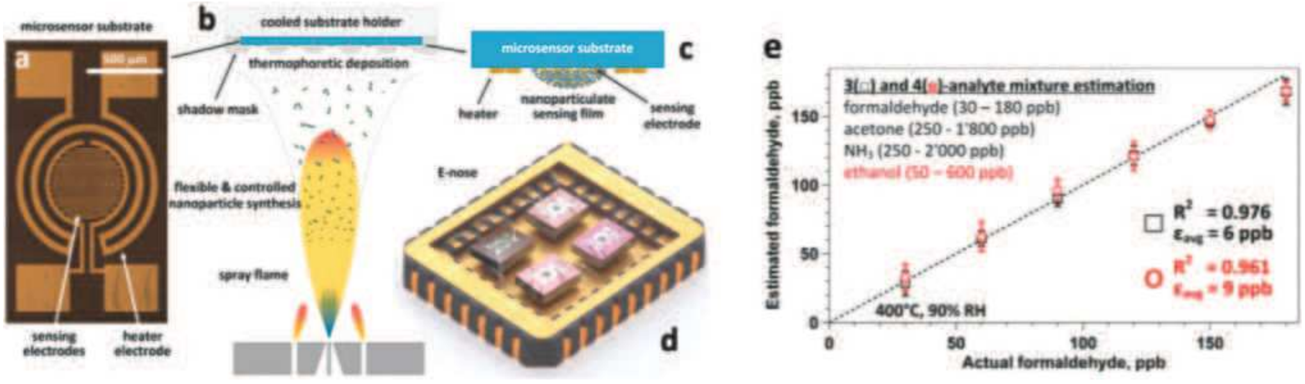

Fig. 7 (a) A microsensor substrate features a circular-shaped sensing area with a set of interdigitated Pt electrodes. This is surrounded by a meandered Pt electrode serving as a heater and a resistance temperature detector. (b and c) Flame-made sensing particles are accurately deposited by thermophoresis on such substrate packages (5 × 4 microsensors) using a shadow mask. (d) After package dicing, Pt:, Si:, Pd: and Ti:SnO_2 microsensors are combined as an array (E-nose) and wire-bonded on a chip carrier. Suspending the sensors minimizes thermal losses and ensures a uniform temperature distribution. (e) E-nose estimation of formaldehyde at 90% RH and in breath-relevant (a) 3- (squares) and (b) 4-analyte mixtures (circles) consisting of formaldehyde (30–180 ppb), acetone (50–1800 ppb), NH_3 (250–2000 ppb), and ethanol (50–600 ppb). Formaldehyde levels are estimated accurately with low error, ε_{avg} = 6 and 9 ppb, despite the significantly higher interferent gas concentrations. Symbols and error bars indicate the estimated average and the variability of different interferent levels. Reprinted with permission from ref. 143. Copyright (2016) American Chemical Society.

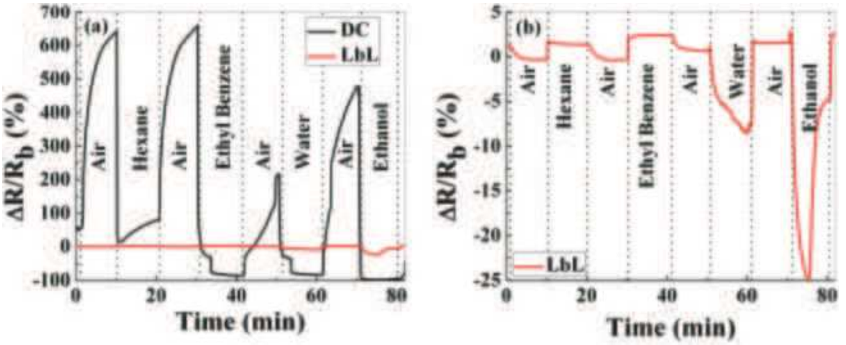

Fig. 8 Normalized resistance, $\Delta R/R_b$, of MNPs prepared by LbL and DC methods. Upon exposure, hexane, ethylbenzene, water and ethanol in the vapor phase at p_a/p_0 = 0.5. (a) Shows both responses on the same scale bar, whereas (b) shows only the response of the LbL device. Reprinted with permission from ref. 155. Copyright (2014) American Chemical Society.

Published on 11 June 2018. Downloaded on 2/1/2025 6:21:23 AM.

limits for the VOCs of interest, low voltage operation, a wide dynamic range, fast response and recovery times, tolerance to humidity, the possibility of being deposited on both solid state and flexible substrates, and compatibility with the standard microelectronic industry.[14,146–151] There are many possible fabrication methods for both solid-state and flexible sensors based on MCMNPs, including drop-casting,[152–155] layer-by-layer (LbL) deposition,[151,155–157] spraying,[158,159] spin-casting,[160] printing,[161–164] self-assembly methods (including drain to-deposit[165]) and others.[166–173] Selecting the fabrication method has a critical impact on the sensor properties. Bashouti *et al.*[155] compared the performance of sensors prepared by 2 fabrication methods: drop casting and LbL (Fig. 8). Using the LbL method, the film was more homogeneous, and gradually increased in thickness and conductivity compared to the film prepared by the drop-casting method.[155] The sensors were exposed to polar and nonpolar VOCs associated with health monitoring, including hexane, ethyl benzene, water and ethanol at p/p_0 = 0.5 (p and p_0 are the partial pressures of the VOC and the VOC vapor pressure, respectively). Interestingly, sensors prepared by the drop-casting method had a higher sensitivity compared to sensors prepared by the other method.[155] Sensors fabricated using LbL methods may be more applicable to high-pressure sensing, where stability and reproducibility are mandatory.[155]

Selection of the substrate properties, especially when a flexible substrate is involved, provides additional opportunities to custom-made sensors with the desired flexibility, stability and sensing performance.[154,156,174,175] Segev-Bar *et al.*[174] reported on the contradictory effect between thickness and load sensitivity of the flexible substrate owing to bending and stretching. Direct control of the chemical and physical properties of nanoparticles (NPs) during synthesis allows significant diversity, and accurate tailoring of the sensing properties, such as sensitivity, selectivity and response time to the desired application.[176] Among these parameters are inorganic particle type (*i.e.* pure metals, metal alloys, semiconducting materials),[177–179] particle shape (*i.e.* sphere, cube, star, rod),[146,180–184] and size (1–100 nm).[185] Such control achieves nearly uniform nano-pores in the composite films.[146,150,176,183,184] In addition, the sensing layer composition (*i.e.* alkylthiols, dialkyl disulfides, DNA, proteins, alkanethiols, and others) is critical, and numerous reports have been published on this topic.[150,176,186–189] Another critical factor is film morphology, which affects sensing properties. The effect of morphology can be divided into several action-based types:[148,190] (i) individual and/or clusters of MCMNPs located at a considerable distance from one another, and, as a result, contribute to resistance changes; (ii) island morphology that permits conductivity due to formation of a single dimensional percolation pathway and/or reduction of the island-to-island distance, and permittivity changes as a results of swelling; (iii) continuous 3-dimensional morphology with numerous percolation pathways,[148,190] and (iv) perforated films that involve 2 simultaneous mechanisms of (a) ionic conduction on the SiO_2 in the domains free from MCMNPs, and (b) tunneling conduction within the MCMNP domains.[190]

A change in the resistivity of the chemiresistor, during exposure to a vaporous phase, can be affected by one or a combination of the known mechanisms related to the activated tunneling model:[149,176] (i) a steric change within the nanomaterial film, such as reversible swelling or aggregation, causing the interparticle distance to increase or decrease, thereby affecting the electrons' tunneling properties,[17,25,36,149] and (ii) changes in the dielectric constant of the organic layer due to charge transfer from/to the layer once exposed to VOCs, especially if the dielectric constants of the sensing layer and the absorbed VOC differ significantly.[25,183,191,192] Exposure to absorbed VOCs with low dielectric constants (*e.g.*, toluene and *n*-hexane) results in decreased permittivity of the organic matrix, causing increased energy of tunneling activation, thereby leading to an increase in resistivity.[192] In a similar manner, VOCs with high dielectric constants reduce the sensor's resistivity. MCMNPs can be integrated into single variable sensors, such as chemiresistors, or into multivariable sensors, such as resonant or non-resonant multivariable electrical sensors and material- or structure-based multivariable photonic sensors. More details about multivariable sensors have been described elsewhere.[188]

4.2.1. Solid state sensors based on monolayer-capped metal nanoparticles. Many studies have been conducted to elucidate the sensing mechanism of solid-state chemiresistors based on MCMNPs.[152,166,193–196] García-Berríos *et al.*[193] investigated the

Published on 11 June 2018. Downloaded on 2/1/2025 6:21:23 AM.

response to various VOCs of sensors capped with a series of straight-chain alkanethiols, including 1-butanethiol, 1-pentanethiol, 1-hexanethiol and others. The VOCs explored that are associated with health conditions included hexane, heptane, *n*-octane, iso-octane, cyclohexane, toluene, ethyl acetate, methanol, ethanol, isopropanol and 1-butanol at room temperature and under dry conditions.[193] The sensors gave rapid and reversible responses, and the resistance response sensitivity to hydrocarbons increased with the length of the capping alkanethiols, whereas with alcohols, negative sensitivities were measured as the alkanethiol length increased.[193] For hydrocarbons and polar analytes, a dual response mechanism was observed, whereas the dielectric constant of the alcohol vapors was not weighty for the sensing mechanism compared to the steric effect.[193] A more comprehensive study was later undertaken by measuring the responses of sensors to 20 different capping ligands to 13 VOCs.[152] The capping ligands were divided into 4 groups: straight-chain alkanethiols (*i.e.* 1-butanethiol, 1-pentanethiol, 1-hexanethiol), branched alkanethiols (*i.e.* 2-methyl-1-propanethiol, 2-methyl-1-butanethiolm 3-methyl-1-butanethiol), aromatic thiols (*i.e.* 1-phenylthiol, 1-naphthalenethiol, 4-biphenylthiol) and composites of 2-phenylethanethiol-capped Au NPs with nonpolymeric aromatic materials (2-phenylethanethiol alone and with naphthalene, biphenyl, anthracene, or terphenyl).[152] The VOCs included the following compounds at different concentrations and dry conditions: hexane, heptane, *n*-octane, iso-octane, cyclohexane, toluene, chloroform, tetrahydrofuran, ethyl acetate, methanol, ethanol, isopropanol and 1-butanol.[152] As expected, the sensing responses for the straight-chain alkanethiol-capped Au-NPs affirmed the expected trends previously reported. According to the data for the branched alkanethiol-capped Au-NPs, the dual response mechanism was not shown, probably due to surface defects.[152] Nevertheless, as the chain length increased, the average resistance-based sensitivities also increased. Sensors with aromatic thiols had higher selectivity to polar vapors due to the π-stacked islands that allowed for voids to polar analyte sorption.[152] By applying PCA and Fisher's linear discriminant as pattern recognition algorithms, the sensor array gave an accurate classification of most vapors, except for those with similar polarity.[152]

As the MCMNP-based sensors are not specific and perform as cross-reactive sensors, each can be exposed to a variety of VOCs of interest in conjugation with pattern recognition algorithms distinguishing between them. Hubble *et al.*[153] screened 132 different capping ligands based on thiols as the sensing layer for Au NP-based chemiresistors for the purpose of discrimination and quantitation of benzene, toluene, ethylbenzene, *p*-xylene, and naphthalene (BTEXN) mixtures at ppm levels in water. The sensors were screened on their baseline resistance values and the relative response to the BTEXN mixtures at 5 ppm.[153] Combination of the sensor array data with random forests analysis also verified the predictive abilities for each individual component concentrations of 100 ppm in water in a mixture.[153] Joseph *et al.*[151] reported on the sensing performance of Pt- and Au-NPs functionalized with 1,9-nonanedithiol (NDT) and dodecylamine, to NH_3, CO, toluene and vapors of water in the range from 300 ppb to 5000 ppm. Increased resistivity was seen with all the sensors upon exposure to VOCs. The sensitivity of the Pt NPs film was 2 to 8 times higher than that of the Au NP film, and gave a detection limit for NH_3 of <100 ppb.[151] Furthermore, response dynamics indicated that NH_3 and CO bind significantly more strongly to the film compared to water and toluene.[151] In another study, 3.5 nm Au NPs functionalized with 4-methylbenzenethiol, 1-hexanethiol and 1-dodecanethiol were examined upon exposure to NO_2 gas and toluene vapors.[197] Detection limits for NO_2 were 0.5 ppm and 8.8 ppt toluene using a 4-methylbenzenethiol functionalized NP film at 22 °C.[197] Detection of CO_2 by NP-based chemiresistors was also reported.[198] The conductivity of the unlinked film increased by up to 31 fold under increasing CO_2 pressure (0–6.6 MPa) at room temperature, whereas it decreased with increasing CO_2 pressure for the linked films with different ligands.[198]

The influence of humidity was also investigated by exposing the sensors to realistic humidity levels mimicking those of actual breath and skin samples. Dovgolevsky *et al.*[183] examined the responses of cubic Pt NPs capped with oleylamine (ODA), 11-mercaptoundecanol, 11-mercaptoundecanoic acid, and benzylmercaptan to VOCs, including hexane, octane, decane, ethyl benzene, ethanol and water at p/p_0 = 0.01–0.5 under humid conditions. Functionalized NPs with nonpolar ligands had a high response to nonpolar VOCs and a low sensitivity to polar ones, especially water.[183] Moreover, sensing capabilities were shown for nonpolar analytes in high humidity environments by exposing an ODA-capped cubic Pt NP sensor to binary mixtures of low concentration octane and realistic humidity levels.[183] Single-stranded DNA-functionalized Au NP-based sensors have also been reported.[189] These sensors were exposed to ethanol, methanol, hexane, dimethyl methyl-phosphonate and toluene at p/p_0 = 0.036 concentration under different relative humidity conditions at room temperature.[189] Exposure to increasing level of humidity resulted in a non-monotonic resistance change that can be explained by the combined effects of hydration induced swelling and ionic conduction.[189] Strong influence of humidity was also observed upon exposures to humid vapors.[189] Homede *et al.*[163] reported a novel printing technique termed self-propelled anti-pinning ink droplet that involves evaporative deposition during the path of motion. By changing the applied actuating forces, different deposition line patterns, thicknesses and morphologies were generated from a single droplet.[163] The sensor array was based on Au NP-sensors capped with 1-hexanethiol, 1-dodecanethiol and 1-ocatadecanethiol prepared by either μ-revulet or spreading patterns.[163,199] The array was exposed to 0–90% RH and a variety of VOCs, including 1-octanol, 1-hexanol, ethanol, decane, hexane, octane, dibutylether and mesitylene at 100–1000 ppb for each. Pattern recognition by discriminant function analysis (DFA) showed a respectable discrimination between the VOCs at all tested RH levels, including the 90% RH level that mimics real breath and skin samples (Fig. 9).[163] Moreover, the results accurately quantified VOC concentrations.[163] Such results indicate the vast potential of the novel printed NPs as a cost-effective technique (*i.e.*, without the need for expensive printing equipment) for

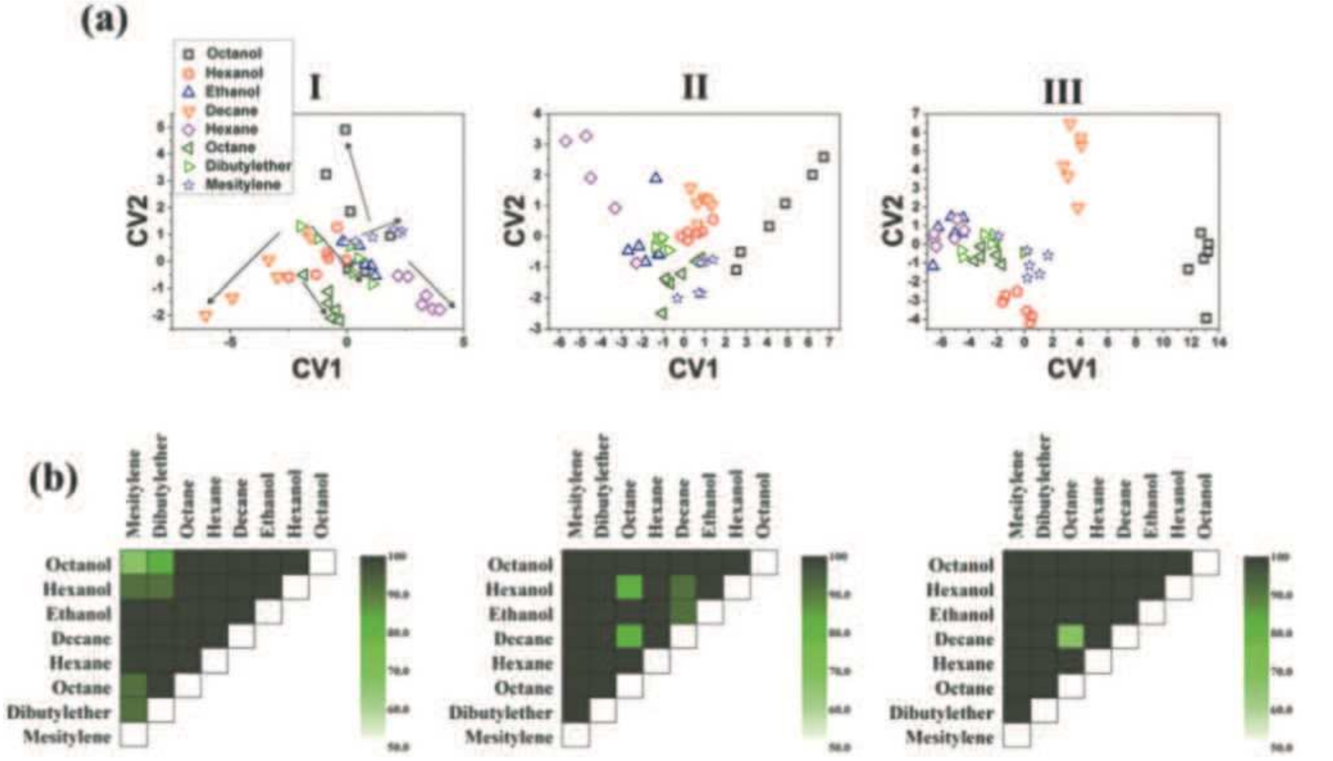

Fig. 9 (a) DFA plots showing the separation between the different VOCs achieved by the sensor array at the 3 humidity levels: (I) 0, (II) 30, and (III) 90%. The arrow directions in (I) indicates increasing concentration of each VOC. (b) Discrimination accuracy between each pair of VOCs achieved by the chemisensor array at the 3 different humidity levels, *i.e.*, (I)–(III) as above. Each square indicates the accuracy of separation between the VOCs from the *x*-axis and the *y*-axis. Reprinted from ref. 163 with permission from Wiley-VCH, copyright 2016.

Published on 11 June 2018. Downloaded on 2/1/2025 6:21:23 AM.

integration into multifunctional devices for health diagnosis and monitoring. Broza *et al.*[200] and Karban *et al.*[201] showed in their studies the capability of an array of MCMNPs in discriminating between simulants of multiple sclerosis (MS) and health, or between different bowel diseases, respectively.

4.2.2. Flexible sensors based on monolayer-capped metal nanoparticles. Chemiresistors based on 4 nm (in diameter) Au NPs capped with NDT and pentaerythritol tetrakis(3-mercapto-propionate) on a flexible high-density polyethylene substrate were tested for detection of toluene, water, 1-propanol and 4-methyl-2-pentanone at from 50 to 10 000 ppm.[157] The tested VOCs had similar vapor pressures, but different polarities.[192] The sensors had higher sensitivity (~30%) to these VOCs during a room temperature operation when 1% tensile strain was applied compared to the absence strain.[157] Shan *et al.*[202] investigated the sensing mechanism of Au NPs capped with decanethiol and NDT on flexible polyethylene terephthalate (PET) as a function of strain and its direction. The sensors were exposed to hexane (826–3300 ppm), ethanol (312–1300 ppm) and acetone (1300–5000 ppm) under ambient conditions and different radii of curvature of concave or convex strains.[202] Response sensitivity analysis showed that the relative changes in response to a particular vapor were ruled by its nature and the sorption, and less by the applied strain.[202] The influence of ligand length as well as the bending state was also elucidated for VOC sensing.[154] Flexible sensors based on Au NPs were capped with alkanethiols, including 1-butanthiol, 1-hexanethiol, 1-decanethiol and 1-octadecanethiol, and were deposited on a Kapton substrate.[154] These sensors were exposed to different VOCs at 3 bending states, including flat. The tested VOCs included compounds associated with health: cyclohexanol, octanol, decanol, mesitylene, octane and decane at increasing concentrations (p/p_0 = 0.01, 0.1, 0.5). Combination of all the bending states increased the discriminatory ability to separate different VOCs in PCA analysis compared to a single bending state.[154] The same report also included Au NPs capped with 1-hexanethiol sensors that were bent to radii of 0.21, 0.26 and 0.28 mm^{-1} as stretched and compressed deformations.[154] These sensors were exposed to VOCs, including hexane, octane, toluene, hexanal, octanol, water and 2-ethylhexanol, at static bending states. PCA analysis, based on the sensing properties, showed an excellent separation between polar and non-polar VOCs, and between the individual VOCs. Furthermore, analysis indicated an orthogonal classification based on the bending groups (Fig. 10). Such a conclusion highlights the ability of a single sensor with multi-parametric sensing to identify the deformation status and its directions even in complex environments as well as the VOCs.[154] Kahn *et al.*[156] tested Au NP-based sensors capped with 2-nitro-4-flouromethylbenzenethiol, 2-naphthalenethiol, 4-chlorobenzenemethanethiol, 3-ethoxy-thiophenol and 4-*tert*butylbenzenethiol on a flexible Kapton substrate. They were evaluated under the mutual effect of different strains, both dynamic and static, along with simulated VOC samples and real breath samples collected from ovarian cancer (OC) patients.

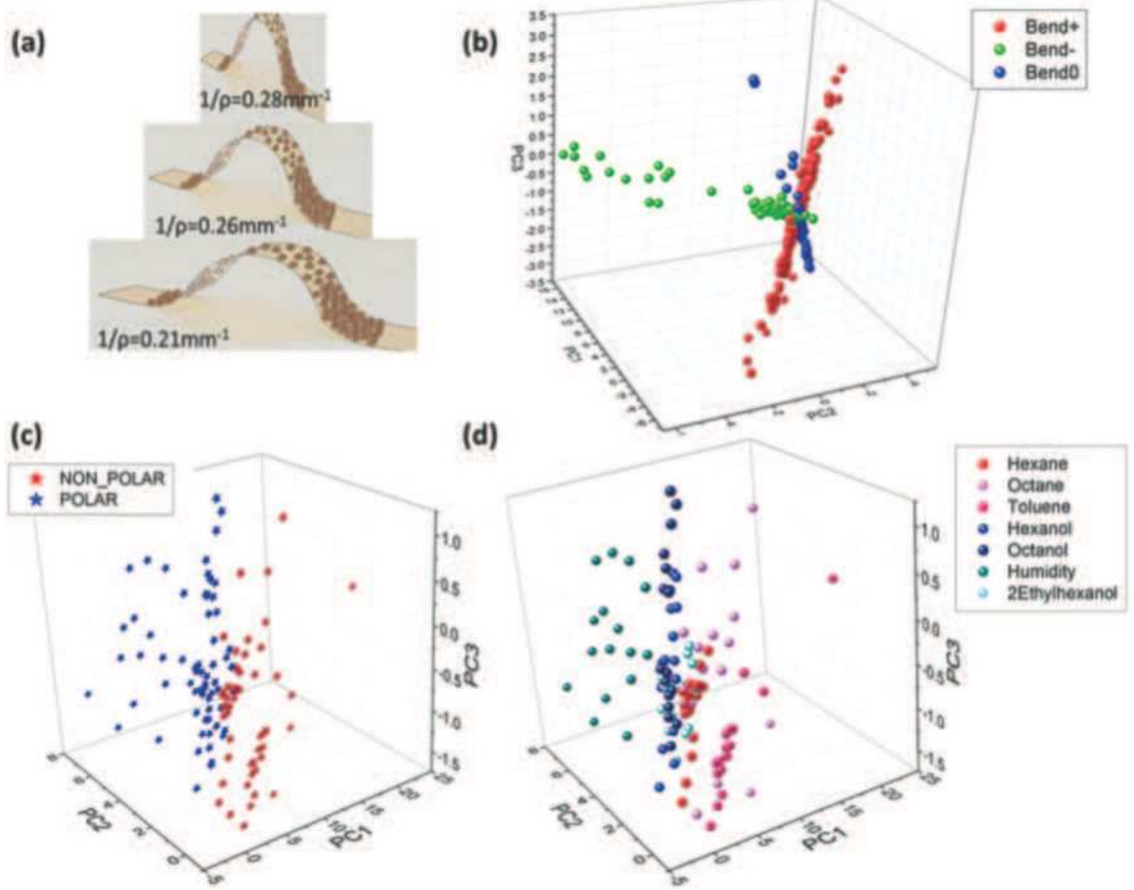

Fig. 10 (a) Illustration of Au-MCNPs deposited on a flexible substrate while been bent to bending radii of 0.21, 0.26 and 0.28 mm^{-1}. PCA plot based on 2 features extracted from the responses of Au-MCNP based sensors to 1-hexanethiol as the encapsulating ligand in response to 7 VOCs. (b) Orthogonal pattern displayed for stretching (Bend+), compressing (Bend−) and flat (Bend0) conditions of flexible sensors. (c) Polar *vs.* non-polar separation. (d) Specific VOC separation. Reprinted from ref. 154 with permission from Wiley-VCH, copyright 2017.

Published on 11 June 2018. Downloaded on 2/1/2025 6:21:23 AM.

The simulated VOC samples included both volatolomics linked with OC, such as styrene and nonanal, and possible confounding factors, such as ethanol and propioaniline at from 50 to 1000 ppb.[156,203] The results show that the VOC-dependent strain response can be sensitive to low concentrations – down to 50 ppb – thus showing the potential for diagnostic applications using exhaled breath.[156]

Paper-based sensors have also been reported.[164] Two nm Au NPs capped with 11-mercaptoundecanoic acid were printed by aerosol jet printing on a 2-layer paper membrane of polyacrylonitrile (PAN)/PET with Ag electrodes.[164] The paper-based sensor was exposed to ethanol at 200–800 ppm and 1-propanol at 50–275 ppm. In addition, methanol, benzene, acetone, hexane and 1-butanol were tested, but no data at the tested concentration were provided. The responses of the paper-based sensors were also compared to sensors based on a polyimide substrate, which is considered a 2D-nonporous substrate. The response sensitivity was higher, for almost all the tested VOCs, for the paper-based sensor compared to the polyimide-based sensor.[164] The sensor was also exposed to human breath and responded to inhalation and exhalation.[164]

Self-healable sensors based on Au NPs for detecting VOC in the skin and breath were also reported.[204] The sensor array included 3-ethoxythiophenol, benzylmercaptan, *tert*-dodecanethiol, hexanethiol, and decanethiol-functionalized NP films.[204] The flexible NP-based sensor was built with 3 layers: a self-healable polymer substrate from self-healing disulfide cross-linked polyurethane (sh-crl-PU),[205] a self-healable silver-polymer (μAg-polyurethane diol) composite (sh-μAgPU) and the sensing layer based on NP films.[204] All the tested VOCs were reported as being emitted from breath and the skin, thus highlighting their importance for healthcare and diagnostics. This list included 2-hexanone, acetic acid, heptane, hexanal, hexane, 3-heptanone, heptanoic acid, hexanoic acid, heptanal, nonanal and nonane.[204] The sensors had excellent healing properties at room temperature, had a fast self-healing within 3 h of being scratched.[204] In addition, these self-healing sensors gave ultra-detection (limit of detection ranged at the ppb level) and classification of health-related VOCs by being combined with pattern recognition methods, both before and after scratching on to the fully healed state (Fig. 11).[204] Nevertheless, all the tested VOCs were exposed under dry conditions, and the effect of humidity associated with real samples[205,206] remains to be evaluated.

The effect of humidity on sensor performance was also studied.[154] Segev-Bar *et al.*[154] reported sensing properties of

Published on 11 June 2018. Downloaded on 2/1/2025 6:21:23 AM.

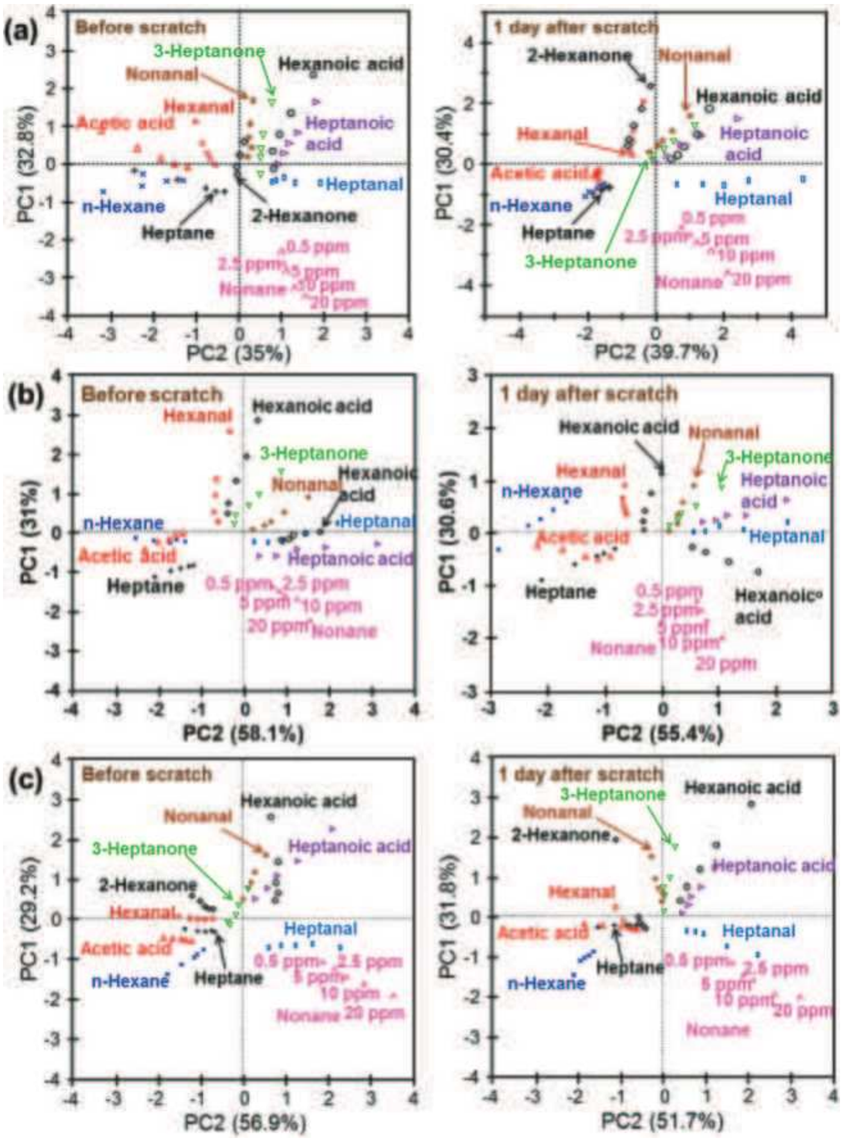

Fig. 11 PCA schemes of the data-set for the sensor array consisting of (a) 5 different sensor parts; (b) benzylmercaptan-, hexanethiol-, and decanethiol-functionalized GNP films; (c) *tert*-dodecanethiol-, hexanethiol-, and decanethiol-functionalized GNP films before scratching and after full healing. After manually scratching and complete healing, the sensor array retained its performance in sensing of VOCs within a satisfactory range. A desirable discrimination feature was also found after the sensor array had fully healed. Reprinted with permission from ref. 204. Copyright (2016) American Chemical Society.

Au NPs on a flexible Kapton substrate with (i) different capping layers including 1-hexanethiol, 1-decanethiol, 1-dodecanethiol, 1-octadecanethiol and 1-naphthalenethiol, and (ii) a set of Au NPs capped with 1-naphthalenethiol with different core diameters of 5, 10, 20 and 50 nm. Measurements were taken at a constant room temperature without applied strain and in the range from 5 to 30% RH.[154] Response times for all the sensors were <20 seconds and there was only a minor drift with time.[154] The results showed a negative response to the tested levels at these RH% for all the sensors. Furthermore, longer capping alkanethiol chains gave higher responses.[154] An additional observation regarding the influence of core diameter gave the highest negative response for the smaller core diameter. The change in response direction for 1-naphthalenethiol sensors with different Au NP diameters suggests the possibility of controlling the diameter to avoid any response to humidity. These findings are consistent with the changes in the dielectric properties of the film once exposed to water vapor with a significantly higher dielectric constant, and which decreases the activation energy for charge transport.[154]

4.3. Carbon nanotube-based sensors

Carbon nanotubes (CNTs) are another promising nanomaterials for a wide range of VOC detection applications. CNT fabrication methods include mostly laser ablation,[207–210] arc-discharge,[211–216] and chemical vapor deposition (CVD).[217–220] These nanomaterials can be divided into 2 groups according to their structure, single-walled (SWCNTs) and multi-walled carbon nanotubes (MWCNTs).

Published on 11 June 2018. Downloaded on 2/1/2025 6:21:23 AM.

CNTs attract the attention of many researchers especially due to their size range, large surface-area-to-volume ratio, electrical and mechanical properties, and their compatibility with other nanomaterials for the generation of enhanced properties. The size ranges 0.8–2 nm for SWCNTs, 5–100 nm for MWCNTs and the length of vertically aligned (VA) CNTs varies from microscale to milliscale.[221,222] MWCNTs have a metallic behavior with a current carrying capability of up to 10^9 A cm^{-2},[223] whereas the SWCNTs have either metallic or semiconducting behavior according to the chiral angle between the hexagons and tube axis.[224,225] Semiconducting CNTs have excellent electrical properties,[226] including high electron mobility (100 000 cm^2 V^{-1} s^{-1}), field effect mobility (79 000 cm^2 V^{-1} s^{-1}),[227] electrical conductivity (10^4 S cm^{-1})[228] and a band gap of ~5 eV with opposite correlation with the diameter.[229] The thermal conductance and thermal conductivity for metallic SWCNTs of 2.6 μm length and a diameter of 1.7 nm at room temperature are approximately 2.4 nW K^{-1} and 3500 W m^{-1} K^{-1}, respectively.[230] For a single tube, the thermal conductivity can reach 3000 W A^{-1} K^{-1}.[231] Both CNTs have excellent mechanical properties and the tensile strength ranges between 11 and 63 GPa for MWCNTs[232] and between 13 and 52 GPa for SWCNTs.[233] An outstanding tensile strength of 100 GPa has been recorded for both MWCNTs and SWCNTs.[234,235] Furthermore, the maximum bending strength for SWCNTs was 28.5 GPa, whereas the range for the MWCNTs was 14.2 to 80 GPa.[236] Not many studies focus on exposure to gas at real-world RH levels, and the present results based mainly on dry conditions. When exposed to water vapor, the conductivity of CNT-based sensors can be changed from p-type to n-type by humidity sorption as the water vapors act as electron donors.[237] Gas sensors based on pristine CNTs may have some limitations, such as low sensitivity due to low adsorption energy or low affinity of the tested gas, lack of selectivity or long recovery time. Functionalization of CNTs with various materials may enhance the sensing abilities and overcome these limitations; however, low detection of nonpolar VOCs and increased sensitivity to water are problematic and limit the detection of gases in breath samples. These major limitations can be explained by several effects.[238] Haick's group proposed that non-polymeric organic materials can act as coating layers that overcome these limitations and could be integrated into online devices for breath monitoring.[238–244]

Pristine and modified CNTs can be integrated into field-effect transistors[238,245–269] and chemiresistors[239–244,246,270–304] on a solid-state configuration with low fabrication costs and power consumption.[305–307]

4.3.1. Solid state carbon nanotube-based field effect transistors. Sensors based on CNTs were investigated as FET (CNTFET) devices for gas sensing applications in many studies,[238,245–268] mostly focused on 2 gases, NO_2 and NH_3, representing oxidizing and reducing gases, respectively. Kong *et al.*[249] reported CNTFETs based on a single SWCNT that was operated under room conditions for the detection of 2–200 ppm NO_2 and NH_3 at 0.1–1% in Ar/air. The sensors had a fast response, 2 to 10 seconds, and fast recovery only at operating temperature pulses of 200 °C.[249] A similar study was conducted with CNTFETs based on a single SWCNT including a polymethylmethacrylate (PMMA) resist as a center-covered configuration, or contact-covered configuration, also tested under ambient conditions.[250] The sensor was exposed to 0–4 ppm NO_2 and 0–400 ppm NH_3. As expected, V_{ON} increased for NO_2 and was reduced for NH_3 exposures.[249] The presence of a PMMA resistor influenced the subthreshold swing. For the center-covered configuration, exposure to NO_2 led to a lower subthreshold swing, but a higher subthreshold swing when exposed to NH_3. Opposite trends were observed for the contact-covered configuration.[250] Sorption of gas molecules on the CNTs/metal interface has also been reported, which mostly affects the characteristics, consistent with the Schottky barrier transistor model on exposure to 0–10 000 ppm NH_3 and 0–200 ppm NO_2 at room temperature.[251–253]

CNTFET based on multi-CWCNTs were reported as room operated sensors for detecting NO_2,[254–257] NH_3,[254,256,257] ethanol,[254] benzene[254] and NO.[257] Chang *et al.*[254] found CNTFETs sensitive to NO_2 at 200–1500 ppm, NH_3 at 30–500 ppm, ethanol at 1000 ppm and benzene at 15 ppm. As expected, the threshold voltage was shifted to the negative gate voltage for reducing gases, such as NH_3, ethanol and benzene, whereas a positive shift occurred on exposure to NO_2. Memory effects were also measured in different atmospheres (Fig. 12).[254] Recovery of the device was achieved by simply an inverse gate voltage for 30 seconds, a negative voltage for recovery from NO_2 exposure and a positive voltage for NH_3 recovery. In this manner, no degradation of the device can occur.[254] A poly(9,9-dioctylfluorene) derivative-based semiconducting-SWCNTs were used as the sensing layer for printed thin-film transistors (TFTs) for detecting NO_2.[255] Printed SWCNT TFTs had superior electrical performance with high on/off ratios (~10^6), highly effective hole mobility (~29.8 cm^2 V^{-1} s^{-1}), small hysteresis, and a small subthreshold swing (142–163 mV dec^{-1}) at a low operating voltage of ±2 V.[255] Furthermore, the sensor was exposed to 40–60 ppm NO_2 at room temperature. The sensing responses were fast (30 seconds), with high sensitivity and good stability. A fast recovery time of 30 seconds was also achieved by integrating a heating resistor applied at 2.5 V on the back of the gas sensor.[255] Similarly, a heating element was integrated into an array of 12 SWCNT-based CNTFET sensors exposed to NO_2 and NH_3 concentrations of 0.4–10 ppm and 4–50 ppm, respectively.[256] Chen *et al.*[257] found an enhanced detection limit of CNTFETs by using ultraviolet illumination at room temperature. Detection limits of 1.51 ± 0.32 ppt, 27.8 ± 6.2 ppt, and 590 ± 120 ppq (parts-per-quadrillion) for NO_2, NH_3 and NO, respectively, were reported.[257] Moreover, by controlling the gate bias, depending on its gate-dependent *I–V* characteristic profile, it can determine the sensitivity of the sensor to the gas being tested.[257] Although the sensitivity of Au/SWCNT to H_2S was strongly dependent upon the size and number of gold nanoparticles, the sensing mechanism was independent of these parameters.

Humidity effects on CNTFET that mimics real world conditions have also been investigated.[258–261] Kim *et al.*[259] reported on the humidity effects on CNTFET devices. Water molecules may shift the CNTFET's transfer characteristics as a function of gate voltage in terms of the hysteresis effect. The mechanisms for water trapping proposed were: (i) water molecules are

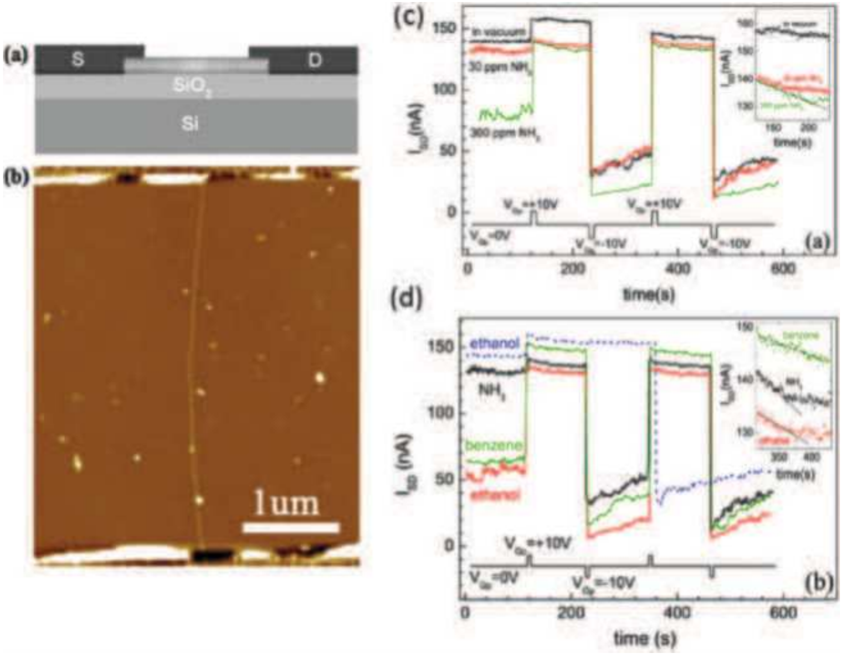

Fig. 12 (a) Schematic diagram of the CNT-based gas sensor. A highly doped Si substrate served as a back gate. (b) AFM image of the sensor used in these experiments. Non-volatile memory effects measured in different atmospheres. (c) Vacuum, 30 ppm NH_3, 300 ppm NH_3; (d) 30 ppm NH_3, 1000 ppm ethanol, 15 ppm benzene I_{SD} is measured as a function of time with V_{SD} = 50 mV and V_G = 0 V, when V_{Gp} = 10 or −10 V is alternately applied. The dashed line in (b) shows I_{SD} in 1000 ppm ethanol obtained from a different device. The insets show the temporal response from t = 120 to 240 s after applying V_{Gp} = 10 V under different conditions. Reprinted from ref. 254 with permission from IOP Publishing, copyright 2007.

Published on 11 June 2018. Downloaded on 2/1/2025 6:21:23 AM.

weakly adsorbed onto the CNT surface and can easily be removed by applying a vacuum, and (ii) bonds creation between the water molecules and the SiO_2 dielectric surface, proximal to the nanotube location. For eliminating undesired hysteresis, passivation of the devices with polymers that create hydrogen bonds with silanol groups, such as PMMA or octadecyltrichlorosilane, may be a solution.[259,260]

CNTFETs functionalized with other elements, such as metals,[262–264] polymers[265–267,269] and polycyclic aromatic hydrocarbons (PAH),[239] were also investigated for enhanced sensing performance. CNTFETs decorated with metal NPs were reported for the detection of several gases and VOCs, including H_2S at 500 ppm, NH_3 at 200 ppm and NO_2 at 5 ppm at room temperature.[262] The authors explored 18 metallic NPs, including Mg, Pd, Pt, and Rh. A multivariate partial least-squares (PLS) regression model was also used to discriminate between the different gases. Mubeen *et al.*[263] reported a Au/SWCNT-based FET device for detecting H_2S at 2–1000 ppb. Au NPs act as nano-Schottky barriers and play a dominant part in hole mobility modulation by gas exposure.[263] A correlation between the work function of the individual metal and the magnitude of electron transfer into the SWNT valence band has been reported in a study that explored the sensing properties of Pt, Pd, Au, and Ag NP decoration on the SWCNTs to 10 ppm of NO gas.[264] Polymer/SWCNT-based CNTFET was also mentioned for increasing the sensitivity and selectivity to the tested gases.[265–267] An *et al.*[265] noted that modified CNTFETs with polypyrrole operated at room temperature for NO_2 detection at 3000 ppm with n-type behavior. Functionalization with polyethyleneimine (PEI) resulted in ultra-sensing of NO_2 down to 100 ppt and lack of sensitivity towards NH_3, whereas functionalization with Nafion blocked NO_2 and allowed selective sensing of NH_3 down to 500 ppm.[266] Recovery was achieved with UV light as in other studies.[257,266,268] The influence of UV illumination on the polymer layer was not assessed. Functionalization of CNTFETs with specific DNA sequences for improving the sensitivity of the sensor in room operation has been published.[267] After exposing the sensors to different vapors, including methanol and propionic acid, the authors found enhanced sensitivity compared to bare devices; this enhancement depended strongly on the DNA sequence used for a specific gas sensing.

Random networks of SWCNT FETs functionalized with tricosane ($C_{23}H_{48}$)[239] and pentadecane ($C_{15}H_{32}$)/dioctyl phthalate ($C_{24}H_{38}O_4$)[239] were evaluated for detecting nonpolar VOCs associated with lung cancer, namely decane and 1,2,4-trimethylbenzene at p/p_0 = 0.004 and room temperature.[238] The pristine CNTFET had p-type behavior, as well as the $C_{23}H_{48}$/SWCNT FET. For the $C_{15}H_{32}$/$C_{24}H_{38}O_4$/SWCNT sensor, n-type characteristics were revealed due to electron-rich functional groups.[238] In addition, hysteresis in the transfer characteristics was significantly different between the devices. This has a significant role under humid conditions; for $C_{23}H_{48}$/SWCNT FET, an extended hysteresis occurred, indicating increased sensitivity to water molecules, whereas almost no hysteresis was seen for $C_{15}H_{32}$/$C_{24}H_{38}O_4$/SWCNT FET and a decreased sensitivity to water molecules was expected.[238]

4.3.2. Solid state carbon nanotube-based chemiresistors. CNT-based chemiresistors have also been thoroughly investigated

Published on 11 June 2018. Downloaded on 2/1/2025 6:21:23 AM.

as gas sensors.[270–299] Pristine CNT-based chemiresistors were well characterized and exposed to several VOCs associated with health monitoring, including NO_2,[270–273] NH_3[274] and ethanol[275] at room temperature. Li *et al.*[270] obtained linear responses to 54–162 ppm NO_2 and a detection limit of 44 ppb. Very slow recovery rates, due to high bonding energy between CNTs and NO_2, were dramatically reduced by UV illumination. In addition, sensitivity variation was <6% for all of the devices tested.[270] Electrokinetic fabrication was also reported for unmodified VA-CNT-based sensors for controlling trapped amounts of CNTs.[271] Both MWCNTs and SWCNTs were fabricated and their initial conductance was proportional to the amount of trapped CNTs. The sensors were exposed to NO_2 at 1–10 ppm, had a slow response time (~50 minutes) and the relative conductance change of the sensors to NO_2 exposure increased proportionally with the initial conductance of the sensors. Furthermore, SWCNT-based sensors had 2–6 times higher normalized sensitivity than those that were MWCNT-based, probably due to the greater abundance of the semi-conducting tubes being correlated with the sensor's response.[271] A VA-CNT-based sensor with a response time of <1 min was exposed to 0.5–1000 ppm NH_3,[274] but no details about the temperature of operation and the recovery process were provided. A recent comparison between a SWCNT random network and a SWCNT aligned network was reported for NO_2 detection in the range of 0.5 to 20 ppm.[272] Detection limits were 125 ppt and 165 ppt for the SWCNT-random network and the SWCNT aligned network, respectively. A decrease in the sensor's detection limit upon reduction in the aligned network-based density also occurred. Furthermore, sensors based on random networks of SWCNTs achieved not only a higher detection limit, but a better signal-to-noise ratio compared to the other investigated fabrication methods.[272] In another study, VA-CNTs on a titanium/Au-coated glass plate were fabricated by electrophoresis and fissure formation techniques.[273] This sensor had high sensitivity to NH_3 and NO_2 up to 100 ppm, with short recovery time within 40 seconds when a voltage of 5 V was applied for <30 seconds.[273] Ethanol detection was achieved by a VA-MWCNT-based sensor with sensitivities of 0.18% to 1.67% for 50–800 ppm.[275]

For enhancement of sensing properties, CNTs modified with metal NPs,[276–286,301] MOs,[287–290,302–304] polymers[292–296,300,301] and other nanomaterials[238,240–244,297–299] have also been demonstrated. The stability and lifetime of the functionalized CNT-based sensors remain questionable, mainly due to the possibility of oxidation, which may degrade the sensing abilities.

Penza *et al.*[276,277] compared the sensing performances of VA-CNTs[276] and random networks of CNTs[277] based on metal functionalization and bare MWCNTs over an operation range of 20–250 °C. The sensors were exposed to individual gases[277] and mixtures[276] of gases, such as NH_3 and NO_2. A temperature of 150 °C proved to be optimal for detecting NO_2 and NH_3.[276,277] Metal/CNT-based sensors were more sensitive than unmodified sensors for 0.1–33 ppm NO_2 and 5–1000 ppm NH_3.[276,277] Furthermore, random networks of CNTs achieved higher sensitivity compared to VA-CNTs.[276,277] In order to increase the sensitivity, responses of the VA-CNT-based array were combined with PCA-based pattern recognition for clear separation of the responses to NO_2 and NH_3 from other compounds.[276] Full recovery was achieved with temperature pulses.[276] Several studies have explored the possibility of integrating different forms of CNTs as the basis of an acetone sensor used for potential diabetic diagnostics; however, none of them evaluated real-world breath samples. An array of 32 sensors based on nanotubes decorated with pristine metal (Pd, Au) and polymer was developed to selectively sense acetone and other gases at ppm levels.[301] Abdelhalim *et al.*[278] measured the sensing performance of an array with pristine and CNTs modified with Pd, Ag, Ti and Cr NPs and with different diameters to NH_3, ethanol, CO and CO_2 under ambient conditions. The optimal combination of the metallic NPs was 1.5 nm Au, 1.0 nm Cr and 0.2 nm Pd. Array responses were combined with PCA analysis for exposures to individual gases, as also to mixtures of 2 gases, NH_3/ethanol and NH_3/CO at different ratios in each mixture. PCA analysis showed that the response to a mixture of 2 gases lies in the plane between the data points of each gas separately.[278] Penza *et al.*[279] also reported on a room temperature operated sensor based on Ag/VA-MWCNTs with a selective response for 1 ppm NO_2. Ag NP functionalization was also successfully reported for detection of 2 ppm H_2S under ambient conditions, yet the recovery of the sensors has not been achieved due to the strong chemical interaction between H_2S and Ag NPs.[280] Benzene[281] and NO_2[281,282] detection at room temperature was achieved with MWCNTs decorated with different metal NPs. Modified sensors were exposed to 50–500 ppb benzene and 0.5–6.5 ppm NO_2. Selective detection of NO_2 and insensitivity towards other gases, such as benzene[281] and ethanol,[282] were accomplished with Au-MWCNTs. Functionalization with Rh, Pd and Ni NPs responded oppositely for both VOCs that were tested.[281] Full and rapid recovery was accomplished by temperature pulses.[281,282] Further reports on NO_2 detection by a Au/CNT layer have also been published.[283–286] VA-CNTs decorated with 6 nm Au NPs of different lengths were exposed to NO_2 0.5–1 ppm with 0 and 50% RH under operation at room temperature.[283] The optimal length for NO_2 sensing was 300 μm. When the RH was increased to 50%, all the sensors had increased responses regardless of the nanotube length. A heat-pulse of 150 °C was used for reducing the recovery time.[283] Au/MWCNTs were recently investigated for detecting 0.1–10 ppm NO_2, 5–1000 ppm NH_3 and 0.1–10 ppm H_2S over a temperature range of 100–200 °C.[284] The optimal operational temperature was 150 °C, and by controlling the gold loading, it was possible to control the sensing responses and the selectivity to different gases. The lowest Au content (0.3 at%) gave the highest response to NO_2, whereas MWCNTs functionalized with the highest Au content (1.1 at%) gave the highest responses to H_2S gas (Fig. 13). Furthermore, binary gas mixtures, NO_2/H_2S and NO_2/NH_3, were tested for evaluating the detection of NO_2 gas.[284] Charlier *et al.*[285] found that Au/MWCNTs with selective responses to 0.5–6.5 ppm NO_2 at room temperature, had a responsiveness up to 6% for 500 ppb, and 12% for 6.5 ppm NO_2. Nevertheless, 20 min were required to reach a steady state resistance value after gas injection. The response time can be reduced at a higher

 This journal is © The Royal Society of Chemistry 2018

Published on 11 June 2018. Downloaded on 2/1/2025 6:21:23 AM.

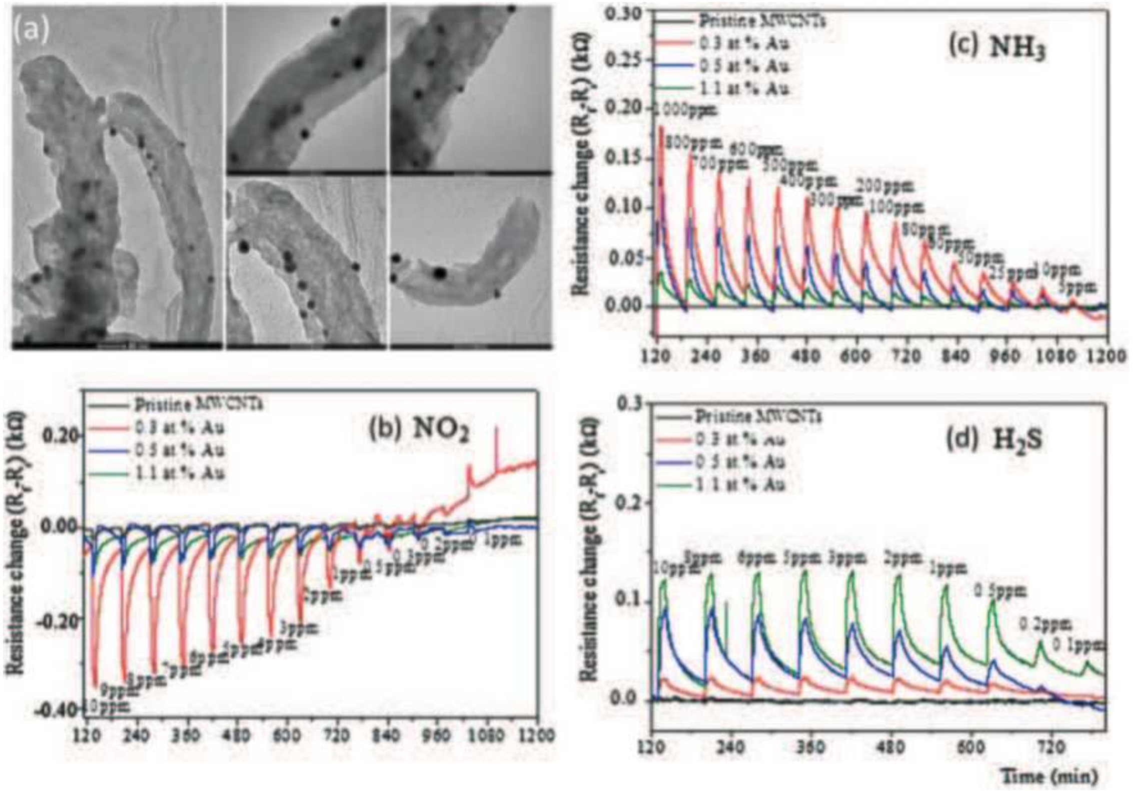

Fig. 13 (a) TEM images of MWCNTs decorated with Au NPs. Scale bar is 50 nm on the left image, and 20 nm for the other images. Time response of chemiresistors based on pristine MWCNT films, and functionalized CNT films with Au loading of 0.3, 0.5 and 1.1 at%, exposed to 10 min pulses of decreasing concentrations of (b) NO_2, (c) NH_3 and (d) H_2S at a sensor temperature of 150 °C. Reprinted from ref. 284 with permission from Elsevier, copyright 2016.

operational temperature, yet the sensitivity at 150 °C was not better than that at room temperature.[285] Random networks of 5 nm Au modification of the sidewalls of the CNTs, operated at an optimal temperature of 200 °C, were exposed to several gases, including NO_2 and NH_3, at 0.5–10 and 5–1000 ppm, respectively.[286] As expected, Au/CNTs gave a p-type response, namely an increase in resistance on exposure to reducing gases and a decrease in electrical resistance on exposure to NO_2.[286] MO nanomaterials are also excellent candidates for hybrid CNT sensors.[287–290,302–304] An antimony–CNT–tin oxide (Sb–CNT–SnO_2) thin film was fabricated and exposed to 100–1000 ppm of formaldehyde, ammonia, benzene and toluene.[287] This sensor had high sensitivity along with short recovery and response times compared to a bare CNT device. Furthermore, the electric field on the surface of the Sb–CNT–SnO_2 thin film was considered the reason for the rapid response to polar gases. Ethanol detection was reported by hybrid SWCNTs/Nb-Pt co-doped TiO_2 thin films.[288] Films with different SWCNT contents were exposed at a sequence of operating temperatures from 290 to 400 °C to 100–1000 ppm ethanol. The optimal content of SWCNTs was 0.01% by weight and the optimal operation temperate range lay between 290–320 °C. Detection of NO_2 was demonstrated by a hybrid of SnO_2/MWCNTs.[289,290] This sensor was exposed to 100–500 ppm NO_2 and had a high sensitivity, with total recovery after heating the chamber to 150 °C.[290] Xiong *et al.*[302] developed CNT cantilevers coated with WO_3 for detecting a single acetone molecule based on changes in resonant frequency. Fabricated multi-wall CNT–SnO_2 sensors enabled selective detection of ppm levels of acetone, and furthermore discriminating it from both ethanol and acetaldehyde.[303] Ding *et al.*[304] developed a photo-induced SWNT–TiO_2 core/shell hybrid nanostructure that had a detection limit of several ppm to acetone vapors at room temperature.

Polymer/CNT nanocomposites have also been reported for gas sensing applications.[291–296] A VA-CNTs and poly(vinyl acetate)/polyisoprene (PVAc/PI) binary polymer composite film sensor was reported as being sensitive to cyclohexane and ethanol at high concentrations.[291] Polyaniline (PANI)/MWCNTs were investigated for selective detection of NH_3 gas at 0.05–150 ppm.[292–294] A detection limit of 50 ppb was achieved, with a response time in the order of minutes and the recovery time of up to several hours.[293] Another study reported a rapid response (6–24 seconds) and recovery time (35–62 seconds) along with high responses for the nanocomposite-based sensor compared to unmodified MWCNTs.[294] Acetone detection was achieved by a PMMA/MWCNT-based sensor with a response time of 2–5 seconds at room temperature.[295] Poly(*m*-aminobenzene sulfonic acid) (PABS)/SWNT random networks were sensitive to NH_3, NO_2 and water vapor.[296] The sensor had excellent detection limits of 100 ppb for NH_3 and 20 ppb for NO_2. Adjustment of the initial resistance impacted on the sensitivity towards the gases tested. Upon exposure to RH levels of 10–70%, a response time of 2–3 min

Published on 11 June 2018. Downloaded on 2/1/2025 6:21:23 AM.

and full recovery time of <10 min were achieved after purging with dry air.[296]

Evans *et al.*[297] reported zeolite/SWCNT nanocomposites for 10 ppm NO_2 detection at room temperature and at different humidities related to real-world conditions. The choice of zeolites, which are known as molecular sieves, was due to their selective adsorption properties to targeted vapors, depending on their composition, hydrophilicity and loading. The results showed that zeolite/SWCNTs mixed as a layer configuration significantly reduced the sensor's responses to 25–75% RH. These findings show promise in that a composite of zeolite/SWCNTs can reduce the cross-sensitivity of the sensors to water vapor. Nevertheless, regeneration of the sensing layer after multiple exposures to water vapors required heat treatment at 150 °C.[297] An array of metalloporphyrin/SWCNTs has been reported for gas detection at several RH levels.[298] Several VOCs were tested, which included pentane, hexane, acetone, methanol, cyclohexane, ethanol, *p*-xylene and others at high ppm level concentrations. In combination with the PCA pattern recognition algorithm, the responses were accurately classified into 5 classes: alkanes, ketones, alcohols, aromatic hydrocarbons and amines. Furthermore, upon comparing the responses of $[Co^{III}(tpp)]ClO^{4-}$ and $[Cu^{II}(tpp)]$-based devices to VOCs at 2% RH in N_2 and 10% RH in air, there were no substantial effects on sensor responses.[298] Xie *et al.*[299] used VA-MWCNTs modified with amino groups for the selective detection of formaldehyde from 20 to 200 ppb at room temperature. The sensors had high selectivity and fast response times to formaldehyde, and a negligible response to methanol, ethanol, acetone, ammonia and carbon dioxide each at 200 ppb.[299]

Haick's group demonstrated an essential focus in the detection of nonpolar VOCs under real humid conditions.[238,240–244] An array of 10 random networks of SWCNTs coated with nonpolymeric organic materials was exposed to mixtures of VOCs found in the breath samples of lung cancer patients[239] and of rats suffering from chronic renal failure[240] at different RH levels. The nonpolymeric organic materials varied in their chain length, branching, aromatic configuration and functional groups.[239,240] Due to this diversity, more interactions between the absorbed VOCs and the sensing layer became possible, and a combination with pattern recognition algorithms could discriminate between similar alkane compounds and their concentrations.[308] At the first stage, the sensors were exposed to styrene, hexane, octane, trimethyl benzene and water at 0.01–100 ppm under ambient conditions.[239] Based on pattern recognition, the sensor array discriminated well between different polar and nonpolar VOCs when exposed to one VOC at a time, and showed clear concentration dependence.[239] Moreover, mixtures of VOCs, mimicking breath samples from healthy controls and lung cancer patients, were prepared at different RH levels: 1, 10 and 80%. PCA analysis showed a clearly opposite trend between the discriminative power and the RH level,[239] *i.e.* as the RH level decreased, the discriminative power increased. A preconcentration method for reducing RH 50 times resulted in excellent discrimination in a similar way to the simulated samples at 1% RH.[239] Similar results were obtained based on simulated samples of chronic renal failure, including phenol, undecane, 2-ethyl hexanol and acetic acid at 10 and 80% RH.[240] Zilberman *et al.*[241] reported on additional organic nanomaterials, discotic hexa-*peri*-hexabenzocoronene (HBC) derivatives, used as a capping layer for random networks of SWCNTs. Two different derivatives were explored, namely HBC-C_{12} and HBC-$C_{6,2}$, to both polar and nonpolar VOCs associated with lung cancer. The sensors were exposed to decane, octane, methanol and water at $p/p_0 = 1$. A clear increase in the response to the nonpolar and methanol VOCs was observed for both HBC derivative-based sensors compared to the pristine SWCNT sensor (Fig. 14). Functionalization with films of HBC-$C_{6,2}$ at much lower surface coverage compared to HBC-C_{12} improved sensing properties. The responses of pristine and HBC-C_{12}/SWCNT sensor to water exposure were identical within the margin of error.[241] In a further study, the sensitivity to different VOCs was investigated as a function of HBC-C_{12} coverage in the hybrid with SWCNTs.[242] Comprehensive works about arrays of SWCNTs covered with different PAH have been published.[243,244] These arrays were exposed to different polar and non-polar VOCs and water at a concentration range of $p/p_0 = 0.05–1$ and at different RH levels of 5 to 80%.[243,244] Such arrays gave 90.3%[243] and 97.2%[244] accuracy in discriminating between all the VOCs tested and water at 5% RH. For 80% RH conditions, 100%[243] and 88.9%[244] accuracies were achieved. These results demonstrate the potential of the PAH derivatives to critically affect the sensing properties by proper selection of the side-groups, concentration and the type of solvent.

4.3.3. Flexible carbon nanotube-based chemiresistors. CNT-based sensors are capable of withstanding high strains;[309,310] nevertheless, a serious limitation is to detect small strains or pressures.[310,311] Integration of CNTs into flexible sensors for gas sensing applications is well studied and many reports have been published, mostly on CNT-based chemiresistors.[312–329] A large series of flexible substrates have been investigated, including polydimethylsiloxane (PDMS),[312,313] plastic,[314–316] paper,[317,324] PET[318–322,324,327] and polyimide.[321,323,328] The majority focus on detection of NH_3,[314–316] H_2S,[316,318] NO,[316,319] NO_2,[316,319–324] other VOCs and water.[313,317,327,328] Rigoni *et al.*[314,315] detected NH_3 with sensors based on ITO/SWCNTs and pristine SWCNTs using flexible plastic substrates operating at room temperature. The ITO/SWCNT sensor had higher sensitivity towards concentrations >200 ppb, whereas similar responses were recorded for both sensors at lower concentrations. Detection limits of 13 and 3 ppb were estimated for ITO/SWCNTs and pristine SWCNT-based sensors, respectively. Upon exposure to 45–60% RH, opposite responses were recorded, and this behavior will allow, in the future, the combination of both sensors for discriminating the presence of water or ammonia under real-world conditions.[314,315] Fe_2O_3/SWCNT composite films on plastic substrates had high sensitivity to several gases, including 10–200 ppm NH_3, 1–100 ppm H_2S, 10–100 ppm NO and 10–100 ppm NO_2 at room temperature.[316] The changes in the responses of a Fe_2O_3/SWNT composite film gas sensor under different bending angles up to 180° after exposure to 20 ppm H_2S were negligible compared to a flat sensor. Asad *et al.*[318] reported a flexible and selective H_2S gas sensor based on CuNPs/SWCNTs on a PET substrate for detecting 5–100 ppm

 This journal is © The Royal Society of Chemistry 2018

Published on 11 June 2018. Downloaded on 2/1/2025 6:21:23 AM.

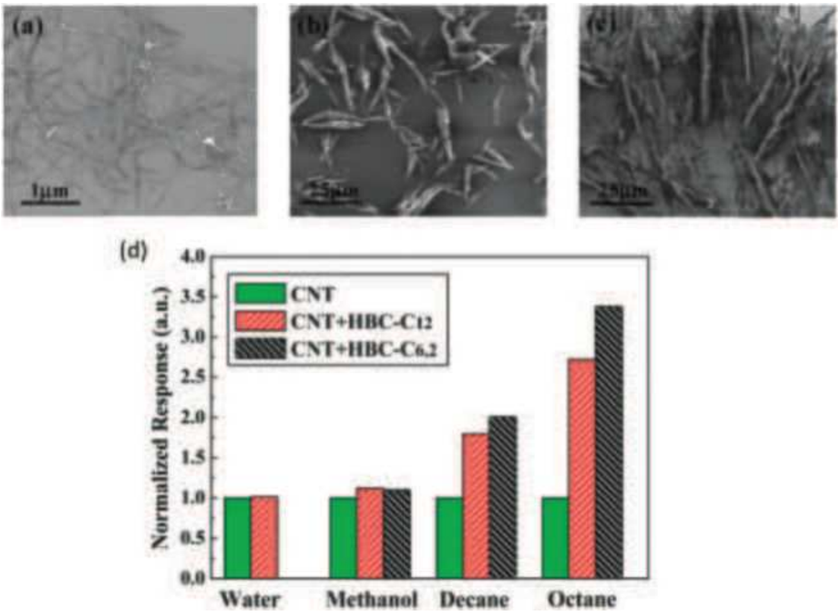

Fig. 14 SEM of (a) RN-CNT cast from dimethylformamide (DMF) solution; (b) HBC-$C_{6,2}$ structures cast from 10^{-3} M solution in xylene; and (c) HBC-C_{12} structures cast from 10^{-4} M solution in toluene. (d) Response of 2 kinds of HBC-functionalized RN-CNT sensors, normalized to the response of the corresponding pristine sensor, to water, methanol, decane, and octane at $p_a/p_0 = 1$. Reprinted with permission from ref. 241. Copyright (2009) American Chemical Society.

under both dry and humid conditions (40% RH) at room temperature (Fig. 15). A fast response and a recovery time of 10 and 15 seconds, respectively, were obtained for 5 ppm gas. A change of 5% in the response occurred upon bending up to 16 mm towards the gas compared to the flat position. Long-term evaluation of the sensor's performance to 5 ppm gas under ambient conditions resulted in a negligible change over a period of at least 30 days. Furthermore, the sensor was

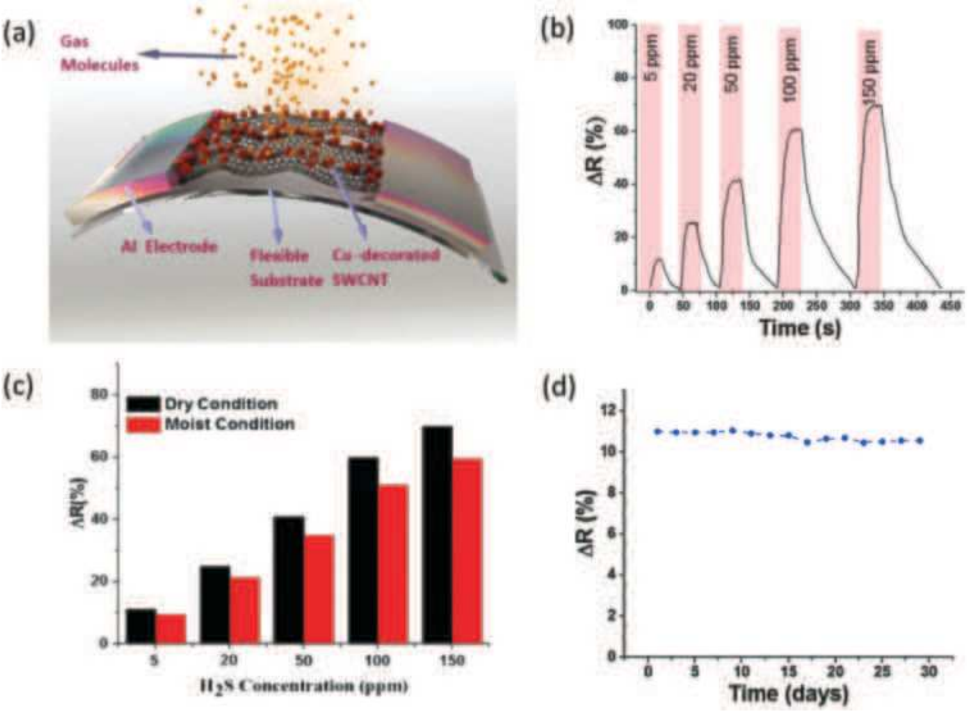

Fig. 15 (a) A schematic of the fabricated SWCNT-based gas sensors on a flexible substrate. (b) Sensor response to different H_2S gas concentrations at room temperature. (c) Final response of the Cu-SWCNT based flexible sensor at 40% relative humidity and in a dry environment at room temperature. (d) Long-term evaluation of the sensor's response to 5 ppm H_2S gas at room temperature. Reprinted from ref. 318 with permission from Elsevier, copyright 2015.

Published on 11 June 2018. Downloaded on 2/1/2025 6:21:23 AM.

exposed to the gas under both dry and 40% RH conditions, and its response decreased ~15% at 40% RH. WO_3/MWCNT hybrid[320] and reduced graphene oxide (RGO)/WO_3/MWCNT hybrid[321] had high sensitivity and selectivity to 0.5–10 ppm NO_2 at room temperature with detection limits of 0.1 and 1 ppm, respectively. Shorter responses and recovery times were found with the RGO/WO_3/MWCNT hybrid.[321] Bending tests of up to 90° tested after exposure to gas showed <2% change in the response compared to the flat state. Further mechanical test using 10^6 bending cycles showed no significant degradation. In a humid environment, the baseline resistance shifted upward by 5%. Multilayers of a MWCNTs/PAH hybrid-based sensor on a PET substrate[322] and a VA-CNTs/RGO hybrid film on a polyimide substrate[323] were developed as NO_2 sensors operating at room temperature. The concentration range tested was 0.5–20 ppm. Both the sensors were bent at different angles upon exposure to NO_2; less than 5% change in the response was recorded in comparison to measurements on flat sensors. In order to achieve full recovery, UV radiation[322] and heat treatment[323] were used.

A sensor based on SWNTs on a flexible PET substrate was used for sensing high concentration organic vapors of hexane, toluene, acetone, chloroform, acetonitrile, methanol and water.[327] The highest responses came from polar VOCs and water. A change of <4% in response occurred with bending downward 10 mm to saturated gas compared to the flat position. The sensing properties of a sensor based on AuNP and amine modified MWCNT on a polyimide substrate to 120–5800 ppm polar (propanol and ethanol) and non-polar (hexane, toluene, trichloroethylene and chloroform) VOCs and 4730–25 000 ppm water were also measured.[328] PCA analysis showed discrimination between all these gases and their concentrations. Bending without exposure resulted in a negligible change in resistance.

4.3.4. Flexible carbon nanotube-based field effect transistors. A FET sensor based on a AuNP/MWCNT hybrid on a PET substrate was able to detect down to 300 ppb ammonia with a limit of 255 ± 20 ppb at room temperature,[330] but there was no full recovery. A bending test carried out without exposing the sensor to the gas, but significant change in the conductance was observed after 200 bending cycles.

4.4. Graphene-based sensors

Graphene (GR) is another potential material for gas sensing due to its numerous attractive properties. GR is a 2-D sheet with sp^2 bonds between the carbon atoms, providing a very large surface area of 2630 m^2 g^{-1}. It is also compatible with chemical functionalization, and possesses extraordinary electrical, thermal and mechanical properties.[331] GR is a p-type semiconductor in nature that does not contain metallic impurities as CNTs.[332] It has high carrier mobility (200 000 cm^2 V^{-1} s^{-1}) with a carrier density of 10^{12} cm^{-2} and very low resistivity of 10^{-6} Ω at room temperature.[333,334] High thermal conductivity (~3000 W m^{-1} K^{-1}) and mechanical strength (>1060 GPa) have also been reported.[335,336] These properties, along with biocompatibility[337] and high flexibility,[338,339] make this nanomaterial suitable for many medical purposes. Nowadays, production of GR is simpler and bulk quantities can be produced by the CVD method,[340–342] "unzipping" CNTs;[343,344] and the most efficient method for producing GR monolayers is intercalation of small molecules in a graphite lattice and its exfoliation.[345,346] However, not all the methods are suitable for deposition on flexible substrates.[341,347] Preparation of GR oxide[348–351] and RGO has been well described.[352–354] By controlling GR production and its modification, an ultra-sensitive sensing performance is gained.[355] GR and RGO were extensively reported as sensing materials for FETs,[356–373] chemiresistors[63,374–425] and other sensors[426] in both solid state and flexible designs.

4.4.1. Solid state graphene-based field effect transistors. Unfunctionalized GR and RGO-based FET sensors have been produced for detecting NO_2,[356–358,370] NH_3,[357,359–364] ethanol[365] and humidity[372] mostly at room temperature. Many fabrication parameters influence the sensing performance of the sensors, including substrate[359] and gate type,[360] GR/RGO preparation fabrication methods,[357,358,361–364,370] and numbers of layers.[356] GR gated by ionic liquid FET sensors had a sensitive and fast response to 9–2400 ppm NH_3, with a calculated detection limit of 130 ppb.[360] The current–voltage curve shifted 0.057 V per 10-fold increase in NH_3 concentration. These results are similar to GR FET with a SiO_2 gate, but with a lower operating gate voltage. A NO_2 doped GR FET was tested for detection of 2–80 ppm ammonia with an estimated limit of 200 ppb (Fig. 16).[364] Nevertheless, this doping method was not completely stable. Lu *et al.*[363] reported an RGO-based FET that specifically, upon positive gate potential (n-type operation), possessed outstanding sensitivity to 1% NH_3. Recovery time was 12 min at V_g = 40 V compared to hours or days for p-mode operated FETs. For detection of NO_2, single- and multi-layered GR-based FETs were exposed to 2.5–50 ppm NO_2.[356] The single-layered GR-based device had a high sensitivity of up to 2.5 ppm, whereas the multi-layered device was of low sensitivity. Cui *et al.*[370] reported a FET based on voltage-activated GR for detection of 0.05–1 ppm NO_2. This process created more defects and oxygen functional groups that are primarily responsible for its sensing performance.

For enhancement of the sensing performance of different VOCs and gases, functionalization of GR or RGO with different nanomaterials, such as MOs,[366,367] metals,[368] polymers[373] and DNA,[369] was established under room temperature conditions. Mao *et al.*[366] found a SnO_2/RGO hybrid that selectively detected NO_2. A SnO_2/RGO FET was exposed to 1–100 ppm NO_2 and a detection limit of 1 ppm NO_2 was estimated. However, the recovery was slow, mainly due to the high-energy binding sites on the RGO possibly delaying recovery. Hybrids of Pd/RGO, with electrodes covered with grown graphene by the CVD method, sensitively detected 2–420 ppb NO with a response time of several hundred seconds for low concentrations (Fig. 17).[368] The authors proposed that this ultra-sensitivity was due to the switch from the symmetric Schottky barrier to an asymmetric n-type barrier at the contact between Pd NPs and RGO after NO exposure. Cuong *et al.*[367] successfully demonstrated detection of 2 ppm H_2S with ZnO/GR composites. Detection of 0.25–25 ppm NH_3 was achieved with a poly-3-hexylthiophene (P3HT)/GR

View Article Online

Published on 11 June 2018. Downloaded on 2/1/2025 6:21:23 AM.

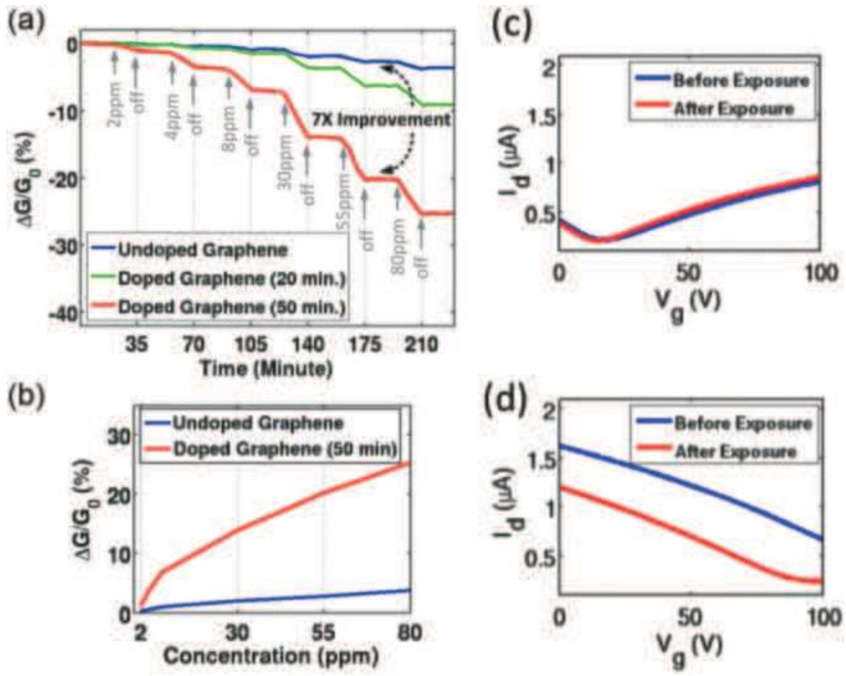

Fig. 16 (a) Sensitivity of as-fabricated graphene and NO_2-doped graphene sensors to NH_3 gas. (b) Sensitivity as a function of NH_3 concentration. I_d–V_g curves of (c) as-fabricated GR-FET and (d) NO_2-doped GR-FET before and after exposure to NH_3. Reprinted from ref. 364 with permission from American Institute of Physics, copyright 2016.

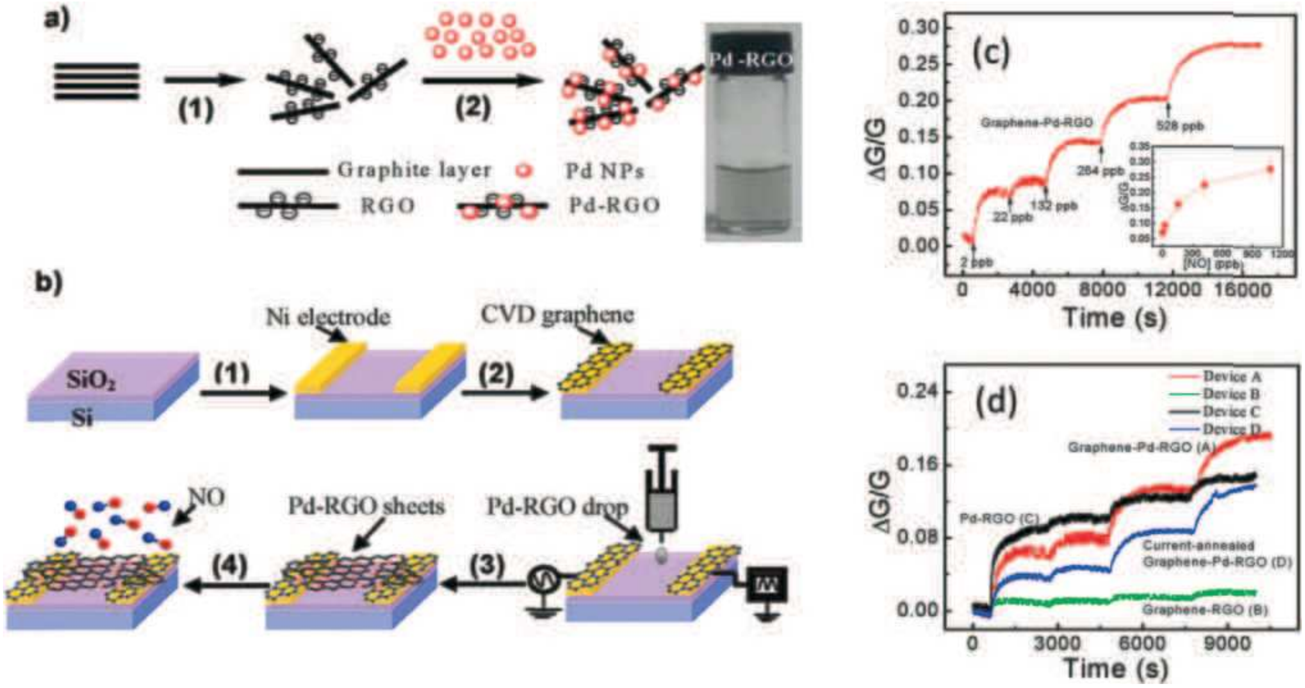

Fig. 17 (a) Schematic illustration of the process for the preparation of Pd–RGO composites: (1) RGO synthesis and (2) Pd decoration of RGO. The inset photograph is the diluted Pd–RGO nanosheet suspension used for ac-DEP. (b) Schematics of graphene–Pd–RGO device fabrication and gas sensing test: (1) Ni electrode fabrication, (2) chemical vapor deposition (CVD) growth of graphene, (3) ac-DEP of Pd–RGO nanosheets, and (4) sensor measurement. (c) Relative change in conductance normalized by initial conductance ($\Delta G/G$), real-time sensitivity dependence of device A of graphene–Pd–RGO exposed to several totally infused NO gas molecules with time durations of 1, 10, 60, 120, and 240 s corresponding to the concentrations of 2, 22, 132, 264, and 528 ppb, respectively. The inset demonstrates steady-state $\Delta G/G$ *versus* the concentration of total infused NO gas molecules ranging from 2 ppb to 1 ppm. (d) Typical relative changes in conductance of the devices *versus* time; the step-shaped conductance response corresponds to 1, 10, 60, and 120 s pulses of NO gas: 2, 22, 132, and 264 ppb, respectively. Reprinted with permission from ref. 368. Copyright (2011) American Chemical Society.

Published on 11 June 2018. Downloaded on 2/1/2025 6:21:23 AM.

nanocomposite based FET operating at room temperature, as the addition of GR to the polymer matrix provided a large surface area for absorption of gas molecules.[373]

4.4.2. Solid-state graphene-based chemiresistors. Many researchers of gas detection propose integration of both modified and pristine GRs and RGOs into chemiresistors.[374–416,425] Pristine GRs and RGOs were thoroughly studied for detecting gases and VOCs, such as NO_2,[375–383,415] NH_3,[375,377,378,381,382] NO[381] and toluene,[384] mostly at room temperature. Hwang *et al.*[377] explored the effect of GR's geometric characteristics on the chemical sensing properties of NO_2 and NH_3. These findings lead to the conclusion that the L/w ratio of an individual GR sheet dominated NH_3 sensing, with no significant effect of the number of GR layers.[377] The influence of the number of layers was measured as a function of 1–25 ppm NO_2 detection, and a bilayer of GR proved to be optimal regarding the number of layers.[415] A slow recovery was observed for all the structures.

Ethanol-based GR nanomesh sensors possessed sensitivities for both 1–10 ppm NO_2 and 5–100 ppm NH_3, with limits of detection of 15 and 160 ppb, respectively.[378] A similar detection limit for NO_2 was found with the epitaxial GR 6H-SiC based sensor.[379] Ozone treated GR-based sensor showed a more sensitive response to 0.2–200 ppm NO_2, with a detection limit of 1.3 ppb. Recovery was achieved with a dry air flow for 30 min.[380] Extraordinary detection limits were obtained by pristine GR continuous illumination with UV light.[381] During exposure, the sensor achieved detection limits of 158 ppq to NO, 2.06 ppt for NO_2, 33.2 ppt for NH_3 and 103 ppt for water. For NO sensing, the detection limit was 300% better than the sensitivity of CNTs under the same conditions. An 80% recovery was achieved within several min.[381] 300 ppb toluene was detected with a polycrystalline GR ribbon-based sensor.[384] In some reports, full and fast recovery was enhanced by joule-heating[382] and UV illumination.[383]

Further improvement of the sensing properties was attained by GR hybrids with other nanomaterials, including MO[374,385–399,416] metals,[400–405] polymers[406–410,425] and organic materials.[411–414] Many investigators have found several MO/GR hybrids for detecting NO_2 in the range of 0.5–200 ppm.[385–393] A 3.2 wt% WO_3/GR nanocomposite film had high sensitivity to 0.5–20 ppm NO_2 at room temperature and 40% RH. A response of 769% during the exposure to 5 ppm NO_2 was achieved with response and recovery times of 9 and 18 min, respectively.[391] The humidity influence on sensing performance was measured over the range of 30–80% RH upon exposure to 5 ppm NO_2. As expected, the response of the sensor decreased as the RH increased. Long-term stability testing resulted in no significant change in the response for at least 45 days.[391] Dong *et al.*[392] reported α-Fe_2O_3/RGO nanocomposites exhibiting a high and selective response of 151% to 90 ppm NO_2 at room temperature, with a detection limit down to 0.18 ppm. The recovery time was in the range of 44–1648 seconds.[392] SnO_2/RGO and In-doped SnO_2(IDTO)/RGO were also prepared as selective NO_2 gas sensors for 1–100 ppm concentration at room temperature, with a detection limit of 300 ppb.[393] However, recovery times were very slow.[393]

Detection of other gases, such as NH_3,[394] acetone,[395,396] H_2S,[395,397,416] formaldehyde[374] and acetylene,[398,399] was also examined by several composites with MO/GRs. For detection of 100–500 ppm NH_3, Cu_2O/RGO composites[394] and an array of SnO_2/RG and CuO/GR[374] were investigated at room temperature. The Cu_2O/RGO-based sensor achieved a high and selective response of 104% to 200 ppm NH_3, with response and recovery times of 28 and 206 seconds, respectively.[394] The sensor array of SnO_2/RG and CuO/GR, in combination with neural network-based signal processing technologies, could recognize and predict the composition of NH_3 and formaldehyde in a gas mixture, with a shorter recovery time of 110 seconds.[374]

Detection of acetone and H_2S, which are associated with diabetes and halitosis, has been reported several times.[395–397,416] Choi *et al.*[395] showed the responses of SnO_2/RGO to these gases as a function of RGO load and temperature at 85–95% RH. There was a sensitive response of 34% to 5 ppm hydrogen sulfide at an optimized temperature of 200 °C with 0.01 wt% RGO loading, whereas a sensitive response of 10% to 5 ppm acetone was achieved by increasing the RGO loading to 5 wt% and the operational temperature to 350 °C. For optimized temperatures, the detection limits were 1 ppm and 100 ppb for hydrogen sulfide and acetone, respectively.[395] A similar detection limit of 120 ppb was found for acetone by the same researchers, demonstrating a selective response with a Co_3O_4/RGO/Ir NP composite at 300 °C and 90% RH.[396] Combination of SnO_2 quantum wires with RGO sheets showed a sensitive and selective detection of 10–100 ppm H_2S at room temperature, with a fully reversible response and a detection limit of 43 ppb.[416] A selective 1–1000 ppm acetylene detection was achieved with ZnO/Ag/GRO films.[398,399] An optimum temperature of 150 °C with a 3 wt% Ag-loaded ZnO/GRO hybrid showed a sensitive response of 21.2% to 100 ppm acetylene gas,[399] and a detection limit of 1 ppm at 11% RH and the optimized temperature.

A AuNPs/GR-based device was evaluated for detecting 15–58 ppm ammonia at room temperature.[401] Recovery time was fast because of a combination of dry air and IR light during the entire recovery process; the detection limit was 6 ppm. Tran *et al.*[402] compared the sensing performance between RGO/AgNWs and RGO/AgNPs to 15–100 ppm NH_3 at room temperature. The RGO/AgNW sensor was more sensitive than RGO/AgNP and AgNP sensors. Cr/GR had a sensitive response to NO_2 under ambient conditions with a detection limit of 90 ppb (Fig. 18).[403] The sensor was exposed to 0.18–12.2 ppm NO_2, with a 540 second recovery time following exposure to 0.732 ppm NO_2. Composites of PIL-mediated Fe_3O_4/RGO and Ag/RGO,[404] as also Gr functionalized with Ag or Au,[405] had sensitive responses to several polar VOCs, including ethanol and acetone.

Graphene oxide/polypyrene (GO/PPr) composite films were used for selective 24–500 ppm toluene detection at room temperature.[406] Addition of PPr led to a more continuous and porous morphology that improved its sensing and mechanical properties. Fast response and recovery times were found. Polymeric modifications are also commonly used for detection of NH_3.[407–410] PANI/GR nanocomposites gave excellent responses to 1–6400 ppm NH_3;[408,409] however, the recovery process and time were inconsistent.[408,409] A 30 day stability test for exposure to NH_3 showed that the response of the PANI/GR to NH_3 decreased and

 This journal is © The Royal Society of Chemistry 2018

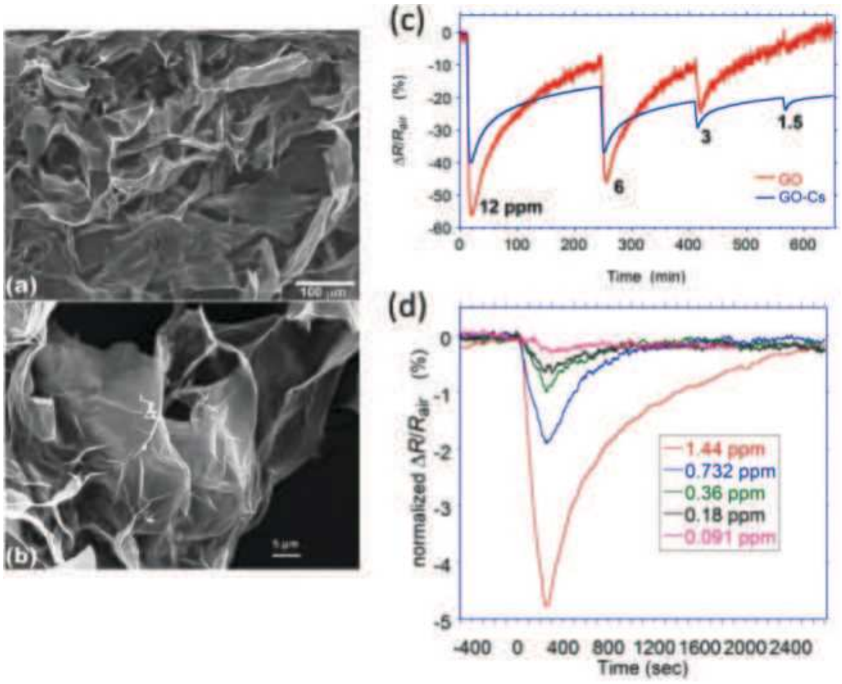

Fig. 18 (a) Low magnification and (b) high magnification SEM images of graphite oxide flakes. Response of (c) GO-Cs and GO based sensors towards NO_2 with concentrations >1 ppm; (d) a GO-Cs based gas sensor after exposure to different concentrations of NO_2 ranging from 0.091 to 1.44 ppm. Reprinted from ref. 403 with permission from Beilstein-Institut, copyright 2014.

then stabilized after 9 days.[409] A hybrid of P3HT/RGO was tested for selective detection of 10–50 ppm NH_3 at room temperature;[410] response and recovery times were relatively short, 141 and 488 seconds, respectively, following exposure to 10 ppm NH_3.

RGO reduced by pyrrole sensors were fabricated and tested with NH_3 at room temperature.[412,413] The range used was 1 ppb to 100 ppm NH_3, which gave a fast recovery under IR illumination.[412,413] The sensor achieved fast response times, such as 1.4 seconds on exposure to 1 ppb NH_3.[412] Sulfonated RGO (S-G) and ethylenediamine-modified RGO (EDA-G)-based sensors were examined for the selective detection of 1–45 ppm NO_2.[414] The detection limits of these modified sensors were 3.6 ppm for S-G and 0.07 ppm for EDA-G, and full recovery was achieved in 30 min.

4.4.3. Flexible graphene-based chemiresistors. Flexible sensors based on GR and RGO are relatively new, and only a few reports have been published on their gas sensing applications. Detection of NO_2,[417–423] NH_3[422–424] and ethanol[63] by flexible Gr-based chemiresistors has been reported. A multilayer GR film on a PI substrate has an enhanced sensitivity to 0.2–5 ppm NO_2 at room temperature.[417] Less than 5% change in the resistance upon exposure to NO_2 was seen compared to the flat state. In order to improve the recovery time, heating elements[418,419] and UV radiation[420] were used. Flexible GR gas sensors integrated with an internal heater[418,419] and a RGO film on a PET substrate with external UV radiation[420] could detect 0.5–200 NO_2. During the bending test, <5% change in the response of the GR film upon exposure to 1 ppm NO_2 was observed compared to an unbent sensor.[419] In contrast, the RGO film showed 12–26% change in resistance as compared to flat measurement.[420] Addition of Pd and Al NPs was investigated in terms of sensing performance of flexible GR-based sensors at 150 °C.[422] A Pd/GR device was sensitive to 5–100 ppm NH_3, whereas Al/GR was sensitive to 1.2–5 ppm NO_2. Pd NPs accumulated the hole carriers of GR and Al depleted hole carriers. An Al/GR sensor was exposed to 1.2 ppm NO_2 with bending for up to 10^4 cycles. Resistance changes were negligible in comparison to the flat sensor even after 3 months. RGO decorated with Ag NPs has also been reported for detecting 0.5–100 ppm NO_2 and 13 ppt NH_3 at room temperature, with fast response and recovery times of 12 and 20 seconds, respectively.[423] An ink-jet printed poly(3,4-ethylenedioxythiophene):poly(styrenesulfonate) (PEDOT:PSS)/GR gas sensor had a high selective response to 25–1000 ppm NH_3 and diethylamine at room temperature compared to a PEDOT:PSS-based sensor (Fig. 19).[424] Upon bending to up to 70° angle under an exposure to 500 ppm NH_3, the gas response to NH_3 increased as the bending angle increased; for an angle of 70°, a 6.2% change in the resistance occurred, probably due to enhanced swelling due to the bending extension. A ZnO nanorod/GR hybrid based on a metal foil was reported to be sensitive to 10–50 ppm ethanol at room temperature and possessed excellent mechanical or electrical properties on flexural deformation to a bending radius of <0.8 cm for up to 100 times.[63] Yang *et al.*[371] demonstrated a 200 ppm NO_2 detection at room temperature by GR-FET on a paper substrate under a strain of 0.5%, with <7% change in the response compared to relaxed conditions.

4.5. Silicon nanowire-based sensors

Silicon nanowires (SiNWs) are excellent candidates for gas sensing applications because they have several advantages, including large

Published on 11 June 2018. Downloaded on 2/1/2025 6:21:23 AM.

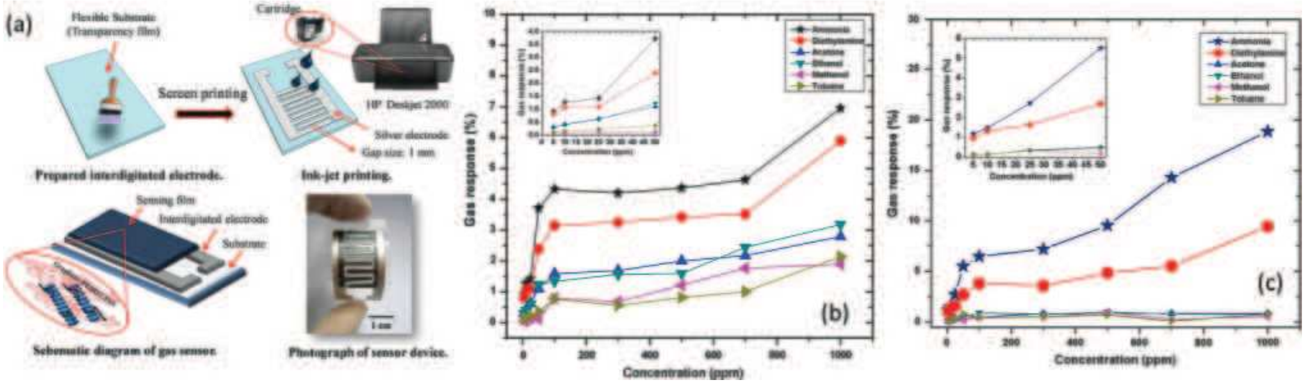

Fig. 19 (a) Schematic diagram of the gas sensor fabrication process. Gas responses of flexible printed (b) PEDOT:PSS and (c) graphene–PEDOT:PSS gas sensors to different concentrations of VOCs at room temperature. Reprinted from ref. 424 with permission from Elsevier, copyright 2014.

Published on 11 June 2018. Downloaded on 2/1/2025 6:21:23 AM.

carrier mobility,[427,428] tunable properties afforded by controlling the doping levels[429] and/or chemical functionalization,[430,431] compatible with very-large-scale integration (VLSI) processes, and compatible with complementary metal-oxide-semiconductor (CMOS) technologies.[432,433] Furthermore, SiNW synthesis can be prepared by several methods, including CVD, pulsed laser deposition (PLD), thermal evaporation, template-assisted growth, and reactive ion etching (RIE).[434–436] Although the sensing capabilities of bare SiNWs to polar VOCs are excellent, detection of non-polar VOCs remains problematic. Functionalization of the SiNWs with different coating layers overcomes this obstacle. Even though there are many reports on SiNW based devices in sensing application, only a small portion sampled in the gas phase.[436–467]

4.5.1. Solid-state silicon nanowire-based field effect transistors. Han *et al.*[443] showed a chemically gated FET based on hybrid integration of 1D SiNWs and 2D SnO_2 thin films. This sensor was exposed to several gases, including 100 ppm NO and 10–100 ppm NH_3. In 2011, Niskanen *et al.*[444] reported on an unmodified SiNW that can be used in combination with pattern recognition algorithms as versatile chemical vapor sensors without additional functionalization. They exposed the sensors to acetone, ethanol and water with 100% accuracy, whereas methanol, ethanol and 2-propanol were classified with 96% accuracy under ambient conditions. Haick's research group has published a number of systematic studies on the fundamentals and applications of functionalized SiNW-based FETs for both polar and non-polar VOC detection.[445–453] Paska *et al.*[445] reported on the sensing properties of SiNW functionalized with hexyltrichlorosilane (HTS) to both polar (water, ethanol, 1-butanol, 1-hexanol, 1-octanol and 1-decanol) and nonpolar (*n*-hexane, *n*-octane and *n*-decane) VOCs at ppm levels in 15% RH air. HTS modification reduced the Si–O–Si bond fraction between adjacent molecules and greatly enhanced sensitivity to both polar and nonpolar VOCs. The sensing mechanism of the nonpolar ones can be explained in terms of an indirect interaction in which nonpolar VOC molecules induce conformational changes in the organic monolayer. The sensing mechanism of polar VOCs is based on direct interaction *via* VOC-induced changes in the SiNW charge carriers, most probably due to electrostatic interaction between the SiNW and polar VOCs. In a subsequent article, Paska *et al.*[446] modeled the detection process based on changes in the carrier mobility, voltage threshold, off-current, off-voltage and subthreshold swing of the devices to non-polar VOCs. The devices were modified with alkyl trichlorosilanes with different alkyl lengths to explore the interactive effect of hysteresis and surface chemistry upon exposure to both polar and non-polar VOCs.[447] The density of the unpassivated Si–OH groups (trap states) on the SiNW surface had a crucial effect on the hysteresis characteristics of the gated silicon nanowire sensors relative to the effect of the hydrophobicity or molecular density of the organic monolayer. Wang *et al.*[448,449] investigated the effects of chain length and functional groups on the sensing properties of silicon nanowires to different various VOCs at 0.01–0.08 p_0/p concentration (p_0 and p represent VOC's partial pressure and the total vapor pressure, respectively). Functionalization of SiNW FETs with longer monolayers of alkane-backbone silane enhanced the selectivity of the sensors to non-polar VOCs.[448]

Conjugation of an array of modified SiNWs with pattern recognition algorithms for discrimination of a variety of VOCs has also been reported.[450,451] Ermanok *et al.*[451] found by a combination with DFA, the potential to discriminate between polar and nonpolar VOCs at 0.01–0.08 p_0/p concentration, as also between the individual VOCs within each group. Combination with an artificial intelligence algorithm on SiNW FET device parameters provided high selectivity to specific VOCs in both single-component and multi-component environments, even in mixtures that contained counteracting compounds of similar structures or chemical properties to those of the target VOC. Concentration was also estimated (Fig. 20).[450] Shehada *et al.*[452,453] reported successful discrimination of simulated samples based on related VOCs to gastric cancer (GC) and lung cancer and environmental confounding factors using an array of modified SiNW FETs.

4.5.2. Solid-state silicon nanowire-based chemiresistors. Bare SiNWs, fabricated by the vapor–liquid–solid mechanism, were used to detect 175–700 ppm NH_3.[455] SiNWs-decorated with TeNPs lowered the detection range to 10–400 ppm NH_3.[456] Bare SiNWs, fabricated by tri-layer nanoimprinting were reported as

Published on 11 June 2018. Downloaded on 2/1/2025 6:21:23 AM.

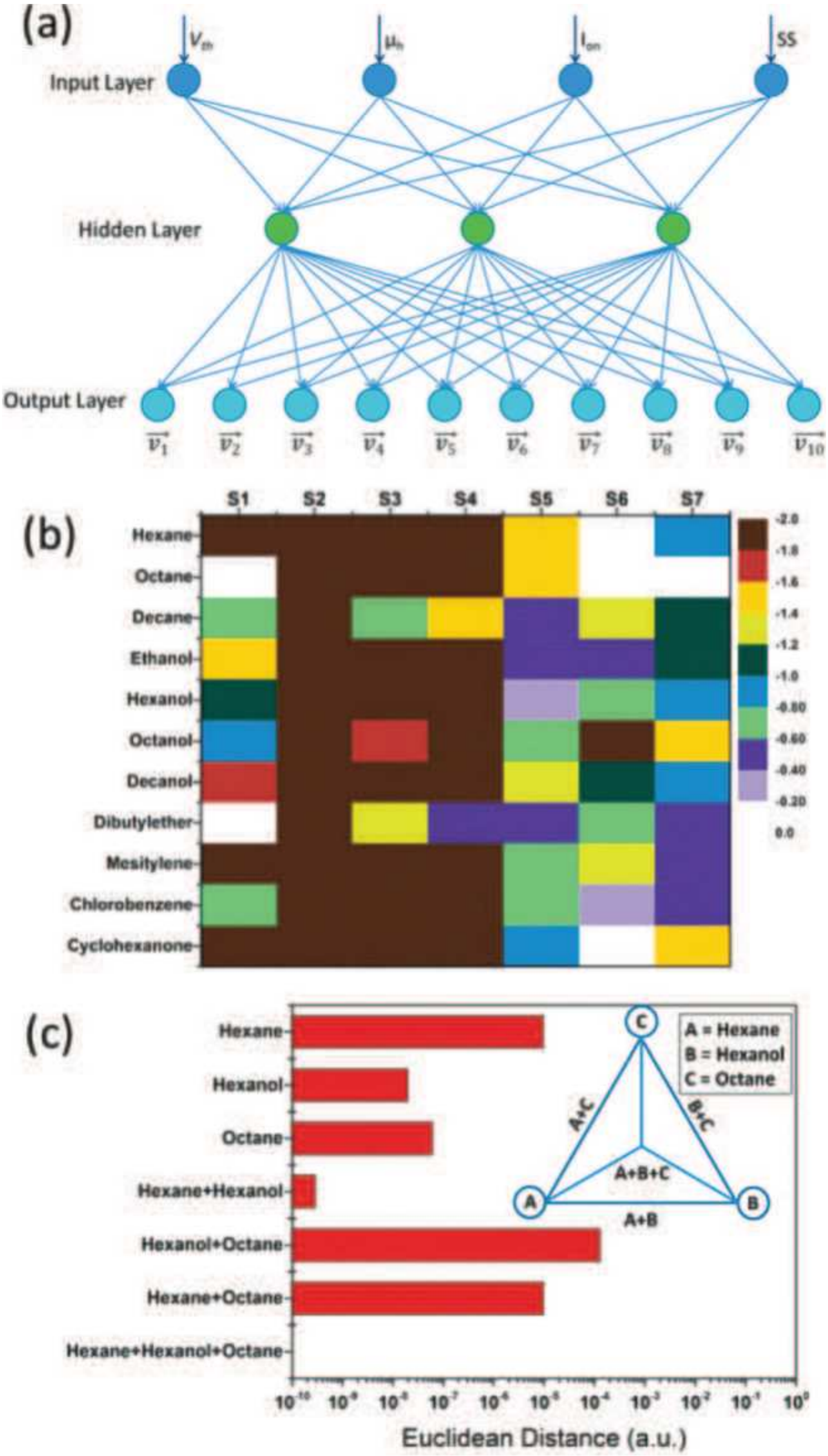

Fig. 20 (a) Schematic illustration of an ANN model for VOC recognition. (b) Hot plots of logarithm Euclidean distance at VOC concentration of p_a/p_0 = 0.08. (c) Euclidean distance of ANN outputs using sensor S3 to identify hexane, hexanol, octane, and their binary and ternary mixtures. All the VOC concentrations are at p_a/p_0 = 0.08. Inset: Schematics of the relationship between single VOCs and their binary and ternary mixtures in ANN outputs. Reprinted with permission from ref. 450. Copyright (2014) American Chemical Society.

chemiresistors for the detection of NO_2[457,458] and NH_3.[457] The authors fabricated 2 nanowire sensors with different line-widths and a thin-film device for comparison. The sensors were sensitive to 250 ppm NO_2[457,458] and NH_3[457] at room temperature, especially for the narrower device due to its larger surface to volume ratio.[457] SiNWs coated with thinner layers of AuNPs gave bigger sensor responses to samples at 30–95% RH at room temperature.[454] As RH increased, a monotonic decrease of the

View Article Online

Published on 11 June 2018. Downloaded on 2/1/2025 6:21:23 AM.

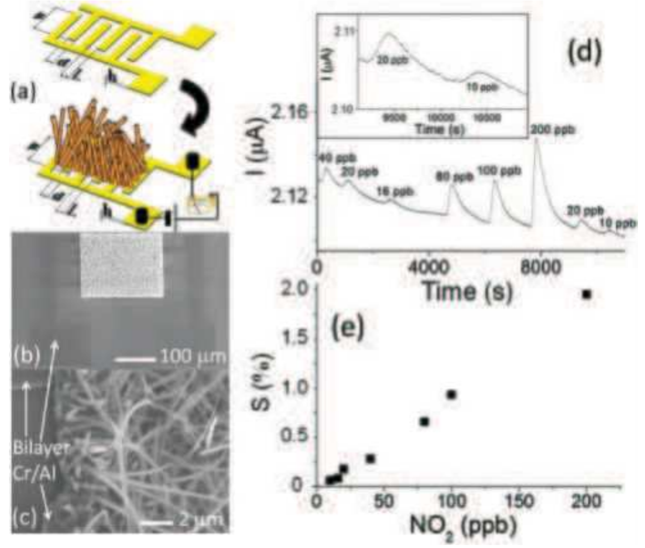

Fig. 21 (a) Sketch of the interdigitated structure used to realize the sensor and the designed Si NW sensor. (b) SEM images of the finite chemoresistive sensor, and (c) Si NW network details for the interdigitated structure with L = 6 μm. (d) Response curve of a typical sensor with a finger gap value of L = 21 μm. The sensor response is the current change due to NO_2 concentration variation at a bias voltage of 1 V. The inset shows the response and recovery behaviors of the device at 20 and 10 ppb. (e) Normalized current variation, defined as $S = 100\Delta I/I_0 = 100(I - I_0)/I_0$, as a function of NO_2 concentration at a selected response time of 3 min. I and I_0 are the electrical current in NO_2/humid nitrogen mixture and only humid nitrogen, respectively. Reprinted from ref. 461 with permission from AIP Publishing, copyright 2012.

current occurred. Modification of peptides on SiNW enhanced sensitivity to 100 ppm NH_3 and acetone at room temperature.[459] Gao *et al.*[460] described a device based on bare SiNWs fabricated by bilayer nanoimprint and angle deposition that had higher sensitivity (155% at 60 nm width of SNW) to 250 ppm NO_2 compared to previous publications.[457,458] In 2012, Cuscunà *et al.*[461] showed an alternative on-chip fabrication method for NO_2 sensors. The authors exploited SiNWs directly grown onto a selected area, over and between pre-patterned interdigitated electrodes defined on oxidized silicon wafers and exposed to ammonia plasma. This sensor was ultrasensitive to 10 ppb gas at room temperature. A similar detection level for NO_2 in humidified air was achieved by a device based on periodically porous top electrodes on a vertical SiNW array (Fig. 21).[461] Furthermore, this device had a sensitive response to 1 ppb NH_3.

Sensors based on ZnO nanorod/porous SiNWs[463] and SiNW/WO_3NW nanocomposites with a cactus-like structure[464] were used for 0.25–50 ppm NO_2 detection at room temperature. A WO_3 nanowires/porous silicon composite proved to be sensitive and selective to NO_2 at sub-ppm levels, with a fast response-recovery characteristic at an operating temperature of 100 °C.[465] Bare porous SiNWs prepared by metal-assisted chemical etching were sensitive to 500 ppb–5000 ppm NO at room temperature, with a fast response and excellent reversibility.[466]

4.5.3. Flexible silicon nanowire-based field effect transistors. Only a single study was found in the literature describing the use of flexible FET devices based on SiNWs, even though many such sensors have been described, using the same solid-state structures. McAlpine *et al.*[467] showed that SiNWs on flexible plastic form a sensitive FET device for detecting 20 ppb–20 ppm NO_2 under ambient conditions. The array of 4r FETs is based on modified (alkane-, aldehyde- and amino-silanes) and unmodified SiNW fabricated on a flexible plastic and exposed to 1000 ppm acetone and hexane. This nanoarray was capable of distinguishing between the gases.

4.6. Surface acoustic wave sensors

Surface acoustic wave (SAW) sensors are based on frequency modulation of waves that are traveling along the surface of an elastic substrate. They are sensitive to physical phenomenon as their amplitudes decay exponentially with substrate depth.[468] The sensing mechanism is based on a frequency shift of the sensing layer, which can be caused by changes in conductivity, stress effect, mass loading and viscoelastic effect as a result of gas absorption.[468,469] Selection of a proper sensing layer is critical in order to enhance good sensitivity and selectivity to the target gas. Atmospheric conditions may influence the propagation of waves. These conditions include temperature, pressure and humidity.[468] Several materials have been used as sensing layers for a variety of gases and VOCs, including MOs,[470–476] GR,[477–479] polymers[480–484] and CNTs.[485–489]

4.6.1. Metal oxide-based surface acoustic wave. Tang *et al.*[470] described a room temperature SAW sensor for the selective detection of NH_3 based on Co_3O_4/SiO_2 composite films. A 50% Co_3O_4 loading into the film gave the best sensing properties to 1–60 ppm NH_3. They recorded the sensor's stability and reproducibility at room temperature. ZnO/SiO_2 bi-layer porous nanofilms on a ST-cut quartz SAW sensor successfully detected 5–120 ppm NH_3 at room temperature.[471] The sensing performance was dependent on the value of sheet conductivity of the films. As a result, the bi-layer nanofilms were more sensitive to NH_3 compared to single layer films, and depended on the thickness of the top ZnO layer. A 60 nm layer of ZnO resulted in a frequency shift of 2000 Hz at 30 ppm of gas, with good repeatability and stability. Pristine SiO_2, TiO_2 and composite SiO_2/TiO_2 films of 200 nm thickness were coated on quartz SAW sensors.[472] Upon exposure to 1–40 ppm, NH_3, TiO_2 and SiO_2/TiO_2 films gave positive frequency shifts, whereas the SiO_2 film gave a negative frequency shift. According to these results, the negative frequency shift was mainly due to the increase of NH_3 mass loading on the sensing film, whereas the positive frequency shift upon exposure to NH_3 gas was related to the condensation of the hydroxyl groups on the film leading to its stiffer and lighter nature. Humidity was also explored, as it has a significant effect on the sensing performance because the water absorbed on the film surface may provide an active site to absorb NH_3, thus enhancing the sensitivity of the sensors. A SAW sensor, based on a composite SiO_2/TiO_2 film, was much more sensitive to NH_3 at low concentration levels, with a response of 2 kHz while displaying fast response and recovery, excellent selectivity and stability.[472] A ferroelectric lead zirconium titanate (PZT)-based SAW sensor was explored as an NO_2 sensor[473]

 This journal is © The Royal Society of Chemistry 2018

Published on 11 June 2018. Downloaded on 2/1/2025 6:21:23 AM.

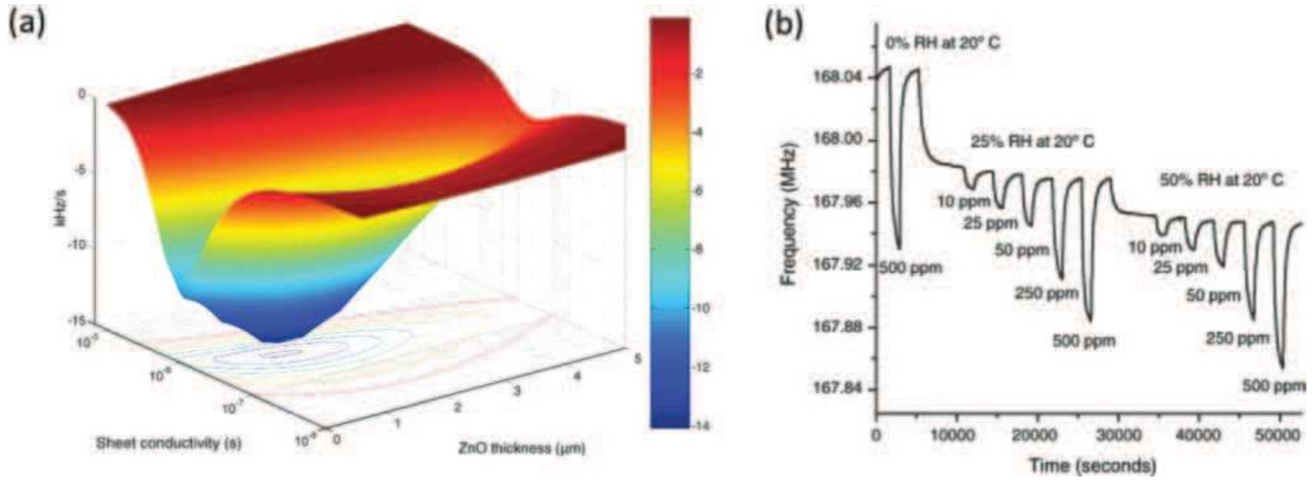

Fig. 22 (a) Sensitivity of a layered structure. (b) Response of ethanol at an operating temperature of 300 °C RH of 0, 25 and 50% (measured at 20 °C). Reprinted from ref. 475 with permission from Elsevier, copyright 2006.

by being exposed to 80–250 ppm gas at room temperature. A linear calibration curve was obtained with a sensitivity of 9.6 Hz ppm^{-1}. Ultrasensitive detection of NO_2 was achieved with a ZnO-based quartz SAW resonator.[474] This sensor exposed to 0.4–16 ppm gas at room temperature was highly sensitive and selective. Ippolito *et al.*[475] exposed an optimized SAW sensor based on ZnO/WO_3 to 10–500 ppm ethanol under both dry and humid conditions. The magnitude of the response decreased with increasing RH levels and decreasing operating temperatures (Fig. 22). Frequency shifts of 119, 90 and 86 kHz upon exposure to 500 ppm ethanol in synthetic air at room temperature were observed for 0, 25 and 50% RH, respectively. The largest response to 500 ppm of ethanol was seen after the operating temperature had increased to 300 °C. Recently, an amino-functionalized iron oxide NP-based SAW sensor was explored as a VOC that was sensitive at room temperature.[476] It was exposed to 25–150 ppm butanol, 400–1200 ppm isopropanol, 200–1000 ppm toluene and 50–200 ppm xylene over a low concentration range. Sensitive responses to these VOCs were demonstrated, together with fast response times of a few min with good reproducibility. The calculated limits of detection for each one of the VOCs was ~1 ppm for butanol, 12 ppm for isopropanol, 3 ppm for toluene and 0.5 ppm for xylene.

4.6.2. Graphene-based surface acoustic wave. GR-Based SAWs have also featured in the literature.[477–479] Thomas *et al.*[478] investigated a GR-based SAW sensor for detecting ppm-levels of NO_2. The sensor had a sensitive response of 25 Hz ppm^{-1}. A GR-like nano-sheet/$LiTaO_3$ SAW sensor was fabricated and investigated for detecting 60–1000 ppm CO gas.[479] A GR/$LiNbO_3$-based SAW device was used to detect a relative humidity in the range of 25 to 85% RH at room temperature.[490] In the low RH range (RH < 50%), a frequency downshift of 1.38 kHz/1% RH change occurred, whereas for the high RH range (RH > 50%), a 2.6 kHz/1% RH frequency downshift occurred.

4.6.3. Polymer-based surface acoustic wave. An array of SAW sensors based on 20 different polymers was evaluated for the detection of a variety of VOCs, including toluene, acetone, ethanol and methanol in dry air each at 120–1300 ppm.[480] The array was most sensitive and selective to toluene exposure. Furthermore, PLS regression was applied to the sensor responses, being obtained from ternary mixtures of the analytes. Stahl *et al.*[481] reported on an array of 8 surface transverse wave (STW) sensors coated with different polymers, which were used at semi-annual intervals for a 3 year period for detecting 3 different VOCs, chloroform, octane and xylene. Among the polymers used as sensing layers were poly(butyl methacrylate), polyepichlorohydrin (PECH) and PDMS, *etc.* The following concentrations were used of each VOC: 2.3–11.6 ppm chloroform, 11.5–34.4 ppm octane and 0.8–4.5 ppm xylene. Radar charts were plotted based on the sensors' responses to obtain characteristic patterns for each VOC. Furthermore, no significant time-related changes were seen over the test period.[481] Liu *et al.*[482] demonstrated a high-frequency SAW (1.56 GHz) humidity sensor based on electrospun CeO_2 NPs/polyvinylpyrrolidone (PVP) nanofibers. The sensor was exposed to 11–95% RH; due to an additional acoustoelectric loading effect arising from the enlarged electrical conductivity of nanofibers at high RH levels, the frequency response was improved compared to a SAW sensor based on pure PVP nanofibers. Addition of inorganic CeO_2 NPs into PVP also enhanced this humidity sensor, with an excellent frequency response and superior long-term stability. A polypyrrole nanofiber surface acoustic wave gas sensor was tested for 2.1–8.5 ppm NO_2 at room temperature.[483] The frequency shift due to the sensor's responses measured 4.5 kHz at 2.1 ppm NO_2. The sensor's performance was also assessed during a 5 day period. A SAW sensor based on a PANI/In_2O_3 nanofiber nanocomposite was NO_2 and CO sensitive at room temperature.[484] This sensor was exposed to 0.51–8.5 ppm NO_2 and 60–1000 ppm CO in dry air; fast response and recovery times with good repeatability were observed.

4.6.4. Carbon nanotube-based surface acoustic wave. SAW sensors based on CNT have also been reported.[485–488] Penza *et al.*[486,487] investigated SAW sensors coated with either SWCNTs or MWCNTs dispersed in ethanol or toluene for detecting VOCs

Published on 11 June 2018. Downloaded on 2/1/2025 6:21:23 AM.

at room temperature. The VOCs included ethanol, ethylacetate and toluene. Selectivity to VOCs can be controlled by the type of organic solvent used to disperse the CNTs, as also the number of layers of the CNTs. For example, the highest sensitivity to ethanol was obtained with SWCNTs in ethanol, whereas for the other VOCs it was with SWCNTs in toluene.[486] The calculated limits of detection were 1.3 ppm for ethanol, 1.6 ppm for ethylacetate and 1.2 ppm for toluene.[486] SAWs, based on nanocomposites of PECH and polyetherurethane with different percentages of MWCNTs, were tested on exposure to octane and toluene at room temperature.[488] The sensors gave a high selective response to toluene and octane, but no response to other gases, such as H_2, NH_3, NO_2 and CO. Addition of small percentages of MWCNTs to the polymer increased the sensitivity to toluene, but hardly changed the response to octane.

4.7. Piezoelectric sensors

Piezoelectric sensors are commonly used for gas-phase detection in a variety of applications.[491,492] The sensor measures fundamental oscillating frequency changes of a quartz crystal resonator as a result of molecule adsorption from the gaseous phase onto its surface. The performance of a quartz crystal microbalance (QCM) system can be controlled by different coating layers selected from a wide range of MOs,[493–497] polymers[498–513] and other nanomaterials.[514–517] Environmental parameters, including temperature and humidity, must be taken into account as they affect the resonant frequency.[501,512,518]

Detection of humidity was demonstrated by several QCM sensors.[512,513,516] A QCM sensor, based on mesoporous silicate SBA-15 with monodisperse hexagonal lamelliform, was prepared as a sensitive humidity sensor.[512] The sensitivity of the sensor exposed to 10–100% RH at room temperature was directly proportional to the film's thickness. A SBA-15 film of 20 nm thickness had the best sensing properties giving 12.32 Hz/RH% with a response time of $\sim$12 seconds and a recovery time of 16 seconds (Fig. 23). The long-term stability of the sensor was tested at 97% RH for 42 days. A fibrous composite of polyacrylic acid (PAA)/PVA membrane was developed as a sensitive coating layer of a QCM sensor to detect humidity.[513] This sensor was exposed to 6–95% RH range and had high sensitivity especially in the range of 20–95% RH compared to a flat PAA/PVA film. The nanofiber PAA membranes were remarkably sensitive to humidity compared to fibrous PAA/PVA membranes. Long-term stability of a PAA nanofiber-based QCM was also measured over 30 days and a good stability was observed. Yao *et al.*[516] investigated the performance of a chemically derived graphene oxide (GO) thin film coating deposited at different thicknesses on a QCM sensor for humidity detection. The sensor was exposed to 6.4–93.5% RH environments at room temperature. As the thickness of the film increased, the sensor became less sensitive to humidity compared with thinner coating layers. Long-term stability was measured for 3 weeks.

4.7.1. Metal oxide-based quartz crystal microbalance. ZnO NW-based QCM sensors with different thicknesses of the sensing layer were prepared and tested for detecting 40–1000 ppm NH_3 at room temperature.[493] A fast response time of 5 seconds was observed for each gas concentration tested. The sensors showed good frequency stability and reproducibility. ZnO nanorods were also explored for selective detection of NH_3 at room temperature.[494] QCM-based ZnO nanorods were exposed to 50–200 ppm NH_3 with a 9.1 Hz frequency shift to 50 ppm. The response and recovery times varied with NH_3 concentration. These gas sensors also showed good reproducibility and high stability. Thin ITO film-based QCMs were demonstrated as an NO detection sensor;[495–497] they were exposed to 295–1770 ppm NO and had an approximately linear negative frequency shift when exposed to an NO-doped atmosphere.[495] Hu *et al.*[496] found good sensitivity of an ITO-coated QCM sensor to 60–1180 ppm at room temperature. Furthermore, a distinct negative frequency shift of 110 Hz within 600 seconds was observed upon exposure to 60 ppm gas. However, the responses of all these reported sensors were irreversible.[495–497]

4.7.2. Polymer-based quartz crystal microbalance. Electrospun nanofibers of cross-linkable PAA and PVA with different

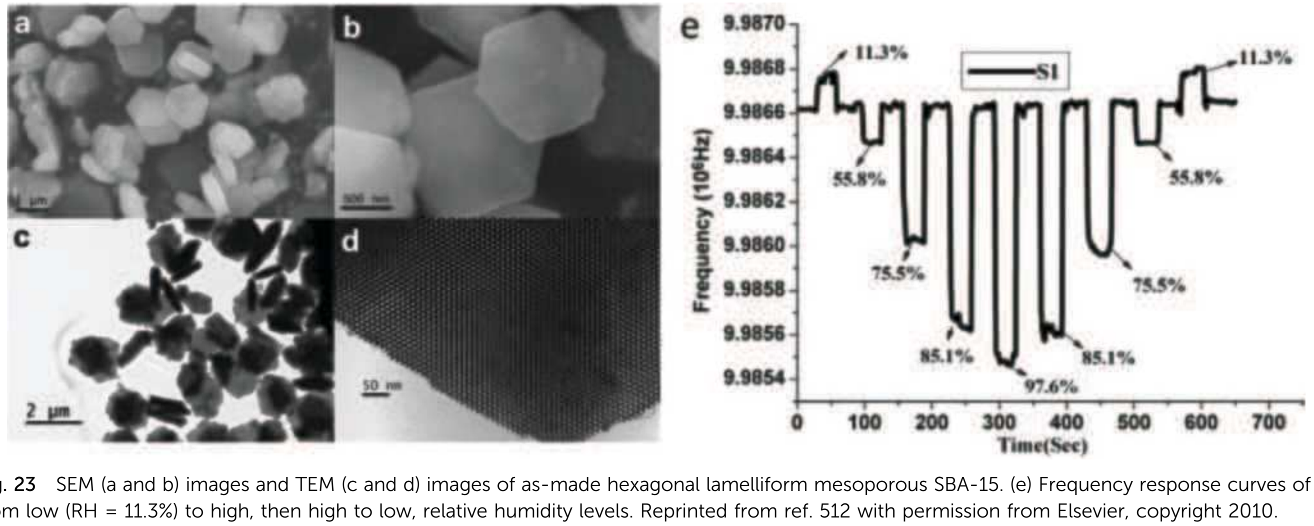

Fig. 23 SEM (a and b) images and TEM (c and d) images of as-made hexagonal lamelliform mesoporous SBA-15. (e) Frequency response curves of S1 from low (RH = 11.3%) to high, then high to low, relative humidity levels. Reprinted from ref. 512 with permission from Elsevier, copyright 2010.

 This journal is © The Royal Society of Chemistry 2018

weight ratios of PAA/PVA were deposited on the surface of QCM for detecting 50–200 ppm NH_3 at room temperature and 50–60% RH.[498] The sensing performance was affected mainly by the content of the PAA component in the nanofibers, the relative humidity and the concentration of NH_3. Palaniappan *et al.*[499] detected NO with phthalocyanine/silica hybrid films on a QCM at room temperature. Their results showed sensitivities up to 25 ppb Hz^{-1}. Cross-sensitivity to CO was also evaluated. A QCM coated with cross-linked poly(styrene-*co*-chloromethyl styrene) was fabricated to develop an NO_2 gas sensor.[500] The results indicate a sensitivity of 1200 Hz h^{-1} for 50 ppm NO_2, yet gas exposure irreversibly decreased the oscillation frequencies of all the devices tested.

Formaldehyde detection has also been demonstrated with QCM sensors.[503,504] Zhang *et al.*[503] tested a QCM sensor coated with 3D polystyrene (PS) fibers functionalized with polyethyleneimine with 3–140 ppm gas at room temperature. The sensor showed selective and sensitive responses to the gas. PEI/PVA nanofibers at a weight ratio of 1.6/1, formed from the cosolvent of water and ethanol, were also reported to be formaldehyde sensitive.[504] QCM-based PEI/PVA nanofibers were found to selectively respond to 10–255 ppm gas at room temperature. During exposure to formaldehyde, the sensor was exposed to humidity conditions from 10 to 70% RH. Exposure to 255 ppm formaldehyde at 10% RH gave no response, whereas responses of the QCM sensor were augmented in increasing relative humidity (Fig. 24).

Ayad *et al.*[505] described a PANI-coated QCM sensor for detecting alcohol vapors, such as ethanol, methanol, 2-propanol and 1-propanol, in the ppm range at room temperature. A linear correlation was noted between concentrations of alcohol vapors in the ppm range and the frequency shift. Increasing film thickness led to more hydrogen bond formation with the PANI chains.

Si *et al.*[506] fabricated and tested 8 QCM sensors coated with different conducting polymers of thiophene and its derivatives with a variety of VOCs, including toluene at 200–5000 ppm, and *p*-xylene, acetic acid, acetone, ethanol, 1-octanol, acetonitrile and water vapor at 5000 ppm. By combining with PCA analysis, the array discriminated between polar and non-polar VOCs. Calixarene film-coated QCM sensors were used for detecting VOCs, including aromatics, chlororganics, ketones, and alcohols.[507]

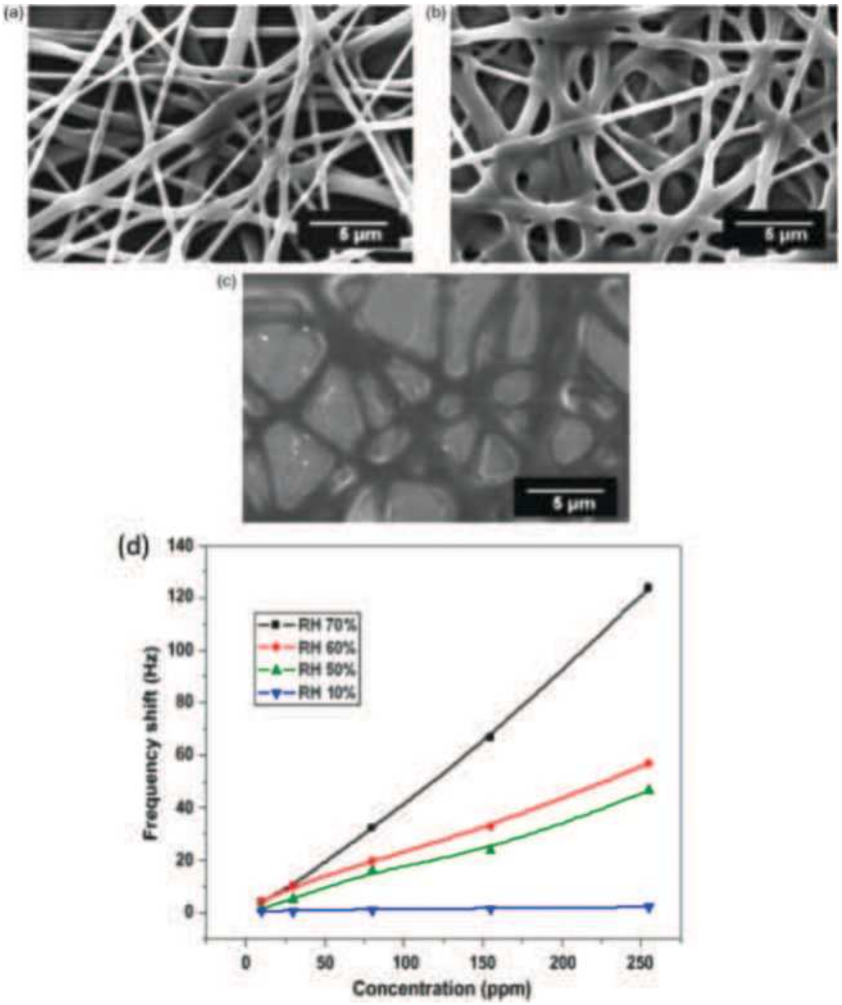

Fig. 24 FE-SEM images of fibrous membranes at different weight ratios of PEI/PVA: (a) 0.8/1; (b) 1.6/1; and (c) 3.2/1. (d) Response of the fibrous membrane coated QCM sensor with a PEI/PVA weight ratio of 1.6/1 formed from the cosolvent of water and ethanol, exposed to 10, 30, 80, 155, and 255 ppm formaldehyde at different RHs. Reprinted from ref. 504 with permission from Elsevier, copyright 2010.

Published on 11 June 2018. Downloaded on 2/1/2025 6:21:23 AM.

Published on 11 June 2018. Downloaded on 2/1/2025 6:21:23 AM.

The calixarene molecule acts as the host whereas different VOCs act as guests. Structural changes of the numbers of aryl fragments and the binding of different functional groups to upper and lower rims lead to sensing of various VOCs with different sensitivity and selectivity. Phosphorous-containing calixarenes showed maximal sensitivity to the tested VOCs with a limit of detection in the ppm range. PEGylated lipids (polyethylene glycol) containing disulphide as supports have also been used as QCM coating layers.[508] These sensors were exposed to a wide range of VOCs, including alcohols, esters, acids and aldehydes each at 100 ppm. PCA analysis showed separation between the different VOCs into 10 distinct groups. QCM-coated with a PVC blended lipid membrane was exposed to methanol, ethanol, chloroform, acetone and benzene at high ppm levels.[509] By combining with PCA analysis, the sensor showed good separation and classification of VOCs. An array of 8 phthalocyanine-coated QCM sensors together with an artificial neural network (ANN) was used to find the composition of gas mixtures.[510] Three binary systems were used in this study, namely: ethanol–acetone, ethanol–trichloroethylene, and acetone–trichloroethylene. By using the ANN algorithm, the success rate of the system in identifying the component concentrations was $>84.5\%$, with overall average prediction error of 10.6%. Toluene and *p*-xylene molecular imprinted polymers, composed of methyl methacrylate as a monomer and divinylbenzene as a cross-linking agent, were prepared in the presence of toluene and *p*-xylene.[511] By adding the solvent molecules, specific recognition sites inside the polymer matrix were created. Each sensor reacted with the preferred VOC in the range of 170–540 ppm, but with slow response rates.

4.7.3. Other nanomaterial-based quartz crystal microbalance. TiO_2/poly(acrylic acid) ultrathin film QCM sensors were fabricated and exposed to 0.3–15 ppm NH_3 at different humidities.[514] There was a linear response to NH_3 concentration. Moreover, no frequency changes were recorded upon exposure to NH_3 over a 30–70% RH range. The limit of detection was estimated at 0.1 ppm. PVP/RGO nanocomposites were sprayed on a QCM sensor for selective NO_2 sensing at room temperature.[515] The sensor exposed to 20–100 ppm had a higher sensitivity and a shorter recovery time than a PVP-based QCM sensor. Tao *et al.*[517] developed a QCM-based sensor for detecting acetone in the breath of diabetic patients. The authors used a room temperature ionic liquid (1-butyl-3-methylimidazolium tetrafluoroborate) as a selective sensing material coating the QCM. The solubilization of acetone in the ionic liquid leads to a decrease in density and viscosity of the film, resulting in a positive frequency shift of the QCM. A linear response to varying acetone concentrations was achieved, with a detection limit of 5 ppm. Confounding VOCs commonly present in breath (*e.g.*, isoprene) did not interfere with the response.[517]

4.8. Colorimetric sensors

Colorimetric sensors are based on chemo-responsive indicators, which chemically react and change their color differentially upon exposure to different chemical species.[519,520] Color changes are measurable by scanning the sensor array before and after an exposure, thereby identifying and quantifying the species. Chemo-responsive indicators include (i) pH indicators that respond to Brønsted acidity/basicity (*i.e.*, hydrogen bonding), (ii) metal salts that respond to redox reactions, (iii) metal ion-containing dyes that respond to Lewis basicity (*i.e.*, electron pair donation); (iv) dyes with large permanent dipoles (*i.e.*, solvatochromic dyes), and (v) nucleophilic indicators that respond to electrophilic analytes.[519,520] A colorimetric sensor array has many advantages, such as being a fast, small and portable device with the possibility for customization. Nevertheless, many challenges need to be overcome; for example, an array is operated in an irreversible manner, meaning it has to be a disposable single-use device. Another challenge is reproducibility of the printing and imaging, in addition to difficulties in determining individual components in a mixture.[521] Yet another involves the sensing non-polar VOCs compared to the abilities of other sensors.[519]

Suslick *et al.*[522] described an array of 24 colorimetric sensors based on a variety of chemo-responsive dyes for detection of a wide range of VOCs at room temperature. These chemo-responsive dyes included Lewis acid/base dyes, Brønsted acidic or basic dyes, and dyes with large permanent dipoles. This array was exposed to alcohols, aldehydes, esters, ketones, and other compounds. Linear combinations of the RGB responses to the 24 dyes, in combination with PCA analysis, could discriminate between different VOCs. Furthermore, the selected dyes were essentially unresponsive to water vapor. Sensitivity at the ppb-level was demonstrated for VOCs, such as thiols, amines and carboxylic acids. In further investigations, colorimetric arrays were tested with up to 100 different VOCs and different humidities (Fig. 25),[523–526] and similar results were obtained.

Detection and quantification of NH_3, NO_2 and H_2S was investigated by a colorimetric array with different chemo-responsive dyes.[527,528] Significant color changes upon exposure to defined concentrations of each analyte were observed. The extrapolated limits of detection for NH_3, NO_2 and H_2S were 0.08, 0.03 and 0.06 ppm, respectively,[527] values significantly below the permissible exposure limits of the gases. Sen *et al.*[529] detected 0.05–50 ppm H_2S at room temperature with an array of chemo-responsive dyes, with an exposure time of up to 30 min to reach equilibrium. PCA analysis gave a 100% classification at all concentrations tested. A polymeric optical waveguide based on PET foils for detection of 5–20 ppm NH_3 has been recorded.[530] The planar waveguide configuration was intended to improve the transducer sensitivity and simplify the fabrication process. The sensor had fast response times and good sensitivity to the gas. Endo *et al.*[531] have described selective detection of benzene, toluene, acetone and xylene by a colorimetric sensor based on a glass substrate with a 3D colloidal crystal and PDMS elastomer. A polydiacetylene/GR-stacked composite film-based colorimetric sensor was tested with 0.01–10% of a variety of VOCs, including methanol.[532]

Humidity detection by a colorimetric sensor has also been reported.[533] A carbon-coated copper/polymer porous hybrid film was synthesized for humidity sensing resulting from plasmon resonance and interference. No change in color was observed up

 This journal is © The Royal Society of Chemistry 2018

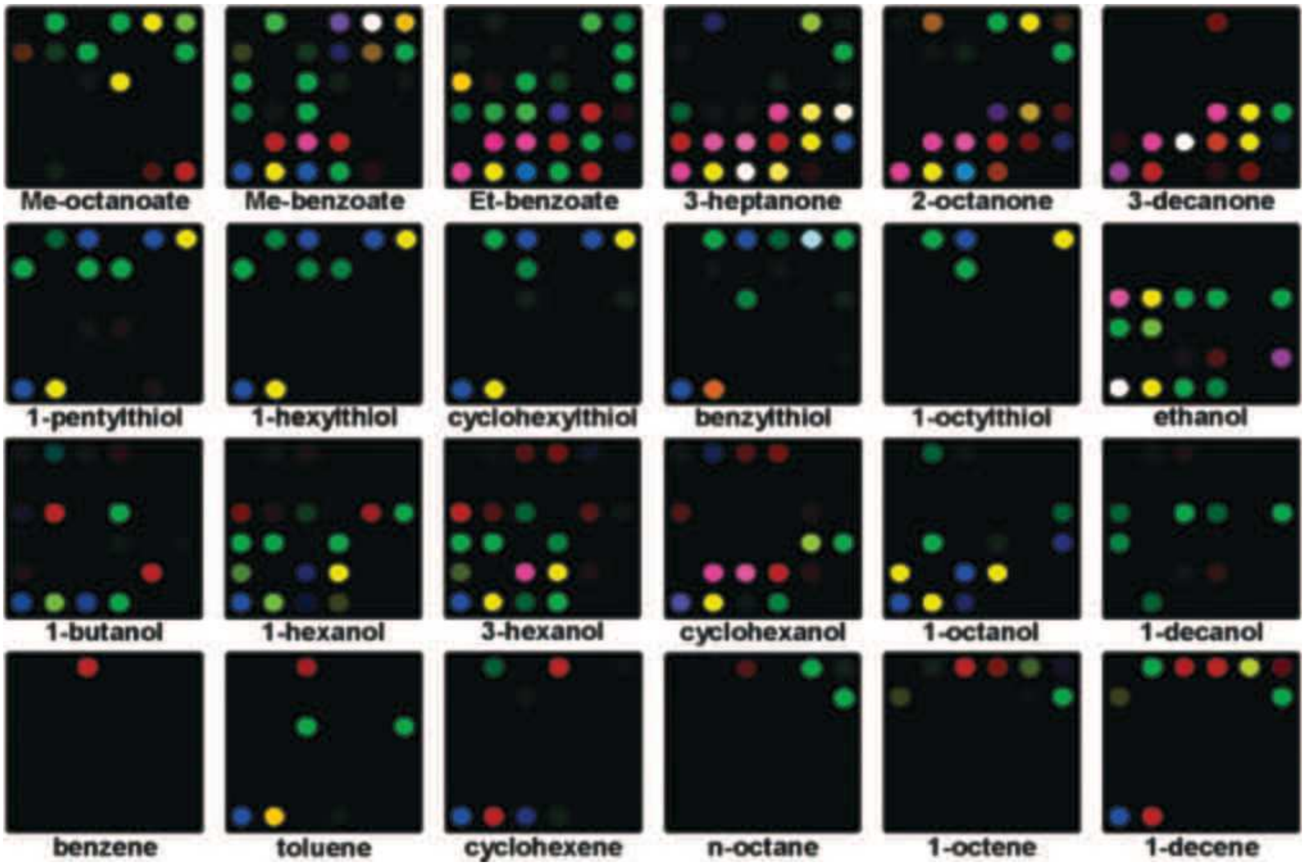

Fig. 25 Colorimetric array response for 24 representatives of less responsive organic compounds at their vapor pressure at 295 K; color range expanded from 4 to 8 bits per color (8–17 expanded to 0–255). Reprinted with permission from ref. 523. Copyright (2006) American Chemical Society.

Published on 11 June 2018. Downloaded on 2/1/2025 6:21:23 AM.

to 50% RH. At approximately 65% RH, the color of the film shifted to green and the absorption peak also shifted. At 70% RH, the color of the film shifted to blue. The absorption shifted by 50 nm per 1% increase in RH from 70 to 80% RH.

4.9. Optical sensors

Optical fibers seem to be promising gas sensors.[534,535] These sensors can be characterized by the location at which the transduction between light and measurement takes place, namely inside or outside the fiber, termed intrinsic and extrinsic, respectively.[534] Intrinsic optical fibers are more frequently used for detection of gases and VOCs, as a passive layer of the fiber is replaced with a small segment of gas-sensitive nanomaterials, such as polymers,[536–543] GR[544–547] and MOs.[534,535,548] Upon exposure to gases, optical and/or structural changes occur in the reactive layer of the fiber, which evoke a change in the reflective index of the optical fiber changing its transmission properties.[534] Sensitivity of optical fibers to gases can be increased by bending or using hollow core fibers, resulting in an increase in the penetration depth in the sensing area by increasing the coupling of the optical power in the evanescent field.[534,536,547,549] Optical fibers have short response and recovery times, along with on-line monitoring possibilities.[534,535,538]

4.9.1. Metal oxide-based optical fiber. Bismuth oxide (Bi_2O_3)-clad modified optical fibers were used to detect 100–500 ppm methanol, acetone, NH_3 and ethanol at room temperature.[548] There was gas selectivity for methanol, ethanol and NH_3, with sensitivities of 212×10^{-3} kPa^{-1}, 109×10^{-3} kPa^{-1} and 15×10^{-3} kPa^{-1}, respectively.

4.9.2. Polymer-based optical fiber. A U-bent optical fiber sensor modified with an organic–inorganic hybrid of PAH/silica NPs and infused with tetrakis (4-sulfophenyl)porphine (TPSS) was tested as a vapor sensor (Fig. 26).[536] The sensor was exposed to different alcoholic vapors and water. As expected, TPSS worked as a selective absorption site for methanol, leading to the highest response. Silva *et al.*[537] reported on an optical fiber coated with a sensitive film of poly[methyl(3,3,3-trifluoropropyl)siloxane] for detection of pentane, heptane, octane, decane, benzene, toluene and other VOCs in human breath at room temperature. Detection limits ranged from 0.8 pmol L^{-1}, for heptane to 9.5 pmol L^{-1} for decane. The fiber responses also had high linearity and stability. In a further study, a portable optical fiber coated with the same polymer was used to detect benzene, toluene, ethylbenzene, *p*-xylene, *m*-xylene and *o*-xylene in real-time monitoring.[538] The fiber had high sensitivity to the vapors tested, with high linearity and detection limits in the range of a few nanograms. In yet another study, an optical fiber was coated with a vapochromic material for sensing acetone and dichloromethane.[550] Changes in the transmitted optical power were up to 13.5 dB as a result of exposures to different concentrations of the vapors.

Published on 11 June 2018. Downloaded on 2/1/2025 6:21:23 AM.

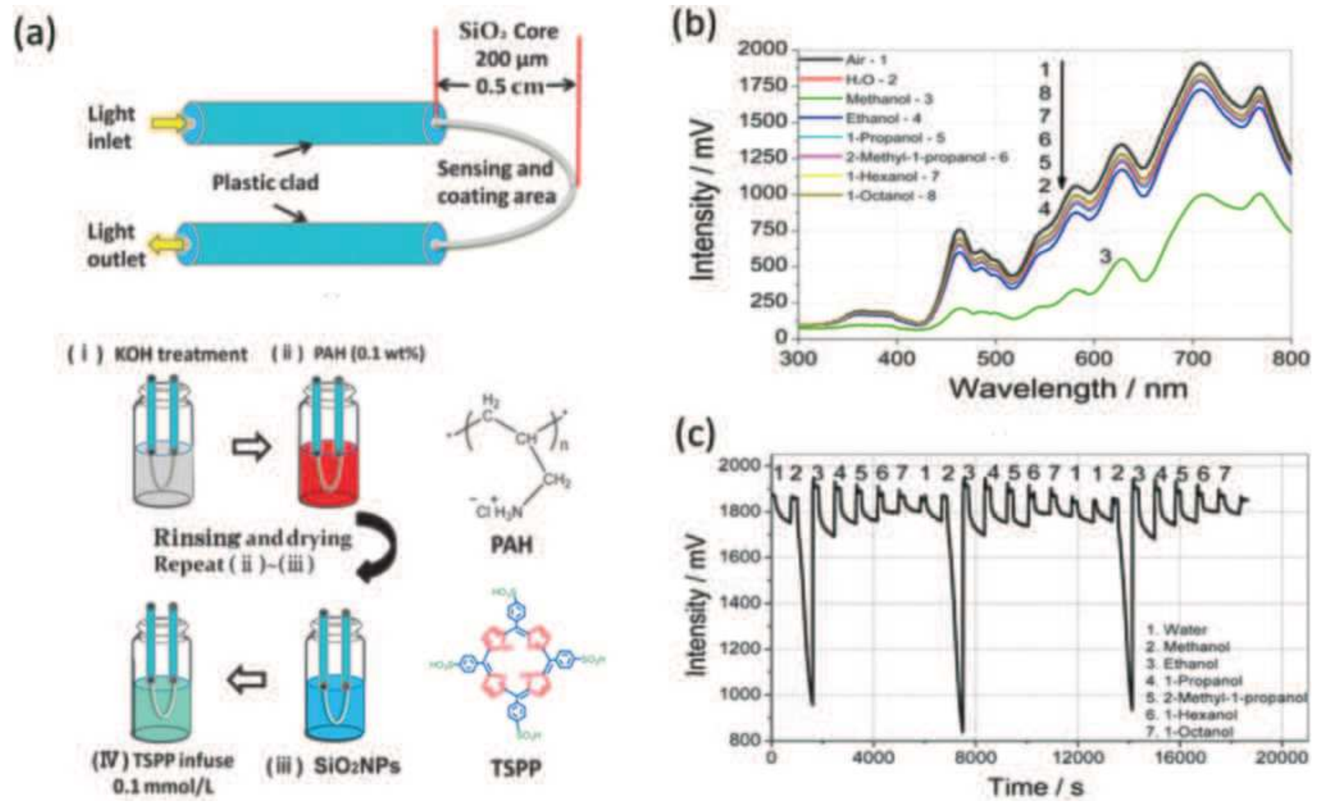

Fig. 26 (a) Schematic of a U-bent optical fiber and the coating process of a TSPP-infused PAH/SiO_2 alternate thin film. (b) Changes in the transmission spectra of the U-bent OF with a TSPP-infused (PAH/SiO_2)10 film after exposure to water and alcohol vapors. (c) Dynamic responses at 700 nm of the sensor after exposure to water and alcohol vapors. Reprinted from ref. 536 with permission from Wiley-VCH, copyright 2017.

Elosúa *et al.*[540] could detect ethanol, methanol and isopropanol with a thin layer of a different vapochromic material on an optical fiber. Changes up to 3 dB in the reflected optical power were found upon exposure to 21.5–125 mmol L^{-1} gas. A lutetium bisphthalocyanine thin layer was also used as a sensitive layer on an optical fiber for detection of a variety of vapors at room temperature.[541] The fiber was exposed to acetic acid, ethanol, *n*-butyl acetate and hexanal at different concentrations in the range from 4 to 88 mmol L^{-1}. As expected, the highest attenuation of the reflected optical power was seen with acetic acid, the most oxidant compound, with a variation of 9.5 dB at the highest concentration. For sensitive detection of aromatic vapors, an optical fiber coated with calixarene has been tested.[542] Once the nanoscale cavity in the calixarene has entrapped a VOC molecule, there is a change in the refractive index. This fiber had been exposed to toluene, benzene, cyclohexane and hexane. As expected, the fiber gave the highest response to toluene and the lowest to hexane. Sensitivity to toluene of 231 ppm was reached, while ensuring a reversible response with a fast recovery time. An electrospun polypyrrole–polyethylene oxide coated optical fiber was fabricated and exposed to different VOCs, including 0.1–10 ppm NH_3, 6–10 ppm acetone and 2–10 ppm methanol and ethanol.[543] The sensor was highly sensitivity to ammonia, methanol and ethanol vapors, but less to acetone.

4.9.3. Graphene-based optical fiber. A reduced GR oxide coated hollow core fiber exposed to 30–90% RH[547] showed sensitivity of up to 0.22 dB/% RH, with good repeatability and a fast response time. A reduced graphene oxide coated PMMA optical fiber proved sensitive to 0–500 ppm ethanol and methanol at room temperature.[544] The sensor had good sensitivities of 0.065 and 0.038 counts per ppm ethanol and methanol, respectively. Optical fibers based on Pt NP-incorporated graphene oxide possessed high sensitivity to 0–120 ppm NH_3 at room temperature.[545] The Pt-decorated sensor had a sensitivity of 10.2 pm ppm^{-1}, 3 times higher than the sensitivity of a fiber based on reduced Gr alone. An optimal concentration of Pt NPs was 185.2 mg L^{-1}. A linear response was obtained for NH_3 concentrations <80 ppm. In another study, optical-fibers based on different concentrations of Ag NPs on exfoliated graphene oxide sheets were tested for detection of 0–500 ppm NH_3.[546] The optimal concentration was 0.1 M AgNP, which yielded 1.5 and 2.2 times higher sensitivity than 0.6 M AgNP/Gr oxide composite and reduced graphene oxide coated sensors, respectively.

5. *In vitro*-based disease diagnostics

5.1. Cancerous diseases

5.1.1. Lung cancer

5.1.1.1. Monolayer-capped metal nanoparticle- and carbon nanotube-based sensors. Many studies have reported the ability of VOCs to serve as biomarkers for lung cancer based on

Published on 11 June 2018. Downloaded on 2/1/2025 6:21:23 AM.

histologic and genetic classification after analysis of the head-space of cancer cell lines by different analytical methods.[551–557] However, not many have dealt with the ability of cross-reactive nanomaterial-based devices to discriminate between lung cancer cell lines with different histologies and genetic mutations. Comprehensive *in vitro* studies conducted by Barash *et al.*[558] reported 100% accuracy for discriminating between 7 different non-small cell lung cancer (NSCLC) cell lines from their growth medium using an array of cross reactive nanomaterial sensors based on organically modified GNPs. A volatolomic signature obtained by applying the PCA algorithm on the multidimensional responses of the nanoarray also successfully discriminated between breath prints of LC patients and healthy controls.[558] In a complementary study, the same NSCLC cell lines could be successfully discriminated from immortal bronchial epithelium (IBE) cell lines, which served as healthy controls, with 96% sensitivity, 86% specificity and 93% accuracy. NSCLC cell lines were also distinguished from small cell lung cancer (SCLC) cell lines with 100% sensitivity, 75% specificity and 96% accuracy. Furthermore, an artificial intelligence nanoarray could discriminate sub-histologies of NSCLC cell lines with 90% accuracy by applying support vector machine (SVM) cross-validation (Fig. 27).[559]

In a different study, DFA analysis of the same cross-reactive nanomaterial-based device resulted in accuracies varying between 84 and 96% for genetic mapping of 3 selected genes related to targeted therapy in LC: EGFR, KRAS and EML4-ALK fusion.[560] Headspace samples of 6 cell lines representing EGFRmut oncogene, 4 representing mutant KRAS, one cell line with EML4-ALK fusion and 7 lung cancer cell lines of wild type (WT) to all 3 tested genes were collected. Nine volatolomic signatures of the GNPs' responses were constructed using a DFA model by comparing each mutation with a large group of WT cells, between every 2 mutations, and between each mutation and a small group of lung cancer cell lines, which were WT to all. Classification success of the global 9 test array was calculated by global leave-one-out cross validation, giving 76% accuracy (Fig. 28).[560]

The above-mentioned studies were conducted on lung cancer cell lines, which provide an excellent model for simulating LC diversity; however, in aiming to assign specific volatolomic signatures to distinct genetic mutation, a more precise and delicate model is required. For this purpose Davies *et al.*[561] have developed a human bronchial epithelial cell (HBEC) model. HBEC provides a more traceable system to follow the effect of minimal genetic alterations by using the parental cell line in which each additional genetic lesion can be studied. With this in mind, cancer cells bearing either KRAS mutation knock-down of T53 or both were compared. Headspace samples were analyzed by means of artificial intelligence nanoarray in combination with DFA, resulting in accuracies above 77% in multiple comparison analysis.[561] To increase the discriminative power in this study, a combined array of organic-coated GNPs and organic-modified CNTs was used. DFA results demonstrate eNOSE ability to differentiate between lung cancer cells with minimal genetic alterations based on volatiles emitted into their microenvironment. The volatolomic signature can now be translated into barcodes to provide an easy and accurate tool for genetic mapping of LC, which can be used in the clinics for biopsy classification, testing tumor margins and other purposes.

5.1.1.2. Polymer composite. In a different study, LC sub histologies, including 4 types of NSCLC, 1 type of metastatic squamous cell carcinoma of the lung and 1 mesothelioma, were successfully classified and discriminated from each other as well as from normal fibroblasts and smooth muscle cells by applying PCA analysis on responses obtained from a polymer composite electronic nose.[562] The electronic nose classification success was estimated by calculating Mahalanobis distances, which were in most cases >3, indicating distinct volatolomic signatures of the different aerodigestive track malignant cell lines.[562]

5.1.2. Breast cancer

5.1.2.1. Metal oxide sensors. Many studies have proven the efficiency of breast cancer screening by the analysis of breath VOCs. However, only one *in vitro* study was reported on breast

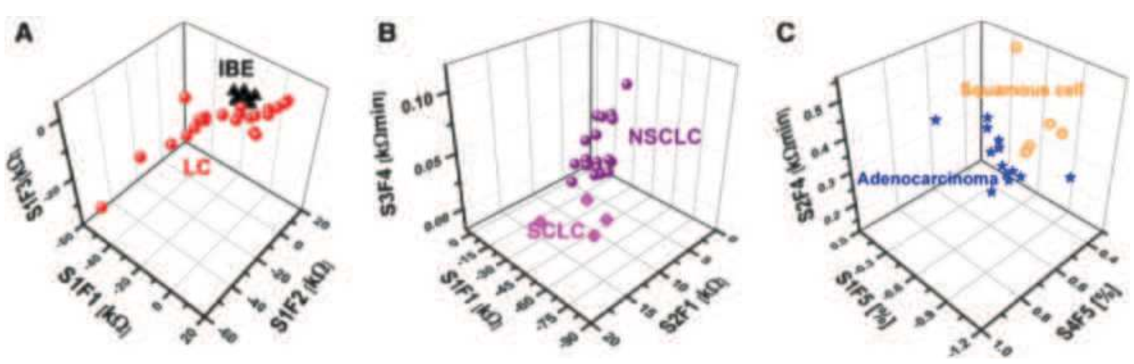

Fig. 27 SVM analysis from the GNPs' multidimensional data output for optimal separation between headspace samples from: (A) LC and IBE cells; (B) SCLC and NSCLC; and from (C) subcategories of NSCLC (*i.e.*, adenocarcinoma and squamous cell carcinoma cells). The pseudo-3D representation of the selected features illustrates the separation between histologically different LC subtypes. F1 is the ΔR_{start}, F2 is the ΔR_{mid}, F3 is the ΔR_{end}, F4 is the $\Delta R/R_0$, and F5 is the area under the normalized response signal (note: ΔR is the net resistance change upon exposure and R_0 is the baseline resistance). S1–S4 refers to the sensors being tested. Reprinted from ref. 559 with permission from Elsevier, copyright 2012.

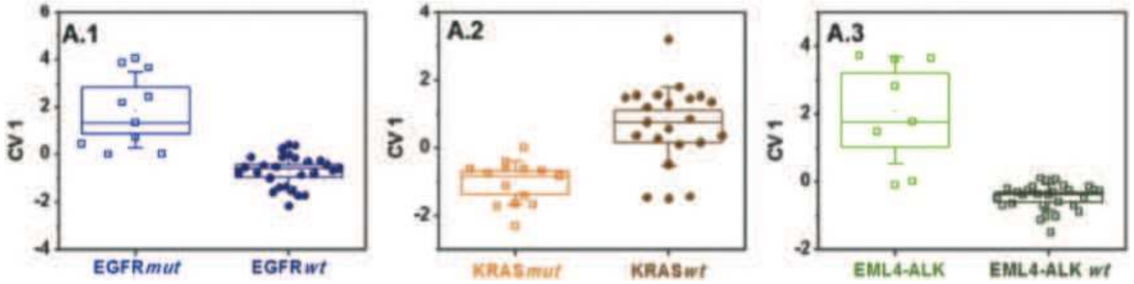

Fig. 28 DFA plots of CV1 calculated from the responses of GNP sensors to the headspace samples of NSCLC cell-lines having EGFRmut, KRASmut or EML4-ALK oncogenes, or genetic mutations that are WT to the former 3. The standard deviation of the CV1 values is represented by the error bars. The boxes represent the 95% CI of the CV1 values, corresponding to 1.96 × SE. Subpanels A.1–A.3 represent the 3 primary general DFA models applied simultaneously to the data and contain all 37 headspace samples. Each point represents one sample. The samples were obtained from multiple cell-lines having the oncogenes of interest. Reprinted from ref. 560 with permission from Elsevier, copyright 2013.

Published on 11 June 2018. Downloaded on 2/1/2025 6:21:23 AM.

cancer cell lines, suggesting that VOC detection might provide an insight into the biochemical and tumorigenic characteristics of the disease.[563] By using a self-temperature modulated metal oxide semiconductor gas sensor, researchers could distinguish between the headspace VOCs of non-transformed breast control cells MCF10a, 5 transformed breast cancer cell lines MDA231, culture media without cells and distilled water (Fig. 29). Breast control cell lines were also discriminated from 5 different types of cancer cell lines (MDA231, MCF-7, ZR751, BT474, SKBR3) by applying a PCA model. The PLS-DA classification model resulted in 88% sensitivity and 80% specificity for the discrimination between cancer cells and non-transformed controls.[563] Interestingly, the PLS-DA model could detect breast cancer cell lines with low cell doubling times and those positive to estrogen receptor with 88% and 85% accuracy, respectively. Breast cancer cell lines expressing progesterone receptor (PgR) and HER2 overexpression were classified with lower accuracies (<75%). These results suggest the possibility that headspace analysis provides cost-effective prognostic and therapeutic molecular information that might have a huge impact on the clinical outcomes due to accurate and fast tumor genetic mapping.[563]

5.1.3. Prostate cancer

5.1.3.1. Metal oxide sensors. Since prostate cancer is the most common cancer in men worldwide and as the performance

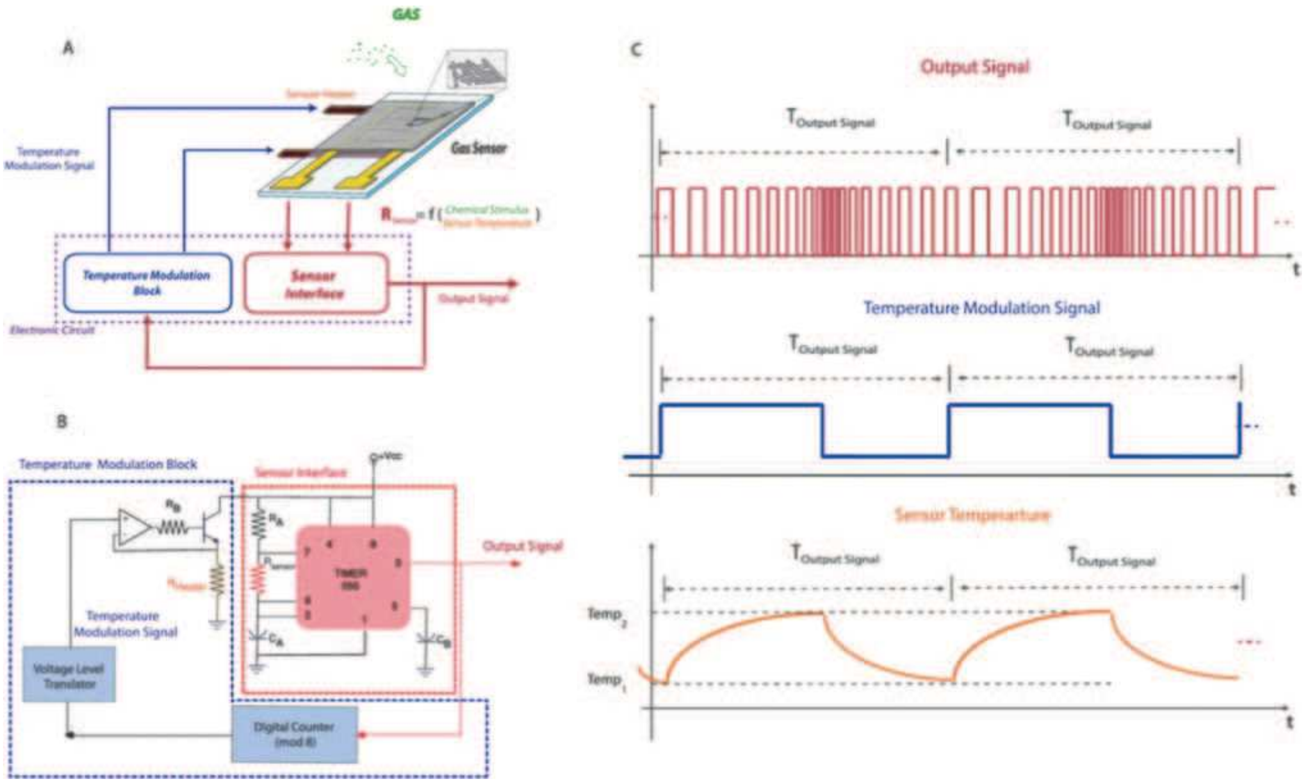

Fig. 29 (A) Schematic of the self-modulation sensor. (B) Circuit implementation used in this work; (C) from top to bottom, examples of output signal, temperature modulation signal and sensor temperature. Reprinted from ref. 563 with permission from Nature Publishing Group, copyright 2015.

Published on 11 June 2018. Downloaded on 2/1/2025 6:21:23 AM.

of prostate specific antigen tests is not optimal, there is a need for a new biomarker. An *in vitro* study of telomerase-immortalized prostate epithelial cell line EP-156T and prostate adenocarcinoma cell line LNCaP was conducted to test the ability of an electronic nose to distinguish between benign and malignant prostate cell lines. The e-nose device consists of an ion mobility cell, which consists of 8 electrode strips and a metal oxide semiconductor cell.[564] PCA analysis was used for dimension reduction and visualization of the sensor's responses. Linear discriminate classifiers were used to distinguish between cell lines and water, cell lines from media, and between benign and cancer cells. M-fold cross validation was used to calculate misclassification rates (MCRs), which ranged from 1.4 to 1.7%. Discrimination between the malignant and nonmalignant cell lines resulted in 2.6–3.6% MCRs. Testing of cell lines and empty media gave a definite conclusion, *i.e.* that cells modify the volatolomic signature of their media.[564]

5.1.4. Hepatocellular carcinoma

5.1.4.1. Monolayer-capped metal nanoparticle- and carbon nanotube-based sensors. In a headspace study of 36 cell cultures with high metastatic potential (HMP) and low metastatic potential (LMP), and from normal cells, samples were collected and analyzed by means of nanomaterial based sensors with DFA as the pattern recognition algorithm. Classification success was determined by leave-one-out cross-validation. The artificial intelligence nanoarray used was composed of cross-reactive chemiresistors based on organic functionalized GNPs and SWCNTs.[565] Resistance recording from different combinations of sensors in the array served as the input to the DFA model, which resulted in 97% accuracy for the discrimination of HCC cell lines from normal cell headspace samples. Accuracy was >96% for the comparisons of HCC and LMP compared to normal cells. The comparison between HCC-LMP and HCC-HMP was 96.7% accuracy, with 83% sensitivity and 100% specificity.[565]

5.1.5. Gastric cancer

5.1.5.1. Multi-walled carbon nanotube/Au–Ag electrochemical biosensor. Headspace VOCs of MGC-803 gastric cancer (GC) cells and GSE-1 gastric mucus cells were sampled and analyzed by a MWCNTs/Au–Ag electrochemical biosensor with different modified electrodes (MWNTs/Au–Ag/glass carbon electrode (GCE), MWNTs/Ag/GCE, MWNTs/Au/GCE, MWNTs/GCE and bare GCE).[566] Preliminary experiments with the different electrodes suggested that the MWCNTs/Au–Ag nanocomposites formed a more efficient electrode sensing film than other nanomaterials, which can be explained by the improved absorbing properties of MWCNTs/Au–Ag/GCE due to a synergistic effect. The different *I–V* curves from the GC cells, the mucosa cells, and the cell-free medium were recorded and compared; clear differences were seen between the curves. The investigators also exposed the electrochemical sensors to 3-octanone and butanone, found only in the headspace of the GC cell lines. Results of the simulated gas exposure might explain the excellent separation between the *I–V* curves of the 2 types of cell lines, implying that butanone and 3-octanone had more electrochemical activity compared to other compounds, as detected by GC-MS.[566]

5.1.6. Brain cancers

5.1.6.1. Polymer–carbon black composite films. Odor signatures of organs taken from chicken, such as liver and heart, as well as human glioblastoma (U251) and human melanoma tumor (A2058) cell lines, have been investigated. Headspace VOCs were analyzed by an electronic nose composed of 16 sensors with different coated polymer–carbon black composite films.[567] Comparison between glioblastoma and melanoma tumor cell lines and their medium resulted in 44% and 77% accuracies, respectively. Comparing the initial number of cultured cells, melanoma cell lines did not produce any significant difference in volatile signature, whereas there was a 49% variation for the glioblastoma cells. However, successful variation of the headspace signature was achieved when comparing the 2 types of cancer cells, as also in comparing the 2 types of fresh organ tissues.[567]

5.1.7. Melanoma. Skin cancer is the most common form of cancer in the United States. Melanoma is the deadliest of skin cancers, as it can rapidly spread to distant parts of the body. The incidence of melanoma has risen rapidly over the past 30 years; the rate of diagnosis has increased by 2–3% per year from 2004 to 2013 among both men and women aged 50 or older,[568] with an associated economic burden estimated at $932.5 million per year across all age groups.[569] Early detection of melanoma is critical for improving survival rate: if detected when still confined to the primary site, the melanoma 5 year survival rate is 98.2%.[570]

5.1.7.1. Quartz crystal microbalance sensors. An array of 7 QCM chemical sensors coated with different metalloporphyrins has been used for identifying unique fingerprints for nevus and melanoma based on headspace samples.[571–573] Three melanoma cancer cell lines, a synovial sarcoma and a thyroid cancer cell line were grown on a Petri dish covered with stainless-steel cylinders for headspace collection.[571] Score plots of the first 2 principal components (PCs) obtained from the PCA model based on responses of the QCM sensors to the headspace discriminated well between melanomas and other tumor cell lines (Fig. 30). In a different study by this group, 4 QMB sensors coated with different metalloporphyrins could distinguish melanoma cell lines from different tumor cells by applying the PCA algorithm.[572] These results imply that there is a unique volatolomic signature produced by melanoma cells that elicits response from the metalloporphyrin-coated sensors and suggests the potential use of these sensors for mole detection straight from the skin. To establish their hypothesis, these investigators also ran *in vitro* trials by smelling melanoma xerographs from nude mice in an *in vivo* experiment.[573] *In vivo* data led to the conclusion that tumor xerographs produce a distinctive VOC pattern that can be differentiated from the volatolomic signature of a tumor-free control. Thus, an appropriate and well-tuned sensor array is capable of detecting and monitoring tumor growth by continually analyzing headspace VOCs.

5.2. Detection of bacteria

5.2.1. Metal-oxide sensors. Accurate and fast bacterial identification may be of considerable importance both in

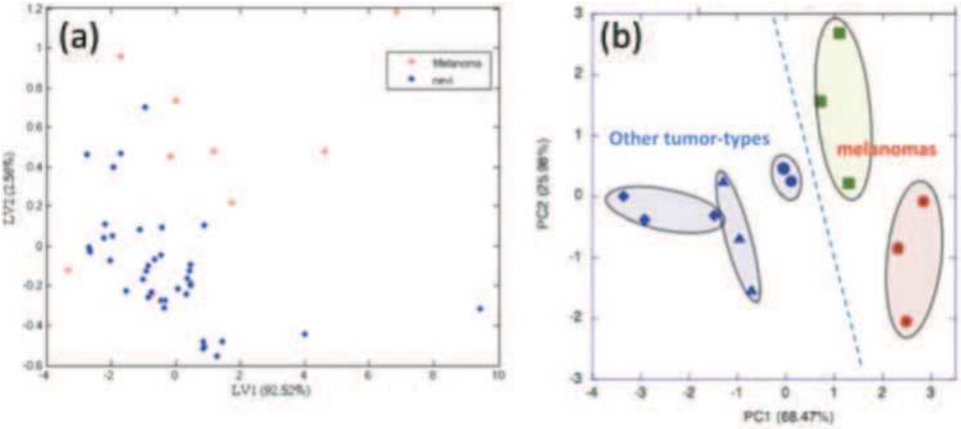

Fig. 30 (a) Score plot of the first 2 latent variables (LVs) of the PLS model built on the data collected from *in vivo* control and melanoma skin lesions. Good discrimination between the 2 groups I seen, and is confirmed by a correct classification percentage of 87%; (b) score plot of the first 2 PCs of the PCA model built on the cell culture headspace measurements. A good discrimination can be seen between melanomas and other tumors along the principal component 1 (PC1) (68.5% of the total variance). Reprinted from ref. 571 with permission from Elsevier, copyright 2009.

Published on 11 June 2018. Downloaded on 2/1/2025 6:21:23 AM.

medicine and industry. Clinician prescriptions of appropriate and the most suitable antibiotic treatment are dependent on bacterial identification, which can take a long time. During this delay, the infection may spread and as a consequence sepsis continues to be one of the leading causes of death in USA and Europe. In industry, regulatory processes may cause unnecessary delays due to bacterial infections.[574–576] This is the reason for many studies using different electronic noses for headspace detection of different pathogenic bacteria.[575,577–585] In one study, headspace samples (360 samples per bacterium) of 2 pathogenic microorganisms, *Escherichia coli* (*E. coli*) and *Staphylococcus aureus* (*S. aureus*), were successfully discriminated using an array of 6 different metal oxide semiconducting gas sensors selected to target hydrocarbons, alcohols, aldehydes/heteroatoms, polar and non-polar molecules.[586] Samples were classified by applying multi-layer perceptron (MLP) with a back-propagation (BP) learning algorithm. The best performance of the MLP resulted in correct classification of 96.1% of all test vectors, with only 2.2% incorrectly classified and 1.7% unknown.

5.2.2. Electroconductive polymers. Furthermore, an array of 14 electroconductive polymer, semi-micro chemoresistive sensors could differentiate between 12 different bacteria and one pathogenic yeast with a 93.4% classification success by applying a neutral network.[580] An array of 8 QCMs, 8 MOs and 4 electrochemical gas sensors was used to discriminate between *E. coli* and *Enterobacter aerogenes*. PCA analysis was applied to discriminate between the different bacteria and between different periods during bacterial growth.[582]

5.2.3. Colorimetric sensor array. In another study, *E. coli*, *Pseudomonas aeruginosa*, *S. aureus* and *Moraxella catarrhalis* were discriminated by means of a colorimetric sensor array with 100% accuracy using a logistic regression classification algorithm for the sensor's responses to headspace samples.[581] These results imply that different bacteria produce distinct volatile metabolites due to different enzymatic pathways, resulting in unique volatolomic signatures in their headspace samples. In a further study using disposable colorimetric sensors, researchers discriminated not only between different bacterial species, but between different strains of the same species. All strains and their antibiotic resistant forms were successfully classified with 98.8% accuracy within 10 h (Fig. 31).[577] Whereas in most studies a single headspace sample taken from a closed environment is examined, in this research the VOCs produced by the bacterium were also tested over time, indicating different phases of the bacterial growth.

5.3. Tuberculosis

5.3.1. Electroconductive polymer. Few studies have reported the ability of electronic noses to identify Tuberculosis (TB) through detection of headspace VOCs in an *in vitro* setup. It was reported elsewhere that 37 headspace samples of TB-positive and TB-negative cultures were analyzed by means of an electronic nose composed of 14 conducting polymers.[587] Sixty-one samples previously clinically diagnosed as positive or negative by conventional microbiology methods were analyzed after headspace sampling. A neural network differentiated between *M. tuberculosis* and a sterile culture with a 100% prediction rate. DFA analysis was applied to check the model's ability to detect blind samples, which also resulted in 100% accuracy. In a second trial, discrimination of 96% between 61 samples with *M. tuberculosis* and related species was achieved by applying the neural network algorithm. DFA analysis was also applied to check the model on a blind sample, which gave absolute identification of the blind samples. Applying the neural network to sensor parameters obtained from exposures to sputum samples resulted in a prediction rate of 96%.

In a different study by Fend *et al.*[588] a gas sensor array composed of 14 conducting polymers detected 3 different *Mycobacterium* species (*M. tuberculosis*, *M. avium*, and *M. scrofulaceum*) and *Pseudomonas aeruginosa* in the headspace of cultures, and could differentiate between them and empty medium. Furthermore, applying the DFA model on the first 4 PCs obtained through PCA analysis resulted in clear differentiation between the 3 *Mycobacterium* spp. Blind analysis of 15 samples gave 100% accuracy.

View Article Online

Published on 11 June 2018. Downloaded on 2/1/2025 6:21:23 AM.

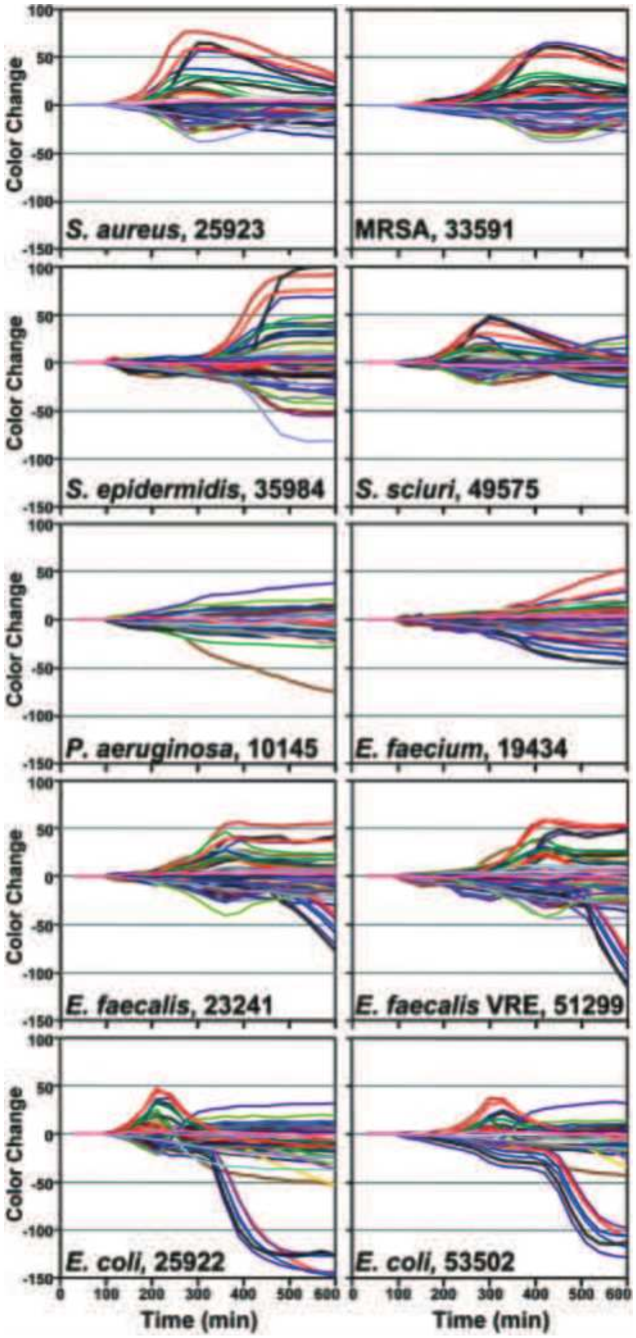

Fig. 31 Time response profiles for 10 different bacterial strains (name and American Type Culture Collection number given in each panel). For each bacterial strain, the color change values *versus* time are plotted for all color channels at each time-point. Reprinted (adapted) with permission from ref. 577. Copyright (2011) American Chemical Society.

In a complementary study in which negative pooled sputum samples were spiked with the 3 different bacteria, the electronic nose discriminated between them with 100% success. For sensitivity calculation, 6 different concentrated *M. tuberculosis* suspensions were analyzed. In this experiment, 2 of the 14 blind samples were incorrectly classified, resulting in a high detection limit of 1×10^4 mycobacteria per mL. In a complementary spiked sputum trial, all 12 blind samples were correctly identified. The performance of the electronic nose was validated from 330 clinical samples of 50 positive control samples, which consisted of non-TB sputum samples spiked with 10^8 *M. tuberculosis* cells per mL, 50 negative non-TB sputum samples from Netherlands, and 280 samples from Africa. Good separation was obtained between TB-positive and TB-negative samples by applying PCA analysis.[588]

6. Breath-based disease diagnostics

6.1. Cancerous diseases

6.1.1. Lung cancer

6.1.1.1. Monolayer-capped metal nanoparticle- and carbon nanotube-based sensors. Many studies have shown the ability of an artificial intelligence nanoarray composed of GNP chemiresistors in the screening, detection, classification and genetic mapping of lung cancer through extended breath analysis from hundreds of volunteers. A series of studies conducted by Haick *et al.* on a monolayer gold nanoparticle sensor array have proven the ability of an electronic nose to discriminate between early and late stages of LC with 88% accuracy, and between different histologies (*e.g.* SCLC and NSCLC) and sub-histologies (*e.g.* adenocarcinoma and squamous cell carcinoma) of LC with 93% and 88% accuracy, respectively.[7,17,191,589] In a recent study of Shlomi *et al.*[590] 19 LC patients harboring the EGFR mutation were successfully discriminated from 34 LC patients who were EGFR wild type with 83% accuracy, 79% sensitivity and 85% specificity. Furthermore, early LC patients (n = 16) were discriminated from patients with benign nodules (n = 30) with 87% accuracy, positive predictive (PPV) and negative predictive values (NPV), using an array of 40 cross-reactive chemiresistors, based on organically functionalized GNPs, as well as SWCNTs, capped with PAHs.[590] In another study, 144 breath samples were collected from 39 advanced LC patients. The investigators could discriminate between LC patients after surgery and monitoring response to therapy using only one GNP sensor with only 59% accuracy. However, DFA analysis of the collective responses obtained from the nanoarray had the ability to monitor changes in tumor response across therapy and also indicate lack of any further response to therapy with 85% success.[591] Using the same array of GNPs, the researchers could detect LC at early onset and monitor breath volatolomics after LC resection. DFA maps clearly differentiated between LC patients and volunteers with benign nodules before and after surgery. Breath prints of LC volunteers before resection were clearly distinguished from those of patients after surgery. Moreover, clear discrimination was obtained between patients with benign nodules pre- and post-surgery (Fig. 32).[7] Furthermore, 4 of the most common cancers in developed countries – lung, breast, colorectal and prostate – were compared and could be discriminated from each other, as also from healthy states, regardless of some confounding factors (including age, gender, and smoking habits). PCA analysis of GNPs' responses to 5 breath fingerprints with minimal overlap between them indicates a high potential of these sensors to serve as first-line tools in cancer screening.[592]

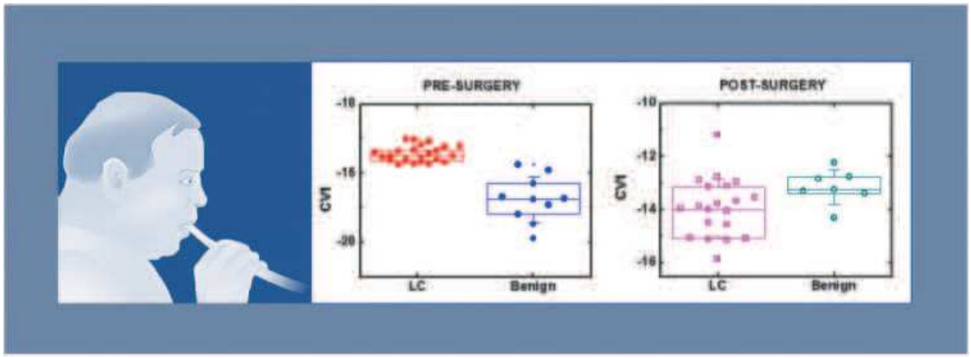

Fig. 32 DFA models that discriminated between lung cancer (LC) and benign states before surgery, and LC and benign states after surgery. Each point represents a single breath sample; 1–2 breath samples were collected per patient. Reprinted from ref. 7 with permission from Elsevier, copyright 2013.

Published on 11 June 2018. Downloaded on 2/1/2025 6:21:23 AM.

6.1.1.2. Polymer composite sensors. Breath signatures of LC patients obtained from the PCA plot of the Cyranose 320 responses could successfully be distinguished from those of subjects with chronic obstructive pulmonary disease (COPD).[593,594] About 85% of the samples were correctly classified by linear canonical discriminate analysis with 3.73 Mahalanobis distance between the group means.[593] Furthermore, breath signatures of LC patients could be discriminated from healthy controls with 90% accuracy and 2.96 Mahalanobis distance. In another study, breath signatures of 14 individuals with bronchogenic carcinoma were discriminated from those of 45 healthy controls using an electronic nose composed of 32 polymer composite sensors.[594] The applied mathematical algorithm used for analyzing the sensor responses was PCA in combination with Mahalanobis distance for discrimination between the classes. A separate group of 76 volunteers (14 with cancer and 62 tumor-free) was used for constructing a cancer prediction model by the SVM algorithm, which gave 71.4% sensitivity and 91.9% specificity.[594] However, since the data were collected and validated on patients with relatively advanced LC, there is a need to modify the model to be more suitable for a wider population.

6.1.1.3. Quartz crystal microbalance sensors. In a separate study, a total of 42 breath samples were collected from patients with different types of lung cancer. An electronic nose composed of 8 QCM sensors coated with different metalloporphyrins was used to detect cancer-diseased patients, and distinguish between cancer-diseased and post-surgery patients, by applying PLS-DA on the sensor's response.[595] A total accuracy of 90.3% was achieved with 100% of the patients, with LC being correctly identified. In following research, the same set of porphyrins coating 8 QCM sensors together with the PLS-DA algorithm for data classification was used.[596] For this purpose, breath samples from 36 healthy controls, 28 patients with LC and 28 patients with diverse lung diseases were collected. Classification between LC patients and healthy controls, as well as between lung cancer and other lung disease patients, as also between LC and lung disease together with healthy controls was successfully achieved with 85, 92.8 and 89.3% sensitivity, respectively. Artificial cancerous breath samples were also generated by adding 3 suspected volatile biomarkers to the breath of 2 healthy controls. In this case, the sensor's response to the healthy volunteers drifted significantly towards the values obtained for the lung cancer signatures, but they did not overlap. In a separate study, QCM sensors were used to analyze the content of the breath collected from 30 subjects who consented to the breath test.[597] Among them, 10 formed the control group and 20 subjects with radiological lesions were divided into adenocarcinoma (ADK) and squamous cell carcinoma (SCC) groups. Breath samples were collected both in the traditional manner using collection bags and by pulling excess air during bronchoscopy directly to the electronic nose. Two PLS-DA models were built, aiming to differentiate between LC patients and healthy volunteers, as also between LC histologies, *i.e.* ADK and SCC. Good capability of the sensor array for the discrimination of LC from healthy controls was achieved, both using the breath from the bags and the breath taken straight from the lungs, with the latter having better performance in terms of classification success. For LC histologic classification the PLS-DA model, based on breath taken from the lungs, provided a classification rate of 75%, which is much higher compared to that achieved by analyzing data collected from a breath bag.

6.1.1.4. Surface acoustic wave sensors. An electronic nose system composed of 2 SAW sensors working together as the detector, out of which one was coated with a polymer film of poly isobutylene and the other worked as a reference, in combination with improved ANN, was used for noninvasive diagnosis of lung cancer.[598] For this purpose, breath samples were collected from 42 volunteers, including 15 healthy subjects, 20 LC patients and 7 chronic bronchitis patients. SAW sensors were calibrated using 11 selected VOCs representative of LC detection as follows: a sample with none of the 11 VOCs corresponds to healthy subjects; if one of these VOCs appears in the sample, it is defined as suspected, and if more than one of the VOCs appear in the sample, the output is lung cancer. Breath samples were collected into 2 Tedlar bags, and were then absorbed on Solid-Phase Microextraction (SPME) fibers for pre-concentration. The extracted VOCs from the TD column were absorbed on the polymer-coated SAW sensor one by one, followed by recording 2 parameters: the frequency response (Hz) and the corresponding retention time. Results from the

Published on 11 June 2018. Downloaded on 2/1/2025 6:21:23 AM.

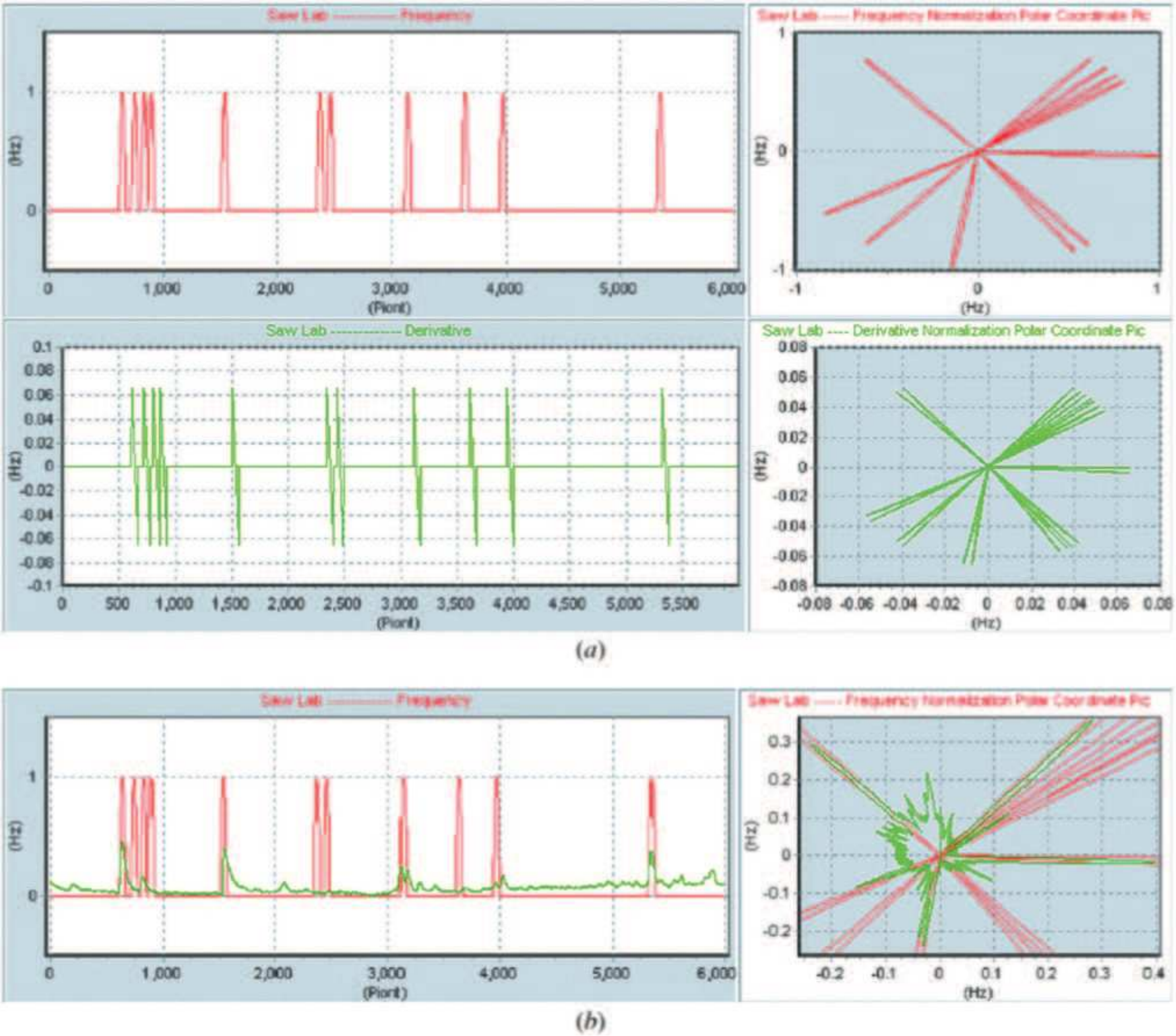

Fig. 33 An image method for lung cancer diagnosis based on a virtual SAW gas sensor array. (a) Ideal response of lung cancer patients, and (b) image response of a lung cancer patient overlapped with the ideal response. Reprinted from ref. 598 with permission from IOP Publishing, copyright 2005.

SAW sensors were translated into 6000 virtual sensor array responses. For patient diagnosis, an image method that combines frequency responses and retention times was applied together with BP-ANN (Fig. 33). For prediction purposes, breath samples from 5 LC patients and 5 healthy volunteers were collected and analyzed by BP-ANN. Results of this blind test showed that 8 of the patients were correctly classified, with one LC patient and one healthy subject being classified as suspect.

6.1.1.5. Colorimetric sensor array. In Mazzone *et al.*,[599] a colorimetric sensor array was designed for noninvasive LC detection through exhaled breath. NSCLC patients (n = 49), controls bearing different pulmonary diseases (n = 73) including COPD, sarcoidosis, pulmonary arterial hypertension and IPF, and healthy volunteers (n = 21) were enrolled. Each colorimetric sensor array was composed of 36 chemically sensitive spots with different sensitivities to VOCs. Each sensor in the array responded in a change in color, which was further analyzed into its RGB components and then to numerical values. Data collected from the sensor array were analyzed by the random forest method using 70% of the data as training, which resulted in 73.3% sensitivity and 72.4% specificity for the diagnosis of LC. In a following study, the contents of exhaled breath taken from 229 volunteers, divided into biopsy-proven untreated LC patients, a control group of individuals at high risk to develop the disease, and individuals with indeterminate lung nodules, were examined using a colorimetric sensor array.[600] Breath signatures of 8 binary groups were compared for identification and characterization of lung cancer with high sensitivities and specificities of >70%. Models developed for discriminating between LC histologies had higher accuracy (90% sensitivity) than those aiming to compare LC patients and healthy

Published on 11 June 2018. Downloaded on 2/1/2025 6:21:23 AM.

volunteers (70% sensitivity). Furthermore, patient characteristics had no influence on the accuracies of the models comparing different features of LC (histology, stage and survival).

6.1.2. Mesothelioma

6.1.2.1. Polymer composite sensors. In a study using Cyranose 320, the detection of mesothelioma (an aggressive cancer mainly related to asbestos exposure) was reported.[601] For this purpose, breath samples were collected from 42 healthy people, 20 volunteers with malignant mesothelioma (MM) approved on the basis of immunohistological diagnosis, 5 volunteers with asbestosis, and 13 with other pleural diseases. The study was conducted in 2 phases: a training phase used to build the model based on PCA analysis, and a blind phase to validate the strength of the model. The Mahalanobis distance between the mean of the groups was also calculated. The results of the training set, which included 10 MM and 10 controls, showed 95% accuracy and a Mahalanobis distance of 4.59. Blind test results (including 10 MM and 32 healthy controls) reached 90% sensitivity and 91% specificity.

6.1.3. Breast cancer

6.1.3.1. Monolayer-capped metal nanoparticle- and carbon nanotube-based sensors. Using an array of cross-reactive GNPs, it was possible to discriminate between 36 female volunteers: 7 were with negative mammography, 16 with benign breast conditions, and 13 women with malignant breast lesions.[602] The first principal component could discriminate between healthy volunteers and women with breast cancer, as well as between women who were negative in mammography and women with benign or malignant lesions. Statistical differences in the tested group were also evaluated by applying Student's t-test for PC1, giving $p < 0.05$ for all comparisons. SVM classification provided 94% sensitivity and 80% specificity for breast cancer screening. In a complementary study from the same group, 276 breast samples were collected from healthy volunteers, women with benign breast lesions and women with breast cancer, characterized by different molecular subtypes.[603] DFA analysis of responses obtained from the GNP sensor array indicated 83% accuracy in the blind test for discriminating breast cancer from non-cancer cases (including healthy volunteers, benign lesions and DCIS). The DFA model sensitivities and specificities for breast cancer molecular subtypes (*viz.* Luminal A, Luminal B, Triple Negative, HER2+, HER2 equivocal) vary from 81 to 88%, and 76 to 96%, respectively.

6.1.4. Ovarian Cancer

6.1.4.1. Monolayer-capped metal nanoparticle-based sensors. An array of 6 modified GNP sensors was used to discriminate between 48 women bearing positive OC, 86 women with benign genital tract neoplasia and 48 tumor-free controls.[203] In this study, the DFA algorithm was applied for multiple binary comparisons of OC compared to non-cancer subjects, and to OC compared to tumor-free cases. Furthermore, early stages of OC were compared to advanced OC, as also to tumor-free volunteers. Classification success of the DFA models for the nanoarray analysis provided accuracies varying between 75 and 90% for the training run, and between 58 and 89% for the blind tests. Furthermore, diagnosis of OC from exhaled breath has recently been accomplished using a dynamic integrated array of flexible sensors based on modified GNPs.[156] For this purpose, breath samples were collected from 43 subjects: 26 were healthy controls and 17 were women previously diagnosed with epithelial OC. Since each sensor in the array can be bent multiple times, it was possible to extract numerous bending-related features from each sensor separately; hence, each sensor acted as a stand-alone array. As a result 82.3% sensitivity, 84.6% specificity and 83.7% accuracy were achieved by applying leave-one-out cross-validation on the DFA model calculated from only single sensor responses. Further verification of the results was obtained by applying k-Fold ($k = 4$) analysis, which gave sensitivity, specificity and accuracy $>80\%$.

6.1.5. Gastric cancer

6.1.5.1. Monolayer-capped metal nanoparticle- and carbon nanotube-based sensors. Monolayer capped GNPs were used to distinguish between GC patients ($n = 37$) and benign gastric conditions ($n = 93$), and between early ($n = 17$) and advanced stage ($n = 18$) GC.[604] Among the benign conditions, patients suffering from ulcers ($n = 32$) were distinguished from those with less severe conditions ($n = 61$). For the first comparison, DFA analysis of the responses obtained from the GNP array resulted in 89% sensitivity and 90% specificity. Comparison of the different stages of GC gave 89% sensitivity and 96% specificity in a blind test. Finally, the volatolomic signature of the benign conditions was 86% accurate. In a following research exercise, an array of artificially intelligent GNPs and SWCNTs coated with different organic ligands successfully distinguished binary comparisons of breath signatures collected from GC patients ($n = 99$; 36 at stages I–II, 59 at stages III–IV), pre-malignant lesion patients ($n = 53$), 7 patients with low grade dysplasia, and 325 cases with the following operative link on gastric intestinal metaplasia (OLGIM) distribution: 155-OLGIM 0, 136-OLGIM I–II and 34 OLGIM III–IV.[605] ROC curves based on canonical variables of the DFA model were plotted and gave calculated AUCs of 76–98% in the different binary comparisons. Potential confounding factors – including *H. pylori*, gender, age, smoking habits and alcohol consumption – were also tested to validate the strength of the calculated DFA model. TP, FN, TN and FP were also validated based on blind experiment. The high accuracies and specificities of this study demonstrated the ability of the artificially intelligent nanoarray based on GNPs and SWCNTs to serve as a clinical tool for GC detection and screening.

6.1.5.2. Silicon nanowire-based field effect transistor. Breath samples were collected and analyzed from 30 GC patients divided into patients at early stage of the disease ($n = 6$), late stage ($n = 13$), and unknown stages of the disease ($n = 11$).[452] The control group included 77 breath samples from 23 patients with early intestinal metaplasia, 9 with ulcer and 45 volunteers with healthy stomachs. Breath signatures were compared by means of molecularly modified SiNW FET. A training set of only one sensor resulted in 87% sensitivity, 81% specificity and 83% accuracy after applying the DFA algorithm for discriminating GC from the control group. The blind test resulted in 71% sensitivity, 89% specificity and 85% accuracy. For the discrimination

This journal is © The Royal Society of Chemistry 2018

Published on 11 June 2018. Downloaded on 2/1/2025 6:21:23 AM.

between early and advanced stages, sensitivity and specificity of 92% and 67%, respectively, were achieved. The influence of the presence of *H. pylori* and smoking habits on breath signatures was also tested, but found to be negligible.

In another study, breath samples collected from 374 volunteers included 129 healthy control, 149 LC patients that were further divided into early and advanced stages, 40 GC patients also divided into early and advanced stages, and 56 patients having asthma and COPD (AC).[453] Signals from the SiNW FET array were analyzed by both the ANN and DFA algorithms. ANN analysis for the comparison of LC and the control group yielded high sensitivity, specificity and accuracy (all >80%). Comparison of GC and the control group gave 87% sensitivity, 98% specificity and 95% accuracy using a different SiNW. Furthermore, the third group of AC that served as either rule-in or rule-out to LC, resulted in 81% specificity and 75% sensitivity. For discrimination between LC and AC a high sensitivity of 92% was observed. An exceptionally high sensitivity of 98% was achieved for discrimination between GC and AC. ANN results were further validated by applying DFA to the same set of binary comparisons.

6.1.6. Colorectal cancer

6.1.6.1. Monolayer-capped metal nanoparticle-based sensors. For the purpose of using GNPs for detecting colorectal cancer (CRC), breath samples were collected from 122 controls, 65 volunteers having CRC that had been confirmed histologically, and 22 classified with advanced adenoma (AA), having adenomatous polyps.[606] Five different DFA models were calculated for 5 separate comparisons: (1) CRC *vs.* control; (2) CRC *vs.* AA; (3) AA *vs.* control; (4) non-advanced AA *vs.* advanced AA; and (5) AA *vs.* the control group. Accuracies in all 5 comparisons were >90% both in the training and the blind set. The effects of age, gender, smoking habits and fasting state were examined, but had no influence on the breath signature.

6.1.7. Squamous cell carcinoma of the head and neck

6.1.7.1. Monolayer-capped metal nanoparticle- and carbon nanotube-based sensors. For the purpose of detection, screening and staging of squamous cell carcinoma of the head and neck (HNSCS), breath samples were collected from 87 volunteers and analyzed by means of an artificial intelligence nanoarray composed of chemically modified GNPs.[607] The study population included 22 patients diagnosed with HNSCC, divided into early and late staging, and also into different tumor locations, *i.e.* larynx or pharynx. Breath samples were also collected from 21 volunteers with benign lesions and 19 healthy subjects. Sensor responses were further analyzed by the DFA model for the design of 5 volatolomic maps. For HNSCC detection and screening sensitivity, specificity and accuracy of 77, 90 and 83%, respectively, were achieved. For HNSCC differential staging of early HNSCC compared to late HNSCC as well as for the comparison based on tumor location, high sensitivities, specificities and accuracies of >90% were reported.

6.1.7.2. Metal oxide sensors. In a different study aimed at enrolling metal oxide-based sensors for HNSCC screening applications, breath samples were collected from 36 cancerous patients and 23 volunteers with different benign conditions.[608] MOs, consisting of triplicates of 4 sensor types (CH_4, CO, NO_x and Pt) were used. Data collected from the sensors analyzed by logistic regression modeling gave 3 false negative and 4 false positive classifications. The model achieved a sensitivity of 90% and a specificity of 80%.

6.2. Pulmonary arterial hypertension

Accumulating evidence points to survival rates and clinical outcomes being significantly enhanced when pulmonary arterial hypertension is diagnosed at its early stages. Nevertheless, it remains a major clinical challenge and relies on an invasive right heart catheterization procedure. The complex pathologies involved in pulmonary arterial hypertension could result in a wide range of biomarkers in the breath. Indeed, there are alterations in the breath volatolome among PAH patients.

6.2.1. Monolayer-capped metal nanoparticle-based sensors. It has been reported that a proof-of-concept study in which an array of GNP-based sensors was used for breath volatolome analysis and successfully detected and classified pulmonary arterial hypertension cases (22 patients and 23 healthy controls).[609] The combined signals obtained from the sensors after exposure were significantly different in samples from each group. After a supervised DFA analysis, a receiver operating characteristic (ROC)-based classifier discriminated between the samples (pulmonary arterial hypertension *vs.* controls) with an accuracy of 92%. A continuation analysis aimed at detecting heritable pulmonary arterial hypertension cases diagnosed a mutation in the bone morphogenetic protein receptor type 2 (BMPR2) gene.[610] The classifier successfully pointed out heritable pulmonary arterial hypertension (n = 7) compared to idiopathic PAH (n = 15), scoring an overall accuracy of 87%. A third model showed a correlation between the signals obtained from the sensor array and the severity of the patient's disease. With 91% accuracy, the samples were rated according to the New York Heart Association severity classification. However, the sample size in this case was relatively small and validation will be required. Currently, a clinical trial for validation (and expansion) is ongoing with a goal of recruiting 400 volunteers, including idiopathic pulmonary arterial hypertension patients, heritable pulmonary arterial hypertension patients, high risk subjects without pulmonary arterial hypertension (asymptomatic relatives of familial pulmonary arterial hypertension cases carrying a BMPR2 mutation) and chronic thromboembolic pulmonary hypertension patients (ClinicalTrials.gov Identifier NCT02782026).

6.2.2. Polymer composite sensors. With similarity in concept, pulmonary arterial hypertension patients were included among a wide group of controls (lung diseases) in a study testing the feasibility of the Cyranose 320 system to detect LC and discriminate it from other common lung diseases.[594] The system was composed of 32 polymer composite sensors, a sampling system, a data acquisition system and a processor (Smiths Detection, Pasadena, CA). For the training phase, the exhaled breath samples of 14 individuals with bronchogenic carcinoma and 45 healthy control subjects were analyzed. PCA and SVM models were obtained and optimized for discrimination

Published on 11 June 2018. Downloaded on 2/1/2025 6:21:23 AM.

between cancer and non-cancer cases. The models were applied to a second independent set of 76 individuals, 14 with and 62 without cancer (including pulmonary arterial hypertension patients). In the validation study, the electronic nose gave 71.4% sensitivity and 91.9% specificity for detecting lung cancer. Positive and negative predictive values were 66.6 and 93.4%, respectively. In this population with a lung cancer prevalence of 18%, positive and negative predictive values were 66.6 and 94.5%, respectively. The authors used GCMS only on 8 LC patients to understand the nature of the composition of the VOC profiles. A similar study, however, was not conducted for other groups, and quantitative comparison between the diseases was not carried out.

6.2.3. Colorimetric sensors. Mazzone *et al.*[599] enrolled 20 pulmonary arterial hypertension patients as a control group to test the feasibility of a chemical-colorimetric sensor array for volatolome collective assessment in the diagnosis of lung cancer (LC). This array is composed of group of 36 spots, containing different chemically sensitive compounds (*e.g.*, metalloporphyrins) impregnated on a disposable cartridge. The colors of these spots change based on the chemicals with which they come into contact. After exposure to breath samples, the changes in color are converted into numerical values for the red, green and blue components of each spot for each scan. This resulted in a 108-dimensional vector (36 spots, 3 values per spot). Random forest analysis was used to explore the discriminative feasibility of the method. The results indicated that the collective breath volatolome of LC patients (n = 49) differed from pulmonary arterial hypertension, idiopathic pulmonary fibrosis (IPF) (n = 15), COPD (n = 18), sarcoidosis (n = 20) and healthy controls (n = 21). Once trained, the sensor array could discriminate between the diseases with a sensitivity of 73.3% and a specificity of 72.4%. The results were not influenced by gender, age, histology or smoking history. In this case, the authors used no complementary analysis for the identification and quantification of the volatile biomarkers that they measured.

6.3. Heart failure

6.3.1. Metal oxide sensors. In a study focused on heart failure (HF), an array of 3 MO-based gas sensors was used to analyze the exhaled breath of 13 patients with decompensated HF (DHF), 16 patients of compensated HF (CHF), and 13 controls without HF (CON).[611] Each of the sensors was modified with a different thin oxide layer with different sensitivity and selectivity for certain gas molecules at different temperatures. For each patient, 10 measurements over 35 min were taken from a 500 mL single breath sample collected from each patient. Three binary DFA classifiers were calculated from the normalized sensor responses. DHF and CHF could be discriminated with an accuracy of 91%, and between CON and CHF it was 97%. The best result was in the discrimination between CON and DHF (100%), but it is of a lesser medical significance.

6.4. Intestinal diseases

6.4.1. Monolayer-capped metal nanoparticle-based sensors. An array of GNP based sensors was trained and programmed for the detection of inflammatory bowel diseases and irritable bowel syndrome using breathprint.[201] Two breath samples were collected from 36 Crohn's disease (CD) and 35 ulcerative colitis (UC) patients – a total of 71 inflammatory bowel disease (IBD) samples – and 26 irritable bowel syndrome (IBS) patients. The first set of samples was analyzed by GCMS to pinpoint the VOCs with discriminative power between the different groups. The "artificial samples" were generated by a gas generator system; these samples, consisting of mixtures of the VOCs corresponding to the significant differences between the study groups, were used as the artificially intelligent training phase for the sensor array. After exposure to the artificial breath mixtures, the most sensitive sensors were chosen for ANN analysis as a training pre-clinical phase. The clinical performance of the chosen sensors was put to the test by analysis of 97 clinical breath samples. In the 6-fold validation method, 92% sensitivity (PPV of 85%), 53% specificity and a total accuracy of 81% when targeting the IBD group were achieved. The area under ROC test was 70%. The same planning and execution strategies were used to discriminate between CD and UC cases. After the training phase with artificial mixtures resulted in an accuracy of 96%, the model was tested with clinical breath samples, giving 75% sensitivity (targeting CD group), 47% specificity and 75% total accuracy, with an area under ROC of 72%.

6.5. Renal diseases

6.5.1. Monolayer-capped metal nanoparticle- and carbon nanotube-based sensors. Four functionalized spherical GNP sensors (out of an array of 20 different sensors) were used to detect chronic kidney disease and disease progression from an analysis of exhaled breath from 62 volunteers.[612] These sensors were chemically modified with 2-ethylhexanethiol, *tert*-dodecanethiol, hexanethiol or dibutyl disulfide as sensing layers, and were used to analyze the exhaled breath of 20 healthy controls and 42 chronic kidney disease (CKD) patients diagnosed at different stages (severity) of the disease, ranging from early stages to end stage failure. Using PCA followed by SVM and leave-one-out cross-validation (LOOCV), the authors used 4 sensing features extracted from 2 sensors to discriminate between healthy controls and early stage CKD patients, giving an accuracy of 79%. Employing a second algorithm, based on using 5 sensing features extracted from 3 sensors, discrimination between advanced stages and end stage CKD was achieved with an accuracy of 85%. Moreover, the authors showed a clear correlation between the sensing signal of a single 2-ethylhexanethiol sensor and the estimated glomerular filtration rate, one of the main clinical indications for renal function. These signals significantly correlated with transition from early-stage to late-stage CKD. This work was supported by quantitative analysis by GCMS indicating disease and/or severity associated volatolomic alterations in the exhaled breath of the patients. Fifteen different compounds were identified in the disease, or in specific stages of the disease progression.

In a continuation study, these authors tested the feasibility of the sensor array, this time including an additional set of random networks of single-walled carbon nanotubes capped

with organic films for identifying patterns of exhaled VOCs of end-stage renal disease (ESRD) and studying the impact of hemodialysis (HD).[613] Exhaled breath samples of the 37 participants (26 ESRD patients treated with HD and 11 healthy subjects) were analyzed using the sensor array, and DFA algorithms were obtained to specify the informative volatolomic patterns. First the DFA model was applied on 80% of the database as a training phase, whereas the remaining samples were used for the validation of the developed model. Including the responses of the 3 sensors (Au NPs with dodecanethiol, gold nanoparticles with *tert*-dodecanethiol, and SWCNTs with sulfated β-cyclodextrin), the model showed that the breath pattern of ESRD patients was distinguishable from controls with an accuracy of 80%. Moreover, the responses of the sensors showed significant variance during HD therapy. Seventeen to twenty breath samples were tested before and 1, 2, 3 and 4 h after the beginning of HD, and showed significant changes along with treatment. Once again, the authors supported their results with quantitative analysis of a second set of breath samples obtained from each patient during the same time intervals, showing that 7 VOCs were altered in patients at ESRD, whereas the concentrations of 2 of them were significantly reduced after 4 h of treatment.

In parallel to the previous 2 clinical studies, the feasibility of the sensor array for the early detection of acute and chronic kidney failure was also tested in an animal model.[240,614] In one study, the volatolomic signature was assessed as a function of time after inducing kidney failure in an ischemic injury model in rats. This was compared to blood and urine biomarkers routinely used in the clinical world. The signature obtained from 3 GNP-based sensors with 3 different organic functionalities – dodecanthiol, octadecanethiol, and 4-chlorobenzenemethanethiol – was altered 1 h after AKI induction, whereas urinary neutrophil gelatinase-associated lipocalin and serum creatinine levels were increased after 2 h. These trends were explained by GCMS analysis showing an accumulation in a specific range of VOCs in the exhaled breath shortly (T1–T4) after disease induction.[614] In a second pre-clinical study, a combination of 10 chemiresistive random network of SWCNTs coated with organic materials, in combination with pattern recognition methods, was used to detect the pattern changes in rats that had undergone induction of CKD. First, the sensors were trained and optimized by exposure to artificial gas mixtures corresponding to the statistically validated VOCs identified by GCMS analysis. The sensors were exposed to 10 breath samples of sham control rats and 7 CKD rats. PCA analysis showed a clear separation between the 2 clusters, corresponding to the groups and indicating 100% detection accuracy.[240]

6.6. Neurological diseases

6.6.1. Parkinson's and Alzheimer's diseases. Diagnosis of Parkinson's disease (PD) is based mainly on the presence of clinical motor symptoms of the disease, which usually appear after the loss of 50–70% of the target dopaminergic neurons in the substantia nigra pars compacta. Non-invasive and objective early detection could reduce misdiagnosis rates and promote early neuroprotective therapy, thus improving clinical outcomes.

6.6.1.1. Monolayer-capped metal nanoparticle- and carbon nanotube-based sensors. To investigate whether damage to the dopaminergic neurons could produce specific alterations in the volatolomic signature, presymptomatic PD in rats was induced and their exhaled breath analyzed by an array of organically functionalized random networks of SWCNTs (RN-CNTs).[615] Dopaminergic neurons were destroyed by the neurotoxin, 6-hydroxydopamine (6-OHDA), which is taken up selectively by neurons expressing a catecholamine transporter, resulting in loss of 55% of dopamine in the striatal tissue homogenate compared to control animals (10 lesioned and 9 sham-operated controls). LOOCV analysis of the DFA algorithm on the output of RN-CNTs functionalized with a layer of α-cyclodextrin or a layer of β-cyclodextrin resulted in a sensitivity, specificity, and accuracy of 80, 100 and 90%, respectively.

A further investigation of a similar model compared rats with mild and severe lesions of dopamine neurons (50 and 70% dopaminergic denervation; n = 8–11 rats per group).[616] In this study, an array of sensors composed of those based on GNPs or SWCNTs, organically functionalized with 4-methoxy-toluenethiol or dibutyl-disulfide, was used to qualitatively analyze breath samples. DFA applied to the responses to exhaled breath samples obtained from a training set of 6-OHDA-lesioned rats showed a clear difference between all the 6-OHDA rats and sham-operated controls (sensitivity, specificity, and accuracy >80%). A difference was also seen between the responses to exhaled air of 5,7-dihydroxytryptamine (DHT)-lesioned rats and controls, which was less clear than that between 6-OHDA and controls (sensitivity 70, specificity 100, and accuracy 84%), as well as between the 5,7-DHT-lesioned rats and all the 6-OHDA lesioned rats (sensitivity 89, specificity 100, accuracy 93%). The exhaled breath of the Sprague-Dawley transgenic rat (Tg) model carrying the human A53T SNCA mutation (n = 8) was analyzed as an additional Parkinsonian model. An array of CNT functionalized with β-cyclodextrin sensors was used to compare the breath signatures of Tg rats and WT controls (n = 10). The analysis gave a distinctive power of sensitivity, specificity, and accuracy of 100, 90 and 94%, respectively (LOOCV). These last results were supported by complementary analysis by GCMS of VOCs obtained from both blood and striatal homogenate samples, which showed a wide range of significant differences in their abundance of VOCs.

A case–control study assessed the use of an array of spherical GNPs (core diameter 3–4 nm) and SWCNTs to detect and classify Parkinsonian cases based on analysis of their exhaled breath.[617] The GNP sensors were organically functionalized with different sensing layers, such as dodecanethiol, 1-dodecanethiol, 4-chlorobenzene methanethiol, whereas the SWCNT-based sensors were organically functionalized with PAH derivatives. In this study, breath samples of 44 idiopathic PD (iPD), 16 non-iPD patients (atypical Parkinsonism) and 37 healthy controls were collected and analyzed. First, comparison of non-iPD with iPD states yielded 88% sensitivity, 88% accuracy and 88% ROC-AUC in the training set samples with known identity. *K*-Fold cross-validation analysis of this comparison scored 81% sensitivity and accuracy, and a 92% negative predictive value.

Published on 11 June 2018. Downloaded on 2/1/2025 6:21:23 AM.

Published on 11 June 2018. Downloaded on 2/1/2025 6:21:23 AM.

A second comparison between atypical Parkinsonism states and healthy subjects scored 94% sensitivity and 85% accuracy in the training set samples with known identity. The validation set of this comparison scored 81% sensitivity and 78% accuracy. The results were unaffected by L-dopa or monoamine oxidase B (MAO-B) inhibitor treatment (AUC = 56% and 59%, respectively). The results were explained by GCMS analysis, which showed significant variations in the concentrations of 4 VOCs in the exhaled breath of these groups.

In a second study, the same system was tested for the potential use of the discrimination between 2 major neurodegenerative disorders, Parkinson's and Alzheimer's disease.[618] Similarly, an array of 2-mercapto-benzoxazole-capped GNPs, 3-mercapto-propionate-capped GNPs and α-cyclodextrin, β-cyclodextrin, carboxy-methylated β-cyclodextrin, hydroxypropyl-β-cyclodextrin or heptakis(2,3,6-tri-*O*-methyl)-β-cyclodextrin modified SWCNTs was used to analyze alveolar breath collected from 57 volunteers (AD patients, PD patients and healthy controls). DFA applied to the responses of different combinations of the sensors could clearly distinguish AD from healthy states, PD from healthy states, and AD from PD states, with a classification accuracy of 85, 78 and 84%, respectively. Supportive GCMS analysis of a second set of breath samples from each subject showed that a range of 24 different VOCs was statistically varied between the study groups.

6.6.1.2. Polymer composite sensors. With the same goal, Bach *et al.* used the Cyranose 320 (C-320) eNose (Smiths Detection Group Ltd, Watford, UK) composed of 32 conducting chemiresistors made from carbon black nanocomposites that change their resistance in response to VOCs.[619] Breath samples were collected from 39 AD, 16 PD, 35 controls and several binary classifiers were obtained by pattern recognition algorithms. Using LOOCV, a sensitivity of 50% and a specificity of 77% were calculated in discriminating between AD and controls. Moreover, the authors reported that a second model was built to discriminate between all 3 groups, in which, after a training phase, an independent group of samples (including 21 AD and 16 controls) were used as a validation set. This resulted in a sensitivity of 69.8% and a specificity of 68.7%. They also used ion mobility spectrometry to explore the quantitative shifts in the composition of exhaled breath. Using a decision tree with 4 VOCs, the method showed a considerably higher accuracy of 94% (95%; CI 88–100) in which only 3 patients were misclassified out of a total of 53 participants.

6.6.1.3. Metal oxide sensors. Other studies have focused on the detection of Alzheimer's disease by breath analysis as a potential method of early diagnosis. For example, using an array of metal oxide semiconductor sensors (iAQ-2000 by Applied-Sensor, Warren, NJ), Mazzatenta *et al.*[620] analyzed the exhaled breath of 59 volunteers, divided into neurodegenerative (n = 15, age range 59–95 years) and healthy (n = 44, age range 19–105 years) subjects. The VOC fingerprint of AD is significantly different from the healthy ones, as was the frequency distribution of VOCs in both AD and control subjects. The fit of the VOC frequency distribution returned 2 distinct Gaussian peaks for the AD (r_2 = 0.56) and healthy subjects (r_2 = 0.98) (Fig. 34a and b). In another study, 11 metal oxide semiconductor sensors were used to analyze 60 breath samples from 40 AD and PD patient groups, and 20 healthy individuals, collected at Kyungpook National University Hospital.[621] Separated into 2 arrays, the authors used the following MOX based gas sensors: TGS 2600, TGS 2602, TGS 2611, TGS 2620, TGS 2610 (Figaro, Osaka, Japan), and MICS 5135, MICS 5132, MICS 2610, and MICS 2610 (e2V Technologies, Chelmsford, Essex, UK). After exposure of the breath samples to the sensors, it was clear that the normalized responses of the sensitivity characteristics for the gas sensors individually in each system were significantly different between the AD, the PD, and the control groups (Fig. 34c). Using different combinations of sensors, clustering analysis of the exhaled samples showed that the AD, PD, and control groups fell into 3 clusters by both sensor arrays. The results demonstrate that the control group could be distinguished from the AD and PD patients based on the PCA and Sammon's mapping methods, with a confidence limit of 95%. A complementary analysis by GCMS analysis of breath samples, to explain the performance of the sensors, showed significant alterations in 5 VOCs, associated with these diseases.

6.6.2. Multiple sclerosis. Multiple Sclerosis (MS) is the most widespread chronic neurological disease affecting young adults. Commonly the disease outbreaks at 20–50 years of age, and is approximately 3 times more frequent in women than in men.[622,623] MS diagnostics is based on clinical characteristics and commonly supported by Magnetic Resonance Imaging (MRI) as a complementary test. A number of VOCs are believed to be present in breath samples, including acetophenone, heptadecane, nonanal, *n*-decanal, and sulfur dioxide.[200] One explanation for the pathogenesis of MS is the toxic effect on the central nervous system of oxidative stress, which is one of the main causes of VOC formation in diseased states.[200]

6.6.2.1. Monolayer-capped metal nanoparticle- and carbon nanotube-based sensors. In two separate follow-up studies, researchers have reported on a homemade e-nose system (NaNose) for MS diagnosis from breath analysis.[200,624] The first study used only sensors based on bilayers of PAHs and SWCNTs (Fig. 35).[624] Data analysis of 51 breath samples showed 80.4% accuracy in classification of MS and healthy people based on a 4-sensor analysis. The second study used a combination of 6 gold nanoparticle sensors combined with 2 PAH-based carbon nanotube sensors.[200] In the latter study, 204 people were sampled in 2 phases (training set and blind set), and subcategories were evaluated for diagnostic purposes. Using ANN analysis, the sensor data allowed discrimination between MS patients and controls, with accuracies up to 90% in the training sets. Blinded sets showed a 95% positive predictive value between MS-remission and control, 86% NPV between relapse and control, and 100% sensitivity with 100% NPV between MS untreated and controls.[200] The authors tested the effect of confounding factors, such as smoking and gender; the results show that the sensor system was not influenced by them. Furthermore, potential biomarkers

Published on 11 June 2018. Downloaded on 2/1/2025 6:21:23 AM.

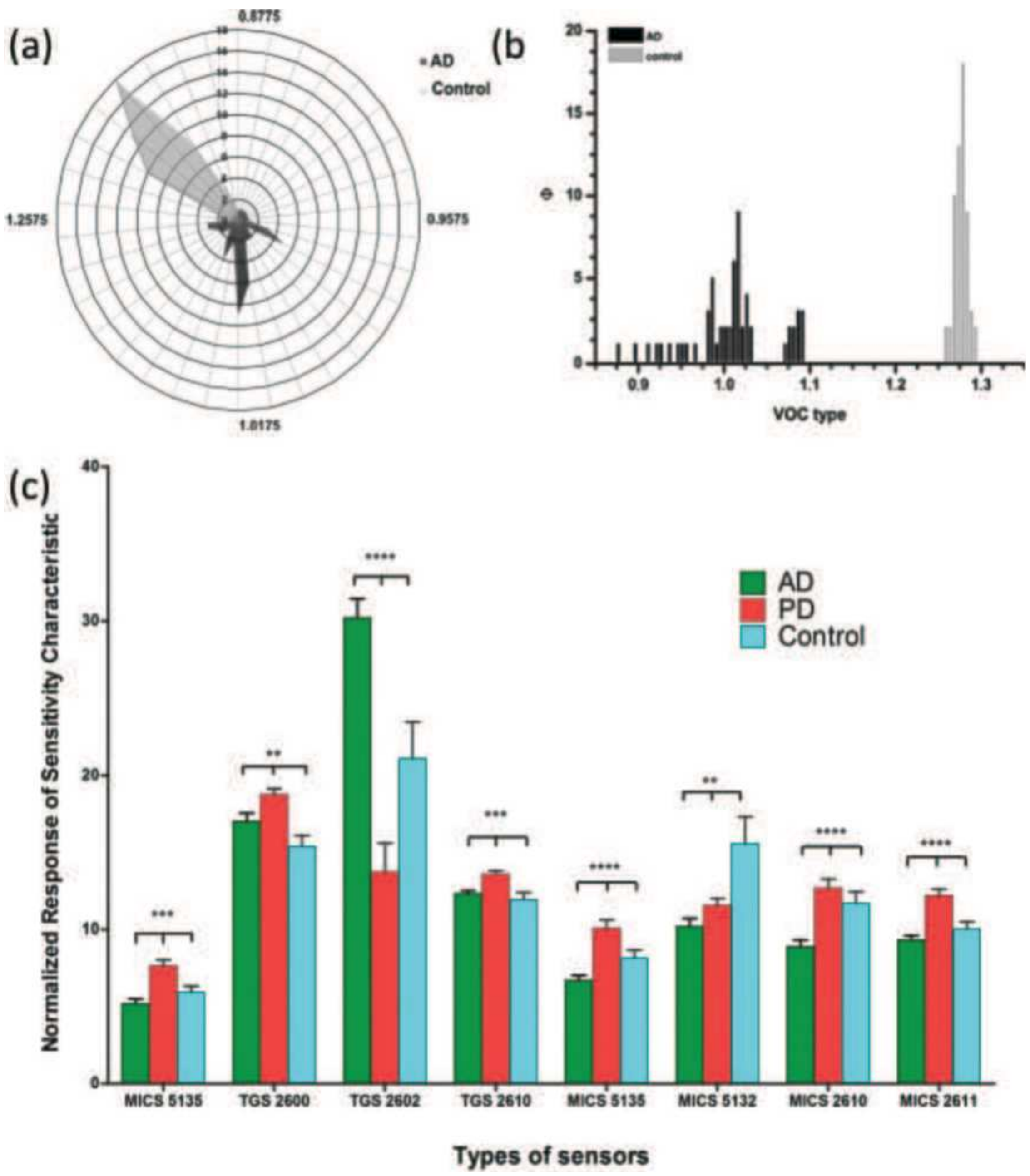

Fig. 34 VOC fingerprint of Alzheimer's disease (AD) and Parkinson's disease (PD) using 2 different MO sensor systems. (a and b) Characterized by a cluster of signals (dark gray) generated by VOCs' relative abundance significantly different from the healthy one (light gray), $p < 0.5$. (a) VOCs' frequency in both AD (fit $r_2 = 0.56$) and controls (fit $r_2 = 0.98$); (b) frequency distribution of VOCs in AD and control. Fit of the VOC frequency distribution shows 2 distinct Gaussian peaks for the AD ($r_2 = 0.56$) and healthy subjects ($r_2 = 0.98$). Reprinted from ref. 620 with permission from Elsevier, copyright 2015. (c) Normalized responses of the sensitivity characteristics for different types of gas sensors. Response sensitivity of exhaled breath samples from the patients with AD, PD and healthy controls. Reprinted from ref. 621 with permission from MDPI, copyrights 2017.

identified in parallel by mass spectrometry were used to evaluate the sensor response. The same array of sensors that achieved classification presented a linear correlated response to changing concentrations of the 3 tested biomarkers.[200]

6.7. Diabetes mellitus

Diabetes mellitus (DM) is an important chronic metabolic disease increasing globally and is truly endangering the social order. A greater number of people are afflicted every year worldwide by this disease due to high-calorie diet and sedentary lifestyle.[625] Based on a World Health Organization (WHO) global report, 422 million adults suffered from diabetes in 2014, compared to 108 million in 1980.[626] Eventually, if not controlled, DM could lead to heart attack, stroke, kidney failure, blindness, and amputations.[626] Due to the everyday discomfort of glucose blood sampling, researchers have been trying to find a non-invasive

Published on 11 June 2018. Downloaded on 2/1/2025 6:21:23 AM.

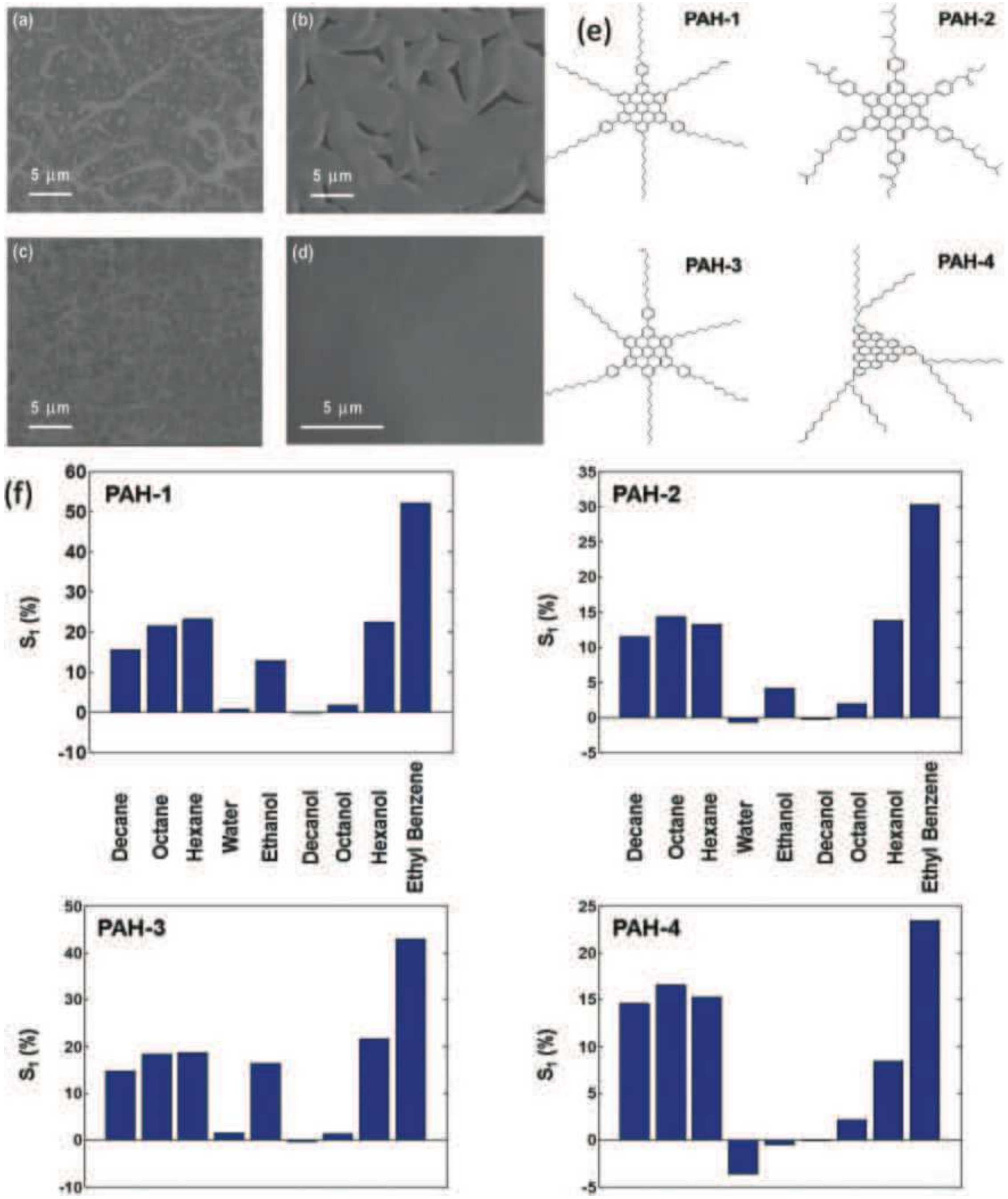

Fig. 35 Field emission scanning electron microscopy (FE-SEM) images of the sensing films made of PAH derivatives on top of random networks of SWCNTs: (a) PAH-1/SWCNT; (b) PAH-2/SWCNT; (c) PAH-3/SWCNT; and (d) PAH-4/SWCNT. (e) Discotic polycyclic aromatic hydrocarbon (PAH) derivatives employed in the study; (f) normalized change of sensor resistance in the middle of the exposure (S1) calculated from sensor responses to the 9 VOCs analyzed at p_a/p_0 = 0.5. Reprinted with permission from ref. 624. Copyright (2011) American Chemical Society.

way for diagnosing diabetes. Different VOCs can act as possible markers related to diabetes; for example, ketones are abundant in urine being the result of excessive fat oxidation by the liver; they are at high levels in diabetic patients.[8,25] In spite of the great interest, only a few sensor-based studies have tested urine VOCs for diabetes, whereas most used spectrometric-based methods.[627–629]

Mass spectrometry studies have shown that breath acetone can serve as a VOC biomarker of diabetes type I.[630] GC-MS analysis of breath samples from diabetic patients contained acetone breath levels at >1.71 ppmv, as opposed to acetone levels of healthy people (<0.76 ppmv).[82] However, other studies have clearly shown wide variations in breath acetone concentration due to diet and natural intra-individual biological variability. This places uncertainty in the use of acetone alone to monitor blood glucose.[631] Nevertheless, many different sensor studies examined acetone in breath sampling using sensors.

This journal is © The Royal Society of Chemistry 2018

Published on 11 June 2018. Downloaded on 2/1/2025 6:21:23 AM.

6.7.1. Metal oxide sensors. Some studies, including chemiresistors based on MO, were used to explore diabetes, although the sensors used were commercial ones not based on nanomaterials. Yan *et al.*[632] used a chamber with 11 sensors, comprising 6 ordinary MOS sensors, 3 temperature modulated MO sensors, a carbon dioxide sensor and a temperature–humidity sensor. First, a simulation experiment with acetone at 0.1 to 20 ppm was evaluated, along with spiked breath samples. The 295 healthy breath samples and 279 diabetic samples were collected and analyzed, and the sensitivity and specificity of diabetes screening were ∼92% and 91%, respectively. Prediction of blood glucose levels by the breath sample also showed moderate correlations.[632] Jaisutti *et al.*[633] developed room-temperature operable gas sensors based on Na-doped p-type ZnO nanoflowers as a potential sensor for diabetes detection. The sensing layer was activated by ultraviolet illumination and the sensors responded to acetone gas (100 ppm, UV intensity of 5 mW cm^{-2}) with a detection limit of 0.2 ppm at room temperature.[633] A FET sensor based on a 10 nm thick indium nitride (InN) layer as an acetone sensor achieved a detection limit of 400 ppb acetone at 200 °C.[31] This sensor was evaluated in the presence of varying concentrations of oxygen, which was done to simulate a response in real breath samples; it showed a good response to a mixture of oxygen and 2.4 ppm of acetone.[31] The sensors were not tested on real breath samples.

6.7.2. Conducting polymer sensors. A portable gas system using a conducting polymer (polypyrrole) sensor array was used as a potential system to analyze diabetic patients' breath.[634] Four polypyrrole thin film sensors of different thicknesses were used to detect acetone. Baseline was calibrated from dry air and voltage variation rates were measured after exposure to acetone. The sensors, initially exposed to acetone concentrations of 50 to 700 ppm, showed increasing responses, but these concentrations were higher than those expected in breath samples. Following this, a breath test was given to six individuals, 3 diabetics and 3 non-diabetics. Discrimination between their breath samples was achieved using the PCA algorithm.[634]

6.7.3. Optical fiber based sensors. Researchers are now developing a sensor device for acetone and isopropanol breath sampling based on an optical fiber probe.[635] Acetone and oxidized nicotinamide adenine dinucleotide (NADH to NAD+) can be reduced in a weak acid environment by secondary alcohol dehydrogenase. Excitation of NADH by 340 nm light will subsequently result in fluorescence emission at 490 nm. Thus, monitoring the decrease in NADH fluorescence intensity should measure acetone.[635] The sensor was tested on breath samples from 25 diabetic patients and 55 healthy controls (Fig. 36). The breath acetone in healthy people had a mean value of 750.0 ± 434.4 ppb, whereas diabetic patients showed a mean concentration value of 1207.7 ± 689.5 ppb. The acetone levels were not influenced by confounding factors, such as gender and age.[635]

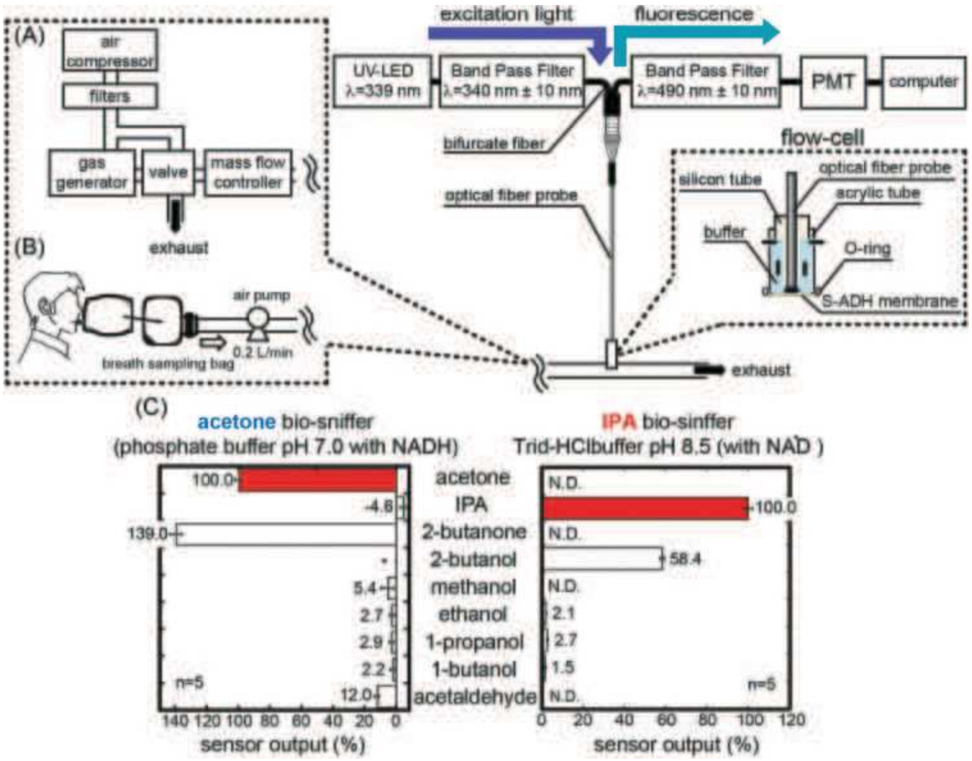

Fig. 36 (A) Schematic diagram of acetone and IPA (isopropanol) biosniffers. The inserted chart shows the structure of the flow-cell. (B) Schematic diagram of breath measurement using biosniffers. The sample breath was delivered to the biosniffer by an air pump, and the flow rate was 200 mL min^{-1}, which was the same as in the standard gas measurement. (C) Selectivity of acetone and IPA biosniffers. N.D.: none detected. *: 2-butanol was not measured by the acetone biosniffer. Reprinted (adapted) with permission from ref. 635. Copyright (2017) American Chemical Society.

Published on 11 June 2018. Downloaded on 2/1/2025 6:21:23 AM.

6.8. Tuberculosis

Diagnosis of TB remains a major global public health challenge. Although sophisticated and accurate diagnostics are now available, they are often non-accessible in TB stricken regions, where old-fashioned and non-accurate tests are still in use. Several reports have pointed out that alteration in exhaled VOCs is found both in cultures and TB patients, indicating that exhaled breath analysis holds the potential for non-invasive diagnostics of TB. Several nano-based sensor technologies have developed and put to the test.

6.8.1. Monolayer-capped metal nanoparticle- and carbon nanotube-based sensors. In a case–control study, 198 breath samples were analyzed by an array of sensors, including MCMNPs and molecularly-modified SWCNTs.[636] The study population consisted of 3 main groups: active TB (n = 64), controls (that included a group of healthy subjects (n = 67), and a group of suspected with TB, yet ruled out by negative smear microscopy, culture and GeneXpert MTB/RIF, n = 67). About 70% of the samples were used as a training set, with the remainder being used as a blind validation set for the sensors' analysis. Interestingly, several sensors gave a discriminative response to the different groups, yet a chemiresistor based on dodecanethiol-capped gold nanoparticles correctly classified 121 of the 138 training samples, with an accuracy of 88%, a sensitivity of 85%, and a specificity of 89%. In the validation set, the same sensor scored a sensitivity of 90%, a specificity of 93% and an accuracy of 92%. The sensor's function was unaffected by possible confounding factors including smoking habits, HIV infection and antibiotic treatment (for up to 2 weeks prior to testing; Fig. 37a). In another study of *Mycobacterium bovis* infected cattle, 6 GNP-based sensors were used to analyze breath samples obtained from 8 TB infected and 4 TB-negative from an infected dairy, and 10 animals from non-infected dairies. The sensors were functionalized with octadecanethiol, decanethiol, 2-naphthalenethiol, 2-mercaptobenzoazole or 2-nitro-4-trifluoromethylbenzenethiol as sensing layers. Applying the DFA algorithm to the responses gave sensitivity and specificity of 100% and 79%, respectively.[637]

6.8.2. Quartz crystal microbalance sensors. In another study, a sensor array made of 8 metalloporphyrin-coated QCM sensors was used to assess the exhaled volatolomic signature of TB patients during their treatment.[638] Exhaled breath samples of 51 TB patients and 20 controls were analyzed before and 2, 7, 14, and 30 days after therapy, which were correlated with clinical and microbiological measurements on sputum samples. Significant differences in the frequency shifts were measured by all 8 sensors when comparing between TB patients and controls at day 0. Pattern recognition analysis that included the signals of all sensors scored 93% accuracy (Fig. 37b). Nanomaterial-based sensors were also examined in cases of bovine TB.[639] An array of 9 MO-based sensors was used to analyze the headspace of cattle sera samples obtained from 11 TB infected and 10 TB-free cattle. DFA and PCA models were obtained using data from 4 sensors that had higher selectivity to oxidizing and flammable compounds, such as nitric oxide,

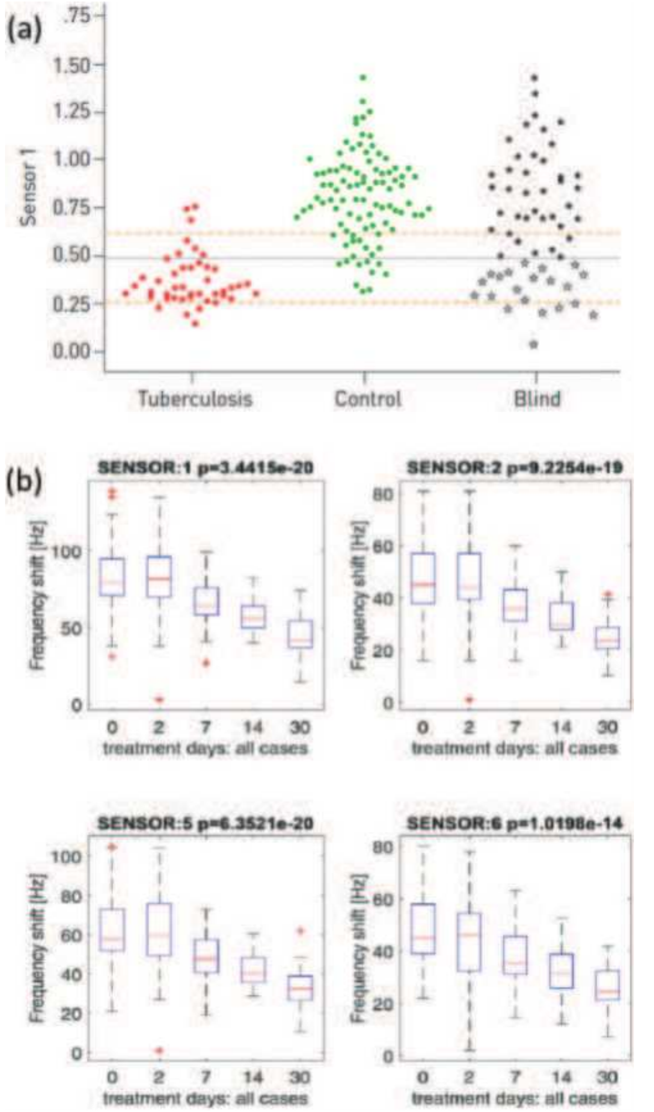

Fig. 37 (a) Dot-plots of the response of a single dodecanethiol-capped gold nanoparticle. Each circle/star represents a single sample. The central dashed line represents Youden's cut-point, and the outer dashed lines represent the cut-points to rule-in and rule-out tuberculosis. Samples from the validation set with responses lower than the threshold were classified as tuberculosis-positive (open stars) or non-tuberculosis-positive (closed stars) according to Youden's cut-point. Reproduced with permission from the ©ERS 2014.[636] (b) Distribution of quartz microbalance (QMB) gas sensors' signals among cases with pulmonary tuberculosis at baseline and on days 2, 7, 14 and 30 after treatment. Reprinted from ref. 638 with permission from Elsevier, copyright 2017.

hydrocarbons and alcohols. Both statistical models resulted in absolute discrimination between the samples.

7. Urine-based disease diagnostics

Urine is a dominant bodily fluid used for diagnostics. Urine diagnostics can be used for detection of bacterial infections, metabolic disorders, protein degradation products and renal dysfunction. It is a complex biofluid containing many components that are influenced by a number of factors, such as diet,

Published on 11 June 2018. Downloaded on 2/1/2025 6:21:23 AM.

age, gender, physical activity and health status.[640–642] Traditionally, clinical urine analysis involves detection of large metabolites directly in the liquid phase, for example, neutrophil gelatinase-associated lipocalin and kidney injury molecule-1 (KIM-1) in the case of acute kidney injury (AKI),[643] or leukocyte esterase urinary tract infection (UTI).[644] However, urine odors have been used for diagnostics for >2000 years ago[640] and recently analysis of VOCs from urine is regaining attention as diagnostic biomarkers.[8] Approximately 280 VOCs have been reported in different studies on healthy people.[21] Most VOCs in the urine are water-soluble and depend on renal filtration.[8] VOCs in urine include a number of chemical families *e.g.*, ketones, aldehydes, acids, amines and others. Other exogenous molecules arising from medical and non-medical drugs are secreted in urine and affect the VOC profile.[8]

7.1. Diabetes mellitus

7.1.1. Metal oxide sensors. Siyang *et al.*[645] reported a homemade e-nose for diabetes diagnosis, using a combination of 8 off-the-shelf MOS sensors. Sensors were commercially made for detection of different compounds, such as alcohol, ammonia and others. The study used 6 pure urine samples that were spiked with different concentrations of glucose from 0 to 2000 mg dL^{-1} and heated to different temperatures between 30 and 45 °C. Using PCA analysis, they showed separation of the control *vs.* high concentration samples at 45 °C, with the ammonia sensor being the most sensitive.

7.2. Malignant diseases of the urinary and genital systems

As discussed above, cancer diseases are widely studied and numerous researches have worked on the field of VOC analysis in cancer. Recent statistics from the United States estimate prostate cancer to be the leading in new cancer cases in 2017, with 19% of the new cases out of all the estimated cancer cases in 2017.[568] Urinary bladder and kidney cancers are the fourth and the sixth new cancer cases, 7% and 5%, respectively.[568] Cancers related to the urinary tract system, bladder and prostate have been conventionally detected by classical imaging techniques, such as ultrasound, computed tomography (CT), positron emission tomography and MRI. Recently there has been a rapid advance in those urological imaging techniques, such as MRI and fusion biopsy.[646] However, these techniques continue to be limited by tissue contrast resolution in MRI and determination of the optimal radiotracer in the case of positron emission tomography.[646] Most cancer cases lack early clinical symptoms and continue asymptomatic until late stages, when therapy options become limited.[647] In recent years, such cancers have been the target for VOC based analysis from urine samples using GC-MS and sensor systems.[647–651]

7.2.1. Metal oxide sensors. Weber *et al.* used a set of commercial 12 MOS and 10 MOS-FET sensors incorporated into a NST 3320 Lab Emission Analyser.[649] The system includes a capacitance-based humidity sensor and an infrared-based CO_2 sensor. The trial included urine samples from 30 patients with bladder cancer (transitional cell carcinoma) prior to surgical intervention, and 59 control people. Data were analyzed using PLS-DA, initially on a training set, that resulted in the best classification between cancerous samples and healthy samples with 70% accuracy. Discrimination of bladder cancer from a control group which included healthy and non-cancerous bladder diseases resulted in a lower accuracy of 65%. Finally, the authors concluded that the tested commercial e-nose is not the best choice for these diagnostics.[649]

A pilot study done by Horstmann *et al.* tested an e-nose with 3 different MOS sensors at 3 temperatures. They tested urine samples from 15 patients with suspected bladder cancer and 21 controls with non-cancerous urological conditions.[652] Using PCA and DA, the authors achieved an overall sensitivity of 75% and specificity of 86% in classifying cancerous *vs.* non-cancerous samples.[652] Since this reference is published in short-note format, the authors did not provide any detailed information on the sensors used or their set-up, making it difficult to evaluate their findings in the current context.[652]

Roine *et al.*[653] used a commercial e-nose system (ChemPro 100) in which detection is based on a combination of an ion mobility chamber and a set of MOS sensors to differentiate cancer from benign cases using headspace of urine samples (Fig. 38). They tested 50 patients with confirmed prostate cancer and 15 patients with benign prostatic hyperplasia.[653] Urine samples were taken prior to operation, and another sample was taken post operation in the case of the control group. DA with leave-one-out cross-validation was used to analyze measurements and reduce the chance for over fitting of the data, followed by ROC curve analysis. Sensitivity and specificity were 78% and 67%, respectively, and AUC was 0.77 between the tested groups.[653]

7.2.2. Quartz crystal microbalance sensors. QCM sensors are based on the piezoelectric properties that, in general, rely on the sensor's response to an applied mechanical stress.[8] The adsorption of molecules from the gas phase on the sensors' surface results in variations in the fundamental oscillating frequency of a thin quartz crystal.[8] This variation can be measured and analyzed for VOC classification. A number of

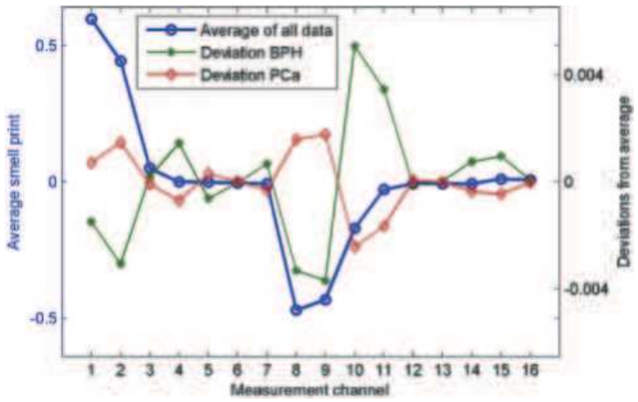

Fig. 38 Combined result of an e-nose MOS and ion mobility system. Total average of all data represents typical smell print of urine. Characteristics of average smell prints of benign prostatic hyperplasia (BPH) and prostate cancer (PCa) groups are represented by deviation from the total average. Reprinted from ref. 653 with permission from Public Library of Science, copyright 2014.

Published on 11 June 2018. Downloaded on 2/1/2025 6:21:23 AM.

QCM studies involved cancer detection from urine samples. All of them used the same set-up based on 8 different QCM sensors, coated by sensing layers of metalloporphyrins and integrated in an e-nose system developed at the University of Rome Tor Vergata.[648,651,654,655] Bernabei *et al.*[648] measured the headspace of urine samples from 113 patients with different urological pathologies. Using PCA and discriminant analysis combined with partial least square (PLS-DA), they could differentiate between healthy and sick people, and achieved gradual distinction between prostate and bladder cancer.[648] In follow-up experiments on prostate cancer, the same group used the same electronic nose set-up on a varying small clinical set of 21 to 41 samples. Using the same PLS-DA methodology, the authors showed fair discrimination between prostate cancer patients and the control group based on first urine samples. The authors reported 93% specificity in the last test.[651,654,655]

7.3. Bacterial and urinary tract infections

Microbial detection and analysis based on odor or VOCs produced by microorganism populations have been widely investigated.[25] Different microorganisms have unique metabolic pathways and can divide into very high numbers that can reach an average of 10^8–10^9 cell per mL culture fluid. As a result, different molecules are produced and degraded at high rates so that they can potentially be detected by analytical and sensor technologies, particularly for clinical applications.[656–659] UTI is a very common bacterial infection at all ages, and one of the most common reasons for hospitalizations due to infections among elderly individuals.[644] The clinical symptoms of UTI are often atypical or asymptomatic, making their diagnosis and management very challenging due to their high prevalence.[644] UTI diagnosis is currently based on the existence of clinical symptoms together with a nitrite strip test and partial measurement of white blood cell count in the urine. Even though urine culture is the gold standard test for UTI detection, it is both costly and time-consuming.[644]

7.3.1. Carbon black polymer sensors. Each sensor element is constructed from films consisting of carbon black particles dispersed in insulating organic polymers. The film's electrical conductivity is provided by the carbon black, whereas the different polymers provide the chemical diversity among elements in the sensor array.[660] Yates *et al.*[661] studied VOCs emitted from urine and blood samples using a commercial electronic nose (Cyrano Sciences C320) together with an Agilent 4440 Chemosensor. The e-nose system consisted of 32 carbon black polymer sensors. Samples of urine from routine hospital screening for UTI were evaluated, with the goal of classifying positive *vs.* negative UTI samples. The authors tested several different classification models and the best accuracy was 80%, using a nonlinear model with Kernel width parameter optimization.[661] In another trial using the polymer-based Cyranose 320 system, they tested urine samples from 13 patients.[662] Using the 32 sensors and PCA analysis, the authors checked only 9 of the 13 samples, out of which only one sample was identified correctly with UTI, 6 samples tested abnormal with mucus, and 2 were classified as healthy.[662]

7.3.2. Conducting polymer sensors. Sensors are constructed using conducting polymers with a specific selection of polymers and doping materials, resulting in responsiveness to different odor groups measured by changes in resistance. Pavlou *et al.*[663] used a commercial electronic nose (BH-114: Bloodhound Sensors, Leeds, UK), which employed 14 conducting polymer (polyaniline) sensors. In a 2-set series experiment, 25 and 45 urine samples from patients were analyzed by incubation in a VOC generation test tube system for 4–5 h. Data were interpreted using neural network analysis.[663] In both experiments the authors correctly identified all but one of the samples compared to standard culture methods. They were able to distinguish among the 4 tested subgroups: normal urine, *Escherichia coli* infected, *Proteus* spp. and *Staphylococcus* spp.[663] Kodogiannis *et al.*[664] used the same commercial system to test urine samples from 45 UTI suspected patients. They collected 5 mL of urine to which an appropriate culture medium was added to a final volume of 20 mL, which was incubated for 5 h at 37 °C. Using an extended normalized radial basis function based on neural networks, they correctly classified all samples using the overall model.[664]

7.4. Intestinal diseases

Inflammatory bowel disease (IBD) relates to a number of inflammatory disorders of the colon and small intestine; most notable are Crohn's disease (CD) and ulcerative colitis (UC).[665] Environmental factors (diet, smoking and personal hygiene), ethnicity, immune system problems, genetic susceptibility, and microbial infections can result in mucosal inflammation.[665] Bacterial diversity in the gastrointestinal tract is immense and difficult to study due to many of the bacteria being non-culturable *in vitro*.[666] The effect of this bacterial colonization, mainly through fermentation-derived VOCs, has been studied for diagnostic proposes.[666]

7.4.1. Metal oxide sensors. A commercial "electronic nose" (Fox 4000; AlphaMOS, Toulouse, France) was used to analyze the chemical signature of urine samples of 24 patients with UC, 24 patients with CD and 14 controls.[666] The system comprises an array of 18 metal oxide gas sensors, with the resistance being modulated in the presence of a target gas/vapor. The system uses 2.5 mL of headspace from the sample bottle, which is directly injected into the electronic nose. The change in resistance of the sensors was measured from an injection time of 120 seconds at a sampling rate of 1 Hz. PCA analysis applied to the signals obtained from the sensors showed clear variance between the 3 groups. A DFA classifier was developed, scoring 70% accuracy in separating the studied groups. A second analytical method was also tested, both as a complementary method and for a comparison between the 2 analytical methods. They used Field Asymmetric Ion Mobility Spectroscopy (FAIMS, Lonestar; Owlstone, UK). After the sample had been ionized, fragments of different sizes and types were introduced between 2 metal plates, and an asynchronous high-voltage waveform was applied. The ionized molecules are then subjected to these high electric fields. The difference in movement of these molecules in this high electric field can be measured, resulting in separation

of the complex mixture. The results of FDA on the FAIMS data for patients with CD, UC, and controls scored an accuracy that exceeded 75%.

Using a similar technology, Covington *et al.* tested another bowel condition, Bile Acid Diarrhea (BAD).[667] BAD is the most common cause of chronic diarrhea that affects ∼5% of the world population. The same AlphaMOS Fox 4000 electronic nose, in combination with an Owlstone Lonestar Field Asymmetric Ion Mobility Spectrometer, was used to sample urine headspace (Fig. 39). They sampled a total of 110 patients: 23 with BAD, 42 with UC and 45 controls.[667] One mL headspace from each 5 mL urine sample was examined using a HS100 autosampler after heating them for 10 min at 60 °C. The sensor's resistance change was measured over 180 seconds from injection at a sampling rate of 1 Hz. Results analyzed by DA gave 79 to 83% accuracy in classification of the tested groups. A complementary GC-MS analysis revealed 2 VOCs, 2-propanol and acetamide, which were significantly elevated in the BAD sample.[667]

7.5. Other medical conditions

7.5.1. Quartz crystal microbalance sensors. An electronic nose-based on QCM technology was used to analyze urine samples containing blood. In different injuries, such as kidney injuries, trace amounts of blood might appear in urine.[668] Here authors used a QCM-based electronic nose with 8 sensors coated with different metalloporphyrins. Urine samples were

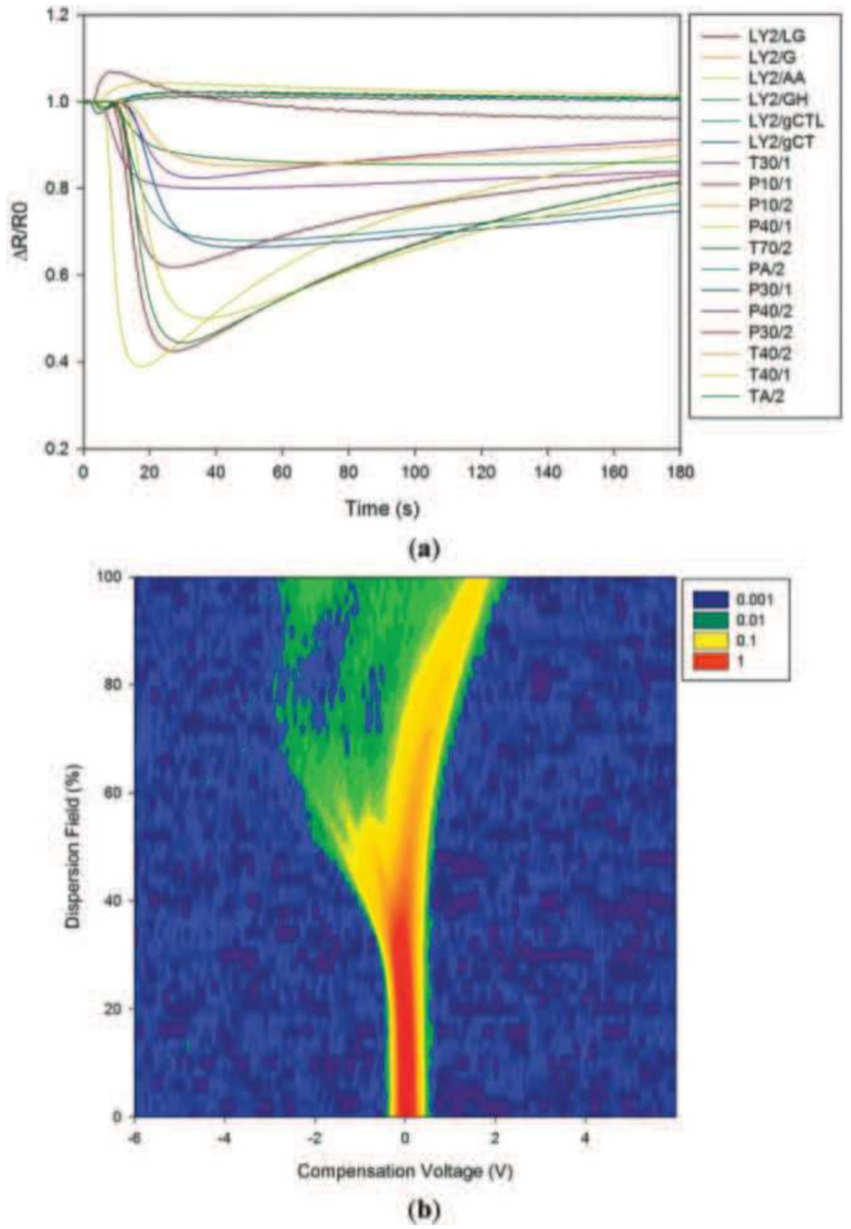

Fig. 39 (a) Raw electronic nose results showing sensor responses to Bile Acid Diarrhea (BAD) of a patient's urine sample. (b) Raw data from the FAIMS instrument response to a BAD patient urine sample. Intensity is in arbitrary units of ion count. Reprinted from ref. 667 with permission from MDPI, copyright 2013.

Published on 11 June 2018. Downloaded on 2/1/2025 6:21:23 AM.

Published on 11 June 2018. Downloaded on 2/1/2025 6:21:23 AM.

collected from children affected by kidney diseases with blood in urine and age-matched healthy children.[668] Samples were sealed in vials for 30 min at 30 °C. No data were provided regarding the volume of urine and headspace samples, or the number of participants. Their results suggest the ability of the system to correctly identify blood-containing urine samples.[668] They also claimed that by using ANN they could correctly determine the pH.[668]

8. Feces-based disease diagnostics

Feces have been gaining interest lately in scientific and clinical studies, mainly due to the recent explosion in microbiome research.[669] The human intestine harbors a variety of micro-organisms that can reach over trillions of cells, consequently creating a microbiome within the human body some 100 times bigger than the human genome.[669,670] Host–microbe relations are essential to many aspects of normal human physiology, such as metabolic activity and immune homeostasis, affecting the balance between health and disease.[671,672] Many fecal metabolomics studies on gut-related issues use Nuclear Magnetic Resonance spectroscopy (NMR) and mass-spectrometry techniques.[673] Dedicated studies on VOCs report on ∼500 compounds emitted from stool samples.[8] Colonic fermentation of different molecules, *e.g.*, amino acids, is responsible for the production of typical fecal odors, such as methane, aliphatic amines, ammonia, branched chain fatty acids, derivatives of phenol or indole, and volatile sulfur-containing compounds. Other VOCs found in fecal headspace, *e.g.*, chloroform, are possibly the outcome of contaminants absorbed from food, water or pollution.[8]

8.1. Malignant diseases

8.1.1. Carbon black polymer sensors. In a proof of concept study done on CRC patients, some authors used the commercial Cyranose 320 system composed of 32 polymer-based sensors to examine volatiles from fecal samples.[674] The study population included 40 patients with CRC, 60 patients with advanced adenomas, and 57 healthy controls. Each stool sample contained about 2 g frozen feces in a sealed vacutainer that was heated to 37 °C for 1 h. Thereafter, the heated vacutainers were connected to the Cyranose in an air-tight closed loop system.[674] The results showed that fecal VOC profiles of CRC patients significantly differed from controls with 85% sensitivity and 87% specificity. Similarly, VOC profiles of advanced adenoma patients differed from controls with 62% sensitivity and 86% specificity.[674]

8.2. Intestinal diseases

8.2.1. Metal oxide sensors. A single metal oxide sensor combined with a GC system was used to differentiate patients with IBD from those with IBS.[675] Here the researchers used a tin and zinc oxide paste to create a MOS sensor that served as the detector of the GC system. Interdigitated gold electrodes with 8 interpenetrating bars were constructed and operated at 450 °C. Sensor measurement was continuous and measured the change in electrical resistance. A GC with a static headspace was used to introduce feces samples using a sulfur column after 10 min of incubation at 50 °C (Shepherd SF 2014). An in-house ANN was used to differentiate between the groups. The authors sampled 181 patients from 3 groups (IBS, IBD, healthy). They achieved a distinction of 76% accuracy between IBS and IBD, whereas IBD *vs.* control and IBS *vs.* control gave mean accuracies of 79% and 54%, respectively.[675] Covington *et al.*[676] studied the plausibility of an electronic nose system based on both MOS and FAIMS technologies. The e-nose contained an array of 6 metal oxide-based sensors, as well as 6 electrochemical sensors, a pellistor (catalytic sensor), and a nondispersive infrared sensor (optical, to monitor CO_2). The MOS sensors operated under a constant current source (100 μA), the voltage across the sensor being amplified and monitored. The system was used to classify patients receiving pelvic radiotherapy, which frequently causes gastrointestinal side-effects.[676] Stool samples were taken before and 4 weeks after radiotherapy from 23 patients with the highest and lowest level of toxicity. Ten mL samples were heated for 1 h at 40 °C and, after dilution with 5 mL water, the sample was divided for e-nose and FAIMS analyses. Samples were introduced into the sensor chamber at 500 mL min^{-1} flow rate with 20% RH lab air. The VOCs emitted were products of fermentation caused by gut microflora.[676] Using PCA of the electronic nose data (and wavelet transform followed by Fisher discriminant analysis of FAIMS), they showed that it was possible to differentiate patients after treatment by their toxicity levels. Furthermore, differences were also identified in their pre-treatment samples and could be correctly classified in 22 of the 23 samples.[676]

Aggio *et al.*[677] used a platform composed of a gas chromatography column coupled to metal oxide gas sensors (OdoReader) to analyze fecal VOC patterns of 152 patients: 33 patients with active inflammatory bowel disease; 50 patients with inactive IBD; 28 patients with IBS and 41 healthy donors. Two mL fecal headspace were collected and injected into the GC column-metal oxide gas sensor system. The sensor was controlled by an electronic circuit monitored by computer software, which records the electrical resistance of the sensor at 0.5 s intervals during each 40 min machine run. The resistance profile of each sample generated by the system was used as the input for pattern recognition algorithms, namely SVM and PLS. Several cross-validation methods were used, including leave-one-out cross-validation, 10-fold cross-validation, double cross-validation and their Monte Carlo variations. The most clinically important findings after double cross-validation were the accuracy of active CD *vs.* irritable bowel syndrome (87%; CI 84–89%) and irritable bowel syndrome *vs.* controls (78%; CI 76–80%; Fig. 40). The same GC-sensor system was also used to investigate whether fecal VOC levels are associated with a response to dietary interventions in patients with IBS.[678] In this study, 93 IBS patients were randomly assigned to a group counselled to follow a diet low in fructans, galacto-oligosaccharides, lactose, fructose and polyols (low-FODMAP diet, $n = 46$) or a group that received placebo dietary advice (sham diet, $n = 47$) for 4 weeks. Fecal samples were collected and analyzed at baseline, and at the end of the 4 week study period. The PLS model to classify

 This journal is © The Royal Society of Chemistry 2018

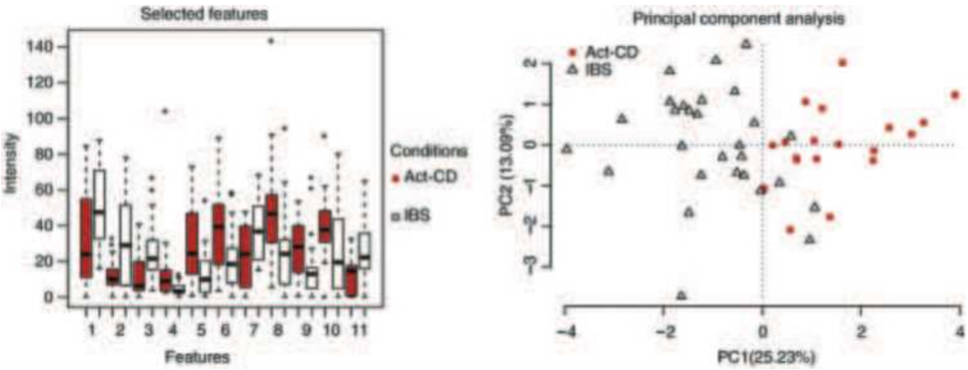

Fig. 40 VOCs were extracted from fecal samples and analyzed using an in-house-developed gas chromatography-sensor device. The profiles of VOCs were further analyzed using an in-house-developed computer algorithm that identified resistance patterns associated with specific medical conditions. This figure shows the selected features used to differentiate 19 samples from patients with active Crohn's disease (Act-CD) and 28 patients with irritable bowel syndrome (IBS), and a 2-component principal component plot based on the selected features. Reprinted from ref. 677 with permission from Wiley-Blackwell, copyright 2017.

Published on 11 June 2018. Downloaded on 2/1/2025 6:21:23 AM.

responders and non-responders on baseline samples showed very high accuracy for both interventions. The PLS model from the low FODMAP diet group predicted responses to the diet with median accuracy, sensitivity and specificity of 100%. However, the same PLS model showed low accuracy when classifying baseline samples from the patients in the control group, indicating that the model was specific for the responses to dietary interventions. In both these last studies, the authors did not identify or report the specific VOCs that contributed to the alterations in the profile shifts.

8.2.2. Polymer composite sensors. de Meij *et al.*[679] tested the ability of Cyranose 320 to discriminate between CD and UC by the volatolomic signature from fecal samples of children. In this case–control study, a total of 83 children (29 CD, 26 UC, and 28 age-matched controls) were enrolled. Two g frozen feces from each child were thawed in a sealed container and placed at 37 °C for 1 h, allowing the headspace to fill with VOCs prior to exposure to the sensors. Using internal cross-validated canonical discriminant analysis, the fecal VOC profiles of patients with UC and CD differed from controls; in active disease (sensitivity, specificity: 100%, 100%, 86% and 67%) and in clinical remission (94%, 94%) and (94%, 94%), respectively. Furthermore, CD-patients differed from UC-patients during active disease (97%, 92%), and after reaching clinical remission (88%, 72%). Quantitative analysis was not performed to address the specific differences in the concentrations of fecal VOCs. In another study by de Meij *et al.*, they tested the use of the Cyranose 320 system to detect necrotizing enterocolitis (NEC) by VOCs, emitted from fecal samples.[680] Samples from infants born at a gestational age of ≤30 weeks were collected daily, up to 28 days of life. The infants were assigned to 3 subgroups: NEC n = 13, sepsis n = 31, and matched controls n = 14.[680] Approximately 0.5 g feces were used in a sealed vacutainer that was heated to 37 °C for 1 h. PCA was used to analyze the results. The authors showed that fecal VOC prints of infants with NEC could be differentiated from controls 2–3 days prior to the onset of clinical symptoms. NEC *vs.* control at days 2 and 3 gave 83% sensitivity, 75% specificity and 89% sensitivity, 89% specificity, respectively.[680]

8.3. Infectious diseases

8.3.1. Metal oxide sensors. A set-up for the detection of *C. difficile* infection by VOC profiling was prepared using a MOS sensor connected to a GC system.[681] Here the authors used a tin and zinc oxide paste to create a MOS sensor that served as the detector in the GC system.[681] An interdigitated gold electrode with 8 interpenetrating bars was constructed and operated at 450 °C. Sensor measurement was continuous and measured the change in electrical resistance. A GC with a static headspace was used to introduce feces after 10 min of incubation at 50 °C. An in-house standard stool sample was used throughout the study, and all samples were from people presenting with diarrhea. Fifty *C. difficile* positive and 50 negative samples were tested. An ANN was used to differentiate between groups. The results showed comparable capability in discriminating between positive and negative samples with sensitivity and specificity of 85% and 80%, respectively.[681]

8.3.2. Carbon black polymer sensors. Similar to the work described in Sections 7.1 and 7.2, the authors used the same Cyranose 320 device, containing 32 polymer-based sensors. The possibility of the e-nose to diagnose late-onset sepsis (LOS) in preterm infants was explored.[682] In a multi-center study, fecal samples from infants at gestational age of ≤30 weeks were collected daily. Samples collected up to 5 days prior to LOS diagnosis were analyzed using the e-nose and compared to matched controls. Approximately 0.5 g feces were used in a sealed vacutainer that was heated to 37 °C for 1 h. Based on the sensors' response and combined with the PCA method, the method could discriminate between fecal VOC-profiles of infants with LOS (n = 36) and controls (n = 40) at 3, 2 and 1 day before the onset of LOS.[682] Sensitivity and specificity were moderate at best and spanned between 57.1% to 75% and 61.5% to 70.8%, respectively.

9. Saliva-based disease diagnostics

Saliva samples have been studied in different clinical and non-clinical applications.[21] VOCs are assumed to reach the saliva by

Published on 11 June 2018. Downloaded on 2/1/2025 6:21:23 AM.

passive diffusion from the blood.[8] Several studies have evidence of a correlation between VOCs in the blood and saliva.[683] In addition to VOCs from blood, saliva can potentially contain VOCs coming from other endogenous sources, such as gingival exudate, the nasal cavity, gastrointestinal reflux, oral cavity microorganisms and exogenous sources, such as food debris, commercial products and environmental pollution.[8]

10. Skin-based disease diagnostics

Skin is the largest human organ being 12–15% of body weight.[684] Skin, in particular sweat, could be informative in a number of clinical observations and diagnosis. For example, by utilizing tattoo-based sensors on the skin, quantitative chemical analysis of the sweat, pH, glucose and sodium levels monitoring is possible.[685–687] An additional approach is based on sampling the gaseous phase, the skin headspace. The skin emits a wide range of VOCs as metabolism products that contribute to a person's body odor. The difference between the secretion glands (eccrine, sebaceous and apocrine glands) and their heterogeneous distribution across the body results in different VOC profiles and thus different odors from assorted body regions. Even though different body regions share a considerable number of compounds, there are significant quantitative and qualitative differences in their VOC profiles. An additional factor comes from different bacterial populations, mostly aerobic, localized in different body regions. Many of these populations decompose long-chain body metabolism products into short-chain VOCs, including ketones, aldehydes, alcohols, and acids.[688] Most of the studies concentrate on the VOC signature from the axillary area, which is rich in apocrine glands and the primary source of human body odor. The emitted odors are of great interest for an almost unlimited number of potential applications, including forensic, biological,[689] diagnostic tools, search for entrapped humans[690] and many others. Diseases or disorders that interfere with the homeostatic harmony of the body result in changes in the skin VOC pattern both in quality and quantity. These changes result in characteristic odors.[25,640,691,692] For example, scarlet fever infection is characterized by a distinctive foul odor from the skin.[693] Investigation of healthy human skin has been reported from many studies.[21,684,688,694–701] de Lacy Costello *et al.* examined 532 different skin VOCs collected from healthy subjects.[21] Despite the enormous potential of the skin VOC pattern for diagnosing diseases, not many studies have been conducted on the subject. One of the major obstacles is there being no protocol standardization nowadays for skin VOC collection and analysis in terms of sampling location, duration, cleaning procedure, sampling methods and spectrometric analysis.[684,690,696,697,699,700,702–712] The most common sampling method is the SPME directly from the skin[702] or from absorbing materials which have been in direct contact with the body.[703,704] The majority of skin-based diagnosis studies focused on a spectrometric analysis of skin-related diseases, such as skin cancer,[703] wounds[640,713–715] and VOCs associated with mosquito attraction.[711,712,716] The implementation of gas sensor technology for disease diagnosis from the skin is still premature and includes reports for only a few diseases.[571,573,703,704,717,718] There is also a tremendous potential for diabetes monitoring by detecting the disease-related VOCs from the skin;[705,706] however, no report on a medical device for this monitoring has been published. With the rapid advancement in flexible gas sensors,[23,46] we hope to see more activity in this field.

10.1. Melanoma cancer

Many efforts have been invested in sampling the headspace of melanoma biopsies, as mentioned in Section 4.1. However, only one study[703] has investigated the VOC pattern associated with melanoma directly from skin headspace. This sampling should reduce the diagnosis period, allowing early screening and detection. D'Amico *et al.*[703] investigated VOC pattern differences emitted by melanoma lesions, nevi and healthy skin, from the same subject by sampling with a gauze pad.

10.1.1. Quartz crystal microbalance sensors. D'Amico *et al.*[703] also reported a gas sensor array for discriminating between nevi and melanomas by using 7 QCM sensors with different metalloporphyrins.[719,720] A metal cylinder with 4 cm diameter was used to isolate and sample the suspected lesions and nevi from 40 subjects. Comparing the responses of each sensor to melanoma lesions and nevi, a large variance in the former samples was observed. Only 2 sensors from the array showed a significantly higher mean value of the response to the melanoma samples compared to nevi. Additional assessment of the array performance was studied in a sub-group of 7 subjects before and after surgical removal of the lesion or nevi. Based on the collected responses, a PLS-DA resulted in 87% correct differentiation between melanoma and nevi samples. Pennazza *et al.*[571] reported the use of a gas sensor array based on 7 QCMs with different metalloporphyrins[721] for the detection of melanoma in both skin and headspace of biopsy samples (as mentioned in Section 4.1). A 4 cm diameter stainless-steel cylinder was used to isolate the investigated lesions among 40 subjects, 10 of whom had melanoma lesions. Each subject was sampled before and after surgical removal. The analysis demonstrated a significant change in the response for each sensor between those taken before and after surgical removal of the melanoma. A further PLS-DA showed a correct classification of 87% among the tested samples with sensitivity and specificity of 70% and 90%, respectively.[571] In a further study, Pennaza *et al.*[573] demonstrated the additional use of the gas sensor array in detecting melanoma development in both mice skin and headspace of biopsy samples (as mentioned in Section 4.1.7). Xenografted mice from 2 melanoma cell lines and tumor-free mice were sampled periodically with a metallic calendric chamber by a gas sensor array based on QCM with different metalloporphyrins: Cu(TBuOPP); Zn(TBuOPP); Fe(TBuOPP); Sn(TBuOPP); Ru(TBuOPP) and Cr(TBuOPP).[721] In a PCA analysis, they showed discrimination between control and xenografted skin headspace samples. Pattern changes as a function of sampling time in coordination with the tumor growth phase were also observed.[573]

 This journal is © The Royal Society of Chemistry 2018

Published on 11 June 2018. Downloaded on 2/1/2025 6:21:23 AM.

10.2. Schizophrenia

Skin-related diseases are not the only diseases associated with detection of VOCs from the skin; studies on schizophrenia detection have also been reported. Schizophrenia is a severe mental disorder characterized by hallucinations, delusions and cognitive deficits. The life-risk of the disorder is $\sim$1% and it is assumed that it is caused by multiple factors, including hereditary and environmental factors. The current methods of diagnosis are based mostly on behavioral signs and neuropsychological testing.

10.2.1. Quartz crystal microbalance sensors. In addition to GC-MS findings, Di Natale *et al.*[704] described a gas sensor analysis of skin odor collected with a cotton compress applied for 30 min on the upper side of the forearm. This study included 27 individuals, subjects with schizophrenia, other mental disorders and healthy volunteers. All patients were treated with anti-psychotic drugs. Prior to sampling, a strict cleaning protocol was used on each subject. After the sampling, the compress cotton was closed in a sealed vial, held at 25 °C for 30 min and then exposed to the sensor chamber. An array of 7 QCM sensors with different metalloporphyrins was used. Metalloporphyrin-based sensors have good sensitivity to carboxylic acids, as the potential candidate for detecting *trans*-3-methyl-2-hexenoic acid, based on previous GC-MS studies.[704,709,710] In a linear discriminant analysis of the gas sensor array, more than 80% correct classifications were achieved.

10.3. Heart failure

10.3.1. Metal oxide sensors. Voss *et al.*[717] reported the sensors' ability to detect heart failure, this time by analyzing skin emitted VOCs. The sensor head was placed in the crook of the arm. Ten measurements were taken for every patient over 35 min in the same air-conditioned room to avoid any environmental influences. In this study, 27 patients with DHF, 25 patients with CHF and 28 controls were involved. This time, DFA analysis of compensated *vs.* decompensated HF resulted in 87% discrimination accuracy and 85% discrimination accuracy between CHF and controls. These authors also reported on significant correlations between the calculated sensors' responses and clinical laboratory parameters, such as blood bicarbonate and pH levels. However, they did not report on the quantitative identification of the VOCs that affected the sensors' performance.

10.4. Renal diseases

10.4.1. Metal oxide sensors. Voss *et al.*[718] assessed the skin/body odor of renal failure patients with an array of 3 thick-film MO-based gas sensors with heater elements. Each of the sensors had a slightly different sensitivity to odorant molecules. Molecular components from the skin of 42 dialysis patients, 20 CKD patients (stages 3–5) and 11 healthy controls were allowed to pass directly to the sensors. Sensor resistance was measured in the range of 200–385 °C, which lasted approximately 2 min. Quadratic discriminant analysis on the corrected signals (normalized to the calibration mixture) of the 1st and 2nd PC from the 2 groups of renal patients gave a correct classification of 95.2% (3 patients were misclassified), whereas correct classification increased to 98.4% (only 1 patient being misclassified) using 1st–3rd PC. The authors also tested the correlation between the PC1 and PC2 for several clinical parameters. For example, they found a significant correlation between the principal components and blood albumin ($r = -0.69$), pH ($r = -0.55$) and creatinine ($r = -0.52$).

11. Hybrid sensor array

Previous sections (5–10) provided detailed references on technological achievements in the VOC diagnostic field. To take diagnostics to the next level, we should use this vast database to improve sensing technology. In this section, we address this issue and provide possible solution and explain how to use the available data of VOC disease-print for improving the technology. The rapid technological development, together with ongoing necessities, drives researchers to develop new nano-based sensors. Currently, as thoroughly discussed above, sensors based on different technologies are being used for VOC sampling *e.g.*, MOS, metal nanoparticles, CNTs, optic, piezoelectric, conducting polymers, and others in a range of applications. Yet in most cases, studies have concentrated on a single array technology and single transduction technology, such as conductance, mass, current and absorption changes. However, in terms of data analysis and pattern recognition, when analyzing electronic sensor array system applications (such as electronic noses), maximum orthogonality is required in the selection of many independent features.[722] Consequently, basing an array on a single transducer clearly leads to a limited power of resolution of such sensor arrays.[722] Therefore, the concept of Hybrid Sensor Arrays (multi-transducer arrays) has been developed, in which different construction techniques are used, leading to lower correlations between the responses of the different sensor types, which in turn strengthens the power of data analysis.[723] Thus, a hybrid array involves multivariable transducers with carefully selected excitation settings to deliver independent outputs allowing recognition of different responses to VOCs (Fig. 41).

While such hybrid arrays have been frequently advocated, they are scarcely used for VOC detection in general, and disease detection in particular. More than 20 years ago, Holmberg *et al.* described the use of a hybrid sensor array based on 3 approaches, MOSFET, chemiresistors and optical sensors for determining the quality of paper by its odor. Using 2 MOFSET and 2 chemiresistors, they achieved the best classification.[724] Ulmer *et al.* showed that an array of 4 different types of transducers could potentially operate better than one.[725] They used a combination of MOS, QCM, electrochemical and calorimetric sensors in the array. System performance was evaluated on food samples, and the results clearly showed a higher resolution power of the tested samples when using different transducers rather than just one.[725] The use of hybrid arrays, however, does not always ensure better classification.

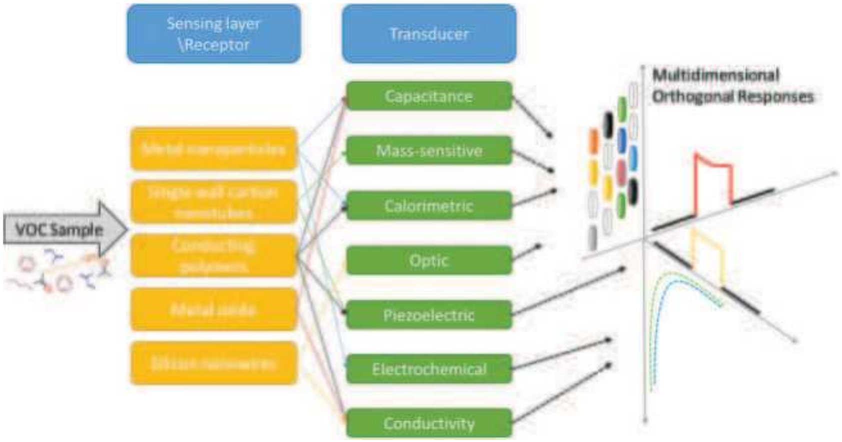

Fig. 41 Hybrid sensors are composed of a variety of sensing layer and different transducers to create multidimensional orthogonal responses.

Published on 11 June 2018. Downloaded on 2/1/2025 6:21:23 AM.

Moreover, a weak visible grouping based on PCA plots or cluster analysis does not necessarily indicate poor classification. The important factor would be to lower the actual quantitative indicator of performance, *i.e.*, the classifiers' test-set error.[723] High dimensionality spaces in pattern recognition can often lead to a bad classification, therefore another important part in the analysis would be the selection of an appropriate feature (sensor). In some cases, it could be that all the sensors together do not lower significantly the test-set error compared to a single class of sensors, but a correctly chosen subset does.[723] Pardo *et al.* evaluated the latter points using an array based on 7 QCM, 8 semiconductor and 4 electrochemical sensors on different data-sets of VOC emitting sources. After analyzing the data based on all sensors and using single or combined technology, they concluded the following among other factors: (1) feature selection increases the operation of any given sensor class by 3 to 5%, even when using just 2 or 3 sensors; (2) the performance achieved with the whole hybrid array (no feature selection) is superior to the performance of the top chosen sensor subsets for any single sensor type; (3) when selecting the best sensor subsets from the hybrid array, the performance increases, leading to a 35% reduction in the classification error in the tested case.[723] Jin *et al.* evaluated the use of 3 different transducers, cantilevers, capacitors, and calorimeters, coated with 5 different sorptive-polymer films.[726] They evaluated VOCs and VOC mixtures with single-transducer arrays of up to 5 sensors and hybrid arrays of up to 15 sensors. They showed that the best hybrid arrays constantly outperform the best single-transducer arrays of similar size. Furthermore, recognition rates were not improved significantly by using more than 5 sensors in a hybrid array for any particular analysis, regardless of difficulty.[726] In a following study, they evaluated the recognition limit for binary and ternary VOC mixtures with a similar hybrid array.[727] A few important conclusions were drawn using a hybrid array; first, the VOCs' relative concentration ratios are considerably more significant factors of performance (recognition of each) than are their absolute concentrations. Second, when performing a targeted-VOC analysis in a mixture, as long as the sensitivity to that VOC is better than its interfering VOCs,

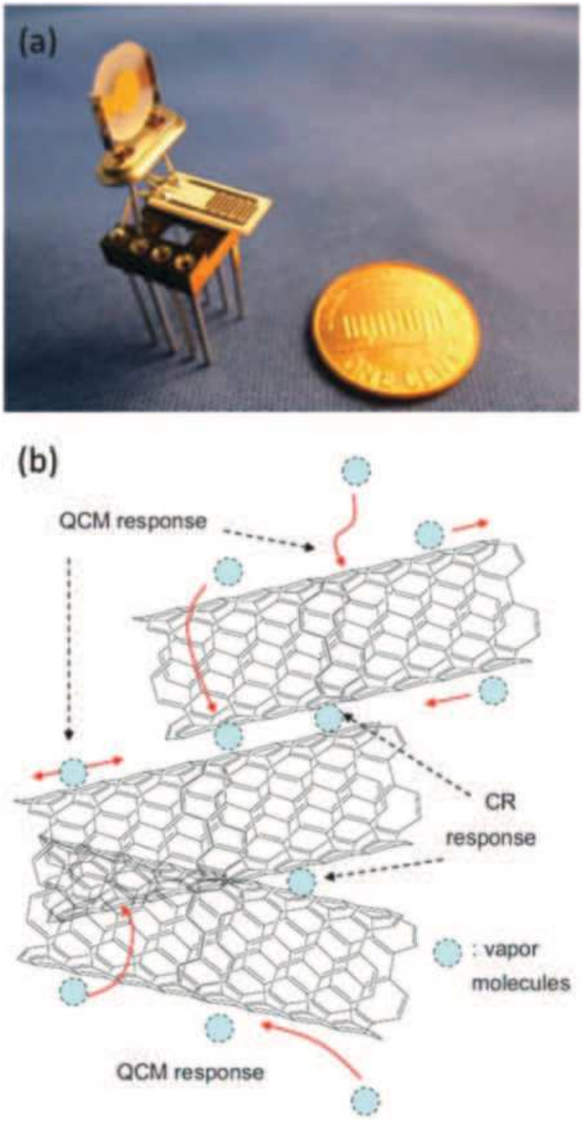

Fig. 42 Hybrid sensing: (a) photo of a hybrid QCM and chemiresistor, (b) scheme for the response mechanism of pristine SWCNT-coated QCMs and chemiresistors. Reprinted from ref. 730 with permission from Elsevier, copyright 2015.

Published on 11 June 2018. Downloaded on 2/1/2025 6:21:23 AM.

it should be identifiable over a range of concentrations even if it is the marginal component.[727] In an *in vitro* study monitoring anti-fungal efficacy based on VOC analysis, researchers used a sensor array hybrid.[728] The hybrid consisted of MOS and MOSFET sensors, but no clear data were provided regarding the separate performance of each type. The use of the hybrid array could possibly discriminate between effective and non-effective concentrations of anti-fungal compounds based on VOCs print.[728] Originally, each component of a hybrid sensor array is not connected, but each is constructed differently, as described above. Yet in some cases, researchers use the same base technology for the receptor part of the sensor while using different technologies for the transducer part. One such example is described in Li *et al.*[729] in which they used the same 4 gold nanoparticle covered ligands as a receptor and connected it to 2 different transducers. In the first case, they placed it as a sensing layer in chemiresistors, and in the second as a sensing layer in QCM, thus creating a hybrid array with a similar receptor and different transducers. They tested 15 different VOCs and their gas evaluation result showed an increase in classification when using the hybrid technologies rather than using just one technology.[729] Similarly, the VOC response to a surface modified SWCNT (as the receptor) and 2 different transducer platforms, chemiresistors (CR) and QCM, was evaluated (Fig. 42a).[730] The study showed that even when using the same coating layer (receptor) on both QCM and CR, the QCM response reached equilibrium faster than CR. The proposed explanation is that vapor molecules take longer to intercalate between the junctions of 2 nanotubes, thus delaying the increase in resistance in the CR (Fig. 42b).[730] With respect to response patterns, this additional mechanism involved in changes of resistance directed the differences between QCM and chemiresistor patterns; thus the same set of SWCNTs will prompt diverse response patterns on 2 diverse sensor array transducers.[730]

In yet another work comparing the performance of 2 types of transducers (QCM, CR) for VOC detection, the authors concluded that response diversity in the hybrid (multi-transducer) array is modestly greater than a similar dimensional (*i.e.*, same number of sensors) single transducer array.[731] The hybrid approach can allow multidimensional measurement that can react to the VOC mixture in a complementary manner, thus providing higher sensitivity and consequently better resolution. Using different transducer platforms on top of different sensing layers introduces an additional degree of freedom for selection and analysis of data. Novel hybrid arrays will bring new ways of thinking about measurements of interfering compounds. Instead of trying to avoid their measurement, the hybrid array will measure them but in a different orthogonal direction than the VOC of interest, allowing clearer observation. Alternatively, studies also refer to an adaptable chemosensor approach in which operating parameters of the system can adapt and change based on application needs and ambient changes.[732] Such adaptation could be on the tunable level of the device itself, such as internal sensing alteration of operation voltages and frequencies, as well as adaptation of post-data analysis with different filters, machine learning and statistical tools that might tune in real-time sensing factors.[732] One example would be using temperature-programmed metal oxide sensors, in which heating operating element changes during analysis, resulting in different responses overcoming complex environmental measurements.[733] Such an approach can provide a single sensing transducer array with the power of a hybrid array.

Arguably, the hybrid approach is indeed the next promising step of these sensors. In sections above (5–10), we have thoroughly provided examples of the specific sensor technologies that have been successfully used until now in the field of medical diagnostics. These sections, together with the ''maps'' of VOC-prints we build in (Fig. 2 and 3) and detailed information summarized in Table S1 (ESI†), should serve as basic information, tutorial-like,

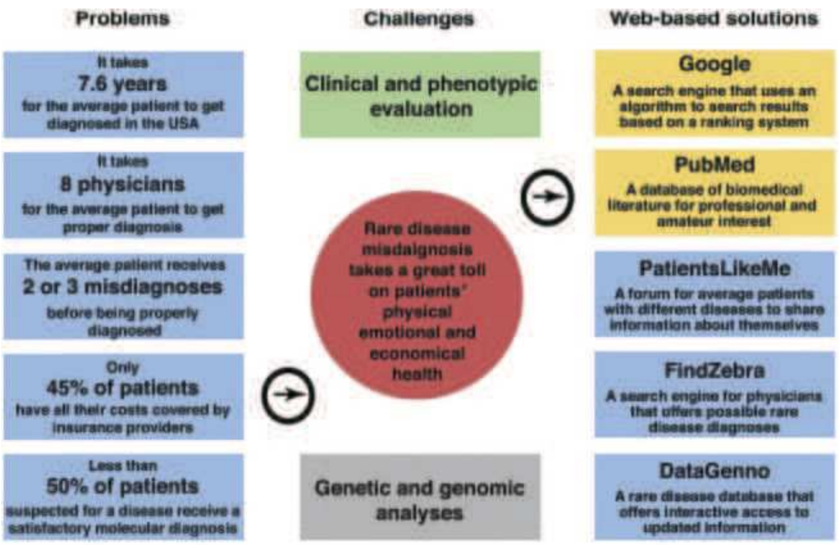

Fig. 43 This illustration presents the main problems in diagnosing diseases, the challenges and part of the web-based solutions that might contribute considerably to patient diagnosis. Reprinted from ref. 734 with permission from Elsevier, copyright 2017.

for designing and developing novel sensor arrays. For example, gastric cancer diagnostics has until now successfully been analyzed from breath by monolayer capped metal nanoparticles, carbon nanotubes and silicon nanowires. This is further supported by *in vitro* data that used carbon nanotubes and electrochemical sensors. Thus, hybrid arrays combining these 3 technologies would be the logical approach for new breath-based gastric cancer systems. From another example in Sections 5–10, one can see that, for diabetes mellitus diagnostics, a metal oxide sensor can be used successfully for urine samples, and also metal oxides, conducting polymers and optical fibers can be used for breath samples. This information can direct a metal oxide sensor towards hybrid sampling of both urine and breath specimens; cross-validation from 2 sources will likely provide improved accuracy and a better sensing system.

12. Disease sense in the ICT era

Disease management is becoming more and more sophisticated, part of which is due to the fast-technological communication leap. With that, many diseases still suffer from problems and challenges for proper diagnosis, as illustrated in the case of rare diseases (Fig. 43).

The information and communication (ICT) era relates to the spatiotemporal integration of data collected by a variety of

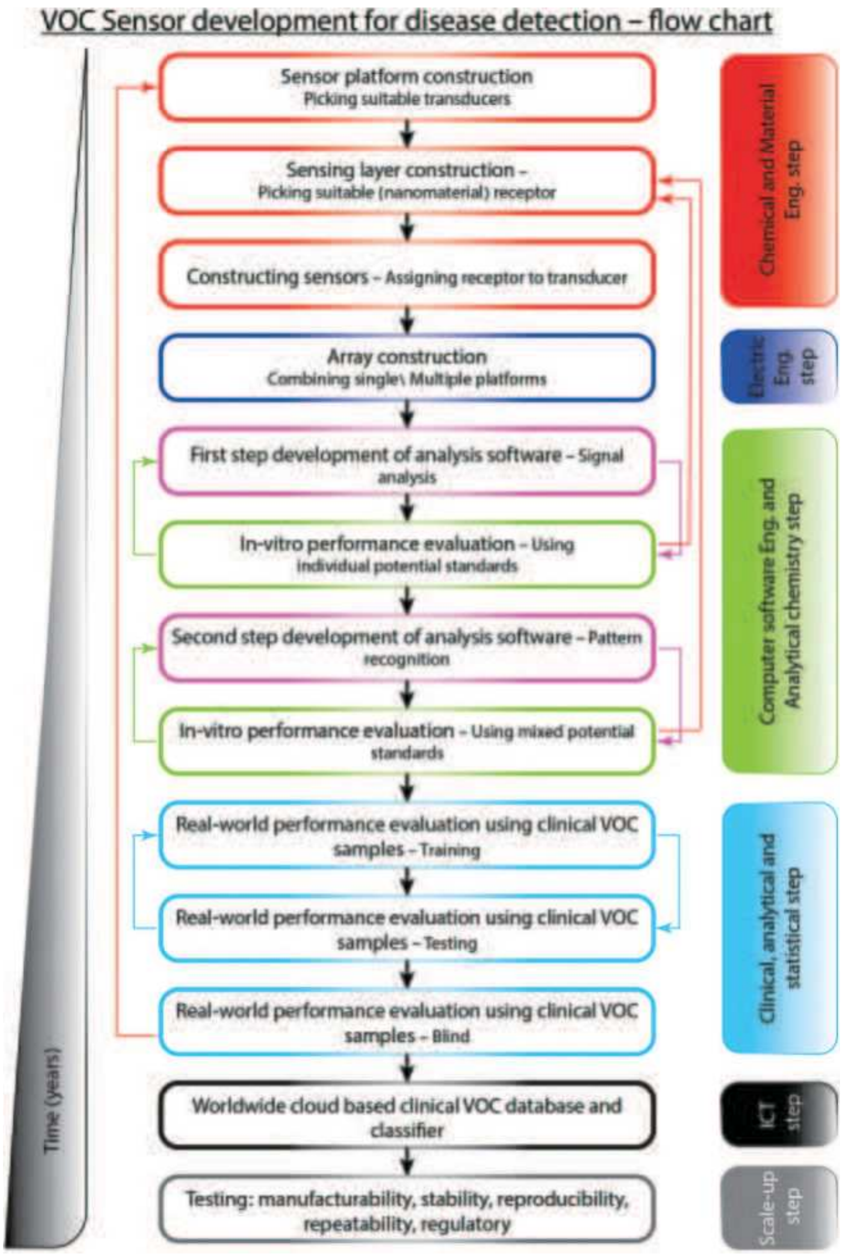

Fig. 44 Workflow process of sensor/sensor array development for clinical use. The process is based on individual steps that are constantly validated and reanalyzed to achieve optimal results. Such a procedure will require years of research and development to mature.

Published on 11 June 2018. Downloaded on 2/1/2025 6:21:23 AM.

This journal is © The Royal Society of Chemistry 2018

Published on 11 June 2018. Downloaded on 2/1/2025 6:21:23 AM.

entities, such as telecommunication, computers, software and databases. Today's globalization makes the data available in seconds. These latter factors together with developing technologies open the options for future high impact point-of-care (POC) diagnostics. POC diagnostics relate to medical tests that can be carried out at the bedside with online immediate results, thereby directing immediate clinical treatment.[735] As a result, no unnecessary delays occur due to preparation, logistics and analysis, thereby providing fast economical care, particularly in developing countries, or in places that lack laboratory infrastructure and trained healthcare personal.[736] Currently a variety of tests are available as POC in clinical applications, such as enzymes, hematology, diabetes mellitus, infectious agents, fertility, metabolites and others.[737] Entering the new era of ICT, 3 main aspects will take important roles – miniaturization, parallel analyses and data networking by ICT.[737] Miniaturization by chip technology, microfluidics and nano-based sensors will reduce sample volume (liquid or gas) and associated procedures.[737] Furthermore, it will enable connection and direct integration into current ICT platforms. Parallel analysis and lab-on-chip approaches enable laboratory scale measurements in a single system. Hybrid system sensor arrays, discussed in the previous section operating on orthogonal platforms, will allow measurements of a range of parameters for general screening purposes using portable systems. ICT platforms, cellphones or other portable devices and potentially human imbedded chips are direct result of these miniaturization and parallelization. Clinical data will be immediately uploaded to cloud-based servers, comparing and supporting deep online analysis. Indeed, for such ideas to be realized, many of the technologies still need standardization for cross-reference analysis and adequate handling of pre- and post-sampling issues; moreover, it will require the clinical community to address personal data security, ethics and political hurdles. Cloud-based servers provide a huge data storage capacity, but constitute a menace to privacy and security of clinical data that potentially can be catastrophic in the wrong hands. Therefore, closed systems are required that can cope with this issue. Moreover, as the future draws us towards online and cloud storage, a new issue arises, namely losing medically relevant data no longer backed up by hardcopy files.

While many sensor technologies are being developed (as we have extensively reviewed above) showing high potential in lab simulations as well as clinical trials, only a few achieve real-world applicability. This is probably the result of the underestimation of the difficulty of the sensing task together with overestimation of sensor array abilities, and the absence of proper data validation.[738] Still, many clinical conditions involve numerous parameters. While parallel analysis can be achieved for some, such as in the hybrid system, others are more problematic and in many cases sensor technologies are not sufficiently sensitive, or do not possess the required resolution power to correctly identify and quantify potential biomarkers or conditions. The long-term function of chemical sensing systems is unknown, and thus changing environments will require adaptive techniques to compensate for changes and reduce the system's power requirements.[732] Therefore, any development and progression of sensors and sensor arrays aimed for clinical use should follow a complex procedure. Such a workflow is based on individual steps that are constantly validated and reanalyzed to achieve optimal results, and will require years of research and development to mature (Fig. 44). The first step is based on chemical and material engineering work; in this part the basis of the sensing system is established, selecting the correct sensor platform, followed by the selection of an appropriate sensing layer and constructing them together. The second step includes electrical engineering work to integrate different sensors to a cohesive single or multi-array platform. The third step includes both software engineering and analytical chemistry for developing analysis software for single and then after multiple pattern samples; this step is continuously checked by single and multiplex analytical standards and varying concentration to evaluate accuracy, precision, sensitivity, selectivity, LOD, dynamic range, linearity, resolution, response time and life cycle. The fourth step includes clinical, analytical and statistical steps, and mainly involves the performance and data analysis of suitable clinical studies, including blind analysis. The fifth step is the ICT integrating system to a cloud-based database. The last step (a multistep) is post-development scale-up that includes testing the system for manufacturability, stability, reproducibility and regulatory function.

13. Conclusion

Clinical diagnostics is one field that does not rest and requires constant improvement in order to cope with new conditions and new diseases. It is well established that efficient screening programs, particularly for diseases, such as cancer, neurological or similar, are crucial for saving and extending life. For example, prognostic data show that early detection can increase survival chance by $>50\%$ in 5 year terms for some cancers. In turn, it will also reduce direct and indirect medical cost, allowing governments and health insurance companies to allocate funding for developing novel drugs and improving current treatments. For these to happen, technological improvement is needed and key players would be superior diagnostic sensor arrays that could be easily integrated into POC systems. More specifically, sensor arrays for volatolomic analysis, which is a promising non-invasive diagnostic approach based on the metabolic changes within the body, can provide the means to achieve this improvement. Many new sensors are being studied using a variety of transducers and an even wider variety of sensing layers. However, most of them are at very preliminary phases of construction and low-complexity *in vitro* evaluation, and serve as proof-of-concept that in most cases do not reflect the real world requirements for applicable diagnostics.

Therefore, the following recommendations are most important:

(1) Multidisciplinary collaborations must be encouraged already from the early stages of the sensor development in order to bridge the vast gap of knowledge transfer between the field of engineering science and the clinical field.

Published on 11 June 2018. Downloaded on 2/1/2025 6:21:23 AM.

(2) Testing and scaling-up of such systems for real-world experiments using worldwide representative populations.

(3) Evaluating the performance of the sensors under 'non-sterile' conditions, which includes consideration of different confounding factors from within the body as well as from the environment. This should be followed by adjustments of suitable techniques in order to test sensors within the true complex settings.

(4) Current empirical and metabolic data estimate the concentrations of medically relevant VOCs to be in the range of ppt and up to hundreds of ppb. Nevertheless, many sensors still do not possess the required sensitivity to detect such levels. Thus new (nano-)materials and approaches, such as hybrid arrays, should be developed to achieve the required sensitivity levels.

(5) Other issues, such as sampling, standardization, logistics and regulation, will as well need further attention to achieve future cohesive diagnostic systems.

The medical world will have to be convinced that VOC detection and identification is valuable for healthcare. The next few years will be critical for such an approach, in which scientists and engineers will need to present the real plausibility of this approach. This will require scale-up, proof of precision and accuracy of the technology. At the same time, we, the researchers of the technology, must be open-minded and listen to the requirements of the medical profession and industry that will bring the products to the market place. Vast funding together with worldwide commercial support could drive this methodology as the next best approach to diagnostics.

Abbreviations

A	Area
AA	Advanced adenoma
AC	Asthma and chronic obstructive pulmonary disease
ac-DEP	Alternating current dielectrophoresis
AD	Alzheimer's disease
ADK	Adenocarcinoma
AKI	Acute kidney injury
ANN	Artificial neural network
AUC	Area under curve
BAD	Bile acid diarrhea
BP	Back-propagation
BTEXN	Benzene, toluene, ethylbenzene, *p*-xylene, and naphthalene
$C_{alveolar}$	Alveolar concentration
CD	Crohn's disease
CHF	Compensated heart failure
CI	Confidence interval
CKD	Chronic kidney disease
cm	Centimeter
CMOS	Complementary metal-oxide-semiconductor
CNTFET	Carbon-nanotube field effect transistor
CNT	Carbon nanotube
COPD	Chronic obstructive pulmonary disease
CR	Chemiresistor
CRC	Colorectal cancer
CT	Computed tomography
CVD	Chemical vapor deposition
DA	Discriminant analysis
dB	Decibel
DC	Drop-casting
DFA	Discriminant function analysis
DHF	Decompensated heart failure
DHT	Dihydroxytryptamine
DM	Diabetes mellitus
DMF	Dimethylformamide
DNA	Deoxyribonucleic acid
E. coli	*Escherichia coli*
EDA-G	Ethylenediamine-modified reduced graphene oxide
EGFR	Epidermal growth factor receptor
EML4-ALK	Echinoderm microtubule-associated protein-like 4 fused to the anaplastic lymphoma kinase gene
ESRD	End-stage renal disease
eV	Electron volt
FAIMS	Field asymmetric ion mobility spectroscopy
FET	Field effect transistor
FN	False negative
FODMAP	Fermentable, oligo-, di-, mono-saccharides and polyols
FP	False positive
GC	Gastric cancer
GC-MS	Gas chromatography linked with mass spectrometry
GCE	Glass carbon electrode
GNP	Gold nanoparticle
GO	Graphene oxide
GPa	Gigapascal
GR	Graphene
HBC	Hexa-*peri*-hexa-benzocoronene
HBEC	Human bronchial epithelial cell
HCC	Hepatocellular carcinoma
HD	Hemodialysis
HER2	Human epidermal growth factor receptor 2
HF	Heart failure
HiPco	High-pressure carbon monoxide disproportionation
HIV	Human immunodeficiency virus
HMP	High metastatic potential
HNSCS	Head and neck squamous cell carcinoma
HTS	Hexyltrichlorosilane
IBD	Inflammatory bowel disease
IBE	Immortal bronchial epithelium
IBS	Irritable bowel syndrome
ICT	Information communications technology
IPA	Isopropanol
iPD	Idiopathic PD
IPF	Idiopathic pulmonary fibrosis
K	Kelvin
kHz	Kilo hertz
KIM-1	Kidney injury molecule-1

 This journal is © The Royal Society of Chemistry 2018

KRAS	Kirsten rat sarcoma
LbL	Layer-by-layer
LC	Lung cancer
LMP	Low metastatic potential
LOOCV	Leave one out cross validation
LOS	Late-onset sepsis
LV	Latent variable
m	Meter
M	Molar
MAO-B	Monoamine oxidase B
MCMNP	Monolayer-coated metal nanoparticle
MCNP	Monolayer-coated nanoparticle
MCR	Misclassification rate
MLP	Multi-layer perceptron
MM	Malignant mesothelioma
MO	Metal oxide
MOFSET	Metal-oxide-semiconductor field-effect transistor
MPa	Megapascal
MRI	Magnetic resonance imaging
MS	Multiple sclerosis
MTB	Mycobacterium tuberculosis
MWCNT	Multi-walled carbon nanotube
NAD+	Oxidized nicotinamide adenine dinucleotide
NADH	Reduced nicotinamide adenine dinucleotide
NDT	1,9-Nonanedithiol
NEC	Necrotizing enterocolitis
nm	Nanometer
NMR	Nuclear magnetic resonance spectroscopy
NP	Nanoparticle
NPV	Negative predictive value
NSCLC	Non-small cell lung cancer
NT	Nanotube
NW	Nanowire
OC	Ovarian cancer
ODA	Oleylamine
OF	Optic fiber
OHDA	Hydroxydopamine
OLGIM	Operative link on gastric intestinal metaplasia
P	Pressure
P3HT	Poly-3-hexylthiophene
PAA	Polyacrylic acid
PABS	Poly(*m*-aminobenzene sulfonic acid)
PAH	Polycyclic aromatic hydrocarbons
PAN	Polyacrylonitrile
PANI	Polyaniline
PC1	Principal component 1
PCA	Principal component analysis
PD	Parkinson's disease
PDMS	Polydimethylsiloxane
PECH	Polyepichlorohydrin
PEDOT:PSS	Poly(3,4-ethylenedioxythiophene): poly(styrenesulfonate)
PEG	Polyethylene glycol
PEI	Polyethyleneimine
PET	Polyethylene terephthalate
PgR	Progesterone receptor
pH	Potential of hydrogen
PI	Polyisoprene
PLD	Pulsed laser deposition
PLS	Partial least squares
PMMA	Polymethylmethacrylate
POC	Point-of-care
ppb	Parts per billion
ppm	Parts per million
ppq	Parts per quadrillion
PPr	Polypyrene
ppt	Parts per trillion
PS	Polystyrene
PSL-DA	Discriminant analysis combined with partial least square
PVA	Poly(vinyl acetate)
PVDF	Poly(vinylidene fluoride)
PVP	Polyvinylpyrrolidone
PPV	Positive predictive value
PZT	Ferroelectric lead zirconium titanate
QCM	Quartz crystal microbalance
RGO	Reduced graphene oxide
RH	Relative humidity
RIF	Rifampicin
RN	Random network
ROC	Receiver operating characteristic
s	Second
S	Siemens
SAW	Surface acoustic wave
SCC	Squamous cell carcinoma
SCLC	Small cell lung cancer
SD	Source-drain
SEM	Scanning electron microscope
S-G	Sulfonated reduced graphene oxide
sh-crl-PU	Self-healing disulfide-cross-linked polyurethane
sh-μAgPU	Self-healable silver-polymer (μag-polyurethane diol) composite
SiNW	Silicon nanowire
STW	Surface transverse wave
SPME	Solid-phase microextraction
SVM	Support vector machine
SWCNT	Single-walled carbon nanotube
TB	Tuberculosis
TEM	Transmission electron microscopy
TFT	Thin-film transistor
Tg	Transgenic rat
TN	True negative
TP	True positive
TPSS	Tetrakis(4-sulfophenyl)porphine
UC	Ulcerative colitis
UTI	Urinary tract infection
UV	Ultraviolet
V	Voltage
VA	Vertically aligned
VOC	Volatile organic compound
W	Watt
WHO	World health organization

Published on 11 June 2018. Downloaded on 2/1/2025 6:21:23 AM.

View Article Online

WT Wild type
wt% Weight percentage
ZIF Zeolitic imidazolate framework
Ω Ohm

Conflicts of interest

There are no conflicts to declare.

Acknowledgements

This work received funding from the Horizon 2020 ICT Program under the SNIFFPHONE (grant agreement no. 644031). This work received funding from the Phase-II Grand Challenges Explorations award of the Bill and Melinda Gates Foundation (Grant ID: OPP1109493). This work received funding from the Britain Israel Research and Academic Exchange Partnership (BIRAX; Grant 62BX14RBHH). The authors acknowledge Prof. Jin Han for fruitful discussions, Dr Viki Kloper for help with artwork production, Ms Shifaa Bdarneh for help with constructing the disease-related VOC table and Dr Falk Fish for a detailed review of the manuscript. R. V. acknowledges The Ariane de Rothschild Foundation for a PhD scholarship.

Published on 11 June 2018. Downloaded on 2/1/2025 6:21:23 AM.

References

1 T. McKeown, *BMJ*, 1983, **287**, 594.
2 W. M. Patterson, *Family Practice*, 1985, **2**, 1–3.
3 World Health Organization (WHO), Introduction in WHO global status report on noncommunicable diseases, Geneva, Switzerland, 2010.
4 M. Kuhn, W. Goebel, D. J. Philpott and P. J. Sansonetti, *Overview of the bacterial pathogens*, 2002.
5 World Health Organization (WHO), Communicable disease alert and response for mass gathering: key considerations, 2008.
6 J. Karnon, E. Goyder, P. Tappenden, S. T. McPhie, I. Towers, J. Brazier and J. Madan, *Health Technol Assess*, 2007, **11**, 1–145.
7 Y. Y. Broza, R. Kremer, U. Tisch, A. Gevorkyan, A. Shiban, L. A. Best and H. Haick, *Nanomedicine: NBM*, 2013, **33**, 92.
8 Y. Y. Broza, P. Mochalski, V. Ruzsanyi, A. Amann and H. Haick, *Angew. Chem., Int. Ed.*, 2015, **54**, 11036–11048.
9 K. Rohlenova, K. Veys, I. Miranda-Santos, K. De Bock and P. Carmeliet, *Trends Cell Biol.*, 2017, **16**, 30201–30205.
10 A. Amann, M. Corradi, P. Mazzone and A. Mutti, *Expert Rev. Mol. Diagn.*, 2011, **11**, 207–217.
11 W. Miekisch, J. K. Schubert and G. F. Noeldge-Schomburg, *Clin. Chim. Acta*, 2004, **347**, 25–39.
12 M. Hakim, Y. Y. Broza, O. Barash, N. Peled, M. Phillips, A. Amann and H. Haick, *Chem. Rev.*, 2012, **112**, 5949–5966.
13 B. Buszewski, M. Kesy, T. Ligor and A. Amann, *Biomed. Chromatogr.*, 2007, **21**, 553–566.
14 M. K. Nakhleh, Y. Y. Broza and H. Haick, *Nanomedicine*, 2014, **9**, 1991–2002.
15 B. J. Hunt and K. M. Jurd, *BMJ*, 1998, **316**, 1328–1329.
16 C. Michiels, *Am. J. Pathol.*, 2004, **164**, 1875–1882.
17 H. Haick, Y. Y. Broza, P. Mochalski, V. Ruzsanyi and A. Amann, *Chem. Soc. Rev.*, 2014, **43**, 1423–1449.
18 D. K. Chan, C. L. Leggett and K. K. Wang, *World J. Gastroenterol.*, 2016, **22**, 1639–1649.
19 P. Trefz, S. Kamysek, P. Fuchs, P. Sukul, J. K. Schubert and W. Miekisch, *J. Breath Res.*, 2017, **11**, 1752–7163.
20 G. A. Mills and V. Walker, *J. Chromatogr. B: Biomed. Sci. Appl.*, 2001, **753**, 259–268.
21 B. de Lacy Costello, A. Amann, H. Al-Kateb, C. Flynn, W. Filipiak, T. Khalid, D. Osborne and N. M. Ratcliffe, *J. Breath Res.*, 2014, **8**, 1–29.
22 L. Dormont, J. M. Bessiere and A. Cohuet, *J. Chem. Ecol.*, 2013, **39**, 569–578.
23 R. Vishinkin and H. Haick, *Small*, 2015, **11**, 6142–6164.
24 A. Amann, P. Mochalski, V. Ruzsanyi, Y. Y. Broza and H. Haick, *J. Breath Res.*, 2014, **8**, 016003.
25 Y. Y. Broza and H. Haick, *Nanomedicine*, 2013, **8**, 785–806.
26 N. Barsan and U. Weimar, *J. Electroceram.*, 2001, **7**, 143–167.
27 M. E. Franke, T. J. Koplin and U. Simon, *Small*, 2006, **2**, 36–50.
28 P. I. Gouma, A. K. Prasad and K. K. Iyer, *Nanotechnology*, 2006, **17**, S48.
29 E. N. Dattoli, A. V. Davydov and K. D. Benkstein, *Nanoscale*, 2012, **4**, 1760–1769.
30 Z. Fan and J. G. Lu, *IEEE Trans. Nanotechnol.*, 2006, **5**, 393–396.
31 K.-W. Kao, M.-C. Hsu, Y.-H. Chang, S. Gwo and J. A. Yeh, *Sensors*, 2012, **12**, 7157–7168.
32 H. J. Kim and J. H. Lee, *Sens. Actuators, B*, 2014, **192**, 607–627.
33 G. Korotcenkov, *Mater. Sci. Eng., B*, 2007, **139**, 1–23.
34 C. M. Hangarter, N. Chartuprayoon, S. C. Hernández, Y. H. Choa and N. V. Myung, *Nano Today*, 2013, **8**, 39–55.
35 P. C. Chen, F. N. Ishikawa, H. K. Chang, K. Ryu and C. Zhou, *Nanotechnology*, 2009, **20**, 125503.
36 U. Tisch and H. Haick, *MRS Bull.*, 2010, **35**, 797–803.
37 S. Chakraborty, A. Sen and H. S. Maiti, *Sens. Actuators, B*, 2006, **115**, 610–613.
38 J. Tamaki, T. Maekawa, N. Miura and N. Yamazoe, *Sens. Actuators, B*, 1992, **9**, 197–203.
39 S. J. Kim, I. S. Hwang, C. W. Na, I. D. Kim, Y. C. Kang and J. H. Lee, *J. Mater. Chem.*, 2011, **21**, 18560–18567.
40 Y. H. Cho, Y. N. Ko, Y. C. Kang, I. D. Kim and J. H. Lee, *Sens. Actuators, B*, 2014, **195**, 189–196.
41 T. Sahm, W. Rong, N. Bârsan, L. Mädler and U. Weimar, *Sens. Actuators, B*, 2007, **127**, 63–68.
42 Y. J. Hong, J. W. Yoon, J. H. Lee and Y. C. Kang, *Chem. – Eur. J.*, 2014, **20**, 2737–2741.
43 S. J. Kim, C. W. Na, I. S. Hwang and J. H. Lee, *Sens. Actuators, B*, 2012, **168**, 83–89.
44 H. Zhao, B. Rizal, G. McMahon, H. Wang, P. Dhakal, T. Kirkpatrick, Z. Ren, T. C. Chiles, M. J. Naughton and D. Cai, *ACS Nano*, 2012, **6**, 3171–3178.

 This journal is © The Royal Society of Chemistry 2018

Published on 11 June 2018. Downloaded on 2/1/2025 6:21:23 AM.

45 C. X. Wang, L. W. Yin, L. Y. Zhang, D. Xiang and R. Gao, *Sesnors*, 2010, **10**, 2088–2106.
46 H. Jin, Y. S. Abu-Raya and H. Haick, *Adv. Healthcare Mater.*, 2017, **6**, 1700024–1700044.
47 W. T. Koo, S. J. Choi, N. H. Kim, J. S. Jang and I. D. Kim, *Sens. Actuators, B*, 2016, **223**, 301–310.
48 K. I. Choi, H. J. Kim, Y. C. Kang and J. H. Lee, *Sens. Actuators, B*, 2014, **194**, 371–376.
49 P. M. Perillo and D. F. Rodríguez, *J. Alloys Compd.*, 2016, **657**, 765–769.
50 Z. P. Li, Q. Q. Zhao, W. L. Fan and J. H. Zhan, *Nanoscale*, 2011, **3**, 1646–1652.
51 Y. Zhang, J. P. Li, G. M. An and X. L. He, *Sens. Actuators, B*, 2010, **144**, 43–48.
52 J. S. Jang, S. J. Choi, S. J. Kim, M. Hakim and I. D. Kim, *Adv. Funct. Mater.*, 2016, **26**, 4740–4748.
53 R. Xing, L. Xu, J. Song, Q. L. Zhou, D. Liu and H. W. Song, *Sci. Rep.*, 2015, **5**, 10717.
54 D. Koziej, N. Bârsan, U. Weimar, J. Szuber, K. Shimanoe and N. Yamazoe, *Chem. Phys. Lett.*, 2005, **410**, 321–323.
55 K. Großmann, S. Wicker, U. Weimar and N. Barsan, *Phys. Chem. Chem. Phys.*, 2013, **15**, 19151–19158.
56 P. Shankar and J. B. B. Rayappa, *Sci. Lett. J.*, 2015, **4**, 1–18.
57 H. R. Kim, A. Haensch, I. D. Kim, N. Barsan, U. Weimar and J. H. Lee, *Adv. Funct. Mater.*, 2011, **21**, 4456–4463.
58 N. Ma, K. Suematsu, M. Yuasa, T. Kida and K. Shimanoe, *ACS Appl. Mater. Interfaces*, 2015, **7**, 5863–5869.
59 K. Suematsu, M. Sasaki, N. Ma, M. Yuasa and K. Shimanoe, *ACS Sens.*, 2016, **1**, 913–920.
60 J. W. Yoon, J. S. Kim, T. H. Kim, Y. J. Hong, Y. C. Kang and J. H. Lee, *Small*, 2016, **12**, 4229–4240.
61 C. S. Lee, Z. Dai, S. Y. Jeong, C. H. Kwak, B. Y. Kim, D. H. Kim, H. W. Jang, J. S. Park and J. H. Lee, *Chem. – Eur. J.*, 2016, **22**, 7102–7107.
62 H. Y. Li, L. Huang, X. X. Wang, C. S. Lee, J. W. Yoon, J. Zhou, X. Guo and J. H. Lee, *RSC Adv.*, 2017, **7**, 3680–3685.
63 J. Yi, J. M. Lee and W. I. Park, *Sens. Actuators, B*, 2011, **155**, 264–269.
64 K. Alving, E. Weitzberg and J. M. Lundberg, *Eur. Respir. J.*, 1993, **6**, 1368–1370.
65 J. Morton, R. L. Henry and P. S. Thomas, *Pediatr Pulmonol*, 2006, **41**, 929–936.
66 H. G. Moon, Y. R. Choi, Y. S. Shim, K. I. Choi, J. H. Lee, J. S. Kim, S. J. Yoon, H. H. Park, C. Y. Kang and H. W. Jang, *ACS Appl. Mater. Interfaces*, 2013, **5**, 10591–10596.
67 S. T. Krishnan, J. P. Devadhasan and S. Kim, *Anal. Bioanal. Chem.*, 2017, **409**, 21–31.
68 S. Davies, P. Spanel and D. Smith, *Kidney Int.*, 1997, **52**, 223–228.
69 S. DuBois, S. Eng, R. Bhattacharya, S. Rulyak, T. Hubbard, D. Putnam and D. J. Kearney, *Dig. Dis. Sci.*, 2005, **50**, 1780–1784.
70 D. J. Kearney, T. Hubbard and D. Putnam, *Dig. Dis. Sci.*, 2002, **47**, 2523–2530.
71 D. Mutschall, K. Holzner and E. Obermeier, *Sens. Actuators, B*, 1996, **36**, 320–324.
72 C. Imawan, F. Solzbacher, H. Steffes and E. Obermeier, *Sens. Actuators, B*, 2000, **64**, 193–197.
73 S. S. Sunu, E. Prabhu, V. Jayaraman, K. I. Gnanasekar, T. K. Seshagiri and T. Gnanasekaran, *Sens. Actuators, B*, 2004, **101**, 161–174.
74 V. Srivastava and K. Jain, *Sens. Actuators, B*, 2008, **133**, 46–52.
75 I. Jimenez, M. A. Centeno, R. Scotti, F. Morazzoni, J. Arbiol, A. Cornet and J. R. Morante, *J. Mater. Chem.*, 2004, **14**, 2412–2420.
76 C. Zamani, O. Casals, T. Andreu, J. R. Morante and A. Romano-Rodriguez, *Sens. Actuators, B*, 2009, **140**, 557–562.
77 P. Gouma, K. Kalyanasundaram, X. Yun, M. Stanacevic and L. Wang, *IEEE Sens. J.*, 2010, **10**, 49–53.
78 A. T. Güntner, M. Righettoni and S. E. Pratsinis, *Sens. Actuators, B*, 2016, **223**, 266–273.
79 P. Galassetti, B. Novak, D. Nemet, C. Rose-Gottron, D. M. Cooper, S. Meinardi, R. Newcomb, F. Zaldivar and D. R. Blake, *Diabetes Technol. Ther.*, 2005, **7**, 115–123.
80 S. J. Kim, S. J. Choi, J. S. Jang, H. J. Cho and I. D. Kim, *Acc. Chem. Res.*, 2017, **50**, 1587–1596.
81 J. King, A. Kupferthaler, K. Unterkofler, H. Koc, S. Teschl, G. Teschl, W. Miekisch, J. Schubert, H. Hinterhuber and A. Amann, *J. Breath Res.*, 2009, **3**, 027006.
82 C. Deng, J. Zhang, X. Yu, W. Zhang and X. Zhang, *J. Chromatogr. B: Anal. Technol. Biomed. Life Sci.*, 2004, **810**, 269–275.
83 S. J. Choi, I. Lee, B. H. Jang, D. Y. Youn, W. H. Ryu, C. O. Park and I. D. Kim, *Anal. Chem.*, 2013, **85**, 1792–1796.
84 S. J. Choi, S. J. Kim, H. J. Cho, J. S. Jang, Y. M. Lin, H. L. Tuller, G. C. Rutledge and I. D. Kim, *Small*, 2016, **12**, 911–920.
85 S. J. Kim, S. J. Choi, J. S. Jang, N. H. Kim, M. Hakim, H. L. Tuller and I. D. Kim, *ACS Nano*, 2016, **10**, 5891–5899.
86 M. Righettoni, A. Tricoli and S. E. Pratsinis, *Anal. Chem.*, 2010, **82**, 3581–3587.
87 M. Righettoni, A. Tricoli and S. E. Pratsinis, *Chem. Mater.*, 2010, **22**, 3152–3157.
88 M. Righettoni and A. Tricoli, *J. Breath Res.*, 2011, **5**, 037109.
89 J. Shin, S. J. Choi, I. Lee, D. Y. Youn, C. O. Park, J. H. Lee, H. L. Tuller and I. D. Kim, *Adv. Funct. Mater.*, 2013, **23**, 2357–2367.
90 W. T. Koo, S. J. Choi, J. S. Jang and I. D. Kim, *Sci. Rep.*, 2017, **7**, 45074.
91 K. H. Kim, S. J. Kim, H. J. Cho, N. H. Kim, J. S. Jang, S. J. Choi and I. D. Kim, *Sens. Actuators, B*, 2017, **241**, 1276–1282.
92 W. T. Koo, S. Yu, S. J. Choi, J. S. Jang, J. Y. Cheong and I. D. Kim, *ACS Appl. Mater. Interfaces*, 2017, **9**, 8201–8210.
93 M. Righettoni, A. Tricoli, S. Gass, A. Schmid, A. Amann and S. E. Pratsinis, *Anal. Chim. Acta*, 2012, **738**, 69–75.
94 S.-J. Choi, I. Lee, B.-H. Jang, D.-Y. Youn, W.-H. Ryu, C. O. Park and I.-D. Kim, *Anal. Chem.*, 2013, **85**, 1792–1796.
95 W.-T. Koo, S.-J. Choi, J.-S. Jang and I.-D. Kim, *Sci. Rep.*, 2017, **7**, 45074.
96 N.-H. Kim, S.-J. Choi, S.-J. Kim, H.-J. Cho, J.-S. Jang, W.-T. Koo, M. Kim and I.-D. Kim, *Sens. Actuators, B*, 2016, **224**, 185–192.

Published on 11 June 2018. Downloaded on 2/1/2025 6:21:23 AM.

97 O. Lupan, V. Postica, N. Wolff, O. Polonskyi, V. Duppel, V. Kaidas, E. Lazari, N. Ababii, F. Faupel and L. Kienle, *Small*, 2017, **13**, 1602868.
98 N. H. Kim, S. J. Choi, S. J. Kim, H. J. Cho, J. S. Jang, W. T. Koo, M. K. Kim and I. D. Kim, *Sens. Actuators, B*, 2016, **224**, 185–192.
99 J.-W. Yoon and J.-H. Lee, *Lab Chip*, 2017, **17**, 3537–3557.
100 J. Rao, A. Yu, C. Shao and X. Zhou, *ACS Appl. Mater. Interfaces*, 2012, **4**, 5346–5352.
101 S. Xiong, J. Xu, D. Chen, R. Wang, X. Hu, G. Shen and Z. L. Wang, *CrystEngComm*, 2011, **13**, 7114–7120.
102 T. Jinkawa, G. Sakai, J. Tamaki, N. Miura and N. Yamazoe, *J. Mol. Catal. A: Chem.*, 2000, **155**, 193–200.
103 H. Fan, T. Zhang, X. Xu and N. Lv, *Sens. Actuators, B*, 2011, **153**, 83–88.
104 M. Siemons and U. Simon, *Sens. Actuators, B*, 2007, **126**, 181–186.
105 L. Wang, Z. Lou, J. Deng, R. Zhang and T. Zhang, *ACS Appl. Mater. Interfaces*, 2015, **7**, 13098–13104.
106 Q. Hao, S. Liu, X. Yin, Z. Du, M. H. Zhang, L. Li, Y. Wang, T. Wang and Q. Li, *CrystEngComm*, 2011, **13**, 806–812.
107 F. Zhang, H. Yang, X. Xie, L. Li, L. Zhang, J. Yu, H. Zhao and B. Liu, *Sens. Actuators, B*, 2009, **141**, 381–389.
108 A. Tangerman, *Int. Dent. J.*, 2002, **52**, 201–206.
109 M. K. Nakhleh, M. Quatredeniers and H. Haick, *Oral Dis.*, 2017, 1–11.
110 N. S. Ramgir, S. K. Ganapathi, M. Kaur, N. Datta, K. P. Muthe, D. K. Aswal, S. K. Gupta and J. V. Yakhmi, *Sens. Actuators, B*, 2010, **151**, 90–96.
111 X. Xue, L. Xing, Y. Chen, S. Shi, Y. Wang and T. Wang, *J. Phys. Chem. C*, 2008, **112**, 12157–12160.
112 Y. Qin, F. Zhang, Y. Chen, Y. Zhou, J. P. Li, A. Zhu, Y. Luo, Y. Tian and J. Yang, *J. Phys. Chem. C*, 2012, **116**, 11994–12000.
113 F. Zhang, A. Zhu, Y. Luo, Y. Tian, J. Yang and Y. Qin, *J. Phys. Chem. C*, 2010, **114**, 19214–19219.
114 X. Liang, T. H. Kim, J. W. Yoon, C. H. Kwak and J. H. Lee, *Sens. Actuators, B*, 2015, **209**, 934–942.
115 H. S. Woo, C. H. Kwak, I. D. Kim and J. H. Lee, *J. Mater. Chem. A*, 2014, **2**, 6412–6418.
116 I. Giebelhaus, E. Varechkina, T. Fischer, M. Rumyantseva, V. Ivanov, A. Gaskov, J. R. Morante, J. Arbiol, W. Tyrra and S. Mathur, *J. Mater. Chem. A*, 2013, **1**, 11261–11268.
117 J. Chen, K. Wang, L. Hartman and W. Zhou, *J. Phys. Chem. C*, 2008, **112**, 16017–16021.
118 S. Steinhauer, E. Brunet, T. Maier, G. C. Mutinati, A. Köck, O. Freudenberg, C. Gspan, W. Grogger, A. Neuhold and R. Resel, *Sens. Actuators, B*, 2013, **187**, 50–57.
119 J. Tamaki, K. Shimanoe, Y. Yamada, Y. Yamamoto, N. Miura and N. Yamazoe, *Sens. Actuators, B*, 1998, **49**, 121–125.
120 I. S. Hwang, J. K. Choi, S. J. Kim, K. Y. Dong, J. H. Kwon, B. K. Ju and J. H. Lee, *Sens. Actuators, B*, 2009, **142**, 105–110.
121 S. Steinhauer, E. Brunet, T. Maier, G. C. Mutinati and A. Köck, *Sens. Actuators, B*, 2013, **186**, 550–556.
122 X. Gao, C. Li, Z. Yin and Y. Chen, *RSC Adv.*, 2015, **5**, 37703–37709.
123 H. L. Yu, L. Li, X. M. Gao, Y. Zhang, F. Meng, T. S. Wang, G. Xiao, Y. J. Chen and C. L. Zhu, *Sens. Actuators, B*, 2012, **171**, 679–685.
124 S. Kabcum, N. Tammanoon, A. Wisitsoraat, A. Tuantranont, S. Phanichphant and C. Liewhiran, *Sens. Actuators, B*, 2016, **235**, 678–690.
125 J. W. Yoon, Y. J. Hong, Y. C. Kang and J. H. Lee, *RSC Adv.*, 2014, **4**, 16067–16074.
126 C. H. Liu, L. Zhang and Y. J. He, *Thin Solid Films*, 1997, **304**, 13–15.
127 S. Ma, J. Jia, Y. Tian, L. Cao, S. Shi, X. Li and X. Wang, *Ceram. Int.*, 2016, **42**, 2041–2044.
128 Y. Wang, Y. Wang, J. Cao, F. Kong, H. Xia, J. Zhang, B. Zhu, S. Wang and S. Wu, *Sens. Actuators, B*, 2008, **131**, 183–189.
129 S. Matsuda, T. Kamo, J. Imahashi and F. Nakajima, *Ind. Eng. Chem. Fundam.*, 1982, **21**, 18–22.
130 S. Y. Jeong, J. W. Yoon, T. H. Kim, H. M. Jeong, C. S. Lee, Y. C. Kang and J. H. Lee, *J. Mater. Chem. A*, 2017, **5**, 1446–1454.
131 H. M. Jeong, H. J. Kim, P. Rai, J. W. Yoon and J. H. Lee, *Sens. Actuators, B*, 2014, **201**, 482–489.
132 H. J. Kim, J. W. Yoon, K. I. Choi, H. W. Jang, A. Umar and J. H. Lee, *Nanoscale*, 2013, **5**, 7066–7073.
133 J. W. Yoon, Y. J. Hong, G. D. Park, S. J. Hwang, F. Abdel-Hady, A. A. Wazzan, Y. C. Kang and J. H. Lee, *ACS Appl. Mater. Interfaces*, 2015, **7**, 7717–7723.
134 S. J. Hwang, K. I. Choi, J. W. Yoon, Y. C. Kang and J. H. Lee, *Chem. – Eur. J.*, 2015, **21**, 5872–5878.
135 H. S. Woo, C. H. Kwak, J. H. Chung and J. H. Lee, *Sens. Actuators, B*, 2015, **216**, 358–366.
136 H. S. Woo, C. H. Kwak, J. H. Chung and J. H. Lee, *ACS Appl. Mater. Interfaces*, 2014, **6**, 22553–22560.
137 K. Xu, J. Zou, S. Tian, Y. Yang, F. Zeng, T. Yu, Y. Zhang, X. Jie and C. Yuan, *Sens. Actuators, B*, 2017, **246**, 68–77.
138 B. Y. Kim, J. H. Ahn, J. W. Yoon, C. S. Lee, Y. C. Kang, F. Abdel-Hady, A. A. Wazzan and J. H. Lee, *ACS Appl. Mater. Interfaces*, 2016, **8**, 34603–34611.
139 H. M. Jeong, J. H. Kim, S. Y. Jeong, C. H. Kwak and J. H. Lee, *ACS Appl. Mater. Interfaces*, 2016, **8**, 7877–7883.
140 J. H. Kim, H. M. Jeong, C. W. Na, J. W. Yoon, F. Abdel-Hady, A. A. Wazzan and J. H. Lee, *Sens. Actuators, B*, 2016, **235**, 498–506.
141 S. Liu, Z. Wang, H. Zhao, T. Fei and T. Zhang, *Sens. Actuators, B*, 2014, **197**, 342–349.
142 M. Drobek, J.-H. Kim, M. Bechelany, C. Vallicari, A. Julbe and S. S. Kim, *ACS Appl. Mater. Interfaces*, 2016, **8**, 8323–8328.
143 A. T. Güntner, V. Koren, K. Chikkadi, M. Righettoni and S. E. Pratsinis, *ACS Sens.*, 2016, **1**, 528–535.
144 S. J. Choi, K. H. Ku, B. J. Kim and I. D. Kim, *ACS Sens.*, 2016, **1**, 1124–1131.
145 H. G. Moon, Y. H. Jung, S. D. Han, Y. S. Shim, B. Shin, T. Lee, J. S. Kim, S. Lee, S. C. Jun and H. H. Park, *ACS Appl. Mater. Interfaces*, 2016, **8**, 20969–20976.

 This journal is © The Royal Society of Chemistry 2018

Published on 11 June 2018. Downloaded on 2/1/2025 6:21:23 AM.

146 E. Dovgolevsky, U. Tisch and H. Haick, *Small*, 2009, **5**, 1158–1161.
147 L. Y. Wang, J. Luo, M. J. Schadt and C. J. Zhong, *Langmuir*, 2010, **26**, 618–632.
148 Y. Joseph, B. Guse, T. Vossmeyer and A. Yasuda, *J. Phys. Chem. C*, 2008, **112**, 12507–12514.
149 U. Tisch and H. Haick, *Rev. Chem. Eng.*, 2010, **26**, 171–179.
150 M. C. Daniel and D. Astruc, *Chem. Rev.*, 2004, **104**, 293–346.
151 Y. Joseph, B. Guse, A. Yasuda and T. Vossmeyer, *Sens. Actuators, B*, 2004, **98**, 188–195.
152 E. García-Berríos, T. Gao, J. C. Theriot, M. D. Woodka, B. S. Brunschwig and N. S. Lewis, *J. Phys. Chem. C*, 2011, **115**, 6208–6217.
153 L. J. Hubble, J. S. Cooper, A. Sosa-Pintos, H. Kiiveri, E. Chow, M. S. Webster, L. Wieczorek and B. Raguse, *ACS Comb. Sci.*, 2015, **17**, 120–129.
154 M. Segev-Bar, N. Bachar, Y. Wolf, B. Ukrainsky, L. Sarraf and H. Haick, *Adv. Carbon Mater. Technol.*, 2017, **2**, 1600206.
155 M. Y. Bashouti, A. S. de la Zerda, D. Geva and H. Haick, *J. Phys. Chem. C*, 2014, **118**, 1903–1909.
156 N. Kahn, O. Lavie, M. Paz, Y. Segev and H. Haick, *Nano Lett.*, 2015, **15**, 7023–7028.
157 N. Olichwer, E. W. Leib, A. H. Halfar, A. Petrov and T. Vossmeyer, *ACS Appl. Mater. Interfaces*, 2012, **4**, 6151–6161.
158 H. Wohltjen and A. W. Snow, *Anal. Chem.*, 1998, **70**, 2856–2859.
159 Q.-Y. Cai and E. T. Zellers, *Anal. Chem.*, 2002, **74**, 3533–3539.
160 H. L. Zhang, S. D. Evans, J. R. Henderson, R. E. Miles and T. H. Shen, *Nanotechnology*, 2002, **13**, 439.
161 W. Zhao, T. Rovere, D. Weerawarne, G. Osterhoudt, N. Kang, P. Joseph, J. Luo, B. Shim, M. Poliks and C.-J. Zhong, *ACS Nano*, 2015, **9**, 6168–6177.
162 J. Herrmann, K. H. Muller, T. Reda, G. R. Baxter, B. Raguse, G. J. J. B. de Groot, R. Chai, M. Roberts and L. Wieczorek, *Appl. Phys. Lett.*, 2007, **91**, 183105.
163 E. Homede, M. Abo Jabal, R. Ionescu and H. Haick, *Adv. Funct. Mater.*, 2016, **26**, 6359–6370.
164 J. Lombardi, M. D. Poliks, W. Zhao, S. Yan, N. Kang, J. Li, J. Luo, C.-J. Zhong, Z. Pan and M. Almihdhar, *IEEE 67th Electronic Components and Technology Conference (ECTC)*, Orlando, FL, 2017, pp. 764–771.
165 G. Yang and D. T. Hallinan Jr, *Nanotechnology*, 2016, **27**, 225604.
166 L. Wang, L. Wang, X. Shi, N. N. Kariuki, M. Schadt, G. R. Wang, Q. Rendeng, J. Choi, J. Luo and S. Lu, *J. Am. Chem. Soc.*, 2007, **129**, 2161–2170.
167 H. Moreira, J. Grisolia, N. M. Sangeetha, N. Decorde, C. Farcau, B. Viallet, K. Chen, G. Viau and L. Ressier, *Nanotechnology*, 2013, **24**, 095701.
168 C. Farcau, N. M. Sangeetha, H. Moreira, B. Viallet, J. Grisolia, D. Ciuculescu-Pradines and L. Ressier, *ACS Nano*, 2011, **5**, 7137–7143.
169 C. Farcau, H. Moreira, B. Viallet, J. Grisolia, D. Ciuculescu-Pradines, C. Amiens and L. Ressier, *J. Phys. Chem. C*, 2011, **115**, 14494–14499.
170 N. M. Sangeetha, N. Decorde, B. Viallet, G. Viau and L. Ressier, *J. Phys. Chem. C*, 2012, **117**, 1935–1940.
171 L. Y. Wang, J. Luo, J. Yin, H. Zhang, J. M. Wu, X. Shi, E. Crew, Z. Xu, Q. Rendeng and S. Lu, *J. Mater. Chem.*, 2010, **20**, 907–915.
172 C. Xu and X. Wang, *Small*, 2009, **5**, 2212–2217.
173 Y. G. Sun and H. H. Wang, *Appl. Phys. Lett.*, 2007, **90**, 213107.
174 M. Segev-Bar, A. Landman, M. Nir-Shapira, G. Shuster and H. Haick, *ACS Appl. Mater. Interfaces*, 2013, **5**, 5531–5541.
175 M. Segev-Bar and H. Haick, *ACS Nano*, 2013, **7**, 8366–8378.
176 H. Haick, *J. Phys. D: Appl. Phys.*, 2007, **40**, 7173–7186.
177 O. Masala and R. Seshadri, *Annu. Rev. Mater. Res.*, 2004, **34**, 41–81.
178 B. L. Cushing, V. L. Kolesnichenko and C. J. O'Connor, *Chem. Rev.*, 2004, **104**, 3893–3946.
179 C. J. Murphy, *Anal. Chem.*, 2002, **74**, 520A–526A.
180 J. Yin, P. P. Hu, J. Luo, L. Wang, M. F. Cohen and C. J. Zhong, *ACS Nano*, 2011, **5**, 6516–6526.
181 X. H. Qian and H. S. Park, *J. Mech. Phys. Solids*, 2010, **58**, 330–345.
182 X. H. Qian and H. S. Park, *Nanotechnology*, 2010, **21**, 365704.
183 E. Dovgolevsky, G. Konvalina, U. Tisch and H. Haick, *J. Phys. Chem. C*, 2010, **114**, 14042–14049.
184 E. Dovgolevsky and H. Haick, *Small*, 2008, **4**, 2059–2066.
185 F. Xiao, J. Song, H. Gao, X. Zan, R. Xu and H. Duan, *ACS Nano*, 2011, **6**, 100–110.
186 T. Yonezawa, K. Yasui and N. Kimizuka, *Langmuir*, 2001, **17**, 271–273.
187 D. Rautaray, A. Kumar, S. Reddy, S. R. Sainkar and M. Sastry, *Cryst. Growth Des.*, 2002, **2**, 197–203.
188 R. A. Potyrailo, *Chem. Soc. Rev.*, 2017, **46**, 5311–5346.
189 K. Fu, S. Li, X. Jiang, Y. Wang and B. G. Willis, *Langmuir*, 2013, **29**, 14335–14343.
190 M. Segev-Bar, G. Shuster and H. Haick, *J. Phys. Chem. C*, 2012, **116**, 15361–15368.
191 G. Peng, U. Tisch, O. Adams, M. Hakim, N. Shehada, Y. Y. Broza, S. Billan, R. Abdah-Bortnyak, A. Kuten and H. Haick, *Nat. Nanotechnol.*, 2009, **4**, 669–673.
192 Y. Joseph, A. Peic, X. Chen, J. Michl, T. Vossmeyer and A. Yasuda, *J. Phys. Chem. C*, 2007, **111**, 12855–12859.
193 E. García-Berríos, T. Gao, M. D. Woodka, S. Maldonado, B. S. Brunschwig, M. W. Ellsworth and N. S. Lewis, *J. Phys. Chem. C*, 2010, **114**, 21914–21920.
194 Y. Joseph, I. Besnard, M. Rosenberger, B. Guse, H. G. Nothofer, J. M. Wessels, U. Wild, A. Knop-Gericke, D. Su and R. Schlögl, *J. Phys. Chem. B*, 2003, **107**, 7406–7413.
195 N. Krasteva, Y. Fogel, R. E. Bauer, K. Müllen, Y. Joseph, N. Matsuzawa, A. Yasuda and T. Vossmeyer, *Adv. Funct. Mater.*, 2007, **17**, 881–888.
196 N. Krasteva, B. Guse, I. Besnard, A. Yasuda and T. Vossmeyer, *Sens. Actuators, B*, 2003, **92**, 137–143.
197 M. D. Hanwell, S. Y. Heriot, T. H. Richardson, N. Cowlam and I. M. Ross, *Colloids Surf., A*, 2006, **284**, 379–383.
198 J. P. Choi, M. M. Coble, M. R. Branham, J. M. DeSimone and R. W. Murray, *J. Phys. Chem. C*, 2007, **111**, 3778–3785.
199 M. A. Jabal, E. Homede, L. M. Pismen, H. Haick and A. M. Leshansky, *Eur. Phys. J.-Spec. Top.*, 2017, **226**, 1307–1324.

Published on 11 June 2018. Downloaded on 2/1/2025 6:21:23 AM.

200 Y. Y. Broza, L. Har-Shai, R. Jeries, J. C. Cancilla, L. Glass-Marmor, I. Lejbkowicz, J. S. Torrecilla, X. Yao, X. Feng, A. Narita, K. Mullen, A. Miller and H. Haick, *ACS Chem. Neurosci.*, 2017, **8**, 2402–2413.
201 A. Karban, M. K. Nakhleh, J. C. Cancilla, R. Vishinkin, T. Rainis, E. Koifman, R. Jeries, H. Ivgi, J. S. Torrecilla and H. Haick, *Adv. Healthcare Mater.*, 2016, **5**, 2339–2344.
202 S. Shan, W. Zhao, J. Luo, J. Yin, J. C. Switzer, P. Joseph, S. Lu, M. Poliks and C. J. Zhong, *J. Mater. Chem. C*, 2014, **2**, 1893–1903.
203 H. Amal, D. Y. Shi, R. Ionescu, W. Zhang, Q. L. Hua, Y. Y. Pan, L. Tao, H. Liu and H. Haick, *Int. J. Cancer*, 2015, **136**, E614–E622.
204 H. Jin, T. P. Huynh and H. Haick, *Nano Lett.*, 2016, **16**, 4194–4202.
205 T. P. Huynh and H. Haick, *Adv. Mater.*, 2015, **28**, 138–143.
206 G. Konvalina and H. Haick, *ACS Appl. Mater. Interfaces*, 2012, **4**, 317–325.
207 R. L. van der Waals, G. M. Berger and T. M. Ticich, *Appl. Phys. A: Mater. Sci. Process.*, 2003, **77**, 885–889.
208 C. D. Scott, S. Arepalli, P. Nikolaev and R. E. Smalley, *Appl. Phys. A: Mater. Sci. Process.*, 2001, **72**, 573–580.
209 N. Braidy, M. M. El Khakani and G. G. Botton, *J. Mater. Res.*, 2002, **17**, 2189–2192.
210 S. Arepalli, *J. Nanosci. Nanotechnol.*, 2004, **4**, 317–325.
211 S. Iijima and T. Ichihashi, *Nature*, 1993, **363**, 603–605.
212 M. V. Antisari, R. Marazzi and R. Krsmanovic, *Carbon*, 2003, **41**, 2393–2401.
213 C. Journet, W. K. Maser, P. Bernier and A. Loiseau, *Nature*, 1997, **388**, 756.
214 H. Li, L. Guan, Z. Shi and Z. Gu, *J. Phys. Chem. B*, 2004, **108**, 4573–4575.
215 Z. Shi, Y. Lian, X. Zhou, Z. Gu, Y. Zhang, S. Iijima, L. Zhou, K. T. Yue and S. Zhang, *Carbon*, 1999, **37**, 1449–1453.
216 J. L. Hutchison, N. A. Kiselev, E. P. Krinichnaya, A. V. Krestinin, R. O. Loutfy, A. P. Morawsky, V. E. Muradyan, E. D. Obraztsova, J. Sloan and S. V. Terekhov, *Carbon*, 2001, **39**, 761–770.
217 H. Dai, *Carbon Nanotubes*, 2001, 29–53.
218 L. C. Qin, D. Zhou, A. R. Krauss and D. M. Gruen, *Appl. Phys. Lett.*, 1998, **72**, 3437–3439.
219 V. K. Varadan and J. Xie, *Smart Mater. Struct.*, 2002, **11**, 610.
220 K. Mukhopadhyay and G. N. Mathur, *J. Nanosci. Nanotechnol.*, 2002, **2**, 197–201.
221 R. Masud, I. Dauda Sh, M. Asyraf, S. Jarin and A. Tomal, *Sens. Rev.*, 2017, **37**, 127–136.
222 K. Hata, D. N. Futaba, K. Mizuno, T. Namai, M. Yumura and S. Iijima, *Science*, 2004, **306**, 1362–1364.
223 B. Q. Wei, R. Vajtai and P. M. Ajayan, *Appl. Phys. Lett.*, 2001, **79**, 1172–1174.
224 M. J. Treacy, T. W. Ebbesen and J. M. Gibson, *Nature*, 1996, **381**, 678.
225 W. Zhang, Z. Zhu, F. Wang, T. Wang, L. Sun and Z. Wang, *Nanotechnology*, 2004, **15**, 936.
226 P. Avouris, Z. Chen and V. Perebeinos, *Nat. Nanotechnol.*, 2007, **2**, 605–615.
227 T. Dürkop, S. A. Getty, E. Cobas and M. S. Fuhrer, *Nano Lett.*, 2004, **4**, 35–39.
228 T. W. Ebbesen, H. J. Lezec, H. Hiura, J. W. Bennett, H. F. Ghaemi and T. Thio, *Nature*, 1996, **382**, 54–56.
229 M. S. Dresselhaus, G. Dresselhaus and P. C. Eklund, *Science of fullerenes and carbon nanotubes: their properties and applications*, Elsevier, Orlando, Florida, 1996.
230 E. Pop, D. Mann, Q. Wang, K. Goodson and H. Dai, *Nano Lett.*, 2006, **6**, 96–100.
231 P. Kim, L. Shi, A. Majumdar and P. L. McEuen, *Phys. Rev. Lett.*, 2001, **87**, 215502.
232 M.-F. Yu, O. Lourie, M. J. Dyer, K. Moloni, T. F. Kelly and R. S. Ruoff, *Science*, 2000, **287**, 637–640.
233 M.-F. Yu, B. S. Files, S. Arepalli and R. S. Ruoff, *Phys. Rev. Lett.*, 2000, **84**, 5552.
234 B. Peng, M. Locascio, P. Zapol, S. Li, S. L. Mielke, G. C. Schatz and H. D. Espinosa, *Nat. Nanotechnol.*, 2008, **3**, 626–631.
235 J.-P. Salvetat, G. A. D. Briggs, J.-M. Bonard, R. R. Bacsa, A. J. Kulik, T. Stöckli, N. A. Burnham and L. Forró, *Phys. Rev. Lett.*, 1999, **82**, 944.
236 E. W. Wong, P. E. Sheehan and C. M. Lieber, *Science*, 1997, **277**, 1971–1975.
237 A. Zahab, L. Spina, P. Poncharal and C. Marliere, *Phys. Rev. B: Condens. Matter Mater. Phys.*, 2000, **62**, 10000.
238 G. Peng, U. Tisch and H. Haick, *Nano Lett.*, 2009, **9**, 1362–1368.
239 G. Peng, E. Trock and H. Haick, *Nano Lett.*, 2008, **8**, 3631–3635.
240 H. Haick, M. Hakim, M. Patrascu, C. Levenberg, N. Shehada, F. Nakhoul and Z. Abassi, *ACS Nano*, 2009, **3**, 1258–1266.
241 Y. Zilberman, U. Tisch, W. Pisula, X. Feng, K. Müllen and H. Haick, *Langmuir*, 2009, **25**, 5411–5416.
242 Y. Zilberman, U. Tisch, G. Shuster, W. Pisula, X. Feng, K. Mullen and H. Haick, *Adv. Mater.*, 2010, **22**, 4317–4320.
243 Y. Zilberman, R. Ionescu, X. Feng, K. Müllen and H. Haick, *ACS Nano*, 2011, **5**, 6743–6753.
244 N. Bachar, L. Mintz, Y. Zilberman, R. Ionescu, X. Feng, K. Müllen and H. Haick, *ACS Appl. Mater. Interfaces*, 2012, **4**, 4960–4965.
245 J. Zhao, A. Buldum, J. Han and J. P. Lu, *Nanotechnology*, 2002, **13**, 195.
246 T. Zhang, S. Mubeen, N. V. Myung and M. A. Deshusses, *Nanotechnology*, 2008, **19**, 332001.
247 P. Bondavalli, P. Legagneux and D. Pribat, *Sens. Actuators, B*, 2009, **140**, 304–318.
248 M. Magno, V. Jelicic, K. Chikkadi, C. Roman, C. Hierold, V. Bilas and L. Benini, *IEEE Sens. J.*, 2016, **16**, 8329–8337.
249 J. Kong, N. R. Franklin, C. Zhou, M. G. Chapline, S. Peng, K. Cho and H. Dai, *Science*, 2000, **287**, 622–625.
250 X. Liu, Z. Luo, S. Han, T. Tang, D. Zhang and C. Zhou, *Appl. Phys. Lett.*, 2005, **86**, 243501.
251 I. Dube, D. Jiménez, G. Fedorov, A. Boyd, I. Gayduchenko, M. Paranjape and P. Barbara, *Carbon*, 2015, **87**, 330–337.
252 J. Zhang, A. Boyd, A. Tselev, M. Paranjape and P. Barbara, *Appl. Phys. Lett.*, 2006, **88**, 123112.

This journal is © The Royal Society of Chemistry 2018

Published on 11 June 2018. Downloaded on 2/1/2025 6:21:23 AM.

253 N. Peng, Q. Zhang, C. L. Chow, O. K. Tan and N. Marzari, *Nano Lett.*, 2009, **9**, 1626–1630.
254 Y. W. Chang, J. S. Oh, S. H. Yoo, H. H. Choi and K.-H. Yoo, *Nanotechnology*, 2007, **18**, 435504.
255 C. Zhou, J. Zhao, J. Ye, M. Tange, X. Zhang, W. Xu, K. Zhang, T. Okazaki and Z. Cui, *Carbon*, 2016, **108**, 372–380.
256 J. Li, Y. Lu, Q. Ye, L. Delzeit and M. Meyyappan, *Electrochem. Solid-State Lett.*, 2005, **8**, H100–H102.
257 G. Chen, T. M. Paronyan, E. M. Pigos and A. R. Harutyunyan, *Sci. Rep.*, 2012, **2**, 1–7.
258 M. Rinkiö, M. Y. Zavodchikova, P. Törmä and A. Johansson, *Phys. Status Solidi B*, 2008, **245**, 2315–2318.
259 W. Kim, A. Javey, O. Vermesh, Q. Wang, Y. Li and H. Dai, *Nano Lett.*, 2003, **3**, 193–198.
260 S. A. McGill, S. G. Rao, P. Manandhar, P. Xiong and S. Hong, *Appl. Phys. Lett.*, 2006, **89**, 163123.
261 D. Sung, S. Hong, Y.-H. Kim, N. Park, S. Kim, S. L. Maeng and K.-C. Kim, *Appl. Phys. Lett.*, 2006, **89**, 243110.
262 A. Star, V. Joshi, S. Skarupo, D. Thomas and J.-C. P. Gabriel, *J. Phys. Chem. B*, 2006, **110**, 21014–21020.
263 S. Mubeen, J. H. Lim, A. Srirangarajan, A. Mulchandani, M. A. Deshusses and N. V. Myung, *Electroanalysis*, 2011, **23**, 2687–2692.
264 D. R. Kauffman and A. Star, *Nano Lett.*, 2007, **7**, 1863–1868.
265 K. H. An, S. Y. Jeong, H. R. Hwang and Y. H. Lee, *Adv. Mater.*, 2004, **16**, 1005–1009.
266 P. Qi, O. Vermesh, M. Grecu, A. Javey, Q. Wang, H. Dai, S. Peng and K. J. Cho, *Nano Lett.*, 2003, **3**, 347–351.
267 C. Staii, A. T. Johnson, M. Chen and A. Gelperin, *Nano Lett.*, 2005, **5**, 1774–1778.
268 R. J. Chen, N. R. Franklin, J. Kong, J. Cao, T. W. Tombler, Y. Zhang and H. Dai, *Appl. Phys. Lett.*, 2001, **79**, 2258–2260.
269 S. Badhulika, N. V. Myung and A. Mulchandani, *Talanta*, 2014, **123**, 109–114.
270 J. Li, Y. Lu, Q. Ye, M. Cinke, J. Han and M. Meyyappan, *Nano Lett.*, 2003, **3**, 929–933.
271 J. Suehiro, G. Zhou, H. Imakiire, W. Ding and M. Hara, *Sens. Actuators, B*, 2005, **108**, 398–403.
272 D. Kumar, P. Chaturvedi, P. Saho, P. Jha, A. Chouksey, M. Lal, J. S. B. S. Rawat, R. P. Tandon and P. K. Chaudhury, *Sens. Actuators, B*, 2017, **240**, 1134–1140.
273 S. M. Jung, H. Y. Jung and J. S. Suh, *Sens. Actuators, B*, 2009, **139**, 425–428.
274 Y. Jiang, P. Wang and L. Lin, *IEEE 24th International Conference on Micro Electro Mechanical Systems*, Cancun, 2011, pp. 396–399.
275 S. J. Young and Z. D. Lin, *Microsyst. Technol.*, 2016, 1–4.
276 M. Penza, R. Rossi, M. Alvisi and E. Serra, *Nanotechnology*, 2010, **21**, 105501.
277 M. Penza, G. Cassano, R. Rossi, M. Alvisi, A. Rizzo, M. A. Signore, T. Dikonimos, E. Serra and R. Giorgi, *Appl. Phys. Lett.*, 2007, **90**, 173123.
278 A. Abdelhalim, M. Winkler, F. Loghin, C. Zeiser, P. Lugli and A. Abdellah, *Sens. Actuators, B*, 2015, **220**, 1288–1296.
279 M. Penza, R. Rossi, M. Alvisi, M. A. Signore, G. Cassano, D. Dimaio, R. Pentassuglia, E. Piscopiello, E. Serra and M. Falconieri, *Thin Solid Films*, 2009, **517**, 6211–6216.
280 D. W. H. Fam, A. I. Y. Tok, A. Palaniappan, P. Nopphawan, A. Lohani and S. G. Mhaisalkar, *Sens. Actuators, B*, 2009, **138**, 189–192.
281 R. Leghrib, A. Felten, F. Demoisson, F. Reniers, J.-J. Pireaux and E. Llobet, *Carbon*, 2010, **48**, 3477–3484.
282 E. H. Espinosa, R. Ionescu, C. Bittencourt, A. Felten, R. Erni, G. Van Tendeloo, J.-J. Pireaux and E. Llobet, *Thin Solid Films*, 2007, **515**, 8322–8327.
283 P. R. Mudimela, M. Scardamaglia, O. González-León, N. Reckinger, R. Snyders, E. Llobet, C. Bittencourt and J.-F. Colomer, *Beilstein J. Nanotechnol.*, 2014, **5**, 910.
284 E. Dilonardo, M. Penza, M. Alvisi, C. Di Franco, R. Rossi, F. Palmisano, L. Torsi and N. Cioffi, *Sens. Actuators, B*, 2016, **223**, 417–428.
285 J.-C. Charlier, L. Arnaud, I. V. Avilov, M. Delgado, F. Demoisson, E. H. Espinosa, C. P. l. Ewels, A. Felten, J. Guillot and R. Ionescu, *Nanotechnology*, 2009, **20**, 375501.
286 M. Penza, R. Rossi, M. Alvisi, G. Cassano and E. Serra, *Sens. Actuators, B*, 2009, **140**, 176–184.
287 J. Liu, Z. Guo, F. Meng, Y. Jia and J. Liu, *J. Phys. Chem. C*, 2008, **112**, 6119–6125.
288 N. Van Hieu, N. Van Duy, P. T. Huy and N. D. Chien, *Physica E*, 2008, **40**, 2950–2958.
289 R. Ionescu, E. H. Espinosa, R. Leghrib, A. Felten, J. J. Pireaux, R. Erni, G. Van Tendeloo, C. Bittencourt, N. Canellas and E. Llobet, *Sens. Actuators, B*, 2008, **131**, 174–182.
290 E. H. Espinosa, R. Ionescu, B. Chambon, G. Bedis, E. Sotter, C. Bittencourt, A. Felten, J.-J. Pireaux, X. Correig and E. Llobet, *Sens. Actuators, B*, 2007, **127**, 137–142.
291 C. Wei, L. Dai, A. Roy and T. B. Tolle, *J. Am. Chem. Soc.*, 2006, **128**, 1412–1413.
292 L. He, Y. Jia, F. Meng, M. Li and J. Liu, *Mater. Sci. Eng., B*, 2009, **163**, 76–81.
293 T. Zhang, M. B. Nix, B. Y. Yoo, M. A. Deshusses and N. V. Myung, *Electroanalysis*, 2006, **18**, 1153–1158.
294 S. Abdulla, T. L. Mathew and B. Pullithadathil, *Sens. Actuators, B*, 2015, **221**, 1523–1534.
295 J. K. Abraham, B. Philip, A. Witchurch, V. K. Varadan and C. C. Reddy, *Smart Mater. Struct.*, 2004, **13**, 1045.
296 T. Zhang, S. Mubeen, E. Bekyarova, B. Y. Yoo, R. C. Haddon, N. V. Myung and M. A. Deshusses, *Nanotechnology*, 2007, **18**, 165504.
297 G. P. Evans, D. J. Buckley, A.-L. Adedigba, G. Sankar, N. T. Skipper and I. P. Parkin, *ACS Appl. Mater. Interfaces*, 2016, **8**, 28096–28104.
298 S. F. Liu, L. C. H. Moh and T. M. Swager, *Chem. Mater.*, 2015, **27**, 3560–3563.
299 H. Xie, C. Sheng, X. Chen, X. Wang, Z. Li and J. Zhou, *Sens. Actuators, B*, 2012, **168**, 34–38.
300 S. Sharma, S. Hussain, S. Singh and S. S. Islam, *Sens. Actuators, B*, 2014, **194**, 213–219.
301 Y. Lu, C. Partridge, M. Meyyappan and J. Li, *J. Electroanal. Chem.*, 2006, **593**, 105–110.

Published on 11 June 2018. Downloaded on 2/1/2025 6:21:23 AM.

302 X. Xiong and M. Xia, Carbon nanotube-based ultra-sensitive breath acetone sensor for non-invasive diabetes diagnosis, University of Bridgeport, 2012.
303 S. Ahmadnia-Feyzabad, A. A. Khodadadi, M. Vesali-Naseh and Y. Mortazavi, *Sens. Actuators, B*, 2012, **166**, 150–155.
304 M. Ding, D. C. Sorescu and A. Star, *J. Am. Chem. Soc.*, 2013, **135**, 9015–9022.
305 C. Gao, Z. Guo, J.-H. Liu and X.-J. Huang, *Nanoscale*, 2012, **4**, 1948–1963.
306 E. Llobet, *Sens. Actuators, B*, 2013, **179**, 32–45.
307 A. Goldoni, L. Petaccia, S. Lizzit and R. Larciprete, *J. Phys.: Condens. Matter*, 2009, **22**, 013001.
308 T. Gao, M. D. Woodka, B. S. Brunschwig and N. S. Lewis, *Chem. Mater.*, 2006, **18**, 5193–5202.
309 E. T. Thostenson and T. W. Chou, *Adv. Mater.*, 2006, **18**, 2837–2841.
310 D. J. Lipomi, M. Vosgueritchian, B. C. Tee, S. L. Hellstrom, J. A. Lee, C. H. Fox and Z. Bao, *Nat. Nanotechnol.*, 2011, **6**, 788–792.
311 T. Yamada, Y. Hayamizu, Y. Yamamoto, Y. Yomogida, A. Izadi-Najafabadi, D. N. Futaba and K. Hata, *Nat. Nanotechnol.*, 2011, **6**, 296–301.
312 C. S. Woo, C. H. Lim, C. W. Cho, B. Park, H. Ju, D. H. Min, C. J. Lee and S. B. Lee, *Microelectron. Eng.*, 2007, **84**, 1610–1613.
313 B. Li, M. G. Hahm, Y. L. Kim, H. Y. Jung, S. Kar and Y. J. Jung, *ACS Nano*, 2011, **5**, 4826–4834.
314 F. Rigoni, S. Tognolini, P. Borghetti, G. Drera, S. Pagliara, A. Goldoni and L. Sangaletti, *Procedia Eng.*, 2014, **87**, 716–719.
315 F. Rigoni, G. Drera, S. Pagliara, A. Goldoni and L. Sangaletti, *Carbon*, 2014, **80**, 356–363.
316 C. Hua, Y. Shang, Y. Wang, J. Xu, Y. Zhang, X. Li and A. Cao, *Appl. Surf. Sci.*, 2017, **405**, 405–411.
317 A. Arena, N. Donato, G. Saitta, A. Bonavita, G. Rizzo and G. Neri, *Sens. Actuators, B*, 2010, **145**, 488–494.
318 M. Asad, M. H. Sheikhi, M. Pourfath and M. Moradi, *Sens. Actuators, B*, 2015, **210**, 1–8.
319 D. Fu, H. Lim, Y.-F. Shi, X. Dong, S. G. Mhaisalkar, Y. Chen, S. Moochhala and L. J. Li, *J. Phys. Chem. C*, 2008, **112**, 650–653.
320 U. Yaqoob, D.-T. Phan, A. S. M. I. Uddin and G.-S. Chung, *Sens. Actuators, B*, 2015, **221**, 760–768.
321 U. Yaqoob, A. S. M. I. Uddin and G.-S. Chung, *Sens. Actuators, B*, 2016, **224**, 738–746.
322 P. G. Su, C. T. Lee, C. Y. Chou, K. H. Cheng and Y. S. Chuang, *Sens. Actuators, B*, 2009, **139**, 488–493.
323 H. Y. Jeong, D. S. Lee, H. K. Choi, D. H. Lee, J. E. Kim, J. Y. Lee, W. J. Lee, S. O. Kim and S. Y. Choi, *Appl. Phys. Lett.*, 2010, **96**, 213105.
324 S. Ammu, V. Dua, S. R. Agnihotra, S. P. Surwade, A. Phulgirkar, S. Patel and S. K. Manohar, *J. Am. Chem. Soc.*, 2012, **134**, 4553–4556.
325 K. Cattanach, R. D. Kulkarni, M. Kozlov and S. K. Manohar, *Nanotechnology*, 2006, **17**, 4123.
326 Y. Wang, Z. Yang, Z. Hou, D. Xu, L. Wei, E. S. W. Kong and Y. Zhang, *Sens. Actuators, B*, 2010, **150**, 708–714.
327 K. Parikh, K. Cattanach, R. Rao, D. S. Suh, A. Wu and S. K. Manohar, *Sens. Actuators, B*, 2006, **113**, 55–63.
328 C. Tasaltin and F. Basarir, *Sens. Actuators, B*, 2014, **194**, 173–179.
329 P. Wan, X. Wen, C. Sun, B. K. Chandran, H. Zhang, X. Sun and X. Chen, *Small*, 2015, **11**, 5409–5415.
330 K. Lee, V. Scardaci, H.-Y. Kim, T. Hallam, H. Nolan, B. E. Bolf, G. S. Maltbie, J. E. Abbott and G. S. Duesberg, *Sens. Actuators, B*, 2013, **188**, 571–575.
331 M. Pumera, A. Ambrosi, A. Bonanni, E. L. K. Chng and H. L. Poh, *TrAC, Trends Anal. Chem.*, 2010, **29**, 954–965.
332 M. Pumera, *Chem. – Eur. J.*, 2009, **15**, 4970–4978.
333 W. Yuan and G. Shi, *J. Mater. Chem. A*, 2013, **1**, 10078–10091.
334 D. S. L. Abergel, V. Apalkov, J. Berashevich, K. Ziegler and T. Chakraborty, *Adv. Phys.*, 2010, **59**, 261–482.
335 A. K. Geim and K. S. Novoselov, *Nat. Mater.*, 2007, **6**, 183–191.
336 A. H. C. Neto, F. Guinea, N. M. R. Peres, K. S. Novoselov and A. K. Geim, *Rev. Mod. Phys.*, 2009, **81**, 109.
337 S. Agarwal, X. Zhou, F. Ye, Q. He, G. C. K. Chen, J. Soo, F. Boey, H. Zhang and P. Chen, *Langmuir*, 2010, **26**, 2244–2247.
338 Y. Wang, J. Liu, L. Liu and D. D. Sun, *Nanoscale Res. Lett.*, 2011, **6**, 241.
339 Z. Chen, W. Ren, L. Gao, B. Liu, S. S. Pei and H.-M. Cheng, *Nat. Mater.*, 2011, **10**, 424–428.
340 N. G. Shang, P. Papakonstantinou, M. McMullan, M. Chu, A. Stamboulis, A. Potenza, S. S. Dhesi and H. Marchetto, *Adv. Funct. Mater.*, 2008, **18**, 3506–3514.
341 A. Reina, X. Jia, J. C. Ho, D. Nezich, H. C. Son, V. Bulovic, M. S. Dresselhaus and J. Kong, *Nano Lett.*, 2009, **9**, 30–35.
342 X. Li, W. Cai, J. An, S. Kim, J. Nah, D. Yang, R. Piner, A. Velamakanni, I. Jung, E. Tutuc, S. K. Banerjee, L. Colombo and R. S. Ruoff, *Science*, 2009, **324**, 1312–1314.
343 D. V. Kosynkin, A. L. Higginbotham, A. Sinitskii, J. R. Lomeda, A. Dimiev, B. K. Price and J. M. Tour, *Nature*, 2009, **458**, 872.
344 L. Jiao, L. Zhang, X. Wang, G. Diankov and H. Dai, *Nature*, 2009, **458**, 877.
345 P. K. Ang, S. Wang, Q. Bao, J. T. L. Thong and K. P. Loh, *ACS Nano*, 2009, **3**, 3587–3594.
346 J. T. Robinson, F. K. Perkins, E. S. Snow, Z. Wei and P. E. Sheehan, *Nano Lett.*, 2008, **8**, 3137–3140.
347 Q. Yu, J. Lian, S. Siriponglert, H. Li, Y. P. Chen and S. S. Pei, *Appl. Phys. Lett.*, 2008, **93**, 113103.
348 W. S. Hummers and R. E. Offeman, *J. Am. Chem. Soc.*, 1958, **80**, 1339.
349 D. C. Marcano, D. V. Kosynkin, J. M. Berlin, A. Sinitskii, Z. Sun, A. Slesarev, L. B. Alemany, W. Lu and J. M. Tour, *ACS Nano*, 2010, **4**, 4806–4814.
350 D. Li, M. B. Muller, S. Gilje, R. B. Kaner and G. G. Wallace, *Nat. Nanotechnol.*, 2008, **3**, 101–105.
351 C. D. Zangmeister, *Chem. Mater.*, 2010, **22**, 5625–5629.
352 S. Stankovich, D. A. Dikin, R. D. Piner, K. A. Kohlhaas, A. Kleinhammes, Y. Jia, Y. Wu, S. B. T. Nguyen and R. S. Ruoff, *Carbon*, 2007, **45**, 1558–1565.
353 Y. Shao, J. Wang, M. Engelhard, C. Y. Wang and Y. Lin, *J. Mater. Chem.*, 2010, **20**, 743–748.

 This journal is © The Royal Society of Chemistry 2018

Published on 11 June 2018. Downloaded on 2/1/2025 6:21:23 AM.

354 L. J. Cote, R. Cruz-Silva and J. Huang, *J. Am. Chem. Soc.*, 2009, **131**, 11027–11032.
355 F. Schedin, A. K. Geim, S. V. Morozov, E. Hill, P. Blake, M. I. Katsnelson and K. S. Novoselov, *Nat. Mater.*, 2007, **6**, 652–655.
356 R. Pearce, T. Iakimov, M. Andersson, L. Hultman, A. L. Spetz and R. Yakimova, *Sens. Actuators, B*, 2011, **155**, 451–455.
357 K. Yu, P. Wang, G. Lu, K.-H. Chen, Z. Bo and J. Chen, *J. Phys. Chem. Lett.*, 2011, **2**, 537–542.
358 G. Lu, L. E. Ocola and J. Chen, *Nanotechnology*, 2009, **20**, 445502.
359 Z. Ben Aziza, Q. Zhang and D. Baillargeat, *Appl. Phys. Lett.*, 2014, **105**, 254102.
360 A. Inaba, K. Yoo, Y. Takei, K. Matsumoto and I. Shimoyama, *Sens. Actuators, B*, 2014, **195**, 15–21.
361 M. Gautam and A. H. Jayatissa, *J. Appl. Phys.*, 2012, **112**, 064304.
362 J. Zhang, P. Hu, R. Zhang, X. Wang, B. Yang, W. Cao, Y. Li, X. He, Z. Wang and W. O'Neill, *J. Mater. Chem.*, 2012, **22**, 714–718.
363 G. Lu, K. Yu, L. E. Ocola and J. Chen, *Chem. Commun.*, 2011, **47**, 7761–7763.
364 S. M. Mortazavi Zanjani, M. M. Sadeghi, M. Holt, S. k. F. Chowdhury, L. Tao and D. Akinwande, *Appl. Phys. Lett.*, 2016, **108**, 033106.
365 B. Chen, H. Liu, X. Li, C. Lu, Y. Ding and B. Lu, *Appl. Surf. Sci.*, 2012, **258**, 1971–1975.
366 S. Mao, S. Cui, G. Lu, K. Yu, Z. Wen and J. Chen, *J. Mater. Chem.*, 2012, **22**, 11009–11013.
367 T. V. Cuong, V. H. Pham, J. S. Chung, E. W. Shin, D. H. Yoo, S. H. Hahn, J. S. Huh, G. H. Rue, E. J. Kim and S. H. Hur, *Mater. Lett.*, 2010, **64**, 2479–2482.
368 W. Li, X. Geng, Y. Guo, J. Rong, Y. Gong, L. Wu, X. Zhang, P. Li, J. Xu and G. Cheng, *ACS Nano*, 2011, **5**, 6955–6961.
369 Y. Lu, B. R. Goldsmith, N. J. Kybert and A. T. C. Johnson, *Appl. Phys. Lett.*, 2010, **97**, 083107.
370 S. Cui, H. Pu, E. C. Mattson, Z. Wen, J. Chang, Y. Hou, C. J. Hirschmugl and J. Chen, *Anal. Chem.*, 2014, **86**, 7516–7522.
371 G. Yang, C. Lee, J. E. Kim, F. Ren and S. J. Pearton, *Phys. Chem. Chem. Phys.*, 2013, **15**, 1798–1801.
372 T. Hayasaka, Y. Kubota, Y. Liu and L. Lin, *19th International Conference on Solid-State Sensors, Actuators and Microsystems (TRANSDUCERS)*, Kaohsiung, 2017, pp. 2103–2106.
373 S. Tiwari, A. K. Singh, S. K. Balasubramanian, W. Takashima and R. Prakash, *J. Nanosci. Nanotechnol.*, 2016, **16**, 9634–9641.
374 D. Zhang, J. Liu, C. Jiang, A. Liu and B. Xia, *Sens. Actuators, B*, 2017, **240**, 55–65.
375 J. D. Fowler, M. J. Allen, V. C. Tung, Y. Yang, R. B. Kaner and B. H. Weiller, *ACS Nano*, 2009, **3**, 301–306.
376 T. H. Han, Y.-K. Huang, A. T. L. Tan, V. P. Dravid and J. Huang, *J. Am. Chem. Soc.*, 2011, **133**, 15264–15267.
377 S. J. Hwang, J. Lim, H. G. Park, W. K. Kim, D.-H. Kim, I. S. Song, J. H. Kim, S. Lee, D. H. Woo and S. C. Jun, *Curr. Appl. Phys.*, 2012, **12**, 1017–1022.
378 R. K. Paul, S. Badhulika, N. M. Saucedo and A. Mulchandani, *Anal. Chem.*, 2012, **84**, 8171–8178.
379 M. W. K. Nomani, R. Shishir, M. Qazi, D. Diwan, V. B. Shields, M. G. Spencer, G. S. Tompa, N. M. Sbrockey and G. Koley, *Sens. Actuators, B*, 2010, **150**, 301–307.
380 M. G. Chung, D. H. Kim, H. M. Lee, T. Kim, J. H. Choi, D. k. Seo, J.-B. Yoo, S.-H. Hong, T. J. Kang and Y. H. Kim, *Sens. Actuators, B*, 2012, **166**, 172–176.
381 G. Chen, T. M. Paronyan and A. R. Harutyunyan, *Appl. Phys. Lett.*, 2012, **101**, 053119.
382 F. Yavari, Z. Chen, A. V. Thomas, W. Ren, H.-M. Cheng and N. Koratkar, *Sci. Rep.*, 2011, **1**, 166.
383 G. Ko, H.-Y. Kim, J. Ahn, Y.-M. Park, K.-Y. Lee and J. Kim, *Curr. Appl. Phys.*, 2010, **10**, 1002–1004.
384 A. Salehi-Khojin, D. Estrada, K. Y. Lin, M. H. Bae, F. Xiong, E. Pop and R. I. Masel, *Adv. Mater.*, 2012, **24**, 53–57.
385 S. Srivastava, K. Jain, V. N. Singh, S. Singh, N. Vijayan, N. Dilawar, G. Gupta and T. D. Senguttuvan, *Nanotechnology*, 2012, **23**, 205501.
386 L. T. Hoa, H. N. Tien, V. H. Luan, J. S. Chung and S. H. Hur, *Sens. Actuators, B*, 2013, **185**, 701–705.
387 S. Liu, B. Yu, H. Zhang, T. Fei and T. Zhang, *Sens. Actuators, B*, 2014, **202**, 272–278.
388 X. Liu, J. Cui, J. Sun and X. Zhang, *RSC Adv.*, 2014, **4**, 22601–22605.
389 F. Gu, R. Nie, D. Han and Z. Wang, *Sens. Actuators, B*, 2015, **219**, 94–99.
390 H. Zhang, J. Feng, T. Fei, S. Liu and T. Zhang, *Sens. Actuators, B*, 2014, **190**, 472–478.
391 P.-G. Su and S.-L. Peng, *Talanta*, 2015, **132**, 398–405.
392 Y.-l. Dong, X.-f. Zhang, X.-l. Cheng, Y.-m. Xu, S. Gao, H. Zhao and L.-h. Huo, *RSC Adv.*, 2014, **4**, 57493–57500.
393 S. Cui, Z. Wen, E. C. Mattson, S. Mao, J. Chang, M. Weinert, C. J. Hirschmugl, M. Gajdardziska-Josifovska and J. Chen, *J. Mater. Chem. A*, 2013, **1**, 4462–4467.
394 H. Meng, W. Yang, K. Ding, L. Feng and Y. Guan, *J. Mater. Chem. A*, 2015, **3**, 1174–1181.
395 S.-J. Choi, B.-H. Jang, S.-J. Lee, B. K. Min, A. Rothschild and I.-D. Kim, *ACS Appl. Mater. Interfaces*, 2014, **6**, 2588–2597.
396 S.-J. Choi, W.-H. Ryu, S.-J. Kim, H.-J. Cho and I.-D. Kim, *J. Mater. Chem. B*, 2014, **2**, 7160–7167.
397 Z. Zhang, R. Zou, G. Song, L. Yu, Z. Chen and J. Hu, *J. Mater. Chem.*, 2011, **21**, 17360–17365.
398 A. S. M. I. Uddin, K.-W. Lee and G.-S. Chung, *Sens. Actuators, B*, 2015, **216**, 33–40.
399 A. S. M. I. Uddin, D.-T. Phan and G.-S. Chung, *Sens. Actuators, B*, 2015, **207**, 362–369.
400 S. Cui, S. Mao, Z. Wen, J. Chang, Y. Zhang and J. Chen, *Analyst*, 2013, **138**, 2877–2882.
401 M. Gautam and A. H. Jayatissa, *Solid-State Electron.*, 2012, **78**, 159–165.
402 Q. T. Tran, H. T. M. Hoa, D.-H. Yoo, T. V. Cuong, S. H. Hur, J. S. Chung, E. J. Kim and P. A. Kohl, *Sens. Actuators, B*, 2014, **194**, 45–50.
403 C. Piloto, M. Notarianni, M. Shafiei, E. Taran, D. Galpaya, C. Yan and N. Motta, *Beilstein J. Nanotechnol.*, 2014, **5**, 1073.

Published on 11 June 2018. Downloaded on 2/1/2025 6:21:23 AM.

404 T. T. Tung, M. Castro and J.-F. Feller, *12th IEEE International Conference on Nanotechnology (IEEE-NANO)*, Birmingham, 2012, pp. 1–5.
405 M. Gautam and A. H. Jayatissa, *J. Appl. Phys.*, 2012, **112**, 114326.
406 L. Zhang, C. Li, A. Liu and G. Shi, *J. Mater. Chem.*, 2012, **22**, 8438–8443.
407 X. Huang, N. Hu, L. Zhang, L. Wei, H. Wei and Y. Zhang, *Synth. Met.*, 2013, **185**, 25–30.
408 X. Huang, N. Hu, R. Gao, Y. Yu, Y. Wang, Z. Yang, E. S.-W. Kong, H. Wei and Y. Zhang, *J. Mater. Chem.*, 2012, **22**, 22488–22495.
409 Z. Wu, X. Chen, S. Zhu, Z. Zhou, Y. Yao, W. Quan and B. Liu, *Sens. Actuators, B*, 2013, **178**, 485–493.
410 Z. Ye, Y. Jiang, H. Tai and Z. Yuan, *Integr. Ferroelectr.*, 2014, **154**, 73–81.
411 X. L. Huang, N. T. Hu, Y. Y. Wang and Y. F. Zhang, *Adv. Mat. Res.*, 2013, **669**, 79–84.
412 N. Hu, Z. Yang, Y. Wang, L. Zhang, Y. Wang, X. Huang, H. Wei, L. Wei and Y. Zhang, *Nanotechnology*, 2014, **25**, 025502.
413 Y. Wang, L. Zhang, N. Hu, Y. Wang, Y. Zhang, Z. Zhou, Y. Liu, S. Shen and C. Peng, *Nanoscale Res. Lett.*, 2014, **9**, 251.
414 W. Yuan, A. Liu, L. Huang, C. Li and G. Shi, *Adv. Mater.*, 2013, **25**, 766–771.
415 Y. Seekaew, D. Phokharatkul, A. Wisitsoraat and C. Wongchoosuk, *Appl. Surf. Sci.*, 2017, **404**, 357–363.
416 Z. Song, Z. Wei, B. Wang, Z. Luo, S. Xu, W. Zhang, H. Yu, M. Li, Z. Huang and J. Zang, *Chem. Mater.*, 2016, **28**, 1205–1212.
417 H. K. Choi, H. Y. Jeong, D.-S. Lee, S.-Y. Choi and C.-G. Choi, *Carbon Lett.*, 2013, **14**, 186–189.
418 M. W. Jung, S. Myung, W. Song, M.-A. Kang, S. H. Kim, C.-S. Yang, S. S. Lee, J. Lim, C.-Y. Park and J.-O. Lee, *ACS Appl. Mater. Interfaces*, 2014, **6**, 13319–13323.
419 H. Choi, J. S. Choi, J. S. Kim, J. H. Choe, K. H. Chung, J. W. Shin, J. T. Kim, D. H. Youn, K. C. Kim and J. I. Lee, *Small*, 2014, **10**, 3685–3691.
420 V. Dua, S. P. Surwade, S. Ammu, S. R. Agnihotra, S. Jain, K. E. Roberts, S. H. Park, R. S. Ruoff and S. K. Manohar, *Angew. Chem., Int. Ed.*, 2010, **49**, 2154–2157.
421 C. Lee, J. H. Ahn, K. B. Lee, D. S. Kim and J. E. Kim, *Thin Solid Films*, 2012, **520**, 5459–5462.
422 B. Cho, J. Yoon, M. G. Hahm, D.-H. Kim, A. R. Kim, Y. H. Kahng, S.-W. Park, Y.-J. Lee, S.-G. Park and J.-D. Kwon, *J. Mater. Chem. C*, 2014, **2**, 5280–5285.
423 L. Huang, Z. L. Wang, J. Zhang, J. Pu, Y. Lin, S. Xu, L. Shen, Q. Chen and W. Shi, *ACS Appl. Mater. Interfaces*, 2014, **6**, 7426–7433.
424 Y. Seekaew, S. Lokavee, D. Phokharatkul, A. Wisitsoraat, T. Kerdcharoen and C. Wongchoosuk, *Org. Electron.*, 2014, **15**, 2971–2981.
425 M.-S. Kim, S. Kim, H. J. Kong, O. S. Kwon and H. Yoon, *J. Phys. Chem. C*, 2016, **120**, 18289–18295.
426 X. Peng, J. Chu, A. Aldalbahi, M. Rivera, L. Wang, S. Duan and P. Feng, *Appl. Surf. Sci.*, 2016, **387**, 149–154.
427 Y. Cui, Z. Zhong, D. Wang, W. U. Wang and C. M. Lieber, *Nano Lett.*, 2003, **3**, 149–152.
428 D. Braga and G. Horowitz, *Adv. Mater.*, 2009, **21**, 1473–1486.
429 Y. Cui, X. Duan, J. Hu and C. M. Lieber, *J. Phys. Chem. B*, 2000, **104**, 5213–5216.
430 R. M. Penner, *Annu. Rev. Anal. Chem.*, 2012, **5**, 461–485.
431 M. Y. Bashouti, K. Sardashti, S. W. Schmitt, M. Pietsch, J. Ristein, H. Haick and S. H. Christiansen, *Prog. Surf. Sci.*, 2013, **88**, 39–60.
432 M. Mescher, L. C. P. M. De Smet, E. J. R. Sudhölter and J. H. Klootwijk, *J. Nanosci. Nanotechnol.*, 2013, **13**, 5649–5653.
433 E. Stern, J. F. Klemic, D. A. Routenberg, P. N. Wyrembak, D. B. Turner-Evans, A. D. Hamilton, D. A. LaVan, T. M. Fahmy and M. A. Reed, *Nature*, 2007, **445**, 519–522.
434 X. Liu, P. R. Coxon, M. Peters, B. Hoex, J. M. Cole and D. J. Fray, *Energy Environ. Sci.*, 2014, **7**, 3223–3263.
435 R. Dussart, T. Tillocher, P. Lefaucheux and M. Boufnichel, *J. Phys. D: Appl. Phys.*, 2014, **47**, 123001.
436 R. Ghosh and P. K. Giri, *Nanotechnology*, 2016, **28**, 012001.
437 A. Cao, E. Sudhölter and L. de Smet, *Sensors*, 2014, **14**, 245.
438 T. Yoshizumi and Y. Miyahara, in *Different Types of Field-Effect Transistors – Theory and Applications*, ed. M. M. Pejovic and M. M. Pejovic, InTech, Rijeka, 2017, ch. 08.
439 X. Chen, C. K. Y. Wong, C. A. Yuan and G. Zhang, *Sens. Actuators, B*, 2013, **177**, 178–195.
440 S. Clavaguera, A. Carella, L. Caillier, C. Celle, J. Pécaut, S. Lenfant, D. Vuillaume and J.-P. Simonato, *Angew. Chem., Int. Ed.*, 2010, **49**, 4063–4066.
441 V. Passi, F. Ravaux, E. Dubois, S. Clavaguera, A. Carella, C. Celle, J.-P. Simonato, L. Silvestri, S. Reggiani and D. Vuillaume, *IEEE Electron Device Lett.*, 2011, **32**, 976–978.
442 S. Clavaguera, N. Raoul, A. Carella, M. Delalande, C. Celle and J.-P. Simonato, *Talanta*, 2011, **85**, 2542–2545.
443 J. W. Han, T. Rim, C.-K. Baek and M. Meyyappan, *ACS Appl. Mater. Interfaces*, 2015, **7**, 21263–21269.
444 A. O. Niskanen, A. Colli, R. White, H. W. Li, E. Spigone and J. M. Kivioja, *Nanotechnology*, 2011, **22**, 295502.
445 Y. Paska, T. Stelzner, S. Christiansen and H. Haick, *ACS Nano*, 2011, **5**, 5620–5626.
446 Y. Paska, T. Stelzner, O. Assad, U. Tisch, S. Christiansen and H. Haick, *ACS Nano*, 2011, **6**, 335–345.
447 Y. Paska and H. Haick, *ACS Appl. Mater. Interfaces*, 2012, **4**, 2604–2617.
448 B. Wang and H. Haick, *ACS Appl. Mater. Interfaces*, 2013, **5**, 5748–5756.
449 B. Wang and H. Haick, *ACS Appl. Mater. Interfaces*, 2013, **5**, 2289–2299.
450 B. Wang, J. C. Cancilla, J. S. Torrecilla and H. Haick, *Nano Lett.*, 2014, **14**, 933–938.
451 R. Ermanok, O. Assad, K. Zigelboim, B. Wang and H. Haick, *ACS Appl. Mater. Interfaces*, 2013, **5**, 11172–11183.
452 N. Shehada, G. Bronstrup, K. Funka, S. Christiansen, M. Leja and H. Haick, *Nano Lett.*, 2015, **15**, 1288–1295.
453 N. Shehada, J. C. Cancilla, J. S. Torrecilla, E. S. Pariente, G. Bronstrup, S. Christiansen, D. W. Johnson, M. Leja,

This journal is © The Royal Society of Chemistry 2018

View Article Online

Published on 11 June 2018. Downloaded on 2/1/2025 6:21:23 AM.

M. P. Davies, O. Liran, N. Peled and H. Haick, *ACS Nano*, 2016, **10**, 7047–7057.
454 H. T. Hsueh, T. J. Hsueh, S. J. Chang, F. Y. Hung, W. Y. Weng, C. L. Hsu and B. T. Dai, *IEEE Sens. J.*, 2011, **11**, 3036–3041.
455 L. Ni, E. Jacques, R. Rogel, A.-C. Salaün, L. Pichon and G. Wenga, *Procedia Eng.*, 2012, **47**, 240–243.
456 L. Yang, H. Lin, Z. Zhang, L. Cheng, S. Ye and M. Shao, *Sens. Actuators, B*, 2013, **177**, 260–264.
457 J. Wan, S.-R. Deng, R. Yang, Z. Shu, B.-R. Lu, S.-Q. Xie, Y. Chen, E. Huq, R. Liu and X.-P. Qu, *Microelectron. Eng.*, 2009, **86**, 1238–1242.
458 C. Gao, S.-R. Deng, J. Wan, B.-R. Lu, R. Liu, E. Huq, X.-P. Qu and Y. Chen, *Microelectron. Eng.*, 2010, **87**, 927–930.
459 M. C. McAlpine, H. D. Agnew, R. D. Rohde, M. Blanco, H. Ahmad, A. D. Stuparu, W. A. Goddard Iii and J. R. Heath, *J. Am. Chem. Soc.*, 2008, **130**, 9583–9589.
460 C. Gao, Z.-C. Xu, S.-R. Deng, J. Wan, Y. Chen, R. Liu, E. Huq and X.-P. Qu, *Microelectron. Eng.*, 2011, **88**, 2100–2104.
461 M. Cuscunà, A. Convertino, E. Zampetti, A. Macagnano, A. Pecora, G. Fortunato, L. Felisari, G. Nicotra, C. Spinella and F. Martelli, *Appl. Phys. Lett.*, 2012, **101**, 103101.
462 H. J. In, C. R. Field and P. E. Pehrsson, *Nanotechnology*, 2011, **22**, 355501.
463 J. Liao, Z. Li, G. Wang, C. Chen, S. Lv and M. Li, *Phys. Chem. Chem. Phys.*, 2016, **18**, 4835–4841.
464 W. Zhang, M. Hu, X. Liu, Y. Wei, N. Li and Y. Qin, *J. Alloys Compd.*, 2016, **679**, 391–399.
465 S. Ma, M. Hu, P. Zeng, M. Li, W. Yan and Y. Qin, *Sens. Actuators, B*, 2014, **192**, 341–349.
466 K.-Q. Peng, X. Wang and S.-T. Lee, *Appl. Phys. Lett.*, 2009, **95**, 243112.
467 M. C. McAlpine, H. Ahmad, D. Wang and J. R. Heath, *Nat. Mater.*, 2007, **6**, 379.
468 W. P. Jakubik, *Thin Solid Films*, 2011, **520**, 986–993.
469 M. C. Horrillo, M. J. Fernández, J. L. Fontecha, I. Sayago, M. Garcıa, M. Aleixandre, J. P. Santos, L. Arés, J. Gutiérrez and I. Gracia, *Thin Solid Films*, 2004, **467**, 234–238.
470 Y.-L. Tang, Z.-J. Li, J.-Y. Ma, H.-Q. Su, Y.-J. Guo, L. Wang, B. Du, J.-J. Chen, W. Zhou and Q.-K. Yu, *J. Hazard. Mater.*, 2014, **280**, 127–133.
471 Y.-L. Tang, Z.-J. Li, J.-Y. Ma, Y.-J. Guo, Y.-Q. Fu and X.-T. Zu, *Sens. Actuators, B*, 2014, **201**, 114–121.
472 Y. Tang, D. Ao, W. Li, X. Zu, S. Li and Y. Q. Fu, *Sens. Actuators, B*, 2018, **254**, 1165–1173.
473 L. Rana, R. Gupta, R. Kshetrimayum, M. Tomar and V. Gupta, *Surf. Coat. Technol.*, 2018, **343**, 89–92.
474 L. Rana, R. Gupta, M. Tomar and V. Gupta, *Sens. Actuators, B*, 2017, **252**, 840–845.
475 S. J. Ippolito, A. Ponzoni, K. Kalantar-Zadeh, W. Wlodarski, E. Comini, G. Faglia and G. Sberveglieri, *Sens. Actuators, B*, 2006, **117**, 442–450.
476 D. Matatagui, O. Kolokoltsev, J. M. Saniger, I. Gràcia, M. J. Fernández, J. L. Fontecha and M. d. C. Horrillo, *Sensors*, 2017, **17**, 2624.
477 I. Sayago, D. Matatagui, M. J. Fernández, J. L. Fontecha, I. Jurewicz, R. Garriga and E. Muñoz, *Talanta*, 2016, **148**, 393–400.
478 S. Thomas, M. Cole, A. De Luca, F. Torrisi, A. C. Ferrari, F. Udrea and J. W. Gardner, *Procedia Eng.*, 2014, **87**, 999–1002.
479 R. Arsat, M. Breedon, M. Shafiei, P. G. Spizziri, S. Gilje, R. B. Kaner, K. Kalantar-zadeh and W. Wlodarski, *Chem. Phys. Lett.*, 2009, **467**, 344–347.
480 D. Amati, D. Arn, N. Blom, M. Ehrat, J. Saunois and H. M. Widmer, *Sens. Actuators, B*, 1992, **7**, 587–591.
481 U. Stahl, A. Voigt, M. Dirschka, N. Barié, C. Richter, A. Waldbaur, F. J. Gruhl, B. E. Rapp, M. Rapp and K. Länge, *Sensors*, 2017, **17**, 2529.
482 Y. Liu, H. Huang, L. Wang, D. Cai, B. Liu, D. Wang, Q. Li and T. Wang, *Sens. Actuators, B*, 2016, **223**, 730–737.
483 L. Al-Mashat, H. D. Tran, W. Wlodarski, R. B. Kaner and K. Kalantar-Zadeh, *Sens. Actuators, B*, 2008, **134**, 826–831.
484 A. Z. Sadek, W. Wlodarski, K. Shin, R. B. Kaner and K. Kalantar-Zadeh, *Nanotechnology*, 2006, **17**, 4488.
485 M. David, M. Arab, C. Martino, L. Delmas, F. Guinneton and J.-R. Gavarri, *Thin Solid Films*, 2012, **520**, 4786–4791.
486 M. Penza, F. Antolini and M. V. Antisari, *Sens. Actuators, B*, 2004, **100**, 47–59.
487 M. Penza, F. Antolini and M. Vittori-Antisari, *Thin Solid Films*, 2005, **472**, 246–252.
488 I. Sayago, M. J. Fernández, J. L. Fontecha, M. C. Horrillo, C. Vera, I. Obieta and I. Bustero, *Sens. Actuators, B*, 2012, **175**, 67–72.
489 M. N. Hamidon and Z. Yunusa, *Progresses in Chemical Sensor*, ed. W. Wang, InTech, Rijeka, 2016, ch. 8.
490 Y. J. Guo, J. Zhang, C. Zhao, P. A. Hu, X.-T. Zu and Y. Q. Fu, *Optik*, 2014, **125**, 5800–5802.
491 S. K. Vashist and P. Vashist, *Sensors*, 2011, 1–13.
492 F. L. Dickert, O. Hayden and M. E. Zenkel, *Anal. Chem.*, 1999, **71**, 1338–1341.
493 X. Wang, J. Zhang and Z. Zhu, *Appl. Surf. Sci.*, 2006, **252**, 2404–2411.
494 N. Van Quy, V. A. Minh, N. Van Luan, V. N. Hung and N. Van Hieu, *Sens. Actuators, B*, 2011, **153**, 188–193.
495 J. Zhang, J. Q. Hu, F. R. Zhu, H. Gong and S. J. O'shea, *Sens. Actuators, B*, 2002, **87**, 159–167.
496 J. Hu, F. Zhu, J. Zhang and H. Gong, *Sens. Actuators, B*, 2003, **93**, 175–180.
497 J. Zhang, J. Hu, Z. Q. Zhu, H. Gong and S. J. O'Shea, *Colloids Surf., A*, 2004, **236**, 23–30.
498 B. Ding, J. Kim, Y. Miyazaki and S. Shiratori, *Sens. Actuators, B*, 2004, **101**, 373–380.
499 A. l. Palaniappan, S. Moochhala, F. E. H. Tay, X. Su and N. C. L. Phua, *Sens. Actuators, B*, 2008, **129**, 184–187.
500 M. Matsuguchi, Y. Kadowaki and M. Tanaka, *Sens. Actuators, B*, 2005, **108**, 572–575.
501 Z. Ying, Y. Jiang, X. Du, G. Xie, J. Yu and H. Wang, *Sens. Actuators, B*, 2007, **125**, 167–172.
502 Z. Ying, Y. Jiang, X. Du, G. Xie, J. Yu and H. Tai, *Eur. Polym. J.*, 2008, **44**, 1157–1164.

Published on 11 June 2018. Downloaded on 2/1/2025 6:21:23 AM.

503 C. Zhang, X. Wang, J. Lin, B. Ding, J. Yu and N. Pan, *Sens. Actuators, B*, 2011, **152**, 316–323.
504 X. Wang, B. Ding, M. Sun, J. Yu and G. Sun, *Sens. Actuators, B*, 2010, **144**, 11–17.
505 M. M. Ayad, G. El-Hefnawey and N. L. Torad, *J. Hazard. Mater.*, 2009, **168**, 85–88.
506 P. Si, J. Mortensen, A. Komolov, J. Denborg and P. J. Møller, *Anal. Chim. Acta*, 2007, **597**, 223–230.
507 I. A. Koshets, Z. I. Kazantseva, Y. M. Shirshov, S. A. Cherenok and V. I. Kalchenko, *Sens. Actuators, B*, 2005, **106**, 177–181.
508 B. Wyszynski, P. Somboon and T. Nakamoto, *Sens. Actuators, B*, 2008, **130**, 857–863.
509 A. K. M. S. Islam, Z. Ismail, M. N. Ahmad, B. Saad, A. R. Othman, A. Y. M. Shakaff, A. Daud and Z. Ishak, *Sens. Actuators, B*, 2005, **109**, 238–243.
510 A. Özmen, F. Tekce, M. A. Ebeoğlu, C. Taşaltın and Z. Z. Öztürk, *Sens. Actuators, B*, 2006, **115**, 450–454.
511 M. Matsuguchi and T. Uno, *Sens. Actuators, B*, 2006, **113**, 94–99.
512 Y. Zhu, H. Yuan, J. Xu, P. Xu and Q. Pan, *Sens. Actuators, B*, 2010, **144**, 164–169.
513 X. Wang, B. Ding, J. Yu, M. Wang and F. Pan, *Nanotechnology*, 2009, **21**, 055502.
514 S.-W. Lee, N. Takahara, S. Korposh, D.-H. Yang, K. Toko and T. Kunitake, *Anal. Chem.*, 2010, **82**, 2228–2236.
515 J. L. Huang, G. Xie, Y. Zhou, T. Xie, H. Tai and G. J. Yang, *7th International Symposium on Advanced Optical Manufacturing and Testing Technologies: Smart Structures and Materials for Manufacturing and Testing*, 2014, vol. 9285, pp. 92850B.
516 Y. Yao, X. Chen, H. Guo and Z. Wu, *Appl. Surf. Sci.*, 2011, **257**, 7778–7782.
517 W. Tao, P. Lin, S. Liu, Q. Xie, S. Ke and X. Zeng, *Sensors*, 2017, **17**, 194.
518 F. Pascal-Delannoy, B. Sorli and A. Boyer, *Sens. Actuators, A*, 2000, **84**, 285–291.
519 V. S. A. Piriya, P. Joseph, S. C. G. K. Daniel, S. Lakshmanan, T. Kinoshita and M. Sivakumar, *Mater. Sci. Eng., C*, 2017, **78**, 1231–1245.
520 J. R. Askim, M. Mahmoudi and K. S. Suslick, *Chem. Soc. Rev.*, 2013, **42**, 8649–8682.
521 M. J. Kangas, R. M. Burks, J. Atwater, R. M. Lukowicz, P. Williams and A. E. Holmes, *Crit. Rev. Anal. Chem.*, 2017, **47**, 138–153.
522 K. S. Suslick, N. A. Rakow and A. Sen, *Tetrahedron*, 2004, **60**, 11133–11138.
523 M. C. Janzen, J. B. Ponder, D. P. Bailey, C. K. Ingison and K. S. Suslick, *Anal. Chem.*, 2006, **78**, 3591–3600.
524 C. Zhang and K. S. Suslick, *J. Am. Chem. Soc.*, 2005, **127**, 11548–11549.
525 N. A. Rakow and K. S. Suslick, *Nature*, 2000, **406**, 710–713.
526 K. S. Suslick, *MRS Bull.*, 2004, **29**, 720–725.
527 L. Feng, C. J. Musto, J. W. Kemling, S. H. Lim and K. S. Suslick, *Chem. Commun.*, 2010, **46**, 2037–2039.
528 S. H. Lim, L. Feng, J. W. Kemling, C. J. Musto and K. S. Suslick, *Nat. Chem.*, 2009, **1**, 562–567.
529 A. Sen, J. D. Albarella, J. R. Carey, P. Kim and W. B. McNamara, *Sens. Actuators, B*, 2008, **134**, 234–237.
530 J. Courbat, D. Briand, J. Wöllenstein and N. F. D. Rooij, *Procedia Chem.*, 2009, **1**, 576–579.
531 T. Endo, Y. Yanagida and T. Hatsuzawa, *Sens. Actuators, B*, 2007, **125**, 589–595.
532 X. Wang, X. Sun, P. A. Hu, J. Zhang, L. Wang, W. Feng, S. Lei, B. Yang and W. Cao, *Adv. Funct. Mater.*, 2013, **23**, 6044–6050.
533 N. A. Luechinger, S. Loher, E. K. Athanassiou, R. N. Grass and W. J. Stark, *Langmuir*, 2007, **23**, 3473–3477.
534 C. Elosua, I. R. Matias, C. Bariain and F. J. Arregui, *Sensors*, 2006, **6**, 1440–1465.
535 M. Hernaez, C. Zamarreño, S. Melendi-Espina, L. Bird, A. Mayes and F. Arregui, *Sensors*, 2017, **17**, 155.
536 H. Okuda, T. Wang and S. W. Lee, *Electron. Comm. JPN*, 2017, **100**, 43–49.
537 L. I. B. Silva, A. C. Freitas, T. A. P. Rocha-Santos, M. E. Pereira and A. C. Duarte, *Talanta*, 2011, **83**, 1586–1594.
538 L. I. B. Silva, T. A. P. Rocha-Santos and A. C. Duarte, *Talanta*, 2009, **78**, 548–552.
539 C. Bariáin, I. R. Matías, I. Romeo, J. Garrido and M. Laguna, *Appl. Phys. Lett.*, 2000, **77**, 2274–2276.
540 C. Elosúa, C. Bariáin, I. R. Matías, F. J. Arregui, A. Luquin and M. Laguna, *Sens. Actuators, B*, 2006, **115**, 444–449.
541 C. Bariain, I. R. Matıas, C. Fernandez-Valdivielso, F. J. Arregui, M. L. Rodrıguez-Méndez and J. A. De Saja, *Sens. Actuators, B*, 2003, **93**, 153–158.
542 S. M. Topliss, S. W. James, F. Davis, S. P. J. Higson and R. P. Tatam, *Sens. Actuators, B*, 2010, **143**, 629–634.
543 S. Bagchi, R. Achla and S. K. Mondal, *Sens. Actuators, B*, 2017, **250**, 52–60.
544 T. Kavinkumar, D. Sastikumar and S. Manivannan, *Proc. SPIE*, 2014, **9270**, DOI: 10.1117/12.2071841.
545 C. Yu, Y. Wu, X. Liu, F. Fu, Y. Gong, Y.-J. Rao and Y. Chen, *Sens. Actuators, B*, 2017, **244**, 107–113.
546 T. Kavinkumar and S. Manivannan, *Ceram. Int.*, 2016, **42**, 1769–1776.
547 R. Gao, D.-F. Lu, J. Cheng, Y. Jiang, L. Jiang and Z.-M. Qi, *Sens. Actuators, B*, 2016, **222**, 618–624.
548 M. Manjula, B. Karthikeyan and D. Sastikumar, *Opt. Lasers Eng.*, 2017, **95**, 78–82.
549 W. S. Wong and A. Salleo, *Flexible electronics: materials and applications*, Springer Science & Business Media, 2009.
550 C. Bariain, I. R. Matıas, I. Romeo, J. Garrido and M. Laguna, *Appl. Phys. Lett.*, 2000, **77**, 2274–2276.
551 X. Chen, F. Xu, Y. Wang, Y. Pan, D. Lu, P. Wang, K. Ying, E. Chen and W. Zhang, *Cancer*, 2007, **110**, 835–844.
552 W. Filipiak, A. Sponring, A. Filipiak, C. Ager, J. Schubert, W. Miekisch, A. Amann and J. Troppmair, *Cancer Epidemiol., Biomarkers Prev.*, 2010, **19**, 182–195.
553 W. Filipiak, A. Sponring, T. Mikoviny, C. Ager, J. Schubert, W. Miekisch, A. Amann and J. Troppmair, *Cancer Cell Int.*, 2008, **8**, 17.
554 D. Smith, T. Wang, J. Sulé-Suso, P. Španěl and A. E. Haj, *Rapid Commun. Mass Spectrom.*, 2003, **17**, 845–850.

This journal is © The Royal Society of Chemistry 2018

Published on 11 June 2018. Downloaded on 2/1/2025 6:21:23 AM.

555 A. Sponring, W. Filipiak, C. Ager, J. Schubert, W. Miekisch, A. Amann and J. Troppmair, *Cancer Biomarkers*, 2010, **7**, 153–161.
556 A. Sponring, W. Filipiak, T. Mikoviny, C. Ager, J. Schubert, W. Miekisch, A. Amann and J. Troppmair, *Anticancer Res.*, 2009, **29**, 419–426.
557 J. Sulé-Suso, A. Pysanenko, P. Španěl and D. Smith, *Analyst*, 2009, **134**, 2419–2425.
558 O. Barash, N. Peled, F. R. Hirsch and H. Haick, *Small*, 2009, **5**, 2618–2624.
559 O. Barash, N. Peled, U. Tisch, P. A. Bunn Jr., F. R. Hirsch and H. Haick, *Nanomedicine*, 2012, **8**, 580–589.
560 N. Peled, O. Barash, U. Tisch, R. Ionescu, Y. Y. Broza, M. Ilouze, J. Mattei, P. A. Bunn, Jr., F. R. Hirsch and H. Haick, *Nanomedicine*, 2013, **9**, 758–766.
561 M. Davies, O. Barash, R. Jeries, N. Peled, M. Ilouze, R. Hyde, M. Marcus, J. Field and H. Haick, *Br. J. Cancer*, 2014, **111**, 1213.
562 K. B. Gendron, N. G. Hockstein, E. R. Thaler, A. Vachani and C. W. Hanson, *Otolaryngol.–Head Neck Surg.*, 2007, **137**, 269–273.
563 L. Lavra, A. Catini, A. Ulivieri, R. Capuano, L. B. Salehi, S. Sciacchitano, A. Bartolazzi, S. Nardis, R. Paolesse, E. Martinelli and C. D. Natale, *Sci. Rep.*, 2015, **5**, 13246.
564 A. Roine, M. Tolvanen, M. Sipilainen, P. Kumpulainen, M. A. Helenius, T. Lehtimaki, J. Vepsalainen, T. A. Keinanen, M. R. Hakkinen, J. Koskimaki, E. Veskimae, A. Tuokko, T. Visakorpi, T. L. Tammela, T. Sioris, T. Paavonen, J. Lekkala, H. Helle and N. K. J. Oksala, *Future Oncol.*, 2012, **8**, 1157–1165.
565 H. Amal, L. Ding, B. B. Liu, U. Tisch, Z. Q. Xu, D. Y. Shi, Y. Zhao, J. Chen, R. X. Sun, H. Liu, S. L. Ye, Z. Y. Tang and H. Haick, *Int. J. Nanomed.*, 2012, **7**, 4135–4146.
566 Y. X. Zhang, G. Gao, H. J. Liu, H. L. Fu, J. Fan, K. Wang, Y. S. Chen, B. J. Li, C. L. Zhang, X. Zhi, L. He and D. X. Cui, *Theranostics*, 2014, **4**, 154–162.
567 B. Kateb, M. Ryan, M. Homer, L. Lara, Y. Yin, K. Higa and M. Y. Chen, *NeuroImage*, 2009, **47**, T5–T9.
568 R. L. Siegel, K. D. Miller and A. Jemal, *Ca-Cancer J. Clin.*, 2017, **67**, 7–30.
569 G. P. Guy, D. U. Ekwueme, F. K. Tangka and L. C. Richardson, *Am. J. Prev. Med.*, 2012, **43**, 537–545.
570 T. Abaffy, M. G. Möller, D. D. Riemer, C. Milikowski and R. A. DeFazio, *Metabolomics*, 2013, **9**, 998–1008.
571 G. Pennazza, M. Santonico, A. Bartolazzi, R. Paolesse, C. Di Natale, R. Bono, V. Tamburrelli, S. Cristina and A. D'Amico, *Procedia Chem.*, 2009, **1**, 995–998.
572 A. Bartolazzi, M. Santonico, G. Pennazza, E. Martinelli, R. Paolesse, A. D'Amico and C. D. Natale, *Sens. Actuators, B*, 2010, **146**, 483–488.
573 G. Pennazza, M. Santonico, E. Martinelli, R. Paolesse, V. Tamburrelli, S. Cristina, A. D'Amico, C. Di Natale and A. Bartolazzi, *Sens. Actuators, B*, 2011, **154**, 288–294.
574 F. R. Coelho and J. O. Martins, *Rev. Assoc. Med. Bras.*, 2012, **58**, 498–504.
575 L. S. Jackson, *J. Agric. Food Chem.*, 2009, **57**, 8161–8170.
576 S. Riedel and K. C. Carroll, *J. Infect. Chemother.*, 2010, **16**, 301–316.
577 J. R. Carey, K. S. Suslick, K. I. Hulkower, J. A. Imlay, K. R. Imlay, C. K. Ingison, J. B. Ponder, A. Sen and A. E. Wittrig, *J. Am. Chem. Soc.*, 2011, **133**, 7571–7576.
578 M. Holmberg, F. Gustafsson, E. G. Hörnsten, F. Winquist, L. E. Nilsson, L. Ljung and I. Lundström, *Biotechnol. Tech.*, 1998, **12**, 319–324.
579 M. Holmberg, F. Gustafsson, E. Hörnsten, F. Winquist, L. Nilsson, L. Ljung and I. Lundström, *Biotechnol. Tech.*, 2004, **12**, 319–324.
580 T. Gibson, O. Prosser, J. Hulbert, R. Marshall, P. Corcoran, P. Lowery, E. Ruck-Keene and S. Heron, *Sens. Actuators, B*, 1997, **44**, 413–422.
581 C. L. Lonsdale, B. Taba, N. Queralto, R. A. Lukaszewski, R. A. Martino, P. A. Rhodes and S. H. Lim, *PLoS One*, 2013, **8**, e62726.
582 C. McEntegart, W. Penrose, S. Strathmann and J. Stetter, *Sens. Actuators, B*, 2000, **70**, 170–176.
583 M. Moens, A. Smet, B. Naudts, J. Verhoeven, M. Ieven, P. Jorens, H. Geise and F. Blockhuys, *Lett. Appl. Microbiol.*, 2006, **42**, 121–126.
584 J. Karasinski, S. Andreescu, O. A. Sadik, B. Lavine and M. N. Vora, *Anal. Chem.*, 2005, **77**, 7941–7949.
585 I. A. Casalinuovo, D. Di Pierro, M. Coletta and P. D. Francesco, *Sensors*, 2006, **6**, 1428–1439.
586 J. Gardner, M. Craven, C. Dow and E. Hines, *Meas. Sci. Technol.*, 1998, **9**, 120.
587 A. K. Pavlou, N. Magan, J. M. Jones, J. Brown, P. R. Klatser and A. P. Turner, *Biosens. Bioelectron.*, 2004, **20**, 538–544.
588 R. Fend, A. H. Kolk, C. Bessant, P. Buijtels, P. R. Klatser and A. C. Woodman, *J. Clin. Microbiol.*, 2006, **44**, 2039–2045.
589 N. Peled, M. Hakim, P. A. Bunn, Y. E. Miller, T. C. Kennedy, J. Mattei, J. D. Mitchell, F. R. Hirsch and H. Haick, *J. Thorac. Oncol.*, 2012, **7**, 1528–1533.
590 D. Shlomi, M. Abud, O. Liran, J. Bar, N. Gai-Mor, M. Ilouze, A. Onn, A. Ben-Nun, H. Haick and N. Peled, *J. Thorac. Oncol.*, 2017, **12**, 1544–1551.
591 I. Nardi-Agmon, M. Abud-Hawa, O. Liran, N. Gai-Mor, M. Ilouze, A. Onn, J. Bar, D. Shlomi, H. Haick and N. Peled, *J. Thorac. Oncol.*, 2016, **11**, 827–837.
592 G. Peng, M. Hakim, Y. Y. Broza, S. Billan, R. Abdah-Bortnyak, A. Kuten, U. Tisch and H. Haick, *Br. J. Cancer*, 2010, **103**, 542–551.
593 S. Dragonieri, J. T. Annema, R. Schot, M. P. C. van der Schee, A. Spanevello, P. Carratu, O. Resta, K. F. Rabe and P. J. Sterk, *Lung Cancer*, 2009, **64**, 166–170.
594 R. F. Machado, D. Laskowski, O. Deffenderfer, T. Burch, S. Zheng, P. J. Mazzone, T. Mekhail, C. Jennings, J. K. Stoller, J. Pyle, J. Duncan, R. A. Dweik and S. C. Erzurum, *Am. J. Respir. Crit. Care Med.*, 2005, **171**, 1286–1291.
595 C. Di Natale, A. Macagnano, E. Martinelli, R. Paolesse, G. D'Arcangelo, C. Roscioni, A. Finazzi-Agro and A. D'Amico, *Biosens. Bioelectron.*, 2003, **18**, 1209–1218.

Published on 11 June 2018. Downloaded on 2/1/2025 6:21:23 AM.

596 A. D'Amico, G. Pennazza, M. Santonico, E. Martinelli, C. Roscioni, G. Galluccio, R. Paolesse and C. Di Natale, *Lung Cancer*, 2010, **68**, 170–176.
597 M. Santonico, G. Lucantoni, G. Pennazza, R. Capuano, G. Galluccio, C. Roscioni, G. La Delfa, D. Consoli, E. Martinelli, R. Paolesse, C. Di Natale and A. D'Amico, *Lung Cancer*, 2012, **77**, 46–50.
598 X. Chen, M. F. Cao, Y. Li, W. J. Hu, P. Wang, K. J. Ying and H. M. Pan, *Meas. Sci. Technol.*, 2005, **16**, 1535–1546.
599 P. J. Mazzone, J. Hammel, R. Dweik, J. Na, C. Czich, D. Laskowski and T. Mekhail, *Thorax*, 2007, **62**, 565–568.
600 P. J. Mazzone, X. F. Wang, Y. M. Xu, T. Mekhail, M. C. Beukemann, J. Na, J. W. Kemling, K. S. Suslick and M. Sasidhar, *J. Thorac. Oncol.*, 2012, **7**, 137–142.
601 E. A. Chapman, P. S. Thomas, E. Stone, C. Lewis and D. H. Yates, *Eur. Respir. J.*, 2012, **40**, 448–454.
602 G. Shuster, Z. Gallimidi, A. H. Reiss, E. Dovgolevsky, S. Billan, R. Abdah-Bortnyak, A. Kuten, A. Engel, A. Shiban, U. Tisch and H. Haick, *Breast Cancer Res. Treat.*, 2011, **126**, 791–796.
603 O. Barash, W. Zhang, J. M. Halpern, Q. L. Hua, Y. Y. Pan, H. Kayal, K. Khoury, H. Liu, M. P. A. Davies and H. Haick, *Oncotarget*, 2015, **6**, 44864–44876.
604 Z. Xu, Y. Y. Broza, R. Ionsecu, U. Tisch, L. Ding, H. Liu, Q. Song, Y. Pan, F. Xiong and K. Gu, *Br. J. Cancer*, 2013, **108**, 941.
605 H. Amal, M. Leja, K. Funka, R. Skapars, A. Sivins, G. Ancans, I. Liepniece-Karele, I. Kikuste, I. Lasina and H. Haick, *Gut*, 2016, **65**, 400–407.
606 H. Amal, M. Leja, K. Funka, I. Lasina, R. Skapars, A. Sivins, G. Ancans, I. Kikuste, A. Vanags, I. Tolmanis, A. Kirsners, L. Kupcinskas and H. Haick, *Int. J. Cancer*, 2016, **138**, 229–236.
607 M. Gruber, U. Tisch, R. Jeries, H. Amal, M. Hakim, O. Ronen, T. Marshak, D. Zimmerman, O. Israel and E. Amiga, *Br. J. Cancer*, 2014, **111**, 790–798.
608 N. Leunis, M. L. Boumans, B. Kremer, S. Din, E. Stobberingh, A. G. Kessels and K. W. Kross, *Laryngoscope*, 2014, **124**, 1377–1381.
609 S. Cohen-Kaminsky, M. Nakhleh, F. Perros, D. Montani, B. Girerd, G. Garcia, G. Simonneau, H. Haick and M. Humbert, *Am. J. Respir. Crit. Care Med.*, 2013, **188**, 756–759.
610 M. K. Nakhleh, H. Haick, M. Humbert and S. Cohen-Kaminsky, *Eur. Respir. J.*, 2017, **49**, 01897.
611 K. Witt, C. Fischer, S. Reulecke, V. Kechagias, R. Surber, H. R. Figulla and A. Voss, *Biomed. Tech.*, 2013, **58**, 1–2.
612 O. Marom, F. Nakhoul, U. Tisch, A. Shiban, Z. Abassi and H. Haick, *Nanomedicine*, 2012, **7**, 639–650.
613 S. Assady, O. Marom, M. Hemli, R. Ionescu, R. Jeries, U. Tisch, Z. Abassi and H. Haick, *Nanomedicine*, 2014, **9**, 1035–1045.
614 M. K. Nakhleh, H. Amal, H. Awad, A. Gharra, N. Abu-Saleh, R. Jeries, H. Haick and Z. Abassi, *Nanomedicine*, 2014, **10**, 1767–1776.
615 U. Tisch, Y. Aluf, R. Ionescu, M. Nakhleh, R. Bassal, N. Axelrod, D. Robertman, Y. Tessler, J. P. Finberg and H. Haick, *ACS Chem. Neurosci.*, 2012, **3**, 161–166.
616 J. P. M. Finberg, Y. Aluf, Y. Loboda, M. K. Nakhleh, R. Jeries, M. Abud-Hawa, S. Zubedat, A. Avital, S. Khatib, J. Vaya and H. Haick, *ACS Chem. Neurosci.*, 2018, **9**, 291–297.
617 M. K. Nakhleh, S. Badarny, R. Winer, R. Jeries, J. Finberg and H. Haick, *Parkinsonism Relat. Disord.*, 2015, **21**, 150–153.
618 U. Tisch, I. Schlesinger, R. Ionescu, M. Nassar, N. Axelrod, D. Robertman, Y. Tessler, F. Azar, A. Marmur, J. Aharon-Peretz and H. Haick, *Nanomedicine*, 2013, **8**, 43–56.
619 J. P. Bach, M. Gold, D. Mengel, A. Hattesohl, D. Lubbe, S. Schmid, B. Tackenberg, J. Rieke, S. Maddula, J. I. Baumbach, C. Nell, T. Boeselt, J. Michelis, J. Alferink, M. Heneka, W. Oertel, F. Jessen, S. Janciauskiene, C. Vogelmeier, R. Dodel and A. R. Koczulla, *PLoS One*, 2015, **10**, e0132227.
620 A. Mazzatenta, M. Pokorski, F. Sartucci, L. Domenici and C. D. Giulio, *Respir. Physiol. Neurobiol.*, 2015, **209**, 81–84.
621 H. C. Lau, J. B. Yu, H. W. Lee, J. S. Huh and J. O. Lim, *Sensors*, 2017, **17**, 1783.
622 R. Milo and A. Miller, *Autoimmun. Rev.*, 2014, **13**, 518–524.
623 J. H. Noseworthy, C. Lucchinetti, M. Rodriguez and B. G. Weinshenker, *N. Engl. J. Med.*, 2000, **343**, 938–952.
624 R. Ionescu, Y. Broza, H. Shaltieli, D. Sadeh, Y. Zilberman, X. Feng, L. Glass-Marmor, I. Lejbkowicz, K. Mullen, A. Miller and H. Haick, *ACS Chem. Neurosci.*, 2011, **2**, 687–693.
625 J. Banerjee, V. Nema, Y. Dhas and N. Mishra, *Biochimie*, 2017, **139**, 9–19.
626 World Health Organization (WHO), Global report on diabetes, World Health Organization, 2016.
627 K. Krönert, M. Künzel, B. Reutter, C. Zimmermann, H. M. Liebich, D. Luft and M. Eggstein, *Diabetes Res. Clin. Pract.*, 1990, **10**, 161–165.
628 H. M. Liebich, *J. Chromatogr. B: Biomed. Sci. Appl.*, 1983, **273**, 67–75.
629 S. Esfahani, N. M. Sagar, I. Kyrou, E. Mozdiak, N. O'Connell, C. Nwokolo, K. D. Bardhan, R. P. Arasaradnam and J. A. Covington, *Biosensors*, 2016, **6**, 4.
630 D. Smith, P. Španěl, A. A. Fryer, F. Hanna and G. A. A. Ferns, *J. Breath Res.*, 2011, **5**, 022001.
631 P. Španěl, K. Dryahina, A. Rejšková, T. W. E. Chippendale and D. Smith, *Physiol. Meas.*, 2011, **32**, N23.
632 K. Yan, D. Zhang, D. Wu, H. Wei and G. Lu, *IEEE Trans. Biomed. Eng.*, 2014, **61**, 2787–2795.
633 R. Jaisutti, M. Lee, J. Kim, S. Choi, T.-J. Ha, J. Kim, H. Kim, S. K. Park and Y.-H. Kim, *ACS Appl. Mater. Interfaces*, 2017, **9**, 8796–8804.
634 J.-B. Yu, H.-G. Byun, M.-S. So and J.-S. Huh, *Sens. Actuators, B*, 2005, **108**, 305–308.
635 P.-J. Chien, T. Suzuki, M. Tsujii, M. Ye, I. Minami, K. Toda, H. Otsuka, K. Toma, T. Arakawa and K. Araki, *Anal. Chem.*, 2017, **89**, 12261–12268.
636 M. K. Nakhleh, R. Jeries, A. Gharra, A. Binder, Y. Y. Broza, M. Pascoe, K. Dheda and H. Haick, *Eur. Respir. J.*, 2014, **43**, 1522–1525.

 This journal is © The Royal Society of Chemistry 2018

Published on 11 June 2018. Downloaded on 2/1/2025 6:21:23 AM.

637 N. Peled, R. Ionescu, P. Nol, O. Barash, M. McCollum, K. VerCauteren, M. Koslow, R. Stahl, J. Rhyan and H. Haick, *Sens. Actuators, B*, 2012, **171**, 588–594.
638 N. M. Zetola, C. Modongo, O. Matsiri, T. Tamuhla, B. Mbongwe, K. Matlhagela, E. Sepako, A. Catini, G. Sirugo, E. Martinelli, R. Paolesse and C. Di Natale, *J. Infect.*, 2017, **74**, 367–376.
639 Y. S. Cho, S. C. Jung and S. Oh, *Lett. Appl. Microbiol.*, 2015, **60**, 513–516.
640 M. Shirasu and K. Touhara, *J. Biochem.*, 2011, **150**, 257–266.
641 B. de Lacy Costello, N. M. Ratcliffe and D. Smith, *Volatile organic compounds (VOCs) found in urine and stool*, Elsevier, Amsterdam, The Netherlands, 2013.
642 D. Samudrala, B. Geurts, P. A. Brown, E. Szymańska, J. Mandon, J. Jansen, L. Buydens, F. J. M. Harren and S. M. Cristescu, *Metabolomics*, 2015, **11**, 1656–1666.
643 E. V. Schrezenmeier, J. Barasch, K. Budde, T. Westhoff and K. M. Schmidt-Ott, *Acta Physiol.*, 2017, **219**, 556–574.
644 A. Masajtis-Zagajewska and M. Nowicki, *Clin. Chim. Acta*, 2017, **471**, 286–291.
645 S. Siyang, C. Wongchoosuk and T. Kerdcharoen, *The 5th 2012 Biomedical Engineering International Conference*, Ubon Ratchathani, 2012, pp. 1–4.
646 A. Srivastava, L. M. Douglass, V. Chernyak and K. L. Watts, *Curr. Neurol. Neurosci. Rep.*, 2017, **18**, 69.
647 M. Monteiro, N. Moreira, J. Pinto, A. S. Pires-Luís, R. Henrique, C. Jerónimo, M. d. L. Bastos, A. M. Gil, M. Carvalho and P. Guedes de Pinho, *J. Cell. Mol. Med.*, 2017, **21**, 2092–2105.
648 M. Bernabei, G. Pennazza, M. Santonico, C. Corsi, C. Roscioni, R. Paolesse, C. Di Natale and A. D'Amico, *Sens. Actuators, B*, 2008, **131**, 1–4.
649 C. M. Weber, M. Cauchi, M. Patel, C. Bessant, C. Turner, L. E. Britton and C. M. Willis, *Analyst*, 2011, **136**, 359–364.
650 A. Roine, E. Veskimäe, A. Tuokko, P. Kumpulainen, J. Koskimäki, T. A. Keinänen, M. R. Häkkinen, J. Vepsäläinen, T. Paavonen and J. Lekkala, *J. Urol.*, 2014, **192**, 230–235.
651 M. Santonico, G. Pennazza, A. D. Asimakopoulos, D. Del Fabbro, R. Miano, R. Capuano, E. Finazzi-Agrò and A. D'Amico, *Procedia Eng.*, 2014, **87**, 320–323.
652 M. Horstmann, D. Steinbach, C. Fischer, A. Enkelmann, M.-O. Grimm and A. Voss, *J. Urol.*, 2015, **193**, e560–e561.
653 A. Roine, T. Saviauk, P. Kumpulainen, M. Karjalainen, A. Tuokko, J. Aittoniemi, R. Vuento, J. Lekkala, T. Lehtimäki and T. L. Tammela, *PLoS One*, 2014, **9**, e114279.
654 A. D'Amico, M. Santonico, G. Pennazza, R. Capuano, G. Vespasiani, D. Del Fabbro, R. Paolesse, C. Di Natale, E. Martinelli and E. F. Agrò, *Procedia Eng.*, 2012, **47**, 1113–1116.
655 A. D. Asimakopoulos, D. Del Fabbro, R. Miano, M. Santonico, R. Capuano, G. Pennazza, A. D'amico and E. Finazzi-Agro, *Prostate Cancer Prostatic Dis.*, 2014, **17**, 206–211.
656 J.-L. Kim, L. Elfman, Y. Mi, G. Wieslander, G. Smedje and D. Norbäck, *Indoor Air*, 2007, **17**, 153–163.
657 S. Zehm, S. Schweinitz, R. Würzner, H. P. Colvin and J. Rieder, *Curr. Microbiol.*, 2012, **64**, 271–275.
658 T. Nawrath, G. F. Mgode, B. Weetjens, S. H. E. Kaufmann and S. Schulz, *Beilstein J. Org. Chem.*, 2012, **8**, 290.
659 M. Nakhleh, R. Jeries, A. Gharra, A. Binder, Y. Y. Broza, M. Pascoe, K. Dheda and H. Haick, *Eur. Respir. J.*, 2014, **43**, 1522–1525.
660 M. C. Lonergan, E. J. Severin, B. J. Doleman, S. A. Beaber, R. H. Grubbs and N. S. Lewis, *Chem. Mater.*, 1996, **8**, 2298–2312.
661 J. W. T. Yates, M. J. Chappell, J. W. Gardner, C. S. Dow, C. Dowson, A. Hamood, F. Bolt and L. Beeby, *Comput. Methods Programs Biomed.*, 2005, **79**, 259–271.
662 T. M. A. Sabeel, F. K. CheHarun, S. E. Eluwa and S. M. A. Sabeel, *International conference on computing, electrical and electronic engineering (ICCEEE)*, Khartoum, 2013, pp. 1–4.
663 A. K. Pavlou, N. Magan, C. McNulty, J. M. Jones, D. Sharp, J. Brown and A. P. F. Turner, *Biosens. Bioelectron.*, 2002, **17**, 893–899.
664 V. S. Kodogiannis, J. N. Lygouras, A. Tarczynski and H. S. Chowdrey, *IEEE Transactions on Information Technology in Biomedicine*, 2008, **12**, 707–713.
665 D. C. Baumgart and S. R. Carding, *Lancet*, 2007, **369**, 1627–1640.
666 R. P. Arasaradnam, N. Ouaret, M. G. Thomas, N. Quraishi, E. Heatherington, C. U. Nwokolo, K. D. Bardhan and J. A. Covington, *Inflammatory Bowel Dis.*, 2013, **19**, 999–1003.
667 J. A. Covington, E. W. Westenbrink, N. Ouaret, R. Harbord, C. Bailey, N. O'Connell, J. Cullis, N. Williams, C. U. Nwokolo and K. D. Bardhan, *Sensors*, 2013, **13**, 11899–11912.
668 C. Di Natale, A. Mantini, A. Macagnano, D. Antuzzi, R. Paolesse and A. D'Amico, *Physiol. Meas.*, 1999, **20**, 377.
669 O. Deda, H. G. Gika, I. D. Wilson and G. A. Theodoridis, *J. Pharm. Biomed. Anal.*, 2015, **113**, 137–150.
670 A. Vrieze, F. Holleman, E. G. Zoetendal, W. M. De Vos, J. B. L. Hoekstra and M. Nieuwdorp, *Diabetologia*, 2010, **53**, 606–613.
671 L. Dethlefsen, M. McFall-Ngai and D. A. Relman, *Nature*, 2007, **449**, 811–818.
672 E. Holmes, J. V. Li, T. Athanasiou, H. Ashrafian and J. K. Nicholson, *Trends Microbiol.*, 2011, **19**, 349–359.
673 K. S. Smirnov, T. V. Maier, A. Walker, S. S. Heinzmann, S. Forcisi, I. Martinez, J. Walter and P. Schmitt-Kopplin, *Int. J. Med. Microbiol.*, 2016, **306**, 266–279.
674 T. G. Meij, I. B. Larbi, M. P. Schee, Y. E. Lentferink, T. Paff, J. S. Terhaar sive Droste, C. J. Mulder, A. A. Bodegraven and N. K. Boer, *Int. J. Cancer*, 2014, **134**, 1132–1138.
675 S. F. Shepherd, N. D. McGuire, B. P. J. de Lacy Costello, R. J. Ewen, D. H. Jayasena, K. Vaughan, I. Ahmed, C. S. Probert and N. M. Ratcliffe, *J. Breath Res.*, 2014, **8**, 026001.
676 J. A. Covington, L. Wedlake, J. Andreyev, N. Ouaret, M. G. Thomas, C. U. Nwokolo, K. D. Bardhan and R. P. Arasaradnam, *Sensors*, 2012, **12**, 13002–13018.
677 R. B. Aggio, P. White, H. Jayasena, B. de Lacy Costello, N. M. Ratcliffe and C. S. Probert, *Aliment. Pharmacol. Ther.*, 2017, **45**, 82–90.

Published on 11 June 2018. Downloaded on 2/1/2025 6:21:23 AM.

678 M. Rossi, R. Aggio, H. M. Staudacher, M. C. Lomer, J. O. Lindsay, P. Irving, C. Probert and K. Whelan, *Clin. Gastroenterol. Hepatol.*, 2017, **6**, 31201–31206.
679 T. G. de Meij, N. K. de Boer, M. A. Benninga, Y. E. Lentferink, E. F. de Groot, M. E. van de Velde, A. A. van Bodegraven and M. P. van der Schee, *J. Crohns Colitis*, 2014, DOI: 10.1016/j.crohns.2014.09.004.
680 T. G. J. de Meij, M. P. C. van der Schee, D. J. C. Berkhout, M. E. van de Velde, A. E. Jansen, B. W. Kramer, M. M. van Weissenbruch, A. H. van Kaam, P. r. Andriessen and J. B. van Goudoever, *J. Pediatr.*, 2015, **167**, 562–567.
681 N. D. McGuire, R. J. Ewen, B. de Lacy Costello, C. E. Garner, C. S. J. Probert, K. Vaughan and N. M. Ratcliffe, *Meas. Sci. Technol.*, 2014, **25**, 065108.
682 D. J. C. Berkhout, H. J. Niemarkt, M. Buijck, M. M. Van Weissenbruch, P. Brinkman, M. A. Benninga, A. H. van Kaam, B. W. Kramer, P. r. Andriessen and N. K. H. de Boer, *J. Pediatr. Gastroenterol. Nutr.*, 2017, **65**, e47–e52.
683 R. M. Nagler, O. Hershkovich, S. Lischinsky, E. Diamond and A. Z. Reznick, *J. Invest. Med.*, 2002, **50**, 214–225.
684 M. Gallagher, C. J. Wysocki, J. J. Leyden, A. I. Spielman, X. Sun and G. Preti, *Br. J. Dermatol.*, 2008, **159**, 780–791.
685 A. Koh, D. Kang, Y. Xue, S. Lee, R. M. Pielak, J. Kim, T. Hwang, S. Min, A. Banks, P. Bastien, M. C. Manco, L. Wang, K. R. Ammann, K.-I. Jang, P. Won, S. Han, R. Ghaffari, U. Paik, M. J. Slepian, G. Balooch, Y. Huang and J. A. Rogers, *Sci. Transl. Med.*, 2016, **8**, 366ra165.
686 A. J. Bandodkar, V. W. S. Hung, W. Jia, G. Valdés-Ramírez, J. R. Windmiller, A. G. Martinez, J. Ramírez, G. Chan, K. Kerman and J. Wang, *Analyst*, 2013, **138**, 123–128.
687 http://www.aarp.org/health/conditions-treatments/info-2017/color-changing-tattoos-health-monitoring-fd.html.
688 Z.-M. Zhang, J.-J. Cai, G.-H. Ruan and G.-K. Li, *J. Chromatogr. B: Anal. Technol. Biomed. Life Sci.*, 2005, **822**, 244–252.
689 M. Kusano, E. Mendez and K. G. Furton, *J. Forensic Sci.*, 2013, **58**, 29–39.
690 P. Mochalski, K. Unterkofler, H. Hinterhuber and A. Amann, *Anal. Chem.*, 2014, **86**, 3915–3923.
691 K. Liddell, *Postgrad. Med. J.*, 1976, **52**, 136–138.
692 W. Z. D. Stitt and L. A. Goldsmith, *Arch. Dermatol.*, 1995, **131**, 997–999.
693 P. J. Honig, I. J. Frieden, H. J. Kim and A. C. Yan, *Pediatrics*, 2003, **112**, 1427–1429.
694 B. W. M. Moeskops, M. M. L. Steeghs, K. Van Swam, S. M. Cristescu, P. T. J. Scheepers and F. J. M. Harren, *Physiol. Meas.*, 2006, **27**, 1187.
695 C. Di Natale, A. Macagnano, R. Paolesse, E. Tarizzo, A. Mantini and A. D'Amico, *Sens. Actuators, B*, 2000, **65**, 216–219.
696 Y. Y. Broza, L. Zuri and H. Haick, *Sci. Rep.*, 2014, **4**, 1–6.
697 P. Martínez-Lozano and J. F. de la Mora, *J. Am. Soc. Mass Spectrom*, 2009, **20**, 1060–1063.
698 L. Dormont, J.-M. Bessière and A. Cohuet, *J. Chem. Ecol.*, 2013, **39**, 569–578.
699 P. Mochalski, J. King, K. Unterkofler, H. Hinterhuber and A. Amann, *J. Chromatogr. B: Anal. Technol. Biomed. Life Sci.*, 2014, **959**, 62–70.
700 S. Riazanskaia, G. Blackburn, M. Harker, D. Taylor and C. L. P. Thomas, *Analyst*, 2008, **133**, 1020–1027.
701 A. M. Curran, S. I. Rabin, P. A. Prada and K. G. Furton, *J. Chem. Ecol.*, 2005, **31**, 1607–1619.
702 H. Kataoka, *Anal. Sci.*, 2011, **27**, 893.
703 A. D'amico, R. Bono, G. Pennazza, M. Santonico, G. Mantini, M. Bernabei, M. Zarlenga, C. Roscioni, E. Martinelli and R. Paolesse, *Skin Res. Technol.*, 2008, **14**, 226–236.
704 C. Di Natale, R. Paolesse, P. Comandini, G. Pennazza, E. Martinelli, S. Rullo, M. Roscioni, C. Roscioni and A. Finazzi-Agro, *Med. Sci. Monit.*, 2005, **11**, CR366–CR375.
705 C. Turner, *Expert Rev. Mol. Diagn.*, 2011, **11**, 497–503.
706 N. Yamane, T. Tsuda, K. Nose, A. Yamamoto, H. Ishiguro and T. Kondo, *Clin. Chim. Acta*, 2006, **365**, 325–329.
707 C. Turner, B. Parekh, C. Walton, P. Španěl, D. Smith and M. Evans, *Rapid Commun. Mass Spectrom.*, 2008, **22**, 526–532.
708 Z. Zhang and G. Li, *Microchem. J.*, 2010, **95**, 127–139.
709 S. G. Gordon, K. Smith, J. L. Rabinowitz and P. R. Vagelos, *J. Lipid Res.*, 1973, **14**, 495–503.
710 K. Smith, G. F. Thompson and H. D. Koster, *Science*, 1969, **166**, 398–399.
711 U. R. Bernier, M. M. Booth and R. A. Yost, *Anal. Chem.*, 1999, **71**, 1–7.
712 U. R. Bernier, D. L. Kline, D. R. Barnard, C. E. Schreck and R. A. Yost, *Anal. Chem.*, 2000, **72**, 747–756.
713 M. Shirasu, S. Nagai, R. Hayashi, A. Ochiai and K. Touhara, *Biosci., Biotechnol., Biochem.*, 2009, **73**, 2117–2120.
714 A. N. Thomas, S. Riazanskaia, W. Cheung, Y. Xu, R. Goodacre, C. L. Thomas, M. S. Baguneid and A. Bayat, *Wound Repair Regen.*, 2010, **18**, 391–400.
715 H.-G. Byun, K. C. Persaud and A. M. Pisanelli, *ETRI J.*, 2010, **32**, 440–446.
716 J. G. Logan, M. A. Birkett, S. J. Clark, S. Powers, N. J. Seal, L. J. Wadhams, A. J. Mordue and J. A. Pickett, *J. Chem. Ecol.*, 2008, **34**, 308.
717 A. Voss, K. Witt, C. Fischer, S. Reulecke, W. Poitz, V. Kechagias, R. Surber and H. R. Figulla, *Conf. Proc. IEEE Eng. Med. Biol. Soc.*, 2012, **7**, 6346852.
718 A. Voss, V. Baier, R. Reisch, K. von Roda, P. Elsner, H. Ahlers and G. Stein, *Ann. Biomed. Eng.*, 2005, **33**, 656–660.
719 A. D'Amico, C. Di Natale, A. Macagnano, F. Davide, A. Mantini, E. Tarizzo, R. Paolesse and T. Boschi, *Biosens. Bioelectron.*, 1998, **13**, 711–721.
720 A. D'Amico, C. Di Natale, R. Paolesse, A. Macagnano and A. Mantini, *Sens. Actuators, B*, 2000, **65**, 209–215.
721 C. Di Natale, R. Paolesse and A. D'Amico, *Sens. Actuators, B*, 2007, **121**, 238–246.
722 H. Ulmer, J. Mitrovics, U. Weimar and W. Göpel, *Sens. Actuators, B*, 2000, **65**, 79–81.
723 M. Pardo, L. G. Kwong, G. Sberveglieri, K. Brubaker, J. F. Schneider, W. R. Penrose and J. R. Stetter, *Sens. Actuators, B*, 2005, **106**, 136–143.
724 M. Holmberg, F. Winquist, I. Lundström, J. W. Gardner and E. L. Hines, *Sens. Actuators, B*, 1995, **27**, 246–249.
725 H. Ulmer, J. Mitrovics, G. Noetzel, U. Weimar and W. Göpel, *Sens. Actuators, B*, 1997, **43**, 24–33.

This journal is © The Royal Society of Chemistry 2018

726 C. Jin, P. Kurzawski, A. Hierlemann and E. T. Zellers, *Anal. Chem.*, 2008, **80**, 227–236.
727 C. Jin and E. T. Zellers, *Anal. Chem.*, 2008, **80**, 7283–7293.
728 N. P. Pont, C. A. Kendall and N. Magan, *Mycopathologia*, 2012, **173**, 93–101.
729 C.-L. Li, Y.-F. Chen, M.-H. Liu and C.-J. Lu, *Sens. Actuators, B*, 2012, **169**, 349–359.
730 H.-L. Lu, C.-J. Lu, W.-C. Tian and H.-J. Sheen, *Talanta*, 2015, **131**, 467–474.
731 K. Scholten, L. K. Wright and E. T. Zellers, *IEEE Sens. J.*, 2013, **13**, 2146–2154.
732 R. Gutierrez-Osuna and A. Hierlemann, *Annu. Rev. Anal. Chem.*, 2010, **3**, 255–276.
733 D. C. Meier, B. Raman and S. Semancik, *Annu. Rev. Anal. Chem.*, 2009, **2**, 463–484.
734 R. E. Pogue, D. P. Cavalcanti, S. Shanker, R. V. Andrade, L. R. Aguiar, J. L. de Carvalho and F. F. Costa, *Drug Discovery Today*, 2017, **23**, 187–195.
735 P. K. Drain, E. P. Hyle, F. Noubary, K. A. Freedberg, D. Wilson, W. R. Bishai, W. Rodriguez and I. V. Bassett, *Lancet Infect. Dis.*, 2014, **14**, 239–249.
736 D. Kuupiel, V. Bawontuo and T. P. Mashamba-Thompson, *Diagnostics*, 2017, **7**, 58.
737 P. B. Luppa, C. Müller, A. Schlichtiger and H. Schlebusch, *TrAC, Trends Anal. Chem.*, 2011, **30**, 887–898.
738 K. J. Johnson and S. L. Rose-Pehrsson, *Annu. Rev. Anal. Chem.*, 2015, **8**, 287–310.

Published on 11 June 2018. Downloaded on 2/1/2025 6:21:23 AM.

ADVANCED MATERIALS
www.advmat.de

UV–Ozone Interfacial Modification in Organic Transistors for High-Sensitivity NO_2 Detection

Wei Huang, Xinming Zhuang, Ferdinand S. Melkonyan, Binghao Wang, Li Zeng, Gang Wang, Shijiao Han, Michael J. Bedzyk, Junsheng Yu, Tobin J. Marks,* and Antonio Facchetti**

A new type of nitrogen dioxide (NO_2) gas sensor based on copper phthalocyanine (CuPc) thin film transistors (TFTs) with a simple, low-cost UV–ozone (UVO)-treated polymeric gate dielectric is reported here. The NO_2 sensitivity of these TFTs with the dielectric surface UVO treatment is ≈400× greater for [NO_2] = 30 ppm than for those without UVO treatment. Importantly, the sensitivity is ≈50× greater for [NO_2] = 1 ppm with the UVO-treated TFTs, and a limit of detection of ≈400 ppb is achieved with this sensing platform. The morphology, microstructure, and chemical composition of the gate dielectric and CuPc films are analyzed by atomic force microscopy, grazing incident X-ray diffraction, X-ray photoelectron spectroscopy, and Fourier transform infrared spectroscopy, revealing that the enhanced sensing performance originates from UVO-derived hydroxylated species on the dielectric surface and not from chemical reactions between NO_2 and the dielectric/semiconductor components. This work demonstrates that dielectric/semiconductor interface engineering is essential for readily manufacturable high-performance TFT-based gas sensors.

Economic growth and quality-of-life improvement during the past decades have dramatically increased worldwide energy consumption. Most of the energy needs for transportation, domestic, and industrial usage originate from combustion of fossil fuels, which are not renewable and release toxic gases into the atmosphere.[1] Especially in developing countries, massive production of harmful gases has become problematic to society.[2] Therefore, accurate, cost-effective means of monitoring and detecting air quality is of paramount importance since these gases do not only pose environmental concerns but also endanger public health.[3]

Among the harmful environmental gases, nitrogen dioxide (NO_2) is particularly dangerous.[4] Although short-term exposure (1–3 h) of healthy individuals to NO_2 at concentrations <1 ppm does not typically induce adverse pulmonary effects, long-term exposure to low NO_2 concentrations (≈1 ppm) can impair respiratory function and increase the risk of emphysema and bronchitis.[5] Individuals with asthma or allergies experience negative pulmonary function at NO_2 concentration as low as 0.2–0.6 ppm.[6] Moreover, ≥10 ppm NO_2 concentration causes immediate distress, including edema, nose and throat irritation, and >100 ppm can cause death by asphyxiation.[7] Furthermore, NO_2 is a component of acid rain and accelerates the formation of microscopic particles in the air.

Currently several technologies, including chemiluminescent, electrochemical, resistive, and optical sensing are used for NO_2 detection.[8] Although some commercial sensors have low limits of detection (LOD), usually below ppm, even ppt level, and good selectively, they usually are limited by high cost and/or high operating power consumption. In contrast, simple, readily manufactured sensors based on organic thin-film transistors (OTFTs) potentially offer low-cost, low power consumption, and high performance, and are regarded as promising candidates for next-generation gas sensors.[9–12] Moreover, OTFT-based gas sensors can be integrated with standard integrated circuit (IC) technologies, making it possible to fabricate smart, portable electronic noses integrable with cell-phones, laptops, and smart appliances.[13]

To enhance OTFT sensor performance, typical approaches have focused on the organic semiconductor (OSCs) and gate dielectric materials.[14–17] Thus, Huang used naphthalene-tetracarboxylic diimide derivatives and copper phthalocyanine (CuPc) to fabricate OTFT sensor arrays, showing the potential for rapid and selective volatile analyte detection.[18] Sensitive dielectric and interface design was also adopted for realizing high-performance TFT-based gas, light, and biosensors.[19–21] Shaymurat

Dr. W. Huang, X. Zhuang, S. Han, Prof. J. Yu
State Key Laboratory of Electronic Thin Films and Integrated Devices
School of Optoelectronic Information
University of Electronic Science and Technology of China (UESTC)
Chengdu 610054, China
E-mail: jsyu@uestc.edu.cn

Dr. W. Huang, Prof. F. S. Melkonyan, Dr. B. Wang, Dr. G. Wang, Prof. T. J. Marks, Prof. A. Facchetti
Department of Chemistry
Northwestern University
2145 Sheridan Road, Evanston, IL 60208, USA
E-mail: t-marks@northwestern.edu; a-facchetti@northwestern.edu

L. Zeng, Prof. M. J. Bedzyk
Department of Materials Science and Engineering and Applied Physics Program
Northwestern University
2220 Campus Drive, Evanston, IL 60208, USA

Prof. A. Facchetti
Flexterra Inc.
8025 Lamon Avenue, Skokie, IL 60077, USA

The ORCID identification number(s) for the author(s) of this article can be found under https://doi.org/10.1002/adma.201701706.

DOI: 10.1002/adma.201701706

© 2017 WILEY-VCH Verlag GmbH & Co. KGaA, Weinheim

developed high-performance SO_2 sensors with a 0.5 ppm LOD using an air gap as the dielectric layer.[22] Another strategy focuses on creating highly sensitive organic semiconductors via chemical modifications which combine charge-carrying cores with peripheral analyte receptors. Implementing semiconductors with functional groups that strongly bind the target analyte can enhance both sensitivity and selectivity.[23–27] Thus, Katz increased OTFT ammonia response using strongly Lewis acidic tris(pentafluorophenyl)borane as the receptor, affording an LOD of 350 ppb.[24] Utilizing optimized device geometries is also effective in enhancing TFT gas sensor performance. Thus, ultrathin and/or structured OSCs can enlarge the surface-to-volume ratio, thereby enhancing sensitivity and lowering the response time.[28–32] Mirza reported NO_2 gas sensing with vapor-deposited ultrathin/sub-monolayer pentacene films which exhibited high sensitivity, fast response/recovery time, an LOD of 100 ppb, and excellent reproducibility.[33] Top-gate OTFTs with sensitive gate dielectrics were also reported by Klug to detect low-concentrations of NH_3.[34] From these results it can be surmised that high-sensitivity OTFT gas sensors can require highly sensitive OSCs and unconventional TFT structures, which may introduce complexity and increase fabrication cost. Thus, achieving high-performance devices while preserving straightforward, low-cost fabrication would be highly desirable.[35]

Here we realize ultrasensitive OTFT-based NO_2 sensors by implementing a simple, low-cost UV–ozone (UVO) treatment of the gate dielectric. We show that the dielectric surface chemistry, and thus the interfacial trap density, can be controlled by simply adjusting the UVO treatment time (t_{UVO}). These trap sites influence the OTFT performance for both unexposed and NO_2 vapor exposed devices. Upon NO_2 exposure, the analyte molecules strongly adsorb to the UVO-induced functional groups, which is essential for achieving ultrasensitive devices. The sensitivities at $[NO_2]$ = 1 and 30 ppm are enhanced by ≈50 and ≈400 times, respectively, upon t_{UVO} optimization. Furthermore, the enhanced NO_2 adsorption on the UVO-treated dielectric surface enables these devices to retain NO_2 at room temperature in ambient, essential for cost-effective NO_2 sensor integration.

The present TFT configuration and device fabrication process are shown in **Figure 1**a. Glass with patterned ITO substrates as gate electrodes were cleaned by sonication in acetone, deionized (DI) water, and isopropyl alcohol for 15 min, followed by O_2 plasma treatment for 5 min (20 W). Poly(vinyl alcohol) (PVA, M_w ≈ 146 000–186 000; 40 mg mL^{-1} in high purity water) was spin-coated onto the ITO substrates and annealed at 70 °C for 1 h. Polystyrene (PS, M_w ≈ 280 000; 30 mg mL^{-1} in *o*-xylene) was then spin-coated on top and baked at 120 °C for 1.0 h to remove residual solvents. A double-layer dielectric is used here to provide good adhesion to the gate electrode and achieve excellent insulating properties. Prior to deposition of the organic semiconductor, the dielectric film was exposed to UV light at 185 and 253.7 nm (UVO-Cleaner 42, Jelight

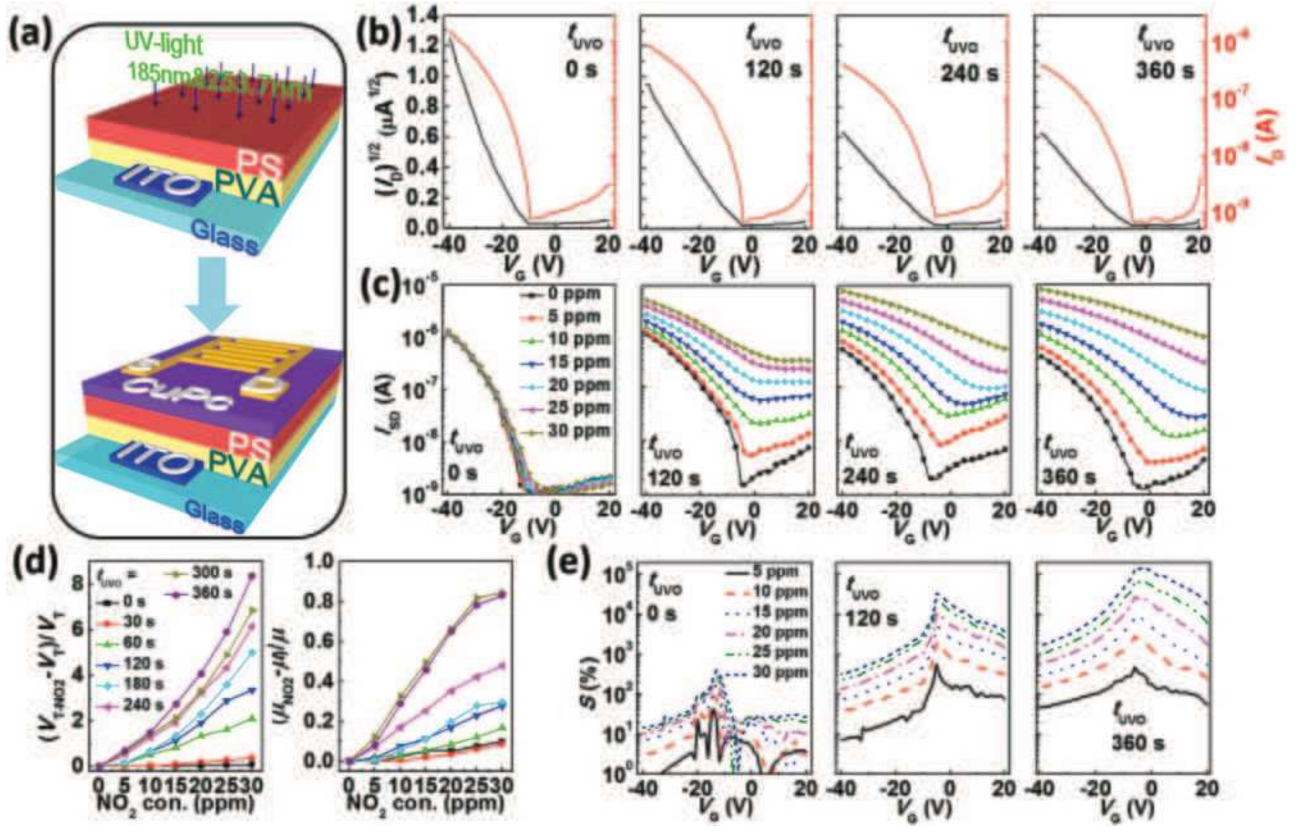

Figure 1. a) Bottom gate CuPc OTFT structure and UVO treatment of the gate dielectric surface. b) Transfer curves for different t_{UVO} values. c) Transfer curves for the indicated t_{UVO} values and different NO_2 concentrations. d) Percentage V_T and mobility variations as a function of t_{UVO} and NO_2 concentrations. e) Sensitivity (S)–V_G plots for the indicated t_{UVO} values and NO_2 concentrations.

© 2017 WILEY-VCH Verlag GmbH & Co. KGaA, Weinheim

15214095, 2017, 31, Downloaded from https://advanced.onlinelibrary.wiley.com/doi/10.1002/adma.201701706 by Nanjing Tech University, Wiley Online Library on [31/01/2025]. See the Terms and Conditions (https://onlinelibrary.wiley.com/terms-and-conditions) on Wiley Online Library for rules of use; OA articles are governed by the applicable Creative Commons License

15214095, 2017, 31, Downloaded from https://advanced.onlinelibrary.wiley.com/doi/10.1002/adma.201701706 by Nanjing Tech University, Wiley Online Library on [31/01/2025]. See the Terms and Conditions (https://onlinelibrary.wiley.com/terms-and-conditions) on Wiley Online Library for rules of use; OA articles are governed by the applicable Creative Commons License

Table 1. Summary of the CuPc TFT sensor performance parameters for different t_{UVO} of the gate dielectric.

t_{UVO} [s]	0	30	60	120	180	240	300	360
I_{ON} [10^{-6} A]	1.6 ± 0.3	1.9 ± 0.5	1.2 ± 0.2	0.92 ± 0.15	0.52 ± 0.09	0.41 ± 0.11	0.32 ± 0.07	0.29 ± 0.09
V_{ON} [V]	−10.5 ± 2.1	−7.2 ± 1.8	−4.7 ± 2.5	−4.2 ± 4.1	−4.5 ± 3.6	−5.1 ± 2.3	−5.0 ± 1.9	−4.7 ± 2.6
μ [10^{-3} cm^2 V^{-1} s^{-1}]	3.1 ± 0.4	3.2 ± 0.5	1.6 ± 0.2	1.3 ± 0.3	0.79 ± 0.08	0.56 ± 0.4	0.49 ± 0.06	0.51 ± 0.04
V_T [V]	−14.2 ± 2.1	−12.3 ± 1.5	−9.1 ± 2.3	−7.1 ± 3.1	−7.1 ± 2.1	−6.8 ± 1.5	−6.8 ± 2.0	−7.0 ± 2.1

Company Inc.) for a time (t_{UVO}) varying from 0 to 360 s. Next, a 10 nm CuPc film was deposited by thermal evaporation. The transistors were then completed by depositing 40 nm thick gold source and drain electrodes via thermal evaporation using a metal shadow mask to form a channel length of 100 µm and width of 1.0 cm. Before establishing the response of the platform to NO_2, the effect of t_{UVO} on the CuPc TFT performance was investigated, and data are summarized in **Table 1**.

Figure 1b and Figure S1 (Supporting Information) indicate that the *I–V* transfer characteristics are strongly affected by t_{UVO}. Thus, the on-current (I_{ON}, drain current measured at $V_D = V_G = -40$ V) of the untreated device (≈1.6×10^{-6} A) first slightly increases to ≈1.9×10^{-6} A for $t_{UVO} = 30$ s, next decreases to ≈1.2×10^{-6} and ≈3.2×10^{-7} A as t_{UVO} is increased from 60 to 300 s, respectively, and finally stabilizes at ≈3.2×10^{-7} A for $t_{UVO} > 300$ s. Meanwhile, a shift of the turn-on voltage (V_{ON}, the gate voltage at which the current start increasing sharply) toward positive values is observed [V_{ON} (t_{UVO}) = −10.5 V (0 s); −7.2 V (30 s); −4.7 V (60 s)]. When t_{UVO} exceeds 60 s, V_{ON} stabilizes at ≈−4.5 V. Moreover, as t_{UVO} increases from 30 to 300 s, the on/off current ratio monotonically falls from ≈2×10^3 ($t_{UVO} = 30$ s) to ≈4×10^2 ($t_{UVO} = 300$ s). To verify that the TFT performance variations are not due to major changes in the gate insulator dielectric properties, the leakage current and capacitance of the PVA/PS gate dielectric for different UVO exposure times were measured in MIM capacitors of the structure, ITO/dielectric/Au (Figure S2, Supporting Information). The leakage current and capacitance values of all samples exhibit small variations with t_{UVO}, with the current density remaining <≈10^{-7} A cm^{-2} (at ±20 V) and the capacitance slightly increasing from 10.2 nF cm^{-2} ($t_{UVO} = 0$ s) to 11.9 nF cm^{-2} ($t_{UVO} = 360$ s). This reflects the mild etching effects of UVO treatment on polymer films,[36] with the present dielectric layer thickness contracting by ≈20 nm (from 530 to 510 nm) for $t_{UVO} = 360$ s.

From the *I–V* characteristics, the FET performance parameters were extracted using standard MOSFET equations,[3,13] and mobility (μ) and threshold voltage (V_T) data are summarized in Table 1. The TFT data indicate that OTFT performance of these devices stabilize when $t_{UVO} > 300$ s. Thus, μ decreases from 3.1×10^{-3} cm^2 V^{-1} s^{-1} ($t_{UVO} = 0$ s) to 1.3×10^{-3} cm^2 V^{-1} s^{-1} ($t_{UVO} = 120$ s) to 4.9×10^{-4} cm^2 V^{-1} s^{-1} ($t_{UVO} = 300$ s). In parallel, a consistent positive shift of V_T is observed [V_T (t_{UVO}) = −14.2 V (0 s); −9.1 V (60 s); −7.1 V (120 s)], and stabilizes at ≈−7.0 V ($t_{UVO} \geq 120$ s). Variations of the electrical parameters with t_{UVO} in our OTFTs are in line with previous reports of pentacene TFTs with UVO-treated polymeric dielectrics.[37–39]

The sensor performance of the present CuPc TFTs with/without UVO-treated dielectric surfaces was characterized using gaseous NO_2 as the analyte in the 5–30 ppm concentration range. The details of the *I–V* parameters are summarized in Table S1 (Supporting Information). In a typical experiment, the OTFT sensor was placed in an airtight test chamber (≈2.4 mL), and a mixture of dry air and 50 ppm NO_2 gas mixed in appropriate concentrations was introduced into the test chamber by mass flow controllers (see Figure S3, Supporting Information). The flow rate in the experiment was fixed at 100 sccm (standard cm^3 min^{-1}), and OTFT *I–V* measurements were carried out after 2 min of NO_2 exposure in the test chamber. Figure 1c and Figure S4 (Supporting Information) show that the NO_2 sensitivity of this platform strongly depends on UVO processing and t_{UVO}. The *I–V* characteristics clearly demonstrate that after the dielectric surface is exposed to UVO, the NO_2 sensitivity of the *I–V* characteristics increase dramatically. Thus, for TFTs with a pristine PS/PVA dielectric, as the [NO_2] increases, there is only a slightly increase of the I_{ON} [from 1.19×10^{-6} A ([NO_2] = 0 ppm) to 1.27×10^{-6} A ([NO_2] = 15 ppm) to 1.36×10^{-6} A ([NO_2] = 30 ppm)] and a small shift of V_{ON} [from −12 V (0 ppm NO_2) to −9 V (15 ppm NO_2) to −8 V (30 ppm NO_2)]. No obvious off-current (I_{OFF}) variations were observed, which remains at ≈10^{-9} A. In marked contrast, for the UVO-treated devices, the transfer curves exhibit considerable variations upon gas exposure. For the device with w I_{ON} increases from 1.14×10^{-6} A ([NO_2] = 0 ppm) to 1.77×10^{-6} A ([NO_2] = 15 ppm) to 3.18×10^{-6} A ([NO_2] = 30 ppm), V_{ON} shifts from −4 V ([NO_2] = 0 ppm) to 1 V ([NO_2] = 15 ppm) to 8 V ([NO_2] = 30 ppm), and I_{OFF} increases from 1.47×10^{-9} A ([NO_2] = 0 ppm) to 1.3×10^{-8} A ([NO_2] = 15 ppm) to 6.06×10^{-8} A ([NO_2] = 30 ppm). Furthermore, when exposed to NO_2, the variations of the transfer curves enlarge as t_{UVO} increases up to ≥300 s, at which time they stabilize. Thus, as shown in Figure 1c and Figure S4 (Supporting Information), for $t_{UVO} = 300$ s, I_{ON} increases from 2.89×10^{-7} A ([NO_2] = 0 ppm) to 1.11×10^{-6} A ([NO_2] = 15 ppm) to 4.75×10^{-6} A ([NO_2] = 30 ppm) while V_{ON} shifts considerably from −5 V ([NO_2] = 0 ppm) to 13 V ([NO_2] = 15 ppm) to >20 V ([NO_2] = 30 ppm). At [NO_2] = 30 ppm the transfer curve becomes almost linear without a distinct off state in the gate voltage (V_G) region of +20 to −40 V.

The corresponding TFT μ and V_T parameters for different t_{UVO} and [NO_2] values are summarized in **Table 2**. Untreated devices exhibit minimal μ (4.0×10^{-3} to 4.4×10^{-3} cm^2 V^{-1} s^{-1}) and V_T (−14.8 to −13.3 V) changes upon NO_2 exposure. However, as the t_{UVO} increases, μ increases with increasing [NO_2], along with a large positive V_T shift. For example, μ for the transistor with a $t_{UVO} = 120$ s monotonously increases from 1.8×10^{-3} cm^2 V^{-1} s^{-1} ([NO_2] = 0 ppm) to 2.4×10^{-3} cm^2 V^{-1} s^{-1} ([NO_2] = 30 ppm) and the V_T shifts from −7.2 V ([NO_2] = 0 ppm) to +17 V ([NO_2] = 30 ppm). Furthermore, for the OTFT with a $t_{UVO} = 300$ s, μ increases from 8.3×10^{-4} cm^2 V^{-1} s^{-1} ([NO_2] = 0 ppm) to 1.5×10^{-3} cm^2 V^{-1} s^{-1} ([NO_2] = 30 ppm)

© 2017 WILEY-VCH Verlag GmbH & Co. KGaA, Weinheim

Table 2. Summary of mobility and threshold voltage variations for different t_{UVO}-treated devices as a function of NO_2 concentration.

		t_{UVO} [s]							
NO_2 con. [ppm]		0	30	60	120	180	240	300	360
0	μ (10^{-3} cm^2 V^{-1} s^{-1})	4.0	3.5	2.8	1.8	1.6	1.4	0.8	0.8
5		4.0	3.5	2.7	1.9	1.6	1.5	0.9	0.9
10		4.1	3.6	2.9	2.0	1.7	1.7	1.1	1.1
15		4.2	3.6	3.0	2.0	1.8	1.8	1.2	1.2
20		4.2	3.6	3.0	2.1	1.9	1.9	1.4	1.4
25		4.3	3.7	3.1	2.3	2.1	2.0	1.5	1.5
30		4.4	3.8	3.3	2.4	2.1	2.1	1.5	1.5
0	V_T [V]	−14.8	−12.7	−8.8	−7.2	−7.0	−7.6	−7.5	−7.1
5		−14.7	−12.7	−7.7	−6.1	−5.9	−3.5	−2.6	−2.1
10		−14.4	−12.1	−4.0	−2.5	−2.1	2.2	3.0	3.6
15		−14.2	−11.3	−1.4	1.1	2.5	7.2	8.5	12.4
20		−13.9	−10.1	3.1	6.6	9.3	17.1	17.2	21.9
25		−13.6	−9.0	5.6	13.7	18.2	25.5	29.3	34.9
30		−13.3	−7.8	9.8	17.0	28.2	39.3	43.9	52.3

while V_T shifts from −7.5 V ([NO_2] = 0 ppm) to +43.9 V ([NO_2] = 30 ppm). The relative variations of both μ and V_T are summarized in Figure 1d, which also indicates that the TFT parameter shifts tend to saturate when $t_{UVO} \geq 300$ s, in agreement with the *I*–*V* characteristics.

An important sensor performance parameter is the sensitivity (*S*) to the given analyte. As is standard for TFT sensors,[22,28,29] the sensitivity (*S*) here for gas analyte is given by using Equation (1), where I_{Gas} is the drain current of the transistor

$$S = \left(\frac{I_{Gas} - I_0}{I_0}\right) \times 100\% \quad (1)$$

when exposed to gas analyte and I_0 is the drain current of the unexposed transistor. As shown in Figure 1e and Figure S5 (Supporting Information), the sensitivity to NO_2 increases as t_{UVO} increases, and since the transfer curve is V_G dependent, the sensitivity also depends on V_G with the highest sensitivity lying in the −15 to 0 V V_G region. Upon NO_2 exposure, the transfer curve shifts toward the upper-left corner of the transfer plot (Figure S4, Supporting Information), along with an enlarged I_{OFF}. Clearly, the highest sensitivity will not be in the region where the transistors are in the on-state, but close to the region where the original turn-on voltages are.

As shown in Figure 1e and Figure S5 (Supporting Information), for V_G = −40 V, the sensitivity at [NO_2] = 30 ppm is the largest and increases from ≈400% (t_{UVO} = 0 s) to ≈35 000% (t_{UVO} = 120 s) to ≈90 000% (t_{UVO} = 240 s), and saturates at t_{UVO} = ≈300 s with a sensitivity as high as ≈160 000%. Even for a low [NO_2] of 5 ppm, high sensitivity of ≈500% is achieved for the device with t_{UVO} = 360 s. In contrast, although the sensitivity at $V_D = V_G = -40$ V is relatively low, ≈1600% for [NO_2] = 30 ppm, it is equally important since the saturation region usually has a much higher signal-to-noise ratio, the reliability of the data is enhanced. From Figure S5 (Supporting Information), the sensitivity in saturation ($V_D = V_G = -40$ V) for a [NO_2] = 30 ppm increases from ≈15% (t_{UVO} = 0 s) to ≈300% (t_{UVO} = 120 s) to ≈1000% (t_{UVO} = 240 s), and reaches maximum of ≈1600% at t_{UVO} = 360 s.

To illuminate the origin of the increased detection performance, the film morphologies of the gate dielectric (on top of ITO) and of the organic semiconductor (on top of the dielectric) were first probed by atomic force microscopy (AFM). Such film morphologies are known to affect the sensing performance of OTFT-based gas sensors.[40,41] AFM images of the dielectric films with various t_{UVO} treatments indicate negligible topological variations (**Figure 2**a and Figure S6, Supporting Information), with all films remaining very smooth with an RMS roughness of 0.24–0.29 nm. More interestingly, the CuPc films grown on the dielectric treated for different t_{UVO}'s also exhibit similar morphologies, which are characterized by small grains with ≈25 nm dimensions and a considerable density of grain boundaries (Figure 2b). The phase images of these films (see Figure 2c) further confirm that the semiconductor grain topology/mechanical properties and density remain unchanged with various t_{UVO}'s. A large density of grain boundaries will facilitate analyte diffusion to the dielectric/semiconductor interface, which can enhance the sensing performance of OTFTs.[42] However, since the quality of the grain boundaries does not change with t_{UVO}, these data demonstrate that this semiconductor morphology simply creates the condition for large sensitivity but it cannot underlie the detection mechanism in the present sensors. Moreover, grazing incidence X-ray diffraction (GIXRD) measurements (Figure 2d) indicate that all CuPc films exhibit the same strong (200) diffraction at $2\theta = 6.9°$.[43] By fitting the diffraction peaks to a Gaussian function, a full width at the half maximum (FWHM) of 0.55° ± 0.1° is obtained for all the (200) diffraction peaks, indicating that UVO treatment does not significantly affect CuPc crystallite dimensions (≈14.5 nm) or orientation on the dielectric film. Thus, semiconductor and dielectric film morphologies are

 © 2017 WILEY-VCH Verlag GmbH & Co. KGaA, Weinheim

15214095, 2017, 31, Downloaded from https://advanced.onlinelibrary.wiley.com/doi/10.1002/adma.201701706 by Nanjing Tech University, Wiley Online Library on [31/01/2025]. See the Terms and Conditions (https://onlinelibrary.wiley.com/terms-and-conditions) on Wiley Online Library for rules of use; OA articles are governed by the applicable Creative Commons License

15214095, 2017, 31, Downloaded from https://advanced.onlinelibrary.wiley.com/doi/10.1002/adma.201701706 by Nanjing Tech University, Wiley Online Library on [31/01/2025]. See the Terms and Conditions (https://onlinelibrary.wiley.com/terms-and-conditions) on Wiley Online Library for rules of use; OA articles are governed by the applicable Creative Commons License

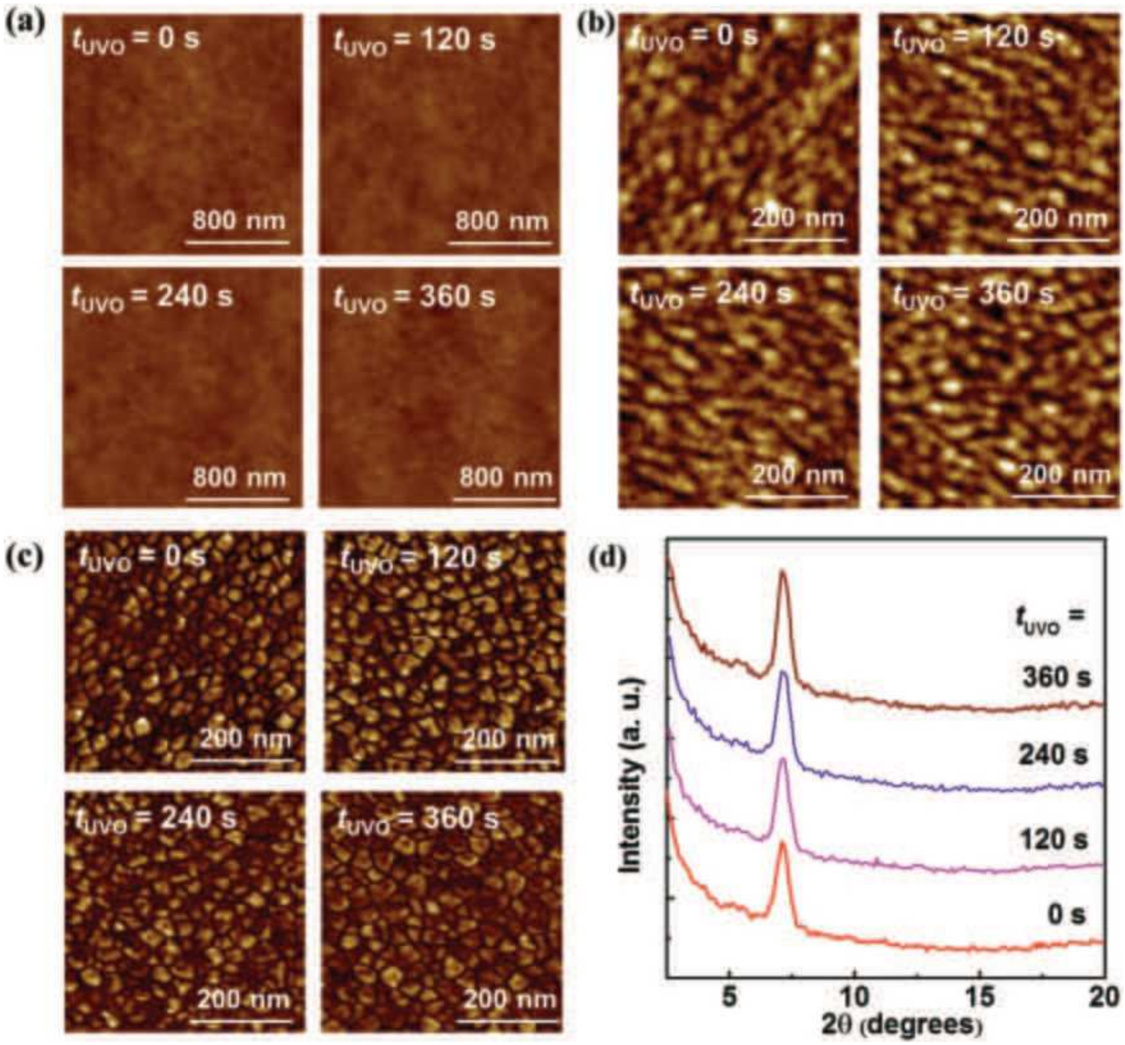

Figure 2. AFM images for the indicated t_{UVO} values of a) the PVA/PS gate dielectric films; b) CuPc (height image) and c) corresponding phase image films; d) GIXRD spectra of CuPc films grown on the PVA/PS gate dielectric as a function of t_{UVO}.

irrelevant to the enhanced sensitivities, which must therefore be chemical in nature.

X-ray photoelectron spectroscopy (XPS) was next utilized to analyze the surface chemical changes of the dielectric upon UVO treatment. As shown in **Figure 3**a and Figure S7 (Supporting Information), UVO introduces large densities of oxygen-containing species on the PS surface. In pristine PS/PVA films, there is no oxygen on the surface and carbon 1s peak fits well with only C—C/C—H components at 285.0 ± 0.1 eV and a C=C π–π* satellites at ≈292.5 eV. After UVO treatment, features corresponding to C—O, C=O, and O—C=O at 286.5 ± 0.1, 288.0 ± 0.1, and 289.5 ± 0.1 eV, respectively, can be identified.[44,45] Moreover, the density of the newly generated functional groups can be controlled by adjusting t_{UVO}, as shown in Table S2 (Supporting Information). To monitor the stability of the surface functional groups, the t_{UVO} = 360 s dielectric sample was aged in air for one month, and then analyzed by XPS. As shown in Figure S8 (Supporting Information), similar carbon content is obtained for the pristine and aged PS surfaces, with only a slightly decrease in C content (from 65.1% to 64.5%) and a small increase of O content (from 34.9% to 35.5%), indicating that UVO-generated functional groups on the surface are stable in air. The existence of new carbon-oxygen groups was also confirmed by Fourier transform infrared spectroscopy (FT-IR) on pristine and UVO-treated PS/PVA films. In Figure 3b and Figure S9 (Supporting Information), the vibrational peaks at ≈1452, ≈1492, and ≈1600 cm^{-1}, and multipeaks in the 2800–3110 cm^{-1} range from all dielectric films indicate the existence of the principle chemical bonds (C—C and C—H) revealing that UVO treatment only changes the surface chemical environment of the top PS film.[46–48] However, a new peak at ≈1728 cm^{-1} intensifies as the t_{UVO} increases, indicating the generation of C=O and/or O—C=O groups.[49] Moreover, the weak broad band at ≈3500 cm^{-1} for treated films implies that hydroxyl groups are produced, possibly originating from C—OH, and COOH functional groups as well as physisorbed water molecules.

PS is known to be an excellent gate dielectric material for both n-type and p-type organic semiconductor TFTs.[50] However, PS surface treatment with UVO is known to affect OSC

© 2017 WILEY-VCH Verlag GmbH & Co. KGaA, Weinheim

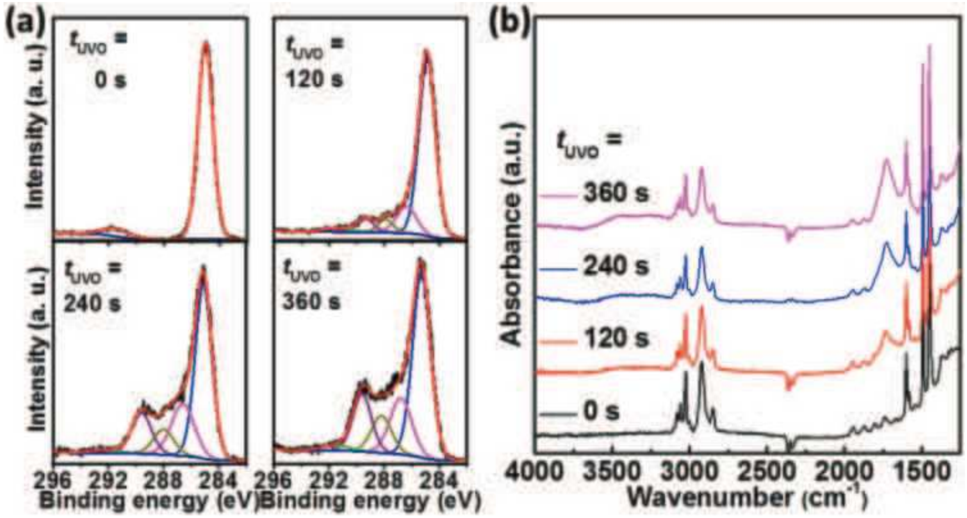

Figure 3. a) C 1s XPS and b) FT-IR of PVA/PS dielectric films as a function of t_{UVO}.

transport characteristics.[51] For the present PVA/PS-CuPc devices, the slightly reduced I_{ON} ($\approx 1.6 \times 10^{-6} \rightarrow \approx 3.2 \times 10^{-7}$ A) and μ ($\approx 3 \times 10^{-3} \rightarrow \approx 5 \times 10^{-4}$ cm^2 V^{-1} s^{-1}) upon UVO treatment ($t_{UVO} = 0 \rightarrow 360$ s) reveal that shallow traps are created by the UVO processing (Figure S1, Supporting Information, and Table 1). Furthermore, a slight shift of V_T in the positive direction is observed, however considering the nonlinearity of $(I_{ON})^{1/2}$ versus V_G, an accurate estimation of the trap density variation from V_T variation is not possible. When the devices are exposed to the analyte, the NO_2 molecules freely diffuse through the semiconductor grain boundaries and reach the PS/CuPc interface.[52–54] As described previously, these devices exhibit significant changes in *I*–*V* characteristics, with substantial increases of both I_{ON} and μ as well as a dramatic negative shift of V_T to positive values. The shifts in all of these parameters increase as [NO_2] increases (Table 2). Furthermore, plots of $(I_{ON})^{1/2}$ versus V_G show excellent linearity (**Figure 4**a and Figure S10, Supporting Information), enabling calculation of the trap density variation from ΔV_T utilizing the equation $\Delta N_{tr} = \frac{C_i \Delta V_T}{e}$.[55] Note, close inspection of the *I*–*V* characteristics points to a positively shifted V_T and an enlarged subthreshold slope (SS), which is due to a simultaneous detrapping and doping processes. This result is in agreement with charge transport studies on doped CuPc based OTFT.[56] Here, the approximate energy of the donor states, deduced from the TFT characteristics, results from a shallow and narrow doping state shifting the V_T but not affecting SS, and a second doping state which is distributed from the band edge to a binding energy of about 0.3 eV, leading to a broadened subthreshold region.[57] Thus, SS variation will not only be dominated by the detrapping process, which would decrease SS. However, both detrapping and/or hole doping processes will always shift V_T to the positive direction, thus it can be utilized to simulate the overall trap state variations.[56] Details of the state density variations are summarized in Table S3 (Supporting Information) and Figure 4b. As an example, the state density of the devices with $t_{UVO} = 0$ s varies by $\approx 15.3 \times 10^{10}$ cm^{-2} on going from the unexposed to the 30 ppm NO_2 exposed device, while those with $t_{UVO} = 360$ s exhibit a substantial density variations of 689.0×10^{10} cm^{-2} over the same analyte concentration range. Clearly, the higher the [NO_2], the more mobile holes are generated in the semiconductor channel.

A key question is how combined UVO surface activation and NO_2 chemistry enable the present sensing mechanism. As discussed above, the pristine PVA/PS dielectric has a low polarity surface covered with carbon–hydrogen bonds and should have negligible binding affinity for NO_2 molecules. As shown in Figure 1c and Figure S4 (Supporting Information), the devices without UVO treatment of the dielectric layer exhibit minimal changes in the *I*–*V* characteristics on NO_2 exposures as high as 30 ppm. This result indicates that although NO_2 is a strong oxidizing agent[58] and can efficiently penetrate the organic semiconductor, it is apparently unable to chemically oxidize (p-dope) the bulk of CuPc and when no NO_2 adsorption on the dielectric surface occurs, since this process alone would greatly enhance CuPc bulk conductivity and therefore I_{OFF} of all of the present OTFTs, including those without or with brief dielectric UVO exposure. On the other hand, upon UVO treatment, large densities of oxygenated polar functionalities are produced on the dielectric surface (Figure 3), which should efficiently adsorb polar molecules such as NO_2 via hydrogen bonding or van der Waals interactions.[59] Polarization effects would then induce positive charges in the semiconductors (Figure 4c). Furthermore, the polar UVO exposed surfaces may also have significant quantities of adsorbed water molecules, which would react with $NO_2 + O_2$ to produce nitric acid.[60] Highly acidic, strongly adsorbed HNO_3 can protonate/dope the first semiconductor monolayer yet is volatile enough to evaporate under low pressures to recover sensor function (vide infra).

To demonstrate the interfacial origin of sensing mechanism and corroborate that it is due to the UVO of the PS surface, we carried FT-IR experiments. Initial experiments, performed on the exact CuPc(semiconductor)/PS/PVA(dielectric) layers used in the TFT measurements (Figure S11, Supporting Information), were inconclusive since the IR peaks of the thick dielectric (C—C and C—H PS stretching modes)[61] overlap and are stronger than that possibly ascribable to NO_2 (≈ 1600 cm^{-1}).[62]

© 2017 WILEY-VCH Verlag GmbH & Co. KGaA, Weinheim

15214095, 2017, 31, Downloaded from https://advanced.onlinelibrary.wiley.com/doi/10.1002/adma.201701706 by Nanjing Tech University, Wiley Online Library on [31/01/2025]. See the Terms and Conditions (https://onlinelibrary.wiley.com/terms-and-conditions) on Wiley Online Library for rules of use; OA articles are governed by the applicable Creative Commons License

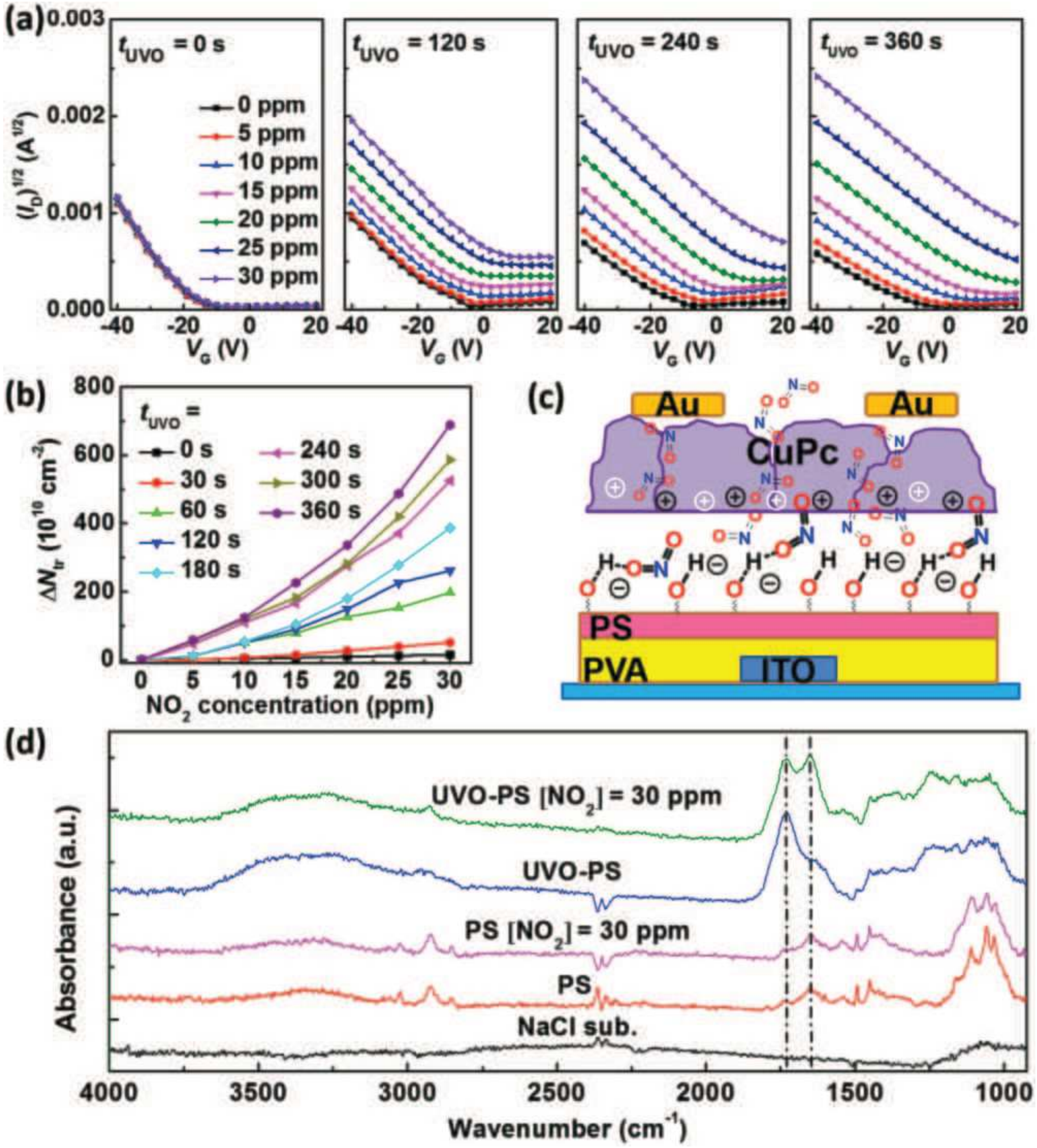

Figure 4. a) Square root of drain current versus gate voltage plots for different t_{UVO} values at the indicated NO_2 concentrations. b) Trap density as a function of NO_2 concentration for different t_{UVO}. c) Schematic representations of the NO_2 diffusion process through the semiconductor grain boundaries and mobile positive charge carrier formation by NO_2 adsorption to the UVO-treated PS surface (black positive charges) and field-effect (white positive charges). d) FT-IR of the indicated samples with t_{UVO} = 0 or 360 s and [NO_2] = 0 or 30 ppm (5 min).

However, these data support that no bulk penetration/adsorption of NO_2 into the dielectric nor chemical reactions with CuPc/PS/PVA occur. Thus, we utilized an alternative platform consisting of a thin PS film (≈10 nm) on IR-transparent NaCl to minimize noise and retain the dielectric surface characteristics. As shown in Figure 4d, PS on NaCl shows distinct vibrational peaks at ≈1452, ≈1492, ≈1600, and ≈2800–3110 cm^{-1}, in agreement with the literature.[48] After sample expose to 30 ppm NO_2 for 5 min, no newly generated peaks are detected. UVO-exposed PS samples (t_{UVO} = 360 s) reveal the formation of an intense

 © 2017 WILEY-VCH Verlag GmbH & Co. KGaA, Weinheim

15214095, 2017, 31, Downloaded from https://advanced.onlinelibrary.wiley.com/doi/10.1002/adma.201701706 by Nanjing Tech University, Wiley Online Library on [31/01/2025]. See the Terms and Conditions (https://onlinelibrary.wiley.com/terms-and-conditions) on Wiley Online Library for rules of use; OA articles are governed by the applicable Creative Commons License

15214095, 2017, 31, Downloaded from https://advanced.onlinelibrary.wiley.com/doi/10.1002/adma.201701706 by Nanjing Tech University, Wiley Online Library on [31/01/2025]. See the Terms and Conditions (https://onlinelibrary.wiley.com/terms-and-conditions) on Wiley Online Library for rules of use; OA articles are governed by the applicable Creative Commons License

peak at ≈1728 cm^{-1}, assigned to C═O functional groups,[49] as well as increased/broadening of the IR hydroxyl region (3000–3500 cm^{-1}). Importantly, the IR spectrum of the UVO-treated PS films exposed to 30 ppm NO_2 for 5 min exhibits a new strong peak at ≈1630 cm^{-1}, assignable to adsorbed NO_2. Note for PS films, the peak at ≈1650 cm^{-1} is expected for monosubstituted aromatic rings.[48] Thus, the combined IR experiments demonstrate that UVO generates new polar groups on the PS surface, NO_2 does not chemically react with the dielectric bulk/surface, and that our UVO-treated dielectric platform has the ability to physically adsorb NO_2.

Real-time sensitivities were next measured for devices biased at $V_D = V_G = -40$ V, responding to the dynamic switching to low NO_2 exposures (1, 2, 4, 6, 8, and 10 ppm, **Figure 5**a). The sensitivity at all the concentrations increases dramatically for devices with longer t_{UVO}'s. For example, the sensitivity at 6 ppm NO_2 increases from 10% (t_{UVO} = 0 s) to ≈700% (t_{UVO} = 120 s), ≈1800% (t_{UVO} = 240 s), and ≈3200% (t_{UVO} = 360 s). In fact, the sensitivity enhancement is so large that the response in devices without UVO-treated dielectric cannot be displayed (Figure 5a), and a separate plot with a much smaller *Y*-axis is required (inset of Figure 5a). Furthermore, for a low 1 ppm NO_2 concentration, the sensitivity for t_{UVO} = 0 s devices is only ≈4%, which can barely be integrated for practical applications. However, for devices with t_{UVO} = 360 s, the sensitivity for 1 ppm NO_2 increases to a remarkable ≈200%, which is distinguishable with any sample analyzer. Due to the limitation of the test equipment, 1 ppm is the lowest concentration of NO_2 that can be reliably utilized in these experiments. However, the estimated LOD for this platform can be calculated using Equation (2),[33,63] where η = 1.645 corresponds to the 90% confidence

$$Y_{LOD} = \frac{-a \times b + \sqrt{a^2 \times b^2 - \left(b^2 - \eta^2 \times \Delta b^2\right)\left(a^2 - \eta^2 \times \Delta a^2\right)}}{b^2 - \eta^2 \times \Delta b^2} \quad (2)$$

level, *a*, *b*, Δ*a*, and Δ*b* are the parameters extracted from the linear fit of Figure S12 (Supporting Information), based on sensitivities of the device with t_{UVO} = 120 s. This yields a LOD of 415 ppb. These sensitivities are among the highest reported to date for NO_2 sensors based on either resistor (*S* as high as 900% for 20 ppm NO_2, and 300% for 5 ppm NO_2) or TFT (*S* as high as 120% for 30 ppm NO_2, and 80% for 5 ppm NO_2) architectures.[5,30,64–67] Note that earlier studies demonstrated LOD for NO_2 lower than ≈53 ppb, the concentration which is considered safe by the U.S. Environmental Control Agency.[68–70] However, our current sensor platform can detect concentrations much lower than those which are of concern for "long-term exposure" (≈1 ppm).[5] Thus, our very simple TFT structure with a sensitivity of ≈400 ppb is more than sufficient to immediately alert that the environment is becoming unsafe and precautions should be taken. Furthermore, even lower LODs could be possible by, for instance, optimizing the dielectric surface

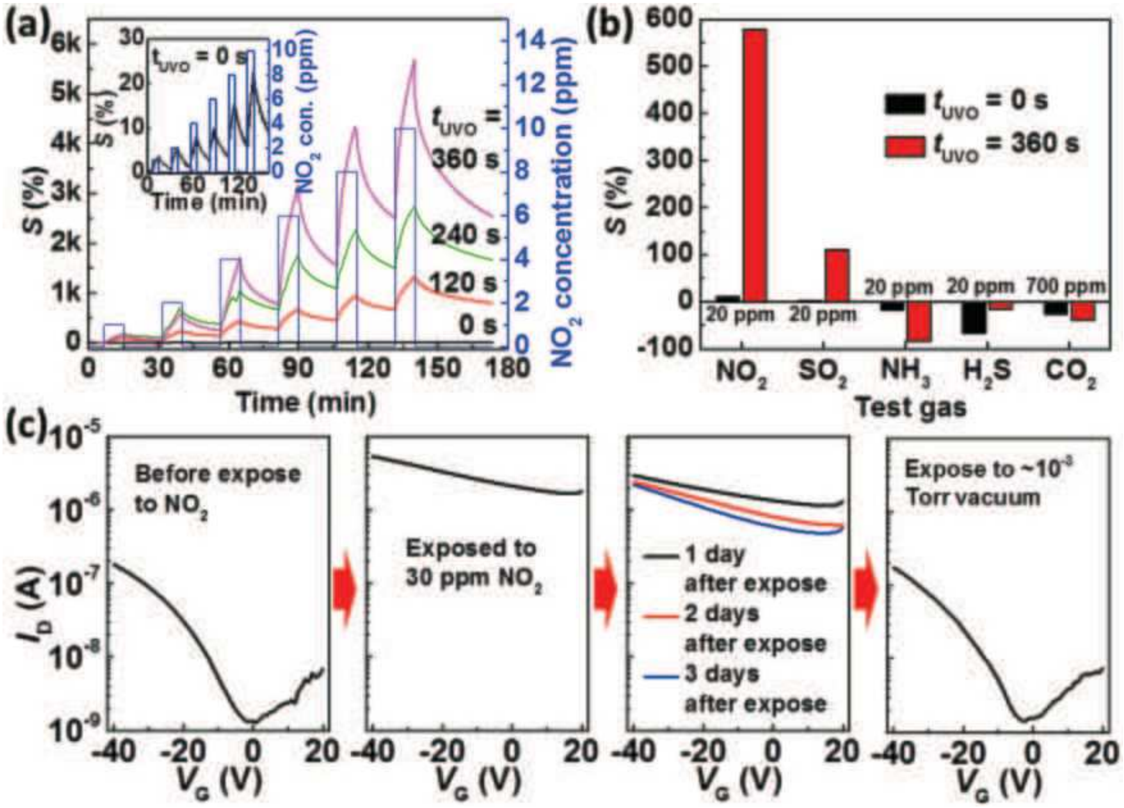

Figure 5. a) Real-time sensitivity ($V_D = V_G = -40$ V) responding to dynamic switching between NO_2 concentrations. b) Sensitivities of sensors with both t_{UVO} = 0 and 360 s for 20 ppm NO_2, SO_2, NH_3, H_2S, and 700 ppm CO_2. c) Transfer curves of a transistor with 360 s UVO-treated dielectric when tested under different environments.

© 2017 WILEY-VCH Verlag GmbH & Co. KGaA, Weinheim

chemistry using other polymers, vary the TFT channel lengths, and the organic semiconductor type/film morphology,[42] all approaches that will not enhances device complexity or costs.

For practical applications, selectivity is another important parameter, which was tested for the devices with both $t_{UVO} = 0$ and 360 s at concentrations of 20 ppm for NO_2, SO_2, NH_3, and H_2S and at a concentration of 700 ppm for CO_2 (since dry air is utilized as the carrier gas which itself contains ≈400 ppm CO_2, thus more CO_2 is mixed into dry air to have a higher concentration of CO_2). As shown in Figure 5b. it is clear that for the optimized platform (t_{UVO} = 360 s) all of the other gases are at least 6× less sensitive than NO_2. Thus, for NO_2 the sensitivity increased from 10% (t_{UVO} = 0 s) to almost 600% (t_{UVO} = 360 s) at a concentration of 20 ppm whereas for SO_2 (the most sensitive of the other gases) sensitivity increased from 3% (t_{UVO} = 0 s) to 110% (t_{UVO} = 360 s) for the same concentration. The TFT sensitivity for the other gases at the same concentration is far less and, equally important, the current variation is opposite compared to those of NO_2 and SO_2. Thus, the sensitivity for NH_3 increased from –17% (t_{UVO} = 0 s) to –84% (t_{UVO} = 360 s), while for H_2S, the sensitivity decreased from –66% (t_{UVO} = 0 s) to –16% (t_{UVO} = 360 s). For the apolar CO_2, even at the high concentration of 700 ppm, the sensor with/without UVO treatment both revealed a relatively low sensitivity of <38%. From Figure 5b, it can be concluded that our sensors are extremely sensitive to NO_2 compared to other four kinds of common gas pollutants. Moreover, this platform can distinguish between strongly oxidizing/polar (NO_2, SO_2) versus reducing/polar gases (NH_3 and H_2S) and weekly acidic/apolar (CO_2) one. These differences could be further optimized by properly optimizing t_{UVO}, fabricating platforms with TFT arrays with dielectric surfaces with different t_{UVO} (currently under investigation), and using other semiconducting materials.

Finally, note that after TFT exposure to NO_2, the recovery times of the *I*–*V* characteristics for the UVO-treated devices are relatively long. As shown in Figure 5c, when the UVO-treated TFT (t_{UVO} = 360 s) is exposed to 30 ppm NO_2 for 5 min, the subsequent transfer curve remains flat, not recovering the original shape after storage in air for 3 d. However, by placing the NO_2 exposed device in ≈10^{-3} Torr vacuum, the transfer curve quickly recovers to its original shape (Figure 5c), corroborating that NO_2 is likely physisorbed on the dielectric/semiconductor interface rather than irreversibly reacting. Moreover, when the device characteristics are recovered in vacuum, they can be reused (Figure S13, Supporting Information). Although the recovery of the UVO-treated transistors is not fast and complete (Figure 5c), it is possible to accelerate recovery by increasing the working temperature, typical of other OTFT gas sensors.[68] That physisorption is stable under the present conditions indicates that the current platform can be utilized as an integrator to record maximum NO_2 exposure.[24] Coupled with a proper alarm, such a device could be used to signal when dangerous NO_2 levels are reached.

In summary, OTFT-based NO_2 sensors with ultrahigh sensitivities and good selectivities have been realized via UVO treatment of the gate dielectric surface. UVO generates oxygen-containing functional groups capable of strongly interacting with NO_2, which underlies the sensing mechanism. The NO_2 device sensitivity approaches ≈200% and ≈160 000% for NO_2 concentrations of 1 and 30 ppm, respectively. This simple processing addresses the low sensitivity and high LOD of conventional gas sensors based on OTFTs. Thus, we believe that the present strategy, together with the advantages of simple OTFT fabrication, opens new opportunities to realize high-performance, low-cost, portable electronic noses.

Experimental Section

Transistor Fabrication: PVA (M_w = 146 000–186 000, 99+% hydrolyzed, Sigma-Aldrich) was dissolved in DI water with a concentration of 4 wt%. PS (average M_w ≈ 280 000, Sigma-Aldrich) was dissolved in xylene with a concentration of 3 wt%. Indium tin oxide[48] glass substrate was cleaned sequentially in an ultrasonic bath with acetone, deionized water, and isopropyl alcohol for 15 min each. PVA was then spin-coated at 3000 rpm for 1 min, and annealed at 70 °C for 1 h on a hot plate. PS was then spin-coated at 3000 rpm for 1 min. After dielectric layer coating, the substrates were baked at 120 °C for 1 h to completely remove residual solvents. Prior to the deposition of organic semiconductors, the dielectrics were exposed to UV light of 185 and 253.7 nm (UVO-Cleaner 42, Jelight Company Inc.) for 0–360 s. 10 nm CuPc was then evaporated under 4×10^{-6} Torr at a rate of 0.2–0.3 Å s^{-1}. The transistors were completed by depositing the source and drain electrodes of 40 nm gold via thermal evaporation using a metal shadow mask under 4×10^{-6} Torr at a rate of 0.5–1 Å s^{-1}.

Device Characterization and Sensor Evaluation: TFT characterization was performed in air on a custom probe station using an Agilent 1500 semiconductor parameter analyzer. The electron mobility (μ) was calculated in the saturation region using Equation (3)

$$I_D = \frac{WC_i}{2L} \mu (V_G - V_T)^2 \quad (3)$$

where C_i is the capacitance per unit area of dielectric layer, and W and L are channel width and length, respectively. For sensing tests, the OTFT sensor was stored in an airtight test chamber (≈2.4 mL). A mixture of dry air and certain gas analyte (50 ppm standard NO_2 gas, 50 ppm standard SO_2 gas, 50 ppm standard NH_3 gas, 50 ppm standard H_2S gas, or 1000 ppm standard CO_2 gas) in appropriate concentrations was introduced into the test chamber by mass flow controllers. The flow rate in the test was fixed at 100 sccm (standard cm^3 min^{-1}).

Film Characterization: AFM film topographies were imaged with a Veeco Dimension Icon scanning Probe Microscope using tapping mode. GIXRD measurements were carried out on a Rigaku SmartLab workstation (CuKα, λ = 1.542 Å) with an incident angle (α = 0.5°). XPS was performed on Thermo Scientific ESCALAB 250Xi at a base pressure of 4.5×10^{-10} mbar (UHV). FT-IR spectra were collected by Nexus 870 spectrometer (Thermo Nicolet) with a single reflection horizontal ATR accessory having a diamond ATR crystal fixed at incident angle of 45°.

Supporting Information

Supporting Information is available from the Wiley Online Library or from the author.

Acknowledgements

The authors thank AFOSR (FA9550-15-1-0044), the Northwestern University MRSEC (NSF DMR-1121262), Polyera Corp., the Foundation for Innovation Research Groups of the NSFC (Grant No. 61421002), the National Science Foundation of China (NSFC) (Grant No. 61675041), and the Project of Science and Technology of Sichuan Province (Grant No. 2016FZ0100) for support of this research. F.S.M. was supported

© 2017 WILEY-VCH Verlag GmbH & Co. KGaA, Weinheim

15214095, 2017, 31, Downloaded from https://advanced.onlinelibrary.wiley.com/doi/10.1002/adma.201701706 by Nanjing Tech University, Wiley Online Library on [31/01/2025]. See the Terms and Conditions (https://onlinelibrary.wiley.com/terms-and-conditions) on Wiley Online Library for rules of use; OA articles are governed by the applicable Creative Commons License

15214095, 2017, 31, Downloaded from https://advanced.onlinelibrary.wiley.com/doi/10.1002/adma.201701706 by Nanjing Tech University, Wiley Online Library on [31/01/2025]. See the Terms and Conditions (https://onlinelibrary.wiley.com/terms-and-conditions) on Wiley Online Library for rules of use; OA articles are governed by the applicable Creative Commons License

by Award No. 70NANB14H012 from U.S. Department of Commerce, National Institute of Standards and Technology as part of the Center for Hierarchical Materials Design (CHiMaD). This work made use of the J.B. Cohen X-Ray Diffraction Facility, EPIC facility, Keck-II facility, and SPID facility of the NUANCE Center at Northwestern University, which received support from the MRSEC program (NSF DMR-1121262); the International Institute for Nanotechnology (IIN); the Keck Foundation; and the State of Illinois. W.H. and B.H.W. thank the joint-Ph.D. program supported by China Scholarship Council for fellowships. A.F. thanks the Shenzhen Peacock Plan project (KQTD20140630110339343).

Conflict of Interest

The authors declare no conflict of interest.

Keywords

interface trap, nitrogen dioxide sensors, organic thin-film transistors, UV–ozone

Received: March 27, 2017
Published online: June 14, 2017

[1] P. Nejat, F. Jomehzadeh, M. M. Taheri, M. Gohari, M. Z. Abd Majid, *Renewable Sustainable Energy Rev.* **2015**, *43*, 843.
[2] Y. X. He, Y. Xu, Y. X. Pang, H. Y. Tian, R. Wu, *Renewable Energy* **2016**, *89*, 695.
[3] Y. Guo, G. Yu, Y. Liu, *Adv. Mater.* **2010**, *22*, 4427.
[4] G. B. Hamra, F. Laden, A. J. Cohen, O. Raaschou-Nielsen, M. Brauer, D. Loomis, *Environ. Health Perspect.* **2015**, *123*, 1107.
[5] A. Das, R. Dost, T. Richardson, M. Grell, J. J. Morrison, M. L. Turner, *Adv. Mater.* **2007**, *19*, 4018.
[6] T. W. Hesterberg, W. B. Bunn, R. O. McClellan, A. K. Hamade, C. M. Long, P. A. Valberg, *Crit. Rev. Toxicol.* **2009**, *39*, 743.
[7] R. Pohanish, *Sittig's Handbook of Toxic and Hazardous Chemicals and Carcinogens*, 6th ed., William Andrew, New York, **2011**.
[8] A.-M. Andringa, C. Piliego, I. Katsouras, P. W. M. Blom, D. M. d. Leeuw, *Chem. Mater.* **2014**, *26*, 773.
[9] Y. Zang, D. Huang, C. A. Di, D. Zhu, *Adv. Mater.* **2016**, *28*, 4549.
[10] A. Luzio, F. G. Ferré, F. D. Fonzo, M. Caironi, *Adv. Funct. Mater.* **2014**, *24*, 1790.
[11] K.-J. Baeg, M. Caironi, Y.-Y. Noh, *Adv. Mater.* **2013**, *25*, 4210.
[12] M. J. Sung, A. Luzio, W.-T. Park, R. Kim, E. Gann, F. Maddalena, G. Pace, Y. Xu, D. Natali, C. de Falco, L. Dang, C. R. McNeill, M. Caironi, Y.-Y. Noh, Y.-H. Kim, *Adv. Funct. Mater.* **2016**, *26*, 4984.
[13] C. Zhang, P. Chen, W. Hu, *Chem. Soc. Rev.* **2015**, *44*, 2087.
[14] S. J. Han, W. Huang, W. Shi, J. S. Yu, *Sens. Actuators, B* **2014**, *203*, 9.
[15] S. J. Han, X. M. Zhuang, W. Shi, X. Yang, L. Li, J. S. Yu, *Sens. Actuators, B* **2016**, *225*, 10.
[16] K. H. Cheon, J. Cho, Y. H. Kim, D. S. Chung, *ACS Appl. Mater. Interfaces* **2015**, *7*, 14004.
[17] M. Magliulo, A. Mallardi, R. Gristina, F. Ridi, L. Sabbatini, N. Cioffi, G. Palazzo, L. Torsi, *Anal. Chem.* **2013**, *85*, 3849.
[18] W. G. Huang, J. Sinha, M. L. Yeh, J. F. M. Hardigree, R. LeCover, K. Besar, A. M. Rule, P. N. Breysse, H. E. Katz, *Adv. Funct. Mater.* **2013**, *23*, 4094.
[19] W. Shi, X. E. Yu, Y. F. Zheng, J. S. Yu, *Sens. Actuators, B* **2016**, *222*, 1003.
[20] H. Zhang, X. Guo, J. Hui, S. Hu, W. Xu, D. Zhu, *Nano Lett.* **2011**, *11*, 4939.
[21] M. Magliulo, K. Manoli, E. Macchia, G. Palazzo, L. Torsi, *Adv. Mater.* **2015**, *27*, 7528.
[22] T. Shaymurat, Q. Tang, Y. Tong, L. Dong, Y. Liu, *Adv. Mater.* **2013**, *25*, 2269.
[23] Y. Zang, F. Zhang, D. Huang, C. A. Di, Q. Meng, X. Gao, D. Zhu, *Adv. Mater.* **2014**, *26*, 2862.
[24] W. G. Huang, K. Besar, R. LeCover, A. M. Rule, P. N. Breysse, H. E. Katz, *J. Am. Chem. Soc.* **2012**, *134*, 14650.
[25] W. G. Huang, K. Besar, R. LeCover, P. Dulloor, J. Sinha, J. F. M. Hardigree, C. Pick, J. Swavola, A. D. Everett, J. Frechette, M. Bevan, H. E. Katz, *Chem. Sci.* **2014**, *5*, 416.
[26] F. X. Werkmeister, T. Koide, B. A. Nickel, *J. Mater. Chem. B* **2016**, *4*, 162.
[27] K. C. See, A. Becknell, J. Miragliotta, H. E. Katz, *Adv. Mater.* **2007**, *19*, 3322.
[28] M. Mirza, J. Wang, D. Li, S. A. Arabi, C. Jiang, *ACS Appl. Mater. Interfaces* **2014**, *6*, 5679.
[29] L. Li, P. Gao, M. Baumgarten, K. Mullen, N. Lu, H. Fuchs, L. Chi, *Adv. Mater.* **2013**, *25*, 3419.
[30] H. D. Fan, W. Shi, X. G. Yu, J. S. Yu, *Synth. Met.* **2016**, *211*, 161.
[31] M. W. G. Hoffmann, J. D. Prades, L. Mayrhofer, F. Hernandez-Ramirez, T. T. Järvi, M. Moseler, A. Waag, H. Shen, *Adv. Funct. Mater.* **2014**, *24*, 595.
[32] D. Khim, G. S. Ryu, W. T. Park, H. Kim, M. Lee, Y. Y. Noh, *Adv. Mater.* **2016**, *28*, 2752.
[33] M. Mirza, J. W. Wang, L. Wang, J. He, C. Jiang, *Org. Electron.* **2015**, *24*, 96.
[34] A. Klug, M. Denk, T. Bauer, M. Sandholzer, U. Scherf, C. Slugovc, E. J. W. List, *Org. Electron.* **2013**, *14*, 500.
[35] J. Zhang, X. Liu, G. Neri, N. Pinna, *Adv. Mater.* **2016**, *28*, 795.
[36] J. R. Vig, J. Vac, *Sci. Technol., A* **1985**, *3*, 1027.
[37] W. Huang, X. G. Yu, H. D. Fan, J. S. Yu, *Appl. Phys. Lett.* **2014**, *105*, 093302.
[38] S. J. Han, J. H. Kim, J. W. Kim, C. K. Min, S. H. Hong, D. H. Kim, K. H. Baek, G. H. Kim, L. M. Do, Y. Park, *J. Appl. Phys.* **2008**, *104*, 013715.
[39] A. Wang, I. Kymissis, V. Bulovic, A. I. Akinwande, *Appl. Phys. Lett.* **2006**, *89*, 112109.
[40] Y. S. Rim, S. H. Bae, H. Chen, N. De Marco, Y. Yang, *Adv. Mater.* **2016**, *28*, 4415.
[41] W. Huang, J. S. Yu, X. G. Yu, W. Shi, *Org. Electron.* **2013**, *14*, 3453.
[42] D. Duarte, A. Dodabalapur, *J. Appl. Phys.* **2012**, *111*, 044509.
[43] A. C. Cruickshank, C. J. Dotzler, S. Din, S. Heutz, M. F. Toney, M. P. Ryan, *J. Am. Chem. Soc.* **2012**, *134*, 14302.
[44] D. O. H. Teare, C. Ton-That, R. H. Bradley, *Surf. Interface Anal.* **2000**, *29*, 276.
[45] M. M. Browne, G. V. Lubarsky, M. R. Davidson, R. H. Bradley, *Surf. Sci.* **2004**, *553*, 155.
[46] Y. Konuklu, M. Ostry, H. O. Paksoy, P. Charvat, *Energy Build.* **2015**, *106*, 134.
[47] E. Biazar, R. Zeinali, N. Montazeri, K. Pourshamsian, M. J. Behrouz, A. Asefnejad, A. Khoshzaban, G. Shahhosseini, M. S. Najafabadi, R. Abyani, H. Jamalzadeh, M. Fouladi, S. R. Hagh, A. S. Khamaneh, S. Kabiri, S. H. Keshel, A. Mansourkiaei, *Int. J. Nanomed.* **2010**, *5*, 549.
[48] D. Olmos, E. V. Martin, J. Gonzalez-Benito, *Phys. Chem. Chem. Phys.* **2014**, *16*, 24339.
[49] A. N. Yusilawati, M. Maizirwan, I. Sopyan, M. S. Hamzah, K. H. Ng, C. S. Wong, *Adv. Mater. Res.* **2011**, *264*, 1532.
[50] M. H. Yoon, C. Kim, A. Facchetti, T. J. Marks, *J. Am. Chem. Soc.* **2006**, *128*, 12851.
[51] N. V. Subbarao, M. Gedda, P. K. Iyer, D. K. Goswami, *ACS Appl. Mater. Interfaces* **2015**, *7*, 1915.
[52] X. Li, Y. D. Jiang, G. Z. Xie, H. L. Tai, P. Sun, B. Zhang, *Sens. Actuators, B* **2013**, *176*, 1191.
[53] J. H. Park, J. E. Royer, E. Chagarov, T. Kaufman-Osborn, M. Edmonds, T. Kent, S. Lee, W. C. Trogler, A. C. Kummel, *J. Am. Chem. Soc.* **2013**, *135*, 14600.

 © 2017 WILEY-VCH Verlag GmbH & Co. KGaA, Weinheim

[54] J. H. Park, S. Lee, A. C. Kummel, *J. Vac. Sci. Technol., B* **2015**, *33*, 030604.
[55] V. Podzorov, E. Menard, A. Borissov, V. Kiryukhin, J. A. Rogers, M. E. Gershenson, *Phys. Rev. Lett.* **2004**, *93*, 086602.
[56] J. Park, J. E. Royer, C. N. Colesniuc, F. I. Bohrer, A. Sharoni, S. Jin, I. K. Schuller, W. C. Trogler, A. C. Kummel, *J. Appl. Phys.* **2009**, *106*, 034505.
[57] R. A. Street, M. L. Chabinyc, F. Endicott, *Phys. Rev. B* **2007**, *76*, 045208.
[58] A. M. Andringa, W. S. C. Roelofs, M. Sommer, M. Thelakkat, M. Kemerink, D. M. de Leeuw, *Appl. Phys. Lett.* **2012**, *101*, 153302.
[59] J. Y. Dai, J. M. Yuan, *Chem. Phys.* **2012**, *405*, 161.
[60] G. Murdachaew, M. E. Varner, L. F. Phillips, B. J. Finlayson-Pitts, R. B. Gerber, *Phys. Chem. Chem. Phys.* **2013**, *15*, 204.
[61] L. Harris, G. W. King, *J. Chem. Phys.* **1934**, *2*, 51.
[62] E. Flores, J. Viallon, P. Moussay, F. Idrees, R. I. Wielgosz, *Anal. Chem.* **2012**, *84*, 10283.
[63] H. Zheng, B. Ramalingam, V. Korampally, S. Gangopadhyay, *Appl. Phys. Lett.* **2013**, *103*, 193305.
[64] W. Yuan, L. Huang, Q. Zhou, G. Shi, *ACS Appl. Mater. Interfaces* **2014**, *6*, 17003.
[65] C. J. Qiu, Y. W. Dou, Q. L. Zhao, W. Qu, J. Yuan, Y. M. Sun, M. S. Cao, *Chin. Phys. Lett.* **2008**, *25*, 3590.
[66] N. Iqbal, A. Afzal, N. Cioffi, L. Sabbatini, L. Torsi, *Sens. Actuators, B* **2013**, *181*, 9.
[67] S. Ji, H. Wang, T. Wang, D. Yan, *Adv. Mater.* **2013**, *25*, 1755.
[68] A. M. Andringa, N. Vlietstra, E. C. P. Smits, M. J. Spijkman, H. L. Gomes, J. H. Klootwijk, P. W. M. Blom, D. M. de Leeuw, *Sens. Actuators, B* **2012**, *171*, 1172.
[69] A. Oprea, U. Weimar, E. Simon, M. Fleischer, H. P. Frerichs, C. Wilbertz, M. Lehmann, *Sens. Actuators, B* **2006**, *118*, 249.
[70] J. Li, Y. J. Lu, Q. Ye, M. Cinke, J. Han, M. Meyyappan, *Nano Lett.* **2003**, *3*, 929.

 © 2017 WILEY-VCH Verlag GmbH & Co. KGaA, Weinheim

15214095, 2017, 31, Downloaded from https://advanced.onlinelibrary.wiley.com/doi/10.1002/adma.201701706 by Nanjing Tech University, Wiley Online Library on [31/01/2025]. See the Terms and Conditions (https://onlinelibrary.wiley.com/terms-and-conditions) on Wiley Online Library for rules of use; OA articles are governed by the applicable Creative Commons License

ACS SENSORS

pubs.acs.org/acssensors Review

Mitigation of Humidity Interference in Colorimetric Sensing of Gases

Jingjing Yu,[#] Di Wang,[#] Vishal Varun Tipparaju, Francis Tsow, and Xiaojun Xian*

Cite This: *ACS Sens.* 2021, 6, 303−320 Read Online

ACCESS | Metrics & More | Article Recommendations

ABSTRACT: Colorimetric sensing technologies have been widely used for both quantitative detection of specific analyte and recognition of a large set of analytes in gas phase, ranging from environmental chemicals to biomarkers in breath. However, the accuracy and reliability of the colorimetric gas sensors are threatened by the humidity interference in different application scenarios. Though substantial progress has been made toward new colorimetric sensors development, unless the humidity interference is well addressed, the colorimetric sensors cannot be deployed for real-world applications. Although there are comprehensive and insightful review articles about the colorimetric gas sensors, they have focused more on the progress in new sensing materials, new sensing systems, and new applications. There is a need for reviewing the works that have been done to solve the humidity issue, a challenge that the colorimetric gas sensors commonly face. In this review paper, we analyzed the mechanisms of the humidity interference and discussed the approaches that have been reported to mitigate the humidity interference in colorimetric sensing of environmental gases and breath biomarkers. Finally, the future perspectives of colorimetric sensing technologies are also discussed.

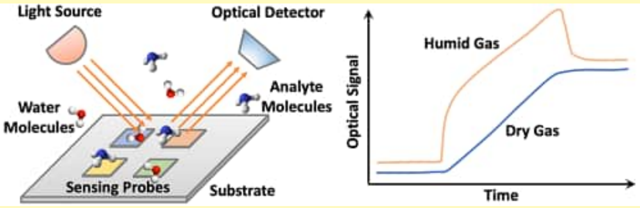

KEYWORDS: *colorimetric sensor, humidity interference, gas sensor, environmental sensor, colorimetric sensor array, hydrophobicity, volatile organic compound, air pollutant, breath biomarker*

As one of the most important chemical sensing techniques, colorimetric sensing has many unique advantages, such as high selectivity, multiplexed sensing capability, high compatibility with digital imaging systems, and low cost. Colorimetric sensing refers to the techniques of detecting the presence and concentration of an analyte via a chemical reaction that accompanies color change. Colorimetric sensors have been widely used in analyte detection in both liquid phase and gas phase.[1−4] The most successful applications include pH paper, pregnancy test strips, and gas detector tubes. Substantial progress has been made to advance colorimetric sensing technologies for broader applications in gas phase analyte sensing, ranging from monitoring chemicals in the ambient air to detecting biomarkers in human breath.[1,4−10] The colorimetric sensing technologies can be used for both quantitative detection of specific analyte and recognition and classification of a large set of analytes. Although there are comprehensive and insightful review articles in the field of colorimetric gas sensors,[1,5,7] they have focused more on the progress in new sensing materials, new sensing systems, and new applications. There is a need for reviewing the challenges that the colorimetric gas sensors have commonly faced, which set barriers for this technology from solving real-world problems. In this review paper, we are dedicated to one of the universal challenges to most colorimetric gas sensors, the humidity interference.

No matter whether in the ambient environment or in human breath, water vapor is everywhere. Humidity refers to the amount of water vapor in the air. It can be expressed as absolute humidity and relative humidity (RH). Absolute humidity describes the concentration of water vapor in gas regardless of temperature, normally in grams per cubic meter. The relative humidity is defined as the ratio of the water vapor to saturated water vapor at a given temperature, typically in percentage. The concentration of water vapor in the atmosphere ranges from ~10 ppm in the coldest regions of the Earth's atmosphere up to 5% by volume in hot, humid air masses.[11] In human breath, the relative humidity is considered to be 100%, meaning the water vapor is saturated at breath temperature.[12,13] Considering that the concentrations of most of the analytes of interest in ambient air or human breath are in the ppm or ppb level, the concentration of vapor is significantly higher than that of the target analytes. This presents a huge challenge to most gas sensors, ranging from semiconductor metal oxide sensors,[14,15] electrochemical sensors,[16,17] solid-state sensors (resistive, capacitive, amperometric, and potentiometric),[18−20] optical sensors,[21] and mass-based sensors,[22,23] to the colorimetric sensors[24,25] for environmental gas[26] or breath biomarker detection.[27]

Special Issue: Commemorating NJ Tao

Received: August 7, 2020
Accepted: October 9, 2020
Published: October 21, 2020

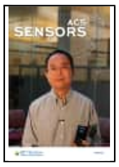

Downloaded via NANJING TECH UNIV on February 1, 2025 at 06:37:10 (UTC).
See https://pubs.acs.org/sharingguidelines for options on how to legitimately share published articles.

© 2020 American Chemical Society

https://dx.doi.org/10.1021/acssensors.0c01644
ACS Sens. 2021, 6, 303−320

This review paper is intended to summarize the approaches that have been used to mitigate the humidity interference in colorimetric sensing of gaseous analytes. Different kinds of colorimetric sensing platforms for gas detection are covered by this review, such as the "lock-and-key" colorimetric sensors and the colorimetric sensing arrays. However, gas detector tubes will not be included in this review since the detector tube technology is very mature and has been widely used in different applications for decades.[28,29] The humidity interference also exists in gas detector tubes, but it has been well addressed by using humidity correction or desiccation.[30−33] The patents related to colorimetric sensing of gases are not included in this review paper.

Because of the intrinsic differences, we divide the humidity mitigating approaches into two groups: one for environmental gas detection and the other for breath biomarker detection. The major difference between these two groups is the humidity level. The environmental humidity is subject to changes in ambient condition, in the range of 10−100% RH, while the humidity level in the breath is saturated at 100% RH. The challenges of mitigating humidity interference under these two conditions are different: one needs to deal with the humidity variation in environmental gas sensing while dealing with water condensation in breath biomarker sensing.

■ COLORIMETRIC GAS SENSING PLATFORMS

To better understand the mechanism of humidity interference, we need to review the colorimetric sensing principle and sensing platforms briefly. Colorimetry is a conventional method in analytical chemistry. It measures the analytes in the sample through chemical reactions that are accompanied by color change. As for the detection of analytes in gas phase, typically, a gas−solid-phase reaction model can be applied. Typically, the sensing probes are incorporated in a solid substrate, such as plate, membrane, or film, and the analytes in the gas phase are actively delivered or passively diffused into a reaction chamber, where they encounter and react with the sensing probes. The following processes are involved in the gas−solid-phase reaction: (1) adsorption of analyte molecules on the surface of the substrate; (2) diffusion of analyte molecules within the matrix of the substrate; and (3) chemical reaction between gas analyte with sensing probes causing color change on the substrate. Generally speaking, the higher the analyte concentration in the gas phase, the faster the color development rate; the higher the gas flow rate, the faster the color development.

Similar to the biosystems, there are two kinds of colorimetric sensing platforms: (1) the lock-and-key sensing platform, where the sensing probe is highly selective to a specific analyte, e.g., the antibody−antigen interaction; (2) the colorimetric sensor array, where the sensing probes in the array cross-react with a large set of analytes, such as the mammalian olfactory system. As for the lock-and-key sensing platform, the concentration of the analyte can be quantified by the optical signal,[24,34,35] while in the colorimetric sensor array, a large set of analytes can be classified through pattern recognition based on the high dimensional data.[8,36,37]

"Lock-and-Key" Colorimetric Sensor. The "lock-and-key" type of colorimetric sensor uses specific sensing probes as the receptors to react with specific analytes, similar to the relationship between a lock and a key. Cross-sensitivity should be avoided in this type of sensor for the sake of high selectivity and accuracy. This is fundamentally different from the colorimetric sensor array, which takes advantage of the cross-sensitivity.

An optical detection system is needed to monitor the color change, as shown in Figure 1. It consists of a light source, a

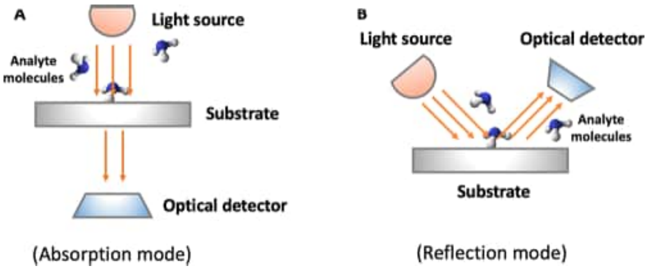

Figure 1. Schematic of the absorption mode and reflection mode in colorimetric sensing.

substrate, and an optical detector. The optical signals that can be used for analyte quantification typically include transmission (or absorbance) and reflectance. In the absorbance mode, the substrate locates in between the light source and the optical detector. The color change on the substrate during analyte exposure causes the optical absorbance change, which is monitored by the optical detector. As for the reflection mode, the light source and the optical detector locate on the same side of the substrate. Similarly, the optical reflection change due to the color change on the substrate is monitored by the optical detector. To quantify the gas sample introduced in the detection system, either the flow rate or the volume of the gas will be controlled.

To quantify the analyte concentration, information on both the kinetics and the thermodynamics can be used. The kinetics information refers to the color development rate, and the thermodynamics information refers to the finalized color development when the chemical reaction reaches equilibrium. Typically, the real-time absorbance or reflection signal will be monitored in kinetics-based colorimetric sensing. Figure 2 shows how to quantify the concentration in a "lock-and-key" colorimetric carbon monoxide (CO) sensor.[24] The slope of the absorbance change during CO injection was calculated to determine the CO concentration. As for the thermodynamics-based sensing, the absorbance or reflection signal before and after analyte exposure will be measured and compared.[38]

Colorimetric Sensor Array. Unlike a "lock-and-key" colorimetric sensor, which leverages the colorimetric chemical reaction between analytes and sensing probes with high specificity, a colorimetric sensor array (or optoelectronic nose) takes advantage of the cross-sensitivity between a large set of analytes and sensor arrays through nonspecific interactions, mimicking the mammalian olfactory system.[1,39] These nonspecific interactions include both physical adsorptions and chemical reactions, ranging from van der Waals interactions, dipole−dipole interactions, hydrogen bonding interactions, salt bridges interactions, $\pi-\pi$ stacking complexation, Bronsted acid−base interactions, and donor−acceptor interactions, to ion−ion interactions.[1] The colorimetric array is typically prepared by printing cross-reactive and hydrophobic dyes, such as Brønsted acidic or basic dyes, Lewis acid/base dyes, redox dyes, colorants with large permanent dipoles, and chromogenic aggregative materials onto hydrophobic membranes.[1] In the colorimetric sensor array, the color map created by the entire sensor arrays rather than the response from each

https://dx.doi.org/10.1021/acssensors.0c01644
ACS Sens. 2021, 6, 303−320

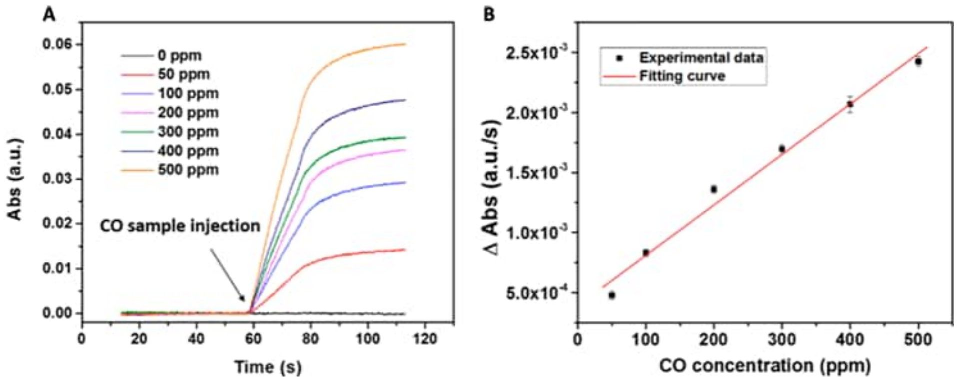

Figure 2. Quantification of analyte concentration in a "lock-and-key" colorimetric CO sensor. (A) Optical responses of colorimetric CO sensor to different concentrations (0, 50, 100, 200, 300, 400, and 500 ppm) of CO gas. (B) Calibration of sensor response to CO. The slope of the absorbance during CO injection increases linearly with the CO concentration. Reproduced with permission from ref 24. Copyright 2018 American Chemical Society.

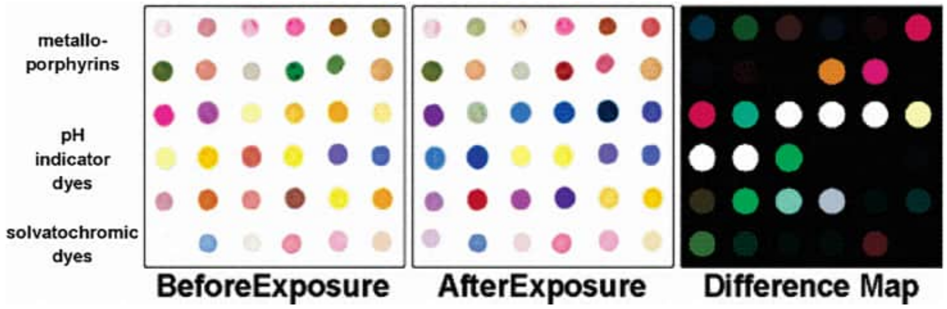

Figure 3. Image of the 36-dye colorimetric sensor array before exposure (left) and after exposure to decylamine (middle) after equilibration at full vapor pressure at 295 K. A subtraction of the two images yields a difference vector. This vector is usefully visualized using a difference map (right). Adapted with permission from ref 40. Copyright 2006 American Chemical Society.

sensor carries the chemical information on the analytes, like the fingerprint. To obtain this chemical fingerprint, the images of the array before and after analyte exposure are captured by digital imaging devices, such as the flatbed scanners, digital cameras, contact image sensors, and cell phones,[1] from which the color difference map of the sensor array can be quantified and visualized for analysis, as shown in Figure 3.[40]

The major data analysis methods for the colorimetric sensor array are pattern recognition methods, which can be categorized into (1) descriptive methods, including hierarchical cluster analysis (HCA) and principal component analysis (PCA); and (2) classification methods, including linear discriminant analysis (LDA), support vector machines (SVMs), and artificial neural networks (ANNs).[1] Figure 4A,B shows the results of HCA and PCA methods for toxic industrial chemicals (TICs)[41] and volatile organic compounds (VOCs)[40] analysis. Though in most of the cases, these data analysis methods are used for the classification of a set of analytes, sometimes they can also be used for quantification of the analyte concentrations,[25,42] as shown in Figure 4C.

■ MECHANISMS OF HUMIDITY INTERFERENCE

Humidity can interfere with the colorimetric sensing through either the physical interaction or the chemical interaction, as shown in Figure 5. Physically, water molecules can be adsorbed onto the surface of the sensing probes, the substrates, or the optical components, resulting in physical changes in the sensing system.

Silica plate is widely used as the substrate for fabricating colorimetric sensors. Humidity can interfere with the colorimetric sensing by changing the transmittance of the silica plate, and the RH-induced absorbance change is in the VIS-NIR (visible−near-infrared) region (Figure 6).[43] This type of humidity effect is reversible since it is only a physical adsorption−desorption process.

As illustrated in Figure 5A, the thickness of the water layer on the sensing substrate may change with humidity, especially for the sol−gel matrix. Usually, the thickness of the water layer increases along with increase in the relative humidity, leading to more gas analytes dissolving and sensor signal increasing.[44]

Humidity can cause swelling of the hydrogels (Figure 5B), a commonly used polymer matrix in colorimetric sensing systems, and introduce false signals to the transducers, such as the shift of the diffraction wavelength.[45]

Chemical changes due to humidity can be much more complicated. The penetration of water vapor into the hydrophobic plastic film in a solid-state colorimetric sensor could possibly cause some degree of plasticization action in the matrix, resulting in an increase of sensitivity.[46] In some colorimetric sensing systems, water is able to catalyze the chemical reaction.[24] For some colorimetric sensors, water molecules take part in the chemical reaction, and the environmental humidity could play a subtle role in affecting the sensor performance. Li et al. found that the Pd(II)-silica porous microsphere sensor-based cumulative colorimetric sensor array was actually independent of the humidity change over a wide range, from 10% to 90%, as shown in Figure 7.[25] They concluded that since water was a necessary component in the chemical reaction of Pd(II) reduction, the concentration of water vapor in 10−90% RH was far more than

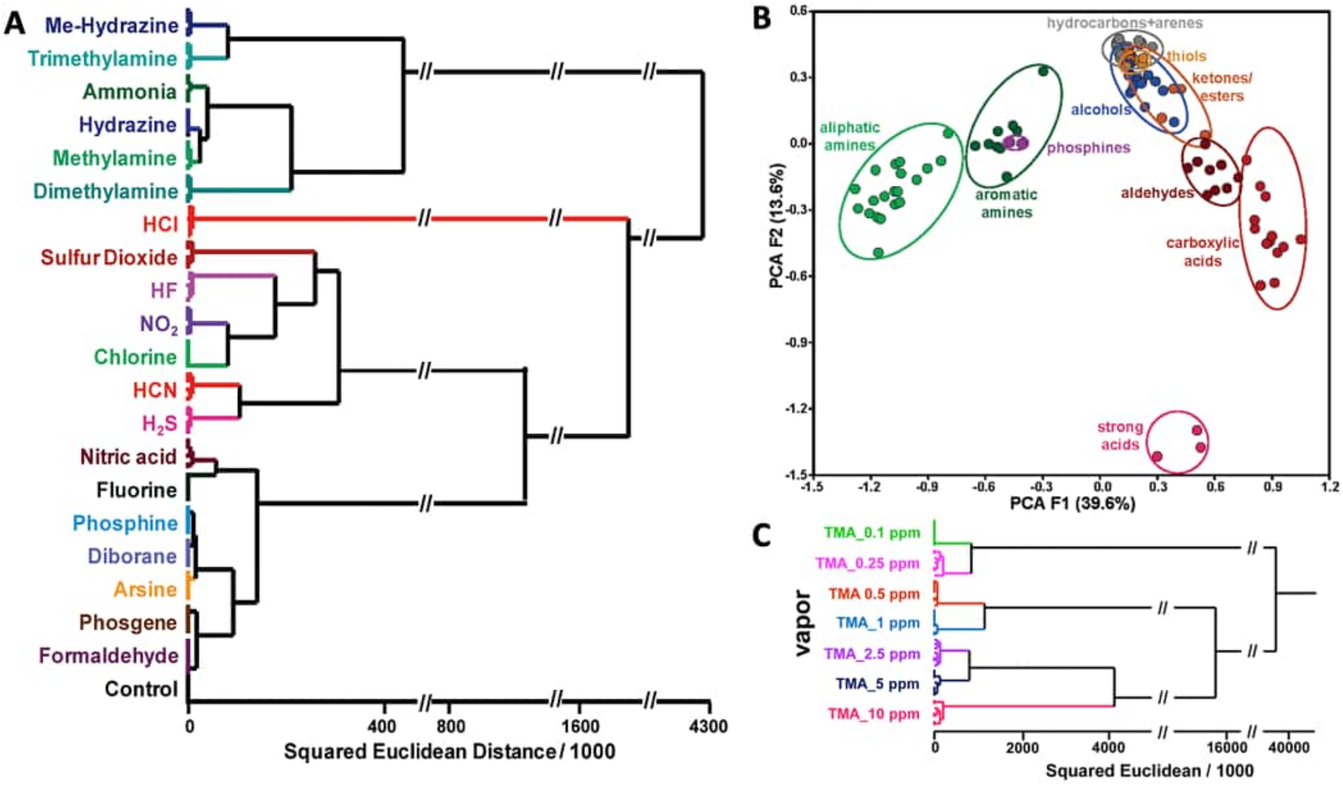

Figure 4. Data analysis methods for colorimetric sensor array. (A) Hierarchical cluster analysis for 20 toxic industrial chemicals (TICs) at (immediately dangerous to life or health) (IDLH) concentrations and a control. (B) Two principal components of the colorimetric sensor array from the response data averages of the 100 VOCs at 295 K, at their full vapor pressure using principal component analysis (PCA) method. (C) Dendrogram of hierarchical cluster analysis (HCA) for trimethylamine (TMA) at different concentrations in gases. Reproduced with permission from refs 41, 40, and 42 Copyright 2010, 2006, and 2016 American Chemical Society.

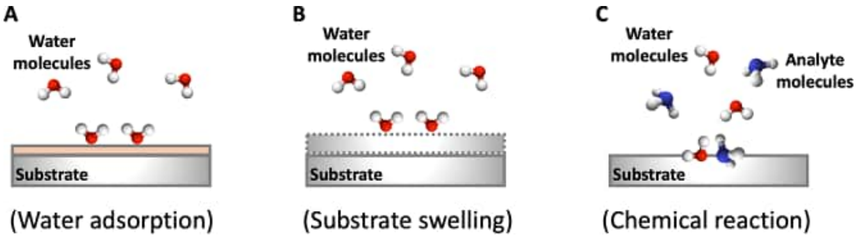

Figure 5. Illustration of the possible mechanisms of humidity interference: (A) water adsorption, (B) substrate swelling, and (C) chemical reaction.

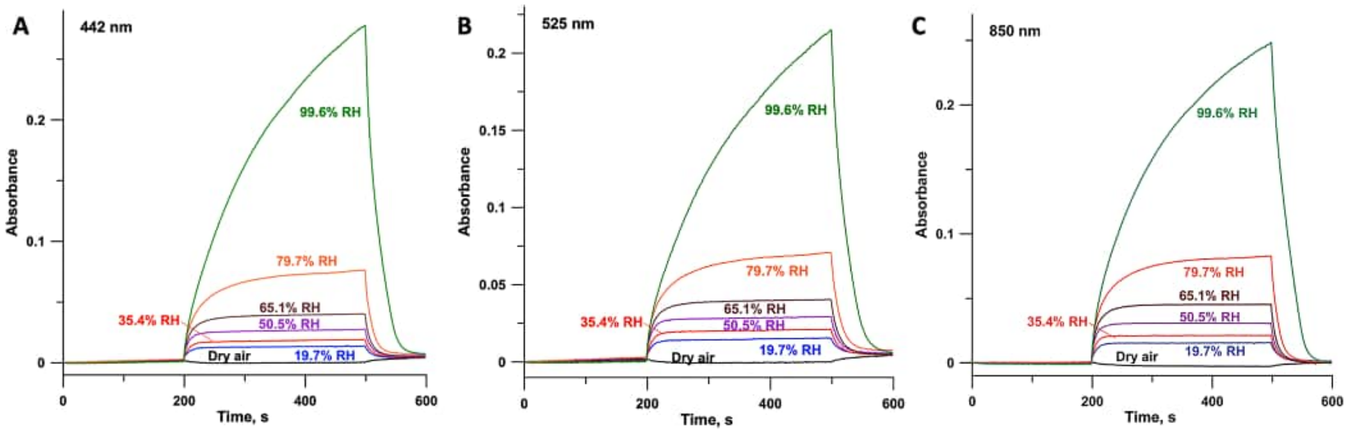

Figure 6. Measured absorbance of the silica plate under different relative humidities at the wavelengths of (A) 442 nm, (B) 525 nm, and (C) 850 nm. Reproduced with permission from ref 43. Copyright 2009 American Chemical Society.

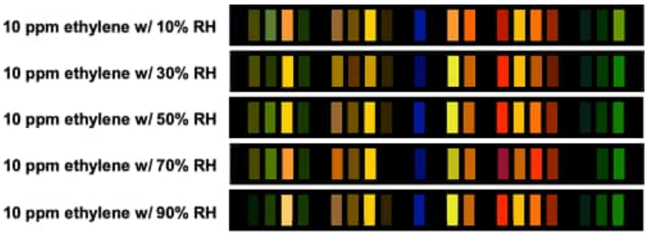

Figure 7. Responses of Pd(II)-silica porous microsphere sensors-based cumulative colorimetric sensor array to 10 ppm ethylene in the presence of 10% to 90% relative humidity. Reproduced with permission from ref 25. Copyright 2019 American Chemical Society.

enough for the chemical reaction, and hence the sensor was immune to the humidity variation.

In a colorimetric sensing platform, the physical interaction and the chemical interaction can happen simultaneously, which makes the humidity interference hard to predict. The entanglement between physical change and chemical change could also result in a nonlinear relationship between the humidity level and the sensor response, creating additional difficulties for signal compensation and correction. To avoid the burden on signal processing and algorithm development, desiccants, Nafion tubing, and hydrophobic substrates are often used to eliminate or repel the water vapor from the chemical reactions. On the other hand, it is also possible to take advantage of the effect that humidity brings to the sensing system to promote the performance of the sensors for analyte detection. This strategy is usually used in breath biomarker detection, since the humidity level in breath is high and constant.

■ MITIGATION OF HUMIDITY INTERFERENCE IN COLORIMETRIC SENSING OF ENVIRONMENTAL GASES

Colorimetric sensors, including the "lock-and-key" colorimetric sensors and colorimetric sensor arrays, have been widely applied in different kinds of environmental gas sensing. The environmental gases here refer to pollutants and chemical vapors existed in home settings, offices, and industrial settings that are harmful to human health; chemical warfare agents and explosives; and odors of food and beverage. Based on the applications, we classify the analytes into five categories:

(1) Volatile organic compounds (VOCs): VOCs are organic chemicals which show high vapor pressure at room temperature. VOCs are released by household products, building materials, furniture, gasoline, and other products. They can cause short-term and long-term adverse health effects, such as irritation, headache, nausea, or cancers.[47]

(2) Air-quality-related pollutants: These refer to six air-quality-related principal pollutants (or "criteria pollutants") identified by the EPA (Environmental Protection Agency), which are harmful to people and the environment.[48]

(3) Toxic industrial chemicals: These refer to industrial chemicals that are manufactured, stored, transported, and used throughout the world.[49] These chemicals are very toxic and could have short-term health effects or risks to exposed individuals.

(4) Chemical warfare agents and explosives: These refer to warfare agents such as nerve agents, poisons, and explosives, which are critical to homeland security.[7]

(5) Odors of food and beverage: These refer to the VOCs released by food and beverage products, such as pork, fish, chicken, turkey, squid, sausage, rice, fruit, coffee, tea, vinegar, beer, wine, honey, and other drinks.[5] These odors can be used for the safety and quality assessment of foods and beverages.

The analytes, sensing probes, substrates, and humidity concerns of the colorimetric sensors for environmental gases detection are summarized in Table 1.

As discussed before, the concentration of water vapor in the atmosphere ranging from ~10 ppm in the coldest regions of the Earth's atmosphere up to 5% by volume in hot, humid air masses.[11] This wide humidity variation presents a practical challenge for the colorimetric sensing system design. The sensing probes and the substrates are the key factors to determine the humidity effect of the colorimetric sensors. As suggested in Table 1, increasing the hydrophobicity of the sensing probes and the substrates is a widely adopted method for mitigating the humidity interference. Besides this materials engineering approach, there are also other methods that can address the humidity interference from the aspects of sample conditioning and signal compensation.

Using Desiccants to Reduce Humidity Level. To avoid the physical and chemical interactions of water molecules with the sensing system, a straightforward way is to remove the water vapor in the gas sample before it is delivered to the sensing chamber. This can be achieved by implementing a dehumidifier to the sensing system. The ambient humidity level can be reduced to almost zero by applying the desiccants for the pretreatment of the gas sample. Kawanura et al. developed a hand-held colorimetric toluene gas sensor for the prevention of sick building syndrome.[62] It was found that the colorimetric chemical reaction between toluene and the sensing probe, I_2O_5, was inhibited by high humidity. A hand-crafted dehumidifier made from calcium chloride was incorporated in the gas pretreatment system (Figure 8A), through which the gas sample was passed to bring the humidity down to ~19% for sensitive detection of toluene. With the help of the dehumidifier, the colorimetric sensor was able to detect toluene at a concentration of 0.5 ppm, as shown in Figure 8B.

Because of the continuous exposure to the ambient environment, the efficiency of the desiccants for dehumidification will drop. Usually, the desiccants will be packed in a cartridge, which can be replaced when its performance drops to a certain level.

Using the Matrices of Substrates for Humidity Regulation. The matrix of the substrates can not only immobilize the sensing probes but also modulate the local environment for the colorimetric sensing.[1] Thus, using hydrophobic materials as the matrices and substrates to immobilize sensing probes is a very effective way to mitigate the humidity interference.[1] The hydrophobicity and the hydrophilicity reflect how easily the water molecules can physically adsorb on the surface of the materials. If this water adsorption process can be blocked, the humidity interference can be mitigated.

Hydrophobic plates/membranes/films are widely used to minimize the humidity interference to the colorimetric sensors. As listed in Table 1, the hydrophobic substrates used in preparing the colorimetric sensors include both organic polymers and inorganic materials, such as reverse-phase silica thin-layer-chromatography plates,[36,37] polyethylene terephthalate (PET) film,[41,52,53] cellulose acetate membrane,[56] silica gel

Table 1. Summary of Colorimetric Sensing of Environmental Gases

categories	analytes	sensing probes	substrates	humidity concerns
Volatile organic compounds	Hydrocarbons, alcohols, amines, ethers, aldehydes, ketones, carboxylic acids, thioethers, phosphines, phosphites, thiols, arenes, and halocarbons.[36,37,40]	Metalloporphyrins, free-base porphyrins, pH indicators, and solvatochromic dyes.[36,37,40]	Reverse-phase silica thin-layer-chromatography plates.[36,37]	Hydrophobic dyes and substrates were selected to avoid humidity interference in 10 to >95% RH.[37]
Air quality related pollutants	Particulate matter, ozone, nitrogen dioxide, sulfur dioxide, carbon monoxide, and lead.[24,50,51]	Redox indicators,[50] and potassium disulfitopalladate (II).[24]	Silica G TLC plates.[24,50]	The sensing performance does depend on the humidity of the ambient air.[24]
Toxic industrial chemicals	Industrial chemicals that are manufactured, stored, transported, and used throughout the world, such as ammonia, arsine, chlorine, diborane, dimethylamine, fluorine, formaldehyde, hydrogen chloride, hydrogen cyanide, hydrogen fluoride, hydrogen sulfide, hydrazine, methylamine, methyl hydrazine, nitric acid, nitrogen dioxide, phosgene, phosphine, sulfur dioxide, and trimethylamine et al.[41,52−54]	Sol−gel-colorant solutions,[41,52,53] and mononuclear iron(II) neutral complex.[54]	Polyethylene terephthalate (PET) film.[41,52,53]	Hydrophobic dyes in hydrophobic matrixes rendering the sensor array much less sensitive to changes in humidity.[41,52,53]
Chemical warfare agents and explosives	Nerve agents like sarin, soman, and tabun; poisons, like mustard gas, lewisite, phosgene, phosgene oxime, cyanide, mace, and ricin; explosives like TNT (2,4,6-trinitrotoluene), DNT (2,4 dinitrotoluene), HMX (octahydro-1,3,5,7-tetranitro-1,3,5,7-tetrazocine), Tetryl (2,4,6-trinitrophenylmethylnitramine), DDNP (diazodinitrophenol), RDX (cyclotrimethylenetrinitramine), PA (picric acid), DMNB (2,3-dimethyl-2,3-dinitrobutane), and TATP (triaceonetriperoxide).[7]	Functionalized gold nanoparticles;[55] oxime-modified PDA (OX-PDA) liposomes;[56] push−pull chromophores;[57] Brady's reagent, metal salts, pH indicators, porphyrins, redox-sensitive dyes, solvatochromic dyes, and Strong base.[58,59]	Cellulose acetate membrane filter,[56] silica gel plate,[57] and polypropylene membranes.[59]	No color changes were detected at the humidity of saturated H_2O atmosphere[57] due to the use of hydrophobic dyes in hydrophobic matrixes, the colorimetric sensor array is very insensitive to changes in ambient humidity.[58]
Odors of food and beverage	Relevant volatile organic chemicals from food and beverage.[5,60]	Porphyrins and metalloporphyrins, pH indicators.[5]	C2 reverse phase silica gel plates, polyvinylidene fluoride (PVDF) membrane, silica gel plates, TiO_2 nanoporous film, polyvinylidene difluoride membrane, acetate sheet.[5]	The insensitivity of colorimetric sensors to changes in humidity is due to the hydrophobic dyes and hydrophobic matrixes.[60] To analyze samples with high humidity, the dyes and the membrane must be hydrophobic.[61]

plate,[57] polypropylene membranes,[59] poly(vinylidene fluoride) (PVDF) membrane,[63−65] acetate sheet,[5] and TiO_2 nanoporous film.[66]

Since the sensing probes are incorporated on the substrate surface, to mitigate the humidity interference, the sensing probes should be hydrophobic as well. The combination of hydrophobic substrates with water-insoluble, hydrophobic dyes has been proved to be a very effective strategy in reducing humidity interference for colorimetric sensor array.[67] Suslick's group has tried many ways to fabricate these hydrophobic colorimetric sensor arrays, mostly through directly printing hydrophobic dyes on the various hydrophobic substrate, such as reverse-phase silica gel thin-layer-chromatography plates[36,37] and PVDF.[63−65] Feng and Suslick et al. demonstrated that by immobilizing hydrophobic colorants on an extremely hydrophobic PVDF membrane, highly sensitive colorimetric detection of gaseous formaldehyde was achieved in 10−90% RH.[64] As shown in Figure 9, the colorimetric sensor array generated almost identical color difference maps at different humidity levels in the range of 10−90% RH without (Figure 9A) and with (Figure 9B) formaldehyde exposure, suggesting a high stability against humidity variation.

By proper choice of hydrophobic dyes and hydrophobic substrates, the colorimetric sensing array could even be immune to 100% RH. Janzen and Suslick et al. demonstrated that colorimetric sensor arrays were nonresponsive to humidity changes in the range of 11−100% RH, as shown in Figure 10.[40] The excellent humidity stability was essential for the sensor array to differentiate 100 VOCs.

Among different kinds of hydrophobic substrates, PVDF film seems to be a popular choice in different applications. The effectiveness of PVDF film for improving the reproducibility of the colorimetric sensing array was also proven by Wang et al. in volatile compound detection.[68] They evaluated the reproducibility of the sensing array for *n*-heptane (n-Hep), ammonia (NH_3), and chlorine hydride (HCl) detection in the humidity range of 0−100% and found no significant differences in sensor responses (Figure 11A). They attributed the excellent immunity of the sensing array to humidity to the superhydrophobic matrix of the PVDF film. The effectiveness of PVDF substrate to improve the stability of the colorimetric sensor against humidity changes was also reported by Zhong et al. in biogenic amines detection (Figure 11B).[69]

Printing soluble dyes onto porous membranes seems to be straightforward for colorimetric sensor fabrication, but implementing the nonpermeable pigments in the colorimetric sensors requires substantial chemical engineering work. Generally, a host material needs to be used for forming the nanoporous pigments via the sol−gel method.[70,71] In this way, the nanoporous pigments can be created to improve the sensitivity, durability, stability, and hydrophobicity of the colorimetric sensors.[52] Suslick's group has embedded a broad range of chemically responsive pigments into ormosils (organically modified silicate) to form nanoporous pigments and then printing these sol−gel colorants onto hydrophobic membranes to fabricate colorimetric sensor arrays.[41,52,53,72] Ormosil sol−gel matrix is an excellent porous matrix, mainly due to its high stability, large surface area, and good hydrophobicity. The colorimetric sensor arrays produced by this method are unaffected by the variations of relative humidity from 10% to 90%, as shown in Figure 12.[41] Moreover, by properly selecting the silane precursors and hydrophobic membranes, the

https://dx.doi.org/10.1021/acssensors.0c01644
ACS Sens. 2021, 6, 303−320

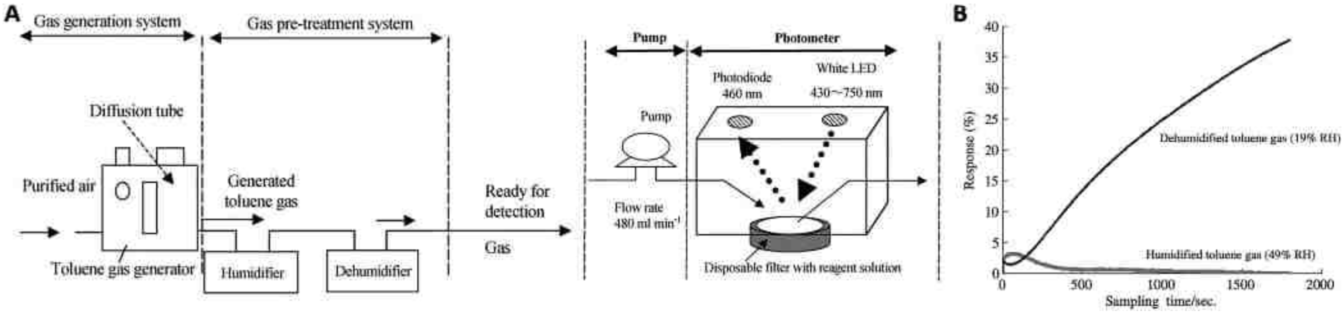

Figure 8. Using a dehumidifier to reduce the humidity of the gas sample. (A) Schematic diagrams of toluene gas generation system, pretreatment system, and the gas sensing system. (B) Effect of humidity (%) and response (%) at toluene gas concentration of 0.5 ppm. Reproduced with permission from ref 62. Copyright 2006 Elsevier B.V.

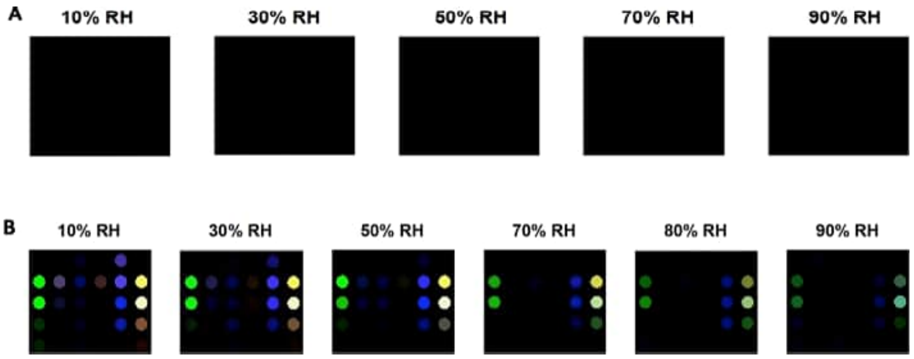

Figure 9. Hydrophobic colorimetric sensor array for highly sensitive formaldehyde detection. (A) Difference maps of the colorimetric sensor array to various humidity from 10% to 90% RH. (B) Difference maps of the colorimetric sensor array to formaldehyde at 1.5 ppm at different levels of relative humidity at room temperature. Reproduced with permission from ref 64. Copyright 2010 American Chemical Society.

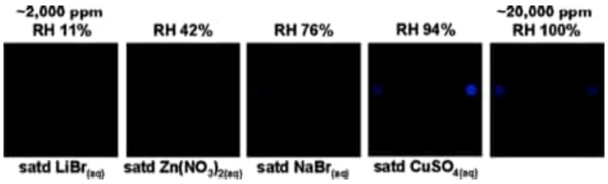

Figure 10. Difference map of colorimetric sensor array to different saturated aqueous salt solutions at 295 K. The array shows no response to humidity change in the range of 11−100% RH. Reproduced with permission from ref 40. Copyright 2006 American Chemical Society.

colorimetric sensor arrays can even be used for hydrophilic analytes detection in liquid.[73,74]

By adding plasticizer to the polymer-based colorimetric film, the hydrophobicity of the polymer matrix can be enhanced, as well as the immunity to humidity. Courbat et al. reported a colorimetric ammonia (NH_3) sensor by mixing pH indicator, polymers, and hydrophobic plasticizer.[75] The sensor showed high humidity dependence (Figure 13A), but the humidity interference could be modulated by the plasticizer. Compared to tributyl phosphate (TBP), dioctyl phthalate (DOP) was very effective in suppressing the humidity effect due to its strong hydrophobic nature (Figure 13B).

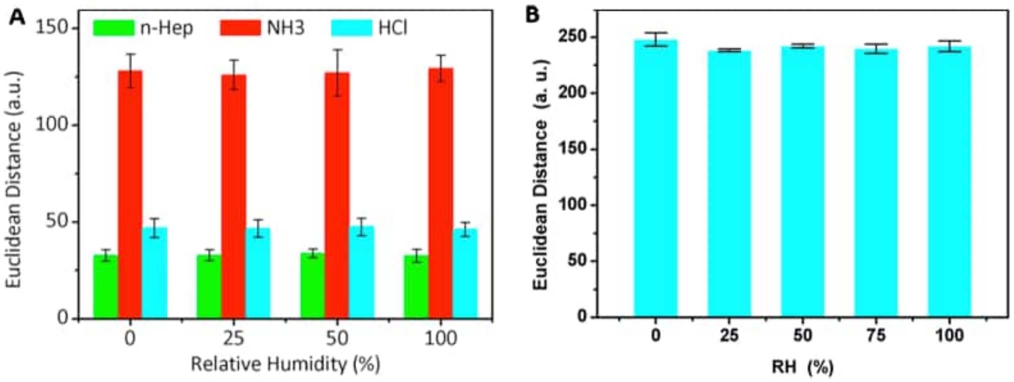

Figure 11. Humidity influence on colorimetric sensor arrays based on PVDF film. (A) Reproducible responses of colorimetric sensor arrays to volatile compounds: *n*-heptane (n-Hep), ammonia (NH_3), and chlorine hydride (HCl) in different relative humidity (RH). (B) Influence of humidity on the response of the arrays to trimethylamine (TMA) at concentration of 10 ppm. Reproduced with permission from refs 68,69. Copyright 2019 and 2018 Elsevier B.V.

https://dx.doi.org/10.1021/acssensors.0c01644
ACS Sens. 2021, 6, 303−320

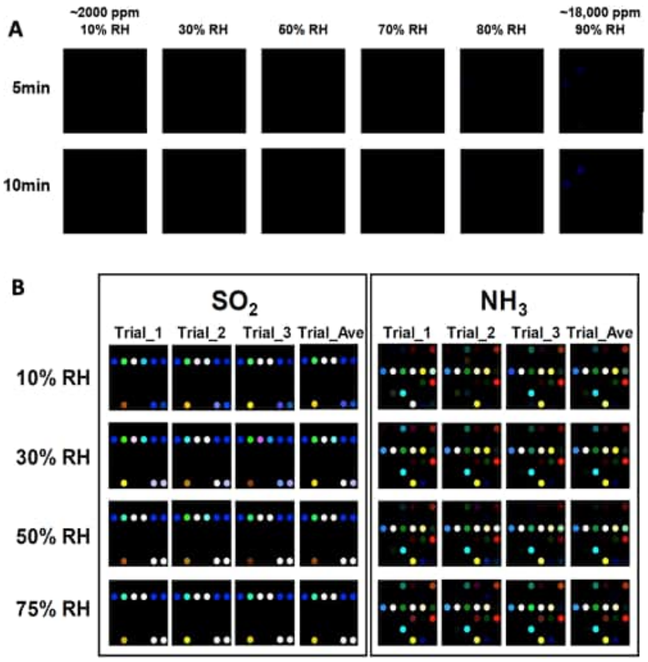

Figure 12. Humidity effect on colorimetric sensor array based on ormosil matrix. (A) Colorimetric sensor array has no response to variations in humidity from 10% to 90% RH (i.e., the color difference maps are black). (B) Difference maps of the colorimetric sensor array for ammonia and sulfur dioxide at IDLH (immediately dangerous to life or health) concentrations at different humidity levels after 2 min of exposure time. Reproduced with permission from ref 41. Copyright 2010 American Chemical Society.

Inorganic film with nanoporous structures could be another alternative for mitigating humidity interference in colorimetric sensing systems. Huang et al. developed a TiO_2-based nanoporous film as the sensing substrate for colorimetric detection of meat-borne trimethylamine (TMA), a key indicator for meat spoilage.[66] The TiO_2-based nanoporous film was impervious to humidity change. As shown in Figure 14, the colorimetric sensor gave almost no response to humidity change in the range of 30−70% RH.

Instead of increasing the hydrophobicity of the film matrix, humectant can also be introduced to buffer the variation of the ambient humidity. Qin et al. added glycerol, a hygroscopic substance, to the silica plate-based colorimetric formaldehyde (HCHO) sensor to serve as the humidity buffer to stabilize the humidity level in the sensor matrix.[34] The variation of the sensor response to HCHO was less than ±15% despite the humidity change in the range of 5−90%, as shown in Figure 15. Study also indicated that hydroxide, an active component in the sensing recipe to control the basicity for chemical reaction, played an important role in improving the stability of the sensor against humidity.[76]

Hydrogels are 3D cross-linked, highly water-swellable, hydrophilic polymer networks that have been widely used in sensing. Hydrogels can serve as a host matrix to immobilize the sensing probes and regulate the diffusion of the analytes in the sensing network.[77] Devadhasan et al. reported a disposable hydrogel test strip consisting of titanium nanoparticles, poly(vinyl alcohol) (PVA) hydrogel, and chemically responsive pH indicators for sensing toxic gases such as hydrogen fluoride (HF), chlorine (Cl_2), ammonia (NH_3), and formaldehyde (CH_2O).[78] The colorimetric detection was based on the color change of dyes due to acid−base reactions. Humidity analysis suggested that the test strips were unresponsive to changes in relative humidity, due to the use of glutaraldehyde as a cross-linking agent. It was reported that glutaraldehyde preserved the structure of PVA hydrogel film from swelling and shrinking up to 95% humidity exposure.[78] Since the colorimetric sensor strip was mechanically stable regardless of the ambient humidity, changes in humidity did not affect the sensing performance of the hydrogel test strips, as shown in Figure 16.

Using Humidity Sensor to Signal Compensation. Instead of avoiding the humidity interference, one can establish the relationship between the humidity level and its impact on the optical signals and then remove the humidity-induced sensor response from the overall signal. This method is more practical when the humidity dependence of the sensor is predictable. Lin et al. found that though the colorimetric CO sensor they developed was significantly dependent on the ambient humidity level, the sensitivity of the sensor followed a linear relationship with the humidity level, as shown in Figure 17.[24] This sensing behavior was due to the catalytic effect and could be simply corrected by implementing a numerical compensation algorithm based on the signal from an additional humidity sensor.

Maruo et al. developed an indigo carmine-coated paper for ambient ozone detection (Figure 18A).[79] The sensor, which consisted of cellulose sheet, humectant, acid, and indigo carmine, gave a color change from blue to white upon ozone

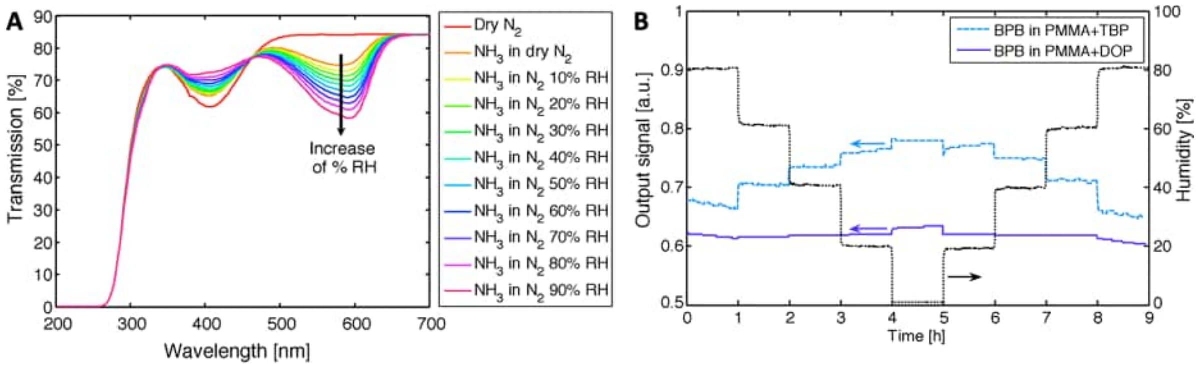

Figure 13. Using hydrophobic plasticizer to minimize the humidity effect on colorimetric ammonia sensor. (A) Transmission spectra of the bromophenol blue (BPB) + PMMA+ tributyl phosphate (TBP) film in 100 ppm of NH_3 for different RH levels. (B) Humidity response of the BPB + PMMA films with either TBP or dioctyl phthalate (DOP) as plasticizer when they were exposed to 5 ppm of NH_3 for different RH levels. Reproduced with permission from ref 75. Copyright 2009 Elsevier B.V.

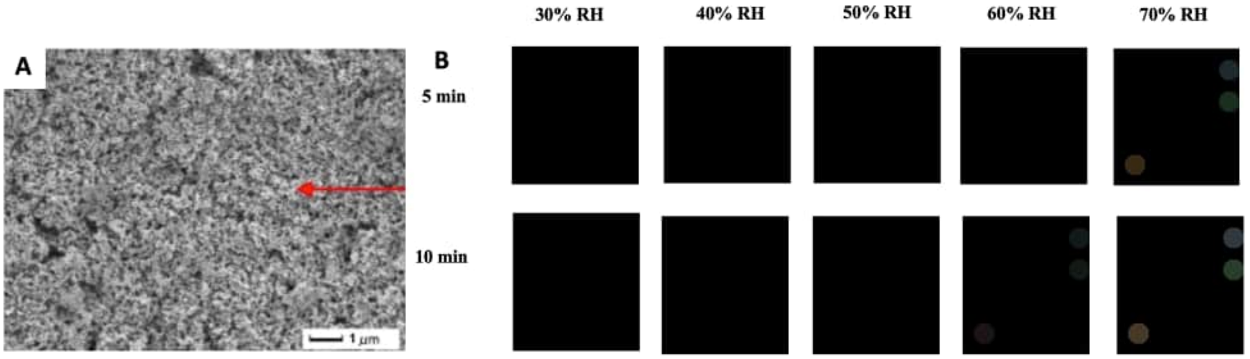

Figure 14. Nanoporous TiO_2 film-based colorimetric sensor array is unaffected by humidity in the range of 30–70% RH. (A) SEM images of nanoporous TiO_2 film, (B) nanoporous colorimetric sensor array under different levels of humidity exposure. Reproduced with permission from ref 66. Copyright 2016 Elsevier B.V.

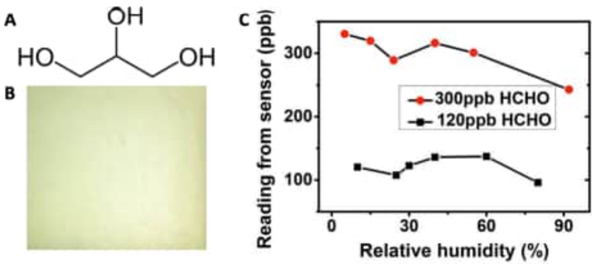

Figure 15. Using humectant to minimize the humidity effect on colorimetric formaldehyde (HCHO) sensor: (A) molecular structure of glycerol; (B) silica plate-based formaldehyde sensor with glycerol; and (C) sensor responses to HCHO (120 and 300 ppb) at different relative humidity levels (5–90%). Reproduced with permission from ref 34. Copyright 2015 IEEE.

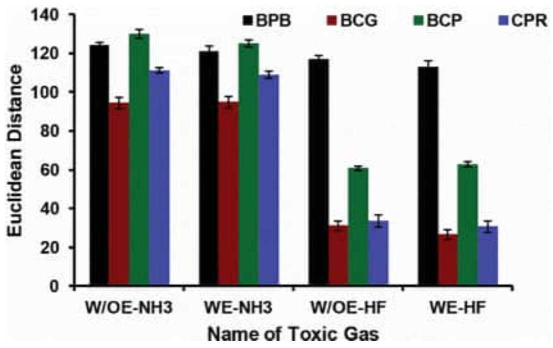

Figure 16. Humidity effect on a colorimetric hydrogel test strip for toxic gas detection. Color Euclidean distance shows that the sensor has no detectable response to 50% RH at room temperature. (W/OE: without exposure at 50% RH; WE: with exposure at 50% RH). Reproduced with permission from ref 78. Copyright 2017 Elsevier B.V.

exposure, as shown in Figure 18B. They found that the response of the sensor to ozone was highly influenced by the relative humidity, but the relationship between the relative humidity and the optical signal could be described by formulas. Three equations were used to correct the humidity interference in the RH range of 10–55%, 55–70%, and 70–85%, respectively, as shown in Figure 18C.

Environmental humidity variation makes the humidity interference in colorimetric sensing of environmental gases less predictable. Three kinds of methods have been used to mitigate the humidity inference, including the use of (1) desiccants to reduce humidity level; (2) matrices of substrates for humidity regulation; and (3) humidity sensor to signal compensation. Among these methods, incorporating hydrophobic dyes/pigments with hydrophobic plates/membranes/films is the most widely used approach in different applications.

■ MITIGATION OF HUMIDITY INTERFERENCE IN COLORIMETRIC SENSING OF BREATH BIOMARKERS

Human breath contains thousands of chemical compounds, the concentrations of which range from parts per trillion (ppt) to percent level.[80,81] Because of the partial pressure equilibrium between the pulmonary capillary blood and alveolar gas in the lung,[82] the chemical biomarkers in the exhaled air can be monitored for evaluating the health conditions of the human body, such as diagnostics of diseases or measurement of metabolism. Compared to blood tests, breath analysis offers a noninvasive diagnostic method. The biomarkers discovered in breath can be used for diagnosis of diseases such as (1) VOCs for lung cancer diagnosis, (2) nitric oxide, carbon monoxide, and ammonia for inflammatory lung diseases, (3) acetone for diabetes, (4) malondialdehyde, ethane, pentane, and methylated alkanes for oxidative stress in breast cancer, heart transplant rejection, and bronchial asthma, and (5) benzene in exhaled air for occupational hygiene.[83−85]

Conventional breath analysis equipment like GC-MS and SIFT-MS are bulky and expensive,[86,87] and more attention has been paid to the development of portable breath analyzers.[88,89] Chemical sensors are the core parts of breath analyzers. Electrochemical sensors, metal oxide sensors, spectroscopy sensors, and electronic noses have been used for building portable breath analyzers.[90] Colorimetric sensor is another promising candidate for sensing breath biomarkers in portable or wearable breath analyzers because of their excellent selectivity, high sensitivity, low cost, and the potential to be miniaturized. In addition, the colorimetric sensor array is an optoelectronic nose that can detect VOC fingerprints for lung cancer diagnosis.[1,8,91,92]

The exhaled breath is nearly saturated with water at body temperature, which is important to keep the airway surfaces moist and enhance the gas exchange.[93,94] The nearly 100% RH in a breath sample and the potential water condensation on the sensor surface can be a huge challenge to the colorimetric sensing of breath biomarkers, considering that most of the biomarkers are in ppm or even ppb level. By overcoming the humidity challenge, many colorimetric sensors have been reported in successfully detecting different kinds of breath

https://dx.doi.org/10.1021/acssensors.0c01644
ACS Sens. 2021, 6, 303−320

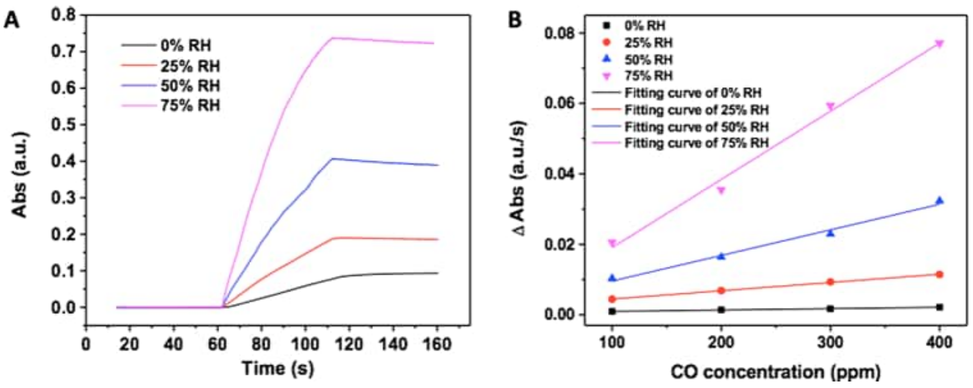

Figure 17. Humidity interference correction by numerical compensation. (A) CO sensor responses to 100 ppm of CO gas samples under different humidity levels. (B) Sensor responses to CO with various concentrations under different relative humidity levels. Reproduced with permission from ref 24. Copyright 2018 American Chemical Society.

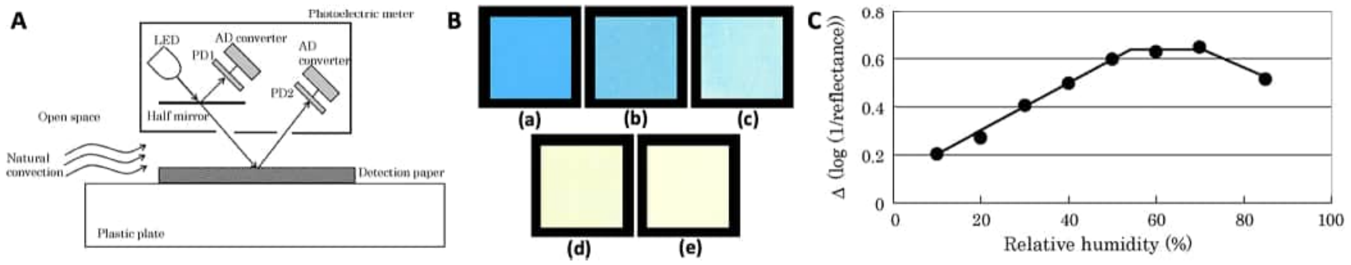

Figure 18. Humidity correction by formulas. (A) Schematic diagrams of the optical sensing system. (B) Photographs of ozone detection paper after exposure to 0−640 ppb × h ozone at 25°C, and 60% RH: (a) 0 ppb, (b) 129 ppb, (c) 268 ppb, (d) 416 ppb, and (e) 640 ppb. (C) Relationship between relative humidity and optical signal for 380 ppb ozone detection and the inset shows the correction formulas at different RH ranges. Reproduced with permission from ref 79. Copyright 2009 Elsevier B.V.

biomarkers. The sensing systems include both the "lock-and-key" colorimetric sensors and colorimetric sensor arrays.

The breath biomarkers, clinical correlation, related diseases, sensing probes, substrates, and humidity concerns of these colorimetric sensors are summarized in Table 2.

Among these colorimetric breath sensors, some are compatible with the highly humid breath condition, while others need interventions to mitigate the humidity interference.

Colorimetric Sensors Compatible with Highly Humid Breath Condition. The concentrations of breath oxygen and carbon dioxide carry important relevant health information about the metabolic rate (energy expenditure) and the pulmonary system (COPD, Capnography). Yu et al. developed an innovative breath O_2 sensing method based on the yellowing of newspaper.[95] They introduced UV light as a switch to activate the selective reaction between O_2 and newspaper, and the results showed that the sensor could reliably detect breath O_2 at 100% RH. Zhao et al. reported a colorimetric device for carbon dioxide detection in real breath.[96] This device utilized abundant water in exhaled gas to mitigate the humidity interference on the sensor. The sensing cartridge was coated with a pH indicator for CO_2 monitoring. When the warm exhaled gas contacts the sensing cartridge, water vapor condensed and formed an aqueous layer with an initial pH of 9.05. The pH decreased as CO_2 in exhaled gas dissolved in the aqueous layer, leading to proton release and color change of the pH indicator, as shown in Figure 19.

The detection of breath O_2 and CO_2 is relatively easy because the volume concentrations of O_2 and CO_2 in breath are at the percent level. To detect the ppm level breath biomarkers, the sensing probes must be functional at a high humidity level. Cha et al. reported the colorimetric sensing of hydrogen sulfide (H_2S) down to the sub-ppm level at 90% RH in breath for halitosis diagnosis using the lead acetate ($Pb(Ac)_2$) anchored nanofibers.[10] Neus Jornet-Martínez et al. developed a solid-phase plasmonic colorimetric sensor based on silver nanoparticle-coated nylon membrane and demonstrated its capability of detecting hydrogen sulfide-like compounds in breath samples.[108] They studied the influence of humidity on the colorimetric sensor in the range of 20−100% RH at room temperature (20 °C). The results showed that the sensitivity of the sensor is retained in 50−96% RH but decreased only at low humidity level, e.g., 20% RH, as shown in Figure 20.

Ito et al. reported a colorimetric sensing approach of using 4-nitrophenylhydrazine (4-NPH)-coated porous glass to detect breath acetone.[112] During their humidity interference study, they observed that when humidity increased, though the concentration of the sensing probe, 4-NPH, in the pores of the glass substrate was decreased, the volume of the solution actually increased. These two opposite changes canceled each other out, which made the performance of the sensor chip unaffected by humidity level.

Using Desiccants to Reduce Breath Humidity Level. For the sensing probes that are sensitive to humidity, additional interventions are needed for accurate breath biomarker detection. To ensure the accuracy of the breath biomarker detection, usually an efficient sample conditioning system was adopted to remove potential interferants and reduce the humidity level. Prabhakar et al. developed a miniaturized mouthpiece packed with desiccant particles to condition the humidity in real breath samples for breath analysis (Figure 21A).[113] The desiccation efficiency of the mouthpiece can be adjusted by the geometry of the mouthpiece, such as length and

Table 2. Summary of Colorimetric Sensing of Breath Biomarkers

breath biomarkers	clinical correlation	related diseases	sensing probes	substrates	humidity concerns
Oxygen (O_2)	Oxygen consumption related to metabolism.	Oxygen uptake, metabolic rate.[95]	Lignin[95]	Paper[95]	The sensor is capable of selectively detecting oxygen in human breath at high humidity (100%).[95]
Carbone dioxide (CO_2)	CO_2 production related to metabolism.	COPD (chronic obstructive pulmonary disease), asthma,[96] and capnography.[46,97]	pH indicators, such as *m*-cresol purple,[98] cresol red,[98] phenol red,[46] and thymol blue.[96]	Hydrophobic plastic film (ethyl cellulose) incorporated with a phase-transfer agent (tetraoctyl ammonium hydroxide)[46]	The colorimetric CO_2 sensor showed stable performance at 37 °C and 100% humidity.[46]
Acetone	Breath acetone is correlated with blood glucose (BG) and blood ketone levels.[99,100]	Diabetes[99,100]	Resorcinol[35,101]	Nafion membrane[35,101]	The incorporated organic acids played an important role in mitigating the humidity interference to the Nafion membrane catalysts in humid breath environment.[35]
Nitric oxide (NO)	NO level is elevated in asthma with inflammatory.	Asthma[102,103]	Cobalt tetraphenylporphyrin (CoTPP)[104] and phenylenediamine derivatives.[105]	Polystyrene (PSt), ethylcellulose (EC), polycyclohexyl methacrylate (PCHMA),[104] and microporous cellulose membrane.[105]	The hydrophobic polymer matrix significantly improved the humidity resistance.[104] A Nafion tubing was integrated to avoid the humidity interference.[105]
Hydrogen sulfide (H_2S)	An endogenous gaseous signaling molecule and potential therapeutic agent.[106]	Bad breath[107]	Lead(II) acetate,[10] silver nanoparticles,[108] and copper(II) complex.[107]	Polyacrylonitrile nanofibers,[10] nylon membrane,[108] and Prozorb substrate.[107]	Sensor can detect sub-ppm of H_2S gas in high relative humidity (~90% RH).[10] The sensitivity of the sensor is retained in 50−96% RH, but decreased at low humidity level, e.g., 20% RH.[108]
Ammonia (NH_3)	Associated with blood urea nitrogen	Renal failure[109−111]	pH indicators[109,110]	Hydrogel film (with the combination of polyvinyl alcohol, titanium oxide nanoparticles, and hydroquinone)[109]	The hydrogel film with the combination of other chemicals provides a versatile matrix to minimize the environmental interference.[109]
VOCs	Volatile organic compounds (VOCs) related to metabolic changes within cancer cells.	Lung cancer[8,91,92]	Metalloporphyrins, pH indicators, and dyes with large permanent dipoles.[8,91,92]	Poly(vinylidene fluoride) membrane[92]	Sensor is stable against humidity because of the high hydrophobicity of polyvinylidene fluoride.[92]

diameter, as shown in Figure 21B. By carefully choosing the desiccants, the mouthpiece can selectively remove the water molecules while leaving the analytes of interest intact, which was very useful for the breath analyzers. Figure 21C showed the performance of calcium chloride-packed mouthpiece, where ~70% of the humidity was removed while analytes like acetone, carbon dioxide, nitric oxide, and oxygen were preserved.

Mazzone et al. implemented the desiccant in front of the colorimetric sensor array to reduce humidity interference so that the VOCs related to the lung cancer were reliably detected in the breath (Figure 22).[114] In addition, temperature was controlled at 30 °C to minimize the condensation of water vapor.

Using Nafion Tubing for Humidity Regulation. Nafion is a copolymer of tetrafluoroethylene and perfluoro-3,6-dioxa-4-methyl-7-octene-sulfonic acid. It is very inert to chemical attack and highly permeable to water, making it an ideal material for drying and humidifying gas samples. Prabhakar et al. developed an ultrasensitive colorimetric sensor for nitrogen oxides detection aiming at both breath analysis and environmental monitoring.[105] The phenylenediamine derivative (*o*-phenylenediamine and diethyl-*p*-phenylenediamine) coated microporous cellulose membrane was capable of detecting ppb level NO in exhaled breath, showing a high potential for asthma diagnosis. As shown in Figure 23A, the sensor signal was interfered with by high humidity (~100% RH). The shape of the response curve suggested that the humidity interference was probably due to condensation of water on the optical pathway. After implementing a Nafion tubing to the inlet of the sensing chamber, the humidity interference was effectively avoided (Figure 23A). The performance of the sensing platform for breath sample analysis was validated against the gold standard method (Figure 23B), which also proved the effectiveness of this humidity mitigating approach.

Using the Matrices of Substrates for Humidity Regulation. As discussed in previous sections, the combination of hydrophobic dyes with hydrophobic substrates is very effective in reducing the humidity interference. This approach also works for breath biomarker detection.[92] Zhong et al. reported the recognition of VOCs in breath for lung cancer screening using colorimetric sensor arrays in a wide humidity range.[92] Their experimental results indicated that the sensor responses were quite stable in 0−100% RH range, as shown in Figure 24A. The superhydrophobic material of PVDF and the micro- to nanostructure of the substrate were both believed to contribute to the hydrophobicity and, thus, the high sensing stability against humidity variation.

Mills et al. developed a solid-state colorimetric sensor for breath-by-breath CO_2 detection by using a hydrophobic plastic film (ethyl cellulose) incorporated with a phase-transfer agent (tetraoctyl ammonium hydroxide) and a hydrophilic pH-sensitive dye (phenol red).[46] The colorimetric CO_2 sensor showed stable performance at 37 °C and 100% humidity, as shown in Figure 24B. It was speculated that the hydrophobic plastic film and the phase-transfer agent played an important role in mitigating the humidity interference.

Shiba et al. reported a humidity-resistive colorimetric breath NO sensor that was fabricated by dispersing the sensing probe in a hydrophobic polymer matrix.[104] They dispersed cobalt tetraphenylporphyrin (CoTPP) in three kinds of hydrophobic polymer film matrix: polystyrene (PSt), ethylcellulose (EC), and polycyclohexyl methacrylate (PCHMA) (Figure 25A). It was found that the hydrophobic polymer matrix not only significantly improved the humidity resistance (Figure 25B)

https://dx.doi.org/10.1021/acssensors.0c01644
ACS Sens. 2021, 6, 303−320

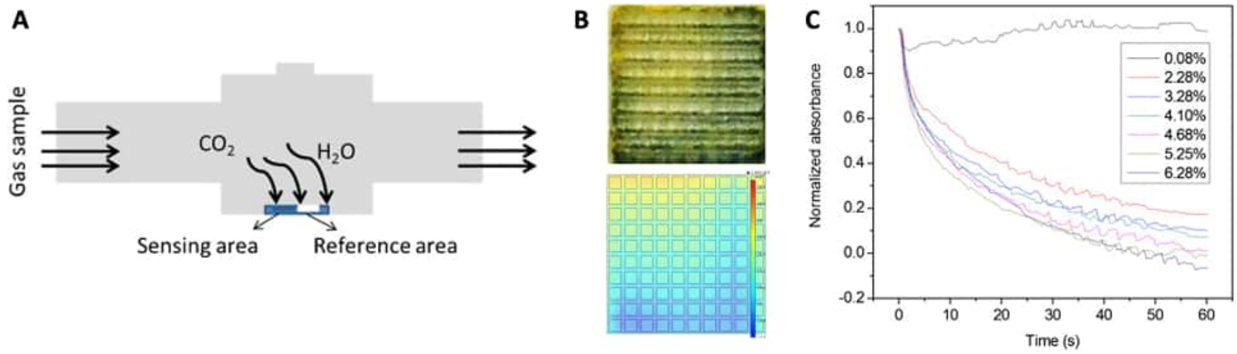

Figure 19. High humidity assisted breath biomarkers detection. (A) Schematic diagram of breath CO_2 sensing system. (B) Real and simulated color development on sensor chip after 6.5 s of exposure to CO_2 sample. (C) Normalized absorbance vs time at the CO_2 concentration of 0.08−6.28%. Reproduced with permission from ref 96. Copyright 2013 Elsevier B.V.

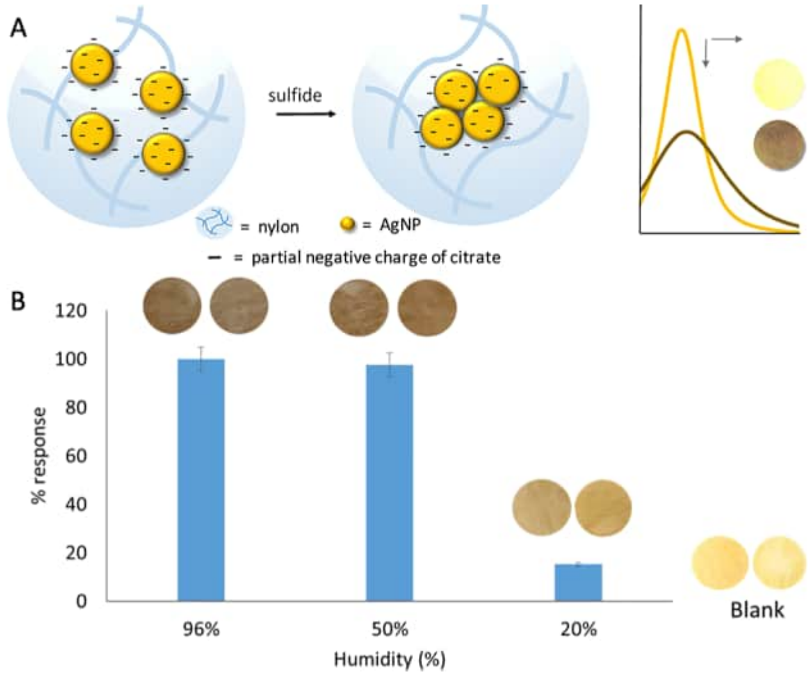

Figure 20. Plasmonic colorimetric sensor for breath hydrogen sulfide detection. (A) Colorimetric sensing principle. (B) Influence of humidity on the response of the sensor for 2500 ppvb H_2S at 20 °C. Reproduced with permission from ref 108. Copyright 2019 American Chemical Society.

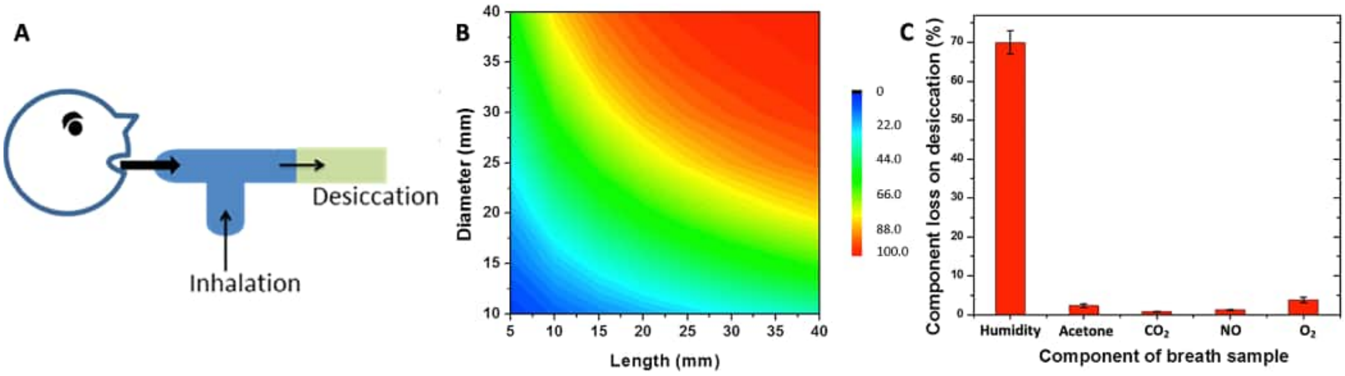

Figure 21. Online sample conditioning mouthpiece for reducing breath humidity. (A) Schematic of the online sample conditioning mouthpiece. (B) Desiccation efficiency simulated as a function of tube geometry for a given volumetric flow rate. (C) Selective removal of humidity by the desiccant mouthpiece over other chemical components of interest. Adapted with permission from ref 113. Copyright 2012 American Chemical Society.

but also dramatically increased the sensitivity, which made the sensor capable of detecting NO in humid breath for asthma diagnosis.

Worrall et al. reported an approach of using organic acid incorporated Nafion membranes to maintain the catalytic capability in colorimetric detection of exhaled acetone in the

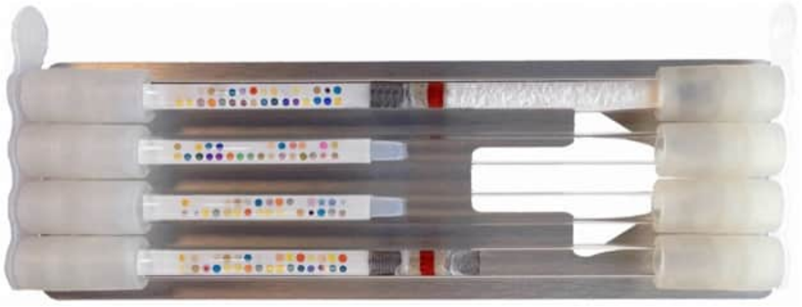

Figure 22. Colorimetric sensor array cartridge with integrated desiccant. There are 4 channels for airflow, each containing 32 sensor elements. The top channel contains a desiccant and oxidizer. Reprinted from ref 114 with permission of the American Thoracic Society. Copyright 2015 American Thoracic Society.

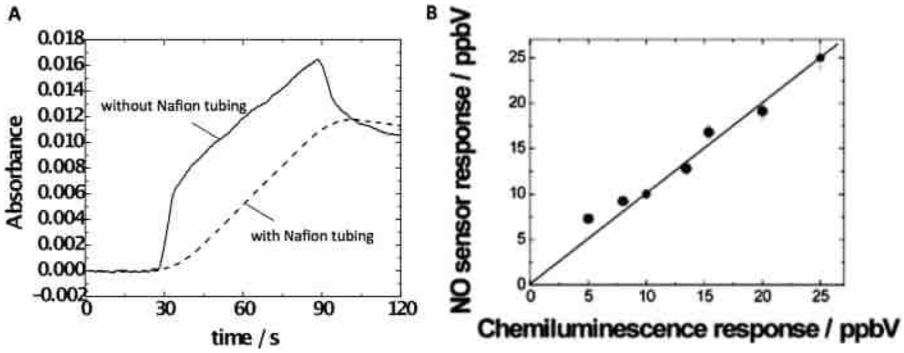

Figure 23. Using Nafion tubing to avoid humidity interference. (A) Sensor response at ∼100% relative humidity with and without a Nafion tubing located at the inlet of the sensing chamber. (B) Evaluation of the sensor accuracy with a Nafion tubing for real sample analysis. Comparison of NO concentration determined by the present sensor vs NO concentration determined by the gold standard method (chemiluminescence). Reproduced with permission from ref 105. Copyright 2010 American Chemical Society.

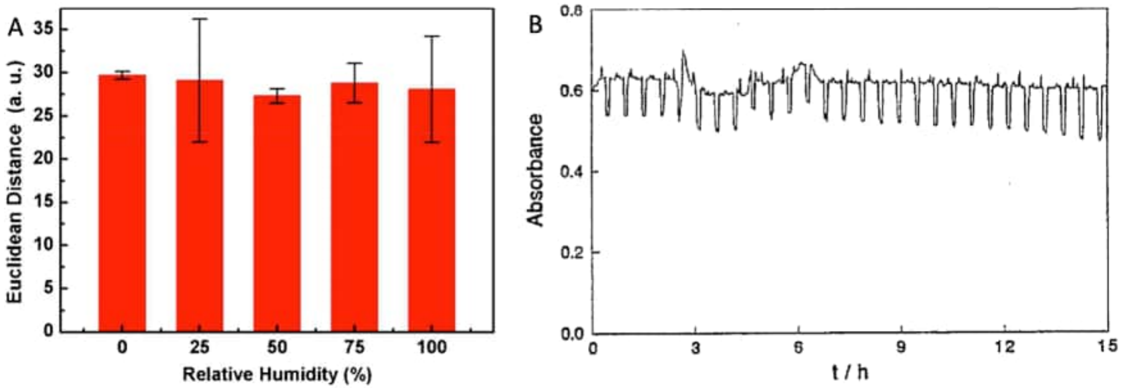

Figure 24. (A) Stability of the colorimetric sensor array for breath VOCs detection against humidity. (B) Response curve of the colorimetric CO_2 sensor exposed to an alternating atmosphere of N_2 and 5% CO_2 at 100% relative humidity and 37 °C. Reproduced with permission from refs 92 and 46. Copyright 2018 Springer and 1997 Elsevier B.V.

humid breath environment for diabetes diagnosis.[35] The colorimetric detection was based on the chemical reaction between breath acetone and membrane-immobilized resorcinol, producing colored flavan, as shown in Figure 26A.[35] In order to preserve the catalytic activity of Nafion membrane, organic acids such as vanillic or tiglic were immobilized in the membrane. Though these acids cannot serve as catalysts in the reaction, they can modulate the ionization of the perfluorosulfonic acid (PSA) groups in Nafion to catalyze the chemical reaction between acetone and resorcinol. This modulation was based on the phenomena that ionization of acids was lower in acidic solvent than in water and the pK_a of strong acid increased in weak acid relative to water. Thus, the incorporated organic acids played an important role in mitigating the humidity interference to the Nafion membrane catalysts in humid breath environment. The schemes of this mechanism were presented in Figure 26B. The performance of the membrane for acetone detection at 100% RH was presented in Figure 26C, which proved the effectiveness of this approach for exhaled acetone detection in humid human breath.

Since the humidity level in breath is around 100% RH, the challenge for a colorimetric breath sensor is not the humidity variation but the water condensation. Though some colorimetric sensors are compatible with the highly humid breath condition, many of them still need interventions to mitigate the humidity interference. Three kinds of methods have been used

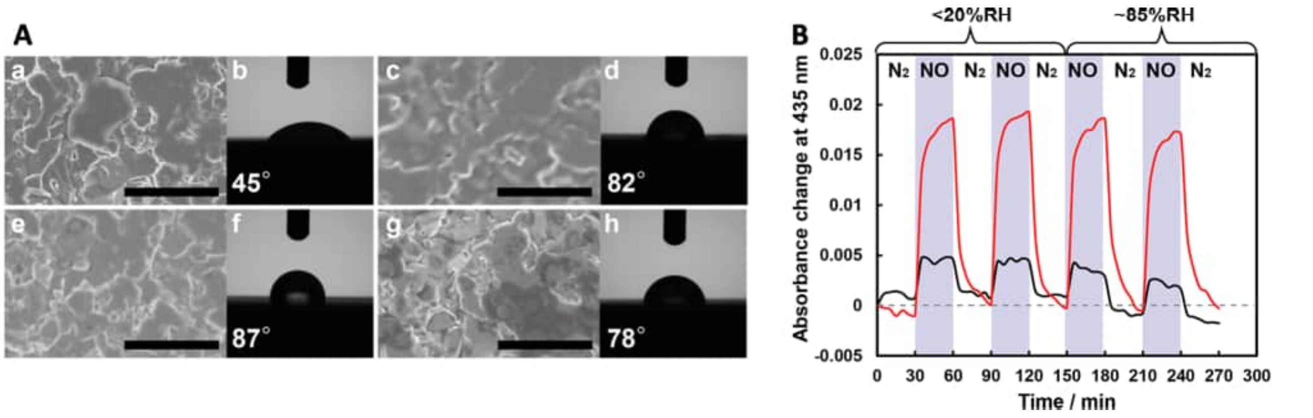

Figure 25. Improving humidity resistance by dispersing sensing probe in hydrophobic polymer matrix. (A) Top surface structures and water-drop contact angle measurements of the (a,b) cobalt tetraphenylporphyrin (CoTPP), (c,d) cobalt tetraphenylporphyrin ethylcellulose (CoTPP-EC), (e,f) cobalt tetraphenylporphyrin polystyrene (CoTPP-PSt), and (g,h) cobalt tetraphenylporphyrin polycyclohexyl methacrylate (CoTPP-PCHMA) films (Scale bar: 5 μm). (B) 1 ppm of NO sensing at 100 °C under low humidity (<20 RH%) and high humidity (85 RH%) conditions for CoTPP-EC film (red solid line) and CoTPP film (black solid line). Reproduced with permission from ref 104. Copyright 2020 MDPI.

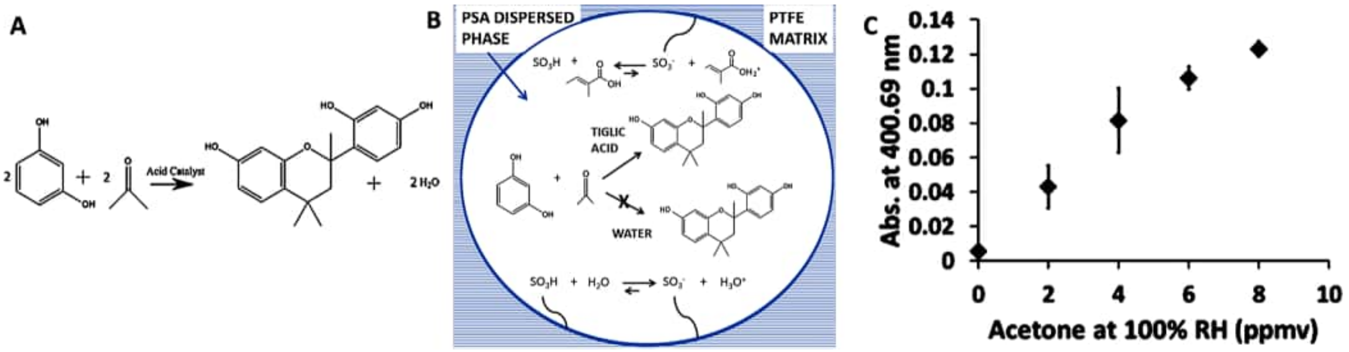

Figure 26. Water-resistant polymeric acid membrane catalyst for acetone detection in the exhaled breath. (A) Nafion-catalyzed reaction between resorcinol and acetone. (B) Mechanism for preservation of PSA catalytic activity in humid environments. Without tiglic acid (TA), PSA is a strong acid that readily deprotonates in the presence of water and can no longer catalyze the reaction. With TA, PSA is a weak acid with sufficient protonation even in the presence of water to catalyze the reaction. (C) Membrane response with acetone exposure at 100% RH. Reproduced with permission from ref 35. Copyright 2018 American Chemical Society.

to mitigate the humidity inference, including the use of (1) desiccants to reduce humidity level; (2) Nafion tubing for humidity regulation; and (3) matrices of substrates for humidity regulation. Among these methods, using the matrices of substrates for humidity regulation is the widely used approach. Compared to environmental gas detection, breath biomarker detection requires special consideration about the breath sampling system, because the exhalation flow rate and the backpressure could affect the test results.[115] Generally speaking, the online mode (meaning the subject directly exhales to the sensors/devices) is more likely to have the water condensation issue than the offline mode (meaning the breath sample of the subject is collected in a bag first and then delivered to the sensors/devices for detection).

■ CONCLUSIONS AND FUTURE PERSPECTIVES

Colorimetric sensors have been widely used for sensing environmental gases and breath biomarkers. Unlike other gas sensors, which are mostly based on physical interactions between the analytes and the receptors, colorimetric sensors use a broad range of chemical interactions for analyte detection. Depending on whether the sensing is based on specific binding or nonspecific binding, colorimetric sensors can be classified into two types: the "lock-and-key" colorimetric sensor and the colorimetric sensor array. The former is mostly used for quantitative detection of a specific analyte, while the latter is widely used for recognition and classification of a large set of analytes. Colorimetric sensor array is the dominating technique in colorimetric sensing, mainly due to its powerful sensing capability for providing high-dimensional data.

Humidity interference is a universal threat to the accuracy and stability of the colorimetric sensors. It can interfere with the colorimetric sensing through either the physical or the chemical way. The methods for mitigating humidity interference include (1) using desiccants to reduce humidity level, (2) using humidity sensor for signal compensation, (3) using Nafion tubing for humidity regulation, and (4) using the matrices of substrates for humidity regulation humidity. The analyte concentration may be reduced when external dehumidifying intervention is introduced to the sensing system, because of either physical adsorption or chemical adsorption of the analyte molecules on the desiccants or filters. This could be problematic when the concentration of the analyte in the gas is too low. Usually, the materials of the desiccants or filters need to be carefully selected to minimize the analyte loss. Substrate/matrix engineering is the primary approach to mitigate the humidity effect in colorimetric sensing, because this approach solves the humidity interference intrinsically and can make the sensor fabrication simple and efficient. Furthermore, the substrate/

matrix engineering approach could avoid the unwanted analyte loss, an issue that the desiccation methods could have.

The most widely used approach for mitigating the humidity interference is to incorporate hydrophobic sensing elements on hydrophobic substrates. Though this approach has been proven to be effective, it limits the options of choosing other kinds of sensing elements. The hydrophilicity could add another dimension for the colorimetric sensing array to expand its sensing power.

The colorimetric sensor array has shown its advantage over conventional e-nose technologies because it can include a broad range of physical and chemical intermolecular interactions, ranging from van der Waals interactions, dipole−dipole interactions, hydrogen bonding interactions, salt bridges interactions, π−π stacking complexation, Bronsted acid−base interactions, donor−acceptor interactions, to ion−ion interactions. Nevertheless, the mammalian olfactory system operates in an aqueous environment, meaning the sensing mechanism is water-compatible. To advance the current colorimetric sensor array, more efforts are needed to further understand and mimic the working principles of the mammalian olfactory system.

Coating plays a central role in preparing the colorimetric sensor cartridge. The conventional way is to cast the sensing element solution on a proper substrate and then let it dry. Though this method is effective, it also faces two technical challenges. One is the well-known “coffee-ring” effect, which could cause uneven distribution of the sensing elements in the sensing spots, and the other is the variation associated with the coating process, e.g., spot size variation, batch-to-batch variation, and crystallization. These challenges can cause precision and reproducibility issues for colorimetric sensing. New coating methods need to be developed to address these challenges.

Both environmental gas monitoring and breath biomarker detection require a multiplex sensing platform on which multiple analytes can be detected simultaneously. Colorimetric sensor is complementary to other sensing techniques, especially other optical sensing methods. A hybrid approach of integrating different sensing systems together could offer a full solution for certain applications, such as environmental health study.

The Internet of things (IoT) is an emerging technology that could change industry, business, and our daily life. It can connect billions of devices around the world to the Internet for data collecting and sharing. Sensors play an essential role in data collection. Moreover, smartphones are also integrating more and more sensors to achieve more functions for a better user experience. Though physical sensors that are compatible with modern electronics are widely available, such as proximity sensor, accelerometer, gyroscope, magnetometer, GPS, microphone, barometer, ambient light sensor, thermometer, humidity sensor, and biometric sensor, the proper chemical sensors are still missing. An IoT and smartphone compatible chemical sensor should be miniaturized, multiplexed, low power, and low cost. Colorimetric sensor array could be a potential candidate because it is compatible with the complementary metal-oxide-semiconductor (CMOS) imaging electronics and can collect high-dimensionality data about the chemicals. However, three major challenges remain: (1) how to further reduce the size of the colorimetric sensing array while maintaining its sensitivity and reproducibility; (2) how to make the colorimetric sensing array reusable rather than disposable; (3) how to make the colorimetric sensors immune to environmental changes, such as humidity and temperature variations. Considering that the chemical sensors to be implemented in IoT and smartphones should be miniaturized and mass-manufacturable, chemical engineering on the sensing probes and substrate matrices are needed to address the challenges of both the reusability and environmental stability.

To expand the use of colorimetric gas sensors in broad applications, the sensor performance should be competitive with other leading gas sensing technologies in the field, such as semiconductor metal oxide sensors, electrochemical sensors, solid-state sensors (resistive, capacitive, amperometric, and potentiometric), optical sensors, and mass-based sensors. The sensor performance could be evaluated from three different aspects: analytical performance, physical dimension, and power consumption. In terms of analytical performance, originating from its chemically specific nature, the colorimetric sensors can be highly sensitive and selective, compared to the sensors that are based on physical interactions between analytes and receptors. However, on the other hand, the reversibility and lifetime of the colorimetric sensors could also be a challenge because of the high specificity and affinity of chemical interactions. As for the physical dimensions, the size of the colorimetric sensors could be further reduced by grafting the advances of complementary metal oxide semiconductor (CMOS) imaging electronics, which could promote their applications in wearables and IoT. The power consumption of the colorimetric sensors can be very low, especially when miniaturized LEDs and CMOS imagers are used for the optical readout. This attributes the colorimetric sensors the competitive advantage over sensing techniques that require heating elements, such as semiconductor metal oxide sensors.

Colorimetric sensors have unique strengths in collecting chemical information about the environment and our health. We envision that the next-generation colorimetric sensors could play an important role in advancing smartphones, smart home, wearable devices, mobile health, and the Internet of things.

■ AUTHOR INFORMATION

Corresponding Author

Xiaojun Xian − *Center for Bioelectronics and Biosensors, The Biodesign Institute, Arizona State University, Tempe, Arizona 85287, United States*; Email: xiaojun.xian@asu.edu

Authors

Jingjing Yu − *Center for Bioelectronics and Biosensors, The Biodesign Institute, Arizona State University, Tempe, Arizona 85287, United States*; orcid.org/0000-0001-8985-8502

Di Wang − *Center for Bioelectronics and Biosensors, The Biodesign Institute, Arizona State University, Tempe, Arizona 85287, United States*; orcid.org/0000-0003-1581-4982

Vishal Varun Tipparaju − *Center for Bioelectronics and Biosensors, The Biodesign Institute, Arizona State University, Tempe, Arizona 85287, United States*

Francis Tsow − *Center for Bioelectronics and Biosensors, The Biodesign Institute, Arizona State University, Tempe, Arizona 85287, United States*

Complete contact information is available at: https://pubs.acs.org/10.1021/acssensors.0c01644

Author Contributions

[#]J.Y. and D.W. contributed equally. The manuscript was written through contributions of all authors. The manuscript was conceived and drafted by X.X. and revised by all the authors. All

authors have given approval to the final version of the manuscript.

Notes

The authors declare no competing financial interest.

■ ACKNOWLEDGMENTS

We dedicate this paper to Prof. Nongjian Tao, our forever mentor. This work was supported by National Institutes of Health (NIH) (Project Numbers: 4R44ES029006-02 and 5R44ES029006-03).

■ REFERENCES

(1) Li, Z.; Askim, J. R.; Suslick, K. S. The optoelectronic nose: colorimetric and fluorometric sensor arrays. *Chem. Rev.* **2019**, *119* (1), 231−292.
(2) Gunnlaugsson, T.; Glynn, M.; Tocci, G. M.; Kruger, P. E.; Pfeffer, F. M. Anion recognition and sensing in organic and aqueous media using luminescent and colorimetric sensors. *Coord. Chem. Rev.* **2006**, *250* (23−24), 3094−3117.
(3) Zhou, X.; Lee, S.; Xu, Z.; Yoon, J. Recent progress on the development of chemosensors for gases. *Chem. Rev.* **2015**, *115* (15), 7944−8000.
(4) Azzouz, A.; Vikrant, K.; Kim, K.-H.; Ballesteros, E.; Rhadfi, T.; Malik, A. K. Advances in colorimetric and optical sensing for gaseous volatile organic compounds. *TrAC, Trends Anal. Chem.* **2019**, *118*, 502−516.
(5) Huang, X.-w.; Zou, X.-b.; Shi, J.-y.; Li, Z.-h.; Zhao, J.-w. Colorimetric sensor arrays based on chemo-responsive dyes for food odor visualization. *Trends Food Sci. Technol.* **2018**, *81*, 90−107.
(6) Schmitt, K.; Tarantik, K. R.; Pannek, C.; Wöllenstein, J. Colorimetric materials for fire gas detection-A review. *Chemosensors* **2018**, *6* (2), 14.
(7) Kangas, M. J.; Burks, R. M.; Atwater, J.; Lukowicz, R. M.; Williams, P.; Holmes, A. E. Colorimetric sensor arrays for the detection and identification of chemical weapons and explosives. *Crit. Rev. Anal. Chem.* **2017**, *47* (2), 138−153.
(8) Mazzone, P. J.; Hammel, J.; Dweik, R.; Na, J.; Czich, C.; Laskowski, D.; Mekhail, T. Diagnosis of lung cancer by the analysis of exhaled breath with a colorimetric sensor array. *Thorax* **2007**, *62* (7), 565−568.
(9) Hou, C.; Lei, J.; Huo, D.; Song, K.; Li, J.; Luo, X.; Yang, M.; Fa, H. Discrimination of lung cancer related volatile organic compounds with a colorimetric sensor array. *Anal. Lett.* **2013**, *46* (13), 2048−2059.
(10) Cha, J.-H.; Kim, D.-H.; Choi, S.-J.; Koo, W.-T.; Kim, I.-D. Sub-parts-per-million hydrogen sulfide colorimetric sensor: lead acetate anchored nanofibers toward halitosis diagnosis. *Anal. Chem.* **2018**, *90* (15), 8769−8775.
(11) Wallace, J. M.; Hobbs, P. V. *Atmospheric science: An introductory survey*, 2nd ed.; 2006; pp 483−483.
(12) Mutlu, G. M.; Garey, K. W.; Robbins, R. A.; Danziger, L. H.; Rubinstein, I. Collection and analysis of exhaled breath condensate in humans. *Am. J. Respir. Crit. Care Med.* **2001**, *164* (5), 731−737.
(13) Rosias, P. P. R.; Dompeling, E.; Hendriks, H. J. E.; Heijnens, J. W. C. M.; Donckerwolcke, R. A. M. G.; Jobsis, Q. Exhaled breath condensate in children: Pearls and pitfalls. *Pediatric Allergy and Immunology* **2004**, *15* (1), 4−19.
(14) Dey, A. Semiconductor metal oxide gas sensors: A review. *Mater. Sci. Eng., B* **2018**, *229*, 206−217.
(15) Masikini, M.; Chowdhury, M.; Nemraoui, O. Review-metal oxides: Application in exhaled breath acetone chemiresistive sensors. *J. Electrochem. Soc.* **2020**, *167* (3), 037537.
(16) Wei, P.; Ning, Z.; Ye, S.; Sun, L.; Yang, F. H.; Wong, K. C.; Westerdahl, D.; Louie, P. K. K. Impact analysis of temperature and humidity conditions on electrochemical sensor response in ambient air quality monitoring. *Sensors* **2018**, *18* (2), 59.
(17) Pang, X. B.; Shaw, M. D.; Gillot, S.; Lewis, A. C. The impacts of water vapour and co-pollutants on the performance of electrochemical gas sensors used for air quality monitoring. *Sens. Actuators, B* **2018**, *266*, 674−684.
(18) Tai, H. L.; Duan, Z. H.; He, Z. Z.; Li, X.; Xu, J. L.; Liu, B. H.; Jiang, Y. D. Enhanced ammonia response of $Ti_3C_2T_x$ nanosheets supported by TiO_2 nanoparticles at room temperature. *Sens. Actuators, B* **2019**, *298*, 126874.
(19) Zhang, Y.; Zhang, J.; Jiang, Y.; Duan, Z.; Liu, B.; Zhao, Q.; Wang, S.; Yuan, Z.; Tai, H. Ultrasensitive flexible NH_3 gas sensor based on polyaniline/$SrGe_4O_9$ nanocomposite with ppt-level detection ability at room temperature. *Sens. Actuators, B* **2020**, *319*, 128293.
(20) Tai, H. L.; Duan, Z. H.; Wang, Y.; Wang, S.; Jiang, Y. D. Paper-based sensors for gas, humidity, and strain detections: A review. *ACS Appl. Mater. Interfaces* **2020**, *12* (28), 31037−31053.
(21) Brook, T. E.; Narayanaswamy, R. Polymeric films in optical gas sensors. *Sens. Actuators, B* **1998**, *51* (1−3), 77−83.
(22) Addabbo, T.; Fort, A.; Mugnaini, M.; Vignoli, V.; Baldi, A.; Bruzzi, M. Quartz-crystal microbalance gas sensors based on TiO_2 nanoparticles. *IEEE Trans. Instrum. Meas.* **2018**, *67* (3), 722−730.
(23) Matsuguchi, M.; Kadowaki, Y. Poly(acrylamide) derivatives for QCM-based HCl gas sensor applications. *Sens. Actuators, B* **2008**, *130* (2), 842−847.
(24) Lin, C.; Xian, X.; Qin, X.; Wang, D.; Tsow, F.; Forzani, E.; Tao, N. High performance colorimetric carbon monoxide sensor for continuous personal exposure monitoring. *ACS Sensors* **2018**, 3 (2), 327−333.
(25) Li, Z.; Suslick, K. S. Colorimetric sensor array for monitoring CO and ethylene. *Anal. Chem.* **2019**, *91* (1), 797−802.
(26) Spinelle, L.; Gerboles, M.; Villani, M. G.; Aleixandre, M.; Bonavitacola, F. Field calibration of a cluster of low-cost available sensors for air quality monitoring. Part A: Ozone and nitrogen dioxide. *Sens. Actuators, B* **2015**, *215*, 249−257.
(27) Tai, H. L.; Wang, S.; Duan, Z. H.; Jiang, Y. D. Evolution of breath analysis based on humidity and gas sensors: Potential and challenges. *Sens. Actuators, B* **2020**, *318*, 128104.
(28) Bäther, W. Detector tube technology. *Sens. Update* **1998**, *4* (1), 81−108.
(29) National Security Technologies LLC, Portable colorimetric tubes for chemical vapor detection market survey report; 2014.
(30) *Dräger-tubes & CMS-Handbook*, 16th ed.; Dräger Safety AG & Co., 2011.
(31) *Gastec gas detection system - detector tube list*, 22nd ed.; Gastec Co., 2015.
(32) *Gas detection tubes and sampling handbook*, 2nd ed.; RAE Systems Inc., 2013.
(33) *Sensidyne colorimetric gas detector tube handbook*; Sensidyne, LP, 2013.
(34) Qin, X.; Wang, R.; Tsow, F.; Forzani, E.; Xian, X.; Tao, N. A colorimetric chemical sensing platform for real-time monitoring of indoor formaldehyde. *IEEE Sens. J.* **2015**, *15* (3), 1545−1551.
(35) Worrall, A. D.; Qian, Z.; Bernstein, J. A.; Angelopoulos, A. P. Water-resistant polymeric acid membrane catalyst for acetone detection in the exhaled breath of diabetics. *Anal. Chem.* **2018**, *90* (3), 1819−1826.
(36) Rakow, N. A.; Suslick, K. S. A colorimetric sensor array for odour visualization. *Nature* **2000**, *406* (6797), 710−713.
(37) Rakow, N. A.; Sen, A.; Janzen, M. C.; Ponder, J. B.; Suslick, K. S. Molecular recognition and discrimination of amines with a colorimetric array. *Angew. Chem., Int. Ed.* **2005**, *44* (29), 4528−4532.
(38) Bridgeman, D.; Corral, J.; Quach, A.; Xian, X.; Forzani, E. Colorimetric humidity sensor based on liquid composite materials for the monitoring of food and pharmaceuticals. *Langmuir* **2014**, *30* (35), 10785−10791.
(39) Askim, J. R.; Mahmoudi, M.; Suslick, K. S. Optical sensor arrays for chemical sensing: The optoelectronic nose. *Chem. Soc. Rev.* **2013**, *42* (22), 8649−8682.
(40) Janzen, M. C.; Ponder, J. B.; Bailey, D. P.; Ingison, C. K.; Suslick, K. S. Colorimetric sensor arrays for volatile organic compounds. *Anal. Chem.* **2006**, *78* (11), 3591−3600.

(41) Feng, L.; Musto, C. J.; Kemling, J. W.; Lim, S. H.; Zhong, W.; Suslick, K. S. colorimetric sensor array for determination and identification of toxic industrial chemicals. *Anal. Chem.* **2010**, *82* (22), 9433−9440.

(42) Li, Z.; Li, H.; LaGasse, M. K.; Suslick, K. S. Rapid quantification of trimethylamine. *Anal. Chem.* **2016**, *88* (11), 5615−5620.

(43) Ohira, S.-I.; Dasgupta, P. K.; Schug, K. A. Fiber optic sensor for simultaneous determination of atmospheric nitrogen dioxide, ozone, and relative humidity. *Anal. Chem.* **2009**, *81* (11), 4183−4191.

(44) Kim, J.; Yoo, H.; Ba, V. A. P.; Shin, N.; Hong, S. Dye-functionalized sol-gel matrix on carbon nanotubes for refreshable and flexible gas sensors. *Sci. Rep.* **2018**, *8*, 1 DOI: 10.1038/s41598-018-30481-y.

(45) Jang, J.; Kang, K.; Raeis-Hosseini, N.; Ismukhanova, A.; Jeong, H.; Jung, C.; Kim, B.; Lee, J.-Y.; Park, I.; Rho, J. Self-powered humidity sensor using chitosan-based plasmonic metal-hydrogel-metal filters. *Adv. Opt. Mater.* **2020**, *8* (9), 2070038.

(46) Mills, A.; Lepre, A.; Wild, L. Breath-by-breath measurement of carbon dioxide using a plastic film optical sensor. *Sens. Actuators, B* **1997**, *39* (1−3), 419−425.

(47) Volatile organic compounds' impact on indoor air quality; https://www.epa.gov/indoor-air-quality-iaq/volatile-organic-compounds-impact-indoor-air-quality.

(48) Criteria air pollutants; https://www.epa.gov/criteria-air-pollutants.

(49) Toxic industrial chemicals (TICs) guide; https://www.osha.gov/SLTC/emergencypreparedness/guides/chemical.html.

(50) Lin, C.; Zhu, Y.; Yu, J.; Qin, X.; Xian, X.; Tsow, F.; Forzani, E. S.; Wang, D.; Tao, N. Gradient-based colorimetric sensors for continuous gas monitoring. *Anal. Chem.* **2018**, *90* (8), 5375−5380.

(51) Kring, E. V.; Lautenberger, W. J.; Baker, W. B.; Douglas, J. J.; Hoffman, R. A. A new passive colorimetric air monitoring badge system for ammonia, sulfur-dioxide and nitrogen-dioxide. *Am. Ind. Hyg. Assoc. J.* **1981**, *42* (5), 373−381.

(52) Lim, S. H.; Feng, L.; Kemling, J. W.; Musto, C. J.; Suslick, K. S. An optoelectronic nose for the detection of toxic gases. *Nat. Chem.* **2009**, *1* (7), 562.

(53) Feng, L.; Musto, C. J.; Kemling, J. W.; Lim, S. H.; Suslick, K. S. A colorimetric sensor array for identification of toxic gases below permissible exposure limits. *Chem. Commun.* **2010**, *46* (12), 2037−2039.

(54) Guo, Y.; Xue, S.; Dirtu, M. M.; Garcia, Y. A versatile iron(II)-based colorimetric sensor for the vapor-phase detection of alcohols and toxic gases. *J. Mater. Chem. C* **2018**, *6* (15), 3895−3900.

(55) Yue, G.; Su, S.; Li, N.; Shuai, M.; Lai, X.; Astruc, D.; Zhao, P. Gold nanoparticles as sensors in the colorimetric and fluorescence detection of chemical warfare agents. *Coord. Chem. Rev.* **2016**, *311*, 75−84.

(56) Lee, J.; Seo, S.; Kim, J. Colorimetric detection of warfare gases by polydiacetylenes toward equipment-free detection. *Adv. Funct. Mater.* **2012**, *22* (8), 1632−1638.

(57) Chulvi, K.; Gavina, P.; Costero, A. M.; Gil, S.; Parra, M.; Gotor, R.; Royo, S.; Martinez-Manez, R.; Sancenon, F.; Vivancos, J.-L. Discrimination of nerve gases mimics and other organophosphorous derivatives in gas phase using a colorimetric probe array. *Chem. Commun.* **2012**, *48* (81), 10105−10107.

(58) Li, Z.; Bassett, W. P.; Askim, J. R.; Suslick, K. S. Differentiation among peroxide explosives with an optoelectronic nose. *Chem. Commun.* **2015**, *51* (83), 15312−15315.

(59) Askim, J. R.; Li, Z.; LaGasse, M. K.; Rankin, J. M.; Suslick, K. S. An optoelectronic nose for identification of explosives. *Chemical Science* **2016**, *7* (1), 199−206.

(60) Li, Z.; Suslick, K. S. Portable optoelectronic nose for monitoring meat freshness. *ACS Sensors* **2016**, *1* (11), 1330−1335.

(61) Zhang, C.; Bailey, D. P.; Suslick, K. S. Colorimetric sensor arrays for the analysis of beers: A feasibility study. *J. Agric. Food Chem.* **2006**, *54* (14), 4925−4931.

(62) Kawamura, K.; Vestergaard, M.; Ishiyama, M.; Nagatani, N.; Hashiba, T.; Tamiya, E. Development of a novel hand-held toluene gas sensor: Possible use in the prevention and control of sick building syndrome. *Measurement* **2006**, *39* (6), 490−496.

(63) Suslick, K. S.; Rakow, N. A.; Sen, A. Colorimetric sensor arrays for molecular recognition. *Tetrahedron* **2004**, *60* (49), 11133−11138.

(64) Feng, L.; Musto, C. J.; Suslick, K. S. A simple and highly sensitive colorimetric detection method for gaseous formaldehyde. *J. Am. Chem. Soc.* **2010**, *132* (12), 4046−4047.

(65) Lim, S. H.; Kemling, J. W.; Feng, L.; Suslick, K. S. A colorimetric sensor array of porous pigments. *Analyst* **2009**, *134* (12), 2453−2457.

(66) Xiao-Wei, H.; Zhi-Hua, L.; Xiao-Bo, Z.; Ji-Yong, S.; Han-Ping, M.; Jie-Wen, Z.; Li-Min, H.; Holmes, M. Detection of meat-borne trimethylamine based on nanoporous colorimetric sensor arrays. *Food Chem.* **2016**, *197*, 930−936.

(67) LaGasse, M. K.; Rankin, J. M.; Askim, J. R.; Suslick, K. S. Colorimetric sensor arrays: Interplay of geometry, substrate and immobilization. *Sens. Actuators, B* **2014**, *197*, 116−122.

(68) Wang, Y.; Zhong, X.; Huo, D.; Zhao, Y.; Geng, X.; Fa, H.; Luo, X.; Yang, M.; Hou, C. Fast recognition of trace volatile compounds with a nanoporous dyes-based colorimetric sensor array. *Talanta* **2019**, *192*, 407−417.

(69) Zhong, X.; Huo, D.; Fa, H.; Luo, X.; Wang, Y.; Zhao, Y.; Hou, C. Rapid and ultrasensitive detection of biogenic amines with colorimetric sensor array. *Sens. Actuators, B* **2018**, *274*, 464−471.

(70) Dunbar, R. A.; Jordan, J. D.; Bright, F. V. Development of chemical sensing platforms based on sol-gel-derived thin films: Origin of film age vs performance trade offs. *Anal. Chem.* **1996**, *68* (4), 604−610.

(71) Jeronimo, P. C. A.; Araujo, A. N.; Montenegro, M. Optical sensors and biosensors based on sol-gel films. *Talanta* **2007**, *72* (1), 13−27.

(72) Lin, H.; Suslick, K. S. A colorimetric sensor array for detection of triacetone triperoxide vapor. *J. Am. Chem. Soc.* **2010**, *132* (44), 15519−15521.

(73) Lim, S. H.; Musto, C. J.; Park, E.; Zhong, W.; Suslick, K. S. A colorimetric sensor array for detection and identification of sugars. *Org. Lett.* **2008**, *10* (20), 4405−4408.

(74) Musto, C. J.; Lim, S. H.; Suslick, K. S. Colorimetric detection and identification of natural and artificial sweeteners. *Anal. Chem.* **2009**, *81* (15), 6526−6533.

(75) Courbat, J.; Briand, D.; Damon-Lacoste, J.; Wollenstein, J.; de Rooij, N. F. Evaluation of pH indicator-based colorimetric films for ammonia detection using optical waveguides. *Sens. Actuators, B* **2009**, *143* (1), 62−70.

(76) Kim, Y. H.; Yang, Y. J.; Kim, J. S.; Choi, D. S.; Park, S. H.; Jin, S. Y.; Park, J. S. Non-destructive monitoring of apple ripeness using an aldehyde sensitive colorimetric sensor. *Food Chem.* **2018**, *267*, 149−156.

(77) Buenger, D.; Topuz, F.; Groll, J. Hydrogels in sensing applications. *Prog. Polym. Sci.* **2012**, *37* (12), 1678−1719.

(78) Devadhasan, J. P.; Kim, D.; Lee, D. Y.; Kim, S. Smartphone coupled handheld array reader for real-time toxic gas detection. *Anal. Chim. Acta* **2017**, *984*, 168−176.

(79) Maruo, Y. Y.; Kunioka, T.; Akaoka, K.; Nakamura, J. Development and evaluation of ozone detection paper. *Sens. Actuators, B* **2009**, *135* (2), 575−580.

(80) de Lacy Costello, B; Amann, A; Al-Kateb, H; Flynn, C; Filipiak, W; Khalid, T; Osborne, D; Ratcliffe, N M A review of the volatiles from the healthy human body. *J. Breath Res.* **2014**, *8* (1), 014001.

(81) Pereira, J.; Porto-Figueira, P.; Cavaco, C.; Taunk, K.; Rapole, S.; Dhakne, R.; Nagarajaram, H.; Câmara, J. S. Breath analysis as a potential and non-invasive frontier in disease diagnosis: an overview. *Metabolites* **2015**, *5* (1), 3−55.

(82) Piiper, J.; Scheid, P. Blood gas equilibration in lungs. In *Pulmonary Gas Exchange, Vol. I: Ventilation, Blood Flow, and Diffusion*, West, J. B., Ed.; Academic Press, Inc.: New York, 1980; pp 131−163.

(83) Cao, W.; Duan, Y. Breath analysis: Potential for clinical diagnosis and exposure assessment. *Clin. Chem.* **2006**, *52* (5), 800−811.

(84) Di Francesco, F.; Fuoco, R.; Trivella, M.G.; Ceccarini, A. Ceccarini, Breath analysis: Trends in techniques and clinical applications. *Microchem. J.* **2005**, *79* (1−2), 405−410.

(85) Kharitonov, S. A.; Barnes, P. J. Exhaled markers of pulmonary disease. *Am. J. Respir. Crit. Care Med.* **2001**, *163* (7), 1693−1722.

(86) Smith, D.; Spanel, P. Application of ion chemistry and the SIFT technique to the quantitative analysis of trace gases in air and on breath. *Int. Rev. Phys. Chem.* **1996**, *15* (1), 231−271.

(87) Spanel, P.; Smith, D. Progress in sift-ms: Breath analysis and other applications. *Mass Spectrom. Rev.* **2011**, *30* (2), 236−267.

(88) Patterson, C. S.; McMillan, L. C.; Longbottom, C.; Gibson, G. M.; Padgett, M. J.; Skeldon, K. D. Portable optical spectroscopy for accurate analysis of ethane in exhaled breath. *Meas. Sci. Technol.* **2007**, *18* (5), 1459−1464.

(89) Righettoni, M.; Tricoli, A. Toward portable breath acetone analysis for diabetes detection. *J. Breath Res.* **2011**, *5* (3), 037109.

(90) Tisch, U.; Haick, H. Chemical sensors for breath gas analysis: The latest developments at the breath analysis summit 2013. *J. Breath Res.* **2014**, *8* (2), 027103.

(91) Mazzone, P. J.; Wang, X.-F.; Xu, Y.; Mekhail, T.; Beukemann, M. C.; Na, J.; Kemling, J. W.; Suslick, K. S.; Sasidhar, M. Exhaled breath analysis with a colorimetric sensor array for the identification and characterization of lung cancer. *J. Thorac. Oncol.* **2012**, *7* (1), 137−142.

(92) Zhong, X.; Li, D.; Du, W.; Yan, M.; Wang, Y.; Huo, D.; Hou, C. Rapid recognition of volatile organic compounds with colorimetric sensor arrays for lung cancer screening. *Anal. Bioanal. Chem.* **2018**, *410* (16), 3671−3681.

(93) Effros, R. M.; Biller, J.; Foss, B.; Hoagland, K.; Dunning, M. B.; Castillo, D.; Bosbous, M.; Sun, F.; Shaker, R. A simple method for estimating respiratory solute dilution in exhaled breath condensates. *Am. J. Respir. Crit. Care Med.* **2003**, *168* (12), 1500−1505.

(94) Horvath, I.; Hunt, J.; Barnes, P. J. Exhaled breath condensate: methodological recommendations and unresolved questions. *Eur. Respir. J.* **2005**, *26* (3), 523−548.

(95) Yu, J.; Qin, X.; Xian, X.; Tao, N. Oxygen sensing based on the yellowing of newspaper. *ACS Sensors* **2018**, *3* (1), 160−166.

(96) Zhao, D.; Miller, D.; Shao, D.; Xian, X.; Tsow, F.; Iglesias, R. A.; Forzani, E. S. A personal device for analyzing carbon dioxide in real time and real breath: Experimental investigation and computational simulation. *Sens. Actuators, B* **2013**, *183*, 627−635.

(97) Rabitsch, W.; Nikolic, A.; Schellongowski, P.; Kofler, J.; Kraft, P.; Krenn, C. G.; Staudinger, T.; Locker, G. J.; Knöbl, P.; Hofbauer, R. Evaluation of an end-tidal portable ETCO2 colorimetric breath indicator (COLIBRI). *American Journal of Emergency Medicine* **2004**, *22* (1), 4−9.

(98) Mills, A.; Chang, Q.; McMurray, N. Equilibrium studies on colorimetric plastic film sensors for carbon-dioxide. *Anal. Chem.* **1992**, *64* (13), 1383−1389.

(99) Tassopoulos, C. N.; Barnett, D.; Fraser, T. R. Breath-acetone and blood-sugar measurements in diabetes. *Lancet* **1969**, *293* (7609), 1282.

(100) Saasa, V.; Beukes, M.; Lemmer, Y.; Mwakikunga, B. Blood ketone bodies and breath acetone analysis and their correlations in type 2 diabetes mellitus. *Diagnostics* **2019**, *9* (4), 224.

(101) Worrall, A. D.; Bernstein, J. A.; Angelopoulos, A. P. Portable method of measuring gaseous acetone concentrations. *Talanta* **2013**, *112*, 26−30.

(102) Smith, A. D.; Cowan, J. O.; Filsell, S.; McLachlan, C.; Monti-Sheehan, G.; Jackson, P.; Taylor, D. R. Diagnosing asthma - Comparisons between exhaled nitric oxide measurements and conventional tests. *Am. J. Respir. Crit. Care Med.* **2004**, *169* (4), 473−478.

(103) Smith, A. D.; Cowan, J. O.; Brassett, K. P.; Herbison, G. P.; Taylor, D. R. Use of exhaled nitric oxide measurements to guide treatment in chronic asthma. *N. Engl. J. Med.* **2005**, *352* (21), 2163−2173.

(104) Shiba, S.; Yamada, K.; Matsuguchi, M. Humidity-resistive optical NO gas sensor devices based on cobalt tetraphenylporphyrin dispersed in hydrophobic polymer matrix. *Sensors* **2020**, *20* (5), 1295.

(105) Prabhakar, A.; Iglesias, R. A.; Wang, R.; Tsow, F.; Forzani, E. S.; Tao, N. Ultrasensitive detection of nitrogen oxides over a nanoporous membrane. *Anal. Chem.* **2010**, *82* (23), 9938−9940.

(106) Toombs, C. F.; Insko, M. A.; Wintner, E. A.; Deckwerth, T. L.; Usansky, H.; Jamil, K.; Goldstein, B.; Cooreman, M.; Szabo, C. Detection of exhaled hydrogen sulphide gas in healthy human volunteers during intravenous administration of sodium sulphide. *Br. J. Clin. Pharmacol.* **2010**, *69* (6), 626−636.

(107) Carpenter, T. S.; Rosolina, S. M.; Xue, Z.-L. Quantitative, colorimetric paper probe for hydrogen sulfide gas. *Sens. Actuators, B* **2017**, *253*, 846−851.

(108) Jornet-Martinez, N.; Hakobyan, L.; Argente-Garcia, A. I.; Molins-Legua, C.; Campíns-Falcó, P. Nylon-supported plasmonic assay based on the aggregation of silver nanoparticles: In situ determination of hydrogen sulfide-like compounds in breath samples as a proof of concept. *ACS Sensors* **2019**, *4* (8), 2164−2172.

(109) Krishnan, S. T.; Son, K. H.; Kim, N.; Viswanath, B.; Kim, S.; An, J. H. Development of simple and sensitive hydrogel based colorimetric sensor array for the real-time quantification of gaseous ammonia. *Mater. Sci. Eng., C* **2017**, *72*, 583−589.

(110) Krishnan, S. T.; Devadhasan, J. P.; Kim, S. Recent analytical approaches to detect exhaled breath ammonia with special reference to renal patients. *Anal. Bioanal. Chem.* **2017**, *409* (1), 21−31.

(111) Limeres, J.; Garcez, J. F.; Marinho, J. S.; Loureiro, A.; Diniz, M.; Diz, P. A breath ammonia analyser for monitoring patients with end-stage renal disease on haemodialysis. *Br. J. Biomed. Sci.* **2017**, *74* (1), 24−29.

(112) Ito, K.; Kawamura, N.; Suzuki, Y.; Maruo, Y. Colorimetric detection of gaseous acetone based on a reaction between acetone and 4-nitrophenylhydrazine in porous glass. *Microchem. J.* **2020**, *159*, 105428.

(113) Prabhakar, A.; Iglesias, R. A.; Shan, X.; Xian, X.; Zhang, L.; Tsow, F.; Forzani, E. S.; Tao, N. Online sample conditioning for portable breath analyzers. *Anal. Chem.* **2012**, *84* (16), 7172−7178.

(114) Mazzone, P. J.; Wang, X.-F.; Lim, S.; Jett, J.; Choi, H.; Zhang, Q.; Beukemann, M.; Seeley, M.; Martino, R.; Rhodes, P. Progress in the development of volatile exhaled breath signatures of lung cancer. *Ann. Am. Thorac. Soc.* **2015**, *12* (5), 752−757.

(115) American Thoracic Society; European Respiratory Society. ATS/ERS recommendations for standardized procedures for the online and offline measurement of exhaled lower respiratory nitric oxide and nasal nitric oxide, 2005. *Am. J. Respir. Crit. Care Med.* **2005**, *171* (8), 912−930.

https://dx.doi.org/10.1021/acssensors.0c01644
ACS Sens. 2021, 6, 303−320

nanomaterials

MDPI

Article

Effect of Humidity on Light-Activated NO and NO_2 Gas Sensing by Hybrid Materials

Abulkosim Nasriddinov [1,2], Marina Rumyantseva [1,*], Elizaveta Konstantinova [3,4,5], Artem Marikutsa [1], Sergey Tokarev [1,6], Polina Yaltseva [1], Olga Fedorova [1,6] and Alexander Gaskov [1]

1 Chemistry Department, Moscow State University, 119991 Moscow, Russia; naf_1994@mail.ru (A.N.); artem.marikutsa@gmail.com (A.M.); pergeybokarev@gmail.com (S.T.); yal-polina@yandex.ru (P.Y.); fedorova@ineos.ac.ru (O.F.); gaskov@inorg.chem.msu.ru (A.G.)
2 Faculty of Materials Science, Moscow State University, 119991 Moscow, Russia
3 Physics Department, Moscow State University, 119991 Moscow, Russia; liza35@mail.ru
4 Faculty of nano-, bio-, information and cognitive technologies, Moscow Institute of Physics and Technology, Dolgoprudny, 141700 Moscow Region, Russia
5 National Research Center "Kurchatov Institute", 123182 Moscow, Russia
6 A.N. Nesmeyanov Institute of Organoelement Compounds RAS, 119991 Moscow, Russia
* Correspondence: roum@inorg.chem.msu.ru; Tel.: +7-495-939-5471

Received: 1 April 2020; Accepted: 30 April 2020; Published: 9 May 2020

check for updates

Abstract: Air humidity is one of the main factors affecting the characteristics of semiconductor gas sensors, especially at low measurement temperatures. In this work we analyzed the influence of relative humidity on sensor properties of the hybrid materials based on the nanocrystalline SnO_2 and In_2O_3 and Ru (II) heterocyclic complex and verified the possibility of using such materials for NO (0.25–4.0 ppm) and NO_2 (0.05–1.0 ppm) detection in high humidity conditions (relative humidity (RH) = 20%, 40%, 65%, 90%) at room temperature during periodic blue (λ_{max} = 470 nm) illumination. To reveal the reasons for the different influence of humidity on the sensors' sensitivity when detecting NO and NO_2, electron paramagnetic resonance (EPR) spectroscopy and diffuse reflectance infrared Fourier transform spectroscopy (DRIFTS) investigations were undertaken. It was established that the substitution of adsorbed oxygen by water molecules causes the decrease in sensor response to NO in humid air. The influence of humidity on the interaction of sensitive materials with NO_2 is determined by the following factors: the increase in charge carrier's concentration, the decrease in the number of active sites capable of interacting with gases, and possible substitution of chemisorbed oxygen with NO_2^- groups.

Keywords: organic–inorganic hybrid materials; tin dioxide; indium oxide; Ru(II) complex; nitrogen monoxide NO; nitrogen dioxide NO_2; semiconductor gas sensor; humidity effect

1. Introduction

Nitric oxide (NO) and nitrogen dioxide (NO_2) gases produced by fuel combustion, electric power plant boilers and industrial plants [1,2] pose a serious problem for both the environment and human health [3,4]. On the other hand, several investigations showed that nitric oxide in exhaled samples of breath condensate can indicate lung diseases and respiratory tract inflammation [5–8]. An early exhaled NO test will enable the correct diagnosis to guide therapy and prevent progression of the disease. The difficulty of quantifying NO in exhaled air is due to the low level of its concentration (20–200 ppb) against the background of a wide range of interfering impurities (CO, CO_2, acetone, etc.), as well as high relative humidity (RH) in the range of 90–100%.

Currently, the most reliable method for quantitative analysis of NO is based on the reaction between NO and ozone O_3, accompanied by chemiluminescence, the intensity of which is proportional to the NO content in the sample. Chemiluminescence occurs in the infrared (IR) range when excited electrons in the NO_2 molecule pass to lower energy levels.

Direct determination of nitric oxide in the gas phase is possible using semiconductor gas sensors. The advantages of gas sensors in comparison with methods of chemiluminescent analysis, mass spectrometry, gas chromatography, etc. are the ability to obtain results in real time without sample processing, small size, low power consumption, relatively low price. The analysis of the exhaled air can be performed directly by the patient without the participation of specially trained personnel. Therefore, the sensors used for this application have to be stable in ambient humidity, sensitive and selective to NO in the ppb range.

The humidity of the environment is one of the main factors affecting the efficiency, device lifetime and long-term stability of semiconductor gas sensors. The influence of water vapor on the characteristics of semiconductor gas sensors becomes especially significant at low temperatures, and this is most evident at room temperature. Most studies on the sensor properties of semiconductor metal oxides to NO gas in ambient humidity confirm the deterioration of the main sensor characteristics: response and recovery times, sensor signal, and sensitivity much more than toward NO_2. Moreover, in some cases, the enhancement of sensor signal is observed [9–13]. These results indicate the complex nature of the interaction of target gases with semiconductor oxide materials in the presence of water vapor. Further and deeper investigation of the gas-sensing mechanism in humid air, especially at room temperature, could prevent and avoid its negative impact.

Our previous studies [14,15] demonstrated high sensitivity and selectivity of photosensitized organic–inorganic hybrid materials toward NO and NO_2 detection at room temperature under visible light activation. In this work, we analyzed the influence of relative humidity on sensor properties of the hybrid materials based on nanocrystalline SnO_2 and In_2O_3 and Ru (II) heterocyclic complex as photosensitizer and verified the possibility of using such materials for NO and NO_2 detection in high ambient humidity conditions at room temperature during periodic blue light-emitting diode (LED, λ_{max} = 470 nm) illumination.

2. Materials and Methods

Nanocrystalline SnO_2 and In_2O_3 were synthesized by the chemical precipitation method and a heteroleptic Ru(II) complex was used as a photosensitizer. A detailed scheme of their synthesis is given in our previous work [14]. Hybrid materials were prepared by adsorption of Ru (II) complex on the surface of semiconductor oxides.

The composition and microstructure of the materials obtained were investigated by X-ray diffraction (XRD), Raman spectroscopy, scanning electron microscopy (SEM), high-resolution transmission electron microscopy (HRTEM), energy-dispersive X-ray (EDX) spectroscopy, X-ray photoelectron spectroscopy (XPS), and Brunauer–Emmett–Teller (BET) measurements; the optical properties and thermal stability were studied via ultraviolet/visible (UV/Vis) absorption spectroscopy and thermogravimetric analysis (TGA), respectively, and were discussed in detail previously [14]. Surface spin centers were studied by electron paramagnetic resonance (EPR) spectroscopy. The EPR spectra were recorded at 110 K to reduce thermally induced signal broadening by the Bruker ELEXSYS−580 spectrometer (X-band, sensitivity is ~10^{10} spin/G, Bruker, Billerica, MA, USA). The values of g-factors and spin center concentrations were calculated based on Mn^{2+} and $CuCl_2 \cdot 2H_2O$ standard samples, respectively. Additionally, SnO_2 sample modified with Ru was used in order to understand the influence of the Ru on concentration of the surface radicals. Ruthenium acetylacetonate (Sigma-Aldrich, >97%, St. Louis, MO, USA) with a concentration of 1% (weight) per metal was used as a precursor. Chemical modification of nanocrystalline SnO_2 was carried out by impregnation with the alcohol solution of $Ru(acac)_3$. The obtained powder was annealed for 24 h at the lowest temperature necessary for decomposition of precursor (T = 265 °C).

Samples characteristics (composition, microstructure parameters, photoresponse in dry pure air) are summarized in Table 1.

Table 1. Sensor characteristics of synthesized materials.

Sample	Phase Composition	d_{XRD} [1], nm	d_{TEM} [2], nm	S_{surf} [3], m^2/g	Average Pore Diameter, nm	$\frac{[Ru]}{[Ru]+[M]}$ [4], *at.* %	R_{av} [5], Ohm Pure Air	S_{Ph} [6], in Pure Air (λ = 470 nm)
SnO_2	SnO_2, cassiterite	4 ± 1	4 ± 1	115 ± 5	3–5; 70–80	-	7.8·10[4]	1.00
SnO_2+RuITP		7 ± 1	7 ± 2	90 ± 5	3–4	1.2 ± 0.1	6.9·10[5]	1.22
In_2O_3	In_2O_3, bixbyite					-	1.7·10[4]	1.25
In_2O_3+RuITP						2.1 ± 0.2	5.8·10[5]	1.95

[1] crystallite size from X-ray diffraction (XRD); [2] particle size from transmission electron microscopy (TEM); [3] specific surface area; [4] obtained by energy-dispersive X-ray spectroscopy (EDX) on thick films: M = Sn for SnO_2+RuITP sample; M = In for In_2O_3+RuITP sample; [5] resistances; [6] effective photoresponse.

In order to study the reactivity of the hybrid materials in an atmosphere of NO and NO_2, in situ diffuse reflectance infrared Fourier transform spectroscopy (DRIFTS) was carried out on a Perkin-Elmer Spectrum One Fourier Transform Infrared spectrometer (Perkin Elmer Inc., Beaconsfield, UK) at room temperature. The spectra were registered in the range of 4000–1000 cm^{-1} with a resolution of 4 cm^{-1} and accumulation of 30 scans under a controlled gas flow rate of 100 mL/min. The samples were preheated at 50 °C for 1 h and cooled down to 25 °C in dry air. The gas mixtures containing 5 ppm of NO_2 or 50 ppm of NO were prepared by dilution of certified gas mixtures 10 ± 1 ppm of NO_2 in N_2 or 100 ± 5 ppm of NO in N_2, respectively (Monitoring, St. Petersburg, Russia). The purified air from a pure air generator (Granat-Engineering Co. Ltd., Moscow, Russia) was used as background and carrier gas.

For gas sensor measurements, the sensitive materials were deposited on specially designed dielectric micro-hotplates (Al_2O_3), covered with Pt contact electrodes for resistance measurements and with a Pt heater. The square-shape micro-hotplates with dimensions of 0.9 × 0.9 × 0.15 mm were fully covered by the samples. The thickness of the films, estimated from the preliminary calibration carried out by scanning electron microscopy, was about 1 µm. Direct current DC measurements have been carried out at room temperature under constant flux of 100 mL/min NO/air (0.25–4.0 ppm NO in dry air) or NO_2/air (0.05–1.0 ppm NO_2 in dry air) gas mixtures in dry and humid air (RH = 20–90%) under periodic blue (λ_{max} = 470 nm) light illumination. In this procedure the illumination of the sensor element is carried out in a pulsed mode with a short period (2 min "on"–2 min "off"). The steady state can be characterized by the minimum resistance R_{light}, which is achieved during the sensor illumination, and the maximum R_{dark}, which is achieved in the dark period. The resistive response can be calculated as the ratio $S = R_{dark\,gas}/R_{dark\,air}$ of dark resistances (measured at the end of 2 min "light off" period) at a given NO_x concentration $R_{dark\,gas}$ and in pure air $R_{dark\,air}$. A relative humidity both in DRIFTS and sensor measurements was set and controlled by Humidifier P-2 (Cellkraft AB, Sweden). A detailed description of the sensor fabrication and measurements setup can be found in our previous works [14,15].

3. Results and Discussion

In this study the gas sensor measurements of the obtained materials were performed when detecting NO (0.25–4.0 ppm) and NO_2 (0.05–1.0 ppm) in dry and humid atmosphere (RH = 20%, 40%, 65%, 90%) at room temperature. The samples were illuminated with a blue LED (λ_{max} = 470 nm) in pulsed mode during 2 hours under dry air or in humid air (depending on the type of measurements) before the first measurement to reach a stable resistance value, and irradiation was kept during the whole experiment in gas phase.

Electrical resistance measurements are plotted in Figure 1a,b only for the SnO_2+RuITP and In_2O_3+RuITP hybrid materials, respectively, since all samples show a similar trend in the presence of both NO and NO_2 in a humid atmosphere: resistance increases with stepwise increasing concentration

of introduced gases, which is typical for *n*-type semiconductors in the presence of oxidizing gases. With an increase in the concentration of NO and NO_2, a slow and partial accumulation of adsorbates on the surface of materials occurs, and with a decrease in concentration it takes longer for the resistance to reach its initial stationary state, since photodesorption of residual adsorbates proceeds slowly. Therefore, the resistance values for the same concentrations with a stepwise increase and decrease in concentration do not coincide. In the ideal case they should be equal, but due to the kinetic inhibition of the processes at room temperature, they differ slightly at low concentrations. The same phenomenon may also be observed even in the case of gas sensors operating during dynamic thermal heating [16].

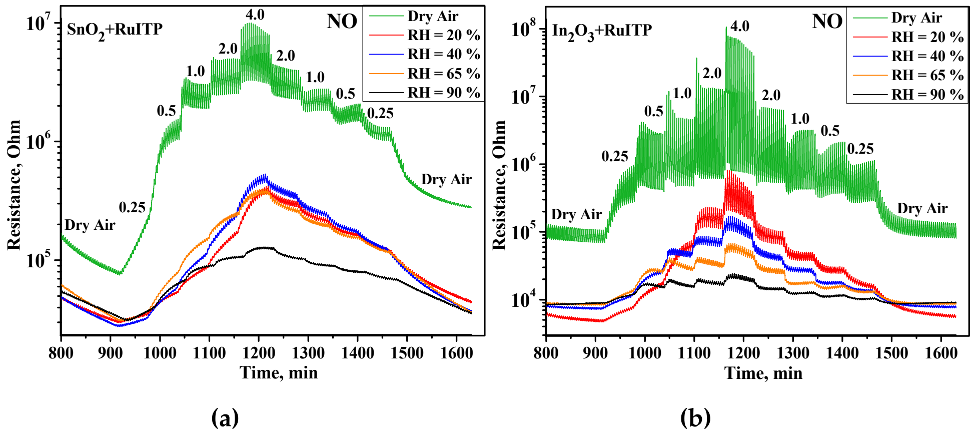

Figure 1. Room temperature electrical resistance change of the (**a**) SnO_2+RuITP and (**b**) In_2O_3+RuITP hybrid materials under periodic illumination during stepwise increase and decrease of the NO concentration in dry air and humid air.

The interaction of *n*-type semiconductor oxides with NO_2 in dry air accompanied by a decrease in electrical conductivity can be described with following reactions:

$$NO_{2(gas)} = NO_{2(ads)} \quad (1)$$

$$NO_{2(gas)} + e^- = NO_2^-{}_{(ads)} \quad (2)$$

$$2\,NO_{2(gas)} + \frac{1}{2}\,O_{2(gas)} + 2\,e^- = NO_2^-{}_{(ads)} + NO_3^-{}_{(ads)} \quad (3)$$

$$NO_2^-{}_{(ads)} + \frac{1}{2}\,O_{2(gas)} = NO_3^-{}_{(ads)} \quad (4)$$

At the same time NO sensing in dry air is determined by the oxidation with chemisorbed oxygen:

$$NO_{(gas)} + \frac{1}{2}\,O_{2(gas)} + e^- = NO_2^-{}_{(ads)}. \quad (5)$$

As discussed in [14], the interaction of NO and NO_2 with the surface of semiconductor oxides occurs in a similar way, through the mechanism of adsorption with the localization of electrons of the semiconductor conduction band. The lower sensitivity of hybrid materials to NO (Figure 2), compared to NO_2 (Figure 3), should be due to different initial steps in the detection routes. In reaction with NO_2, it is a simple one-electron reduction (reaction (2)) favored by the strong oxidative activity of nitrogen dioxide. The interaction with NO (reaction (5)) is essentially an oxidation of the target gas mediated by oxygen on the surface of the semiconductor oxide. This should be the main reason for the different sensitivities to NO_2 and NO, although the surface species produced in both interaction pathways are similar. Figures 1–3 show that an increase in relative humidity in the range 0–90% leads to a decrease in the baseline resistance of all materials, as well as to a change in the sensor signal when interacting with nitrogen oxides.

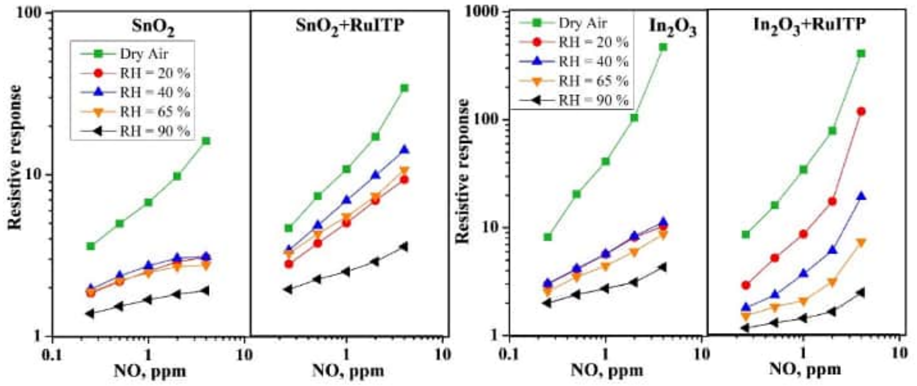

(a) (b)

Figure 2. Resistive response of (**a**) SnO_2, SnO_2+RuITP and (**b**) In_2O_3, In_2O_3+RuITP samples depending on NO concentration in dry and humid air at room temperature.

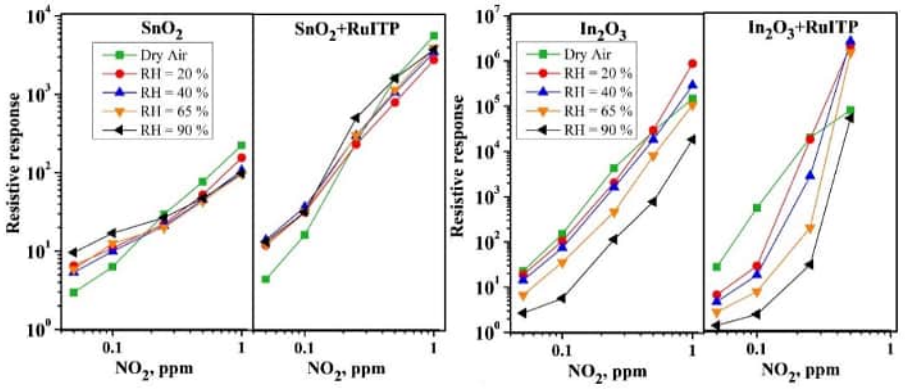

(a) (b)

Figure 3. Resistive response of (**a**) SnO_2, SnO_2+RuITP and (**b**) In_2O_3, In_2O_3+RuITP samples depending on NO_2 concentration in dry and humid air at room temperature.

The main trends in a change in the resistive response to NO and NO_2 depending on air humidity can be summarized as follows:

(i) When detecting NO for all samples with an increase in air humidity there is a decrease in the resistive response (Figure 2a,b);

(ii) For SnO_2-based samples, when detecting low NO_2 concentrations ($C(NO_2) < 0.25$ ppm), the resistive response increases with RH (Figure 3a,b);

(iii) For In_2O_3 based samples, with increasing humidity there is an increase in the resistive response when detecting high concentrations of NO_2 ($C(NO_2) \geq 0.5$ ppm). Note that for $C(NO_2) = 1$ ppm, the value of the dark resistance of In_2O_3+RuITP hybrid sample exceeds the upper measurement limit of the device used (10^{10} Ohm). Therefore, the point corresponding to $C(NO_2) = 1$ ppm for In_2O_3+RuITP hybrid sample is not shown in Figure 3.

The main contribution to the electrophysical properties of semiconductor gas sensors at room temperature in air is mainly made by adsorbed oxygen and water molecules. Temperature has a significant influence on the predominant form of chemisorbed oxygen, as well as the concentration of adsorbed oxygen and hydroxyl groups on the surface of semiconductor oxides, which determined the width of an electron-depleted layer near the surface of crystal grains of the semiconductor [17,18]. It was shown [19] that water is desorbed at temperatures above 100 °C, O_2 molecules—at temperatures above 250 °C, OH groups are removed when heated above 500 °C from the surface of SnO_2 films. Moreover, the density of adsorbed oxygen atoms increases in the temperature range from 200 °C to 500 °C, and then begins to decrease (desorption predominates). This means that molecular oxygen,

surface water and OH groups, on the one hand, and chemisorbed forms of oxygen, on the other hand, compete for the same active sites on the surface of metal oxides. At low operating temperatures at the surface of *n*-type semiconductor metal oxides, the dissociative adsorption of H_2O is energetically more preferable than dissociative adsorption of oxygen and surface becomes hydroxylated [20,21]. Moreover, different types of chemisorbed oxygen ions can exist at different temperature ranges: molecular (O_2^-) is formed at temperature lower than 150 °C and atomic (O^-, O^{2-})—at higher temperatures [17,19,22]. That is why in our experiments at room temperature in air flow we can assume that a depletion layer at the surface of SnO_2 and In_2O_3 is mainly formed by O_2^- species.

There are several mechanisms explaining the increase in conductivity in humid air described in the literature [17,21]:

(i) During dissociative adsorption of H_2O isolated hydroxyl groups are formed due to the acid-base interaction of OH with the lattice Sn (Lewis acid). The released hydrogen atom reacts with the lattice or chemisorbed oxygen (Lewis base) with the formation of rooted hydroxyl group and injection of an electron into the conduction band [23,24]:

$$H_2O_{(gas)} + Sn_{(lat)} + O_{(lat)} = [Sn_{(lat)}\text{-}OH] + [O_{(lat)}H]\cdot + e^- \quad (6)$$

(ii) Another mechanism involves the formation of the OH groups, which bind to the Sn atom, and ionization of the oxygen vacancy, which provides additional electrons:

$$H_2O_{(gas)} + 2Sn_{(lat)} + O_{(lat)} = 2[Sn_{(lat)}\text{-}OH] + V_O\cdot + 2e^- \quad (7)$$

(iii) The electron affinity of the acceptor surface states can change after interaction with OH^- or H^+. Also chemisorbed oxygen ions can be replaced or rearranged by the water molecules [25,26]:

$$H_2O_{(gas)} + 2Sn_{(lat)} + O_2{}^-{}_{(ads)} = 2[Sn_{(lat)}\text{-}OH] + e^- \quad (8)$$

To find out the reasons for the different influence of humidity on the sensor sensitivity of SnO_2 and In_2O_3 semiconductor oxides and hybrid samples when detecting NO and NO_2, EPR and DRIFTS investigations were undertaken. The influence of the relative humidity on the concentration of the surface radicals was investigated by EPR spectroscopy. Figure 4a,b shows the EPR spectra of SnO_2 and SnO_2, modified with Ru. The EPR signal with an intense central line (g = 2.0030 ± 0.0005) and weak lateral satellites (g = 2.0210 ± 0.0005 and g = 1.9833 ± 0.0005), according to literature [27,28], belong to O_2^- radicals.

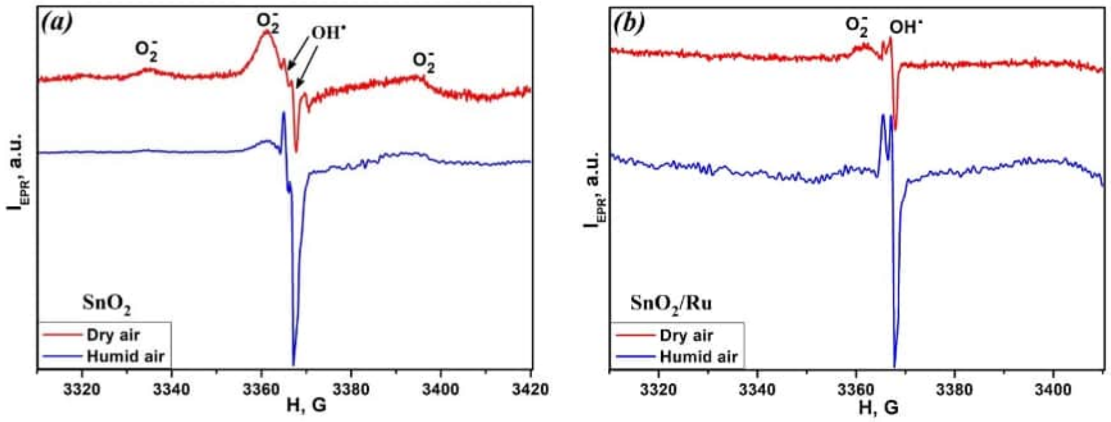

(**a**) (**b**)

Figure 4. Electron paramagnetic resonance (EPR) spectra of SnO_2 (**a**) and SnO_2/Ru (**b**) in dry air and after exposure to humid air.

An EPR signal with parameters g = 2.0021 ± 0.0005 and g = 2.0009 ± 0.0005 for tin dioxide based materials is not described in the literature. However, it is known that for TiO_2 sample the EPR signal with close g-factors belongs to OH· radicals [27,29,30]. The additional EPR measurements were carried out for samples differing in the degree of surface hydration to check out the hypothesis of the formation of hydroxyl radicals on the surface of SnO_2. Hydrated samples were obtained by passing saturated water vapor over SnO_2 and SnO_2/Ru powders during 24 h. It can be seen from Figure 4a,b that the EPR spectra of samples processed by this method demonstrate an increase in signal intensity and concentration with g = 2.0021 ± 0.0005 and g = 2.0009 ± 0.0005. Thus, the nature of this signal is not in doubt and corresponds to OH· radicals. Note that the intensity of the EPR signal from O_2^- radicals (and, accordingly, their concentration) in hydrated samples decreased. The concentrations of O_2^- and OH· radicals on the surface of SnO_2 and SnO_2/Ru samples in dry and humid conditions presented in Table 2 indicate that O_2^- radicals can be easily replaced by OH· radicals in a humid atmosphere. Changes in the ratio of OH·/O_2^- concentrations in dry and humid air for SnO_2 and SnO_2/Ru are close and are 6.5 and 6.3, respectively.

Table 2. Concentrations (spin/g) of O_2^- and OH· radicals on the surface of SnO_2 and SnO_2/Ru samples in dry and humid conditions.

Conditions	SnO_2			SnO_2/Ru		
	O_2^-	OH·	OH·/O_2^-	O_2^-	OH·	OH·/O_2^-
Dry air	8×10^{14}	1.9×10^{14}	0.24	2.5×10^{14}	4.4×10^{14}	1.2
Humid air	2.8×10^{14}	4.3×10^{14}	1.54	10^{14}	1.1×10^{15}	11

Figure 5 shows the DRIFT spectra of the nanocrystalline SnO_2 and In_2O_3 and hybrid materials after 1 h interaction with NO (50 ppm) and NO_2 (5 ppm) in dry and humid air (RH = 65%) at room temperature. There are a lot of IR bands in dry air comparing with the humid atmosphere. Moreover, the appearance of nearly identical absorption IR bands corresponding to NO_x^- groups upon adsorption of NO and NO_2 indicates an almost similar interaction nature of these two molecules with the surface of SnO_2 and In_2O_3 semiconductors. Recently, the same results were obtained for nanocrystalline WO_3 with different particle size by the authors of [31] and for SnO_2-based sensors in [32].

The bands assignments observed upon interaction of NO and hybrid samples have been fully discussed in our previous paper [14]. Additional bands observed during interaction of NO_2 with analyzing samples in dry and humid air are summarized in Table S1 (Supplementary Information) [32–35].

The most intense bands at the 1145–1205 cm^{-1} and 1205–1220 cm^{-1} regions correspond to chelating and bridging bidentate NO_2^- groups. The nitrate species NO_3^- can be assumed at the wide range of frequency: 970–1040, 1180–1600 and 1260–1300 cm^{-1}. The bands between 1605 and 1680 cm^{-1} are attributed to adsorbed NO_2, which can be overlapped with bending H_2O vibration modes. Adsorbed NO_2 molecules were obtained during adsorption of NO, NO+O_2 or NO_2 on the surface of different catalysts and metal oxides [36,37]. It is difficult for some peaks to exactly distinguish and determine the structure type of the adsorbed species, because different type of nitrites and nitrates have IR-activated bonds in the same wavenumber region.

For In_2O_3 and its hybrid sample, a broad band appears at 2110 cm^{-1} in a humid atmosphere in the presence of NO. This band has also been detected by other researchers [38] and has been assigned either to nitrosonium ion NO^+ or nitronium ion NO_2^+. However, most authors tend to assume that during the adsorption of NO/O_2 in the presence of moisture this band is attributed to NO^+ ion, which produces with the participation of the acidic hydroxyls, but NO_2^+ often arise after NO_2 adsorption [39,40]. Moreover, the appearance of this band correlates with the disappearance of the OH stretching band at 3610 cm^{-1}. There is an additional band at 1720 cm^{-1} appearing upon NO_2 adsorption in humid air on the surface of SnO_2+RuITP hybrid sample. This band is attributed to dinitrogen tetraoxide N_2O_4, which can be easily formed by NO_2 dimerization [41].

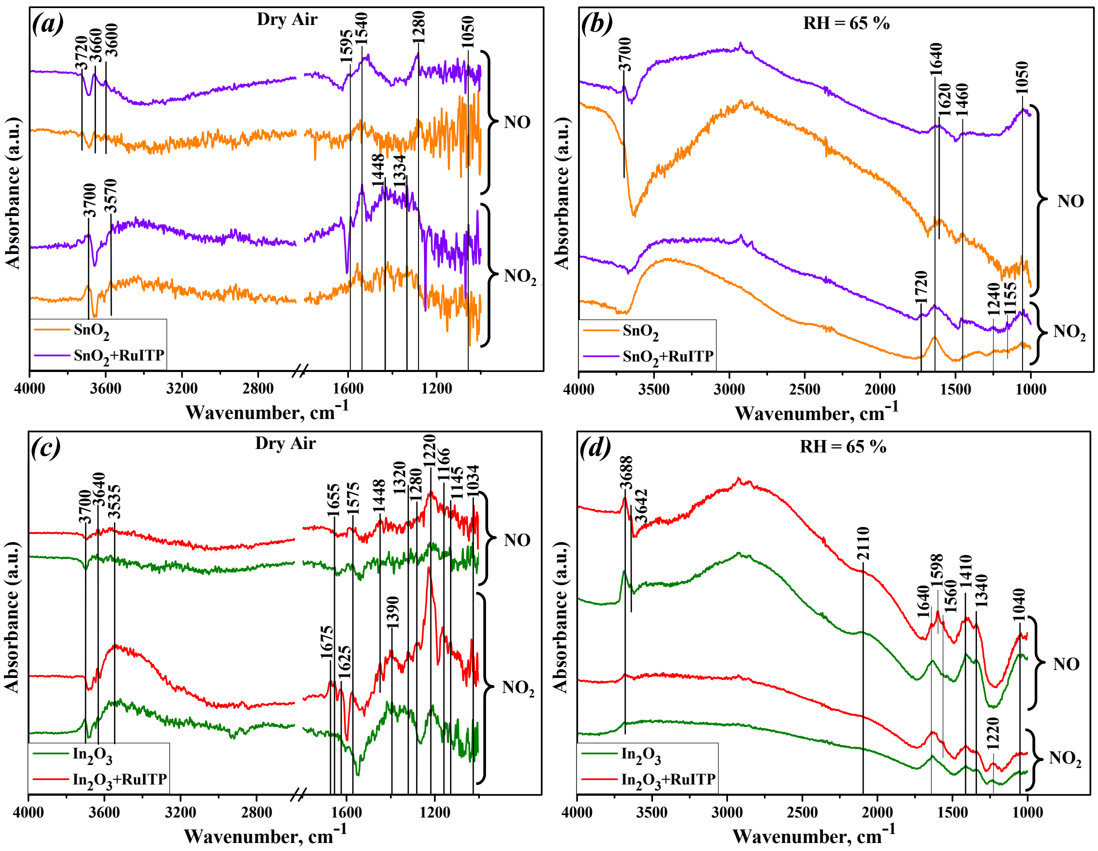

Figure 5. In situ diffuse reflectance infrared Fourier transform spectroscopy (DRIFT) spectra of the nanocrystalline SnO_2 and hybrid SnO_2+RuITP samples in dry (**a**) and humid (RH = 65%) air (**b**); nanocrystalline In_2O_3 and hybrid In_2O_3+RuITP samples in dry (**c**) and humid (RH = 65%) air (**d**) after 60 min exposure of the NO (50 ppm) and NO_2 (5 ppm) at room temperature.

The high wavenumber region is characterized by surface hydroxyl groups. The spectra in O-H stretching (3200–3700 cm^{-1}) and H–O–H bending (1610–1640 cm^{-1}) regions look similar for all samples. The negative sharp bands produced in these regions are associated with the interaction and/or replacement of hydroxyls by adsorbed NO_x^- species. The background of this frequency range of the spectrum increased in a humid atmosphere due to the adsorption of a large number of water molecules.

In dry gas phase the hybrid materials have additional and more intense bands than pure nanocrystalline metal oxides. Moreover, NO_2 adsorption compared to NO adsorption leads to an increase in the intensity of bands associated with nitrite and nitrate species. This effect can be explained through the following assumptions:

(i) Sensitization of SnO_2 and In_2O_3 semiconductors with a heterocyclic Ru (II) complex leads to an increase in the number of charge carriers (electrons) in the conduction band of semiconductor matrix, which enhance the interaction with the electrophylic NO_2 molecules;

(ii) NO_2 as a strong oxidizing gas will either capture electrons in direct competition with a greater advantage than oxygen, since its electron affinity is 5 times greater ($E_{ea}(NO_2)$ = 2.27 eV, E_{ea} (O_2) = 0.44 eV) and increases the sensor resistance; or react with the surface chemisorbed oxygen ions, or can replace it in the competitive adsorption (reactions 2 and 3);

(iii) The electron affinity of nitrogen monoxide (E_{ea}(NO) = 0.03 eV) is lower than that of both NO_2 and O_2 [42]. There is an unpaired electron on the antibonding 2π orbital of the NO molecule, thus it is difficult for it to accept an electron or replace the oxygen. That's why there wasn't appear any bands attributed to nitrosyl anion (NO^-) or NO dimers in NO adsorption spectra (Figure 5). On the other hand, the formation of nitrate and nitrite groups after the interaction of materials with NO

can be associated with the oxidation of nitrogen monoxide molecules by chemisorbed oxygen on the surface of semiconductor oxides [31]. G.X. et al. have investigated the NO adsorption and oxidation over various SnO_2 surfaces by density functional theory (DFT) calculations [43]. It was concluded that NO molecules are mainly adsorbed on the SnO_{2-x} (110) surface containing pre-adsorbed O_2 and oxidize to form NO_2^- species.

In a humid atmosphere a notable difference among spectra can be found: the band at 1220 cm^{-1} for In_2O_3 and In_2O_3+RuITP samples (Figure 5d), that corresponds to a bidentate nitrite group, has appeared only after NO_2 exposure and was not observed in the presence of NO. This band is less intense and shifted to the 1240 cm^{-1} position for SnO_2 and SnO_2+RuITP samples (Figure 5b). Considering the above position and the results of EPR studies, we can safely assume that in a humid atmosphere hydroxyl groups replace chemisorbed oxygen molecules, which play a key role in the oxidation of NO molecules. As a result, no absorption bands corresponding to bidentate nitrite groups are observed. Previously, Sergent et al. [44] have been investigated the influence of the different nitrite and nitrate species on the electrical response of nanocrystalline SnO_2 and TiO_2 powders and established that it is bidentate nitrate species that are responsible for the change in conductivity of SnO_2 and TiO_2 and sensor response to NO_2. Thus, for successful detection of low NO concentrations in a humid atmosphere, additional modification of the sensitive material is necessary, for example, by introducing an additional layer that acts as a filter to absorb water molecules, or by introducing an additional catalyst that ensures the oxidation of NO to NO_2.

4. Conclusions

The analysis of data obtained by EPR and DRIFTS methods, together with the dependence of the resistive response of samples in the presence of NO and NO_2 in dry and humid air, allows us to reach the following conclusions:

(i) When detecting NO, a decrease in the resistive response is observed with an increase in air humidity for all samples. This is due to a decrease in the number of adsorbed NO_2^- particles on the surface of materials (according to DRIFTS). The reason for this, in turn, is the substitution of adsorbed oxygen by water molecules (according to EPR data), which makes it difficult to oxidize NO by reaction (5). For successful detection of low NO concentrations in a humid atmosphere, additional modification of the sensitive material is necessary.

(ii) The influence of humidity on the interaction of sensitive materials with NO_2 is complex. It can be concluded that for SnO_2-based samples, an increase in the electron concentration in accordance with the reactions (6–8) stimulates reaction (2), which leads to an increase in the sensor response in the region of low NO_2 concentrations. When the NO_2 concentration increases, the limiting factor is the number of active sites capable of interacting with gases, which decreases with increasing humidity. For In_2O_3-based samples, a decrease in the sensor response to low NO_2 concentrations with increasing humidity indicates that an increase in the concentration of charge carriers in accordance with the reactions (6)–(8) is not a determining factor. At the same time, the dependence of the sensor response of In_2O_3-based samples to high NO_2 concentrations on air humidity is non-monotonic. The maximum response value is observed at RH = 20% and RH = 40%. It can be assumed that under these conditions, the reaction of substitution of chemisorbed oxygen with NO_2^- groups makes an additional contribution to the formation of the sensor response:

$$NO_{2(gas)} + O_2^-{}_{(ads)} = NO_2^-{}_{(ads)} + O_{2(gas)} \quad (9)$$

Due to the significant difference in the electron affinities O_2 and NO_2 molecules, at high NO_2 concentration the equilibrium (9) will be shifted to the right, and chemisorbed NO_2^- species should form deeper acceptor levels in the band gap of In_2O_3 that will increase the resistance of In_2O_3 based samples.

Supplementary Materials: The following are available online at http://www.mdpi.com/2079-4991/10/5/915/s1: Table S1: Frequency ranges and structures of IR bands observed in DRFIT spectra in 5 ppm NO_2/air and 50 ppm NO/air atmosphere on the surface of prepared samples in dry and humid (RH = 65%) air at room temperature.

Author Contributions: Conceptualization, M.R., O.F. and A.G.; data curation, A.N., E.K. and P.Y.; formal analysis, A.N., E.K. and P.Y.; investigation, A.N., A.M., E.K., S.T., and P.Y.; methodology, A.N., M.R., E.K. and O.F.; supervision, M.R.; writing of the original draft, A.N., M.R., and E.K.; writing of review and editing, M.R. and A.G. All authors have read and agreed to the published version of the manuscript.

Funding: This research was funded by the Russian Science Foundation, grant number 19-19-00357.

Acknowledgments: The EPR measurements were performed using the facilities of the Collective Use Center at the Moscow State University. The IR spectral experiments were carried out using the equipment purchased by funds of Lomonosov Moscow State University Program of the Development.

Conflicts of Interest: The authors declare no conflict of interest.

References

1. World Health Organization (WHO). WHO Guidelines for Indoor Air Quality: Selected Pollutants. Available online: www.euro.who.int_data/assets/pdf_file/0009/128169/e94535/pdf (accessed on 30 March 2020).
2. Menil, F.; Coillard, V.; Lucat, C. Critical Review of Nitrogen Monoxide Sensors for Exhaust Gases of Lean Burn Engines. *Sens. Actuators B* **2000**, *67*, 1–23. [CrossRef]
3. Witschi, H. Ozone, nitrogen dioxide and lung cancer: A review of some recent issues and problems. *Toxicology* **1988**, *48*, 1–20. [CrossRef]
4. Seinfeld, J.H.; Pandis, S.N. Chapter 20. Wet Deposition. In *Atmospheric Chemistry and Physics: From Air Pollution to Climate Change*, 3rd ed.; John Wiley & Sons, Inc.: Hoboken, NJ, USA, 2006; pp. 856–888.
5. Barnes, P.J. Nitric Oxide and Airway Disease. *Ann. Med.* **1995**, *27*, 389–393. [CrossRef] [PubMed]
6. Lunt, A.; Ahmed, N.; Rafferty, G.F.; Dick, M.; Rees, D.; Height, S.; Thein, S.L.; Greenough, A. Airway and alveolar nitric oxide production, lung function, and pulmonary blood flow in sickle cell disease. *Pediatri. Res.* **2016**, *79*, 313–317. [CrossRef] [PubMed]
7. Pijnenburg, M.W.H.; De Jongste, J.C. Exhaled nitric oxide in childhood asthma: A review. *Clin. Exp. Allergy* **2008**, *38*, 246–259. [CrossRef]
8. Righettoni, M.; Amann, A.; Pratsinis, S.E. Breath analysis by nanostructured metal oxides as chemoresistive gas sensors. *Mater. Today* **2015**, *18*, 163–171. [CrossRef]
9. Hyodo, T.; Urata, K.; Kamada, K.; Ueda, T.; Shimizu, Y. Semiconductor-type SnO_2-based NO_2 sensors operated at room temperature under UV-light irradiation. *Sens. Actuators B Chem.* **2017**, *253*, 630–640. [CrossRef]
10. Liu, B.; Luo, Y.; Li, K.; Wang, H.; Gao, L.; Duan, G. Room-Temperature NO_2 Gas Sensing with Ultra-Sensitivity Activated by Ultraviolet Light Based on SnO_2 Monolayer Array Film. *Adv. Mater. Interfaces* **2019**, *1900376*, 1–10.
11. Fomekong, R.L.; Saruhan, B. Influence of Humidity on NO_2-Sensing and Selectivity of Spray-CVD Grown ZnO Thin Film above 400 °C. *Chemosensors* **2019**, *7*, 42. [CrossRef]
12. Belghachi, A.; Collins, R.A. The effects of humidity on phthalocyanine NO_2 and NH_3 sensors. *J. Phys. D Appl. Phys* **1990**, *23*, 223–227. [CrossRef]
13. Ling, Z.; Leach, C. The effect of relative humidity on the NO_2 sensitivity of a SnO_2/WO_3 heterojunction gas sensor. *Sens. Actuators B* **2004**, *102*, 102–106. [CrossRef]
14. Nasriddinov, A.; Rumyantseva, M.; Shatalova, T.; Tokarev, S.; Yaltseva, P.; Fedorova, O.; Khmelevsky, N.; Gaskov, A. Organic-inorganic hybrid materials for room temperature light-activated sub-ppm NO detection. *Nanomaterials* **2020**, *10*, 70. [CrossRef]
15. Rumyantseva, M.; Nasriddinov, A.; Vladimirova, S.; Fedorova, O.; Tokarev, S.; Krylov, I.; Drozdov, K.; Baranchikov, A.; Gaskov, A. Photosensitive organic-inorganic hybrid materials for room temperature gas sensor applications. *Nanomaterials* **2018**, *8*, 671. [CrossRef]
16. Ruhland, B.; Becker, T.; Muller, G. Gas-kinetic interactions of nitrous oxides with SnO_2 surfaces. *Sens. Actuators B* **1998**, *50*, 85–94. [CrossRef]
17. Bârsan, N.; Weimar, U. Conduction model of metal oxide gas sensors. *J. Electroceram.* **2001**, *7*, 143–167. [CrossRef]

18. Rumyantseva, M.N.; Makeeva, E.A.; Badalyan, S.M.; Zhukova, A.A.; Gaskov, A.M. Nanocrystalline SnO_2 and In_2O_3 as materials for gas sensors: The relationship between microstructure and oxygen chemisorption. *Thin Solid Film.* **2009**, *518*, 1283–1288. [CrossRef]
19. Korotcenkov, G.; Brinzari, V.; Golovanov, V.; Blinov, Y. Kinetics of gas response to reducing gases of SnO_2 films, deposited by spray pyrolysis. *Sens. Actuators B* **2004**, *98*, 41–45. [CrossRef]
20. Egashira, M.; Nakashima, M.; Kawasuma, S.; Selyama, T. Temperature programmed desorption study of water adsorbed on metal oxides. Part 2. Tin oxide surfaces. *J. Phys. Chem.* **1981**, *85*, 4125–4130. [CrossRef]
21. Bârsan, N.; Schweizer-Berberich, M.; Gopel, W. Fundamental and practical aspects in the design of nanoscaled SnO_2 gas sensors: A status report. *Fresenius J. Anal. Chem.* **1999**, *365*, 287–304. [CrossRef]
22. Yamazoe, N.; Sakai, G.; Shimanoe, K. Oxide semiconductor gas sensors. *Catal. Surv. Asia* **2003**, *7*, 63–75. [CrossRef]
23. Davydov, A.A. Chapter 2. The Nature of Oxide Surface Centers. In *Molecular Spectroscopy of Oxide Catalyst Surfaces*; Sheppard, N.T., Ed.; John Wiley & Sons Ltd.: Chichester, UK, 2003.
24. Heiland, G.; Kohl, D. Chapter 2. Physical and Chemical Aspects of Oxidic Semiconductor Gas Sensors. In *Chemical Sensor Technology*; Seiyama, T., Ed.; Elsevier Science: Amsterdam, The Netherlands, 1988; Volume 1, pp. 15–38.
25. Morrison, S.R. Chapter 5. Bonding of Foreign Species at the Solid Surface. In *The Chemical Physics of Surfaces*, 2nd ed.; Springer Science+ Business Media: New York, NY, USA, 1990; pp. 173–220.
26. Henrich, V.A.; Cox, P.A. Chapter 6. Molecular Adsorption on Oxides. In *The Surface Science of Metal Oxides*; Cambridge University Press: Cambridge, UK, 1994; pp. 247–370.
27. Gurlo, A. Interplay between O_2 and SnO_2: Oxygen ionosorption and spectroscopic evidence for adsorbed oxygen. *Chem. Phys. Chem.* **2006**, *7*, 2041–2052. [CrossRef]
28. Konstantinova, E.A.; Pentegov, I.S.; Marikutsa, A.V.; Rumyantseva, M.N.; Gaskov, A.M.; Kashkarov, P.K. EPR study of nanocrystalline tin dioxide. *Phys. Status Solidi C* **2011**, *8*, 1957–1960. [CrossRef]
29. Konstantinova, E.A.; Weidmann, J.; Dittrich, T. Influence of adsorbed water and oxygen on the photoluminescence and EPR of por-TiO_2 (anatase). *J. Porous Mater.* **2000**, *7*, 389–392. [CrossRef]
30. Weidmann, J.; Dittrich, T.; Konstantinova, E.A.; Lauermann, I.; Uhlendorf, I.; Koch, F. Influence of oxygen and water related surface defects on the sensitized TiO_2 solar cell. *Sol. Energy Mater. Sol. Cells* **1999**, *56*, 153–165. [CrossRef]
31. Yang, L.; Marikutsa, A.; Rumyantseva, M.; Konstantinova, E.; Khmelevsky, N.; Gaskov, A. Quasi similar routes of NO_2 and NO sensing by nanocrystalline WO_3: Evidence by in situ drift spectroscopy. *Sensors* **2019**, *19*, 3405. [CrossRef]
32. Leblanc, E.; Perier-Camby, L.; Thomas, G.; Gibert, R.; Primet, M.; Gelin, P. NO_x adsorption onto dehydroxylated or hydroxylated tin dioxide surface. Application to SnO_2-based sensors. *Sens. Actuators B* **2000**, *62*, 67–72. [CrossRef]
33. Hadjiivanov, K.I. Identification of neutral and charged N_xO_y surface species by IR spectroscopy. *Catal. Rev. Sci. Eng.* **2000**, *42*, 71–144. [CrossRef]
34. Nakamoto, K. *Infrared and Raman Spectra of Inorganic and Coordination Compounds. Part A: Theory and Applications in Inorganic Chemistry*, 6th ed.; John Wiley & Sons, Inc.: New Jersey, NJ, USA, 2009.
35. Socrates, G. *Infrared and Raman Characteristic Group Frequencies: Tables and Charts*, 3rd ed.; John Wiley & Sons Ltd.: Chichester, UK, 2001.
36. Guglielminotti, E.; Boccuzzi, F. Nitric Oxide Adsorption and Nitric Oxide-Carbon Monoxide Interaction on Ru/ZnO Catalyst. *J. Catal.* **1993**, *141*, 486–493. [CrossRef]
37. Valden, M.; Keiski, R.; Xiang, N.; Pere, J.; Aaltonen, J.; Pessa, M.; Maunula, T.; Savimaki, A.; Lahti, A.; Harkonen, M. Reactivity of Pd/Al_2O_3, $Pd/La_2O_3–Al_2O_3$ and $Pd/LaAlO_3$ Catalysts for the Reduction of NO by CO: CO and NO Adsorption. *J. Catal.* **1996**, *161*, 614–625. [CrossRef]
38. Chao, C.C.; Lunsford, J.H. Adsorption of nitric oxide on Y-type zeolites. Low-temperature infrared study. *J. Am. Chem. Soc.* **1971**, *93*, 6794–6800. [CrossRef]
39. Kantcheva, M.; Bushev, V.; Hadjiivanov, K. Nitrogen dioxide adsorption on deuteroxylated titania (anatase). *J. Chem. Soc. Faraday Trans.* **1992**, *88*, 3087–3089. [CrossRef]
40. Gil, B.; Datka, J.; Kubacka, A.; Janas, J.; Sulikowski, B. NO adsorption on the active sites of Co- and/or in-containing ferrierite catalysts for the CH_4-SCR-NO process. *Stud. Surf. Sci. Catal.* **2005**, *158*, 1137–1144.

41. Djonev, B.; Tsyntsarski, B.; Klissurski, D.; Hadjiivanov, K. IR spectroscopic study of NO_x adsorption and NO_x–O_2 coadsorption on Co^{2+}/SiO_2 catalysts. *J. Chem. Soc. Faraday Trans.* **1997**, *93*, 4055–4063. [CrossRef]
42. Chen, E.S.; Wentworth, W.E.; Chen, E.C.M. The electron affinities of NO and O_2. *J. Mol. Struct.* **2002**, *606*, 1–7. [CrossRef]
43. Xu, G.; Zhang, L.; He, C.; Ma, D.; Lu, Z. Adsorption and oxidation of NO on various SnO_2(110) surfaces: A density functional theory study. *Sens. Actuators B* **2015**, *221*, 717–722. [CrossRef]
44. Sergent, N.; Epifani, M.; Comini, E.; Faglia, G.; Pagnier, T. Interactions of nanocrystalline tin oxide powder with NO_2: A Raman spectroscopic study. *Sens. Actuators B* **2007**, *126*, 1–5. [CrossRef]

© 2020 by the authors. Licensee MDPI, Basel, Switzerland. This article is an open access article distributed under the terms and conditions of the Creative Commons Attribution (CC BY) license (http://creativecommons.org/licenses/by/4.0/).

ACS APPLIED NANO MATERIALS

www.acsanm.org Article

Au-WO_3 Nanowire-Based Electrodes for NO_2 Sensing

Hongying Lin, Junhe Wang, Shiqiang Xu, Qiang Zhang, Yongqiang Cheng, Dan Han, Hongtao Wang, and Kai Zhuo*

Cite This: ACS Appl. Nano Mater. 2022, 5, 14311−14319 Read Online

ACCESS | Metrics & More | Article Recommendations | Supporting Information

ABSTRACT: In this work, a selective and highly sensitive gas sensor using tungsten oxide (WO_3) nanofibers was fabricated via electrospinning. WO_3 was functionalized with gold nanoparticles by magnetron sputtering at different sputtering times to obtain Au films with thicknesses of 1, 5, 10, and 15 nm. The sensing performance of Au film composite nanomaterials with different Au layer thicknesses was tested at 100−250 °C and different nitrogen dioxide (NO_2) concentrations ranging from 200 to 1000 ppb. The findings showed that the 10 nm Au−WO_3 composite nanomaterial sensor had the most significant improvement in the performance of the pristine WO_3 sensor compared with other Au−WO_3 composite nanomaterial sensors, and the optimal operating temperature of the sensor was 175 °C. The composite nanomaterial sensor exhibited excellent selectivity when exposed to different gases and also exhibited high sensibility when exposed to low concentrations of NO_2 under high humidity (80%). The mechanism of gas sensor performance improvement was also investigated.

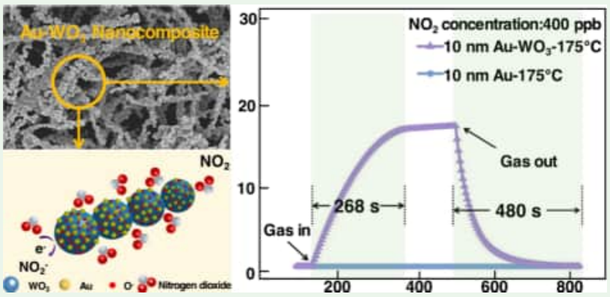

KEYWORDS: *NO_2, WO_3, Au, gas sensors, electronic sensitization, chemical sensitization*

Downloaded via NANJING TECH UNIV on February 1, 2025 at 06:39:44 (UTC).
See https://pubs.acs.org/sharingguidelines for options on how to legitimately share published articles.

1. INTRODUCTION

In the last few decades, toxic chemicals and harmful gases have been discharged into the air due to the rapid development of science and technology and the increasing demand for fossil fuels.[1,2] The continuous discharge of toxic chemicals and gases poses major threats to both the ecological environment and human health.[3] In these harmful gases, nitrogen dioxide (NO_2) causes severe health challenges and environmental pollution.[4,5] The World Health Organization included NO_2 into a list of the most dangerous yet common pollutants. NO_2 is a reddish-brown, flammable, explosive, and irritating gas.[6] Several studies have shown that prolonged exposure to low concentrations of NO_2 can cause respiratory tract infection, which can result in the inflammation of the throat and nose, leading to dizziness, and nausea.[7,8] In more severe cases, poisoning with NO_2 causes serious life-threatening emphysema, asthma, bronchitis, and lung cancer. For health and safety reasons, exposure to low concentrations of NO_2 should not exceed 15 min.[8,9] Therefore, the development of a reliable, rapid, and highly sensitive NO_2 sensor is vital.

Gas sensors containing metal oxide semiconductors have attracted a lot of attention from scientists and engineers because of their excellent performance, remarkable stability, and compatibility.[10−12] Metal oxide semiconductors (MOSs), including CuO, SnO_2, Co_3O_4, tungsten oxide (WO_3), ZnO, NiO, and In_2O_3[13−15] are very popular materials for applications related to the detection of volatile organics and toxic gases in the atmosphere As a typical N-type semiconductor, WO_3 with a wide bandgap of 2.6 eV has attracted considerable research attention because of its superior stability and high electron mobility and has been extensively applied in gas sensors.[16−19] However, the single tungsten trioxide sensor is characterized by low sensitivity, high operating temperature, and instability.[20−22] There are several strategies to improve the practical application and performance of WO_3-based gas sensing.[23,24] WO_3 doping with noble metals (Au, Ag, Pt, and Pd) is a general strategy (one-step hydrothermal synthesis method).[25,26] The precious metals prepared via this method may be unevenly distributed and easy to polymerize. However, these metal thin films can be prepared and uniformly distributed onto target materials via a magnetron sputtering technology. Some studies have reported a WO_3 film grown on an alumina substrate, and Au nanoparticles were then sputtered on the WO_3 film. At 350 °C, the response of the WO_3-based sensor to 5 ppm of toxic H_2S gas was approximately 163.[27] Yu et al. designed a new structure Au@SnO_2 core−shell nanosphere and applied it in gas sensors. Compared with the pure SnO_2 nanospheres, the new Au@SnO_2 core−shell nanospheres have a higher carbon monoxide (CO) response.[28] Porous WO_3/SnO_2 nanofibers, synthesized

Received: May 25, 2022
Accepted: August 30, 2022
Published: September 16, 2022

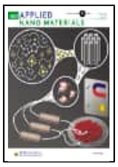

© 2022 American Chemical Society

by electrospinning, also improved the sensing performance when coated with the Pd catalyst (by chemical deposition).[29] The resulting composite Pd@WO_3/SnO_2 material was incorporated into a sensor capable of detecting acetone traces (0.05−10 ppm), and it exhibited more excellent gas sensitive performance.[29] The combination of WO_3 and the Au thin film has been shown to be a good prospect in detecting NO_2.[11,18,30,31] Due to the excellent gas sensing properties of WO_3, WO_3-based and derived sensors will become the most stable and mass-produced sensing materials in the future.

In this study, WO_3 nanofibers were prepared via electrospinning technology after which they were coated with an Au layer by magnetron sputtering. To prepare composites with different Au contents, we varied Au deposition times. Our sensing tests revealed that the response of the Au−WO_3 sensor based NO_2 gas sensor was much better than that of unmodified WO_3 sensors. During the experiment, the sensor electrode sheets can be prepared in batches. About 80 sensor electrodes were used for all the tests in this experiment. Therefore, after a certain improvement in the preparation process, the Au−WO_3 sensor is expected to be experimentally manufactured on a large scale. Finally, the sensor mechanism of Au−WO_3 composite nanomaterials for NO_2 gas detection is discussed.

2. EXPERIMENTAL DESIGN

2.1. Materials. Ammonium metatungstate hydrate (AMT, 99.5% purity), absolute ethanol, *N*,*N*-dimethylformamide (DMF, 99.5% purity), and polyvinylpyrrolidone (PVP, k88-96 grade) were obtained from Sinopharm Chemical Reagent. All chemicals were used without further purification.

2.2. Preparation of WO_3 Nanofibers. WO_3 nanofibers were prepared via the electrostatic spinning method. As shown in Figure 1a, 2.55 g of AMT was dissolved in 6 mL of de-ionized water. Subsequently, 9 mL of DMF and 1.8 g of PVP were added to the above mixture solution, and the mixture was stirred with a magnetic stirrer for 12 h at room temperature. The obtained homogeneous precursor solution was transferred into syringes and injected into a stainless steel nozzle (G21, Nano NC) for electrospinning. The mixed solution was electrospun at a voltage of 18 kV and a propulsion speed of 0.001 mm·s^{-1} at indoor temperature. The distance of the syringe front end to collector was 18 cm and the drum was rotated at 1500 rpm. Collected nanofibers were dried at 60 °C overnight in an oven. Then, the dried samples were heated to 700 °C in a muffle furnace at a heating rate of 2 °C/min in air and maintained for 3 h to obtain WO_3 nanofibers (Figure 1b).

2.3. Preparation of Au−WO_3 Nanocomposite Electrodes. WO_3 nanofibers were deposited on the surface of the electrode using spraying technology. WO_3 spraying agents were prepared using ethanol solvent, which resulted in a thinner and more uniform layer due to the rapid evaporation rate of ethanol. The WO_3 nanofibers were sonicated to disperse in an ethanol solution, which was then sprayed onto the electrode surface, which was heated to 80 °C to eliminate the solvent.

Au thin films were deposited on the WO_3 nanofiber electrode via magnetron sputter deposition. The growth rate of thin films was approximately 0.1 nm/s. After different deposition times, such as 1, 5, 10, and 15 nm, gold films were obtained on the WO_3 nanofiber electrode (size: 4 mm × 4 mm × 0.5 mm). After the inductively coupled plasma test, it was found that the Au content of 1 nm Au−WO_3 nanocomposite, 5 nm Au−WO_3 nanocomposite, 10 nm Au−WO_3 nanocomposite, and 15 nm Au−WO_3 nanocomposite was 0.15, 0.75, 1.48, and 2.27%, respectively (detailed experimental data can be found in Supporting Information Table S1). The detailed sputtering process parameters for the deposition of Au thin films are presented in the Supporting Information (Table S2). The preparation process of the sensor electrode is shown in Figure 1c.

2.4. Gas-Sensing Tests. The gas-sensing tests were performed using an IST-500E intelligent gas-sensing platform manufactured by the Beijing Sino Aggtech. During the sensing test, dry air was used as the background gas and the Au−WO_3 sensor was kept in a closed chamber. After the base resistance was stabilized, the gas sensitivity test could be performed. The test gas (NO_2) was diluted with dry air for the required concentration using the PC-controlled mass flow controllers. The electrode was mounted on a temperature-controlled heater. The electrode was directly connected to the test probe. The sensor response was determined as R_a/R_g or R_g/R_a, where R_a and R_g refer to the resistance in air and target gas, respectively. The volume of this dynamic gas-sensing test system was 0.1 L. Due to the small volume of the gas-sensitive chamber, the filling time is not considered, so the response time and recovery time are the real response of the sensor to the target gas response speed and recover speed. A schematic of the sensing setup is shown in Figure 2.

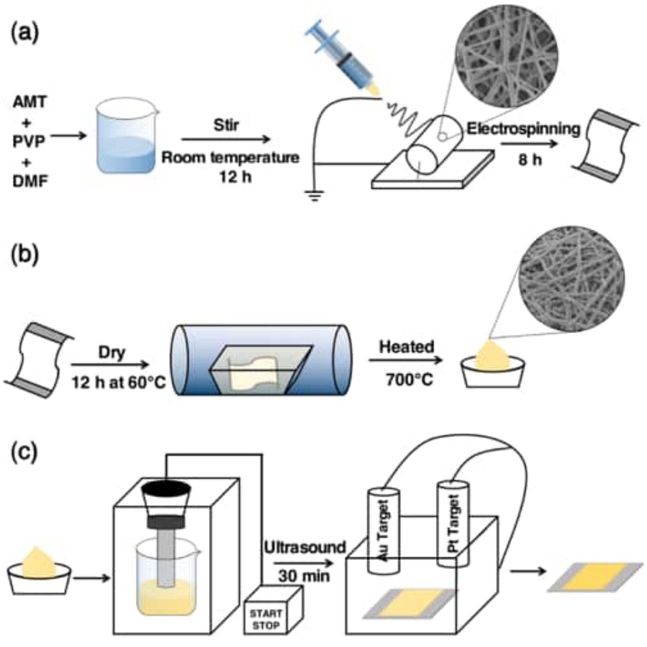

Figure 1. Process flowchart of Au−WO_3 nanocomposite electrodes. (a) Process of electrospinning. (b) Electrospun nanofibers are decomposed into WO_3 nanofibers at high temperature. (c) Manufacturing process of the Au−WO_3 sensor electrode sheet.

3. RESULTS AND DISCUSSION

3.1. Structural and Morphological Characterization. X-ray diffraction (XRD, Bruker, Karlsruhe, Germany) is one of the most used techniques for the structural characterization of

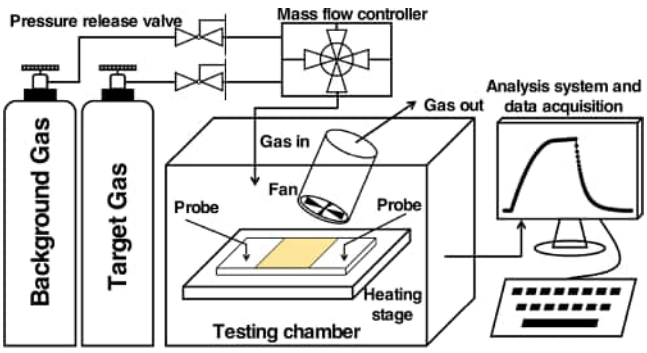

Figure 2. Diagram of the experimental setup used to test the gas sensitivity of NO_2.

https://doi.org/10.1021/acsanm.2c02289
ACS Appl. Nano Mater. 2022, 5, 14311−14319

crystalline materials. The characteristic peaks of the samples were consistent with those of monoclinic WO_3 (JCPDS No. 89-4476) and the characteristic peak of the cubic structured Au (111) plane (JCPDS No. 04-0784) was observed (see Figure 3a).[32] The relative strength of the Au diffraction peak was weaker than the diffraction peaks of WO_3, possibly owing to the small amount of Au. No other XRD peaks were detected, confirming the high purity of the samples.

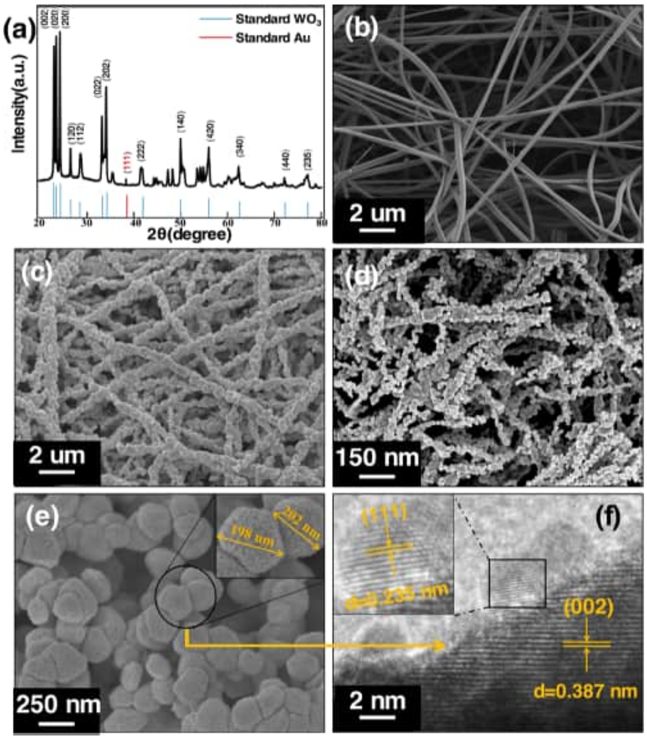

Figure 3. (a) XRD spectrum of Au−WO_3 nanocomposites, (b) the SEM images of WO_3 nanofibers acquired after electrospinning. (c,d) SEM images of WO_3 nanofibers and Au−WO_3 nanocomposites. (e,f) TEM images and HRTEM images of Au−WO_3 nanocomposites.

The surface morphology of WO_3 nanofibers and Au−WO_3 nanocomposite samples was examined using a scanning electron Hitachi S-4800 microscope. The scanning electron microscopy (SEM) image at high magnification gave accurate and clear shapes of the sample, which can be used to calculate the average grain size of the sample surfaces precisely. Figure 3c illustrates that the WO_3 nanofibers consist of abundant nanoparticles, which are beneficial for the diffusion of the gas. N_2 adsorption−desorption tests revealed 1.6 m^2/g surface area of the Au−WO_3 nanocomposite. This value agrees with the values reported for the similar materials of the same type. Therefore, we speculate that the substantial increase in the response of Au−WO_3 nanocomposites to NO_2 is mainly due to the addition of Au thin films. Figure 3d illustrates the SEM image of Au−WO_3 nanocomposite samples and Figure 3e illustrates the transmission electron microscopy (TEM) image of Au−WO_3 nanocomposites. According to Figure 3e, the TEM image further demonstrates the microstructure of the chain. The WO_3 nanoparticles were 180−220 nm in diameter. TEM also revealed a very even Au layer on the WO_3 nanoparticles. The presence of Au effectively speeded up the transfer of electrons on the face of the material and improved the kinetics of chemical adsorption on the surface of the material, which enhanced the dissociation reaction of gas molecules and thus improved the sensing performance of the sensor.[11,20,27,28,30,31]

The microstructure of the Au−WO_3 nanocomposite samples was examined using a high-resolution TEM (HRTEM) performed using a JEOL JEM-2100 setup. According to the HRTEM, the lattice fringes 0.387 nm apart (see Figure 3f) correspond to the (002) plane of the WO_3, while those 0.235 nm apart were assigned to the (111) planes of metallic Au.

X-ray photoelectron spectroscopy (XPS) spectra of W 4f, O 1s, and Au 4f are labeled in Figure 4a, and no other elements were detected. This result shows the effective preparation of Au−WO_3 nanocomposites, which also confirms the XRD result. The high-resolution W 4f XPS spectrum recorded for pristine WO_3exhibited two peaks 35.4 and 37.5 eV (see in Figure 4b) with the corresponding spin−orbit split equal to 2.1 eV. Thus, these values were assigned to W $4f_{5/2}$ and W $4f_{7/2}$ states, respectively.[33] At the same time, analogous peaks recorded for the Au−WO_3 composite were positioned at slightly higher binding energies because of the charge transfer between WO_3 and Au. The high-resolution O 1s spectrum of pristine WO_3 revealed three sub-peaks at 530.1, 531.0, and 532.4 eV, which correspond to the crystal lattice oxygen, oxygen vacancies, and chemisorbed oxygen species (H_2O, O_2), respectively (see Figure 4c). Analogous peaks in the XPS spectrum recorded for the 10 nm Au−WO_3 composite were located at 530.1, 531.0, and 532.3 eV, respectively. Table 1 shows that the oxygen vacancy content in 10 nm Au−WO_3 was higher (13.5%) than in pristine WO_3 (which was equal to 11.8%). The presence of oxygen vacancies is very beneficial for the overall sensing capability of a material. Thus, the higher the oxygen vacancy content, the better the sensing properties, including more favorable gas adsorption and increased response amplitude. The Au 4f XPS spectrum contained two peaks at 87.4 and 83.7 eV, which were ascribed to Au $4f_{7/2}$ and Au $4f_{5/2}$ states, respectively (see Figure 4f), the presence of which confirms the single metallic state of Au in the Au−WO_3 nanocomposites.

3.2. Gas-Sensing Properties. Different working temperatures significantly affected the gas-sensing performances of MOSs because the content of the ionized species (O_2^-, O^-, and O^{2-}) strongly depends on temperature. Figure 5a shows sensing responses at 100−250 °C of the sensors based on WO_3, 1 nm Au−WO_3, 5 nm Au−WO_3, 10 nm Au−WO_3, and 15 nm Au−WO_3. When exposed to 1 ppm of NO_2, all sensors exhibited a typical mountain-shaped curve. As shown in Figure 5a, the optimum operating temperature of a pristine WO_3 sensor is 150 °C; however, after sputtering Au films, the optimum operating temperature of all Au−WO_3 sensors became 175 °C. This phenomenon may be attributed to the chemical sensitization effect (spill-over effect), which can increase the coverage of negative oxygen active ions on the surface of sensitive materials. During the dissociation of precious metals, oxygen molecules dissociated into the more reactive ·O, thus reducing the operating temperature. As shown in Figure 5a, to achieve the same response value, the required operating temperature of the Au−WO_3 sensor is much lower than that of the original WO_3 sensor, and a higher surface negative oxygen ion coverage requires more energy as activation energy. Therefore, the optimum operating temperature of the Au−WO_3 sensor is increased. In Figure 5b, the R_a of pristine WO_3 and Au−WO_3 sensors decreases with increasing temperature. The resistance values of WO_3 and

https://doi.org/10.1021/acsanm.2c02289
ACS Appl. Nano Mater. 2022, 5, 14311−14319

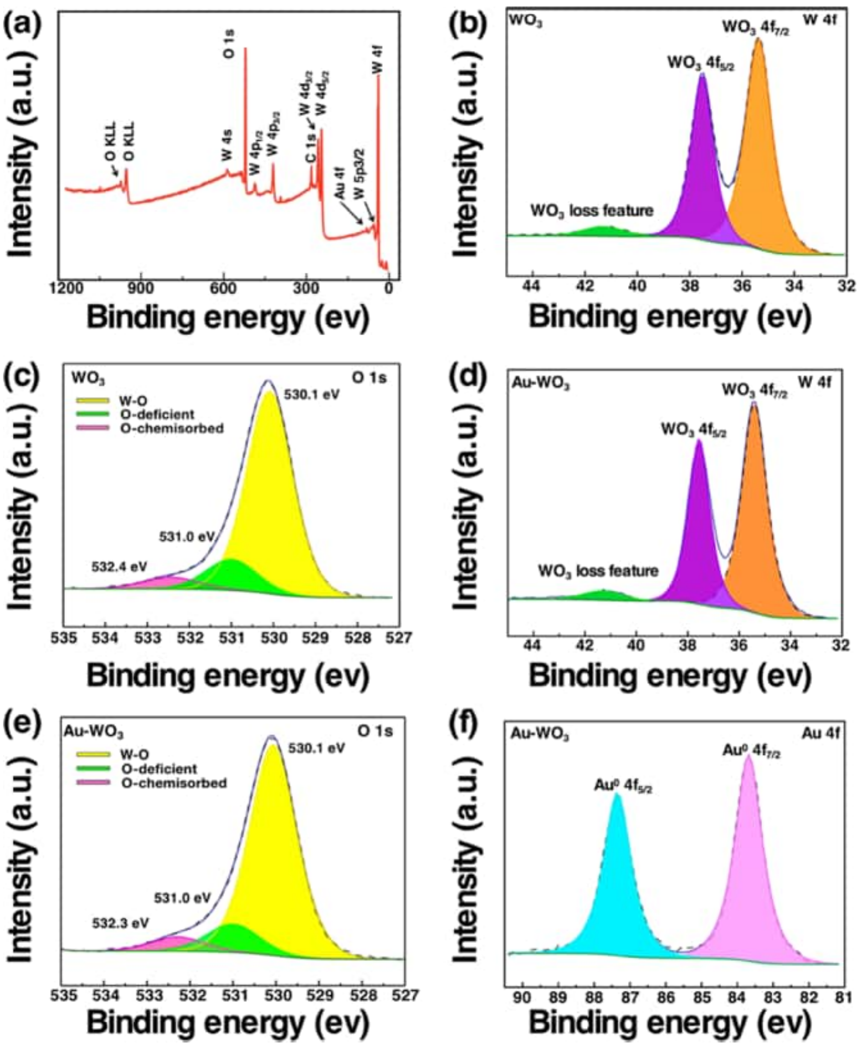

Figure 4. (a) Wide scan spectrum of Au−WO_3 nanocomposites. (b) W 4f spectrum of WO_3 nanocomposites, (c) the O 1s spectrum of WO_3 nanocomposites. XPS spectra of Au−WO_3 nanocomposites: (d) W 4f spectrum, (e) O 1s spectrum, and (f) Au 4f spectrum.

Table 1. Composition in O 1s of Pure WO_3 and 10 nm Au−WO_3

material	lattice oxygen	O-vacancy	O-chemisorbed
WO_3	6.5%	11.8%	81.7%
10 nm Au−WO_3	5.8%	13.5%	80.7%

Au−WO_3 sensors in the air at the working temperature of 175 °C were 10.3 and 0.088 MΩ, respectively. Higher Au contents in the composite increased its chemisorbed oxygen content on the surface. This leads to an increase in the base resistance of the sensor, but the resistance of the composite material also depends on the material itself. The resistance of the precious metal Au itself is much lower than that of WO_3, so as the Au content increases, the resistance of the composite material decreases.

Our next tests were exposure of sensors based on Au, WO_3, and Au−WO_3 to 50−1000 ppb of NO_2 at 176 °C and recording of their response−recovery (see Figure 5c). A sensor based on Au had no response to any concentration of NO_2 gas. Compared to the pristine WO_3 sensor, the sensor performance of the Au−WO_3 sensor was significantly better after Au addition judging by the response of the Au−WO_3 sensor, which was 12 times higher that of the pristine WO_3 sensor to 400 ppb of NO_2 gas. Figure 5d displays the response−recovery curves of sensors of pristine WO_3 and Au−WO_3 exposed to 400 ppb NO_2 gas at 175 °C. The response−recovery times of the pristine WO_3 and Au−WO_3 sensors to 400 ppb NO_2 gas were 67 s/73 s and 268 s/480 s, respectively. Under the same conditions, the response/recovery times of the Au−WO_3 sensors were longer than pristine WO_3 sensors. Due to the addition of precious metals, the response value of the Au−WO_3 sensor is 30 times that of the original WO_3 sensor. At the same NO_2 concentration, in order to achieve a higher response, the composite needs to adsorb more NO_2, so the reaction time will be longer. We tested the response cycles of the Au−WO_3 sensors to 200, 400, and 800 ppb NO_2 gas at 175 °C, and the response values were around 10.2, 17.1, and 28.6, respectively (Figure 5e). There was no considerable baseline drift observed, which proved that the Au−WO_3 sensor showed good reproducibility under different concentrations of NO_2 gas.

Both sensors showed relatively good linearity (see Figure 5f) with the corresponding equations expressed as $Y_1 = 0.001022X + 0.964765$ and $Y_2 = 3.964214X + 0.030703$ for the WO_3- and Au−WO_3-containing sensors. The corresponding correlation coefficients (R^2) were equal to 0.99798 and 0.99128,

https://doi.org/10.1021/acsanm.2c02289
ACS Appl. Nano Mater. 2022, 5, 14311−14319

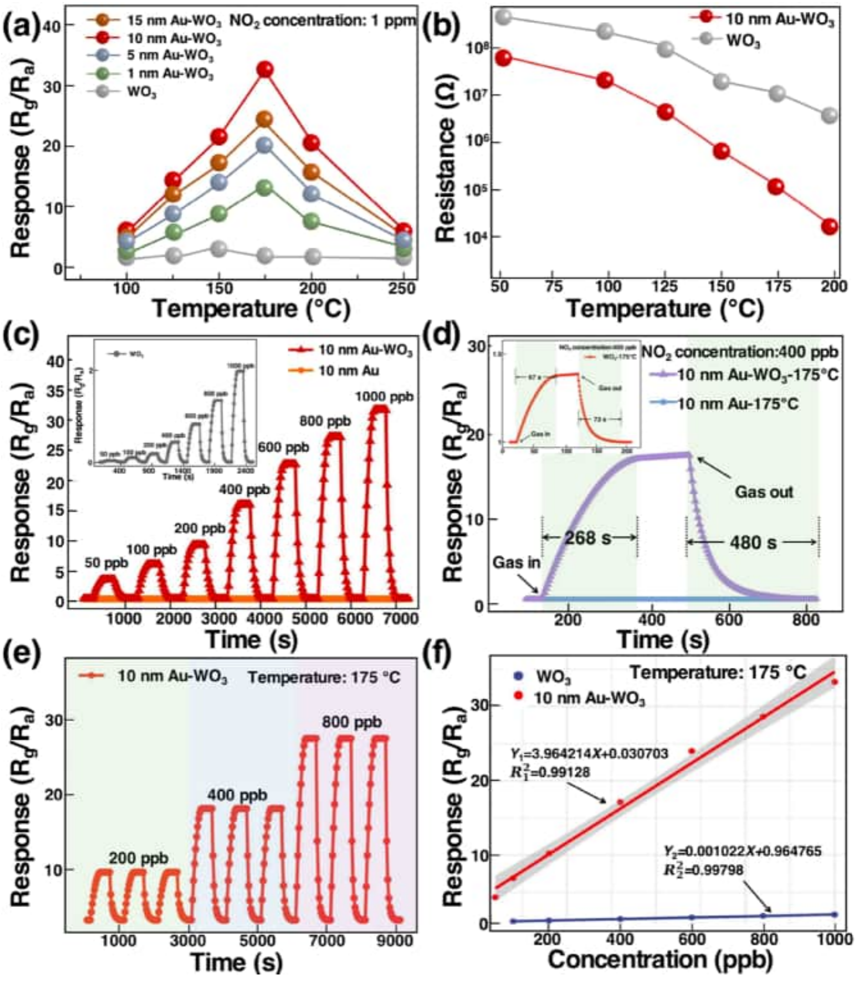

Figure 5. (a) Temperature-dependent sensing responses of the sensors based on pristine WO_3 and Au−WO_3 nanocomposites toward 1 ppm NO_2. (b) Resistances of the pristine WO_3 and Au−WO_3 sensors at different temperatures in air. (c) Dynamic response/recovery curves of three sensors to 50−1000 ppb NO_2 gas at 175 °C. (d) Response−recovery curves of the three sensors to 400 ppb NO_2 gas at 175 °C. (e) Reproducibility of the Au−WO_3 for three cycles at 175 °C. (f) Fitting of the sensor response curves of various NO_2 contents.

respectively. The limits of detection (LODs) were calculated using eq 2.

$$\sigma = \sqrt{\sum (S_i - S)^2/N} \quad (1)$$

where S_i is experimental data, S is obtained from the curve fitting, and N is the number of data points used for the fitting.

$$\text{LOD} = 3\sigma/S \quad (2)$$

where σ and S are the root-mean-square deviations (calculated using the fifth-order polynomial fit of base response versus time) and the slope value of the linear fit of the sensor response plotted as a function of the NO_2 content, respectively.[35,36]

According to eq 2, the Au−WO_3 sensor has a detection limit of 9.13 ppt for NO_2 using experimental data.

Figure 6a illustrates the response trend of the five sensors (pristine WO_3, 1 nm Au−WO_3, 5 nm Au−WO_3, 10 nm Au−WO_3, and 15 nm Au−WO_3) with the concentration of NO_2 (200−1000 ppb) at 175 °C. The 10 nm Au−WO_3-containing sensor showed the strongest response to1 ppm of NO_2 equal to 33.3. The reason that 10 nm Au−WO_3 exhibited the highest response can be explained as follows. With the increase of the Au content, the proportion of oxygen adsorbed on the surface of the material gradually increased, and the chemical sensitization effect was gradually enhanced.[34] The response also became higher with increasing Au content at this time. At excessively high Au contents, despite increased adsorbed oxygen, Au nanoparticles aggregate and, as a result, form independent charge transmission channels, thus, reducing the electronic sensitization. Systems containing 1 nm Au−WO_3 and 5 nm Au−WO_3 composites did not show sufficient electronic or chemical sensitization to be efficient active materials for gas sensors. A sensor containing 10 nm Au−WO_3 as an active sensor material showed the best performance due to the optimum electronic and chemical sensitization achieved by the most appropriate Au doping.

To determine the sensing selectivity, a mixture containing 1 ppm of NO_2, and 100 ppm of each ammonia, sulfur dioxide, acetone, ethanol, and methanol was added to the sensing setup at 175 °C containing our sensors (see Figure 6b). The exposure of the Au−WO_3 sensor to 1 ppm of NO_2 together with 100 ppm of five other gases demonstrated a much lower signal toward these other gases, which confirms the excellent selectivity of our sensor to NO_2. The response of the Au−WO_3 sensor to 400 ppb of NO_2 and its resistance in air was checked every 5 days (see Figure 6d). We observed stable sensing responses to this mixture for 20 days, which emphasizes the

https://doi.org/10.1021/acsanm.2c02289
ACS Appl. Nano Mater. 2022, 5, 14311−14319

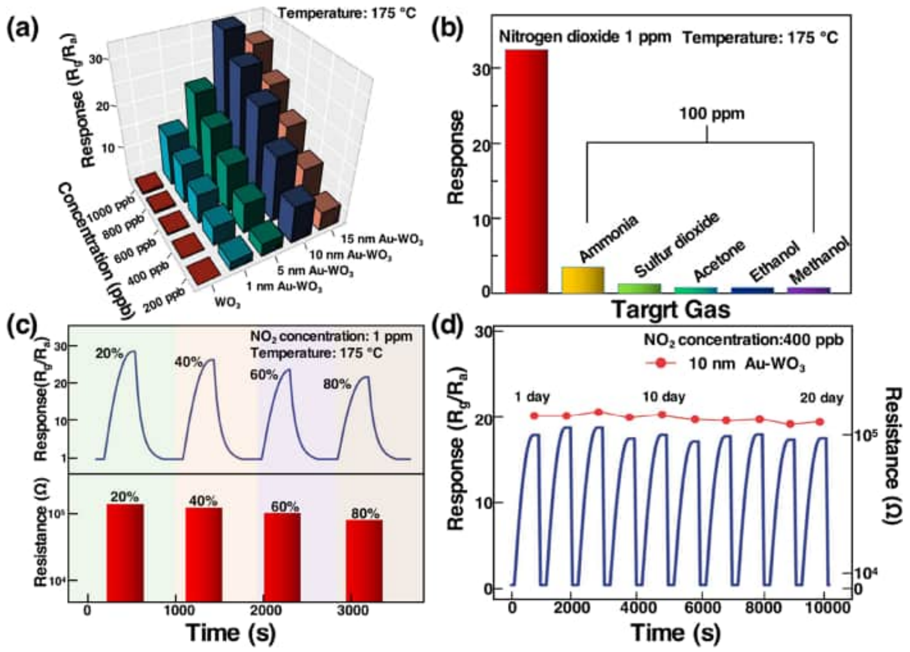

Figure 6. (a) Gas-sensing responses of the sensors of different samples as a function of NO_2 concentration at 175 °C. (b) Selectivity of the sensor to various gases at 175 °C. (c) Dynamic response−recovery curves of the Au−WO_3 sensor at 20−80% relative humidity (RH) toward 1 ppm NO_2 gas. Resistance value of the Au−WO_3 sensor at 20−80% RH in air at optimum operating temperature. (d) Stability tests toward 400 ppb NO_2 gas of the Au−WO_3 sensor for 20 days, at optimum operating temperature.

Table 2. Performance of Gas Sensors Prepared in This Work and in the Literature

materials	working condition	gas	concentration, ppm	response	T_{res}/T_{rec}	refs
SnSe nanostructured film	RT	NO_2	5	172%[a]	5 s/232 s	37
TiO_2−Sn_3O_4	RT	NO_2	10	285.7%[a]	9 s/60 s	38
Cu_2O−CuO microflowers	187	NO_2	1	10.2[b]	35 s/47 s	39
IGZO nano films	250 °C	NO_2	25	800[b]	5 min/25 min	40
porous ZnO NSs	200 °C	NO_2	10	74.6[b]		41
MoS_2/rGO	90 °C	NO_2	5	27.6%[a]		42
CuO nanoflakes/rGO	RT	NO_2	5	400.8[b]	6.8 s/55.1 s	43
In_2O_3 cubes/rGO	RT	NO_2	5	60.8[b]	180 s/240 s	44
Au−WO_3 nanofibers	250 °C	C_2H_6O	100	3.43[b]		45
Ag−WO_3 nanoplates	200 °C	CH_4O	100	1.2[b]		46
α-Fe_2O_3−ZnO−Au nanorods	225 °C	$C_4H_{10}O$	100	113[b]		47
Au−WO_3 nanofibers	175 °C	NO_2	0.4	10.2[b]	268 s/480 s	this work
			1	34.3[b]	253 s/495 s	

[a]Response = $\frac{|R_g - R_a|}{R_a}$. [b]Response = $\frac{R_g}{R_a}$ or $\frac{R_a}{R_g}$.

continuous cycling tests for 20 days can reveal the long-term stability of the sensor.

We also analyzed how humidity affects our Au−WO_3-based gas sensor (see Figure 6c) relative to 1 ppm of NO_2 at 175 °C and different RH. When the RH was increased from 20 to 80%, the response and resistance of the gas sensor gradually decreased. This phenomenon may be attributed to NO_2 reacting with water to form nitric oxide and nitric acid at higher humidity. However, the response value of the sensor can retain more than 80% at a relatively high RH of 80% compared with the response value at 20% RH. Therefore, the Au−WO_3-based sensor shows strong potential to detect NO_2. Table 1 compares the differences between various nanomaterial sensors and our Au−WO_3 sensor. Au−WO_3 sensor in this work has certain advantages in terms of sensitivity, low concentration detection, and time of response and recovery (Table 2).

3.3. Gas-Sensing Mechanism. Gas-interactions with WO_3, an n-type semiconductor, toward NO_2 could be explained using a loss model. The mechanism can also be interpreted as a typical adsorption-oxidation-desorption reaction.[6] The reduction of electrons on the WO_3 surface leads to electron depletion layer formation on the surface, which subsequently increased the resistance. The chemical absorption of O species strongly correlates with the sensor operating temperature: (1) below 100 °C, the most stable O species were O_2^-;[20,39] (2) in the 100−300 °C range, the predominantly O form is O^-; (3) above 300 °C, mostly O^{2-} forms.[48] This process can be explained using eqs 3−6 and shown in Figure 7.

$$O_2(g) \rightarrow O_2(ads) \quad (3)$$

$$O_2(ads) + e^- \rightarrow O_2^-(ads)\ (100°C) \quad (4)$$

$$O_2^-(ads) + e^- \rightarrow 2O^-(ads)\ (100 - 300°C) \quad (5)$$

$$O^-(ads) + e^- \rightarrow O^{2-}(ads)\ (\geq 300°C) \quad (6)$$

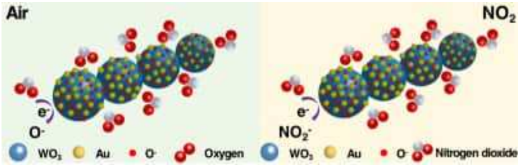

Figure 7. Gas-sensing mechanism of the Au−WO_3 sensor in air and NO_2.

The NO_2 molecules exhibited a strong electron withdrawing property and chemisorption on the WO_3 nanofibers due to its stronger electron affinity energy (220 kJ/mol) than O_2 (20 kJ/mol). NO_2 molecules react with chemisorbed oxygen ions and trap their electrons. Both formed and adsorbed ions will react with each other, facilitating the overall reaction shown in eq 10 below.[49] In this study, when the WO_3 nanofiber-based sensor was exposed to NO, electrons from the WO_3 conduction band were transferred to NO_2.[50−52] This, in turn, leads to additional electron consumption and, as a result, resistance increase of the overall sensor (see eqs 7−10 and Figure 10).[30,53,54] Upon Au introduction into WO_3 nanofibers, the electron depletion layer became thicker due to the increased oxygen vacancy content. The O vacancies generated by the strong metal-support interaction effect also participate in the redox reaction, creating even more absorbed oxygen and NO_2 species.[55] Thus, the catalytic effect and contribution of Au introduce more and easily available surface oxygen species. Occurrence of these redox reactions would drastically change in Au−WO_3 composite resistance, which explains the better performance of the Au−WO_3 relative to its undoped counterpart. The reactions discussed above can be schematically expressed as shown below (see eqs 11−15).

$$NO_2(g) \rightarrow NO_2(ads) \quad (7)$$

$$NO_2(ads) + e^- \rightarrow NO_2^-(ads) \quad (8)$$

$$NO_2(g) + O^- + 2e^- \rightarrow NO_2^-(ads) + O^{2-} \quad (9)$$

$$NO_2^-(ads) + 2O^- + e^- \rightarrow NO_2(g) + 2O^{2-} \quad (10)$$

$$O_2(ads) \rightarrow 2O(Au) \quad (11)$$

$$NO_2(g) \rightarrow NO_2(ads) + O\ ads\ (Au) \quad (12)$$

$$O\ ads(Au) + e^- \rightarrow O^-\ ads\ (Au) \quad (13)$$

$$O\ ads(Au) \rightarrow O\ ads(WO_3) \quad (14)$$

$$O\ ads(WO_3) + e^- \rightarrow O^-\ ads(WO_3) \quad (15)$$

We compared the sensing responses of Au−WO_3- and WO_3-based sensors toward NO_2 and determined a significant contribution of the Au film to the sensor performance. Catalytic contribution of the Au film involves surface barrier reduction and NO_2 adsorption enhancement (eq 8). However, Au, with its outstanding oxygen dissociation ability, can only adsorb and bond with active O molecules.[56,57] In this case, as a result of a spill-over effect, numerous O molecules would accept electrons from the WO_3 conduction band, and become the reactive ions, diffusing to the WO_3 surface. This, in turn, will increase the chemisorbed oxygen content on the sensor surface.[30,37] As seen in eq 9, a high content of O ions can improve the NO_2 sensing process, thus resulting in a higher response. Additionally, the presence of an Au film will facilitate NO_2 adsorption/desorption, the O-molecule-ion transformation, and the interaction of NO_2 and chemisorbed O active species, all of which contributes to the outstanding performance of the Au−WO_3-based sensor.

4. CONCLUSIONS

In summary, chain-like WO_3 nanofibers were effectively prepared via electrospinning, and then an Au film was sputtered on the surface of WO_3 nanofibers using magnetron sputtering. The chain-like Au−WO_3 nanocomposites are composed of irregular nanospheres with diameters ranging from 180 to 200 nm. The microscopic and bulk properties of Au−WO_3 nanocomposites were thoroughly analyzed. The addition of Au to WO_3 significantly improved the sensitivity of the corresponding sensor to NO_2. The temperature of the best performing Au−WO_3-based sensor was 175 °C at which its response to 1 ppm of NO_2 (equal to 33.3) was 17 times than that of pristine WO_3. Moreover, the Au−WO_3 sensor exhibited stable reproducibility, good linearity, and excellent selectivity. The NO_2 LOD for the Au−WO_3 sensor was 9.13 ppt. Humidity tests showed that the Au−WO_3 sensor had a relatively high response to NO_2 gas in a high humidity environment of 80% RH. The paper also describes in detail the sensing mechanism of our novel Au−WO_3-based sensor using the data on WO_3 carrier mobility, Au−WO_3 nanocomposite structures, and the catalytic effect of Au. The excellent performance of the Au−WO_3-based sensor relative to NO_2 indicates its very strong potential for real-life applications.

■ ASSOCIATED CONTENT

Supporting Information

The Supporting Information is available free of charge at https://pubs.acs.org/doi/10.1021/acsanm.2c02289.

Table S1: Percentage of Au content in different samples measured by inductively coupled plasma; Table S2: Process parameters for the deposition of Au thin films via magnetron sputtering technique (PDF)

■ AUTHOR INFORMATION

Corresponding Author

Kai Zhuo − *Mirco-Nano System Research Center, College of Information and Computer, Taiyuan University of Technology, Taiyuan, Shanxi 030024, China;* orcid.org/0000-0003-2966-9376; Email: zhuokai@tyut.edu.cn

Authors

Hongying Lin − *Mirco-Nano System Research Center, College of Information and Computer, Taiyuan University of Technology, Taiyuan, Shanxi 030024, China*

Junhe Wang − *Mirco-Nano System Research Center, College of Information and Computer, Taiyuan University of Technology, Taiyuan, Shanxi 030024, China*

Shiqiang Xu − *Mirco-Nano System Research Center, College of Information and Computer, Taiyuan University of Technology, Taiyuan, Shanxi 030024, China*

Qiang Zhang − *Mirco-Nano System Research Center, College of Information and Computer, Taiyuan University of*

https://doi.org/10.1021/acsanm.2c02289
ACS Appl. Nano Mater. 2022, 5, 14311−14319

Technology, Taiyuan, Shanxi 030024, China; orcid.org/0000-0001-6034-3919
Yongqiang Cheng − *Mirco-Nano System Research Center, College of Information and Computer, Taiyuan University of Technology, Taiyuan, Shanxi 030024, China*
Dan Han − *Mirco-Nano System Research Center, College of Information and Computer, Taiyuan University of Technology, Taiyuan, Shanxi 030024, China;* orcid.org/0000-0002-4517-6507
Hongtao Wang − *Mirco-Nano System Research Center, College of Information and Computer, Taiyuan University of Technology, Taiyuan, Shanxi 030024, China*

Complete contact information is available at:
https://pubs.acs.org/10.1021/acsanm.2c02289

Notes

The authors declare no competing financial interest.

ACKNOWLEDGMENTS

This work was supported by Research Project Supported by Shanxi Scholarship Council of China (HGKY2019026), International Cooperation of Science and Technology Projects in Shanxi Province (201903D421044), and the Sensor for disease markers of nitrogen compounds in exhaled breath (62031022). We thank LetPub (www.letpub.com) for its linguistic assistance during the preparation of this manuscript.

REFERENCES

(1) Mhamdi, H.; Azaiez, K.; Fiorido, T.; Benabderrahmane Zaghouani, R.; Lazzari, J. L.; Bendahan, M.; Dimassi, W. Room temperature NO_2 gas sensor based on stain-etched porous silicon: Towards a low-cost gas sensor integrated on silicon. *Inorg. Chem. Commun.* **2022**, *139*, No. 109325.

(2) Hong, H. S.; Ha, N. H.; Thinh, D. D.; Nam, N. H.; Huong, N. T.; Thi Hue, N.; Hoang, T. V. Enhanced sensitivity of self-powered NO_2 gas sensor to sub-ppb level using triboelectric effect based on surface-modified PDMS and 3D-graphene/CNT network. *Nano Energy* **2021**, *87*, No. 106165.

(3) Zhou, L.; Hu, Z.; Wang, P.; Gao, N.; Zhai, B.; Ouyang, M.; Zhang, G.; Chen, B.; Luo, J.; Jiang, S.; Li, H.-Y.; Liu, H. Enhanced NO_2 sensitivity of SnO_2 SAW gas sensors by facet engineering. *Sens. Actuators, B* **2022**, *361*, No. 131735.

(4) Khudadad, A. I.; Yousif, A. A.; Abed, H. R. Effect of heat treatment on WO_3 nanostructures based NO_2 gas sensor low-cost device. *Mater. Chem. Phys.* **2021**, *269*, No. 124731.

(5) Lu, Q.; Huang, L.; Hao, X.; Li, W.; Wang, B.; Wang, T.; Liang, X.; Liu, F.; Wang, C.; Lu, G. Mixed potential type NH3 sensor based on YSZ solid electrolyte and metal oxides (NiO, SnO_2, WO_3) modified $FeVO_4$ sensing electrodes. *Sens. Actuators, B* **2021**, *343*, No. 130043.

(6) Hu, Y.; Li, T.; Zhang, J.; Guo, J.; Wang, W.; Zhang, D. High-sensitive NO_2 sensor based on p-$NiCo_2O_4$/n-WO_3 heterojunctions. *Sens. Actuators, B* **2022**, *352*, No. 130912.

(7) Chen, Z.; Wang, J.; Umar, A.; Wang, Y.; Li, H.; Zhou, G. Three-Dimensional Crumpled Graphene-Based Nanosheets with Ultrahigh NO_2 Gas Sensibility. *ACS Appl. Mater. Interfaces* **2017**, *9*, 11819−11827.

(8) Li, T.; Shen, Y.; Zhao, S.; Zhong, X.; Zhang, W.; Han, C.; Wei, D.; Meng, D.; Ao, Y. Sub-ppm level NO_2 sensing properties of polyethyleneimine-mediated WO_3 nanoparticles synthesized by a one-pot hydrothermal method. *J. Alloys Compd.* **2019**, *783*, 103−112.

(9) Xiao, B.; Wang, D.; Wang, F.; Zhao, Q.; Zhai, C.; Zhang, M. Preparation of hierarchical WO_3 dendrites and their applications in NO_2 sensing. *Ceram. Int.* **2017**, *43*, 8183−8189.

(10) Dai, Z.; Xu, L.; Duan, G.; Li, T.; Zhang, H.; Li, Y.; Wang, Y.; Wang, Y.; Cai, W. Fast-response, sensitivitive and low-powered chemosensors by fusing nanostructured porous thin film and IDEs-microheater chip. *Sci. Rep.* **2013**, *3*, 1669.

(11) Zeb, S.; Peng, X.; Shi, Y.; Su, J.; Sun, J.; Zhang, M.; Sun, G.; Nie, Y.; Cui, Y.; Jiang, X. Bimetal Au-Pd decorated hierarchical WO_3 nanowire bundles for gas sensing application. *Sens. Actuators, B* **2021**, *334*, No. 129584.

(12) Franke, M. E.; Koplin, T. J.; Simon, U. Metal and metal oxide nanoparticles in chemiresistors: does the nanoscale matter? *Small* **2006**, *2*, 36−50.

(13) Choi, S.-W.; Kim, S. S. Room temperature CO sensing of selectively grown networked ZnO nanowires by Pd nanodot functionalization. *Sens. Actuators, B* **2012**, *168*, 8−13.

(14) Zhang, D.; Yang, Z.; Yu, S.; Mi, Q.; Pan, Q. Diversiform metal oxide-based hybrid nanostructures for gas sensing with versatile prospects. *Coord. Chem. Rev.* **2020**, *413*, No. 213272.

(15) Yin, L.; Qu, G.; Guo, P.; Zhang, R.; Sun, J.; Chen, D. Construction and enhanced low-temperature H_2S-sensing performance of novel hierarchical CuO@WO_3 nanocomposites. *J. Alloys Compd.* **2019**, *785*, 367−373.

(16) Ghosh, S.; Saha, M.; Paul, S.; De, S. K. Maximizing the photo catalytic and photo response properties of multimodal plasmonic Ag/WO(3-x) heterostructure nanorods by variation of the Ag size. *Nanoscale* **2015**, *7*, 18284−18298.

(17) Shen, Y.; Chen, X.; Wang, W.; Gong, Y.; Chen, S.; Liu, J.; Wei, D.; Meng, D.; San, X. Complexing surfactants-mediated hydrothermal synthesis of WO_3 microspheres for gas sensing applications. *Mater. Lett.* **2016**, *163*, 150−153.

(18) Jie, X.; Zeng, D.; Zhang, J.; Xu, K.; Wu, J.; Zhu, B.; Xie, C. Graphene-wrapped WO_3 nanospheres with room-temperature NO_2 sensing induced by interface charge transfer. *Sens. Actuators, B* **2015**, *220*, 201−209.

(19) Wang, C. J.; Yao, W. X.; Xia, T. X. A small-load-omitting criterion based on probability fatigue. *Int. J. Fatigue* **2014**, *68*, 224−230.

(20) Zhao, S.; Shen, Y.; Zhou, P.; Zhong, X.; Han, C.; Zhao, Q.; Wei, D. Design of Au@WO_3 core−shell structured nanospheres for ppb-level NO_2 sensing. *Sens. Actuators, B* **2019**, *282*, 917−926.

(21) Chen, D.; Hou, X.; Li, T.; Yin, L.; Fan, B.; Wang, H.; Li, X.; Xu, H.; Lu, H.; Zhang, R.; Sun, J. Effects of morphologies on acetone-sensing properties of tungsten trioxide nanocrystals. *Sens. Actuators, B* **2011**, *153*, 373−381.

(22) Chen, D.; Ge, L.; Yin, L.; Shi, H.; Yang, D.; Yang, J.; Zhang, R.; Shao, G. Solvent-regulated solvothermal synthesis and morphology-dependent gas-sensing performance of low-dimensional tungsten oxide nanocrystals. *Sens. Actuators, B* **2014**, *205*, 391−400.

(23) Fan, G.; Chen, D.; Li, T.; Yi, S.; Ji, H.; Wang, Y.; Zhang, Z.; Shao, G.; Fan, B.; Wang, H.; Xu, H.; Lu, H.; Zhou, Y.; Zhang, R.; Sun, J. Enhanced room-temperature ammonia-sensing properties of polyaniline-modified WO_3 nanoplates derived via ultrasonic spray process. *Sens. Actuators, B* **2020**, *312*, No. 127892.

(24) Chen, D.; Hou, X.; Wen, H.; Wang, Y.; Wang, H.; Li, X.; Zhang, R.; Lu, H.; Xu, H.; Guan, S.; Sun, J.; Gao, L. The enhanced alcohol-sensing response of ultrathin WO_3 nanoplates. *Nanotechnology* **2009**, *21*, No. 035501.

(25) Yin, L.; Chen, D.; Feng, M.; Ge, L.; Yang, D.; Song, Z.; Fan, B.; Zhang, R.; Shao, G. Hierarchical Fe_2O_3@WO_3 nanostructures with ultrahigh specific surface areas: microwave-assisted synthesis and enhanced H_2S-sensing performance. *RSC Adv.* **2015**, *5*, 328−337.

(26) Yin, L.; Chen, D.; Hu, M.; Shi, H.; Yang, D.; Fan, B.; Shao, G.; Zhang, R.; Shao, G. Microwave-assisted growth of In_2O_3 nanoparticles on WO_3 nanoplates to improve H_2S-sensing performance. *J. Mater. Chem. A* **2014**, *2*, 18867−18874.

(27) Punginsang, M.; Zappa, D.; Comini, E.; Wisitsoraat, A.; Sberveglieri, G.; Ponzoni, A.; Liewhiran, C. Selective H_2S gas sensors based on ohmic hetero-interface of Au-functionalized WO_3 nanowires. *Appl. Surf. Sci.* **2022**, *571*, No. 151262.

(28) Yu, Y.-T.; Dutta, P. Examination of Au/SnO_2 core-shell architecture nanoparticle for low temperature gas sensing applications. *Sens. Actuators, B* **2011**, *157*, 444−449.

(29) Zhang, J.; Zhang, L.; Leng, D.; Ma, F.; Zhang, Z.; Zhang, Y.; Wang, W.; Liang, Q.; Gao, J.; Lu, H. Nanoscale Pd catalysts decorated WO_3−SnO_2 heterojunction nanotubes for highly sensitive and selective acetone sensing. *Sens. Actuators, B* **2020**, *306*, No. 127575.
(30) Ponnuvelu, D. V.; Dhakshinamoorthy, J.; Prasad, A. K.; Dhara, S.; Kamruddin, M.; Pullithadathil, B. Geometrically Controlled Au-Decorated ZnO Heterojunction Nanostructures for NO_2 Detection. *ACS Appl. Nano Mater.* **2020**, *3*, 5898−5909.
(31) Shen, Y.; Li, T.; Zhong, X.; Li, G.; Li, A.; Wei, D.; Zhang, Y.; Wei, K. Ppb-level NO_2 sensing properties of Au-doped WO_3 nanosheets synthesized from a low-grade scheelite concentrate. *Vacuum* **2020**, *172*, No. 109036.
(32) Wang, D.; Deng, L.; Cai, H.; Yang, J.; Bao, L.; Zhu, Y.; Wang, X. Bimetallic PtCu Nanocrystal Sensitization WO_3 Hollow Spheres for Highly Efficient 3-Hydroxy-2-butanone Biomarker Detection. *ACS Appl. Mater. Interfaces* **2020**, *12*, 18904−18912.
(33) Nayak, A. K.; Ghosh, R.; Santra, S.; Guha, P. K.; Pradhan, D. Hierarchical nanostructured WO_3−SnO_2 for selective sensing of volatile organic compounds. *Nanoscale* **2015**, *7*, 12460.
(34) Wang, H.; Li, Y.; Wang, C.; Li, Y.; Bai, J.; Liu, Y.; Zhou, L.; Liu, F.; Shimanoe, K.; Lu, G. N-pentanol sensor based on ZnO nanorods functionalized with Au catalysts. *Sens. Actuators, B* **2021**, *339*, No. 129888.
(35) Hung, C. M.; Dat, D. Q.; Van Duy, N.; Van Quang, V.; Van Toan, N.; Van Hieu, N.; Hoa, N. D. Facile synthesis of ultrafine rGO/ WO_3 nanowire nanocomposites for highly sensitive toxic NH_3 gas sensors. *Mater. Res. Bull.* **2020**, *125*, No. 110810.
(36) Kuang, D.; Wang, L.; Guo, X.; She, Y.; Du, B.; Liang, C.; Qu, W.; Sun, X.; Wu, Z.; Hu, W.; He, Y. Facile hydrothermal synthesis of Ti_3C_2Tx-TiO_2 nanocomposites for gaseous volatile organic compounds detection at room temperature. *J. Hazard. Mater.* **2021**, *416*, No. 126171.
(37) Rani, S.; Kumar, M.; Sheoran, H.; Singh, R.; Singh, V. N. Rapidly responding room temperature NO_2 gas sensor based on SnSe nanostructured film. *Mater. Today Commun.* **2022**, *30*, No. 103135.
(38) Chen, D.; Yu, W.; Wei, L.; Ni, J.; Li, H.; Chen, Y.; Tian, Y.; Yan, S.; Mei, L.; Jiao, J. High sensitive room temperature NO_2 gas sensor based on the avalanche breakdown induced by Schottky junction in TiO_2-Sn_3O_4 nanoheterojunctions. *J. Alloys Compd.* **2022**, *912*, No. 165079.
(39) Wang, N.; Tao, W.; Gong, X.; Zhao, L.; Wang, T.; Zhao, L.; Liu, F.; Liu, X.; Sun, P.; Lu, G. Highly sensitive and selective NO_2 gas sensor fabricated from Cu_2O-CuO microflowers. *Sens. Actuators, B* **2022**, *362*, No. 131803.
(40) Eadi, S. B.; Shin, H.-J.; Nguyen, K. T.; Song, K.-W.; Choi, H.-W.; Kim, S.-H.; Lee, H.-D. Indium-gallium−zinc oxide (IGZO) thin-film gas sensors prepared via post-deposition high-pressure annealing for NO_2 detection. *Sens. Actuators, B* **2022**, *353*, No. 131082.
(41) Sik Choi, M.; Young Kim, M.; Mirzaei, A.; Kim, H.-S.; Kim, S.-I.; Baek, S.-H.; Won Chun, D.; Jin, C.; Hyoung Lee, K. Selective, sensitive, and stable NO_2 gas sensor based on porous ZnO nanosheets. *Appl. Surf. Sci.* **2021**, *568*, No. 150910.
(42) Jung, M. W.; Kang, S. M.; Nam, K.-H.; An, K.-S.; Ku, B.-C. Highly transparent and flexible NO_2 gas sensor film based on MoS_2/ rGO composites using soft lithographic patterning. *Appl. Surf. Sci.* **2018**, *456*, 7−12.
(43) Bai, H.; Guo, H.; Wang, J.; Dong, Y.; Liu, B.; Xie, Z.; Guo, F.; Chen, D.; Zhang, R.; Zheng, Y. A room-temperature NO_2 gas sensor based on CuO nanoflakes modified with rGO nanosheets. *Sens. Actuators, B* **2021**, 337.
(44) Yang, W.; Wan, P.; Zhou, X.; Hu, J.; Guan, Y.; Feng, L. Additive-free synthesis of In_2O_3 cubes embedded into graphene sheets and their enhanced NO_2 sensing performance at room temperature. *ACS Appl. Mater. Interfaces* **2014**, *6*, 21093−21100.
(45) Yang, X.; Salles, V.; Kaneti, Y. V.; Liu, M.; Maillard, M.; Journet, C.; Jiang, X.; Brioude, A. Fabrication of highly sensitive gas sensor based on Au functionalized WO_3 composite nanofibers by electrospinning. *Sens. Actuators, B* **2015**, *220*, 1112−1119.
(46) Kaneti, Y. V.; Yue, J.; Jiang, X.; Yu, A. Controllable Synthesis of ZnO Nanoflakes with Exposed $(10\bar{1}0)$ for Enhanced Gas Sensing Performance. *J. Phys. Chem. C* **2013**, *117*, 13153−13162.
(47) Chen, D.; Yin, L.; Ge, L.; Fan, B.; Zhang, R.; Sun, J.; Shao, G. Low-temperature and highly selective NO-sensing performance of WO_3 nanoplates decorated with silver nanoparticles. *Sens. Actuators, B* **2013**, *185*, 445−455.
(48) Tseng, Y. T.; Lin, J. C.; Ciou, Y. J.; Hwang, Y. R. Fabrication of a novel microsensor consisting of electrodeposited ZnO nanorod-coated crossed Cu micropillars and the effects of nanorod coating morphology on the gas sensing. *ACS Appl. Mater. Interfaces* **2014**, *6*, 11424−11438.
(49) Guo, F.; Feng, C.; Zhang, Z.; Wu, H.; Zhang, C.; Feng, X.; Lin, S.; Xu, C.; Zhang, B.; Bai, H. A room-temperature and ppb-level NO_2 sensor based on n-CdS/p-CuO heterojunction modified with rGO nanosheets. *Sens. Actuators, B* **2022**, *364*, No. 131898.
(50) Qi, J. J.; Gao, S.; Chen, K.; Yang, J.; Zhao, H. W.; Guo, L.; Yang, S. H. Vertically aligned, double-sided, and self-supported 3D WO_3 nanocolumn bundles for low-temperature gas sensing. *J. Mater. Chem. A* **2015**, *3*, 18019−18026.
(51) Bai, S.; Fu, H.; Zhao, Y.; Tian, K.; Luo, R.; Li, D.; Chen, A. On the construction of hollow nanofibers of ZnO-SnO_2 heterojunctions to enhance the NO_2 sensing properties. *Sens. Actuators, B* **2018**, *266*, 692−702.
(52) Xiao, B.; Wang, D.; Song, S.; Zhai, C.; Wang, F.; Zhang, M. Fabrication of mesoporous In_2O_3 nanospheres and their ultrasensitive NO_2 sensing properties. *Sens. Actuators, B* **2017**, *248*, 519−526.
(53) Li, Q.; Zeng, W.; Li, Y. Metal oxide gas sensors for detecting NO_2 in industrial exhaust gas: Recent developments. *Sens. Actuators, B* **2022**, *359*, No. 131579.
(54) Wang, B.-R.; Wang, R.-Z.; Liu, L.-Y.; Wang, C.; Zhang, Y.-F.; Sun, J.-B. WO_3 Nanosheet/$W_{18}O_{49}$ Nanowire Composites for NO_2 Sensing. *ACS Appl. Nano Mater.* **2020**, *3*, 5473−5480.
(55) Bai, H.; Guo, H.; Feng, C.; Wang, J.; Liu, B.; Xie, Z.; Guo, F.; Chen, D.; Zhang, R.; Zheng, Y. A room-temperature chemiresistive NO_2 sensor based on one-step synthesized SnO_2 nanospheres functionalized with Pd nanoparticles and rGO nanosheets. *Appl. Surf. Sci.* **2022**, *575*, No. 151698.
(56) Majhi, S. M.; Rai, P.; Yu, Y. T. Facile Approach to Synthesize Au@ZnO Core-Shell Nanoparticles and Their Application for Highly Sensitive and Selective Gas Sensors. *ACS Appl. Mater. Interfaces* **2015**, *7*, 9462−9468.
(57) Ju, D. X.; Xu, H. Y.; Qiu, Z. W.; Zhang, Z. C.; Xu, Q.; Zhang, J.; Wang, J. Q.; Cao, B. Q. Near Room Temperature, Fast-Response, and Highly Sensitive Triethylamine Sensor Assembled with Au-Loaded ZnO/SnO_2 Core-Shell Nanorods on Flat Alumina Substrates. *ACS Appl. Mater. Interfaces* **2015**, *7*, 19163−19171.

https://doi.org/10.1021/acsanm.2c02289
ACS Appl. Nano Mater. 2022, 5, 14311−14319

Sensors & Actuators: B. Chemical 326 (2021) 128910

Contents lists available at ScienceDirect

Sensors and Actuators: B. Chemical

journal homepage: www.elsevier.com/locate/snb

Novel Ag-SnO_2-βC_3N_4 ternary nanocomposite based gas sensor for enhanced low-concentration NO_2 sensing at room temperature

Dipyaman Mohanta, Md. Ahmaruzzaman *

Department of Chemistry, National Institute of Technology, Silchar, Assam, 788010, India

ARTICLE INFO

Keywords:
NO_2 sensor
Room temperature sensing
Schottky contact
n-n heterojunction

ABSTRACT

Herein, a novel gas sensor based on Ag nanoparticles, tin oxide and carbon nitride nanocomposite (Ag-SnO_2-βC_3N_4) was fabricated and its utility towards room-temperature NO_2 gas sensing was investigated. TheAg-SnO_2-βC_3N_4 ternary nanocomposite was characterized with transmission electron microscopy (TEM), Energy dispersive X-ray (EDX) analysis, X-ray diffraction (XRD), X-ray photoelectron spectroscopy (XPS) etc. The ternary heterojunction based sensor responded strongly to low concentrations of NO_2 gas at room-temperature. For 5 ppm NO_2 concentration, an excellent response time of 27.2 s for 90 % of response was achieved with higher recovery time of 110.5 s. The ternary nanocomposite sensor showed synergistic response which is ~1.14 times and ~1.9 times of that of the SnO_2-C_3N_4 and pristine SnO_2 sensors, respectively. The superior sensitivity of the ternary nanocomposite sensor is attributed to the higher conductivity and catalytic activity of the Ag-SnO_2-βC_3N_4 ternary nanocomposite. The enhanced sensing properties of Ag-SnO_2-βC_3N_4 sensor were discussed from the points of SnO_2-βC_3N_4 n-n heterojunction and Ag-SnO_2-βC_3N_4 Schottky contact on the basis of depletion layer model.

1. Introduction

Nitrogen dioxide (NO_2) is a common toxic and harmful gas, and primarily comes from burning fuel at high temperature and exhaust of motor vehicle [1]. The increased levels of NO_2 in cities causes dramatic environmental pollution as well as severe damages on human respiratory tract [2,3]. Therefore, the development of efficient sensing materials for the detection of low concentration NO_2 gas is greatly significant for both environmental protection and human health.

Recently, semiconducting nanostructured materials have found immense attention as efficient gas sensing materials due to high sensitivity, selectivity, stability and cost efficiency [4]. The high sensing performance of metal oxide semiconductor sensors can be attributed to high surface-to-volume ratio, band gap tunability, tunable surface defects and profuse active sites [5–7]. Indeed, various semiconducting metal oxides like SnO_2, ZnO, WO_3, TiO_2 etc. have been successfully used for fabrication of high performance NO_2 gas sensors [8–11]. However, such sensors need to operate at high temperatures of 100-400 °C to achieve excellent sensing properties. Moreover, high temperature operation may result in high power consumption and in some cases, may result in ignition of flammable and explosive gases [12]. Therefore, various integrated sensors such as doped metal oxides, nano-heterojunctions, nanocomposites etc. have been developed by modulating morphology, electronic structure or introducing facets, oxygen vacancies etc. to achieve highly sensitive gas sensing performance even at room temperature [13–17].

Gas sensing response is strongly affected by the interactions of the sensor surface with the gas molecules and thus, the role of heterojunction is very crucial for enhanced gas sensing properties [18]. In heterojunction sensors, the surface interaction of two dissimilar crystalline solids may result in a depletion layer at the interface in order to equalize the Fermi energy of the materials. This flow of charge carriers can alter the resistance of the material, thereby, making the heterojunction synergistically sensitive towards oxidizing/reducing gases [18–19]. Recently, n-n- junctions like ZnO/CdO [20], WO_3/ZnO [21], SnO_2-Sn_3O_4 [22], ZnO/SnO_2 [23], SnO_2/C_3N_4 [24] etc. were extensively utilized for improved NO_2 gas sensing performance. At the n–n junction interface, there occur transfer electrons from the materials with a high Fermi level to the one with a low Fermi level in order to equalize the Fermi energy. This results in the generation of a depletion layer, thereby, narrowing the conduction channel and enhancing the response.

Furthermore, it has been reported that hybridization of noble metal

* Corresponding author.
E-mail address: md_a2002@rediffmail.com (Md. Ahmaruzzaman).

https://doi.org/10.1016/j.snb.2020.128910
Received 18 May 2020; Received in revised form 9 September 2020; Accepted 13 September 2020
Available online 16 September 2020
0925-4005/© 2020 Elsevier B.V. All rights reserved.

nanoparticles with semiconductor metal oxide alters the Schottky barriers at the metal-semiconductor interface [25–27]. Noble metals such as Au, Ag, Pt and Pd have the Femi level much lower than that of semiconductor metal oxides like SnO_2, ZnO, and WO_3 and the electrons tend to flow from metal oxide to metal to achieve equilibrium, thus, significantly decreasing the conductivity [28–31]. In most cases, this facilitates large increase in sensing response, dynamic characteristics and decrease in optimal operating temperature.

In the present study, we focused on the fabrication of a novel NO_2 sensor based on Ag-SnO_2-C_3N_4 nanohybrid. The ternary nanoheterojunction generates Ag-SnO_2 Schottky junction and SnO_2-C_3N_4 n-n heterojunction which can collectively enhance the NO_2 sensing performances at room temperature.

2. Experimental Section

2.1. Materials and methods

All the reagents were of AR grade and used as received. Stannic chloride pentahydrate ($SnCl_4.5H_2O$), Silver nitrate ($AgNO_3$), Urea (NH_2CONH_2), Sodium hydroxide (NaOH) and distilled water were obtained from Sigma Aldrich.

Transmission electron microscopy (TEM) was carried out on JEOL, 9JSM-100CX equipment with an accelerating voltage of 60-200 kV. Energy dispersive X-ray (EDX) analysis was performed using the same instrument. Powder X-ray diffraction (XRD) patterns were obtained using Philips X'PERT powder X-ray diffractometer with Cu-Kα1 radiation (λ = 1.5406 Å) with a scan speed 2O/min at room temperature. BET analysis and BJH adsorption-desorption measurements were carried out by means of Quanta Chrome Nova 1000 gas adsorption analyzer. X-ray photoelectron spectroscopy (XPS) of the material was performed by PHI 5000 Versa Prob II spectrometer.

2.2. Synthesis

The Ag-SnO_2-βC_3N_4 nanocomposite was fabricated using hydrothermal technique reported elsewhere with modifications [32]. Firstly, βC_3N_4 was synthesized using urea as a precursor. Urea (2.5 g) was taken in porcelain crucible with appropriate lid and heated in muffle furnace at 550 °C for 4 h to get the βC_3N_4 powder. For the synthesis of SnO_2-βC_3N_4, 3.5 g of $SnCl_4.5H_2O$ was dissolved in 30 mL distilled water and stirred thoroughly. To this, freshly prepared βC_3N_4 powder was added and sonicated for 30 min to get homogenous dispersion. The basic pH of the mixture was maintained by adding NaOH (1 M) solution drop wise. The resultant mixture was transferred to Teflon lined hydrothermal autoclave and heated at 150 °C for 12 h. The product thus obtained was washed several times and dried to get SnO_2-βC_3N_4 nanocomposite.

For the synthesis of Ag-SnO_2-βC_3N_4 nanocomposite, *Iresine herbstii* leaf extract was utilized as reported elsewhere in our previous work [33]. To an aqueous suspension of SnO_2-βC_3N_4 nanocomposite (10 mg, 20 mL), silver nitrate solution (2 mg, 10 mL) was added and sonicated for about 15 min. The mixture was then added drop wise to the *Iresine herbstii* leaf extract (50 mL) with constant stirring and maintaining a constant temperature of 70 °C for 2 h. The resultant solution was then irradiated with thirty 10 s microwave shots (Power 1400 Watts) to get yellowish brown mass which was centrifuged, washed several times and

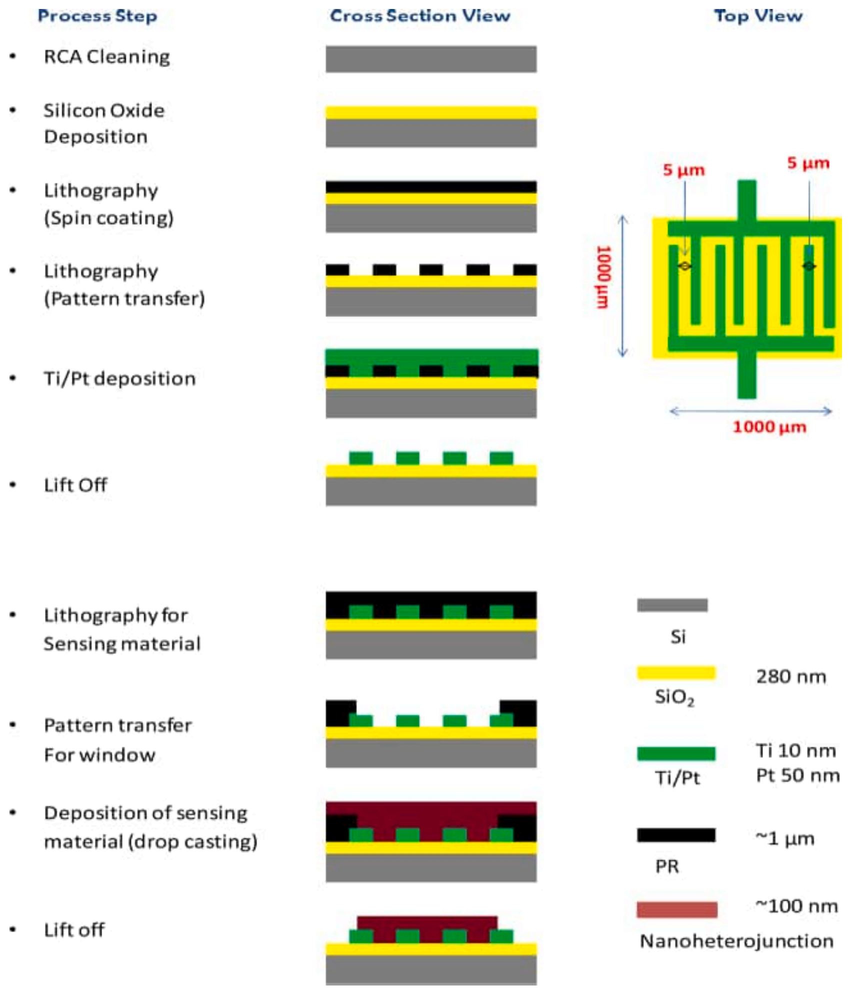

Scheme 1. Schematic representation of fabrication and dimensions of Ti/Pt IDEs.

air dried to get the Ag-SnO_2-βC_3N_4 nanocomposite.

2.3. Fabrication of the sensor and measurement of the sensing response

The gas sensors were fabricated using MEMS technology. The Ti/Pt inter digital electrode (IDE) was fabricated by lithography technique as represented in Scheme 1. The sensing film was coated on the device using drop coating technique. The nanocomposite precursor dispersion was drop casted at 500 rpm centrifuge condition and dried at 60 °C. The sensing characteristics were recorded using a bench system at room temperature (~ 27 °C). The electrical measurements were carried out using KEITHLY 2450-SourceMeter under a constant applied voltage (1 V), while injecting different concentrations of target gases at a flow rate of 500 mL min^{-1}. The gas response was recorded using the following equation,

$$Response\ (\%) = \frac{I_{air} - I_{gas}}{I_{air}} X\ 100$$

where, I_{air} and I_{gas} are the current values of the sensors in background gas and in presence of NO_2 or other test gases, respectively.

3. Results and discussion

3.1. Characterization

3.1.1. Transmission electron microscopy

The transmission electron microscopic technique (TEM) has been employed to investigate the morphology of the synthesized Ag-SnO_2-βC_3N_4 nanocomposite. The TEM micrograph (Fig.1 **(a)**) showed the coupling of faintly elliptical Ag nanoparticles having diameter in the range ~ 20-50 nm embedded within βC_3N_4 matrix. Deep inspection revealed that the Ag nanoparticles were surface decorated with quantum sized SnO_2 nanoparticles (diameter in the range ~3 ± 0.5 - ~2 ± 0.5 nm) of uniform size and shape (Fig.1 **(b)**), thereby, corroborating the composition of the desired Ag-SnO_2-βC_3N_4 nanocomposite. The HRTEM micrograph (Fig. 1**(c)**) centered on the junction of Ag and SnO_2 revealed the lattice fringes of 0.236 nm indicating (111) plane of Ag (JCPDS 89-3722) and 0.335 nm resembling (110) plane of tetragonal rutile SnO_2 (JCPDS 88-0287). The concentric rings in the SAED pattern (Fig. 1**(d)**) revealed the polycrystalline nature of Ag-SnO_2-βC_3N_4. The rings coinciding with both (110) plane SnO_2 and (111) plane of Ag were successfully identified and found analogous to the XRD patterns.

3.1.2. FESEM and EDAX mapping

The field emission scanning electron microscopic (FESEM) image showed the porous flake like surface morphology of the elemental composition of the Ag-SnO_2-βC_3N_4 nanocomposite (Fig. 2**(a)**). The elemental composition of the nanocomposite was also confirmed by the FESEM-EDAX mapping (Fig. 2 **(b-f)**) which revealed the presence of different elements like Sn, O, Ag, C and N using different color indicators.

3.1.3. X-ray diffraction analysis

X-ray diffraction technique (XRD) has been utilized to investigate the

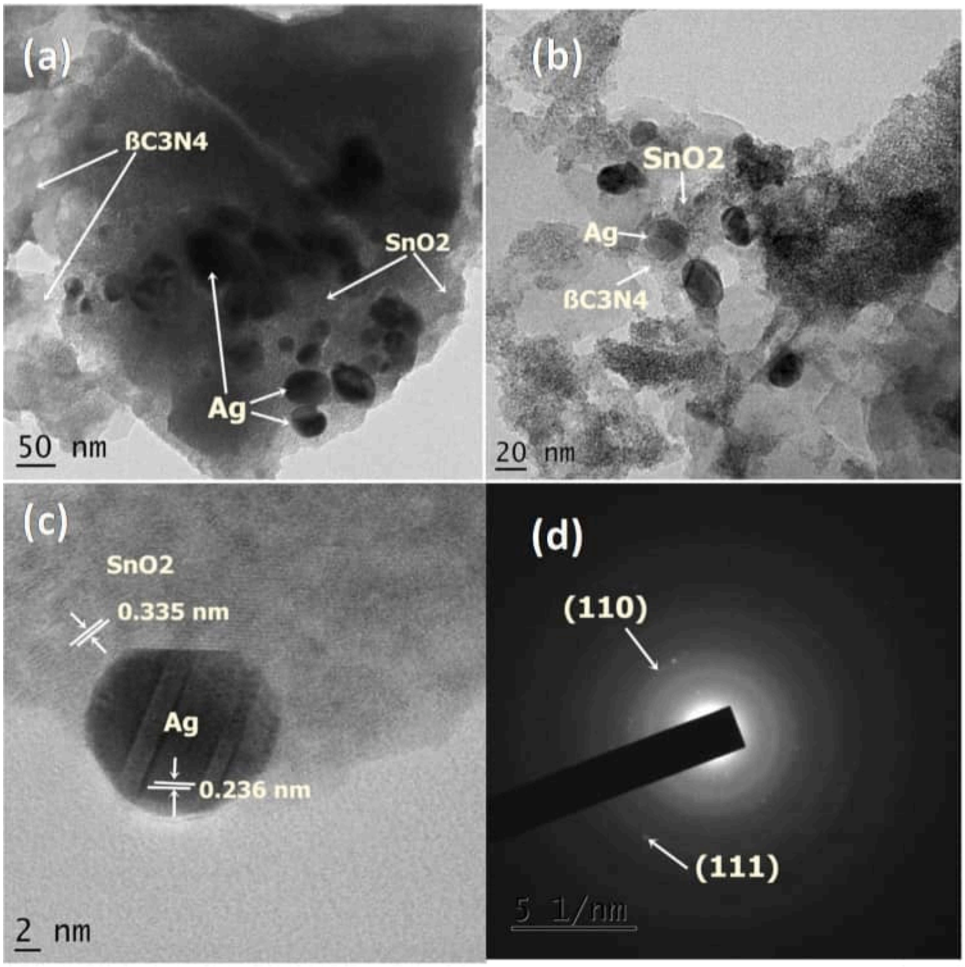

Fig. 1. (a-b) TEM micrographs of Ag-SnO_2-βC_3N_4 nanocomposite dispersed in ethanol (c) HRTEM micrograph centered on Ag-SnO_2 junction (d) SAED pattern of the Ag-SnO_2-βC_3N_4 nanocomposite.

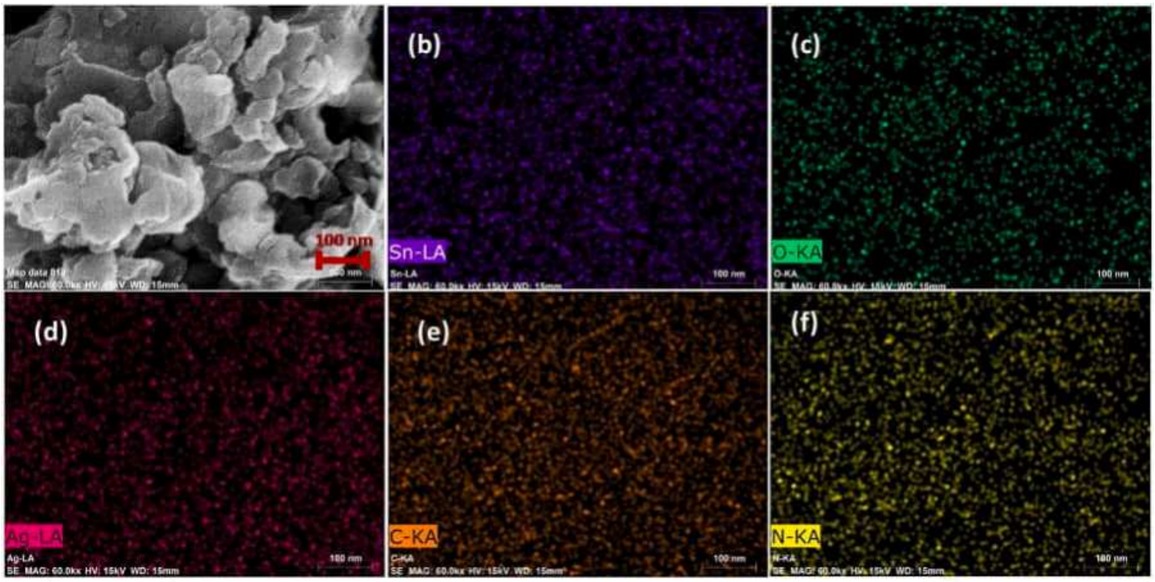

Fig. 2. (a) FESEM micrograph of Ag-SnO_2-βC_3N_4 nanocomposite (b-f) EDAX mapping of various elements like Sn, O, Ag, C and N.

crystal structure and phase constitution of the synthesized Ag-SnO_2-βC_3N_4 nanocomposite and presented in Fig. 3. Pristine tin oxide revealed diffraction peaks at 2θ values of 26.6°, 33.7°, 37.9°, 51.6° and 65.8° which are indexed to be (110), (101), (200), (211) and (301) planes of tetragonal SnO_2 (JCPDS 88-0287). The XRD pattern of Ag-SnO_2-βC_3N_4 nanocomposite showed dominant diffraction planes corresponding to (111), (211) and (301) planes of SnO_2 along with (111) and (200) planes of face-centered cubic silver (JCPDS 89-3722) centered at 2θ values 38.1° and 44.3°, respectively. Moreover, sharp diffraction peaks at 2θ values of 27.8°, 32.5°, 46.4° and 57.1° corresponding to (110), (200), (111) and (220) planes of hexagonal carbon nitride (βC_3N_4) (JCPDS 50-1512) were also evident in the XRD pattern of Ag-SnO_2-βC_3N_4 nanocomposite implying the successful coupling of Ag-SnO_2 moiety in βC_3N_4 matrix. The broadness of the SnO_2 peaks in comparison to sharp peaks of Ag and βC_3N_4 suggested the smaller sizes of SnO_2 crystals which is also evident from the TEM images.

3.1.4. *BET surface area analysis*

The BET surface area and BJH pore size distribution of Ag-SnO_2-βC_3N_4 nanocomposite were characterized by nitrogen adsorption-desorption method. Ag-SnO_2-βC_3N_4 exhibited typical type IV isotherm with characteristic H1 hysteresis loop which is associated with the capillary condensation taking place at the mesopores (Fig. 4)) [34]. The H1 hysteresis is also indicative of cylindrical pore geometry with high pore size uniformity and facile pore connectivity [35]. The BET surface area of Ag-SnO_2-βC_3N_4 was calculated to be 86.5 m^2/g. Also, Ag-SnO_2-βC_3N_4 exhibited BJH pore diameter and cumulative mesopore volume of 10.61 nm and 0.88 cm^3/g respectively. The high surface area and mesoporosity of the composite could provide more binding sites, enhancing the gas sensing property.

3.1.5. *X-ray photoelectron spectroscopic analysis*

To examine the surface chemical composition and chemical status of the functional material, X-ray photoelectron spectroscopy (XPS) was performed and represented in Fig. 5. The XPS survey spectrum (Fig. 5 **(a)**) displayed the presence of Ag, Sn, C, N and O elements confirming the composition of Ag-SnO_2-βC_3N_4 nanocomposite. The high resolution XPS spectrum of Ag (Fig. 5 **(b)**) showed two characteristic peaks at 373.07 eV and 366.98 eV which are attributed to Ag 3d 3/2 and Ag 3d 5/2 respectively, indicating the formation of metallic Ag. The existence of

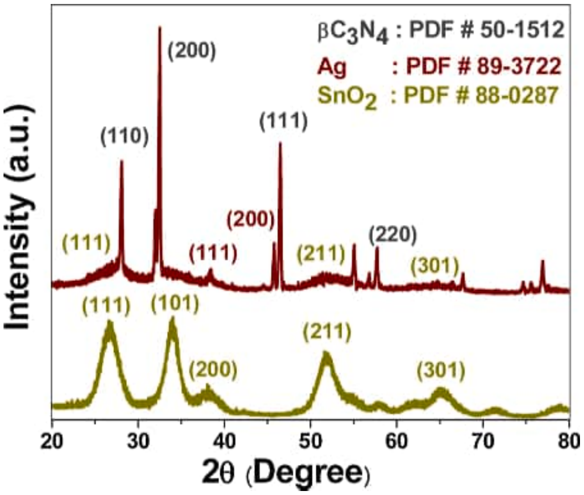

Fig. 3. X-ray diffraction pattern of pristine SnO_2 and Ag-SnO_2-βC_3N_4 nanocomposite.

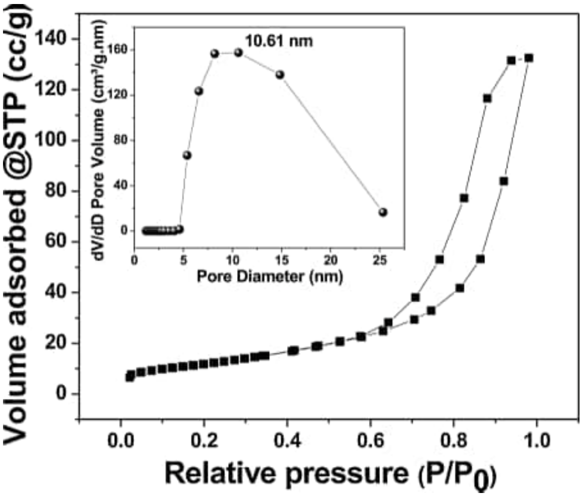

Fig. 4. N_2 adsorption-desorption isotherm and BJH pore size distribution of Ag-SnO_2-βC_3N_4.

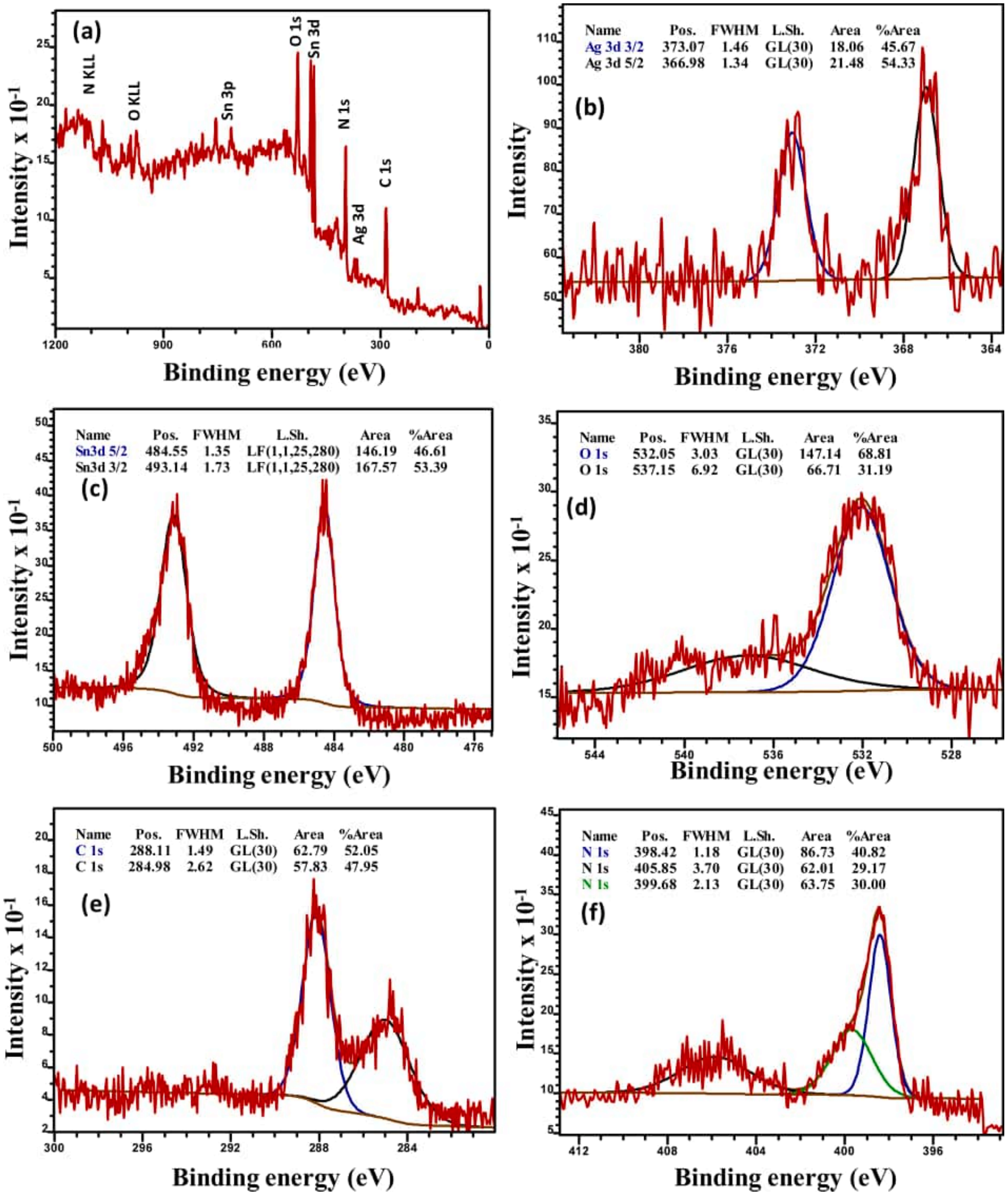

Fig. 5. (a) XPS survey spectrum; High resolution XPS spectrum of (b) Ag 3d (c) Sn 3d (d) O 1s (e) C 1s (f) N 1s of Ag-SnO_2-βC_3N_4.

Ag (I) ions as impurity was nullified due to the absence of any satellite peaks at 375.8 eV and 369.6 eV [36]. Fig. 5 **(c)** displayed the Sn 3d spectrum consisting of two peaks at 484.55 eV and 493.14 eV assigned to Sn3d 5/2 and Sn3d 3/2 respectively, indicating the formation of SnO_2. The O1 s peak (Fig. 5 **(d)**) showed a distinct peak at 532.05 eV which can be indexed to the Sn-O bond. On deconvolution, low intensity peak at 537.15 eV evolved which may be due to other oxygen functionalities and adsorbed water [37]. The C1 s peak can be deconvoluted into two peaks centered at 284.98 eV and 288.1 eV, which are attributed to sp^2 C-C bonds and sp^2 C-N bonds respectively. In addition, N1 s peak exhibited three signals at 398.42 eV, 399.68 eV and 405.85 eV. The two major signals at 398.42 eV and 399.68 eV correspond to two different types of sp^2 hybridized nitrogen viz., N_α (bonded to two neighboring atoms) and N_β (bonded to three different neighboring atoms) respectively. The weak signal at 405.85 eV is attributed to uncondensed terminal amino groups [38].

3.2. Gas sensing characteristics of Ag-SnO_2-βC_3N_4 nanocomposite based sensor

To investigate the NO_2 sensing properties of the Ag-SnO_2-βC_3N_4 nanocomposite, three different Ti/Pt IDE sensors were fabricated with

pristine SnO_2, SnO_2-C_3N_4 and Ag-SnO_2-βC_3N_4 by drop casting method. Fig. 6 **(a)** demonstrated the response and recovery curves of pristine SnO_2, SnO_2-$C_3N_4$and Ag-SnO_2-βC_3N_4 based sensors towards 1 ppm, 3 ppm and 5 ppm concentrations of NO_2 exposure at room temperature. It was evident that the injection of the NO_2 gas resulted in a steep decrease of the current values in all the three sensors and on stopping the gas flow, the current values gradually recovers itself to the initial value. This is attributed to the oxidizing nature of the injected NO_2 gas which can withdraw electron density from the catalyst system, thereby, decreasing the current values. The SnO_2, SnO_2-C_3N_4 and Ag-SnO_2-βC_3N_4 sensor exhibited maximum responses of 61.2 %, 53.8 % and 32.2 % respectively at 5 ppm NO_2 concentration. The Ag-SnO_2-βC_3N_4 sensor thus showed synergistic sensing behavior which is ~1.14 times and ~1.9 times superior to the SnO_2-C_3N_4 and pristine SnO_2 sensors, respectively.

Further, it has also been observed that the gas sensing response readily increases with increasing NO_2 concentrations from 1 ppm to 5 ppm. The sensor response of Ag-SnO_2-βC_3N_4 nanocomposite for 1 ppm, 3 ppm and 5 ppm NO_2 concentrations were found to be 38.6 %, 54.9 % and 61.2 % respectively, thereby indicating the utility of nanocomposite sensor for low concentration detection of NO_2 gas. A linear curve fitting of the sensor responses versus NO_2 concentration for SnO_2, SnO_2-C_3N_4 and Ag-SnO_2-βC_3N_4 based sensors at different concentrations has been represented in Fig. 6**(b)**. The sensitivity is determined from the slopes of the plots of response versus concentration [39]. The Ag-SnO_2-βC_3N_4 nanocomposite exhibited superior sensitivity compared to SnO_2-C_3-$N_4$and SnO_2 which may be attributed to the superior adsorption of NO_2 gas molecules over the surface of Ag-SnO_2-βC_3N_4 nanocomposite sensor.

Furthermore, the short response/recovery time and good reproducibility are very important indexes for the sensor device. A typical response recovery curve of Ag-SnO_2-βC_3N_4 nanocomposite sensor at 5 ppm NO_2 concentration is represented in Fig. 6 **(c)**. The response time (T_{res}) for reaching 90 % variation in the response upon exposure to gas is found to be 27.2 s and the recovery time (T_{rec}) of the sensor to reach 90 % of its initial response was found to be 110.5 s [40]. Thus, the Ag-SnO_2-βC_3N_4 nanocomposite sensor presented quite short response time and delayed recovery time towards room temperature NO_2 sensing which was compared with recently reported sensing materials with special emphasize on SnO_2 based nanocomposite sensors and presented in Table 1 [8,22–23,36,41–46]. Moreover, to investigate the repeatability of sensing performance, two cycles of the response profiles with varying concentrations has been shown in Fig. 6**(d)** [47]. It was observed that the sensor maintained its initial response properties upon six successive sensing tests, even two cycles of varying concentration of the gas. This indicated that the Ag-SnO_2-βC_3N_4 nanocomposite sensor possesses good repeatability and stability after six consecutive runs. All these observations are indicative of the potential of Ag-SnO_2-βC_3N_4 nanocomposite towards high performance NO_2 sensors at low concentration under room temperature.

The long-term stability of the Ag-SnO_2-βC_3N_4 nanocomposite sensor was investigated for successive 20 days and presented in Fig. 7 **(a)**. The average response for 5 ppm NO_2 concentration over 20 days was found to be 57.63 % with relative standard deviation of 2.2 %. Also, to investigate the selectivity of the Ag-SnO_2-βC_3N_4 nanocomposite sensor towards NO_2 gas, responses for acetone, ethanol and chlorine gas at 5 ppm concentrations were also recorded at room temperature and given

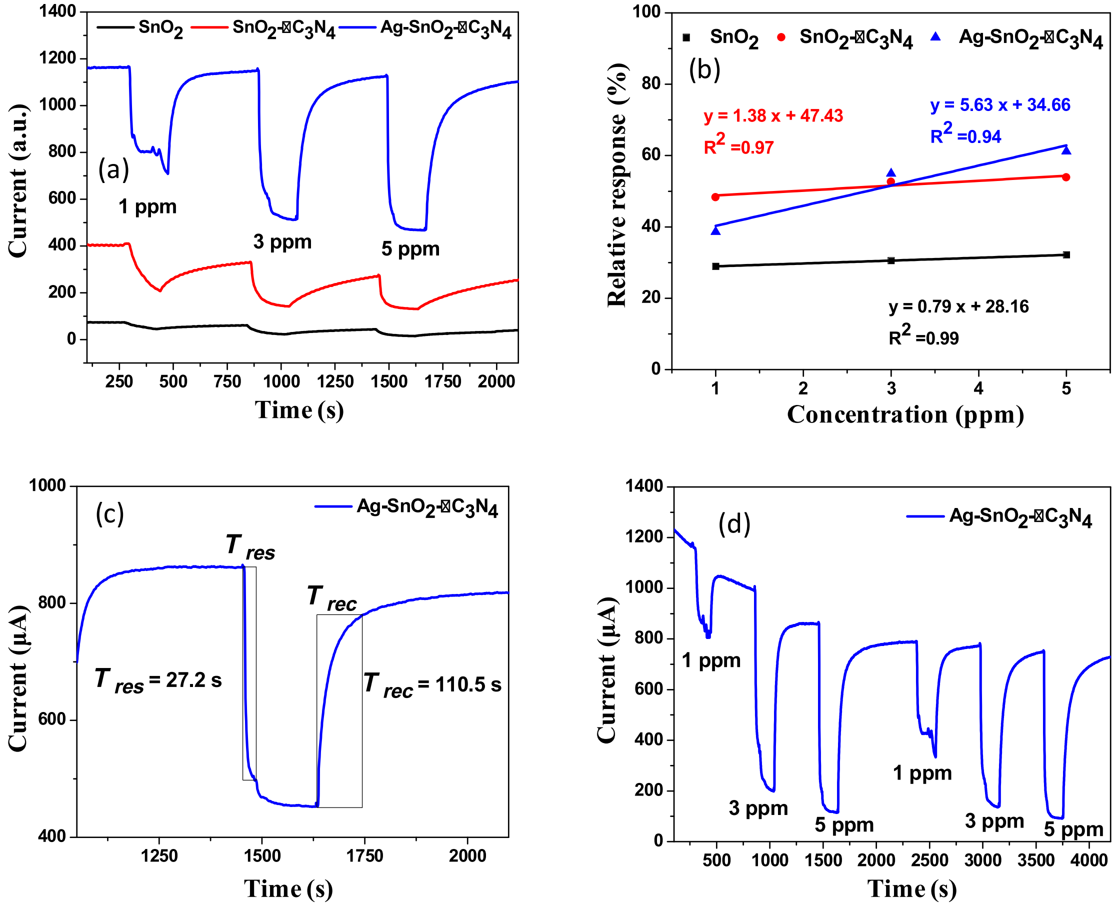

Fig. 6. Transient relative response of SnO_2, SnO_2-βC_3N_4 and Ag-SnO_2-βC_3N_4 sensors to 1 ppm, 3 ppm and 5 ppm concentration of NO_2 gas (b) Linear fitting of the responses of SnO_2, SnO_2-βC_3N_4 and Ag-SnO_2-βC_3N_4 sensors as a function of NO_2 concentration (c) Dynamic response-recovery curve of Ag-SnO_2-βC_3N_4 sensor at 5 ppm NO_2 concentration (d) The repeatability testing of response-recovery curves at different concentrations.

Table 1
Comparison of NO_2 sensing performance of Ag-SnO_2-βC_3N_4 with other reported sensing materials with special emphasize on SnO_2 based nanocomposite sensors

Materials	NO_2 gas concentration	Operating conditions	Response and recovery time	Reference
SnO_2	5 ppm	RT, UV	276 s/1140 s	[8]
SnO_2-Sn_3O_4	5 ppm	150 °C	62 s/22 s	[22]
SnO_2-ZnO	5 ppm	90 °C	68 s/83 s	[23]
AgNP-SnO_2-rGO	5 ppm	RT	49 s/339 s	[36]
ZnO/rGO	1 ppm	RT	75 s/132 s	[41]
SnO_2 with rich oxygen vacancy	5 ppm	RT	331 s/1057 s	[42]
SnO_2/RGOH	100 ppb	RT	117 s/260 s	[43]
SnO_2-rGO-PR	**5 ppm**	**RT**	**12 s/17 s**	[44]
SnO_2 loaded with exfoliated graphene	5 ppm	RT	7 min/–	[45]
3 D Ag-rGO aerogel	0.08 ppm	RT	75 s/89.5 s	[46]
Ag-SnO_2-βC_3N_4 nanoheterojunction	5ppm	**RT**	**27.2 s/110.5 s**	**This work**

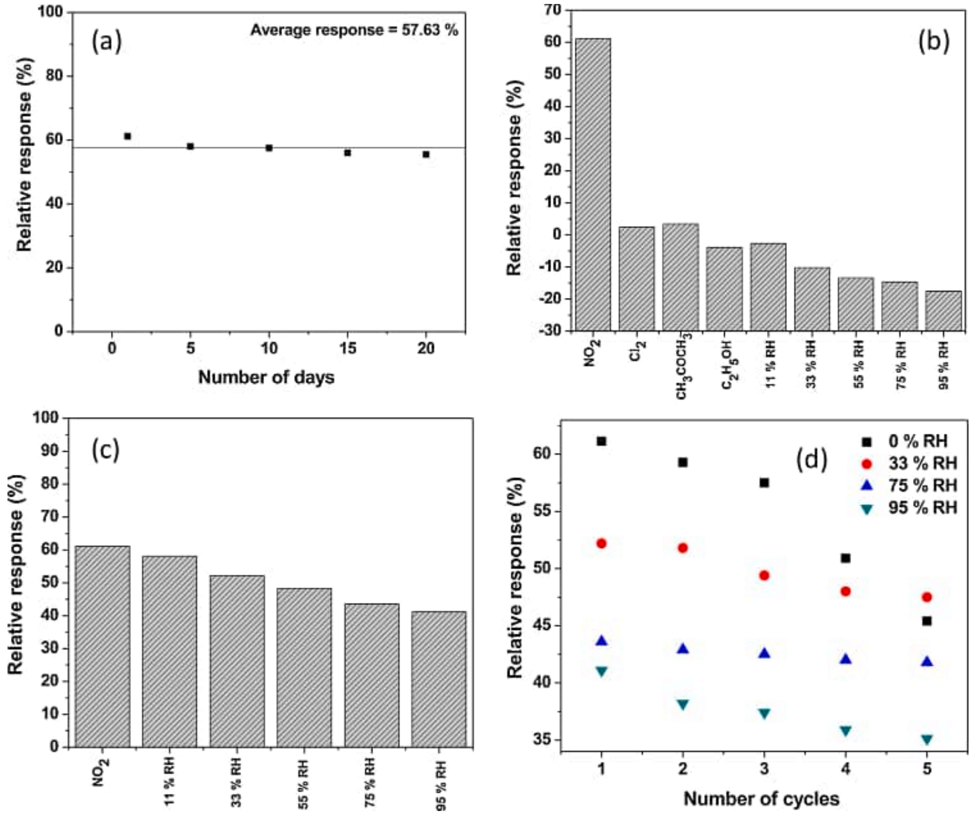

Fig. 7. (a)Long term stability of Ag-SnO_2-βC_3N_4 sensor response (b) Selectivity histogram of Ag-SnO_2-βC_3N_4 sensor (c) Effect of relative humidity on the NO_2 sensing performance (d)Percentage response during the sensing cycles to 5 ppm NO_2 at RT under varying humid atmospheres.

in Fig. 7 **(b)**. Apparently, the response value for NO_2 is much greater than that of the other target gases. This is attributed to the fact that the bond energy of O-NO is much lower compared to H-OC_2H_5, H-CH_2COCH_3 and Cl-Cl and hence the adsorption energy of NO_2 gas on the sensor surface is much larger compared to other gases at room temperature [36,48–49]. Besides, the presence of Ag-SnO_2-βC_3N_4 hetero-interface may increase the electronic conductivity and catalytic activity of the sensor surface, thereby resulting in the increase of selectivity of the sensor at room temperature.

Additionally, the response of Ag-SnO_2-βC_3N_4 towards humidity was also examined. The sensor has low response to the relative humidity (RH) of 11 %. Although, a slight increase in the relative response towards humidity was observed in the range 33-95 % RH, the interference is quite low. Further, in order to confirm the effect of relative humidity on NO_2 sensing performance, the relative responses of the sensor were recorded under different humid conditions (Fig. 7 **(c)**).With increasing the humidity of atmosphere, a gradual decrease in relative response towards target NO_2 was observed. Under 95 % RH, the relative response of Ag-SnO_2-βC_3N_4 sensor towards NO_2 gas (5 ppm) was found to be decreasing by ~33 %. This decrease in relative response is attributed to the completive adsorption of the target gas and water vapor on the sensor surface [50]. Water molecules tend to react with chemisorbed anionic oxygen species, thereby getting adsorbed over the sensor surface and facilitating release of electrons back to the conduction channel ($H_2O_{gas} + O^-_{ads} \longrightarrow 2OH_{ads} + e^-$) [51–52]. This results in the decrease in the resistance of the sensing material and also the relative response towards target NO_2 gas.

Moreover, the effect of repeated exposure of the Ag-SnO_2-βC_3N_4 sensor towards NO_2 gas (5 ppm) under varying humid atmospheres has also been investigated and presented in Fig. 7 **(d)**. After five consecutive sensing cycles, the NO_2 sensing response was found to be decreased by ~26 % in dry air, ~9 % in 33% RH, ~4 % in 75 % RH and ~15 % in 95 % RH. The steep decrease in sensing response under dry atmosphere is attributed to the incomplete recovery of NO_2 gas and ionosorption of reduced gas species (NO_2^-) over the sensor surface [53]. With increasing the humidity in the range 33-75 % RH, the sensing responses were found

to be fairly stable with variation in the range 9-4 % after five consecutive cycles. With increasing humidity level at room temperature, the proton within the physisorbed water may react with ionosorbed NO_2^- species ($H^+ + NO_2^- \longrightarrow HNO_2$), thereby stimulating the recovery of Ag-SnO_2-βC_3N_4 sensor and hence maintaining the stable sensing performance [54]. However, in 95 % RH, the observed decrease in the repeatability of response with consecutive cycles is attributed to coverage of active sites of sensor by hydroxyl surface groups due to physisorption of water vapor.

Thus, the results revealed that the Ag-SnO_2-βC_3N_4 nanocomposite sensor exhibits good long-term stability and selectivity towards NO_2 and reliability under humid conditions which are imperative for its practical application.

3.3. *Mechanism discussion of the enhanced sensing properties of Ag-SnO_2-βC_3N_4 nanocomposite based NO_2 sensor*

The exact gas sensing mechanism quite elusive, but a plausible mechanism of enhanced sensing performance based on the degree of interfacial charge perturbation on the sensor surface is presented in Fig. 8. The response of resistance-based sensor is highly dependent on the modulation of resistance by the adsorption and desorption of target gas on the sensor surface. SnO_2 is a n-type semiconductor with a band gap of 3.5 eV. When exposed to air, it interacts with oxygen by transferring electrons from conduction band (CB) to the adsorbed oxygen to generate chemisorbed oxygen species (O^-, O^{2-}, O_2^- etc.). As a result, a surface depletion layer is created at the surface, thereby, decreasing the carrier concentration and increasing the resistance of the sensor. On exposure to an oxidizing gas like NO_2, it further widens the depletion layer of the SnO_2 by accepting electrons from the surface, resulting in a sharp decrease in the current values of the sensor (NO_2 (g) + $e^- \longrightarrow$ 2NO (g) + O^-_{ads}) [48]. After stopping the supply of target gas, the recovery of current occurs due to the release of trapped electrons by the adsorbed NO_2 gas molecules.

Compared with pristine SnO_2 sensor, the binary SnO_2-βC_3N_4 sensor exhibited superior sensing performance due to the formation of n-n heterojunction interface. Referring to literature reports, the conduction band position, work function and energy band gap of SnO_2 and βC_3N_4 are E_{CB}= -4.5 eV (vs. vacuum), W=4.9 eV, E_g=3.5 eV and E_{CB} = -3.37 eV (vs. vacuum), W =4.15 eV and E_g=2.7 eV respectively [49,55]. Due to the formation of n-n heterojunction, the electrons from conduction band of βC_3N_4 tend to flow to the conduction band of SnO_2 until the Fermi levels gets equalized. As a result, an electron depletion layer is created at the interface, which can promote abundant amount of oxygen adsorption at the interface. The interaction of target NO_2 gas with greater amount of chemisorbed oxygen results in greater sensing response in SnO_2-βC_3N_4 sensor compared to the pristine SnO_2 sensor.

Moreover, the hybridization of Ag nanoparticles to SnO_2-C_3N_4 to forms nano-Schottky junction which may further enhance the sensing performance both by chemical sensitization and electronic sensitization. The chemical sensitization occurs at the catalytic surface of noble Ag nanostructures where the molecular oxygen can get adsorbed and dissociate to generate reactive oxygen atoms [56]. The generated oxygen atoms may undergo a spill over process on to the SnO_2/C_3N_4 semiconductor junction and withdraw electrons to produce anionic oxygen species at the interface [56–57]. As a result, high electrostatic potential generated at the junction thereby increasing the resistance due to band bending leaving Ag nanostructure unchanged (Fig. 8) [56–59]. Additionally, the adsorption of target gas molecules on to the catalytically active sites of Ag nanostructures may lower the work function of the moiety [59–60].

In electronic sensitization, the excited electrons from conduction bands of SnO_2 and g-C_3N_4 can travel to Ag nanostructure due to the lower work function of Ag (4.26 eV) [36] (Fig. 8). The depletion zone thus created around the interfaces of Ag, SnO_2 and/or βC_3N_4 may modulate the resistance and surface reactivity at room temperature. Thus, the heterojunction structure acts as a lever to modulate the charge concentration and charge mobility of the sensor surface, thereby enhancing the sensing properties. The substantial improvement of the response is attributed to the larger change in resistance caused by the increased participation of electrons and creation of depletion zones due to the heterojunction structure of the Ag-SnO_2-βC_3N_4 nanocomposite.

4. Conclusion

In summary, a new NO_2 sensor has been successfully fabricated by using Ag-SnO_2-βC_3N_4 hybrids as sensing materials, which have been prepared by hydrothermal synthesis method. The gas sensing results indicate that the introduction of Ag NPs into the SnO_2-βC_3N_4 hybrids significantly enhanced the NO_2 sensing performances at room temperature, compared to SnO_2-βC_3N_4 hybrids. This improvement in gas sensing response can be attributed to the coupled effect of nano-Schottky junction and n-n heterojunction on the electronic structure of ternary Ag-SnO_2-βC_3N_4 nanocomposite sensor.

CRediT authorship contribution statement

Dipyaman Mohanta: Data curation, Writing - original draft, Methodology, Software. **Md. Ahmaruzzaman:** Supervision, Investigation.

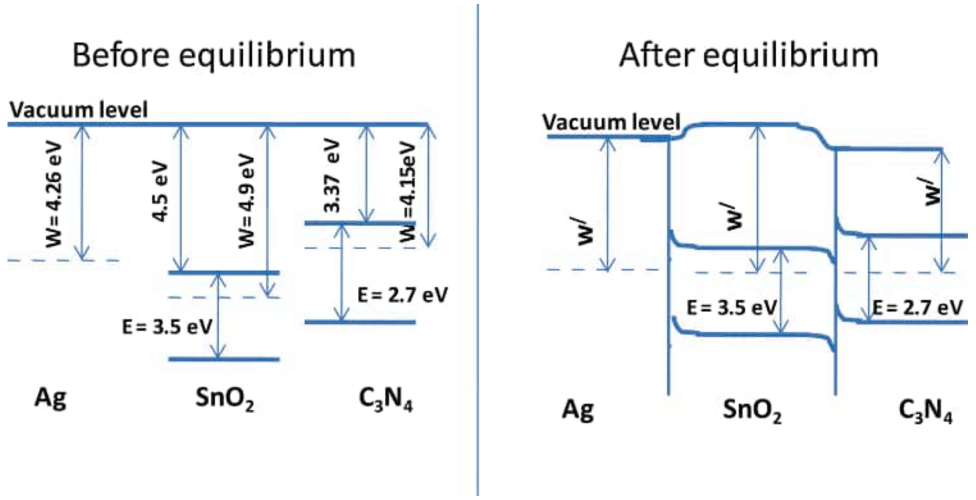

Fig. 8. Energy band configurations of Ag, SnO_2, βC_3N_4 and Ag-SnO_2-βC_3N_4 heterojunction before and after equilibrium.

Declaration of Competing Interest

The authors declare that they have no known competing financial interests or personal relationships that could have appeared to influence the work reported in this paper.

Acknowledgements

Authors would like to thank CeNSE, IISc Bangalore, ACMS IIT Kanpur, CSMCRI Bhavnagar, SAIF NEHU for all the instrumentation facilities. The Director of NIT Silchar is gratefully acknowledged for financial assistance.

References

[1] A.V. Agrawal, R. Kumar, S. Venkatesan, A. Zakhidov, G. Yang, J. Bao, M. Kumar, Photoactivated mixed in-plane and edge-enriched p-Type MoS_2 flake-based NO_2 sensor working at room temperature, ACS sensors 3 (5) (2018) 998–1004.
[2] J. Zeng, Y. Xu, J. Yu, X. Zhang, X. Zhang, H. Jin, J. Jian, Compact Yttria-Stabilized Zirconia Based Total NOx Sensor with a Dual Functional Co_3O_4/NiO Sensing Electrode, ACS sensors 4 (8) (2019) 2150–2155.
[3] E. Wu, Y. Xie, B. Yuan, H. Zhang, X. Hu, J. Liu, D. Zhang, Ultrasensitive and fully reversible NO_2 gas sensing based on p-type $MoTe_2$ under ultraviolet illumination, ACS sensors 3 (9) (2018) 1719–1726.
[4] Z. Li, H. Li, Z. Wu, M. Wang, J. Luo, H. Torun, Y. Fu, Advances in designs and mechanisms of semiconducting metal oxide nanostructures for high-precision gas sensors operated at room temperature, Materials Horizons 6 (3) (2019) 470–506.
[5] P.T. Moseley, Progress in the development of semiconducting metal oxide gas sensors: a review, Measurement Science and Technology 28 (8) (2017), 082001.
[6] Y.F. Sun, S.B. Liu, F.L. Meng, J.Y. Liu, Z. Jin, L.T. Kong, J.H. Liu, Metal oxide nanostructures and their gas sensing properties: a review, Sensors 12 (3) (2012) 2610–2631.
[7] A. Dey, Semiconductor metal oxide gas sensors: A review, Materials Science and Engineering: B 229 (2018) 206–217.
[8] T. Hyodo, K. Urata, K. Kamada, T. Ueda, Y. Shimizu, Semiconductor-type SnO_2-based NO_2 sensors operated at room temperature under UV-light irradiation, Sensors and Actuators B: Chemical 253 (2017) 630–640.
[9] V.L. Patil, S.A. Vanalakar, P.S. Patil, J.H. Kim, Fabrication of nanostructured ZnO thin films based NO_2 gas sensor via SILAR technique, Sensors and Actuators B: Chemical 239 (2017) 1185–1193.
[10] Z. Zhu, S.J. Lin, C.H. Wu, R.J. Wu, Synthesis of TiO_2 nanowires for rapid NO_2 detection, Sensors and Actuators A: Physical 272 (2018) 288–294.
[11] S.S. Shendage, V.L. Patil, S.A. Vanalakar, S.P. Patil, N.S. Harale, J.L. Bhosale, P. S. Patil, Sensitive and selective NO_2 gas sensor based on WO_3 nanoplates, Sensors and Actuators B: Chemical 240 (2017) 426–433.
[12] S. Park, S. An, Y. Mun, C. Lee, UV-enhanced NO_2 gas sensing properties of SnO_2-core/ZnO-shell nanowires at room temperature, ACS applied materials & interfaces 5 (10) (2013) 4285–4292.
[13] A. Dey, Semiconductor metal oxide gas sensors: A review, Materials Science and Engineering: B 229 (2018) 206–217.
[14] E.R. Waclawik, J. Chang, A. Ponzoni, I. Concina, D. Zappa, E. Comini, G. Sberveglieri, Functionalised zinc oxide nanowire gas sensors: Enhanced NO_2 gas sensor response by chemical modification of nanowire surfaces, Beilstein journal of nanotechnology 3 (1) (2012) 368–377.
[15] S.W. Choi, A. Katoch, G.J. Sun, S.S. Kim, Bimetallic Pd/Pt nanoparticle-functionalized SnO_2 nanowires for fast response and recovery to NO_2, Sensors and Actuators B: Chemical 181 (2013) 446–453.
[16] Y. Gui, K. Tian, J. Liu, L. Yang, H. Zhang, Y. Wang, Superior triethylamine detection at room temperature by {-112} faceted WO_3 gas sensor, Journal of hazardous materials 380 (2019), 120876.
[17] Y. Zhong, W. Li, X. Zhao, X. Jiang, S. Lin, Z. Zhen, H. Zhu, High-response room-temperature NO_2 sensor and ultrafast humidity sensor based on SnO_2 with rich oxygen vacancy, ACS applied materials & interfaces 11 (14) (2019) 13441–13449.
[18] J.M. Walker, S.A. Akbar, P.A. Morris, Synergistic effects in gas sensing semiconducting oxide nano-heterostructures: A review, Sensors and Actuators B: Chemical 286 (2019) 624–640.
[19] D.R. Miller, S.A. Akbar, P.A. Morris, Nanoscale metal oxide-based heterojunctions for gas sensing: a review, Sensors and Actuators B: Chemical 204 (2014) 250–272.
[20] H. Naderi, S. Hajati, M. Ghaedi, J.P. Espinos, Highly selective few-ppm NO gas-sensing based on necklace-like nanofibers of ZnO/CdO nn type I heterojunction, Sensors and Actuators B: Chemical 297 (2019), 126774.
[21] J. Sun, L. Sun, N. Han, J. Pan, W. Liu, S. Bai, A. Chen, Ordered mesoporous WO_3/ZnO nanocomposites with isotype heterojunctions for sensitive detection of NO_2, Sensors and Actuators B: Chemical 285 (2019) 68–75.
[22] W. Zeng, Y. Liu, J. Mei, C. Tang, K. Luo, S. Li, Z. He, Hierarchical SnO_2–Sn_3O_4 heterostructural gas sensor with high sensitivity and selectivity to NO_2, Sensors and Actuators B: Chemical 301 (2019), 127010.
[23] S. Bai, H. Fu, Y. Zhao, K. Tian, R. Luo, D. Li, A. Chen, On the construction of hollow nanofibers of ZnO-SnO_2 heterojunctions to enhance the NO_2 sensing properties, Sensors and Actuators B: Chemical 266 (2018) 692–702.
[24] Y. Zou, Y. Xie, S. Yu, L. Chen, W. Cui, F. Dong, Y. Zhou, SnO_2 quantum dots anchored on g-C_3N_4 for enhanced visible-light photocatalytic removal of NO and toxic NO_2 inhibition, Applied Surface Science 496 (2019), 143630.
[25] P. Rai, S.M. Majhi, Y.T. Yu, J.H. Lee, Noble metal@ metal oxide semiconductor core@ shell nano-architectures as a new platform for gas sensor applications, RSC Advances 5 (93) (2015) 76229–76248.
[26] Z. Yuan, R. Li, F. Meng, J. Zhang, K. Zuo, E. Han, Approaches to enhancing gas sensing properties: A review, Sensors 19 (7) (2019) 1495.
[27] F. Xu, H.F. Lv, S.Y. Wu, H.O. Ho-Pui, Light-activated gas sensing activity of ZnO nanotetrapods enhanced by plasmonic resonant energy from Au nanoparticles, Sensors and Actuators B: Chemical 259 (2018) 709–716.
[28] T. Li, W. Zeng, Z. Wang, Quasi-one-dimensional metal-oxide-based heterostructural gas-sensing materials: a review, Sensors and Actuators B: Chemical 221 (2015) 1570–1585.
[29] S.B. Kondawar, A.M. More, H.J. Sharma, S.P. Dongre, Ag-SnO_2/Polyaniline composite nanofibers for low operating temperature hydrogen gas sensor, Journal of Materials Nano Science 4 (1) (2017) 13–18.
[30] T. Zhai, H. Xu, W. Li, H. Yu, Z. Chen, J. Wang, B. Cao, Low-temperature in-situ growth of SnO_2 nanosheets and its high triethylamine sensing response by constructing Au-loaded ZnO/SnO_2 heterostructure, Journal of Alloys and Compounds 737 (2018) 603–612.
[31] C. Wang, S. Zhang, L. Qiu, S.A. Rasaki, F. Qu, T. Thomas, M. Yang, Ru-decorated WO_3 nanosheets for efficient xylene gas sensing application, Journal of Alloys and Compounds 826 (2020), 154196.
[32] A. Mohammad, M.R. Karim, M.E. Khan, M.M. Khan, M.H. Cho, Biofilm-Assisted Fabrication of Ag@ SnO_2-g-C_3N_4 Nanostructures for Visible Light-Induced Photocatalysis and Photoelectrochemical Performance, The Journal of Physical Chemistry C 123 (34) (2019) 20936–20948.
[33] D. Mohanta, S. Raha, M. Ahmaruzzaman, Biogenic green synthetic route for Janus type Ag: SnO_2 asymmetric nanocomposite arrays: Plasmonic activation of wide band gap semiconductors towards photocatalytic degradation of Doripenem, Materials Letters 230 (2018) 203–206.
[34] T. Endo, Gas Adsorption on Surface of Solid Materials. Measurement Techniques and Practices of Colloid and Interface Phenomena, Springer, Singapore, 2019, pp. 119–127.
[35] R. Bardestani, G.S. Patience, S. Kaliaguine, Experimental methods in chemical engineering: specific surface area and pore size distribution measurements—BET, BJH, and DFT, The Canadian Journal of Chemical Engineering 97 (11) (2019) 2781–2791.
[36] Z. Wang, Y. Zhang, S. Liu, T. Zhang, Preparation of Ag nanoparticles-SnO_2 nanoparticles-reduced graphene oxide hybrids and their application for detection of NO_2 at room temperature, Sensors and Actuators B: Chemical 222 (2016) 893–903.
[37] D. Mohanta, K. Barman, S. Jasimuddin, M. Ahmaruzzaman, MnO doped SnO_2 nanocatalysts: Activation of wide band gap semiconducting nanomaterials towards visible light induced photoelectrocatalytic water oxidation, Journal of colloid and interface science 505 (2017) 756–762.
[38] M. Kim, S. Hwang, J.S. Yu, Novel ordered nanoporous graphitic carbon nitride with C_3N_4 stoichiometry as a support for Pt-Ru anode catalyst in DMFC, J. Mater. Chem 17 (2007) 1656–1659.
[39] W. Li, H. Xu, T. Zhai, H. Yu, Z. Chen, Z. Qiu, B. Cao, Enhanced triethylamine sensing properties by designing Au@ SnO_2/MoS_2 nanostructure directly on alumina tubes, Sensors and Actuators B: Chemical 253 (2017) 97–107.
[40] D. Zhang, J. Liu, C. Jiang, A. Liu, B. Xia, Quantitative detection of formaldehyde and ammonia gas via metal oxide-modified graphene-based sensor array combining with neural network model, Sensors and Actuators B: Chemical 240 (2017) 55–65.
[41] Y. Xia, J. Wang, J.L. Xu, X. Li, D. Xie, L. Xiang, S. Komarneni, Confined formation of ultrathin ZnO nanorods/reduced graphene oxide mesoporous nanocomposites for high-performance room-temperature NO_2 sensors, ACS applied materials & interfaces 8 (51) (2016) 35454–35463.
[42] M. Zhang, H.C. Su, Y. Rheem, C.M. Hangarter, N.V. Myung, A rapid room-temperature NO_2 sensor based on tellurium–SWNT hybrid nanostructures, The Journal of Physical Chemistry C 116 (37) (2012) 20067–20074.
[43] J. Wu, Z. Wu, H. Ding, Y. Wei, W. Huang, X. Yang, X. Wang, Three-dimensional graphene hydrogel decorated with SnO_2 for high-performance NO_2 sensing with enhanced immunity to humidity, ACS Applied Materials & Interfaces 12 (2) (2020) 2634–2643.
[44] Z. Wang, T. Han, T. Fei, S. Liu, T. Zhang, Investigation of microstructure effect on NO_2 sensors based on SnO_2 nanoparticles/reduced graphene oxide hybrids, ACS applied materials & interfaces 10 (48) (2018) 41773–41783.
[45] N. Tammanoon, A. Wisitsoraat, C. Sriprachuabwong, D. Phokharatkul, A. Tuantranont, S. Phanichphant, C. Liewhiran, Ultrasensitive NO_2 sensor based on ohmic metal–semiconductor interfaces of electrolytically exfoliated graphene/flame-spray-made SnO_2 nanoparticles composite operating at low temperatures, ACS applied materials & interfaces 7 (43) (2015) 24338–24352.
[46] Q. Li, D. Chen, J. Miao, S. Lin, Z. Yu, Y. Han, Z. An, Ag modified 3D reduced graphene oxide aerogel-based sensor with embedded microheater for fast response and high-sensitive detection of NO_2, ACS Applied Materials & Interfaces. (2020).
[47] P.G. Su, L.Y. Yang, NH_3 gas sensor based on Pd/SnO_2/RGO ternary composite operated at room-temperature, Sensors and Actuators B: Chemical 223 (2016) 202–208.
[48] Z. Wang, S. Gao, T. Fei, S. Liu, T. Zhang, Construction of ZnO/SnO_2 heterostructure on reduced graphene oxide for enhanced nitrogen dioxide sensitive performances at room temperature, ACS sensors 4 (8) (2019) 2048–2057.

[49] R. Kumar, N. Goel, M. Kumar, UV-activated MoS_2 based fast and reversible NO_2 sensor at room temperature, ACS sensors 2 (11) (2017) 1744–1752.
[50] H.R. Kim, A. Haensch, I.D. Kim, N. Barsan, U. Weimar, J.H. Lee, The role of NiO doping in reducing the impact of humidity on the performance of SnO_2-Based gas sensors: synthesis strategies, and phenomenological and spectroscopic studies, Advanced Functional Materials 21 (23) (2011) 4456–4463.
[51] C.A. Zito, T.M. Perfecto, D.P. Volanti, Impact of reduced graphene oxide on the ethanol sensing performance of hollow SnO_2 nanoparticles under humid atmosphere, Sensors and Actuators B: Chemical 244 (2017) 466–474.
[52] C. Struzzi, M. Scardamaglia, J. Casanova-Cháfer, R. Calavia, J.F. Colomer, A. Kondyurin, C. Bittencourt, Exploiting sensor geometry for enhanced gas sensing properties of fluorinated carbon nanotubes under humid environment, Sensors and Actuators B: Chemical 281 (2019) 945–952.
[53] A. Stănoiu, C.E. Simion, S. Somăcescu, NO_2 sensing mechanism of ZnO–Eu_2O_3 binary oxide under humid air conditions, Sensors and Actuators B: Chemical 186 (2013) 687–694.
[54] W. Yan, M.A. Worsley, T. Pham, A. Zettl, C. Carraro, R. Maboudian, Effects of ambient humidity and temperature on the NO_2 sensing characteristics of WS_2/graphene aerogel, Applied Surface Science 450 (2018) 372–379.
[55] H. Li, H. Yu, X. Quan, S. Chen, Y. Zhang, Uncovering the key role of the fermi level of the electron mediator in a Z-scheme photocatalyst by detecting the charge transfer process of WO_3-metal-gC_3N_4 (Metal= Cu, Ag, Au), ACS applied materials & interfaces 8 (3) (2016) 2111–2119.
[56] S. Basu, P.K. Basu, Nanocrystalline metal oxides for methane sensors: role of noble metals, Journal of Sensors 2009 (2009).
[57] V.P. Dinesh, A. Sukhananazerin, P. Biji, An emphatic study on role of spill-over sensitization and surface defects on NO_2 gas sensor properties of ultralong ZnO@Au heterojunction NRs, Journal of Alloys and Compounds 712 (2017) 811–821.
[58] G. Tofighi, D. Degler, B. Junker, S. Müller, H. Lichtenberg, W. Wang, J. D. Grunwaldt, Microfluidically synthesized Au, Pd and AuPd nanoparticles supported on SnO_2 for gas sensing applications, Sensors and Actuators B: Chemical 292 (2019) 48–56.
[59] A. Esfandiar, S. Ghasemi, A. Irajizad, O. Akhavan, M.R. Gholami, The decoration of TiO_2/reduced graphene oxide by Pd and Pt nanoparticles for hydrogen gas sensing, international journal of hydrogen energy 37 (20) (2012) 15423–15432.
[60] Y. Wang, C. Liu, Z. Wang, Z. Song, X. Zhou, N. Han, Y. Chen, Sputtered SnO_2: NiO thin films on self-assembled Au nanoparticle arrays for MEMS compatible NO_2 gas sensors, Sensors and Actuators B: Chemical 278 (2019) 28–38.

Dipyaman Mohanta is a senior research scholar in the Dept. of Chemistry, NIT silchar. He has passed M.Sc. in Chemistry from Assam University. He has published several papers in international journals as well as in various national and international conferences.

Md. Ahmaruzzaman is currently working as an Associate Professor in the Department of Chemistry, National Institute of Technology Silchar. He received his postgraduate degree in M.Sc. (Chemistry) from North Bengal University after his graduation from the same University. He secured first class first position (Gold Medalist) in M.Sc. (Chemistry). He also received his postgraduate degree in M.Tech. (Energy Science & Technology) from Jadavpur University. He obtained his Ph.D. degree from Indian Institute of Technology Delhi. He is Associate Editor of the Frontiers in Environmental Science Journal. He is also the Editorial Board Member of the various international Journals. He has produced seven PhDs and supervised more than 20 projects at the post graduate level. He has published more than 150 research papers in international journals as well as in various national and international conferences. He has more than 6000 Citations in International Journals. He is a reviewer of more than 50 peer-reviewed International and National journals. He was awarded 3 year honorary membership to prestigious American Chemical Society.

Journal of Hazardous Materials 411 (2021) 125120

Contents lists available at ScienceDirect

Journal of Hazardous Materials

journal homepage: www.elsevier.com/locate/jhazmat

Research Paper

Edge-enriched WS_2 nanosheets on carbon nanofibers boosts NO_2 detection at room temperature

Yongshan Xu [a,1], Jiayue Xie [a,1], Yunfan Zhang [b], FengHui Tian [b], Chen Yang [a], Wei Zheng [a], Xianghong Liu [a,c,*], Jun Zhang [a,**], Nicola Pinna [d]

[a] College of Physics, Center for Marine Observation and Communications, Qingdao University, Qingdao 266071, China
[b] State Key Laboratory of Bio-Fibers and Eco-Textiles, College of Chemistry and Chemical Engineering, Qingdao University, Qingdao 266071, China
[c] Key Laboratory of Advanced Energy Materials Chemistry (Ministry of Education), Nankai University, Tianjin 300071, China
[d] Institut für Chemie and IRIS Adlershof, Humboldt-Universität zu Berlin, Brook-Taylor-Str. 2, 12489 Berlin, Germany

ARTICLE INFO

Editor: Dr. H. Zaher

Keywords:
Nanosheets
TMDs
DFT calculation
Edge activity
NO_2 detection

ABSTRACT

Two-dimensional (2D) transition metal dichalcogenides (TMDs) hold great promise for room temperature (RT) NO_2 sensors. However, the exposure of the edges of TMDs with high adsorption capability and electronic activity remains a great obstacle to achieve high sensor sensitivity. Herein, we demonstrate a high-performance RT NO_2 gas sensor based on WS_2 nanosheets/carbon nanofibers (CNFs) composite with abundant intentionally exposed WS_2 edges. Few-layer WS_2 nanosheets are anchored on CNFs through a hydrothermal process. The approach permits to achieve a coating presenting an optimized active surface area and accessibility of the sensing layers. The exposure of WS_2 edges remarkably improves the sensing properties. Consequently, the WS_2@CNFs composite exhibits excellent selectivity to NO_2 at RT with improved response and much lower detection limit in comparison to the WS_2 and CNFs counterparts. Density functional theory (DFT) calculations verify a surprisingly strong NO_2 adsorption on WS_2 edge sites (adsorption energy 3.40 eV) with a partial charge transfer of 0.394e, while a week adsorption on the basal surface of WS_2 (adsorption energy 0.25 eV) with a partial charge transfer of 0.171e. The strategy proposed herein will be instructive to the design of efficient material structures for low-power NO_2 sensors with optimized performances.

1. Introduction

With the emergence of the Internet of Things (IoT), the demand for various high-performance sensors is dramatically increasing (Swan, 2012; Yuan et al., 2019; Hancke and de Carvalho, 2013; Chen et al., 2020). In particular, metal oxide semiconductor (MOS) gas sensors have attracted enormous attention for detection of toxic and explosive molecules because of their easy manufacture, low cost, high sensitivity, high stability, fast response speed and simple sensing mechanism (Walker et al., 2019; Xu et al., 2018; Cho et al., 2020). However, MOS sensors usually work at high temperature (200–450 °C) with high power consumption for heating (Xu et al., 2020; Kumar et al., 2020; Ikram et al., 2019), which is not compatible with applications in IoT and sensor networks requiring low power consumption (Zhang et al., 2016; Kim et al., 2020; Majhi et al., 2021). Room temperature (RT) gas sensors have recently attracted enormous interests due to the zero-power consumption that does not require heating (Kumar et al., 2020; Zhang et al., 2016; Xu et al., 2020), however the relatively low molecular activity on MOS under RT often leads to low sensor response (Wang et al., 2020).

Due to the versatile semiconducting properties and graphene-like structures (Zhang et al., 2019; Zhu et al., 2019; Wang et al., 2019), two-dimensional (2D) transition metal dichalcogenides (TMDs) such as MoS_2 and WS_2 are widely studied in diverse fields, including gas sensor (Liu et al., 2017; Koo et al., 2018; Cha et al., 2017; Zheng et al., 2020). In particular, 2D TMDs are promising candidates as sensing layers for gas sensors due to their vulnerable change of carrier concentration on adsorption of gaseous molecules (Anichini et al., 2018). Among the many harmful gases, NO_2 is the most common gas in chemical synthesis and industrial production, which may cause serious respiratory diseases when the NO_2 concentration exceeds 1 ppm in atmosphere (Ikram et al.,

* Corresponding authors at: College of Physics, Center for Marine Observation and Communications, Qingdao University, Qingdao 266071, China
** Corresponding author.
E-mail addresses: xianghong.liu@qdu.edu.cn (X. Liu), jun@qdu.edu.cn (J. Zhang).
[1] Y.X. and J.X. contributed equally to this work.

https://doi.org/10.1016/j.jhazmat.2021.125120
Received 18 October 2020; Received in revised form 23 December 2020; Accepted 10 January 2021
Available online 13 January 2021
0304-3894/© 2021 Published by Elsevier B.V.

2019). The United States Environmental Protection Agency (U.S. EPA) has formulated an exposure limit of 53 ppb (Zhou et al., 2019; Pham et al., 2019; U.S.E.P., 2016). In addition, NO_2 gas can cause great damage to the environment, such as acid rain (Ou et al., 2015).

Although 2D TMDs gas sensors have been applied to detect NH_3, NO_2, as well as H_2S (Tang et al., 2019; Li et al., 2017; Mayorga-Martinez et al., 2015; Kim et al., 2018), the role played by the basal surface and edges of TMDs is still of great concern in gas sensing. Cho and co-workers (Cho et al., 2015) revealed that the vertically aligned MoS_2 exhibited better NO_2 sensing properties than horizontally aligned MoS_2 nanosheets, due to the low molecular adsorption energy on TMDs edge sites (Choi et al., 2017; Ko et al., 2016; Kim et al., 2019; Shim et al., 2018). In an attempt to increase the exposure of edges of TMDs to improve the gas sensing properties, Cha et al. (2017) reported WS_2 nanoflakes functionalized multi-channel carbon nanofibers (MTCNFs) to detect NO_2. The WS_2@MTCNFs based sensors exhibited notable response of 15% to 1 ppm NO_2 at RT with the response/recovery time of 5.7/3.73 min. Later, Koo et al. (2018) synthesized few-layered WS_2 nanosheets decorated hollow carbon nanocages for RT NO_2 gas sensor, which showed a response of 48.2% to 5 ppm NO_2 and a detection limit of 100 ppb. Although the introduction of carbon materials into WS_2 improved the conductivity and the sensing properties, the sensor performances are still to be optimized.

Herein, we propose a sheet-on-tube structure in order to maximize the exposure of TMDs edges. As a proof-of-concept, 2D WS_2 nanosheets are grown on CNFs via a facile hydrothermal route, which offers abundant highly reactive sites for NO_2 adsorption. The structure-property relationships of the WS_2@CNFs composite for NO_2 detection at RT have been investigated by various characterizations. Experiments reveal that the WS_2@CNFs sensor delivers excellent gas sensing properties in terms of outstanding selectivity, fast response/recovery times and very low detection limit, as well as repeatability. The improved sensing properties are attributed to the synergistic interaction of edge-rich WS_2 nanosheets and high conductivity CNFs. In addition, density functional theory (DFT) simulations also indicate that the edge sites are more active than the basal surface of WS_2 nanosheets, which leads to strong NO_2 adsorption and a more effective charge transfer.

2. Experimental details

2.1. Materials

Tungsten hexachloride (WCl_6, 99.5%), methyl orange ($C_{14}H_{14}N_3SO_3Na$, A.R.), pyrrole (C_4H_5N, ≥ 98%), thioacetamide (C_2H_5NS, ≥ 99%) and ferric chloride ($FeCl_3$, ≥ 97%) were purchased Sinopham Chemical Reagent Co. Ltd. All chemicals were used without further purification.

2.2. Synthesis of CNFs

The CNFs were synthesized using a procedure similar to that described in Ref. (Ma et al., 2019). 0.327 g of methyl orange (MO) was dissolved in 200 ml of distilled water under stirring, and then added 1.622 g of $FeCl_3$ and 0.7 ml of pyrrole (Py) into the solution after the MO was dispersed evenly. After continuous stirring for 12 h, the solution was washed by centrifugation with distilled water and ethanol until a neutral pH is reached, and then vacuum dried overnight to obtain polypyrrole (Ppy) nanofibers. Finally, the Ppy was annealed at 600 °C for 5 h under Ar atmosphere to convert into CNFs.

2.3. Preparation of WS_2 and WS_2@CNFs

The WS_2 nanosheets were synthesized through a hydrothermal process reported before (Reddy et al., 2017). 0.9 g of WCl_6 and 1.7 g of thioacetamide were added into 30 ml of distilled water under ultrasonication for 30 min. Then 100 mg of as-prepared CNFs were added into the solution and ultrasonication for 50 min. The obtained solution was transferred to a Teflon-lined stainless steel autoclave (50 ml) and heated at 200 °C for 12 h. The final products were washed with distilled water and ethanol several times, then dried at 80 °C and annealed at 600 °C for 5 h under Ar atmosphere to obtain the ultrathin WS_2 nanosheets functionalized CNFs. In addition, the same hydrothermal process without addition of CNFs was carried out to synthesize pristine WS_2 nanosheets for comparison.

2.4. Materials characterization

X-ray diffraction (XRD) patterns of the samples were obtained by a Rigaku Smartlab with Cu Kα radiation (40 kV, 40 mA) at RT. Scanning electron microscope (SEM) images were obtained with Zeiss sigma 300. Transmission electron microscope (TEM) and High-resolution TEM (HRTEM) was carried out on a FEI JEM-2010 with an acceleration voltage of 200 kV. Scanning transmission electron microscopy (STEM) and energy dispersive spectroscopy (EDS) were performed on a FEI TALOS F200. Surface composition of the samples was performed by X-ray photoelectron spectroscopy (XPS, Thermo ESCALAB 250, Al Kα 1486.6 eV). Raman spectra was collected on Renishaw with Ar^+ laser excitation at 532 nm. Ultraviolet photoelectron spectroscopy (UPS) was obtained from Thermo Fisher Scientific ESCALAB 250xi, using He-Iα 21.2 eV light, and a pass energy and energy resolution of 1.2 and 0.01 eV.

2.5. Fabrication of gas sensor

First, a glass plate was used as the substrate to prepare the interdigital electrodes using Ti/Au (10/30 nm) in a Thermal Evaporation System. Then 100 μL of the ultrasonically dispersed samples (0.1 g in 1 ml ethanol) was dropped onto the interdigital electrodes to form the sensing layers.

2.6. Gas sensing characterization

The gas sensing characteristics were examined using a home-made static test equipment. The sensor was connected to the test equipment (Keithley 2400) to measure the electrical current evolution. The gas concentration to be measured is generated by injecting a calculated volume of NO_2 or liquid onto the heating plate. The response (S) was calculated by using the sensor resistance before (R_a) and after (R_g) exposure to target gases, $S = R_a/R_g$ for p-type semiconductor for oxidizing gases and $S = R_g/R_a$ for reducing gases. The response time is defined as the time taken to reach 90% of the full response after exposure to analytes. The recovery time is the time taken to return to 90% after the analyte is removed.

2.7. Density functional theory (DFT) calculation

All of the DFT simulations were performed using the $Dmol^3$ package in the Material Studio. The generalized gradient approximation (GGA) functional of Perdew–Burke–Ernzerhof (PBE) was adopted to express the exchange correlation terms. The 1 × 2 × 1 Monkhorst-Pack k-points were used. The vacuum distance is set as 30 Å to eliminate interference between interlayers. The adsorption energy (E_{ads}) is utilized to evaluate the interaction between NO_2 and WS_2 nanosheets, which can be calculated as

$$E_{ads} = E_{mol} + E_{surface} - E_{mol/surface} \quad (1)$$

where E_{mol} is the energy of an isolated NO_2 molecule in the vacuum, $E_{surface}$ is the energy of clean surface slab and $E_{mol/surface}$ is the total energy of the surface with NO_2 adsorption. According to this equation, a positive value of E_{ads} indicates a favorable adsorption. Mulliken population is used to check the charge transfer between gas and surface.

3. Results and discussion

3.1. Structural and morphological characteristics

Fig. 1a illustrates the synthesis process of the sensing materials and final sensors. First, Ppy tubes were generated by the polymerization of Py, and then further annealed to obtain CNFs. Next, the prepared CNFs and the precursors of WS_2 were hydrothermally reacted together, and the resulting products were annealed to prepare ultrathin WS_2 sheets functionalized CNFs. In addition, the sensor used for the test is obtained by drop-coating ultrasonically dispersed sensing materials onto the surface of interdigital electrodes. Additional information can be found in the Experimental details.

The crystalline structure and phase composition of WS_2 and WS_2@CNFs were investigated by XRD analysis, as shown in Fig. 1b. The dominant diffraction peaks in the XRD patterns correspond to the (002), (004), (100), (103), and (110) planes of hexagonal WS_2 phase (JCPDS card NO. 84-1398), demonstrating that the precursor of tungsten successfully transforms to WS_2. The broadening of diffraction peaks and apparent asymmetry in (100) and (110) planes indicate the nanoscale dimension of WS_2 with ultrathin thickness (Zeng et al., 2016). The nanocrystal size calculated according to the Scherrer formula is 4.7 nm, which is about the thickness of 5–6 layers of WS_2 (Liu et al., 2020). This result is consistent with the TEM shown in Fig. 2f. In addition, some impurity peaks can be seen in WS_2@CNFs, which is attributed to the partial oxidation of WS_2 to WO_3 during the annealing process.

The morphology of CNFs, WS_2 and WS_2@CNFs were identified by SEM. As can be seen from Fig. 1c and d, the average diameter of as-synthesized CNFs is 150 nm, and some nanofibers have a square hollow structure and nothing is attached to the surface. Fig. 1e shows the low magnification SEM image of WS_2, which prove that the as-synthesized WS_2 consists of nanosheets aggregated in spherical structures. From the high magnification SEM image (Fig. 1f), it can be seen that the prepared nanosheets are packed tightly with width ranging from 50 to 150 nm. With the participation of CNFs, the WS_2 nanosheets are closely confined on the surface of the CNFs (Fig. 1g). As can be seen from Fig. 1h, the WS_2 nanosheets are attached vertically to the surface of CNFs, exposing abundant edge sites. No aggregation of WS_2 is observed, indicating that the CNFs can be used as an efficient substrate for the nucleation and growth of WS_2 nanosheets.

The microstructure of CNFs, WS_2 and WS_2@CNFs were investigated in detail by TEM and HRTEM. The hollow structure of the CNFs can be clearly seen from Fig. 2a. The HRTEM of CNFs in Fig. 2b reveals an amorphous structure. The TEM image of pristine WS_2 reveal an aggregation of the nanosheets, as shown in Fig. 2c. The lattice spacing in the HRTEM of the WS_2 (Fig. 2d) is estimated to be 0.27 nm, corresponding to the (100) basal planes of WS_2 (Zeng et al., 2016; Koo et al., 2018). The TEM in Fig. 2e shows the hierarchical sheet-on-tube structure of WS_2@CNFs. Ultrathin WS_2 nanosheets are uniformly distributed on the surface of the CNFs, exposing abundant free-standing edges. The loose structure and the rich space between the nanosheets can facilitate the gas adsorption and diffusion. The HRTEM image of WS_2@CNFs (Fig. 2f) confirms that the WS_2 nanosheets on CNFs have three to five layers. The lattice spacing in Fig. 2f is measured to be 0.62 nm, corresponding to the (002) planes of WS_2 (Zeng et al., 2016; Alagh et al., 2021). STEM and EDS mapping were also carried out to confirm the distribution of WS_2 nanosheets onto the CNFs. As can be seen from Fig. 2g, the homogeneous distribution of W, S, and C demonstrates the WS_2 nanosheets are uniformly grafted on the surface of CNFs.

Raman spectra of CNFs, WS_2 and WS_2@CNFs samples is shown in

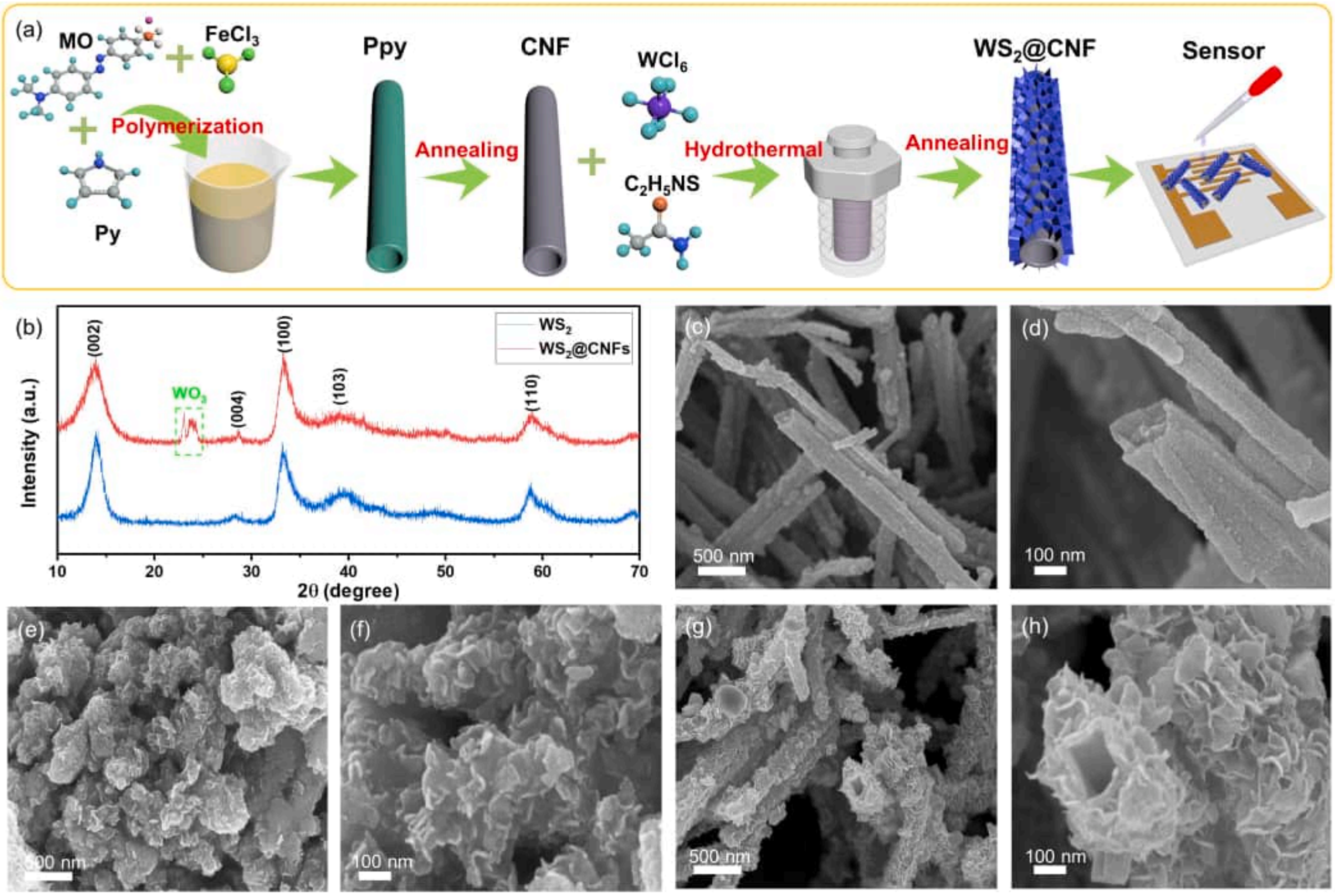

Fig. 1. (a) Schematic illustration of fabrication of the sensor based on WS_2@CNFs. (b) XRD pattern of WS_2 and WS_2@CNFs. SEM images of (c, d) CNFs, (e, f) WS_2, and (g, h) WS_2@CNFs.

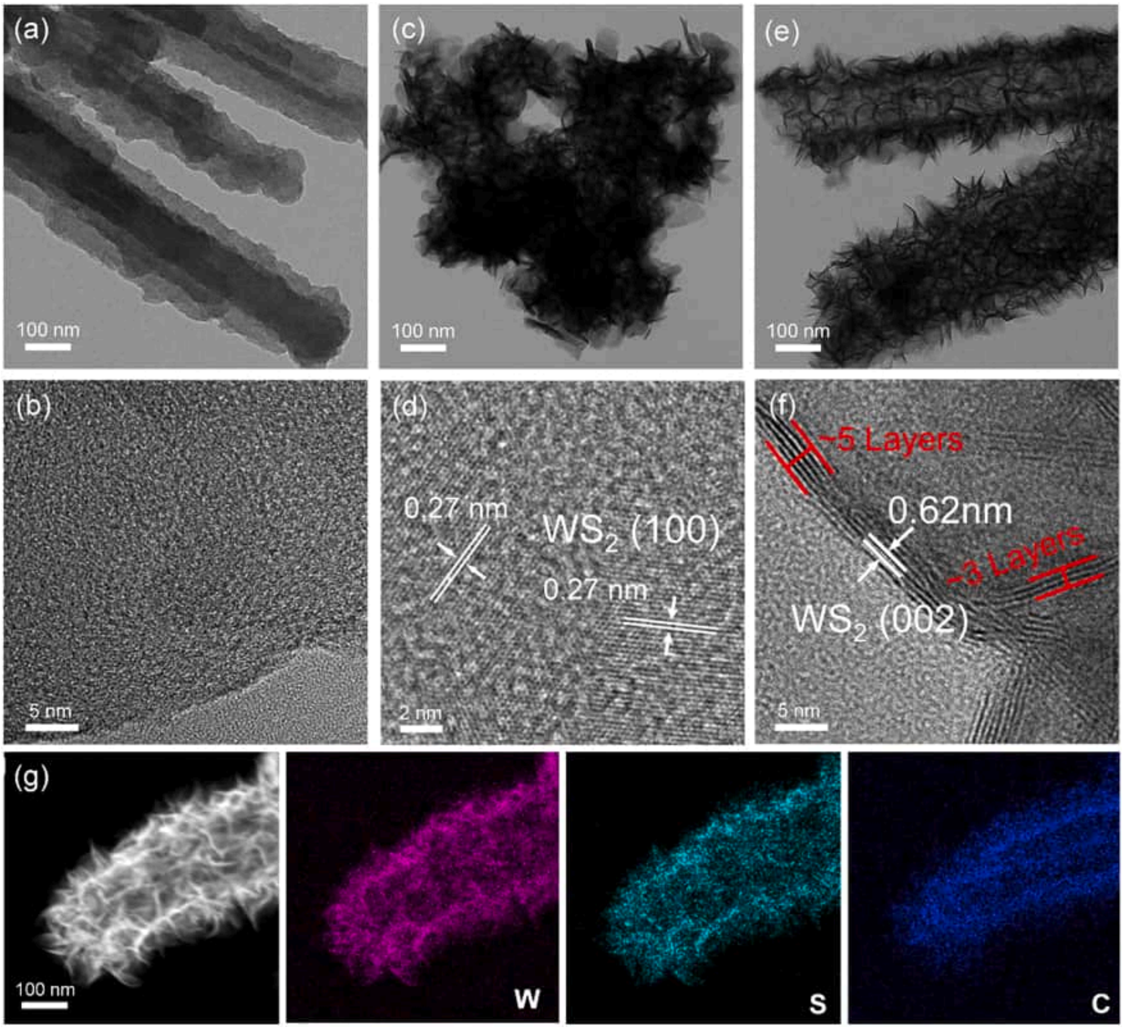

Fig. 2. TEM and HRTEM images of (a, b) CNFs, (c, d) WS_2, and (e, f) WS_2@CNFs. (g) STEM and EDS elemental mapping images of WS_2@CNFs.

Fig. 3a. The peak located at 348 cm^{-1} in WS_2 and WS_2@CNFs is the E^1_{2g} range for in-plane vibrations of WS_2, and the peak at 414 cm^{-1} in WS_2 and WS_2@CNFs can be assigned to A_{1g} range out-of-plane vibrations of WS_2. Besides the two typical peaks for WS_2, the fingerprint peaks of "D" (1336 cm^{-1}) and "G" (1592 cm^{-1}) within the spectra range from 1200 to 1800 cm^{-1} in the CNFs and WS_2@CNFs can be attributed to the CNFs (Wang et al., 2019; Berkdemir et al., 2013). In addition, a unique feature observed in the Raman spectra is that the intensity ratio of E^1_{2g}/A_{1g} decreases from 1.3 for bulk WS_2 to 1.2 for WS_2@CNFs, indicating a dominant exposure of the edge sites (Cho et al., 2015).

Fig. 3b–d shows the XPS spectra of WS_2 and WS_2@CNFs. As can be seen in Fig. 3b, the W 4f core level spectrum of the samples exhibits the W $4f_{7/2}$, W $4f_{7/2}$ and W $5p_{3/2}$ peaks at 33.28, 35.38, and 38.78 eV for WS_2, respectively. Meanwhile, the peaks located at 36.38 and 38.68 eV can be attributed to WO_3, indicating that the crystalline WS_2 is partially oxidized to WO_3 due to the high reactivity in air (Cha et al., 2017; Gao et al., 2016). For S 2p high-resolution spectrum of WS_2 and WS_2@CNFs (Fig. 3c), the strong absorption peaks in 162.88 and 164.08 eV can be assigned to S $2p_{3/2}$ and S $2p_{1/2}$, demonstrating that the existence of S^{2-}. Furthermore, it can be seen that the S 2p spectrum of two samples are offset by 0.4 eV, suggesting the charges transfer between WS_2 and CNFs, which contribute to close contacts in the heterojunctions (Han et al., 2019). WS_2 has a higher work function than that of the CNFs, resulting in the electrons in CNFs transferred to WS_2 (Cha et al., 2017). Moreover, the C 1s spectrum is shown in Fig. 3d and can be matched with C–C, C–N or C–O and C=O at 284.58, 285.78, 288.68 and 290.78 eV, respectively (Wu et al., 2020).

3.2. Characterization of gas sensing performances

The gas sensing performances of CNFs, WS_2, and WS_2@CNFs were evaluated upon exposure to NO_2 in dry condition at RT to verify distinct edge effects of WS_2 on the surface of CNFs. Fig. 4a exhibits the dynamic resistance transients of CNFs, WS_2, and WS_2@CNFs towards 10 ppm of NO_2, it is observed that the resistance of the samples decreases gradually when exposed to NO_2, and then gradually returns to the original state with the removal of NO_2, showing the typical p-type semiconductor property. The initial resistance of CNFs, WS_2 and WS_2@CNFs are 26.1, 54.3, and 33.3 MΩ, respectively, demonstrating that the presence of carbon can increase the conductivity of the sensing materials. In terms of response and recovery time (τ_{res} and τ_{rec}), compared with the short τ_{res} (38 s) and long τ_{rec} (427 s) of CNFs and the long τ_{res} (90 s) and short τ_{rec} (39 s) of WS_2, the WS_2@CNFs sensor shows a relatively moderate response and recovery speed (54 and 305 s). The relatively long response and recovery is due to that the gas molecules lack sufficient energy to overcome the barrier during adsorption and desorption at RT. In addition, the WS_2@CNFs displays a larger resistance change than that of CNFs and WS_2 in NO_2 atmosphere. This result indicates that WS_2@CNFs has the highest response value. The corresponding response of CNFs, WS_2 and WS_2@CNFs to 10 ppm NO_2 are 1.63, 1.13, and 2.11, respectively, as shown in Fig. 4b, revealing the significant promotion by the edge-enriched WS_2@CNFs hierarchical structure. Furthermore, the

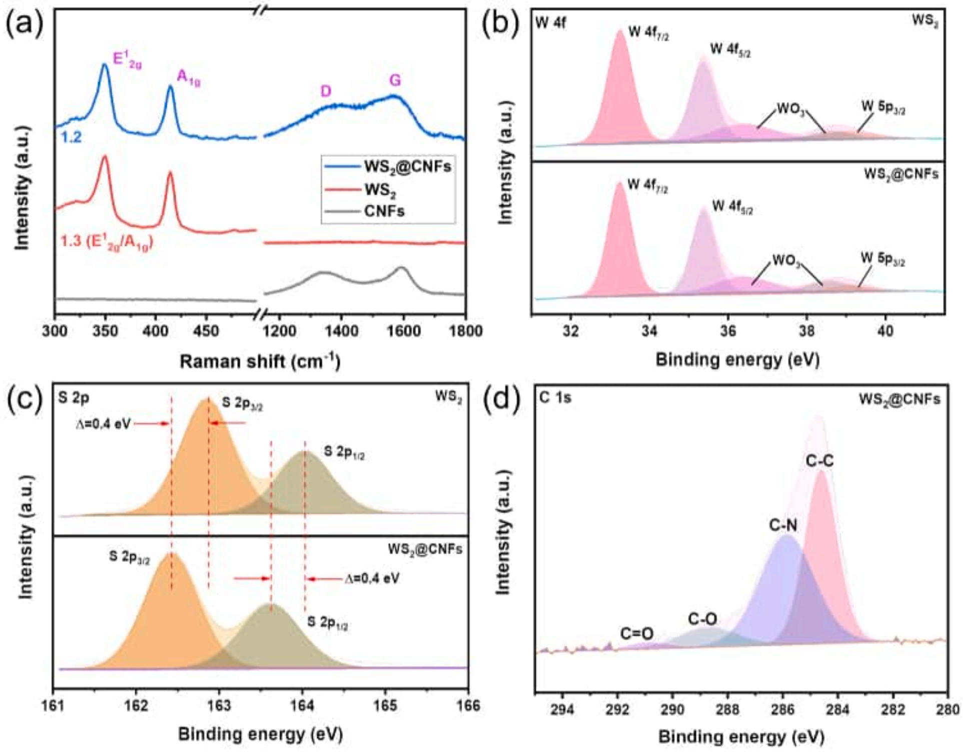

Fig. 3. (a) Raman spectra of CNFs, WS_2 and WS_2@CNFs. XPS spectra of WS_2 and WS_2@CNFs showing (b) W 4f and (c) S 2p core level spectrum, and (d) C 1s core level spectrum of WS_2@CNFs.

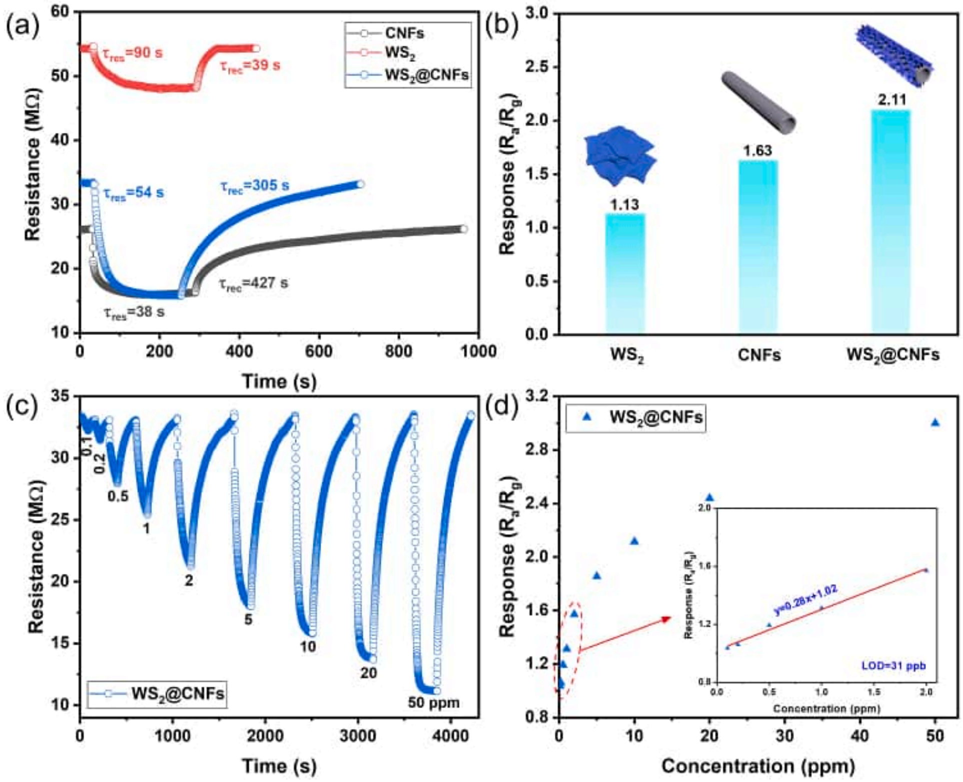

Fig. 4. (a) Dynamic resistance transients of CNFs, WS_2, and WS_2@CNFs towards 10 ppm of NO_2 at RT, and (b) corresponding response. (c) Dynamic resistance transients of WS_2@CNFs towards 0.1–50 ppm of NO_2 at RT, and (d) response versus NO_2 concentration plot of WS_2@CNFs.

response of CNFs, WS_2 and WS_2@CNFs to 10 ppm NO_2 at different temperature was measured and shown in Fig. S1a. It can be seen that the response of WS_2 and WS_2@CNFs at 150 °C is 5.12 and 6.9, respectively. At the same time, the activity of gas molecules is higher at high temperature, and it is easier to overcome the barrier of adsorption and desorption, showing a faster response and recovery speed (Wang et al., 2010). The response and recovery time of WS_2@CNFs at 150 °C is only 23 and 94 s (Fig. S1b). Fig. 4c shows the dynamic resistance transients of WS_2@CNFs towards 0.1–50 ppm of NO_2. It is clear that the higher the concentration of NO_2, the greater the change of resistance of the sensor. Meanwhile, WS_2@CNFs exhibits the fast response and recovery speed at each concentration. The responses of WS_2@CNFs to 0.1–50 ppm of NO_2 are 1.04, 1.06, 1.19, 1.31, 1.57, 1.86, 2.11, 2.44 and 3, respectively. The response values are plotted as a function of NO_2 concentration in Fig. 4d. A linear relationship between the sensor response and the concentration of 0.1–2 ppm was obtained: $y = 0.28x + 1.02$, which reveals the sensitivity of WS_2@CNFs is 0.28 ppm^{-1}. Although 0.1 ppm of NO_2 was the lowest concentration tested experimentally, the theoretical limit of detection (LOD) of WS_2@CNFs is calculated to be 31 ppb (The detailed calculation process is in the Supporting Information S4). Since NO_2 exposure causes skin damage and respiratory disorders, the U.S. EPA has regulated a limit of exposure to NO_2 as 53 ppb (U.S.E.P., 2016). The superior LOD of WS_2@CNFs is far beyond the threshold limit value, indicating its great application potential for NO_2 gas sensor.

In terms of practical application of gas sensors, a favorable feature of the sensor is to selectively distinguish NO_2 gas among other analytes. Fig. 5a displays the response of CNFs, WS_2, and WS_2@CNFs on exposure to diverse interfering gases including 50 ppm NO_2, triethylamine, NH_3, N-butanol, acetone, ethanol, methanol, formaldehyde, H_2, CO_2, CO, H_2S, and SO_2. It can be seen that the CNFs shows almost no differentiation among various gases due to the very low response to each gas. However, when WS_2 is functionalized on CNFs, the response to NO_2 is greatly improved, while the response to other gases is not increased. The results indicate that the edge-rich WS_2@CNFs structure can effectively improve the selectivity to NO_2. It is believed that the significant variation of selectivity originated from high density of WS_2 edges grafted on the surface of CNFs and that WS_2 edges can improve the adsorption of NO_2 molecules due to the higher adsorption energy for NO_2 molecules and achieve significant charge transfer, resulting in good reactivity with adsorbed oxygen molecules compared with other reducing and oxidizing gases at RT (Cha et al., 2017; Ikram et al., 2020). Principal Component Analysis (PCA) is a powerful statistical technique for data analysis, which can provide the information of the gas sensor to discriminate target gas component. Fig. 5b shows the PCA plot of WS_2@CNFs by determining gas response, response and recovery times upon exposure to various gases. The PCA result reveals that WS_2@CNFs based sensor is able to successfully identify NO_2 from all the chemical analytes tested.

For practical application, the influence of ambient humidity on the gas sensor response must be considered. As shown in Fig. 5c, the resistance of WS_2@CNFs in air decreases with the increase of humidity from 30% to 90%, which implies the sensor resistance in air is affected by the humidity. The corresponding response of WS_2@CNFs is displayed in Fig. S2, the response under 90% humidity is lower than those under lower humidity. This is because water molecules adsorbed on the surface of WS_2@CNFs are in competition with the adsorption of NO_2 molecules (Guo et al., 2017).

Fig. 5d shows dynamic resistance transients of WS_2@CNFs toward 50 ppm of NO_2 at RT for 5 consecutive cycles. It is clear that all 5 exposures to NO_2 show almost the same level of resistance change, in terms of both resistance reduction and response-recovery speed. After ten months, the sensor was tested again for 5 consecutive times, and the results were not significantly different from before (Fig. 5e). Moreover, the relatively stable responses of two tests are presented in the Fig. 5f, indicating that the sensor based on edge-rich WS_2@CNFs structure has an excellent repeatability and stability.

In comparison to other edge-emphasized sensors (Table S1), although some sensors can show higher response value and fast response-recovery speed, they often require heating or the use of

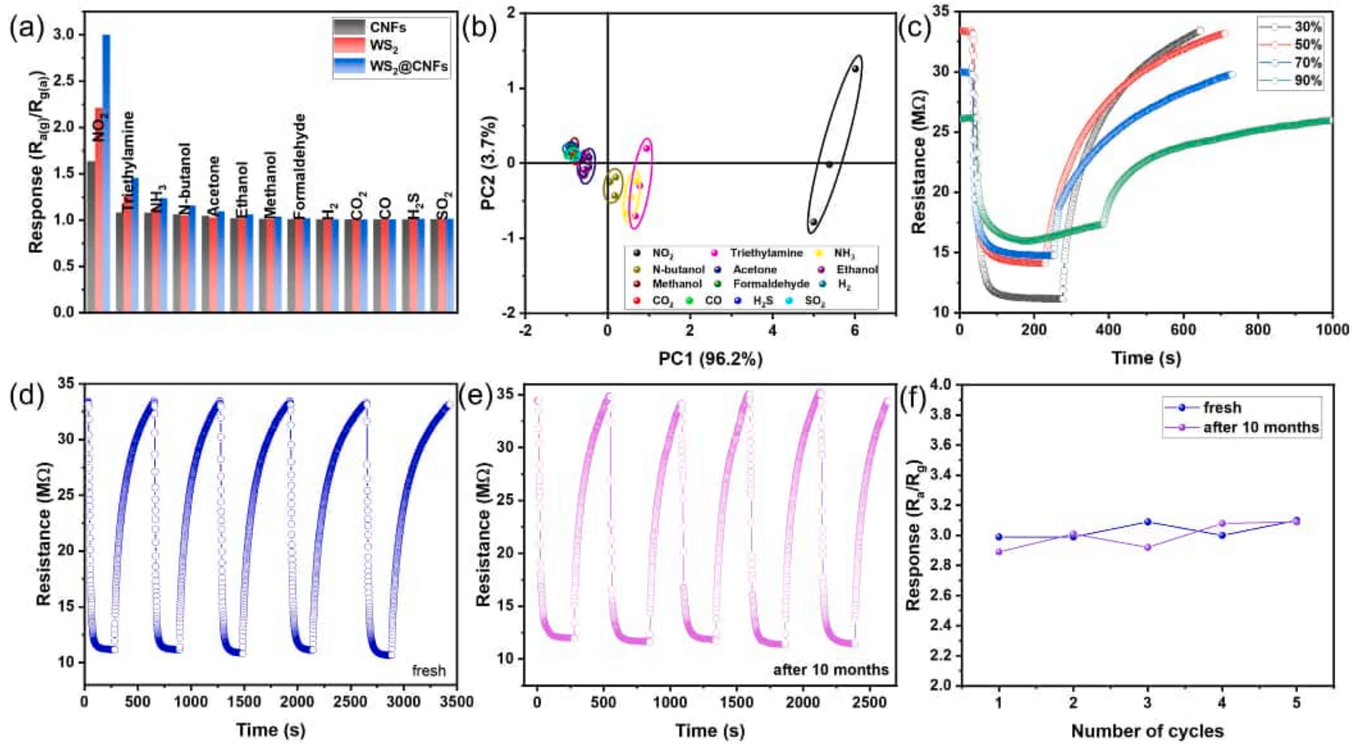

Fig. 5. (a) The selectivity of CNFs, WS_2, and WS_2@CNFs to 50 ppm of various gases. (b) Principal Component Analysis (PCA) plot of WS_2@CNFs. (c) Dynamic resistance change curves of WS_2@CNFs at different humidity at RT. (d) Dynamic resistance transients of fresh WS_2@CNFs and (e) WS_2@CNFs after 10 months exposed to 50 ppm of NO_2 for five times and (f) corresponding response.

ultraviolet (UV) light assisted methods to achieve this (Shim et al., 2018; Li et al., 2019; Agrawal et al., 2018). Furthermore, although some sensors have low LOD, their response are also weak (Cha et al., 2017; Ikram et al., 2020). Thus, the WS_2@CNFs reported in this work exhibits remarkable advantages including response value, response-recovery speed and LOD at RT.

The sensing mechanism of CNFs, WS_2, and WS_2@CNFs is based on a charge transfer between the sensing materials and the target gases (Cha et al., 2017; Cho et al., 2015). As an oxidizing gas, NO_2 plays a role as an electron acceptor. As can be seen in Fig. 6a, upon exposure to NO_2, NO_2 molecules began to be adsorbed on the surface of CNFs and attracted electrons from CNFs. As a result, the number of holes increases in p-type CNFs and the resistance of CNFs decreases. For pure WS_2 nanosheets (Fig. 6b), as it is a tightly aggregated multilayer structure, only a minimum of active sites is exposed to the surroundings. Hence, gas adsorption can only occur on partially exposed surfaces, and this results in a low response. As for the WS_2@CNFs (Fig. 6c), the enhanced gas sensing properties can be attributed to the increased specific surface area and the significant increase of edge sites originated from the grafted growth of WS_2 nanosheets on the surface of the CNFs. This hierarchical structure improves the adsorption and diffusion of NO_2 within the space between WS_2 nanosheets. Thus, the abundant WS_2 edges promote the adsorption of NO_2 as well as the reaction kinetics. In addition, the hollow structure of WS_2@CNFs allowed significantly increased reaction sites on CNFs to NO_2 molecules. Furthermore, electron deficiency in WS_2 could be compensated by electrons donated from CNFs. Therefore, the additional change of resistance in WS_2@CNFs through electron transfer between WS_2 and CNFs result in excellent sensing properties compared to that of pure CNFs and WS_2. The work function of CNFs and WS_2 was obtained from UPS, as shown in Fig. S3, which can be calculated as $\Phi = h\nu - (E_{co} - E_f)$, where E_{co} is the secondary electron cut-off energy and E_f is the fermi level of the sample (Forti et al., 2017). The value of CNFs and WS_2 obtained in this way is 3.6 and 6.2 eV, respectively. The corresponding band diagram of WS_2@CNFs is shown in Fig. 6d. Due to the different work function of the CNFs and WS_2, electrons in the CNFs are favorably transferred to the WS_2 nanosheets, leaving holes in CNFs. The obtained gas sensing characteristics curves in Fig. 4a indicate that CNFs and WS_2@CNFs possess a p-type sensing property with decrease in resistance toward NO_2, which is known as an oxidizing gas (Cha et al., 2017). When the sensor is exposed to NO_2, the electrons are further transferred to NO_2. When the sensor is exposed to air again, NO_2 is desorbed from the surface of sensing materials and returns electrons to the system.

3.3. DFT calculation

In order to understand the improved sensing performance of the hybrid WS_2@CNFs materials, DFT calculations are performed to simulate the adsorption and charge transfer between NO_2 gas and two WS_2 surfaces, including the basal (001) and the edge (010) surfaces. The results are shown in Fig. 7, indicating that the existence of WS_2 edges is crucial to the improvement of the sensing performance. On the basal surface (Fig. 7a and b), it is found that the maximum NO_2 adsorption energy is only 0.25 eV, irrespective of the different adsorption sites including W, S and hole. This result reveals the NO_2 is subject to a weak adsorption on the basal surface of WS_2. On the edge surface (Fig. 7c), the adsorption energy at W site is surprisingly as high as 3.40 eV, showing a very strong adsorption. This strong adsorption can induce a large charge transfer compared to the adsorption on the basal planes, thereby leading to a remarkable sensitivity of WS_2@CNFs. Based on DFT simulation, the partial charge transfer from WS_2 edges to NO_2 is 0.394 e with W atoms serving as the adsorption sites, while the charge transfer related to NO_2 adsorption on the basal surface is only 0.171 e. Therefore, the above DFT calculation clearly point out that the edges of WS_2 are more active than the basal surface for NO_2 adsorption to achieve a significant sensor response, which is in good agreement with our experimental results.

To further understand the charge transfer mechanism, a charge density analysis of NO_2 adsorption on the WS_2 (010) surface, i.e., the edge sites, is shown in Fig. 7c. As can be seen, when adsorption occurs, electrons are more concentrated between surface W and O atom of NO_2. This is also consistent with the larger charge transfer amount of 0.394 e and the phenomenon that the distance between W and O atoms is decreased from the initial distance of 2.856–2.137 Å. This further confirms that the exposure of edges is crucial for improving the sensor sensitivity of NO_2 detection.

4. Conclusion

In conclusion, a highly sensitive and selective NO_2 gas sensor is successfully developed based on WS_2@CNFs nanocomposites with

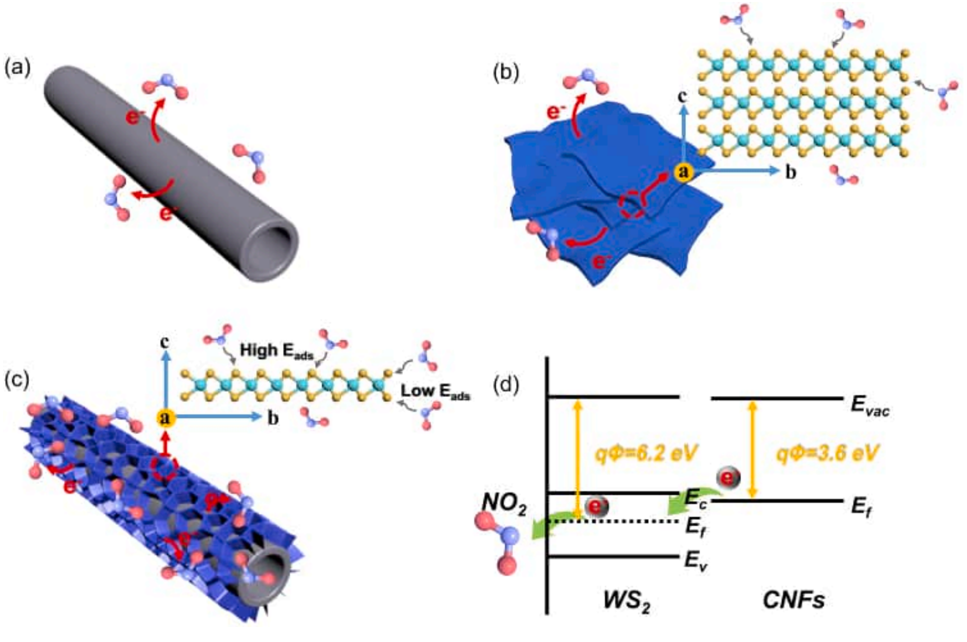

Fig. 6. Schematic illustration of the sensing mechanism of (a) CNFs, (b) WS_2, and (c) WS_2@CNFs, and (d) band diagram for WS_2@CNFs.

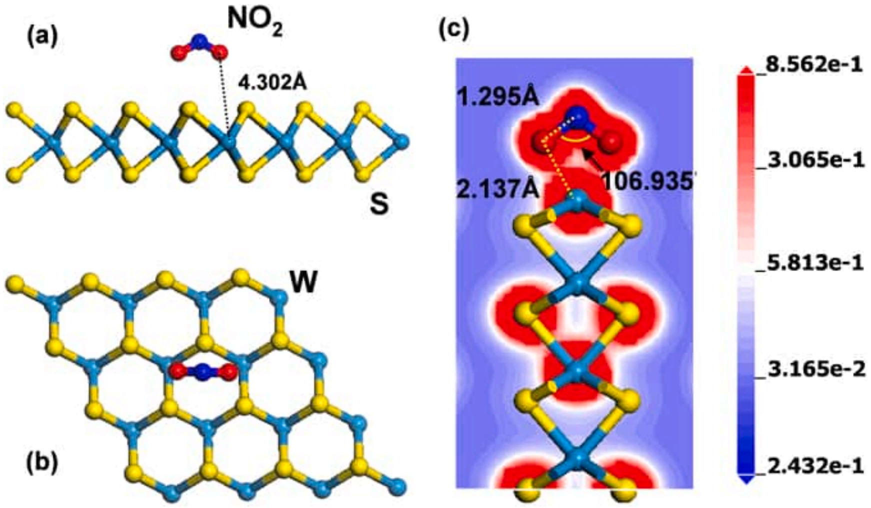

Fig. 7. Adsorption conformations and charge density of NO_2 molecule on WS_2 surfaces. (a) Side and (b) Top view of NO_2 on the WS_2 (001) surface. (c) The charge density diagram of NO_2 adsorption on the (010) surface.

abundant intentionally exposed WS_2 edge sites. The WS_2@CNFs sensor delivers remarkable sensing response, excellent repeatability and selectivity, as well as ultralow LOD towards NO_2 detection, making our material suitable for practical application. The improved response and selectivity is due to the synergistic contribution from WS_2 edge-rich structure and high conductivity of CNFs. The underlying sensing mechanism is interpreted by DFT calculations, which demonstrated that the edge sites of WS_2 are more beneficial to NO_2 adsorption with an improved electron transfer compared to the basal surface of WS_2. This work contributes to the understanding of sensing mechanism of TMDs materials and provides new hints to develop high performance RT gas sensors by designing edge-enriched TMDs nanostructures.

CRediT authorship contribution statement

Yongshan Xu and Jiayue Xie conducted material synthesis, analyzed the data and wrote the manuscript. Yunfan Zhang and Fenghui Tian contributed to DFT calculation and analysis. Chen Yang and Wei Zheng contributed to the gas sensor test. Xianghong Liu, Jun Zhang and Nicola Pinna reviewed and revised the manuscript. The corresponding authors Xianghong Liu and Jun Zhang ensure that the descriptions are accurate and submission of this manuscript was agreed by all authors.

Declaration of Competing Interest

The authors declare that they have no known competing financial interests or personal relationships that could have appeared to influence the work reported in this paper.

Acknowledgments

This work is financially supported by the National Natural Science Foundation of China (No. 61971252 and 51972182), the Shandong Provincial Natural Science Foundation (ZR2020JQ27 and ZR2019BF008), the Youth Innovation Team Project of Shandong Provincial Education Department (2020KJN015) and Qingdao Applied Fundamental Research Project (19-6-2-71-cg).

Appendix A. Supporting information

Supplementary data associated with this article can be found in the online version at doi:10.1016/j.jhazmat.2021.125120.

References

Agrawal, A.V., Kumar, R., Venkatesan, S., Zakhidov, A., Yang, G., Bao, J., Kumar, M., Kumar, M., 2018. Photoactivated mixed in-plane and edge-enriched p-type MoS_2 flake-based NO_2 sensor working at room temperature. ACS Sens. 3, 998–1004.

Alagh, A., Annanouch, F.E., Umek, P., Bittencourt, C., Sierra-Castillo, A., Haye, E., Colomer, J.F., Llobet, E., 2021. CVD growth of self-assembled 2D and 1D WS_2 nanomaterials for the ultrasensitive detection of NO_2. Sens. Actuators B Chem. 326, 128813.

Anichini, C., Czepa, W., Pakulski, D., Aliprandi, A., Ciesielski, A., Samorì, P., 2018. Chemical sensing with 2D materials. Chem. Soc. Rev. 47, 4860–4908.

Berkdemir, A., Gutiérrez, H.R., Botello-Méndez, A.R., Perea-López, N., Elías, A.L., Chia, C.-I., Wang, B., Crespi, V.H., López-Urías, F., Charlier, J.-C., Terrones, H., Terrones, M., 2013. Identification of individual and few layers of WS_2 using Raman spectroscopy. Sci. Rep. 3, 1755.

Cha, J.-H., Choi, S.-J., Yu, S., Kim, I.-D., 2017. 2D WS_2-edge functionalized multi-channel carbon nanofibers: effect of WS_2 edge-abundant structure on room temperature NO_2 sensing. J. Mater. Chem. A 5, 8725–8732.

Chen, W.Y., Jiang, X., Lai, S.-N., Peroulis, D., Stanciu, L., 2020. Nanohybrids of a MXene and transition metal dichalcogenide for selective detection of volatile organic compounds. Nat. Commun. 11, 1302.

Cho, I., Sim, Y.C., Cho, M., Cho, Y.-H., Park, I., 2020. Monolithic micro light-emitting diode/metal oxide nanowire gas sensor with microwatt-level power consumption. ACS Sens. 5, 563–570.

Cho, S.Y., Kim, S.J., Lee, Y., Kim, J.-S., Jung, W.-B., Yoo, H.-W., Kim, J., Jung, H.-T., 2015. Highly enhanced gas adsorption properties in vertically aligned MoS_2 layers. ACS Nano 9, 9314–9321.

Choi, W., Choudhary, N., Han, G.H., Park, J., Akinwande, D., Lee, Y.H., 2017. Recent development of two-dimensional transition metal dichalcogenides and their applications. Mater. Today 20, 116–130.

Forti, S., Rossi, A., Büch, H., Cavallucci, T., Bisio, F., Sala, A., Menteş, T.O., Locatelli, A., Magnozzi, M., Canepa, M., Müller, K., Link, S., Starke, U., Tozzini, V., Coletti, C., 2017. Electronic properties of single-layer tungsten disulfide on epitaxial graphene on silicon carbide. Nanoscale 9, 16412–16419.

Gao, J., Li, B., Tan, J., Chow, P., Lu, T.-M., Koratkar, N., 2016. Aging of transition metal dichalcogenide monolayers. ACS Nano 10, 2628–2635.

Guo, H., Lan, C., Zhou, Z., Sun, P., Wei, D., Li, C., 2017. Transparent, flexible, and stretchable WS_2 based humidity sensors for electronic skin. Nanoscale 9, 6246–6253.

Han, Y., Liu, Y., Su, C., Wang, S., Li, H., Zeng, M., Hu, N., Su, Y., Zhou, Z., Wei, H., 2019. Interface engineered WS_2/ZnS heterostructures for sensitive and reversible NO_2 room temperature sensing. Sens. Actuators B Chem. 296, 126666.

Hancke, G.P., de Carvalho e Silva, B., Hancke Jr., G.P., 2013. The role of advanced sensing in smart cities. Sensors 13, 393–425.

Ikram, M., Liu, L., Liu, Y., Ma, L., Lv, H., Ullah, M., He, L., Wu, H., Wang, R., Shi, K., 2019. Fabrication and characterization of a high-surface area MoS_2@WS_2 heterojunction for the ultra-sensitive NO_2 detection at room temperature. J. Mater. Chem. A 7, 14602–14612.

Ikram, M., Lv, H., Liu, Z., Khan, M., Liu, L., Raziq, F., Bai, X., Ullah, M., Zhang, Y., Shi, K., 2020. Rational design of MoS_2/C_3N_4 hybrid aerogel with abundant exposed edges for highly sensitive NO_2 detection at room temperature. Chem. Mater. 32, 7215–7225.

Kim, Y., Kwon, K.C., Kang, S., Kim, C., Kim, T.H., Hong, S.-P., Park, S.Y., Suh, J.M., Choi, M.-J., Han, S., Jang, H.W., 2019. Two-dimensional NbS_2 gas sensors for selective and reversible NO_2 detection at room temperature. ACS Sens. 4, 2395–2402.

Kim, Y., Lee, S., Song, J.-G., Ko, K.Y., Woo, W.J., Lee, S.W., Park, M., Lee, H., Lee, Z., Choi, H., Kim, W.-H., Park, J., Kim, H., 2020. 2D transition metal dichalcogenide

heterostructures for p- and n-type photovoltaic self-powered gas sensor. Adv. Funct. Mater. 30, 2003360.
Kim, Y.H., Phan, D.T., Ahn, S., Nam, K.H., Park, C.M., Jeon, K.J., 2018. Two-dimensional SnS_2 materials as high-performance NO_2 sensors with fast response and high sensitivity. Sens. Actuators B 255, 616–621.
Ko, K.Y., Song, J.G., Kim, Y., Choi, T., Shin, S., Lee, C.W., Lee, K., Koo, J., Lee, H., Kim, J., Lee, T., Park, J., Kim, H., 2016. Improvement of gas-sensing performance of large-area tungsten disulfide nanosheets by surface functionalization. ACS Nano 10, 9287–9296.
Koo, W.-T., Cha, J.-H., Jung, J., Choi, S.-J., Jang, J.-S., Kim, D.-H., Kim, I.-D., 2018. Few-layered WS_2 nanoplates confined in Co, N-doped hollow carbon nanocages: abundant WS_2 edges for highly sensitive gas sensors. Adv. Funct. Mater. 28, 1802575.
Kumar, R., Liu, X., Zhang, J., Kumar, M., 2020. Room-temperature gas sensors under photoactivation: from metal oxides to 2D materials. Nano Micro Lett. 12, 164.
Li, X., Li, X., Li, Z., Wang, J., Zhang, J., 2017. WS_2 nanoflakes based selective ammonia sensors at room temperature. Sens. Actuators B 240, 273–277.
Li, Y., Song, Z., Li, Y., Chen, S., Li, S., Li, Y., Wang, H., Wang, Z., 2019. Hierarchical hollow MoS_2 microspheres as materials for conductometric NO_2 gas sensors. Sens. Actuators B 282, 259–267.
Liu, D., Hong, J., Li, X., Zhou, X., Jin, B., Cui, Q., Chen, J., Feng, Q., Xu, C., Zhai, T., Suenaga, K., Xu, H., 2020. Synthesis of $^{2}H–^{1}T'$ WS_2-ReS_2 heterophase structures with atomically sharp interface via hydrogen-triggered one-pot growth. Adv. Funct. Mater. 30, 1910169.
Liu, X., Ma, T., Pinna, N., Zhang, J., 2017. Two-dimensional nanostructured materials for gas sensing. Adv. Funct. Mater. 27, 1702168.
Ma, T., Liu, X., Sun, L., Xu, Y., Zheng, L., Zhang, J., 2019. MoS_2 nanosheets@N-carbon microtubes: a rational design of sheet on-tube architecture for enhanced lithium storage performances. Electrochim. Acta 293, 432–438.
Majhi, S.M., Mirzaei, A., Kim, H.W., Kim, S.S., Kim, T.W., 2021. Recent advances in energy-saving chemiresistive gas sensors: a review. Nano Energy 79, 105369.
Mayorga-Martinez, C.C., Ambrosi, A., Eng, A.Y.S., Sofer, Z., Pumera, M., 2015. Metallic 1T-WS_2 for selective impedimetric vapor sensing. Adv. Funct. Mater. 25, 5611–5616.
Ou, J.Z., Ge, W., Carey, B., Daeneke, T., Rotbart, A., Shan, W., Wang, Y., Fu, Z., Chrimes, A.F., Wlodarski, W., Russo, S.P., Li, Y.X., Kalantar-zadeh, K., 2015. Physisorption-based charge transfer in two-dimensional SnS_2 for selective and reversible NO_2 gas sensing. ACS Nano 9, 10313–10323.
Pham, T., Li, G., Bekyarova, E., Itkis, M.E., Mulchandani, A., 2019. MoS_2-based optoelectronic gas sensor with sub-parts-per-billion limit of NO_2 gas detection. ACS Nano 13, 3196–3205.
Reddy, D.A., Park, H., Ma, R., Kumar, D.P., Lim, M., Kim, T.K., 2017. Heterostructured WS_2-MoS_2 ultrathin nanosheets integrated on CdS nanorods to promote charge separation and migration and improve solar-driven photocatalytic hydrogen evolution. ChemSusChem 10, 1563–1570.
Shim, Y.-S., Kwon, K.C., Suh, J.M., Choi, K.S., Song, Y.G., Sohn, W., Choi, S., Hong, K., Jeon, J.-M., Hong, S.-P., Kim, S., Kim, S.Y., Kang, C.-Y., Jang, H.W., 2018. Synthesis of numerous edge sites in MoS_2 via SiO_2 nanorods platform for highly sensitive gas sensor. ACS Appl. Mater. Interfaces 10, 31594–31602.
Swan, M., 2012. Sensor mania! the internet of things, wearable computing, objective metrics, and the quantified self 2.0. J. Sens. Actuator Netw. 1, 217–253.
Tang, H., Li, Y., Sokolovskij, R., Sacco, L., Zheng, H., Ye, H., Yu, H., Fan, X., Tian, H., Ren, T.-L., Zhang, G., 2019. Ultra-high sensitive NO_2 gas sensor based on tunable polarity transport in CVD-WS_2/IGZO p-N heterojunction. ACS Appl. Mater. Interfaces 11, 40850–40859.
U.S.E.P. Agency, 2016. Air Trends Summary Report, ⟨https://www3.epa.gov/ttn/naaqs/standards/nox/s_nox_history.html#3⟩.
Walker, J.M., Akbar, S.A., Morris, P.A., 2019. Synergistic effects in gas sensing semiconducting oxide nano-heterostructures: a review. Sens. Actuators B 286, 624–640.
Wang, C., Yin, L., Zhang, L., Xiang, D., Gao, R., 2010. Metal oxide gas sensors: sensitivity and influencing factors. Sensors 10, 2088–2106.
Wang, D., Luo, F., Lu, M., Xie, X., Huang, L., Huang, W., 2019. Chemical vapor transport reactions for synthesizing layered materials and their 2D counterparts. Small 15, 1804404.
Wang, J., Fan, S., Xia, Y., Yang, C., Komarneni, S., 2020. Room-temperature gas sensors based on ZnO nanorod/Au hybrids: visible-light-modulated dual selectivity to NO_2 and NH_3. J. Hazard. Mater. 381, 120919.
Wang, X., Gu, D., Li, X., Lin, S., Zhao, S., Rumyantseva, M.N., Gaskov, A.M., 2019. Reduced graphene oxide hybridized with WS_2 nanoflakes based heterojunctions for selective ammonia sensors at room temperature. Sens. Actuators B 282, 290–299.
Wu, H., Chen, X., Qian, C., Yan, H., Yan, C., Xu, N., Piao, Y., Diao, G., Chen, M., 2020. Confinement growth of layered WS_2 in hollow beaded carbon nanofibers with synergistic anchoring effect to reinforce Li^+/Na^+ storage performance. Small 16, 2000695.
Xu, T., Liu, Y., Pei, Y., Chen, Y., Jiang, Z., Shi, Z., Xu, J., Wu, D., Tian, Y., Li, X., 2018. The ultra-high NO_2 response of ultra-thin WS_2 nanosheets synthesized by hydrothermal and calcination processes. Sens. Actuators B 259, 789–796.
Xu, Y., Zheng, W., Liu, X., Zhang, L., Zheng, L., Yang, C., Pinna, N., Zhang, J., 2020a. Platinum single atoms on tin oxide ultrathin films for extremely sensitive gas detection. Mater. Horiz. 7, 1519–1527.
Xu, Y., Zheng, L., Yang, C., Zheng, W., Liu, X., Zhang, J., 2020b. Oxygen vacancies enabled porous SnO_2 thin films for highly sensitive detection of triethylamine at room temperature. ACS Appl. Mater. Interfaces 12, 20704–20713.
Yuan, H., Aljneibi, S.A.A.A., Yuan, J., Wang, Y., Liu, H., Fang, J., Tang, C., Yan, X., Cai, H., Gu, Y., Pennycook, S.J., Tao, J., Zhao, D., 2019. ZnO nanosheets abundant in oxygen vacancies derived from metal-organic frameworks for ppb-level gas sensing. Adv. Mater. 31, 1807161.
Zeng, X., Ding, Z., Ma, C., Wu, L., Liu, J., Chen, L., Ivey, D.G., Wei, W., 2016. Hierarchical nanocomposite of hollow N-doped carbon spheres decorated with ultrathin WS_2 nanosheets for high-performance lithium-ion battery anode. ACS Appl. Mater. Interfaces 8, 18841–18848.
Zhang, J., Liu, X.H., Neri, G., Pinna, N., 2016. Nanostructured materials for room-temperature gas sensors. Adv. Mater. 28, 795–831.
Zhang, Y., Yao, Y., Sendeku, M.G., Yin, L., Zhan, X., Wang, F., Wang, Z., He, J., 2019. Recent progress in CVD growth of 2D transition metal dichalcogenides and related heterostructures. Adv. Mater. 31, 1901694.
Zheng, W., Xu, Y., Zheng, L., Yang, C., Pinna, N., Liu, X., Zhang, J., 2020. MoS_2 Van der Waals p–n junctions enabling highly selective room-temperature NO_2 sensor. Adv. Funct. Mater. 30, 2000435.
Zhou, J., Cheng, X.-F., Gao, B.-J., Yu, C., He, J.-H., Xu, Q.-F., Li, H., Li, N.-J., Chen, D.-Y., Lu, J.-M., 2019. Detection of NO_2 down to one ppb using ion-in-conjugation-inspired polymer. Small 15, 1803896.
Zhu, W., Low, T., Wang, H., Ye, P., Duan, X., 2019. Nanoscale electronic devices based on transition metal dichalcogenides. 2D Mater. 6, 032004.

Materials Research Bulletin 158 (2023) 112053

Contents lists available at ScienceDirect

Materials Research Bulletin

journal homepage: www.elsevier.com/locate/matresbu

Review

Semiconductor-metal-oxide-based nano-composites for humidity sensing applications

Ankit Kumar [a,b], Gaurav Gupta [a], Komal Bapna [a,b,*], D.D. Shivagan [a,b]

[a] Temperature and Humidity Metrology, CSIR- National Physical Laboratory, New Delhi 110012, India
[b] Academy of Scientific & Innovative Research, CSIR- National Physical Laboratory, Ghaziabad 201002, India

ARTICLE INFO

Keywords:
Humidity sensor
Semiconductor metal oxides
Nano-composites
Surface morphology
Relative humidity

ABSTRACT

Humidity sensing is of great significance in agriculture, pharmaceutical, medical, environmental, etc. Among various types of materials, semiconductor metal oxides (SMOs) are widely studied as humidity sensors due to their easy fabrication, chemical inertness, low-cost and portability. The review presents the humidity sensors based on various SMO nanostructures and their composites with the recent developments. Basic sensing characteristics of a humidity sensor, relative humidity generation methods, sensing mechanism and influence of material engineering are illustrated. The review elaborates the theoretical aspects of water adsorption and the experimental outcomes. It is revealed that there are still challenges to achieve humidity sensors with best of all sensing characteristics, to bring them into practical applications. The possibility of flexible SMO nano-composites for wearable devices in healthcare sector and artificial intelligence is discussed. The review is concluded with the gap and ideas for future developments of these SMO nano-composites for dynamic humidity sensing applications.

1. Introduction

The physical, chemical, mechanical and biological properties of all living and non-living species (materials) are affected by even a trace amount of water present in the atmosphere. Measurement of this small amount of water is of utmost importance and sometimes very critical in many areas such as health and safety, product quality etc. The amount of water content in a solid and liquid component is called “moisture”, whereas the amount of water vapours present in gas is referred to as “humidity”. The term “humidity” can be expressed as the amount of water vapour present in the air at a given temperature. Measurement of humidity of gas is termed as hygrometry. The humidity measurement results can be expressed in different units, such as absolute humidity (AH), relative humidity (%RH), dew and frost Point (DP & FP) and parts per million (ppm), depending upon the measurement methods [1]. Absolute humidity is applicable for primary measurements, which require a direct measure of the amount of water vapour in the air. Absolute humidity is referred to as the ratio of the mass of water vapour to the unit volume of air at a given temperature and pressure and is expressed as:

$$AH = \frac{Amount\ of\ water\ vapour\ (g)}{Volume\ of\ air(m^3)} \tag{1}$$

In contrast, relative humidity is referred to as the ratio of water content present in the given volume to the water needed for saturation for the same volume at a constant temperature and is expressed as:

$$\%RH = \frac{Amount\ of\ water\ vapour\ present\ in\ given\ volume}{Amount\ of\ Water\ vapour\ required\ for\ saturation} \times 100 \tag{2}$$

Since, at a given temperature, air cannot hold more than a definite amount of water in the form of vapour, the state is defined as a saturated state. The saturated state varies with temperature and pressure. Hence, relative humidity shows dependency on temperature and pressure. Vapour pressure exerted at saturation is defined as saturation vapour pressure. At a constant temperature, relative humidity can be expressed as the ratio of vapour pressure present to the saturated vapour pressure of air and defined as:

$$\%RH = \frac{Actual\ vapour\ Pressure\ (bar\ or\ kPa)}{Saturated\ vapour\ Pressure\ (bar\ or\ kPa)} x\ 100 \tag{3}$$

The water vapour molecule starts condensing to liquid or ice in the condition when vapour pressure becomes equal to the saturation vapour pressure, and it depends on the temperature of the environment. The Dew point is the temperature (above 0°C) at which water vapour starts condensing into liquid water. If the temperature is below 0°C, on further

* Corresponding author at: Temperature and Humidity Metrology, CSIR- National Physical Laboratory, New Delhi 110012, India.
E-mail address: komal.bapna@nplindia.org (K. Bapna).

https://doi.org/10.1016/j.materresbull.2022.112053
Received 3 June 2022; Received in revised form 7 September 2022; Accepted 30 September 2022
Available online 3 October 2022
0025-5408/© 2022 Elsevier Ltd. All rights reserved.

lowering the temperature, the water condenses and forms frost. Thus, the frost point is referred to the temperature at which the frost begins to form. Dew and frost point measurements are independent of pressure and are used to determine very low humidity levels.

Humidity plays a significant role in agriculture, pharmaceutical, chemical, textile, food and health industries, scientific research, environmental climates, etc. [2–7]. In medical applications, humidity sensors have been utilized to monitor human breathing for the detection of respiratory disorders [8–10]. In agricultural sectors, humidity sensors are used to monitor crop growth. In the food industry, it is necessary to monitor the humidity level. Fruits and vegetables must be kept in a specified humid condition to keep them fresh for a long time. Humidity measurement is also important for human comfort [11]. Humidity sensing studies have been progressing rapidly in industrial and household applications. Continuous monitoring of humidity with high accuracy and precision is not only a difficult but also a complex task. %RH is the most used parameter in the measurement of humidity because it can be measured with simpler and cost-effective methods. Hygrometers/Humidity sensors are the devices that convert amounts of water vapours in some measuring parameters like resistance [12,13], capacitance [14–16], impedance [17,18], quartz crystal microbalance (QCM) [19–21], optical properties [22,23], etc. Key parameters for an excellent humidity sensor are high sensitivity towards water molecules, high accuracy, long-term repeatability, chemically inertness, fast response and recovery time, low drift, low hysteresis, work on a wide range of temperature and humidity and cost-effectiveness. A summary of the parameters of humidity sensors is given in Table 1.

Widely studied materials as humidity sensors are ceramics such as Al_2O_3, SiO_2, perovskites (ABO_3), spinel (AB_2O_4), SMOs such as ZnO, SnO_2, TiO_2, CuO, WO_3, etc., polymers such as Polyvinylpyrrolidone (PVP), poly vinyl alcohol (PVA), poly vinyl aniline (PANI) etc., carbon materials such as carbon nanotubes (CNTs), graphene, carbon fibres, among others. The classification of humidity sensors based on different materials and their advantages and disadvantages is illustrated in Section 3.2. On the basis of a literature survey on various materials, it appears that SMOs are promising to fulfil the maximum criteria for excellent humidity sensors. These are earth-abundant, environment friendly and chemically inert. The advantages of SMOs which attracted the researchers to be utilized for humidity sensors include their ease of fabrication, high humidity sensing response over a wide range of temperatures, chemically inert, low cost and portability [25]. Humidity sensors based on SMOs nanostructures have been widely studied in the past few years because of their brilliant performance. Recent development toward miniaturization based on these compounds has several advantages, including enhancement of accuracy, reliability and cost-effectiveness. Nanosized materials possess unique physical, chemical and mechanical properties in comparison with bulk due to their large surface-to-volume ratio [26]. In general, the surface-to-volume ratio of nanomaterials increases with a decrease in nano-particle size. SMO nanostructures are easy to synthesize, environmentally friendly, earth bounteous and low-cost [27]. The sensing properties depend on surface morphology, presence of defects, doping elements, etc. Different techniques have been used for the fabrication of these materials to change their physical morphology, which directly correlates to the sensor's performance.

Table 1
Summary of parameters of humidity sensors.

Parameters	Definition
Sensitivity	The ratio of the sensor output obtained against change in unit %RH
Accuracy	Closeness to the standard %RH value.
Response/Recovery time	Time required by the sensor to reach 90% of the actual value.
Drift	Variation in output value with same input with respect to time.
Hysteresis	The difference in an output signal with respect to the input signal when measured in increasing and decreasing order, respectively.
Repeatability	The closeness of output values with the same repeated input values under the same conditions of measurements.
Temperature dependence	The sensor output is a function of temperature at a constant humidity value.

In the present paper, we have comprehensively discussed the background of humidity sensing, the various sensing methods and their working, the classification of humidity sensors and a detailed study on SMO humidity sensors with the current research status in this area. The present review is segmented into eight sections. After the introduction, in Section 2, we have defined various methods to generate relative humidity in real practice. Section 3 details the classification of the humidity sensors based on two different approaches. Section 4 emphasizes the SMOs - based humidity sensors. The advantages, limitations and modifications to improve the humidity sensing properties of SMOs based nanostructures have been discussed in detail. In Section 5, we have addressed the water vapour interaction with SMO surfaces to comprehend the humidity sensing phenomenon. Sections 6–9 describes the humidity sensing characteristics of different SMOs based nanocomposites. Change in the sensing behavior with the alteration of surface morphology of SMOs by different methods has been described comprehensively. It also includes the theoretical background behind the water sorption mechanism for different SMOs. The overview of SMOs based sensors and their advantages/disadvantages is described in Section 10. In Section 11, the possibility of SMOs being utilized in the healthcare sector has evolved. Section 12 concludes and discusses the present gap and future prospects of these SMO-based nanostructures as humidity sensors.

2. Methods of generation of relative humidity

The performance of the humidity sensor has to be verified according to their application before utilizing them. Calibration of the sensor with standard relative humidity values assures the correct measurement. The quality of the humidity sensor defines by its performance characteristics like sensitivity, response/recovery time, drift, hysteresis, reproducibility, etc. The known humidity generation mechanism that remains stable with time at a defined temperature is the most important part of the standardization/calibration of the humidity sensor. Different methods to generate the relative humidity are discussed below:

2.1 Saturated salt solution method.
2.2 Two-Pressure (2P) humidity generation method.
2.3 Two-Temperature (2T) humidity generation method.
2.4 Two-Pressure Two-Temperature (2P-2T) humidity generation method.
2.5 Mix flow method.

2.1. Saturated salt solution method

It is a very economical and simplest method for relative humidity generation. A saturated salt solution generates specific and predictable relative humidity in a closed container. When a saturated salt solution is placed in a closed container, two processes occur simultaneously; the dissolution of undissolved salts and the evaporation of water from the solution. At a particular point, the rate of both the processes becomes equal and attains an equilibrium, and the system attains a specific relative humidity which remains constant with time provided that the temperature is constant. Different salt solutions have been used to generate different relative humidity environments and act as relative humidity "fixed points" [28]. The equilibrium %RH values obtained for various salts at room temperature are shown in Fig. 1.

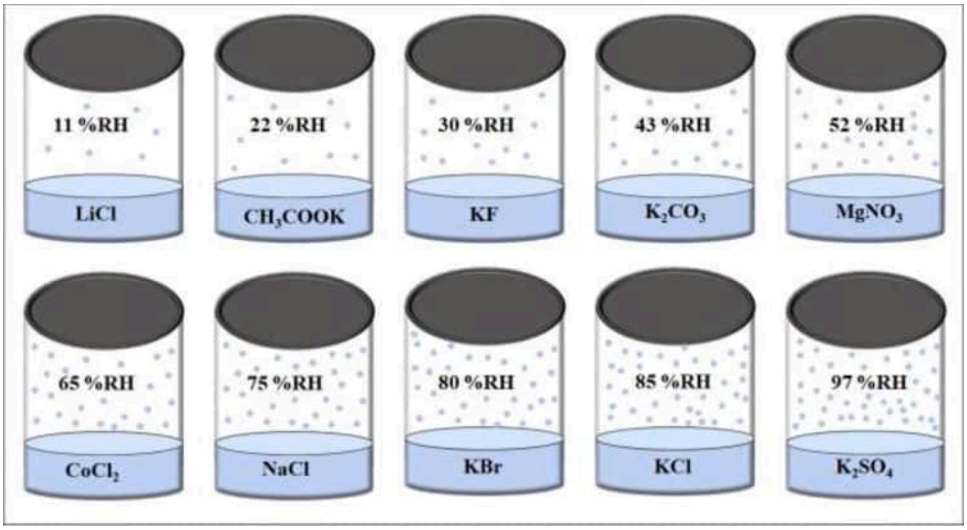

Fig. 1. Saturated salt solution generating different %RH environment in a closed container.

2.2. Two-pressure (2P) humidity generation method

Since %RH depends on the temperature and pressure, by keeping the temperature constant, %RH can be varied with a change in the pressure. The two-pressure method is the most commonly used method for the realization of relative humidity in national metrological institutes of various countries as a primary standard [29,30]. This method consists of two chambers, a saturated and a test chamber, both having a constant temperature. The saturated chamber consists of high pressure (P_S) with a relative humidity of 100%, while the test chamber consists of low pressure (P_T), usually atmospheric pressure. Both chambers are connected with an expansion valve that controls the flow of saturated air. Saturated air flows from the saturated chamber to the test chamber through the expansion valve, where the gas is isothermally expanded at low pressure in the test chamber. The desired humidity is achieved in the test chamber, which is equal to the ratio of pressure between the test and saturated chamber and is as given:

$$\%RH = \frac{P_T}{P_s} x\ 100 \tag{4}$$

The schematic diagram of the working principle of the two-pressure method is shown in Fig. 2.

2.3. Two-temperature (2T) humidity generation method

The two-temperature method is also a primary method to generate desired humidity environment. It also consists of two chambers: a saturated chamber and a test chamber, both consist of constant pressure, but the saturated chamber has a relatively low temperature compared to the test chamber. The saturated air is passed into the test chamber, having a relatively high temperature. As the mixing ratio is constant, thus relative humidity decreases with an increase in the temperature. By comparison of the saturation vapour pressure at various temperatures and the temperature of two chambers, any desired relative humidity can be achieved. The schematic diagram of the working principle of the two-temperature humidity generator is shown in Fig. 3.

2.4. Two-pressure two-temperature (2P-2T) humidity generation method

This primary method combines the two-pressure principle with the two-temperature principle to form a hybrid system which exhibits the benefits of both system architectures [31]. Here we allow the difference in the temperature of both the saturated and test chamber and can be changed independently. This method requires measurement of temperature as well as the pressure of both saturated and test chamber to obtain resultant relative humidity environment in a test chamber. By allowing the temperature difference, a wider range of relative humidity can be generated with less pressure range of saturated chamber.

2.5. Mix gas flow method

In this method, a mixed stream of dry and saturated air is passed in a definite proportion into the test chamber to generate the desired relative humidity environment. The relative humidity is then generated in the

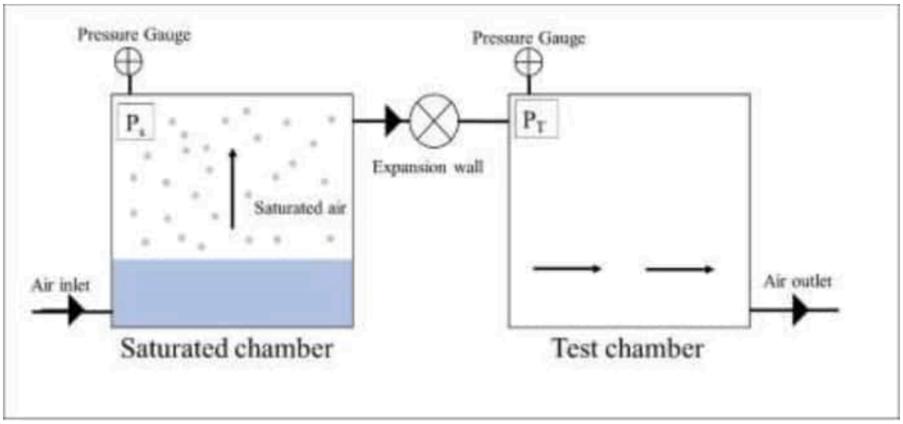

Fig. 2. Schematic diagram of two-pressure humidity generator.

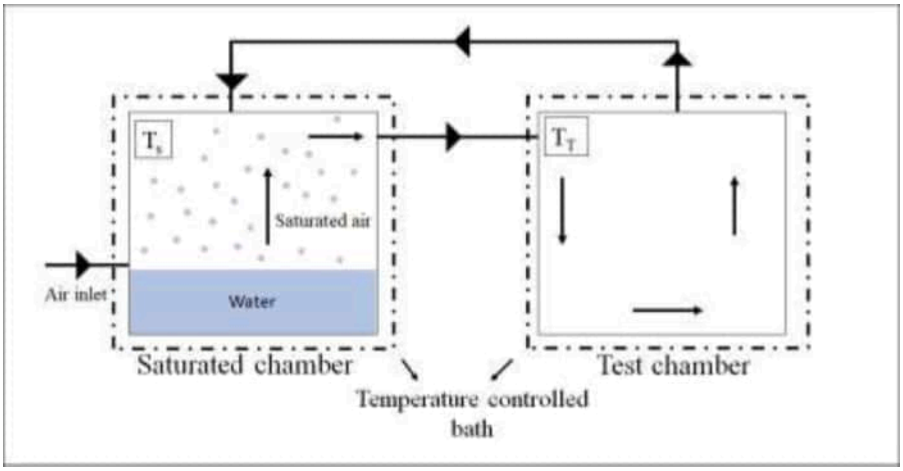

Fig. 3. Schematics of two-temperature humidity generator.

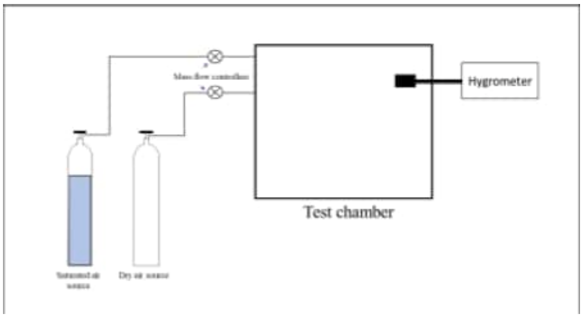

Fig. 4. Schematics of mix flow method.

test chamber from the known flow rates of the two streams of gas which is given by the ratio of the flow parameters. This method is rather simple and widely used in metrological laboratories for calibration purposes. The schematic diagram is shown in Fig. 4.

3. Classification of humidity sensors

Principally, when water molecules of the surrounding gas get adsorbed on the active material surface, it causes some change in measurable physical and chemical properties of the sensing material, which alters corresponding to the relative humidity change. Humidity sensors are classified into two categories based on:

- Sensing parameters.
- Sensing materials.

The classification of humidity sensors based on sensing parameters is shown in Fig. 5.

3.1. *Classification of humidity sensors based on sensing parameters*

3.1.1. *Gravimetric hygrometers*

The determination of the mass of a given material is called gravimetry. The gravimetric method, being a direct method, provides the most precise and accurate measurement of water vapour in hygrometry. Therefore, it is often used as a primary standard against which other methods and instruments are compared and calibrated. This method involves removing all the water from a sample and weighing it to determine the density and volume of the dry sample. The first gravimetric hygrometer at National Institute for Standard and Technology (NIST) was developed in 1948 by Wexler [32]. Based on the

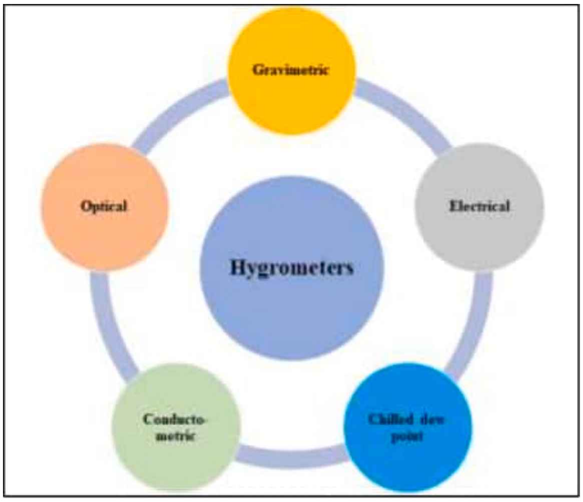

Fig. 5. Classification of humidity sensors based on the change in the physical properties with change in the humidity.

construction, gravimetric sensors can be further divided into different categories. In gravimetric hygrometry, the mass-sensitive device transforms the mass change at a specifically modified surface into a measurable parameter which includes quartz crystal microbalance (QCM), surface acoustic wave (SAW), etc.

Quartz crystal microbalance (QCM) based humidity sensors. The QCM method has been extensively used to analyze the properties related to mass change on any surface. Due to its advantage of ultra-high sensitivity towards water molecules adsorbed on sensing film deposited on the crystal surface, it has attracted researchers working on humidity sensors [33]. A typical QCM humidity sensor is made of a mass-sensitive material film deposited on an electrode-integrated quartz crystal resonator. When there is a variation in the amount of water molecule, during adsorption or desorption, deposited on the sensing material, the composite resonator shows an oscillating frequency shift, revealing a change in humidity. The shift in the resonance frequency (Δf) can be calculated in terms of the change in mass of the QCM, as given by the Sauerbrey equation [34]:

$$\Delta f = -\left(\frac{2f_0^2}{A\sqrt{\rho_q \mu_q}}\right)\Delta \mathrm{m} \tag{5}$$

where f_0 is the fundamental resonance frequency of the QCM (when no

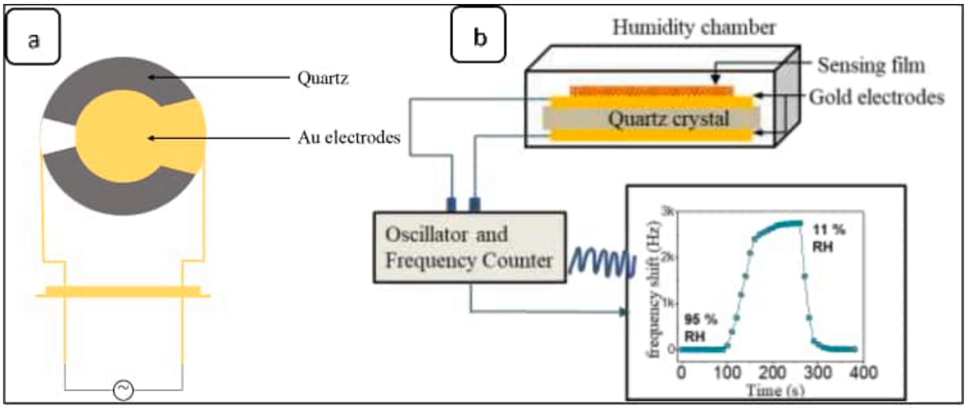

Fig. 6. (a) Schematic diagram of quartz crystal and (b) QCM device.

water molecules were adsorbed), ρ_q is quartz crystal density, μ_q is shear modulus, and A is the area of the electrode defined on the QCM. In the past few years, researchers have been depositing different new humidity sensing materials with QCM to study their humidity sensing behavior [35–39]. During the adsorption process, the water molecules deposited on the surface increase the mass of the crystal. As a result, the oscillating frequency of the QCM increases sharply. Also, during the desorption process, the QCM can return to its initial resonance frequency. The schematic diagram of quartz crystal and QCM device presenting the humidity sensing response are shown in Fig. 6(a and b), respectively.

Surface acoustic wave (SAW) humidity sensors. The device which detects the change in humidity as a result of variation in the surface acoustic wave comes under SAW humidity sensors. Acoustic wave velocity is altered by changes in the physical or chemical properties of the sensor surface, including mass, conductance, viscoelasticity, etc. The SAW is transformed into a measurable electric signal by the piezoelectric effect in the output-input integrated transducer as a result of any change in wave corresponding to the change in output electric signal. SAW humidity sensors have piezoelectric materials as the acoustic layer, which shows the change in SAW corresponding to the change in relative humidity. Polymers [40–42], GO [43], CNTs [44], SMOs [45], among others, are some of the communal materials used in SAW humidity sensors. The pictorial diagram of the working principle of a SAW humidity sensor is shown in Fig. 7. Fig. 7(a) shows a SAW device where a delay line, which consists of humidity sensitive layer, lies in between Interdigitated electrodes (IDEs) deposited on a piezoelectric substrate. The signal is applied to input IDTs through a transducer, which generates the surface wave. After the propagation through the humidity sensing layer, this surface wave is converted into an electrical signal at output IDTs and thus analyzed for different humidity environments. The propagation of SAW through a humidity sensing layer is shown in Fig. 7 (b). The change in frequency of a typical SAW humidity sensor with relative humidity is shown in Fig. 7(c).

3.1.2. Electrical hygrometers

The hygrometers, which show the change in the electrical properties of the materials concerning the change in relative humidity, come under electrical hygrometers. An electric hygrometer may be defined as an instrument used to determine the water content of air by the measurement of electrical properties like resistance, impedance, or capacitance of the hygroscopic materials with a change in humidity. Electrical hygrometers generally constitute a substrate and conducting IDEs, at which humidity-sensitive material thin film is being deposited. The common schematic diagram for resistive (R), impedance (Z) and capacitance (C) types of electrical humidity sensors is shown in Fig. 8.

Resistive humidity sensors. These sensors show the change in resistance when come in contact with water molecules. Resistive humidity sensors have many advantages such as being easy to fabricate, low power consumption, simplicity in operation, ease of miniaturization, and cost-effectiveness and hence widely studied. When a water molecule gets adsorbed on the sensor surface, the water molecule gets ionized in the presence of an electric field, generating a large number of charge carriers, hydronium ion (H_3O^+). This, in turn, causes a change in resistance due to the mobility of protons generated by the dissociation of water molecules adsorbed on the surface. Hence, change in relative humidity can be evaluated by measuring the resistance change. Typically, the change in resistance varies from 1kΩ to 100 MΩ.

Capacitive humidity sensors. These sensors show the change in capacitance when water molecules get adsorbed on the sensing material. The variation in capacitance is due to the change in the dielectric constant of the sensing material. The adsorbed water molecule, being polar, strengthens the polarization effect and increases the dielectric constant of the sensing film. Generally, the change in capacitance is in the order of 0.2–0.5 pF per unit %RH change at room temperature. Similar to resistive humidity sensors, capacitive sensors possess a simple structure with high reliability, low fabrication cost, and better selectivity [14,46].

Impedance humidity sensors. Impedance humidity sensors work based on the change in impedance when water molecules get adsorbed on the sensing film corresponding to a change in the relative humidity. These types of sensors are good candidates for detecting relatively low humidity changes. A sinusoidal voltage is applied at a particular frequency, and the resultant impedance change is calculated by measuring the current. Working with these impedance humidity sensors requires high-performance impedance spectroscopy, which lacks their general use and makes them a bit expensive [47].

3.1.3. Coulometric hygrometers

A Coulometric hygrometer consists of an electrolytic cell in which a conducting film is electrolyzed continuously by an applied direct current voltage, and the cell current is measured as a function of the water vapour content. The first time, a coulometric hygrometer was described by Keidel in 1959 to determine the water content in the air [48]. In a coulometric hygrometer, electrolysis of adsorbed water molecules takes place, which decomposes to H_2 and O_2. Generally, a strong desiccant material, tetraphosphorous decaoxide (P_4O_{10}), is coated on the metal electrodes to enhance the adsorption of water molecules. When electrical voltage is applied to the electrode, the water vapour adsorbed on P_4O_{10} is dissociated into H_2 and O_2, generating a finite current. This reaction requires a minimum 2V DC for dissociation of water molecules,

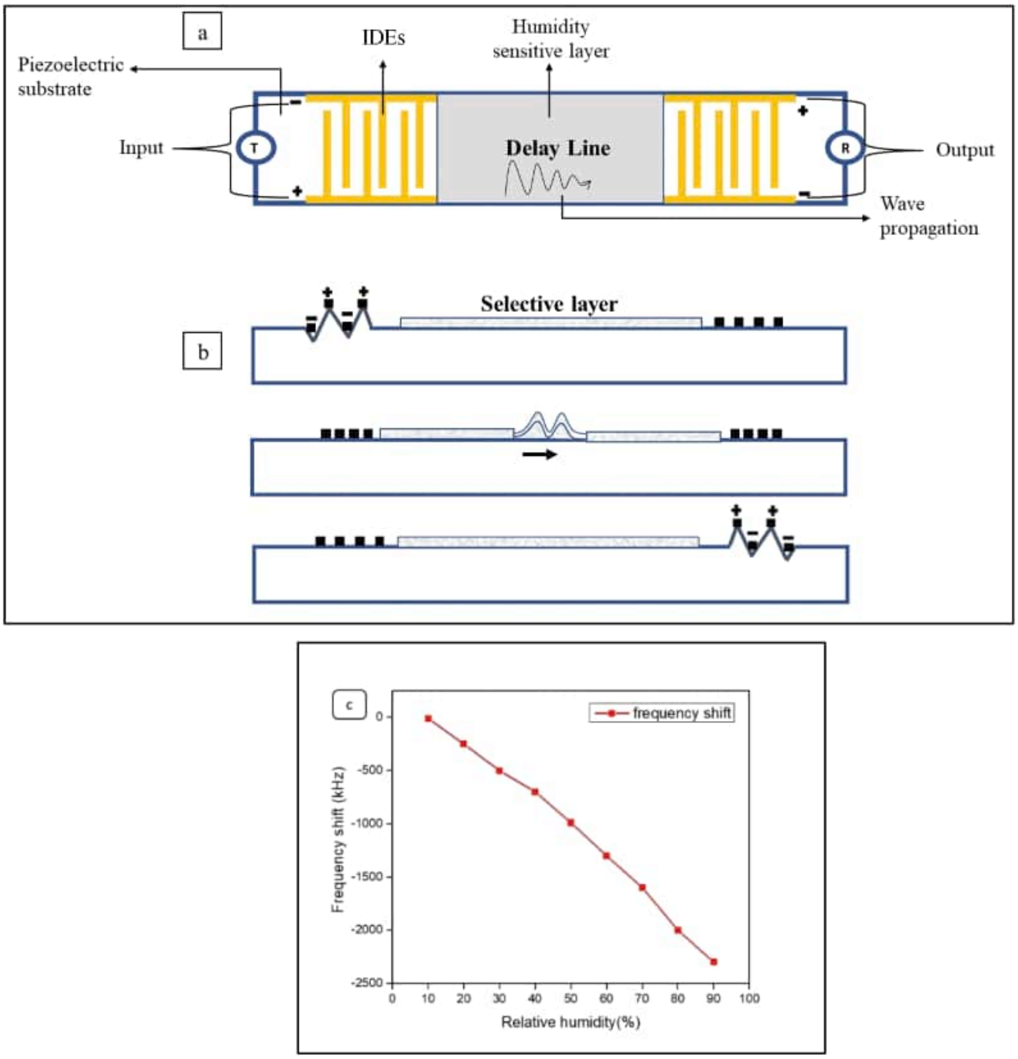

Fig. 7. (a) Schematic diagram of the working principle of a SAW humidity sensor, (b) the propagation of SAW over the surface of piezoelectric substrate and (c) the change in frequency with relative humidity.

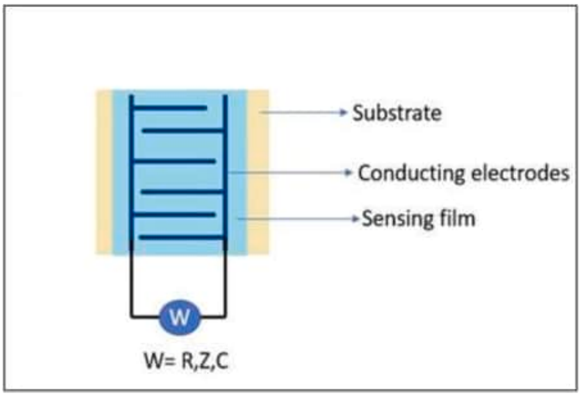

Fig. 8. Schematic of humidity sensing mechanism of resistance (R), impedance (Z) and capacitance (C) based humidity sensors.

and the increase in current with an increase in the number of water molecules corresponds to an increase in relative humidity. A pictorial diagram to illustrate the working principle of the coulometric hygrometer is shown in Fig. 9.

3.1.4. *Optical hygrometers*

The optical properties of the fibre are another important tool for measuring humidity change. Optical fibre humidity sensors show changes in the optical properties of incident light, such as transmittance, dielectric properties and refractive index caused by the adsorption of water molecules when passed through the optical fibre coated with humidity sensing material. A schematic diagram of the basic working principle of an optical fibre-based humidity sensor is shown in Fig. 10. Optical fibres have several advantages, such as immunity to electromagnetic interference, high thermal stability, chemical inertness, low attenuation, etc. [22]. Also, their small size and simple geometry make them lightweight systems [11]. Despite this, the high cost of optical instruments limits the commercial use of optical fibre-based sensors. David Gomez et al. have reported polymeric optical fibre coated with a hydrophilic film of poly(allylamine hydrochloride) and SiO_2

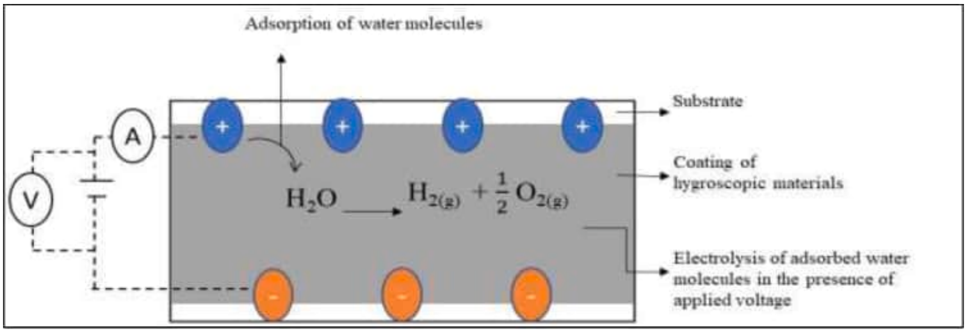

Fig. 9. Pictorial diagram illustrating the working principle of coulometric hygrometers.

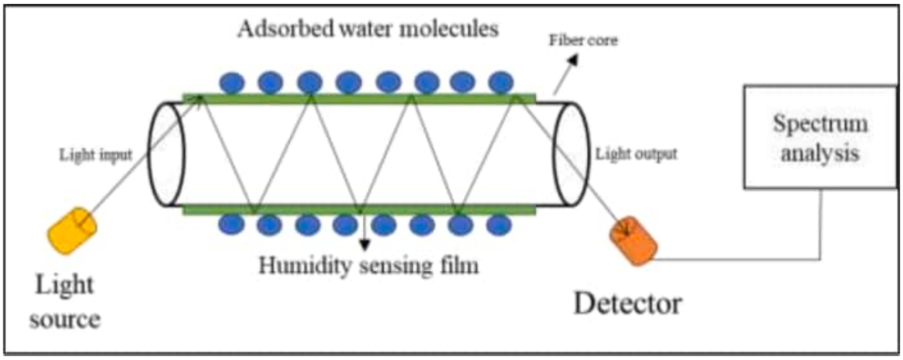

Fig. 10. Schematic diagram of working principle of optical hygrometers.

mesoporous nanoparticles [49]. The sensor shows the reduction in light transmission with the increase in the relative humidity as the refractive index of coated film onto optical fibre changes with a response time of 1.5 s. Due to the very low response time, the given sensor was also used for breath monitoring.

3.1.5. *Chilled mirror dew point hygrometer*

Chilled mirror-based dew-point sensors work on the principle of the condensation method [50]. If a surface exposed to air at a particular % RH less than 100% is cooled, a temperature will be reached at which the relative humidity of the air close to the surface is 100%, and thereafter dew will be deposited on the cooled surface. In this device, the sample air is drawn to the metallic mirror surface through piping. As the mirror cools, condensation forms when its surface temperature falls below the dew point temperature. The temperature in the metallic mirror is measured using a platinum resistance thermometer (PRT) placed at the back of the mirror, and the result is taken as dew-point temperature. In the chilled-mirror technique, the mirror plays an important role which is built from silver and copper due to good thermal conductivity and coated with an inert metal such as gold, rubidium, etc. to prevent the mirror from stain and oxidation. At a particular temperature, as water vapours condense on the mirror, the reduction of the reflected light, due to the light-scattering effect of droplets, is detected by the photodetector, and the dew point is calculated. The schematic diagram of the working principle of the chilled mirror-based dew-point sensor is shown in Fig. 11. The instrument consists of an air inlet which is surrounded by an electric heater that prevents prior condensation of the air sample. The air samples are passed through the mirror, where temperature can be controlled. The mirror is placed with PRT for accurate measurement of dew point temperature.

3.2. *Classifications of humidity sensors based on materials used*

Numerous water-sensitive materials have been used as humidity sensors which include ceramics, such as SiO_2, Al_2O_3, semiconductors such as ZnO, TiO_2, SnO_2, CuO and NiO, perovskites such as $BaMO_3$ (M = Ti, Hf, Sn, Ce, Nb, Zr, etc.), $MnTiO_3$ [51], $SrCeO_3$ [52], $SrMnO_3$ [53], $GdAlO_3$ [54] etc, spinels such as $MgCr_2O_4$ [55], $MgAl_2O_4$ [56], $ZnCr_2O_4$ [57], $ZnAl_2O_4$ [58], $CoFe_2O_4$ [59], $MgFe_2O_4$ [60,61], etc, organic polymers such as PVP [41,62], PVA [63,64], PDA [37,65], PANI [66,

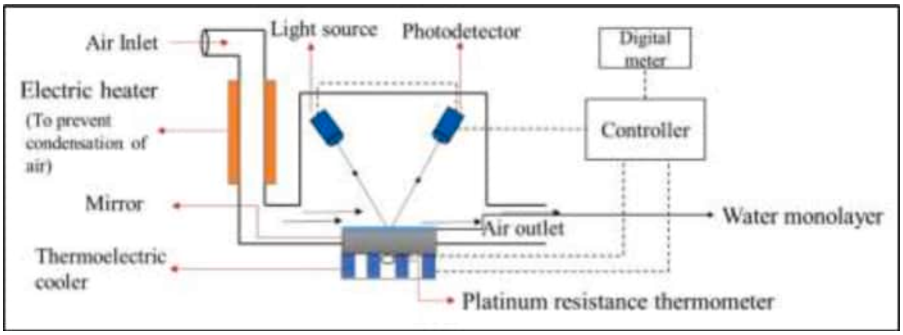

Fig. 11. Schematic diagram of chilled mirror dew point hygrometer.

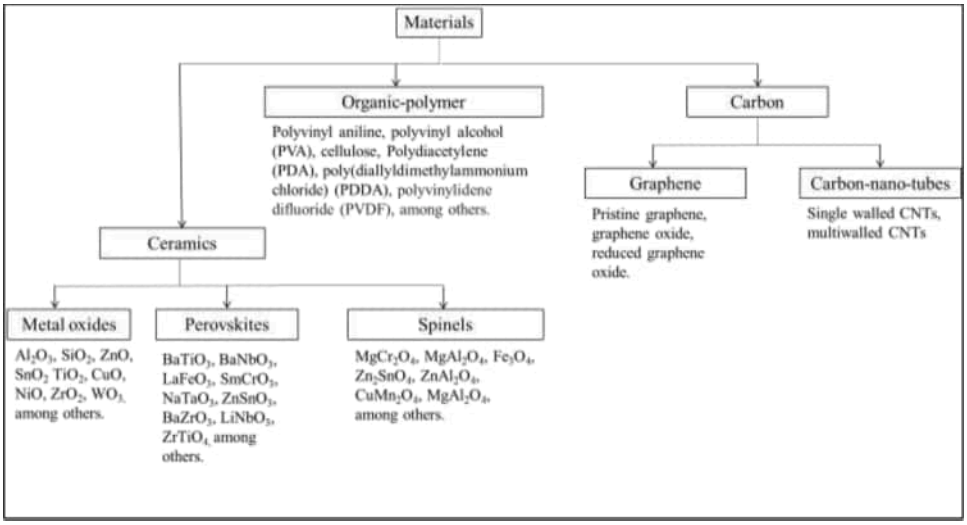

Fig. 12. Classification of various humidity sensing materials.

67], PDDA [68], PVDF [69] etc., carbon material such as porous carbon, CNTs and graphene derivatives like graphene oxide and reduced graphene oxide [70–74]. Classification of humidity sensors based on different materials used in the sensors is shown in Fig. 12.

The humidity sensing properties of perovskite oxides (ABO_3) are attributed to the presence of surface defects with trapped electrons, such as ionized oxygen vacancies. The electron trapped by chemisorbed oxygen ions is retransferred upon exposure to water molecules because of the displacement of oxygen ion by hydroxyl ions ($O^{2-} + H^+ \rightarrow OH^- + e^-$) and are restored to the conduction band resulting in increased conductivity [75]. The stability of perovskites allows the partial substitution of other ions at both A- and B-sites without changing the basic perovskite structure. Such substitution results in oxygen vacancies, variable B-site cation valences and crystalline structure defects, which directly or indirectly affect their performance in sensing applications [76]. Some of the limitations of perovskites are high sintering temperature, post calcination, and the need of sophisticated equipment for synthesis, which certainly increases the production cost, elongated preparation processes, and is eco-unfriendly [77]. Perovskites have not been popular as humidity sensors due to these various limitations.

The humidity sensors based on polymers have several advantages, such as lightweight, cost-effective, flexible, easy to fabricate and possessing good mechanical stability [2]. Polymers comprise repeated polar functional groups like fluoride, hydroxy, amino, or carbonyl, which attract the water molecules in the environment through hydrogen bonding. Since hydrogen bonding is weak so there is an equilibrium between adsorption and desorption, which shifts according to the water molecules present in the environment that corresponds to humidity change. But polymers show a lack of conductivity and large hysteresis due to the adsorption of water cluster inside bulk polymer, which results in distorting sensing polymer. This results in decreasing the lifetime of the sensor. Furthermore, the response/ recovery time for many polymers was shown long, even in the order of minutes which limits their use. Moreover, the polymer-based sensors are not suitable for high temperature, and high humidity working environments and are prone to deterioration when exposed to some solvents or electrical shocks.

Humidity sensors based on Carbon materials have attracted researchers due to their rich surface chemistry, high thermal and electrical conductivity and structure design ability. Carbon nanomaterials like CNTs [78], graphene [79], carbon black [80] and carbon nanofibers [66] possess high surface-to-volume ratio, chemical inertness, and one of the most important advantages is their flexibility which helps in making flexible sensors used in many applications [81]. Despite these many advantages, carbon nanomaterials show low selectivity towards water molecules due to the hydrophobic nature of carbon. Additionally, poor reproducibility, surface contamination and long-term drift limit their use. Also, environmental pollution and explosion hazards are big disadvantages of these materials, and the functionalization and oxidation process of CNTs and graphene takes a long time.

Spinel-type AB_2O_4 compounds have also been studied for applications in humidity/gas sensors, as they exhibit high porosity, large surface/volume ratio, low price and chemically stable structure. Spinel have a number of water adsorption sites in the form of multi-cation sites. Hence, in spinel, the reactivity of the surface mainly depends on the cation distribution along with the morphology and nanostructures, which are hugely governed by the synthesis technique, thermal treatment and doping. Similar to the perovskites, along with Grotthus mechanism, previously adsorbed and ionized oxygen species (O^- and O^{2-}) on the sensor surface hugely affect the humidity sensing mechanism [82]. However, the high values of electrical resistivity of these materials make them difficult to be used in practical applications [83].

After having a detailed discussion on the properties and challenges associated with different materials, we now overview the progress and advances in the work carried out on SMO-based humidity sensors. As discussed in the introduction section, SMOs show remarkable advantages such as high mechanical strength, physical and thermal stability and chemical inertness, low cost, environmentally friendly etc. When compared to other classes of materials and hence attracted the researchers to be utilized them for humidity sensing applications.

4. Semiconductor-metal-oxides as humidity sensor

Nanosized SMOs have attracted researchers for many years due to their significant electrical and optical properties, which are highly useful in fabricating nanoscale optoelectronic and electronic devices with multifunctionality [84]. Metal oxides are ionic compounds having positively charged metal ions and negatively charged oxygen ions and can behave as conductors, semiconductors and insulators. SMOs are classified as n-type, in which electrons are responsible for conductions, and p-type, in which holes are responsible for conduction. Exceptional intrinsic properties of SMOs like high surface-to-volume ratio, rich surface physics and high adsorption/desorption activity attracted the researchers to be chosen for integration in sensors [27]. The unique structure of SMOs comprising grain boundaries, porous structure, and rich surface areas makes them a suitable candidate for electrochemical sensor application. SMOs are the most commonly used materials among others for humidity sensor fabrication. In addition, 2D SMOs nanomaterials, including nanosheets, nanoplates, and nanowalls, among

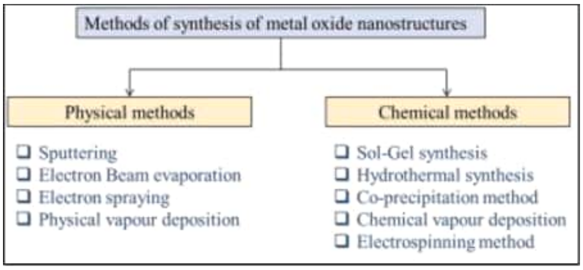

Fig. 13. Different methods of synthesis of nanostructures of metal oxides.

others, have novel properties like large surface-to-volume ratio, enhanced sensitivity, low power consumption and high mechanical durability, which attracted the attention of researchers [85]. Humidity sensors based on SMOs such as ZnO, TiO_2, SnO_2, CuO and NiO are commonly studied as humidity sensors due to their ease of fabrication, huge sensing response, low cost and good compatibility with microelectronic processes. These SMOs have revealed excellent humidity sensing properties due to the possibility to further tailor their band gap by defect engineering. Furthermore, sensing characteristics can be improved by modifying their surface morphology, particle size variation and composite formation with other sensing materials.

The physical, chemical and electronic properties of SMOs can be changed by modification in their structure, particle size, stoichiometry and doping [86]. The metal oxides can be synthesized by two methods; physical and chemical methods [87]. The physical method involves a top to down strategy in which bulk material is depleted systematically for the generation of nanoparticles. During the process, no chemical reaction takes place. While in the chemical method, the bottom-up approach has been applied in which the atoms or molecules react and combine to form the nanoparticles. The properties of nanoparticles like size, structure and composition can be controlled and varied by the synthesis method and the experimental conditions, which can be useful in various applications like sensing devices, catalysis, electronic devices, etc. The different methods of synthesis of metal oxide nanostructures are shown in Fig. 13.

5. Humidity sensing mechanism on SMOs surface

A lot of research has been done in past years on the water vapour interaction with SMOs surfaces. Gorden E. Brown Jr. et al. reported a review article where they studied the role of geometrical and electronic structure and defects reactivity towards the water molecules [88]. Based on experimental observations performed on the atomically clean substrate, the nature of reactive sites has been studied. Water adsorption on the SMOs surface is a donor-acceptor type interaction with no change in the valence state of water molecules and a slight change in the electronic state of the SMOs surface. This forms the basis of the mechanism of humidity sensing phenomena on SMOs. Adsorption of water molecules, when interacting with the surface of SMOs, takes place in successive layers where the adsorption phenomena differ from layer to layer. The first layer is formed by the dissociative adsorption of water molecules on the surface-active sites in the form of hydroxyl group (OH^-). The OH^- group of each water molecule is chemically adsorbed on the cationic species present on the surface. Hence, at low humidity, there is chemisorption of water molecules in hydroxyl form at SMOs surfaces [89], which is highly intact and immune to any change in the humid environment. After chemisorption, successive water vapours get physically adsorbed to the first formed chemisorbed hydroxyl layer by strong hydrogen bonding. With a further increase in humidity, the stack grows from monolayer to multilayer. After the first physisorbed layer, other layers are less ordered due to weak hydrogen bonding, which makes the water molecules more mobile to form continuous dipoles through the surface. This in turn results in an increase in dielectric constant as well as bulk conductivity.

The electronic conductivity in the chemisorbed layer is mainly due to the free electrons, which act as the charge carriers and proton immigration as a result of hydrogen hopping between adjacent OH^-. Therefore, the electronic conductivity decreases or increases depending on the p- or n-type nature of semiconductors [1]. The electronic conduction, as well as proton conduction, is possible at low humidity levels where the only chemisorbed layer of water molecule is present. For proton conductance, OH^- groups might dissociate to provide mobile H^+ through tunnelling. In the physisorbed layer, conductance is due to the Grotthus mechanism. The physisorbed water molecules dissociate in H^+ and OH^- ions which act as a charge carrier. The high conductivity of H^+ and OH^- ions in bulk water occurs due to the tunnelling of H^+ and OH^- ions from one water molecule to the next via hydrogen bonding [90]. The interaction of water molecules and bulk conductivity due to the Grotthus mechanism is shown in Fig. 14.

Furthermore, humidity sensing performance is influenced by grain size, surface-to-volume ratio and surface defects of crystalline which can be altered by defects, doping, compounding, composite formation etc.

5.1. Role of oxygen vacancies

Water molecules, due to their polar nature, show a tendency to reside on oxygen vacancy defects. Oxygen vacancies present in the surface layer are the reason behind dissociative adsorption of the H_2O molecule, which occurs due to the migration of one proton (H^+) to a neighbouring oxygen atom. The dissociative adsorption of one water molecule forms two hydroxyl groups for each oxygen vacancy. Hence, the amount of oxygen vacancies at the surface determines the amount of water dissociation [91]. Surface oxygen vacancy may provide a high charge density and strong electrostatic field for the dissociation of the adsorbed water molecules, which is beneficial for proton hopping transportation in the case of lower %RH. Hence, oxygen vacancies present on the surface play

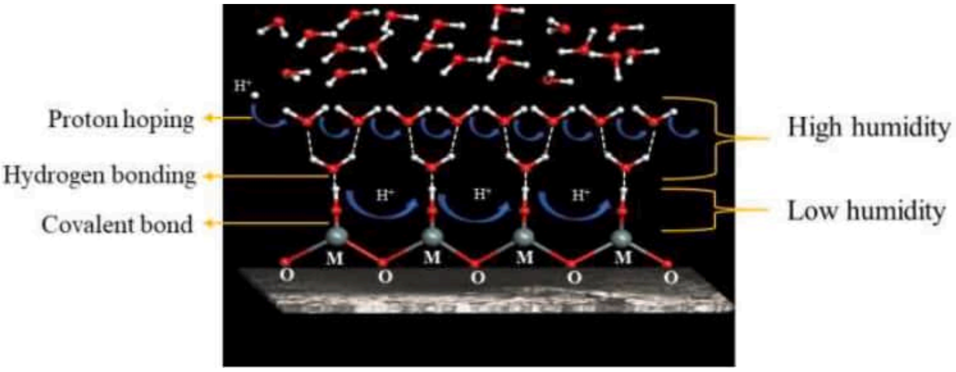

Fig. 14. Interaction of water molecules on the SMOs surface.

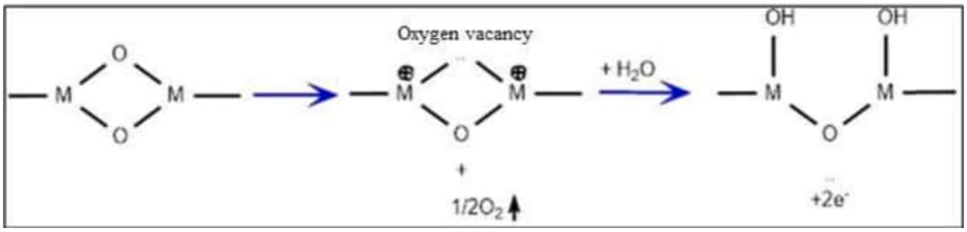

Fig. 15. The mechanism of chemisorption of water molecule on oxygen vacancy site of SMOs.

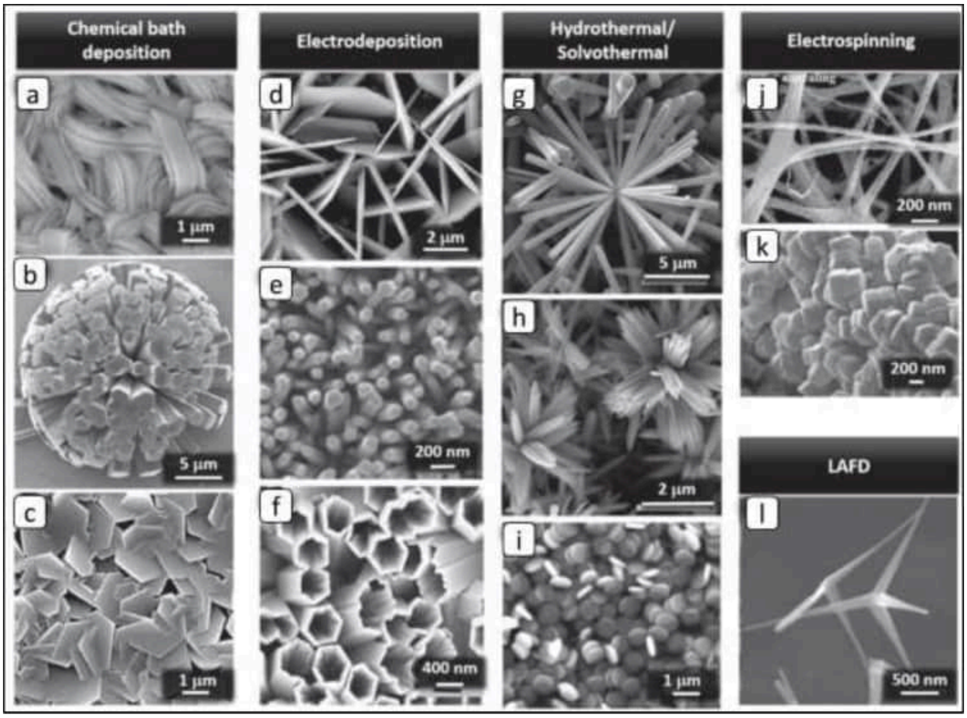

Fig. 16. Different nanostructures of ZnO produced by numerous synthesis methods: (a, b, c) chemical bath deposition, (d–f) electrodeposition, (g–i) hydrothermal/solvothermal synthesis, (j, k) electrospinning and (l) Laser assisted flow deposition (LAFD) [27]. Copyright 2019 IOP Science.

a key role in controlling the humidity sensing performance. The mechanism of chemisorption of water molecules at oxygen vacancy sites on SMOs is shown in Fig. 15.

5.2. Role of dopant

Doping of other elements such as K, Mg, Ga, Er, Eu, Au, Mn, Sb, Sr, among others, is found to result in the enhancement of oxygen vacancies to maintain charge neutrality and also improvement in surface morphology. Depending on their ionic valency, they can further add charge carriers on the surface, producing a strong electric field. This favours more ionization of water molecules and hence high H^+ density. Subsequently, increase in the humidity sensing performance of SMOs-based humidity sensors [92–95].

5.3. Role of composite formation

The composite formation of SMOs with other hygroscopic materials like polymers, graphene oxides, SMOs, functionalized CNTs, etc., is also an effective way to enhance the sensitivity of SMO-based humidity sensors. The polar functional groups like hydroxyl, carbonyl, amine, etc., can form hydrogen bonding with water molecules [2,14,84,96–98]. This favours the water molecules to be adsorbed on the surface. Since surface polarity also plays a significant role in humidity sensing, it can be influenced by varying the number of functional groups of composite materials. Many researchers have proposed improvements in humidity sensor performance with the composite formation in past few years.

Now we discuss the humidity sensing characteristics of various SMO nanocomposites in detail in the following sections:

6. ZnO and its nanocomposites as humidity sensor

ZnO is an n-type wide bandgap (3.2–3.4 eV) semiconductor with a hexagonal wurtzite structure [99,100]. Depending upon the growth conditions and the source materials, a wide range of nanostructures of ZnO have been synthesized like nanorods, nanowires, nanotubes, nanosheets, nanoneedles, nanodiscs, nanospiral, rings nanohelixes, nanobows, nanopropellers, and nanocages [100–106]. A pictorial view of different nanostructures of ZnO can be found in Fig. 16, as given by Nunes et al. [27].

The reason behind the studies of ZnO nanostructures as humidity sensing material is their exceptional properties like controllable surface morphology, wide change in resistance, ease of formation, good operating stability over a wide range of temperatures and %RH, chemical inertness, low-priced synthesis and portability [92]. ZnO nanostructures exhibit not only a large surface-to-volume ratio but also rich surface vacancies, which results in large active sites for chemisorption and, therefore, highly sensitive and selective to change in the ambient conditions [96].

Dulub et al. [107] have studied the water adsorption on ZnO ($10\bar{1}0$)

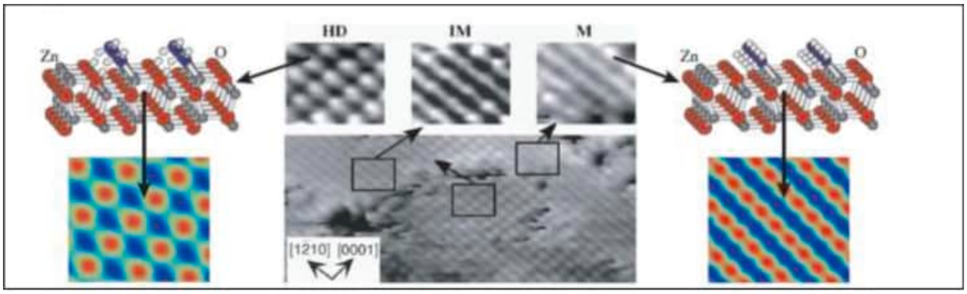

Fig. 17. STM image of full water monolayer on the ZnO ($10\bar{1}0$) surface at room temperature [107]. Copyright 2005 APS physics.

surface using DFT. It was observed that the water adsorption stabilizes in the two lowest degenerate energy configurations: the water molecules possess a half-dissociated state in which every second molecule is dissociated, and the other configuration consists of a fully molecular water monolayer. STM image of full water monolayer on the ZnO ($10\bar{1}0$) at room temperature is shown in Fig. 17. The layer consists of three different structures; a half-dissociated monolayer (HD), a molecular overlayer (MD) and an intermediate structure of both half-dissociated and molecular layer (IM). Being degenerate in energy, there is a balance between the two states, causing a fluctuation between dissociated and undissociated water molecules, which is so fast that it gives an intermediate state when measured with a conventional technique like STM. Similarly, Calzolari et al. [108] have studied DFT on adsorption of a water molecule on the nonpolar ZnO ($10\bar{1}0$) surface. They concluded that at low coverage, water molecules are largely spaced and adsorb in undissociated form. But with an increase in coverage, the partial dissociation of adsorbed water takes place. They also claimed that oxygen vacancies are not additionally effective to favour water dissociation. They have suggested that these results are contradictory to the earlier reported studies, and hence the peculiar behavior of oxygen vacancy in ZnO has to be understood more deeply based on experimental and theoretical investigations. Raymand et al. [109] further performed temperature-dependent DFT calculations on ZnO ($10\bar{1}0$) surface at three different temperatures of 10K, 300K and 600K. It has been found that there is an equilibrium between molecular and dissociated water resulting in half dissociated, half molecular water monolayer. Equilibrium is not reached at low temperature (T= 10K). But, at T=300K and T= 600K, equilibrium is reached between molecular/dissociative adsorption in less than 20 ps.

As discussed in the sensing mechanism that humidity sensing is a surface phenomenon, so humidity sensing characteristics like sensitivity can be increased by increasing the number of surface adsorption sites. The significant factors of ZnO to be utilized as a humidity sensor are large surface-active sites for adsorption of water molecules, high electrical conductivity and surface oxygen vacancies (which boosts the dissociative adsorption of water molecules).

Many researchers have synthesized undoped ZnO nanomaterials for humidity sensing applications. ZnO nanostructures-based humidity sensors with thin films [110], nanowire [111], nanorod [112], nanoparticle [113] and nanofiber [114] sensing elements have been proposed in the past few decades. Tsai et al. [25] have proposed a resistive type humidity sensor in which they have grown ZnO nanosheets on 500 nm-thick SiO_2 coated silicon wafer substrate. The sensor exhibits a response/recovery time of 37/3s, respectively, over the %RH range of 12-96%, with good sensing linearity and low hysteresis. The schematic of the device structure of the prepared ZnO-nanostructured based sensor is shown in Fig. 18(a). The sensor characteristics as a humidity sensor are shown in Fig. 18(b–d).

Gupta et al. [12] obtained mesoporosity in chemically deposited highly crystalline 2D ZnO nanosheets by simple heat treatment. XPS spectra reveal the oxygen ion to be present at the lattice site while the oxygen vacancy and chemisorbed oxygen species lie on the surface of heat-treated ZnO nanosheets (referred to as MZNS). FESEM images reveal the mesoporosity in MZNS samples, as shown in Fig. 19(a and b). It can be seen that MZNS samples have preserved their sheet-like morphology with the introduction of multiple defects (inset of Fig. 19 (b)). The humidity sensor was synthesized on an ITO substrate by itching ~100 μm thick regime on the conducting surface to separate the source and drain. The schematics of humidity sensing, along with device

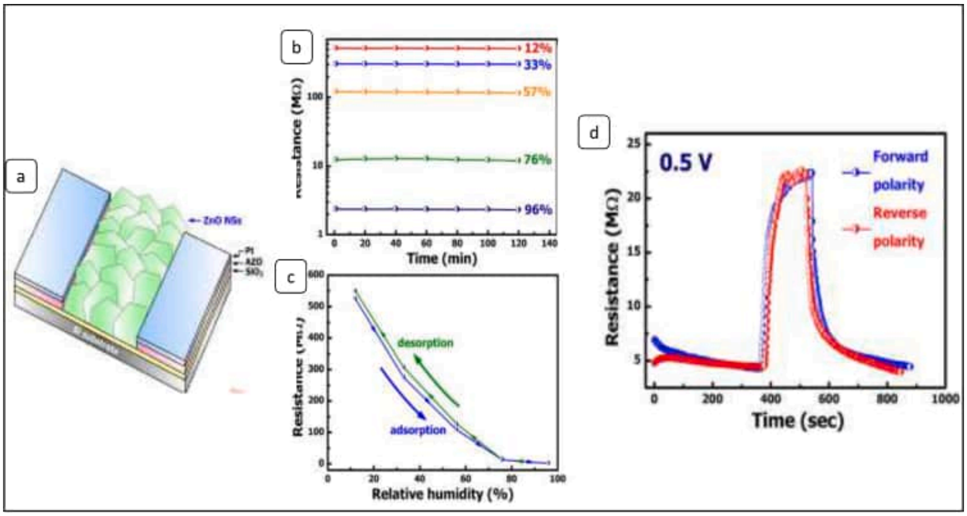

Fig. 18. (a) The schematic picture of the ZnO nano-sheet device, (b) sensor stability with time at different %RH ranges from 12 to 96%, (c) typical hysteresis characteristics and (d) measurement of response and recovery time at %RH between 76 and 96 under an applied voltage of 0.5 [25]. Copyright 2014 Elsevier.

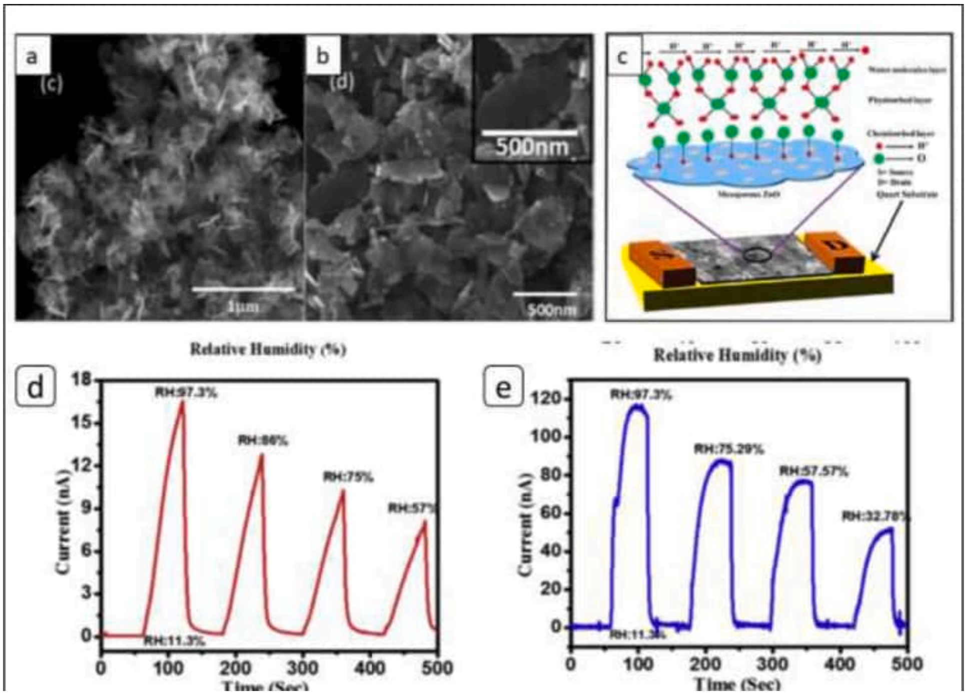

Fig. 19. (a, b) Low and highly magnified FESEM image of heat-treated mesoporous ZnO nanosheets, (c) schematic diagram of fabricated humidity sensor along with sensing mechanism, (d and e) current as a function of time at different %RH of pristine and MZNS samples respectively [12]. Copyright 2019 Elsevier.

design, are shown in Fig. 19(c). The measured currents for pristine and MZNS are plotted with respect to time by varying %RH, respectively and are shown in Fig. 19(d and e) respectively. It is found that MZNS show improved response and recovery time of 25s and 5 s, respectively, than the pristine one with better stability.

Despite previous studies, there is still room for researchers to study the humidity sensing behavior of various undoped ZnO in various morphologies like nanoneedles, nanodiscs, nanospiral, nanorings, nanohelixes, nanobows, nanopropellers, nanocages, etc., which have not been explored till now as a humidity sensor. Further, conventional

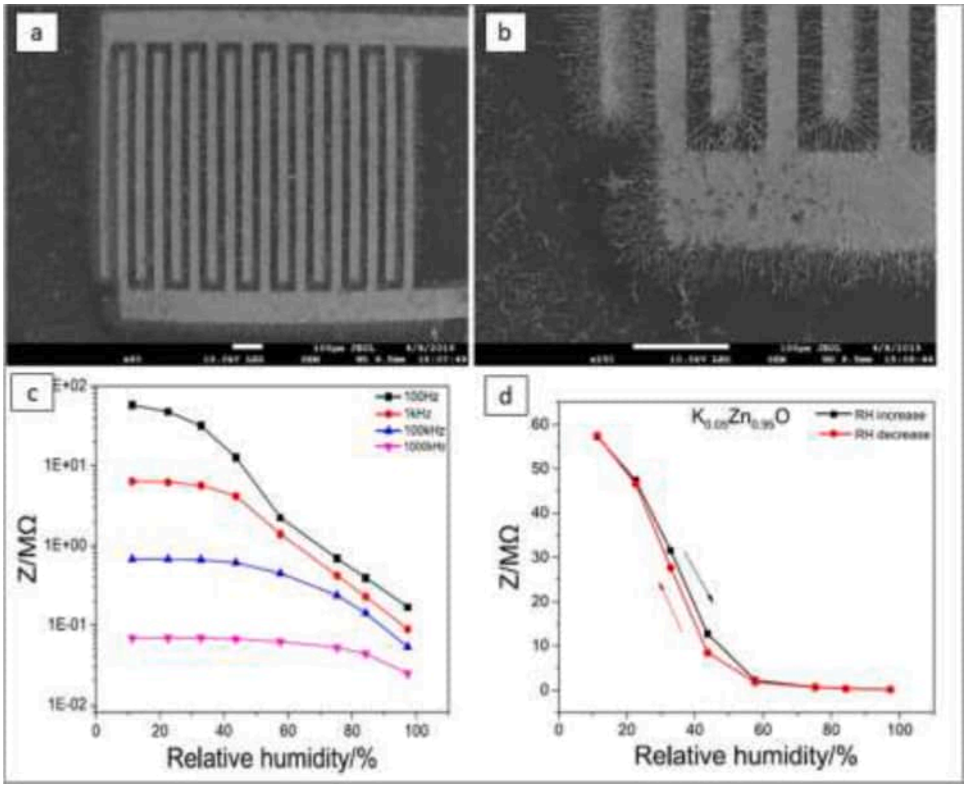

Fig. 20. SEM images of (a) low and (b) highly magnified of $K_{0.05}Zn_{0.95}O$ sensing nanowires distributed regularly over IDEs, (c) impedance vs %RH and (d) absorption–desorption behavior at different %RH [93]. Copyright 2019 springer.

pure ZnO-based humidity sensors are limited in practical applications because of poor linearity [115], relatively less sensitivity towards water molecules due to their hydrophobic nature and relatively high response/recovery time.

Since humidity sensing is a surface phenomenon, so, sensor characteristics like sensitivity, response time, recovery time, accuracy, and drift chemical stability can be improved by engineering in the surface morphology. In order to improve the humidity sensing characteristics, Gu et al. [93] have proposed a K-doped ZnO nanowire, synthesized by hydrothermal method, as an impedance-based humidity sensor. As shown in Fig. 20(a and b), the nanowires are arranged regularly and connected across the IDEs. It has been observed that 5% K-doped ZnO humidity sensor shows maximum sensitivity, low response and recovery time (~8 s and ~2 s respectively), minor hysteresis and superior reproducibility. The variation of impedance with %RH values for $K_{0.05}Zn_{0.95}O$ humidity sensor at different frequencies is represented in Fig. 20(c). A negligible hysteresis can be seen in Fig. 20(d). The reason behind the enhancement of sensitivity is attributed to the generation of oxygen vacancies due to the doping process (which can be occupied by water molecules), which results in increased surface-active sites for the chemisorption of water molecules. With a combination of Complex Impedance Spectroscopy and Freundlich curve fitting, the authors proposed that by tuning the amount of K doping, one can control the surface activity of the material and hence the humidity sensing properties.

In the continuous work of doping in ZnO nanomaterial, Yu et al. [116] prepared Eu-doped ZnO-based impedance type humidity sensor synthesized by the sol-gel method. The addition of Eu in ZnO results in uniform morphology with grain size from 50 nm to 75 nm, which enhances the surface area for the adsorption of water molecules. Furthermore, doping of Eu increases the oxygen vacancies, which increases the number of water molecules adsorbed on the ZnO surface. The sensor showed excellent sensing linearity, small hysteresis, lower response and recovery time of 5s and 19s, respectively. Hong et al. [45] have proposed a SAW humidity sensor based on Ga-doped ZnO nanorod thin film. Ga dopant is found to decrease the grain size of the ZnO seed layers. Subsequently, a decrease in the diameter and increase in the length of ZnO nanorods is observed, which results in the enhancement of the sensitivity of SAW sensor. Peng et al. [117] have studied the performance of impedance type humidity sensor based on Mn-doped ZnO nanopowder prepared by sol-gel technique. Mn doping induces defects on the grain surface, which act as water adsorption sites. Besides this, it also introduces a substantial number of free electrons on the surface, producing a strong electric field on the surface, which results in more ionization of water molecules and hence high H^+ density. This effect is seen in the 6% Mn-doped ZnO nano-powder as greatly improved sensitivity. Furthermore, I-V curves of 6% Mn-doped sample exhibit linear behavior (as shown in Fig. 21(c) at all %RH values, suggesting gold contact is perfectly ohmic with the Mn-doped nanopowder. The SEM image of Mn-doped ZnO nano-powder (not shown here) reveals that grains acquire polyhedron structures with defined facets, edges and vertices. The response and recovery time were reported to be 6s and 20s respectively. The humidity sensor characteristics of the above-discussed sensor are shown in Fig. 21(a and b).

Similarly, composite formations with other hygroscopic materials like polymers, graphene oxides, functionalized CNTs, etc., are also the effective way to improve the sensitivity toward water molecule which contains the polar functional groups that help in the interaction of water molecules with the sensor surface. There are reports where researchers have shown improvements in humidity sensor performance with the composite formation. Patil et al. [2] have studied PVA-modified ZnO nanowires-based humidity sensors. SEM image of ZnO nanowires in Fig. 22(a–c) reveals the diameter of around 40 nm and length of nanowires up to a few microns. The hexagonal structure is confirmed by the tip of the ZnO nanowire. The improvement in the sensor output of the PVA-modified sensor is observed and attributed to the hygroscopic nature of PVA. The humidity sensing characteristics of the sensor are shown in Fig. 22(d and e). PVA-modified ZnO nanowires sensor showed sensitivities of 4.6 kΩ/0.1%RH. Furthermore, sensor revealed good stability, fast response and recovery time, high repeatability and low hysteresis of 1-2%.

Velumani et al. [118] have prepared a composite of two SMOs, i.e. a ZnO-SnO_2 composite thin film-based impedance type humidity sensor prepared by magnetron sputtering to see how the system behaves towards humidity sensing. It is found that the composites show an improved response (~21 s) due to the enhanced surface area and the defect sites. Despite this, the composite shows poor sensitivity than undoped SnO_2. The authors suggest that the sensitivity can further be improved by optimizing the Sn/Zn ratio in such composites. Very recently, Li et al. [119] prepared a ZnO/SnO_2 humidity sensor by a simple solvothermal method by varying the ZnO concentration (accordingly, named Z/S-1 to Z/S-4), and the sensing mechanism was investigated by Complex Impedance Spectra. The composite is found to show suitable linearity and stability, small hysteresis, high response, and response and recovery times of 35 s and 8 s, respectively. SEM images (as shown in Fig. 23(a-c)) reveal ZnO to be a rod-like structure while SnO_2 as a particle of diameter 1 μm. In composite, ZnO was uniformly attached to SnO_2 structures to provide an enhanced surface area. TEM images (Fig. 23(d and e)) further show the lattice fringes of SnO_2 and ZnO; having the lattice spacings of 0.14 and 0.34 nm corresponding to the (200) plane of ZnO, and the (110) plane of SnO_2, respectively. It is seen Fig. 23(f) that a composite with 0.041 g of ZnO (Z/S-2) shows the best linearity behavior as a function of %RH at a frequency of 100Hz. Fig. 23(g) shows the response and recovery characteristics of the humidity sensor, and it was observed that the impedance changes at different %RH remained pretty constant, indicating that the sensor has suitable repeatability.

Burman et al. [120] have further studied ZnO/MoS_2 nanocomposite-based resistive humidity sensor by mixing the MoS_2 nanoflakes with ZnO (synthesised by chemical bath deposition). MoS_2 is known to have inherent defects in its structure, and it was envisaged to have improved proton conductivity inside the water layer. The composite showed a response of ~30 times at 35% RH and ~301 times at

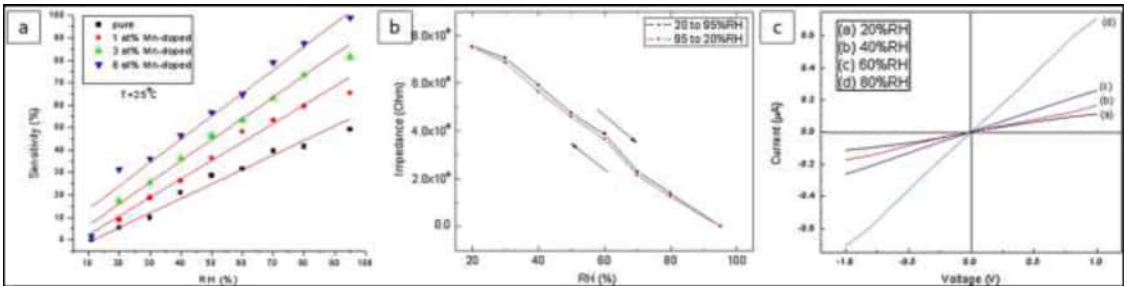

Fig. 21. (a) Sensitivity as a function of % RH for different contents of Mn in ZnO, (b) hysteresis characteristic of the 6% Mn-doped ZnO nanopowder at different humidity levels and (c) I–V curves in different %RH conditions at room temperature [117]. Copyright 2012 Elsevier.

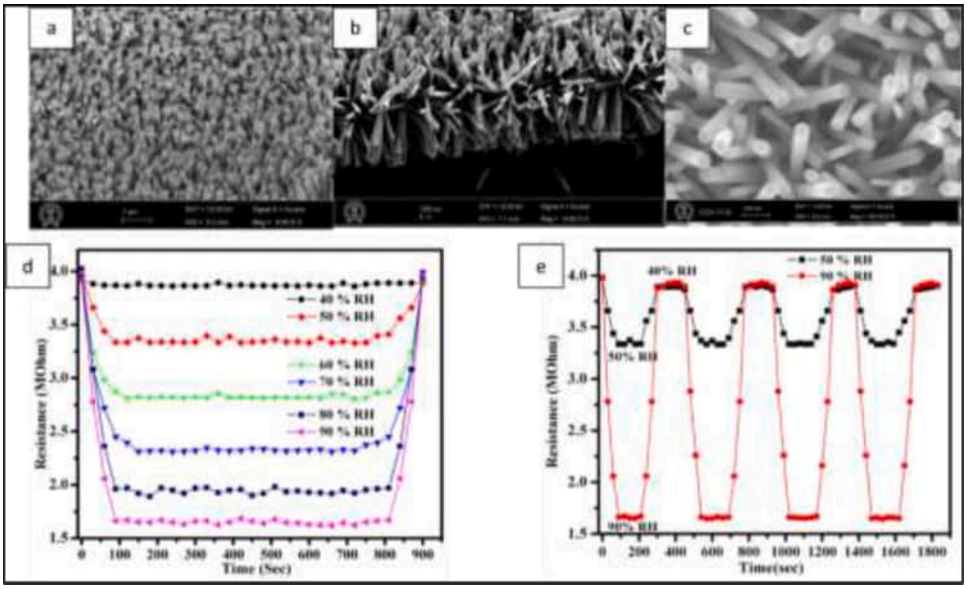

Fig. 22. SEM images of ZnO nanowire structures (a) from top view, (b) 45° incline angle and (c) with higher magnification, (d) sensor response at different %RH levels and (e) the repeatability of the sensor at 50 and 90%RH [2]. Copyright 2017 Elsevier.

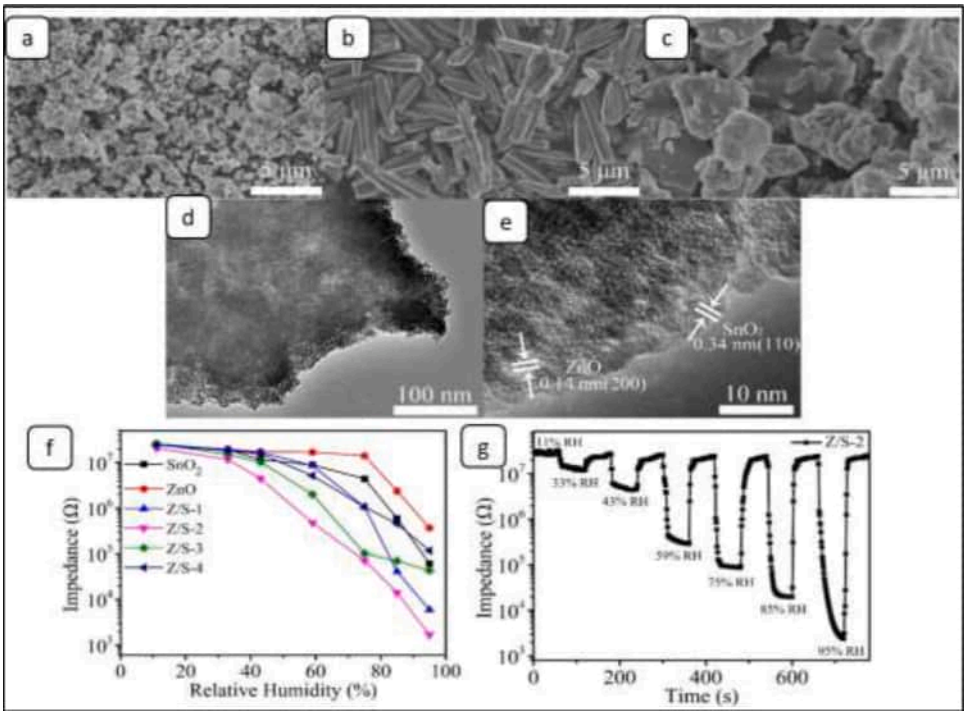

Fig. 23. SEM images of (a) SnO_2, (b) ZnO, and (c) Z/S-2 composite, (d, e) TEM spectra of Z/S-2 composite, (f) impedance changes of humidity sensors with different ZnO concentrations at 100 Hz and (g) response and recovery features of Z/S-2 humidity sensor at different %RH [119]. Copyright 2017 Springer.

85% RH, much higher compared to pristine MoS_2 nanoflakes or ZnO nanowires. The sensors show high reproducibility, fast response and recovery time with slight hysteresis. The authors finally fabricated a prototype sensor on a printed circuit board which was controlled over Wi-Fi based on an android application. The prototype sensor with Smartphone interfacing and humidity sensing behavior have been discussed by the authors.

Researchers have made efforts in humidity sensors based on the heterojunction of ZnO, which could promote a faster electron transfer phenomenon. Wang et al. [121] demonstrated a humidity sensor based on a ZnO/porous GaN (PGAN) heterojunction deposited by the spraying process. An etched GaN film with an Al_2O_3 layer was used as the substrate. ZnO film was then deposited on an insulating layer deposited on the PGAN substrate. Ag was deposited on ZnO and GaN surfaces as top and bottom electrodes. The schematic of the sensor design and the various steps involved in the fabrication is shown in Fig. 24(a). The authors suggested that in this type of heterostructure, due to the diode nature, the sensing mechanism is mainly due to electronic conduction. A pictorial diagram is given for understanding the interaction of water molecules with heterostructure surfaces, as shown in Fig. 24(b). The measured response and recovery times are 7s and 13s, respectively, when %RH is changed from 96% to 12%. The humidity response for ZnO

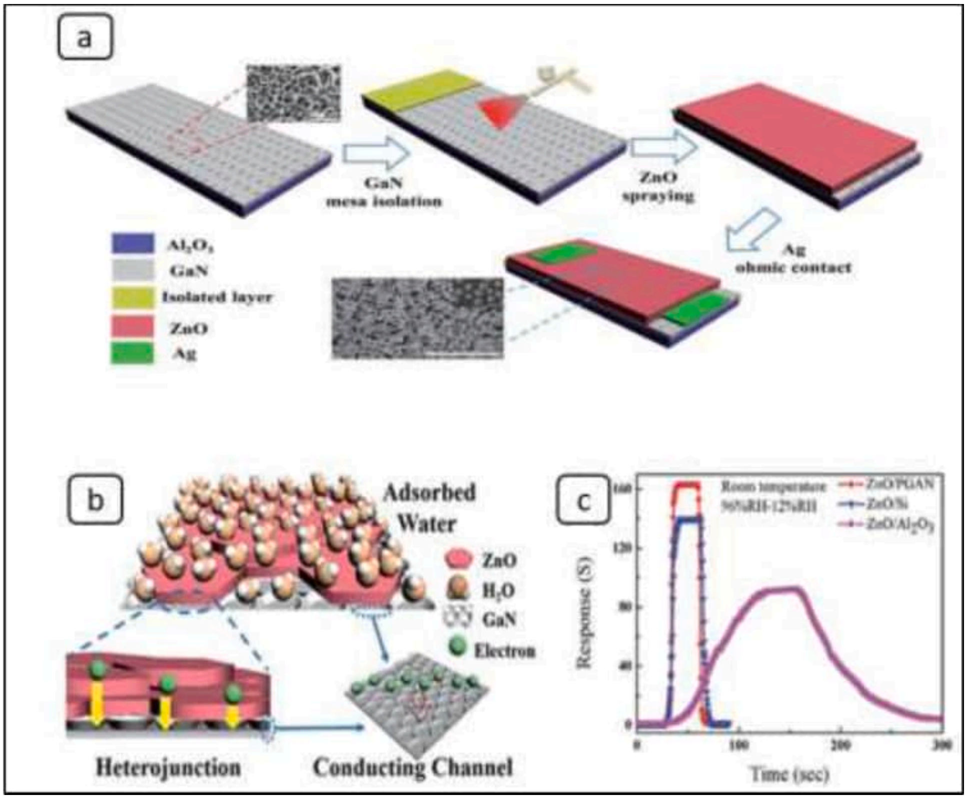

Fig. 24. (a) Schematic view of the ZnO/PGAN hetero-junction for humidity sensor, (b) pictorial diagram to show the interaction of H_2O molecule with ZnO/PGAN heterostructure which illustrates the electron transport at ZnO vis-à-vis porous surface of GAN and (c) response behaviors of ZnO/PGAN, ZnO/Si and ZnO/Al_2O_3 based humidity sensors [121]. Copyright 2019 Royal Society of Chemistry.

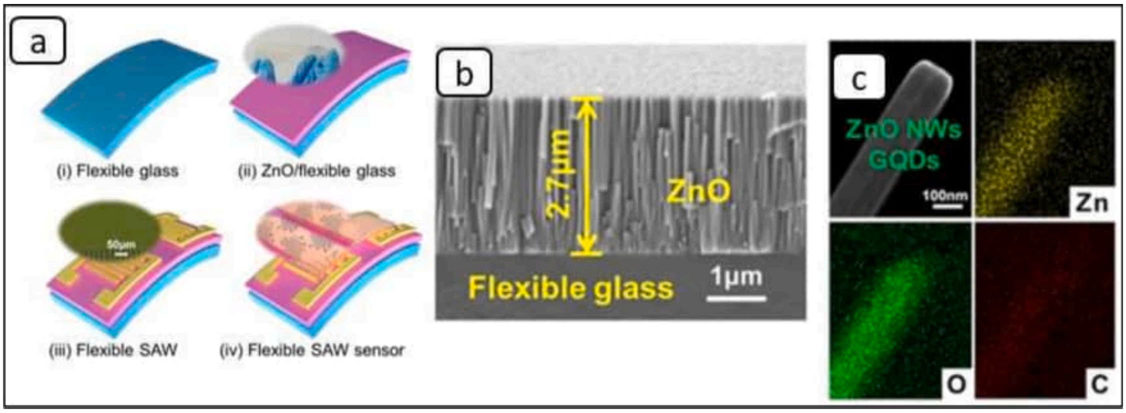

Fig. 25. (a) Schematic illustration of the fabrication process for the ZnO NWs and GQDs based SAW sensor, (b) Cross-sectional view of the ZnO layer and (c) The mapping of Zn, O, and C elements in
composite of ZnO NW and GQD with EDS analysis, illustrating the coating of GQDs on the ZnO NWs [102]. Copyright 2020 American Chemical Society.

grown on Si and Al_2O_3 substrates and the ZnO/PGAN heterojunction are shown in Fig. 24(c). The author claimed that these are much improved results compared to any other films of ZnO on other substrates, hence can be suitable for various applications.

In recent work, Wu et al. [102] have synthesized a new sensing SAW layer by integrating ZnO nanowires with graphene quantum dots (GQDs) on a flexible glass substrate. Fig. 25(a) shows the schematic picture of the various steps involved in the fabrication of ZnO and GQDs-based SAW sensors. The cross-sectional SEM image (Fig. 25(b)) demonstrates the columnar growth of ZnO nanocrystals in a direction perpendicular to the substrate, as revealed by SEM images. The elemental distribution in the composite layer was also obtained using the energy-dispersive spectroscopy (EDS) analysis, which suggests that the GQDs composed of C and O are indeed coated on the ZnO nanowires (Fig. 25(c)). The developed sensor showed ultrahigh humidity sensitivity of 40.16 kHz/% RH, along with its excellent stability and repeatability, which may be attributed to the large surface areas of ZnO nanowires and large numbers of hydrophilic functional groups of GQDs.

In continuation to the study of ZnO and its composites-based humidity sensor, the summary of ZnO-modified humidity sensors with sensor characteristics is given in Table 2.

Table 2
Humidity sensing performances of ZnO modified humidity sensors.

S. No	Material	Type	Humidity range (% RH)	Method of synthesis	Response time(s)	Recovery time(s)	Remarks	Refs.
**Pure ZnO**								
1.	ZnO nanoparticles	Impedance	11-95	**Hydrothermal**	31	15	The sensor exhibited high sensitivity (14,415), good linearity, small hysteresis (0.9%), by introducing alkaline nature in synthesized ZnO sample	[92]
2.	Flowerlike ZnO nanorods	Impedance	11-95	**Wet-Chemical**	5	10	High sensitivity, rapid response and recovery with the novel ZnO flowerlike nanostructure.	[122]
3.	ZnO nanowires	Optical fibre	35-60	**Sol-gel**	-	-	The sensitivity of the sensor is approximately 0.0205nm/% RH with slope linearity of more than 99.58 and a limit of detection of 0.02%.	[123]
**Doped-ZnO**								
4.	Ce doped ZnO thin film	Impedance	11-95	**Sol-gel**	13	17	Narrow hysteresis loop, a good, wide range of response from 10^8- $10^4\Omega$ in the humidity range.	[124]
5.	Mn doped ZnO nano-powder	Impedance	11-95	**Sol-gel**	6	20	Three order change in conductivity from 20% to 95%	[117]
6.	Ga doped ZnO nanorods	Surface acoustic wave	10-90	**Sol-gel**	-	-	Enhance in sensitivity of SAW humidity sensor.	[45]
7.	Sn doped ZnO nanorods	Resistive	40-90	**Sol-gel**	230	30	Small crystallite size, pore channels due to Sn doping enhanced the adsorption of water molecules.	[125]
8.	Cu doped ZnO thin film	Resistive	15-95	**Sol-gel**	32	47	Improved sensitivity, low hysteresis, and better response/ recovery time compared to pristine ZnO.	[126]
9.	Er doped ZnO nanoparticles	Impedance	11-95	**Wet-chemical**	32	39	Three order more sensitivity compared to pristine one, improved linearity, low hysteresis and fast response/ recovery time.	[127]
10.	Mg doped ZnO microspheres	Impedance	11-95	**Sol-gel**	24	12	1.5 mol% Mg-doped ZnO exhibits four order more sensitive than pristine one.	[128]
**ZnO-Composites**								
11.	ZnO-PVA Nanowire	Resistive	40-90	**Hydrothermal growth**	40	60	Improved stability, high repeatability, high sensitivity, fast response and recovery with low hysteresis of 1-2 %.	[2]
12.	ZnO-cellulose nanocomposite	Impedance	40-90	**Aqueous chemical bath deposition**	8	10	High sensitivity of 4.487 MΩ/ %RH.	[17]
13.	ZnO- Poly(diphenylamine sulfonic acid) (PSDA)-3-mercaptopropyltrimethoxysilane (MPTMS)	Impedance	12-95	**Sol-gel**	90	60	Three order more sensitivity, linear response, and low hysteresis within 4%, good repeatability and high stability.	[129]
14.	ZnO-PVDF nanocomposite	Resistive	5-95	**Hydrothermal growth**	30	51	Fast response and recovery with good stability.	[130]
15.	ZnO-graphene nanocomposite	Capacitive	0-85	**Composite formation**	1	2	Improvement in detection range of the sensors while maintaining a fast transient response.	[84]
16.	ZnO-rGO-Cu	Resistive	11-97	**One-pot synthesis method**	19	42	Low humidity hysteresis and stable humidity sensing ability.	[131]
17.	ZnO-polypyrrole composite	Resistive	5-95	***In-situ* polymerization and composite formation**	12	8	1000 times better electrical conductivity than pristine one.	[132]
18.	ZnO/chitosan/SWCNTs	Resistive	11-97	**Hydrothermal**	-10	-	The composite shows nonlinear which may correspond to the water sorption nature of chitosan.	[133]

7. TiO_2 and its nanocomposites as humidity sensor

TiO_2 behaves as an n-type wide bandgap (3.2–3.35 eV) semiconductor. It usually exists in three phases in the crystalline structure, including rutile, anatase, and brookite [3,134]. The most stable form of TiO_2 is rutile while anatase and brookite are metastable states which eventually transform into the rutile phase when exposed to high temperatures. The reported temperature for phase transfer from amorphous to anatase occurs at a temperature of approximately 500°C and further transforms to the rutile phase at 500–600°C [135,136]. However, in thin-film form, the phase of TiO_2 can be controlled by controlling the growth parameters. TiO_2 possess a high refractive index of 2.71, 2.52 and 2.64 for rutile, anatase and brookite, respectively [137]. TiO_2 thin films have a high dielectric constant ranging from 50 to 100 (depending on film thickness) and high resistance range [138].

In the past few years, researchers have reported numerous techniques to synthesize TiO_2 nanostructures such as sol-gel method [139], wet-chemical synthesis [140], thermal-evaporation [141], sputtering [142], electrodeposition [143], hydrothermal and solvothermal synthesis [144], microwave irradiation [145], etc. TiO_2 exhibits various nano-morphologies such as nanorods, nanowires, nanobelts, nanotubes, nanofibers, among others [146–151]. In TiO_2, high chemical inertness, low-cost fabrication and fast response/recovery time make them suitable for use in fibre optics, photovoltaics, biocompatible materials, and dye-sensitized solar cells [152].

When exploited as a humidity sensor, TiO_2 materials show hydrophilic nature due to the intrinsic presence of Ti^{3+} defect sites or oxygen vacancies, at which adsorption of water molecules takes place [91]. The amount of oxygen vacancies at the surface determines the amount of water dissociation, as discussed earlier. TiO_2 anatase phase is extensively studied as a sensing material because it has a highly sensitive/reactive surface that results in high water adsorption capacity. On the other side, Brookite favours molecular as well as dissociative adsorption of the water molecule. Some experimental data suggest that these two mechanisms may occur simultaneously. Perron et al. [153] have performed an experimental and theoretical calculation to understand water sorption on the rutile phase of TiO_2. Angle resolved X-ray photoemission spectroscopy spectra (ARPES) was collected to investigate the interaction of water in different surface layers. ARPES findings suggest that the first water molecule layer is strongly bonded to the surface while bonding degrades towards the upper layers. Based on DFT calculation, the author studied the role of hydrogen bonding in influencing water sorption and studied the possibility of molecular and dissociated water by considering six different geometries with different numbers of hydrogen bonds. The authors concluded that molecular sorption of water is energetically favoured, but dissociative one can also be predicted because it could be stabilized by hydrogen bonding. Li et al. [154] reveal that in the anatase phase, water molecules get adsorb on the anatase (101) plane and adsorption energy is found to be 0.71 eV, while there is a very less probability of water dissociation on the TiO_2 surface. Similarly, there are reports which suggest dissociative adsorption as the most stable structure, while some published the result with the molecular favoured structure [155,156]. These results indicate that surface defects water coverage layer and thickness of slab considered in DFT play an important role and influence the stability of different water sorption structures. Hence, the adsorption phenomenon needs to be investigated considering variation in these parameters. To improve the humidity sensing characteristics, researchers have introduced porosity and modified the TiO_2 nanostructures by various means.

Recently, exploring the undoped TiO_2 as a humidity sensor, Shen et al. [157] have fabricated a self-powered sensor based on TiO_2 nanowires, which produce voltage due to the diffusive intervasion of adsorbed water vapour on TiO_2 nanowire. The fabricated sensor and SEM image of TiO_2 nanowires are shown in Fig. 26(a and b), respectively. The SEM images show loosely aggregated nanowires, having a diameter of about 50−200 nm. The XPS spectrum shows that TiO $_2$ nanowires exhibit some intrinsic oxygen vacancies, as expected. The sensor works in the %RH range of 20–90% and the output voltage obtained may be due to the diffusive penetration of ions along with the widespread network of nano-channels in the TiO_2 structure. The response and recovery times were found to be 4.5s and 2.8s, respectively. This fabricated humidity sensor is flexible as shown in Fig. 26(a), which retained its sensor characteristics over thousands of times of bending cycles. The humidity sensor characteristics, including sensitivity and response/recovery time of the given sensor, are shown in Fig. 26(c–f).

Towards the progress on undoped TiO_2, in another report, Duborg et al. [146] have proposed a miniaturized and flexible resistive type humidity sensor based on TiO_2 nanoparticles deposited on a flexible PET substrate by a screen-printing method. The photograph and SEM images of the printed sensors are shown in Fig. 27(a) and Fig. 27(b and c), respectively. The fabrication process is low-cost and does not require annealing and further treatment with chemicals. The I-V characteristics

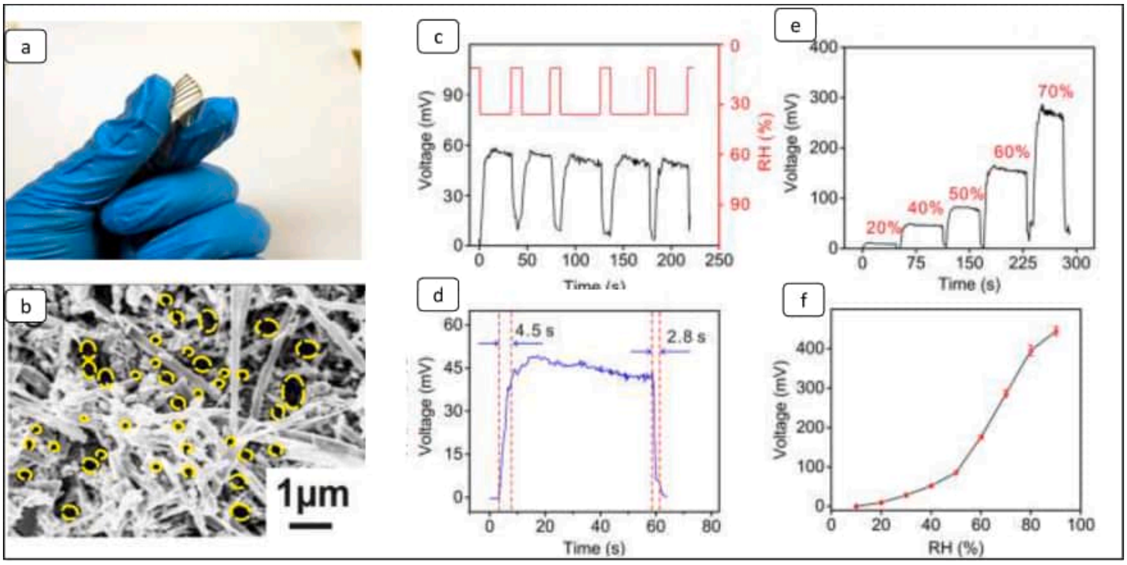

Fig. 26. **(a)** Image of a flexible TiO_2 nanowires device, (b) SEM image of TiO_2 nanowires, (c) repeatability of voltage in response to a periodical change in %RH, (d) measurement of response and recovery time, (e) voltage change with periodic change in the %RH value and (f) voltage vs %RH graph [157]. Copyright 2019 American Chemical Society.

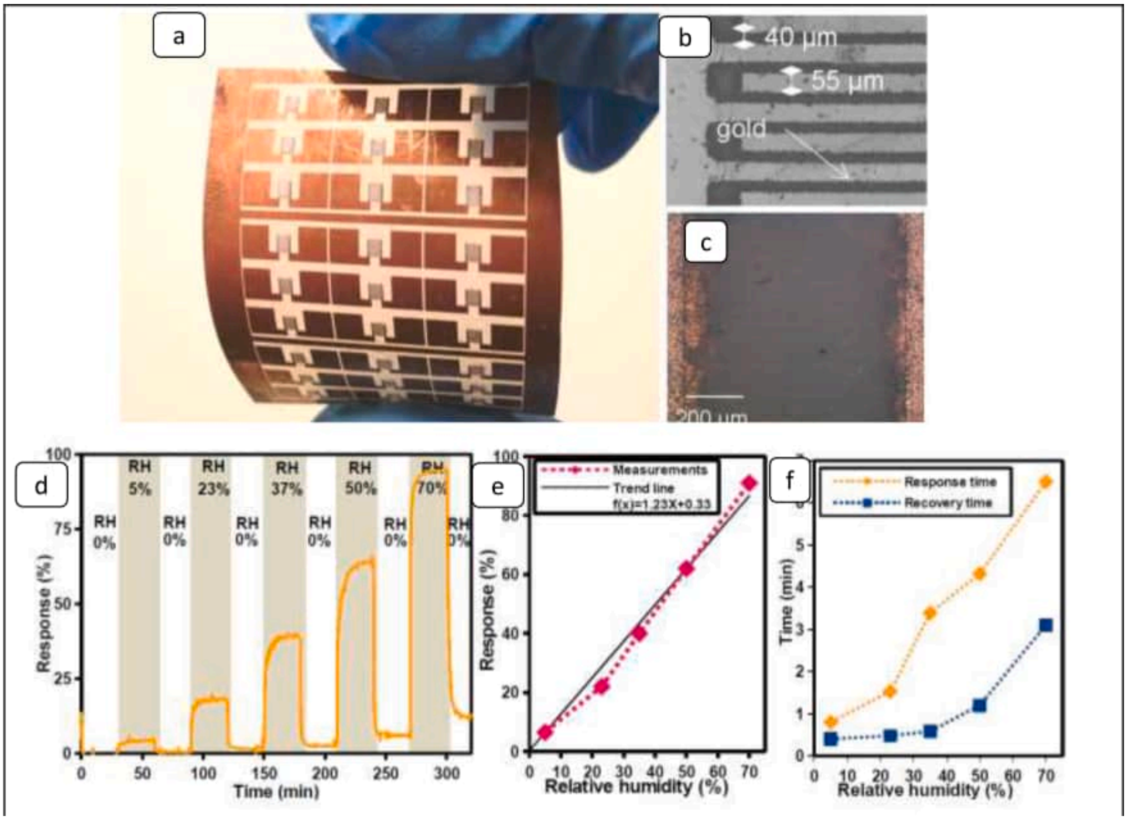

Fig. 27. (a) Image of IDEs printed on PET substrate, (b) SEM image of the IDEs, (c) SEM image of the fabricated sensor after deposition by screen printing, (d) sensor response with %RH change ranging from 0 to 70 and stability with time, (e) sensor response at different %RH value and (f) response and recovery curve of a given sensor [146]. Copyright 2017 MDPI.

of the senor showed good linearity and potential for low power generation. Also, the sensor exhibits a linear response in the range from 5 to 70%RH. The fabricated sensor and its humidity sensor characteristics are shown in Fig. 27(d–f). It was observed that the sensors showed response even in the very low %RH, which may be due to the highly porous surface that results in a large surface area and more favourable sites for the adsorption of water molecules.

Zhang et al. [158] synthesized undoped TiO_2 into different nanostructures, such as hollow balls, nanosphere, and nanoflower as QCM humidity sensors, by different chemical routes. The humidity sensing characteristics showed that surface activity plays a crucial role. The metal-organic framework-derived hollow ball-like TiO_2 material as a QCM sensor revealed superior humidity sensing characteristics in a wide range of very low and (till now) high %RH ranges. The sensor exhibits high sensitivity (33.8 Hz/%RH), good repeatability and remarkably short dynamic response/recovery times (5 s/2 s). The stability of the sensors was evaluated based on the quality factor which is the ratio of peak frequency to the bandwidth at half maxima in conductance spectra, which is found to be highest for the hollow ball-like structures. Shinde et al. [159] recently reported a facile synthesis of undoped TiO_2 nanoflower using a modified hydrothermal method and its humidity sensing. They also studied its cytotoxic activity toward human cells, as discussed later in this review article. Fig. 28(a and b) shows the SEM images of as synthesised TiO_2 nanoflower revealing petals of 20–15 mm long, bound in bundles, forming the typical TiO_2 flower-like growth. Response and recovery time were obtained as 143 s and 33 s, respectively (Fig. 28(c)).

Further, extensive investigations have been carried out to enhance the surface properties of TiO_2-based humidity sensors by varying the

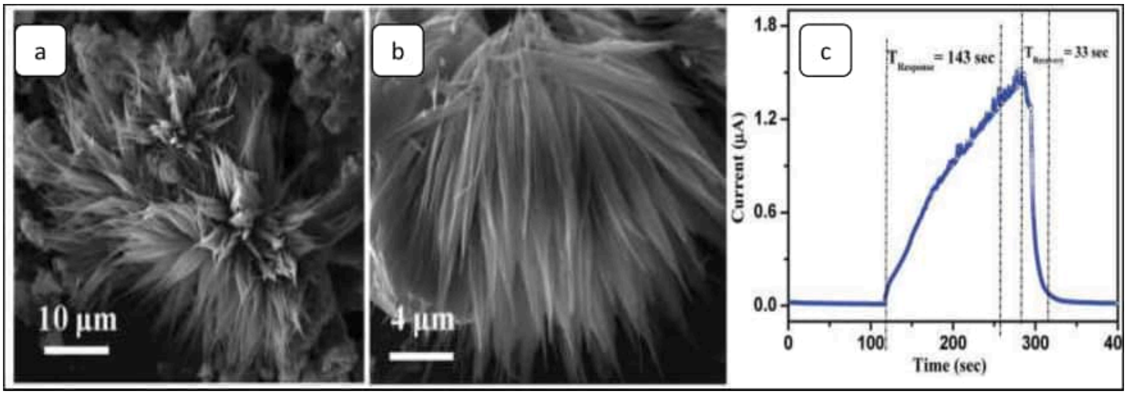

Fig. 28. (a,b) SEM images for TiO_2 nanoflower at low and high magnifications and (c) response and recovery time pattern for developed TiO_2 nanostructure [159]. Copyright 2019 IEEE.

synthesis method, doping of other elements, composite formation, etc., to improve sensitivity and stability and to decrease response/recovery time. Doping of other elements, such as Ce, La, K, Cu, Na Cu, Li, Mg, etc., is suggested to be one of the most effective methods to improve the humidity sensing properties. In past years, researchers have reported many articles on doping of various metals and oxides to get high-performance TiO_2-based humidity sensors.

Farzaneh et al. [35] have proposed a QCM humidity sensor based on Cu-doped TiO_2 films synthesized by the sol-gel method. SEM images of pure TiO_2 and Cu-doped TiO_2 showing rough surface morphology results in the higher surface area for adsorption of water molecules which are obtained due to the sol-gel route. Authors have found an increase in the adsorption energy and charge transfer values of the water molecules in the case of Cu-doped TiO_2. Also, Farzaneh et al. [151] have proposed a Ru-doped TiO_2 nanofibers QCM humidity sensor fabricated by the electrospun technique. Authors also performed DFT calculation of anatase and rutile structure of doped and undoped TiO_2 and revealed that Ru-doping increases the adsorption energy of water molecules by about 46% in the anatase phase and 87% in the rutile phase, which results in a higher tendency of water molecules to interact with Ru-doped TiO_2. Consequently, the doping of the Ru atom in both structures leads to a stronger interaction between the water molecule and the surface. The enhanced sensitivity of Ru-doped TiO_2 nanofibers towards the water molecules could be attributed to more favourable charge transfer between the adsorbed water molecules and the surface. SEM images are shown for the calcined samples (Fig. 29(a and b)), revealing that doped nanofibers are thicker than pure TiO_2, and calcination has opened up the nanofiber networking. The comparison of humidity sensing characteristics of Ru-doped and undoped TiO_2 nanofibers is shown in Fig. 29(c and d).

Zhang et al. [160] synthesized a rutile TiO_2 nanofiber humidity sensor, combined with Mg^{2+} and Na^{+} via electrospinning, followed by calcination method. The detailed synthesis procedure is shown in Fig. 30 (a). It is observed that the TiO_2 containing Mg^{2+} and Na^{+} are nanofiber in structure with an average diameter of 200 nm. The hysteresis behavior of the sensor is shown in Fig. 30(b), which reveals a negligible difference in the adsorption and desorption. The authors have claimed that the sensor possesses high response and recovery time of 1 s and 2 s, respectively, good reproducibility and, importantly, anti-fogged

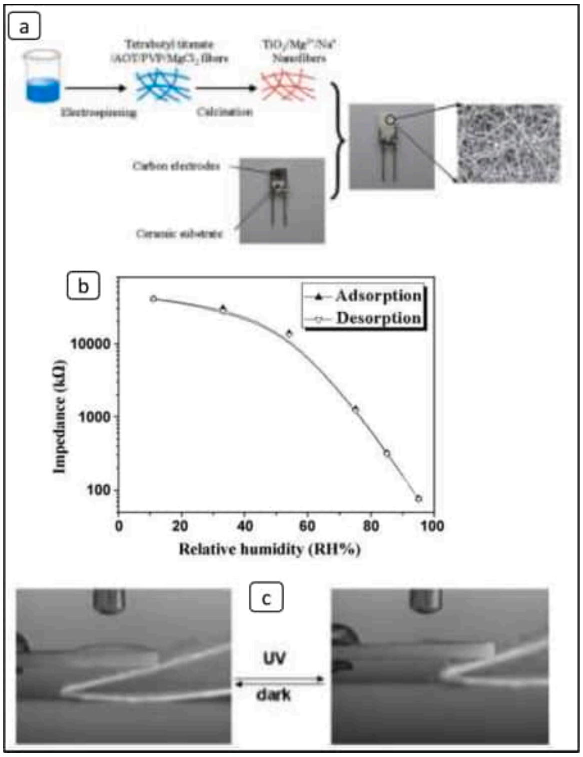

Fig. 30. (a) Schematic diagrams of the steps to fabricate Mg^{2+}/Na^{+}-doped TiO_2 nanofiber mats for humidity measurement via electrospinning and calcination, (b) hysteresis behavior and (c) change in water contact angle under the illumination of UV light [160]. Copyright 2009 Elsevier.

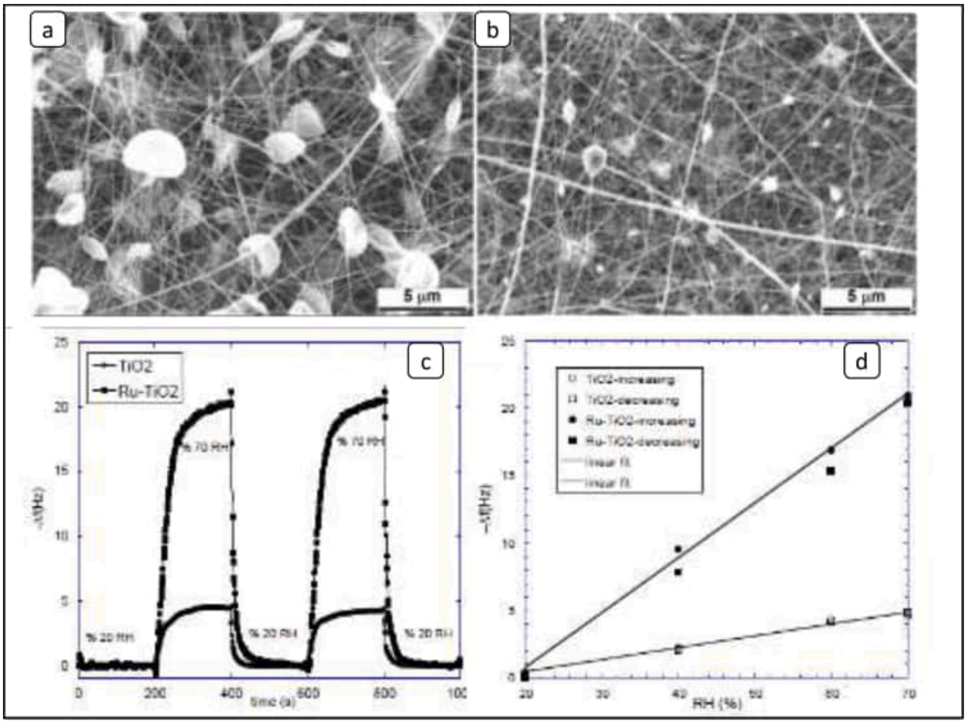

Fig. 29. (a,b) SEM image of calcinated undoped and Ru-doped TiO_2 nanofibers, respectively, (c) frequency shift for adsorption and desorption cycle between 20%RH and 70%RH for undoped and Ru-doped TiO_2 nanofibers and (d) linear relation between frequency shift and %RH [151]. Copyright 2020 Elsevier.

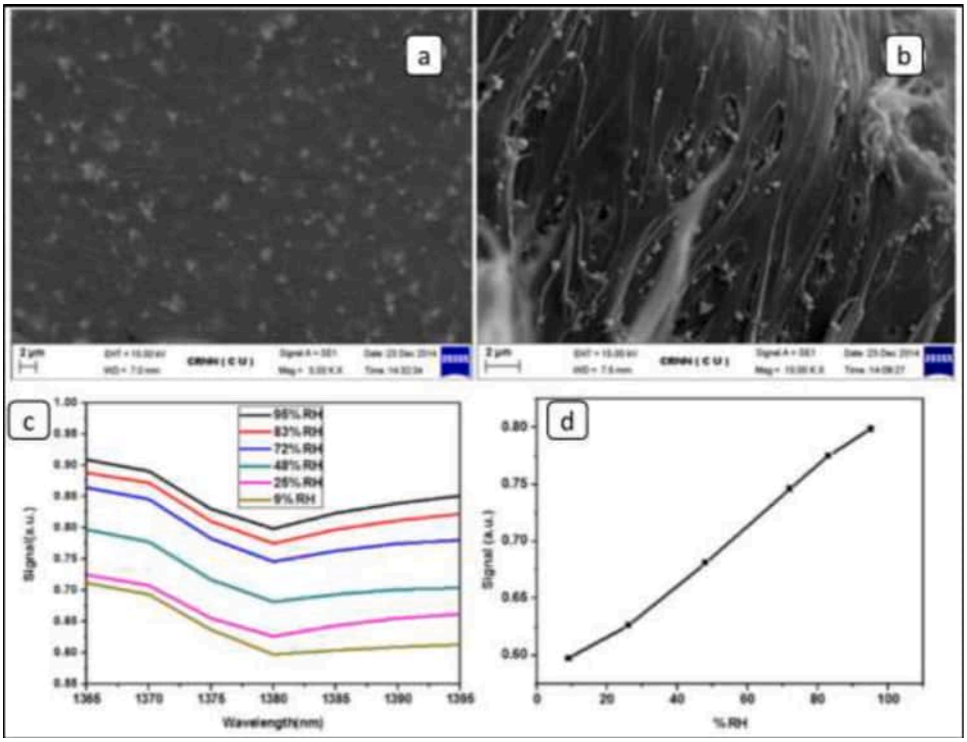

Fig. 31. (a) FESEM image of PVA-TiO_2 composite film, (b) Cross-sectional view of the film, (c) variation in the intensity of the signal at various %RH of light having a wavelength of 1380 nm and (d) variation in Transmission signal at different %RH at 1380nm wavelength [161]. Copyright 2017 Exeley.

properties when comes under UV radiation where a water contact angle changes to nearly 0° (as shown in Fig. 30(c)) endowing the humidity sensor with the anti-fogged properties.

Here, we discuss some work reported on the composites of TiO_2. Khatua et al. [161] proposed an optical fibre-based humidity sensor based on polyvinyl alcohol (PVA)-TiO_2 nanoparticle polymeric composite material. FESEM image shows a homogeneous distribution of TiO_2 throughout the PVA membrane, as shown in Fig. 31(a). The cross-sectional view in Fig. 31(b) shows large numbers of pores on the surface, which may lead to an increase in the surface-active sites for the adsorption of water molecules and hence subsequently increase the sensitivity of the given sensor. The humidity sensing characteristic of synthesized polymer composite is evaluated by depositing them on optical fibre. Since the water has a strong absorption around 1380 nm, a change in the transmission peak of PVA-TiO_2 nanoparticles at 1380 nm was observed with a change in the %RH value. The change in the

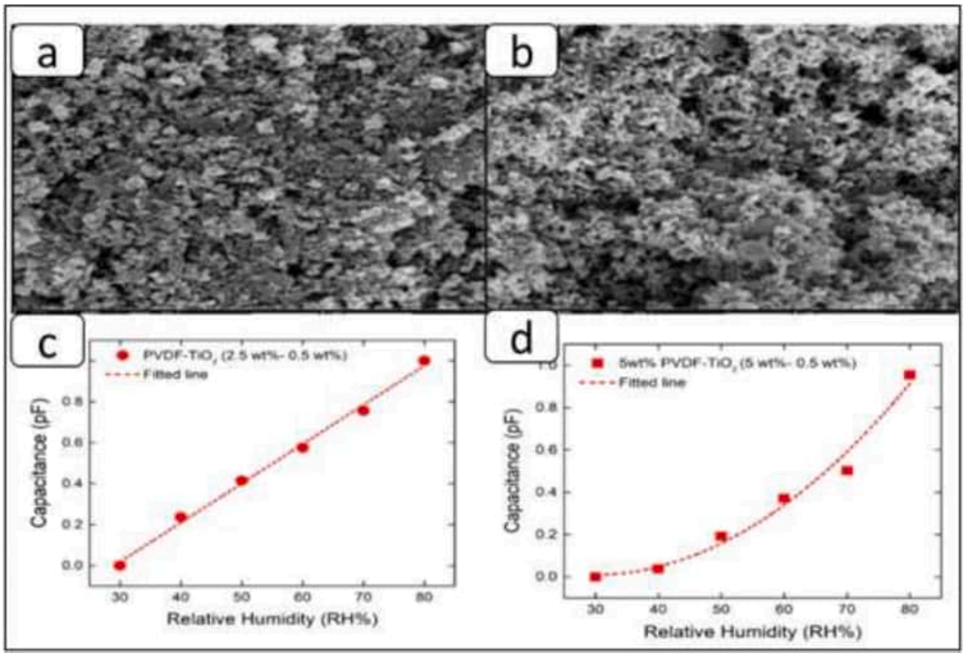

Fig. 32. (a, b) Unetched and etched surface of PVDF-TiO_2 sensing film and (c, d) change in capacitance with %RH of PVDF-TiO_2 (2.5 wt%- 0.5 wt%) and PVDF-TiO_2 (5 wt%- 0.5 wt%), respectively [162]. Copyright 2019 Elsevier.

refractive index of sensing material due to adsorption/desorption of water alters the intensity of the transmission peak around 1380 nm, as shown in Fig. 31(c). The fabricated sensor exhibited not only good sensing performance but also excellent repeatability with a good response and recovery time of 27 s and 30 s, respectively. The humidity sensing response of a given sensor with varying %RH is shown in Fig. 31 (d).

Mallick et al. [162] proposed a capacitive type humidity sensor based on polyvinylidene fluoride (PVDF)-TiO_2 nanocomposites. The morphological studies of FESEM and AFM confirmed the surface roughness of composite thin film etched by acetone which results in the enhancement of surface-active sites for adsorption of water, as shown in Fig. 32(a and b). The sensor showed linear and stable response over the relative humidity ranging from 30 to 80%RH with a response and recovery time of 45s and 11s, respectively and minimum hysteresis of 6%. The change in the capacitance with a relative humidity of the PVDF-TiO_2 based sensor of different compositions are shown in Fig. 32(c and d), respectively.

To enhance the humidity sensing properties, Gu et al. [163] introduced 1D ZnO nanostructures into TiO_2 nanostructures to combine the high chemical and physical stability, good sensitivity as well as fast response and recovery time characteristics of 1D ZnO nanostructure. In this study, ZnO/TiO_2 core/shell nanorod arrays were fabricated through sol-gel processes. The microscopic studies, as shown in Fig. 33(a and b), demonstrated that the majority of the anatase TiO_2 nanoparticles had covered the ZnO nanorods. The humidity sensing performances of pristine TiO_2 films, ZnO nanorods and the composite are shown in Fig. 33(c and d), suggesting the response, as well as the sensitivity of the TiO_2, coated ZnO nanorods were considerably enhanced. Although, the response and recovery behavior of the substrate were not affected much by the composite formation. The study indicates that synergic effect of nanostructured composites of ZnO-TiO_2 leads to enhance in surface morphology and hydrophilicity, results in improvement in humidity sensing properties.

A detailed view of TiO_2 modified Humidity sensors is presented in Table 3.

8. SnO_2 and its nanocomposites as humidity sensors

The oxides of tin usually exist in two forms- tin (IV) oxide, generally known as stannic oxide (SnO_2) and tin (II) monoxide, generally known as stannous oxide (SnO). SnO_2 behaves as an n-type semiconductor with a wide energy band gap (~ 3.6 eV) and exhibits a tetragonal structure, similar to the rutile structure [170]. In contrast, SnO behaves as a p-type semiconductor having a direct and indirect bandgap of 2.5–3.0 eV and 0.5–0.7 eV, respectively. SnO is a metastable phase formed at the initial stage of oxidation of Sn, which converts to SnO_2 when exposed to higher temperatures [171,172].

Researchers have invented many techniques to yield SnO_2 nanostructures and thin films, including hydrothermal method [173], precipitation method [174], sol-gel technique [175], RF magnetron sputtering [176], spray pyrolysis [177], among others. SnO_2 exists in various nano-morphologies like nanorods, nanobelts, nano-whiskers, nanowires, nanoflowers, nanospheres, etc. [178–182]. SnO_2 nanostructures have advantages of high sensitivity, high chemical inertness, non-toxicity and long lifetime, which attracted the researchers to be utilized them as a material for humidity detection [183]. Furthermore, SnO_2 nanostructures exhibit high surface area, good reversibility, ease of fabrication and low production cost [184].

Many researchers have studied DFT calculations and confirmed the adsorption of a water molecule on the SnO_2 surface [185]. Lindan et al. [186] have studied water chemistry at the SnO_2(110) surface using DFT techniques. The authors claimed that besides the surface morphology, the surface geometry of SMOs also plays an essential role in controlling

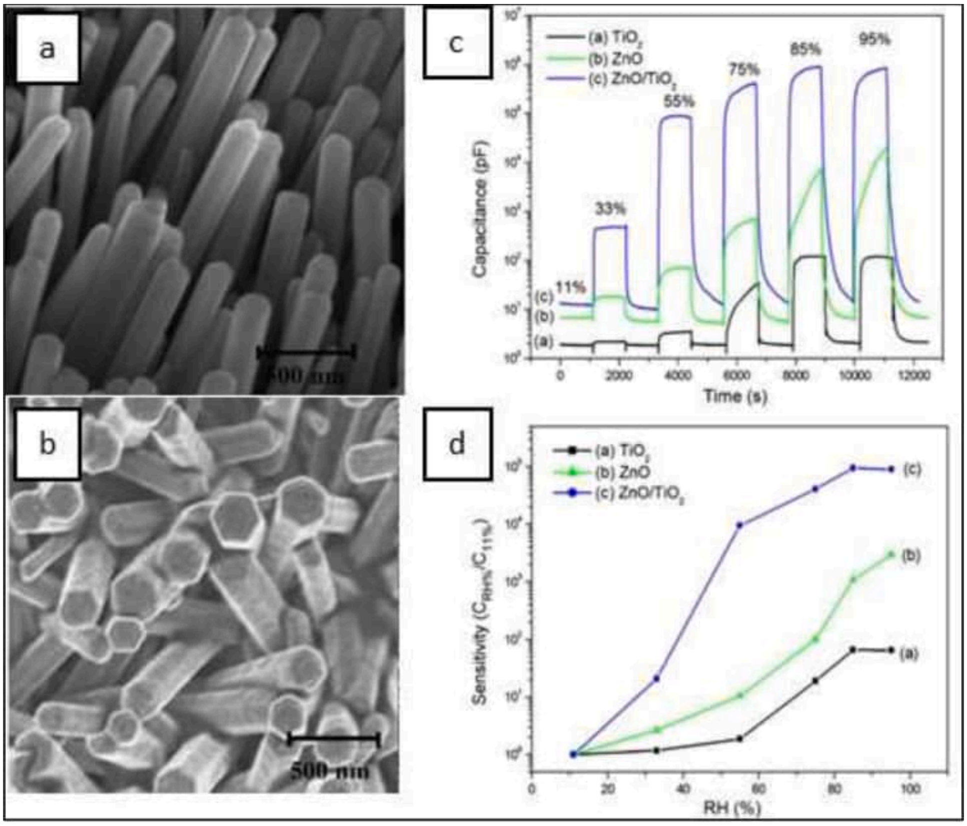

Fig. 33. SEM images of (a, b) ZnO nanorods and ZnO-TiO_2 composite, respectively, (c) capacitance of sensors for pristine TiO_2, pristine ZnO nanorod arrays and ZnO-TiO_2 composites at different humidity levels and (d) sensitivity as a function of % RH for different sensor types [163]. Copyright 2011 Elsevier.

Table 3
Sensing performances of TiO2 -modified humidity sensors.

S.No	Material	Type	Humidity range (%RH)	Method of synthesis	Response time (s)	Recovery time (s)	Remarks	Refs.
**Pure-TiO_2**								
1.	TiO_2 nanoparticle	Optical fibre	24-95	**Sol-gel**	0.01	0.06	Sensitivity of 27.1 mV/%RH, good reversibility and repeatability.	[164]
2.	TiO_2 nanosphere	QCM	0-97	**Hydrothermal**	6	3	Excellent repeatability and sensitivity of porous hollow ball-like TiO_2 nanostructures derived from Metal-organic frameworks.	[158]
3.	TiO_2-nanoflowers	Resistive	20-95	**Hydrothermal**	-	-	Sensor showed a high sensitivity of 485.7 /RH% along with comparably good repeatability, little hysteresis, and high degree of chemical stability.	[165]
**Doped-TiO_2**								
4.	Cu-doped TiO_2 film	QCM	30-70	**Sol-gel**	-	-	The Cu-doped in TiO_2 results in the enhancement of adsorption energy and charge-transfer values of the water molecule.	[35]
5.	Co-doped mesoporous TiO_2	Resistive	9-90	**Multicomponent self-assembly**	~24	~24	Increase in the sensor response with cobalt content.	[94]
6.	KCl-doped TiO_2 nanofibre	impedance	11-95	**Electrospinning and calcination**	~3	~3	KCl-doped TiO_2 nanofibers show high sensitivity, fast response/recovery time with good stability and reproducibility with mixed anatase and rutile structures.	[166]
7.	LiCl-doped TiO_2 nanofibre	Impedance	11-95	**Electrospinning and calcination**	3	7	Improved response and recovery time, good reproducibility with improved linearity and stability.	[167]
8.	Mg^{2+}/Na^{+}-doped TiO_2 nanofiber	Impedance	11-95	**Electrospinning and calcination**	2	1	Good reproducibility, fast response and recovery time.	[160]
9.	Ru-doped TiO_2 nanofibre	Quartz Crystal microbalance	20-70	**Electrospinning and calcination**	-	-	Ru-doped TiO_2 nanofibers show enhanced sensitivity due to more favourable charge-transfer between the adsorbed water molecules and the surface.	[151]
**TiO_2-Composite**								
10.	TIO_2-PVA	Optical Fibre	9-95	**Hydrothermal**	27	30	TiO_2-PVA composite showed pseudo-linear change with capacitance over 9-95%	[161]
11.	TiO_2-Polyaniline	Impedance	25-95	**Chemical polymerisation**	60	100	The composite has shown stable sensing ability over a period of one month.	[152]
12.	TiO_2-graphene	Impedance	12-90	**Sol-gel**	128	68	High sensing response, greater BET surface area of 169.5 m^2/g with negligible hysteresis of 0.39%	[168]
13.	TiO_2-polystyrene sulfonic sodium	Impedance	33-95	**Dip-coating**	80	20	High sensitivity, fast response/recovery time with low hysteresis.	[169]

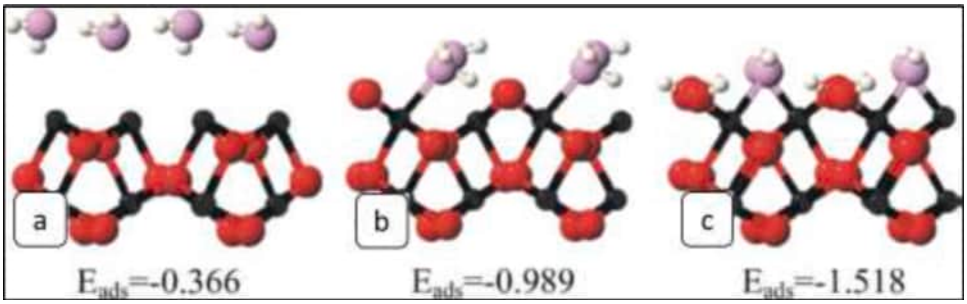

Fig. 34. Adsorption geometry for full monolayer water coverage for (a) reduced SnO_2(101) surface, (b) molecular adsorption and (c) dissociative adsorption on stoichiometric SnO_2(101) surface [187]. Copyright 2006 Elsevier.

water adsorption. They have suggested that inter-molecular interaction with surface sites creates a balance between molecular and dissociate adsorption and hence controls the water sorption on any surface. When compared their results with DFT calculation on the TiO_2 surface, they explained that H-bonding is less advantageous for water adsorption on SnO_2 due to the greater bond length of SnO_2 compared with TiO_2. Since the water molecules cannot be stabilised by H-bonding hence, dissociative adsorption on SnO_2 is dominant to form a monolayer coverage with an adsorption energy of 1.74 eV. In continuation, Batzill et al. [187] have studied DFT and photoemission calculations on stoichiometric and non-stoichiometric SnO_2(101) surface and concluded that dissociative adsorption is energetically favourable on stoichiometric surfaces while not possible on the reduced surface. While molecular water can adsorb on both reduced and stoichiometric surfaces but more strongly on the stoichiometric surface. The predictions are also confirmed by valence band photoemission studies and core-level spectra. The authors suggest that as the effects of initial water adsorption influences the band bending, water adsorption at defect sites can been studied by studying the band bending. Fig. 34 shows the adsorption geometries for full monolayer coverage with calculated adsorption energies.

Researchers have reported many articles based on SnO_2 nanostructures as humidity sensors in past few years. Li et al. [188] have proposed a resistive type humidity sensor based on ordered SnO_2 nanostructures prepared by nanosphere lithography with the magnetron sputtering technique. AFM image reveals that the SnO_2 nanostructures exhibit classical honeycomb structure, which provide a large surface area, as shown in Fig. 35(a). The hysteresis behavior of the humidity sensor is shown in Fig. 35(b). The sensor shows response and recovery time of 32s and 42s, respectively, for 11–96% RH with minimum hysteresis of <5%. The humidity sensing characteristics of the given sensor are shown in Fig. 35(c–e).

Qu et al. [189] have proposed impedance type humidity senor based on hierarchical SnO_2 twig-like microstructures prepared by a facile hydrothermal reaction. SEM image in Fig. 36(a and b) shows the hierarchical SnO_2 architectures are in a twiggy shape and the end of branches are bulging revealing dendritic growth. The impedance of the SnO_2 microstructures-based sensor exhibit excellent humidity sensing performance at room temperature. The humidity sensing response of the mentioned structure is shown in Fig. 36(c).

Parthibavarman et al. [190] have proposed a resistive type humidity sensor based on spherical nanoparticles of SnO_2 prepared by microwave irradiation method with an operating frequency of 2.45 GHz. The crystallite size was in the range of 20– 25 nm. The sensor showed super sensitivity within the %RH of 5–95%RH with low response and recovery time of 32 s and 25 s, respectively. The sensor showed nearly the same result after six months which concluded the excellent stability and reproducibility.

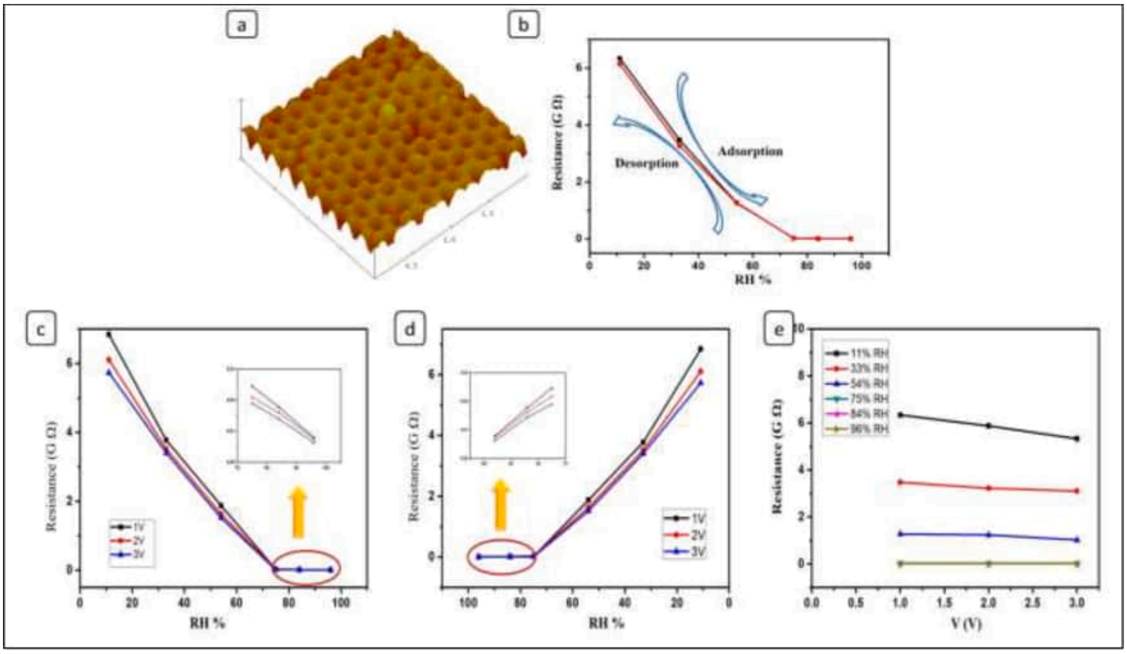

Fig. 35. (a) AFM image of SnO_2 nanostructure, (b) hysteresis curve, (c) change in resistance with variation in relative humidity from (c) low to high relative humidity (11% to 96%), (d) from high to low relative humidity (96% to 11%) and (e) the resistance -voltage curve at different %RH [188]. Copyright 2017 MDPI.

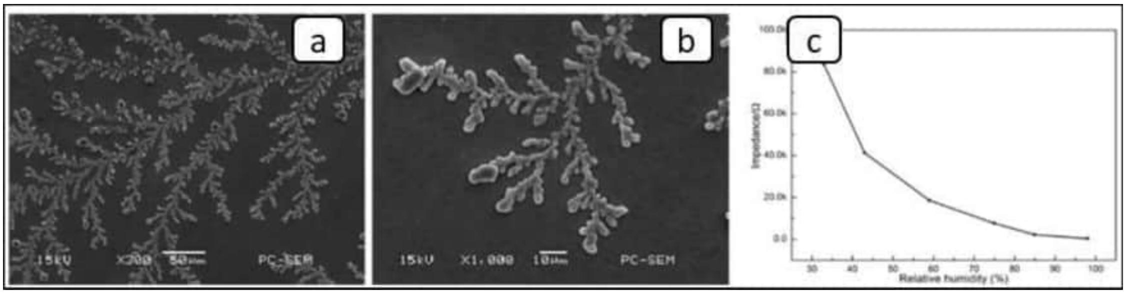

Fig. 36. (a) SEM images of SnO_2 microstructures, (b) at higher magnification, and (c) change in Impedance with a relative humidity of SnO_2-based sensor [189]. Copyright 2017 Elsevier.

Further continuing the work-doping elements, such as Li, Zn, Fe, Ni, Ag, etc., have been used to improve the humidity sensing characteristics, such as sensitivity, response/recovery time and reproducibility for SnO_2-based humidity sensors. Yin et al. [191] have proposed a resistive type humidity sensor based on 1-D Li-doped SnO_2 porous nanofibers synthesized by electrospinning. The optimized Li-doped SnO_2 porous nanofibers exhibited up to 15 times higher response (85% RH) as compared to pristine ones when tested at 5 V and room temperature. The reason behind the enhancement in response can be mainly attributed to the alkaline Li bonding interface to create a strong adhesion to water molecules, which can effectively induce a current change. Tomer et al. [192] have developed impedance type humidity sensor based on mesoporous Ag/SnO_2 synthesized by nano casting route using silica (SBA-15) as a hard template. HRTEM image revealed ordered 2D p6mm regular hexagonal mesostructures with a long range of pore channels, as shown in Fig. 37(a and b) by black and white lines. Using SBA-15 silica as a hard template has the advantages of getting a high surface area and wider pore channels in the SnO_2 nanostructure, which facilitates smoother movement of charge carriers across the SnO_2 surface. Furthermore, increase in the number of oxygen vacancies on the surface due to Ag dopants which provide more adsorption sites for water molecules on the surface. The response and recovery time was found to be 4s and 6.5 s, respectively. The humidity sensing response of Ag/SnO_2-based sensor at different operating frequencies is shown in Fig. 37(c) and the adsorption-desorption process at different humidity values is shown in Fig. 37(d).

Sabarilakshmi et al. [95] have proposed a resistive type humidity sensor based on W-doped SnO_2 nanostructure synthesized by hydrothermal method. TEM images of both pure and different wt% of W-doped SnO_2 nanoparticles show elongated spherical-shaped rod-like morphology with particle sizes ranging from 12 to 20 nm, as shown in Fig. 38(a–c). The 10 wt% W-doped sample exhibits high sensitivity and stability as compared to a pristine one. The average response and recovery time for pure SnO_2 were found to be 45s and 33s, respectively, while 38 s and 25 s, respectively, for the 10 wt% W-doped SnO_2 sample. The reason behind the increase in sensitivity is due to the increase in oxygen vacancies and surface-active sites resulting in an increase in the number of adsorptions of water molecules on the sensor surface. The comparison of humidity sensor characteristics of undoped and W-doped sensors is shown in Fig. 38(d and e).

Furthermore, researchers have reported enhancement in humidity sensitivity of SnO_2 by composite formation with other hygroscopic materials such as graphene oxide, polymers, CNTs, SMOs, among others. Zhang et al. [14] have proposed a capacitive type flexible humidity sensor based on SnO_2/rGO nanocomposite film deposited on polyimide substrate with integrated microelectrodes. SEM image in Fig. 39(a) shows the SnO_2 nanocrystals attached to the surface of rGO sheets. Fig. 39(b) plot shows the change in capacitance with change in %RH value of given sensor at different operating frequencies of 10, 40, 70 and 100 kHz, respectively. With an increase in %RH, the capacitance of the

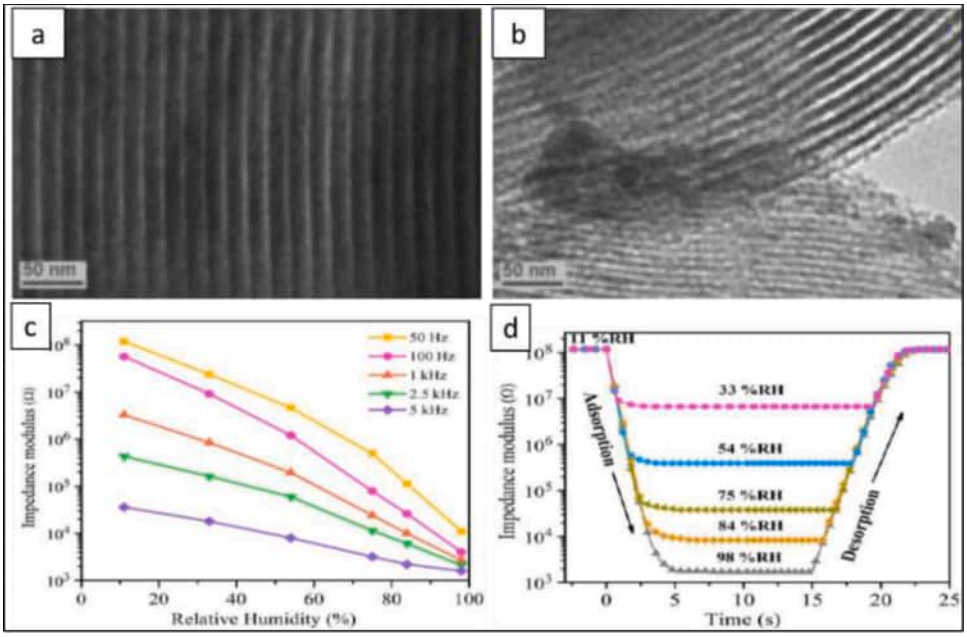

Fig. 37. (a, b) HRTEM image of Ag/SnO_2 showing uniform channels with long-range order, (c) change in impedance with %RH at various frequencies, (d) response and recovery time at humidity levels between 11 and 98%RH [192]. Copyright 2016 Elsevier.

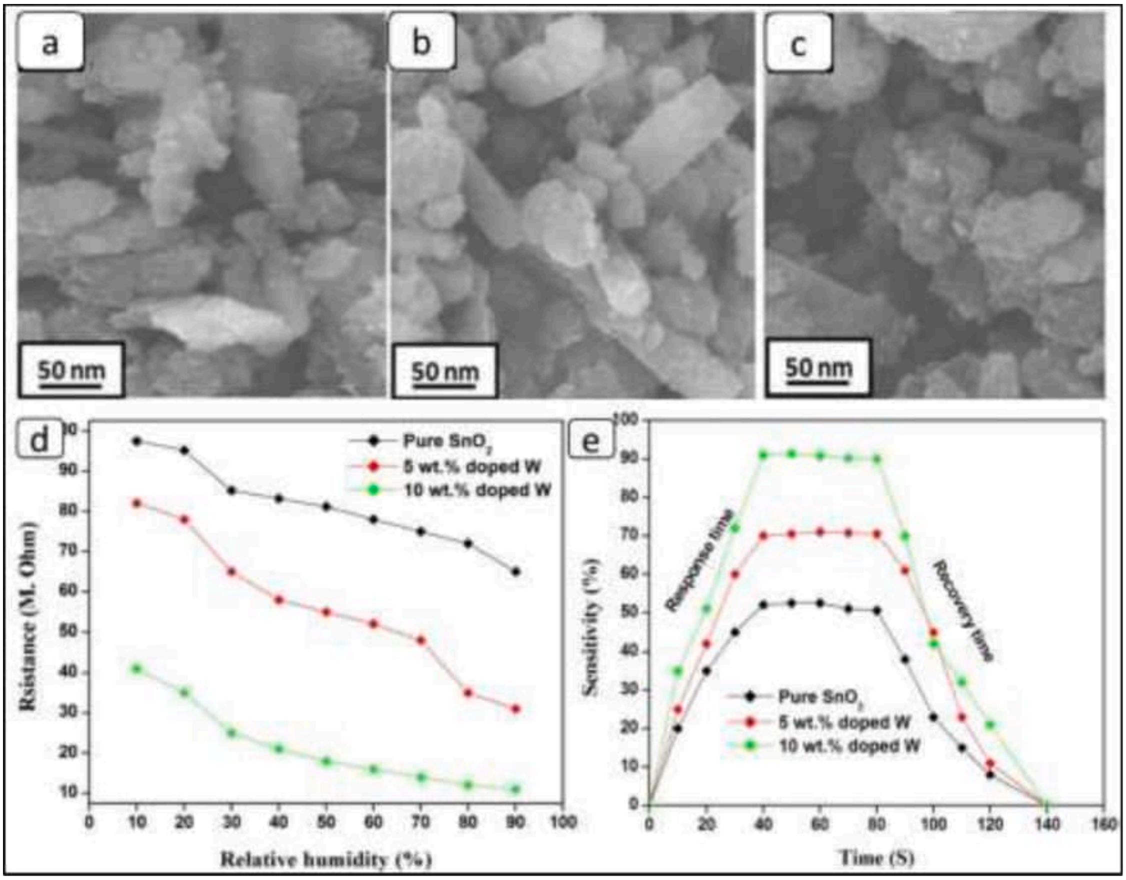

Fig. 38. (a–c) TEM images of pure SnO_2, 5 wt% W and 10 wt% W, respectively, (d) change in resistance with respect to %RH of pure and W-doped SnO_2 and (e) response/recovery time of pure and W-doped SnO_2 [95]. Copyright 2016 Springer.

sensor increases due to the adsorption of water molecules which strengthens the polarization effect and dielectric constant. The overall humidity characteristics, along with the response and recovery time of the sensor at different %RH changes, are shown in Fig. 39(b and c).

Su et al. [193] have proposed impedance type thin film humidity sensor based on Ag microwires, polypyrrole and SnO_2 ternary composite (Ag MWs/PPy/SnO_2) synthesized by one-step UV-irradiation photo-polymerization. High-magnification SEM image in Fig. 40(a–c) shows that the micro-wires with diameters (0.22 to 0.25 μm) were embedded and wrapped and more dispersed inside the Ag MWs/PPy/ternary composite. The humidity sensor based on Ag MWs (6 mg)/PPy (0.1 g)/SnO_2 ternary composite film exhibits high sensitivity, negligible hysteresis <1.8% RH, low ambient temperature coefficient, and improved response and recovery time of 46 s and 54 s respectively with long-term stability. The authors have also studied an important aspect of the synthesized humidity sensor, i.e. temperature dependency, although in a small range of temperature range of 15°C–35°C. It has been found that the impedance of thin film decreases with an increase in the temperature due to an increase in the mobility of the charge carrier by heat energy. The humidity sensor characteristics, along with temperature dependency, are shown in Fig. 40(d & e).

Zhang et al. [194] have proposed impedance and capacitive type humidity sensor based on MoS_2 modified SnO_2 nanocomposite synthesized by facile hydrothermal route. SEM images (Fig. 41(a and b)) show

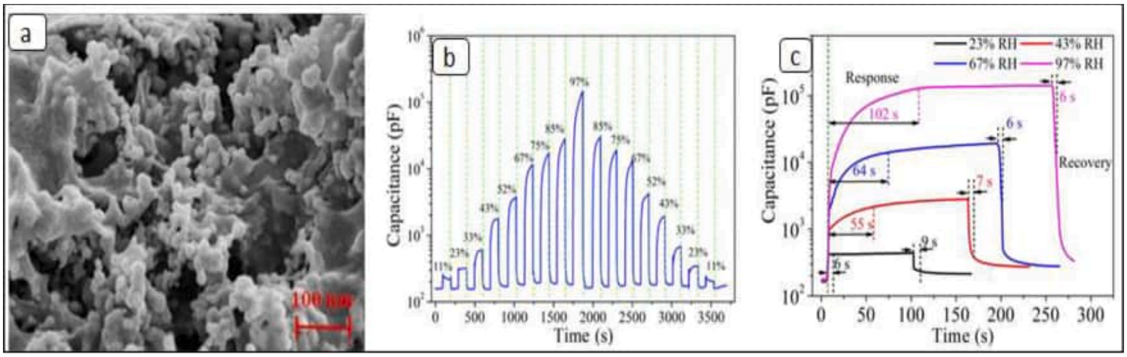

Fig. 39. (a) SEM image of SnO_2/rGO composite, (b) capacitance change of the SnO_2/rGO hybrid composite sensor under different %RH and (c) response and recovery curve at different %RH [14]. Copyright 2016 Elsevier.

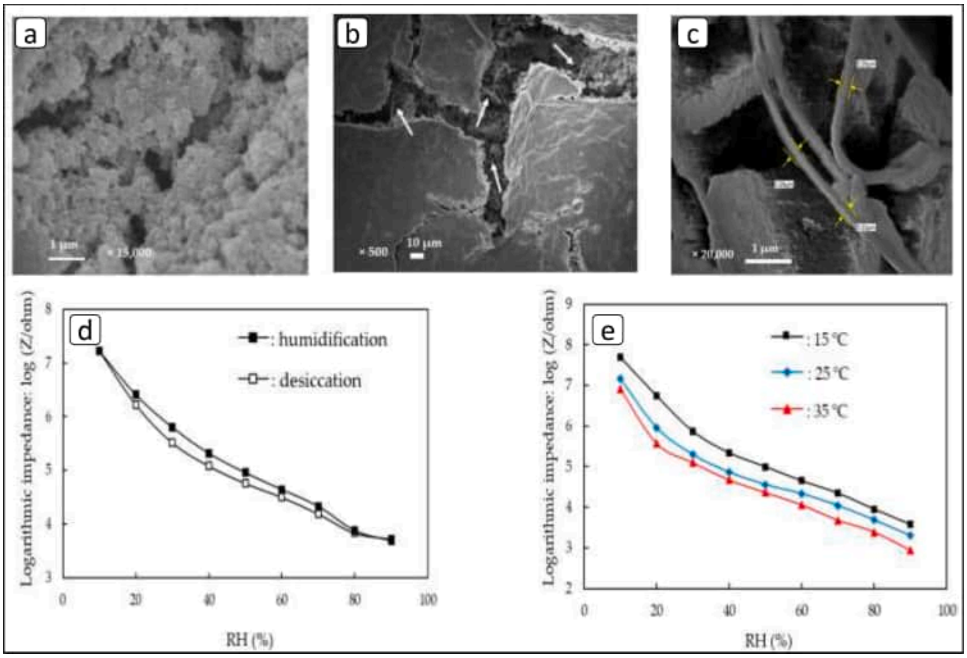

Fig. 40. (a–c) high- magnification SEM image of Ag/MWs/PPy/SnO_2 composite film, (d) hysteresis curve of the composite film sensor and (e) effect of temperature on the sensor response [193]. Copyright 2020 MDPI.

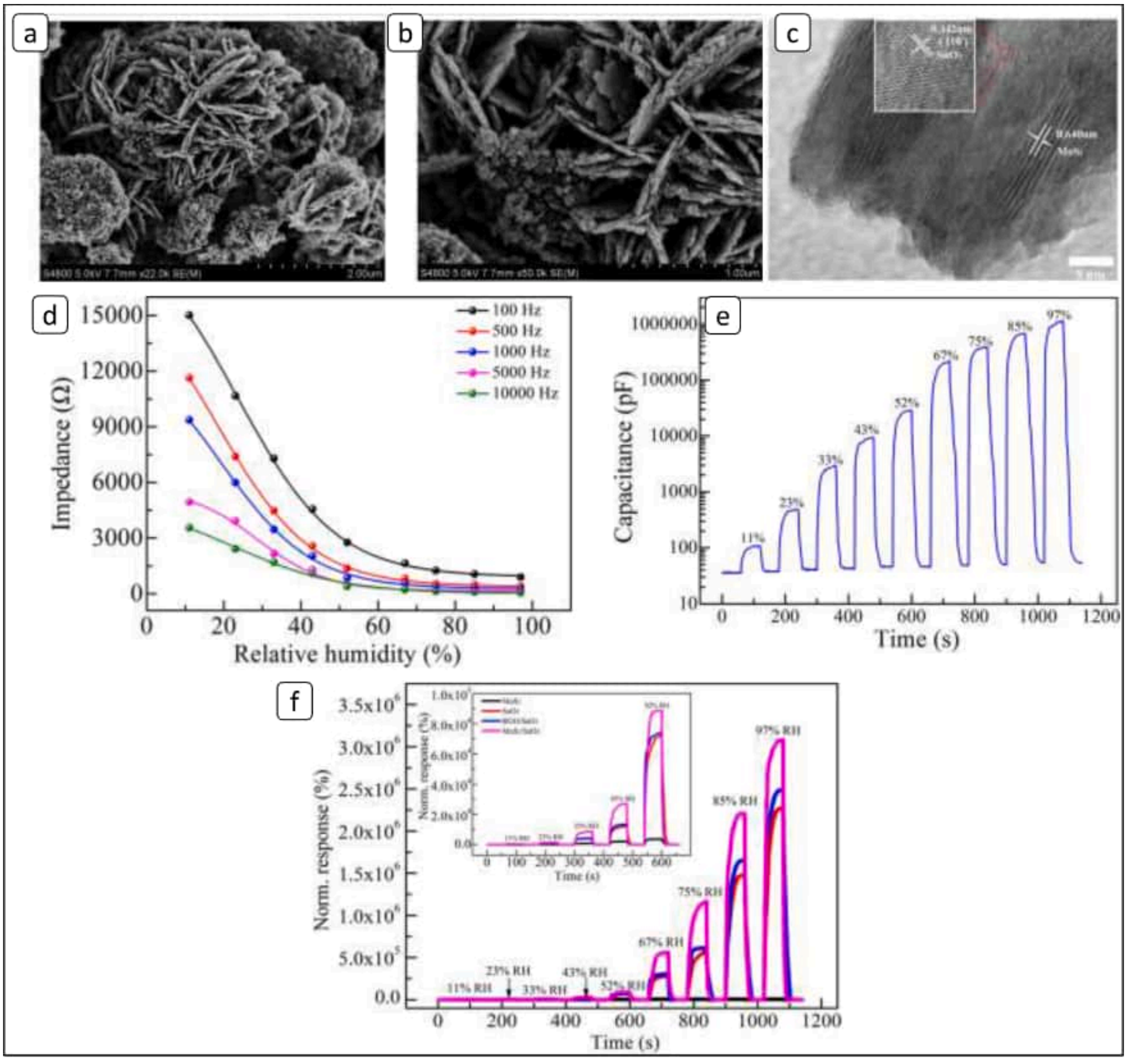

Fig. 41. (a and b) SEM images, (c) HRTEM image, (d) impedance as a function of relative humidity, (e) capacitance as a function of relative humidity; of MoS_2/SnO_2 and (f) humidity response of MoS_2/SnO_2 with pure MoS_2, pure SnO_2 and rGO/SnO_2 thin films [194]. Copyright 2016 American Chemical Society.

Table 4
Sensing performances of SnO_2 modified humidity sensors.

S. No	Material	Type	Humidity range (%RH)	Method of synthesis	Response time (Second)	Recovery time (second)	Remarks	Refs.
**Pure-SnO_2**								
1.	SnO_2 nanowires	QCM	11-97	**Spin Coating**	10	3	Low-cost humidity sensor with low power consumption.	[183]
2.	SnO_2 nanoparticles	Resistive	5-95	**Microwave-irradiation wet chemical**	32	25	Simple, effective and low-cost synthesis and highly sensitive	[190]
3.	SnO_2 dodecahedral nanocrystals	Impedance	11-95	**Hydrothermal**	4	13	Quick response/recovery time, low hysteresis, high sensitivity, great linearity and good stability.	[195]
**Doped-SnO_2**								
4.	Li-doped SnO_2 nanofibers	Resistive	33-85	**Electrospinning and calcination**	1	1	Li^+-doped SnO_2 nanofibers exhibited 15 times higher response than a pristine one.	[191]
5.	Ni-doped SnO_2 nanoparticles	Resistive	0-100	**Microwave-irradiation wet chemical**	54	84	Ni-SnO_2 sensors exhibit high sensitivity with quick detection.	[24]
6.	Sb-SnO_2 nanowire	Resistive	22-40	**Vapour liquid solid**	-	-	The sensor exhibits high sensitivity towards humidity.	[196]
7.	Ag-doped SnO_2 nanoparticles	Impedance	11-98	**Hydrothermal**	4	6.5	Exhibits fast response and recovery time, good linearity, low hysteresis, high stability and selectivity.	[192]
8.	KCl-doped SnO_2 nanofibers	Impedance	11-95	**Electrospinning and calcination**	5	6	High sensitivity, rapid response/recovery time with good linearity and long-term stability.	[197]
**SnO_2-Composite**								
9.	SnO_2-graphene nanocomposite film	Capacitance	11-97	**Hydrothermal**	102	-	Nanocomposite exhibited high sensitivity, rapid response and recovery times and good long-term stability.	[14]
10.	SnO_2-polyaniline	Resistive	5-95	***In-situ* Polymerization**	26	30	High sensitivity (0.22% RH^{-1}), good linearity and quick response.	[198]
11.	SnO_2-Chitosan Hybrid Nanowires	Resistive	25-75	**Physical Vapour deposition**	-	-	Exhibit better sensing performance under UV light radiation.	[199]
12.	SnO_2-MoS_2 nano-composite	Surface Acoustic wave	10-90	**Hydrothermal**			The sensor exhibits excellent linearity, high sensitivity (0.78 kHz/%RH), and low hysteresis with good repeatability and long-term stability.	[200]
13.	SnO_2-WS_2 nano-composite	Resistive	11-95	**Hydrothermal**	-	50	SnO_2/WS_2 nano-composite was found to be 8.5 and 862.8 times higher than that of pristine SnO_2 and WS_2 nanosheets respectively.	[201]
14.	SnO_2-ZnO nanoparticle film	Resistive	40-90	**Spin coating**	-	-	Enhancement in surface area due to composite formation due to a decrease in grain size and crystallite size.	[202]

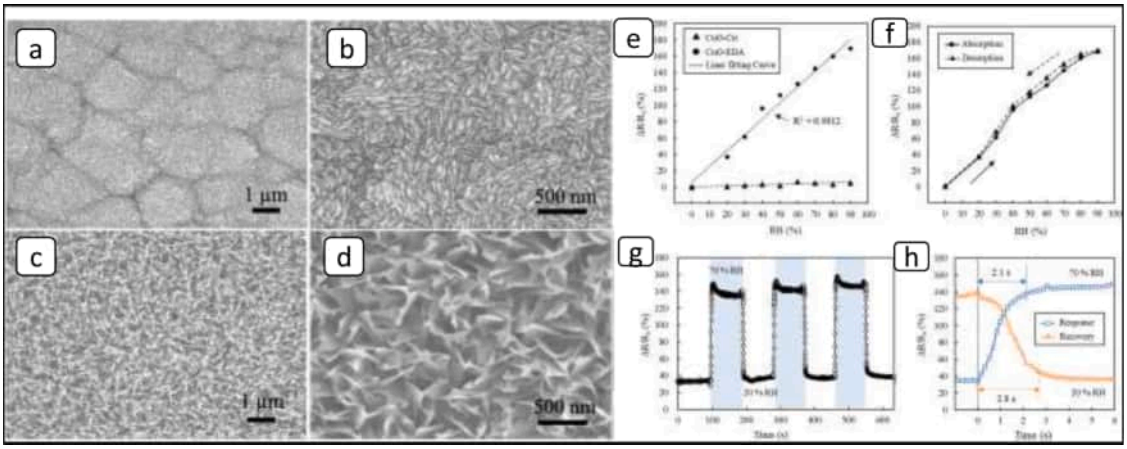

Fig. 42. FESEM image of (a and b) CuO-Cit, (c and d) CuO-EDA thin films, (e) humidity sensing response of CuO-Cit and CuO-EDA thin films, (f) hysteresis curve of CuO-EDA, (g and h) repeatability and response/recovery graph of CuO-EDA, respectively [220]. Copyright 2022 Elsevier.

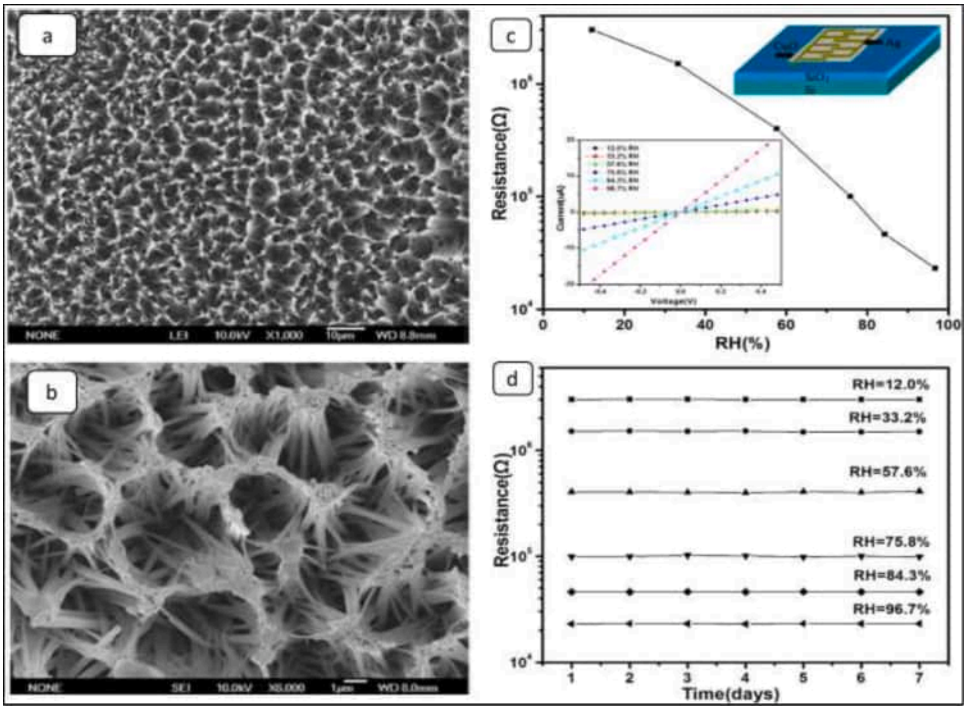

Fig. 43. SEM images of CuO honeycombs synthesized by two-step electrochemical deposition at different voltages for 4 h: (*a*), (*b*) $E = 5$V, (*c*) change in resistance with %RH plot at room temperature and (d) resistance value stability various %RH levels [224]. Copyright 2009 IOPscience.

that MoS_2/SnO_2 hybrid constructed by the MoS_2 nanosheets uniformly decorated with SnO_2 nanocrystals. Lattice fringe spacing for the composite is found to be 0.342 nm as determined using HRTEM image (Fig. 41(c)), which is attributed to the (110) plane of the SnO_2 nanocrystals. The impedance and capacitance change with %RH are shown in Fig. 41(d and e). The MoS_2/SnO_2 hybrid nanocomposite-based humidity sensor exhibited excellent sensing properties, with a low response/recovery time of less than 5/13s and acceptable repeatability. The report suggested that tailoring the SnO_2 nanostructure by MoS_2 can significantly improve the humidity-sending properties. The humidity-sensing responses of the MoS_2/SnO_2 film sensor along with pure MoS_2, pure SnO_2, and rGO/ SnO_2, are shown in Fig. 41(f).

With further study of SnO_2 and its nanocomposites, the summary of SnO_2-modified humidity sensors with sensor characteristics is shown in Table 4.

9. CuO and its nanocomposites as humidity sensors

CuO is a p-type semiconductor having a monoclinic structure with a narrow indirect bandgap of 1.2 eV [203,204]. The p-character is usually due to the presence of intrinsic Cu vacancies [205]. In the past few years, researchers have reported several techniques to produce copper oxide nanostructures and thin films, including hydrothermal synthesis [206], microwave irradiation [18], sol-gel [207], spray pyrolysis [208], electrodeposition [209], sputtering [210], among others. CuO has been synthesized in various nano-morphologies such as nanowires [211], nanorods [212], nenobelts [213], nanowhiskers [214], nanocubes [215], nanospheres [216], etc. Researchers have utilized CuO nanostructures for several applications, including solar cells, photocatalysis, electrodes in lithium-ion batteries, gas and humidity sensors, among others [217–219].

In literature, we found that humidity sensing studies of p-type semiconductor materials are scarce. Most of the reports are on n-type semiconductors such as SnO_2, TiO_2, ZnO, etc. CuO has been considered to be used as humidity sensor because of its high surface activity, oxygen adsorption capability and good adhesion capability with flexible substrate, which makes it suitable for device applications [220]. In the present review, we have elaborated on the work carried out on CuO-based humidity sensors with various nanostructures. CuO (111) surface is the most stable surface and hence widely chosen to investigate the surface phenomena [221]. However, to understand the adsorption of water molecules on the CuO surface at the molecular level, very few reports are available in the literature. Yu et al. [222] have performed DFT calculations for the most stable CuO (111) surface. They have found that on a clean CuO (111) surface, there is molecular well as dissociative desorption of H_2O molecule with an adsorption energy of 0.71 eV and 0.69 eV, respectively. Since there is a very small difference between both molecular adsorption and dissociated adsorption energies, both adsorptions can coexist on CuO (111) surface. For one H_2O molecule, molecular H_2O adsorption is preferred while for two, three and four H_2O molecules, co-adsorption is preferred for mixed (undissociated and dissociated). The surface termination plays a key role in determining the interaction of water molecules with any material. Zhang et al. [223] studied the DFT calculations of adsorption of water molecules on different CuO(111) surfaces, including stoichiometric, oxygen-vacancy and oxygen-rich surfaces. The authors have taken various parameters into consideration to study the adsorption of H_2O on these three surfaces, such as bond lengths, charge transfer energy, surface adsorption sites, etc. They suggested that chemisorbed H_2O prefers to dissociate into OH and H species rather than dehydrogenation. When compared their results with the oxygen vacancy and oxygen-rich surface, it is found that the water dissociation into OH^- and H^+ is still more favourable on the oxygen-rich CuO (111) surface. Thermodynamically, it is found that the formation of OH species on stoichiometric surface is endothermic in nature while the dissociative adsorption on oxygen-vacancy and oxygen-rich surfaces is exothermic with a higher energy by oxygen-rich surface. It is revealed that the dissociation barrier of H_2O is smaller on oxygen vacancy surface compared to stoichiometric surface suggesting oxygen vacancy to exhibit strong water dissociation. Also, oxygen rich atom promotes the formation of OH^- by first dehydrogenation of H_2O (thermodynamically and kinetically). Different factors favour different processes towards the adsorption of H_2O on the

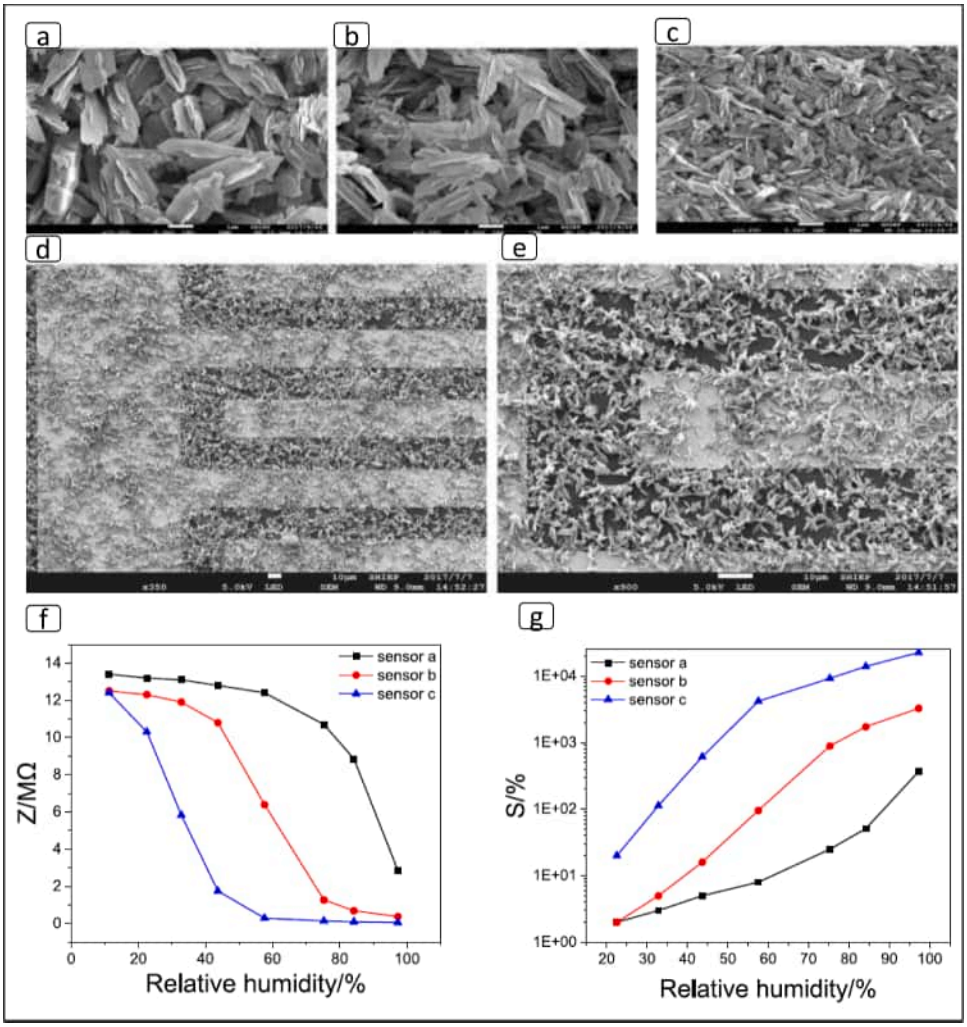

Fig. 44. (a–c) SEM images of CuO nanosheets synthesized by hydrothermal method with different NaOH concentrations of 0.5, 0.125 and 0.05 mol/l, respectively, (d, e) SEM images of deposited material on the IDEs, (f) impedance changes of different sizes of CuO nanosheets at various %RH and (g) sensitivity of different sizes of CuO nanosheets at various %RH [225]. Copyright 2019 Springer.

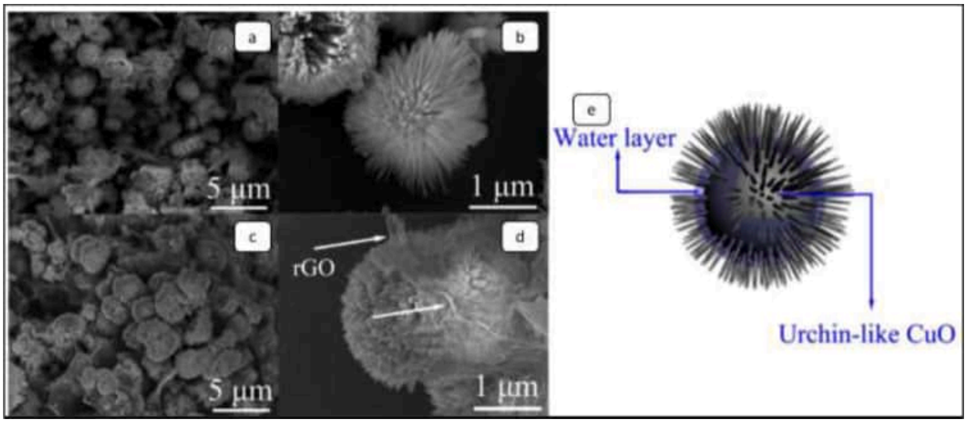

Fig. 45. SEM images of (a, b) CuO, (c, d) CuO/rGO composites and (e) pictorial Diagram of the Capillary Condensation Phenomenon on the Surface of Urchinlike CuO [226]. Copyright 2014 American Chemical Society.

CuO (111) surface. Thus the adsorption phenomenon exhibits a rich surface physics and needs to be further analyzed.

Recently, Nitta et al. [220] proposed a resistive type humidity sensor based on CuO nanostructures deposited on PET substrate by spin spray method using trisodium citrate and ethylene diamine as complexing agents referred as CuO-Cit and CuO-EDA, respectively. The SEM images of CuO-Cit show (Fig. 42(a and b)) tiny multi-layered nanosheets which are aggregated to each other to form dense film, while CuO-EDA (Fig. 42 (c and d)) shows nanosheets located separately like grass-like nanosheets. In Fig. 42(e), the CuO-Cit shows a negligible response to %RH

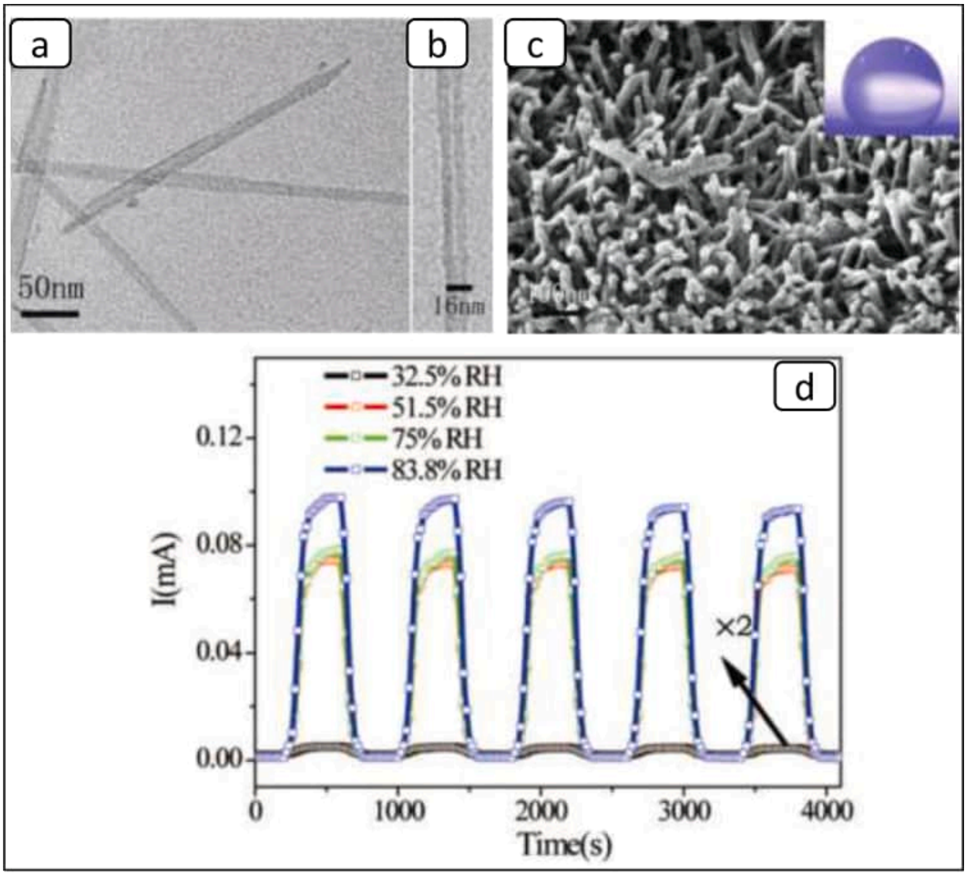

Fig. 46. TEM image of (a) P(O-B-EG-B)/CuO hollow nanowires, (b) single hollow nanowire (high magnification), (c) typical SEM image of hollow hybrid nanowires; inset show the shape of water droplets on the surface of a substrate, (d) dynamic current response of the sensor between dry air (5%) and different RH (32.5–83.8%) at 30 °C [227]. Copyright 2010 IOPscience.

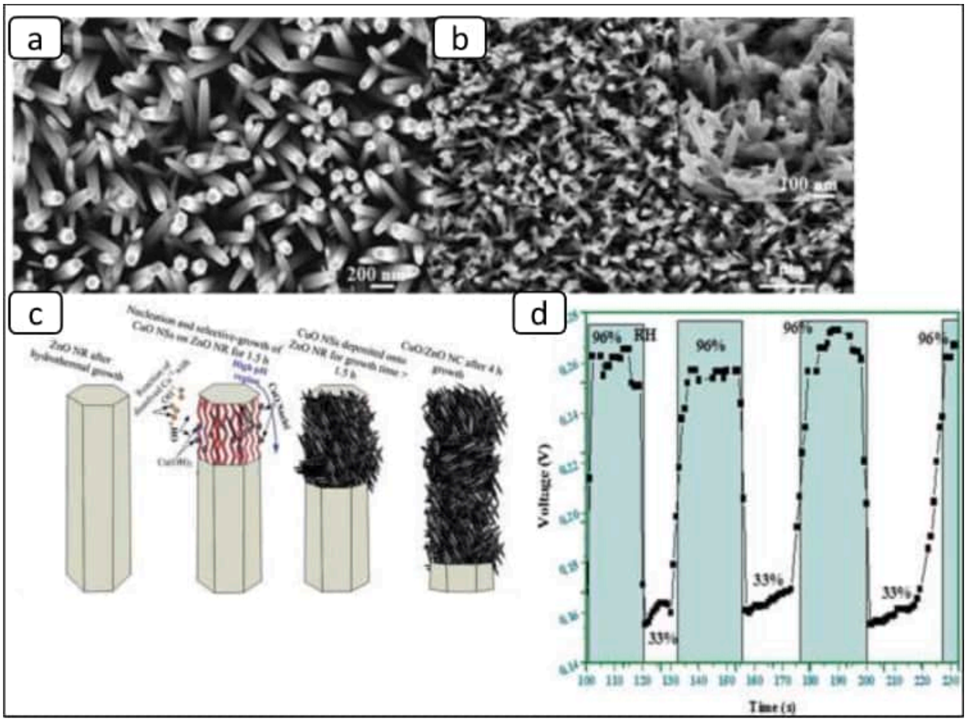

Fig. 47. SEM images of (a) ZnO Nanorods with a length of around 1.5 mm and a diameter of around 80–120 nm, (b) CuO nanostructures grown on ZnO nanorods showing the growth of CuO nanostructures on the sidewalls of ZnO nanorods, (c) schematic diagram of the growth steps of CuO/ZnO nano coral structures, and (d) dynamic humidity response of sensor at low and high humidity levels [228]. Copyright 2012 Royal Society of Chemistry.

compared to CuO-EDA. The result indicates that surface morphology plays a significant role in humidity sensing. The humidity sensing characteristics of the CuO-EDA sensor are shown the Fig. 42(f–h). The unusual behavior of resistance with an increase in %RH is observed as compared to n-type SMOs, which the authors have attributed to decrease in hole concentration of CuO occurring because of the release of trapped electrons on adsorption of water molecules. The CuO-EDA shows excellent humidity-sensing performance with high sensitivity, good repeatability and a fast response/recovery time of 2.1 s and 2.8 s, respectively.

Towards the progress of undoped CuO as a humidity sensor, Xu et al. [224] have synthesized resistive type humidity sensor based on undoped CuO honeycomb nanowires via two-step electrochemical deposition. SEM images in Fig. 43(a and b) show a honeycomb structure comprised of several CuO nanowires that provide a large surface-to-volume ratio. Furthermore, its rough surface morphology and presence of pore structures are responsible for a large number of adsorptions of water molecules. The humidity sensing response of the sensor is shown in Fig. 43(c and d). The resistance of the film is shown to be decreased with increasing %RH in an almost linear fashion with approximately two orders of magnitude (10^6–10^4) over the range of 12–97%RH.

In another recent report on undoped CuO-based humidity sensors, Gu et al. [225] studied the impact of size on the humidity sensing property of CuO nanosheets synthesized by the hydrothermal method. SEM image of CuO nanosheets with different NaOH solution concentrations is shown in Fig. 44(a, b and c) respectively for the different sizes. With the decrease in the concentration of NaOH, the size of the CuO nanosheets decreases, which was labelled as sample 1, sample 2 and sample 3, respectively. SEM images of the sample between the Ti/Au integrated electrodes are shown in Fig. 44(d and e). The humidity sensing results showed that with the decrease in size of CuO nanosheets, sensitivity increased as the surface-to-volume ratio increased. With the decrease in size, the sensitivity of sensors comes out to be 369%, 3278%, 22,611% at 97.3% RH, respectively, and the response time under 11.3–97.3%RH were 53s, 49s, and 32s, respectively. The humidity sensing response of different CuO nanosheets is shown in Fig. 44(f and g).

Wang et al. [226] synthesized a humidity sensor based on CuO/rGO composite with an Urchin-like CuO structure. The urchin-like structure of CuO was confirmed by SEM image (Fig. 45(a, b)). Morphological studies suggest that the rGO and CuO connect well with each other, as shown in Fig. 45(c, d). The sensor fabricated with the composite shows relatively good humidity sensing characteristics like response/recovery time as compared with the pristine one. The authors explain the capillary condensation at a lower %RH level which may be caused by the pores formed in the gap of the spines on urchinlike CuO, which facilitates a higher impedance response for humidity-sensing. A schematic picture of the capillary condensation phenomenon is shown in Fig. 45 (e). The increase in the impedance has been explained with the help of the Schottky junction formed at the interface of CuO and rGO nanostructures.

Yuan et al. [227] introduced a simple route to synthesize polyethene glycol di-butenetrioate (O-B-EG-B)–CuO hybrid nanowires with CuO shells. Here, O-B-EG-B was bound around CuO or entrapped in interior CuO. TEM images of the hybrid and single nanowire-based structures are shown in Fig. 46(a and b). SEM image suggests hollow nanowires are upstanding and get separated from each other due to the hydrophilic shells constructed from polar PEG segments (Fig. 46(c)). The sensor showed high sensitivity towards the %RH. A change in resistance by about two orders of magnitude is observed for the humidity range from 5% to 83.8%, as revealed by Fig. 46(d). Moreover, a humidity sensor based on this type of nanowire showed not only long-term stability but also exhibited excellent reversibility to moisture changes in the air. The sensor was kept at a moisture level of 83.8%RH for about one month, even after that, the sensor recovered its original resistance when it came to air with 32.5%RH. This result confirms that the humidity sensitivity of these hollow hybrid nanowires is mainly due to the physical interaction rather than the chemical interaction between vapour and hybrid wires.

Nanocoral structures of p-type CuO/n-type ZnO were grown by hydrothermal approach by Zainelabdin et al. [228], where the CuO was grown on pre-deposited ZnO nanorods. SEM images of ZnO nanorods and CuO/ZnO nanostructures are shown in Fig 47(a and b), respectively. Different steps in the growth of nano coral structure of CuO/ZnO is shown in Fig. 47(c), which explains the effect of acidic condition on the ZnO nanorods and the hydroxylation–dehydroxylation processes. The nano coral humidity sensor is found to show a dramatic increase in the sensitivity factor and response and recovery time of 6s and 7s, respectively, as elaborated in Fig. 47(d).

These results show that p-type CuO nanostructures can also be a good candidate for the humidity sensor, although their sensing mechanism is quite intriguing. Moreover, they have not been exploited much for humidity sensing applications. This provides room for researchers to develop and investigate p-type CuO-based nanocomposites for humidity sensing. Some of the work reported on CuO-based humidity sensors are summarized below in Table 5:

Table 5
Sensing performances of CuO modified humidity sensors.

S. No.	Material	Type	Humidity range (%RH)	Method of synthesis	Response time (Second)	Recovery time(second)	Remarks	Refs.
1.	CuO nanowire	Impedance	20-90	**DC sputtering**	-	-	Samples with a larger film thickness and a longer average CuO nanowire length show a high sensing response.	[47]
2.	CuO-rGO	Impedance	11-98	**Microwave-assisted hydrothermal**	2	17	Sensors exhibited relatively good humidity-sensing performance with fast response time and response time.	[226]
3.	CuO-ZnO Nanocorals	Resistive	30-90	**Hydrothermal**	6	7	The sensor shows a high sensing response to humidity variations.	[228]
4.	$CuO-TiO_2$ nanocomposite	Resistive	10-98	**Microwave-assisted hydrothermal**	162	428	Better sensitivity over relative humidity ranges from 10 to 98%.	[229]
5.	CuO- polyethylene oxide/ MWCNTs	Resistive	30-90	**Electrospinning**	3	22	High stability, flexibility and versatility for several industrial applications	[230]
6.	CuO-polyaniline	Resistive	5-75	**Chemical polymerisation**	~30	~30	Better sensitivity, linearity, and quicker response time.	[231]
7.	CuO-Polyaniline	Resistive	10-95	**Chemical oxidative polymerization**	40	55	4.5 ohm/%RHsensitivity.	[98]

10. Overview of SMOs humidity sensors performance

This section summarises the comparison between different SMOs discussed in this review paper. Nanostructured SMOs are able to detect relative humidity in a vast range of values between 0 and 100 %RH, with each individual having its own merits and demerits.

ZnO presents a good detection range humidity sensor; however, it has the main disadvantage of being hydrophobic in nature. The water contact angle of ZnO surfaces comes out to be more than 90° suggesting its hydrophobic nature, which results in its less sensitivity towards the water molecules [232], however, its hydrophobic nature can be improved by making its composites with hydrophilic materials like polymers, GO, rGO, etc. as discussed in earlier section.

On the other hand, TiO_2 is highly hydrophilic due to the intrinsic presence of Ti^{3+} defect sites or oxygen vacancies, at which adsorption of water molecules takes place. The water contact angle of the TiO_2 film surface is $\leq 20°$, which shows its superior hydrophilic nature [233]. TiO_2-based humidity sensors usually have a low surface-to-volume ratio which results in limited sensitivity, high resistance or poor charge transfer process of single phased TiO_2 material [168]. These limitations may be overcome either by modifying its surface structure and morphology or by composite formation with conducting materials like graphene, polymer, GO, etc. Many research articles have been proposed based on ZnO/TiO_2 nanocomposites in order to develop humidity sensor with superior sensor characteristics.

SnO_2-based nanostructures have been attracting more interest due to their high chemical stability, wide operating temperature range, non-toxicity and low cost. Pristine SnO_2 is hydrophilic in nature, with the water contact angle in a range of 11-48°, which makes it selective for water molecules [234,235]. The main limiting factor for SnO_2 to be exploited as a humidity sensor in practical applications is its very high resistance at lower %RH, which does not change abruptly at higher %RH values. Besides this, SnO_2-based humidity sensors mostly require high operating temperatures [14]. As discussed in the above section, these limitations can be overcome by preparing the composites of SnO_2 with the conducting polymers (PANI), graphene, GO, rGO, metal oxides, etc.

It is found that there is comparatively less literature available on p-type semiconductors to be studied as humidity sensors. Among p-type semiconductors, CuO has been considered to be used as humidity sensor because of its high surface activity, oxygen adsorption capability, hydrophilicity and good adhesion capability with flexible substrate, which makes it suitable for device applications [220,236,237]. The electronic conductance in p-type semiconductors decreases with an increase in the %RH due to a decrease in the concentration of holes due to water adsorption. But the ionic conductance follows the same trends as n-type semiconductors, where protons are the charge carriers. As the electronic and ionic conductance show reverse contribution to the conductivity when exposed to the water molecules and hence the resistance of CuO-based nanostructures have been found to increase/decrease depending upon the dominant conduction mechanism [220,238–240]. Thus, in p-type semiconductors, the resultant change in conductivity is limited due to the involvement of two reverse conduction mechanism.

It is to be noted that these SMOs are also prone to various gases present in the atmosphere. Through proper optimization of morphology, structure and surface properties by synthesis process, doping or composites formations, one can reduce the selectivity of these SMOs towards these gases while improving their limitations and hence allow them to perform as more reliable humidity sensor.

Overall, due to their various advantages, the SMOs based humidity sensors are widely used in diversified sectors. The high mechanical strength, resist to chemical attack, long-term stability, and thermal stability allow them to be used in areas with harsh conditions, such as textile industries, automobiles, meteorology, etc. and are generally used in agriculture, food industry, medical, pharmaceutical, etc. which demands continuous monitoring of relative humidity in the presence of foreign molecules at different temperatures. Areas such as production industries, thermal plants, furnaces, ovens, etc. require humidity to be monitored at elevated temperatures (>200°C). At these temperatures, polymers and carbon materials are chemically unstable and hence, SMOs offer the ultimate choice to operate as humidity sensors in these areas. Furthermore, the non-toxic nature adds a new dimension to SMOs for their use in wearable devices for human breath analysis which has been discussed in the next section.

11. SMOs for breath monitoring

Recently researchers have emphasized the respiration condition as a tool for disease monitoring and clinical diagnostics. This is a non-

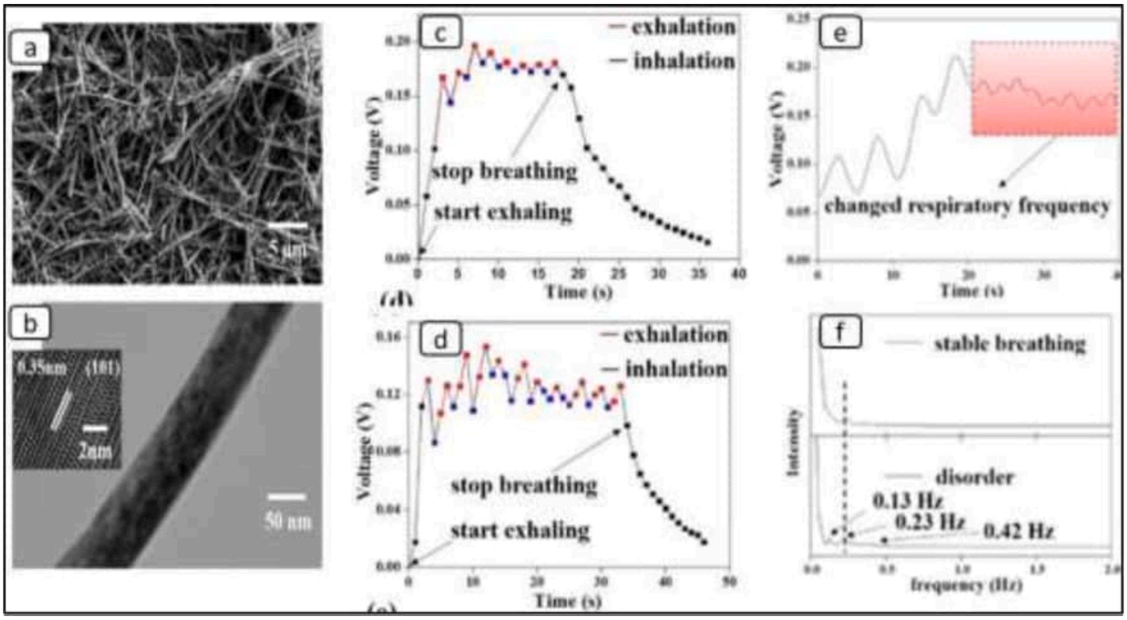

Fig. 48. Surface structure of TiO_2 nanowire network (a) SEM image, (b) TEM image showing a lattice spacing of 0.35 nm corresponding to (101) plan of anatase TiO_2, (c, d) voltage response of the sensor at different nasal breath frequencies (e) Change in the respiratory frequency (f) emergence of an extra peak in the frequency-domain diagrams in case of a respiratory disorder [241]. Copyright 2019 IOPscience.

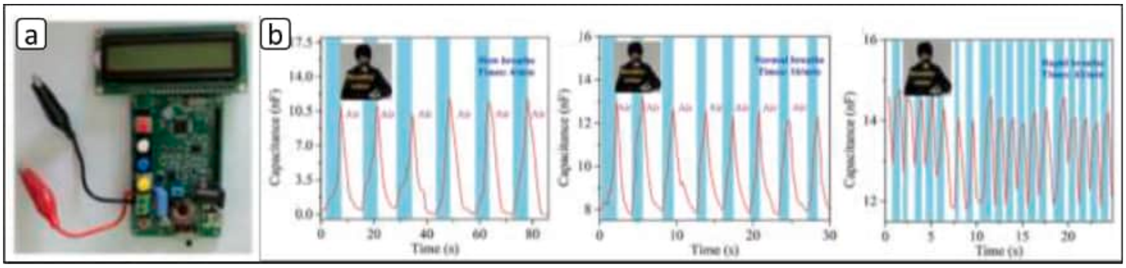

Fig. 49. (a) The image of the portable sensing device and (b) the capacitive response under different respiratory rates [242]. Copyright 2021 Royal Society of Chemistry.

invasive, painless and cost-effective technique and rapidly provides information about the disease at the early stage. The main indicators of respiration, i.e., breath rate, rhythm, volume and gas components, are informative for health, including physical/mental activity and emotions of living subjects. Skin moisture is also an important parameter in judging human health. Smart humidity sensors-based devices with high stretchability, fast response/ recovery times, skin compatibility, and self-powered are some features that have to look for in wearable medical applications. Recent work by Xiao et al. [241] has reported self-powered, mechanically flexible TiO_2 nanowire networks synthesized using a low-cost electrophoresis method for monitoring human breath. SEM image (Fig. 48(a)) suggests the nanowires are spread on the ITO substrate to form a layered network structure. TEM image (Fig. 48 (b)) reveals the lattice spacing for nanowires to be 0.35nm, corresponding to the (101) plane of anatase structure. The structure of nanowire networks is highly flexible and shows very good repeatability even after 10,000 times bending. The sensor exhibits a response time of ~ 3.6 s and recovery times of ~ 14 s with a very high sensitivity ability. The device can generate a voltage when exposed to a moist environment due to water vapour diffusion along the nano-channels in TiO_2 nanowire networks.

For real-time breath monitoring, the structure was integrated with a microcontroller unit and an analogue-to-digital converter to realize the remote control function. The sensor can detect the humidity changes between inhalation and exhalation and generate voltage signals that follow the frequency of human breath (as shown in Fig. 48(c and d) without needing an external power source. The inhalation makes the voltage signal drop, while the exhalation increases the voltage. With an FFT analysis, authors have shown that when the tester's breathing is abnormal, an anomaly appears in the pattern of the peaks, which can be observed directly from the frequency domain image. A comparison between normal respiration and the case of any respiratory disorders in the breathing pattern is shown in Fig. 48(e and f). Thus, the number of peaks in the frequency domain is a direct indication of human health. This gives a quick and easy detection method of human health disorders and further monitoring. In recent work, Gong et al. [242] have proposed a portable Cerium oxide/graphitic carbon nitride nanocomposites (CeO_2/g-C_3N_4) based multifunctional self-powered capacitive type wearable humidity sensor for real-time respiratory detection and skin dryness monitoring. The sensor could communicate with the mobile smartphone through wireless communication technologies and can be worn on the forehead, neck, arms or knees. The photograph of the fabricated device and capacitance response under different respiratory rates are shown in Fig. 49.

In another paper, Zhang et al. [243] reported a high-performance flexible tin disulphide nanoflowers/reduced graphene oxide (SnS_2/rGO) nanohybrid thin film structure screen printed on PET substrate and demonstrated the potential ability to monitor human breath with different frequency, human cough, and finger approach in various applications for humidity sensing. Pang et al. [244] have improved the humidity performance by introducing poly (3, 4-ethylenedioxythiophene)-poly(styrenesulfonate) (PEDOT: PSS) and Ag colloids to the graphene. The sample exhibited excellent capability of monitoring different breathing patterns, including normal and deep respiration. In a similar work, Wang et al. [245] proposed a self-powered flexible humidity sensing device based on poly (vinyl alcohol)/$Ti_3C_2T_x$ (PVA/MXene) nanofibers film deposited on a flexible PET substrate and driven by the monolayer MoS_2 Piezoelectric nanogenerator (PENG) which converts mechanical energy to electrical energy. The fast response/recovery time makes it suitable to detect human breathing rate. Also, the flexible humidity sensor driven by PENG exhibits excellent performance in detecting human skin surface moisture. Liu et al. [21] have proposed QCM based chitosan/polypyrrole composite humidity sensor for respiratory monitoring system combined with an app to accurately determine normal and abnormal breathing and demonstrate the potential respiratory monitoring of patients. The sensor could quickly and accurately detect the change in the respiration rate, depth, and rhythm. Based on the waveform generation, the sensor can judge the respiration type and

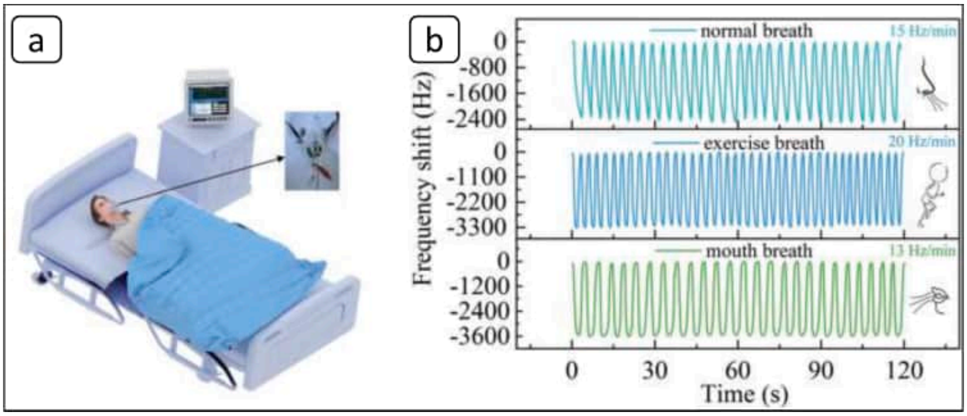

Fig. 50. (a) Schematic diagram of clinical respiratory monitoring, (b) Breathing test results in normal, exercise and mouth breathing [21]. Copyright 2021 Royal Society of Chemistry.

generate a respiratory diagnosis report. The schematic diagram for clinical respiratory monitoring and breathing test results are shown in the Fig. 50.

These studies suggest an ultrafast, repetitive, stable, non-toxic humidity sensor offers a very good option to be used to monitor human respiration precisely to capture the quick changes in the breathing pattern and diagnose the abnormal conditions of asthma, apnea, choking etc. Further work is in progress to control and read the patterns of these sensors with artificial intelligence (AI) to bring the best possible information without any delays. Besides humidity sensing, SMOs offer a potential candidate to record the breathing patterns in terms of different gas components to diagnose early detection of certain malfunctioning human organs. Zhang et al. [246] have reported a review article where they discussed the SMOs nanostructures for the application of wearable biomarker gas sensors to detect human diseases. Wang et al. [247] have proposed a self-powered organ-like $Ti_3C_2T_x$ MXene/ metal-organic framework CuO gas sensor for NH_3 with a detection range of 0 to 100 ppm at room temperature with excellent response. Since NH_3 exists in human exhaled breath and can be used as a biomarker for the end-stage renal disease of kidney patient, the said sensor can be a good candidate as breath analyzer for monitoring kidney related problems. As human health is an important parameter to be measured, in this area, more studies have to be performed for SMO-based nanostructures and their composites considering their rich surface physics and the distinct humidity sensing characteristics.

12. Conclusion and future perspectives

This review comprehensively presented and summarized the SMO nano-composites based humidity sensors. The humidity sensors have been classified based on the different measuring parameters such as resistance, capacitance, impedance, QCM, optical, surface acoustic wave, etc. and different materials like SMOs, perovskites, spinel, polymers and carbon-based materials, which have been shortly discussed. SMOs based nanostructures are superior candidates for humidity sensors due to their inherent properties, including high surface-to-volume ratio and high surface-active sites. Furthermore, SMOs are commonly used because they are earth-abundant, affordable, nontoxic, chemically inert, and well-suited for cost-effective wet-chemical routes. The humidity sensing mechanism of SMOs has been discussed. The role of oxygen vacancies, dopants and composite formation is illustrated in detail. The underline physics behind the water adsorption on different SMOs (ZnO, TiO_2, SnO_2 and CuO) surfaces has been discussed based on DFT calculations. DFT calculations suggest that ZnO and TiO_2 majorly favour molecular adsorption of water, while SnO_2 and CuO majorly favour dissociative adsorption, which is further upended by the multilayer physisorption of molecular water. The studies revealed the importance of the different surface terminations of these oxides, as humidity sensing is a surface phenomenon. Besides this, surface defects and surface geometry are also important parameters to influence water sorption activities. Therefore, more theoretical studies need to be performed to understand the humidity sensing behaviors of SMOs with different morphology and nanostructures. Such studies should also be extended for SMO nanocomposites to optimize them for the best humidity-sensing characteristics. The discrete SMO nanostructures, including nanosheets, nanowires, nanotubes, nanofibers and nanoflowers based humidity sensors, have been illustrated, and it has been observed that an increase in the surface areas, defects and polarity significantly influence the humidity sensor characteristics, although the results are not similar for different groups. It is observed that among SMOs, n-type semiconductors such as ZnO, TiO_2 and SnO_2 have been extensively studied as compared to p-type semiconductors such as CuO, NiO, etc., towards humidity sensors. In this review, we have tried to bring the merits and demerits of each SMO as a humidity sensor.

Nevertheless, it is concluded that there is plenty of room to further develop and investigate different nanostructures to improve the humidity sensing characteristics such as poor linearity, low sensitivity, long-term stability, and high response/recovery time/efficiency. The sensing properties can further be enhanced by doping of other elements and composite formation with polymers and carbon materials like GO, rGO, CNTs, which have been discussed in detail. The doping and composites formation can improve the morphological, structural and optical/band engineering properties of SMOs nanostructures and subsequently improve the sensor's characteristics. There is still no consensus on response time, recovery time, hysteresis or the humidity range. To use these sensors for practical applications, one has to work for humidity sensors with the best of all these characteristics.

Miniaturization of sensors maintaining vital sensor characteristics as well as flexible and low-cost devices for practical applications are further significant challenges for researchers nowadays. The use of polymer and the paper-based substrate is an option to produce a superior device due to the advantages of being low-cost, flexible, lightweight, and recyclable advantages. Furthermore, these flexible substrates permit large-scale production and can be easily engaged in printing methods. In recent years, researchers have focused on the development of new technology based on the concept of wearable and self-powered flexible humidity sensors for health monitoring. This work is at an initial stage and needs to be explored. As very few reports are available on these studies, after identifying the potential features of SMOs, one has to look into the possibility to diagnose SMOs' compatibility with the flexible substrates to exploit the rich surface physics of these structures for dynamic humidity sensing applications. This may improve the performance of the humidity sensors and provide a suitable solution for use in wearable devices in the healthcare sector and have applications in artificial intelligence.

Another critical aspect of humidity measurements is accurate and precise humidity measurements at different operating temperatures. This is important for biomedical applications, such as monitoring different breathing patterns, clinical incubators, specialized ICUs, and in many other fields. So, the given humidity sensor must be calibrated with standard humidity reference at regular intervals. The different methods for the generation of relative humidity environment have been discussed in this review which can be used to standardize the humidity sensors and to establish their humidity sensing behavior for practical applications. To be able to withstand harsh environmental conditions, the humidity sensing properties of SMOs nanocomposites must be tested in a wide range of temperatures to get better device performance and endurance in dynamic measurements.

Declaration of Competing Interest

The authors declare that they have no known competing financial interests or personal relationships that could have appeared to influence the work reported in this paper.

Acknowledgment

We thank Prof. Venu Gopal Achanta, Director, CSIR-NPL and Dr. Sanjay Yadav, Head, Physico-Mechanical Metrology Division, for their constant support and encouragement. Ankit Kumar acknowledges the CSIR for the award of Senior Research Fellowship. We acknowledge our colleagues Dr. Umesh Pant, Hansraj Meena, Parvesh Kumari, Ashish Bhatt, Anshul Singh, and Saroj Sharma from Temperature and Humidity Metrology for their help during this work.

References

[1] Z. Chen, C. Lu, Humidity sensors: a review of materials and mechanisms, Sens. Lett. 3 (4) (2005) 274–295, https://doi.org/10.1166/sl.2005.045.
[2] S. Patil, N. Ramgir, S. Mukherji, V.R. Rao, PVA modified ZnO nanowire based microsensors platform for relative humidity and soil moisture measurement, Sens. Actuators B Chem. 253 (2017) 1071–1078, https://doi.org/10.1016/j.snb.2017.07.053.

[3] B.C. Yadav, R. Srivastava, A. Yadav, T. Shukla, Synthesis and characterization of ZnO/ZnNb $_2$ O $_6$ nanocomposite and its application as humidity and LPG sensor, Int. J. Green Nanotechnol. 3 (1) (2011) 56–71, https://doi.org/10.1080/19430892.2011.574539.
[4] D. Bridgeman, J. Corral, A. Quach, X. Xian, E. Forzani, Colorimetric humidity sensor based on liquid composite materials for the monitoring of food and pharmaceuticals, Langmuir 30 (35) (2014) 10785–10791, https://doi.org/10.1021/la502593g.
[5] P. Jayaraman, C. Sengottaiyan, K. Krishnan, Self-assembled polymer thin films towards nanoarchitectonics for respiration monitoring, J. Nanosci. Nanotechnol. 20 (5) (2020) 2893–2901, https://doi.org/10.1166/jnn.2020.17458.
[6] A.T. Güntner, V. Koren, K. Chikkadi, M. Righettoni, S.E. Pratsinis, E-nose sensing of low-ppb formaldehyde in gas mixtures at high relative humidity for breath screening of lung cancer? ACS Sens. 1 (5) (2016) 528–535, https://doi.org/10.1021/acssensors.6b00008.
[7] M. Javaid, A. Haleem, S. Rab, R.Pratap Singh, R. Suman, Sensors for daily life: a review, Sens. Int. 2 (2021), 100121, https://doi.org/10.1016/j.sintl.2021.100121.
[8] A. Tételin, C. Pellet, C. Laville, G. N'Kaoua, Fast response humidity sensors for a medical microsystem, Sens. Actuators B Chem. 91 (1–3) (2003) 211–218, https://doi.org/10.1016/S0925-4005(03)00090-X.
[9] H.G. Moon, Y. Jung, S.D. Han, Y.S. Shim, W.S. Jung, T. Lee, S. Lee, J.H. Park, S. H. Baek, J.S. Kim, H.H. Park, C. Kim, C.Y. Kang, All villi-like metal oxide nanostructures-based chemiresistive electronic nose for an exhaled breath analyzer, Sens. Actuators B Chem. 257 (2018) 295–302, https://doi.org/10.1016/j.snb.2017.10.153.
[10] H.S. Kim, J.H. Kang, J.Y. Hwang, U.S. Shin, Wearable CNTs-based humidity sensors with high sensitivity and flexibility for real-time multiple respiratory monitoring, Nano Converg. 9 (1) (2022) 35, https://doi.org/10.1186/s40580-022-00326-6.
[11] S. Sikarwar, B.C. Yadav, Opto-electronic humidity sensor: A review, Sens. Actuators Phys. 233 (2015) 54–70, https://doi.org/10.1016/j.sna.2015.05.007.
[12] S.P. Gupta, A.S. Pawbake, B.R. Sathe, D.J. Late, P.S. Walke, Superior humidity sensor and photodetector of mesoporous ZnO nanosheets at room temperature, Sens. Actuators B Chem. 293 (2019) 83–92, https://doi.org/10.1016/j.snb.2019.04.086.
[13] R. Akram, M. Saleem, Z. Farooq, M. Yaseen, Z.M. Almohaimeed, Q. Zafar, Integrated capacitive- and resistive-type bimodal relative humidity sensor based on 5,10,15,20-tetraphenylporphyrinatonickel(II) (TPPNi) and Zinc Oxide (ZnO) nanocomposite, ACS Omega 7 (34) (2022) 30590–30600, https://doi.org/10.1021/acsomega.2c04313.
[14] D. Zhang, H. Chang, P. Li, R. Liu, Q. Xue, Fabrication and characterization of an ultrasensitive humidity sensor based on metal oxide/graphene hybrid nanocomposite, Sens. Actuators B Chem. 225 (2016) 233–240, https://doi.org/10.1016/j.snb.2015.11.024.
[15] H. Yan, Z. Chen, L. Zeng, Z. Wang, G. Zheng, R. Zhou, The effect of rGO-doping on the performance of SnO_2/rGO flexible humidity sensor, Nanomaterials 11 (12) (2021) 3368, https://doi.org/10.3390/nano11123368.
[16] D. Shen, Y. Liu, M. Yu, F. Kong, B. Xin, Y. Liu, Bioinspired flexible and highly responsive PVDF-based humidity sensors for respiratory monitoring, Polymer 254 (2022), 125103, https://doi.org/10.1016/j.polymer.2022.125103.
[17] K. Sahoo, B. Mohanty, A. Biswas, J. Nayak, Role of hexamethylenetetramine in ZnO-cellulose nanocomposite enabled UV and humidity sensor, Mater. Sci. Semicond. Process. 105 (2020), 104699, https://doi.org/10.1016/j.mssp.2019.104699.
[18] S. Felix, R.B.P. Chakkravarthy, A.N. Grace, Microwave assisted synthesis of copper oxide and its application in electrochemical sensing, IOP Conf. Ser. Mater. Sci. Eng. 73 (2015), 012115, https://doi.org/10.1088/1757-899X/73/1/012115.
[19] X. Zheng, R. Fan, C. Li, X. Yang, H. Li, J. Lin, X. Zhou, R. Lv, A fast-response and highly linear humidity sensor based on quartz crystal microbalance, Sens. Actuators B Chem. 283 (2019) 659–665, https://doi.org/10.1016/j.snb.2018.12.081.
[20] Z. Li, M. Teng, R. Yang, F. Lin, Y. Fu, W. Lin, J. Zheng, X. Zhong, X. Chen, B. Yang, Y. Liao, Sb-doped WO_3 based QCM humidity sensor with self-recovery ability for real-time monitoring of respiration and wound, Sens. Actuators B Chem. 361 (2022), 131691, https://doi.org/10.1016/j.snb.2022.131691.
[21] X. Liu, D. Zhang, D. Wang, T. Li, X. Song, Z. Kang, A humidity sensing and respiratory monitoring system constructed from quartz crystal microbalance sensors based on a chitosan/polypyrrole composite film, J. Mater. Chem. A 9 (25) (2021) 14524–14533, https://doi.org/10.1039/D1TA02828F.
[22] L. Alwis, T. Sun, K.T.V. Grattan, Optical fibre-based sensor technology for humidity and moisture measurement: review of recent progress, Measurement 46 (10) (2013) 4052–4074, https://doi.org/10.1016/j.measurement.2013.07.030.
[23] R. Verma, S. Pathak, K.K. Dey, S. Sikarwar, B.C. Yadav, A.K. Srivastava, Facile synthesized zinc oxide nanorod film humidity sensor based on variation in optical transmissivity, Nanoscale Adv. 4 (13) (2022) 2902–2912, https://doi.org/10.1039/D1NA00893E.
[24] V. Manikandan, I. Petrila, S. Vigneselvan, R.S. Mane, B. Vasile, R. Dharmavarapu, S. Lundgaard, S. Juodkazis, J. Chandrasekarang, A reliable chemiresistive sensor of nickel-doped tin oxide (Ni-SnO_2) for sensing carbon dioxide gas and humidity, RSC Adv. 10 (7) (2020) 3796–3804, https://doi.org/10.1039/C9RA09579A.
[25] F.S. Tsai, S.J. Wang, Enhanced sensing performance of relative humidity sensors using laterally grown ZnO nanosheets, Sens. Actuators B Chem. 193 (2014) 280–287, https://doi.org/10.1016/j.snb.2013.11.069.
[26] S. Talam, S.R. Karumuri, N. Gunnam, Synthesis, characterization, and spectroscopic properties of ZnO nanoparticles, ISRN Nanotechnol. 2012 (2012) 1–6, https://doi.org/10.5402/2012/372505.
[27] D. Nunes, A. Pimentel, A. Gonçalves, S. Pereira, R. Branquinho, P. Barquinha, E. Fortunato, R. Martins, Metal oxide nanostructures for sensor applications, Semicond. Sci. Technol. 34 (4) (2019), 043001, https://doi.org/10.1088/1361-6641/ab011e.
[28] Institute of measurement and control, Gatton (GB). Gatton Section; National Physical Lab., Teddington (GB), Ed., A guide to the measurement of humidity. 1996.
[29] D.M. Abd El-Galil, E. Mahmoud, NIS two-pressure humidity generator, Int. J. Metrol. Qual. Eng. 7 (3) (2016) 303, https://doi.org/10.1051/ijmqe/2016012.
[30] J.S. Tawale, G. Gupta, A. Mohan, A. Kumar, A.K. Srivastava, Growth of thermally evaporated SnO_2 nanostructures for optical and humidity sensing application, Sens. Actuators B Chem. 201 (2014) 369–377, https://doi.org/10.1016/j.snb.2014.04.099.
[31] H. Kitano, T. Niwa, N. Ochi, C. Takahashi, Saturator efficiency and uncertainty of NMIJ two-pressure two-temperature humidity generator, Int. J. Thermophys. 29 (5) (2008) 1615–1622, https://doi.org/10.1007/s10765-008-0494-x.
[32] C.W. Meyer, J.T. Hodges , R.W. Hyland , G.E. Scace , J. Valencia-Rodriguez , and J.R. Whetstone, Automated Continous-Flow Gravimetric as a Primary Humidity Standard, in: C.W. Meyer, et al. (Eds.), Proceedings of ISHM 2006, The 5th International Symposium on Humidity and Moisture, 2006.
[33] X. Ding, X. Chen, X. Chen, X. Zhao, N. Li, A QCM humidity sensor based on fullerene/graphene oxide nanocomposites with high quality factor, Sens. Actuators B Chem. 266 (2018) 534–542, https://doi.org/10.1016/j.snb.2018.03.143.
[34] C. Lu, A.W. Czanderna, Applications of Piezoelectric Quartz Crystal Microbalances, Elsevier Science, Amsterdam, 2014.
[35] A. Farzaneh, A. Mohammadzadeh, M.D. Esrafili, O. Mermer, Experimental and theoretical study of TiO_2 based nanostructured semiconducting humidity sensor, Ceram. Int. 45 (7) (2019) 8362–8369, https://doi.org/10.1016/j.ceramint.2019.01.144.
[36] K.N. Chappanda, O. Shekhah, O. Yassine, S.P. Patole, M. Eddaoudi, K.N. Salama, The quest for highly sensitive QCM humidity sensors: the coating of CNT/MOF composite sensing films as case study, Sens. Actuators B Chem. 257 (2018) 609–619, https://doi.org/10.1016/j.snb.2017.10.189.
[37] D. Zhang, D. Wang, P. Li, X. Zhou, X. Zong, G. Dong, Facile fabrication of high-performance QCM humidity sensor based on layer-by-layer self-assembled polyaniline/graphene oxide nanocomposite film, Sens. Actuators B Chem. 255 (2018) 1869–1877, https://doi.org/10.1016/j.snb.2017.08.212.
[38] H. Tai, Y. Zhen, C. Liu, Z. Ye, G. Xie, X. Du, Y. Jiang, Facile development of high performance QCM humidity sensor based on protonated polyethylenimine-graphene oxide nanocomposite thin film, Sens. Actuators B Chem. 230 (2016) 501–509, https://doi.org/10.1016/j.snb.2016.01.105.
[39] A. Erol, S. Okur, N. Yağmurcukardeş, M.Ç. Arıkan, Humidity-sensing properties of a ZnO nanowire film as measured with a QCM, Sens. Actuators B Chem. 152 (1) (2011) 115–120, https://doi.org/10.1016/j.snb.2010.09.005.
[40] C. Caliendo, E. Verona, A. D'Amico, A. Furlani, G. Iucci, M.V. Russo, Surface acoustic wave humidity sensor, Sens. Actuators B Chem. 16 (1–3) (1993) 288–292, https://doi.org/10.1016/0925-4005(93)85197-I.
[41] Q. Lin, Y. Li, M. Yang, Highly sensitive and ultrafast response surface acoustic wave humidity sensor based on electrospun polyaniline/poly(vinyl butyral) nanofibers, Anal. Chim. Acta 748 (2012) 73–80, https://doi.org/10.1016/j.aca.2012.08.041.
[42] A. Buvailo, Y. Xing, J. Hines, E. Borguet, Thin polymer film based rapid surface acoustic wave humidity sensors, Sens. Actuators B Chem. 156 (1) (2011) 444–449, https://doi.org/10.1016/j.snb.2011.04.080.
[43] X. Le, X. Wang, J. Pang, Y. Liu, B. Fang, Z. Xu, C. Gao, Y. Xu, J. Xie, A high performance humidity sensor based on surface acoustic wave and graphene oxide on AlN/Si layered structure, Sens. Actuators B Chem. 255 (2018) 2454–2461, https://doi.org/10.1016/j.snb.2017.09.038. Feb.
[44] L. Sheng, C. Dajing, C. Yuquan, A surface acoustic wave humidity sensor with high sensitivity based on electrospun MWCNT/Nafion nanofiber films, Nanotechnology 22 (26) (2011), 265504, https://doi.org/10.1088/0957-4484/22/26/265504.
[45] H.S. Hong, G.S. Chung, Controllable growth of oriented ZnO nanorods using Ga-doped seed layers and surface acoustic wave humidity sensor, Sens. Actuators B Chem. 195 (2014) 446–451, https://doi.org/10.1016/j.snb.2013.12.120.
[46] C.-L. Zhao, M. Qin, W.-H. Li, Q.-A. Huang, Enhanced performance of a CMOS interdigital capacitive humidity sensor by graphene oxide, in: Z. K. X. Yuan (Ed.), 2011 16th International Solid-State Sensors, Actuators and Microsystems Conference (TRANSDUCERS 2011), IEEE, 2011.
[47] H.T. Hsueh, T.J. Hsueh, S.J. Chang, F.Y. Hung, T.Y. Tsai, W.Y. Weng, C.L. Hsu, B. T. Dai, CuO nanowire-based humidity sensors prepared on glass substrate, Sens. Actuators B Chem. 156 (2) (2011) 906–911, https://doi.org/10.1016/j.snb.2011.03.004.
[48] M. Detjens, T. Hübert, C. Tiebe, U. Banach, Coulometric trace humidity measurement in technical gases, Rev. Sci. Instrum. 89 (8) (2018), 085004, https://doi.org/10.1063/1.5008463.
[49] D. Gomez, S.P. Morgan, B.R. Hayes-Gill, R.G. Correia, S. Korposh, Polymeric optical fibre sensor coated by SiO_2 nanoparticles for humidity sensing in the skin microenvironment, Sens. Actuators B Chem. 254 (2018) 887–895, https://doi.org/10.1016/j.snb.2017.07.191.

[50] R.S. Jachowicz, W.J. Makulski, Optimal measurement procedures for a dew point hygrometer system, IEEE Trans. Instrum. Meas. 42 (4) (1993) 828–833, https://doi.org/10.1109/19.234493.
[51] H.Y. He, J.F. Huang, L.Y. Cao, J.P. Wu, Humidity sensitivity of $MnTiO_3$ film prepared via chemical solution deposition process, Sens. Actuators B Chem. 132 (1) (2008) 5–8, https://doi.org/10.1016/j.snb.2008.01.017.
[52] H. Iwahara, H. Uchida, J. Kondo, Galvanic cell-type humidity sensor using high temperature-type proton conductive solid electrolyte, J. Appl. Electrochem. 13 (3) (1983) 365–370, https://doi.org/10.1007/BF00941609.
[53] C. Doroftei, L. Leontie, Nanocrystalline $SrMnO_3$ perovskite prepared by sol–gel self-combustion method for sensor applications, J. Sol Gel Sci. Technol. 97 (1) (2021) 146–154, https://doi.org/10.1007/s10971-020-05419-4.
[54] C. Doroftei, L. Leontie, Porous nanostructured gadolinium aluminate for high-sensitivity humidity sensors, Materials 14 (22) (2021) 7102, https://doi.org/10.3390/ma14227102.
[55] T. Nitta, Z. Terada, S. Hayakawa, Humidity-sensitive electrical conduction of $MgCr_2O_4$-TiO_2 porous ceramics, J. Am. Ceram. Soc. 63 (5–6) (1980) 295–300, https://doi.org/10.1111/j.1151-2916.1980.tb10724.x.
[56] Z. Wang, C.L. Chang, X. Zhao, W. Qian, X. Zhang, Z. Xie, B.H. Hwang, C. Hu, J. Shen, R. Hui, $MgAl_2O_4$-based humidity-sensing material for potential application in PEM fuel cells, J. Power Sources 190 (2) (2009) 351–355, https://doi.org/10.1016/j.jpowsour.2008.08.078.
[57] Y. Yokomizo, S. Uno, M. Harata, H. Hiraki, K. Yuki, Microstructure and humidity-sensitive properties of $ZnCr_2O_4$-$LiZnVO_4$ ceramic sensors, Sens. Actuators 4 (1983) 599–606, https://doi.org/10.1016/0250-6874(83)85073-2.
[58] B. Cheng, Z. Ouyang, B. Tian, Y. Xiao, S. Lei, Porous $ZnAl_2O_4$ spinel nanorods: high sensitivity humidity sensors, Ceram. Int. 39 (7) (2013) 7379–7386, https://doi.org/10.1016/j.ceramint.2013.02.077.
[59] M. Hashim, Alimuddin, S. Kumar, S.E. Shirsath, R.K. Kotnala, J. Shah, R. Kumar, Synthesis and characterizations of Ni^{2+} substituted cobalt ferrite nanoparticles, Mater. Chem. Phys. 139 (2–3) (2013) 364–374, https://doi.org/10.1016/j.matchemphys.2012.09.019.
[60] J. Shah, R.K. Kotnala, Humidity sensing exclusively by physisorption of water vapors on magnesium ferrite, Sens. Actuators B Chem. 171–172 (2012) 832–837, https://doi.org/10.1016/j.snb.2012.05.079.
[61] R.K. Kotnala, J. Shah, B. Singh, H. kishan, S. Singh, S.K. Dhawan, A. Sengupta, Humidity response of Li-substituted magnesium ferrite, Sens. Actuators B Chem. 129 (2) (2008) 909–914, https://doi.org/10.1016/j.snb.2007.10.002.
[62] W. Yan, D. Zhang, X. Liu, X. Chen, C. Yang, Z. Kang, Guar gum/ethyl cellulose-polyvinyl pyrrolidone composite-based quartz crystal microbalance humidity sensor for human respiration monitoring, ACS Appl. Mater. Interfaces 14 (27) (2022) 31343–31353, https://doi.org/10.1021/acsami.2c08434.
[63] E.M. Amin, N.C. Karmakar, B. Winther-Jensen, Polyvinyl-alcohol (PVA)-based RF humidity sensor in microwave frequency, Prog. Electromagn. Res. B 54 (2013) 149–166, https://doi.org/10.2528/PIERB13061716.
[64] A. Gastón, F. Pérez, J. Sevilla, Optical fiber relative-humidity sensor with polyvinyl alcohol film, Appl. Opt. 43 (21) (2004) 4127, https://doi.org/10.1364/AO.43.004127.
[65] L. Wang, J. Xu, X. Wang, Z. Cheng, J. Xu, Facile preparation of N-rich functional polymer with porous framework as QCM sensing material for rapid humidity detection, Sens. Actuators B Chem. 288 (2019) 289–297, https://doi.org/10.1016/j.snb.2019.02.058. Jun.
[66] V.P. Anju, P.R. Jithesh, S.K. Narayanankutty, A novel humidity and ammonia sensor based on nanofibers/polyaniline/polyvinyl alcohol, Sens. Actuators Phys. 285 (2019) 35–44, https://doi.org/10.1016/j.sna.2018.10.037.
[67] Y. Li, C. Deng, M. Yang, A novel surface acoustic wave-impedance humidity sensor based on the composite of polyaniline and poly(vinyl alcohol) with a capability of detecting low humidity, Sens. Actuators B Chem. 165 (1) (2012) 7–12, https://doi.org/10.1016/j.snb.2011.12.037.
[68] D. Zhang, J. Tong, B. Xia, Humidity-sensing properties of chemically reduced graphene oxide/polymer nanocomposite film sensor based on layer-by-layer nano self-assembly, Sens. Actuators B Chem. 197 (2014) 66–72, https://doi.org/10.1016/j.snb.2014.02.078.
[69] J.M. Corres, Y.R. Garcia, F.J. Arregui, I.R. Matias, Optical fiber humidity sensors using PVdF electrospun nanowebs, IEEE Sens. J. 11 (10) (2011) 2383–2387, https://doi.org/10.1109/JSEN.2011.2123881.
[70] J. Wu, Y.M. Sun, Z. Wu, X. Li, N. Wang, K. Tao, G.P. Wang, Carbon nanocoil-based fast-response and flexible humidity sensor for multifunctional applications, ACS Appl. Mater. Interfaces 11 (4) (2019) 4242–4251, https://doi.org/10.1021/acsami.8b18599.
[71] P. Zhu, H. Ou, Y. Kuang, L. Hao, J. Diao, G. Chen, Cellulose nanofiber/carbon nanotube dual network-enabled humidity sensor with high sensitivity and durability, ACS Appl. Mater. Interfaces 12 (29) (2020) 33229–33238, https://doi.org/10.1021/acsami.0c07995.
[72] A. Fort, A. Lo Grasso, M. Mugnaini, E. Panzardi, V. Vignoli, QCM measurements of RH with nanostructured carbon-based materials: part 1-theory and model, Chemosensors 10 (8) (2022) 315, https://doi.org/10.3390/chemosensors10080315.
[73] Y.T. Huang, C.C. Huang, Y.Y. Chen, C.Y. Su, Y.C. Tsai, Effect of substrate topography for graphene-based humidity sensors, Jpn. J. Appl. Phys. 58 (SD) (2019) SDDD04, https://doi.org/10.7567/1347-4065/ab0546.
[74] X. Zhang, D. Maddipatla, A.K. Bose, S. Hajian, B.B. Narakathu, J.D. Williams, M. F. Mitchell, M.Z. Atashbar, Printed carbon nanotubes-based flexible resistive humidity sensor, IEEE Sens. J. 20 (21) (2020) 12592–12601, https://doi.org/10.1109/JSEN.2020.3002951.
[75] E. Traversa, Ceramic sensors for humidity detection: the state-of-the-art and future developments, Sens. Actuators B Chem. 23 (2–3) (1995) 135–156, https://doi.org/10.1016/0925-4005(94)01268-M.
[76] Z. Wang, L. Shi, F. Wu, S. Yuan, Y. Zhao, M. Zhang, Structure and humidity sensing properties of $La_{1-x}K_xCo_{0.3}Fe_{0.7}O_{3-\delta}$ perovskite, Sens. Actuators B Chem. 158 (1) (2011) 89–96, https://doi.org/10.1016/j.snb.2011.05.046.
[77] Z. Wu, J. Yang, X. Sun, Y. Wu, L. Wang, G. Meng, D. Kuang, X. Guo, W. Qu, B. Du, C. Liang, X. Fang, X. Tang, Y. He, An excellent impedance-type humidity sensor based on halide perovskite $CsPbBr_3$ nanoparticles for human respiration monitoring, Sens. Actuators B Chem. 337 (2021), 129772, https://doi.org/10.1016/j.snb.2021.129772.
[78] C.L. Cao, C.G. Hu, L. Fang, S.X. Wang, Y.S. Tian, C.Y. Pan, Humidity sensor based on multi-walled carbon nanotube thin films, J. Nanomater. 2011 (2011) 1–5, https://doi.org/10.1155/2011/707303.
[79] S. Borini, R. White, D. Wei, M. Astley, S. Haque, E. Spigone, N. Harris, J. Kivioja, T. Ryhänen, Ultrafast Graphene Oxide Humidity Sensors, ACS Nano 7 (12) (2013) 11166–11173, https://doi.org/10.1021/nn404889b.
[80] K. Jiang, T. Fei, F. Jiang, G. Wang, T. Zhang, A dew sensor based on modified carbon black and polyvinyl alcohol composites, Sens. Actuators B Chem. 192 (2014) 658–663, https://doi.org/10.1016/j.snb.2013.11.004.
[81] J.M. Tulliani, B. Inserra, D. Ziegler, Carbon-based materials for humidity sensing: a short review, Micromachines 10 (4) (2019) 232, https://doi.org/10.3390/mi10040232.
[82] Y. Kumar, A. Sharma, P.M. Shirage, Shape-controlled $CoFe_2O_4$ nanoparticles as an excellent material for humidity sensing, RSC Adv. 7 (88) (2017) 55778–55785, https://doi.org/10.1039/C7RA11072C.
[83] I. Petrila, F. Tudorache, Humidity sensor applicative material based on copper-zinc-tungsten spinel ferrite, Mater. Lett. 108 (2013) 129–133, https://doi.org/10.1016/j.matlet.2013.06.066.
[84] G. Hassan, J. Bae, C.H. Lee, A. Hassan, Wide range and stable ink-jet printed humidity sensor based on graphene and zinc oxide nanocomposite, J. Mater. Sci. Mater. Electron. 29 (7) (2018) 5806–5813, https://doi.org/10.1007/s10854-018-8552-z.
[85] E. Modaresinezhad, S. Darbari, Realization of a room-temperature/self-powered humidity sensor, based on ZnO nanosheets, Sens. Actuators B Chem. 237 (2016) 358–366, https://doi.org/10.1016/j.snb.2016.06.097.
[86] R. Das, A. Kumar, Y. Kumar, S. Sen, P.M. Shirage, Effect of growth temperature on the optical properties of ZnO nanostructures grown by simple hydrothermal method, RSC Adv. 5 (74) (2015) 60365–60372, https://doi.org/10.1039/C5RA07135F.
[87] M. Parashar, V.K. Shukla, R. Singh, Metal oxides nanoparticles via sol–gel method: a review on synthesis, characterization and applications, J. Mater. Sci. Mater. Electron. 31 (5) (2020) 3729–3749, https://doi.org/10.1007/s10854-020-02994-8.
[88] G.E. Brown, V.E. Henrich, W.H. Casey, D.L. Clark, C. Eggleston, A. Felmy, D. W. Goodman, M. Grätzel, G. Maciel, M.I. McCarthy, K.H. Nealson, D. A. Sverjensky, M.F. Toney, J.M. Zachara, Metal oxide surfaces and their interactions with aqueous solutions and microbial organisms, Chem. Rev. 99 (1) (1999) 77–174, https://doi.org/10.1021/cr980011z.
[89] T.A. Blank, L.P. Eksperiandova, K.N. Belikov, Recent trends of ceramic humidity sensors development: a review, Sens. Actuators B Chem. 228 (2016) 416–442, https://doi.org/10.1016/j.snb.2016.01.015.
[90] T. Miyake, M. Rolandi, Grotthuss mechanisms: from proton transport in proton wires to bioprotonic devices, J. Phys. Condens. Matter 28 (2) (2016), 023001, https://doi.org/10.1088/0953-8984/28/2/023001.
[91] R. Schaub, P. Thostrup, N. Lopez, E. Lægsgaard, I. Stensgaard, J.K. Nørskov, F. Besenbacher, Oxygen vacancies as active sites for water dissociation on rutile TiO_2 (110), Phys. Rev. Lett. 87 (26) (2001), 266104, https://doi.org/10.1103/PhysRevLett.87.266104.
[92] Zhang Yu, Zhang, Li, Effects of pH on high-performance ZnO resistive humidity sensors using one-step synthesis, Sensors 19 (23) (2019) 5267, https://doi.org/10.3390/s19235267.
[93] Y. Gu, Z. Ye, N. Sun, X. Kuang, W. Liu, X. Song, L. Zhang, W. Bai, X. Tang, Preparation and properties of humidity sensor based on K-doped ZnO nanostructure, J. Mater. Sci. Mater. Electron. 30 (20) (2019) 18767–18779, https://doi.org/10.1007/s10854-019-02230-y.
[94] Z. Li, A.A. Haidry, B. Gao, T. Wang, Z. Yao, The effect of Co-doping on the humidity sensing properties of ordered mesoporous TiO_2, Appl. Surf. Sci. 412 (2017) 638–647, https://doi.org/10.1016/j.apsusc.2017.03.156.
[95] M. Sabarilakshmi, K. Janaki, A facile and one step synthesis of W doped SnO_2 nanopowders with enhanced humidity sensing performance, J. Mater. Sci. Mater. Electron. 28 (7) (2017) 5329–5335, https://doi.org/10.1007/s10854-016-6191-9.
[96] J. Xu, M. Bertke, X. Li, H. Mu, H. Zhou, F. Yu, G. Hamdana, A. Schmidt, H. Bremers, E. Peiner, Fabrication of ZnO nanorods and Chitosan@ZnO nanorods on MEMS piezoresistive self-actuating silicon microcantilever for humidity sensing, Sens. Actuators B Chem. 273 (2018) 276–287, https://doi.org/10.1016/j.snb.2018.06.017.
[97] D. Zhang, H. Chang, R. Liu, Humidity-sensing properties of one-step hydrothermally synthesized tin dioxide-decorated graphene nanocomposite on polyimide substrate, J. Electron. Mater. 45 (8) (2016) 4275–4281, https://doi.org/10.1007/s11664-016-4630-2.
[98] P. Singh, S.K. Shukla, Structurally optimized cupric oxide/polyaniline nanocomposites for efficient humidity sensing, Surf. Interfaces 18 (2020), 100410, https://doi.org/10.1016/j.surfin.2019.100410.

[99] D.C. Look, Recent advances in ZnO materials and devices, Mater. Sci. Eng. B 80 (1–3) (2001) 383–387, https://doi.org/10.1016/S0921-5107(00)00604-8.
[100] Z.L. Wang, Nanostructures of zinc oxide, Mater. Today 7 (6) (2004) 26–33, https://doi.org/10.1016/S1369-7021(04)00286-X.
[101] M.H. Jali, H.R.A. Rahim, M.A.M. Johari, H.H.M. Yusof, A. Ahmad, S. Thokchom, K. Dimyati, S.W. Harun, Humidity sensing using microfiber-ZnO nanorods coated glass structure, Optik 238 (2021), 166715, https://doi.org/10.1016/j.ijleo.2021.166715.
[102] J. Wu, C. Yin, J. Zhou, H. Li, Y. Liu, Y. Shen, S. Garner, Y. Fu, H. Duan, Ultrathin glass-based flexible, transparent, and ultrasensitive surface acoustic wave humidity sensor with ZnO nanowires and graphene quantum dots, ACS Appl. Mater. Interfaces 12 (35) (2020) 39817–39825, https://doi.org/10.1021/acsami.0c09962.
[103] S. Mohammadnejad, S. Ahadzadeh, M. Nouri Rezaie, Effect of ZnO nanorods and nanotubes on the electrical and optical characteristics of organic and perovskite light-emitting diodes, Nanotechnology 32 (24) (2021), 245204, https://doi.org/10.1088/1361-6528/abe893.
[104] P. Luo, M. Xie, J. Luo, H. Kan, Q. Wei, Nitric oxide sensors using nanospiral ZnO thin film deposited by GLAD for application to exhaled human breath, RSC Adv. 10 (25) (2020) 14877–14884, https://doi.org/10.1039/D0RA00488J.
[105] C. Li, Y. Lin, F. Li, L. Zhu, F. Meng, D. Sun, J. Zhou, S. Ruan, Synthesis and highly enhanced acetylene sensing properties of Au nanoparticle-decorated hexagonal ZnO nanorings, RSC Adv. 5 (106) (2015) 87132–87138, https://doi.org/10.1039/C5RA16552K.
[106] M. Que, C. Lin, J. Sun, L. Chen, X. Sun, Y. Sun, Progress in ZnO Nanosensors, Sensors 21 (16) (2021) 5502, https://doi.org/10.3390/s21165502.
[107] O. Dulub, B. Meyer, U. Diebold, Observation of the dynamical change in a water monolayer adsorbed on a ZnO surface, Phys. Rev. Lett. 95 (13) (2005), 136101, https://doi.org/10.1103/PhysRevLett.95.136101.
[108] A. Calzolari, A. Catellani, Water adsorption on nonpolar ZnO(101¯0) surface: a microscopic understanding, J. Phys. Chem. C 113 (7) (2009) 2896–2902, https://doi.org/10.1021/jp808704d.
[109] D. Raymand, A.C.T. van Duin, D. Spångberg, W.A. Goddard, K. Hermansson, Water adsorption on stepped ZnO surfaces from MD simulation, Surf. Sci. 604 (9–10) (2010) 741–752, https://doi.org/10.1016/j.susc.2009.12.012.
[110] N.F. Hsu, M. Chang, C.H. Lin, Synthesis of ZnO thin films and their application as humidity sensors, Microsyst. Technol. 19 (11) (2013) 1737–1743, https://doi.org/10.1007/s00542-013-1830-z.
[111] Q. Wan, Q.H. Li, Y.J. Chen, T.H. Wang, Positive temperature coefficient resistance and humidity sensing properties of Cd-doped ZnO nanowires, Appl. Phys. Lett. 84 (16) (2004) 3085–3087, https://doi.org/10.1063/1.1707225.
[112] S.J. Young, K.W. Yuan, ZnO nanorod humidity sensor and dye-sensitized solar cells as a self-powered device, IEEE Trans. Electron. Devices 66 (9) (2019) 3978–3981, https://doi.org/10.1109/TED.2019.2926021.
[113] S. Jagtap, K.R. Priolkar, Evaluation of ZnO nanoparticles and study of ZnO–TiO_2 composites for lead free humidity sensors, Sens. Actuators B Chem. 183 (2013) 411–418, https://doi.org/10.1016/j.snb.2013.04.010.
[114] Y.J. Liu, H.D. Zhang, J. Zhang, S. Li, J.C. Zhang, J.W. Zhu, M.G. Gong, X.X. Wang, Y.Z. Long, Effects of Ce doping and humidity on UV sensing properties of electrospun ZnO nanofibers, J. Appl. Phys. 122 (10) (2017), 105102, https://doi.org/10.1063/1.5000443.
[115] P. Li, S. Yu, H. Zhang, Preparation and performance analysis of Ag/ZnO humidity sensor, Sensors 21 (3) (2021) 857, https://doi.org/10.3390/s21030857.
[116] S. Yu, H. Zhang, C. Lin, M. Bian, The enhancement of humidity sensing performance based on Eu-doped ZnO, Curr. Appl. Phys. 19 (2) (2019) 82–88, https://doi.org/10.1016/j.cap.2018.11.015.
[117] X. Peng, J. Chu, B. Yang, P.X. Feng, Mn-doped zinc oxide nanopowders for humidity sensors, Sens. Actuators B Chem. 174 (2012) 258–262, https://doi.org/10.1016/j.snb.2012.07.011.
[118] M. Velumani, S.R. Meher, Z.C. Alex, Impedometric humidity sensing characteristics of SnO_2 thin films and SnO_2–ZnO composite thin films grown by magnetron sputtering, J. Mater. Sci. Mater. Electron. 29 (5) (2018) 3999–4010, https://doi.org/10.1007/s10854-017-8342-z.
[119] F. Li, P. Li, H. Zhang, Preparation and research of a high-performance ZnO/SnO_2 humidity sensor, Sensors 22 (1) (2021) 293, https://doi.org/10.3390/s22010293.
[120] D. Burman, D.S. Choudhary, P.K. Guha, ZnO/MoS_2 -based enhanced humidity sensor prototype with android app interface for mobile platform, IEEE Sens. J. 19 (11) (2019) 3993–3999, https://doi.org/10.1109/JSEN.2019.2896208.
[121] C. Wang, H. Huang, M.-R. Zhang, W.X. Song, L. Zhang, R. Xi, L.J. Wang, G.B. Pan, A ZnO/porous GaN heterojunction and its application as a humidity sensor, Nanoscale Adv. 1 (3) (2019) 1232–1239, https://doi.org/10.1039/C8NA00243F.
[122] Q. Qi, T. Zhang, Q. Yu, R. Wang, Y. Zeng, L. Liu, H. Yang, Properties of humidity sensing ZnO nanorods-base sensor fabricated by screen-printing, Sens. Actuators B Chem. 133 (2) (Aug. 2008) 638–643, https://doi.org/10.1016/j.snb.2008.03.035.
[123] A. Lokman, H. Arof, S.W. Harun, Z. Harith, H.A. Rafaie, R.M. Nor, Optical fiber relative humidity sensor based on inline mach–zehnder interferometer with ZnO nanowires coating, IEEE Sens. J. 16 (2) (2016) 312–316, https://doi.org/10.1109/JSEN.2015.2431716.
[124] M. Anbia, S.E.M. Fard, Humidity sensing properties of Ce-doped nanoporous ZnO thin film prepared by sol-gel method, J. Rare Earths 30 (1) (2012) 38–42, https://doi.org/10.1016/S1002-0721(10)60635-7.
[125] A.S. Ismail, M.H. Mamat, N.D.M. Sin, M.F. Malek, A.S. Zoolfakar, A.B. Suriani, A. Mohamed, M.K. Ahmad, M. Rusop, Fabrication of hierarchical Sn-doped ZnO nanorod arrays through sonicated sol−gel immersion for room temperature, resistive-type humidity sensor applications, Ceram. Int. 42 (8) (2016) 9785–9795, https://doi.org/10.1016/j.ceramint.2016.03.071.
[126] S.K. Misra, N.K. Pandey, Study of activation energy and humidity sensing application of nanostructured Cu-doped ZnO thin films, J. Mater. Res. 31 (20) (2016) 3214–3222, https://doi.org/10.1557/jmr.2016.322.
[127] M. Zhang, H. Zhang, L. Li, K. Tuokedaerhan, Z. Jia, Er-enhanced humidity sensing performance in black ZnO-based sensor, J. Alloys Compd. 744 (2018) 364–369, https://doi.org/10.1016/j.jallcom.2018.02.109.
[128] C. Lin, H. Zhang, J. Zhang, C. Chen, Enhancement of the humidity sensing performance in Mg-doped hexagonal ZnO microspheres at room temperature, Sensors 19 (3) (2019) 519, https://doi.org/10.3390/s19030519.
[129] Ş. Dinç Zor, H. Cankurtaran, Impedimetric humidity sensor based on nanohybrid composite of conducting poly(diphenylamine sulfonic acid, J. Sens. 2016 (2016) 1–9, https://doi.org/10.1155/2016/5479092.
[130] M.V. Arularasu, M. Harb, R. Vignesh, T.V. Rajendran, R. Sundaram, PVDF/ZnO hybrid nanocomposite applied as a resistive humidity sensor, Surf. Interfaces 21 (2020), 100780, https://doi.org/10.1016/j.surfin.2020.100780.
[131] D. Kuntal, S. Chaudhary, A.B.V.K. Kumar, R. Megha, C.H.V.V. Ramana, Y.T. Ravi Kiran, S. Thomas, D. Kim, rGO/ZnO nanorods/Cu based nanocomposite having flower shaped morphology: AC conductivity and humidity sensing response studies at room temperature, J. Mater. Sci. Mater. Electron. 30 (16) (2019) 15544–15552, https://doi.org/10.1007/s10854-019-01931-8.
[132] S.K. Shukla, C.S. Kushwaha, A. Shukla, G.C. Dubey, Integrated approach for efficient humidity sensing over zinc oxide and polypyrole composite, Mater. Sci. Eng. C 90 (2018) 325–332, https://doi.org/10.1016/j.msec.2018.04.054.
[133] H. Dai, N. Feng, J. Li, J. Zhang, W. Li, Chemiresistive humidity sensor based on chitosan/zinc oxide/single-walled carbon nanotube composite film, Sens. Actuators B Chem. 283 (2019) 786–792, https://doi.org/10.1016/j.snb.2018.12.056.
[134] K. Atamnia, H. Satha, M. Bououdina, Synthesis and characterisation of TiO_2 nanostructures for photocatalytic applications, Int. J. Nanoparticles 10 (3) (2018) 225, https://doi.org/10.1504/IJNP.2018.094048.
[135] K. Yanagisawa, J. Ovenstone, Crystallization of anatase from amorphous titania using the hydrothermal technique: effects of starting material and temperature, J. Phys. Chem. B 103 (37) (1999) 7781–7787, https://doi.org/10.1021/jp990521c.
[136] O. Khatim, M. Amamra, K. Chhor, A.M.T. Bell, D. Novikov, D. Vrel, A. Kanaev, Amorphous–anatase phase transition in single immobilized TiO_2 nanoparticles, Chem. Phys. Lett. 558 (2013) 53–56, https://doi.org/10.1016/j.cplett.2012.12.019.
[137] X. Rocquefelte, F. Goubin, H.J. Koo, M.H. Whangbo, S. Jobic, Investigation of the origin of the empirical relationship between refractive index and density on the basis of first principles calculations for the refractive indices of various TiO_2 phases, Inorg. Chem. 43 (7) (2004) 2246–2251, https://doi.org/10.1021/ic035383r.
[138] M. Takeuchi, T. Itoh, H. Nagasaka, Dielectric properties of sputtered TiO_2 films, Thin Solid Films 51 (1) (1978) 83–88, https://doi.org/10.1016/0040-6090(78)90215-8.
[139] M.J. Alam, D.C. Cameron, Preparation and characterization of TiO_2 thin films by Sol-Gel method", J. Sol-Gel Sci. Technol. 25 (2) (2002) 137–145, https://doi.org/10.1023/A:1019912312654.
[140] K.R. Patil, S.D. Sathaye, Y.B. Khollam, S.B. Deshpande, N.R. Pawaskar, A. B. Mandale, Preparation of TiO_2 thin films by modified spin-coating method using an aqueous precursor, Mater. Lett. 57 (12) (2003) 1775–1780, https://doi.org/10.1016/S0167-577X(02)01067-4.
[141] J.-M. Wu, H.C. Shih, W.-T. Wu, Electron field emission from single crystalline TiO_2 nanowires prepared by thermal evaporation, Chem. Phys. Lett. 413 (4–6) (2005) 490–494, https://doi.org/10.1016/j.cplett.2005.07.113.
[142] S. Takeda, S. Suzuki, H. Odaka, H. Hosono, Photocatalytic TiO_2 thin film deposited onto glass by DC magnetron sputtering, Thin Solid Films 392 (2) (2001) 338–344, https://doi.org/10.1016/S0040-6090(01)01054-9.
[143] S. Karuppuchamy, D.P. Amalnerkar, K. Yamaguchi, T. Yoshida, T. Sugiura, H. Minoura, Cathodic electrodeposition of TiO_2 thin films for dye-sensitized photoelectrochemical applications, Chem. Lett. 30 (1) (2001) 78–79, https://doi.org/10.1246/cl.2001.78.
[144] T.D. Dongale, S.S. Shinde, R.K. Kamat, K.Y. Rajpure, Nanostructured TiO_2 thin film memristor using hydrothermal process, J. Alloys Compd. 593 (2014) 267–270, https://doi.org/10.1016/j.jallcom.2014.01.093.
[145] D. Di Claudio, A.R. Phani, S. Santucci, Enhanced optical properties of sol–gel derived TiO_2 films using microwave irradiation, Opt. Mater. 30 (2) (2007) 279–284, https://doi.org/10.1016/j.optmat.2006.10.032.
[146] G. Dubourg, A. Segkos, J. Katona, M. Radović, S. Savić, G. Niarchos, C. Tsamis, V. C.-Bengin, Fabrication and characterization of flexible and miniaturized humidity sensors using screen-printed TiO_2 nanoparticles as sensitive layer, Sensors 17 (8) (2017) 1854, https://doi.org/10.3390/s17081854.
[147] H. Li, Q. Yu, Y. Huang, C. Yu, R. Li, J. Wang, F. Guo, S. Jiao, S. Gao, Y. Zhang, X. Zhang, Peng Wang, L. Zhao, Ultralong rutile TiO_2 nanowire arrays for highly efficient dye-sensitized solar cells, ACS Appl. Mater. Interfaces 8 (21) (2016) 13384–13391, https://doi.org/10.1021/acsami.6b01508.
[148] X. Zhao, X. Chen, X. Yu, P. Du, N. Li, X. Chen, Humidity-sensitive properties of TiO_2 nanorods grown between electrodes on Au interdigital electrode substrate, IEEE Sens. J. 17 (19) (2017) 6148–6152, https://doi.org/10.1109/JSEN.2017.2732829.
[149] S. Atalay, S. Erdemoğlu, V.S. Kolat, T. İzgi, E. Akgeyik, H.C. Yılmaz, H. Kaya, F. E. Atalay, A rapid response humidity sensor for monitoring human respiration

with TiO_2-based nanotubes as a sensing layer, J. Electron. Mater. 49 (5) (2020) 3209–3215, https://doi.org/10.1007/s11664-020-08006-z.
[150] G. Chen, S. Ji, H. Li, X. Kang, S. Chang, Y. Wang, G. Yu, J. Lu, J. Claverie, Y. Sang, H. Liu, High-energy faceted SnO_2 -coated TiO_2 nanobelt heterostructure for near-ambient temperature-responsive ethanol sensor, ACS Appl. Mater. Interfaces 7 (44) (2015) 24950–24956, https://doi.org/10.1021/acsami.5b08630.
[151] A. Farzaneh, M.D. Esrafili, Ö. Mermer, Development of TiO_2 nanofibers based semiconducting humidity sensor: adsorption kinetics and DFT computations, Mater. Chem. Phys. 239 (2020), 121981, https://doi.org/10.1016/j.matchemphys.2019.121981.
[152] S. Kotresh, Y.T. Ravikiran, H.G.R. Prakash, S.C.V. Kumari, Polyaniline-titanium dioxide composite as humidity sensor at room temperature, Nanosyst. Phys. Chem. Math. (2016) 732–739, https://doi.org/10.17586/2220-8054-2016-7-4-732-739. Aug.
[153] H. Perron, J. Vandenborre, C. Domain, R. Drot, J. Roques, E. Simoni, J. J. Ehrhardt, H. Catalette, Combined investigation of water sorption on TiO_2 rutile (110) single crystal face: XPS vs. periodic DFT, Surf. Sci. 601 (2) (2007) 518–527, https://doi.org/10.1016/j.susc.2006.10.015.
[154] W.K. Li, X.Q. Gong, G. Lu, A. Selloni, Different reactivities of TiO_2 polymorphs: comparative DFT calculations of water and formic acid adsorption at anatase and brookite TiO_2 surfaces, J. Phys. Chem. C 112 (17) (2008) 6594–6596, https://doi.org/10.1021/jp802335h.
[155] M. Menetrey, A. Markovits, C. Minot, Reactivity of a reduced metal oxide surface: hydrogen, water and carbon monoxide adsorption on oxygen defective rutile TiO_2(110), Surf. Sci. 524 (1–3) (2003) 49–62, https://doi.org/10.1016/S0039-6028(02)02464-0.
[156] J. Ahdjoudj, A. Markovits, C. Minot, Hartree–Fock periodic study of the chemisorption of small molecules on TiO_2 and MgO surfaces, Catal. Today 50 (3–4) (1999) 541–551, https://doi.org/10.1016/S0920-5861(98)00489-1.
[157] D. Shen, M. Xiao, Y. Xiao, G. Zou, L. Hu, B. Zhao, L. Liu, W.W. Duley, Y.N. Zhou, Self-powered, rapid-response, and highly flexible humidity sensors based on moisture-dependent voltage generation, ACS Appl. Mater. Interfaces 11 (15) (2019) 14249–14255, https://doi.org/10.1021/acsami.9b01523.
[158] D. Zhang, H. Chen, P. Li, D. Wang, Z. Yang, Humidity sensing properties of metal organic framework-derived hollow ball-like TiO_2 coated QCM sensor, IEEE Sens. J. 19 (8) (2019) 2909–2915, https://doi.org/10.1109/JSEN.2018.2890738.
[159] P.V. Shinde, S. Gagare, C.S. Rout, D.J. Late, TiO_2 nanoflowers based humidity sensor and cytotoxic activity, RSC Adv. 10 (49) (2020) 29378–29384, https://doi.org/10.1039/D0RA05007E.
[160] H. Zhang, Z. Li, L. Liu, C. Wang, Y. Wei, A.G. MacDiarmid, Mg^{2+}/Na^{+}-doped rutile TiO_2 nanofiber mats for high-speed and anti-fogged humidity sensors, Talanta 79 (3) (2009) 953–958, https://doi.org/10.1016/j.talanta.2009.05.035.
[161] C. Khatuaa, I. Chinyaa, D. Sahab, S. Das, R. Sen, A. Dhar, Modified clad optical fibre coated with PVA/TiO_2 nano composite for humidity sensing application, Int. J. Smart Sens. Intell. Syst. 8 (3) (2015) 1424–1442, https://doi.org/10.21307/ijssis-2017-813.
[162] S. Mallick, Z. Ahmad, F. Touati, R.A. Shakoor, Improvement of humidity sensing properties of PVDF-TiO_2 nanocomposite films using acetone etching, Sens. Actuators B Chem. 288 (2019) 408–413, https://doi.org/10.1016/j.snb.2019.03.034.
[163] L. Gu, K. Zheng, Y. Zhou, J. Li, X. Mo, G.R. Patzke, G. Chen, Humidity sensors based on ZnO/TiO_2 core/shell nanorod arrays with enhanced sensitivity, Sens. Actuators B Chem. 159 (1) (2011) 1–7, https://doi.org/10.1016/j.snb.2010.12.024.
[164] R. Aneesh, S.K. Khijwania, Titanium dioxide nanoparticle based optical fiber humidity sensor with linear response and enhanced sensitivity, Appl. Opt. 51 (12) (2012) 2164, https://doi.org/10.1364/AO.51.002164.
[165] H. Jeong, Y. Noh, D. Lee, Highly stable and sensitive resistive flexible humidity sensors by means of roll-to-roll printed electrodes and flower-like TiO_2 nanostructures, Ceram. Int. 45 (1) (2019) 985–992, https://doi.org/10.1016/j.ceramint.2018.09.276.
[166] Q. Qi, Y. Feng, T. Zhang, X. Zheng, G. Lu, Influence of crystallographic structure on the humidity sensing properties of KCl-doped TiO_2 nanofibers, Sens. Actuators B Chem. 139 (2) (2009) 611–617, https://doi.org/10.1016/j.snb.2009.03.041.
[167] Z. Li, H. Zhang, W. Zheng, W. Wang, H. Huang, C. Wang, A.G. MacDiarmid, Y. Wei, Highly sensitive and stable humidity nanosensors based on LiCl doped TiO_2 electrospun nanofibers, J. Am. Chem. Soc. 130 (15) (2008) 5036–5037, https://doi.org/10.1021/ja800176s.
[168] W.D. Lin, C.T. Liao, T.C. Chang, S.H. Chen, R.J. Wu, Humidity sensing properties of novel graphene/TiO_2 composites by sol–gel process, Sens. Actuators B Chem. 209 (2015) 555–561, https://doi.org/10.1016/j.snb.2014.12.013.
[169] A. Sun, L. Huang, Y. Li, Study on humidity sensing property based on TiO_2 porous film and polystyrene sulfonic sodium, Sens. Actuators B Chem. 139 (2) (2009) 543–547, https://doi.org/10.1016/j.snb.2009.03.064.
[170] F.A. Akgul, C. Gumus, A.O. Er, A.H. Farha, G. Akgul, Y. Ufuktepe, Z. Liu, Structural and electronic properties of SnO_2,", J. Alloys Compd 579 (2013) 50–56, https://doi.org/10.1016/j.jallcom.2013.05.057.
[171] A. Togo, F. Oba, I. Tanaka, K. Tatsumi, First-principles calculations of native defects in tin monoxide, Phys. Rev. B 74 (19) (2006), 195128, https://doi.org/10.1103/PhysRevB.74.195128.
[172] J.H. Shin, J.Y. Song, Y.H. Kim, H.M. Park, Low temperature and self-catalytic growth of tetragonal SnO nanobranch, Mater. Lett. 64 (9) (2010) 1120–1122, https://doi.org/10.1016/j.matlet.2010.02.028.
[173] H.C. Chiu, C.S. Yeh, Hydrothermal synthesis of SnO_2 nanoparticles and their gas-sensing of alcohol, J. Phys. Chem. C 111 (20) (2007) 7256–7259, https://doi.org/10.1021/jp0688355.
[174] C.A. Ibarguen, A. Mosquera, R. Parra, M.S. Castro, J.E. Rodríguez-Páez, Synthesis of SnO_2 nanoparticles through the controlled precipitation route, Mater. Chem. Phys. 101 (2–3) (2007) 433–440, https://doi.org/10.1016/j.matchemphys.2006.08.003.
[175] M. Aziz, S. Saber Abbas, W.R. Wan Baharom, Size-controlled synthesis of SnO_2 nanoparticles by sol–gel method, Mater. Lett. 91 (2013) 31–34, https://doi.org/10.1016/j.matlet.2012.09.079.
[176] Y. Zhang, K. Yu, G. Li, D. Peng, Q. Zhang, F. Xu, W. Bai, S. Ouyang, Z. Zhu, Synthesis and field emission of patterned SnO_2 nanoflowers, Mater. Lett. 60 (25–26) (2006) 3109–3112, https://doi.org/10.1016/j.matlet.2006.02.053.
[177] F. P. Delgado, W.A. Flores, M.M. Yoshida, A.A. Elguezabal, P. Santiago, R. Dia, J. A. Ascencio, Structural analysis and growing mechanisms for long SnO_2 nanorods synthesized by spray pyrolysis, Nanotechnology 16 (6) (2005) 688–694, https://doi.org/10.1088/0957-4484/16/6/011.
[178] A. Amutha, M. Vigneswari, S. Amirthapandian, B.K. Panigrahi, P. Thangadurai, Facile synthesis and characterization of microstructure and optical properties of pure and Zn doped SnO_2 nanorods, J. Clust. Sci. (2021), https://doi.org/10.1007/s10876-021-02089-2.
[179] F. Hernandezramirez, A. Tarancon, O. Casals, J. Arbiol, A. Romanorodriguez, J. Morante, High response and stability in CO and humidity measures using a single SnO_2 nanowire, Sens. Actuators B Chem. 121 (1) (2007) 3–17, https://doi.org/10.1016/j.snb.2006.09.015.
[180] Y.P. Sun, Y.F. Zhao, H. Sun, F.C. Jia, P. Kumar, B. Liu, Synthesis and room-temperature H_2S sensing of Pt nanoparticle-functionalized SnO_2 mesoporous nanoflowers, J. Alloys Compd. 842 (2020), 155813, https://doi.org/10.1016/j.jallcom.2020.155813.
[181] M.G. Masteghin, R.A. Silva, D.C. Cox, D.R.M. Godoi, S.R.P. Silva, M.O. Orlandi, The role of surface stoichiometry in NO_2 gas sensing using single and multiple nanobelts of tin oxide, Phys. Chem. Chem. Phys. 23 (16) (2021) 9733–9742, https://doi.org/10.1039/D1CP00662B.
[182] X. Chen, R. Cai, P. Liu, W. Liu, K. Liu, Preparation and electrochemical performance of reduced graphene and SnO_2 nanospheres composite materials for lithium-ion batteries and sodium-ion batteries, ChemistrySelect 6 (13) (2021) 3192–3198, https://doi.org/10.1002/slct.202100877.
[183] N. Gao, H.Y. Li, W. Zhang, Y. Zhang, Y. Zeng, H. Zhixiang, J. Liu, J. Jiang, L. Miao, F. Yi, H. Liu, QCM-based humidity sensor and sensing properties employing colloidal SnO_2 nanowires, Sens. Actuators B Chem. 293 (2019) 129–135, https://doi.org/10.1016/j.snb.2019.05.009. Aug.
[184] S. Karthick, H.S. Lee, S.J. Kwon, R. Natarajan, V. Saraswathy, Standardization, calibration, and evaluation of tantalum-nano rGO-SnO_2 composite as a possible candidate material in humidity sensors, Sensors 16 (12) (2016) 2079, https://doi.org/10.3390/s16122079.
[185] R.A. Evarestov, A.V. Bandura, E.V. Proskurov, Plain DFT and hybrid HF-DFT LCAO calculations of SnO_2 (110) and (100) bare and hydroxylated surfaces, Phys. Status Solidi B 243 (8) (2006) 1823–1834, https://doi.org/10.1002/pssb.200541412.
[186] P.J.D. Lindan, Water chemistry at the SnO_2(110) surface: the role of inter-molecular interactions and surface geometry, Chem. Phys. Lett. 328 (4–6) (2000) 325–329, https://doi.org/10.1016/S0009-2614(00)00963-5.
[187] M. Batzill, W. Bergermayer, I. Tanaka, U. Diebold, Tuning the chemical functionality of a gas sensitive material: water adsorption on SnO_2(101), Surf. Sci. 600 (4) (2006) 29–32, https://doi.org/10.1016/j.susc.2005.11.034.
[188] W. Li, J. Liu, C. Ding, G. Bai, J. Xu, Q. Ren, J. Li, Fabrication of ordered SnO_2 nanostructures with enhanced humidity sensing performance, Sensors 17 (10) (2017) 2392, https://doi.org/10.3390/s17102392.
[189] X. Qu, M.H. Wang, Y. Chen, W.J. Sun, R. Yang, H.P. Zhang, Facile synthesis of hierarchical SnO_2 twig-like microstructures and their applications in humidity sensors, Mater. Lett. 186 (2017) 182–185, https://doi.org/10.1016/j.matlet.2016.09.131.
[190] M. Parthibavarman, V. Hariharan, C. Sekar, High-sensitivity humidity sensor based on SnO_2 nanoparticles synthesized by microwave irradiation method, Mater. Sci. Eng. C 31 (5) (2011) 840–844, https://doi.org/10.1016/j.msec.2011.01.002.
[191] M. Yin, F. Yang, Z. Wang, M. Zhu, M. Liu, X. Xu, Z. Li, A fast humidity sensor based on Li^{+}-doped SnO_2 one-dimensional porous nanofibers, Materials 10 (5) (2017) 535, https://doi.org/10.3390/ma10050535.
[192] V.K. Tomer, S. Duhan, A facile nanocasting synthesis of mesoporous Ag-doped SnO_2 nanostructures with enhanced humidity sensing performance, Sens. Actuators B Chem. 223 (2016) 750–760, https://doi.org/10.1016/j.snb.2015.09.139.
[193] P.G. Su, P.H. Lu, Electrical and humidity-sensing properties of impedance-type humidity sensors that were made of Ag Microwires/PPy/SnO_2 ternary composites, Chemosensors 8 (4) (2020) 92, https://doi.org/10.3390/chemosensors8040092.
[194] D. Zhang, Y. Sun, P. Li, Y. Zhang, Facile fabrication of MoS_2 -modified SnO_2 hybrid nanocomposite for ultrasensitive humidity sensing, ACS Appl. Mater. Interfaces 8 (22) (2016) 14142–14149, https://doi.org/10.1021/acsami.6b02206.
[195] H. Feng, C. Li, T. Li, F. Diao, T. Xin, B. Liu, Y. Wang, Three-dimensional hierarchical SnO_2 dodecahedral nanocrystals with enhanced humidity sensing properties, Sens. Actuators B Chem. 243 (2017) 704–714, https://doi.org/10.1016/j.snb.2016.12.043.
[196] M. Zhuo, Y. Chen, J. Sun, H Zhang, D. Guo, H. Zhang, Q. Li, T. Wang, Q. Wan, Humidity sensing properties of a single Sb doped SnO_2 nanowire field effect transistor, Sens. Actuators B Chem. 186 (2013) 78–83, https://doi.org/10.1016/j.snb.2013.05.043.

[197] X. Song, Q. Qi, T. Zhang, C. Wang, A humidity sensor based on KCl-doped SnO_2 nanofibers, Sens. Actuators B Chem. 138 (1) (2009) 368–373, https://doi.org/10.1016/j.snb.2009.02.027.

[198] S.K. Shukla, S.K. Shukla, P.P. Govender, E.S. Agorku, A resistive type humidity sensor based on crystalline tin oxide nanoparticles encapsulated in polyaniline matrix, Microchim. Acta 183 (2) (2016) 573–580, https://doi.org/10.1007/s00604-015-1678-2.

[199] O. Sisman, N. Kaur, G. Sberveglieri, E. Núñez-Carmona, V. Sberveglieri, E. Comini, UV-enhanced humidity sensing of chitosan–SnO_2 hybrid nanowires, Nanomaterials 10 (2) (2020) 329, https://doi.org/10.3390/nano10020329.

[200] L. Lu, J. Liu, Q. Li, Z. Yi, J. Liu, X. Wang, X. Chen, B. Yang, High performance SnO_2 /MoS_2 -based surface acoustic wave humidity sensor with good linearity, IEEE Sens. J. 19 (23) (2019) 11027–11033, https://doi.org/10.1109/JSEN.2019.2937186.

[201] Y. Chen, Y. Pei, Z. Jiang, Z. Shi, J. Xu, D. Wu, T. Xu, Y. Tian, X. Wang, X. Li, Humidity sensing properties of the hydrothermally synthesized WS_2-modified SnO_2 hybrid nanocomposite, Appl. Surf. Sci. 447 (2018) 325–330, https://doi.org/10.1016/j.apsusc.2018.03.154.

[202] A.S. Ismail, M.H. Mamat, M.F. Malek, M.M. Yusoff, R. Mohamed, N.D.M. Sin, A. B. Suriani, M. Rusop, Heterogeneous SnO_2/ZnO nanoparticulate film: facile synthesis and humidity sensing capability, Mater. Sci. Semicond. Process. 81 (2018) 127–138, https://doi.org/10.1016/j.mssp.2018.03.022.

[203] C.H.B. Ng, W.Y. Fan, Shape evolution of Cu_2O nanostructures via kinetic and thermodynamic controlled growth, J. Phys. Chem. B 110 (42) (2006) 20801–20807, https://doi.org/10.1021/jp061835k.

[204] N.R. Dhineshbabu, V. Rajendran, N. Nithyavathy, R. Vetumperumal, Study of structural and optical properties of cupric oxide nanoparticles, Appl. Nanosci. 6 (6) (2016) 933–939, https://doi.org/10.1007/s13204-015-0499-2.

[205] Y. Peng, Z. Zhang, T. Viet Pham, Y. Zhao, P. Wu, J. Wang, Density functional theory analysis of dopants in cupric oxide, J. Appl. Phys. 111 (10) (2012), 103708, https://doi.org/10.1063/1.4719059.

[206] A. Ananth, S. Dharaneedharan, M.S. Heo, Y.S. Mok, Copper oxide nanomaterials: synthesis, characterization and structure-specific antibacterial performance, Chem. Eng. J. 262 (2015) 179–188, https://doi.org/10.1016/j.cej.2014.09.083.

[207] Z.N. Kayani, M. Umer, S. Riaz, S. Naseem, Characterization of copper oxide nanoparticles fabricated by the sol–gel method, J. Electron. Mater. 44 (10) (2015) 3704–3709, https://doi.org/10.1007/s11664-015-3867-5.

[208] D. Majumdar, T.A. Shefelbine, T.T. Kodas, H.D. Glicksman, Copper (I) oxide powder generation by spray pyrolysis, J. Mater. Res. 11 (11) (1996) 2861–2868, https://doi.org/10.1557/JMR.1996.0361.

[209] L. Chen, S. Shet, H. Tang, H. Wang, T. Deutsch, Y. Yan, J. Turnera, M. A.-Jassim, Electrochemical deposition of copper oxide nanowires for photoelectrochemical applications, J. Mater. Chem. 20 (33) (2010) 6962, https://doi.org/10.1039/c0jm01228a.

[210] A. Rydosz, K. Dyndał, W. Andrysiewicz, D. Grochala, K. Marszałek, GLAD magnetron sputtered ultra-thin copper oxide films for gas-sensing application, Coatings 10 (4) (2020) 378, https://doi.org/10.3390/coatings10040378.

[211] A.S. Ethiraj, D.J. Kang, Synthesis and characterization of CuO nanowires by a simple wet chemical method, Nanoscale Res. Lett. 7 (1) (2012) 70, https://doi.org/10.1186/1556-276X-7-70.

[212] C. Yang, X. Su, F. Xiao, J. Jian, J. Wang, Gas sensing properties of CuO nanorods synthesized by a microwave-assisted hydrothermal method, Sens. Actuators B Chem. 158 (1) (2011) 299–303, https://doi.org/10.1016/j.snb.2011.06.024.

[213] R.M. Mohamed, F.A. Harraz, A. Shawky, CuO nanobelts synthesized by a template-free hydrothermal approach with optical and magnetic characteristics, Ceram. Int. 40 (1) (2014) 2127–2133, https://doi.org/10.1016/j.ceramint.2013.07.129.

[214] N. Mukherjee, B. Show, S. K. Maji, U. Madhu, S. K. Bhar, B. C. Mitra, G. G. Khan, A. Mondal, CuO nano-whiskers: Electrodeposition, Raman analysis, photoluminescence study and photocatalytic activity, Mater. Lett. 65 (21–22) (2011) 3248–3250, https://doi.org/10.1016/j.matlet.2011.07.016.

[215] L. Hou, C. Zhang, L. Li, C. Du, X. Li, X.-F. Kang, W. Chen, CO gas sensors based on p-type CuO nanotubes and CuO nanocubes: morphology and surface structure effects on the sensing performance, Talanta 188 (2018) 41–49, https://doi.org/10.1016/j.talanta.2018.05.059.

[216] W. Jia, E. Reitz, P. Shimpi, E.G. Rodriguez, P.X. Gao, Y. Lei, Spherical CuO synthesized by a simple hydrothermal reaction: concentration-dependent size and its electrocatalytic application, Mater. Res. Bull. 44 (8) (2009) 1681–1686, https://doi.org/10.1016/j.materresbull.2009.04.003.

[217] R. Katal, S. Masudy-panah, E.Y.J. Kong, N. Dasineh Khiavi, M.H.D. Abadi Farahani, X. Gong, Nanocrystal-engineered thin CuO film photocatalyst for visible-light-driven photocatalytic degradation of organic pollutant in aqueous solution, Catal. Today 340 (2020) 236–244, https://doi.org/10.1016/j.cattod.2018.12.019.

[218] H. Kidowaki, T. Oku, T. Akiyama, A. Suzuki, B. Jeyadevan, J. Cuya, Fabrication and characterization of CuO-based solar cells, J. Mater. Sci. Res. 1 (1) (2011) p138, https://doi.org/10.5539/jmsr.v1n1p138.

[219] Z. Deng, Z. Ma, Y. Li, Y. Li, L. Chen, X. Yang, H.E. Wang, B.L. Su, Boosting lithium-ion storage capability in CuO nanosheets via synergistic engineering of defects and pores, Front. Chem. 6 (2018) 428, https://doi.org/10.3389/fchem.2018.00428.

[220] R. Nitta, H.E. Lin, Y. Kubota, T. Kishi, T. Yano, N. Matsushita, CuO nanostructure-based flexible humidity sensors fabricated on PET substrates by spin-spray method, Appl. Surf. Sci. 572 (2022), 151352, https://doi.org/10.1016/j.apsusc.2021.151352.

[221] J. Hu, D. Li, J.G. Lu, R. Wu, Effects on electronic properties of molecule adsorption on CuO surfaces and nanowires, J. Phys. Chem. C 114 (40) (2010) 17120–17126, https://doi.org/10.1021/jp1039089.

[222] X. Yu, X. Zhang, H. Wang, G. Feng, High coverage water adsorption on the CuO (111) surface, Appl. Surf. Sci. 425 (2017) 803–810, https://doi.org/10.1016/j.apsusc.2017.07.086.

[223] J. Zhang, R. Zhang, B. Wang, L. Ling, Insight into the adsorption and dissociation of water over different CuO(111) surfaces: the effect of surface structures, Appl. Surf. Sci. 364 (2016) 758–768, https://doi.org/10.1016/j.apsusc.2015.12.211.

[224] J. Xu, K. Yu, J. Wu, D. Shang, L. Li, Y. Xu, Z. Zhu, Synthesis, field emission and humidity sensing characteristics of honeycomb-like CuO, J. Phys. Appl. Phys. 42 (7) (2009), 075417, https://doi.org/10.1088/0022-3727/42/7/075417.

[225] Y. Gu, H. Jiang, Z. Ye, N. Sun, X. Kuang, W. Liu, G. Li, X. Song, L. Zhang, W. Bai, X. Tang, Impact of size on humidity sensing property of copper oxide nanoparticles, Electron. Mater. Lett. 16 (1) (2020) 61–71, https://doi.org/10.1007/s13391-019-00181-4.

[226] Z. Wang, Y. Xiao, X. Cui, P. Cheng, B. Wang, Y. Gao, X. Li, T. Yang, T. Zhang, G. Lu, Humidity-sensing properties of urchinlike CuO nanostructures modified by reduced graphene oxide, ACS Appl. Mater. Interfaces 6 (6) (2014) 3888–3895, https://doi.org/10.1021/am404858z.

[227] C. Yuan, Y. Xu, Y. Deng, N. Jiang, N. He, L. Dai, CuO based inorganic–organic hybrid nanowires: a new type of highly sensitive humidity sensor, Nanotechnology 21 (41) (2010), 415501, https://doi.org/10.1088/0957-4484/21/41/415501.

[228] A. Zainelabdin, G. Amin, S. Zaman, O. Nur, J. Lu, L. Hultman, M. Willande, CuO/ZnO Nanocorals synthesis via hydrothermal technique: growth mechanism and their application as humidity sensor, J. Mater. Chem. 22 (23) (2012) 11583, https://doi.org/10.1039/c2jm16597j.

[229] C. Ashok, K. Venkateswara Rao, Microwave-assisted synthesis of CuO/TiO_2 nanocomposite for humidity sensor application, J. Mater. Sci. Mater. Electron. 27 (8) (2016) 8816–8825, https://doi.org/10.1007/s10854-016-4907-5.

[230] W. Ahmad, B. Jabbar, I. Ahmad, B.M. Jan, M.M. Stylianakis, G. Kenanakis, R. Ikram, Highly sensitive humidity sensors based on polyethylene oxide/CuO/multi walled carbon nanotubes composite nanofibers, Materials 14 (4) (2021) 1037, https://doi.org/10.3390/ma14041037.

[231] S. Ashokan, P. Jayamurugan, V. Ponnuswamy, Effects of CuO and oxidant on the morphology and conducting properties of PANI:CuO hybrid nanocomposites for humidity sensor application, Polym. Sci. Ser. B 61 (1) (2019) 86–97, https://doi.org/10.1134/S1560090419010020.

[232] H. Ghannam, A. Chahboun, M. Turmine, Wettability of zinc oxide nanorod surfaces, RSC Adv. 9 (65) (2019) 38289–38297, https://doi.org/10.1039/C9RA05378F.

[233] M.K. Lee, Y.C. Park, Contact angle relaxation and long-lasting hydrophilicity of sputtered anatase TiO_2 thin films by novel quantitative XPS analysis, Langmuir 35 (6) (2019) 2066–2077, https://doi.org/10.1021/acs.langmuir.8b03258.

[234] M. Khalfallah, N. Guermat, W. Daranfed, N. Bouarissa, H. Bakhti, Hydrophilic nickel doped porous SnO_2 thin films prepared by spray pyrolysis, Phys. Scr. 95 (9) (2020), 095805, https://doi.org/10.1088/1402-4896/aba8c5.

[235] Talinungsang, D. Dhar Purkayastha, M.G. Krishna, Dopant controlled photoinduced hydrophilicity and photocatalytic activity of SnO_2 thin films, Appl. Surf. Sci. 447 (2018) 724–731, https://doi.org/10.1016/j.apsusc.2018.04.028.

[236] M.Y. Ghotbi, Z. Rahmati, Nanostructured copper and copper oxide thin films fabricated by hydrothermal treatment of copper hydroxide nitrate, Mater. Des. 85 (2015) 719–723, https://doi.org/10.1016/j.matdes.2015.07.081.

[237] N. Al armouzi, M. Mabrouki, Effect of annealing temperature on the structural, physical, chemical, and wetting properties of copper oxide thin films, Mater. Today Proc. 13 (2019) 771–776, https://doi.org/10.1016/j.matpr.2019.04.039.

[238] H.T. Hsueh, T.J. Hsueh, S.J. Chang, F.Y. Hung, T.Y. Tsai, W.Y. Weng, C.L. Hsu, B. T. Dai, CuO nanowire-based humidity sensors prepared on glass substrate, Sens. Actuators B Chem. 156 (2) (2011) 906–911, https://doi.org/10.1016/j.snb.2011.03.004. Aug.

[239] S.B Wang, C.H. Hsiao, S.J. Chang, K.T. Lam, K.H. Wen, S.J. Young, S.C. Hung, B. R. Huang, CuO nanowire-based humidity sensor, IEEE Sens. J. 12 (6) (2012) 1884–1888, https://doi.org/10.1109/JSEN.2011.2180375.

[240] J. Wang, W. Zeng, Research progress on humidity-sensing properties of Cu-based humidity sensors: a review, J. Sens. 2022 (2022) 1–29, https://doi.org/10.1155/2022/7749890. May.

[241] Y. Xiao, D. Shen, G. Zou, A. Wu, L. Liu, W.W. Duley, Y.N. Zhou, Self-powered, flexible and remote-controlled breath monitor based on TiO_2 nanowire networks, Nanotechnology 30 (32) (2019), 325503, https://doi.org/10.1088/1361-6528/ab1b93.

[242] L. Gong, X. Wang, D. Zhang, X. Ma, S. Yu, Flexible wearable humidity sensor based on cerium oxide/graphitic carbon nitride nanocomposite self-powered by motion-driven alternator and its application for human physiological detection, J. Mater. Chem. A 9 (9) (2021) 5619–5629, https://doi.org/10.1039/D0TA11578A.

[243] D. Zhang, Z. Xu, Z. Yang, X. Song, High-performance flexible self-powered tin disulfide nanoflowers/reduced graphene oxide nanohybrid-based humidity sensor driven by triboelectric nanogenerator, Nano Energy 67 (2020), 104251, https://doi.org/10.1016/j.nanoen.2019.104251.

[244] Y. Pang, J. Jian, T. Tu, Z. Yang, J. Ling, Y. Li, X. Wang, Y. Qiao, H. Tian, Y. Yang, T.L. Ren, Wearable humidity sensor based on porous graphene network for respiration monitoring, Biosens. Bioelectron. 116 (2018) 123–129, https://doi.org/10.1016/j.bios.2018.05.038.

[245] D. Wang, D. Zhang, P. Li, Z. Yang, Q. Mi, L. Yu, Electrospinning of flexible poly (vinyl alcohol)/MXene nanofiber-based humidity sensor self-powered by

monolayer molybdenum diselenide piezoelectric nanogenerator, Nano-Micro Lett. 13 (1) (2021) 57, https://doi.org/10.1007/s40820-020-00580-5.
[246] D. Zhang, Z. Yang, S. Yu, Q. Mi, Q. Pan, Diversiform metal oxide-based hybrid nanostructures for gas sensing with versatile prospects, Coord. Chem. Rev. 413 (2020), 213272, https://doi.org/10.1016/j.ccr.2020.213272.
[247] D. Wang, D. Zhang, Y. Yang, Q. Mi, J. Zhang, L. Yu, Multifunctional latex/polytetrafluoroethylene-based triboelectric nanogenerator for self-powered organ-like MXene/metal–organic framework-derived CuO nanohybrid ammonia sensor, ACS Nano 15 (2) (2021) 2911–2919, https://doi.org/10.1021/acsnano.0c09015.

Sensors & Actuators: B. Chemical 283 (2019) 61–68

Contents lists available at ScienceDirect

Sensors and Actuators B: Chemical

journal homepage: www.elsevier.com/locate/snb

Enhanced humidity resistance of porous SiNWs via OTS functionalization for rarefied NO_2 detection

Yuxiang Qin[a,b,c,*], Yunqing Jiang[a], Liming Zhao[a]

[a] School of Microelectronics, Tianjin University, Tianjin 300072, China
[b] Tianjin Key Laboratory of Imaging and Sensing Microelectronic Technology, Tianjin University, Tianjin 300072, China
[c] Key Laboratory for Advanced Ceramics and Machining Technology, Ministry of Education, School of Materials Science and Engineering, Tianjin University, Tianjin 300072, China

ARTICLE INFO

Keywords:
Porous silicon nanowires
Humidity resistance
Gas detection
Octadecyltrichlorosilane

ABSTRACT

Porous silicon nanowires (SiNWs) is considered as one of promising candidates for high-performance NO_2 sensor application in human health and quality life owing to its fast response and stable operation at room temperature. However, the development of SiNWs-based gas sensors for stable detection of ppb-level NO_2 in practical high-humidity environments remains a challenge. In this study, octadecyltrichlorosilane (OTS) was utilized to modify partially insensitive SiNWs surface with the Si-O bond, yielding an immunity to high humidity in gas detection process. The modified OTS provides an umbrella-like barrier blocking water molecules attraction on the nanowire surface which disinhibits the main adsorption sites and sensing channels of the sensor. The OTS-modified porous SiNWs (OTS/SiNWs) sensor exhibited a practical capability to detect 5 ppb NO_2 at humidity of 25%–55% RH and a response of 1.8% to 50 ppb NO_2 at 75% RH at room temperature. Moreover, the fast response/recovery characteristics and good reversibility of the OTS/SiNWs sensor was observed even under high humidity conditions. In addition, the OTS/SiNWs sensor displayed excellent stability to humidity variations. The enhanced gas sensing performance is attributed to a marriage of porous structure of SiNWs and OTS. The simple modification for SiNWs-based gas sensor will broaden new avenues, not only in chemical trace detection, but also in high humidity health monitoring.

1. Introduction

Gas sensors are attracting major interest due to their significance for security, environment, healthy and comfort [1–13]. Developing gas sensors with high performance, low power, and easy integration is demanded urgently for modern intelligent sensors application. Intelligent gas sensors in human well-being should meet higher requirements such as miniaturization, precision, energy saving and immunity to humidity [3,14–18]. For most of gas sensors, despite of their high sensitivity and fast response, external heating is generally required to guarantee the optimal properties of gas sensing at elevated temperatures [19–30]. Sensors with external heating not only reduce portability but also increase thermal safety hazards for practical applications. The effective and direct way to solve this problem is to develop gas sensors that can operate at room temperature.

Silicon nanowires-based gas sensors have been considered as extremely promising candidates for the next generation gas sensor with high sensitivity at room temperature, as well as with the advantages of good compatibility and high specific surface area for gas adsorption [31]. However, as a gas sensor operating at room temperature, the silicon nanowires-based gas sensor have to confront a key problem that is the interference of ambient humidity especially for the severe performance degradation or even invalid in high humidity applications [15,17,32,33]. In et al. [34] highlighted silicon nanowires arrays have the capability for detecting the lowest concentration level of 10 ppb NO_2 at < 10% RH but only the response towards 500 ppb and 1 ppm NO_2 were shown in moisture air (30% RH). Fahad et al. reported a 3.5-nm-thin silicon channel transistors which showed a weak response (≈20%) to 1 ppm NO_2 at 45 ~ 50% RH [35]. Besides, the work of M Gao et al. about silicon-based sensors also demonstrated that, when operating under high humidity (80% RH) conditions, the lower detection limit of the sensor stagnates at a large ppm level for other gas detection [36]. Despite important advances made in silicon nanowires-based gas sensors, the development of silicon nanowires-based gas sensors for stable detection of ppb-level NO_2 in high-humidity environments remains a challenge. The main reason for interference of

* Corresponding author at: School of Microelectronics, Tianjin University, Weijin Road, NO. 92, Nankai District, Tianjin 300072, China.
E-mail address: qinyuxiang@tju.edu.cn (Y. Qin).

https://doi.org/10.1016/j.snb.2018.12.013
Received 14 August 2018; Received in revised form 3 December 2018; Accepted 3 December 2018
Available online 04 December 2018
0925-4005/ © 2018 Elsevier B.V. All rights reserved.

humidity is that the water molecule preferentially occupies the adsorption sites and interacts with the gas sensors to generate electrical signal interference because of the humidity sensing of silicon surface [37–39]. Therefore, the excellent ability against water molecules is crucial for silicon nanowires-based gas sensors working under high relative humidity in practical application.

An promising approach in the use of superhydrophobic structure for reducing interference of humidity was described by Wu et al. [19]. In this aspect, octadecyltrichlorosilane (OTS) has become an emerging and effective strategy to functionalize silicon-based materials with unique superhydrophobic structures [40–45]. OTS can combine with SiO_2 to form a self-assembled monolayer (SAM) which could reduce the surface energy of silicon surface and then make the surface hydrophobic [42]. However, the main drawback of OTS SAM is their tiny specific surface area and poor conductivity, thus making OTS incapable of producing rapid and strong sensing signals at room temperature.

Herein, to weaken the adverse effect of water molecules on gas-sensing response at high humidity, OTS functionalization was preformed to create a near superhydrophobic surface for porous silicon nanowires (SiNWs). In this study, an umbrella-like waterproof layer of OTS was formed on the surface of porous SiNWs arrays. The porous SiNWs, fabricated via metal assistant chemical etching (MACE), provide a high specific surface area and effective adsorption sites. The near superhydrophobic surfaces were created by applying OTS on insensitive surface with the Si-O bond, for offering the capability of stable gas detection under high humidity [44,45]. During modification, organic molecules hardly enter into the nanopores of porous SiNWs in a short time, ensuring the effectiveness of gas molecule adsorption channels in the as-formed OTS/SiNWs [40]. The OTS-functionalized SiNWs (OTS/SiNWs) could detect the trace NO_2 down to 5 ppb at 25%–55% RH. Due to the ingenious structure, the porous SiNWs functionalized with OTS displays a good response ($\Delta R/R_a$) of 1.8% towards 50 ppb NO_2 at 75% RH.

2. Experimental section

2.1. Fabrication of SiNWs and OTS/SiNWs

In our previous work, we have described an effective routine for fabricating porous silicon nanowires gas sensors with agglomeration structure [46,47]. Before etching, the cut Si pieces (1 x 2 cm) were sequentially washed in acetone, ethanol, hot mixture solution of H_2SO_4/H_2O_2 (volume ratio of 3:1), and deionized (DI) water for 10 min each. The cleaned Si pieces were dipped into 10% HF solution for 1 min, and then soaked in the mixed solution of 0.0035 M $AgNO_3$ and 6 M HF for another 1 min to modulate the surface state. MACE of SiNWs arrays were performed through immersing the pre-treated silicon wafer pieces in the mixture solution composed of 6 M HF and 0.8 M H_2O_2 for 1 h at room temperature. After the etching process, the as-prepared samples were rinsed thoroughly with DI water and then dipped into 44 wt% HNO_3 solution for 10 min to remove the metal Ag formed during SiNWs etching.

To prepare OTS-functionalized SiNWs, the MACE-induced SiNWs were dried in IR dryer for 5 min, followed by conjugation with 1 vol % OTS in toluene for 1 min at room temperature. Finally, the samples were cleaned by chloroform ($CHCl_3$) for three times and dried in air, yielding the OTS-functionalized porous SiNWs, which is named as OTS/SiNWs.

2.2. Characterization of SiNWs and OTS/SiNWs

Microstructure characterization and gas-sensing measurement: Field emission scanning electron microscope (FESEM, Hitachi S4800) and field emission transmission electron microscopy (FETEM, TECNAI G2F-20) were used to characterize the microstructure of as-fabricated samples. Fourier transform infrared spectroscope (Nicolet Avatar 370) was utilized for measurement of FT-IR spectra with IR radiation in the range of 700-3500 cm^{-1} using the KBr pellet method. The Brunauer-Emmett-Teller (BET) gas-sorption measurement (Quantachrome, Quadrasorb-S1) were carried out to obtain the specific surface area and the Barrett-Joyner-Halenda (BJH) method was used for analyzing the pore size distribution. A video-based contact angle measurement system (JY-82B, China) was employed for measuring the water contact angles (WCAs) of the samples and each sample were measured 3 times by dispensing 9 μL drops of DI water.

2.3. Fabrication and evaluation of sensor

Top point electrodes of Pt with thickness of about 500–600 nm were deposited on the surface of samples by DC magnetron sputtering to form a sensor device. The gas-sensing properties of the sensor samples were evaluated at room temperature with a homemade static gas-sensing characterization system consisting of a test chamber, a flat heating plate, a programmable digital multimeter with automatic range adjustment and a data acquisition system [48]. Commercial standard NO_2 gas (1000 ppm) was introduced into the test chamber by static volumetric method. That is, a predetermined volume of gas was injected into the chamber directly by a microsyringe to get the desired concentration. The resistance change of the sensor was continuously monitored and collected by a computer connected digital multimeter with sampling interval of 1 s. The test equipment is set in a humidity-controlled testing chamber, and during the measurement, the ambient relative humidity (RH) was varied from 25% to 75% (i.e., 25%, 35%, 45%, 55%, 65%, 75%, respectively). The error bars were obtained by repeated four experiments and averaged on the same sample.

3. Results and discussion

3.1. Characterization of OTS/SiNWs

Fig. 1 shows the ideal process for the fabrication of OTS-modified porous SiNWs (OTS/SiNWs) sensor with a good humidity resistance. Firstly, the porous SiNWs was fabricated by an well-established MACE method [46]. In air, SiNWs are oxidized partially to form a thin layer of SiO_2 on the surface, which is generally insensitive upon gas exposure. For bare SiNWs shown in Fig. 1, the water molecules adsorb on the nanowire surface and meanwhile interact with the adsorbed NO_2 molecules, not only occupying some effective sites for gas adsorption but also inducing large resistance signal interference [20,49]. However, the insensitive SiO_2 regions on the surface of SiNWs easily conjugate OTS with Si-O bond to achieve superhydrophobic properties [42,50]. After superhydrophobic treatment by OTS, the surface uneasily bonding to OTS, such as non-oxidized SiNWs surfaces or the channel of nanopores, is still bare and keeps active for gas adsorption (as shown in the ideal OTS/SiNWs model in Fig. 1). For OTS-functionalized SiNWs, i.e., OTS/SiNWs, the molecules of OTS modified on the surface of SiO_2 regions provide an umbrella-like barrier blocking water molecules attraction on the nanowire surface, which not only weakens the influence of ambient humidity but also assures the effective sites available for gas adsorption.

Our previous work revealed that the tip-cluster configuration in bundling nanowire array is a kind of highly sensitive structure for gas sensor applications. The same method was employed for fabricating the tip-cluster structure of porous SiNWs gas sensors and this structure shows nearly unchanged morphology after OTS functionalization, despite of the increased spacing among the bundles (Fig. 2(a) and (b)). The tip-cluster structure with the modification of OTS is more conducive to improving hydrophobic properties of the nanowires array, compared to vertical SiNWs [51]. The wettability is studied by comparing the water contact angles (WCAs) of porous SiNWs with the WCAs of the OTS/SiNWs. Fig. 2(c) shows that the SiNWs without OTS treatment has a small contact angle of 53°, indicating the surface of the

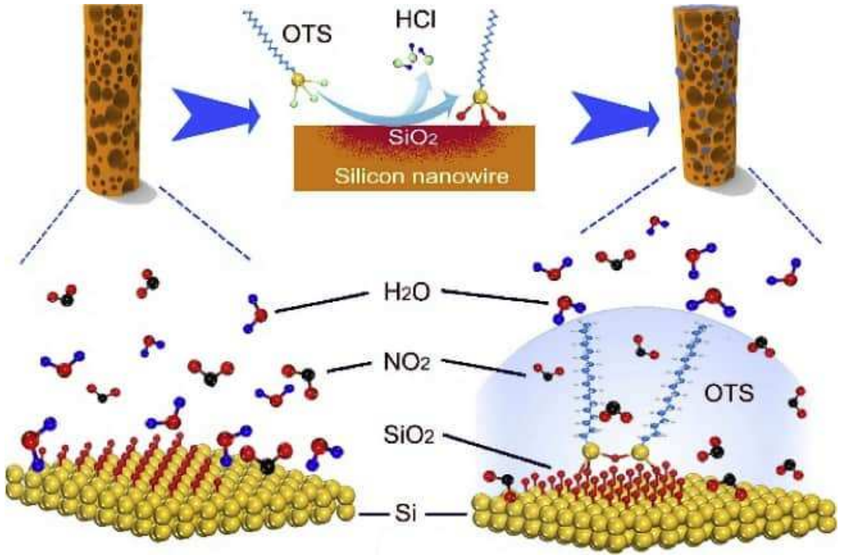

Fig. 1. Schematic illustration of construction of OTS-modified porous SiNWs for NO_2 detection under high humidity condition.

unfunctionalized SiNWs is hydrophilic (contact angle < 90°). Whereas, the porous SiNWs with 60 s-OTS modification exhibit a large contact angle of 146° as shown in Fig. 2(d), which is an indication of a near superhydrophobic surface (contact angle > 150°). Note that the contact angle is the average value of water droplets being dropped at three different positions of the same sample. The obvious water-repelling characteristic exhibited by OTS/SiNWs is thought to be capable of weakening the inhibitory effect of humidity on gas-sensing remarkably. Above measurements of contact angle confirm the high effectiveness of OTS functionalization in achieving perfect superhydrophobicity. In fact, fast covering of OTS on SiO_2 surface is easily achieved due to its growth kinetics [52]. Thus the wettability of SiNWs could be modulated conveniently by adjusting the treatment time of OTS simply. In the experiments, 10 s of OTS treatment can obtain a contact angle of 122° (see Fig.S1†), which is much larger than the angle from the untreated bare SiNWs (53°). However, the hydrophobicity of OTS/SiNWs can not be further improved significantly when the OTS treatment time is extended from 60 s to 300 s. The OTS/SiNWs with 300 s-OTS treatment shows a very similar contact angle as the 60s-functionalized sample (149° and 146° for 300 s- and 60 s-OTS treatment, respectively, see Fig. S1†).

The porous structure of the pure SiNWs and OTS/SiNWs was

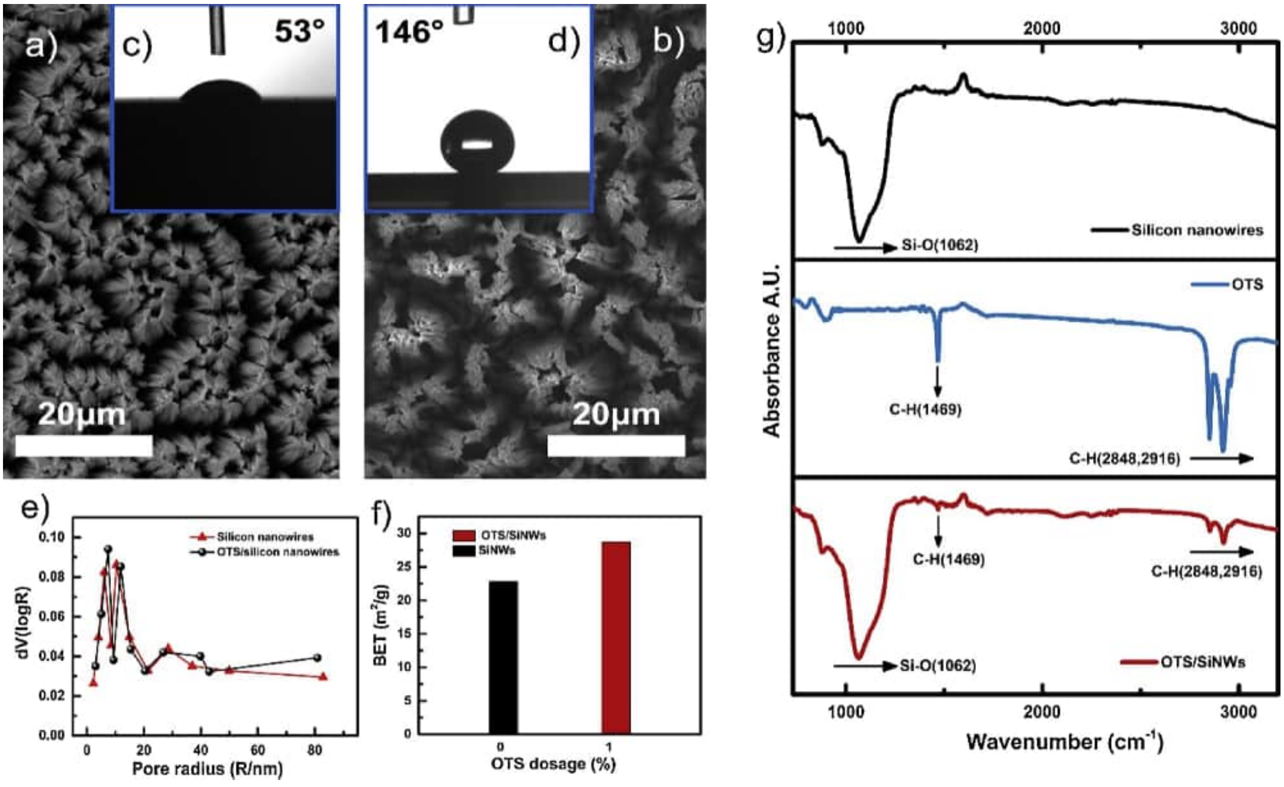

Fig. 2. (a, b) Top view SEM images of SiNWs and OTS/SiNWs. (c, d) The contact angles of water droplets on SiNWs and OTS/SiNWs. BJH pore-size distribution functions (e) and BET specific surface area (f) of SiNWs and OTS/SiNWs. (g) FTIR spectra of pure SiNWs (black line), the pure OTS (blue line), the OTS/SiNWs (red line) (For interpretation of the references to colour in this figure legend, the reader is referred to the web version of this article).

investigated by N_2 gas absorption at 77 K. As shown in Fig. 2(e), the pore size distribution of the porous SiNWs mainly concentrated in 5–20 nm without drastically varying before and after OTS modification. Due to the porous structure of SiNWs, the OTS/SiNWs exhibits a high BET specific surface area of 28.7 $m^2 g^{-1}$ with a tiny increase compared to the porous SiNWs (22.8 $m^2 g^{-1}$), which facilitates the surface adsorption of gas molecules (Fig. 2(f)).

FTIR is effectively utilized to investigate the alteration of chemical bonding induced by surface modification. As shown in Fig. 2(g), significant absorption peaks located in 700-3500 cm^{-1} are observed for both pure porous SiNWs and OTS/SiNWs. Between the pure SiNWs and OTS/SiNWs, the 1060 cm^{-1} peak is ascribed to Si–O stretching vibration [53]. The pure OTS shows strong absorbance in the ranges of 1469, 2848 and 2916 cm^{-1}, which is attributed to the C–H scissoring vibration, C–H symmetrical stretching vibration and C–H asymmetrical stretching vibration [41,53]. Compared to the pure SiNWs, the sharper and stronger vibration stretch peaks of C–H bonding at 2848 and 2916 cm^{-1} of the OTS/SiNWs demonstrates successful formation of OTS. The integrated area above the absorption curve is proportional to the monolayer density [41]. The absorption peaks of the OTS/SiNWs shift to lower intensity in contrast to those of the pure OTS, meaning the lower OTS density.

In addition, the OTS/SiNWs was inspected by TEM and high-resolution TEM (HRTEM). Well-defined silicon nanowires with diameter of 0.28-0.5 μm are observed, as shown in Fig. 3(a) and Fig. S2†. From the cross-sectional SEM view of the SiNWs and OTS/SiNWs in Fig. S2†, it can be observed that the detected signal is significantly weakened after OTS modification, suggesting uniform OTS assembly on the whole nanowires. In more detail, the enlarged TEM image in Fig. 3(b) shows that the nanopores in average size of 3–10 nm densely and uniformly distribute on whole nanowires, which is consistent with the pore size distribution results. HRTEM in Fig. 3(c) gives further characterization on the OTS/SiNWs, displaying Si lattice with a Si (100) interlayer spacing of 0.328 nm. It also shows clearly that the crystal lattice has been destroyed, due to etching into porous silicon nanowires.

3.2. Gas sensing performance and correlation with humidity

The gas sensing response of the SiNWs and OTS/SiNWs sensors is calculated based on a relative resistance change equation:

$$Response = \frac{\Delta R}{R_a} = \frac{|R_a - R_g|}{R_a} \times 100\% \quad (1)$$

where R_a and R_g are the resistance of the sensor before and after exposure to target gas (NO_2). As observed in Fig. 4(a) and (b), the OTS/SiNWs sensor shows a response value of 41.6% at 65% RH, which is four times higher than the free SiNWs without OTS functionalization (9.7%). It demonstrates that the surface OTS functionalization is highly beneficial for enhancement of gas sensing response of SiNWs in high humidity environment. As schematically illustrated in Fig.1, the response of bare SiNWs is affected by the interaction of the target gas and the water molecules, which not only interferes with the detection of the resistance change but also occupies the adsorption sites of NO_2 molecules. In particular, under the high humidity environment, NO_2 can react with the water molecules adsorbed on the sensing surface to form nitric acid ($3NO_2 + H_2O \rightarrow NO + 2HNO_3$), as it occurs in acid rain formation. The disproportionation reaction occurring under high humidity ambient, to some extent, obstructs the direct interaction between NO_2 and SiNWs. Moreover, the formed HNO_3 could induce an electron doping as behaved on the graphene [54], which weakens the magnitude of resistance decrease of SiNWs towards NO_2 exposure. However, after modifying OTS onto SiO_2 regions, the as-formed OTS/SiNWs have the water-repelling umbrella-like microstructure, which will block water molecules from attracting on the surface of SiNWs. Thus the sensing response of OTS-functionalized SiNWs at high humidity will be directly related to the OTS coverage-induced hydrophobicity; appropriate hydrophobicity (or OTS coverage on the gas-sensing surface) of SiNWs is beneficial to improve the response capability at high humidity. For instance, in present work, the OTS/SiNWs sensor with 60 s of OTS treatment shows the highest response magnitude (13.7% to 50 ppb NO_2) in contrast to the one with 10 s- or 300 s-OTS treatment when operating at high relative humidity of 65% (see Fig. S1).

Fig. 4(a) shows a response time of 15 s and a recovery time of 60 s for SiNWs sensor towards 250 ppb NO_2 at 65% RH. Here, the response time (t_s) and recovery time (t_c) are respectively defined as the times required for the sensor resistance to reach 90% of the equilibrium value after the test gas is introduced or released [55]. From the dynamic response curve of the OTS/SiNWs in Fig. 4(b), a shorter response time of 9 s is observed upon exposure to the same 250 ppb NO_2 at the same humidity of 65%. To give a reasonable comparison to the recovery time of both sensors, we define the mean recovery time (Δt_c) of sensor as follow:

$$\Delta t_c = \frac{t_c}{\Delta R / R_a} \quad (2)$$

where t_c is recovery time of the gas sensor, $\Delta R/R_a$ is the response of the gas sensor towards a special concentration of NO_2. Fig. S3† shows the calculated Δt_c values for the SiNWs and OTS/SiNWs sensors upon exposure to NO_2 gas ranging from 5 to 250 ppb at different relative humidity. As shown in Fig. S3†, the Δt_c values of OTS/SiNWs are always shorter than that of the SiNWs, indicating that the functionalization of OTS is conducive to reducing the interference of NO_2 molecule diffusion. Fig. 4(c) gives the dynamic responses of SiNWs and OTS/SiNWs sensor to 50 ppb NO_2 at a high humidity of 75%. As observed, the SiNWs is highly susceptible to relative humidity; it shows a negligible response at 75% RH. In contrast, the OTS/SiNWs still displays a response of 1.8% to 50 ppb NO_2 at such high humidity, demonstrating enhanced immunity of the OTS-functionalized SiNWs to high humidity.

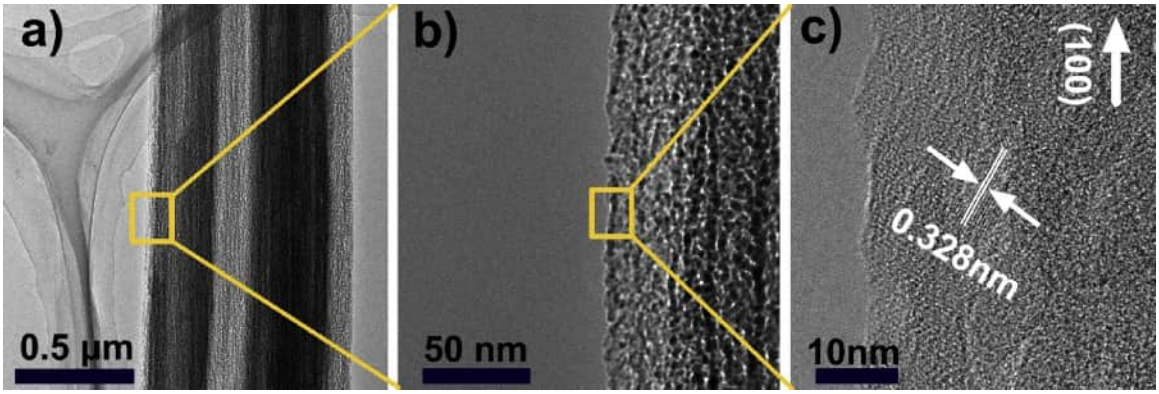

Fig. 3. TEM (a, b) and HRTEM (c) overview images of the OTS/SiNWs.

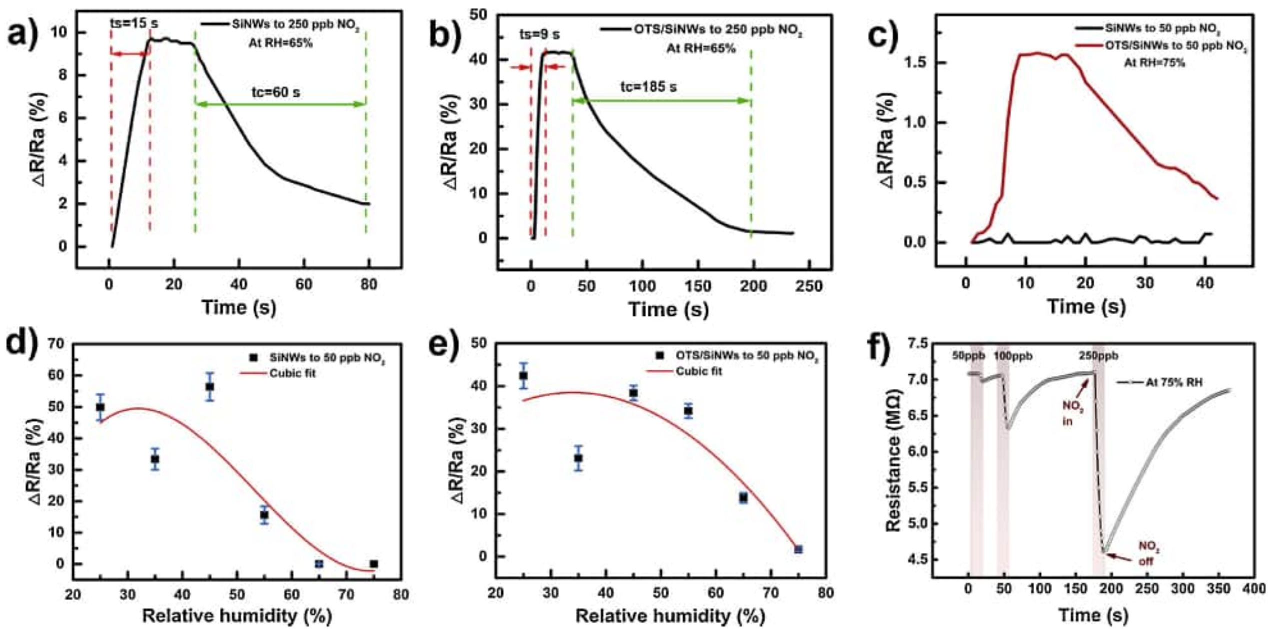

Fig. 4. (a, b) Dynamic responses to 250 ppb NO_2 at 65% RH for SiNWs sensor (a) and OTS/SiNWs sensor (b); (c) Dynamic responses of OTS/SiNWs and SiNWs sensors to 50 ppb NO_2 at 75% RH; (d, e) Response value versus relative humidity and corresponding cubic fits (red line) for SiNWs sensor (d) and OTS/SiNWs sensor (e) towards 50 ppb NO_2; (f) Dynamic response of OTS/SiNWs sensor to varying concentration of NO_2 at 75% RH (For interpretation of the references to colour in this figure legend, the reader is referred to the web version of this article).

The response value of OTS/SiNWs sensor towards 50 ppb NO_2 at 75% RH is higher than the result reported by Wu et al. at 70% RH (0.3%) [19].

To investigate the relationship between response and relative humidity, the cubic fit was calculated by all of response values of the SiNWs and OTS/SiNWs sensors to 50 ppb NO_2 at different humidity. The fitting equations are detailed in Table S1†, and the fitting results are shown in Fig. 4(d) and (e). According to the cubic fit curves of both sensors in Fig. 4(d) and (e), the response of both SiNWs and OTS/SiNWs are stable at high values when the relative humidity is less than 45%. With increasing relative humidity, the response values of the SiNWs sensor decline rapidly. Compared with the SiNWs, the response degradation of OTS/SiNWs sensor for humidity variations is more sluggish according to cubic fit curve. The slow downtrend of OTS/SiNWs sensor with humidity increasing is attributed to its near superhydrophobicity which prevents water molecules adsorbing on the surface of SiNWs. Therefore, in the case of OTS modification, the negative influence of water molecules on sensing response can be weakened to a great degree. It should be noted that, at 35% RH, the gas responses of both sensors show a sudden drop. A similar result, i.e., abnormal response decreases at 30% RH for SiNWs sensor, had ever been reported by H *J* In, et al [34]. As demonstrated, differing from the adverse effect on metal oxides, water appeared to improve the sensing response of SiNWs sensor at very low analyte concentrations. However, the SiNWs sensor showed an obviously lower response at 30% RH than at a much low relative humidity (< 10% RH). The peculiar response drop of SiNWs sensor at 30–40% RH is assumed to correlative with the intrinsic nature of silicon sensing materials and the complex interactions occurring among NO_2, adsorbed water molecules and the silicon surface. Thus for the SiNWs sensor, there might be an optimal range of ambient humidity in which the sensing response is more enhanced. A water dipole screening of the surface-NO_2 dipole was ever suggested to explain the response enhancement of epitaxial graphene under humidity condition (at 40% RH) [37]. It is not sure whether the dipole screening mechanism is adoptable for the SiNWs sensor. Further investigation based on DFT calculations on co-adsorption of NO_2 and water molecules might be helpful and required. Fig. 4(f) shows the dynamic response of the OTS/SiNWs sensor to varying concentration of NO_2 (50–250 ppb) at a much higher humidity of 75%. A sharp decline in resistance from 7.1 to 4.6 MΩ is observed when the 250 ppb NO_2 gas injected in the chamber. After the wet air with 75% RH is injected into the chamber, the sensor resistance gradually recovery to 7.04 MΩ. The nearly complete recovery of baseline resistance indicates a good reversibility of the OTS-functionalized sensor at high humidity. At other humidity of 35–65% RH, the OTS/SiNWs sensor also exhibits better reversibility than the SiNWs sensor (see Fig. S4†). The good reversibility of the OTS/SiNWs sensor exhibited at high humidity can be attributed to its superhydrophobicity induced by surface functionalization of OTS. For the bare SiNWs sensor, the reversibility deteriorates obviously with the increase of humidity, companying a difficult recovery of sensor resistance to 90% initial resistance value after release of NO_2 gas.

To visualize the resemblance and discrepancy among different samples, we performed radar plots of response variation of different NO_2 concentration for the SiNWs and OTS/SiNWs sensors at 25%–75% RH, as shown in Fig. 5. The detailed response values in the radar charts are listed in Table 1. For the SiNWs and OTS/SiNWs sensors, the lowest detection concentration of NO_2 can reach 5 ppb at 25% RH and the response values are 22.8% and 17%, respectively, due to the advantages of the top cluster structure (Fig. 5(a)).

Further from the radar plots of Fig. 5(a) and (b), we can observe that the OTS/SiNWs sensor has highly sensitive characteristic to rarefied NO_2 as low as 5 ppb at 25–35% RH and the response values are comparable to the SiNWs sensors. At 45% RH in Fig. 5(c), the OTS/SiNWs sensor exhibits significantly higher response than SiNWs sensor when exposure to NO_2 with low concentrations. Continuously increase relative humidity to 55%, the response values of the OTS/SiNWs sensor towards 5–250 ppb NO_2 show a slight decrease, as shown in Fig. 5(c–d) and Table 1. However for SiNWs sensor, the response to 5–50 ppb NO_2 is hardly detected, and upon exposure to 100 ppb NO_2, there is a response degradation more than 72% when the relative humidity increases from 45% to 55% (see Table 1). Compared with the rapidly response decline of SiNWs, the OTS/SiNWs sensor exhibits the capability for detecting rarefied NO_2 down to 10 ppb at much high relative humidity of 65%, with a response value of approximately 2.5% (Fig. 5(e) and Table 1). And at 65% RH, the response value of the OTS/SiNWs sensor towards 250 ppb NO_2 is four times higher than that of

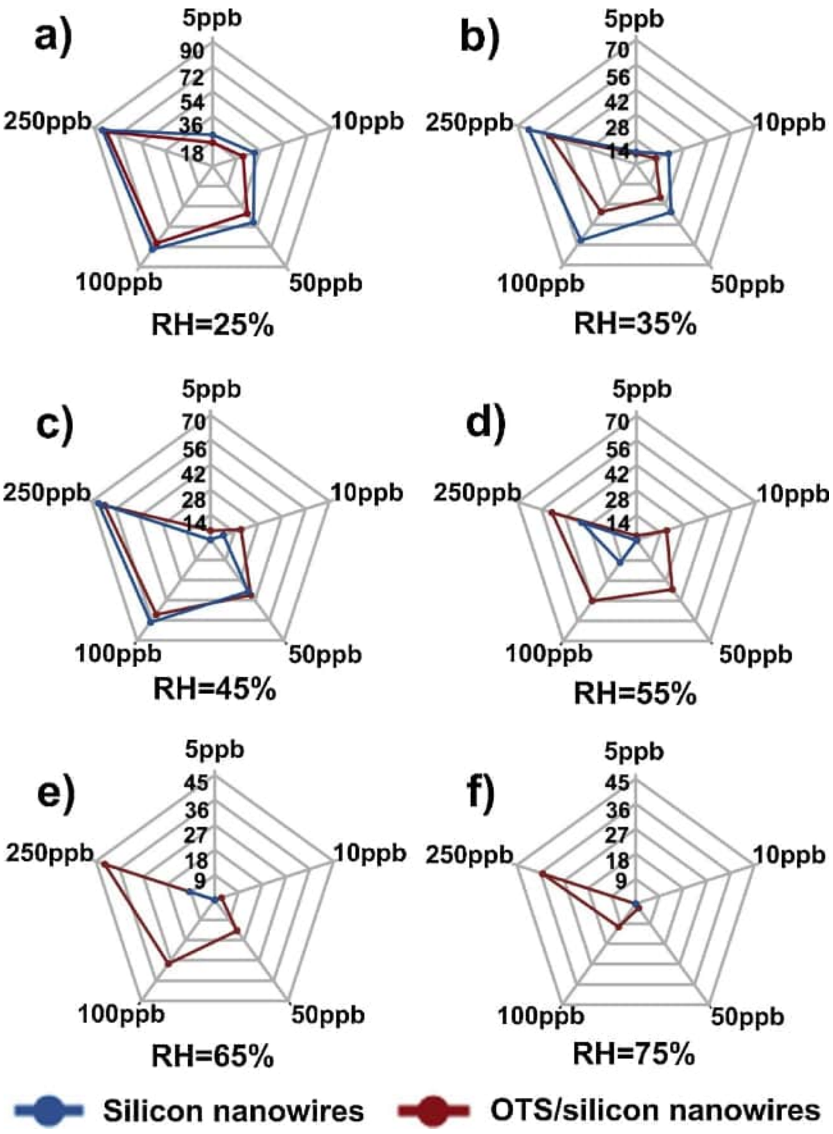

Fig. 5. (a–f) Radar plots of response of different NO_2 concentration for the SiNWs (blue) and OTS/SiNWs (red) sensors at 25%, 35%, 45%, 55%, 65%, 75%, respectively (For interpretation of the references to colour in this figure legend, the reader is referred to the web version of this article).

Table 1
Response values of the SiNWs and OTS/SiNWs sensors towards different NO_2 concentrations under 25–75% RH.

Response (%)	NO_2 conc. (ppb)									
	SiNWs					OTS/SiNWs				
RH	5	10	50	100	250	5	10	50	100	250
**25%**	22.8	31.6	50	74.3	83.8	17	23	42.4	68.9	80.5
**35%**	7	19.1	33.4	52.8	63.2	5.9	11.5	23.1	33.2	51.1
**45%**	/	7.5	35.9	57.4	66	5.11	17.8	38.4	52	62.5
**55%**	/	/	/	15.6	32.5	2.44	17.8	34.2	42.1	50
**65%**	/	/	/	/	9.7	/	2.5	13.7	28.6	41.6
**75%**	/	/	/	/	/	/	/	1.8	10.3	35.1

SiNWs sensor. When the relative humidity reaches 75%, the OTS/SiNWs sensor shows a response of 1.8 to 50 ppb NO_2. While for the SiNWs sensor, it seems to be completely invalid at 75% RH, even if the NO_2 concentration is as high as 250 ppb (Fig. 5(f)). Therefore, according to the humidity partition of Fig. 5, when the humidity is less than 45%, the silicon nanowires with the top aggregate structure play a dominant role and the humidity does not interfere significantly. With humidity increasing from 45% to 65%, the sensing response of the SiNWs sensor are deteriorating without near superhydrophobic protection, which weakens not only the response signal but also the detection capability for rarefied gas. Once the humidity achieves 75% or above, no response can be observed below 250 ppb NO_2 by the SiNWs sensor. Comparatively, the OTS/SiNWs sensor only show a response degradation towards rarefied NO_2 ($\leq$10 ppb), while the response towards 50–250 ppb NO_2 still maintains well. Above results indicate a good effect on humidity resistance for surface modification by OTS.

Table S2† shows the response comparisons of the present OTS/SiNWs sensor to some ever reported NO_2 sensors based on SiNWs and other related nanomaterials at different humidity levels. Observe that the OTS/SiNWs sensor exhibits more excellent properties in humidity resistance; it shows much higher responses at a wide range of humidity (25–75% RH) towards NO_2 with a much lower concentration in contrast to the mentioned sensors. It further demonstrates that OTS modification to SiNWs presented here is especially effective in developing NO_2-sensing sensors with humidity immunity, and for the OTS/SiNWs sensor, the detection of ppb level rarefied NO_2 under high humidity (such as 75% RH) is capable at room temperature.

Based on the detailed response values shown in Table S4†, we made linear fitting for the response value versus NO_2 concentration and

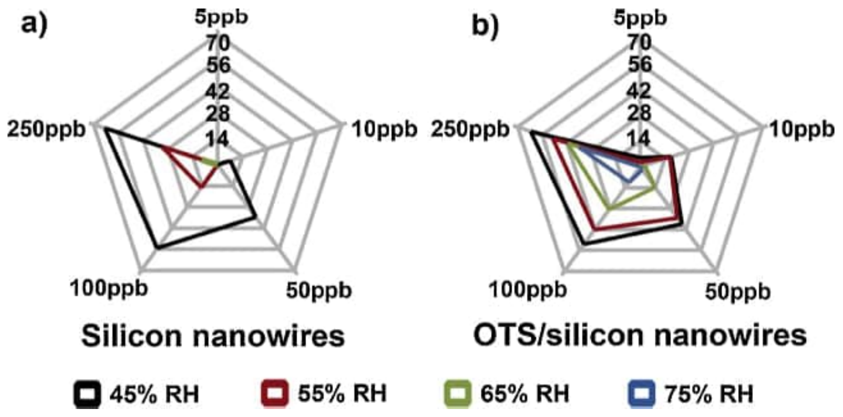

Fig. 6. Radar charts of response variation of different NO_2 concentration for the SiNWs (a) and OTS/SiNWs (b) sensors at 45%–75% RH.

further calculate the limit of detection (LOD) of the OTS/SiNWs sensor under different relative humidity, according to $LOD = 3SD_{noise}/S$, where SD_{noise} is the standard deviation of sensor noise before NO_2 exposure and S is the sensor sensitivity, i.e., the slope of the linear part of the calibration curve [56,57]. The calculated S and LOD of OTS/SiNWs sensor are shown in Table S3†. Observe that an ultralow LOD of 0.52 ppb NO_2 at 25% RH is deduced. At much high humidity of 75%, the OTS-functionalized sensor still gives an extremely low LOD of 0.82 ppb. Such low LODs are attributed to both the high sensitivity and the low noise level (~ 0.04) of the as-developed OTS/SiNWs sensor. The deduced LODs at different humidity for OTS/SiNWs sensor are much lower than that of many other NO_2 sensors ever reported, such as 3D reduced graphene oxide and TTC-SiNWs (see Table S2†).

To achieve a better understanding of the enhancement of humidity-resisting by modification of OTS, the response variations for SiNWs and OTS/SiNWs sensors at high humidity are demonstrated in the radar chart in Fig. 6. The response values of SiNWs decreases geometrically (6.8 times drop towards 250 ppb NO_2) and the detectable range of NO_2 is rapidly reduced as the humidity rises from 45% (black line) to 65% (green line). Comparatively, the OTS/SiNWs has shown relatively stable decline to the same concentration of NO_2 under different humidity. Therefore, every curve presented detectable concentration ranges has a larger area as shown in Fig. 6(b), indicating a broader sensitive range of humidity for OTS/SiNWs. It is crucial in practice capable of gas detection in a wide range of ambient humidity. For the SiNWs sensor, not only the sensitive range reduces but the response time elongates with increasing humidity, as shown in Table S4† which lists the response times and recovery times of SiNWs and OTS/SiNWs gas sensors towards different NO_2 concentration under 25–75% RH. Prolonging of the response time is due to water molecules distributed on the silicon nanowires make the diffusion of NO_2 molecules slow [49,58]. This shortcoming is well compensated in the OTS/SiNWs sensor, whose response time is kept within 10 s towards 5–250 ppb NO_2 gas exposure (see Table S4†). The recovery time of the SiNWs sensor gradually shorten with the humidity increasing, because the sensor resistance cannot be completely recovered to its baseline value and thus the t_c shown in the table is actually less than the defined 90% recovery time. Comparatively, the OTS/SiNWs sensor exhibits good reversibility at high humidity of 75% as revealed by Fig.4 (f). The repeatability of the functionalized sensor under high humidity conditions was further checked by evaluating the dynamic characteristic of the OTS/SiNWs sensor in five continuous cycles of response-recovery to a fixed concentration of NO_2 in 100 ppb at 75% RH. As shown in Fig. S5†, almost identically dynamic characteristics for every gas in – out cycle demonstrate satisfactory repeatability of the sensor. Continuous measurements in a much longer duration of 22 days further verify the good stability of the OTS/SiNWs sensor. Fig. S6† is the results from the OTS/SiNWs sensor towards 250 ppb NO_2 exposure at 75% RH. The acceptable attenuation of about 18% in response value after 22 days of measurement demonstrate a satisfactory stability of the OTS-functionalized sensor. Moreover, the stable responses of OTS/SiNWs sensor at high ambient humidity of 75% further indicates the effectiveness of OTS in enhancing humidity resistance of SiNWs during gas detection at room temperature.

The selectivity of OTS/SiNWs sensor was further investigated. Sensing response of the SiNWs and OTS/SiNWs sensors to other seven gases (100 ppm of NO, ethanol, acetone, methanol, methane, O_2 and 1 ppm of NH_3) were measured at 25% RH and 65% RH, respectively, and compared with those to 0.1 ppm NO_2. Fig. S7† shows the results from the OTS/SiNWs sensor. The bare SiNWs sensor exhibits a similar response feature as that of the OTS-functionalized sensor at 25% RH. Considering the big concentration difference of tested gases, the OTS/SiNWs sensor exhibits acceptable selectivity to NO_2, as evidence of the biggest response value to 0.1 ppm NO_2 than to other gases with much higher concentrations. Ambient humidity shows little effect on selectivity characteristic of the OTS-functionalized sensor, despite the fact that the response of the sensor to each type of gas is significantly reduced at much high relative humidity of 65%.

4. Conclusions

We herein present a kind of OTS-functionalized porous SiNWs (OTS/SiNWs) gas sensor with good immunity to humidity, low consumption and good reversibility. In this novel OTS/SiNWs with umbrella-like arrangement of OTS, the insensitive SiO_2 layer of porous SiNWs was efficiently used to bond OTS achieving near superhydrophobicity of SiNWs. The as-fabricated OTS/SiNWs sensor displays short response times (within 10 s over the whole NO_2 concentration) and reversible sensing behavior under high humidity (75% RH). In virtue of the synergy of porous silicon nanowires and OTS, detections of NO_2 with the low concentration of 5 ppb below 55% RH and 50 ppb at 75% RH are achieved respectively. We believe that the remarkable sensor performance, obtained with a simple fabrication process, significantly enlarges the potential of silicon-based sensor in practical application, such as the detection of human breathing and outdoor trace toxic gas detection.

Acknowledgement

This work was financially supported by the National Natural Science Foundation of China (Nos. 61574100, 61274074).

Appendix A. Supplementary data

Supplementary material related to this article can be found, in the online version, at doi:https://doi.org/10.1016/j.snb.2018.12.013.

References

[1] D. Liu, L. Lin, Q. Chen, H. Zhou, J. Wu, Low power consumption gas sensor created from silicon nanowires/TiO2 core-shell heterojunctions, ACS Sens. 2 (2017) 1491–1497.
[2] Z. Wang, C. Hou, Q. De, F. Gu, D. Han, One-step synthesis of Co-doped In2O3 nanorods for high response of formaldehyde sensor at low temperature, ACS Sens. 3 (2018) 468–475.
[3] M.-Y. Chuang, C.-C. Chen, H.-W. Zan, H.-F. Meng, C.-J. Lu, Organic gas sensor with an improved lifetime for detecting breath ammonia in hemodialysis patients, ACS Sens. 2 (2017) 1788–1795.
[4] R. Kumar, N. Goel, M. Kumar, UV-activated MoS2 based fast and reversible NO2 sensor at room temperature, ACS Sens. 2 (2017) 1744–1752.
[5] J. Ramirez, D. Rodriquez, F. Qiao, J. Warchall, J. Rye, E. Aklile, et al., Metallic nanoislands on graphene for monitoring swallowing activity in head and neck cancer patients, ACS Nano 12 (2018) 5913–5922.
[6] J. Chen, Z. Chen, F. Boussaid, D. Zhang, X. Pan, H. Zhao, et al., Ultra-low-Power smart electronic nose system based on three-dimensional tin oxide nanotube arrays, ACS Nano 12 (2018) 6079–6088.
[7] Z. Ma, P. Chen, W. Cheng, K. Yan, L. Pan, Y. Shi, et al., Highly sensitive, printable nanostructured conductive polymer wireless sensor for food spoilage detection, Nano Lett. 18 (2018) 4570–4575.
[8] M. Cho, J. Yun, D. Kwon, K. Kim, I. Park, High-sensitivity and low-power flexible schottky hydrogen sensor based on silicon nanomembrane, ACS Appl. Mater. Interfaces 10 (2018) 12870–12877.
[9] Y.J. Kwon, S.-W. Choi, S.Y. Kang, M.S. Choi, J.H. Bang, S.S. Kim, et al., Enhancement of the benzene-sensing performance of Si nanowires through the incorporation of TeO2 heterointerfaces and Pd-sensitization, Sens Actuator B-Chem 244 (2017) 1085–1097.
[10] J. Baek, B. Jang, M.H. Kim, W. Kim, J. Kim, H.J. Rim, et al., High-performance hydrogen sensing properties and sensing mechanism in Pd-coated p-type Si nanowire arrays, Sens. Actuator B-Chem. 256 (2018) 465–471.
[11] Y. Pak, N. Lim, Y. Kumaresan, R. Lee, K. Kim, T.H. Kim, et al., Palladium nanoribbon array for fast hydrogen gas sensing with ultrahigh sensitivity, Adv. Mater. 27 (2015) 6945–6952.
[12] A. Lay-Ekuakille, S. Ikezawa, M. Mugnaini, R. Morello, C. De Capua, Detection of specific macro and micropollutants in air monitoring: review of methods and techniques, Measurement 98 (2017) 49–59.
[13] D. Huang, Z. Yang, X. Li, L. Zhang, J. Hu, Y. Su, et al., Three-dimensional conductive networks based on stacked SiO2@graphene frameworks for enhanced gas sensing, Nanoscale 9 (2017) 109–118.
[14] A.T. Güntner, V. Koren, K. Chikkadi, M. Righettoni, S.E. Pratsinis, E-nose sensing of low-ppb formaldehyde in gas mixtures at high relative humidity for breath screening of lung cancer, ACS Sens. 1 (2016) 528–535.
[15] N. Shehada, G. Broenstrup, K. Funka, S. Christiansen, M. Leja, H. Haick, Ultrasensitive silicon nanowire for real-world gas sensing: noninvasive diagnosis of cancer from breath volatolome, Nano Lett. 15 (2015) 1288–1295.
[16] H. Wang, Y. Liu, Z. Wang, M. Yang, Y. Gu, 808 nm-light-excited upconversion nanoprobe based on LRET for the ratiometric detection of nitric oxide in living cancer cells, Nanoscale 10 (2018) 10641–10649.
[17] G. Katwal, M. Paulose, I.A. Rusakova, J.E. Martinez, O.K. Varghese, Rapid growth of zinc oxide nanotube-nanowire hybrid architectures and their use in breast cancer-related volatile organics detection, Nano Lett. 16 (2016) 3014–3021.
[18] G. Ge, Y. Cai, Q. Dong, Y. Zhang, J. Shao, W. Huang, et al., A flexible pressure sensor based on rGO/polyaniline wrapped sponge with tunable sensitivity for human motion detection, Nanoscale 10 (2018) 10033–10040.
[19] J. Wu, Z. Li, X. Xie, K. Tao, C. Liu, K.A. Khor, et al., 3D superhydrophobic reduced graphene oxide for activated NO2 sensing with enhanced immunity to humidity, J. Mater. Chem. A 6 (2018) 478–488.
[20] M. Donarelli, S. Prezioso, F. Perrozzi, L. Giancaterini, C. Cantalini, E. Treossi, et al., Graphene oxide for gas detection under standard humidity conditions, 2d Mater. 2 (2015) 035018.
[21] S.-J. Choi, S. Chattopadhyay, J.J. Kim, S.-J. Kim, H.L. Tuller, G.C. Rutledge, et al., Coaxial electrospinning of WO3 nanotubes functionalized with bio-inspired Pd catalysts and their superior hydrogen sensing performance, Nanoscale 8 (2016) 9159–9166.
[22] F.E. Annanouch, Z. Haddi, S. Vallejos, P. Umek, P. Guttmann, C. Bittencourt, et al., Aerosol-assisted CVD-Grown WO3 nanoneedles decorated with copper oxide nanoparticles for the selective and humidity-resilient detection of H2S, ACS Appl. Mater. Interfaces 7 (2015) 6842–6851.
[23] J. Hao, D. Zhang, Q. Sun, S. Zheng, J. Sun, Y. Wang, Hierarchical SnS2/SnO2 nanoheterojunctions with increased active-sites and charge transfer for ultrasensitive NO2 detection, Nanoscale 10 (2018) 7210–7217.
[24] S. Roso, C. Bittencourt, P. Umek, O. Gonzalez, F. Guell, A. Urakawa, et al., Synthesis of single crystalline In2O3 octahedra for the selective detection of NO2 and H-2 at trace levels, J. Mater. Chem. C Mater. Opt. Electron. Devices 4 (2016) 9418–9427.
[25] H. Long, L. Chan, A. Harley-Trochimczyk, L.E. Luna, Z. Tang, T. Shi, et al., 3D MoS2 aerogel for ultrasensitive NO2 detection and its tunable sensing behavior, Adv. Mater. Interfaces 4 (2017) 1700217.
[26] Y. Qin, Z. Wang, D. Liu, K. Wang, Dendritic composite array of silicon nanowires/WO3 nanowires for sensitive detection of NO2 at room temperature, Mater. Lett. 207 (2017) 29–32.
[27] F. Ricciardella, S. Vollebregt, T. Polichetti, M. Miscuglio, B. Alfano, M.L. Miglietta, et al., Effects of graphene defects on gas sensing properties towards NO2 detection, Nanoscale 9 (2017) 6085–6093.
[28] Y.X. Li, N. Chen, D.Y. Deng, X.X. Xing, X.C. Xiao, Y.D. Wang, Formaldehyde detection: SnO2 microspheres for formaldehyde gas sensor with high sensitivity, fast response/recovery and good selectivity, Sens. Actuator B-Chem. 238 (2017) 264–273.
[29] X. Liu, N. Chen, B. Han, X. Xiao, G. Chen, I. Djerdj, et al., Nanoparticle cluster gas sensor: Pt activated SnO2 nanoparticles for NH3 detection with ultrahigh sensitivity, Nanoscale 7 (2015) 14872–14880.
[30] H. Yu, P. Xu, D.W. Lee, X. Li, Porous-layered stack of functionalized AuNP-rGO (gold nanoparticles-reduced graphene oxide) nanosheets as a sensing material for the micro-gravimetric detection of chemical vapor, J. Mater. Chem. A 1 (2013) 4444–4450.
[31] A. Cao, E.J. Sudholter, L.C. de Smet, Silicon nanowire-based devices for gas-phase sensing, Sensors (Basel) 14 (2013) 245–271.
[32] S.-Y. Cho, H.-W. Yoo, J.Y. Kim, W.-B. Jung, M.L. Jin, J.-S. Kim, et al., High-resolution p-type metal oxide semiconductor nanowire array as an ultrasensitive sensor for volatile organic compounds, Nano Lett. 16 (2016) 4508–4515.
[33] L.M. Lin, D. Liu, Q.F. Chen, H.Z. Zhou, J.M. Wu, A vertical tip-tip contact silicon nanowire array for gas sensing, Nanoscale 8 (2016) 17757–17764.
[34] H.J. In, C.R. Field, P.E. Pehrsson, Periodically porous top electrodes on vertical nanowire arrays for highly sensitive gas detection, Nanotechnology 22 (2011) 355501.
[35] H.M. Fahad, H. Shiraki, M. Amani, C.C. Zhang, V.S. Hebbar, W. Gao, et al., Room temperature multiplexed gas sensing using chemical-sensitive 3.5-nm-thin silicon transistors, Sci. Adv. 3 (2017) e1602557.
[36] M. Gao, M. Cho, H.J. Han, Y.S. Jung, I. Park, Palladium-decorated silicon nanomesh fabricated by nanosphere lithography for high performance, room temperature hydrogen sensing, Small 14 (2018) 1703691.
[37] M. Ridene, I. Iezhokin, P. Offermans, C.F.J. Flipse, Enhanced sensitivity of epitaxial graphene to NO2 by water coadsorption, J. Phys. Chem. C 120 (2016) 19107–19112.
[38] B.R. Tao, J. Zhang, F.J. Miao, H.L. Li, L.J. Wan, Y.T. Wang, Capacitive humidity sensors based on Ni/SiNWs nanocomposites, Sens. Actuator B-Chem. 136 (2009) 144–150.
[39] L.L. Wang, L.P. Kang, H.Y. Wang, Z.P. Chen, X.J. Li, Capacitive humidity sensitivity of SnO2:Sn thin film grown on silicon nanoporous pillar array, Sens. Actuator B-Chem. 229 (2016) 513–519.
[40] Y.L. Wang, M. Lieberman, Growth of ultrasmooth octadecyltrichlorosilane self-assembled monolayers on SiO2, Langmuir 19 (2003) 1159–1167.
[41] Y. Ito, A.A. Virkar, S. Mannsfeld, J.H. Oh, M. Toney, J. Locklin, et al., Crystalline ultrasmooth self-assembled monolayers of alkylsilanes for organic field-effect transistors, J. Am. Chem. Soc. 131 (2009) 9396–9404.
[42] Y. Song, R.P. Nair, M. Zou, Y. Wang, Superhydrophobic surfaces produced by applying a self-assembled monolayer to silicon micro/nano-textured surfaces, Nano Res. 2 (2010) 143–150.
[43] H.L. Yu, J.Q. Zhu, L. Yang, B. Dai, L. Baraban, G. Cuniberti, et al., Superhydrophobic carbon nanotube/silicon carbide nanowire nanocomposites, Mater. Des. 87 (2015) 198–204.
[44] P. Seeharaj, P. Pasupong, E. Detsri, P. Damrongsak, Superhydrophobilization of SiO2 surface with two alkylsilanes for an application in oil/water separation, J. Mater. Sci. 53 (2018) 4828–4839.
[45] M.R. Yousaf, B.S. Yilbas, H. Ali, Assessment of optical transmittance of oil impregnated and non-wetted surfaces in outdoor environment towards solar energy harvesting, Sol. Energy 163 (2018) 25–31.
[46] Y.X. Qin, Y.Q. Jiang, L.M. Zhao, Modulation of agglomeration of vertical porous silicon nanowires and the effect on gas-sensing response, Adv. Eng. Mater. 20 (2018) 1700893.
[47] Y.X. Qin, Y. Liu, Y.Y. Wang, Aligned array of porous silicon nanowires for gas-sensing application, Ecs J. Solid State Sci. Technol. 5 (2016) 380–383.
[48] Y.Q. Wu, M. Hu, Y.X. Qin, X.Y. Wei, S.Y. Ma, D.L. Yan, Enhanced response characteristics of p-porous silicon (substrate)/p-TeO2 (nanowires) sensor for NO2 detection, Sens Actuator B-Chem 195 (2014) 181–188.
[49] W.J. Yan, M.A. Worsley, T. Pham, A. Zettl, C. Carraro, R. Maboudian, Effects of ambient humidity and temperature on the NO2 sensing characteristics of WS2/graphene aerogel, Appl. Surf. Sci. 450 (2018) 372–379.
[50] J. Seo, S. Lee, J. Lee, T. Lee, Guided transport of water droplets on super-hydrophobic-hydrophilic patterned Si nanowires, ACS Appl. Mater. Interfaces 3 (2011) 4722–4729.
[51] S. Hoshian, V. Jokinen, V. Somerkivi, A.R. Lokanathan, S. Franssila, Robust superhydrophobic silicon without a low surface-energy hydrophobic coating, ACS Appl. Mater. Interfaces 7 (2015) 941–949.
[52] S.A. Mirji, Octadecyltrichlorosilane adsorption kinetics on Si(100)/SiO2 surface: contact angle, AFM, FTIR and XPS analysis, Surf. Interface Anal. 38 (2006) 158–165.
[53] J. Xie, X. Jiang, Y. Zhong, Y. Lu, S. Wang, X. Wei, et al., Stem-loop DNA-assisted silicon nanowires-based biochemical sensors with ultra-high sensitivity, specificity, and multiplexing capability, Nanoscale 6 (2014) 9215–9222.
[54] F. Guenes, H. Arezki, D. Pierucci, D. Alamarguy, J. Alvarez, J.-P. Kleider, et al., Tuning the work function of monolayer graphene on 4H-SiC (0001) with nitric acid, Nanotechnology 26 (2015).
[55] Y. Qin, D. Liu, T. Zhang, Z. Cui, Ultrasensitive silicon nanowire sensor developed by a special Ag modification process for rapid NH3 detection, ACS Appl. Mater. Interfaces 9 (2017) 28766–28773.
[56] L. Li, S. He, M. Liu, C. Zhang, W. Chen, Three-dimensional mesoporous graphene aerogel-supported SnO2 nanocrystals for high-performance NO2 gas sensing at low temperature, Anal. Chem. 87 (2015) 1638–1645.
[57] D. Le Thai, D.-J. Kim, T. Tran Quang, D. Vinh Quang, B.-Y. Kim, H.K. Moon, et al., High performance three-dimensional chemical sensor platform using reduced graphene oxide formed on high aspect-ratio micro-pillars, Adv. Funct. Mater. 25 (2015) 883–890.
[58] V.A. Poddubny, N.A. Yushketova, A physicochemical model of sorption processes in NO2 passive sampling with air humidity effects, Environ. Monit. Assess. 185 (2013) 3819–3829.

nanomaterials

Article

Humidity Sensing Ceria Thin-Films

Vilko Mandić [1,*], Arijeta Bafti [1,*], Luka Pavić [2], Ivana Panžić [1], Stanislav Kurajica [1], Jakov-Stjepan Pavelić [1], Zhen Shi [3], Katarina Mužina [1] and Ivana Katarina Ivković [1]

1 Faculty of Chemical Engineering and Technology, Marulićev trg 20, 10000 Zagreb, Croatia; ipanzic@fkit.hr (I.P.); stankok@fkit.hr (S.K.); jpavelic@fkit.hr (J.-S.P.); kmuzina@fkit.hr (K.M.); imunda@fkit.hr (I.K.I.)
2 Ruđer Bošković Institute, Bijenička Cesta 54, 10000 Zagreb, Croatia; luka.pavic@irb.hr
3 College of Materials and Environmental Engineering, Hangzhou Dianzi University, 1158, No.2 Street, Hangzhou 310018, China; zhenshi@hdu.edu.cn
* Correspondence: vmandic@fkit.hr (V.M.); abafti@fkit.hr (A.B.); Tel.: +385-1-4597-226 (V.M.); +385-1-4597-218 (A.B.)

Abstract: Lowering the constitutive domains of semiconducting oxides to the nano-range has recently opened up the possibility of added benefit in the research area of sensing materials, in terms both of greater specific surface area and pore volume. Among such nanomaterials, ceria has attracted much attention; therefore, we chemically derived homogeneous ceria nanoparticle slurries. One set of samples was tape-casted onto a conducting glass substrate to form thin-films of various thicknesses, thereby avoiding demanding reaction conditions typical of physical depositions, while the other was pressed into pellets. Structural and microstructural features, along with electrical properties and derivative humidity-sensing performance of ceria thin-films and powders pressed into pellets, were studied in detail. Particular attention was given to solid-state impedance spectroscopy (SS-IS), under controlled relative humidity (RH) from 30%–85%, in a wide temperature and frequency range. Moreover, for the thin-film setup, measurements were performed in surface-mode and cross-section-mode. From the results, we extrapolated the influence of composition on relative humidity, the role of configuration and thin-film thickness on electrical properties, and derivative humidity-sensing performance. The structural analysis and depth profiling both point to monophasic crystalline ceria. Microstructure analysis reveals slightly agglomerated spherical particles and thin-films with low surface roughness. Under controlled humidity, the shape of the conductivity spectrum stays the same along with an increase in RH, and a notable shift to higher conductivity values. The relaxation is slow, as the thickness of the pellet slows the return of conductivity values. The increase in humidity has a positive effect on the overall DC conductivity, similar to the temperature effect for semiconducting behavior. As for the surface measurement setup, the thin-film thickness impacts the shape of the spectra and electrical processes. The surface measurement setup turns out to be more sensitive to relative humidity changes, emphasized with higher RH, along with an increase in thin-film thickness. The moisture directly affects the conductivity spectra in the dispersion part, i.e., on the localized short-range charge carriers. Moisture sensitivity is a reversible process for thin-film samples, in contrast to pellet form samples.

Keywords: thin-films; relative humidity sensors; ceria nanoparticles; solid-state impedance spectroscopy; grazing incidence X-ray diffraction (GIXRD); atomic force microscopy (AFM); field emission scanning electron microscopy (FESEM)

check for updates

Citation: Mandić, V.; Bafti, A.; Pavić, L.; Panžić, I.; Kurajica, S.; Pavelić, J.-S.; Shi, Z.; Mužina, K.; Ivković, I.K. Humidity Sensing Ceria Thin-Films. *Nanomaterials* **2022**, *12*, 521. https://doi.org/10.3390/nano12030521

Academic Editors: Marcin Runowski and Antonino Gulino

Received: 7 December 2021
Accepted: 28 January 2022
Published: 2 February 2022

Publisher's Note: MDPI stays neutral with regard to jurisdictional claims in published maps and institutional affiliations.

Copyright: © 2022 by the authors. Licensee MDPI, Basel, Switzerland. This article is an open access article distributed under the terms and conditions of the Creative Commons Attribution (CC BY) license (https://creativecommons.org/licenses/by/4.0/).

1. Introduction

In various fields such as industry, agriculture, environmental protection, health monitoring, and household use, the application of humidity sensors is necessary [1]. The inherent characteristics of humidity sensors should include high sensitivity and selectivity, as well as a wide detection range, along with easy and affordable production. [2]. Various

materials can be used for the constitutive parts of humidity sensors, but the advantages of metal oxides lie in their stability (mechanical, chemical, and physical), thermally broad operational range, and fast response and recovery [2]. For example, CeO_2 is a metal oxide used as a humidity sensor, but also as a catalyst, a solid electrolyte in fuel cells, a high-temperature ceramic, coating, etc. [3–6]. For sensing application, the composition of materials is, of course, critical, but morphologically the nanometer dimension of a particle constituent also plays a beneficial role in the advancement of sensing properties, by providing a large specific surface area and pore volume [7]. Therefore, the reports on better sensing performance of nanostructured ceria compared to the bulk material come as no surprise [8,9]. For the preparation of ceria thin-films, a variety of physical deposition methods has been reported [9,10]. Several reports of relatively demanding methods, such as pulsed laser deposition or magnetron sputtering, stand out [10]. Among demanding chemical depositions, it was shown that chemical vapor deposition can be used [11]. Provided the course of the deposition is well optimized, equally successful thin-films can be derived by the much simpler and affordable tape casting deposition of previously precipitated nanoparticles [12].

The reason behind the favorable properties of ceria for sensing lies in their electronic structure: Ce^{3+} ions and oxygen vacancies. Specifically, ceria displays a high charge density, due to the high positive charge and small ionic radius of Ce^{4+} ions. For nanostructured ceria particles, such a strong electric field can also increase the ionization of water molecules on the surface and affect the deeper physiosorbed water [8]. As a consequence, the nano-ceria display great oxygen storage and release capacity via simple Ce^{4+}/Ce^{3+} redox cycles [13–15]. The resistance in ceria films decreases exponentially along with increasing humidity; consequently, an ion-conductivity mechanism dominates the sensing [7,16,17]. As previously stated, nano-structuring plays a considerable role in the performance of a sensor; from the point of view of ceria sensors, Fu et al. investigated hydrothermally prepared ceria nanowires due to their fast sensing response [8] and in terms of their implementation in fast humidity sensors. It was found that the variation in the number of water molecules adsorbed on the surface when changing relative humidity also controlled electrical behavior [18]. On the other hand, the transport of the proton ions on the sample surface is unrestricted due to Grotthuss's chain reaction [19,20]. Reports on doped ceria nanoparticles, regarding humidified atmosphere monitoring adsorption behavior and electrical conductivity, are also available [20]. Under a humidified atmosphere, proton conductivity of dense monocrystalline ceria was in the range 10^{-7} and 10^{-8} S cm^{-1} at ~50 °C. The proton conductivity decreased along with the increase in temperature. Excellent recovery and repeatability for the ceria sensor with the linear response are reported for a wide span of relative humidities, from 11–97%, for sensors prepared by the microwave-assisted method [21]. Moreover, the structural heterogeneity and large surface-to-volume ratio of sintered ceria nanoparticles lead to high values of the dielectric constant in the low-frequency range for the sintered ceria nanoparticles, due to large surface to volume ratio [22]. AC conductivity is constant up to 175 °C, after which it starts to be temperature-sensitive, followed by an increase as the temperature continues to rise. Data on the impedance spectroscopy approach is extremely scarce in the literature for ceria thin-film humidity sensors, especially bearing in mind the different characterization setups and geometries. Moreover, despite the different geometries, the available literature mainly focuses on the resistance response to an increase in humidity, while the frequency of the measurement is fixed [23]. In line with this, there is a need to study this sensing material in a wide range of frequencies and temperatures.

In this work, we present the development of ceria humidity sensor materials in thin-film and bulk configurations. Through a wide range of structural, microstructural, and electrical characterizations, with a particular focus on Solid-State Impedance Spectroscopy (SS-IS), we monitor the influence of the various synthetic and deposition parameters on the humidity sensing properties, as well as other key properties of the derived thin-films. Interestingly we have been able to dedicate measuring setups to differentiate bulk, surface

and cross-section properties, in a wide frequency range from 0.1 to 10^6 Hz. Therefore, the discussion particularly focuses on the functional aspects of different sample and measurement geometry configurations, as well as the influence of film thickness on subsequent electrical properties.

2. Materials and Methods

2.1. Preparation

Ceria powder synthesis has been reported elsewhere [24]. Briefly, 200 mL of 0.2 mol solution of cerium(III) nitrate hexahydrate (p.a., Merck, Darmstadt, Germany) in deionized water were heated to 70 °C and stirred at a rate of 500 rpm. Then 100 mL of 3M ammonia solution (p.a., Alkaloid, Skopje, Northern Macedonia) were added, causing immediate precipitate formation. The stirring speed was increased to 700 rpm and the reaction was carried out for 5 min. The pH value of the reaction mixture was kept at ~9.3 by adding a few drops of concentrated ammonia solution. The formed precipitate was aged for 20 h, after which it was centrifuged, washed with water and ethanol with the help of sonication, and subsequently dried at 60 °C. The obtained product was a fine yellow powder. For the preparation of the thin-film samples, a solution of polyvinyl diethyl fluoride (PVDF, Sigma Aldrich, St. Louis, MO, USA) dissolved in N-methyl pyrrolidine (NMP, 99%, Thermo Fisher Scientific, Waltham, MA, USA) was used as a binder. The powder was mixed with the solution to form a thick suspension. This suspension was consolidated on glass slides by a doctor blade with a clearance of 10, 20, 40, and 80 µm. Two types of glass slides were used, regular microscope slides and fluorine tin oxide glass (FTO, Ossila, Sheffield, UK). Glass slides were washed in acetone and ethanol with the help of sonication before being used as a thin-film substrate. The formed ceria films were dried under a vacuum at 100 °C overnight. A scheme of the synthesis, processing and characterization setups are included in Figure 1a.

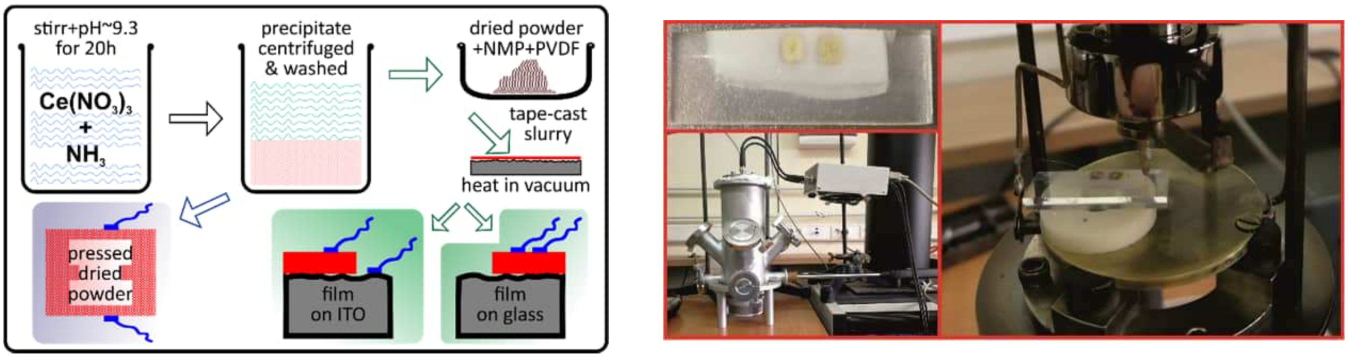

(**a**) (**b**)

Figure 1. (**a**) Scheme of the synthesis, processing and characterization setups; (**b**) CeO_2 sample in the form of thin-film on FTO glass substrate with gold electrodes (surface measurements setup, left up), sample connected to the instrument cell (left down), and setup for relative humidity measurements (right).

2.2. Characterization

Powder X-ray diffraction (PXRD) analysis was performed using an XRD 6000 diffractometer (Shimadzu, Kyoto, Japan) with CuKα radiation. The obtained data were collected in the range of 5–100°2θ in the step mode (step of 0.02°2θ), and the retention time per step was 0.6 seconds. The average crystallite size was calculated from the (200) peak using the Scherrer equation: $d = k\lambda/(\beta\cos\theta)$, in which d represents the average size of the crystallite, k is the Scherrer constant which in a given case of spherical crystallites of cubic symmetry is 0.94, λ is the wavelength of CuKα X-ray radiation and is 0.15418 nm, β is the width at half the height of the diffraction peak corrected for instrumental broadening, and θ is the Bragg angle. The X-ray experiment using dedicated synchrotron X-ray apparatus was

performed at the Materials Characterization by X-ray diffraction (MCX) beamline of the Elettra synchrotron radiation facility (Trieste, Italy) using 2.4 GeV synchrotron radiation delivered using bending magnets. The incident beam energy was 8 keV, corresponding to CuKα_1 radiation. The apparatus rests on a four-axis Huber goniometer equipped with a fast scintillator detector in Bragg-Brentano grazing incidence setup (GIXRD) [25]. The thin-film sample was glued on a flat sample holder and adjusted in position using theta-scan (tilting) and z-scan (height). For these measurements, a CeO_2 thin-film was used at different incidence angles.

Scanning electron microscopy (SEM) was performed using a high-resolution scanning electron microscope JSM-7000F (JEOL, Tokyo, Japan) at a voltage of 10 kV.

N_2 adsorption-desorption isotherms were recorded on Micromeritics ASAP-2000 (Micromeritics Instruments Corp., Norcross, GA, USA) at 77 K. The sample was previously degassed at 100 °C in a dynamic vacuum of 7 mPa. Desorption data were used to calculate the specific surface area of the powder using the Brunauer-Emmett-Teller (BET) method, while the Barret-Joyner-Hallenda (BJH) method was used to calculate the pore size distribution.

The UV-Vis spectra of the powder were collected utilizing QEPRO (Ocean Insight, Duiven, The Netherlands) spectrometer with DH-2000-DUV and ISP-50-8-R-GT integrating sphere. The diffuse reflectance spectrum was recorded in the wavelength range 300–900 nm and BaSO4 was used as a reference. Band-gap energy was calculated via Tauc equation, $(\alpha h\nu)^n = B(h\nu\text{–Eg})$, where α is extinction coefficient, h is the Planck's constant and ν is frequency; the exponent n is associated with the electronic transition in the course of optical absorption process and is theoretically equal to $\frac{1}{2}$ and 2 for indirect and direct allowed transitions, respectively; B is a constant and Eg is band-gap energy [26]. The diffuse reflectance spectra were transformed via Kubelka-Munk transformation of the measured spectrum to Kubelka-Munk function, $\text{F}(R) = (1–R)^2/(2R)$, where F($R$) is proportional to the extinction coefficient (α) and R is the reflectance of the "infinitely thick" layer of the solid [2]. The bandgap energy, Eg, was assessed by plotting $(\text{F}(R)h\nu)^n$ vs. photon energy ($h\nu$), the so-called Tauc's plot, followed by extrapolation of the linear region onto the energy axis.

Atomic force microscopy (AFM) characterization was conducted using CoreAFM (Nanosurf, Liestal, Switzerland) under ambient conditions. For the images acquisition, non-contact (tapping) mode was used. The Tap300Al-G probe with nominal resonant frequency of 300 kHz and tip radius of less than 10 nm turned out to be the best for this characterization. The scanning parameters were initialized using a setpoint of 35 or 55 nN of contact force with an acquisition time of 0.78 s on a 10 × 10 µm surface. Images were processed with the Gwyddion program [27].

The electrical properties of the prepared CeO_2 samples, in the form of thin-films and pellet samples, were studied by solid-state impedance spectroscopy. An impedance analyzer Alpha-A Dielectric Spectrometer, (Novocontrol Technologies, Montabaur, Germany) was used to measure impedance at controlled relative humidity (RH) from 30% to 85%, and for selected samples measurements were also made in a wide temperature and frequency range. A closed in-situ chamber was used to measure impedance in the humidified atmosphere, where humidity conditions were fixed inside, on behalf of the use of specific salts. The humidity is expressed as relative humidity with respect to the capacity of the air to embed the water vapor. For these measurements, both bulk CeO_2 material and CeO_2 thin-films with thickness variation were used. One set of measurements was performed on a polycrystalline sample pressed into a pellet of the approximate thickness of 1 mm and 5 mm in diameter. For the electrical contacts, gold electrodes, 3.8 mm in diameter, were sputtered onto both sides of the pellets using a Sputter Coater SC7620 (Quorum Technologies, Laughton, UK). The impedance temperature-dependent measurements were made in the nitrogen atmosphere over a frequency range from 0.1 Hz to 0.1 MHz and in the temperature range from 30 to 210 °C with a step of 30 °C. A frequency sweep at each temperature was repeated twice. The temperature was controlled with an accuracy of ±0.2 °C. Moreover, the measurements were performed in isothermal mode at the ambient temperature in a

wide range of relative humidity (RH) from 30% to 85%, which was obtained using various salts (their saturated aqueous solutions). For the thin-film configuration, measurements were made in the frequency range from 0.1 Hz to 0.1 MHz at a voltage of 20 mV at ambient temperature in a wide range of RH from 30% to 85%. Two sets of thin-film measurements were performed: (i) surface and (ii) cross-section. In this case, for the electrical contacts, gold electrodes (surface 4 × 2 mm) were deposited 4 mm apart on the sample surface using a Sputter Coater SC7620 (Quorum Technologies, Laughton, UK). To make a connection with the instrument cell, for (i) surface measurements, platinum wires were attached to the surface of gold pads, whereas for (ii) cross-section measurements, platinum wires (Advent Research Materials Ltd, Oxford, UK) were attached to the surface of the FTO glass substrate and one gold pad on thin-film, thus enabling electrical characterization of the film in the cross-sectional geometry, in addition to the surface. In order to check the reliability of the results for both thin-film samples and pellets, measurements were performed three times (each time on different sample areas and samples, respectively) under identical experimental conditions (Figure 1b). The experimental data were analyzed by equivalent circuit modelling using the complex non-linear least-squares (CNLLSQ) fitting procedure and the corresponding parameters were determined using WinFit software, Version 3.2 (Novocontrol Technologies, Montabaur, Germany). The procedure is based on the fitting of the experimental impedance to a suitable equivalent circuit model. The values of the resistance obtained from the fitting procedures, R, and electrode dimensions (d is sample thickness and A is electrode area) were used to calculate the DC conductivity, $\sigma_{DC} = d/(R \times A)$.

3. Results

3.1. Structural and Microstructural Analysis of Powders

The diffraction pattern of the prepared CeO_2 powder sample is shown in Figure 2. All diffraction peaks, with angles and intensities, correspond to fluorite cubic structure ceria (ICDD PDF#34-0394). High purity powders were obtained; no other peaks were observed, indicating the presence of additional phases. The ceria phase yields crystallites in the nanometer size range as indicated by the moderately broadened peaks. The average crystallite size was calculated through the Scherrer equation from the (220) peak, broadening to 23 nm.

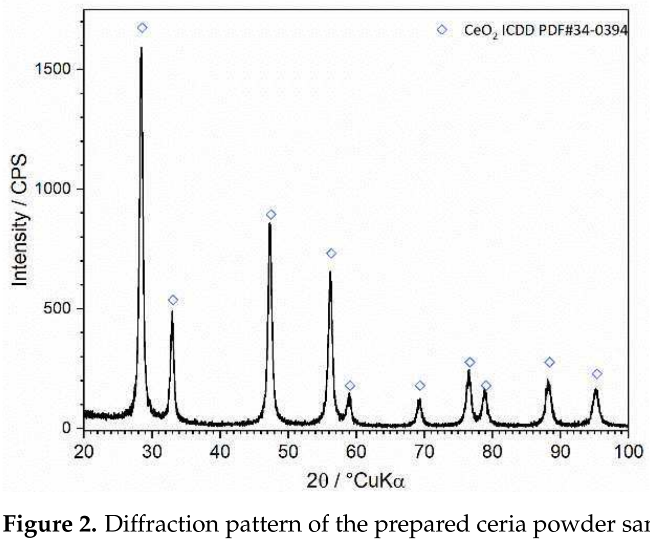

Figure 2. Diffraction pattern of the prepared ceria powder sample.

In Figure 3 micrographs of the same sample at different magnifications are shown. In Figure 3a, small, relatively isotropic particles could be observed, with relatively narrow size distribution, but fairly agglomerated. On the other hand, Figure 3b gives a better perspective on the spherical particles. However, the grain boundaries remain relatively unclear. Thus the image analysis software was unable to accomplish the determination of

particle size; however, it could be estimated that the average particle size is slightly greater than 23 nm.

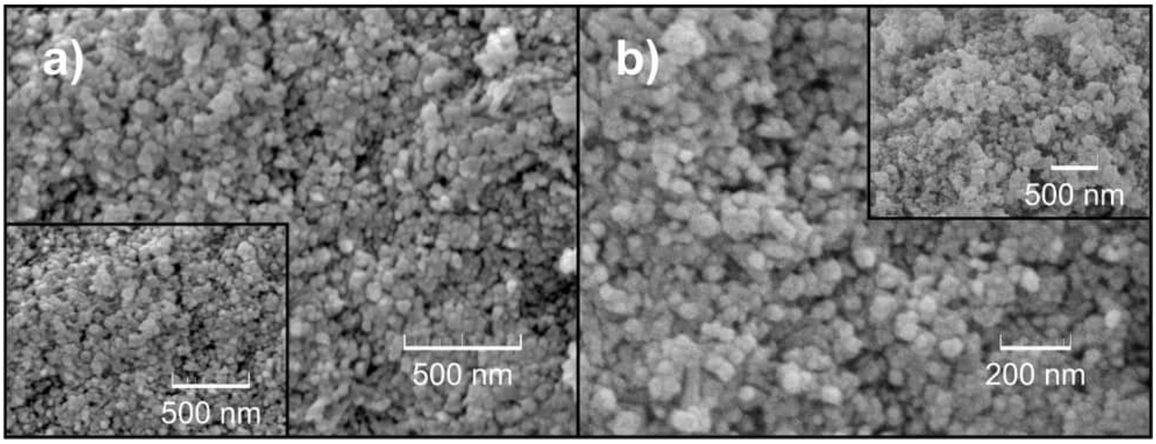

Figure 3. FE-SEM micrographs of the prepared powder CeO_2 sample: (**a**) smaller magnification; (**b**) greater magnification.

The N_2 adsorption-desorption isotherms and pore size distribution are presented in Figure 4a. The isotherms according to the IUPAC classification belong to type IV with a H3 hysteresis loop, which refers to mesoporous materials and indicates the formation of particle aggregates [28]. The specific surface area of the powder sample was calculated to be 38.7 m^2 g^{-1} and a pore volume of 0.15 cm^3 g^{-1}, while the average pore size was calculated as 15.7 nm. It is shown that the pore volume plays a crucial role in terms of sensor performance. While large pore volume (i.e., high surface area) is beneficial for humidity sensor sensitivity due to the ease with which water (H_3O^+ ions) passes easily through the pores and influences electrical conductivity, small pore size is important, to define the detection limit of the sensor. By decreasing the pore size, the quantity of water intake is limited and the influence on electrical conductivity is reduced and controlled, resulting in a lower limit of detection [1,29].

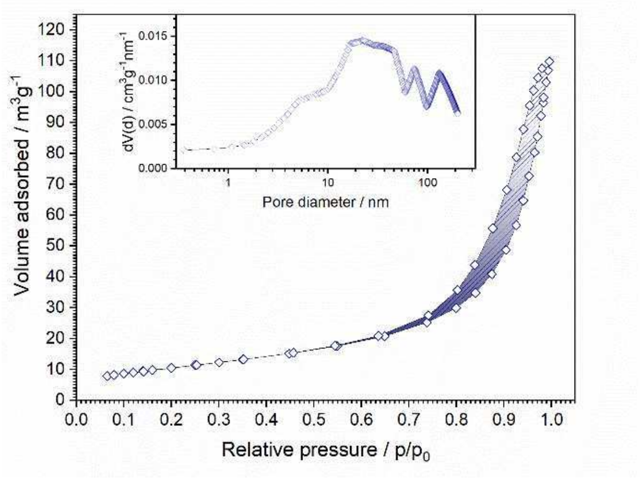

(**a**)

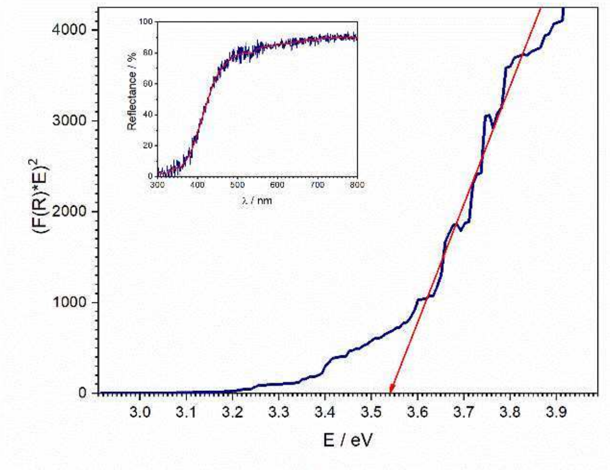

(**b**)

Figure 4. (**a**) N_2 adsorption-desorption isotherms of the prepared powder; (**b**) Tauc's plot for direct transition; and Inset: UV–Vis diffuse reflectance spectra (inset) of the prepared sample.

The bandgap of the powder was estimated from UV-Vis spectroscopy (Figure 4b). The inset in Figure 4b shows the UV–Vis spectrum of the powder. The spectrum shows absorbance in the UV region and reflectance in the visible region. The ceria absorption

edge arises from direct transitions from the top of the valence band (O 2p states) to empty 4f-shells of Ce^{4+} in CeO_2. [30,31]. Therefore, $(F(R)h\nu)^2$ has been plotted versus incident photon energy ($h\nu$) (Tauc's plot) and, by extrapolation of the linear portion onto the energy axis, the value of the direct bandgap energy was obtained as 3.54 eV (Figure 4b). The obtained value is considerably greater than the bulk CeO_2 literature value (3.19 eV) [32], i.e., a blue shift of the absorption band is observed. Such behavior is explained by the alteration of electron energy levels from continuous in bulk to discrete in nanoscale ceria. Blueshift is then a consequence of the fact that electrons have to cross a greater distance in terms of energy, and produce photons of greater energy and shorter-wavelength [33].

3.2. Structural and Microstructural Analysis of Thin-Films

In the grazing incidence diffraction patterns (Figure 5a), the presence of a single phase can be observed which is attributed, as already observed for powder CeO_2 sample, to cerium(IV) oxide (ICDD PDF #34-0394). The depth profiling at different angles of incidence points to the homogeneous ceria phase which is fully in concordance with the tape-casting deposition of previously prepared particulate material.

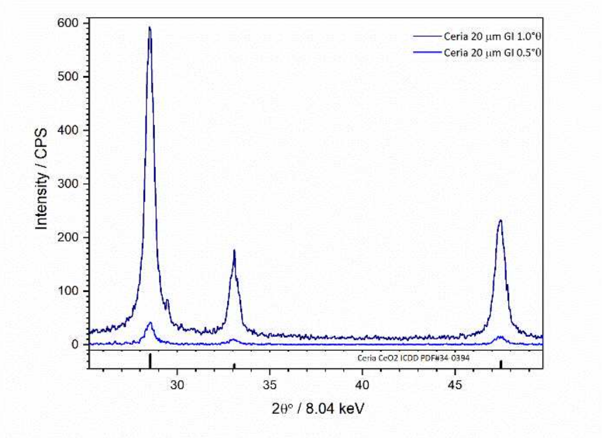

(a)

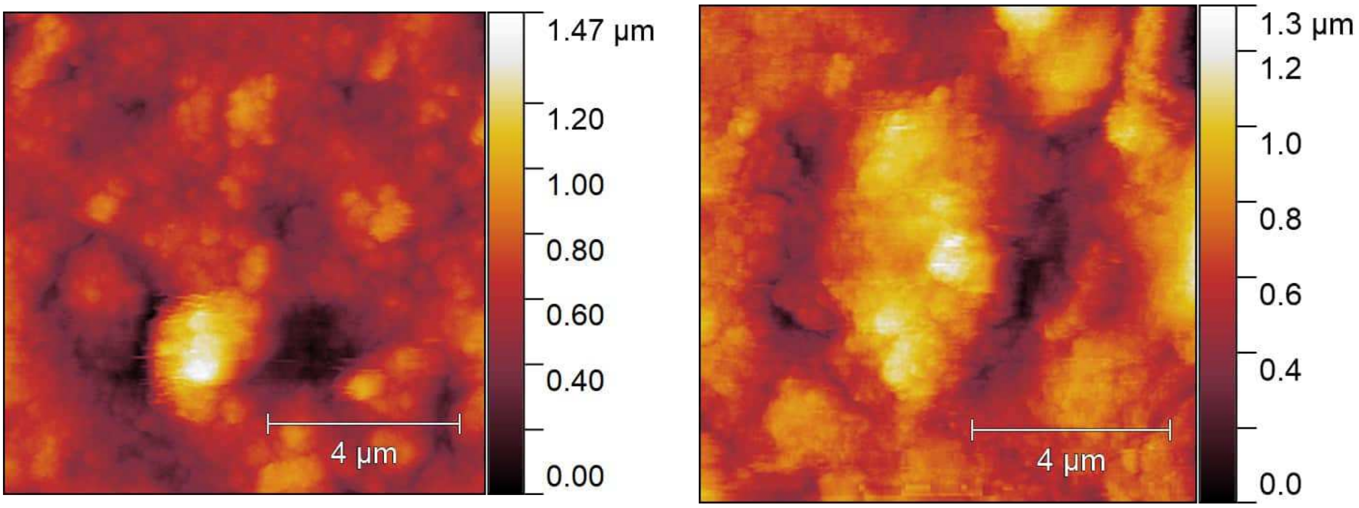

(b)

Figure 5. (**a**) Grazing incidence X-ray diffraction pattern of thin-film sample with 20 µm nominal thickness. (**b**) AFM micrographs of thin-film samples with 40 µm (**left**) and 80 µm (**right**) thickness, where Sq for 40 µm thick sample is 272.79 nm, and for 80 µm thick sample is 287.67 nm, respectively.

Figure 5b shows AFM micrographs of the thin-film with a thickness of 40 and 80 µm, respectively. Based on micrographs, it could be observed that thin-film is crack-free and has a granular surface. Several thin-film samples were examined to determine the range of surface roughness. The surface roughness (*S*q) is determined by squaring each value of Z (height) in the sample and rooting the arithmetic mean of these values. The roughness is thus calculated as the arithmetical average of the absolute heights in the entire height profile of the sample so that the presence of a smaller number of larger deviations can affect the roughness. For the cerium (IV) oxide samples, the topography results are consistent with the film thickness and surface roughness: sq 272.79 nm for the 40 µm thin-film and 287.76 nm for the 80 µm thin-film, which shows that the sample of 40 µm thickness consists of smaller sized particles (or, to be precise, agglomerates) which form a smoother surface than the 80 µm sample.

3.3. Electrical Properties

3.3.1. Thick Pellet Configuration: Temperature-Dependent Conductivity

Figure 6 shows conductivity spectra at different temperatures for the ceria sample in the form of a thick pellet in the heating/cooling cycles. The conductivity isotherms are similar in shape, and different spectral features can be observed. Firstly, it can be observed that heating runs (Figure 6a,c) show a restricted range and are random in terms of the temperature variation of conductivity in comparison to cooling runs (Figure 6b,d). In the latter, a nearly frequency-independent conductivity related to the long-range transport of charge carriers (DC conductivity) is observed at low frequencies, whereas with increasing frequency conductivity dispersion occurs in a power-law fashion due to their localized short-range motions. In the lowest-frequency region of spectra at highest temperatures, a decrease in conductivity is also visible due to the electrode polarization effect at the sample surface [34–38].

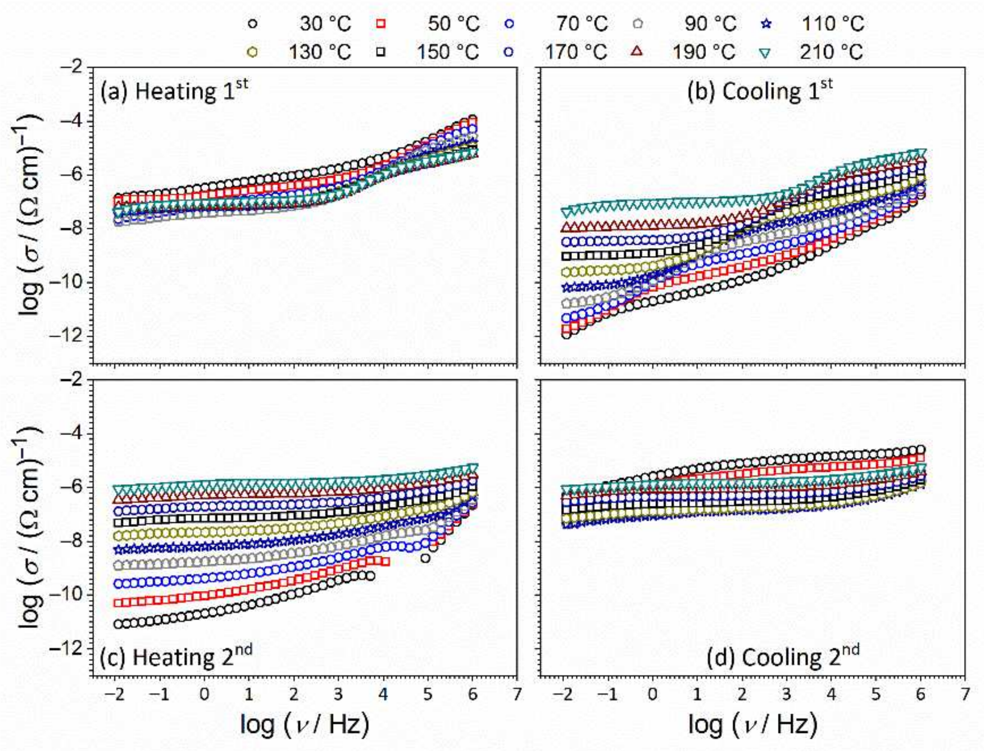

Figure 6. Conductivity spectra for CeO_2 sample in form of a thick pellet (bulk) in: (**a**,**b**) 1st; and (**c**,**d**) 2nd heating/cooling runs, respectively.

The temperature-dependent measurements were performed in a nitrogen atmosphere which resulted in different trends of DC conductivity in the heating/cooling cycles. In the heating runs (Figure 6a,c), the sample is dehydrated and water molecules become desorbed from the surface, while in the cooling runs (Figure 6b,d), the DC conductivity is thermally activated and increases with temperature showing semiconducting behavior, which was not the case in the heating runs [13,21,22].

The conductivity spectra for the CeO_2 sample in the form of a thick pellet measured at 90 °C in the first and second cooling run are shown in Figure 7a. The effect of heating/cooling runs on structural relaxation and the effect on the electrical properties are observed. As mentioned earlier, the conductivity spectra mainly show two typical features: (I) a plateau at low-frequencies that corresponds to the DC conductivity, and (II) dispersion at higher frequencies which is related to the short-range transport of charge carriers. When cycling, the shape of conductivity dispersion changes. Indication of the additional process is observed for the first cooling run in the lower frequency range, which starts to be visible for 90 °C conductivity isotherms (Figure 7a). This additional process shifts along with temperature to higher frequencies. Moreover, in the second cooling run, the process at lower frequencies completely disappears from the spectra.

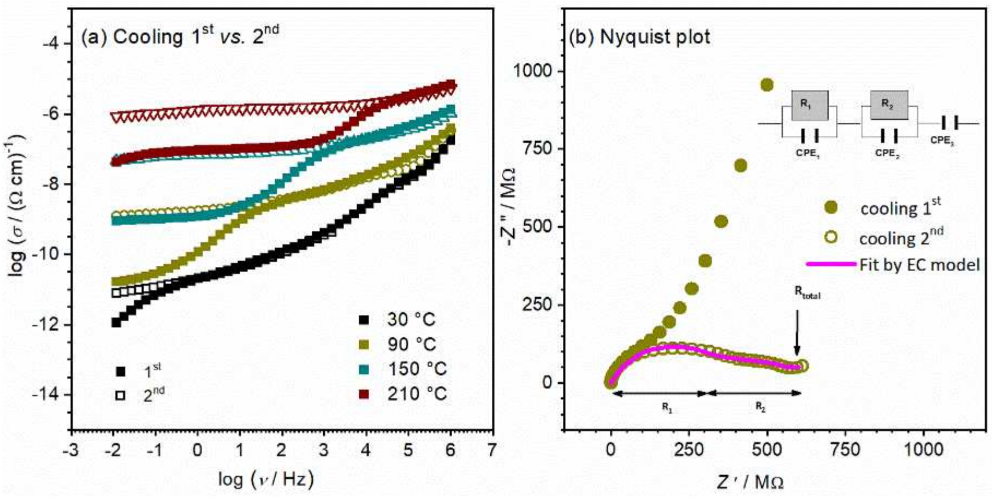

Figure 7. (**a**) Comparison of conductivity spectra at various temperatures in different cooling runs; and (**b**) complex impedance plane (Nyquist plot) for CeO_2 bulk sample in the configuration of pellet at 90 °C.

To shed more light on this interesting effect, the obtained experimental results are presented differently. The Nyquist plot for the CeO_2 sample in the form of a pellet for the corresponding first and second cooling run at 90 °C is presented in Figure 7b. At first glance, it can be seen that the impedance spectra consist of two overlapping but still well-formed semicircles and a low-frequency spur. The presence of multiple semicircles/spurs can be related to different electrical processes in a material [39,40]. The spectrum of the second run shows lower values in comparison to the first, and different ratios of the observed processes. The initial step in the interpretation is to choose an appropriate electrical equivalent circuit (EEC) model and then estimate the parameters of the chosen model. The corresponding equivalent circuit model used for fitting the experimental data for the second run spectrum is shown in Figure 7b and fitting parameters are listed in Table 1.

Table 1. The fitting parameters were obtained from equivalent circuit modelling of complex impedance spectra measured at various temperatures for a CeO_2 sample in the form of a pellet.

Parameters	Temperature /°C		
	**90**	**150**	**210**
R_1/Ω	3.01×10^8	5.09×10^6	2.79×10^5
$A_1/(s^\alpha\ \Omega^{-1})$	4.05×10^{-11}	6.06×10^{-11}	7.97×10^{-11}
α_1	0.72	0.80	0.82
C_1 */F	3.70×10^{-12}	8.03×10^{-12}	7.59×10^{-12}
R_2/Ω	3.15×10^8	5.44×10^6	2.20×10^5
$A_2/(s^\alpha\ \Omega^{-1})$	1.55×10^{-9}	2.69×10^{-9}	6.85×10^{-9}
α_2	0.47	0.59	0.62
C_2 */F	1.55×10^{-10}	1.43×10^{-10}	1.28×10^{-10}
$A_3/(s^\alpha\ \Omega^{-1})$	1.53×10^{-7}	6.28×10^{-7}	5.14×10^{-6}
α_3	0.61	0.46	0.41

* Capacity (*C*) calculated from the equation: $C = A(\omega_{max})^{\alpha-1.}$

According to the appropriate equivalent circuit model used, ongoing processes can be identified and separated based on the order of magnitude of the obtained fitting parameters [41–43]. The complex impedance spectrum is described by two parallel equivalent circuits (R-CPE) connected in series. An individual impedance semicircle can be represented by an equivalent circuit consisting of a resistor and capacitor connected in parallel. Ideally, such a semicircle passes through the origin of a complex plot and yields a low-frequency intercept on the real axis corresponding to the resistance, *R*, of the corresponding process. However, the experimental data obtained show a depressed semicircle whose center is below the real axis, and in such cases, a constant phase element (CPE) is used, instead of the ordinary capacitor in equivalent circuits. The CPE is an empirical impedance function of the type:

$$Z^*{}_{CPE} = \frac{1}{A(i\omega)^\alpha} \quad (1)$$

where A and α are the constants. For the low-frequency spur, the third CPE element connected in series is added to the model. The parameters for each circuit element (*R*, *A*, and α) were obtained directly from the measured impedance data using the complex non-linear least square (CNLLSQ) fitting procedure.

The semicircle at higher frequencies corresponds to the sample bulk (equivalent circuit R1-CPE1), whereas the semicircle at low frequencies (R2-CPE2), is due to the grain boundaries effect. Additionally, the spur at low frequencies is connected with the surface-electrode effect. From the values of resistance, obtained from equivalent circuit modeling along with sample geometry, we evaluated the total DC conductivity (Table 2). From cycle comparison in Figure 7a,b, it can be clearly seen that processes that correspond to bulk have the same extent in both cycles. Furthermore, differences are obvious in the process related to the grain boundaries effect, which is present at lower frequencies as a result of the polycrystalline form of the CeO_2 sample pressed in the form of a pellet. With cycling, this additional contribution to total DC conductivity is diminished by a few orders of magnitude but is still present in the second cooling run. It appears that better connectivity between the grains occurs with cycling, which has a positive impact on conductivity.

Table 2. DC conductivity, σ_{DC}, the activation energy of total DC conductivity, E_{DC}, and activation energies for each particular contribution (grain and grain boundary) for the CeO_2 sample in the form of a pellet.

**CeO_2 Sample—Pellet Form**	
σ_{DC} [a]/$(\Omega\ cm)^{-1}$	1.22×10^{-9}
E_{DC}/eV	0.88
E_g/eV	0.87
E_{gb}/eV	0.90

[a] DC conductivity at 90 °C.

Even though the above-proposed interpretation originally referred to ceramics, [42–44], it can be applied to any other solid materials with conductive crystalline grains and grain boundaries, which is further supported by the determined capacitance values presented in Table 1. The interpretation is directly connected to the volume fraction of each region present in the samples, i.e., small capacity values correspond to large volume fractions (bulk), while large capacity values are attributed to the phase with lower volume fraction. Therefore, order of magnitude values of fitting parameters obtained using equivalent circuit modeling allows the determination of the electrical properties of different regions within the electrode-material system. In general, low capacitance values correspond to the largest volume fraction, bulk, ~10^{-12} F, while higher values between 10^{-11} and 10^{-8} F are related to the processes with lower volume fractions. Hence, the obtained capacitance values in our study for sample CeO_2 in pellet form of 3.70×10^{-12} F and 1.55×10^{-10} F are consistent with grain and grain boundary effects, respectively. Moreover, the activation energy for DC conductivity for the observed process (grain, grain boundary, and total) is calculated from the slope of $\log(1/R_x)$ vs. $1000/T$ presented in Figure 8a and listed in Table 2.

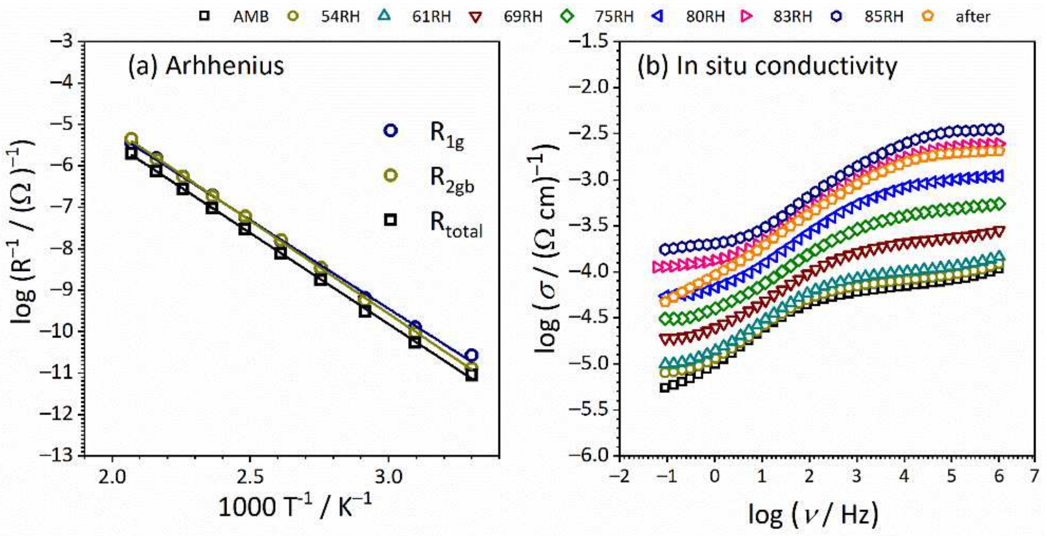

Figure 8. (**a**) Temperature dependence of grain, grain boundary, and total resistance ($\log(1/R)$ vs. $1000/T$) for CeO_2 sample in pellet form. Solid lines represent the least-square linear fits to experimental data; (**b**) Conductivity spectra at ambient temperature for CeO_2 sample in form of pellet measured under controlled relative humidity (RH), from 30% (ambient) up to 85%.

The values for the activation energy related to different processes (i.e., grain and grain boundaries) are close to each other, ranging from 0.87 to 0.90 eV. The approximated total DC conductivity at 90 °C is evaluated to be 2.5×10^{-9} $(\Omega\ cm)^{-1}$ using the equation: $\sigma_{DC} = d/(R_{total} \times S)$ and correlates to the range of semiconductors. Here, it is interesting to observe that the resistance of grain is at the same order of magnitude in comparison to the resistance related to the grain boundary (~10^8), which results in a conductivity decrease of

0.3 orders of magnitude. Thus, we can see a slight blocking effect on long-range charge transfer which hampers the faster transport process in the crystalline grain interior.

3.3.2. Thick Pellet Configuration: Humidity-Dependent Conductivity

In Figure 8b the conductivity spectra recorded in a wide relative humidity (RH) range, from 30% (ambient) to 85% for CeO_2 sample in pellet form, is presented. The spectrum at ambient conditions (30% RH) is similar to the measurements for second heating run (see Figure 6c). One can see that high conductivity values are observed, which are related to the atmosphere and condition measurements. For impedance temperature measurements, nitrogen atmosphere has a negative effect on conductivity, which is the opposite if we are talking about ambient conditions and atmosphere (air). Under ambient conditions, water molecules are adsorbed on the surface and the sample is not dehydrated, resulting in the relatively high conductivity observed [45].

Under controlled humidity, with an increase in RH, the shape of the conductivity spectra remains the same. However, a shift to higher conductivity values along the y-axis is present. Humidity/moisture has a positive effect through a wide frequency range, at the same time on long as well as on short-range charge carriers transport. An interesting effect is observed after the measurements. The relaxation is slow and conductivity values need a long time to return to the initial values. This implies that thickness plays a crucial role in the relaxation process.

To obtain more insight into present processes in controlled relative humidity environments, the impedance data are plotted as the Nyquist plot (see Figure 9). The corresponding equivalent circuit model used to fit the experimental data is shown in Figure 9 and fitting parameters are given in Table 3. The less pronounced semicircle at higher frequencies corresponds to the sample bulk (equivalent circuit R1-CPE1), whereas the dominant semicircle at low frequencies (R2-CPE2), is due to the grain boundaries/surface effects. The proposed interpretation is in line with calculated capacitance values of ~10^{-11} F and 10^{-6} F and related to bulk, along with grain and surface effects, respectively. From the values of determined resistance, along with sample geometry, the total DC conductivity is evaluated (see Table 3). One can see how the increase in humidity has a positive effect on the total DC conductivity, similarly to the temperature effect with semiconducting behavior. In the case of conductivity, an increase in RH from 30 to 85% results in a pronounced jump of one order and half in DC conductivity, from 3.81×10^{-6} $(\Omega\ cm)^{-1}$ to 9.91×10^{-5} $(\Omega\ cm)^{-1}$, respectively.

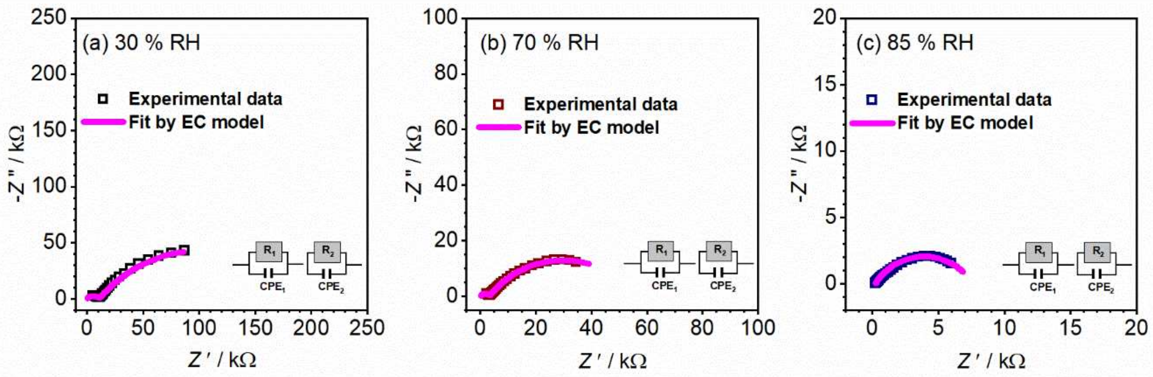

Figure 9. Complex impedance spectra for sample CeO_2 sample in the form of pellet measured at various relative humidities (**a**) 30%, (**b**) 70%, and (**c**) 85%. The symbols (colored open squares) denote experimental values, whereas the solid magenta line corresponds to the best fit. The corresponding equivalent circuit model is composed of multiple parallel combinations of the resistor (R) and the constant-phase element (CPE), used for fitting the data of individual spectra. The goodness of the fit (i.e., chi-squared value) is in the range of 0.001–0.02.

Table 3. The fitting parameters obtained from equivalent circuit modeling of complex impedance spectra measured at various relative humidities (RH) for CeO_2 sample in pellet form.

Parameters	Relative Humidity (RH)		
	**30%**	**70%**	**85%**
R_1/Ω	10901	3345	292
$A_1/(s^{\alpha}\ \Omega^{-1})$	1.43×10^{-8}	2.49×10^{-8}	4.61×10^{-8}
α_1	0.56	0.61	0.63
C_1 */F	1.46×10^{-11}	6.14×10^{-11}	6.35×10^{-11}
R_2/Ω	1.87×10^{5}	5.25×10^{4}	7.31×10^{3}
$A_2/(s^{\alpha}\ \Omega^{-1})$	5.07×10^{-6}	7.71×10^{-6}	2.05×10^{-5}
α_2	0.55	0.58	0.64
C_2 */F	4.85×10^{-6}	4.01×10^{-6}	7.05×10^{-6}
σ_{DC} [a] $/(\Omega\ \text{cm})^{-1}$	3.81×10^{-6}	1.35×10^{-5}	9.91×10^{-5}

* Capacity (C) calculated from the equation: $C = A(\omega_{max})^{\alpha-1}$. [a] DC total calculated from the sum of R_1 and R_2.

Furthermore, noted changes in the conductivity spectra could be described as a result of humidity changes, i.e., the moisture increase and impact on the processes observed and their ratios in the CeO_2 sample in the form of a pellet. Thus, the concentration of charge carriers increases first on the surface of the sample, and with time the influence moves further through the sample, which can be seen by changes in the shape of spectra and corresponding calculated parameters (Figure 9 and Table 3).

As the relative humidity increases, the high-frequency semicircle associated with the bulk process becomes less pronounced and shifts to the edge of the frequency range of the measurements. At the same time, the low-frequency semicircle starts to dominate the spectrum, which could clearly be seen at 85% RH. The change of humidity affects the process starting on the surface and moving inside the sample, and directly impacts the resistivity of the grain boundaries and capacitance values.

3.3.3. Thin-film Configuration: Controlled Relative Humidity

After a detailed investigation of the CeO_2 sample in pellet form and its temperature-dependent electrical properties and sensitivity to the humidified environment, thin-film configuration and corresponding properties of the CeO_2 sample were investigated. The state in mind should be changed from pellet (bulk) mode to thin-film (both surface and cross-section mode). As can be seen, the relaxation of the bulk sample is relatively slow, so the idea is to investigate the influence of sample thickness on sample properties. The conductivity spectra at ambient temperature for CeO_2 thin-film samples with different thicknesses and setup (surface vs. cross-section) measured under controlled relative humidity (RH), from 30% (ambient) to 85%, are presented in Figure 10. The obtained results are interesting in a few aspects. For example, one can see that film thickness (i.e., 40 μm and 80 μm, respectively) has an impact on the shape of conductivity spectra. For different setups, the shape of the spectra also remains the same for each thickness under controlled relative humidity.

At ambient relative humidity (i.e., 30%), the DC surface conductivity is almost the same for 40 and 80 μm (Figure 10), having a value of ~2.25×10^{-7} (Ω square) which implies that film thickness does not directly influence surface conductivity of CeO_2 samples under steady air atmosphere and ambient conditions, just the shape of spectra, as noted before. On the other hand, a comparison of spectra at ambient conditions for cross-section setup reveals an increase in DC conductivity by half an order of magnitude, when the thin-film thickness is increased from 40 to 80 μm. DC conductivity increases from 1.20×10^{-8} $(\Omega\ \text{cm})^{-1}$ to 5.25×10^{-8} (Ω cm$)^{-1}$. The observed effect of thickness is in line with further increase of conductivity at ambient conditions observed for the pellet of CeO_2 sample having a value of total DC conductivity equal to 3.98×10^{-6} $(\Omega\ \text{cm})^{-1}$ and a bulk DC conductivity of 6.92×10^{-5} $(\Omega\ \text{cm})^{-1}$. A direct correlation between surface and cross-section values cannot be made due to the different geometry setups and consequent measuring units.

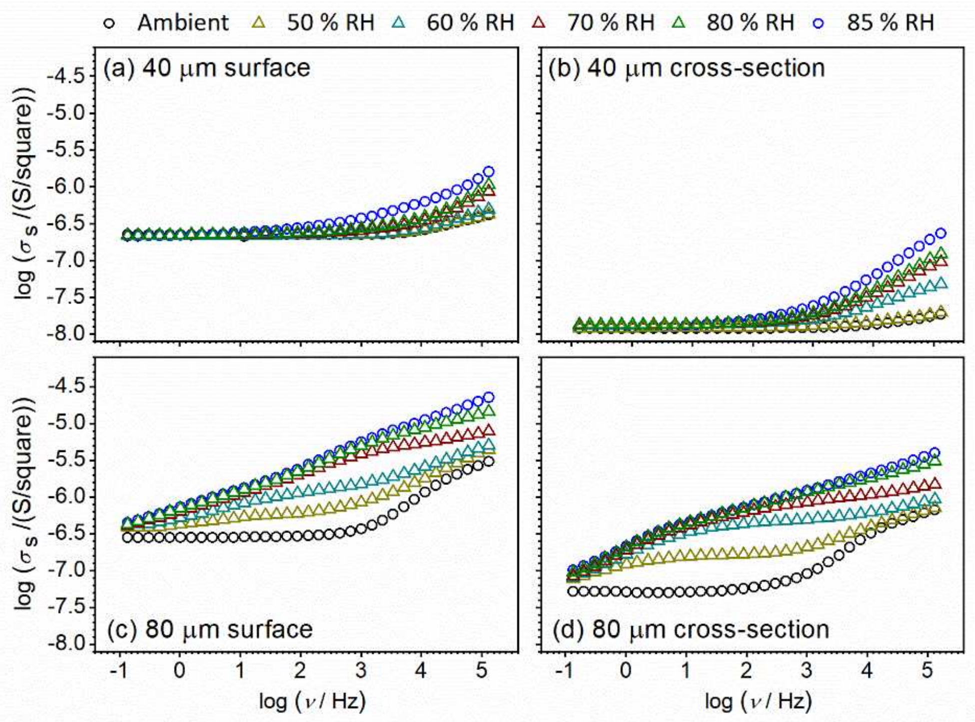

Figure 10. Conductivity spectra for CeO_2 sample prepared in thin-film configuration with a thickness of: (**a**,**b**) 40 μm, and (**c**,**d**) 80 μm.

Interestingly, going back to the shape of the conductivity spectra, differences in spectra with changes in relative humidity for thinner thin-film samples are visible only at higher frequencies (dispersion part of spectra, which corresponds to localized short-range charge carriers) in both configurations (surface vs. cross-section) (Figure 10a,b). At the same time, the DC part of the spectra which corresponds to long-range charge carriers remains unaffected during exposure to a humidified environment. With an increase in thickness from 40 to 80 μm, conductivity starts to be sensitive to the RH conditions in both surface and cross-section configuration, through the wide measured frequency range (Figure 10c,d). Relative humidity influences, at the same time, long-range and localized short-range charge carriers. Moreover, in addition to in-situ measurements, we used another saturated aqueous solution (NaCl) and obtained saturated static values of 70% RH for the studied CeO_2 samples, along with 85% RH. In this way, we can compare the conductivity spectra in in-situ and static modes. In Figure 11, conductivity spectra at two different static RH values, 70% and 85%, respectively, are presented. A good correlation is observed between 70% RH spectra obtained in in-situ and static mode.

The observation that thicker film (80 vs. 40 μm) is, similarly to pellet, more sensitive to relative humidity, foreshadows once again a gradual transition from thin-film to bulk configuration with an increase in thickness. In an attempt to shed more light on the effect observed above on sensitivity to humidified environments, impedance data are presented in the Nyquist plot presentation (Figures 12 and 13).

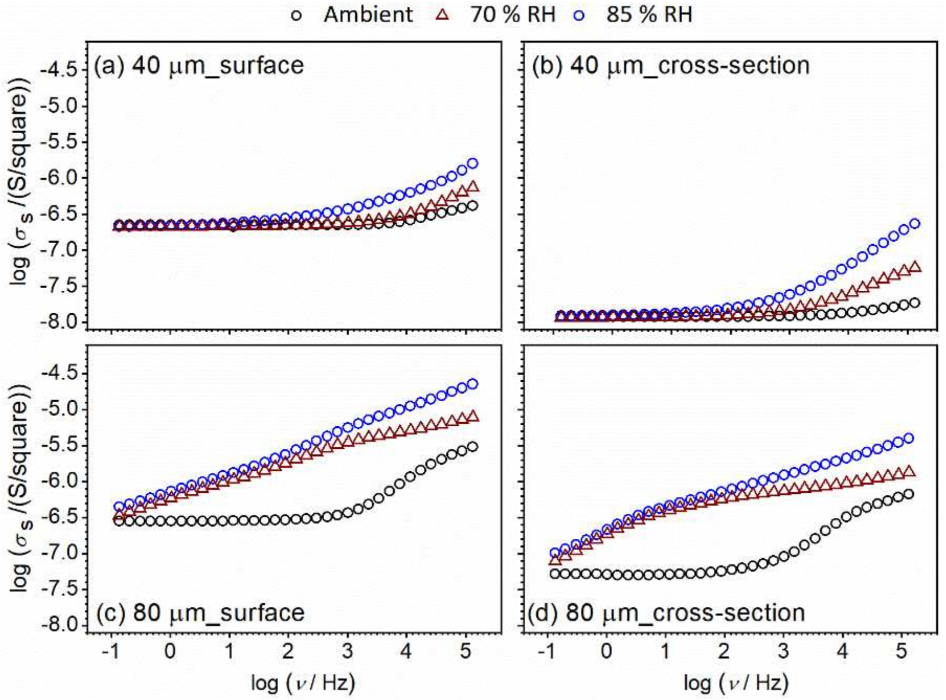

Figure 11. Conductivity spectra for CeO_2 sample prepared in thin-film form with a thickness of: (**a**,**b**) 40 µm; and (**c**,**d**) 80 µm. Different configuration setup: (**a**,**c**) surface; and (**b**,**d**) cross-section, measured at ambient temperature and under controlled relative humidity (RH): ambient, 70% and 85%. Relative humidity stabilization is achieved with a saturated aqueous solution.

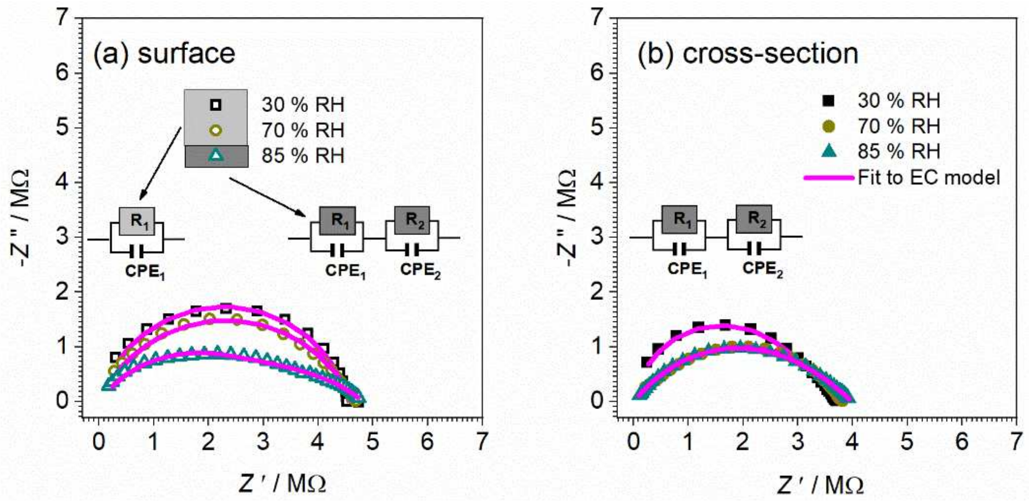

Figure 12. Complex impedance spectra for CeO_2 sample prepared in thin-film form with a thickness of 40 µm measured at ambient temperature with different configuration setups: (**a**) surface and (**b**) cross-section, and under controlled relative humidity (RH): ambient—30%, 70%, and 85%. The symbols (colored open/full squares) denote experimental values, whereas the solid magenta line corresponds to the best fit. The corresponding equivalent circuit model is composed of multiple parallel combinations of the resistor (R) and the constant-phase element (CPE), used for fitting the data of individual spectra. The goodness of fit (i.e., chi-squared value) is in the range of 0.0002–0.006.

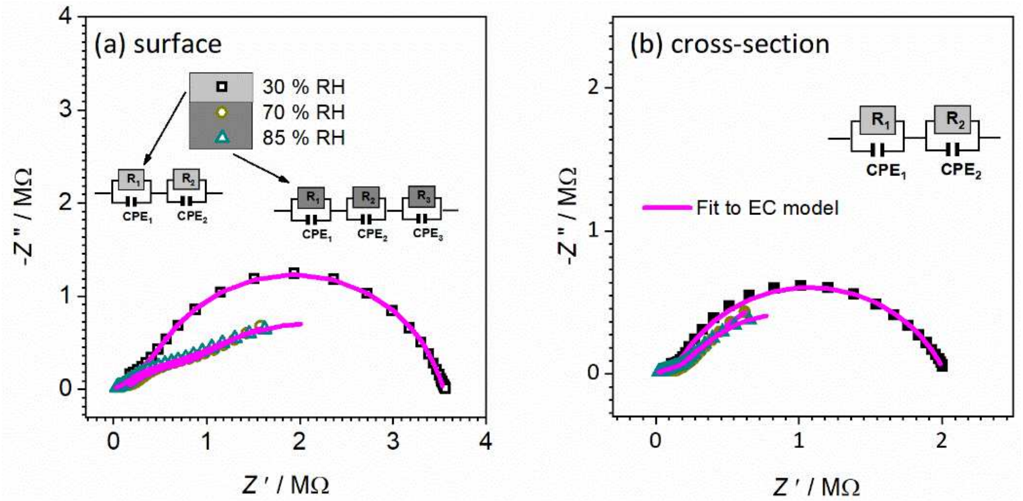

Figure 13. Complex impedance spectra for CeO_2 sample prepared in thin-film form with a thickness of 80 μm measured at ambient temperature in: (**a**) surface, and; (**b**) cross-section configuration, under controlled relative humidity (RH): ambient—30%, 70%, and 85%. The symbols (colored open squares) denote experimental values, whereas the solid magenta line corresponds to the best fit. The corresponding equivalent circuit model is composed of multiple parallel combinations of the resistor (R) and the constant-phase element (CPE), used for fitting the data of individual spectra. The goodness of fit (i.e., chi-squared value) is in the range of 0.0003–0.001.

The corresponding equivalent circuit model used to fit the experimental data for the 40 μm CeO_2 thin-film sample is shown in Figure 12 and fitting parameters obtained from equivalent circuit modeling of complex impedance spectra are given in Table 4.

Table 4. The fitting parameters obtained from equivalent circuit modeling of complex impedance spectra measured at various relative humidity (RH) for CeO_2 sample in the form of thin-film 40 μm: surface and cross-section.

Parameters	Surface Setup/RH%			Cross-Section/RH%		
	30	70	85	30	70	85
R_1/Ω	4.57×10^6	4.64×10^6	2.37×10^6	2.71×10^6	1.84×10^6	2.49×10^6
$A_1/(s^\alpha\ \Omega^{-1})$	1.41×10^{-11}	7.83×10^{-11}	6.23×10^{-10}	4.30×10^{-12}	7.48×10^{-10}	1.81×10^{-9}
α_1	0.83	0.72	0.63	0.94	0.72	0.66
C_1 */F	1.95×10^{-12}	3.60×10^{-12}	1.36×10^{-11}	2.08×10^{-12}	5.77×10^{-11}	1.12×10^{-10}
R_2/Ω	/	/	2.45×10^6	1.00×10^6	1.98×10^6	1.51×10^6
$A_2/(s^\alpha\ \Omega^{-1})$	/	/	2.12×10^{-8}	1.44×10^{-9}	3.06×10^{-9}	2.76×10^{-8}
α_2	/	/	0.45	0.65	0.52	0.45
C_2 */F	/	/	5.71×10^{-10}	4.25×10^{-11}	2.75×10^{-11}	5.68×10^{-10}

* Capacity (*C*) calculated from the equation: $C = A(\omega_{max})^{\alpha-1}$.

For both surface and cross-section measurements, as can be seen, the total DC conductivity values (i.e., total resistance value) remain approximately the same, no matter what the RH conditions, which implies that the RH level does not affect the total resistance/conductivity. On the other hand, for surface and cross-section measurements, the shape of spectra and number of observed processes (semicircles) is gradually changing with higher RH conditions, i.e., a new process is present with moisture increase. For surface setup at ambient and 70% RH conditions, only one electrical process that can be confirmed is related to the process in bulk, which is in line with the calculated capacitance value ~10^{-12} F (Figure 12a). By increasing moisture up to 85% RH, a new process is observed with similar resistance and higher capacitance value which could be related to the effect of moisture on the surface of the thin-film sample. It is interesting to note at this point that

the highest moisture level obtained did not affect the total conductivity but has a positive effect on the bulk process, which is visible in the decrease in the resistance value two-fold (from 4.6 to 2.4 × 10^6 Ω) for a 40 μm thin-film sample measured in surface configuration.

Furthermore, the cross-section measurement configuration shows different trends. In contrast to the surface setup, for all RH conditions, two semicircles are observed related to charge transfer in bulk (high frequencies) and at grain boundaries (low-frequency) with calculated values of the capacitance of ~10^{-12} and ~10^{-10}, respectively. The observed interpretation is in line with that for the pellet (bulk) CeO_2 sample. Turning to the higher RH conditions (70 and 85%), the lower-frequency semicircle is slightly affected by the environment, as confirmed by the change in its shape and corresponding parameters. Due to an increase in RH level, the resistance and capacitance increased (see Figure 12b and Table 4). Based on the following processes and evaluation of complex impedance spectra, it can be concluded that surface measurements turn out to be more sensitive to relative humidity changes, emphasized for higher RH.

Moving forward to thicker thin-film samples, i.e., 80 μm CeO_2, the corresponding equivalent circuit model used for fitting the experimental data is shown in Figure 13 and fitting parameters are given in Table 5. Again, for both surface and cross-section measurements, as can be seen, the total values of DC conductivity values (i.e., total resistance value) remain approximately the same, no matter which RH conditions, except for a small change in the cross-section measurement at the highest RH of 85%. Therefore, it implies that RH level does not affect the total resistance/conductivity, similarly to thinner, 40 μm thick thin-film. Moreover, for both setups at ambient RH, two semicircles are observed at high and low frequencies related to the processes in bulk and grain boundary, respectively.

Table 5. The fitting parameters obtained from equivalent circuit modeling of complex impedance spectra measured at various relative humidities (RH) for CeO_2 sample in the form of thin-film 80 μm: surface and cross-section.

Parameters	Surface Setup/RH%			Cross-Section/RH%		
	30	70	85	30	70	85
R_1/Ω	3.67×10^5	1.59×10^5	1.05×10^5	1.25×10^5	1.58×10^5	2.87×10^5
$A_1/(s^\alpha\ \Omega^{-1})$	2.15×10^{-10}	2.25×10^{-10}	5.54×10^{-8}	6.48×10^{-12}	5.93×10^{-8}	7.43×10^{-7}
α_1	0.71	0.76	0.44	0.98	0.368	0.29
C_1 */F	4.54×10^{-12}	8.87×10^{-12}	7.92×10^{-11}	4.87×10^{-12}	1.95×10^{-11}	1.69×10^{-8}
R_2/Ω	3.17×10^6	6.72×10^5	6.00×10^5	1.89×10^6	1.93×10^6	1.32×10^6
$A_2/(s^\alpha\ \Omega^{-1})$	2.13×10^{-10}	8.14×10^{-8}	8.89×10^{-8}	1.82×10^{-9}	1.08×10^{-6}	1.13×10^{-6}
α_2	0.83	0.54	0.58	0.72	0.70	0.69
C_2 */F	4.78×10^{-11}	6.85×10^{-9}	1.06×10^{-8}	2.00×10^{-10}	1.43×10^{-6}	1.35×10^{-6}
R_3/Ω	–	3.11×10^6	2.98×10^6	–	–	–
$A_3/(s^\alpha\ \Omega^{-1})$	–	4.47×10^{-7}	4.42×10^{-7}	–	–	–
α_3	–	0.59	0.55	–	–	–
C_3*/F	–	5.62×10^{-7}	5.54×10^{-7}	–	–	–

* Capacity (C) calculated from the equation: $C = A(\omega_{max})^{\alpha-1}$.

This effect is similar to the measurements for sample in pellet form and 40 μm sample in cross-section setup where two processes are also observed: as in the 40 μm sample, in this configuration the positive effect of moisture is observed in the bulk process where the decrease in the resistance value is two-fold (from 4.6 to 2.4 × 10^6 Ω) for a 40 μm thin-film sample measured in surface configuration. It seems that, with an increase in thickness, an additional process is also visible for surface set-up measurement. For the surface measurement setup (see Figure 13a), with an increase in RH from ambient level to 70% and 85%, the shape of spectra and number of observed processes (semicircles) gradually changes, i.e., a new process is present with an increase in moisture. This process can be seen in the Nyquist plot as an additional semicircle (the third) at the middle-low frequency range. Moisture for this setup has a strong effect and does not just influence grain boundary

resistance and capacitance, but contributes to a new effect (process). An increase in RH level has a positive effect on the bulk process which is visible in the three-fold decrease in the resistance value (from 3.7 to 1.1 × 10^5 Ω), similarly to the 40 μm thin-film sample measured in the surface configuration. On the other hand, the cross-section setup for 80 μm thick thin-film, see Figure 13b, as mentioned above, has two observed processes. Based on the presented results, we can conclude that surface measurement turns out to be more sensitive to relative humidity changes, emphasized for higher RH, along with an increase in thin-film thickness from 40 to 80 μm.

We also study the relaxation process to ambient conditions for CeO_2 thin-films samples after being exposed to high levels of RH. With moisture variation, the changes occur instantaneously (see Figure 14). The thickness of the film does not affect the relaxation process and remains instant. Therefore, moisture sensitivity is a reversible process for thin-film samples, in contrast to pellet form samples, and could furthermore be investigated with other thickness values to find the boundary for reversibility of humidity sensitivity. Once more, by the relaxation experiment, we showed that moisture directly affects conductivity spectra in the dispersion part, i.e., on the localized short-range charge carriers.

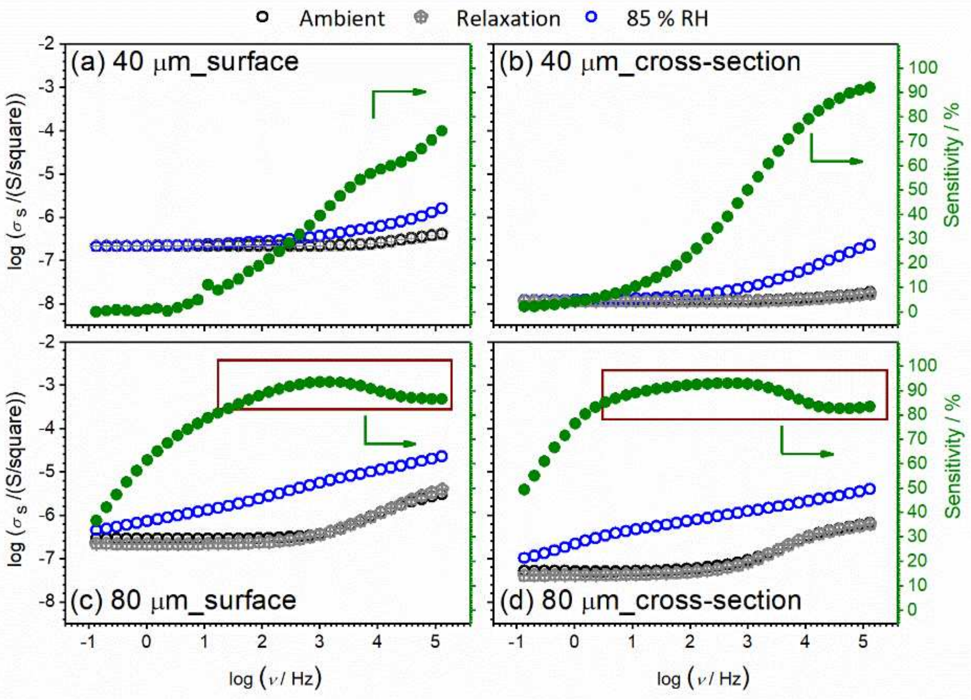

Figure 14. Conductivity spectra for CeO_2 sample prepared in thin-film form with a thickness of (**a**,**b**) 40 μm; and (**c**,**d**) 80 μm. Different configuration setups: (**a**,**c**) surface; and (**b**,**d**) cross-section, measured at ambient temperature and under controlled relative humidity (RH): ambient—30%, 85% and relaxation to ambient after measurements.

Taking the analysis further, we calculated the sensing response (SR) of the thin-film samples with different thicknesses. *SR* was calculated through the whole frequency range of impedance measurements using the expression $SR = 100 \times (R_0 - R_{85})/R_0$, where R_0 is the resistance of the sample at ambient conditions, before exposure to the water vapor (i.e., 30% RH), and R_{85} is the resistance at 85% RH. The humidity sensing response of the studied thin-film sensors is revealed in the right panel in Figure 14. As can be seen, different behavior is observed regarding the measurement configuration and thin-film thickness. In both configurations, humidity sensitivity of 80 um thin-film shows high values, $SR > 80\%$ above 1 Hz and 10 Hz for surface and cross-section, respectively. On the other hand, a constant increase is observed for the thinner sample through the whole frequency range. The observed behavior and trends indicate how the thickness and configuration setup along with the switch from bulk to thin-film play an important role in humidity sensing behavior.

4. Conclusions

Nano-powder of pure ceria was prepared. Pellets were made by pressing the prepared powders. Slurries were prepared and deposited at conductive glass substrates by tape casting method with nominal 10, 20, 40, and 80 μm thickness. Ceria powder yielded crystallites of about 23 nm while slightly greater spherical particles were observed on micrographs, with specific surface area of 38.7 m^2g^{-1} along with a band-gap of 3.55 eV. Depth profiling of tape-casted ceria films shows homogeneous monophasic films with relative roughness of the order of 200 nm (for 80 μm film).

For a bulk sample, the impedance spectra consist of two overlapping, but still, well-shaped, semicircles and a low-frequency spur, corresponding to the sample bulk, the grain boundaries effect and the surface-electrode effect. It was found that, with cycling, there is better connectivity between the grains, which has a positive impact on conductivity.

Under controlled humidity, with an increase in RH, the shape of the conductivity spectrum stays the same, but a shift to higher conductivity values is present. Relaxation is slow and conductivity values need a long time to return to starting values suggesting that the thickness of the pellet plays a crucial role in the relaxation process. The increase in humidity has a positive effect on the total DC conductivity, similar to the temperature effect in semiconducting behavior.

Conductivity spectra at ambient temperature for CeO_2 thin-film samples with different thicknesses and setup (surface vs. cross-section) were measured under controlled relative humidity (RH), from 30% (ambient) up to 85%. At ambient conditions, DC conductivity increases by half order of magnitude for the cross-section setup when the thin-film thickness is increased from 40 to 80 μm.

For the surface measurement setup (80 μm CeO_2), with an increase of RH from ambient level to 70 and 85%, the shape of spectra and number of observed processes (semicircles) gradually changes, i.e., a new process is present along with moisture increase. We can conclude that surface measurement turns out to be more sensitive to relative humidity changes, emphasized for higher RH, along with an increase in thin-film thickness from 40 to 80 μm. Consecutively, moisture sensitivity is a reversible process for thin-film samples, in contrast to pellet form samples.

Author Contributions: Conceptualization, V.M., A.B., L.P.; methodology, V.M., A.B., L.P., I.P., S.K., J.-S.P., Z.S., K.M. and I.K.I.; software, V.M., I.P., A.B. and L.P.; validation, V.M., A.B., L.P. and I.P.; formal analysis, V.M., A.B., L.P., I.P., S.K., J.-S.P., Z.S., K.M. and I.K.I.; investigation, V.M., A.B., L.P., S.K.; resources, V.M., L.P. and S.K.; data curation, V.M., I.P., A.B., L.P. and S.K.; writing (original draft preparation), V.M., A.B., L.P.; writing (review and editing), V.M. and A.B.; visualization, V.M. and A.B.; supervision, V.M.; project administration, V.M. and S.K.; funding acquisition, V.M. and S.K. All authors have read and agreed to the published version of the manuscript.

Funding: This work has been funded by the projects PZS-2019-02-1555 (sensing applicability) in Research Cooperability Program of the Croatian Science Foundation (CSV) funded by the EU from the European Social Fund (ESF) under the Operational Programme Efficient Human Resources 2014–2020, UIP-2019-04-2367 (deriving thin-films) and IP-2018-01-2963 (ceria powder synthesis), of the Croatian Science Foundation, project KK.01.2.1.02.0316 (thin-film characterization) by the European Regional Development Fund (ERDF), Croatian-Chinese bilateral project "Comparison of the doped ceria film catalyst prepared using advanced chemical and physical deposition methods" (humidity sensor testing), EUSMI project E200700427, and Elettra projects 20210495 and 20195555.

Data Availability Statement: Data Availability Statement: Data can be available upon request from the authors.

Acknowledgments: Beamline scientists of the MCX beamline at Synchrotron Elettra Trieste and application scientists of the Nanosurf Langen are gratefully acknowledged for instrumentation access and collaboration. Project Cesar (UP.03.1.1.04.0026), is gratefully acknowledged for instrumentation access. The financial sustenance of the University of Zagreb is gratefully acknowledged.

Conflicts of Interest: The authors declare no conflict of interest. The funders had no role in the design of the study; in the collection, analyses, or interpretation of data; in the writing of the manuscript, or in the decision to publish the results.

References

1. Almar, A.L.; Tarancón, A.; Andreu, T.; Torrell, M.; Hu, Y.; Dezanneau, G.; Morata, A. Mesoporous ceramic oxides as humidity sensors: A case study for gadolinium-doped ceria. *Sens. Actuators B Chem.* **2015**, *216*, 41–48. [CrossRef]
2. Divya, T.; Nikhila, M.P.; Anju, M.; Kusumam, T.A.; Akhila, A.K.; Ravikiran, Y.T.; Renuka, N.K. Nanoceria based thin films as efficient humidity sensors. *Sens. Actuators A Phys.* **2017**, *26*, 85–93. [CrossRef]
3. Sivakumar, A.; Ramya, S.; Dhas, S.S.J.; Almansour, A.I.; Kumar, R.S.; Arumugam, N.; Murugesan, M.; Dhas, S.M.B. Assessment of crystallographic and electronic phase stability of shock wave loaded cubic cerium oxide nanoparticles. *Ceram. Int.* **2022**, *48*, 1963–1968. [CrossRef]
4. Chakraborty, I.; Ghosh, D.; Sathe, S.M.; Dubey, B.K.; Pradhan, D.; Ghangrekar, M.M. Investigating the efficacy of CeO_2 multi-layered triangular nanosheets for augmenting cathodic hydrogen peroxide production in microbial fuel cell. *Electrochim. Acta* **2021**, *398*, 139341. [CrossRef]
5. Kurajica, S.; Munda, I.K.; Dražić, G.; Mandić, V.; Mužina, K.; Bauer, L.; Matijašić, G. Manganese-doped, hydrothermally-derived ceria: The occurrence of birnessite and the distribution of manganese. *Ceram. Int.* **2020**, *46*, 29451–29458. [CrossRef]
6. Salaev, M.A.; Salaeva, A.A.; Kharlamova, T.S.; Mamontov, G.V. Pt–CeO_2-based composites in environmental catalysis: A review. *Appl. Catal. B* **2021**, *295*, 120286. [CrossRef]
7. Traversa, E. Ceramic sensors for humidity detection: The state-of-the-art and future developments. *Sens. Actuators B Chem.* **1995**, *23*, 135–156. [CrossRef]
8. Fu, X.Q.; Wang, C.; Yu, H.C.; Wang, Y.G.; Wang, T.H. Fast humidity sensors based on CeO_2 nanowires. *Nanotechnology* **2007**, *18*, 145503. [CrossRef]
9. Thakur, S.; Patil, P. Rapid synthesis of cerium oxide nanoparticles with superior humidity-sensing performance. *Sens. Actuators B Chem.* **2014**, *194*, 260–268. [CrossRef]
10. Gatea, H.A. The role of substrate temperature on the performance of humidity sensors manufactured from cerium oxide thin films. *J. Mater. Sci. Mater. Electron.* **2020**, *31*, 22119–22130. [CrossRef]
11. Acosta-Silva, Y.J.; Toledano-Ayala, M.; Torres-Delgado, G.; Torres-Pacheco, I.; Méndez-López, A.; Castanedo-Pérez, R.; Zelaya-Ángel, O. Nanostructured CeO_2 thin films prepared by the sol-gel dip-coating method with anomalous behavior of crystallite size and bandgap. *J. Nanomater.* **2019**, *2019*, 5413134. [CrossRef]
12. Kurajica, S.; Macan, J.; Mandić, V.; Galjer, M.; Mužina, K.; Plaisier, J.R. Reinforcing blade-cast photocatalytic-titania thin film by titanate nanotubes. *Mater. Res. Bull.* **2018**, *105*, 142–148. [CrossRef]
13. Wang, B.; Zhu, B.; Yun, S.; Zhang, W.; Xia, C.; Afzal, M.; Cai, Y.; Liu, Y.; Wang, Y.; Wang, H. Fast ionic conduction in semiconductor $CeO_{2-\delta}$ electrolyte fuel cells. *NPG Asia Mater.* **2019**, *11*, 1–12. [CrossRef]
14. Campbell, C.T.; Peden, C.H. Oxygen vacancies and catalysis on ceria surfaces. *Science* **2005**, *309*, 713–714. [CrossRef] [PubMed]
15. Knoblauch, N.; Dörrer, L.; Fielitz, P.; Schmücker, M.; Borchardt, G. Surface controlled reduction kinetics of nominally undoped polycrystalline CeO_2. *Phys. Chem. Chem. Phys.* **2015**, *17*, 5849–5860. [CrossRef]
16. Kulwicki, B.M. Ceramic sensors and transducers. *J. Phys. Chem. Solids* **1984**, *45*, 1015–1031. [CrossRef]
17. Lee, C.Y.; Lee, G.B. Humidity sensors: A review. *Sens. Lett.* **2005**, *3*, 1–15. [CrossRef]
18. Qu, W.; Wlodarski, W. A thin-film sensing element for ozone, humidity and temperature. *Sens. Actuators B Chem.* **2020**, *64*, 42–48. [CrossRef]
19. Anderson, J.H., Jr.; Parks, G.A. Electrical conductivity of silica gel in the presence of adsorbed water. *J. Phys. Chem.* **1968**, *72*, 3662–3668. [CrossRef]
20. Pokhrel, S.; Nagaraja, K.S. Electrical and humidity sensing properties of Chromium (III) oxide–tungsten (VI) oxide composites. *Sens. Actuators B Chem.* **2003**, *92*, 144–150. [CrossRef]
21. Takamura, H.; Takahashi, N. Electrical conductivity of dense nanocrystalline ceria under humidified atmosphere. *Solid State Ion* **2010**, *181*, 100–103. [CrossRef]
22. Zamiri, R.; Abbastabar Ahangar, H.; Kaushal, A.; Zakaria, A.; Zamiri, G.; Tobaldi, D.; Ferreira, J.M.F. Dielectrical properties of CeO_2 nanoparticles at different temperatures. *PLoS ONE* **2015**, *10*, e0122989. [CrossRef]
23. Younis, A.; Loucif, A. Defects mediated enhanced catalytic and humidity sensing performance in ceria nanorods. *Ceram. Int.* **2021**, *47*, 15500–15507. [CrossRef]
24. Chen, H.I.; Chang, H.Y. Synthesis and characterization of nanocrystalline cerium oxide powders by two-stage non-isothermal precipitation. *Solid State Commun.* **2005**, *133*, 593–598. [CrossRef]
25. Plaisier, J.R.; Nodari, L.; Gigli, L.; San Miguel, E.P.R.; Bertoncello, R.; Lausi, A. The X-ray diffraction beamline MCX at Elettra: A case study of non-destructive analysis on stained glass. *Acta Imeko* **2017**, *6*, 71–75. [CrossRef]
26. Valencia, S.; Marín, J.M.; Restrepo, G. Study of the bandgap of synthesized titanium dioxide nanoparticules using the sol-gel method and a hydrothermal treatment. *Open Mater. Sci.* **2009**, *4*, 9–14. [CrossRef]
27. Nečas, D.; Klapetek, P. Gwyddion: An Open-Source Software for SPM Data Analysis. *Open Phys.* **2012**, *10*, 181–188. [CrossRef]

28. Thommes, M.; Kaneko, K.; Neimark, A.V.; Olivier, J.P.; Rodriguez-Reinoso, F.; Rouquerol, J.; Sing, K.S. Physisorption of gases, with special reference to the evaluation of surface area and pore size distribution (IUPAC Technical Report). *Pure Appl. Chem.* **2015**, *87*, 1051–1069. [CrossRef]
29. Seiyama, T.; Yamazoe, N.; Arai, H. Ceramic humidity sensors. *Sens. Actuat.* **1983**, *4*, 85–96. [CrossRef]
30. Dhannia, T.; Jayalekshmi, S.; Kumar, M.S.; Rao, T.P.; Bose, A.C. Effect of iron doping and annealing on structural and optical properties of cerium oxide nanocrystals. *J. Phys. Chem. Solids* **2010**, *71*, 1020–1025. [CrossRef]
31. Zhang, Y.W.; Si, R.; Liao, C.S.; Yan, C.H.; Xiao, C.X.; Kou, Y. Facile alcohothermal synthesis, size-dependent ultraviolet absorption, and enhanced CO conversion activity of ceria nanocrystals. *J. Phys. Chem.* **2003**, *107*, 10159–10167. [CrossRef]
32. Kumar, E.; Selvarajan, P.; Muthuraj, D. Synthesis and characterization of CeO_2 nanocrystals by solvothermal route. *Mater. Res.* **2013**, *16*, 269–276. [CrossRef]
33. Miao, J.J.; Wang, H.; Li, Y.R.; Zhu, J.M.; Zhu, J.J. Ultrasonic-induced synthesis of CeO_2 nanotubes. *J. Cryst. Growth.* **2005**, *281*, 525–529. [CrossRef]
34. Kubuki, S.; Osouda, K.; Ali, A.S.; Khan, I.; Zhang, B.; Kitajou, A.; Okada, S.; Okabayashi, J.; Homonnay, Z.; Kuzmann, E.; et al. ^{57}Fe-Mössbauer and XAFS Studies of Conductive Sodium Phospho-Vanadate Glass as a Cathode Active Material for Na-ion Batteries with Large Capacity. *J. Non-Cryst. Solids* **2021**, *570*, 120998. [CrossRef]
35. Razum, M.; Pavić, L.; Ghussn, L.; Moguš-Milanković, A.; Šantić, A. Transport of potassium ions in niobium phosphate glasses. *J. Am. Ceram. Soc.* **2021**, *104*, 4669–4678. [CrossRef]
36. Jakovac, M.; Klaser, T.; Radatović, B.; Skoko, Ž.; Pavić, L.; Žic, M. Surface Characterization and Conductivity of Two Types of Lithium-Based Glass Ceramics after Accelerating Ageing. *Materials* **2020**, *13*, 5632. [CrossRef]
37. Thoms, E.; Sippel, P.; Reuter, D.; Weiß, M.; Loidl, A.; Krohns, S. Dielectric study on mixtures of ionic liquids. *Sci. Rep.* **2017**, *7*, 7463. [CrossRef] [PubMed]
38. Dubraja, L.A.; Žilić, D.; Olujić, K.; Pavić, L.; Molčanov, K.; Pajić, D. Targeted synthesis of a Cr III–O–VV core oxo-bridged complex: Spectroscopic, magnetic and electrical properties. *New J. Chem.* **2021**, *45*, 6336–6343. [CrossRef]
39. Pavić, L.; Skoko, Ž.; Gajović, A.; Su, D.; Moguš-Milanković, A. Electrical transport in iron phosphate glass-ceramics. *J. Non-Cryst. Solids* **2018**, *502*, 44–53. [CrossRef]
40. Pavicć, L.; Sklepicć, K.; Skoko, Ž.; Tricot, G.; Mošer, P.; Koudelka, L.; Moguš-Milankovicć, A. Ionic conductivity of lithium germanium phosphate glass-ceramics. *J. Phys. Chem. C* **2019**, *123*, 23312–23322. [CrossRef]
41. Page, L.; Adams, N.I. *Principles of Electricity*, 2nd ed.; Van Nostrand: New York, NY, USA, 2019.
42. Irvine, J.T.; Sinclair, D.C.; West, A.R. Electroceramics: Characterization by impedance spectroscopy. *Adv. Mat.* **1990**, *2*, 132–138. [CrossRef]
43. Sinclair, D.C. Characterisation of electro-materials using ac impedance spectroscopy. *Bol. Soc. Esp. Cerám. V* **1995**, *34*, 55–65.
44. Khan, I.; Kuzmann, E.; Nomura, K.; Ali, A.S.; Akiyama, K.; Homonnay, Z.; Sinko, K.; Pavic, L.; Kubuki, S. Structural characterization, electrical and photocatalytic properties of $\alpha-$ and γ-Fe2O3 nanoparticles dispersed in iron aluminosilicate glass. *J. Non-Cryst. Solids* **2021**, *561*, 120756. [CrossRef]
45. Miyake, T.; Rolandi, M. Grotthuss mechanisms: From proton transport in proton wires to bioprotonic devices. *J. Phys. Condens. Matter.* **2015**, *28*, 023001. [CrossRef] [PubMed]

Sensors & Actuators: B. Chemical 374 (2023) 132815

Contents lists available at ScienceDirect

Sensors and Actuators: B. Chemical

journal homepage: www.elsevier.com/locate/snb

Highly-sensitive organic field effect transistor sensors for dual detection of humidity and NO_2

Yangyang Zhu [a], Yiqun Zhang [a], Jiajia Yu [a], Chengren Zhou [a], Chaojie Yang [a], Lu Wang [a], Li Wang [a], Libo Ma [b], Li Juan Wang [a,b,*]

[a] School of Chemical Engineering, Changchun University of Technology, Changchun 130012, China
[b] Institute for Integrative Nanosciences, Leibniz IFW Dresden, Dresden 01069, Germany

ARTICLE INFO

Keywords:
Sensor
Organic field effect transistor
Humidity
Nitrogen dioxide
Dual detection

ABSTRACT

Sensors based on organic field effect transistors have attracted extensive interests, but most sensors are applied to a single analyte detection. Herein, a facile strategy was proposed to optimize the growth of active layers by regulating the thickness of vanadyl-phthalocyanine (VOPc) films and carrying out the thermal annealing. Thanks to the improved film morphology and crystal properties, the carrier mobility of transistors reached 0.15 $cm^2V^{-1}s^{-1}$, humidity and nitrogen dioxide (NO_2) induced multi-parameter response of transistors could be considered as an efficient sensing approach. The sensitivity of threshold voltage reached 0.59 V/% RH ranging from 42%−82% RH. In addition, the gas sensing performance was systematically studied. Sensors based on 10 nm VOPc films achieved a 4710% response to 10 ppm NO_2 gas, with a theoretical limit of detection down to 125.2 ppb, and the response current was improved by two orders of magnitude under the amplification effect of transistor. The obtained gas sensors also exhibited high repeatability after being stored at 90% ambient humidity. It was concluded that different charge carrier transfer mechanism in moisture and NO_2 gas provided a simple idea for tunable applications of phthalocyanine-based transistors.

1. Introduction

In the past few decades, organic field-effect transistors (OFET) have been widely applied in physical, chemical and biological sensors due to their inherent advantages such as room temperature operation, multi-parameter information, signal amplification effects, and mechanical flexibility, to play an increasingly important role in environmental monitoring, medical diagnosis and electronics industry [1–8]. Among various applications, humidity sensors and gas sensors have attracted wide interest because they are closely related to human production and life. For example, monitoring of human respiration and skin humidity level can provide some key information about human health and the extent of wound healing for timely diagnosis and treatment [9–12]. Detection of environmental toxic gases, such as nitrogen dioxide (NO_2) [13,14], sulfur compounds [15], ammonia (NH_3) [16], and volatile organic compounds (VOCs) [17], can effectively prevent potential harm to human health. Sensing behaviors of humidity and gas are mainly attributed to the different interaction mechanisms between active layers and analytes, and are manifested through multi-parameter changes of OFET. Specifically, current, voltage and carrier mobility applied as response mechanisms to humidity or gases have been widely reported [18–21]. However, in most researches, sensors mainly realize the detection of a single analyte, which cannot satisfy the desire for multi-functional transistors.

The existing sensors based on multi-analyte detection can provide more ideas. Zhou et al. have reported organic ambipolar transistors based on 2,5-bis(4-biphenylyl)bithiophene (BP2T) and copper hexadeca-fluorophthalocyanine ($F_{16}CuPc$) heterojunction and utilized different types of carriers for selective responses to multiple analytes including NO_2, NH_3, H_2S, SO_2, higher output signal and stability are obtained [22]. Thus, it is a reliable method to construct heterojunctions of different sensing materials in transistors to achieve multi-analyte response. Li et al. have reported NDI3HU-DTYM2 (NDI) monolayer molecular crystals with a controllable porous structure for NH_3 detection, achieving a low detection limit of 0.1 ppb. In addition, the direct exposure of the conducting channel enables the detection of solid chemicals, the current response to 500 ppb dopamine has been achieved [23]. This research makes it possible to realize multi-analyte detection

* Corresponding author at: School of Chemical Engineering, Changchun University of Technology, Changchun 130012, China.
E-mail addresses: wanglj15@ccut.edu.cn, lijuan.wang@ifw-dresden.de (L.J. Wang).

https://doi.org/10.1016/j.snb.2022.132815
Received 8 May 2022; Received in revised form 20 August 2022; Accepted 7 October 2022
Available online 12 October 2022
0925-4005/© 2022 Elsevier B.V. All rights reserved.

through thickness modulation of active layers. M. Bouvet et al. have reported the response of cobalt phthalocyanine (CoPc) and Co[$(SO_3Na)_{2,3}$Pc] sensors to NH_3 and O_3 gas, which exhibit good linearity in 20–200 ppb O_3 and 20–200 ppm NH_3, and the response of Co[$(SO_3Na)_{2,3}$Pc] sensor to ammonia gas is increased in the presence of humidity [24]. This provides a method like molecular design of sensitive materials for multi-responses to different analytes. It is apparent from the above that the potential of OFET for multifunctional detection has attracted extensive attention. It is worth noting that humidity is often avoided as an important factor affecting the transistor operating at room temperature, so that the dual sensing of humidity and gas is rarely studied. This study adopts a simple strategy to investigate the performance of transistors under humidity and gas.

In this work, transistors for dual detection of humidity and NO_2 gas were prepared by thermally evaporating, *p*-6 P template layer and VOPc sensing films were deposited successively on SiO_2 gate dielectric. Taking advantage of the improved film morphology and crystal properties after thickness modulation and thermal annealing, high-performance transistors were further obtained. Multiple parameters of transistor are highly sensitive to humidity and NO_2 gas and exhibit an opposite variation, which is attributed to the different interaction mechanisms between VOPc and analytes. In addition, the sensors also exhibited low detection limit of NO_2 gas, as well as a considerable repeatability after being stored at 90% RH.

2. Experimental section

2.1. Fabrication of organic films

para-Sexiphenyl (*p*-6 P, 95.0% purity) was purchased from Aladdin Industrial Corporation, Shanghai. Vanadyl-phthalocyanine (VOPc, 99.0% purity) was purchased from Beijing Huawei Ruike Chemical Co., Ltd. The molecular structures of *p*-6 P and VOPc are shown in Fig. 1. All materials were used directly without further purification. The heavily n-doped silicon wafers covered with 300 (±10) nm silicon dioxide (SiO_2) layer were used as the substrates. They were purchased from Suzhou Crystal Silicon Technology Co., Ltd. The devices adopted a bottom gate top contact structure, as shown in Fig. 1. Firstly, the Si/SiO_2 substrates were successively rinsed with acetone, ethanol and deionized water until the surface was free of any impurities. Then, they were purged by nitrogen and dried in an oven at 80 ℃ for 30 min. Afterwards, organic thin films were deposited at a substrate temperature of 180 ℃ by vacuum evaporation method, the vacuum pressure was below 6×10^{-4} Pa. The quartz crystal microbalance was used to monitor the film thickness and the deposition speed (0.2–0.4 nm/min). The thickness of *p*-6 P and VOPc films were 3 nm and 5–30 nm respectively. Finally, the electrodes (Au) with thickness of 40 nm were deposited, the width and length of the channels for OFET are 5000 μm and 250 μm, the width and length of the channels for interdigital electrodes of gas sensor are 97,500 μm and 250 μm.

2.2. Characterization and test

The morphologies of films were characterized by atom force microscopy (AFM, Seiko Instruments Inc., SPI3800N) with tapping mode. Topographical and phase images were obtained simultaneously. The structure of active layers was characterized by D8 Discover thin film diffractometer (Bruker, Germany) with Cu Kα radiation (λ=1.54056 Å, V=40 kV, I=40 mA). It was measured at a step-scan rate of 1°/min and the scattering angle ranged from 2° to 30°.

The electrical characteristics of OFET and sensors were measured by Keithley 2636 A Dual-channel I-V Source Meter. The capacitance-frequency characteristic of SiO_2 dielectric layer was measured by Agilent E4980A LCR Meter. All the tests were performed at room temperature (~20℃).

The schematic diagram of gas sensor test system is shown in Fig. S1. The sensors were placed in a sealed chamber while NO_2 gas with a certain concentration was introduced by mass flow controller system. NO_2 (191.81×10^{-6} V/V) acted as the target gas and the high purity air (80% N_2 and 20% O_2) acted as the carrier gas. The gas flow rate was settled to 200 sccm and 2000 sccm for NO_2 gas and air, respectively. The response current of all sensors was measured at source-drain voltage of 1 V. The gas pulse was kept for 5 or 10 min. In humidity sensing studies, the transistors were exposed in a constant humidity for 10 min, and then the tests were performed, and the humidity remained stable (±1% RH) throughout the test. The gas sensors were exposed in a constant humidity for 10 min and then transferred to a sealed chamber for the exposure of accurate gas concentrations.

3. Results and discussion

3.1. Characterization

Fig. 2 shows the morphologies of organic semiconductor films. It can be seen in Fig. 2a that *p*-6 P shows the mode of island growth after the formation of a continuous and dense film. Fractal islands and nucleation of the third-layer can also be observed, which is similar with the growth behavior at high temperature described in the literature [25]. The large-size domains and continuous *p*-6 P films provides a favorable condition for the growth of VOPc films [26]. Fig. 2b-d show the morphologies of 2 nm, 5 nm and 10 nm VOPc films induced by 3 nm *p*-6 P film, respectively. The VOPc films are composed of many dot-like crystal grains, preferentially aggregating on the *p*-6 P fractal islands. The average size of grains is about 79 nm and the height fluctuates from 3 to 15 nm. As the increase of thickness, the crystal grains grow up through molecular aggregation or merging adjacent particles, and the domains are fully connected to form a continuous film, as shown in Fig. 2d. The improved continuity can result in a significant reduction of defects near the interface of active and dielectric layers, which is beneficial to the carrier transition and the formation of conduction channels [27,28]. Fig. 2e-h show the morphologies of corresponding films annealed at 180 ℃ for 20 min. Thermal annealing promotes the transformation of

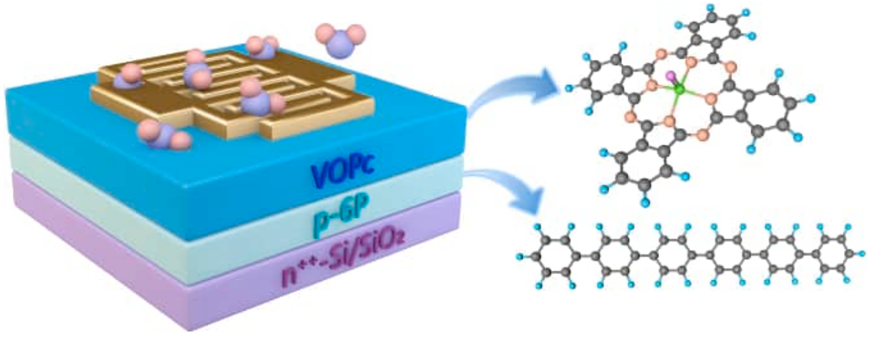

Fig. 1. Schematic diagram of the sensor architecture and the molecule structures of organic semiconductors.

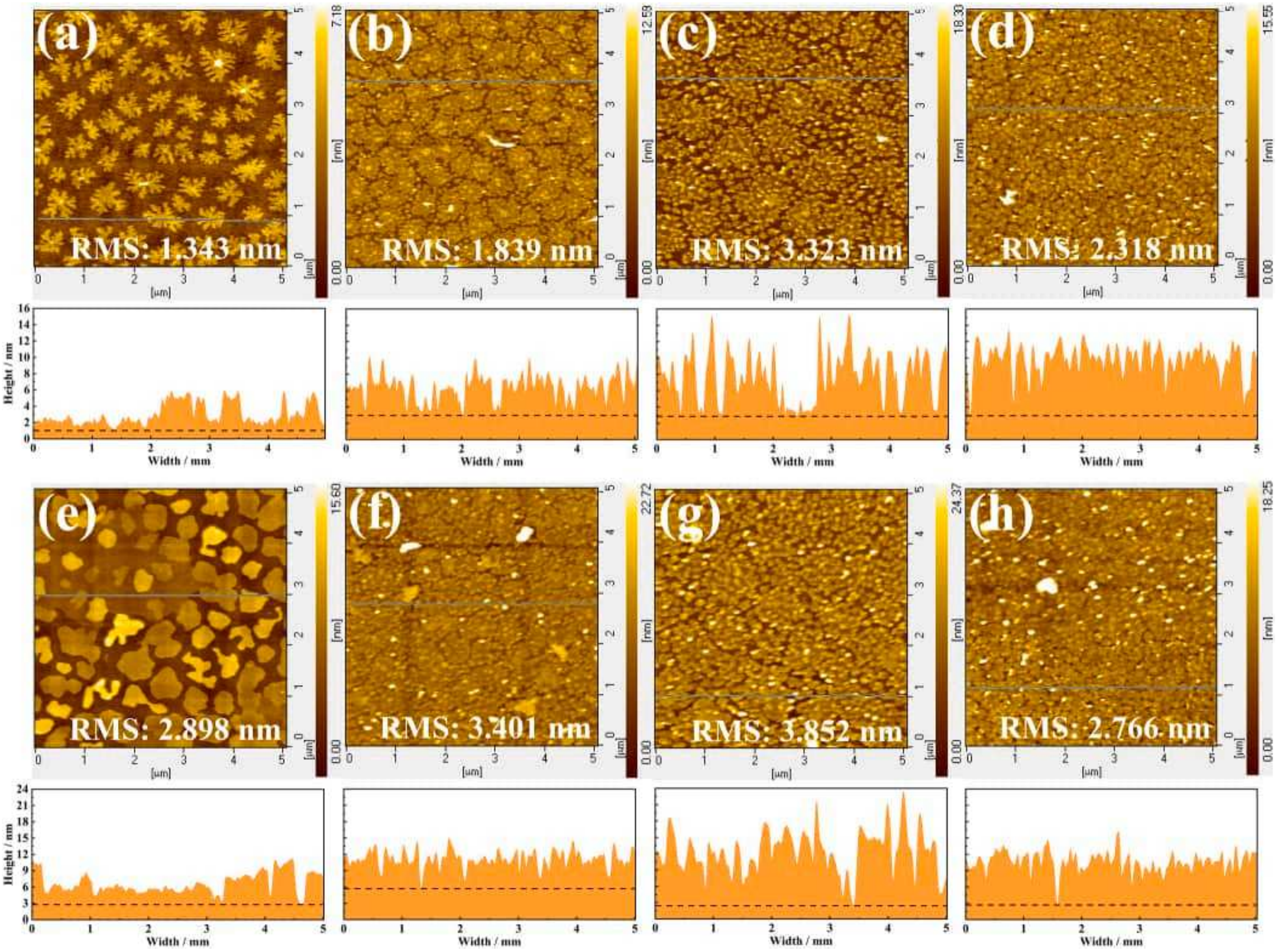

Fig. 2. The AFM images of *p*-6 P and *p*-6 P/VOPc films before and after annealing: (a) *p*-6 P 3 nm. (b) *p*-6 P/VOPc 2 nm. (c) *p*-6 P/VOPc 5 nm. (d) *p*-6 P/VOPc 10 nm. (e-h) are the corresponding morphologies of (a-d) after annealing. The graphs below AFM images corresponds to the cross-sectional morphology at the blue line.

p-6 P fractal islands to compact islands, some adjacent islands further merge to sheet-like films, as shown in Fig. 2e. This is because the annealing temperature provides sufficient activation energy to allow *p*-6 P molecules to freely diffuse and rearrange over the diffusion barrier. Fig. 2f-h exhibit the effects of thermal annealing on VOPc films, which further increases the continuity of VOPc films and reduces the defect between the compact islands. In addition, thermal annealing promotes the fusion of VOPc nanograins, with average size up to 112 nm, as shown in Fig. S2, thus reducing the number of grain boundaries inside the film, to increase the carrier transport efficiency [29–31]. The graphs below AFM images corresponds to the cross-sectional morphology at the blue line. The length of the entire cross-section is about 5 μm. It is obvious that there are dense peaks in the cross-sectional morphologies of Fig. 2b-d, which can be considered as the crystal grains in VOPc films.

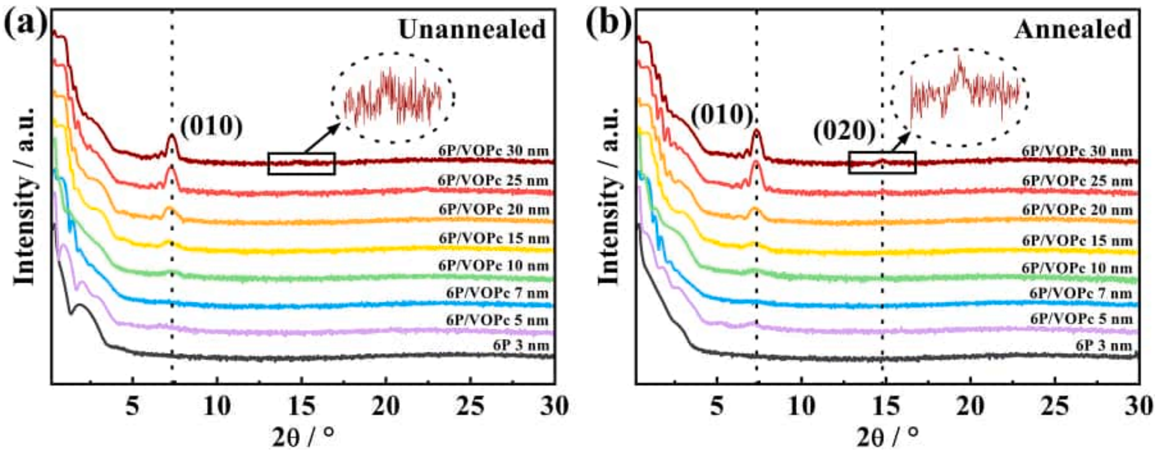

Fig. 3. X-ray diffraction patterns of *p*-6 P/VOPc films with different thickness before and after annealing.

The density and width of peaks reveal the size of grains to some extent, and the gaps between the grains may be conducive to the adsorption and diffusion of gases. After annealing, the density of peaks in the cross-sectional morphologies of Fig. 2f-h decreases and the grain size increases, which is consistent with the results of surface morphology.

In order to study the structure of *p*-6 P/VOPc films, X-ray diffraction (XRD) technique was used to characterize, as shown in Fig. 3. The diffraction pattern of *p*-6 P/VOPc films (10–30 nm) before annealing exhibit only one diffraction peak at 2θ=7.27°, as shown in Fig. 3a. According to the Bragg equation 2dsinθ=λ (λ=0.154056 nm), the interplanar spacing corresponding to the diffraction peak can be calculated as d=1.215 nm, which is similar to that of *p*-6 P/VOPc films deposited on Al_2O_3 dielectric layers [32]. In this case, the diffraction peak at 2θ=7.27° is assigned to the (010) lattice plane. It is suggested that VOPc molecules are dominated by phase II crystalline structure (triclinic, space group $P\bar{1}$, a=12.027 Å, b=12.571 Å, c=8.690 Å, α=96.04°, β=94.80°, γ=68.20°) [33–36]. The appearance of the (010) peak indicates that the VOPc molecules are mainly standing up on the *p*-6 P film, and the π-π stacking direction is parallel to the substrate [32,34, 37]. With the increase of VOPc thickness, the intensity of diffraction peak at 7.27° was significantly enhanced, confirming the improvement of crystallinity in VOPc films. The intensity and position of diffraction peak at 7.27° in Fig. 3b has no obvious change. However, a weak diffraction peak at 2θ=14.8° can be observed in 30 nm VOPc film, corresponding to the interplanar spacing of 0.598 nm, which can be indexed as (020) lattice plane [34,37], indicating the change of VOPc crystal orientation. Therefore, the increase of thickness could not always improve the quality of VOPc films, as evidenced by changes in transistor performance.

3.2. Device performance

The surface morphology and crystal structure of VOPc films provide theoretical basis for the fabrication of high-performance OFET-based sensors. Fig. S3 shows the output and transfer curves of OFET. It is obvious that the increased VOPc thickness and thermal annealing have a positive effect on the performance of transistors. According to the transfer curves at V_{DS}=−50 V, the mobility (μ) and threshold voltage (V_{TH}) of transistors in the saturation region were calculated using the general formulas below:

$$I_{DS} = \frac{\mu C_{ox} W}{2L}(V_G - V_{TH})^2 \quad (1)$$

where I_{DS}, V_G, C_{ox}, W, L denote the source-drain current, gate voltage, capacitance, channel width and length, respectively. The capacitance of SiO_2 was 11.3 nF/cm^2, as shown in Fig. S4. The relevant electrical parameters such as mobility (μ), and threshold voltage (V_{TH}) were extracted in Fig. 4. Compared with thin-film devices (7 nm), 15 times higher mobility and 48% lower threshold voltage is obtained in thick-film device (30 nm) before annealing, which are mainly attributed to the improved crystallinity of VOPc films. Besides, the performance of annealed OFET is further improved, with mobility up to 0.15 cm^{2}V^{-1}s^{-1} (25 nm). The illustration in Fig. 4a depicts the difference in mobility (Δμ) after thermal annealing, which indicates that the improved film morphology also plays an indispensable role. The density of trapped charges (N_t) was estimated using the threshold voltage variation after thermal annealing, as shown in the inset of Fig. 4b. The formula is as follows:

$$N_t = \frac{C_{ox}\Delta V_{TH}}{q} \quad (2)$$

where C_{ox} and q denote the capacitance and unit charge respectively. It implies that the thermal annealing process can quench part of the traps and improve quality of the thin films. The density of quenched traps is about 9.7 × 10^{11} cm^{-2} in 10 nm VOPc films and 8.1 × 10^{10} cm^{-2} in 30 nm VOPc films, respectively. The quenched traps after annealing decreases significantly with the thickness, indicating that thin films have higher trap density than thick films. Additionally, the threshold voltage of thin-film devices after thermal annealing has more improvement, reaching the level of thick-film devices, which provides an idea for the regulation of OFET based on ultra-thin films.

In order to study the multi-parameter response of transistors to humidity, the performance under different humidity is analyzed, as shown in Fig. 5. The output curves under different humidity exhibit typical linear and saturation regions in Fig. 5a, with saturation current (I_{sat}) highly sensitive to humidity, exhibiting a 56.2% decrease at 82% humidity. In transfer curves, the effect of humidity on the off-state current of transistors is not obvious, and changes mainly occur in the sub-threshold region, as shown in Fig. 5b. Meanwhile, the on-off current ratio (I_{on}/I_{off}) of transistors is calculated, to have a 57% decline at 82% humidity. It is apparent in Fig. S5a that the decrease of I_{sat} and I_{on}/I_{off} is highly dependent on humidity, revealing a negative influence of humidity on charge transport properties. Humidity-induced voltaic response can be multifactorial, and carrier mobility can be considered as one of the major factors. Fig. S5b displays the plot of $I_{DS}^{1/2}$ versus gate voltage at different humidity along with the fitting curves in linear regions. The decreased slope of fitting curves indicates a degrade of carrier mobility, and the shifts of threshold voltage can be observed simultaneously, as extracted in Fig. 5c. Compared with the initial mobility, the mobility at 82% humidity is reduced by 38.6%, which indicates the existence of charge interaction between adsorbed water molecules and hole carriers. This can be regarded as trap effect caused by the doping of

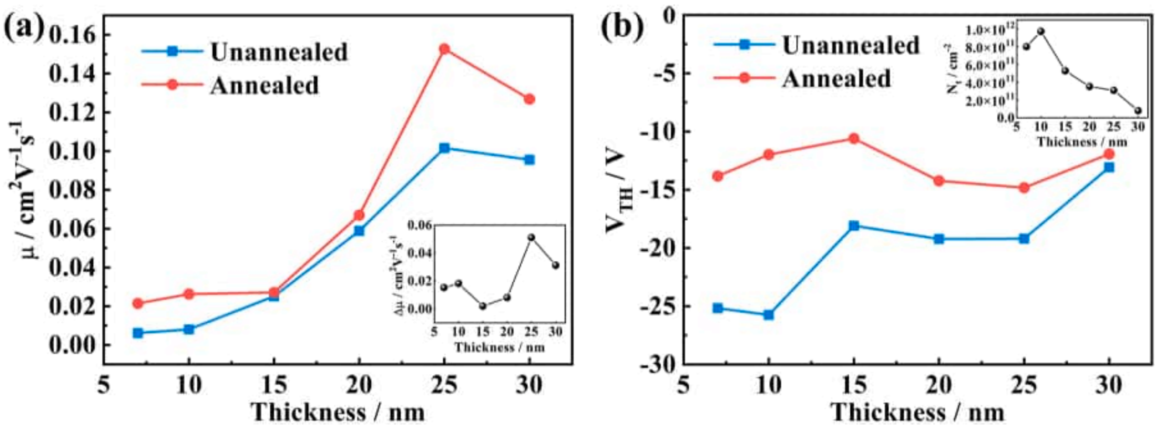

Fig. 4. The electrical parameters of OFET with different VOPc thickness before and after annealing: (a) Mobility (μ) and the variation of mobility difference (Δμ) before and after annealing in illustration; (b) Threshold voltage (V_{TH}) (V_{DS}=−50 V) and the quenched trap density (N_t) after annealing in illustration.

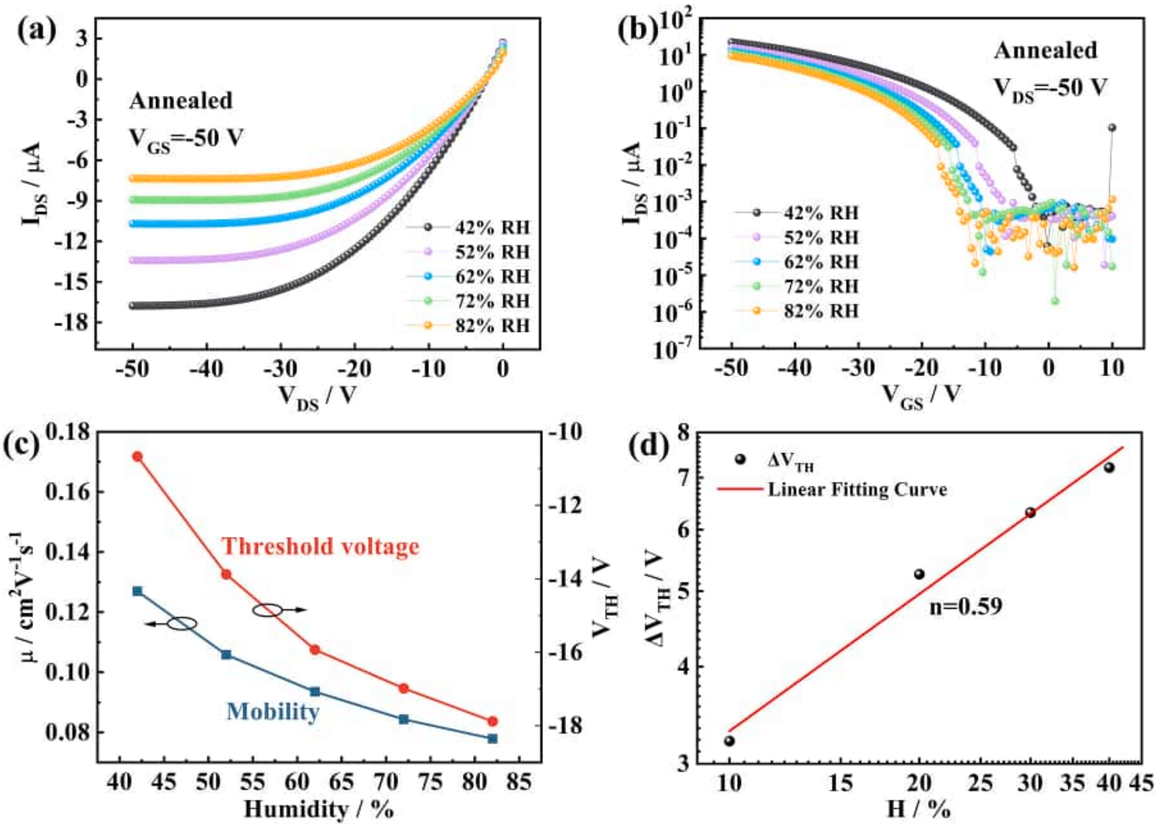

Fig. 5. The electrical properties of OFET with 30 nm VOPc film at different humidity: (a) Output curves (V_{GS}=−50 V). (b) Transfer curves (V_{DS}=−50 V). (c) The variation of mobility (μ) and threshold voltage (V_{TH}). (d) The linear fitting curve of ΔV_{TH} (H = H_A-42%, 42% is the ambient humidity during testing).

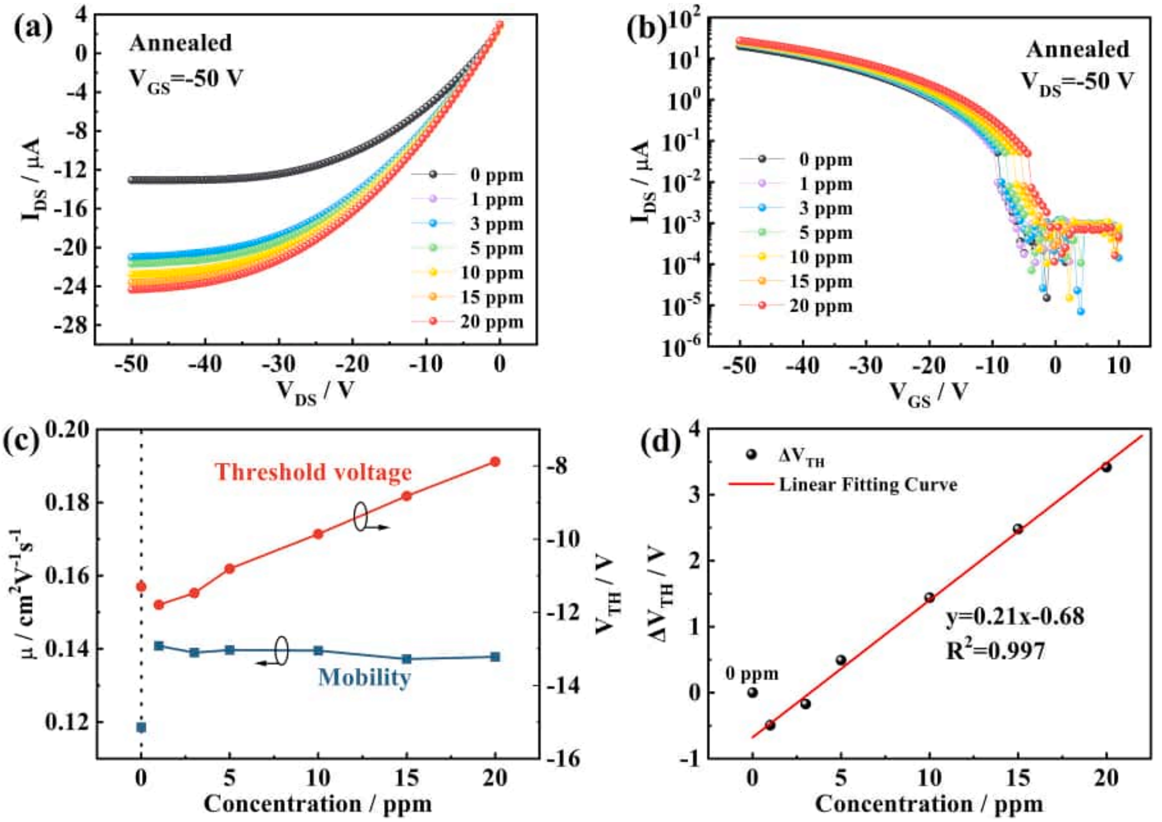

Fig. 6. The electrical properties of OFET with 30 nm VOPc film under different NO_2 gas concentrations: (a) Output curves (V_{GS}=−50 V). (b) Transfer curves (V_{DS}=−50 V). (c) The variation of mobility (μ) and threshold voltage (V_{TH}). (d) The linear fitting curve of ΔV_{TH}.

water molecules. Additionally, the threshold voltage exhibits a large range of variation, as improved by 67.6% at 82% humidity, yielding a well-correlation $\Delta V_{TH} \propto H^{0.59}$ in logarithmic coordinate system, as shown in Fig. 5d. Here, H is defined as the actual measured humidity minus 42% (denote as $H = H_A$-42%). The slope n=0.59 of the straight line can be approximately regarded as the sensitivity of transistor to the humidity range from 42%−82%. The great linearity provides a viable solution for transistor humidity sensors.

Multi-parameter response of transistors to NO_2 gas is opposite to humidity, as shown in Fig. 6. It can be clearly seen in Fig. 6a that the I_{sat} increases significantly upon exposure to NO_2 gas. However, in the process of gas concentration from 1 ppm to 20 ppm, it shows only a 15.7% increase, much lower than that of humidity response. The transfer performance in Fig. 6b also exhibits an obvious change in subthreshold region. Under high gas concentrations, the driving voltage of transistors operating in the subthreshold region is further decreased, which provides an idea towards the low-consumption gas sensors. The I_{on}/I_{off} of transistors under 20 ppm NO_2 gas own a 57.1% increase than 1 ppm, which is shown together with I_{sat} in Fig. S6. Notably, the mobility in different gas concentrations has no obvious change, while the significant increase of mobility between 0 and 1 ppm is most likely to be caused by environmental factors, such as the adsorption of water molecules. The threshold voltage exhibits a decrease of 33.2% as gas concentrations increase from 1 ppm to 20 ppm, as shown in Fig. 6c. The results imply that NO_2 doping only change the carrier concentration in the film, instead of contributing to the carrier transport properties. Moreover, the ΔV_{TH} exhibit an excellent linear relationship with NO_2 gas concentration, as shown in Fig. 6d, with sensitivity up to 0.21 V·ppm^{-1}. As

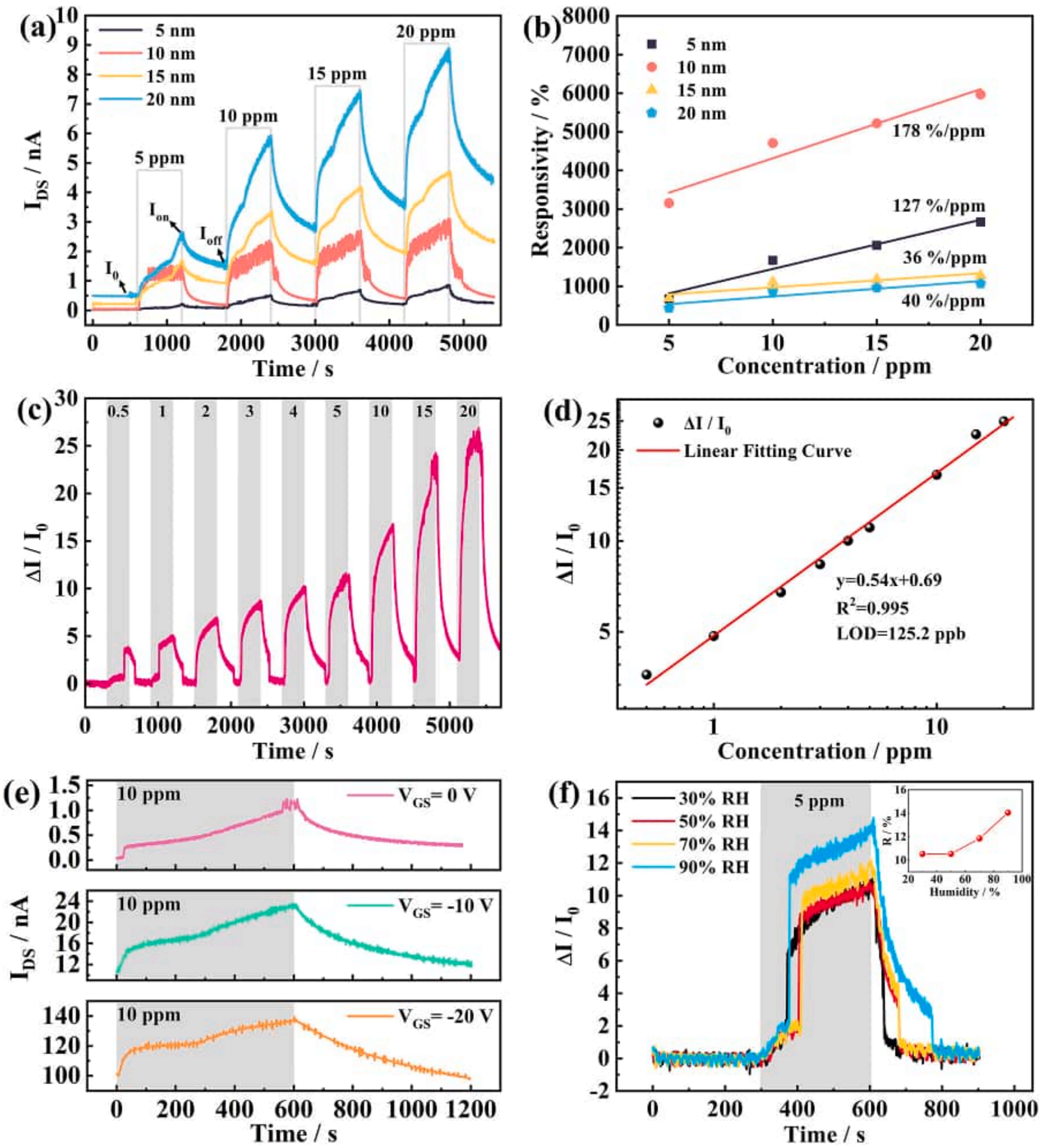

Fig. 7. The detection properties of OFET sensors: (a) The dynamic response curves of sensors with different VOPc thicknesses to 5–20 ppm NO_2 gas. (b) The corresponding responsivity extracted from (a). (c) The relative response of 10 nm VOPc sensors to 0.5–20 ppm NO_2 gas. (d) The linear fitting curve to relative response in (c). (e) The dynamic response curves of 10 nm sensors to 10 ppm NO_2 gas at different gate voltages. (f) The relative response of 10 nm sensors to 5 ppm NO_2 gas at different humidity. The inset shows the responsivity at different humidity.

mentioned above, the effectiveness of transistor multi-parameter variation in the detection of humidity or NO_2 gas is successfully demonstrated.

To further develop high-performance gas sensors, transistors based on interdigitated electrodes were prepared, and the sensing performance was systematically studied. The dynamic response to 5–20 ppm NO_2 gas is shown in Fig. 7a, and the gas pulse were set to 10 min. It is obvious that the baseline and response current increase significantly with VOPc thickness, which is consistent with the analysis of OFET performance. However, the responsivity of gas sensor is not the case. Here, the responsivity is defined as $(I_{on}\text{-}I_{off})/I_0$, where I_{on}, I_{off}, I_0 represent the saturation current, recovering current, and the baseline current, respectively. Among them, the responsivity of 10 nm device to 10 ppm NO_2 gas is up to 4710%, which is four times higher than that of 20 nm device, as shown in Fig. 7b. In addition, with the increase of VOPc thickness, the baseline drift of sensors is more serious, the recovery rate of 10 nm sensor is 85%, while the 30 nm sensor only reaches 53%. This can be attributed to that the number of grain boundaries in VOPc films increases with the thickness, which can promote the adsorption of NO_2 molecules, and the recovery is relatively slower. Moreover, the diffusion distance of NO_2 molecules in VOPc film increases, and the deeply trapped NO_2 molecules may undergo the process of repeated charge exchange during recovery. Similar phenomenon has also occurred even in ultrathin films [38]. Response and recovery time is also an important performance indicator of gas sensors. In this work, the response time (T_{res}) is expressed as the time required for the sensor to reach 50% of the maximum response, and the recovery time (T_{rec}) is expressed as the time required for the sensor to recover to 50%. The response/recovery time increases with VOPc thickness, as shown in Fig. S7, 10 nm device simultaneously obtained a short response/recovery time, while 5 nm devices have the longest response time due to its poor film quality with low carrier transport efficiency. With the increase of gas concentrations, the response/recovery time of different devices decreased significantly. It is apparent from the above results that the performance of sensors with thick-films decreases significantly, which can be attributed to the difficulties in gas diffusion, thus reducing the charge transfer between NO_2 molecules and sensing films. Low-concentration gas detection is an indispensable capability for gas sensors. The relative response $((I\text{-}I_0)/I_0)$ of 10 nm device to 0.5–20 ppm NO_2 gas was studied, and the gas pulse time was set to 5 min, as shown in Fig. 7c. The relative response to 0.5 ppm NO_2 gas reaches 359%, with almost no baseline drift. The linear fitting curve in Fig. 7d is usually used to evaluate the sensitivity and limit of detection (LOD) for gas sensors, the LOD is defined as the ratio of 3 times the standard deviation to the slope. According to the linear fitting results, the theoretical detection limit of 125.2 ppb and sensitivity of 0.54 ppm^{-1} were obtained. Besides, the effect of gate electrical field on the dynamic response of 10 nm devices was investigated, as shown in Fig. 7e. As the gate voltage reach −20 V, the response current increased by two orders of magnitude. Thus, the amplification effect of transistor on sensing signal is successfully realized. Humidity is an important factor affecting the operation of gas sensors at room temperature. There is a great variety of ambient humidity in different regions and weathers [39], calling for higher requirements on the repeatability between humidity and gas dual detection. The sensing performance of 10 nm devices after being stored at different humidity was studied, as shown in Fig. 7 f. The inset shows the responsivity at different humidity, which remains stable at 30%−50% humidity, and exhibits a slight increase when the humidity ranges from 50%−90%. As mentioned above, the higher the humidity, the more severe the equivalent trap effect caused by water molecules. The introduce of NO_2 molecules promotes the desorption of water molecules and release part of the captured hole carriers. The higher the humidity, the more hole carriers are released, resulting in the increased response current. The similar phenomenon has also been observed in CuPc gas sensor in previous study [40]. Overall, the sensors exhibit considerable repeatability at high humidity, which simultaneously proves the reliability of the humidity sensing strategy. The repeatability and long-term stability of devices were studied in Fig. S8-S9. In Fig. S8 a-b, sensors based on *p*-6 P/VOPc 10 nm films have similar response currents and baseline drifts under 5–20 ppm NO_2 gas. The output (c-d) and transfer (e-f) performance of transistors based on *p*-6 P/VOPc 30 nm under different voltages have little change. The performance of the same transistor (*p*-6 P/VOPc 30 nm) after being stored for 63 days in the atmosphere was studied, and neither output nor transfer performance was significantly degraded, as shown in Fig. S9. Repeatable and stable transistor performance underpins the multi-parameter responses to gas and humidity. It can be concluded that the prepared devices have certain repeatability and long-term stability.

The selectivity of gas sensors is crucial for practical applications. Herein, the responses of VOPc and other phthalocyanine materials to different gases were compared, as shown in Table S1 Ref. [2–9,13], and higher selectivity of the sensor to NO_2 is concluded. The response of VOPc sensor to 50 ppm NH_3 and 500 ppm H_2 are about 0.03 and 0.05, respectively, while the response to 10 ppm NO_2 in this work reached 47.1. Compared with NO_2 gas, the response to H_2 and NH_3 is basically negligible, and VOPc has barely response in CO_2 gas, ethanol and acetone vapor [41]. Higher response to NO_2 gas has also been observed in other phthalocyanine materials in Table S1 Ref. [2–9]. In general, the ability of gases in donating or capturing electrons determines the selectivity of sensors to a certain extent [42]. As a strong oxidizing gas, NO_2 has a higher electron affinity and is more likely to interact with VOPc molecules, thereby making it more selective to NO_2 gas. In addition, the enhanced response to NO_2 gas at high humidity (Fig. 7f) demonstrated that VOPc is more prone to react with NO_2 than H_2O molecules. Therefore, it can be inferred that the sensor also has a certain selectivity to NO_2 gas at high humidity compared to the above gases. It is worth mentioned that other oxidizing gases such as Cl_2, O_3 may interfere with the detection results, and corresponding sensor arrays can be utilized to further improve the resolution of target gas [43,44].

To further demonstrate the great performance of the sensor, the basic parameters of common metal phthalocyanine-based devices were summarized, as shown in Table S1. it can be seen that the as-fabricated OFET sensors in this work exhibit certain competitiveness in responsivity, sensitivity, and the detection range of NO_2 or humidity.

3.3. *Humidity and gas sensing mechanism*

Current and voltage as the sensing mechanism are mainly caused by the charge transfer during redox reactions. The sensing behavior is strongly correlated with the morphology and intrinsic properties of VOPc films. The grain boundaries and defects in VOPc polycrystalline films provide convenience for the physical adsorption and diffusion of H_2O and NO_2 molecules [45–48], as evidenced in Fig S10, while the delocalized π-electron system with low ionization energy and high polarization energy in VOPc molecules plays an indispensable role in the chemical adsorption. Different sensing mechanisms of humidity and gas are illustrated in Fig. 8. For the initial device state in Fig. 8a, there are a large number of hole traps inside *p*-6 P/VOPc film. Without considering the electron barrier in Fig. 8b, the hole carriers induced by gate electric field (V_G) fill the traps preferentially, as V_G equals the initial threshold voltage ($V_{TH\text{-}I}$), all the traps are filled with hole carriers. Once exposed to moisture in Fig. 8c, polar water molecules can affect the charge transport in organic materials through charge-dipole interaction. On the one hand, the proton end of the water dipole is attracted by the delocalized π bond in VOPc molecule to form a π hydrogen bond, while the other end with strong electronegativity forms hole traps. On the other hand, the dipole polarization under the electric field makes the system energy disorder. The above results will not only lead to the decrease of hole carriers induced by gate electric field, but also increase the energy barrier for carrier transition, thus affecting the carrier mobility. Similar mechanisms have also been reported in other transistor sensors [49–51]. Furthermore, more hole traps undoubtedly require a larger gate voltage ($V_{TH\text{-}H}$) to induce hole carriers to fill. During gas detection in Fig. 8d,

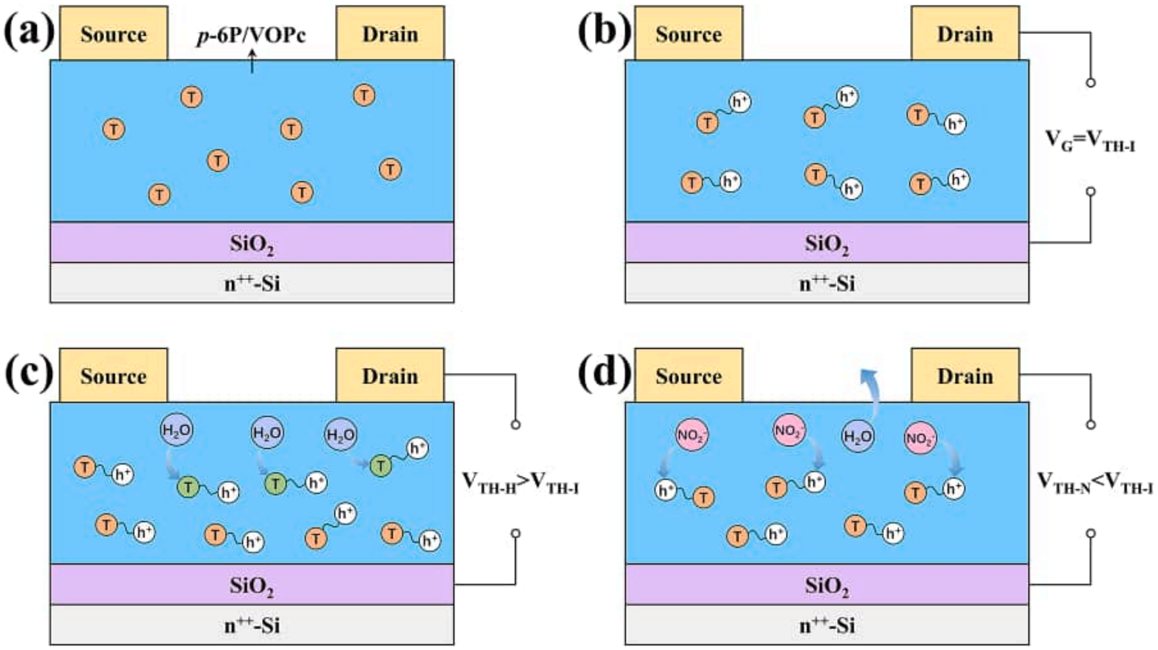

Fig. 8. Schematic diagrams of humidity and NO_2 detection mechanism, related to the threshold voltage changes of transistors: (a) A large number of hole traps (orange) inside the film; (b) When the gate voltage (V_G) equals the initial threshold voltage (V_{TH-I}), the traps inside the film are all filled with hole carriers; (c) Water molecules induce more traps (green) to be filled by hole carriers, to increase the threshold voltage (V_{TH-H}); (d) NO_2 molecules induce more hole carriers to fill the traps, to decrease the threshold voltage (V_{TH-N}). NO_2 gas can also promote the desorption of water molecules. T denotes hole traps, and the arrows denote the adsorption and desorption process of analytes.

however, NO_2 molecules with strong oxidizing can easily capture electrons from VOPc molecules. Thus, the concentration of hole carriers is significantly enhanced, resulting in an increase of electrical conductivity, which can be regarded as p-type doping of the material. The reaction equation is as follows:

$$NO_2\ (gas) + e^- \rightleftharpoons NO_2^-\ (ad) \tag{3}$$

In addition, NO_2 molecules have no negative effect on carrier transport property, which is evidenced by the constant mobility from 1 to 20 ppm in Fig. 6c. Therefore, the number of gate voltage-induced (V_{TH-N}) hole carriers required to fill the traps is greatly reduced. As H_2O molecules and NO_2 gas coexist, it is worth noting that the introduce of NO_2 gas will promote the desorption of water molecules and reduce the hole traps, which is manifested in the enhanced mobility and current response in Fig. 6c (0–1 ppm) and Fig. 7f. Moreover, NO_2 gas plays a major role in the current response while the difference caused by humidity is manifested in the increased current response at high humidity. This further proves the reliability of sensors to detect NO_2 gas at different humidity.

4. Conclusion

In summary, we have demonstrated the feasibility of high-performance transistors based on thickness-optimization and thermal-annealing for humidity and gas detection. The multi-parameter of transistors exhibits a high dependence on humidity, with threshold voltage variation linearly related to humidity ranging from 42% to 82% RH, resulting in a sensitivity of 0.59 V/%RH. In addition, sensors with 10 nm VOPc films also realized a high response to 0.5–20 ppm NO_2 gas, with minor baseline drift and short response/recovery time. The relative response exhibited excellent linearity with gas concentrations, resulting in a theoretical LOD of 125.2 ppb and the sensitivity of 0.54 ppm^{-1}. The sensing behavior was attributed to different interaction mechanisms between charge carrier and H_2O/NO_2 molecules. The result also indicated that OFET sensors own high repeatability between humidity and NO_2 gas. We believe that this study has great potential to realize the practical application of low cost and multifunctional phthalocyanine-based sensors.

CRediT authorship contribution statement

Yangyang Zhu: Conceptualization, Methodology, Software, Writing – original draft, Writing – review & editing. **Yiqun Zhang:** Data curation, Investigation. **Jiajia Yu:** Visualization, Formal analysis. **Chengren Zhou:** Software. **Chaojie Yang:** Methodology, Data curation. **Lu Wang:** Software, Validation. **Li Wang:** Validation. **Libo Ma:** Supervision. **Li Juan Wang:** Supervision, Validation, Writing – review & editing.

Declaration of Competing Interest

The authors declare that they have no known competing financial interests or personal relationships that could have appeared to influence the work reported in this paper.

Data Availability

Data will be made available on request.

Acknowledgments

The authors sincerely acknowledge the financial support from Department of Science and Technology of Jilin Province (20200403146SF). L. J. W. acknowledges the support and funding from China Scholarship Council (CSC). The authors thank the technical support in characterization of organic films and AFM images from Prof. Jidong Zhang at the State Key Laboratory of Polymer Physics and Chemistry, Changchun Institute of Applied Chemistry.

Appendix A. Supporting information

Supplementary data associated with this article can be found in the online version at doi:10.1016/j.snb.2022.132815.

References

[1] H. Li, W. Shi, J. Song, H.J. Jang, J. Dailey, J. Yu, H.E. Katz, Chemical and biomolecule sensing with organic field-effect transistors, Chem. Rev. 119 (2019) 3–35.
[2] V.V. Tran, G. Jeong, K.S. Kim, J. Kim, H.R. Jung, B. Park, J.J. Park, M. Chang, Facile strategy for modulating the nanoporous structure of ultrathin π-conjugated polymer films for high-performance gas sensors, ACS Sens. 7 (2022) 175–185.
[3] M.Y. Lee, H.J. Kim, G.Y. Jung, A.R. Han, S.K. Kwak, B.J. Kim, J.H. Oh, Highly sensitive and selective liquid-phase sensors based on a solvent-resistant organic-transistor platform, Adv. Mater. 27 (2015) 1540–1546.
[4] G. Schwartz, B.C. Tee, J. Mei, A.L. Appleton, D.H. Kim, H. Wang, Z. Bao, Flexible polymer transistors with high pressure sensitivity for application in electronic skin and health monitoring, Nat. Commun. 4 (2013) 1859.
[5] A.N. Sokolov, B.C. Tee, C.J. Bettinger, J.B. Tok, Z. Bao, Chemical and engineering approaches to enable organic field-effect transistors for electronic skin applications, Acc. Chem. Res. 45 (2012) 361–371.
[6] S. Yuvaraja, S.G. Surya, V. Chernikova, M.T. Vijjapu, O. Shekhah, P.M. Bhatt, S. Chandra, M. Eddaoudi, K.N. Salama, Realization of an ultrasensitive and highly selective OFET NO_2 sensor: the synergistic combination of PDVT-10 polymer and porphyrin-MOF, ACS Appl. Mater. Interfaces 12 (2020) 18748–18760.
[7] A. Daus, S. Vaziri, V. Chen, Ç. Köroğlu, R.W. Grady, C.S. Bailey, H.R. Lee, K. Schauble, K. Brenner, E. Pop, High-performance flexible nanoscale transistors based on transition metal dichalcogenides, Nat. Electron. 4 (2021) 495–501.
[8] J. Liu, L. Jiang, J. Shi, C. Li, Y. Shi, J. Tan, H. Li, H. Jiang, Y. Hu, X. Liu, J. Yu, Z. Wei, L. Jiang, W. Hu, Relieving the photosensitivity of organic field-effect transistors, Adv. Mater. 32 (2020), 1906122.
[9] D. McColl, B. Cartlidge, P. Connolly, Real-time monitoring of moisture levels in wound dressings in vitro: an experimental study, Int. J. Surg. 5 (2007) 316–322.
[10] S.D. Milne, I. Seoudi, H. Al Hamad, T.K. Talal, A.A. Anoop, N. Allahverdi, Z. Zakaria, R. Menzies, P. Connolly, A wearable wound moisture sensor as an indicator for wound dressing change: an observational study of wound moisture and status, Int. Wound J. 13 (2016) 1309–1314.
[11] T.Q. Trung, L.T. Duy, S. Ramasundaram, N.-E. Lee, Transparent, stretchable, and rapid-response humidity sensor for body-attachable wearable electronics, Nano Res. 10 (2017) 2021–2033.
[12] W. Jeong, J. Song, J. Bae, K.R. Nandanapalli, S. Lee, Breathable nanomesh humidity sensor for real-time skin humidity monitoring, ACS Appl. Mater. Interfaces 11 (2019) 44758–44763.
[13] X. Zhuang, S. Han, B. Huai, W. Shi, Y. Junsheng, Sub-ppm and high response organic thin-film transistor NO_2 sensor based on nanofibrillar structured TIPS-pentacene, Sens. Actuators B 279 (2019) 238–244.
[14] S. Han, Z. Yang, Z. Li, X. Zhuang, D. Akinwande, J. Yu, Improved room temperature NO_2 sensing performance of organic field-effect transistor by directly blending a hole-transporting/electron-blocking polymer into the cctive layer, ACS Appl. Mater. Interfaces 10 (2018) 38280–38286.
[15] S. Yuvaraja, V.N. Bhyranalyar, S.A. Bhat, S.G. Surya, C.V. Yelamaggad, K. N. Salama, A highly selective electron affinity facilitated H_2S sensor: the marriage of tris(keto-hydrazone) and an organic field-effect transistor, Mater. Horiz. 8 (2021) 525–537.
[16] C.-F. Lu, C.-W. Shih, C.-A. Chen, A. Chin, W.-F. Su, Tuning the morphology of isoindigo donor-acceptor polymer film for high sensitivity ammonia sensor, Adv. Funct. Mater. 28 (2018), 1803145.
[17] P. Lienerth, S. Fall, P. Lévêque, U. Soysal, T. Heiser, Improving the selectivity to polar vapors of OFET-based sensors by using the transfer characteristics hysteresis response, Sens. Actuators B 225 (2016) 90–95.
[18] M.-J. Yin, Z.-R. Li, T.-R. Lv, K.-T. Yong, Q.-F. An, Low-voltage driven flexible organic thin-film transistor humidity sensors, Sens. Actuators B 339 (2021), 129887.
[19] S. Wu, G. Wang, Z. Xue, F. Ge, G. Zhang, H. Lu, L. Qiu, Organic field-effect transistors with macroporous semiconductor films as high-performance humidity sensors, ACS Appl. Mater. Interfaces 9 (2017) 14974–14982.
[20] T. Xie, G. Xie, H. Du, Y. Su, Z. Ye, Y. Chen, Y. Jiang, Two novel methods for evaluating the performance of OTFT gas sensors, Sens. Actuators B 230 (2016) 176–183.
[21] Z. Yang, S. Han, Y. Liu, X. Zhuang, D. Akinwande, J. Yu, Investigation of the atmosphere influence on device characteristics and NO_2 sensing performance of organic field-effect transistors consisting of polymer bulk heterojunction, Org. Electron. 62 (2018) 114–120.
[22] X. Zhou, Z. Wang, R. Song, Y. Zhang, L. Zhu, D. Xue, L. Huang, L. Chi, High performance gas sensors with dual response based on organic ambipolar transistors, J. Mater. Chem. C. 9 (2021) 1584–1592.
[23] H. Li, Y. Shi, G. Han, J. Liu, J. Zhang, C. Li, J. Liu, Y. Yi, T. Li, X. Gao, C. Di, J. Huang, Y. Che, D. Wang, W. Hu, Y. Liu, L. Jiang, Monolayer two-dimensional molecular crystals for an ultrasensitive OFET-based chemical sensor, Angew. Chem. Int. Ed. Engl. 59 (2020) 4380–4384.
[24] T. Sizun, M. Bouvet, Y. Chen, J.-M. Suisse, G. Barochi, J. Rossignol, Differential study of substituted and unsubstituted cobalt phthalocyanines for gas sensor applications, Sens. Actuators B 159 (2011) 163–170.
[25] J. Yang, T. Wang, H. Wang, F. Zhu, G. Li, D. Yan, Ultrathin-film growth of *para*-sexiphenyl (II): formation of large-size domain and continuous thin film, J. Phys. Chem. B 112 (2008) 7821–7825.
[26] J. Dong, Q. Sun, G. Li, H. Su, L. Wang, Y. Zhu, L. Wang, Evaporation process of heterogeneous induced zinc phthalocyanine for organic thin film transistor, Chin. J. Lumin. 42 (2021) 700–707.
[27] H. Wang, F. Zhu, J. Yang, Y. Geng, D. Yan, Weak epitaxy growth affording high-mobility thin films of disk-like organic semiconductors, Adv. Mater. 19 (2007) 2168–2171.
[28] S. Zhang, Y. Zhao, X. Du, Y. Chu, S. Zhang, J. Huang, Gas sensors based on nano/microstructured organic field-effect transistors, Small 15 (2019), 1805196.
[29] V. Rani, A. Sharma, P. Kumar, B. Singh, S. Ghosh, Charge transport mechanism in copper phthalocyanine thin films with and without traps, RSC Adv. 7 (2017) 54911–54919.
[30] D.H. Kwak, Y. Seo, J.E. Anthony, S. Kim, J. Hur, H. Chae, H.J. Park, B. Kim, E. Lee, S. Ko, W.H. Lee, Enhanced gas sensing performance of organic field-effect transistors by modulating the dimensions of triethylsilylethynyl-anthradithiophene microcrystal arrays, Adv. Mater. Interfaces 7 (2020), 1901696.
[31] S. Dong, H. Tian, L. Huang, J. Zhang, D. Yan, Y. Geng, F. Wang, Non-peripheral tetrahexyl-substituted vanadyl phthalocyanines with intermolecular cofacial π-π stacking for solution-processed organic field-effect transistors, Adv. Mater. 23 (2011) 2850–2854.
[32] H. Wang, D. Song, J. Yang, B. Yu, Y. Geng, D. Yan, High mobility vanadyl-phthalocyanine polycrystalline films for organic field-effect transistors, Appl. Phys. Lett. 90 (2007), 253510.
[33] T.V. Basova, V.G. Kiselev, I.S. Dubkov, F. Latteyer, S.A. Gromilov, H. Peisert, T. Chassè, Optical spectroscopy and XRD study of molecular orientation, polymorphism, and phase transitions in fluorinated vanadyl phthalocyanine thin films, J. Phys. Chem. C. 117 (2013) 7097–7106.
[34] Y. Pan, X. Liao, Y. Wu, L. Chen, Y. Zhao, Y. Shen, F. Li, S. Shen, D. Huang, Steady-state photovoltaic and electroreflective spectra in Al/vanadyl phthalocyanine (VOPc, in phase II)/rindium-tin-oxide (ITO) sandwich cell, Thin Solid Films 324 (1998) 209–213.
[35] R.F. Ziolo, C.H. Griffiths, J.M. Troup, Cryatal structure of vanadyl phthalocyanine, phase II, J. Chem. Soc. Dalton Trans. 11 (1980) 2300–2302.
[36] C.H. Griffiths, M.S. Walker, P. Goldstein, Polymorphism in Vanadyl Phthalocyanine, Mol. Cryst. Liq. Cryst. 33 (1976) 149–170.
[37] H. Ohta, T. Kambayashi, K. Nomura, M. Hirano, K. Ishikawa, H. Takezoe, H. Hosono, Transparent organic thin-film transistor with a laterally grown non-planar phthalocyanine channel, Adv. Mater. 16 (2004) 312–316.
[38] X. Wang, S. Ji, H. Wang, D. Yan, Room temperature nitrogen dioxide chemresistor using ultrathin vanadyl-phthalocyanine film as active layer, Sens. Actuators B 160 (2011) 115–120.
[39] Z. Gao, G. Song, X. Zhang, Q. Li, S. Yang, T. Wang, Y. Li, L. Zhang, L. Guo, Y. Fu, A facile PDMS coating approach to room-temperature gas sensors with high humidity resistance and long-term stability, Sens. Actuators B 325 (2020), 128810.
[40] L. Wang, L. Wang, G. Li, Y. Zhu, C. Liu, L. Zeng, S. Zhong, L.J. Wang, Three-dimensional CuPc films decorated with well-ordered PVA parallel nanofiber arrays for low concentration detecting NO_2 sensor, Sens. Actuators B 337 (2021), 129781.
[41] D. Klyamer, A. Sukhikh, N. Nikolaeva, N. Morozova, T. Basova, Vanadyl phthalocyanine films and their hybrid structures with Pd nanoparticles: structure and sensing properties, Sensors 20 (2020) 1893.
[42] W.C. Trogler, Chemical sensing with semiconducting metal phthalocyanines, Struct. Bond 142 (2012) 91–118.
[43] Y. Sivalingam, G. Magna, R. Kalidoss, S. Murugan, D. Chidambaram, V. Nutalapati, S.V. Jayaraman, R. Paolesse, C.D. Natale, Combinatorial selectivity with an array of phthalocyanines functionalized TiO_2/ZnO heterojunction thin film sensors, Nanotechnology 33 (2022), 075503.
[44] L. Song, L. Yang, Z. Wang, D. Liu, L. Luo, X. Zhu, Y. Xi, Z. Yang, N. Han, F. Wang, Y. Chen, One-step electrospun SnO_2/MO_x heterostructured nanomaterials for highly selective gas sensor array integration, Sens. Actuators B 283 (2019) 793–801.
[45] T. Someya, H.E. Katz, A. Gelperin, A.J. Lovinger, A. Dodabalapur, Vapor sensing with α, ω-dihexylquarterthiophene field-effect transistors: The role of grain boundaries, Appl. Phys. Lett. 81 (2002) 3079–3081.
[46] L. Wang, S. Chen, W. Li, K. Wang, Z. Lou, G. Shen, Grain-boundary-induced drastic sensing performance enhancement of polycrystalline-microwire printed gas sensors, Adv. Mater. 31 (2019), 1804583.
[47] P. Yasaei, B. Kumar, R. Hantehzadeh, M. Kayyalha, A. Baskin, N. Repnin, C. Wang, R.F. Klie, Y.P. Chen, P. Král, A. Salehi-Khojin, Chemical sensing with switchable transport channels in graphene grain boundaries, Nat. Commun. 5 (2014) 4911.
[48] A. Hankins, T.C. Willard, A.Y. Liu, M. Paranjape, Role of defects in the sensing mechanism of CNTFET gas sensors, J. Appl. Phys. 128 (2020), 084501.
[49] Z. Zhu, J.T. Mason, R. Dieckmann, G.G. Malliaras, Humidity sensors based on pentacene thin-film transistors, Appl. Phys. Lett. 81 (2002) 4643–4645.
[50] D.H. Dunlap, P.E. Parris, V.M. Kenkre, Charge-dipole model for the universal field dependence of mobilities in molecularly doped polymers, Phys. Rev. Lett. 77 (1996) 3–545.
[51] D. Li, E.-J. Borkent, R. Nortrup, H. Moon, H. Katz, Z. Bao, Humidity effect on electrical performance of organic thin-film transistors, Appl. Phys. Lett. 86 (2005), 042105.

Yangyang Zhu received the B. E. degree in 2018. He is currently pursuing the Ph. D. degree at Changchun University of Technology. His main research interests focus on organic field effect transistors and gas sensors.

Yiqun Zhang received his B. E. degree in 2021. He is a graduate student at Changchun University of Technology. His main research interests focus on organic/metal interface and flexible electronics.

Jiajia Yu is an undergraduate student at Changchun University of Technology. Her research interests focus on the preparation and characterization of organic semiconductor thin films.

Chengren Zhou is an undergraduate student at Changchun University of Technology. His research interests focus on nanocomposites and solution processed organic thin films.

Chaojie Yang is an undergraduate student at Changchun University of Technology. His main research interests include organic phototransistors.

Lu Wang is currently a lecturer at Changchun University of Technology. She received her Ph.D. degree from Changchun University of Technology in 2020. Her research interests include nanomaterials and gas sensors.

Li Wang is currently a lecturer at Changchun University of Technology. Her research interests include organic semiconductor device physics.

Libo Ma is a research group leader in the Institute of Integrative Nanosciences at Leibniz IFW Dresden. He obtained his Ph.D. degree from the Institute of Physics, Chinese Academy of Sciences in 2007. His current research interests include cavity photonics, on-chip photonic integration, and topological photonics.

Li Juan Wang is an associate professor at Changchun University of Technology. She received her Ph. D. degree from Changchun University of Science and Technology in 2008. Her current research interests focus on organic phototransistors, organic solar cells, sensors, organic light-emitting diode and flexible electronics.

Current Applied Physics 19 (2019) 82–88

Contents lists available at ScienceDirect

Current Applied Physics

journal homepage: www.elsevier.com/locate/cap

The enhancement of humidity sensing performance based on Eu-doped ZnO

Shuguo Yu, Hongyan Zhang*, Cunchong Lin, Mingjin Bian

School of Physical Science and Technology, Xinjiang University, Urumqi, 830046, PR China

A R T I C L E I N F O

Keywords:
Sol-gel
Zinc oxide
Eu dopant
Humidity sensors

A B S T R A C T

In this work, a high performance impedance-type humidity sensor based on Europium-doped ZnO with abundant surface oxygen vacancy defects was synthesized by sol-gel method. Response of the Eu-doped ZnO with different molar ratio were investigated by exposing them to humidity environments in wide range of 11–95% RH at room temperature. The Eu-doped ZnO (2 mol%) exhibits a three orders impedance change, along with short response/recovery time (5 s/19 s), low hysteresis and best linearity. Complex impedance spectra indicates that dopant Eu can enhance humidity sensing performance of ZnO, which is resulted from the introduction of Eu^{3+} ions into ZnO structure to produce more defects of surface oxygen vacancy and more active sites on the surface of ZnO. The results show that this is a feasible method to achieve high humidity sensing performance by Eu doped ZnO, which make it a promising candidate for humidity sensing materials and broaden the use of ZnO materials.

1. Introduction

It is well established that metal oxide semiconductors have attracted considerable attentions for fabricating versatile photo electronic devices, detectors and sensors etc. Zinc oxide (ZnO) is an n-type II-VI metal oxide semiconductor with excellent photoelectric characteristics, surface adsorption activity and favorable morphology, which ensures it as attractive candidates for a wide range of application in environmental protection, photodetection, sensing device and other fields [1–5]. Among these applications, nanostructured ZnO based humidity sensors are widely used in increasing demand for daily life, such as agriculture, food storage, industrial process, weather forecast and environment protection et al. [6–11]. Modern humidity sensor most commonly measures the change in electric resistance due to the adsorption/desorption of water molecules in different relative humidity environments [12,13]. It is well known that the mechanism of humidity sensor based on ZnO is important to enhance the humidity sensing performance. For resistance humidity sensors, the most known mechanism for metal oxide semiconductor based humidity sensors is that there exist oxygen vacancy defects on the surface and highly specific surface area [14–16]. In general, O_2 is usually absorbed on the surface of ZnO through physical adsorption, and then it gets negative electrons from ZnO to generate O_2^- adsorbed on ZnO. Oxygen vacancy defects on ZnO surface will facilitate water molecules and subsequently dissociate adsorption of oxygen [15,16]. In another words, water molecules are preferred to be adsorbed on the oxygen vacancy defects, which means more surface oxygen vacancy defects enable more water molecules to be absorbed on the surface of ZnO. Moreover, surface oxygen vacancy can provide high charge density and strongly electrostatic field for the dissociation of the adsorbed water molecules, which is beneficial for proton hopping transportation for the case of lower relative humidity (RH). Furthermore, more adsorption sites are produced by large specific surface area of nanostructured ZnO, which is usually concerned to achieve high sensitivity in the higher RH [17–19].

In recently years, many studies have been achieved to get high effective humidity sensing performance by controlling the structure and the shape of ZnO by doping elements [20–23]. Compared to other dopants, the rare earth (RE) such as europium (Eu) [24], cerium (Ce) [25], erbium (Er) [26] and dysprosium (Dy) [24,27] can be easily used to tune the magnetic, the electrical and the optical properties of ZnO resulted from the RE ions of 4f orbital shielded from outer 6s, 5p and 5d orbitals, weakening the coupling of the surrounding ligands for the application optoelectronic device [28]. It's worth mentioning that the photoluminescence and photocatalytic properties of the rare earth elements doped ZnO are intensively studied and widely used in light-emitting devices and photocatalytic degradation [28,29]. Many methods can be used to prepare Eu-doped ZnO nanoparticles, such as hydrothermal synthesis [29], sol-gel [24] and co-precipitation method [30]. Of all those synthesis techniques, the sol-gel technology is one of common method to prepare Eu-doped ZnO nanostructures due to its simplicity, safety and low cost [31]. According to the survey, there are insufficient information available about impedance humidity sensors based on RE doped ZnO, in particular Eu-doped ZnO. Therefore, there still remains a tremendous challenge to study the mechanism and to

* Corresponding author.
E-mail address: zhanghongyanxj@163.com (H. Zhang).

https://doi.org/10.1016/j.cap.2018.11.015
Received 10 August 2018; Received in revised form 20 October 2018; Accepted 26 November 2018
Available online 28 November 2018
1567-1739/ © 2018 Korean Physical Society. Published by Elsevier B.V. All rights reserved.

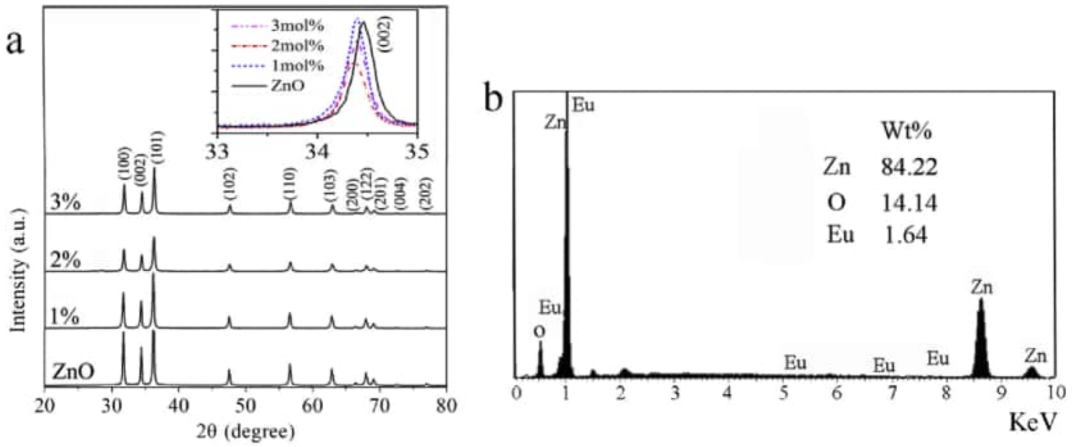

Fig. 1. (a) XRD spectra of undoped ZnO and Eu-doped ZnO. Inset of (a) is the magnified region of (002) peak. (b) EDS spectral analysis of Eu-doped ZnO.

develop effective method for controlling the Eu-doped ZnO nanostructures to improve humidity sensing properties.

On the basis of the above consideration, high humidity sensing performance has been achieved based on Eu-doped ZnO resistance humidity sensor by sol-gel method. The humidity experiments show that the impedance variation of the Eu-doped ZnO humidity sensor is of three orders impedance change, along with short response/recovery time (5 s/19 s), low hysteresis and best linearity in a range of 11–95% RH. The mechanism of humidity sensors based on Eu-doped ZnO is due to numerous oxygen vacancy defects on the surface and highly specific surface area of Eu-doped ZnO. The present results demonstrate that Eu-doped ZnO nanoparticles are suitable for realizing high humidity sensing performance. Considering abundant oxygen vacancy on the surface, high surface adsorption activity, fast response/recovery time and stability make such a realized Eu-doped ZnO as an efficient humidity sensor and will broaden the use of ZnO materials.

2. Experiments

2.1. Materials and characterizations

Zinc acetate ($Zn(CH_3COO)_2{\cdot}2H_2O$), monoethanolamine (MEA), europium oxide (Eu_2O_3) and ethanol were of analytical grade (AR), purchased from Sangon Biotech (China, www.sangon.com). All the reagents used were of analytical grade without any further purification. Deionized water (DI) was used in the hole experiments.

A JEM-2100F transmission electron microscope (Japan, Hitachi) was used to get transmission electron microscopic (TEM) images and S-4800 scanning electron microscope (Japan, Hitachi) was used to get scanning electron micrographs (SEM). A advance diffractometer (Germany, Bruker D8) was used to measure X-ray diffraction measurements (XRD). A Lambda 650 UV–vis spectrophotometer (US, PerkinElmer) was used to obtain absorption spectra with a wavelength range from 200.0 to 1000.0 nm. A Zennium workstation (Germany, ZAHNER) was used to measure resistance of the Eu-doped ZnO humidity sensor at alternating current (AC) 1V with a frequency change from 40 Hz to 100 kHz.

2.2. Preparation of Eu-doped ZnO

Synthesis precursor of Eu-doped ZnO (x mol%) was made by dissolving 2.3 g $Zn(CH_3COO)_2{\cdot}2H_2O$ and Eu_2O_3 (x mol%) in 30 mL mixture solution of DI water and ethanol (v:v = 1:2). The molar ratios of Eu^{3+} ions were 0 mol%, 1 mol%, 2 mol% and 3 mol% respectively. After being magnetically stirred for 5 min at 65 °C, 1 mL MEA was put into it drop by drop. A white homogeneous sol of Eu-doped ZnO was got after being stirred for 2 h. Then this solution was aged at room temperature for 36 h. Finally, the aged sol was heat-treated at 600 °C in a quartz-tube furnace for 2 h in nitrogen (N_2) and then grind it enough [32].

2.3. Humidity sensing experiments

For RH response of the Eu-doped ZnO nanoparticles, the grinding Eu-doped ZnO powder was mixed in a weight ratio of 5:1 by DI water to form a paste. In order to form a sensitive film, a five pair Ag-Pd interdigital electrodes (IDE) ceramic substrate (40 × 70 mm) was coated by the synthesized Eu-doped paste using brush. Then the film was dried at 60 °C for 10 min in vacuum. The controlled humidity environment were set at 11%, 33%, 54%, 75%, 85% and 95% RH through super saturated aqueous solution of LiCl, $MgCl_2$, $Mg(NO_3)_2$, NaCl, KCl and KNO_3 at room temperature, respectively [15,33,34]. To get reliable humidity sensing performance, the sensing device was kept in the flask with 11% RH at the beginning to obtain response transient. After the response sensing got steady, this device was switched to another flask with different RH as quick as possible about 1–2 s to reduce possible interference of response transients. The prepared Eu-doped ZnO sensor was put in a rubber-sealed flask in different RH from 11% to 95% RH.

3. Results and discussions

3.1. Characterizations

XRD spectra of undoped ZnO and Eu-doped ZnO with different molar ratios can be shown in Fig. 1(a). There are sharp and intensive diffraction peaks as (100), (002), (101), (102), (110), (103), (200), (122), (201), (004) and (202) planes of typical hexagonal wurtzite structure for ZnO (JCPDS No. 80-0075 diffraction card), which means the obtained Eu-doped ZnO are good in crystallized form. The magnified XRD spectra show that the diffraction peak of Eu-doped ZnO shifts slightly toward the lower angles compared to that of the undoped ZnO. Such a shift correlates to ZnO lattice expansion since the ionic radius of Eu^{3+} (0.98 Å) are much larger than that of Zn^{2+} (0.77 Å), which indicates that ZnO are successfully doped with Eu^{3+} ions [35]. The chemical composition of the Eu-doped ZnO is identified by EDS spectral analysis. Fig. 1(b) shows the respective elemental concentrations, which reveals that the synthetic products are composed of only Zn, O and Eu.

To study the states of surface oxygen of the Eu-doped ZnO, XPS characterization of O 1s in ZnO or Eu-doped ZnO is shown in Fig. 2. Fig. 2(a) and (b) show a typical O 1s peak of the undoped ZnO with three fitted Gaussian curves centered around at binding energy of 530.4 eV, 531 eV and 532 eV, corresponding to lattice oxygen (O_l), oxygen vacancy defect (O_v) and adsorbed oxygen (O_a) as well as OH^-, respectively [36]. The area ratio of each peak indicates the

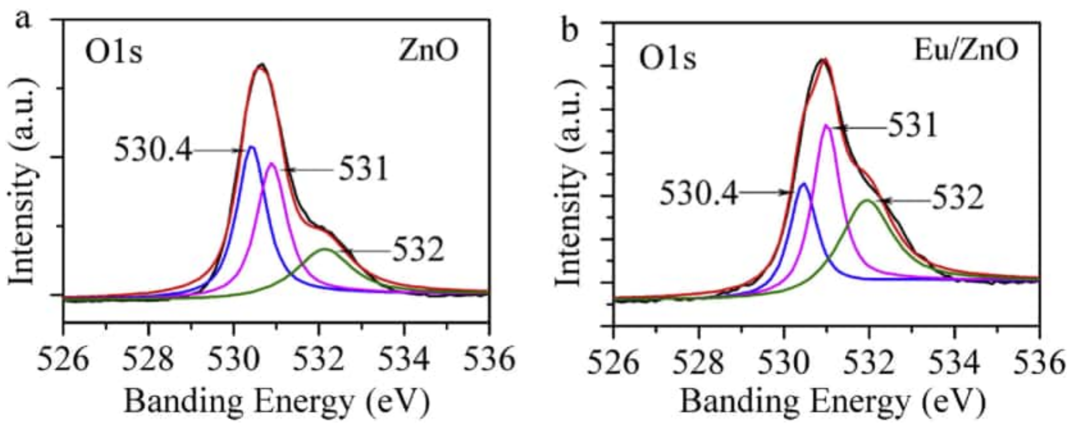

Fig. 2. XPS of O 1s of (a) undoped ZnO and (b) Eu-doped ZnO (2 mol%).

concentration of surface oxygen. Fig. 1(a) confirms that the lattice oxygen varies 41%, the oxygen vacancy defect varies 36% and the adsorbed oxygen varies 23% in undoped ZnO, respectively. But in Fig. 1(b), the lattice oxygen varies 26%, the oxygen vacancy defect varies 39% and the adsorbed oxygen varies 35% in Eu-doped ZnO, respectively. Compared the XPS of the undoped ZnO with the Eu-doped one, it can be seen that dopant Eu increases both the concentration of oxygen vacancy defects and adsorbed oxygen on the surface of Eu-doped ZnO, which means there are abundant oxygen vacancy defects on the surface of Eu-doped ZnO.

Scanning electron microscopy (SEM) was carried out to obtain detail information about the microstructure and morphology of the undoped ZnO, and the Eu-doped ZnO at different molar ratios as shown in Fig. 3. In Fig. 3(a), the undoped ZnO shows irregular disk-like nanostructures and spheroidal nanoparticles. Most of the ZnO nanoparticles are of size in diameter from 60 nm to 200 nm. The surface morphology of Eu-doped ZnO shows a significant morphological improvement as in Fig. 3(b). The addition of rare earth metal Eu in ZnO is beneficial for obtaining more uniform morphology with grain size of Eu-doped ZnO (2 mol%) from 50 nm to 75 nm. Such a shape has advantages of high activity and large specific surface area. Moreover, the differences of shape, particle size and distribution between ZnO and Eu-doped ZnO are observed in Fig. 3(c) and (d) by transmission electron microscopy (TEM) images. In Fig. 3(c), the size of ZnO nanoparticles is in a range from 60 nm to 150 nm and some particles are coalescent, which are consistent with the SEM images. In Fig. 3(d), uniformly distributed spherical nanoparticles can be clearly observed and the average size of Eu-doped ZnO is approximately from 50 nm to 70 nm, which means Eu doping causes the size of ZnO nanoparticles to be smaller and more uniform. As a result, more adsorption sites are produced by Eu-doped ZnO with large specific surface area.

The magnified crystal structure of Eu-doped ZnO is displayed in Fig. 4. It is found that the distance between two adjacent parallel planes lattice fringes is 0.25 nm or 0.52 nm, which agrees well with (101) or (001) planes of the hexagonal wurtzite ZnO or Eu [37,38]. The chemical composition for each Eu-doped ZnO from TEM is determined to be quite close to target compositions.

3.2. UV–vis analysis

Fig. 5(a) depicts the UV–visible diffuse reflectance spectra to investigate the optical absorption properties of ZnO and Eu-doped ZnO

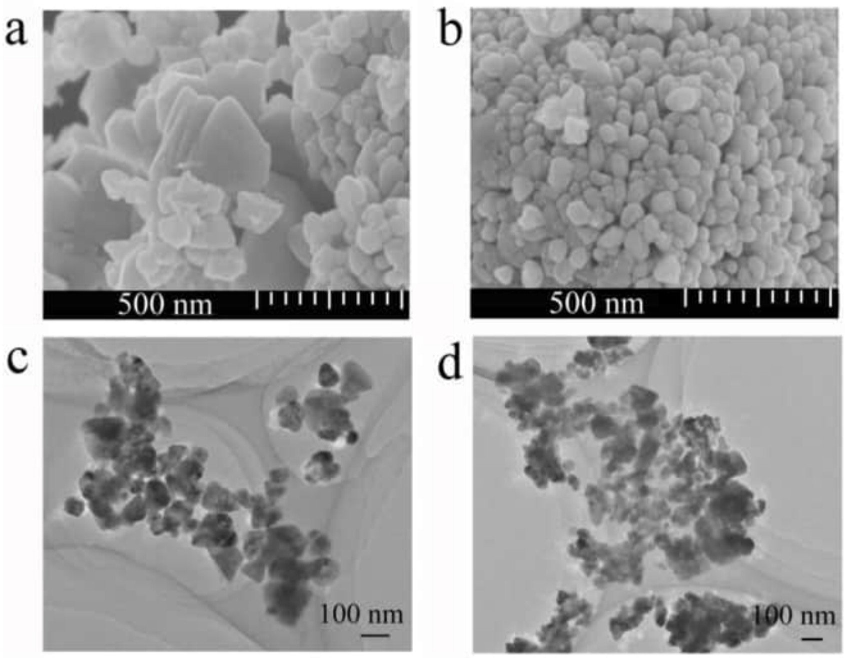

Fig. 3. FESEM images of (a) ZnO and (b) Eu-doped ZnO (2 mol%). TEM images of (c) ZnO and (d) Eu-doped ZnO (2 mol%).

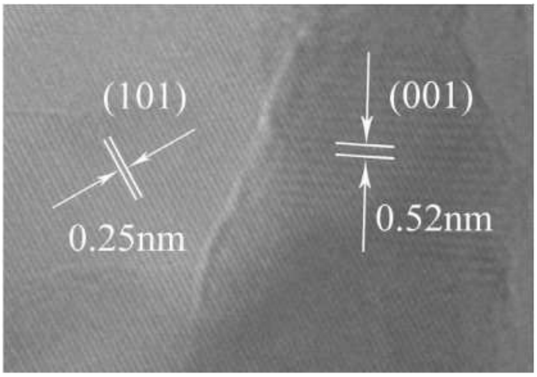

Fig. 4. Most favorable planes for crystallization (101) and (001) and grain boundary defects of Eu-doped ZnO (2 mol%).

with different doping ratios. The absorption peaks around 370 nm are related to excitonic absorption of nanostructured ZnO [38], and the bad gap of Eu-doped ZnO is effectively influenced by the amount of dopant Eu. All absorption band edges of Eu-doped ZnO show blue shifts compared to ZnO bulk value (395.4 nm). With more percentage of Eu^{3+} ion dopant, the peaks are shifts from 390.0 nm to 394.6 nm, which implies there is a growth of agglomeration of nanoparticles and reduction of particles size for the case of Eu-doped ZnO [39]. This result is in consistence with SEM and TEM images in Fig. 3. Fig. 5(b) shows the band gap energies (E_g) of Eu-doped ZnO with different molars calculated from their absorbance spectra by plotting of the square of the Kubelka-Munk function $\alpha h\nu = A(h\nu - E_g)$ with A being the absorbance, h the Plank constant and ν the frequency of light [40]. The band gap energies of the undoped ZnO and the Eu-doped ZnO for different molar ratios 1 mol%, 2 mol% and 3 mol% are 3.07 eV, 2.92 eV, 2.98 eV and 3.00 eV, respectively. The undoped and the Eu-doped ZnO show smaller band gap energies compared to that of typically reported 3.37 eV of traditional ZnO. Moreover, the presence of the Eu dopant can alter the band gap energy of ZnO.

3.3. Humidity sensing performances

Fig. 6(a) indicates the conductivity of Eu-doped ZnO with different molar ratios varying with RH in a range from 11% to 95% with the test frequency at 100 Hz. As observed, there is only one order change for undoped ZnO. Contrarily, a change of five orders for Eu-doped ZnO (1 mol%) is obtained though it's less than linear. In comparison with that of Eu-doped ZnO (1 mol%), Eu-doped ZnO (2 mol%) exhibit three order of magnitude decrease in impedance and good linearity, which means that Eu-doped ZnO (2 mol%) has the best humidity sensing performance. In the humidity sensing process, there is a steep decrease in impedance of Eu-doped ZnO when RH changes from 11% to 75% RH, while the changes of conductivity for Eu-doped ZnO are small when RH increases from 75% to 95%. This phenomenon comes from water molecule adsorptive process on the surface of Eu-doped ZnO, including chemisorption and physisorption. The decrease of impedance is not obvious when RH changes from 75% to 95%, because the water absorption capacity for physisorption layer is weaker than chemisorption layer and a continuous film of liquid water probably have already formed on Eu-doped ZnO (2 mol%). The change of impedance value is so low with linear scale that we can't appreciate it on Fig. 6 (a). Therefore, logarithmic coordinates are used in Fig. 6(b). Fig. 6(b) show that there is a good linear relationship using log coordinates between impedance of Eu-doped ZnO (2 mol%) and RH in the range from 75% to 95%. Based on the above discussions, we can conclude that Eu-doped ZnO (2 mol%) has the best humidity sensing performance, using linear coordinates in the moderate and low humidity area (11%–75% RH) and logarithmic coordinates in the high humidity area (75%–95% RH).

Test frequency has a great impact on the response of humidity sensor. In order to determine the best measuring frequency, as a function of frequency, the humidity response is obtained by varying from 40 Hz to 100 kHz as shown in Fig. 6(c). The impedance response decreases when the frequency increases, and the highest humidity sensitivity with good linearity is achieved at a frequency of 100 Hz. There is almost no observable change for impedance response at higher frequencies, which is result from that water it is difficult to be polarized at higher frequencies. As a result, 100 Hz is chosen to be the optimized working frequency in the following humidity experiments.

Hysteresis effect is usual phenomenon for metal oxide semiconductor humidity sensors, and it is important for sensing performance. Essentially hysteresis is easy to be influenced by RH. Furthermore, it may be induced by chemisorption on the surface of sensing element at initial states. In Fig. 7(a), the sensing curves for most parts of adsorption and desorption process are almost covered over each other, which indicates that there exist good adsorption/desorption properties. Fig. 7(b) depicts the response and the recovery curves of Eu-doped ZnO humidity sensors obtained at 100 Hz with RH from 11% to 95%. The response time or the recovery time is determined by the ability of the Eu-doped ZnO in desorption or adsorption of water vapor and the time for the sensor to achieve 90% of total resistance change for adsorption process or desorption process. For Eu-doped ZnO humidity sensor, the response time is about 5 s and the recovery time is about 19 s, which means that the Eu-doped ZnO humidity sensor performs good repeatability with fast response and recovery time. Moreover, the desorption process (recovery time) needs more time than that of the adsorption process (response time) due to that desorption needs external energy for water for further departing from the surface of Eu-doped ZnO [41].

In order to study the mechanism of humidity sensing for the Eu-

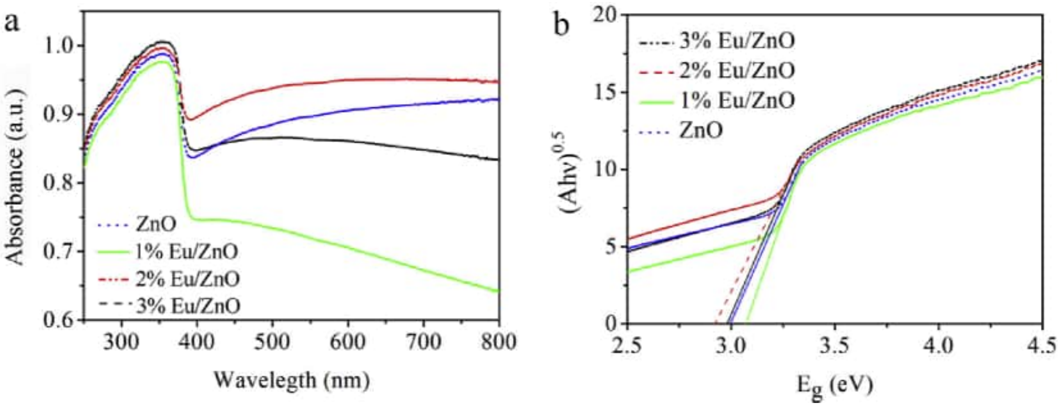

Fig. 5. (a) Absorbance spectra and (b) Plots to determine the band gap energy of the undoped and Eu-doped ZnO with different molar ratios.

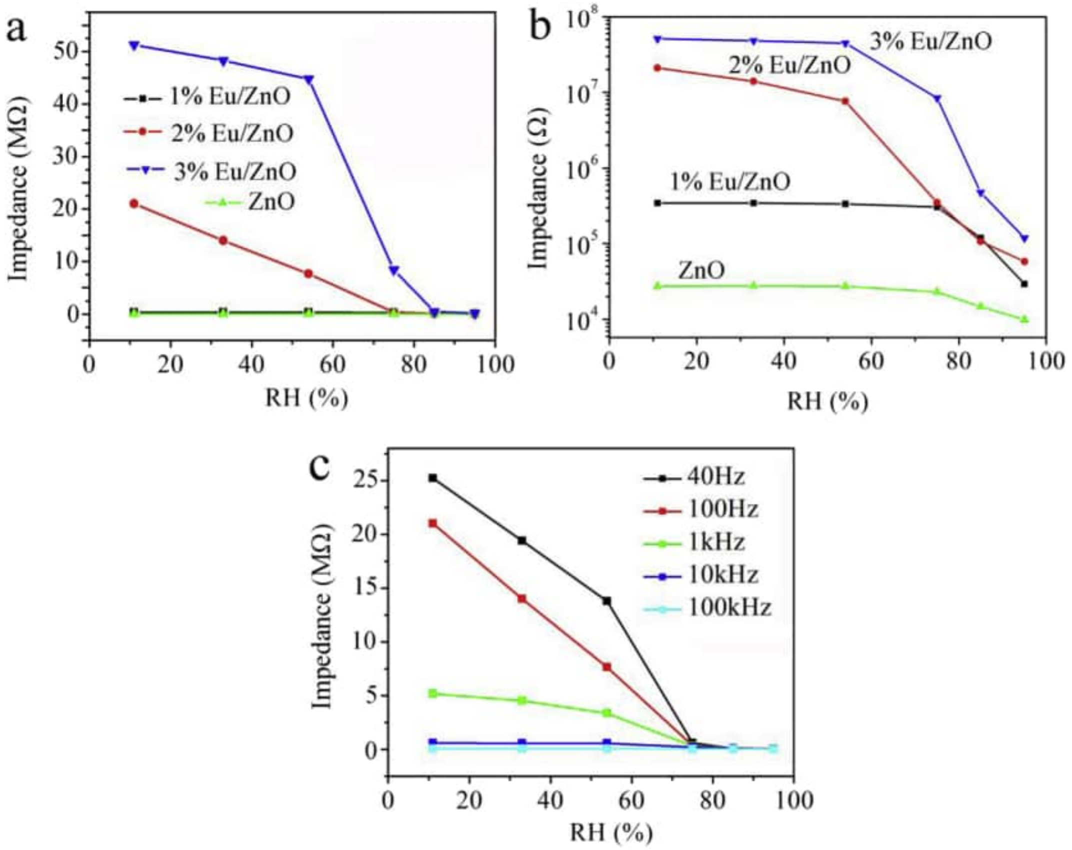

Fig. 6. Impedance versus RH curve of Eu-doped ZnO with different molar ratios with (a) linear coordinates and (b) logarithmic coordinates, respectively. (c) Impedance versus relative humidity curves of Eu-doped ZnO (2 mol%) at different frequencies.

doped ZnO, complex impedance spectra for the Eu-doped ZnO at different RH have been investigated as shown in Fig. 8. At low RH (11%, 33% and 54%), the plots of impedance change are semicircle. This is because that the water molecule adsorptive process on the surface of ZnO is consisted of both chemisorption and physisorption. With low RH, water molecules can be chemisorbed at first on the surface of Eu-doped ZnO because water molecules prefer to be absorbed on the oxygen vacancy defects. In this experiment, XPS results show that there are more vacancy defects of oxygen on the surface of ZnO, which means more water molecules can be absorbed on the surface of ZnO through vacancy defects of oxygen and hence provide high local charge density of dissociation for adsorbed water into OH^- and H_3O^+. Moreover, XPS results show that dopant Eu has increased adsorbed oxygen on the surface of ZnO and provides more quantity of OH species. As a consequence, the enhancement of OH species is useful for proton hopping transportation on the surface and it makes the sensor conductance increase. With further increase of the RH level (75%, 85% and 95% RH), the impedance changes into a straight line. That means the physisorption water layer is on the chemisorption water layer when the adsorbed water molecules increase. In these process, a large amount of H_3O^+ as the dominant charged carriers can accelerate the electrolytic conductive and release protons from the hydration of H_3O^+. Therefore, the smaller and uniformly distributed spherical nanoparticles of Eu-doped ZnO will lead to more active sites and adsorb more water molecules. As a result, the impedance of Eu-doped ZnO is decreased and the response is enhanced with high RH. The mechanism of the enhancement in

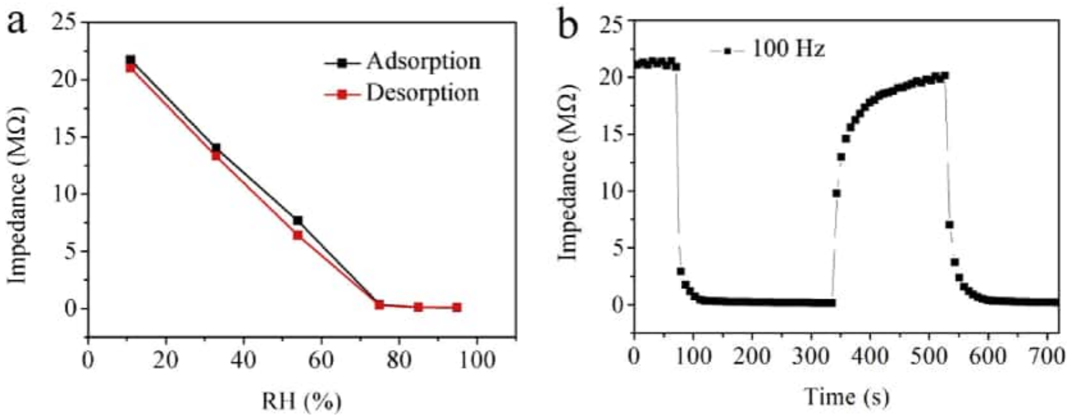

Fig. 7. (a) Humidity hysteresis characteristics and (b) response and recovery properties of Eu-doped ZnO (2 mol%) humidity sensor measured from 11% to 95% RH at 100 Hz.

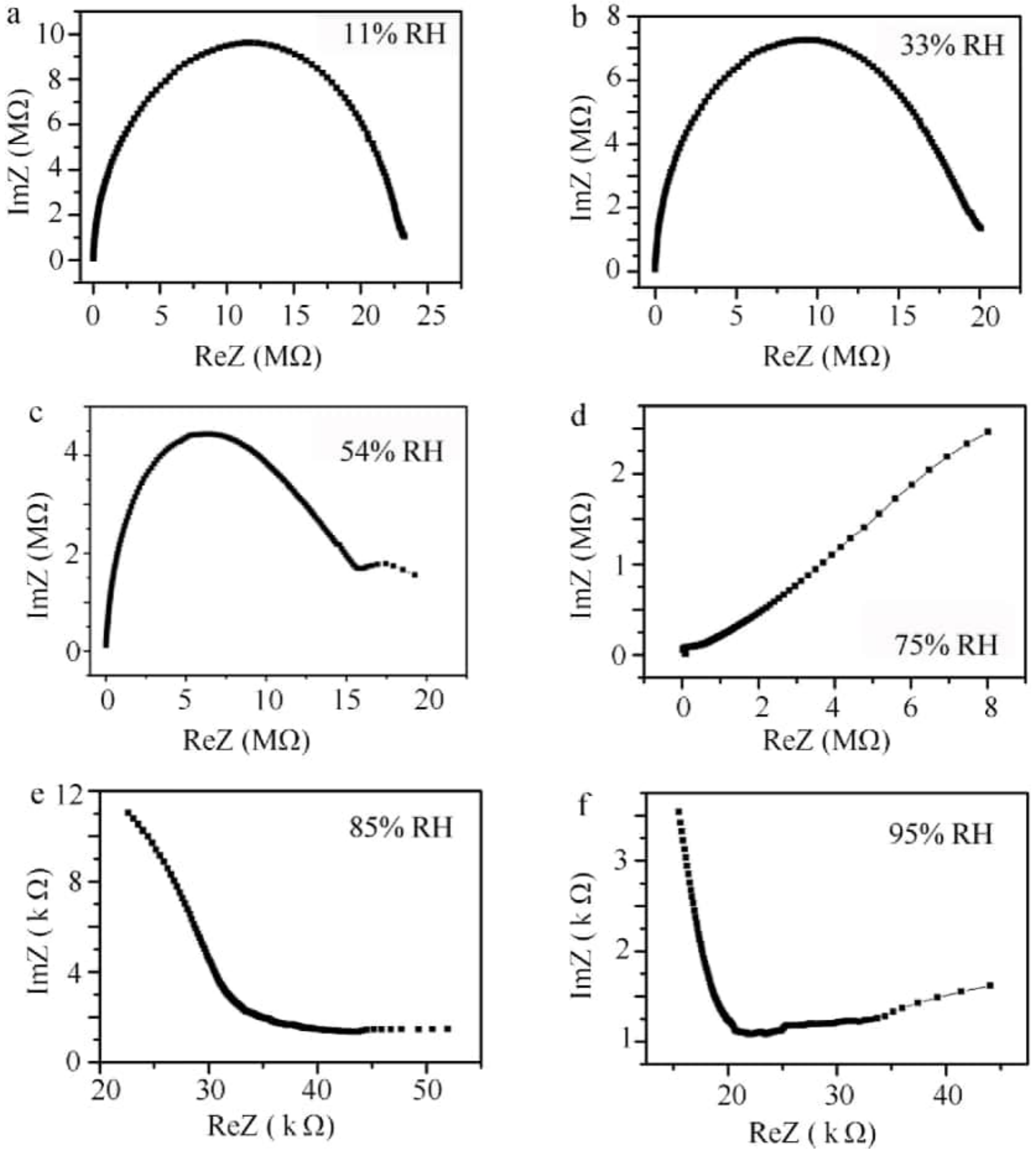

Fig. 8. Complex impedance spectra of the Eu-doped ZnO (2 mol%) at different RH.

sensing performance of Eu-doped ZnO is resulted from more surface oxygen vacancy defects and the smaller and uniformly distributed spherical nanoparticles.

4. Conclusions

In summary, Eu-doped ZnO with high humidity sensing properties was successfully synthesized by a simple and effective sol-gel method. Eu-doped ZnO particles are smaller in size and more uniform in distribution, making Eu-doped ZnO have a larger specific surface area and more active sites. Moreover, the dopant Eu can increase the oxygen vacancy defect concentration, which result in more water molecules that can be adsorbed on the ZnO surface. Humidity sensing impedance based on Eu-doped ZnO shows significant magnitude change of approximately three orders, excellent sensing linearity, small hysteresis, fast response time (5 s) and recovery time (19 s). The superior humidity sensing performance of Eu-doped ZnO is resulted from surface oxygen vacancy defects and large specific surface area caused by Eu doping. Consequently, this work makes the Eu-doped ZnO as an efficient humidity sensor interesting for humidity detection, which will broaden the use of ZnO materials.

Conflict of interest

The authors declared that they have no conflicts of interest to this work. We declare that we do not have any commercial or associative interest that represents a conflict of interest in connection with the work submitted.

Acknowledgements

This work was supported by the National Science Foundation of China (No. 61665011, No. 11504313 and No. 11564039).

References

[1] S. Rackauskas, K. Mustonen1, T. Järvinen, M. Mattila, O. Klimova, H. Jiang, O. Tolochko, H. Lipsanen, E. Kauppinen, A.G. Nasibulin, Synthesis of ZnO tetrapods for flexible and transparent UV sensors, Nanotechnology 23 (9) (2012) 095502.
[2] F.S. Tsaia, S.J. Wang, Enhanced sensing performance of relative humidity sensors using laterally grown ZnO nanosheets, Sens. Actuators B 193 (2014) 280–287.

[3] P. She, K. Xu, S. Zeng, Q. He, H. Sun, Z. Liu, Investigating the size effect of Au nanospheres on the photocatalytic activity of Au-modified ZnO nanorods, J. Colloid Interface Sci. 499 (2017) 76–82.
[4] P.J. Jeon, Y.T. Lee, J.Y. Lim, J.S. Kim, D.K. Hwang, S. Im, Black Phosphorus − Zinc oxide nanomaterial heterojunction for p − n diode and junction field-effect transistor, Nano Lett. 16 (2016) 1293–1298.
[5] S. Rackauskas, N. Barbero, C. Barolo, G. IViscardi, ZnO nanowire application in chemoresistive sensing: a review, Nanomaterials 7 (2017) 381.
[6] V. Havasi, B. Vörédi, Á. Kukovecz, Photocatalytic performance of Sr4Al14O25:Eu, Dy phosphor assisted ZnO: Co^+ Ag nanocomposite under continuous and pulsed illumination, Catal. Today 284 (2017) 107–113.
[7] B.M. Rajbongshi, S.K. Samdarshi, ZnO and Co-ZnO nanorods—complementary role of oxygen vacancy in photocatalytic activity of under UV and visible radiation flux, Mater. Sci. Eng. B 182 (2014) 21–28.
[8] Y. Wang, Y. Yang, L. Xi, X. Zhang, M. Jia, H. Xu, H. Wu, A simple hydrothermal synthesis of flower-like ZnO microspheres and their improved photocatalytic activity, Mater. Lett. 180 (2016) 55–58.
[9] A.D. Mauro, M.E. Fragalà, V. Privitera, G. Impellizzeri, ZnO for application in photocatalysis: from thin films to nanostructures, Mater. Sci. Semicond. Process. 69 (2017) 44–51.
[10] K. Rahimi, A. Yazdani, Improving photocatalytic activity of ZnO nanorods: a comparison between thermal decomposition of zinc acetate under vacuum and in ambient air, Mater. Sci. Semicond. Process. 80 (2018) 38–43.
[11] J. Lin, Z. Luo, J. Liu, P. Li, Photocatalytic degradation of methylene blue in aqueous solution by using ZnO-SnO_2 nanocomposites, Mater. Sci. Semicond. Process. 87 (2018) 24–31.
[12] E. Modaresinezhad, S. Darbari, Realization of a room-temperature/self-powered humidity sensor, based on ZnO nanosheets, Sens. Actuators B 237 (2016) 358– 336.
[13] K. Narimani, F.D. Nayeri, M. Kolahdouz, Fabrication, modeling and simulation of high sensitivity capacitive humidity sensors based on ZnO nanorods, Sens. Actuators B 224 (2016) 338–343.
[14] S. Brahma, C.W. Yang, C.H. Wu, F.M. Chang, T.J. Wu, C.S. Huang, K.Y. Lo, The optical response of ZnO nanorods induced by oxygen chemisorption and desorption, Sens. Actuators B 259 (2018) 900–907.
[15] M. Gong, Y. Li, Y. Guo, X. Lv, X. Dou, 2D TiO_2 nanosheets for ultrasensitive humidity sensing application benefited by abundant surface oxygen vacancy defects, Sens. Actuators B 262 (2018) 350–358.
[16] S. Brahma, C. Yang, C.H. Wu, F.M. Chang, T.J. Wu, C.S. Huang, K.Y. Lo, The optical response of ZnO nanorods induced by oxygen chemisorption and desorption, Sens. Actuators B 259 (2018) 900–907.
[17] M.H. Feng, W.C. Wang, X.J. Li, Capacitive humidity sensing properties of CdS/ZnO sesame-seed-candy structure grown on silicon nanoporous pillar array, J. Alloys Compd. 698 (25) (2017) 94–98.
[18] W. Li, Y. Ma, S. Ji, G. Sun, P. Jin, Synthesis and humidity sensing properties of the VO2(B)@ZnO heterostructured nanorods, Ceram. Int. 42 (7) (2016) 9234–9240.
[19] M. Hassan, A.S. Afify, J.M. Tulliani, Synthesis of ZnO Nanoparticles onto sepiolite needles and determination of their sensitivity toward Humidity, NO2 and H2, J. Mater. Sci. Technol. 32 (6) (2016) 573–582.
[20] K. Joshi, M. Rawat, S.K. Gautam, R.G. Singh, R.C. Ramola, F. Singh, Band gap widening and narrowing in Cu-doped ZnO thin films, J. Alloys Compd. 680 (25) (2016) 252–258.
[21] Z. Quan, X. Liu, Y. Qi, Z. Song, S. Qi, G. Zhou, X. Xu, Robust room temperature ferromagnetism and band gap tuning in nonmagnetic Mg doped ZnO films, Appl. Surf. Sci. 399 (31) (2017) 751–757.
[22] S. Shet, Y. Yan, J. Turner, M.A. Jassim, Effect of gas ambient and varying RF sputtering power for bandgap narrowing of mixed (ZnO:GaN) thin films for solar driven hydrogen production, J. Power Sources 232 (15) (2013) 74–78.
[23] L.T.F. Jule, B. Dejene, A.G. Ali, K.T. Roro, A. Hegazy, N.K. Allam, E.E. Shenawy, Wide visible emission and narrowing band gap in Cd-doped ZnO nanopowders synthesized via sol-gel route, J. Alloy. Comp. 687 (5) (2016) 920–926.
[24] J. Mrázek, L. Spanhel, M. Surynek, M. Potel, V. Matejec, Crystallization properties of RE-doped (RE = Eu, Er, Tm) Zn_2TiO_4 prepared by the sol-gel method, J. Alloys Compd. 509 (2011) 4018–4024.
[25] N. Fifere, A. Airinei, D. Timpu, A. Rotaru, L. Sacarescu, L. Ursu, New insights into structural and magnetic properties of Ce doped ZnO nanoparticles, J. Alloys Compd. 757 (2018) 60–69.
[26] F. Pavón, A. Urbieta, P. Fernández, Luminescence and light guiding properties of Er and Li codoped ZnO nanostructures, J. Lumin. 195 (2018) 396–401.
[27] R.K. Kalaiezhily, G. Saravanan, V. Asvini, N. Vijayan, K. Ravichandran, Tuning violet to green emission in luminomagnetic Dy,Er co-doped ZnO nanoparticles, Ceram. Int. 44 (2018) 19560–19569.
[28] B. B. K.Sarma Sarma, Fabrication of Ag/ZnO heterostructure and the role of surface coverage of ZnO microrods by Ag nanoparticles on the photophysical and photocatalytic properties of the metal-semiconductor system, Appl. Surf. Sci. 410 (15) (2017) 557–565.
[29] J.C. Sin, S.M. Lam, Hydrothermal synthesis of europium-doped flower-like ZnO hierarchical structures with enhanced sunlight photocatalytic degradation of phenol, Mater. Lett. 182 (2016) 223–226.
[30] S. Fabbiyola, L. JohnKennedya, T. Ratnaji, J.J. Vijaya, U. Aruldoss, M. Bououdin, Effect of Fe-doping on the structural, optical and magnetic properties of ZnO nanostructures synthesized by co-precipitation method, Ceram. Int. 42 (2016) 1588–1596.
[31] K.J. Chen, F.Y. Hung, S.J. Chang, S.J. Young, Z.S. Hu, Effects of crystallization on the optical properties of ZnO nano-pillar thin films by sol-gel method, Curr. Appl. Phys. 11 (2011) 1243–1248.
[32] H. Zhang, M. Zhang, C. Lin, J. Zhang, AuNPs hybrid black ZnO nanorods madeby a sol-gel method for highly sensitive humidity sensing, Sensors 18 (218) (2018) 1–10.
[33] Y. Zhang, Y. Chen, Y. Zhang, X. Cheng, C. Feng, L. Chen, J. Zhou, S. Ruan, A novel humidity sensor based on $NaTaO_3$ nanocrystalline, Sens. Actuators B 174 (2012) 485–489.
[34] L.L. Wang, H.Y. Wang, W.C. Wang, K. Li, X.C. Wang, X.J. Li, Capacitive humidity sensing properties of ZnO cauliflowers grown on silicon nanoporous pillar array, Sens. Actuators B 177 (2013) 740–744.
[35] L.V. Trandafilovi, D.J. Jovanovi, X. Zhang, S. Ptasi´nsk, M.D. Drami´canin, Enhanced photocatalytic degradation of methylene blue and methyl orange by ZnO:Eu nanoparticles, Appl. Catal. B Environ. 203 (2017) 740–752.
[36] S. Wu, Z. Chen, T. Wang, X. Ji, A facile approach for the fabrication of Au/ZnO-hollow-sphere-monolayer thin films and their photocatalytic properties, Appl. Surf. Sci. 412 (2017) 69–76.
[37] L.F. Koao, B.F. Dejene, H.C. Swart, S.V. Motloung, T.E. Motaung, Characterization of annealed Eu^{3+}-doped ZnO flower-like morphology synthesized by chemical bath deposition method, Opt. Mater. 60 (2016) 294–304.
[38] T. Liu, W. Chen, Y. Hua, X. Liu, Au/ZnO nanoarchitectures with Au as both supporter and antenna of visible-light, Appl. Surf. Sci. 392 (2017) 616–623.
[39] H.V.S.L. Pessoni, J.Q. Maia, A.F. Jr, Eu-doped ZnO nanoparticles prepared by the combustion reaction method: structural, photoluminescence and dielectric characterization, Mater. Sci. Semicond. Process. 30 (2015) 135–141.
[40] K. Zhao, Y. Lu, N. Lu, Y. Zhao, X. Yuan, H. Zhang, L. Teng, F. Li, Design of $H_3PW_{12}O_{40}/TiO_2$ nano-photocatalyst for efficient photocatalysis under simulated sunlight irradiation, Appl. Surf. Sci. 285 (2013) 616–624.
[41] F.S. Tsai, S.J. Wang, Enhanced sensing performance of relative humidity sensors using laterally grown ZnO nanosheets, Sens. Actuators B 193 (2014) 280–287.

Sensors & Actuators: B. Chemical 325 (2020) 128810

Contents lists available at ScienceDirect

Sensors and Actuators: B. Chemical

journal homepage: www.elsevier.com/locate/snb

A facile PDMS coating approach to room-temperature gas sensors with high humidity resistance and long-term stability

Zhimin Gao [a], Guoshuai Song [a], Xuemin Zhang [a,*], Qian Li [a], Shuang Yang [a], Tieqiang Wang [a], Yunong Li [a], Liying Zhang [a], Lei Guo [b,*], Yu Fu [a,*]

[a] College of Sciences, Northeastern University, Shenyang, 110189, People's Republic of China
[b] Texas A&M Institute of Biosciences & Technology, Houston, TX, 77030, USA

ARTICLE INFO

Keywords:
Room-temperature gas sensor
PDMS coating
Humidity resistance
Long-term stability
Pd decorated TiO_2 nanotubes

ABSTRACT

The interference from relative humidity (RH) poses a critical issue for the practical application of room-temperature gas sensors. We demonstrate herein coating gas sensors with a hydrophobic polydimethylsiloxane (PDMS) layer can significantly enhance their humidity resistance. Pd nanoparticles (NPs) decorated TiO_2 nanotubes (Pd/TiO_2 NTs) is selected as a typical room-temperature gas sensor. By a simple thermal evaporation process, a conformal PDMS layer can be coated on the nanostructured sensing material. PDMS layer not only endows the sensor with a hydrophobic surface to water droplet, but also has a sufficient thickness to effectively prevent the penetration of water molecules. In contrast, other hydrophobic modification layer (such as silane coupling agent) can only make gas sensors superhydrophobic but not able to enhance their humidity resistance. Additionally, PDMS layer improves the long-term stability of Pd/TiO_2 NTs sensors through affording protection of Pd NPs on the surface of gas sensors. Furthermore, PDMS layer is stable enough to resist the degradation during long-term usage. The PDMS coating strategy is simple and can be applied to gas sensors with various morphologies, which promotes the real applications of room-temperature gas sensors in moist environments.

1. Introduction

Room-temperature gas sensors that offer real-time information of the presence of a specific gas at low power consumption have extensive applications in fields such as public security protection, environmental monitoring and medical diagnosis [1–6], and have been considered as an essential part in the system of internet of things [7]. However, relative humidity (RH) is a notorious interferent to room-temperature gas sensors. It is well accepted water molecules will occupy the active sites on the surface of sensing materials, which thereby influences the adsorption and equilibrium processes of the target gas [8–11], thus resulting the sensor response dramatically fluctuating with RH (the loss of sensor's reliability). This problem severely limits the application of gas sensors in real environments with variable ambient humidity.

Catalyst nanoparticles (NPs) decorated semiconductor metal oxide (SMO) is considered as the most promising candidate for commercial room-temperature gas sensors [12,13]. Despite its remarkable advantages including simple fabrication procedure, low cost and high compatibility to current electronic system, this type of sensor suffers from humidity seriously due to the intrinsic hydrophilicity of most SMO [14,15]. Although great efforts have been made to increase the humidity resistance of SMO-based room-temperature gas sensors, such as functionalization of sensor materials with hydrophobic self-assembled monolayers (SAMs), filter layers or some other special additives [16–20], it is still quite challenging to eliminate the negative influences of RH. As a result, it is imperative to develop a facile strategy to improve the humidity resistance of gas sensors for their practical usage.

Polydimethylsiloxane (PDMS) is a stable hydrophobic polymer that has been coated on solid surfaces to enhance their water resistance [21,22]. For example, Zhang et al. reported a simple PDMS-coating treatment that formed a protective hydrophobic layer on the surface of metal-organic frameworks (MOFs) for improving their structural/chemical stability under moisture condition [22]. Inspired by these works, we expect PDMS coating can be used as a passivation layer that enhances the humidity resistance of room-temperature gas sensors. The choice of PDMS has many advantages compared with other polymers and organic SAMs. Firstly, a conformal PDMS layer can be coated on the nanostructured sensing material by a simple thermal evaporation

* Corresponding author.
E-mail addresses: zhangxuemin@mail.neu.edu.cn (X. Zhang), guoleijay@tamu.edu (L. Guo), Fuyu@mail.neu.edu.cn (Y. Fu).

https://doi.org/10.1016/j.snb.2020.128810
Received 6 April 2020; Received in revised form 2 August 2020; Accepted 25 August 2020
Available online 28 August 2020
0925-4005/© 2020 Elsevier B.V. All rights reserved.

process [23]. The PDMS layer not only endows the sensor with a hydrophobic surface to water droplet, but also has a sufficient thickness to effectively prevent the penetration of water molecules. Secondly, PDMS is stable enough to resist the degradation during long-term usage [21]. This is particularly important when most SMO-based sensing materials, such as TiO_2, ZnO and WO_3, have excellent photodecomposition ability. Finally, as most SMO-based gas sensors are sensitized by catalyst NPs to achieve room-temperature sensing, coating PDMS is helpful to afford protection and prevent the aggregation of these catalyst NPs [24]. As a proof of concept, Pd NPs decorated TiO_2 nanotubes (Pd/TiO_2 NTs), a commonly investigated room-temperature H_2 sensor, is selected as a typical SMO-based gas sensor [25,26]. We demonstrate herein coating PDMS remarkably enhances the humidity resistance and long-term stability of Pd/TiO_2 NTs sensors. PDMS coating is stable enough to sustain the photodegradation by TiO_2, while other hydrophobic organic modification layers are decomposed easily. Additionally, the complex nanostructure of NTs also proves the PDMS coating strategy is versatile and can be applied to gas sensors with various morphologies.

2. Experimental section

2.1. Materials

The water used in all the experiments was Milli-Q water (18.2 MΩ cm). PDMS elastomer kits (Sylgard 184) were purchased from Dow Corning (Midland, MI). Sodium hydroxide (NaOH), sodium borohydride ($NaBH_4$), palladium chloride ($PdCl_2$), hydrochloric acid (HCl), ammonium fluoride (NH_4F), ethylene glycol, hexyltriethoxysilane (HTS), 1H,1H,2H,2H-perfluorodecyltriethoxysilane (PFS) were used as received.

2.2. Preparation of Pd/TiO_2 NTs

TiO_2 NTs were prepared by anodization of Ti foil (99%, 22 mm × 22 mm × 0.20 mm) at room temperature in ethylene glycol solution containing 0.5 wt% NH_4F and 3 vol% H_2O [27]. The anodization process was carried out with two Ti foils serving as both the anode and the cathode. The oxidation process consisted of two steps. The anodizing voltage of first step was 60 V and Ti foil was oxidized for 1 h. Then the anodized samples were ultrasonicated in deionized water for 3 min. In the second step, the anodizing voltage was 60 V and the oxidation time was 2 h. After that, the oxidized sample was kept upright, and ultrasonically washed in ethanol solution. Finally, the sample was calcined in a tubular furnace at 450 °C for 3 h in air with heating rate of 10 °C min^{-1}. For the synthesis of Pd NPs, 60 ml freshly prepared $NaBH_4$ solution (1 mM) was added into 15 ml H_2PdCl_4 (10 mM) solution under magnetic stirring. To deposit Pd NPs onto TiO_2 NTs, TiO_2 NTs were immersed into Pd NP solution and kept upright overnight and then dried in in a nitrogen stream.

2.3. Preparation of PDMS coated Pd/TiO_2 NTs (PDMS-Pd/TiO_2 NTs)

PDMS stamps were prepared by adding PDMS prepolymer and crosslinker into glass culture dish at the volume ratio of 10:1, and then heated in an oven at 60 °C for 4 h. The coating process was conducted by heating Pd/TiO_2 NTs in the presence of PDMS stamps (1.46 g) in a sealed glass desiccator (diameter of 120 mm) at 235 °C for various duration and then cooled naturally [23].

2.4. Preparation of HTS (or PFS) modified Pd/TiO_2 NTs

Pd/TiO_2 NTs were placed in a sealed glass container, on the bottom of which was dispensed a few drops of HTS (or PFS). The glass container was put in an oven at 90 °C for 7 h to enable the vapor of HTS (or PFS) to react with the -OH groups on the TiO_2 surface, and then cooled naturally.

2.5. Preparation and characterization of sensing performance of sensors

Conductive silver paste was dropped on the surface of PDMS-Pd/TiO_2 NTs with separation of ~1 cm, and then dried in air. The H_2 sensing performance of as-prepared samples were evaluated using a gas sensing analysis system (CGS-MT, Beijing Elite Tech Co) equipped with a 1 L chamber [28,29]. 1 V voltage was applied between the electrode. The test temperature was controlled by a heating stage underneath the sample with constant temperature of 25 °C. Meanwhile, the ambient humidity was adjusted by humidifier from 25% RH to 75% RH. In the experiment, the concentration of H_2 was controlled through injecting a certain amount of H_2 into the chamber by a microsyringe. Before sensing performance testing, all the freshly prepared sensors were aged in H_2 atmosphere for several hours until they achieved a stable baseline. The sensor response is defined as $(I_g-I_a)/I_a$ [or $(R_a-R_g)/R_g$], where I_g (R_g) and I_a (R_a) are the electrical current (resistance) of sensor in H_2 and air, respectively. The response time is defined as the time required for the variation in current to reach 90% of the equilibrium value after H_2 injection and the recovery time is the time necessary for the sensor to return to 10% above the base current in air after releasing H_2.

2.6. Characterization

The X-ray diffraction (XRD) patterns were recorded on a PANalytical Empyrean instrument equipped with Cu Kα1 radiation source (λ = 1.5406 Å). The morphology and elemental analysis of the samples were examined using a Hitachi SU1080 field emission scanning electron microscopy (SEM) operating at primary electron energy of 15 kV equipped with an energy-dispersive X-ray spectroscopy (EDX) instrument. The surface chemical characteristics of the samples were investigated by X-ray photoelectron spectroscopy (XPS, ESCALAB250). Water contact angle (CA) was measured at room temperature using a drop shape analysis system (DSA 10 MK2, KRÜSS). The organic groups in PDMS-Pd/TiO_2 NTs were characterized by Fourier transform infrared spectroscopy (FTIR, Bruker VERTEX 70).

3. Results and discussions

3.1. Preparation and characterization of PDMS-Pd/TiO_2 NTs

Fig. 1 shows the schematic illustration for the preparation of PDMS-Pd/TiO_2 NTs. Pd/TiO_2 NTs were firstly prepared according to previously reported method [25,27]. To synthesize PDMS-Pd/TiO_2 NTs, the pristine Pd/TiO_2 NTs were placed in a sealed glass container with freshly prepared PDMS stamp. The glass container was kept at 235 °C for a certain of time and then allowed to cool to room temperature naturally. During thermal evaporation, volatile silicone molecules in the form of short PDMS chains will form a conformal layer on the surface of Pd/TiO_2 NTs and then crosslink [23,30], which acts as a passivation layer to eliminate the influence of humidity in the subsequent gas sensing measurements.

Fig. 2A shows the SEM images of pristine Pd/TiO_2 NTs, from which it can be seen that Pd NPs of ~16 nm are well distributed on the surface of TiO_2 NTs (inner diameter of ~100 nm with wall thickness of ~17 nm, see Figure S1A). XRD spectrum reveals TiO_2 NTs have both anatase and rutile phases which are represented as A and R in Figure S1B. No diffraction peaks corresponding to metallic Pd NPs are found in XRD spectrum probably because they are overlapped by the intensive peaks from TiO_2. As shown in Fig. 2B, there is no obvious difference in morphology when Pd/TiO_2 NTs are coated by PDMS, confirming that Pd NPs and TiO_2 NTs both remain stable during PDMS deposition process [24].

According to previous reports, the conformal PDMS layer is only 2-3 nm in thickness [31,32], making it difficult to observe the PDMS layer directly. However, its presence can be identified using other measurements such as EDX, XPS and FTIR. EDX shown as the inset of Fig. 2 and

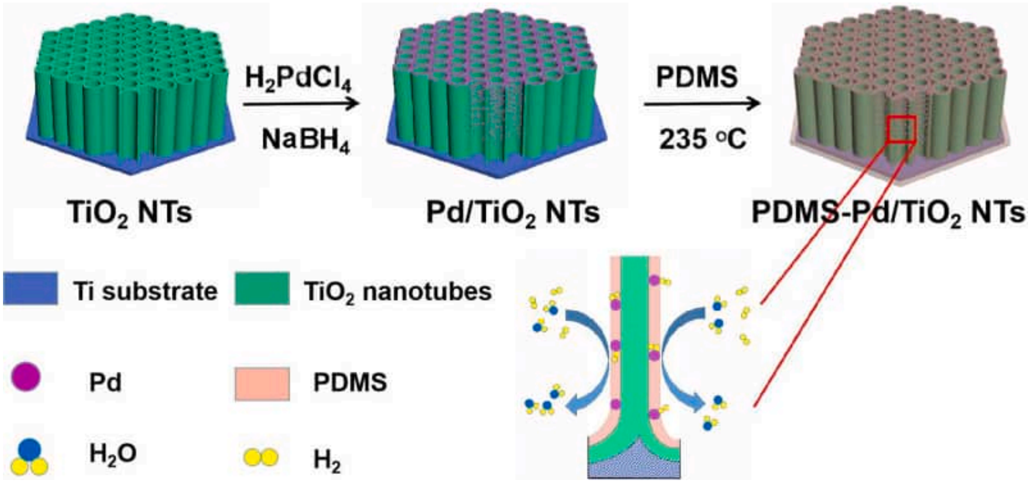

Fig. 1. Schematic illustration of the procedures for the synthesis of PDMS-Pd/TiO_2 NTs. The magnified picture shows the PDMS thin film acts as a filter to eliminate the influence of water molecules on the gas sensors.

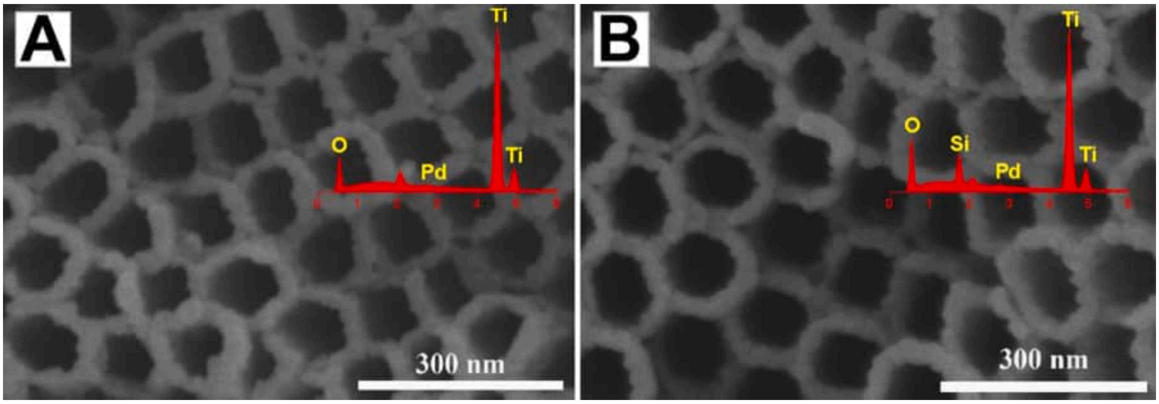

Fig. 2. SEM images of pristine Pd/TiO NTs (A) and PDMS-Pd/TiO_2 NTs (B). The insets of (A) and (B) are EDX spectra of pristine Pd/TiO NTs and PDMS-Pd/TiO_2 NTs.

XPS peak characteristic shown in Fig. 3A and Figure S2 display the appearance of Si peak after Pd/TiO_2 NTs were coated by PDMS. Furthermore, the presence of Si 2p peak at 102.1 eV in XPS spectrum implies Si is not fully oxidized [32], which therefore can be assigned as Si atoms in siloxane framework. In the meanwhile, as shown in Fig. 3B, pristine Pd/TiO_2 NTs exhibit O 1s peak at 529.9 eV, while PDMS-Pd/TiO_2 NTs have additional O 1s shoulder at 532.0 eV that corresponds to the oxygen in siloxane [33]. With respect to Ti, its peak intensity significantly decreases after thermal deposition due to the influence of PDMS layer (shown as Fig. 3C). Additionally, both of binding energies of Ti and Pd show a slightly shift to higher energy after coated by PDMS (shown as Fig. 3C to 3E), suggesting the possible bond formation between the pristine gas sensor and PDMS layer [21,22]. The functional group in PDMS-Pd/TiO_2 NTs is further characterized by FTIR (Figure S3). Compared with pristine Pd/TiO_2 NTs, the appearance of absorption bands at 2960 and 2897 cm^{-1} after thermal deposition can be assigned respectively as the asymmetric and symmetric stretching of -CH_3 groups in PDMS [31].

TiO_2 NTs prepared in our experiment have a complex morphology with tube length of ~20 μm. To verify that PDMS layers could be deposited on the inner parts of TiO_2 NTs, cross-sectional elemental measurement of PDMS coated Pd/TiO_2 NTs are carried out (as shown in Fig. 4). EDX mapping shows Si which derives from to the PDMS layer is well distributed throughout the cross section of sample, indicating the PDMS can diffuse throughout the structure and form a coating layer on the inner wall surface of TiO_2 NTs.

As PDMS is thermally evaporated on the Pd/TiO_2 NTs, we suppose the amount (or the thickness) of PDMS layer can be tuned through adjusting the deposition time. This assumption is proved by the EDX elemental analysis, which shows the atomic ratio of Si/Ti increases from 0.25 to 0.33, and the atomic ratio of Si/Pd increased from 1.5 to 2.0 when the deposition time is prolonged from 3 h to 7 h. This tunability offers us ability to investigate the influence of PDMS thickness on the humidity resistance of as-prepared gas sensors.

The surface wettability of as-prepared gas sensors is characterized by water CA measurement (shown as the insets of Fig. 5). The pristine Pd/TiO_2 NTs shows a superhydrophilic surface that water droplets diffuse into NTs very quickly (static water CA of ~0° and wetting time less than 0.5 s) due to the porous morphology and intrinsic hydrophilic property of TiO_2 [15]. In contrast, PDMS-Pd/TiO_2 NTs exhibit CA larger than 150° (sliding angle less than 3°). This perfect superhydrophobic nature drives from the hydrophobicity of PDMS combined with the surface roughness of Pd/TiO_2 NTs [34]. At the same time, it is interesting to note, independent of the deposition time, all PDMS-Pd/TiO_2 NTs reveal similar superhydrophobicity, even when the PDMS deposition time is as short as 1 h. Water droplets run away easily with almost no adherence to all PDMS-Pd/TiO_2 NTs samples, revealing these samples exhibit perfect resistance to water droplets.

3.2. *Influence of PDMS layer to the sensor performance*

Figure S4 shows the real-time sensing performance of pristine Pd/TiO_2 NTs to H_2 gas of various concentrations at room temperature under 50 % RH. The current of the sensor increases abruptly after the injection

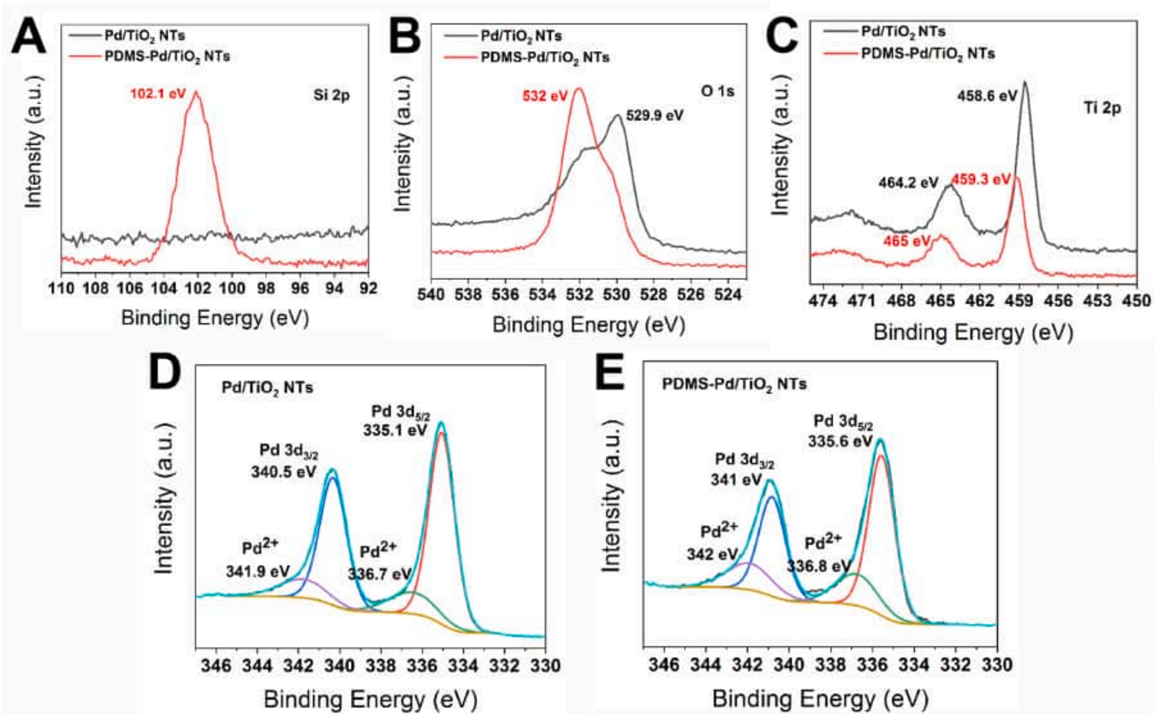

Fig. 3. XPS spectra of Si 2p (A), O 1s (B), and Ti 2p (C) for pristine Pd/TiO_2 NTs and PDMS-Pd/TiO_2 NTs. XPS spectra of Pd before (D) and after (E) PDMS deposition process.

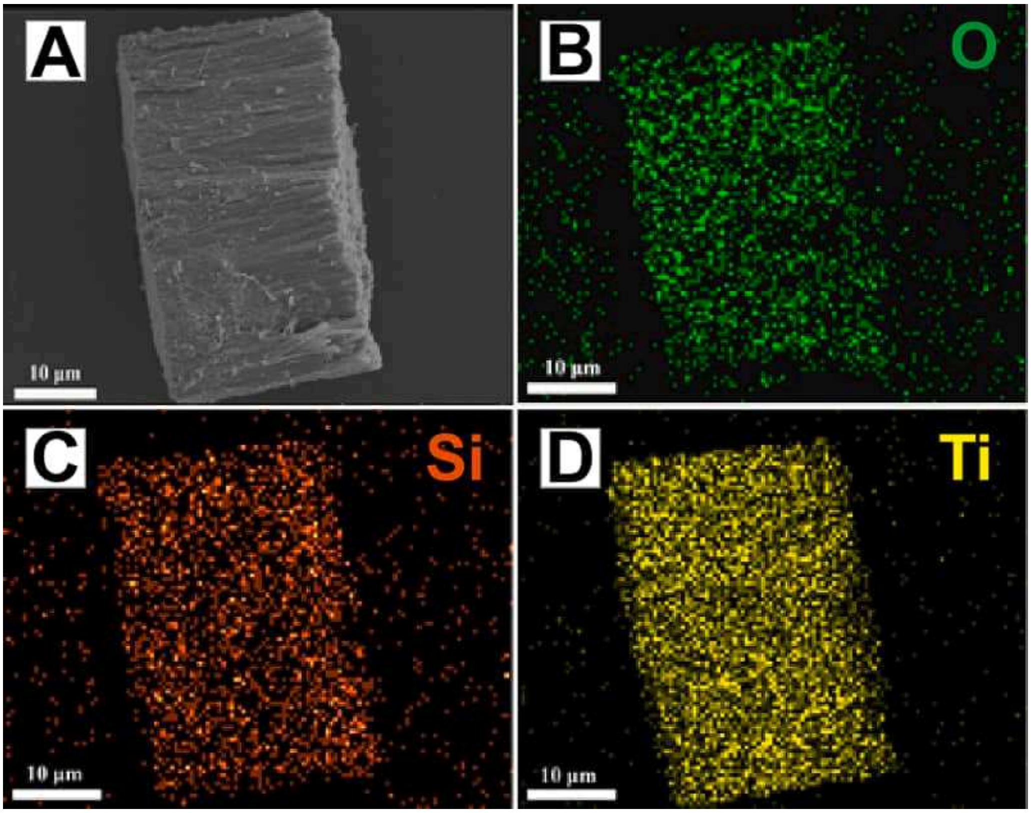

Fig. 4. SEM image of cross-sectional PDMS-Pd/TiO_2 NTs (A) and the EDX mapping of O (B), Si (C) and Ti (D).

of H_2 gas, and then decreases to its initial values when H_2 gas is released. The mechanisms of SMO-based gas sensor have been reported elsewhere [35–37]. In our experiment, the current change is induced by the adsorption/desorption reaction between H_2 and chemisorbed oxygen ions (O_2- at room temperature) on the surface of TiO_2 NTs, and Pd NPs act as both chemical and electronic sensitizer that enable the detection be performed at room temperature. However, this kind of room-temperature gas sensor is easily influenced by RH, because the

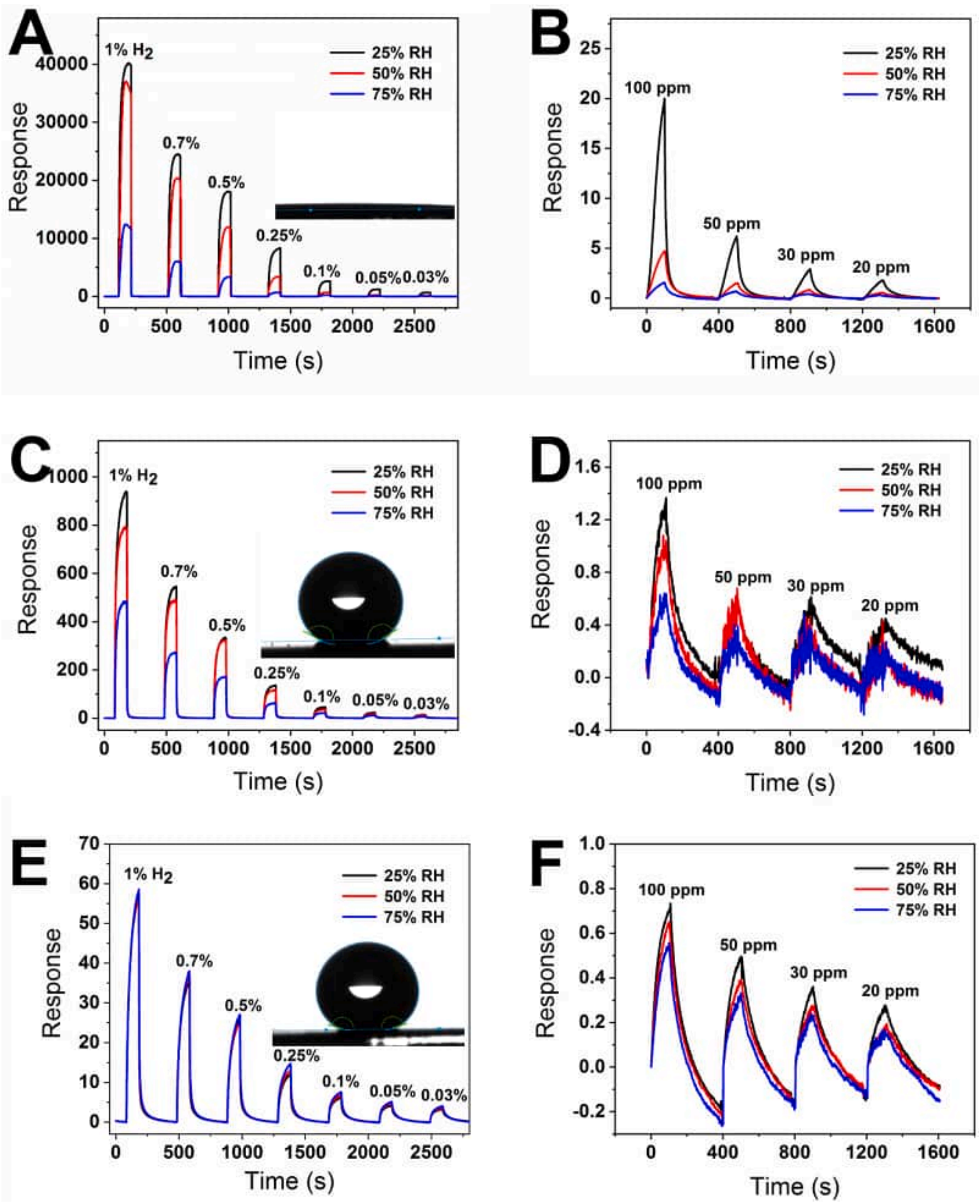

Fig. 5. The real-time responses of pristine Pd/TiO_2 NTs (A and B), Pd/TiO_2 NTs coated by PDMS for 3 h (C and D) and Pd/TiO_2 NTs coated by PDMS for 7 h (E and F) to different concentrations of H_2 under different RH environments at 25 °C. The insets of (A), (C), and (E) show water droplet on each surface.

water molecules can occupy the active sites on the sensor surface and thereby hinder the whole sensing process described above.

Fig. 5A and 5B show the hydrogen sensing response of pristine Pd/TiO_2 NTs under different RH environments (the real-time current variation with respect to H_2 concentration are shown as Figure S5A and S5B). As expected, the sensor response displays a significant decrease, along with the increase of recovery time (Figure S6A and S6B), when the RH of surrounding environment increases. For example, compared with the response value at 25% RH, the response values to 100 ppm H_2 retain only ~25% and ~10%, respectively, when RH of surrounding environment increases to 50% and 75%. This kind of dramatic fluctuation of sensor response with respect to the change of RH makes the reliably quantitative detection of target gas rather difficult in practical applications.

When it comes to PDMS-Pd/TiO_2 NTs, it is interesting to notice although these samples are all superhydrophobic, their resistance to RH are totally different. As for Pd/TiO_2 NTs coated by PDMS for 3 h, it shows no obvious improvements in humidity resistance compared with pristine Pd/TiO_2 NTs in the high H_2 concentration range (Fig. 5C). However, it does reveal some improvements in the low H_2 concentration range (Fig. 5D). For example, the response value to 100 ppm H_2 at 75% RH is ~42% of that at 25% RH, which is higher than the pristine Pd/TiO_2 NTs. In contrast, when PDMS deposition time is prolonged to 7 h, the obtained sample shows excellent humidity resistance (as shown in Fig. 5E-F, S5C-D and S6C-D). RH reveals very limited interference to the response value and response/recovery time in a wide range of H_2 concentrations. The humidity-resistive sensing performance of Pd/TiO_2 NTs coated by PDMS for 7 h is further proved by Figure S7, which shows the variation ratio of base current (or resistance) is lower than 20% when RH of surrounding environment increases from 25% to 75%. With respect to the pristine sample, the variation ratio of base current is more than 150%. The variation of the base current (or resistance) of sample with RH can be attributed to the combined effects from reduced O_2 adsorption and the surface hydroxylation of TiO_2 by water molecules

adsorption [9,10], and our experimental results show coating a conformal PDMS layer definitely suppresses the influence of RH through reducing the water molecule adsorption.

Based on these phenomena, it can also be concluded that surface hydrophobicity is a necessary, but certainly not a sufficient condition for humidity-resistive gas sensors. A superhydrophobic surface can resist water droplet very well, but it may not keep out water molecules in surrounding environments. At the same time, the amount or the thickness of PDMS is an important factor. The influence of RH can be eliminated only when PDMS layer reaches an enough thickness so that it covers all of the sensor surfaces.

To further convince this conclusion, the sensing performances of PFS and HTS modified Pd/TiO_2 NTs under different RH were characterized. Silane coupling agents have been widely used to modify the wettability of a solid surface [38,39]. As shown in Fig. 6, after modified with PFS and HTS, the CA of Pd/TiO_2 NTs increased to 167° and 150°, respectively. PFS modified Pd/TiO_2 NTs have a superior hydrophobicity due to the lower surface energy of F-based functional groups. However, as aforementioned, simply altering the surface wettability from superhydrophilic to superhydrophobic can not endow gas sensor with a good humidity resistance (as shown in Fig. 6 and S8). The response value of PFS (or HTS) modified Pd/TiO_2 NTs to 100 ppm H_2 at 75% RH is ~24% of that at 25% RH. Additionally, the variation ratio of base current is larger than 50% when RH of surrounding environment increases from 25% to 75% (Figure S7 and S9). The steric hindrance prevents silane coupling agents forming a complete passivation monolayer to keep out the adsorption of water molecular on the gas sensor, which therefore is not able to completely eliminate the influence of RH.

Besides perfect humidity-resist property, PDMS has a better chemical stability than other commonly used organic modification layers, such as silane coupling agents, as a result of its unique backbone structure (-Si-O-Si-). The superior chemical stability of PDMS is of particularly importance to SMO-based sensors because most SMOs are perfect photocatalyst so that organic layers on them are easily decomposed during daily usage and lose their functionalities. To characterize the chemical stability of PDMS, PDMS-Pd/TiO_2 NTs were irradiated by UV light (xenon lamp, intensity of 1.35 W/cm^2), and the CA variation of the sample during the irradiation process was measured. UV irradiation accelerates the aging and degradation process of PDMS on TiO_2, ensuring the chemical stability can be characterized in a short time. At the same time, other organic modification layers such as PFS, HTS were selected as references. Fig. 7 shows CA of samples modified with HTS decreases from 151° to ~10° after UV irradiation for 1 h, indicating HTS is easily decomposed on TiO_2 surfaces. PFS has a slightly better stability, the CA of PFS modified samples decreases from 167° to 137° and 33° after UV irradiation for 1 h and 2 h, respectively. Compared with silane coupling agents, PDMS shows a much better resistance to the photodegradation by TiO_2. For PDMS-Pd/TiO_2 NTs, there is only a slight decrease of CA after UV irradiation, from initial 162° to 155° (irradiation time of 1 h) and152° (irradiation time of 2 h), implying PDMS is more stable and more suitable for functionalizing SMO-based devices for practical applications.

For most SMO-based gas sensors, room-temperature sensing abilities are achieved by the decoration of catalyst NPs (such as Ag, Pd and Pt), and their sensing performances have been demonstrated to be greatly influenced by the dispersion and size distribution of decorated catalyst NPs on the SMO surface [37,40,41]. Therefore, to achieve long-term stability, it also requires the decorated catalyst NPs to be highly stable. However, in practical applications, the sensing performance of gas sensors degrades with time, because catalyst NPs tend to aggregate and detach from the SMO as time goes by, or easily be poisoned by weakly-bonded contaminants so that lose their catalyst ability [42–44]. For example, Fig. 8A and S10A shows the sensing performance of pristine Pd/TiO_2 NTs to 500 ppm H_2 gas under a constant RH decreases

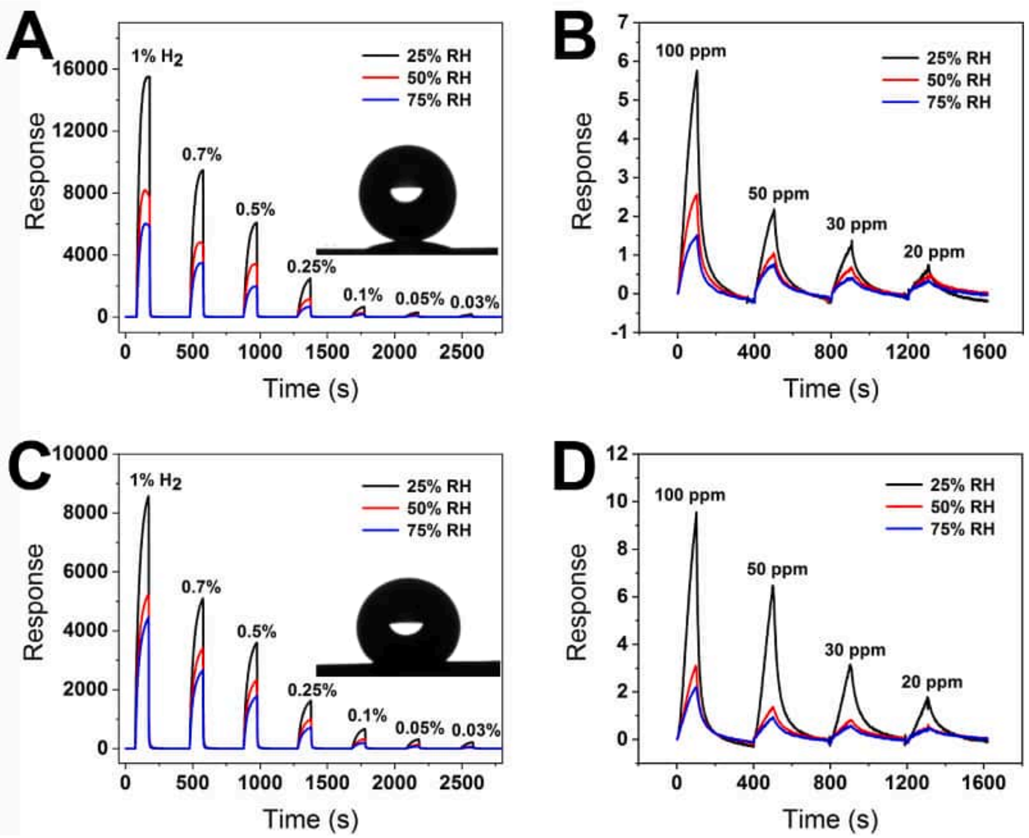

Fig. 6. The real-time response of PFS-Pd/TiO_2 NTs (A and B) and HTS-Pd/TiO_2 NTs (C and D) to different concentrations of H_2 under different RH environments at 25 °C. The insets of (A) and (C) show water droplet on each surface.

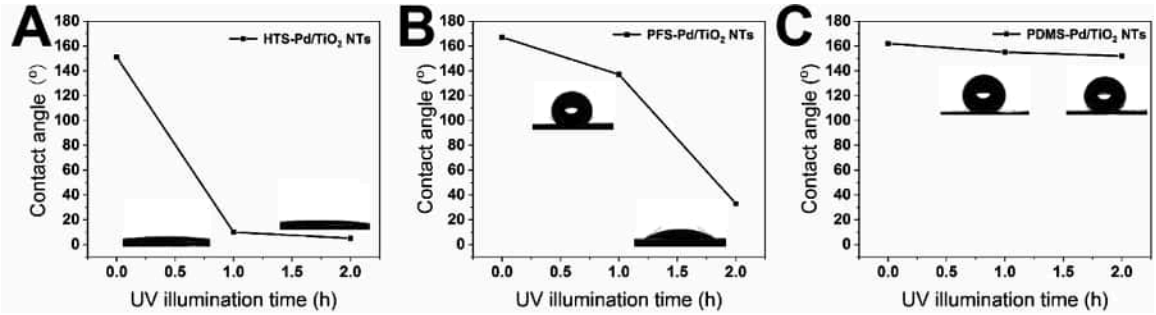

Fig. 7. The contact angle variation of HTS-Pd/TiO_2 NTs (A), PFS-Pd/TiO_2 NTs (B), and PDMS-Pd/TiO_2 NTs (C) under UV illumination. The inset images show water droplet on each surface after 1 h and 2 h UV illumination.

dramatically in 30 days. In sharp contrast, PDMS-Pd/TiO_2 NTs possess stable sensing performance over 2 months (as shown in Fig. 8B, 8C and S10B). Moreover, cyclic test of PDMS-Pd/TiO_2 NTs to 500 ppm H_2 was performed after the sample was aged for 60 days, and the result was given as Fig. 8D. The repeatable and stable response further proves the as-prepared sensors are stable in long-term usage. The improved long-term stability implies PDMS plays a critical role in the stabilization of catalyst NPs by preventing the migration, growth and poisoning of Pd NPs through encapsulation. Similar result was also shown by Huang et al. [24], who demonstrated PDMS layer affords protection to catalyst NPs and improves their long-term stability.

At last, the relationship between the sensitivity and reliability of a gas sensor should be discussed. These two factors are both important parameters to evaluate the sensing performance of gas sensors. High sensitivity means a strong electrical signal for a given analyte concentration. Reliability is a prerequisite to quantitative detection of target gases, which requires the response at a given target-gas concentration should be the same with little degrades with time and little interference by changes in the ambient environment (such as the change of RH). Unluckily, sensitivity and reliability are always restricted by each other, just as we are not able to simultaneously measure the precise position and momentum of a microscopic particle based on the uncertainty principle. That is to say, for most sensors, the more sensitive, the more easily to be interfered by other species or changes in the ambient environment (or the less reliable). As such, these two factors should reach a balance in practical applications. In our experiment, we must admit there is a notable decrease of the sensor response after coated by PDMS layer (this may be due to the PDMS film hinders the contact of H_2 and O_2 with the surface of catalytic Pd NPs and TiO_2 NTs, and the detailed reason for this is still under investigation). However, the response value of PDMS-Pd/TiO_2 NTs to 1% H_2 gas is still much higher than that required by U.S. Department of Energy (according to this requirement, hydrogen sensors should exhibit response larger than 25% to 1% H_2) [45,46]. Moreover, it should also be pointed out PDMS-Pd/TiO_2 NTs can detect H_2 gas with concentration lower than 20 ppm, which is sensitive enough for many applications such as explosive detection and H_2 breath test. Hence, considering the humidity-resistance property and long-term stability, we believe PDMS-Pd/TiO_2 NTs have a better overall

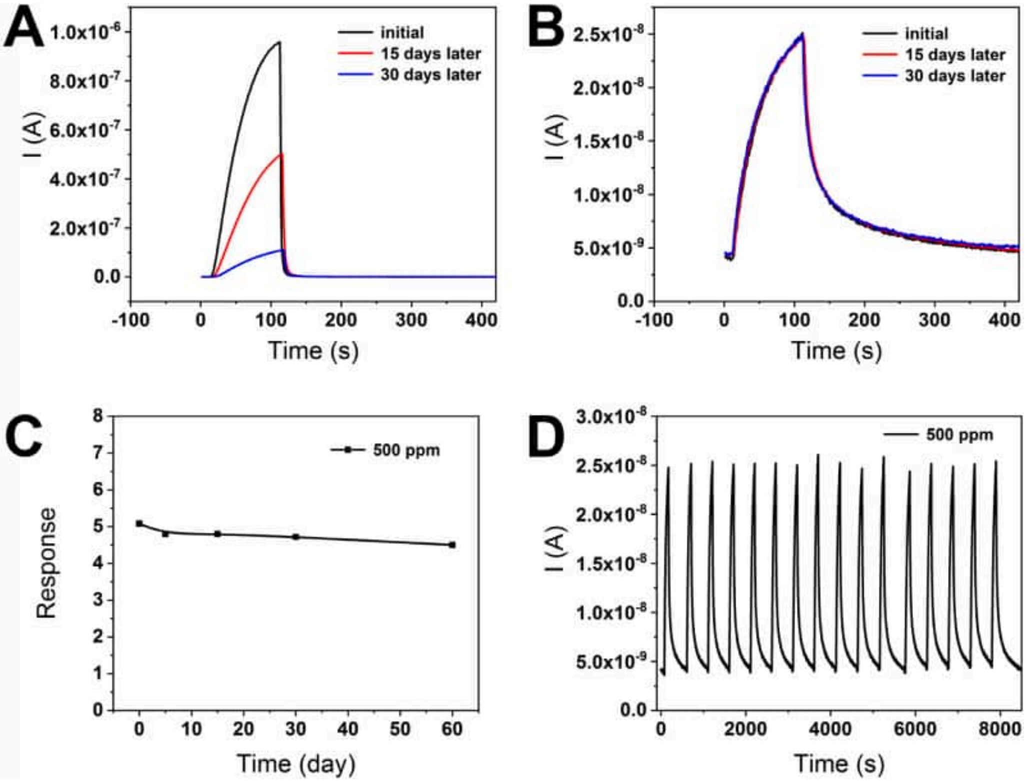

Fig. 8. Characterization of the long-term stability of pristine Pd/TiO_2 NTs (A) and 7 h PDMS-Pd/TiO_2 NTs to 500 ppm H_2 at 25 °C (B, C). D, cyclic test of 7 h PDMS-Pd/TiO_2 NTs to 500 ppm H_2 after the sample was aged for 60 days.

performance than pristine Pd/TiO_2 NTs for practical applications.

The aim of this work is offering a simple and general strategy to improve the humidity resistance and long-term stability of gas sensors through evaporating a layer of PDMS. The effect of PDMS layer can be understood as improving the reliability (including the humidity resistance and long-term stability) at the cost of some response value. We believed this strategy can be applied to room-temperature gas sensors working at ambient environment with variable RH, or other conditions where humidity resistance is more important than response value to achieve a reliable detection.

4. Conclusions

In summary, a simple method to enhance the humidity resistance and long-term stability of room-temperature gas sensor by thermally evaporating PDMS is demonstrated. Pd/TiO_2 NTs that possess a complex nanostructure are selected as a typical gas sensor to demonstrate this method has high controllability and flexibility, and can be extended to gas sensors with variable morphologies. The sensor response of pristine Pd/TiO_2 NTs decreases with the increase of RH, retaining only ~10% response value at 75% RH compared with that at 25% RH. In contrast, at optimized condition, PDMS-Pd/TiO_2 NTs reveal perfect humidity resistance. Experimental results show the thickness of PDMS layer plays an important role. PDMS layer with insufficient thickness or other organic SAM layer are not able to make gas sensors humidity resistive in despite of their superhydrophobic nature, indicating a superhydrophobic surface is not a sufficient condition for humidity-resistive gas sensors. In the meanwhile, we show PDMS has a superior resistance to the photodegradation by TiO_2 than other organic modification layers, which ensures their functionality can be well retained for a long time. Furthermore, PDMS is found to afford protection to catalyst NPs on TiO_2 NTs, which also improves the long-term stability of gas sensor. We believe the PDMS coating method offers a simple yet powerful strategy for designing various SMO-based room-temperature gas sensors with high humidity resistance and long-term stability.

CRediT authorship contribution statement

Zhimin Gao: Methodology, Investigation, Data curation. **Guoshuai Song:** Validation, Formal analysis. **Xuemin Zhang:** Conceptualization, Supervision, Writing - original draft. **Qian Li:** Resources, Formal analysis. **Shuang Yang:** Resources, Formal analysis. **Tieqiang Wang:** Writing - original draft. **Yunong Li:** Funding acquisition. **Liying Zhang:** Funding acquisition. **Lei Guo:** Writing - review & editing. **Yu Fu:** Writing - review & editing.

Declaration of Competing Interest

The authors report no declarations of interest.

Acknowledgements

The authors show their gratitude to Prof. Yang Li at Jilin University for helpful discussions. This work was supported by the National Natural Science Foundation of China (21503037, 21805029, 21805028), the Fundamental Research Funds for the Central Universities (N170503010, N180504005, N180504007), and Open Project of State Key Laboratory of Supramolecular Structure and Materials (sklssm202042, sklssm202008, sklssm202027). Special thanks are due to the instrumental analysis from analytical and testing center, Northeastern University.

Appendix A. Supplementary data

Supplementary material related to this article can be found, in the online version, at doi:https://doi.org/10.1016/j.snb.2020.128810.

References

[1] D.W. Wang, Z.W. Li, J. Zhou, H. Fang, X. He, P. Jena, J.B. Zeng, W.N. Wang, Simultaneous detection and removal of formaldehyde at room temperature: Janus Au@ZnO@ZIF-8 nanoparticles, Nano-Micro Lett. 10 (2018) 4.
[2] D.J. Wales, J. Grand, V.P. Ting, R.D. Burke, K.J. Edler, C.R. Bowen, S. Mintova, A. D. Burrows, Gas sensing using porous materials for automotive applications, Chem. Soc. Rev. 44 (2015) 4290–4321.
[3] S.J. Kim, H.J. Koh, C.E. Ren, O. Kwon, K. Maleski, S.Y. Cho, B. Anasori, C.K. Kim, Y. K. Choi, J. Kim, Y. Gogotsi, H.T. Jung, Metallic $Ti_3C_2T_x$ Mxene gas sensors with ultrahigh signal-to-noise ratio, ACS Nano. 12 (2018) 986–993.
[4] T.A. Blank, L.P. Eksperiandova, K.N. Belikov, Recent trends of ceramic humidity sensors development: a review, Sens. Actuators B Chem. 228 (2016) 416–442.
[5] R. Kumar, O. Al-Dossary, G. Kumar, A. Umar, Zinc oxide nanostructures for NO_2 gas–sensor applications: a review, Nano-Micro Lett. 7 (2015) 97–120.
[6] G. Peng, U. Tisch, O. Adams, M. Hakim, N. Shehada, Y.Y. Borza, S. Billan, R. Abdah-Bortnyak, A. Kuten, H. Haick, Diagnosing lung cancer in exhaled breath using gold nanoparticles, Nat. Nanotechnol. 4 (2009) 669–673.
[7] R.A. Potyrailo, Multivariable sensors for ubiquitous monitoring of gases in the era of internet of things and industrial internet, Chem. Rev. 116 (2016) 11877–11923.
[8] Z.Y. Zhao, M. Knight, S. Kumar, E.T. Eisenbraun, M.A. Carpenter, Humidity effects on Pd/Au-based all-optical hydrogen sensors, Sens. Actuators B Chem. 129 (2008) 726–733.
[9] Y.G. Song, Y.S. Shim, J.M. Suh, M.S. Noh, G.S. Kim, K.S. Choi, B. Jeong, S. Kim, H. W. Jang, B.K. Ju, C.Y. Kang, Ionic-Activated chemiresistive gas sensors for room-temperature operation, Small 15 (2019), 1902065.
[10] O. Lupan, V. Postica, T. Pauporté, B. Viana, M.I. Terasa, R. Adelung, Room temperature gas nanosensors based on individual and multiple networked Au-modified ZnO nanowires, Sens. Actuators B Chem. 299 (2019), 126977.
[11] J. van den Broek, A.T. Güntner, S.E. Pratsinis, Highly selective and rapid breath isoprene sensing enabled by activated alumina filter, ACS Sens. 3 (2018) 677–683.
[12] H.J. Kim, J.H. Lee, Highly sensitive and selective gas sensors using p-type oxide semiconductors: Overview, Sens. Actuators B Chem. 192 (2014) 607–627.
[13] M.E. Franke, T.J. Koplin, U. Simon, Metal and metal oxide nanoparticles in chemiresistors: does the nanoscale matter? small 2 (2006) 36–50.
[14] S. Lee, W. Kim, K. Yong, Overcoming the water vulnerability of electronic devices: a highly water-resistant ZnO nanodevice with multifunctionality, Adv. Mater. 23 (2011) 4398–4402.
[15] X.M. Zhang, J.H. Zhang, Z.Y. Ren, X. Zhang, T. Tian, Y.N. Wang, F.X. Dong, B. Yang, Photoinduced cleaning of water-soluble dyes on patterned superhydrophilic/superhydrophobic substrates, Nanoscale 2 (2010) 277–281.
[16] W. Liu, L. Xu, K. Sheng, C. Chen, X.Y. Zhou, B. Dong, X. Bai, S. Zhang, G.Y. Lu, H. W. Song, APTES-functionalized thin-walled porous WO_3 nanotubes for highly selective sensing of NO_2 in a polluted environment, J. Mater. Chem. A 6 (2018) 10976–10989.
[17] C. Struzzi, M. Scardamaglia, J. Casanova-Chafer, R. Calavia, J.F. Colomer, A. Kondyurin, M. Bilek, N. Britun, R. Snyders, E. Llobet, C. Bittencourt, Exploiting sensor geometry for enhanced gas sensing properties of fluorinated carbon nanotubes under humid environment, Sens. Actuators B Chem. 281 (2019) 945–952.
[18] K. Hassan, G.S. Chung, Fast and reversible hydrogen sensing properties of Pd-capped Mg ultra-thin films modified by hydrophobic alumina substrates, Sens. Actuators B Chem. 242 (2017) 450–460.
[19] J. Wang, P. Yang, X.W. Wei, High-performance, room-temperature, and no-humidity-impact ammonia sensor based on heterogeneous nickel oxide and zinc oxide nanocrystals, ACS Appl. Mater. Interfaces 7 (2015) 3816–3824.
[20] T. Lim, Y. Kang, S.M. Jeong, S. Ju, Thermally nonreactive and chemically reactive metal-oxide nanowire transistor covered with aerogel-microsphere-thin-film based selective filter, Mater. Res. Express 5 (2018), 116402.
[21] S. Wooh, N. Encinas, D. Vollmer, H.J. Butt, Stable hydrophobic metal-oxide photocatalysts via grafting polydimethylsiloxane brush, Adv. Mater. 29 (2017), 1604637.
[22] W. Zhang, Y.L. Hu, J. Ge, H.L. Jiang, S.H. Yu, A facile and general coating approach to moisture/water-resistant metal-organic frameworks with intact porosity, J. Am. Chem. Soc. 136 (2014) 16978–16981.
[23] J.K. Yuan, X.G. Liu, O. Akbulut, J.Q. Hu, S.L. Suib, J. Kong, F. Stellacci, Superwetting nanowire membranes for selective absorption, Nat. Nanotechnol. 3 (2008) 332–336.
[24] G. Huang, Q.H. Yang, Q. Xu, S.H. Yu, H.L. Jiang, Polydimethylsiloxane coating for a palladium/MOF composite: highly improved catalytic performance by surface hydrophobization, Angew. Chem. Int. Ed. 55 (2016) 7379–7383.
[25] C.L. Xiang, Z. She, Y.J. Zou, J. Cheng, H.L. Chu, S.J. Qiu, H.Z. Zhang, L.X. Sun, F. Xu, A room-temperature hydrogen sensor based on Pd nanoparticles doped TiO_2 nanotubes, Ceram. Int. 40 (2014) 16343–16348.
[26] O.K. Varghese, D.W. Gong, M. Paulose, K.G. Ong, E.C. Dickey, C.A. Crimes, Extreme changes in the electrical resistance of titania nanotubes with hydrogen exposure, Adv. Mater. 15 (2003) 624–627.
[27] Q.Y. Wang, X.C. Yang, X.L. Wang, M. Huang, J.W. Hou, Synthesis of N-doped TiO_2 mesosponge by solvothermal transformation of anodic TiO_2 nanotubes and enhanced photoelectrochemical performance, Electrochimica Acta 62 (2012) 158–162.
[28] X.F. Li, T.L. Cao, X.M. Zhang, Y.H. Sang, L.Q. Yang, T.Q. Wang, Y.N. Li, L.Y. Zhang, L. Guo, Y. Fu, Characterization and optimization of the H_2 sensing performance of Pd hollow shells, Sens. Actuators B Chem. 295 (2019) 101–109.
[29] Y.H. Sang, G.S. Song, Z.M. Gao, X.M. Zhang, T.Q. Wang, Y.N. Li, L.Y. Zhang, Y. Fu, L. Guo, Sea urchin-like CuO particles prepared using $Cu_3(PO_4)_2$ flowers as

precursor for high performance ethanol sensing, Nanotechnology 31 (2020), 165504.
[30] J.W. Krumpfer, T.J. McCarthy, Rediscovering silicones: "unreactive" silicones react with inorganic surfaces, Langmuir 27 (2011) 11514–11519.
[31] E.J. Park, Y.K. Cho, D.H. Kim, M.G. Jeong, Y.H. Kim, Y.D. Kim, Hydrophobic polydimethylsiloxane (PDMS) coating of mesoporous silica and its use as a preconcentrating agent of gas analytes, Langmuir 30 (2014) 10256–10262.
[32] M.G. Jeong, H.O. Seo, K.D. Kim, D.H. Kim, Y.D. Kim, D.C. Lim, Quenching of photocatalytic activity and enhancement of photostability of ZnO particles by polydimethylsiloxane coating, J Mater Sci 47 (2012) 5190–5196.
[33] G.M. Ingo, S. Dirè, F. Babonneau, XPS studies of SiO_2-TiO_2 powders prepared by sol-gel process, Appl. Surf. Sci. 70 (1993) 230–234.
[34] X.J. Feng, L. Jiang, Design and creation of superwetting/antiwetting surfaces, Adv. Mater. 18 (2006) 3063–3078.
[35] S.T. Ren, W.J. Liu, One-step photochemical deposition of PdAu alloyed nanoparticles on TiO_2 nanowires for ultra-sensitive H_2 detection, J. Mater. Chem. A 4 (2016) 2236–2245.
[36] A. Kolmakov, D.O. Klenov, Y. Lilach, S. Stemmer, M. Moskovits, Enhanced gas sensing by individual SnO_2 nanowires and nanobelts functionalized with Pd catalyst particles, Nano Lett. 5 (2005) 667–673.
[37] C.M. Chang, M.H. Hon, I.C. Leu, Outstanding H_2 sensing performance of Pd nanoparticle-decorated ZnO nanorod arrays and the temperature-dependent sensing mechanisms, ACS Appl. Mater. Interfaces 5 (2013) 135–143.
[38] X.M. Zhang, J.H. Zhang, Z.Y. Ren, X. Li, X. Zhang, D.F. Zhu, T.Q. Wang, T. Tian, B. Yang, Morphology and wettability control of silicon cone arrays using colloidal lithography, Langmuir 25 (2009) 7375–7382.
[39] Y. Li, L. Li, J.Q. Sun, Bioinspired self-healing superhydrophobic coatings, Angew. Chem. Int. Ed. 49 (2010) 6129–6133.
[40] I.S. Hwang, J.K. Choi, H.S. Woo, S.J. Kim, S.Y. Jung, T.Y. Seong, I.D. Kim, J.H. Lee, Facile control of C_2H_5OH sensing characteristics by decorating discrete Ag nanoclusters on SnO_2 nanowire networks, ACS Appl. Mater. Interfaces 3 (2011) 3140–3145.
[41] H.J. Cho, V.T. Chen, S.P. Qiao, W.T. Koo, R.M. Penner, I.D. Kim, Pt-functionalized PdO nanowires for room temperature hydrogen gas sensors, ACS Sens. 3 (2018) 2152–2158.
[42] E. Brauns, E. Morsbach, S. Kunz, M. Bäumer, W. Lang, A fast and sensitive catalytic gas sensors for hydrogen detection based on stabilized nanoparticles as catalytic layer, Sens. Actuators B Chem. 193 (2014) 895–903.
[43] J.Y. Luo, L. Gong, H.D. Tan, S.Z. Deng, N.S. Xu, Q.G. Zeng, Y. Wang, Study of the catalyst poisoning and reactivation of Pt nanoparticles on the surface of WO_3 nanowire in gasochromic coloration, Sens. Actuators B Chem 171–172 (2012) 1117–1124.
[44] Y. Zhang, J.Q. Xu, P.C. Xu, Y.H. Zhu, X.D. Chen, W.J. Yu, Decoration of ZnO nanowires with Pt nanoparticles and their improved gas sensing and photocatalytic performance, Nanotechnology 21 (2010), 285501.
[45] Office of Energy Efficiency and Renewable Energy (EERE), The Department of Energy Funding Opportunity Announcement: DEPS36-09GO99004, The Department of Energy, Golden, CO, 2009.
[46] X.W. Li, Y. Liu, J.C. Hemminger, R.M. Penner, Catalytically activated palladium@platinum nanowires for accelerated hydrogen gas detection, ACS Nano 9 (2015) 3215–3225.

Zhimin Gao is currently carrying out her academic research for her Master's degree under the guidance of Prof. Xuemin Zhang. Her research mainly focuses on TiO_2 NTs based gas sensors.

Guoshuai Song is currently carrying out his academic research for his Doctor's degree under the guidance of Prof. Fu Yu. His research direction mainly focuses on CuO based gas sensors.

Xuemin Zhang is an associate professor at the Department of Chemistry at the College of Sciences, Northeastern University (China). He received his PhD degree from Jilin University (China) in 2012. His research focuses on the Plasmonics, optical and electronic sensors.

Qian Li is currently carrying out her academic research for her Master's degree under the guidance of Prof. Xuemin Zhang. Her research mainly focuses on gas sensors.

Shuang Yang is currently carrying out her academic research for her Master's degree under the guidance of Prof. Xuemin Zhang. Her research mainly focuses on gas sensors.

Tieqiang Wang is an associate professor at the Department of Chemistry at the College of Sciences, Northeastern University (China). He received his PhD degree from Jilin University (China) in 2013. His research focuses on the Plasmonic sensors.

Yunong Li is a lecture at the Department of Chemistry at the College of Sciences, Northeastern University (China). She received his PhD degree from Nankai University (China) in 2014. Her research focuses on the preparation of hybrid materials for CO_2 chemistry.

Liying Zhang is a lecture at the Department of Chemistry at the College of Sciences, Northeastern University (China). She received her PhD degree from Jilin University (China) in 2017. Her research focuses on the modification of MOF nanosheets.

Lei Guo is a postdoctoral at the Texas A&M Institute of Biosciences & Technology, Houston. He received his PhD degree from Xiamen University (China) in 2014. His research focuses on the biosensors.

Fu Yu is a professor at the Department of Chemistry at the College of Sciences, Northeastern University (China). He received his PhD degree from Jilin University (China) in 2003. His research focuses on the 2D MOF materials.

RESEARCH ARTICLE

ADVANCED FUNCTIONAL MATERIALS
www.afm-journal.de

Flexible, Breathable and Hydrophobic SnO_2–SnS_2–SiO_2/SiO_2 All-Inorganic Self-Supporting Nanofiber Membrane for Ultralow-Concentration NO_2 Sensing Under High Humidity

Jia Liu, Jinniu Zhang, Qian Yu, Yumeng Liu, Xinlei Zhang, Gangqiang Zhu, Yanmin Jia, Hongbing Lu, Jianzhi Gao,* Hongjun Wang,* and Benpeng Zhu**

Inorganic semiconductor gas sensors, being widely utilized in gas-sensing applications, face significant challenges in attaining mechanical flexibility and humidity resistance in wearable sensing fields. Herein, a highly flexible, breathable, and hydrophobic all-inorganic self-supporting nanofiber (NF) gas sensor is developed using electrospinning combined with thermal sulfidation approach. This innovative sensor features a bilayer configuration, with an amorphous SiO_2 nanofiber substrate layer and an interwoven SiO_2 and SnO_2–SnS_2 nanofiber active layer. The relatively low elastic modulus of the amorphous SiO_2 nanofibers, combined with the three-dimensional network interwoven structure, endow the SnO_2–SnS_2–SiO_2/SiO_2 sensor with superior mechanical flexibility. The sensor exhibits excellent sensitivity, selectivity, moisture resistance, and cycling stability (>10 000 cycles at 140° bending) to both high and low concentration NO_2. Notably, an excellent flexible detecting capability of the sensor to NO_2, an asthma-related biomarker, is demonstrated at ultralow concentrations (≈25 ppb) in simulated exhaled breath environments. The enhanced moisture resistance is attributed to the effective inhibition of hydrogen bond formation from H_2O molecules by the Sn—S bonds formed through sulfidation of SnO_2 nanofibers. This work represents a substantial advancement in the universal fabrication of flexible, breathable and moisture-resistant inorganic semiconductor sensors for wearable breath sensing applications.

1. Introduction

Resistive metal oxide semiconductor (MOS) gas sensors are extensively used for gas concentration monitoring owing to their low cost, high sensitivity, and exceptional stability.[1] A tremendous demand for MOS gas sensors is being generated by the rapidly expanding markets of wearable sensing, portable environmental monitoring, and medical pre-diagnostic devices.[2] Unfortunately, conventional MOS gas sensors are unsuitable for the aforementioned applications due to their bulky and rigid structure. In light of this, the development of MOS gas sensors with good flexibility, good air permeability, and high gas-sensing performance, is urgently demanded. Presently, the fabrication of flexible MOS gas sensors predominantly involves coating MOS sensing materials onto flexible substrates with low elastic moduli, such as polymers,[3] paper,[4] and organic fiber membranes.[5] Nevertheless, the substantial intrinsic stiffness of the MOS sensing layer and its weak bonding to the organic substrate frequently result in delamination

J. Liu, Q. Yu, Y. Liu, X. Zhang, G. Zhu, Y. Jia, H. Lu, J. Gao
School of Physics and Information Technology
Shaanxi Normal University
Xi'an 710062, China
E-mail: hblu@snnu.edu.cn; jianzhigao@snnu.edu.cn

J. Zhang
School of Science
Xi'an University of Posts and Telecommunications
Xi'an 710121, China

H. Wang
Department of Physics
Shaanxi University of Science and Technology
Xi'an 710021, China
E-mail: wanghongjun@sust.edu.cn

B. Zhu
School of Integrated Circuit
Wuhan National Laboratory for Optoelectronics
Huazhong University of Science and Technology
Wuhan 430074, China
E-mail: benpengzhu@hust.edu.cn

The ORCID identification number(s) for the author(s) of this article can be found under https://doi.org/10.1002/adfm.202410833

DOI: 10.1002/adfm.202410833

© 2024 Wiley-VCH GmbH

during bending. Additionally, achieving optimal sensing performance of MOS gas sensors typically requires high-temperature aging treatments (300–400 °C) to enhance both stability and crystallinity.[6] Organic substrates, due to their low glass transition temperatures and higher coefficients of thermal expansion,[2,7] are unable to withstand such elevated sensing aging temperatures. Moreover, the dense and plate-like organic substrates exhibit poor gas permeability, hindering the exchange of gas molecules and moisture. This limitation not only impedes the gas sensing reactions but also leads to potential discomfort and skin irritation in wearable applications.[8] Therefore, it is imperative to develop novel approaches for the fabrication of flexible MOS sensors with high flexibility, good thermostability and high breathability.

Flexible gas sensors have promising applications in the biomedical field, particularly in detecting the concentration of biomarker gases in exhaled human breath, which enables painless and non-invasive health evaluations. A well-documented correlation exists between the concentrations of chemical vapors in exhaled breath and various pathological conditions, such as asthma, lung cancer, kidney disease, and diabetes.[9] Specifically, NO_x levels in exhaled breath exceeding 25 ppb are indicative of asthma disease.[9a,10] It has been reported that there are over 800 compounds in human exhaled breath, with certain compounds closely linked to the metabolic processes in the human body.[11] A major challenge for gas sensors in exhaled breath analysis arises from the high relative humidity (RH), which leads to the adsorption of a substantial number of H_2O molecules on the surface of the sensing material. These adsorbed H_2O molecules can induce proton conductivity and inhibit gas-sensing reactions, both of which reduce the gas-sensing performance.[12] In the domain of non-flexible gas sensing, some strategies have been investigated to mitigate the impact of humidity fluctuations on the performance of MOS materials. Approaches such as Sb,[13] Rh,[14] Tb,[15] and Pr[16] atom doping, as well as the loading of Pd,[17] CuO,[18] and CeO_2[19] nanoparticles, have been introduced into MOS materials due to their catalytic properties for water molecules. However, achieving the desired catalytic activity with these introduced species often requires operating temperatures above 300 °C, posing challenges for room temperature applications. Some researchers[6c,20] have developed MOS sensors with enhanced humidity resistance by coating the gas-sensing layer with a hydrophobic overlayer. Although effective, this method introduces a barrier between the sensing layer and the target gas, which delays the gas-sensing reaction at room temperature and requires elevated temperatures to boost gas molecule diffusion rates. An attempt was made by coating a $Ti_3C_2T_x$/ZnO sphere sensing layer on a polyimide flexible substrate for NO_2 detection, achieving a 51.36% response to 100 ppm NO_2 under 90% RH after 1000 bending cycles.[21] Nonetheless, the development of flexible MOS gas sensors capable of sensitive and selective detection of ultralow concentrations of NO_2 (≈25 ppb) under high RH conditions still remains a significant challenge.

In this paper, a highly flexible, breathable, and hydrophobic all-inorganic self-supporting nanofiber (NF) membrane gas sensor is constructed using electrospinning techniques combined with a thermal sulfidation approach. The proposed sensor features a bilayer architecture, with the bottom substrate layer composed of an amorphous SiO_2 NF membrane fabricated via a standard electrospinning technique. The top active layer is an intertwined NF membrane of amorphous SiO_2 NFs and SnO_2–SnS_2 NFs. In situ thermal sulfidation at 400 °C of a two-spinneret electrospun SnO_2–SiO_2 NF layer leads to the formation of a SnO_2–SnS_2 active layer with an n–n heterojunction structure and a hydrophobic surface, contributing to the excellent room-temperature sensing performance of the SnO_2–SnS_2–SiO_2/SiO_2 sensor to ultralow-concentration NO_2 under the high RH of 85%. Drawing from our knowledge in the domain of MOS-based flexible gas sensors, the construction of the all-inorganic and all-nanofiber flexible gas sensor here overcomes the shortcomings of organic substrate-based sensors, offering several distinct advantages: i) the brittleness of crystalline inorganic semiconductors has been attributed to strong chemical bonds and stress accumulation at grain boundaries.[22] In contrast, amorphous inorganic semiconductors exhibit a short-range disordered atomic arrangement with comparatively weaker chemical bonds and lack grain boundaries, thereby resulting in a lower elastic modulus compared to their periodically arranged crystalline counterparts.[22a] This lower elastic modulus of the amorphous SiO_2 NFs, combined with the three-dimensional network interwoven structure, confer superior flexibility to the SnO_2–SnS_2–SiO_2/SiO_2 sensor; ii) amorphous SiO_2 NF substrate exhibits superior thermal stability and a reduced thermal expansion coefficient mismatch with MOS sensing materials (Figure S1, Supporting Information) compared to conventional organic substrates (Table S1, Supporting Information). This compatibility between SiO_2 NFs and MOS sensing materials enables the constructed sensors to withstand high processing temperatures, thereby expanding the range of feasible fabrication techniques and application scenarios for flexible sensors; iii) sensing signal interference from the amorphous SiO_2 NF substrate is negligible due to its low electrical conductivity and outstanding chemical stability; iv) the all-nanofiber three-dimensional network structure imparts high breathability to the sensor, making it suitable for wearable sensing applications; v) the hydrophobic characteristic induced by the sulfidation of SnO_2 NFs endows the sensor with significant potential for detecting ultralow concentration NO_2 in exhaled breath environments. We are confident that the proposed approach for developing breathable and hydrophobic all-inorganic flexible sensors can be broadly applied to other semiconductor-based flexible gas sensors.

2. Results and Discussion

2.1. Characterization of NF Membranes

The fabrication procedure for the flexible self-supporting SnO_2–SnS_2–SiO_2/SiO_2 sensor is depicted in **Figure 1**. Initially, a two-spinneret electrospinning technique was developed to fabricate a hybrid electrospun NF membrane consisting of interwoven Sn—Si precursor functional NF layer and Si precursor NF substrate layer. Then, the hybrid electrospun precursor NF membrane was calcined at 650 °C to produce the SnO_2–SiO_2/SiO_2 NF membrane. Subsequently, thermal sulfidation of SnO_2–SiO_2/SiO_2 membrane was carried out to produce the SnO_2–SnS_2–SiO_2/SiO_2 membrane. Finally, with Au interdigital electrodes deposited on the membrane surface by an ion sputter technique, the flexible self-supporting SnO_2–SnS_2–SiO_2/SiO_2

© 2024 Wiley-VCH GmbH
16163028, 2025, 4, Downloaded from https://advanced.onlinelibrary.wiley.com/doi/10.1002/adfm.202410833 by Nanjing Tech University, Wiley Online Library on [31/01/2025]. See the Terms and Conditions (https://onlinelibrary.wiley.com/terms-and-conditions) on Wiley Online Library for rules of use; OA articles are governed by the applicable Creative Commons License

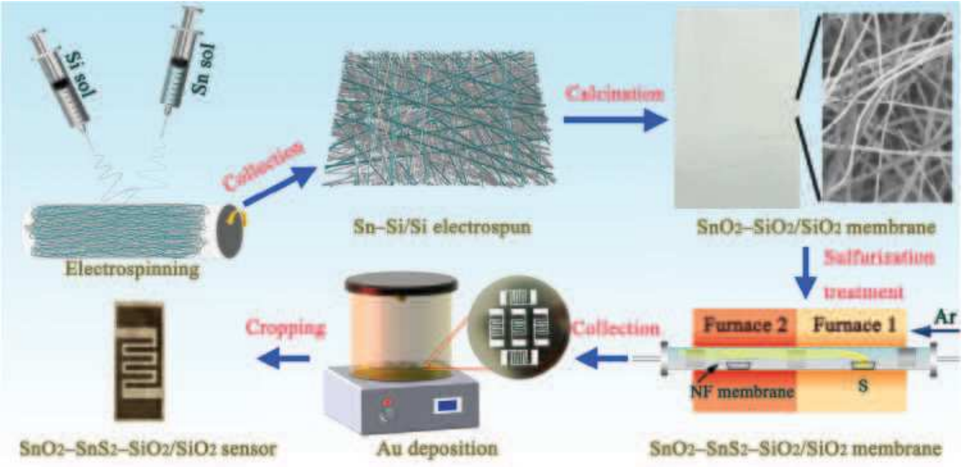

Figure 1. Schematic illustration of the preparation process of flexible SnO_2–SnS_2–SiO_2/SiO_2 sensors.

sensor with superior mechanical flexibility and stability was produced.

Figure 2a displays the photographs of the SnO_2–SiO_2/SiO_2 NF membrane created using the two-spinneret electrospinning approach, exhibiting a smooth surface and self-supporting structure. The remarkable flexibility of the SnO_2–SiO_2/SiO_2 NF membrane is evidenced by its intact state even after undergoing triple folding. A single broad diffraction peak at 22.2° in X-ray diffraction (XRD) pattern (Figure 2b) indicates that the pure SiO_2 NF membrane has an amorphous structure, whereas the hybrid NF membrane derived from Sn─Si/Si precursor NFs using the two-spinneret electrospinning technique contains both amorphous SiO_2 and tetragonal SnO_2 (JCPDS no. 41–1445). The SnO_2–SiO_2/SiO_2 NF membrane comprises two distinct types of ultralong NFs, as illustrated in Figure 2c–e. The NFs with light-color and smooth surface are identified as amorphous SiO_2, while the NFs with dark-color and coarse surface are made up of many tetragonal SnO_2 nanoparticles, as shown by the corresponding high-resolution transmission electron microscopy (HRTEM) image in Figure 2f.

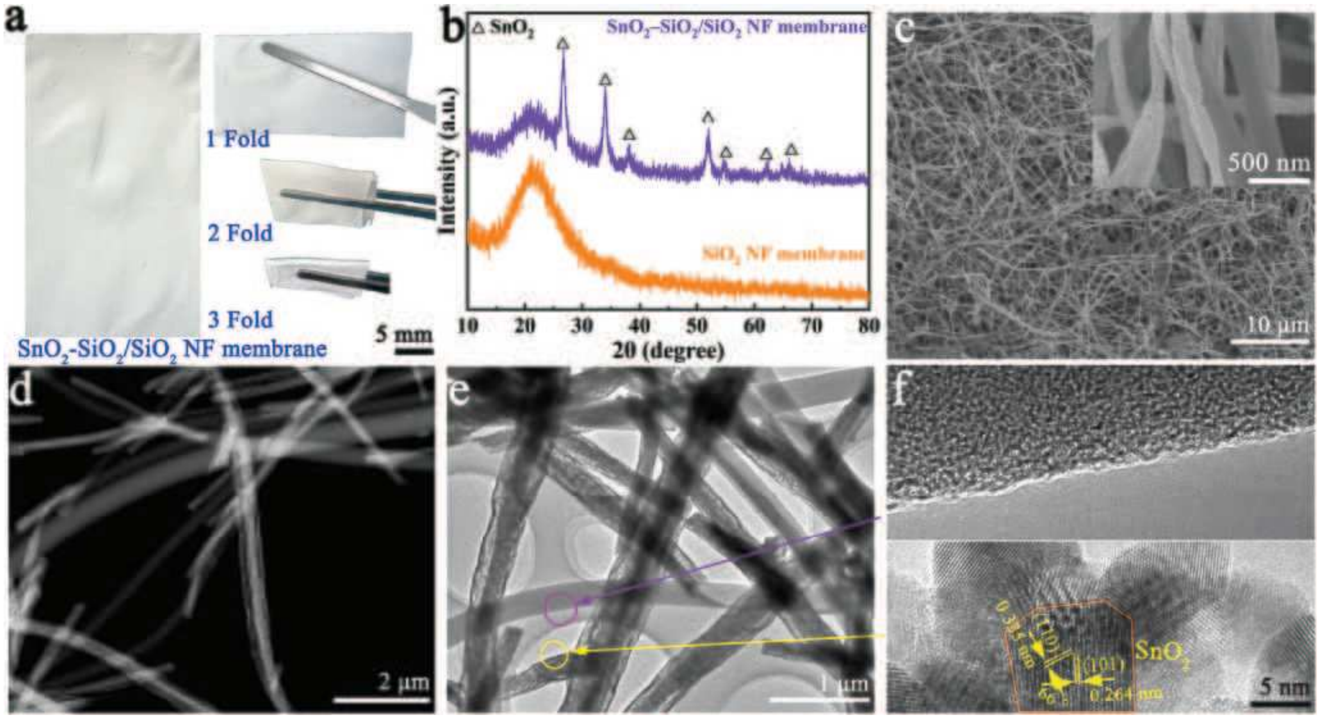

Figure 2. a) Photographs of the flexible SnO_2–SiO_2/SiO_2 NF membranes bent with various folds. b) XRD patterns of SnO_2 and SnO_2–SiO_2/SiO_2 NF membranes. c) Scanning electron microscope (SEM), d) scanning transmission electron microscopy (STEM), and e) transmission electron microscopy (TEM) images of SnO_2–SiO_2/SiO_2 NFs. f) HRTEM images of a single SnO_2 NF and a single SnO_2 NF from SnO_2–SiO_2/SiO_2 NF membrane.

© 2024 Wiley-VCH GmbH

16163028, 2025, 4, Downloaded from https://advanced.onlinelibrary.wiley.com/doi/10.1002/adfm.202410833 by Nanjing Tech University, Wiley Online Library on [31/01/2025]. See the Terms and Conditions (https://onlinelibrary.wiley.com/terms-and-conditions) on Wiley Online Library for rules of use; OA articles are governed by the applicable Creative Commons License

16163028, 2025, 4, Downloaded from https://advanced.onlinelibrary.wiley.com/doi/10.1002/adfm.202410833 by Nanjing Tech University, Wiley Online Library on [31/01/2025]. See the Terms and Conditions (https://onlinelibrary.wiley.com/terms-and-conditions) on Wiley Online Library for rules of use; OA articles are governed by the applicable Creative Commons License

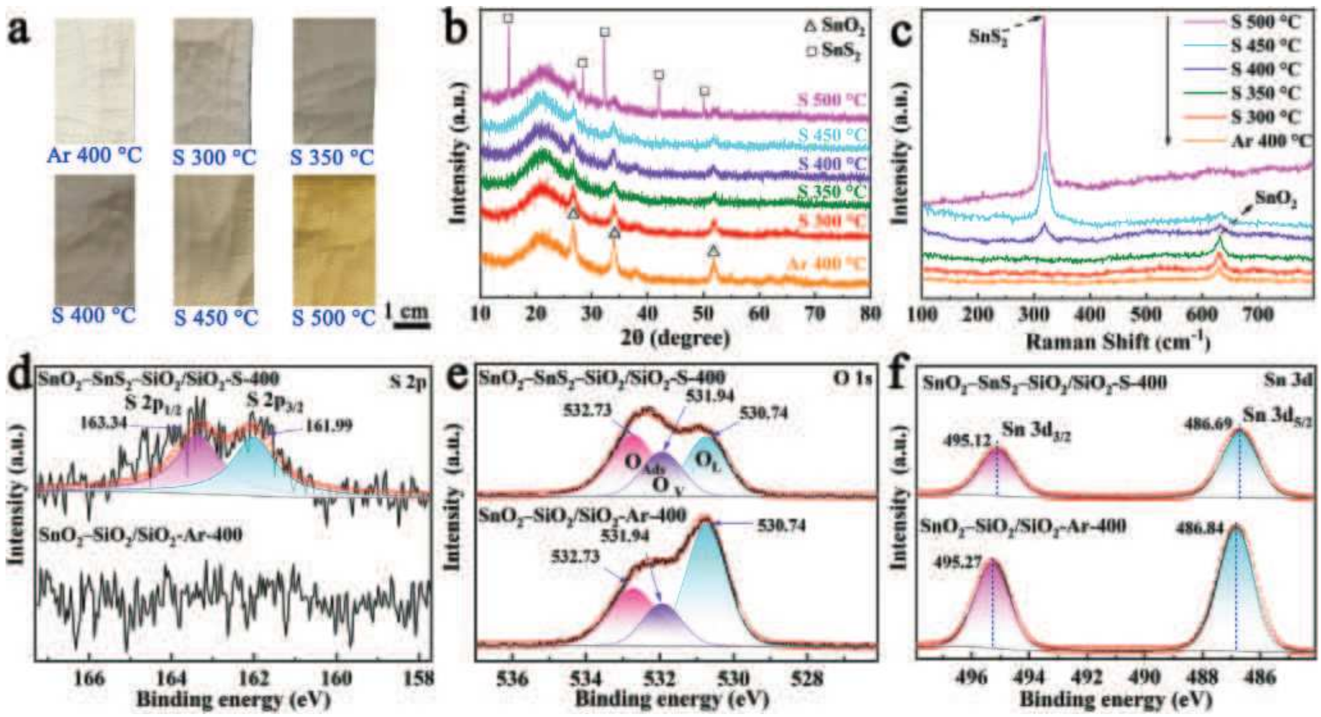

Figure 3. a) Photographs of the various flexible NF membranes prepared by annealing in Ar at 400 °C or thermal sulfidation at various temperatures. b) XRD patterns and c) Raman patterns of various flexible NF membranes. High-resolution XPS spectra of d) S 2p, e) O 1s, and f) Sn 3d of SnO_2–SiO_2/SiO_2-Ar-400 and SnO_2–SnS_2–SiO_2/SiO_2-S-400 NF membranes.

Following sulfidation treatment, the SnO_2–SiO_2/SiO_2 NF membrane maintains a self-supporting structure free of any visible damage (**Figure** 3a). The SnO_2–SiO_2/SiO_2 membrane undergoes a substantial color change through sulfidation at 300 °C, suggesting that a reaction between sulfur and SnO_2 NFs may occur at 300 °C. As the temperature rises, the color gradually transforms from grey to yellow. The membranes of the SiO_2 NFs sulfurized at 400 °C (designated as SiO_2-S-400) (Figure S2, Supporting Information) and the SnO_2–SiO_2/SiO_2 NFs annealed in Ar at 400 °C (designated as SnO_2–SiO_2/SiO_2-Ar-400) retain their original white color. XRD results in Figure 3b show a sequential decrease in SnO_2 peak intensities of the SnO_2–SiO_2/SiO_2 membranes as the sulfidation temperature rises from 300 to 500 °C. Several new diffraction peaks, corresponding to the hexagonal phase SnS_2 (JCPDS no. 23–0677), are detected at the sulfidation temperature of 500 °C. More sensitive structure characterization was further performed by Raman spectra in Figure 3c. The vibrational model at 631 cm^{-1} is associated with the A1g optical modes of SnO_2.[23] An obvious vibrational model at 317 cm^{-1}, which is related with the A1g optical modes of SnS_2, appears at the sulfidation temperature of 400 °C. A sequential decrease in SnO_2 Raman peak intensity and a gradual increase in SnS_2 Raman peak intensity are exhibited with the increase of sulfidation temperature from 300 to 500 °C and from 400 to 500 °C, respectively. These results are consistent with the XRD findings, suggesting that more SnO_2 is transformed into SnS_2 through the sulfidation reaction with increasing sulfidation temperature. The absence of both XRD peaks and Raman vibration modes of SnS_2 at the sulfidation temperatures of 300 and 350 °C is likely due to the low content of SnS_2 phase produced under these conditions. Thermodynamic calculations for the reactions between SnO_2 and gaseous sulfur were performed using HSC Chemistry 6.0 software. The results show that the formation of the SnS_2 phase is thermodynamically favorable within the temperature range of 390–610 °C, as evidenced by the negative Gibbs free energy values (Figure S3, Supporting Information). In contrast, the SnS phase is predicted to form at temperatures exceeding 690 °C.[24]

X-ray photoelectron spectroscopy (XPS) measurements were employed to analyze the chemical state of elements in the membranes annealed at 400 °C in Ar (designated as SnO_2–SiO_2/SiO_2-Ar-400) and in sulfur vapor (designated as SnO_2–SnS_2–SiO_2/SiO_2-S-400). As depicted in Figure 3d, the sulfur element is present solely in a S^{2-} state in the SnO_2–SnS_2–SiO_2/SiO_2-S-400 membrane.[25] Furthermore, SnO_2–SnS_2–SiO_2/SiO_2-400 sample exhibits a lattice oxygen ratio of 32%, which is considerably lower than the 53% observed in the SnO_2–SiO_2/SiO_2-Ar-400 sample (Figure 3e). This decrease in lattice oxygen ratio is a result of the sulfidation reaction, wherein the lattice oxygens in SnO_2 are partially replaced by sulfur to form SnS_2. It is also noted that the two Sn 3d peaks in SnO_2–SnS_2–SiO_2/SiO_2-S-400 sample move toward lower binding energies by 0.15 eV compared to those in the SnO_2–SiO_2/SiO_2-Ar-400 sample (Figure 3f). The primary factor influencing this binding energy shift is the formation of an S—Sn—O bond between SnO_2 and SnS_2.[2,26] Both XPS and Raman results confirm the generation of the SnO_2–SnS_2 composite at a sulfidation temperature of 400 °C.

SEM images in **Figure** 4a,b and Figure S4a,b (Supporting Information) show that the sulfurized membranes still retain their original micro-morphology when the sulfidation temperatures are 300, 350, and 400 °C. After being subjected to sulfidation at

 © 2024 Wiley-VCH GmbH

16163028, 2025, 4, Downloaded from https://advanced.onlinelibrary.wiley.com/doi/10.1002/adfm.202410833 by Nanjing Tech University, Wiley Online Library on [31/01/2025]. See the Terms and Conditions (https://onlinelibrary.wiley.com/terms-and-conditions) on Wiley Online Library for rules of use; OA articles are governed by the applicable Creative Commons License

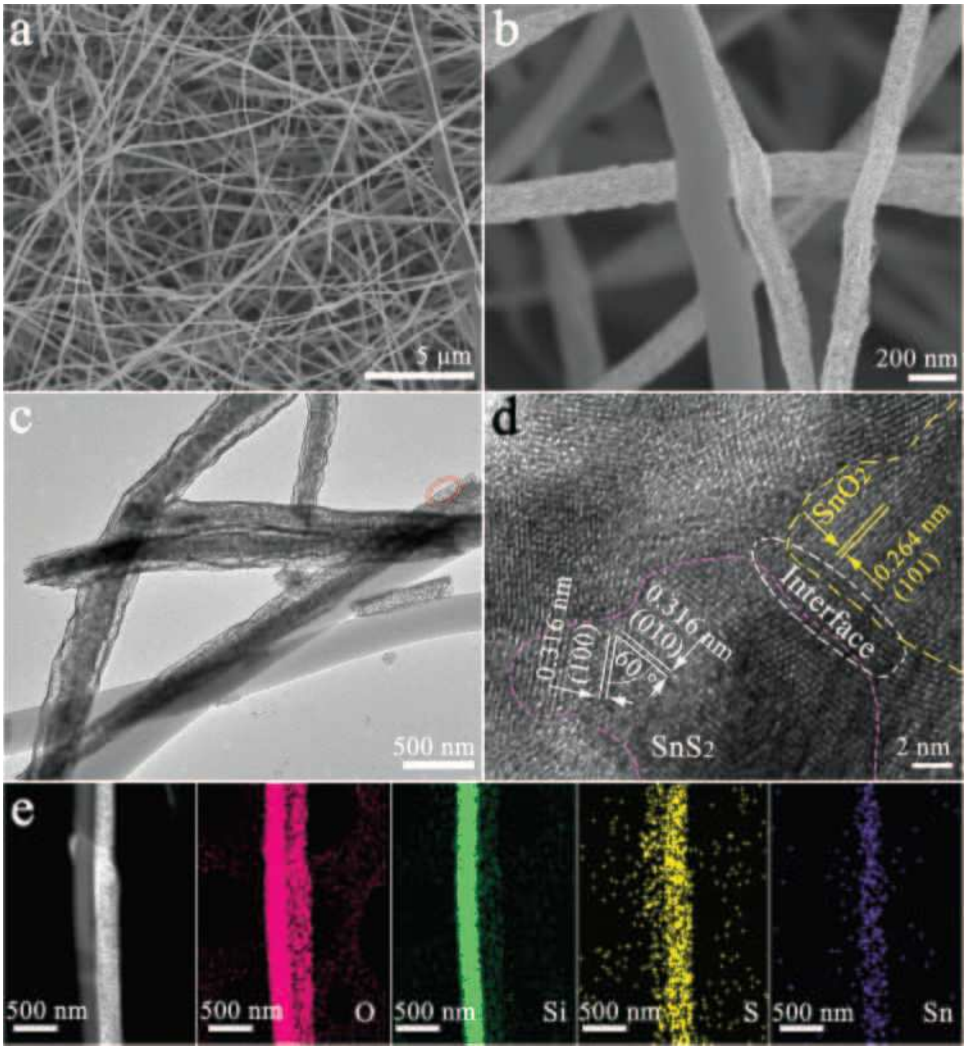

Figure 4. Morphology, structure, and element characterizations of SnO_2–SnS_2–SiO_2/SiO_2-S-400 NF membrane prepared at the sulfidation temperature of 400 °C: a) Low-magnification and b) high-magnification SEM images. c) TEM image of several SiO_2 and SnO_2–SnS_2 NFs. d) HRTEM image taken from the oval area of the single SnO_2–SnS_2 NF in (c). e) Elemental mapping images of O (pink), Si (green), S (yellow), and Sn (purple) for two adjacent SiO_2 and SnO_2–SnS_2 NFs.

400 °C, the SiO_2 NFs maintain their amorphous structure and smooth surface, while the SnO_2 NFs are converted into SnO_2–SnS_2 heterojunction NFs (Figure 4c,d). The lattice distance of 0.264 nm corresponds to the (101) crystal planes of SnO_2, and the lattice distances of 0.316 nm correlate to the (100) or (010) crystal planes of hexagonal SnS_2. The SnO_2–SnS_2 NFs are observed to consist of SnO_2 and SnS_2 nanoparticles, and heterojunction interfaces between tetragonal SnO_2 nanoparticles and hexagonal SnS_2 nanoparticles can be identified, as delineated by the dotted lines in Figure 4d. Elemental mapping findings confirm that the smooth and amorphous NFs contain Si and O elements, while the coarse, porous, and polycrystalline NFs consist of Sn, O, and S elements for the SnO_2–SnS_2–SiO_2/SiO_2-S-400 sample (Figure 4e). When the sulfidation temperature reaches 450 °C, a new morphology of hexagonal microdisks appears in the NF membrane (Figure S4c, Supporting Information). The microdisks, which become the dominating product instead of SnO_2 NFs at the high sulfidation temperature of 500 °C (Figure S4d,e, Supporting Information), are identified to be hexagonal SnS_2 according to the HRTEM result in Figure S4f (Supporting Information). The above results indicate that lower sulfidation temperatures (300, 350, and 400 °C) are beneficial for preserving the original NF structure. However, these temperatures are insufficient for fully converting SnO_2 NFs into SnS_2 NFs, even after 6 repetitive sulfidation cycles (Figure S5, Supporting Information). It is important to note that sulfurization occurs throughout both the surface and interior of the SnO_2 NF layer due to the porous network structure and high gas permeability of the NF membranes (Figure S6, Supporting Information).

Air permeability greatly impacts the comfort of a flexible wearable sensor by determining its ability to exchange moisture and air between the skin and the environment. The permeability of various NF membranes was evaluated by a water molecule transmission test at 25 °C. As shown in **Figure 5**, over a period of seven days, the amounts of traversed water molecules from commercial polyethylene terephthalate (PET) plate, filter paper,

 © 2024 Wiley-VCH GmbH

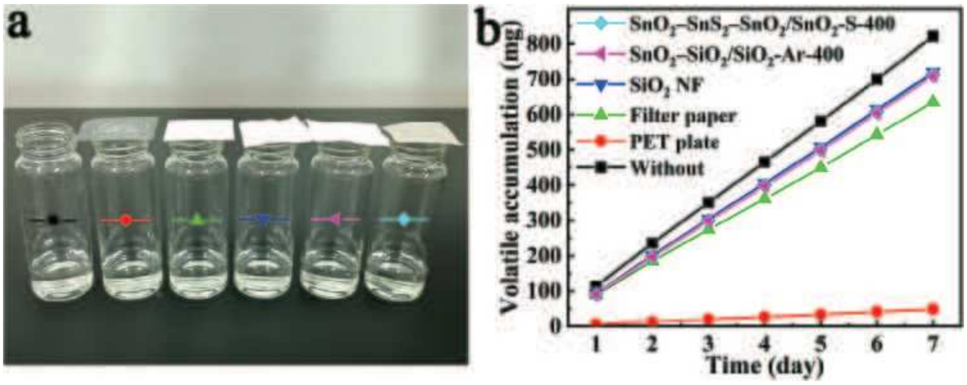

Figure 5. a) Schematic illustration of water vapor transmission experiments. b) Water contents that evaporated from the bottles.

SnO_2–SiO_2/SiO_2-Ar-400 NF membrane, SnO_2–SnS_2–SiO_2/SiO_2-S-400 NF membrane, SiO_2 NF membrane, and absence plate are 50, 630, 689, 709, 713, and 820 mg, respectively. The results indicate that the conventional PET flexible substrate is almost gas-impermeable. In contrast, the homemade SiO_2, SnO_2–SiO_2/SiO_2-Ar-400, and SnO_2–SnS_2–SiO_2/SiO_2-S-400 NF membranes exhibit a good capability to rapidly transport H_2O and various gas molecules, demonstrating their excellent gas permeability. The high gas permeability of various SiO_2-based membranes is a result of their micro/nanopore three-dimensional network NF structure, which promotes rapid air and moisture diffusion through the porous channels.

2.2. Flexibility Mechanism of SnO_2–SnS_2–SiO_2/SiO_2 Membranes

Flexibility, mechanical stability, electrical stability, and gas permeability are critical for the practical application of flexible sensors. The two-spinneret hybrid electrospinning technique is essential for fabricating flexible SnO_2–SnS_2–SiO_2/SiO_2 membranes. Various NFs, such as SiO_2, SnO_2, SnO_2/SiO_2, 400 °C-sulfurized SnO_2 (designated as SnO_2–SnS_2-S-400), and 400 °C-sulfurized SnO_2/SiO_2 (designated as SnO_2–SnS_2/SiO_2-S-400), were additionally synthesized using the single-spinneret electrospinning technique to examine the flexibility mechanism of the SnO_2–SnS_2–SiO_2/SiO_2 NF membranes. As shown in Figure S7a,b (Supporting Information), SnO_2 and SnO_2–SnS_2-S-400 NFs are non-flexible and non-self-supporting. The SnO_2/SiO_2 and SnO_2–SnS_2/SiO_2-S-400 NF membranes can sustain a self-supporting function, but the SnO_2 and SnO_2–SnS_2 surface layers are partly separated from the SiO_2 NF substrate layer (Figure S7c,d, Supporting Information). Therefore, the network interwoven SiO_2 NFs in both the substrate layer and the sensing layer is crucial to the flexible self-supporting characteristics of SnO_2–SnS_2–SiO_2/SiO_2 membranes.

The impact of the crystalline state of SiO_2 NFs on the membrane's flexibility was also investigated. As shown in Figure S8a,d, the SiO_2 NFs remain amorphous, and the NF membrane still retains considerable flexibility when the calcination temperature is up to 900 °C. Nevertheless, the formation of the monoclinic SiO_2 phase occurs at the calcination temperatures over 1000 °C, resulting in the fracture of the NFs and forming a weak self-supporting membrane with limited flexibility (Figure S8b–d, Supporting Information). The poor flexibility of crystalline SiO_2 NF membranes originates from the hard nature of the crystalline SiO_2 NFs and the numerous grain boundaries inside the SiO_2 NFs created during the crystallization process. Therefore, the amorphous characteristic of SiO_2 NFs is important to the membrane's remarkable flexibility and self-supporting ability.

The micro-morphologies, electrical and flexible characterizations of the sensors constructed by SiO_2-S-400, SnO_2–SnS_2–SiO_2/SiO_2-S-400, and SnO_2–SnS_2/SiO_2-S-400 NF membranes were investigated. SnO_2–SnS_2-S-400/PET sensor, which was created by coating SnO_2–SnS_2-S-400 NFs on commercial PET substrates (Figure S9, Supporting Information), was also examined for comparison. SEM images in **Figure 6**a,b show that the SiO_2-S-400 and SnO_2–SnS_2–SiO_2/SiO_2-S-400 sensors maintain undamaged, free of cracks and detachments under a bending state (140°). On the contrary, the SnO_2–SnS_2/SiO_2-S-400 sensor, and particularly the SnO_2–SnS_2-S-400 NF/PET sensor, undergo significant splitting and detachments under a similar bending process. The mechanical durability of the four kinds of sensors were evaluated by analyzing the real-time resistance curves during 140 repetitive bending cycles, as shown in Figure 6e–h. No obvious effect of bending on the resistance variation is found for the SiO_2-S-400 NF membrane sensor, which exhibits invariable insulation characteristics with an exceptionally high electrical resistance of $\approx 3 \times 10^{11}$ Ω (Figure 6e). For the two-spinneret electrospun SnO_2–SnS_2–SiO_2/SiO_2-S-400 sensor, excellent flexibility stability is also revealed, as only a very small resistance variation of 5% is shown upon the 140 repetitive bending cycles with a large bending angle of 140° (Figure 6f). In contrast, for the SnO_2–SnS_2/SiO_2-S-400 and conventional SnO_2–SnS_2-S-400/PET sensors, the resistances quickly rise following the first bending cycle because of the breakage of the superficial SnO_2–SnS_2 NFs, and the corresponding resistance variations are up to 13 520% and 3260%, respectively.

Figure 6i–l shows the results of further flexibility evaluation of the four kinds of sensors using real-time stress curves through 40 bending cycles (0–140°). The SiO_2-based sensors, including SiO_2-S-400, SnO_2–SnS_2–SiO_2/SiO_2-S-400 and SnO_2–SnS_2/SiO_2-S-400 NF, experience strains of −27 to 5 mN when being bent, whereas the traditional PET-based sensor encounters stresses of −174 to 143 mN. Given that the former requires less force to bend at the same angle than the latter, it can be witnessed that the homemade SiO_2 NF-based sensors are more flexible than the traditional flexible PET-based sensor.

 © 2024 Wiley-VCH GmbH

16163028, 2025, 4, Downloaded from https://advanced.onlinelibrary.wiley.com/doi/10.1002/adfm.202410833 by Nanjing Tech University, Wiley Online Library on [31/01/2025]. See the Terms and Conditions (https://onlinelibrary.wiley.com/terms-and-conditions) on Wiley Online Library for rules of use; OA articles are governed by the applicable Creative Commons License

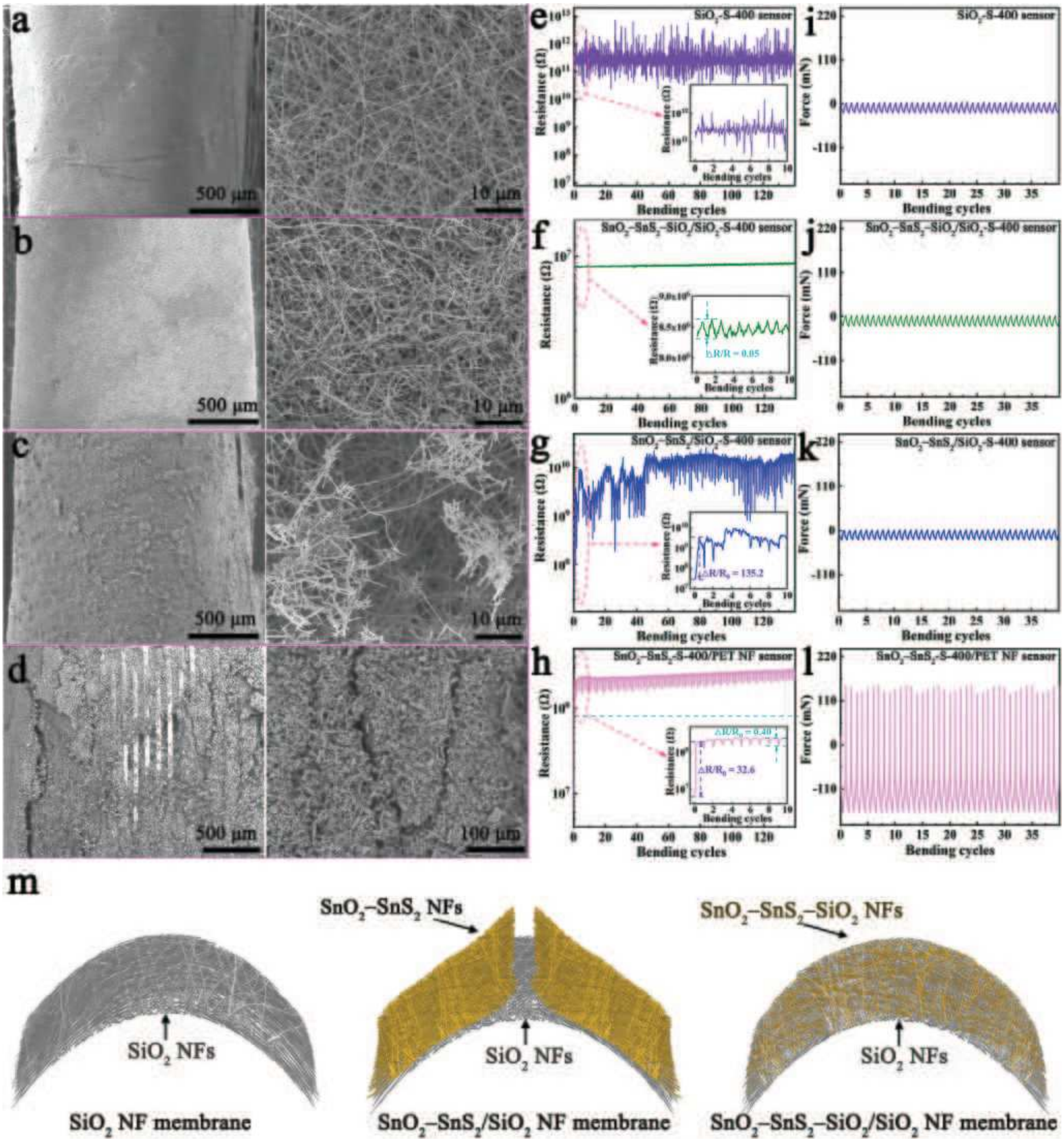

Figure 6. Low-magnification and high-magnification SEM images of a) SiO_2-S-400 NF, b) SnO_2–SnS_2–SiO_2/SiO_2-S-400 NF, and c) SnO_2–SnS_2/SiO_2-S-400 sensors under the bending angle of 140°. d) Low-magnification and high-magnification SEM images of SnS_2–SnO_2-S-400 NF/PET sensor under the bending angle of 140°. Resistances of e) SiO_2-S-400 NF, f) SnO_2–SnS_2–SiO_2/SiO_2-S-400 NF, g) SnO_2–SnS_2/SiO_2-S-400 NF, and h) SnO_2–SnS_2-S-400 NF/PET sensors as a function of the blending cycle. Stress–bending cycle curves of i) SiO_2-S-400 NF, j) SnO_2–SnS_2–SiO_2/SiO_2-S-400 NF, k) SnO_2–SnS_2/SiO_2-S-400 NF, and l) SnO_2–SnS_2-S-400 NF/PET sensors. m) Schematic illustrations of SiO_2, SnO_2–SnS_2/SiO_2, and SnO_2–SnS_2–SiO_2/SiO_2 NF membranes in the bent state.

© 2024 Wiley-VCH GmbH

16163028, 2025, 4, Downloaded from https://advanced.onlinelibrary.wiley.com/doi/10.1002/adfm.202410833 by Nanjing Tech University, Wiley Online Library on [31/01/2025]. See the Terms and Conditions (https://onlinelibrary.wiley.com/terms-and-conditions) on Wiley Online Library for rules of use; OA articles are governed by the applicable Creative Commons License

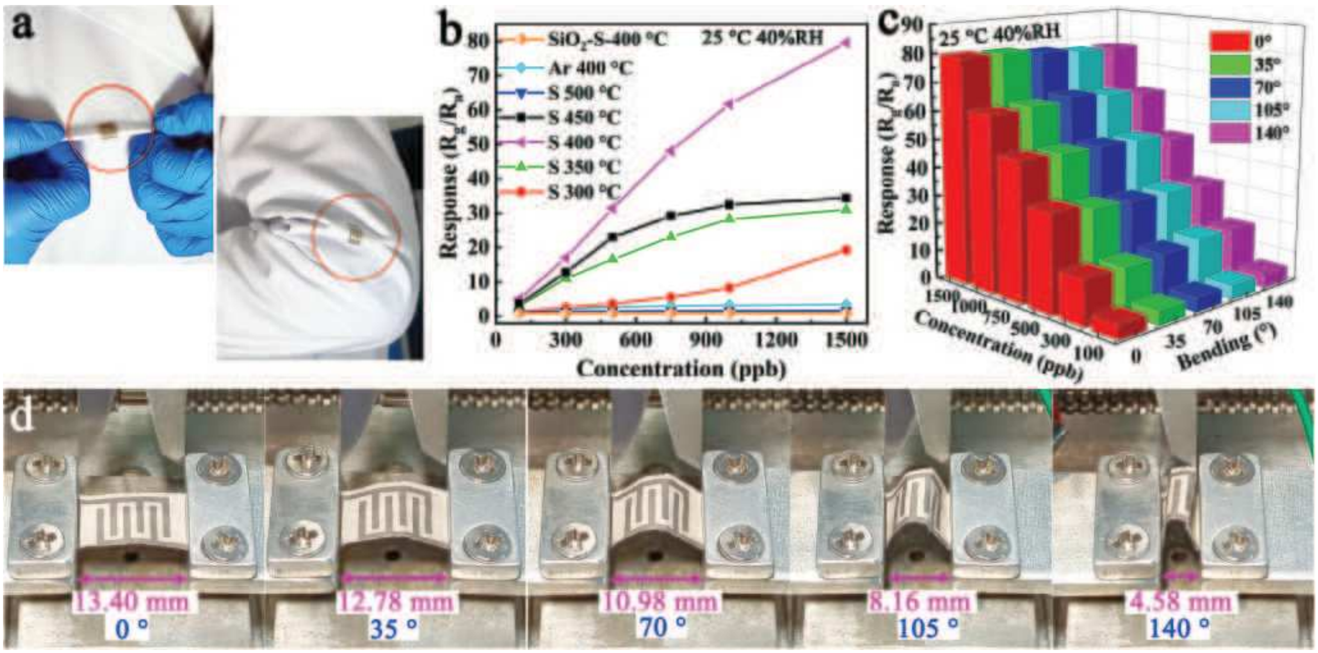

Figure 7. a) Photographs of SnO_2–SnS_2–SiO_2/SiO_2-S-400 NF membrane sensors attached to clothes. b) Responses of various NF membrane sensors to NO_2 with various concentrations under the conditions of 40% RH and 25 °C. c) Responses of the SnO_2–SnS_2–SiO_2/SiO_2-S-400 sensor as functions of bending angle and NO2 concentration. d) Photographs of the SnO_2–SnS_2–SiO_2/SiO_2-S-400 sensor at various bending angles.

The detailed mechanism for the exceptional flexibility of the two-spinneret electrospun SnO_2–SnS_2–SiO_2/SiO_2-S-400 NF membrane, as shown by the schematic diagram in Figure 6m, is ascribed to the following factors based on the aforementioned findings: (i) the amorphous feature of SiO_2 NFs lacks grain boundaries and has a lower elastic modulus, making them more flexible than crystalline SiO_2 NFs. By interweaving these amorphous SiO_2 NFs in a three-dimensional network, the SiO_2 NF substrate layer exhibits significant levels of flexibility and self-support; ii) the barrier effect of Si precursor NFs can reduce the mutual adhesion of Sn precursor NFs during the calcination process (Figure S10, Supporting Information). The resulting SnO_2 NFs thus have a better degree of freedom during bending and deformation, allowing the SnO_2–SnS_2 NFs to release stress through displacements and slight deformations; iii) Si and Sn electrospun precursor NFs have an intertwined network structure. During the calcination process, the relatively lower shrinkage of the Si precursor NFs may help to reduce the overall shrinkage of the Sn precursor NFs. Additionally, the structural framework provided by the Si precursor NFs can restrain the aggregation and regrowth of the Sn precursor NFs, thereby further attenuating their shrinkage during calcination. This effect is illustrated by the SEM images of the SnO_2 NFs (Figure S10a–d, Supporting Information) and SnO_2–SiO_2/SiO_2 NF membrane (Figure S10e,f, Supporting Information); iv) the three-dimensional network interwoven structure between amorphous SiO_2 NFs and SnO_2–SnS_2 NFs contributes crucially to the fantastic flexibility of the SnO_2–SnS_2–SiO_2/SiO_2-S-400 NF membrane. For the single-spinneret electrospun SnO_2–SnS_2/SiO_2-S-400 NF membrane, the crystalline SnO_2–SnS_2 NFs are susceptible to cracking during the bending cycle because of the sustained considerable stress perpendicular to the curved interface. Moreover, the SnO_2–SnS_2 NF layer is also easy to split away from the SiO_2 NF layer because of the limited contact interface and weak bonding force between these two layers. In contrast, for the two-spinneret electrospun SnO_2–SnS_2–SiO_2/SiO_2-S-400 membrane, amorphous SiO_2 NFs with exceptional flexibility are arbitrarily implanted inside the SnO_2–SnS_2 layer, offering a greater buffer space for SnO_2–SnS_2 NFs when subjected to deformation. More importantly, the flexible SiO_2 NFs in the SnO_2–SnS_2 layer function as a structural framework, efficiently bearing and transmitting stress throughout the bending procedure. Cracking of the SnO_2–SnS_2 layer is thus effectively mitigated by the above two functions of the amorphous SiO_2 NFs; (v) the original reticular interwoven structure is well kept during the sulfidation reaction between SnO_2 NFs and sulfur vapor at 400 °C, thus ensuring the stable electrical properties and good mechanical flexibility of the SnO_2–SnS_2/SiO_2-S-400 NF membrane.

2.3. NO_2 Sensing Performance

The flexible SnO_2–SnS_2–SiO_2/SiO_2-S-400 sensor can be affixed to clothing as a proof of concept (**Figure** 7a), demonstrating the good application potential of the sensor in the wearable field. The response–concentration curves for various flexible sensors to different concentrations of NO_2 at 25 °C and 40% RH are shown in Figure 7b. Without sulfidation, the SnO_2–SiO_2/SiO_2-Ar-400 sensor shows low responses to 100–1500 ppb NO_2. The SiO_2 NFs sulfurized at 400 °C exhibit no measurable response to NO_2 and possess a remarkably high resistance. This observation suggests that the SiO_2 NFs primarily serve as a support material within the composite sensors, without contributing to the NO_2 gas-sensing response. The thermal sulfidation treatment within the

© 2024 Wiley-VCH GmbH
16163028, 2025, 4, Downloaded from https://advanced.onlinelibrary.wiley.com/doi/10.1002/adfm.202410833 by Nanjing Tech University, Wiley Online Library on [31/01/2025]. See the Terms and Conditions (https://onlinelibrary.wiley.com/terms-and-conditions) on Wiley Online Library for rules of use; OA articles are governed by the applicable Creative Commons License

16163028, 2025, 4, Downloaded from https://advanced.onlinelibrary.wiley.com/doi/10.1002/adfm.202410833 by Nanjing Tech University, Wiley Online Library on [31/01/2025]. See the Terms and Conditions (https://onlinelibrary.wiley.com/terms-and-conditions) on Wiley Online Library for rules of use; OA articles are governed by the applicable Creative Commons License

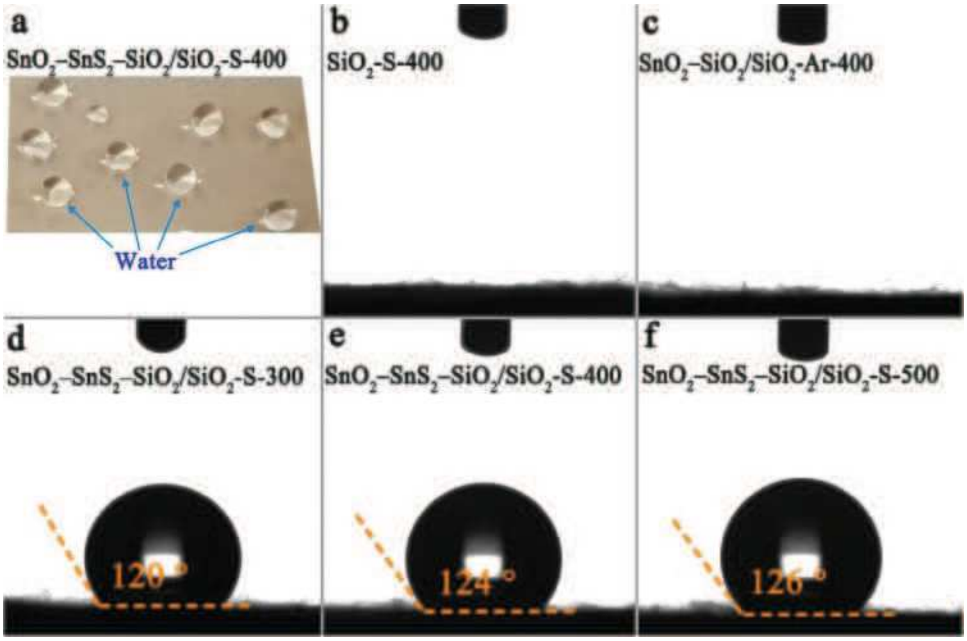

Figure 8. a) Photograph of water droplet shape on the SnO_2–SnS_2–SiO_2/SiO_2-S-400 NF membrane. Optical images of surface contact angle on various fiber membranes: b) SiO_2-S-400, c) SnO_2–SiO_2/SiO_2-Ar-400, d) SnO_2–SnS_2–SiO_2/SiO_2-S-300, e) SnO_2–SnS_2–SiO_2/SiO_2-S-400, and f) SnO_2–SnS_2–SiO_2/SiO_2-S-500.

temperature range of 300–450 °C greatly improves the responses of NF membrane sensors to NO_2. As the sulfidation temperature rises, the response of SnO_2–SnS_2–SiO_2/SiO_2 sensor to NO_2 increases, and is maximum when the sulfidation temperature climbs to 400 °C. Further increase in sulfidation temperature gradually reduces the response to NO_2. For example, the responses of the SnO_2–SnS_2–SiO_2/SiO_2 sensors sulfurized at 300 °C, 350 °C, 400 °C, 450 °C, and 500 °C to 1000 ppb NO_2 are 9.3, 29.3, 63.0, 33.4, and 2.6, respectively. The SnO_2–SnS_2–SiO_2/SiO_2-S-400 sensor exhibits the highest response to NO_2 among various sulfurized NF sensors, which is partly attributed to the formation of effective SnO_2–SnS_2 heterojunctions at the sulfidation temperature of 400 °C. When the sulfidation temperature remains below 400 °C, only a small amount of SnS_2 and SnO_2–SnS_2 heterojunctions are formed. Consequently, the NO_2 sensing performances of SnO_2–SnS_2–SiO_2/SiO_2-S-300 and SnO_2–SnS_2–SiO_2/SiO_2-S-350 sensors are not greatly boosted. When the sulfidation temperature is raised to 450 °C, additional SnS_2 hexagonal nanosheets are generated. The SnS_2 nanosheets and SnO_2 NFs separate and become independent of each other, which is not conducive to the improvement of gas-sensing performance from the perspective of heterojunction effect. After further increasing the sulfidation temperature to 500 °C, the vast majority of SnO_2 NFs are substituted with SnS_2 hexagonal nanosheets. The internal conductance is damaged due to the low interconnection channels between the SnS_2 hexagonal nanosheets, resulting in a high initial resistance and a degraded NO_2 response of the SnO_2–SnS_2–SiO_2/SiO_2-S-500 sensor.

The response stability under various bending angles is a crucial parameter for flexible gas sensors. Figure 7c shows the response bar graph of the SnO_2–SnS_2–SiO_2/SiO_2-400 sensor to various concentrations of NO_2 with different bending angles. The responses of the sensor to 1000 ppb NO_2 at bending angles of 0°, 35°, 70°, 105°, and 140° are 63.0, 63.6, 65.0, 63.7, and 62.4, respectively, with a small standard deviation of 63.540 ± 0.969. Figure 7d shows that the surface of the sensor remains intact at various bending angles without structural degradation. Both findings demonstrate excellent sensing stability toward NO_2 at varied bending angles of the flexible SnO_2–SnS_2–SiO_2/SiO_2-S-400 sensor.

Water molecules in the surroundings play a major role in influencing the sensing performance of gas sensors. Under high humidity, a water layer may form on the surface of sensing materials, altering the electron transfer pathway and reducing the response of gas sensors.[12b] Good humidity resistance of gas sensors is thus highly advantageous for improving their gas-sensing performance. As illustrated in **Figure** 8a, the SnO_2–SnS_2–SiO_2/SiO_2-S-400 NF membrane exhibits a characteristic comparable to that of a natural lotus leaf with excellent hydrophobicity. Spherical beads are formed when water droplets reach the surface of the SnO_2–SnS_2–SiO_2/SiO_2-S-400 NF membrane. The surface wettabilities of various NF membranes were further assessed by contact angle measurements of water, as presented in Figure 8b–f. Water droplets rapidly penetrate the interiors of SiO_2-S-400 and SnO_2–SiO_2/SiO_2-Ar-400 membranes upon contacting their surfaces, indicating the poor moisture resistance of these two non-sulfurized membranes. The membranes sulfurized at 300 °C, 400 °C, and 450 °C exhibit contact angles of 120°, 124°, and 126°, respectively, indicating that SnO_2–SnS_2 composites have strong moisture resistance. This is attributed to the Sn—S chemical bonds created on the surface of $SnO_{2\ NFs}$ after thermal sulfidation treatment. Sulfur atoms hinder the formation of hydrogen bonds with water molecules, thereby impeding the adherence of water molecules to the NF membranes.[27]

 © 2024 Wiley-VCH GmbH

16163028, 2025, 4, Downloaded from https://advanced.onlinelibrary.wiley.com/doi/10.1002/adfm.202410833 by Nanjing Tech University, Wiley Online Library on [31/01/2025]. See the Terms and Conditions (https://onlinelibrary.wiley.com/terms-and-conditions) on Wiley Online Library for rules of use; OA articles are governed by the applicable Creative Commons License

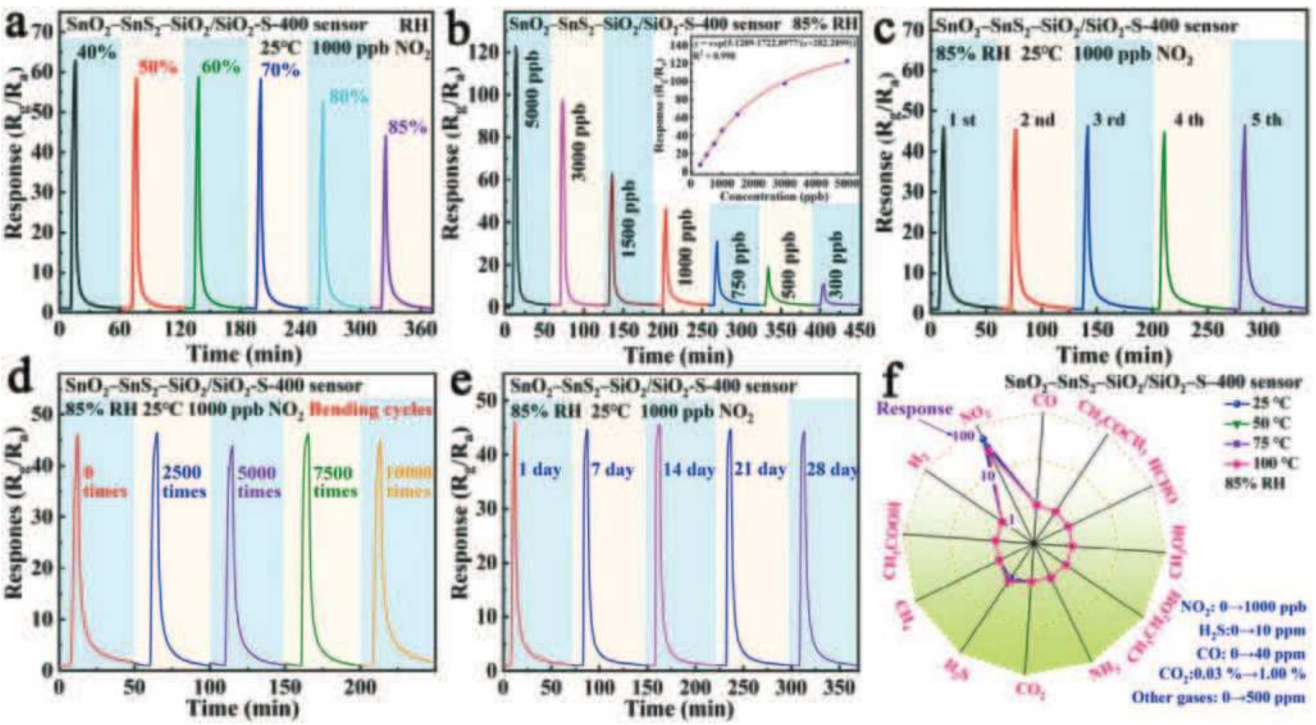

Figure 9. Gas-sensing performances of the flexible SnO_2–SnS_2–SiO_2/SiO_2-S-400 sensor to NO_2 at 25 °C. a) Response–time curves under various RH to 1000 ppb NO_2. b) Response–concentration curves under 85% RH. The inset shows the corresponding response–concentration fitting curve. c) Reproducibility measurements for five cycles to 1000 ppb NO_2 under 85% RH. d) Response–time curves under various bending cycles to 1000 ppb NO_2 under 85% RH. e) Stability measurements for 28 days to 1000 ppb NO_2 under 85% RH. f) Radar map of selectivity measurements to NO_2 against various interfering gases in the temperature range of 25–100 °C under 85% RH.

The subsequent sensing measurements were all carried out on the flexible SnO_2–SnS_2–SiO_2/SiO_2-S-400 sensor, since the optimal sulfidation temperature is determined to be 400 °C according the sensing results in Figure 7b. The gas-sensing performances of the SnO_2–SnS_2–SiO_2/SiO_2-S-400 sensor under various RH were first examined, as shown in **Figure 9**a. When the RH levels are 40, 50, 60, 70, 80, and 85%, the responses of the sensor to 1000 ppb NO_2 at 25 °C are 63.0, 60.0, 59.8, 59.4, 54.0, and 45.3, respectively. The response of the sensor to 1000 ppb NO_2 remains as high as 45.3 under the high RH of 85%, suggesting considerable potential of the sensor to be applied in high RH conditions. The gas-sensing capabilities of the sensor under 85% RH were further explored. The responses of the sensor under 85% RH to 300, 500, 750, 1000, 1500, 3000, and 5000 ppb NO_2 are 7.8, 19.1, 31.0, 45.3, 63.2, 98.0, and 124.1, respectively. An excellent functional relationship between the response and NO_2 concentration is displayed (inset of Figure 9b). Additionally, the sensor also demonstrates excellent response reproducibility to NO_2 under 85% RH (Figure 9c). The flexible stability of the sensor was assessed by measuring its sensing response after multiple bending cycles with a large bending angle of 140°. The specific bending process is illustrated in Video S1 (Supporting Information). As depicted in Figure 9d, the corresponding responses to 1000 ppb NO_2 under 85% RH are 45.3, 46.7, 44.1, 46.3, and 45.2 at bending cycles of 0, 2500, 5000, 7500, and 10 000 times, respectively. This shows that the sensor maintains a stable response toward NO_2 even after 10 000 bending cycles. Figure 9e illustrates the long-term stability of the sensor to 1000 ppb NO_2 at 25 °C under 85% RH. The corresponding responses of the sensor after 1, 7, 14, 21, and 28 days are 45.3, 44.9, 45.8, 44.8, and 44.5, respectively. No distinct degradation in response is found after 28 days, indicating that the sensor has an excellent long-term stability for NO_2 detection under high RH. Figure 9f illustrates the radar map of selectivity measurements of the sensor to a series of interfering gases at different temperatures. When the testing temperatures are 25, 50, 75, and 100 °C, the responses of the sensor to 1000 ppb NO_2 are 45.3, 30.2, 28.2, and 24.1, respectively, while those to the interfering gases with much higher concentrations are all below 1.5 at the involved temperatures, indicating the excellent NO_2 selectivity of the sensor against various interfering gases. All of these findings support the considerable potential of the flexible SnO_2–SnS_2–SiO_2/SiO_2-S-400 sensor for sensitive and selective monitoring of NO_2 concentrations under high RH environments.

2.4. Detection of Ultralow Concentration NO_2 in Exhaled Breath

The good humidity resistance, attractive flexibility, and high sensitivity of the flexible SnO_2–SnS_2–SiO_2/SiO_2-S-400 sensor inspire us to explore its potential application in the wearable field for detection of ultralow concentration NO_2 under high humidity environments. There are several potential medical uses for detecting very low NO_2 concentrations under high RH. For instance, an individual with exhaled breath containing more than 25 ppb NO_x is probably suffering from asthma or

 © 2024 Wiley-VCH GmbH

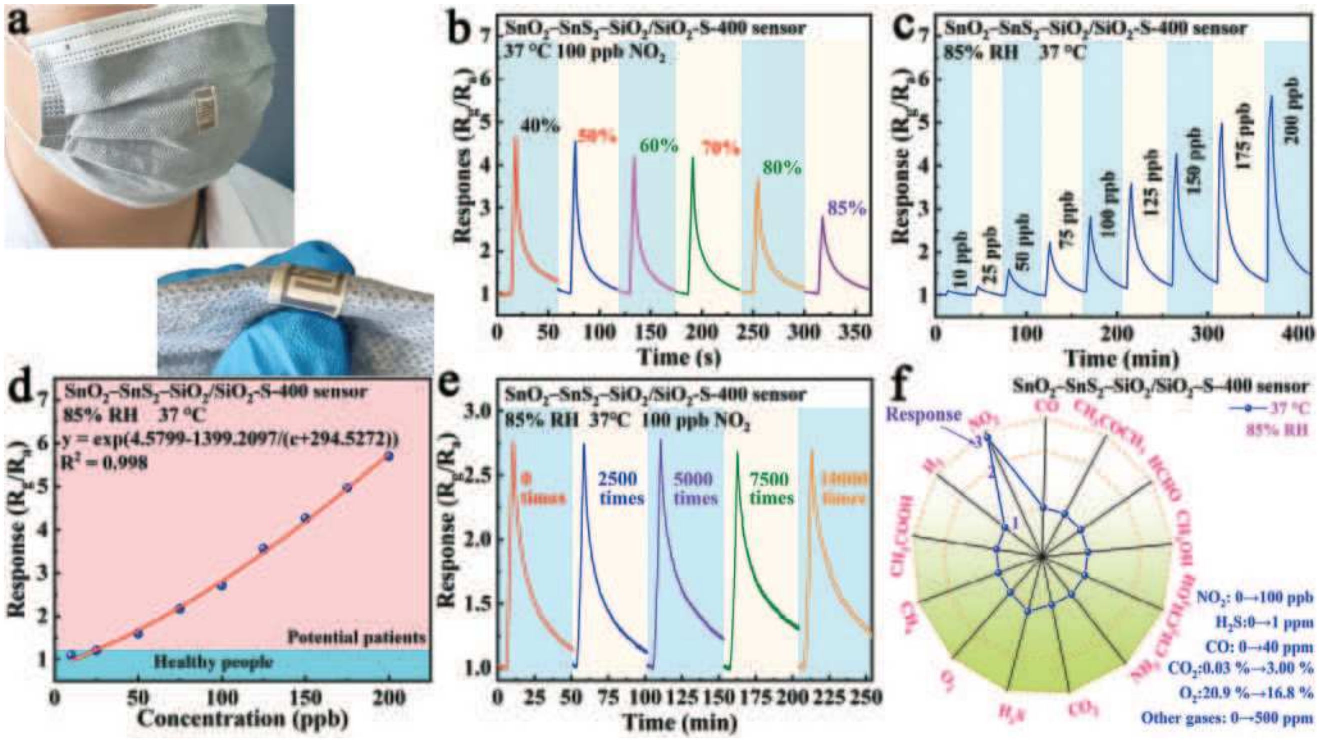

Figure 10. a) Photographs of the flexible SnO_2–SnS_2–SiO_2/SiO_2-S-400 sensors attached to masks. Gas-sensing performances of the SnO_2–SnS_2–SiO_2/SiO_2-S-400 sensor to low-concentration NO_2 at 37 °C. b) Response–time curves under various RH to 100 ppb NO_2. c) Response–concentration curves to NO_2 under 85% RH. d) Response–concentration fitting curve. e) Response–time curves under various bending cycles to 100 ppb NO_2 under 85% RH. f) Radar map of selectivity measurements to NO2 against various interfering gases in exhaled breath at 37 °C and 85% RH.

lung-related illness.[9a,10] Therefore, the gas-sensing performance of the SnO_2–SnS_2–SiO_2/SiO_2-S-400 sensor to low-concentration NO_2 in a gas environment similar to human exhaled breath (85% RH, 37 °C) was examined to assess its suitability for noninvasive breath diagnosis application. As demonstrated in **Figure 10**a, the flexible sensor can be securely affixed to masks, and remains intact when the mask is bent. For additional sensing measurements, the sensor can be linked to a source meter using wires and wiring clips (Figure S11, Supporting Information). Although there is a decrease in the response of the sensor with the increase of RH, an evident response of 2.8 is still revealed for a low-concentration NO_2 (100 ppb) under 85% RH (Figure 10b). Further testing was conducted to evaluate the gas-sensing response of the sensor toward various ultralow concentrations of NO_2 in a simulated human exhaled breath environment, as shown in Figure 10c. The responses of the sensor to 10, 25, 50, 75, 100, 125, 150, 175, and 200 ppb NO_2 at 37 °C and 85% RH are 1.1, 1.2, 1.6, 2.2, 2.7, 3.6, 4.3, 5.0, and 5.7, respectively. Detectable responses for 10 and 25 ppb NO_2 and evident responses for concentrations more than 50 ppb are revealed. A strong function relationship between NO_2 concentration and response value is obtained (Figure 10d), showing that the sensor is capable of effectively identifying the critical health concentration (25 ppb NO_2) at 37 °C and 85% RH. The sensor also exhibits impressive flexible stability to low-concentration NO_2 (100 ppb) under 85% RH, since its response keeps no obvious fluctuation with only a small decrease of 2.6% following very large bending cycles of 10 000 (Figure 10e). The response–recovery curves for three separate batches of SnO_2–SnS_2–SiO_2/SiO_2-S-400 sensors to 100 ppb under 85% RH are displayed in Figure S12 (Supporting Information). The results reveal that the sensors, produced via the electrospinning technique combined with thermal sulfidation approach, maintain good repeatability in their sensing performance. Further investigation was conducted on the anti-interference performance of the sensor against common gases in human breath, as shown in Figure 10f. The sensor shows minimal response to typical interfering gases such as CO (40 ppm), CH_3COCH_3 (500 ppm), HCHO (500 ppm), CH_3OH (500 ppm), CH_3CH_2OH (500 ppm), NH_3 (500 ppm), H_2S (1 ppm), CH_4 (500 ppm), CH_3COOH (500 ppm), and H_2 (500 ppm). The responses to the major gases (O_2 and CO_2) present in human exhaled breath are also negligible. For example, when the O_2 concentration drops from 20.9% in inhaled air to 16.8% in exhaled gas, or the CO_2 concentration rises from 0.03% to 3%, the resistance of the sensor remains constant. This indicates that the sensor has exceptional NO_2 selectivity in an exhaled breath environment. This excellent selectivity can be attributed to three primary factors. First, unlike various interfering gases, electrophilic NO_2 is capable of directly causing a resistance change without the need to interact with surface-adsorbed oxygen species.[2,28] Second, as presented in Table S2 (Supporting Information), SnO_2 and SnS_2 have much greater adsorption capabilities for NO_2, as the adsorption energies of NO_2 on these surfaces are markedly lower than those of other gases. Moreover, NO_2 results in greater Bader charge transfers with SnO_2 and SnS_2 (Table S2, Supporting Information), leading to more significant alterations in their conductivity.[29]

 © 2024 Wiley-VCH GmbH

16163028, 2025, 4, Downloaded from https://advanced.onlinelibrary.wiley.com/doi/10.1002/adfm.202410833 by Nanjing Tech University, Wiley Online Library on [31/01/2025]. See the Terms and Conditions (https://onlinelibrary.wiley.com/terms-and-conditions) on Wiley Online Library for rules of use; OA articles are governed by the applicable Creative Commons License

Third, the sensor operates optimally at RT for NO_2 detection, whereas most interfering gases, such as CH_3COOH, CH_3CH_2OH, HCHO, CH_3COCH_3, and CH_3OH, require temperatures above 300 °C for effective sensing (Figure S13, Supporting Information). At 37 °C, the energy is thus insufficient to drive effective sensing reactions between these interfering gas molecules and the adsorbed oxygen species on the SnO_2–SnS_2 NF surfaces, resulting in their minimal sensing responses.[30] The aforesaid findings demonstrate that the flexible SnO_2–SnS_2–SiO_2/SiO_2-S-400 sensor is sensitive, structurally stable, and highly selective for detecting low-concentration NO_2 in the exhaled breath environment for monitoring asthma-related illnesses.

The possibility of downsizing the SnO_2–SnS_2–SiO_2/SiO_2-S-400 sensor has been examined. Images of the reduced-size SnO_2–SnS_2–SiO_2/SiO_2-S-400 sensors, along with their dynamic response–recovery curves toward 100 ppb NO_2 at 37 °C under 85% RH, are shown in Figure S14 (Supporting Information). The findings clearly demonstrate that the SnO_2–SnS_2–SiO_2/SiO_2-S-400 sensor can be effectively miniaturized without compromising its functionality, since these miniaturized sensors maintain similar NO_2 sensing performance as their larger counterparts.

Sub.: substrate, Con.: concentration, Res.: response, Tem.: temperature, Roc.: radius of curvature, IM-0.1/P-1.0: mixtures of n-type conjugated polymers and a polymer dopant, CT and ET: cotton thread and elastic thread, PAN: polyacrylonitrile, SWCNT: Single walled carbon nanotubes, MWCNT: multiwalled carbon nanotubes, PET: polyethylene terephthalate, PMMA: polymethyl methacrylate, TPU: thermoplastic polyurethane, PI: polyimide.

Table 1 compares the NO_2 sensing performance of the flexible SnO_2–SnS_2–SiO_2/SiO_2-S-400 sensor with that of other flexible sensors. The all-inorganic flexible SnO_2–SnS_2–SiO_2/SiO_2-S-400 sensor stands out by its excellent gas permeability, superior NO_2 sensing performance, good humidity tolerance, and strong mechanical stability over large bending cycles (10 000 times). In particular, the sensor in our case is capable of monitoring ultralow concentration NO_2 (25 ppb) in the exhaled breath environment for potential health-related application.

2.5. Gas Sensing Mechanism

The resistance change caused by the adsorption and desorption of NO_2 molecules on the surface of sensitive materials is the fundamental principle of gas-sensing responses in metal oxide materials.[13,34] In the SnO_2–SnS_2–SiO_2/SiO_2-400 sensor, the insulating SiO_2 NFs, serving as the backbone and substrate material, are unresponsive to NO_2. The SnO_2–SnS_2 NFs are primarily responsible for the NO_2 gas-sensing response. In ambient air, SnO_2–SnS_2 NFs adsorb O_2 molecules, and chemisorbed oxygen ions ($O^-_{2(ads)}$) are generated according to Equations (1) and (2). The discharge of electrons from the conduction band of SnO_2–SnS_2 NFs results in the formation of an electron depletion layer (EDL) on their surface with high resistance. After introducing NO_2 molecules, they adsorb on the surface of SnO_2–SnS_2 NFs, and capture electrons to form $NO^{--}_{2(ads)}$ (Equation (3)). Furthermore, the NO_2 molecules can also engage in an electron-capturing interaction with $O^-_{2(ads)}$, as depicted in Equation (4). As a result of the above processes, the EDL gets thicker and a sharp

Table 1. Sensing performance of various flexible NO_2 sensors.

Materials	Sub.	Con. [ppm]	Res.	Tem. [°C]/Other	Bending angle [°]/Roc. [mm]	Res. decrease/Bending cycles	Gas permeability	Humidity tolerance	Res. to 25 ppb in exhaled breath	Ref.
SnO_2–SnS_2	SiO_2	1	63.0	25 °C/–	140/–	No obvious decrease/10000	Yes	Yes	Yes	This Work
ZnO–rGO	CT and ET	15	1.4	25 °C/–	–/1	No obvious decrease/3000	Yes	No	NO	[31]
Co-SnS_2	PAN	1	1.1	25 °C/–	–/2.5	18.0%/1000	Yes	–	NO	[32]
Co_3O_4–MWCNT	MWCNT	1000	1.32	25 °C/–	120/–	–/–	Yes	No	NO	[33]
TiO_2–g-C_3N_4–SnO_2	PET	1	1.5	25 °C/Uv-light	–/10	No obvious decrease/2800	No	Yes	NO	[34]
In_2Se_3	PMMA	1	1.4	25 °C/–	–/10	50%/1000	No	No	NO	[35]
IM-0.1–P-1.0	TPU	1	3.4	60/–	80/1.9	No obvious decrease/15	No	Yes	NO	[3]
WS_2–$Ti_3C_2T_x$	Paper-based	1	1.2	25 °C/–	60/–	No obvious decrease/500	No	Yes	NO	[36]
Au–SnS_2–Ti_3C_2	PI	1	5.3	25 °C/–	60/–	10%/700	No	No	NO	[37]
Bi_2Se_3	PI	1	1.3	25 °C/–	90/–	–/–	No	Yes	NO	[38]
SWCNT–PdO–Co_3O_4	PI	20	1.3	25 °C/–	–/–	No obvious decrease/4000	No	No	NO	[39]
IGZO	PI	20	1.3	25 °C/–	–/5	No obvious decrease/1000	No	No	NO	[40]
In_2O_3–GO	PI	1	1.32	25 °C/–	–/–	No obvious decrease/100	No	–	NO	[41]

© 2024 Wiley-VCH GmbH

16163028, 2025, 4, Downloaded from https://advanced.onlinelibrary.wiley.com/doi/10.1002/adfm.202410833 by Nanjing Tech University, Wiley Online Library on [31/01/2025]. See the Terms and Conditions (https://onlinelibrary.wiley.com/terms-and-conditions) on Wiley Online Library for rules of use; OA articles are governed by the applicable Creative Commons License

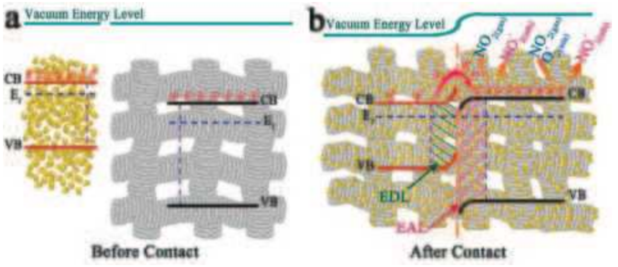

Figure 11. Energy band diagrams of a) SnS_2, SnO_2, and b) SnO_2–SnS_2 heterojunction.

increase in the resistance of the SnO_2–SnS_2–SiO_2/SiO_2-400 sensor is presented.

$$O_{2(gas)} \rightarrow O_{2(ads)} \tag{1}$$

$$O_2 + e^- \rightarrow O^-_{2(ads)} \tag{2}$$

$$NO_2(_{gas}) + e^- \rightarrow NO^-_{2(ads)} \tag{3}$$

$$NO_2(_{gas}) + O^-_{2(ads)} + 2e^- \rightarrow NO^-_{2(ads)} + 2O^-_{(ads)} \tag{4}$$

In comparison with the SnO_2–SiO_2/SiO_2 sensor, the enhanced gas-sensing performance of the SnO_2–SnS_2–SiO_2/SiO_2-S-400 sensor is mainly attributed to the following two aspects. First, the formation of SnO_2–SnS_2 n–n heterojunctions through thermal sulfidation treatment enhances the resistance modulation ability of the sensor. To investigate the charge transfer in the SnO_2–SnS_2 heterojunction, the work functions of SnO_2–SiO_2 NFs and SnO_2–SnS_2–SiO_2 NFs were characterized using ultraviolet photoelectron spectroscopy (UPS),[42] as shown in Figure S15a,b (Supporting Information). The insulating nature of SiO_2 NFs leads to a very low signal contribution in the UPS spectra (Figure S15a–d, Supporting Information). Furthermore, since the SiO_2 NFs are spatially separated from SnO_2 and SnO_2–SnS_2 NFs, without forming heterojunction interfaces, their impact on the UPS spectra, particularly on the relative energy band diagrams of SnO_2 and SnO_2–SnS_2 NFs, is considered to be minimal. The results show that the work functions of SnO_2 and SnO_2–SnS_2 NFs are calculated to be 4.40 and 3.31 eV, respectively. When SnO_2 is thermally sulfureted, a lower work function of SnO_2–SnS_2 is obtained. This occurs because, as illustrated in **Figure 11**a,b, the Fermi level of pure SnO_2 is less than that of SnS_2. After forming the SnO_2–SnS_2 heterojunction, electrons in the conduction band of SnS_2 diffuse toward the SnO_2 conduction band under the action of the built-in electric field, until the Fermi levels of SnO_2 and SnS_2 reach equilibrium. The EDL of SnO_2 NFs is thinner as a result of the aforementioned process. Figure S16 (Supporting Information) shows that for the SnO_2–SnS_2–SiO_2/SiO_2-S-400 and SnO_2–SiO_2/SiO_2-Ar-400 sensors, the R_a values are 6.2 × 10^6 and 1.1 × 10^7 Ω, respectively. This observed drop in resistance of the SnO_2–SnS_2–SiO_2/SiO_2-S-400 sensor matches the direction of electron flow from SnS_2 to SnO_2. These transferred active free electrons on the surface of SnO_2 NFs have the capability to transform the surface adsorbed O_2 into $O^{--}_{2(ads)}$, thus resulting in an increased content of $O^{--}_{2(ads)}$. The increased $O^{--}_{2(ads)}$ promotes the gas-sensing reaction shown in Equation (4), allowing NO_2 molecules to capture more electrons from the SnO_2–SnS_2 NFs, consequently increasing the gas-sensing response of the SnO_2–SnS_2–SiO_2/SiO_2-S-400 sensor. The quantity of SnO_2–SnS_2 heterojunctions is a crucial factor in determining the sensing response of SnO_2–SnS_2–SiO_2/SiO_2 sensors. By increasing the reaction temperature, reaction time or sulfidation cycles, the relative proportion of SnS_2 to SnO_2 can be increased within a specific range, as demonstrated by XRD (Figure 3b; Figure S17a, Supporting Information), Raman spectroscopy (Figure 3c), and composition characterization results (Table S3, Supporting Information). The sensor response initially increases with the rising SnS_2 proportion, but subsequently decreases with further increases (Figure 7b; Figure S17b, Supporting Information). This initial increase in SnS_2 proportion enhances the amount of SnO_2–SnS_2 heterojunctions, thereby improving the NO_2 response. However, beyond the optimal SnO_2–SnS_2 heterojunction amount, further increase in the SnS_2 proportion hinders the effective diffusion of NO_2 molecules into the heterojunction interfaces and inner SnO_2 nanoparticles, resulting in a decline in NO_2 sensing response.

Second, the strong hydrophobicity of the SnO_2–SnS_2–SiO_2/SiO_2-S-400 membrane is also a crucial factor contributing to its enhanced room-temperature NO_2 gas-sensing performance. As demonstrated in Figure S18 (Supporting Information), the response losses for the SnO_2–SnS_2–SiO_2/SiO_2-S-400 and SnO_2–SiO_2/SiO_2-Ar-400 sensors are 28.0% and 60.0%, respectively, when the RH increases from 40% to 85%. Remarkably, the SnO_2–SnS_2–SiO_2/SiO_2-S-400 sensor exhibits only a 5.7% response loss as RH rises from 40% to 70%. The lower response loss of the SnO_2–SnS_2–SiO_2/SiO_2-S-400 sensor, compared to the SnO_2–SiO_2/SiO_2-Ar-400 sensor, is primarily attributed to its enhanced hydrophobicity. After thermal sulfidation treatment, Sn—S chemical bonds are established on the surface of SnO_2 NFs. These sulfur atoms impede the formation of hydrogen bonds by H_2O molecules on the surface of SnO_2–SnS_2 NFs, thereby inhibiting the creation of H_2O sublayers on the membrane surface.[27] As a result, R_a remains relatively stable for the SnO_2–SnS_2–SiO_2/SiO_2-S-400 sensor across different RH levels. Additionally, this hydrophobic characteristic prevents H_2O molecules from occupying the active sites on the SnO_2–SnS_2 NFs. When NO_2 is introduced, the interference from surface-adsorbed H_2O molecules on gas-sensing reactions, especially at high RH, is greatly reduced. This high moisture resistance effectively ensures the sensitive response of the SnO_2–SnS_2–SiO_2/SiO_2-S-400 sensor toward ultralow concentrations of NO_2 in high RH environments.

3. Conclusion

In summary, we have developed a highly flexible, breathable, and hydrophobic all-inorganic self-supporting SnO_2–SnS_2–SiO_2/SiO_2 NF gas sensor using a straightforward electrospinning technique combined with a thermal sulfidation approach. This novel sensor presented a bilayer architecture, with the bottom substrate layer consisting of an amorphous SiO_2 NF membrane, and the top active layer comprising interwoven amorphous SiO_2 and SnO_2–SnS_2 NFs. The relatively low elastic modulus of the amorphous SiO_2 NFs as well as the three-dimensional network interwoven structure conferred superior mechanical flexibility to

© 2024 Wiley-VCH GmbH

16163028, 2025, 4, Downloaded from https://advanced.onlinelibrary.wiley.com/doi/10.1002/adfm.202410833 by Nanjing Tech University, Wiley Online Library on [31/01/2025]. See the Terms and Conditions (https://onlinelibrary.wiley.com/terms-and-conditions) on Wiley Online Library for rules of use; OA articles are governed by the applicable Creative Commons License

16163028, 2025, 4, Downloaded from https://advanced.onlinelibrary.wiley.com/doi/10.1002/adfm.202410833 by Nanjing Tech University, Wiley Online Library on [31/01/2025]. See the Terms and Conditions (https://onlinelibrary.wiley.com/terms-and-conditions) on Wiley Online Library for rules of use; OA articles are governed by the applicable Creative Commons License

the sensor. Exceptional flexible stability was demonstrated with only a very small resistance variation of 5% after 140 repetitive cycles at a large bending angle of 140°. The all-nanofiber-based porous structure imparted high gas permeability to the sensor. The formation of a SnO_2–SnS_2 active layer featuring an n–n heterojunction structure and a hydrophobic surface through in situ thermal sulfidation at an optimal temperature of 400 °C contributed to the excellent sensing performances (sensitivity, selectivity, and cycling stability) of the sensor under high RH of 85%. In particular, the sensor can selectively detect ultralow concentrations of NO_2 (≈25 ppb) with high cycling stability (>10 000 cycles at a 140° bending angle) in simulated human exhaled breath environments, highlighting its potential for wearable applications in noninvasive asthma-related disease diagnosis. This work breaks through the limitations of traditional organic substrate-based flexible gas sensors, offering a universal strategy for developing flexible, breathable, and moisture-resistant inorganic semiconductor sensors for wearable breath-sensing applications.

4. Experimental Details

Preparation of Flexible SnO_2–SiO_2/SiO_2 NF Membrane: The flexible SnO_2–SiO_2/SiO_2 NF membrane was fabricated via a two-spinneret electrospinning technique, as illustrated in Figure 1. Initially, 60.0 mg of H_3PO_4 (75 wt%) and 5.0 mL of tetraethyl orthosilicate were dissolved in 5.0 mL of H_2O and stirred for 4 h. Subsequently, 10.0 g of polyvinyl alcohol solution (10 wt.%) was added to the solution and stirred for 8 h (Si sol). 1.0 g of $SnCl_2$·$2H_2O$ and 0.7 g of polyacrylonitrile were dissolved in 10 mL *N*,*N*-dimethylformamide and stirred in an oil bath at 80 °C for 2 h (Sn sol). The Si sol and Sn sol were then separately transferred into two plastic syringes and mounted onto the electrospinning machine. Parameters such as roller speed, collecting distance, negative voltage, positive voltage, and temperature were set at 200 r min^{-1}, 15 cm, −2.5 and 19 kV, and 50 °C, respectively. During the initial 4 h, Si sol was electrospun at 1.2 mL h^{-1}. Over the subsequent 2.5 h, Si sol and Sn sol were electrospun simultaneously via two spinnerets at 0.2 and 0.8 mL h^{-1}, respectively. The resulting electrospun NF membrane was calcined for 1.5 h at 650 °C in a muffle furnace to produce the flexible SnO_2–SiO_2/SiO_2 NF membrane. SiO_2 NFs and SnO_2 NFs were fabricated by electrospinning using only Si sol and Sn sol, respectively, with the remaining production stages identical to those for the SnO_2–SiO_2/SiO_2 NF membrane.

Preparation of Flexible SnO_2–SnS_2–SiO_2/SiO_2 NF Membranes: The SnO_2–SnS_2–SiO_2/SiO_2 NF membranes were obtained through thermal sulfidation of the SnO_2–SiO_2/SiO_2 NF membranes. This procedure was conducted in a two-furnace system with 0.9 g of sulfur powder and SnO_2–SiO_2/SiO_2 NF membranes (30 mm × 50 mm) as precursors, with Ar as the carrier gas at atmospheric pressure. The sulfur powder was positioned in the center of furnace 1, while the SnO_2–SiO_2/SiO_2 NF membranes were placed in the center of furnace 2. To minimize O_2 in the tube furnace, air was first evacuated with a mechanical pump and then filled with Ar. The evacuation and purging steps were repeated three times. Furnace 1 was heated to 250 °C at a ramp rate of 20 °C min^{-1}, and furnace 2 was heated to 400 °C at the same ramp rate. After maintaining furnace 2 at 400 °C for 15 min, both furnaces were allowed to cool naturally to room temperature, with a continuous 100 sccm Ar flow throughout the process. The calcined samples were collected and labeled as SnO_2–SnS_2–SiO_2/SiO_2-S-400. To determine the optimal thermal sulfidation temperature, SnO_2–SiO_2/SiO_2 NF membranes underwent thermal sulfidation at 300, 350, 450, and 500 °C, while the other steps remained identical to those for SnO_2–SnS_2–SiO_2/SiO_2-S-400. The resulting samples were labeled as SnO_2–SnS_2–SiO_2/SiO_2-300, SnO_2–SnS_2–SiO_2/SiO_2-S-350, SnO_2–SnS_2–SiO_2/SiO_2-S-450, and SnO_2–SnS_2–SiO_2/SiO_2-S-500, respectively. The similar thermal sulfidation was also conducted on SnO_2 NFs at 400 °C, and the resultant sample was labeled as SnO_2–SnS_2-S-400.

Characterizations: Crystal phases were analyzed using XRD (D8 Advance, Bruker, Germany). UPS and elemental chemical states were examined using XPS (ESCALAB MKII, VG Scientific, UK). Morphologies and structures were characterized using a SEM (SU8220, HITACHI) and TEM (Tecnai G^2 F20, FEI). Raman spectra were characterized using a LabRam Aramis Raman Spectrometer (LabRAM Odyssey, Horiba) excited by a 532 nm laser.

Fabrication and Measurement of Flexible Self-Supporting Sensors: The flexible sensors were created by depositing Au interdigital electrodes on the surface of NF membranes using a mask. A photograph of the mask and detailed parameters, including the width and spacing of the interdigital electrodes (0.8 mm), are shown in Figure S19 (Supporting Information). The Au interdigital electrodes were prepared by depositing for 200 s on an ion sputter coater (BAL-TAC, Baltek Inc.). Gas-sensing performance was evaluated by an AES-4SD flexible gas-sensing analysis system (Beijing Elite Tech Co., Ltd, China), with its test chamber shown in Figure S20 (Supporting Information). Target gas flow rate, concentrations, and RH were controlled using a dynamic gas and liquid distribution system (DGL-III) from the same company, as shown by the schematic diagram of this system in Figure S21 (Supporting Information). The target gas flow was introduced and maintained for 300 s. Response is calculated as $S = R_g/R_g$ for reductive gases and $S = R_g/R_a$ for oxidative gases, where R_a and R_g represent the sensor's resistances in air and in the target gas, respectively.

Supporting Information

Supporting Information is available from the Wiley Online Library or from the author.

Acknowledgements

J.L. and J. Z. contributed equally to this work. This work was supported by the National Natural Science Foundation of China (Nos. 12374186, 12074236, and 62304180), the Fundamental Research Funds for the Central Universities (Nos. GK202301001, GK202203002, and LHRCCX23147).

Conflict of Interest

The authors declare no conflict of interest.

Data Availability Statement

The data that support the findings of this study are available in the supplementary material of this article.

Keywords

all-inorganic, biomarker, flexible, gas sensor, hydrophobic, NO_2

Received: June 20, 2024
Revised: August 12, 2024
Published online: September 2, 2024

[1] S. M. Majhi, A. Mirzaei, H. W. Kim, S. S. Kim, T. W. Kim, *Nano Energy* **2021**, *79*, 105369.
[2] X. Li, Y. Tang, W. Cheng, Y. Liu, H. Dong, X. Li, C. Shao, S. Fu, Y. Liu, *Chem. Soc. Rev.* **2023**, *455*, 140768.

 © 2024 Wiley-VCH GmbH

[3] H. Park, D. H. Kim, B. S. Ma, E. Shin, Y. Kim, T. S. Kim, F. S. Kim, I. D. Kim, B. J. Kim, *Adv. Sci.* **2022**, *9*, 2200270.
[4] Z. Liu, J. Xu, D. Chen, G. Shen, *Chem. Soc. Rev.* **2015**, *44*, 161.
[5] C. Han, X. Li, Y. Liu, Y. Tang, M. Liu, X. Li, C. Shao, J. Ma, Y. Liu, *Adv. Sci.* **2021**, *8*, 2102471.
[6] a) M. Yao, W. Li, G. Xu, *Coord. Chem. Rev.* **2021**, *426*, 213479; b) C. K. Nguyen, P. D. Taylor, A. Zavabeti, H. Alluhaybi, S. Almalki, X. Guo, M. Irfan, M. A. Kobaisi, S. J. Ippolito, M. J. S. Spencer, S. Balendhran, A. Roberts, T. Daeneke, K. B. Crozier, Y. Sabri, N. Syed, *Adv. Funct. Mater.* **2024**, *34*, 2309342; c) S. Y. Jeong, Y. K. Moon, J. K. Kim, S. W. Park, Y. K. Jo, Y. C. Kang, J. H. Lee, *Adv. Funct. Mater.* **2020**, *31*, 2007895.
[7] R. Alrammouz, J. Podlecki, P. Abboud, B. Sorli, R. Habchi, *Sens. Actuator A-Phys.* **2018**, *284*, 209.
[8] a) X. Zheng, S. Zhang, M. Zhou, H. Lu, S. Guo, Y. Zhang, C. Li, S. C. Tan, *Adv. Funct. Mater.* **2023**, *33*, 2214880; b) M. Chao, P. Di, Y. Yuan, Y. Xu, L. Zhang, P. Wan, *Nano Energy* **2023**, *108*, 108201; c) Y. Zhou, Y. Zhang, Y. Zhou, L. Zhao, F. Liu, X. Yan, P. Sun, G. Lu, *Nano Energy* **2023**, *117*, 108881.
[9] a) A. Vasilescu, B. Hrinczenko, G. M. Swain, S. F. Peteu, *Biosens. Bioelectron.* **2021**, *182*, 113193; b) T. Hibbard, A. J. Killard, *Crit. Rev. Anal. Chem.* **2011**, *41*, 21; c) A. Sett, T. Rana, U. Rajaji, R. Sha, T. Liu, T. K. Bhattacharyya, *Sens. Actuator A-Phys.* **2022**, *338*, 113507; d) A. Hermawan, T. Amrillah, A. Riapanitra, W. J. Ong, S. Yin, *Adv. Healthcare Mater.* **2021**, *10*, 2100970.
[10] S. Das, M. Pal, *J. Electrochem. Soc.* **2020**, *167*, 037562.
[11] K. H. Kim, S. A. Jahan, E. Kabir, *Trac-Trends Anal. Chem.* **2012**, *33*, 1.
[12] a) J. Liu, W. Wang, G. Li, X. Bian, Y. Liu, J. Zhang, J. Gao, C. Wang, B. Zhu, H. Lu, *J. Alloy. Compd.* **2023**, *934*, 167950; b) J. Hu, C. Zou, Y. Su, M. Li, Y. Han, E. S.-W. Kong, Z. Yang, Y. Zhang, *J. Mater. Chem. A* **2018**, *6*, 17120; c) D. Wang, D. Zhang, J. Guo, Y. Hu, Y. Yang, T. Sun, H. Zhang, X. Liu, *Nano Energy* **2021**, *89*, 106410.
[13] K. Suematsu, M. Sasaki, N. Ma, M. Yuasa, K. Shimanoe, *ACS Sens.* **2016**, *1*, 913.
[14] S. Y. Jeong, J. S. Kim, J. H. Lee, *Adv. Mater.* **2020**, *32*, 2002075.
[15] C. Kwak, T. Kim, S. Jeong, J. Yoon, J. Kim, J. Lee, *ACS Appl. Mater. Interfaces* **2018**, *10*, 18886.
[16] J. Kim, C. W. Na, C. Kwak, H. Li, J. W. Yoon, J. Kim, S. Jeong, J. Lee, *ACS Appl. Mater. Interfaces* **2019**, *11*, 25322.
[17] N. Ma, K. Suematsu, M. Yuasa, T. Kida, K. Shimanoe, *ACS Appl. Mater. Interfaces* **2015**, *7*, 5863.
[18] K. Choi, H. Kim, Y. C. Kang, J. Lee, *Sens. Actuator B-Chem.* **2014**, *194*, 371.
[19] J. W. Yoon, J. S. Kim, T. H. Kim, Y. J. Hong, Y. C. Kang, J. H. Lee, *Small* **2016**, *12*, 4229.
[20] W. Liu, L. Xu, K. Sheng, C. Chen, X. Zhou, B. Dong, X. Bai, S. Zhang, G. Lu, H. Song, *J. Mater. Chem. A* **2018**, *6*, 10976.
[21] Z. Yang, L. Jiang, J. Wang, F. Liu, J. He, A. Liu, S. Lv, R. You, X. Yan, P. Sun, C. Wang, Y. Duan, G. Lu, *Sens. Actuator B-Chem.* **2021**, *326*, 128828.
[22] a) Y. Zhang, S. Liu, J. Yan, X. Zhang, S. Xia, Y. Zhao, J. Yu, B. Ding, *Adv. Mater.* **2021**, *33*, 2105011; b) J. Cho, Q. Li, H. Wang, Z. Fan, J. Li, S. Xue, K. S. N. Vikrant, H. Wang, T. B. Holland, A. K. Mukherjee, R. E. García, X. Zhang, *Nat. Commun.* **2018**, *9*, 2063; c) X. Mao, H. Shan, J. Song, Y. Bai, J. Yu, B. Ding, *CrystEngComm* **2016**, *18*, 1139.
[23] a) C. Wang, Y. Wu, L. Tsai, H. Lee, Y. Hsu, *Nanotechnology* **2021**, *32*, 305706; b) K. Michalec, A. Kusior, A. Mikuła, M. Radecka, *Mater. Res. Lett.* **2021**, *9*, 445; c) D. Chao, P. Liang, Z. Chen, L. Bai, H. Shen, X. Liu, X. Xia, Y. Zhao, S. V. Savilov, J. Lin, Z. X. Shen, *ACS Nano* **2016**, *10*, 10211.
[24] a) T. B. Limbu, B. Adhikari, S. K. Song, B. Chitara, Y. Tang, G. N. Parsons, F. Yan, *RSC Adv.* **2021**, *11*, 38839; b) M. Khajehzadeh, N. Ehsani, H. R. Baharvandi, A. Abdollahi, M. Bahaaddini, A. Tamadon, *Ceram. Int.* **2020**, *46*, 26970.
[25] X. Tian, X. Cui, Y. Xiao, T. Chen, X. Xiao, Y. Wang, *ACS Appl. Mater. Interfaces* **2023**, *15*, 9604.
[26] B. Huang, Q. Zhu, H. Xu, X. Li, X. Li, X. Li, *Sens. Actuator B-Chem.* **2023**, *380*, 133303.
[27] a) Y. Huang, W. Jiao, Z. Chu, S. Wang, L. Chen, X. Nie, R. Wang, X. He, *ACS Appl. Mater. Interfaces* **2019**, *12*, 997; b) J. M. Suh, T. H. Lee, K. Hong, Y. G. Song, S. H. Cho, C. Kang, Y. Shim, D. Lee, K. C. Kwon, H. W. Jang, *Sens. Actuator B-Chem.* **2022**, *369*, 132319.
[28] J. Sun, L. Chen, X. Zhang, S. Miao, *IEEE Sens. J.* **2024**, *24*, 86.
[29] a) Y. Zhang, Y. Jiang, Z. Yuan, B. Liu, Q. Zhao, Q. Huang, Z. Li, W. Zeng, Z. Duan, H. Tai, *Small* **2023**, *19*, 2303631; b) J. Liu, Q. Yu, Y. Liu, X. Zhang, Z. Yang, X. Yin, H. Lu, J. Zhang, J. Gao, B. Zhu, *J. Mater. Chem. A* **2024**, *12*, 17432; c) R. Zhao, T. Wang, M. Zhao, C. Xia, X. Zhao, Y. An, X. Dai, *Phys. Chem. Chem. Phys.* **2017**, *19*, 10470.
[30] J. Zhang, D. Leng, L. Zhang, G. Li, F. Ma, J. Gao, H. Lu, B. Zhu, *J. Alloy. Compd.* **2021**, *853*, 157339.
[31] W. Li, R. Chen, W. Qi, L. Cai, Y. Sun, M. Sun, C. Li, X. Yang, L. Xiang, D. Xie, T. Ren, *ACS Sens.* **2019**, *4*, 2809.
[32] W. Ma, Y. Fu, G. Meng, W. Tan, Y. Wang, J. Tan, *Sens. Actuator B-Chem.* **2023**, *381*, 133429.
[33] K. Rui, X. Wang, M. Du, Y. Zhang, Q. Wang, Z. Ma, Q. Zhang, D. Li, X. Huang, G. Sun, J. Zhu, W. Huang, *ACS Appl. Mater. Interfaces* **2018**, *10*, 2837.
[34] H. Zhao, W. Ge, X. Li, T. Zhao, Z. Luo, R. Wang, S. Wang, S. Shang, Q. Zhang, H. DiWu, H. Lu, J. Zhang, J. Liu, *Chem. Soc. Rev.* **2023**, *474*, 145915.
[35] S. Fan, G. Lu, Y. Hu, W. Zheng, X. Liu, J. Zhang, *Sens. Actuator B-Chem.* **2023**, *396*, 134549.
[36] W. Quan, J. Shi, H. Luo, C. Fan, W. Lv, X. Chen, M. Zeng, J. Yang, N. Hu, Y. Su, H. Wei, Z. Yang, *ACS Sens.* **2023**, *8*, 103.
[37] W. Zhang, W. Wang, Y. Wang, L. Sun, Y. Sun, J. Hu, *Appl. Surf. Sci.* **2023**, *637*, 157957.
[38] Y. Wang, C. Tang, M. Su, Y. Ji, L. Xie, Q. Yang, A. Du, Y. Zhou, J. Yang, *Chem. Soc. Rev.* **2023**, *34*, 107981.
[39] S. J. Choi, H. J. Choi, W. T. Koo, D. Huh, H. Lee, I. D. Kim, *ACS Appl. Mater. Interfaces* **2017**, *9*, 40593.
[40] B. Wang, A. Thukral, Z. Xie, L. Liu, X. Zhang, W. Huang, X. Yu, C. Yu, T. J. Marks, A. Facchetti, *Nat. Commun.* **2020**, *11*, 369.
[41] R. You, D. Han, F. Liu, Y. L. Zhang, L. Geyu, *Sens. Actuator B-Chem.* **2018**, *277*, 114.
[42] J. Zhang, J. Liu, Y. Liu, G. Li, J. Guo, J. Zhang, Q. Zhao, J. Che, L. Li, J. Gao, H. Lu, *Sens. Actuator B-Chem.* **2024**, *400*, 134941.

© 2024 Wiley-VCH GmbH

16163028, 2025, 4, Downloaded from https://advanced.onlinelibrary.wiley.com/doi/10.1002/adfm.202410833 by Nanjing Tech University, Wiley Online Library on [31/01/2025]. See the Terms and Conditions (https://onlinelibrary.wiley.com/terms-and-conditions) on Wiley Online Library for rules of use; OA articles are governed by the applicable Creative Commons License

ACS APPLIED MATERIALS & INTERFACES

Research Article

Cite This: *ACS Appl. Mater. Interfaces* 2018, 10, 39327−39335

www.acsami.org

Synergistic Effect of Doping and Compositing on Photocatalytic Efficiency: A Case Study of $La_2Ti_2O_7$

Zuju Ma,[†,§] Yangwen Li,[†] Yaohui Lv,[†] Rongjian Sa,[‡] Qiaohong Li,[∥] and Kechen Wu*[,‡]

[†]School of Materials Science and Engineering, Anhui University of Technology, Maanshan 243002, China

[‡]Minjiang University, Fuzhou 350108, China

[§]State Key Laboratory of Photocatalysis on Energy and Environment, Fuzhou University, Fuzhou 350116, China

[∥]State Key Laboratory of Structural Chemistry, Fujian Institute of Research on the Structure of Matter, Chinese Academy of Sciences, Fuzhou 350002, China

Supporting Information

ABSTRACT: Charge generation and separation are two key issues in developing a high-efficiency semiconductor for the visible-light-driven photocatalysis. Here, we use the layered perovskite-type wide-gap semiconductor $La_2Ti_2O_7$ (LTO) as a model to systematically explore the synergistic effect of doping (with sulfur or nitrogen) and heterojunction (with graphitic C_3N_4) on improving visible light absorption and photoexcited charge separation by means of density functional theory calculations. It is found that the anion (N or S) doping into the LTO(010) surface can not only shift the optical absorption edge to the visible region, but also creates some partially occupied or unoccupied states in the band gap that would facilitate the formation of recombination centers. For the purpose of promoting electron−hole separation, the (N or S-doped) LTO(010) surfaces were hybridized with the monolayer g-C_3N_4. Interestingly, we found that the (S-doped) LTO/g-C_3N_4 heterostructure forms a type-II heterojunction, with the valence band maximum residing in the (S-doped) LTO and the conduction band minimum in g-C_3N_4, respectively. This band alignment feature facilitates efficient electron−hole separation. Moreover, we found that the S-doped LTO/g-C_3N_4 composite has a short interfacial distance (about 2.1 Å), implying that the interfacial interaction of this composite might be a chemical bond rather than a weak van der Walls interaction. The chemical bonding can enhance charge separation. Our theoretical findings provide design principles for optimizing the photocatalytic performance of the wide-gap photocatalysts and demonstrate that the S-doped LTO/g-C_3N_4 composite would be a potential candidate for the photocatalysis of water splitting.

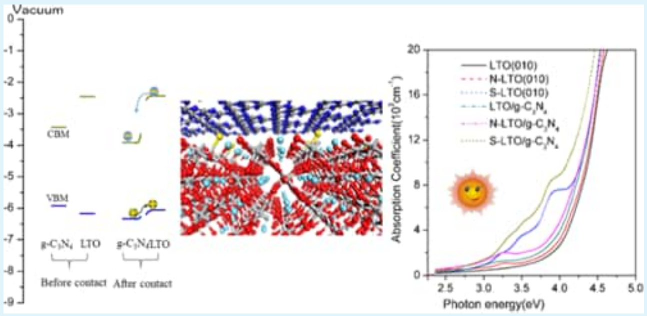

KEYWORDS: *photocatalysis, doping, composite, density functional theory, water splitting*

Downloaded via NANJING TECH UNIV on February 1, 2025 at 06:45:56 (UTC).
See https://pubs.acs.org/sharingguidelines for options on how to legitimately share published articles.

1. INTRODUCTION

Hydrogen is considered as an alternative energy resource to fossil fuels and a promising solution to environmental problems. It can be obtained from renewable water and solar light via semiconductor-based photocatalytic water splitting.[1−8] In the past few decades, many inorganic and organic semiconductor materials have been developed as photocatalysts for water splitting driven by UV light and/or visible light.[9] These photocatalysts, however, still suffer from low quantum efficiency or poor stability under visible light that comprises almost 46% of the solar spectrum. It is generally accepted that large number of photoexcited carriers and effective separation of the photoexcited electrons and holes are two keys to develop high-efficiency visible-light-driven photocatalysts for water splitting.[9]

In the charge generation process, the semiconductor photocatalyst should first possess a relatively small band gap (1.23 eV < E_g < 2.5 eV) for efficient visible-light harvesting. Unfortunately, the reported semiconductor photocatalysts with high photocatalytic activities for hydrogen generation from water splitting, such as n-type TiO_2,[10] $NaTaO_3$,[11] $La_2Ti_2O_7$,[12] and Sr_2NbO_7,[13] mostly have large band gaps (E_g > 3 eV), rendering them only active under UV light (<400 nm) that comprises just 3% of the solar spectrum. Metal or/and nonmetal ion doping is one of the most widely used approaches to making photocatalysts visible-light active. The reports include doped Ti-based oxides, doped Ta-based oxides, doped Zr-based oxides, and so on.[9] While doping with foreign atom species may narrow the band gap of the photocatalyst, the impurities easily create localized states in the forbidden gap and act as charge carrier recombination centers, which would strongly affect the efficiency of the photocatalytic water-

Received: July 19, 2018
Accepted: October 24, 2018
Published: October 24, 2018

© 2018 American Chemical Society

DOI: 10.1021/acsami.8b12178
ACS Appl. Mater. Interfaces 2018, 10, 39327−39335

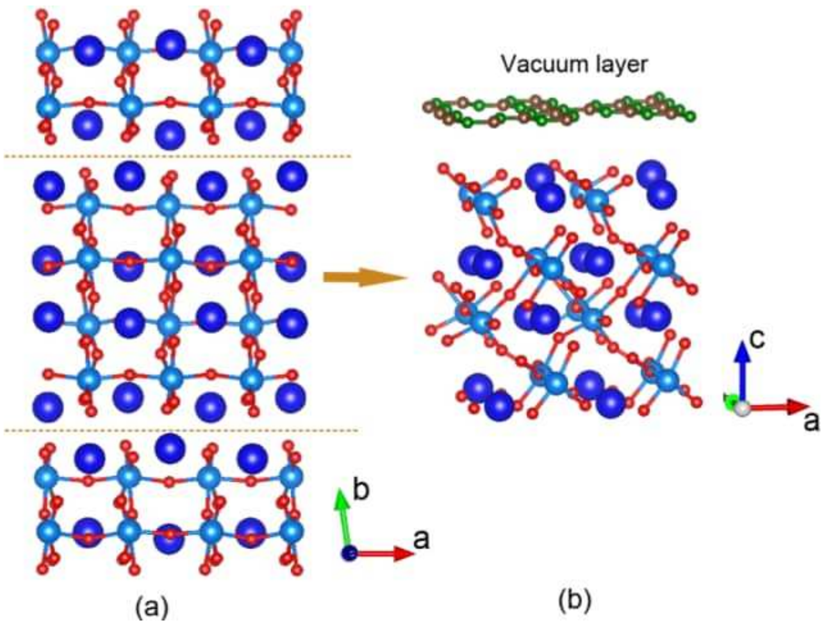

Figure 1. (a) Bulk LTO with a layered perovskite-type structure; (b) the simulated interface between the g-C_3N_4 and the LTO(010) surface model. The atoms are represented by spheres: O, red; La, blue; Ti, indigo blue; N, green; and C, brown.

splitting process. Several approaches have been used to separate the photogenerated electrons and holes, including loading with the cocatalyst, combining with other semiconductor material to form a composite, and modifying the crystal structure and morphology.[9] Of these methods, the approach of constructing composites is an effective strategy for improving photocatalytic activity through efficient charge separation and fast charge transport. In this work, we aim to explore the synergistic effect of doping and heterojunction on the light absorption and charge separation of a wide band gap semiconductor, $La_2Ti_2O_7$ (LTO).

LTO, a layered perovskite-type compound, has received much attention due to its superior photocatalytic activities in the water-splitting reaction.[12,14,15] Unfortunately, the band gap of LTO (3.29−3.82 eV[16−19]) is still too large to absorb the visible light. Considerable efforts have been devoted to narrow its band gap. Meng et al. found that N-doped LTO (N-LTO) nanosheets showed good visible-light photocatalytic activity towards the decomposition of methyl orange.[20] Our previous theoretical research on N-LTO demonstrated that both the dispersed substitutional N atoms at O sites and O vacancy are necessary to shift the valence band (VB) edge upward instead of forming donor levels in the forbidden band.[21] In addition, the doped LTO usually has poor electron mobility. To prolong the life-time of charge carriers, some attentions are devoted to the LTO-based composites, in which the photogenerated electrons and holes could transfer to the opposite direction, resulting in the decrease of recombination.[22−27] Recently, graphitic C_3N_4 (g-C_3N_4)-based composites have attracted intensive interest due to their enhanced vis-light photocatalytic activity compared with g-C_3N_4. These composites include g-C_3N_4/carbon dots,[28] g-C_3N_4/graphene,[29] g-C_3N_4/TiO_2,[30] g-C_3N_4/reduced graphene oxide,[31,32] g-C_3N_4/MoS_2,[33,34] g-C_3N_4/Ag_3PO_4,[35] g-C_3N_4/WO_3,[36] g-C_3N_4/Ag_2O,[37] g-C_3N_4/CeO_2,[38] and so on. More recently, a hybrid N-LTO/g-C_3N_4 nanocomposite has been successfully synthesized, showing an enhanced UV and vis-light photocatalytic activity for water splitting and dye degradation.[23] However, the underlying mechanism for such an improvement remains unclear, and the interfacial electronic structure of the composite is also unexplored. One purpose of this work is to explore the relationship between this enhanced photocatalytic activity and the interfacial microstructure of the N-LTO/g-C_3N_4 composite.

Moreover, Liu et al. have theoretically investigated the effect of anionic doping (N, S, P, and C) and codoping on the geometrical and electronic structure of bulk LTO.[5] They found that a clean and narrow band gap can be obtained by doping S atoms in bulk LTO as the S 3p orbitals are appearing just above the valence band maximum (VBM) edge. By contrast, the donor/acceptor states appear in the band gap of LTO after N, P, or C doping. Due to the appealing band structure of S-doped LTO (S-LTO), we propose that the S-LTO/g-C_3N_4 composite may exhibit better performance than the LTO/g-C_3N_4 and N-LTO/g-C_3N_4 composites. In the present work, large-scale density functional theory (DFT) computations including long-range dispersion correlation have been carried out to accurately predict the structural and electronic properties of the hybrid LTO (N-LTO, S-LTO)/g-C_3N_4 composites. The interfacial interaction was comparatively studied. We demonstrated that the composites can harvest a longer wavelength of the visible light spectrum compared to the pristine LTO(010) surface for visible light photocatalysis. Importantly, we found that the interfacial interaction between the S-LTO(010) slab and g-C_3N_4 might be a chemical bond rather than the van der Walls (vdW) interaction, which can enhance charge separation. In addition, a type-II heterostructure formed in the (S-)LTO/g-C_3N_4 composites is also beneficial for efficient photoinduced charge separation.

2. COMPUTATIONAL DETAILS

Given that LTO has a layered perovskite-type structure, reducing its dimension to an ultrathin nanosheet with few-layer thickness is feasible.[20] To construct the composite of LTO and g-C_3N_4, we cleave a stoichiometric LTO(010) slab from the bulk LTO, as shown in Figure 1a,b. No dangling bond is

DOI: 10.1021/acsami.8b12178
ACS Appl. Mater. Interfaces 2018, 10, 39327−39335

introduced on the top surface of the slab model. An optimized planar g-C_3N_4 layer containing four g-C_3N_4 units sits on a 2 × 1 nine atomic layer stoichiometric LTO(010) slab containing 88 atoms with three bottom layers fixed at the bulk position and is followed by a 15 Å thick vacuum layer to avoid artificial interaction. The lattice constants (a = 11.159 Å, b = 7.811 Å) of the LTO(010) layer were kept fixed in constructing the composites. The lattice constants (a = 12.358 Å, b = 7.135 Å) of the g-C_3N_4 layer were slightly rescaled to match that of the LTO(010) layer. The reason for fixing the LTO slab and relaxing g-C_3N_4 is that in experiment, the LTO slab is much thicker than the monolayer g-C_3N_4, and this makes the lattice constants of g-C_3N_4 more susceptible to the interfacial stress than the bulk LTO.

Our calculations on the pristine g-C_3N_4 monolayer, (N-, S-)LTO(010) slabs, and the hybrid (N-, S-)LTO/g-C_3N_4 composites were performed using the plane-wave basis Vienna ab initio simulation package (VASP) code.[39−41] The projected augmented wave[42] potentials with the valence states 2s and 2p for O, N, and C, 3s and 3p for S, 3d and 4s for Ti, 5s, 5p, 5d, and 6s for La were used to treat the core-valence interaction. The Perdew−Burke−Ernzerhof (PBE) functional,[43] which has been proved to be accurate in predicting the lattice constants of g-C_3N_4 and bulk LTO,[32] was chosen for the structural optimization in this work. The long-range van der Walls (vdW) interaction are expected to be significant in accurately describing the geometry of the complex.[29] Herein, we used the Grimme's scheme[44] (D3) to treat the long-range dispersion interactions between the LTO slab and g-C_3N_4.[44] The dipole correction and spin-polarization were considered in all calculations. For systems with strongly correlated d/f electrons, the local density approximation and general gradient approximation (GGA) are generally unsuitable for predicting the electronic structure.[45,46] To correct this problem, we adopted two methods to calculate the electronic structures of the LTO slab, g-C_3N_4, and the composites. The first is the PBE + U method. Additional "U" term could improve the description of the d/f electronic system by replacing the onsite Coulomb interaction within the chosen functional with a Hubbard term.[47−51] The U value was set to be 5 eV for La f and Ti d states in LTO. This U value has been checked by several previous works.[17,21] The second is the hybrid functional HSE03. We found that the HSE03 functional can well reproduce the band gap of bulk LTO and g-C_3N_4. The obtained band gap of bulk LTO and g-C_3N_4 by the HSE03 functional are 3.85 and 2.50 eV, respectively (see Figure S1 in the Supporting Information), which are in good agreement with the experimental values (3.82 eV for LTO,[18] and 2.75 eV for g-C_3N_4[28]). The two methods were compared to confirm the results.

A Γ-centered 4 × 3 × 1 Monkhorst−Pack grid for the Brillouin zone sampling[52] and a cutoff energy of 450 eV for the plane-wave expansion were found to get convergent lattice parameters of the hybrid LTO/g-C_3N_4 composites. The convergence of force on each atom was set to 0.05 eV/Å.

To obtain the optical absorption properties, the complex dielectric function $\varepsilon(\omega) = \varepsilon_1(\omega) + i\varepsilon_2(\omega)$ has been determined in the random phase approximation from the PBE wavefunctions. The absorption coefficient $\eta(\omega)$ can be obtained based on ε_1 and ε_2.[53,54] The imaginary part of the dielectric function due to direct inter-band transitions is given by the expression[55,56]

$$\varepsilon_2(\hbar\omega) = \frac{2e^2\pi}{\Omega\varepsilon_0}\sum_{k,v,c}|\langle\psi_k^c|u\cdot r|\psi_k^v\rangle|^2\ \delta(E_k^c - E_k^v - E)$$

where, Ω, ω, u, ν, and c are the unit-cell volume, photon frequencies, the vector defining the polarization of the incident electric field, valence and conduction bands (CBs), respectively. The real part of the dielectric function is obtained from ε_2 by a Kramers−Kronig transformation.

3. RESULTS AND DISCUSSION

3.1. Monodoping in LTO(010) Surface. There have been some theoretical studies on the N-doped bulk LTO.[5,21] However, to the best of our knowledge, the doped LTO surface has not been reported theoretically. As the chemical reaction takes place on the surface of the photocatalyst, it is more desirable to study the photocatalytic mechanism based on the doped LTO surface. In this study, we choose N or S substituting on the O site in the LTO(010) surface as the dopants. As N and S atoms both have higher 2p-orbital energies than the O 2p orbitals, doping N or S atom would move the valence bands of LTO upwards, and thus reducing the bandgap.[5] In addition, S atom is isoelectronic as O atom, therefore, the total number of valence electrons of the system remains unchanged after S doping. Figure 2a,b show the most stable N-doped LTO(010) surface (N-LTO) and S-doped LTO(010) surface (S-LTO), respectively, in which both the N_O (N substitutes for O) and S_O atoms tend to locate at the surface of the LTO(010) surface. Closer inspection of the atomic position at the surfaces reveals that, the S_O atom in the top layer slightly moves upward about 0.73 Å, whereas the N_O atom in the second layer is almost undisplaced.

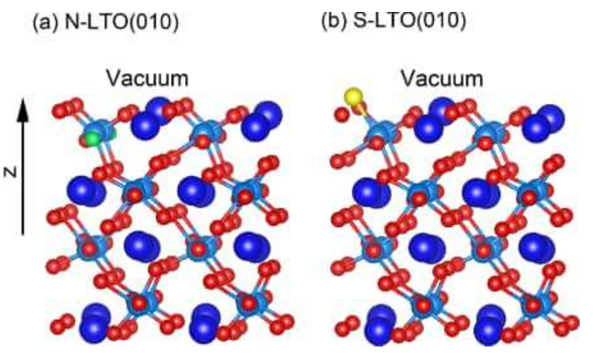

Figure 2. Atomistic representation of the N-doped LTO(010) surface model (a) and S-doped LTO(010) surface model (b). The atoms are represented by spheres: O, red; La, blue; Ti, indigo blue; N, green; S, yellow.

With the optimized structures, we estimated the formation energy for such doping by

$$E_{form} = E_t(D) - E_t(*) + \mu_X - \mu_Y \quad (1)$$

here, $E_t(D)$ and $E_t(*)$ are the total energies of doped and pure LTO, respectively, and μ_X and μ_Y are the atomic potential of the host and dopant atoms, respectively.[6] The calculated (using GGA/PBE) formation energies for the N-LTO and S-LTO are 2.75 and 2.31 eV, respectively. The isovalent S doping was found to be more favorable than the p-type N doping. The conclusion is consistent with the result of N and S-doped bulk LTO.[5]

DOI: 10.1021/acsami.8b12178
ACS Appl. Mater. Interfaces 2018, 10, 39327−39335

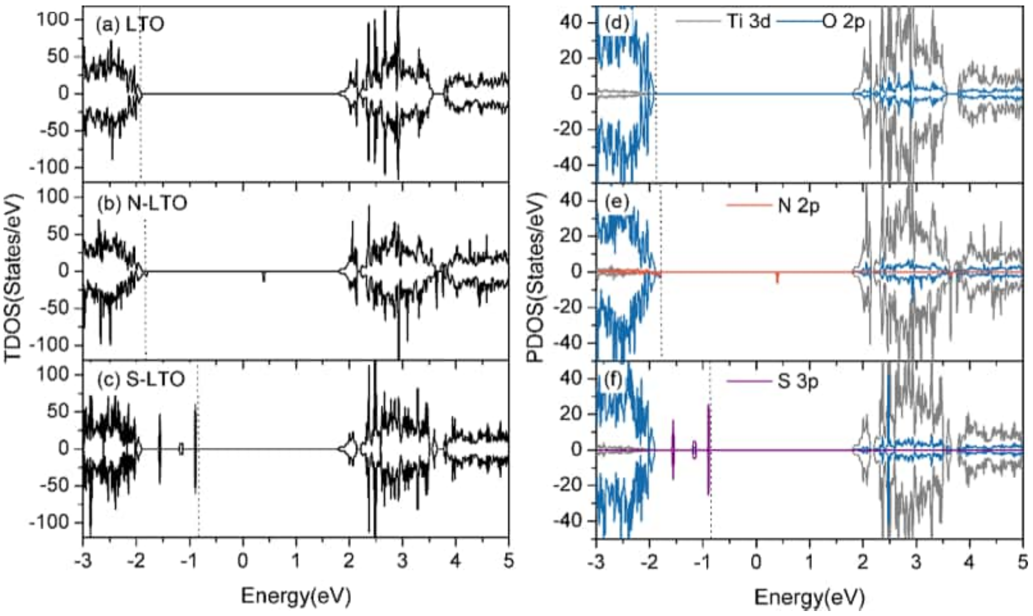

Figure 3. Total DOS and atomic partial DOS (PDOS) of the LTO(010) (a, d), N-doped LTO(010) (b, e), and S-doped LTO(010) (c, f) surface, respectively, calculated using the HSE03 functional. The vertical dash line indicates the Fermi level.

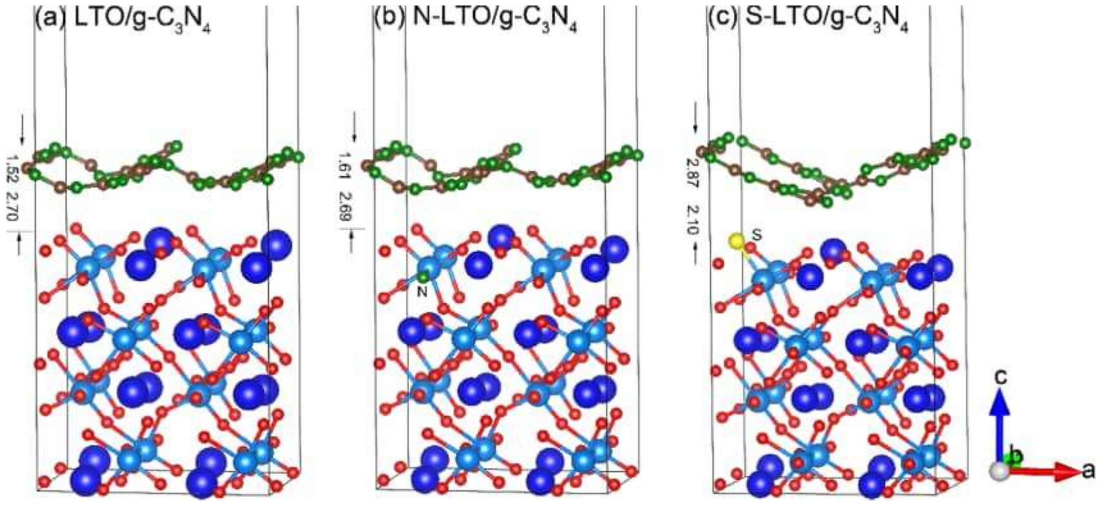

Figure 4. Side view structures of LTO/g-C_3N_4 (a), N-LTO/g-C_3N_4 (b), and S-LTO/g-C_3N_4 (c) heterostructures after geometry optimization. The distance is given in unit of Å.

Next, we investigated the effect of anionic (N or S) doping on the electronic structure of the LTO(010) surface. Figure 3a−c show the calculated total density of states (TDOS) by the HSE03 functional for the LTO(010), N-LTO, and S-LTO surfaces, respectively. The PBE + *U* method gives results similar to the HSE03 method, see Figure 2 in the Supporting Information. The TDOS in the different systems are aligned by referencing to the core levels of the O atom farthest from the impurity. The TDOS of the LTO(010) surface (Figure 3a) shows that the LTO(010) surface has a band gap of 3.71 eV, which is a little smaller than that of bulk LTO (3.85 eV). No surface states are induced inside the forbidden gap as there is no dangling bond (undercoordinated surface atom) on the top surface of the LTO slab model. In the N-LTO surface (Figure 3b), the N 2p orbitals create some unoccupied empty states above the Fermi level, see partial DOS (PDOS) in Figure 3e. Therefore, the effective band gap of the LTO(010) surface is reduced to 2.18 eV after N doping. The impurity states appearing in the band gap might act as an electron−hole recombination center, which will definitely suppress the photocatalytic activities. Our previous study on N-doped bulk LTO[21] suggests that at higher doping level, the localized N 2p states would extend to lower energies and merge into the edge of VBs. As the S atom is isoelectronic to O atom, a clean band gap is obtained after doping S atom in the LTO(010) surface. Because the S 3p orbitals have higher p-orbital energy than the O 2p orbitals, the VBM edge of S-LTO is moved upward by 1.01 eV and the conduction band minimum (CBM) remains nearly unchanged, as seen in Figure 3c,f. Therefore, the effective band gap of the S-LTO surface is reduced to 2.67 eV, which is an appropriate band gap value used in the visible light photocatalysis.

3.2. Hybrid with g-C_3N_4. Although metal or/and non-metal ions doping can effectively reduce the band gap and make photocatalysts visible-light active, the defects often act as electron−hole recombination centers and reduce the photo-

DOI: 10.1021/acsami.8b12178
ACS Appl. Mater. Interfaces 2018, 10, 39327−39335

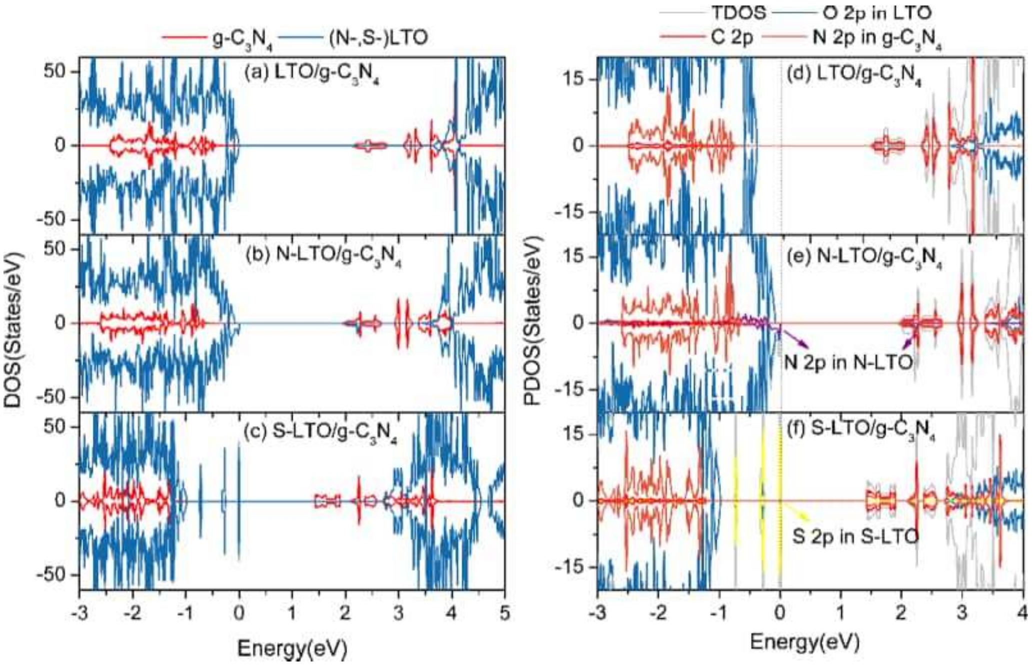

Figure 5. TDOS (a−c) and PDOS (d−f) of the (N-, S-)LTO/g-C_3N_4 composites calculated using the HSE03 functional. The Fermi level was set to zero.

catalytic efficiency.[9] Hybridizing with a two-dimensional material with a suitable electronic structure is an efficient approach to separate the photoexcited electron and hole. Here, we investigate three g-C_3N_4-based composites: LTO/g-C_3N_4, N-LTO/g-C_3N_4, and S-LTO/g-C_3N_4.

3.2.1. Structure and Stability. In our previous study of g-C_3N_4, we found that the buckled g-C_3N_4 monolayer is about 0.59 eV more stable than the planar form.[57] Similarly, in this work, g-C_3N_4 in the three composites also tend to be buckled. The distance between the uppermost and lowermost atoms in the buckled g-C_3N_4 (Figure 4a−c) is about 1.52, 1.61, and 2.87 Å in LTO/g-C_3N_4, N-LTO/g-C_3N_4, and S-LTO/g-C_3N_4, respectively. These values are larger than the value of 1.42 Å in the monolayer-buckled g-C_3N_4.[58] This suggests that the underlying (N-, S-)LTO(010) surfaces have a significant effect on the upper g-C_3N_4. In addition, the g-C_3N_4 in the S-LTO/g-C_3N_4 distorts most seriously, which might result from the upward S atom on the top layer of the S-LTO surface. The separation distance between the uppermost atom in the (N-, S-)LTO slab and the lowermost atom in g-C_3N_4 (Figure 4a−c) are calculated to be 2.70, 2.69, and 2.10 Å for LTO/g-C_3N_4, N-LTO/g-C_3N_4, and S-LTO/g-C_3N_4, respectively. These values, especially that of S-LTO/g-C_3N_4, are a little smaller than that of the other g-C_3N_4-based heterostructures, such as 3.01 Å for SnS_2/g-C_3N_4, 3.03 Å for graphene/g-C_3N_4,[29] and 2.97 Å for MoS_2/g-C_3N_4.[34] This implies that the interfacial interaction between S-LTO and g-C_3N_4 is the chemical bond rather than the typical weak vdW interaction. This type of layered heterojunction composed of different monolayers stacked together by chemical bonds can greatly enhance charge separation.[59] Closer inspection of the atomic position at the interface reveals that the S−Ti bond in the S-LTO surface is prolonged from 2.34 to 2.36 Å in S-LTO/g-C_3N_4. For N-LTO/g-C_3N_4 and LTO/g-C_3N_4, the atoms in the top layer of the (N-)LTO slab are nearly unchanged, implying the relatively weak interaction between the two components.

The thermal stability and interfacial interaction of the heterostructures can be described by the formation energy of the interface, which is defined as

$$E_F = E(\text{hetero}) - E(1) - E(2) \qquad (2)$$

where, E(hetero), E(1), and E(2) are the total energy of the heterostructure, component 1, and component 2, respectively. In general, a negative formation energy indicates that the heterojunction is favorable to form. The interfacial formation energy per unit area is calculated to be −54.3, −53.7, and −57.1 meV/Å^2 for LTO/g-C_3N_4, N-LTO/g-C_3N_4, and S-LTO/g-C_3N_4, respectively. We can see that all the three heterostructures are thermodynamically stable. Moreover, S-LTO/g-C_3N_4 is more stable than the other two composites due to the shortest interlayer distance in the S-LTO/g-C_3N_4 composite.

3.2.2. Electronic Structure. Next, we study the effect of the interfacial interaction between the two components on the electronic structure of the (N-, S-)LTO/g-C_3N_4 composites. As no correction (U) is adopted for g-C_3N_4, the PBE + U method will underestimate the band gap of g-C_3N_4. The TDOS and PDOS of the three composites were calculated by the HSE03 hybrid functional and are displayed in Figure 5a−f to analyze the electronic structure. From the TDOS of the LTO/g-C_3N_4 composite (Figure 5a), we see that the VBM of the composite is mainly dominated by the LTO region and is composed of O 2p states (Figure 5d), while the CBM is mainly contributed by N 2p and C 2p orbitals of the g-C_3N_4 region. A type-II heterostructure is formed between LTO and g-C_3N_4 with both the valence and conduction band edges of g-C_3N_4 below the corresponding LTO counterparts. Under the visible light irradiation, the electrons can be photoexcited from the VBs of both LTO and g-C_3N_4 to their corresponding CBs. Subsequently, the photoexcited electrons in the CBs of LTO would transfer to that of g-C_3N_4 due to the more positive CBM of g-C_3N_4 than that of LTO. In the meantime, the photoexcited holes in the VBs of g-C_3N_4 can be moved to the

DOI: 10.1021/acsami.8b12178
ACS Appl. Mater. Interfaces 2018, 10, 39327−39335

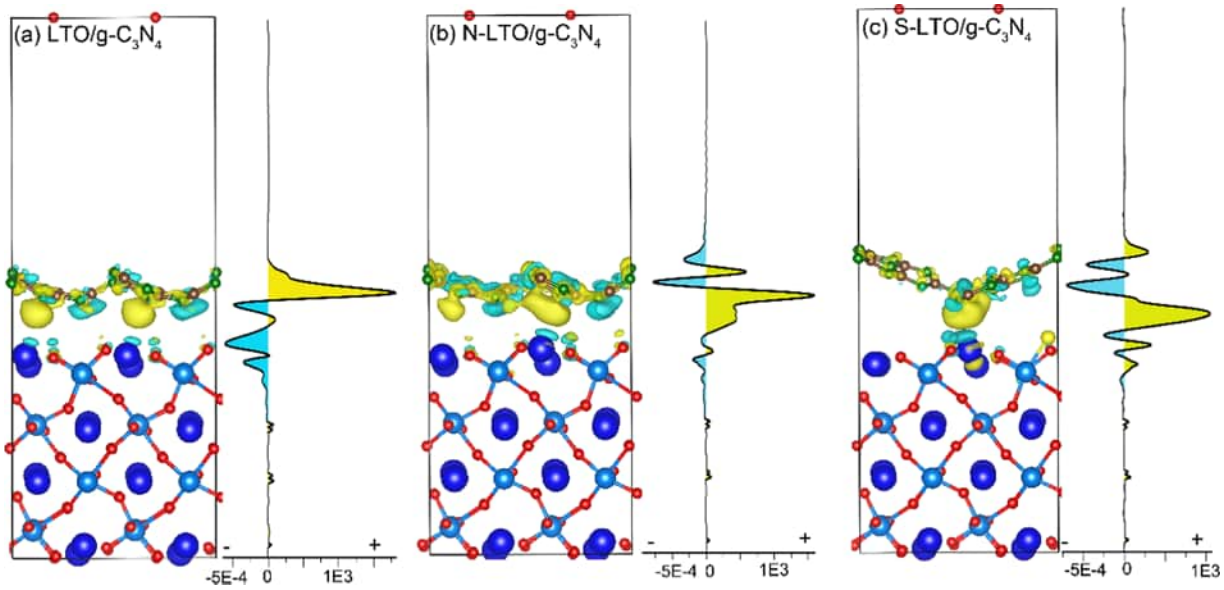

Figure 6. Charge density difference for (a) the LTO/g-C_3N_4 composite, (b) N-LTO/g-C_3N_4 composite, and (c) S-LTO/g-C_3N_4 composite. In the (a–c), the left part is the 3D charge density difference with the yellow and cyan regions representing charge accumulation and depletion, respectively; the isosurface value is 3×10^{-7} e/Å^3; the right part is the corresponding planar averaged charge density difference with units of e/Å.

VBs of LTO due to their VBs offset. Therefore, the photoexcited electron–hole pairs can be effectively separated to the opposite counterpart, where the reduction and oxidation reaction would take place, respectively.

For the case of N-LTO/g-C_3N_4, the VBM is also composed of the N-LTO orbitals, while the CBM consists not only of the g-C_3N_4 orbitals, but also of some unoccupied N 2p states of N-LTO, as seen from PDOS (Figure 5e) of the composites. The unoccupied N 2p states appearing at the CBM may trap the photoexcited electrons from the VBs of N-LTO and facilitate the electron–hole recombination. For the S-LTO/g-C_3N_4 composite (Figure 5f), the VBM of the composite is only composed of S 3p states from the S-LTO slab, meanwhile, the CBM is formed only by the C and N 2p orbitals of g-C_3N_4. Similar to the case of LTO/g-C_3N_4, a type-II band alignment is also present at the interface of S-LTO/g-C_3N_4 heterojunction, which can produce well-separated electron–hole pairs.

3.2.3. Charge Transfer. A substantial charge transfer between the two constituents in the (N-, S-)LTO/g-C_3N_4 composites can be visualized by three-dimensional (3D) charge density difference and the planar averaged charge density difference along the normal direction of the surface. As shown in Figure 6a–c, we see that the charge redistribution mostly occurs at the g-C_3N_4 sheet and the interface region. This indicates that the underling (N-, S-)LTO surface induces significant charge distribution fluctuation of the supernatant g-C_3N_4 sheet. Further away from the outmost surface of (N-, S-)LTO, the surface-induced charge density fluctuation gradually diminishes in the bulk region of (N-, S-)LTO slab. Specifically, in the LTO/g-C_3N_4 composite (Figure 6a), a strong charge accumulation is found on the lower side of the g-C_3N_4 sheet. The outmost La atoms on the LTO(010) surface lose electron, while O atoms gain some electrons. From the planar averaged charge density difference (right part of the Figure 6a), we see that the largest electron accumulation localizes around the g-C_3N_4 sheet with a value of 0.0018 e/Å. The region near the LTO surface are mainly electron depletion. The planar averaged charge density difference clearly demonstrates that the electrons transfer from the LTO(010) surface to the g-C_3N_4 sheet in the composite.

For the N-LTO/g-C_3N_4 composite, the charge redistribution in the interface (Figure 6b) is different from that observed in the LTO/g-C_3N_4 composite. A strong charge accumulation is observed to occur above the outmost La atoms of the N-LTO slab. The electron depletion region mainly localizes on the upper surface of the g-C_3N_4 sheet. There is nearly no charge redistribution around the doped N atoms in the top layer of the N-LTO(010) surface. The planar average charge density difference of N-LTO/g-C_3N_4 (right part of Figure 6b) shows that there is a fluctuation around the g-C_3N_4 sheet, indicating that the charge accumulation and depletion occur simultaneously across the buckled g-C_3N_4 sheet. The upper and lower surface of g-C_3N_4 supplies hole-rich and electron-rich sites, respectively, which would server as active sites for photocatalysis.

For the S-LTO/g-C_3N_4 composite, the charge redistribution in the interface (Figure 6c) is obvious owing to the short interfacial distance. The N atom in the lowest site of the g-C_3N_4 sheet is covalent with the La atom in the top layer of the S-LTO surface. The N atom from g-C_3N_4 gains some electrons, while the La atom from S-LTO loses some electrons. The doping S atom in the S-LTO surface gains some electrons. The planar average charge density difference (right part of Figure 6c) shows that the largest electron accumulation localizes just below the lowest N atom in the g-C_3N_4 sheet, and the largest electron depletion occurs in the g-C_3N_4 sheet.

The effective net electrons from LTO, N-LTO, and S-LTO to g-C_3N_4 were calculated by the Bader method[60] to be 0.036e, 0.034e, and 0.004e respectively. Interestingly, although S-LTO/g-C_3N_4 has the shortest interfacial distance in the three composites, the transfer number of electrons from S-LTO to g-C_3N_4 sheet is the smallest. The net charge distribution results in a polarized field which points from the (N-, S-)LTO to the g-C_3N_4 sheet. As we stated in the section of "Electronic Structure", the type-II band alignment in the (S-)LTO/g-C_3N_4 composite prompts the photoexcited electrons transferring

DOI: 10.1021/acsami.8b12178
ACS Appl. Mater. Interfaces 2018, 10, 39327–39335

from (S-)LTO to g-C_3N_4. There is a competitive role in electron−hole separation between the band alignment and the polarized electric field. Usually, the driving force provided by the band offset is larger than that coming from the polarized field.[34] In our work, the CBs offsets for LTO/g-C_3N_4 and S-LTO/g-C_3N_4 were calculated to be 1.48 and 1.28 V, respectively. The corresponding field strength were estimated to be about 5.48×10^9 and 6.09×10^9 V/m, respectively ($E = U/d$, where U is CBs offset, d is the interfacial distance). We see that S-LTO/g-C_3N_4 possesses larger field strength from the band alignment than that of LTO/g-C_3N_4. Meanwhile, it has the smallest polarized electric field due to the least effective net electrons transferring from S-LTO to g-C_3N_4. Therefore, S-LTO/g-C_3N_4 may possess high separation efficiency of the photoexcited carriers.

3.3. Optical Properties. Finally, we explore their vis-light photocatalytic activity by analyzing the UV−vis optical absorption spectra. The calculated optical absorption spectra (by using HSE03) of the pure LTO(010) surface, N-LTO(010) surface, S-LTO(010) surface, and the hybrid (N-, S-)LTO/g-C_3N_4 composites are shown in Figure 7. For the

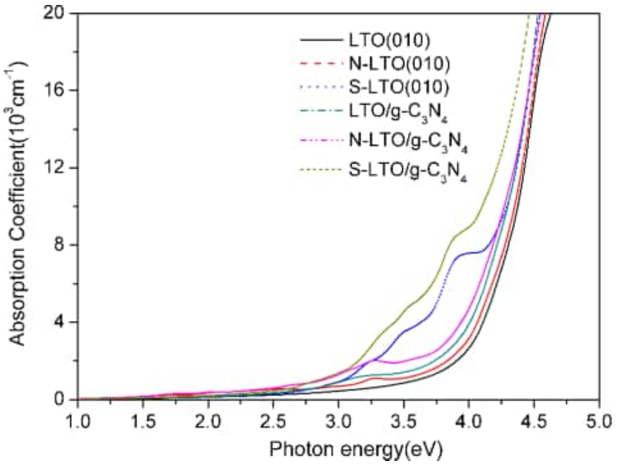

Figure 7. Optical absorption coefficients α of the LTO(010) surface, N-LTO(010) surface, S-LTO(010) surface, and LTO/g-C_3N_4, N-LTO/g-C_3N_4, and S-LTO/g-C_3N_4 composites for the polarization vector perpendicular to the surface.

pure LTO(010) surface, the optical absorption starts at about 3.7 eV and increases sharply with the increasing photon energy, suggesting that it is only active under UV light. The anion (N or S) doping into the LTO(010) surface can shift the optical absorption edge to the visible region. The optical absorption edges are redshifted gradually from the LTO(010) surface to N-LTO(010) to LTO/g-C_3N_4 to N-LTO/g-C_3N_4 to S-LTO(010) to S-LTO/g-C_3N_4. The S-LTO/g-C_3N_4 composite has the strongest absorption in the energy range of 2.7−4 eV.

4. CONCLUSIONS

In summary, we have investigated the (N-, S-)LTO(010) surfaces and the (N-, S-)LTO/g-C_3N_4 composites by a state-of-the-art DFT calculation incorporating long-range dispersion corrections. The results revealed that doping N or S into the LTO(010) surface can greatly decrease the band gap of the LTO surface, enabling the absorption of visible light. After doping with N, the effective band gap of the LTO(010) surface is reduced to 2.18 eV due to some unoccupied empty states above the Fermi level created by the N 2p states. By contrast, A clean band gap with a value of 2.67 eV is obtained after doping the S atom in the LTO(010) surface. To decrease the recombination rate of the photoexcited hole and electron, the three surfaces were hybridized with the g-C_3N_4 sheet. We found that: (i) the S-LTO/g-C_3N_4 composite has the shortest interfacial distance (about 2.1 Å), implying a strong electronic coupling at the interface; (ii) a type-II band alignment is present in S-LTO/g-C_3N_4 and LTO/g-C_3N_4 composites, suggesting that they can produce well-separated electron−hole pairs; (iii) S-LTO/g-C_3N_4 has high separation efficiency of the photoexcited carriers; (iv) the optical absorption edges decrease in the order of LTO(010) > N-LTO(010) > LTO/g-C_3N_4 > N-LTO/g-C_3N_4 > S-LTO(010) > S-LTO/g-C_3N_4. Our results suggest that the S-LTO/g-C_3N_4 composite might be more active than N-LTO/g-C_3N_4 in the water-splitting reaction.

■ ASSOCIATED CONTENT

Supporting Information

The Supporting Information is available free of charge on the ACS Publications website at DOI: 10.1021/acsami.8b12178.

Partial DOS of bulk LTO and g-C_3N_4 calculated using the HSE03 functional and total DOS of the LTO(010), N-doped LTO(010), and S-doped LTO(010) surface by using the PBE + U method (PDF)

■ AUTHOR INFORMATION

Corresponding Author

*E-mail: wkc@fjirsm.ac.cn.

ORCID

Zuju Ma: 0000-0001-5687-862X

Kechen Wu: 0000-0002-9531-2239

Notes

The authors declare no competing financial interest.

■ ACKNOWLEDGMENTS

This work was supported by the National Science Foundation of China (Nos. 21501177, 21771182 and 21673240), the NSF of Fujian Province (Grant 2017J05033), the NSF of Anhui Province (1808085ME138), and the Open Project Program of the State Key Laboratory of Photocatalysis on Energy and Environment (Grant No. SKLPEE-KF201708), Fuzhou University. The authors also gratefully acknowledge the National Supercomputing Center in Shenzhen for providing the computing resources. The authors thank Dr. Zhenxing Fang of Zunyi normal university for useful discussions.

■ REFERENCES

(1) Fujishima, A.; Honda, K. Electrochemical Photolysis of Water at A Semiconductor Electrode. *Nature* **1972**, *238*, 37−38.

(2) Hwang, D. W.; Kirn, H. G.; Lee, J. S.; Kim, J.; Li, W.; Oh, S. H. Photocatalytic Hydrogen Production from Water over m-doped La2Ti2O7 (M = Cr, Fe) under Visible Light Irradiation (Lambda > 420 nm). *J. Phys. Chem. B* **2005**, *109*, 2093−2102.

(3) Kudo, A.; Miseki, Y. Heterogeneous Photocatalyst Materials for Water Splitting. *Chem. Soc. Rev.* **2009**, *38*, 253−278.

(4) Nisar, J.; Pathak, B.; Wang, B.; Kang, T. W.; Ahuja, R. Hole Mediated Coupling in Sr2Nb2O7 for Visible Light Photocatalysis. *Phys. Chem. Chem. Phys.* **2012**, *14*, 4891−4897.

(5) Liu, P.; Nisar, J.; Sa, B.; Pathak, B.; Ahuja, R. Anion−Anion Mediated Coupling in Layered Perovskite La2Ti2O7 for Visible Light Photocatalysis. *J. Phys. Chem. C* **2013**, *117*, 13845−13852.

(6) Nisar, J.; Pathak, B.; Ahuja, R. Screened Hybrid Density Functional Study on Sr2Nb2O7 for Visible Light Photocatalysis. *Appl. Phys. Lett.* **2012**, *100*, No. 181903.

(7) Wang, B. C.; Nisar, J.; Pathak, B.; Kang, T. W.; Ahuja, R. Band Gap Engineering in BiNbO4 for Visible-light Photocatalysis. *Appl. Phys. Lett.* **2012**, *100*, No. 182102.

(8) Liu, P.; Nisar, J.; Pathak, B.; Ahuja, R. Hybrid Density Functional Study on SrTiO3 for Visible Light Photocatalysis. *Int. J. Hydrogen Energy* **2012**, *37*, 11611−11617.

(9) Chen, X.; Shen, S.; Guo, L.; Mao, S. S. Semiconductor-based Photocatalytic Hydrogen Generation. *Chem. Rev.* **2010**, *110*, 6503−6570.

(10) Khan, S. U. M.; Al-Shahry, M.; Ingler, W. B. Efficient Photochemical Water Splitting by A Chemically Modified n-TiO2. *Science* **2002**, *297*, 2243−2245.

(11) Kato, H.; Asakura, K.; Kudo, A. Highly Efficient Water Splitting into H-2 and O-2 over Lanthanum-doped NaTaO3 Photocatalysts with High Crystallinity and Surface Nanostructure. *J. Am. Chem. Soc.* **2003**, *125*, 3082−3089.

(12) Kim, H. G.; Hwang, D. W.; Bae, S. W.; Jung, J. H.; Lee, J. S. Photocatalytic Water Splitting over La2Ti2O7 Synthesized By the Polymerizable Complex Method. *Catal. Lett.* **2003**, *91*, 193−198.

(13) Kudo, A.; Kato, H.; Nakagawa, S. Water Splitting into H-2 and O-2 on New Sr2M2O7 (M = Nb and Ta) Photocatalysts with Layered Perovskite Structures: Factors Affecting the Photocatalytic Activity. *J. Phys. Chem. B* **2000**, *104*, 571−575.

(14) Hwang, D. W.; Kim, H. G.; Kim, J.; Cha, K. Y.; Kim, Y. G.; Lee, J. S. Photocatalytic Water Splitting over Highly Donor-doped (110) Layered Perovskites. *J. Catal.* **2000**, *193*, 40−48.

(15) Kim, H. G.; Hwang, D. W.; Kim, J.; Kim, Y. G.; Lee, J. S. Highly Donor-doped (110) Layered Perovskite Materials as Novel Photocatalysts for Overall Water Splitting. *Chem. Commun.* **1999**, *12*, 1077−1078.

(16) Abe, R.; Higashi, M.; Sayama, K.; Abe, Y.; Sugihara, H. Photocatalytic Activity of R3MO7 and R2Ti2O7 (R = Y, Gd, La; M = Nb, Ta) for Water Splitting into H-2 and O-2. *J. Phys. Chem. B* **2006**, *110*, 2219−2226.

(17) Bruyer, E.; Sayede, A. Density Functional Calculations of the Structural, Electronic, and Ferroelectric Properties of High-k Titanate Re2Ti2O7 (Re = La and Nd). *J. Appl. Phys.* **2010**, *108*, No. 053705.

(18) Hwang, D. W.; Lee, J. S.; Li, W.; Oh, S. H. Electronic Band Structure and Photocatalytic Activity of Ln(2)Ti(2)O(7) (Ln = La, Pr, Nd). *J. Phys. Chem. B* **2003**, *107*, 4963−4970.

(19) Uno, M.; Kosuga, A.; Okui, M.; Horisaka, K.; Yamanaka, S. Photoelectrochemical Study of Lanthanide Titanium Oxides, Ln(2)-Ti(2)O(7) (Ln = La, Sm, and Gd). *J. Alloys Compd.* **2005**, *400*, 270−275.

(20) Meng, F. K.; Hong, Z. L.; Arndt, J.; Li, M.; Zhi, M. J.; Yang, F.; Wu, N. Q. Visible Light Photocatalytic Activity of Nitrogen-doped La2Ti2O7 Nanosheets Originating from Band Gap Narrowing. *Nano Res.* **2012**, *5*, 213−221.

(21) Ma, Z.; Wu, K.; Sa, R.; Li, Q.; He, C.; Yi, Z. Mechanism of Enhanced Photocatalytic Activities on N-doped La2Ti2O7: An Insight from Density-functional Calculations. *Int. J. Hydrogen Energy* **2015**, *40*, 980−989.

(22) Meng, F.; Cushing, S. K.; Li, J.; Hao, S.; Wu, N. Enhancement of Solar Hydrogen Generation by Synergistic Interaction of La2Ti2O7 Photocatalyst with Plasmonic Gold Nanoparticles and Reduced Graphene Oxide Nanosheets. *ACS Catal.* **2015**, *5*, 1949−1955.

(23) Cai, X.; Zhang, J.; Fujitsuka, M.; Majima, T. Graphitic-C3N4 Hybridized N-doped La2Ti2O7 Two-dimensional Layered Composites as Efficient Visible-light-driven Photocatalyst. *Appl. Catal., B* **2017**, *202*, 191−198.

(24) Ao, Y.; Wang, K.; Wang, P.; Wang, C.; Hou, J. Synthesis of Novel 2D-2D p-n Heterojunction BiOBr/La2Ti2O7 Composite Photocatalyst with Enhanced Photocatalytic Performance under Both UV and Visible Light Irradiation. *Appl. Catal., B* **2016**, *194*, 157−168.

(25) Ao, Y.; Wang, K.; Wang, P.; Wang, C.; Hou, J. Fabrication of Novel p-n heterojunction BiOI/La2Ti2O7 Composite Photocatalysts for Enhanced Photocatalytic Performance under Visible Light Irradiation. *Dalton Trans.* **2016**, *45*, 7986−7997.

(26) Ao, Y.; Wang, K.; Wang, P.; Wang, C.; Hou, J. Fabrication of P-type BiOCl/n-type La2Ti2O7 Facet-coupling Heterostructure with Enhanced Photocatalytic Performance. *RSC Adv.* **2016**, *6*, 48599−48609.

(27) Nashim, A.; Martha, S.; Parida, K. M. Heterojunction Conception of n-La2Ti2O7/p-CuO in the Limelight of Photocatalytic Formation of Hydrogen under Visible Light. *RSC Adv.* **2014**, *4*, 14633−14643.

(28) Liu, J.; Liu, Y.; Liu, N.; Han, Y.; Zhang, X.; Huang, H.; Lifshitz, Y.; Lee, S.-T.; Zhong, J.; Kang, Z. Metal-free Efficient Photocatalyst for Stable Visible Water Splitting via A Two-electron Pathway. *Science* **2015**, *347*, 970−974.

(29) Du, A.; Sanvito, S.; Li, Z.; Wang, D.; Jiao, Y.; Liao, T.; Sun, Q.; Ng, Y. H.; Zhu, Z.; Amal, R.; Smith, S. C. Hybrid Graphene and Graphitic Carbon Nitride Nanocomposite: Gap Opening, Electron-Hole Puddle, Interfacial Charge Transfer, and Enhanced Visible Light Response. *J. Am. Chem. Soc.* **2012**, *134*, 4393−4397.

(30) Yu, J.; Wang, S. H.; Low, J. X.; Xiao, W. Enhanced Photocatalytic Performance of Direct Z-scheme g-C3N4-TiO2 Photocatalysts for the Decomposition of Formaldehyde in Air. *Phys. Chem. Chem. Phys.* **2013**, *15*, 16883−16890.

(31) Li, Y. B.; Zhang, H. M.; Liu, P. R.; Wang, D.; Li, Y.; Zhao, H. J. Cross-Linked g-C3N4/rGO Nanocomposites with Tunable Band Structure and Enhanced Visible Light Photocatalytic Activity. *Small* **2013**, *9*, 3336−3344.

(32) Xu, L.; Huang, W.-Q.; Wang, L.-L.; Tian, Z.-A.; Hu, W.; Ma, Y.; Wang, X.; Pan, A.; Huang, G.-F. Insights into Enhanced Visible-Light Photocatalytic Hydrogen Evolution of g-C3N4 and Highly Reduced Graphene Oxide Composite: The Role of Oxygen. *Chem. Mater.* **2015**, *27*, 1612−1621.

(33) Ge, L.; Han, C. C.; Xiao, X. L.; Guo, L. L. Synthesis and Characterization of Composite Visible Light Active Photocatalysts MoS2-g-C3N4 with Enhanced Hydrogen Evolution Activity. *Int. J. Hydrogen Energy* **2013**, *38*, 6960−6969.

(34) Wang, J.; Guan, Z.; Huang, J.; Li, Q.; Yang, J. Enhanced Photocatalytic Mechanism for the Hybrid g-C3N4/MoS2 Nanocomposite. *J. Mater. Chem. A* **2014**, *2*, 7960−7966.

(35) Kumar, S.; Surendar, T.; Baruah, A.; Shanker, V. Synthesis of A Novel and Stable g-C3N4-Ag3PO4 Hybrid Nanocomposite Photocatalyst and Study of the Photocatalytic Activity under Visible Light Irradiation. *J. Mater. Chem. A* **2013**, *1*, 5333−5340.

(36) Huang, L.; Xu, H.; Li, Y. P.; Li, H. M.; Cheng, X. N.; Xia, J. X.; Xu, Y. G.; Cai, G. B. Visible-Light-Induced WO3/g-C3N4 Composites with Enhanced Photocatalytic Activity. *Dalton Trans.* **2013**, *42*, 8606−8616.

(37) Xu, M.; Han, L.; Dong, S. J. Facile Fabrication of Highly Efficient g-C3N4/Ag2O Heterostructured Photocatalysts with Enhanced Visible-Light Photocatalytic Activity. *ACS Appl. Mater. Interfaces* **2013**, *5*, 12533−12540.

(38) Qiao, Q.; Yang, K.; Ma, L.-L.; Huang, W.-Q.; Zhou, B.-X.; Pan, A.; Hu, W.; Fan, X.; Huang, G.-F. Facile In Situ Construction of Mediator-Free Direct Z-Scheme g-C3N4/CeO2 Heterojunctions with Highly Efficient Photocatalytic Activity. *J. Phys. D: Appl. Phys.* **2018**, *51*, No. 275302.

(39) Kresse, G. VASP, 5.3.5. https://www.vasp.at, 2018.

(40) Kresse, G.; Furthmuller, J. Efficient Iterative Schemes for Ab Initio Total-Energy Calculations Using A Plane-Wave Basis Set. *Phys. Rev. B* **1996**, *54*, 11169−11186.

(41) Kresse, G.; Joubert, D. From Ultrasoft Pseudopotentials to the Projector Augmented-Wave Method. *Phys. Rev. B* **1999**, *59*, 1758−1775.

(42) Blöchl, P. E. Projector Augmented-Wave Method. *Phys. Rev. B* **1994**, *50*, 17953−17979.

(43) Perdew, J. P.; Burke, K.; Ernzerhof, M. Generalized Gradient Approximation Made Simple. *Phys. Rev. Lett.* **1996**, *77*, 3865−3868.

(44) Grimme, S. Semiempirical GGA-type Density Functional Constructed with A Long-Range Dispersion Correction. *J. Comput. Chem.* **2006**, *27*, 1787−1799.
(45) Cococcioni, M.; de Gironcoli, S. Linear Response Approach to the Calculation of the Effective Interaction Parameters in the LDA + U Method. *Phys. Rev. B* **2005**, *71*, No. 035105.
(46) Mori-Sánchez, P.; Cohen, A. J.; Yang, W. T. Localization and Delocalization Errors in Density Functional Theory and Implications for Band-Gap Prediction. *Phys. Rev. Lett.* **2008**, *100*, No. 146401.
(47) Anisimov, V. I.; Zaanen, J.; Andersen, O. K. Band Theory and Mott Insulators - Hubbard-U instead of Stoner-I. *Phys. Rev. B* **1991**, *44*, 943−954.
(48) Morgan, B. J.; Watson, G. W. GGA + U Description of Lithium Intercalation into Anatase TiO2. *Phys. Rev. B* **2010**, *82*, No. 144119.
(49) Coquet, R.; Willock, D. J. The (010) Surface of Alpha-MoO3, A DFT + U Study. *Phys. Chem. Chem. Phys.* **2005**, *7*, 3819−3828.
(50) Scanlon, D. O.; Walsh, A.; Morgan, B. J.; Watson, G. W. An Ab Initio Study of Reduction of V2O5 through the Formation of Oxygen Vacancies and Li Intercalation. *J. Phys. Chem. C* **2008**, *112*, 9903−9911.
(51) Scanlon, D. O.; Galea, N. M.; Morgan, B. J.; Watson, G. W. Reactivity on the (110) Surface of Ceria: A GGA plus U Study of Surface Reduction and the Adsorption of CO and NO2. *J. Phys. Chem. C* **2009**, *113*, 11095−11103.
(52) Monkhorst, H. J.; Pack, J. D. Special Points for Brillouin-zone Integrations. *Phys. Rev. B* **1976**, *13*, No. 5188.
(53) Palik, E. D.; Ghosh, G. *Handbook of Optical Constants of Solids*; Academic Press: London, U.K., 1998; Vol. *3*.
(54) Ma, Z.; Yi, Z.; Sun, J.; Wu, K. Electronic and Photocatalytic Properties of Ag3PC4VI (C = O, S, Se): A Systemic Hybrid DFT Study. *J. Phys. Chem. C* **2012**, *116*, 25074−25080.
(55) Read, A. J.; Needs, R. J. Calculation of Optical Matrix Elements with Nonlocal Pseudopotentials. *Phys. Rev. B* **1991**, *44*, No. 13071.
(56) Yu, P.; Cardona, M., *Fundamentals of Semiconductors*. Springer-Verlag: Berlin, 1996.
(57) Ma, Z.; Sa, R.; Li, Q.; Wu, K. Interfacial Electronic Structure and Charge Transfer of Hybrid Graphene Quantum Dot and Graphitic Carbon Nitride Nanocomposites: Insights into High Efficiency for Photocatalytic Solar Water Splitting. *Phys. Chem. Chem. Phys.* **2016**, *18*, 1050−1058.
(58) Wu, H.-Z.; Liu, L.-M.; Zhao, S.-J. The Effect of Water on the Structural, Electronic and Photocatalytic Properties of Graphitic Carbon Nitride. *Phys. Chem. Chem. Phys.* **2014**, *16*, 3299−3304.
(59) Zhou, Y.; Zhang, Y.; Lin, M.; Long, J.; Zhang, Z.; Lin, H.; Wu, J. C. S.; Wang, X. Monolayered Bi2WO6 Nanosheets Mimicking Heterojunction Interface with Open Surfaces for Photocatalysis. *Nat. Commun.* **2015**, *6*, No. 8340.
(60) Henkelman, G.; Arnaldsson, A.; Jonsson, H. A Fast and Robust Algorithm for Bader Decomposition of Charge Density. *Comput. Mater. Sci.* **2006**, *36*, 354−360.

DOI: 10.1021/acsami.8b12178
ACS Appl. Mater. Interfaces 2018, 10, 39327−39335

Chemical Engineering Journal 423 (2021) 130220

Contents lists available at ScienceDirect

Chemical Engineering Journal

journal homepage: www.elsevier.com/locate/cej

High-concentration N-doped $La_2Ti_2O_7$ nanocrystals: Effects of nano-structuration and doping sites on enhancing the photocatalytic activity

Jingwen Wang, Yusuke Asakura [1,*], Takuya Hasegawa, Shu Yin

Institute of Multidisciplinary Research for Advanced Materials, Tohoku University, Sendai, Japan

A R T I C L E I N F O

Keywords:
Nitrogen doping
Layered perovskite
Photocatalysts
Hydrothermal synthesis
NO_x decomposition
$La_2Ti_2O_7$

A B S T R A C T

A series of N-doped $La_2Ti_2O_7$ nanocrystal for photocatalytic NO_x oxidation are successfully prepared by a two-steps process including hydrothermal reaction and heat treatment under NH_3 flow. The effects of different nitrogen doping amounts on the structure, optical property and photocatalytic activity of the obtained N-doped $La_2Ti_2O_7$ are investigated. The photocatalytic activity and stability of the N-doped $La_2Ti_2O_7$ samples are evaluated through photocatalytic oxidation of NO_x under UV and visible light irradiation. The nanosheet morphology of N-doped $La_2Ti_2O_7$ is originated from $La_2Ti_2O_7$ precursor synthesized by hydrothermal reaction. The addition of triethanolamine (TEA) in the hydrothermal reaction largely affects the morphology of $La_2Ti_2O_7$ to form nanocrystal. The light absorption of $La_2Ti_2O_7$ nanocrystal is cut off at 338 nm, while the N-doped $La_2Ti_2O_7$ nanocrystals exhibit the extended light absorption up to 560 nm. It is found that the N doping into $La_2Ti_2O_7$ enhances absorption in the visible light region to exhibit higher activity for photocatalytic oxidation of NO gas. The optimized N-doped $La_2Ti_2O_7$ nanocrystal with 4.79 at% of nitrogen dopant possesses the best photocatalytic NO_x oxidation activity under both ultraviolet and visible light irradiation compared with N-doped $La_2Ti_2O_7$ samples obtained by hydrothermal reaction without TEA or flux synthesis of $La_2Ti_2O_7$ and the subsequent treatment with NH_3. The enhancement of photocatalytic activity should originate from simultaneous achievement of large specific surface area and strong visible light absorption due to the large amount of nitrogen dopants.

1. Introduction

In the past few decades, a number of perovskite-type oxides have been demonstrated to be effective photocatalysts with a wide range of applications, such as solar energy conversion, environmental remediation and so on.[1] The bandgap, or absorption edge of perovskite materials can be controlled in a wide range because of their favorable crystallographic flexibility.[2] In addition, the narrow depletion layers of the perovskite layered materials make the separation of electrons and holes easier than that in other semiconductor materials.[3] Because perovskite-related compounds can be structurally and compositionally modified for desired functions, they have been focused for water spitting under UV and visible light irradiation, CO_2 reduction and decomposition of organic pollutants.[4–7]

NO_x emitted from industries, motor vehicles and other combustion process has aroused increasing concerns about the impact on environment and became global issues. NO is the major compound of NO_x, and it is also one of the pollutants attributed to acid rain and urban smog.[8] Nowadays, using photocatalysts to decompose NO under low reaction temperature has become an effective and promising approach to improve air quality and reduce energy consumption.[9] Recently, lanthanum titanate ($La_2Ti_2O_7$), as a layer-structured perovskite material, has attracted great attention due to its high stability and strong redox ability, which can be applied in many areas including water-splitting to produce H_2 and/or O_2 under UV irradiation,[10,11] photodegradation of organic pollutants,[12] and the photocatalytic conversion of CO_2 by H_2O.[7] However, $La_2Ti_2O_7$ possesses some drawbacks for achieving efficient photocatalytic activity under visible light irradiation: (i) no visible light absorption and (ii) its large particle size. (i) The wide bandgap of $La_2Ti_2O_7$ (*ca.* 3.8 eV) induces insufficient visible light

* Corresponding author.
E-mail address: asa@aoni.waseda.jp (Y. Asakura).
[1] Present address: Kagami Memorial Research Institute for Materials Science and Technology, Waseda University, Japan.

https://doi.org/10.1016/j.cej.2021.130220
Received 4 February 2021; Received in revised form 29 April 2021; Accepted 3 May 2021
Available online 7 May 2021
1385-8947/© 2021 Elsevier B.V. All rights reserved.

absorption. (ii) In a conventional way to synthesize $La_2Ti_2O_7$, a mixture of precursor oxides including La_2O_3 and TiO_2 are ground together and then calcinated at a high temperature for a long duration to allow the interdiffusion of cations.[13] The large size of the formed particles leads to low surface area and long pathway to the surfaces, resulting in a small amount of reaction sites and high frequent recombination of photoexcited carriers to the particle surface. These factors can largely decrease the efficiency of the photocatalytic performance. Therefore, the methods for solving the above-mentioned problems are highly needed for further improvement of photocatalytic activity.

A lot of efforts have been paid to solve the downsides of (i) no visible light absorption and (ii) large particle size of $La_2Ti_2O_7$. (i) Introduction of a foreign element into $La_2Ti_2O_7$ is one of the approaches to control the bandgap. The introduction of foreign cations induces a donor level above the valence band of oxide photocatalyst. Although the presence of the donor level induces visible-light absorption ability, the localized energy states can act as recombination centers of charge carriers, reducing the photocatalytic activity.[14] Anion doping is another strategy to improve the visible light absorption. Nitrogen was reported as an effective anion dopant to narrow the bandgap of TiO_2.[15] Identification of the different positions of introduced nitrogen including interstitial sites or substitutional sites is needed because the nitrogen doping sites were found to have important effects on the photocatalytic activity of nitrogen-doped oxides. However, for the complex metal oxides, most reports confirmed the successful doping of N atoms into lattice based on the characterization but lacked the further investigation of doping sites, which made it insufficient to understand the intrinsic properties of nitrogen doped materials.[16–19] Therefore, we investigate the effects of doping sites on electronic structure and further catalytic activity of $La_2Ti_2O_7$, one of the complex perovskite oxides. In the case of N-doped TiO_2 sample, the N dopant can introduce shallow energy states near the valence band states and the oxygen vacancies to rise the local states below the conduction edge, which can improve the visible light absorption.[20] For $La_2Ti_2O_7$, nitrogen doping can shift the absorption edge to longer wavelength to enhance the visible light photocatalytic activity effectively.[6,21] (ii) The nanoparticle morphology of $La_2Ti_2O_7$ precursor is desired, because $La_2Ti_2O_7$ with nanostructured morphology can increase the reaction sites and suppress the frequency of recombination between photoexcited carriers to enhance the photocatalytic activity.[22] Hojamberdiev *et al.* reported a flux growth method using alkali-metal molybdate for the preparation of $La_2Ti_2O_7$.[23] Even though the average size of the obtained $La_2Ti_2O_7$ platelet decreased with the increase of solute concentration, it was still several micrometers. Although several methods have been effective for improving photocatalytic activity, the photocatalytic activity under visible light irradiation has not been high enough for the practical use. Therefore, the further development is needed to solve the two drawbacks.

Herein, particle size and band structure in $La_2Ti_2O_7$ were controlled by sophisticated hydrothermal reaction and the subsequent ammonia treatment, respectively. Although some papers reported that the hydrothermal synthesis of $La_2Ti_2O_7$ formed comparably smaller particle size than that obtained by a solid-state reaction, the size has been over micrometers.[11,24] In this study, triethanolamine (TEA), which can act as a morphological controller,[25] was added into the precursor solution for the hydrothermal reaction to decrease the particle size of the obtained $La_2Ti_2O_7$ significantly. Also, we found a small amount of nitrogen was doped into the obtained $La_2Ti_2O_7$ from TEA during the hydrothermal reaction, and the pre-doped nitrogen may contribute to the high concentration of nitrogen doping after the nitridation with NH_3. Meanwhile, the influence of nitrogen doping concentration and different nitrogen sites on the photocatalytic activities of these samples were evaluated by the oxidative decomposition of NO gas.

2. Experimental section

2.1. Preparation of $La_2Ti_2O_7$ nanocrystals

$La_2Ti_2O_7$ nanocrystals were synthesized by modified hydrothermal method based on the previous reference.[26] The triethanolamine (TEA, min. 98.0%, Kanto Chemical) was used as an additive. The titanium(IV) bis(ammonium lactato) dihydroxide solution ($C_6H_{18}N_2O_8Ti$, 50 wt% in H_2O, Aldrich, denoted as TAL) as the Ti source is a stable water-soluble compound due to the chelating lactate group. In a typical synthesis, 0.96 mL of TAL and 2 mmol of $La(NO_3)_2{\cdot}6H_2O$ (>99%, Wako Pure Chemicals) were added into 10 mL deionized water. The molar ratio of La/Ti in this mixture was adjusted to 1:1. After stirring for 20 min, an aqueous solution of NaOH (10 mL, 1.5 M) and 8 mmol of TEA were slowly added to the solution dropwise with continuous stirring, respectively. After being well homogenized for 30 min, the mixture was transferred to a 100 mL Teflon-lined stainless-steel autoclave and treated hydrothermally at 240 °C for 24 h, and then cooled to room temperature. After the treatment, the white precipitation was then washed with deionized water and centrifuged several times for separation. The product was subsequently dried under reduced pressure at 60 °C overnight. The obtained white powder was referred as LTO_NC where NC means nanocrystal. The pristine $La_2Ti_2O_7$ with larger particle size than LTO_NC was also prepared by the hydrothermal method without the addition of TEA for comparison. The obtained compound was denoted as LTO_P. Also, $La_2Ti_2O_7$ with platelet micro-morphology was prepared by a flux growth method using NaCl and KCl as the molten salt for comparison (denoted as LTO_Flux).[23]

2.2. Preparation of N-doped $La_2Ti_2O_7$ nanocrystals

In a typical process, nitrogen doped $La_2Ti_2O_7$ nanocrystal (N-LTO_NC) was obtained by heating LTO_NC at 650 °C under NH_3 flow (50 mL min^{-1}) in a tubular furnace. An alumina boat was used to load the $La_2Ti_2O_7$ powder and put in the center of the tubular furnace which equipped with a gas flow meter to control the flow rate. After the reaction time of 3 h, the samples were cooled naturally to room temperature under an NH_3 atmosphere. The sample color changed from white to yellowish after the NH_3 treatment. To study the effect of nitridation temperature on morphology and incorporated nitrogen concentration, the nitridation temperature was changed from 600 to 750 °C and the other conditions were kept the same (The obtained samples were denoted as N-LTO_NC(600), N-LTO_NC(650), N-LTO_NC(700) and N-LTO_NC(750), respectively). In addition, LTO_P, LTO_Flux and the commercial titania (Degussa P25) were also treated with NH_3 (50 mL min^{-1}) at 650 °C for 3 h to form N-doped $La_2Ti_2O_7$ (referred as N-LTO_P, N-LTO_Flux and N-P25, respectively) for comparison. The XRD patterns of LTO_Flux and N-LTO_Flux (Fig. S1a) indicated the pure phase of $La_2Ti_2O_7$.

2.3. Photocatalytic activity test

To evaluate the photocatalytic performance of the obtained nitrogen doped samples, the oxidative decomposition of NO activity was investigated. A glass sample holder with a hollow bottom (1.8 cm × 2 cm × 0.5 mm) was placed into the center of the reaction chamber. The sample (0.05 – 0.06 g) was put into the hollow bottom and pressed tightly to form a fixed surface area of 3.6 cm^2. After calibration of the NO_x analyzer (Yanaco, ECL-88A), the mixed gas including N_2 + NO (2 ppm) and air with 1:1 ratio was input to the reaction chamber. The NO concentration in the inlet gas is maintained at around 1 ppm during the measurement. A 450 W high pressure mercury lamp with a cooling system provided by a Pyrex jacket with cooling water was used as a light source and different filters (> 290 nm (Pyrex glass), > 400 nm (Kenko L41 Super Pro (W) filter) and > 510 nm (a Fuji triacetyl cellulose filter)) were used for selecting the light wavelengths in the photocatalytic

reaction. The temperature is maintained at 20 °C by the circulating cooling system at each measurement. And the relative humidity at this temperature was also maintained at around 23% under the same gas flowing to minimize the influence of humidity on the photocatalytic measurement. The distance of samples and the mercury lamp was kept constant. The irradiation intensity of different light wavelength of $\lambda >$ 290 nm, $\lambda >$ 400 nm and $\lambda >$ 510 nm on the surface of samples were determined as 352, 337 and 243 μmol m^{-2} s^{-1}, respectively. After stabilization under the sample gas flowing and reaching adsorption equilibrium in dark for 10 min, the light was turned on for photocatalytic NO_x test. The measurement under each light wavelength (> 510 nm, > 400 nm, > 290 nm) was performed for 10 min, which was followed by 10 min in dark to reach the initial concentration of NO_x. The gas after the photocatalytic reaction in the chamber was directly transferred to the NO_x analyzer for measurement of NO concentration. In the system of measurement, although NO_2 as a reaction intermediate may be produced during photocatalytic oxidation of NO, the photocatalytic NO_x decomposition activity was determined by measuring the concentration of NO_x gas at the outlet of the reactor after photoirradiation. The conversion ratio of NO is calculated by following equation,

$$NO\ conversion\ ratio\% = \left(\frac{C_0 - C_t}{C_0}\right) \times 100$$

where C_0 and C_t are the initial NO concentration and NO_x concentration at a time 't', respectively. To evaluate the stability of N-LTO_NC (650), the sample was measured by cycling test for successive three cycles. After stabilization in dark for 10 min, the sample was irradiated under light wavelength of > 510, > 400 and > 290 nm in sequence. Additionally, the stability of sample was also evaluated under long-time irradiation of > 290 nm light for 60 min.

2.4. Characterization

The crystalline phases of the obtained samples were characterized with an X-ray diffraction analysis (XRD, Bruker AXS D2 Phaser) with Cu-Kα radiation (λ = 0.15418 nm). The morphologies of the obtained samples were examined by scanning electron microscopy (SEM, JSM-7800F, JEOL). The TEM images were recorded with a transmission electron microscope (TEM, JEM-2000EXII, JEOL) and the high-resolution TEM (HRTEM) were recorded by JEOL JEM-2100F microscope. The surface composition and binding energy of the samples were investigated by X-ray photoelectron spectroscopy (XPS, ULVAC-PHI 5600). The binding energy was calibrated by the C 1 s level at 284.6 eV and Ar^+ sputtering was also utilized to analyze the depth profile of the samples. Fourier transform infrared (FT-IR) spectra were measured by a FT-IR spectrometer (FT/IR-660D-ATR, JASCO). The UV–Vis diffuse reflection spectra (DRS) were collected with a UV–Vis spectrophotometer (V-670, JASCO). The specific surface areas were calculated on the basis of the results of N_2 adsorption measurement at 77 K (NOVA 4200e, Quantachrome Instruments) by the BET method. The photoluminescence (PL) spectra were measured by a spectrofluorometer (JASCO, FP-8500) with a laser (λ = 240 nm) at room temperature. Oxygen vacancies in the solid samples were monitored by electron spin-resonance spectroscopy (ESR, X330, JEOL) at ambient temperature.

3. Results and discussion

3.1. Characterization of structure and morphology of N-doped $La_2Ti_2O_7$

$La_2Ti_2O_7$ with nanosheet morphology was successfully synthesized by a hydrothermal method with the addition of TEA. Fig. 1 shows the XRD patterns of the prepared LTO_NC as well as N-LTO_NC prepared under NH_3 flow at different temperatures. The XRD patterns of LTO_P synthesized without the additive and the corresponding N-LTO_P prepared at 650 °C for 3 h were showed in Fig. S1b. XRD analysis revealed

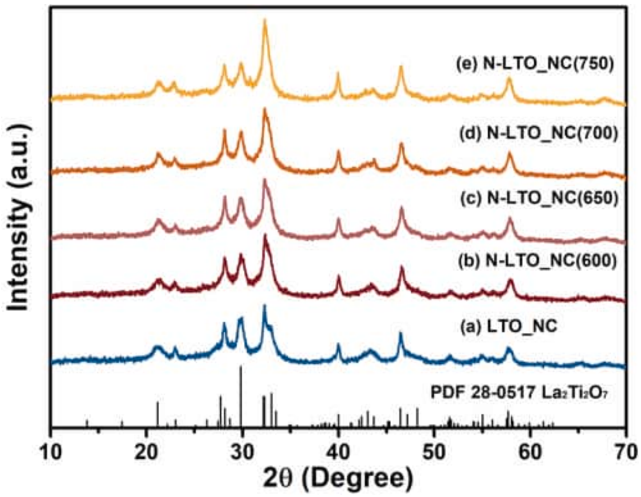

Fig. 1. XRD patterns of standard pattern of $La_2Ti_2O_7$ and as-prepared (a) LTO_NC, nitrogen doped $La_2Ti_2O_7$ heating under different temperature at (b) 600; (c) 650; (d) 700 and 750 °C.

that LTO_NC precursor had a monoclinic structure of $La_2Ti_2O_7$ (ICDD PDF 28–0517), which is the same as the pristine $La_2Ti_2O_7$ synthesized without the addition of TEA. After the NH_3 treatment, all the samples were doped with nitrogen, which was confirmed by the XPS results discussed in the next section. All the nitrogen doped $La_2Ti_2O_7$ samples retained a monoclinic phase, and no diffraction peaks belonging to impurities were detected. The absence of impurity peaks indicates N located in the host structure. The above results suggested that the pure phase of $La_2Ti_2O_7$ crystals was obtained with the addition of TEA additive in the hydrothermal reaction, and the phase was not altered by the nitrogen doping. Also, the overall crystal structure of N-doped $La_2Ti_2O_7$ samples was not largely affected by a range of doping concentration. The N-LTO_NC samples exhibited the similar peak intensity to that of LTO_NC precursor. Compared with the case of LTO_P (Fig. S1b), the decreased intensity and the broaden peaks were observed in the XRD patterns of LTO_NC before and after NH_3 treatment, indicating a low degree of crystallinity of these samples. This result suggested the decrease of particle size by the addition of TEA, which was also consistent well with the TEM images shown below.

Fig. 2 shows the typical TEM images of the as-prepared $La_2Ti_2O_7$ precursors and the nitrogen doped $La_2Ti_2O_7$ samples. LTO_P (Fig. 2a) possessed plate-like morphology with irregular shape and smooth surface. The length of some particles in LTO_P was > 1 μm. On the other hand, the particle size of LTO_NC (Fig. 2b) synthesized with TEA as an additive decreased significantly, resulting in a specific surface area of 69 m^2/g. The value was higher than that of LTO_P (49 m^2/g). The addition of TEA did not largely alter the shape of LTO_NC, while the particle size decreased to below 200 nm. To the best of our knowledge, this is the first report for drastic decrease of the particle size of layered perovskite by simple hydrothermal reaction. The TEM images of the samples after NH_3 treatment at various temperatures (Fig. 2 c-f) indicated that the N-doped $La_2Ti_2O_7$ nanocrystals remained the irregular nano-layered morphology of the precursor. Therefore, N-doping did not alter the nanosheet morphology and the layered structure of the precursor, which was also confirmed by the SEM results (Fig. S2). The particle size of N-LTO_P (Fig. S3a) was larger than that of N-LTO_NC(650) owing to the different sizes of the precursors. From the viewpoint of energy saving, $La_2Ti_2O_7$ precursor with a smaller size was more beneficial for the nitrogen doping under mild conditions compared with the precursor with larger particle size. The unchanged morphology should be attributed to topochemical transformation during the NH_3 treatment between $La_2Ti_2O_7$ crystals and NH_3 species.[27,28] Although the morphology was retained after the NH_3 treatment, the specific surface area of N-LTO_NC(650)

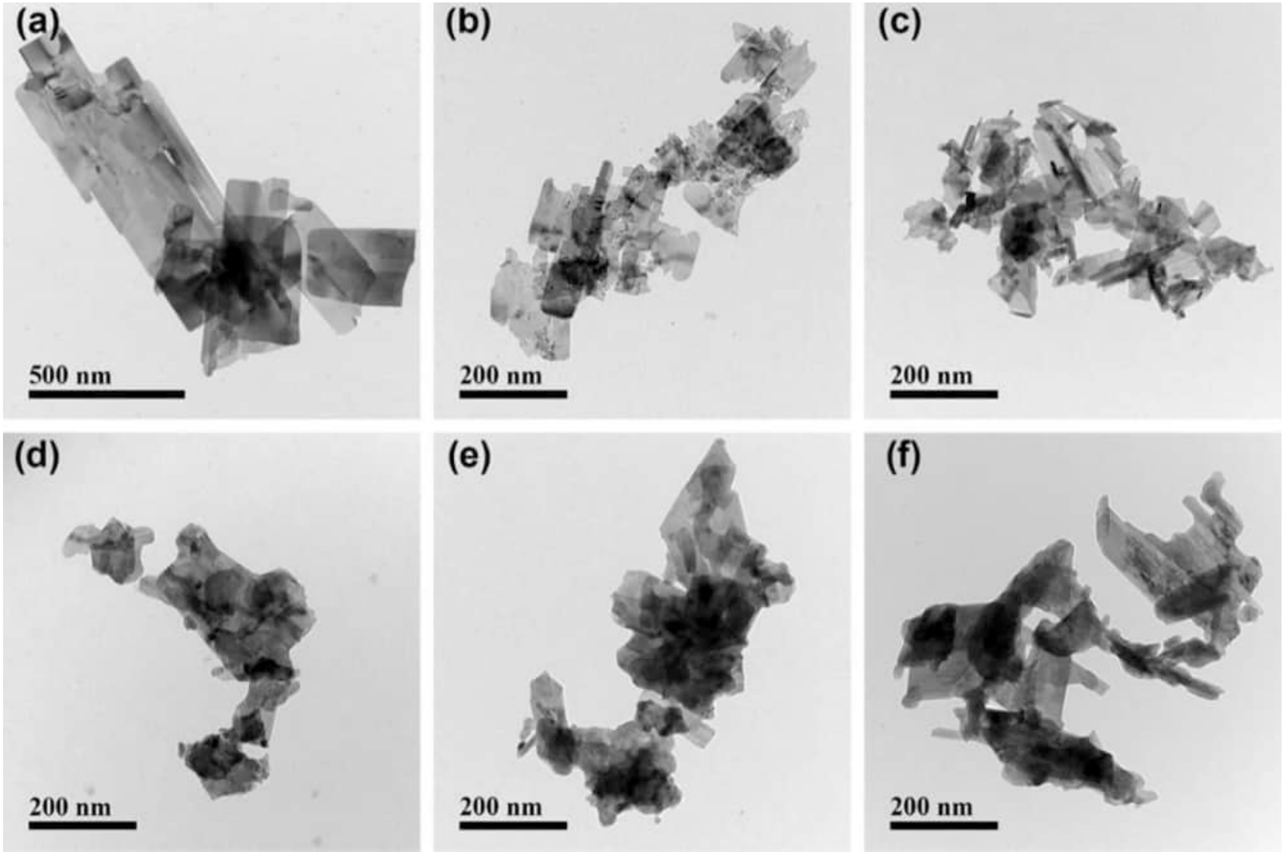

Fig. 2. TEM images of (a) LTO_P; (b) LTO_NC; (c) N-LTO_NC(600); (d) N-LTO_NC(650); (e) N-LTO_NC(700) and (d) N-LTO_NC(750).

declined to 44 m^2/g. The decrease of specific surface area may be attributed to aggregation of the layered particles during NH_3 treatment process at relatively high temperature. From the high-resolution TEM images of LTO_NC and N-LTO_NC(650) (Fig. S2), although some discontinuous atomic lattices were observed in both samples due to the low crystallinity, the lattice spacings of 0.224 nm and 0.272 nm were assigned to the interplanar distance of (212) and ($-$122) planes of $La_2Ti_2O_7$ phase, respectively. Based on above results, nitrogen doping did not affect the crystal structure and the morphology of the $La_2Ti_2O_7$ compounds.

The reaction mechanism of $La_2Ti_2O_7$ nanocrystal was also investigated to understand the effects of the TEA in the hydrothermal reaction. On the one hand, the steric effect of TEA owing to its three-branched structure can lead to the formation of a layer of TEA molecules on the surface of $La_2Ti_2O_7$ nuclei at the beginning stage to restrict the particle size. On the other hand, the TEA molecule acts as a strong chelating agent to coordinate with La^{3+} ions to form La(III)-TEA complex at the initial stage (eq. (1)) during the hydrothermal reaction.[29,30] With the slow hydrolysis of TAL, the titanium precursor, ammonium lactate (AL) is produced, while Ti^{4+} is released and TiO_2 is gradually formed (eq. (2)) in the basic medium.[31,32] Then TiO_2 slowly dissolves in alkaline solution to form TiO_3^{2-} ions (eq. (3)).[33,34] The La^{3+} ions slowly dissociate from La(III)–TEA complex and react with the TiO_3^{2-} ions to form LTO_NC precursor with small particle size (eq. (4)). The rate of aggregation during the reaction is reduced probably due to the chelating property of TEA to form a layer on the surface of nuclei with steric barrier effect, which accounts for the nano-scaled particle size of $La_2Ti_2O_7$ after hydrothermal reaction. The reaction mechanism is suggested as follows:

$$TEA \rightarrow La(III) - TEA\ complex \leftrightarrow^{slow\ \ dissociation} La^{3+} \quad (1)$$

$$TAL \overset{slow\ \ dissociation}{\rightarrow} TiO_2 + AL \quad (2)$$

$$TiO_2\ +\ OH^-\ \rightarrow TiO_3^{2-} + H_2O \quad (3)$$

$$La(III) - TEA\ complex + TiO_3^{2-} \rightarrow La_2Ti_2O_7 + TEA \quad (4)$$

3.2. Optical property

The UV–vis diffuse reflectance spectra of the $La_2Ti_2O_7$ nanocrystal and the nitrogen doped $La_2Ti_2O_7$ are illustrated in Fig. 3a. The nitrogen doped $La_2Ti_2O_7$ exhibited enhanced visible light absorption compared with that of LTO_NC precursor, which should be attributed to the nitrogen doping in the $La_2Ti_2O_7$ lattice. The LTO_NC precursor absorbed the light with wavelength below *ca.* 338 nm, from which the band gap was estimated to be 3.86 eV. As the temperature of NH_3 treatment increased from 600 to 750 °C, the light absorption edge exhibited a gradual redshift toward $\lambda = 560$ nm. The absorption onsets of the nitrogen doped samples treated at 600, 650, 700 and 750 °C were at $\lambda \approx$ 345, 453, 491, and 560 nm, respectively. Thus, the bandgap became narrower as the NH_3 treatment temperature increased, indicating the degree of nitrogen doping strongly affected the visible light absorption of $La_2Ti_2O_7$. The higher N dopant concentration (Table 1) resulted in a larger add-on shoulder on the absorption edge and an enhancement of absorbance in the overall visible region. Among the nitrogen doped samples, N-LTO_NC(600) and N-LTO_NC(750) exhibited a parallel shift of the whole optical absorption edge to the direction towards longer wavelength, which is consistent with some reports indicating that the nitrogen doping in $La_2Ti_2O_7$ induced a narrowing band gap instead of introducing a impurity level in the mid-gap.[12,17] The derived bandgaps of N-LTO_NC(600) and N-LTO_NC(750) estimated from the Tauc plots (Fig. 3b) are 3.74 and 2.57 eV, respectively. However, for

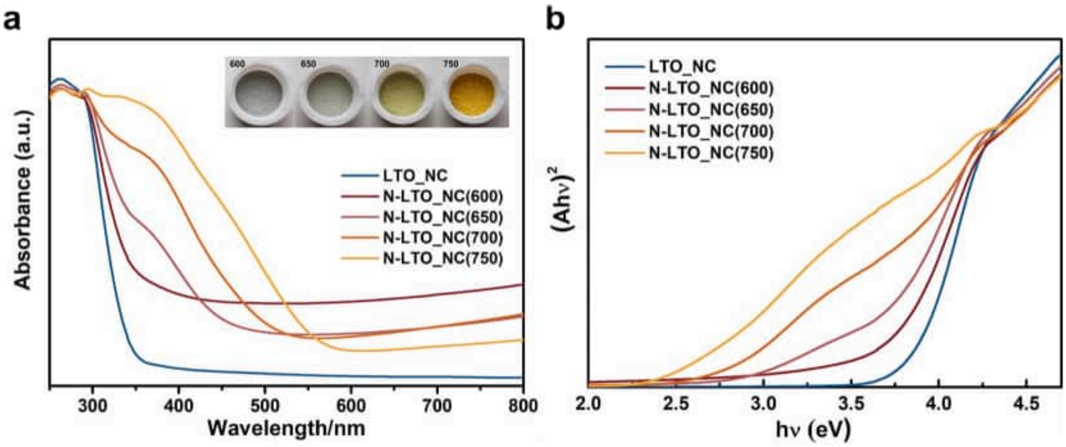

Fig. 3. (a) UV–vis diffuse reflectance spectra and (b) Tauc plots (b) of the $La_2Ti_2O_7$ nanocrystals before and after heat treatment under NH_3 gas flow at different temperatures.

Table 1
Atomic percentage of total nitrogen doping (N_{at}) and different nitrogen sites (substitutional N1 (N-Ti-N), substitutional N2 (O-Ti-N) and interstitial N3) using XPS spectra of N 1s core level of $La_2Ti_2O_7$ nanocrystal before and after NH_3 treatment at different temperatures.

Sample	N_{at} (at%)	**N1** (at%)	**N2** (at%)	**N3** (at%)
LTO_NC	3.50	–	3.50*	–
N-LTO_NC(600)	3.53	0.32	3.04	0.17
N-LTO_NC(650)	4.79	0.48	3.42	0.89
N-LTO_NC(700)	5.71	0.94	3.42	1.35
N-LTO_NC(750)	5.74	1.78	2.78	1.18

*The signal may include the nitrogen of TEA molecules on the oxide surface.

samples treated at 650 and 700 °C, the add-on shoulders were imposed onto the cutoff edge of the absorption spectrum from 350 to 490 nm, probably resulting from the formation of localized state between the band gap.[12] This behavior is similar to that of N-doped TiO_2.[35,36] Therefore, the doping nitrogen at 650 and 700 °C did not lead to band gap transitions. The gaps between the impurity level and conduction band minimum of N-LTO_NC(650) and N-LTO_NC(700) are estimated as 2.84 and 2.74 eV, respectively (Fig. 3b). The DFT calculation in the previous report indicated that this localized mid-gap energy state was formed by the mixture of N 2p, O 2p, Ti 3p and La 5d levels when N substituted for O in the lattice of $La_2Ti_2O_7$.[37] As a result of the N-doping, the color of N-LTO_NC changed from light green to yellowish with the increasing temperature of NH_3 treatment as shown in the inset in Fig. 3a, while the color of the LTO_NC precursor was white. It worth to mention that the charge transfer between dopant and host is also important due to the formation of dopant-induced states within the bandgap.[38] The introduced N 2p states can provide extra photo-generated electrons and additional pathways for the transition of electrons from the valence band, which is beneficial for the photocatalytic activity. [20] However, the mid-gap states of N doping for the enhancement of photocatalytic activity is limited because it also leads to a higher recombination rate, especially in a high concentration of N doping. The results of photocatalytic deNO_x will be discussed in section 3.4.

3.3. Measurement of chemical states and electronic structure

XPS measurements were used to determine the nitrogen configuration and concentration in the lattice of N-doped $La_2Ti_2O_7$. No nitrogen was detected on the surface of the LTO_P samples as verified by the absence of N 1s peak of the XPS survey spectrum of LTO_P. (Fig. S4a) However, a small amount of nitrogen (Table 1) was detected in the LTO_NC precursor synthesized with the TEA additive in the hydrothermal reaction. The state of nitrogen in LTO_NC can come from (i) TEA molecules on the oxide surfaces or (ii) directly doped nitrogen during the hydrothermal reaction. The FT-IR spectrum of LTO_NC shows the peaks around 3300–3700 cm^{-1} assigned to surface hydroxyl group and 1028–1070 cm^{-1} attributed to C-O stretching vibration were stronger than those in the spectrum of LTO_P (Fig. S5), indicating the presence of TEA on the oxide surfaces. On the other hand, the C 1s spectra in the XPS analyses of LTO_P and LTO_NC (Fig. S6a) show only the two peaks ascribed to C–C bond and C-O bond with different component ratios without a peak corresponding to C-N bonding at a binding energy of around ~ 286 eV,[39,40] meaning only a small amount of TEA in LTO_NC. Fig. S6b shows the N 1s core level spectra of LTO_NC sample without and with Ar^+ sputtering. The same peak was observed on the surface and inner layer of this sample, and the intensity after Ar^+ sputtering for 120 s was higher than that of the surface peak. The above results strongly suggested that most of N in LTO_NC should be pre-doped into LTO_NC precursor. The XPS results of the samples after the treatment with NH_3 revealed the presence of La, Ti, O and N elements. These results clearly indicate that the treatment with NH_3 for LTO compounds led to the nitrogen doping into $La_2Ti_2O_7$ precursor.

Usually, the doped nitrogen mainly existed in two states: (1) substitutional N introduced by displacing O atoms in the lattice of $La_2Ti_2O_7$; (2) interstitial N in the interval of the TiO_6 octahedra and bonded with oxygen. The N 1s XPS spectrum of LTO_NC shows only a single peak at a binding energy of around 398.05 eV. After the NH_3 treatment, the N 1s core level spectra of the N-doped samples (Fig. 4a) were deconvoluted into three regions: (N1) between 395 eV and 396 eV, (N2) centered around 398 eV to 399 eV, and (N3) localized at around 400 eV. According to the numerous previous experimental reports,[17,41–43] the position of N1 peak is widely regarded as substitutional sites of nitrogen, indicating that some O atoms in TiO_6 octahedra were substituted by N atoms to form N-Ti-N bond. The peak with binding energy at *ca.* 398 eV (N2) corresponds to N with a lower electron density than the N present in the N–Ti–N structure. The N2 nitrogen is ascribed to substitutional N atoms at oxygen sites with N-Ti-O bond in some reports.[16,17,44] Meanwhile, the chemical state of doped-N at N3 peaks has been still under controversy. Saha and Asahi investigated the N 1s XPS spectra of N-doped TiO_2 and assigned the peak of *ca.* 400 eV as molecularly chemisorbed γ-N_2.[15,45] Although the NH_3 was reported to be decomposed into N_2 and H_2 above 550 °C,[20] the chemisorbed N_2 molecules was strongly argued by Sato and co-workers.[46] Other researcher also considered the N 1s peak of N-doped TiO_2 at *ca.* 399–400 eV correspond to N-O bond (i.e., Ti-O-N)[46] or the other adsorbed species like NO dimers.[47] In addition, the examination of the literatures revealed that N 1s binding energy of $>$ 400 eV was assigned

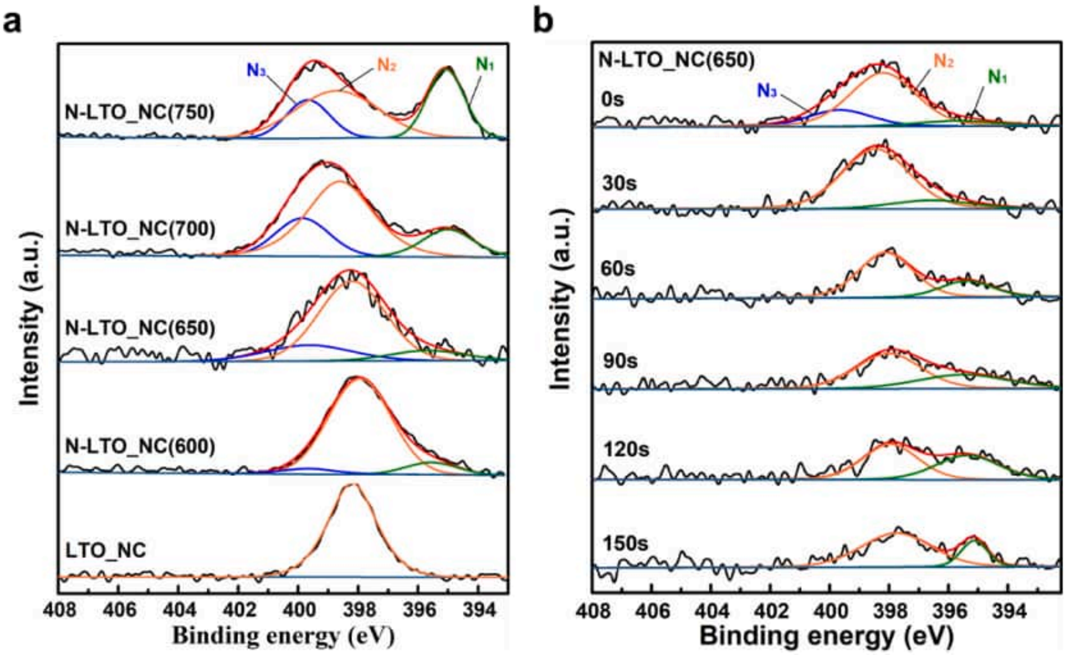

Fig. 4. (a) High resolution N 1s XPS spectra of LTO_NC before and after heat treatment in NH_3 gas flow at different temperatures without Ar^+ sputtering and (b) Ar^+ sputtering duration-dependent high resolution XPS spectra of N 1s of N-LTO_NC(650).

to interstitial N atoms in O-N-Ti linkage.[48,49] The DFT calculation results by Di Valentin *et al.* suggested that the calculated core level binding energy for interstitial N was higher than that of substitutional N in TiO_2. With the combination of experimental and theoretical results, they attributed the peak at 399–400 eV to interstitial N.[50] For N-LTO_NC(650) samples (Fig. 4b), the N3 peak disappeared after Ar^+ sputtering, suggesting that this bond only existed on the surface layer of the N-doped $La_2Ti_2O_7$. This result is consistent with the report that interstitial N only exist in the surface of N-doped sample, while the substitutional N existed not only in the surface but also the deep layer.[44] As the assignment of N3 peak is still disputable, we assume that the peak of N3 corresponds to interstitial nitrogen (Ti-O-N or O-N-Ti) formed after NH_3 treatment. As discussed above, we can summary that the N atoms can be doped into substitutional and interstitial sites in $La_2Ti_2O_7$ after the NH_3 treatment, and the N1 and N2 can be considered as the characteristic peak of substitutional N with N-Ti-N and N-Ti-O bonds, respectively; while N3 can be attributed to interstitial N.

The calculated atomic concentrations of total nitrogen dopants and the different nitrogen states are presented in Table 1. The atomic percentage of total nitrogen doping (N_{at}) on the surface of samples shows an increasing trend from 3.53 at% to 5.74 at% with the increasing temperature for the NH_3 treatment from 600 to 750 °C. Therefore, the N concentration in the $La_2Ti_2O_7$ samples was tuned by changing the NH_3 treatment temperature. The XPS spectra of N-doped $La_2Ti_2O_7$ reported by Meng *et al.* showed only 3.3 at% of nitrogen has been doped into $La_2Ti_2O_7$ after being heated in NH_3 flow.[17] For comparison, only 1.96 at% of N dopants was detected in N-LTO_P sample which prepared under the same conditions with N-LTO_NC(650). These mean that a remarkably large amount of N dopants were introduced into the N-LTO_NC samples. The theoretical calculation in some references suggested that when Ti-N bond was introduced in N-doped TiO_2, the bond length of Ti-O was elongated than that of pure TiO_2.[51,52] The pre-doped N in LTO_NC may also elongate the bond length of Ti-O and weaken this bond, which probably increases the solubility of substitutional nitrogen introduced by NH_3 treatment. The larger N doping amount in N-LTO_NC may be also attributed to the further introduced N from TEA molecules absorbed on the surface of LTO_NC precursor during the NH_3 treatment process. Also, the large surface area of LTO_NC in this study (69 m^2/g) can facilitate the reaction of $La_2Ti_2O_7$ with NH_3, which probably induced the more amount of nitrogen dopants.

In addition, the ratio of N1, N2, and N3 nitrogen in the N-LTO_NC samples changed depending on the treatment temperature for the nitrogen doping. The concentration of N1 gradually increased, while the atomic percentage of N2 decreased with rising nitridation temperature. Namely, the oxygens in N-Ti-O bond (N2) were substituted by further introduced N atoms to form N-Ti-N bonds (N1). These results indicated the high temperature is beneficial for nitrogen atoms to substitute more oxygen atoms in TiO_6 octahedron. This is in agreement with the previous report about N-doped TiO_2 case which reported higher temperature was favorable to the formation of substitutional N species.[53] The increment degree of the interstitial N (N3) with the increase of the treatment temperature was smaller than that of the total substitutional N atoms. It seems to be inconsistent with the previous report which indicated that the formation energy of interstitial N was lower than that of substitutional N in $La_2Ti_2O_7$.[54] However, it was also reported that the amount of interstitial N was limited in a very low range, and the treatment at high temperature led to the replacement of the interstitial N with the substitutional N.[20,53] Therefore, the smaller increment degree of N3 is not conflicting.

Fig. 4b shows the Ar^+ sputtering duration-dependent N 1s XPS spectra of N-LTO_NC(650) sample. Because the Ar^+ sputtering gradually shaves down the surface of the particles, the duration-dependent spectra can inform us the inside of the samples. Before the sputtering, several types of N sites (substitutional / interstitial nitrogen) existed on the surface of the particles like the cases of the other nitrogen doped $La_2Ti_2O_7$ samples. As mentioned above, a weak shoulder peak attributed to the interstitial N (N3) at 399.7 eV was removed by Ar^+ sputtering, meaning that the N dopants were mainly present as substitutional N in the deep layer of N-doped $La_2Ti_2O_7$ nanocrystals. The total amount of N in the surface of N-LTO_NC(650) sample was 4.79 at% and exhibited a descending trend with the increasing sputtering time to 150 s (2.40 at %). The N inside the sample might come from the N source of TEA used in the hydrothermal reaction. The gradual decrease of the N-Ti-N bond (N1) from the inside to the surface of N-LTO_NC(650) indicated that N-Ti-N bond (N1) was favorable forming in the inside of the sample.

Fig. 5 shows the Ti 2p and O 1s regions of LTO_NC and N-LTO_NC

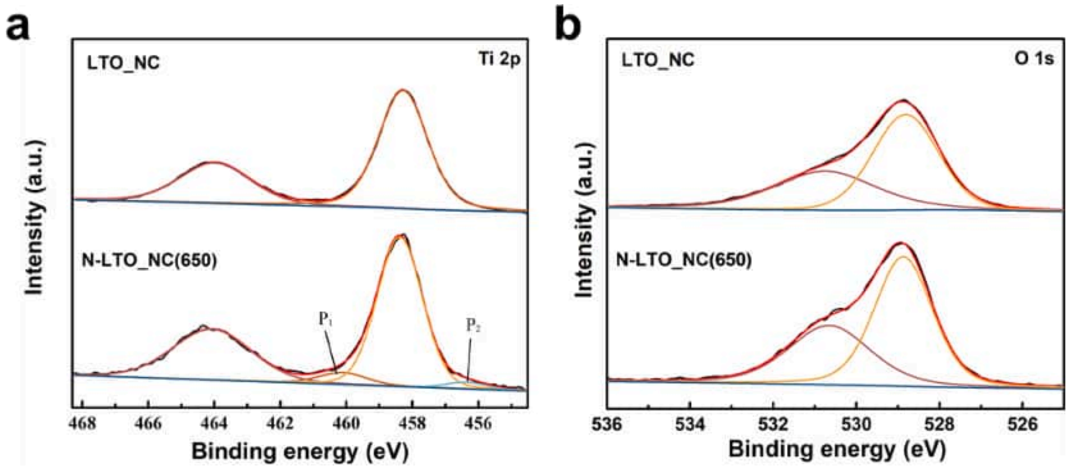

Fig. 5. High resolution XPS spectra of (a) Ti 2p and (b) O 1s of LTO_NC and N-LTO_NC(650).

(650). Both of the samples exhibited two major peaks associated with Ti $2p_{3/2}$ at approximately 458.3 eV and Ti $2p_{1/2}$ at approximately 464.1 eV, which is typical of Ti^{4+} in the perovskite structure of $La_2Ti_2O_7$ and assigned to the Ti–O bond.[17,55] After the NH_3 treatment, the Ti 2p spectra of N-LTO_NC(650) can be deconvoluted into four components. The P_1 peak (456.5 eV) and P_2 peak (460.1 eV) can be attributed to Ti^{3+} formed under reductive atmosphere in the nitridation.[56] The P_2 peak can be corresponded to the Ti-N bond.[57] These means that the nitrogen atoms were successfully doped into the $La_2Ti_2O_7$ lattice after NH_3 treatment. This result is also in agreement with the conclusion of N 1 s core level spectra observed in Fig. 4a.

The O 1s spectra shown in Fig. 5b was fitted into two components to oxygen in the lattice (*ca.* 528.8 eV) and surface hydroxyl groups (*ca.* 530.6 eV), respectively.[58] The O 1s spectra with and without Ar^+ sputtering of N-LTO_NC(650) in Fig. S7a showed the peak at 530.6 eV decreased drastically after Ar^+ sputtering for 150 s, suggesting that the most of the hydroxyl groups only existed on the surface of the sample. The comparison of La 3d core level spectra of LTO_NC precursor and N-LTO_NC(650) sample is shown in Fig. S7b. The figure clearly shows that no obvious change before and after the NH_3 treatment, indicating that there is no effect of nitrogen doping to La ions and the introduced nitrogen atoms prefer to substitute atoms in the TiO_6 octahedron slabs.[49]

In order to determine the energy band structure of these N-doped samples, the valence band spectra in XPS at very low binding energy is shown in Fig. 6a. The estimated valence band edge was shifted to the upward by the treatment with NH_3. The increase of the NH_3 treatment temperature led to gradually upward shift of the valence band edge. With the combination of estimated bandgap energy from the UV–Vis diffuse reflectance spectra (Fig. 3), the schematic illustration of the band structure diagrams of LTO_NC before and after heat treatment in NH_3 gas flow at different temperatures (600–750 °C) is presented at Fig. 6b. The specific bandgap energy, valence band edge and conduction edge values of the samples are listed in Table S1. The hybridization between N 2p orbitals of N dopant and O 2p orbital of the valence band of $La_2Ti_2O_7$ resulted in up-shift of the valence band edge.[59] The conduction band also show decreasing trend by increasing the amount of N dopant. The DFT calculation indicated all the elements in $La_2Ti_2O_7$ contribute to the valence band minimum (VBM) and conduction band maximum (CBM).[37] Thus, the N dopants not only affect the VBM but also change the oxygen amount in $La_2Ti_2O_7$ which may influence the CBM.

3.4. Evaluation of the photocatalytic activities

The photocatalytic activities of the prepared samples were investigated by NO decomposition degree under various wavelengths of light illumination (> 510, > 400 nm, > 290 nm). Fig. 7a presents the photocatalytic NO conversion ratio of LTO_NC and N-LTO_NC(600–750)

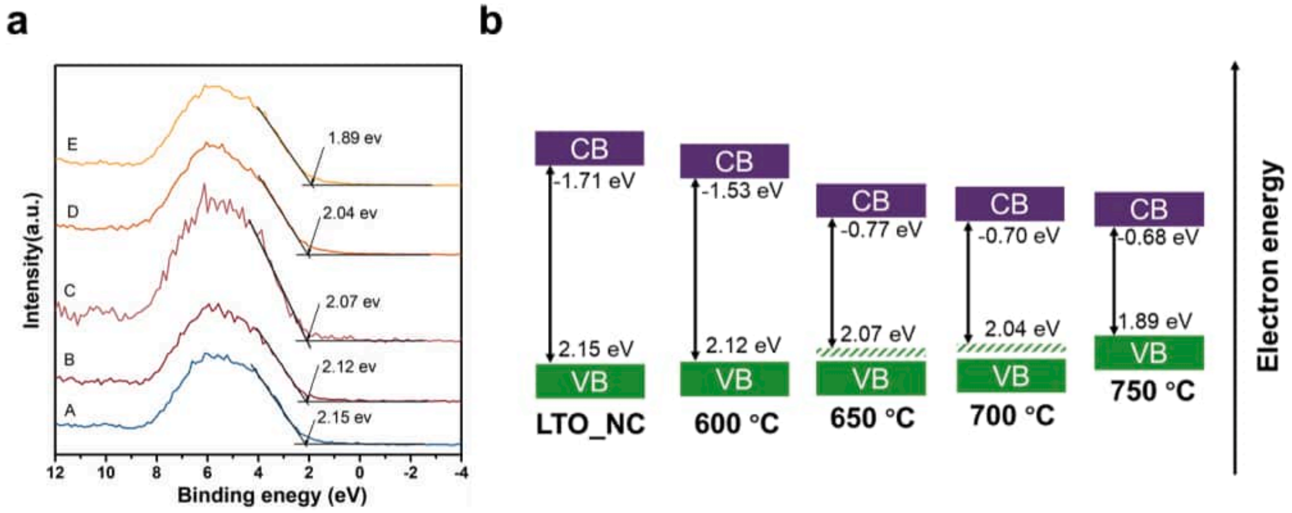

Fig. 6. (a) Valence band spectra vs Fermi level and (b) schematic illustration of band structure diagram of LTO_NC before and after heat treatment in NH_3 gas flow at different temperatures (600–750 °C) (the valence band energy and conduction band energy is vs Fermi level).

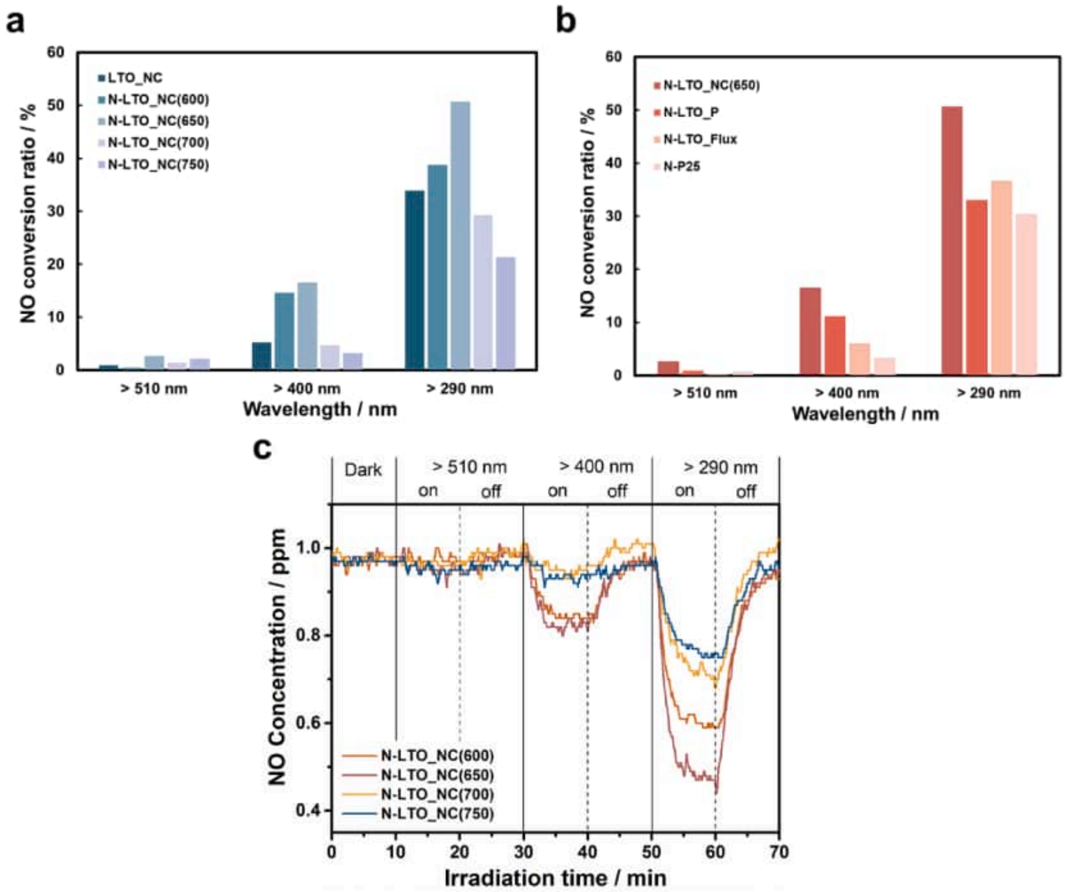

Fig. 7. (a) The photocatalytic decomposition of NO under different light wavelengths (> 510, > 400 and > 290 nm) of (a) LTO_NC and N-LTO_NC(600–750); (b)N-LTO_NC(650), N-LTO_P, N-LTO_Flux and N-P25 and (c) time dependence of NO decomposition activity of N-LTO_NC.

samples. For comparison, N-LTO_P, N-LTO_Flux and N-P25 were also tested for the photocatalytic NO_x oxidative decomposition activity under the same conditions (Fig. 7b). All the samples showed very low activity under the irradiation of long wavelength light (> 510 nm) because of the low energy light irradiation. The photocatalytic activities of N-LTO_NC(650) under the irradiation of the light with a wavelength of > 400 nm and > 290 nm were higher than those of N-LTO_P, N-LTO_Flux and N-P25, indicating the samples obtained by the method in this study exhibited high photocatalytic activity. Around 5 at% of nitrogen was introduced into P-25 measured by XPS analysis. The low activity of N-P25 is due to the high N dopant content with high defect concentration, which has also been proven by J. Wang *et al.*[20]

The time-dependence NO decomposition activity of N-LTO_NC (Fig. 7c) clearly shows the order of the photocatalytic activity over N-LTO_NC samples under both visible and UV light irradiation: N-LTO_NC (650) > N-LTO_NC(600) > N-LTO_NC(700) > N-LTO_NC(750). The highest activity was achieved in N-LTO_NC(650) sample, indicating its stronger NO absorption and decomposition ability. Furthermore, the quantum efficiencies (QE%) and photo-efficiencies (PE%) of the photocatalytic reactions calculated based on our previous research are given in Table S2.[60] The details of calculation were provided in supporting information. All samples possessed low QE% at > 510 nm range. The N-LTO_NC(650) sample also showed the highest QE% and PE% in the light wavelength of 400–500 nm and 290–400 nm ranges, respectively. Although the NH_3 treatments at 600 and 650 °C for LTO_NC led to increase of photocatalytic activity, the samples treated at 700 and 750 °C resulted in a drastic reduction of the photocatalytic performance even lower than that of $La_2Ti_2O_7$ precursor. With the mild N doping, the upward-shifted valence band and narrowed bandgap resulted in enhancement of visible light absorption and production of more photogenerated charge carriers. However, the further upward-shifted valence band led to weakened oxidation activity or deep localized N 2p state above the valence band acted as recombination centers for charge carriers, which can explain the reduced photocatalytic activity of samples treated at over 650 °C. The formation of oxygen vacancies in the high concentration of N doping may be another reason. The more reductive condition in the NH_3 treatment at 700 and 750 °C can lead to the formation of more amount of Ti^{3+} accompanied by oxygen vacancies, which can behave as the recombination centers of charge carriers to reduce the photocatalytic activity.[20]

Photoluminescence (PL) spectra of the pristine and nitrogen doped $La_2Ti_2O_7$ materials were obtained, to reveal the charge carriers separation efficiency (as shown in Fig. 8a). The samples exhibited typical PL peaks around 550 nm, which can be due to the recombination of photogenerated electrons and holes. Compared with LTO_NC, a distinct decrement of PL intensity for N-doped samples was observed, indicating that the introduced nitrogen anions played a key role in promoting the photogenerated electron-hole separation. Although N-LTO_NC(600) exhibited the lowest PL intensity, its photocatalytic activity was lower than N-LTO_NC(650) due to its large bandgap similar with LTO_NC, resulting in the insufficient light absorption. The high concentration of N doping, especially for N-LTO_NC(750) with high amount of substitutional N, is favored for the formation of oxygen vacancies acted as the charge carriers recombination centers, which is consistent with its low photocatalytic activity.[20] The formation of oxygen vacancy can also be confirmed by the ESR spectra (Fig. S8), which displayed a signal at g = 2.004 ascribed to the unpaired electron trapped in the oxygen vacancies in both LTO_NC and N-LTO_NC(650) samples. The oxygen vacancies were first introduced in the $La_2Ti_2O_7$ precursor by the pre-doped N in hydrothermal reaction, and N doping under highly reductive NH_3

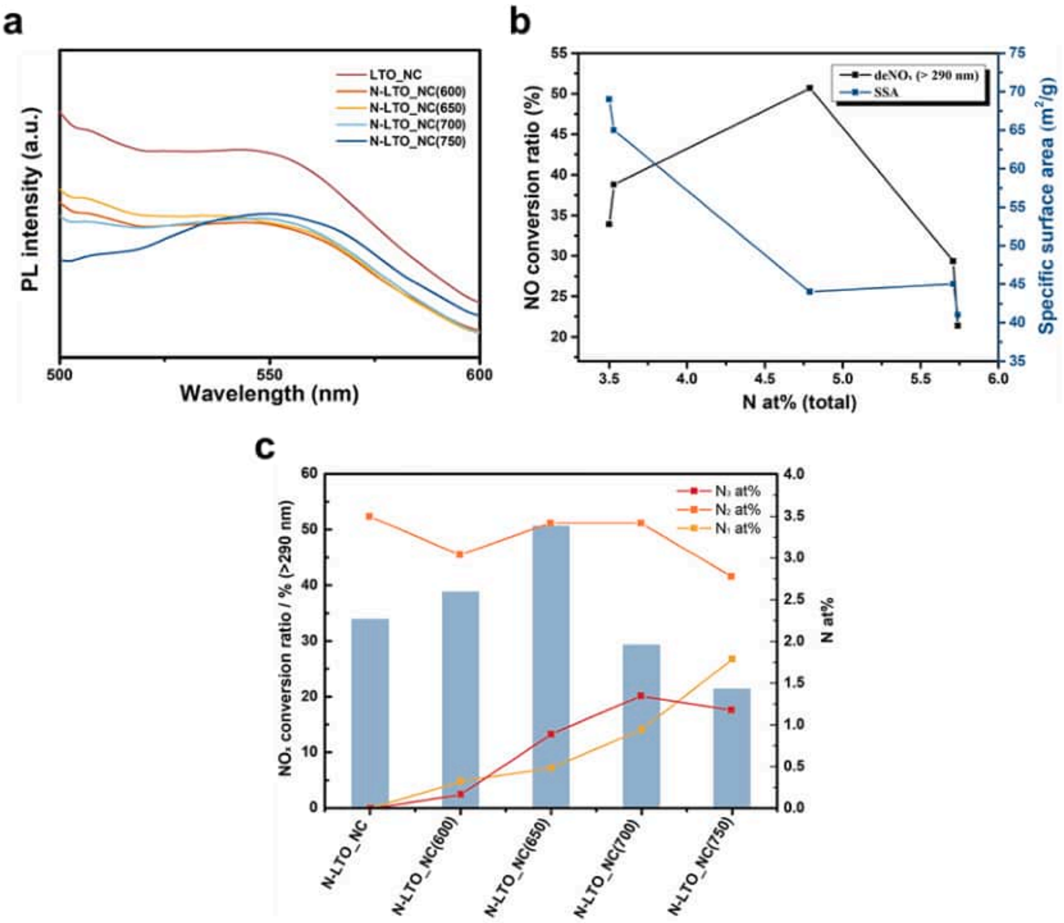

Fig. 8. (a) Photoluminescence spectra the pristine and nitrogen doped $La_2Ti_2O_7$; (b) the relations of total doped nitrogen (at%) with NO conversion ratios (> 290 nm) and specific surface areas; (c) correlation between the photocatalytic NO conversion ratio of LTO_NC*, N-doped $La_2Ti_2O_7$ nanocrystal under various temperature (600–750 °C) under the irradiation of light wavelengths > 290 nm and different nitrogen sites (N1, N2 and N3) calculated from XPS results. (*N2 in LTO_NC may include both the nitrogen species from TEA molecules and pre-doped nitrogen).

flow led to the formation of more oxygen vacancies. The theoretical calculation and experimental results showed that the combination of nitrogen anions and moderate oxygen vacancies are beneficial for the enhancement of photocatalytic activity,[54,61] which might be the reason for the best photocatalytic performance in N-LTO_NC(650). To further understand the enhanced photocatalytic NO_x decomposition activity of the N-doped $La_2Ti_2O_7$, the relation between introduced nitrogen anions with different sites and photocatalytic activity is need to be discussed.

On the one hand, the photocatalytic activity of N-doped $La_2Ti_2O_7$ was strongly related to the amount and the kind of the sites of the nitrogen dopants. The correlations between total atomic nitrogen amount (N_{at} at%) and photocatalytic activity as shown in Fig. 8b suggested that the photocatalytic activity (> 290 nm) increased with the increase of N_{at} at% until an optimal concentration was achieved in the structure of N-LTO_NC(650) sample. The optimal concentration of nitrogen should be less than 5%. When the nitrogen concentration further increased, the NO conversion ratios decreased. It should be noted that the nitrogen at different sites would have different influences on the photocatalytic activity. Fig. 8c shows the correlation between the atomic concentration of the three different nitrogen sites (N1, N2 and N3) on the basis of the XPS results and the photocatalytic activity (> 290 nm). The amount of N2 was not largely different between all the N-LTO_NC samples, and the substituted nitrogen with N-Ti-O bond did not affect the photocatalytic activity significantly. The NH_3 treatment for LTO_NC at 600 °C further introduced more nitrogen species with both substitutional (N1) and interstitial (N3) nitrogen. It was reported that the photocatalytic efficiency can be improved by the presence of both interstitial and substitutional N species in the case of the low concentration of doped nitrogen.[62] Therefore, the photocatalytic activity of N-LTO_NC(600) should be enhanced by the doping of both substitutional N (N1) and interstitial N (N3). According to some references, when the N-Ti-N bond is dominant species among the dopants with the increase of the total N concentration, the N-Ti-N bond (N1) [49,50] and the interstitial N (N3) [40] can possess negative and positive contribution to the visible light photocatalytic activity, respectively. In the case of N-LTO_NC(650), the suitable ratio of N1 and N3 atoms in N-LTO_NC should lead to the achievement of the highest photocatalytic activity. With higher concentration of N in samples treated at 700 and 750 °C, the photocatalytic efficiency decreased probably due to the further introduction of the N1 atoms with negative effect which led to unsuitable ratio of N1 and N3. Therefore, N-LTO_NC(700) and N-LTO_NC(750) with high concentration of N1 species should exhibit lower photocatalytic activity than those of N-LTO_NC(600) and N-LTO_NC(650), although they can absorb more visible light.

On the other hand, N-LTO_P and N-LTO_Flux exhibited lower photocatalytic NO_x decomposition activity than N-LTO_NC(650), although they were prepared under the same conditions of NH_3 treatment with N-LTO_NC(650). Compared to N-LTO_Flux (17 m^2/g), the large specific surface area of N-LTO_NC(650) (44 m^2/g) resulted in high adsorption ability of NO on the surfaces, and its smaller particle size induced less time for photogenerated charge carriers to diffuse from the inside to the surface. Therefore, the recombination of photogenerated electrons and holes should be suppressed to enhance the photocatalytic activity. The

specific surface area (SSA) of other N-LTO_NC samples under various treatment temperature are shown in Table S3. N-LTO_NC nitrided at 650 to 750 °C exhibited similar SSA, indicating that the influence of treatment temperature on the photocatalytic performance depends more on different chemical sites of introduced N dopants. As illustrated in Fig. 8b, the relation of total doped nitrogen (at%) with specific surface areas indicated that the SSA decreased at elevated total doped nitrogen amount. In addition, although the surface area of N-LTO_P (41 m^2/g) was similar to N-LTO_NC(650), the doped N amount of N-LTO_NC(650) (4.79 at%) was over twice than that of N-LTO_P (1.96 at%). These results suggest that both the specific surface area and N doping concentration affect the photocatalytic activity. Consequently, N-doped $La_2Ti_2O_7$ with optimal N concentration and nanocrystal size morphology can be applied as a promising visible light-driven photocatalyst with efficient NO decomposition ability. The photostability of the N-LTO_NC(650) sample was investigated by recycling and long-time irradiation test of the photocatalytic NO degradation. Fig. 9a shows that after three cycles of photocatalytic $deNO_x$ under different irradiation wavelength, there was no obvious decline of the activity N-LTO_NC(650) sample in three successive runs. As shown in Fig. 7, the photocatalytic NO removal efficiency of N-LTO_NC(650) was up to around 50% and remained stable for 60 min under the irradiation of > 290 nm light (Fig. 9b). It is obvious that no loss of photocatalytic ability occurred during the long-time measurement. The XRD patterns and TEM images (Fig. S9) of N-LTO_NC(650) after cycling tests showed that no alteration of crystal structure and morphology were observed, indicating the phase stability of the sample. The XPS spectra (Fig. S10) of La 3d and Ti 2p of N-LTO_NC (650) maintained approximately the same before and after photocatalytic decomposition of NO. The intensity for N 1s spectrum after three cycling tests increased compared with that of unmeasured sample, which could be attributed to the formation of HNO_3 on the surface of the photocatalysts. Meanwhile, the decrease of O 1s bonding (*ca.* 530.6 eV) was related to hydroxyl groups, which might be oxidized to generate hydroxyl radicals and participate in the reaction.[12,63] The above results elucidated that the sample was relatively stable and resistant to photo-corrosion during the recycling and prolonged time irradiation experiments of photocatalytic decomposition of NO.

Considering the band positions and redox potentials of reactants, a possible photocatalytic mechanism of $deNO_x$ reaction of N-doped $La_2Ti_2O_7$ samples might be proposed as follows.[64–66] N-doped $La_2Ti_2O_7$ nanocrystals are excited by light irradiation to produce photoexcited electrons (e^-) and holes (h^+) (eq. (5)). According to some references,[12,67] the valence band maximum position (vs NHE) of $La_2Ti_2O_7$ and N-doped $La_2Ti_2O_7$ were positive than $H_2O/\cdot OH$ (2.37 V vs NHE) and HNO_3/NO (0.94 V vs NHE).[63] Therefore, the photogenerated holes are considered to oxidize water into ·OH radicals (eq. (6)) and NO into HNO_3 (eq. (7)) during the process of photocatalytic NO decomposition. Because of the presence of oxygen from the air in the mixed gas and more negative conduction band position (vs. NHE) [12,67] than the standard redox potential of $O_2/\cdot O_2^-$ (-0.33 V vs NHE), [68] the highly active $\cdot O_2^-$ radical can be generated by the reaction of surface-trapped O_2 and photo-transferred e^- (eq. (8)) and then the ·OOH radical can be further formed in the conduction band (eq. (9)). [65,69,70] The NO can be oxidized by the generated $\cdot O_2^-$, ·OOH or ·OH to produce HNO_3 on the surface of the photocatalysts (eq. (10)). In addition, other intermediated products may also be produced, such as NO_2, N_2O_4 and HNO_2,[71] here we only list some possible basic photocatalytic reactions as follows:

$$N-LTO\_NC + h\nu \rightarrow e_{CB}^- + h_{VB}^+ \quad (5)$$

$$h_{VB}^+ + H_2O \rightarrow \cdot OH + H^+ \quad (6)$$

$$3h_{VB}^+ + NO + 2H_2O \rightarrow HNO_3 + 3H^+ \quad (7)$$

$$e_{CB}^- + O_2 \rightarrow \cdot O_2^- \quad (8)$$

$$\cdot O_2^- + H^+ \rightarrow \cdot OOH \quad (9)$$

$$NO + \left\{\cdot O_2^-, \cdot OOH, \cdot OH\right\} \rightarrow HNO_3 \quad (10)$$

4. Conclusions

Nitrogen-doped $La_2Ti_2O_7$ was achieved by treatment the $La_2Ti_2O_7$ nanocrystal under NH_3 flow at different temperature from 600 to 750 °C to extend the optical absorption into visible region. The obtained N-doped $La_2Ti_2O_7$ nanocrystal possesses the optical absorption of visible light to induce visible-light responding photocatalytic activity. The particle size of the obtained N-doped $La_2Ti_2O_7$ was considerably small by reflecting the size of the LTO_NC precursor which was obtained by hydrothermal reaction under a presence of TEA. The TEA additive in hydrothermal reaction controlled the morphology of LTO_NC within nanoscale size and attributed to the nitrogen in LTO_NC precursor as pre-doped N. The addition of TEA may largely affect the introduced nitrogen states and the substitution degree of nitrogen in N-LTO_NC after NH_3 treatment. The NH_3 treatment can introduce mainly substitutional N (N1) with N-Ti-N bond and interstitial N (N3). The different nitrogen sites affected the photocatalytic NO_x decomposition activity. The photocatalytic activity was independent from the concentration of substitutional N (N2) with N-Ti-O bond. Interstitial nitrogen doping sites (N3) is beneficial for the improvement of the photocatalytic activity of N-doped $La_2Ti_2O_7$, rather than high concentration of substitutional site

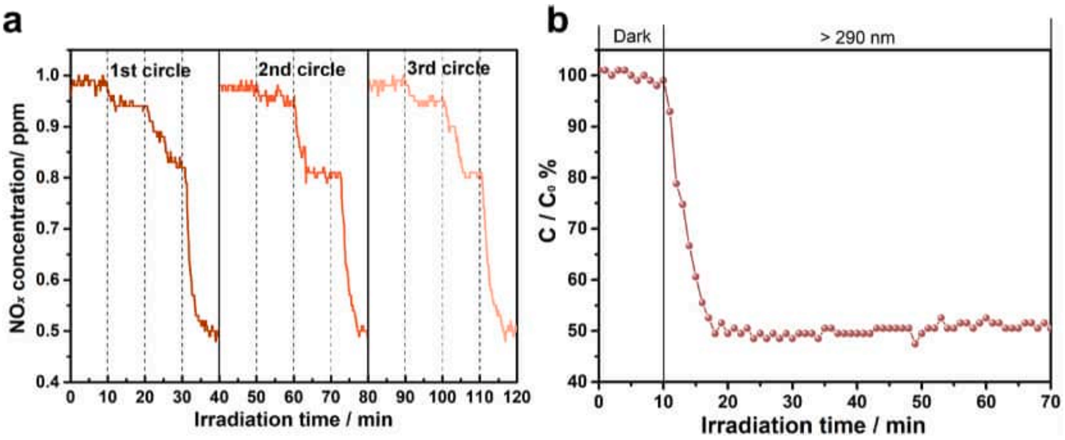

Fig. 9. (a) Recycling tests of photocatalytic NO degradation by N-LTO_NC(650) and (b) the photocatalytic NO removal efficiency of N-LTO_NC(650) for long term irradiation with light wavelength of > 290 nm.

(N1). N-LTO_NC(650) exhibited the highest visible light responsive photocatalytic activity for NO oxidation by the optimization of doped N concentration and the nano-structuration of the particle size. This research has shown that nitrogen doped $La_2Ti_2O_7$ can be tailor-synthesized from the viewpoint of morphology, optical properties, and nitrogen doping concentration to control the photocatalytic performance.

Declaration of Competing Interest

The authors declare that they have no known competing financial interests or personal relationships that could have appeared to influence the work reported in this paper.

Acknowledgments

This research was supported by the Murata Science Foundation, and by the JSPS Grant-in-Aid for Scientific Research on Innovative Areas “Mixed anion” (no. JP16H06439), and by the Dynamic Alliance for Open Innovation Bridging Human, Environment and Materials in Network Joint Research Center for Materials and Devices. The authors are grateful to Ms. S. Ogawa (Tohoku Univ.) for the XPS analyses.

Author Contributions

The manuscript was written through contributions of all authors. All authors have given approval to the final version of the manuscript.

Appendix A. Supplementary data

Supplementary data to this article can be found online at https://doi.org/10.1016/j.cej.2021.130220.

References

[1] G. Zhang, G. Liu, L. Wang, J.T. Irvine, Inorganic perovskite photocatalysts for solar energy utilization, Chem. Soc. Rev. 45 (21) (2016) 5951–5984.

[2] Y. Moriya, T. Takata, K. Domen, Recent progress in the development of (oxy) nitride photocatalysts for water splitting under visible-light irradiation, Coord. Chem. Rev. 257 (13–14) (2013) 1957–1969.

[3] G. Herrera, J. Jiménez-Mier, E. Chavira, Layered-structural monoclinic–orthorhombic perovskite $La_2Ti_2O_7$ to orthorhombic $LaTiO_3$ phase transition and their microstructure characterization, Mater. Charact. 89 (2014) 13–22.

[4] F. Meng, J. Li, Z. Hong, M. Zhi, A. Sakla, C. Xiang, N. Wu, Photocatalytic generation of hydrogen with visible-light nitrogen-doped lanthanum titanium oxides, Catal. Today 199 (2013) 48–52.

[5] K. Onozuka, Y. Kawakami, H. Imai, T. Yokoi, T. Tatsumi, J. Kondo, Perovskite-type $La_2Ti_2O_7$ mesoporous photocatalyst, J. Solid State Chem. 192 (2012) 87–92.

[6] D.W. Hwang, H.G. Kim, J.S. Lee, J. Kim, W. Li, S.H. Oh, Photocatalytic hydrogen production from water over M-doped $La_2Ti_2O_7$ (M= Cr, Fe) under visible light irradiation (λ>420 nm), J. Phys. Chem. B 109 (6) (2005) 2093–2102.

[7] Z. Wang, K. Teramura, S. Hosokawa, T. Tanaka, Photocatalytic conversion of CO_2 in water over Ag-modified $La_2Ti_2O_7$, Appl. Catal. B 163 (2015) 241–247.

[8] J. Ângelo, L. Andrade, L.M. Madeira, A. Mendes, An overview of photocatalysis phenomena applied to NO_x abatement, J. Environ. Manage. 129 (2013) 522–539.

[9] J. Lasek, Y.-H. Yu, J.C. Wu, Removal of NO_x by photocatalytic processes, J. Photochem. Photobiol. C 14 (2013) 29–52.

[10] H. Song, T. Peng, P. Cai, H. Yi, C. Yan, Hydrothermal synthesis of flaky crystallized $La_2Ti_2O_7$ for producing hydrogen from photocatalytic water splitting, Catal. Lett. 113 (1–2) (2007) 54–58.

[11] K. Li, Y. Wang, H. Wang, M. Zhu, H. Yan, Hydrothermal synthesis and photocatalytic properties of layered $La_2Ti_2O_7$ nanosheets, Nanotechnology 17 (19) (2006) 4863.

[12] X. Cai, J. Zhang, M. Fujitsuka, T. Majima, Graphitic-C_3N_4 hybridized N-doped $La_2Ti_2O_7$ two-dimensional layered composites as efficient visible-light-driven photocatalyst, Appl. Catal. B 202 (2017) 191–198.

[13] D.W. Hwang, K.Y. Cha, J. Kim, H.G. Kim, S.W. Bae, J.S. Lee, Photocatalytic degradation of CH_3Cl over a nickel-loaded layered perovskite, Ind. Eng. Chem. Res. 42 (6) (2003) 1184–1189.

[14] A. Kudo, R. Niishiro, A. Iwase, H. Kato, Effects of doping of metal cations on morphology, activity, and visible light response of photocatalysts, Chem. Phys. 339 (1–3) (2007) 104–110.

[15] R. Asahi, T. Morikawa, T. Ohwaki, K. Aoki, Y. Taga, Visible-light photocatalysis in nitrogen-doped titanium oxides, Science 293 (5528) (2001) 269–271.

[16] F. Zou, Z. Jiang, X. Qin, Y. Zhao, L. Jiang, J. Zhi, T. Xiao, P.P. Edwards, Template-free synthesis of mesoporous N-doped $SrTiO_3$ perovskite with high visible-light-driven photocatalytic activity, Chem. Commun. 48 (68) (2012) 8514–8516.

[17] F. Meng, Z. Hong, J. Arndt, M. Li, M. Zhi, F. Yang, N. Wu, Visible light photocatalytic activity of nitrogen-doped $La_2Ti_2O_7$ nanosheets originating from band gap narrowing, Nano Res. 5 (3) (2012) 213–221.

[18] H. Shi, X. Li, H. Iwai, Z. Zou, J. Ye, 2-Propanol photodegradation over nitrogen-doped $NaNbO_3$ powders under visible-light irradiation, J. Phys. Chem. Solids 70 (6) (2009) 931–935.

[19] A. Mukherji, C. Sun, S.C. Smith, G.Q. Lu, L. Wang, Photocatalytic hydrogen production from water using N-doped $Ba_5Ta_4O_{15}$ under solar irradiation, J. Phys. Chem. C 115 (31) (2011) 15674–15678.

[20] J. Wang, D.N. Tafen, J.P. Lewis, Z. Hong, A. Manivannan, M. Zhi, M. Li, N. Wu, Origin of photocatalytic activity of nitrogen-doped TiO_2 nanobelts, J. Am. Chem. Soc. 131 (34) (2009) 12290–12297.

[21] R. Abe, M. Higashi, K. Sayama, Y. Abe, H. Sugihara, Photocatalytic activity of R_3MO_7 and $R_2Ti_2O_7$ (R= Y, Gd, La; M= Nb, Ta) for water splitting into H_2 and O_2, J. Phys. Chem. B 110 (5) (2006) 2219–2226.

[22] H. Chen, G. Yu, G.D. Li, T. Xie, Y. Sun, J. Liu, H. Li, X. Huang, D. Wang, T. Asefa, Unique electronic structure in a porous Ga-In bimetallic oxide nano-photocatalyst with atomically thin pore walls, Angew. Chem. Int. Ed. 55 (38) (2016) 11442–11446.

[23] M. Hojamberdiev, A. Yamaguchi, K. Yubuta, S. Oishi, K. Teshima, Fabrication of $La_2Ti_2O_7$ crystals using an alkali-metal molybdate flux growth method and their nitridability to form $LaTiO_2N$ crystals under a high-temperature NH_3 atmosphere, Inorg. Chem. 54 (7) (2015) 3237–3244.

[24] F. Wang, S. Gu, R. Shang, P. Jing, Y. Wang, W. Li, Fabrication of AgBr/$La_2Ti_2O_7$ hierarchical heterojunctions: boosted interfacial charge transfer and high efficiency visible-light photocatalytic activity, Sep. Purif. Technol. 229 (2019), 115798.

[25] J. Wang, Y. Asakura, S. Yin, Preparation of $(Zn_{1+x}Ge)(N_2O_x)$ nanoparticles with enhanced NO_x decomposition activity under visible light irradiation by nitridation of Zn_2GeO_4 nanoparticles designed precisely, Nanoscale 11 (42) (2019) 20151–20160.

[26] R. Okada, K. Katagiri, Y. Masubuchi, K. Inumaru, Preparation of $LaTiO_2N$ using hydrothermally synthesized $La_2Ti_2O_7$ as a precursor and urea as a nitriding agent, Eur. J. Inorg. Chem. 2019 (9) (2019) 1257–1264.

[27] P. Zhang, M. Fujitsuka, T. Majima, TiO_2 mesocrystal with nitrogen and fluorine codoping during topochemical transformation: efficient visible light induced photocatalyst with the codopants, Appl. Catal. B 185 (2016) 181–188.

[28] V. Kalyani, B.S. Vasile, A. Ianculescu, M.T. Buscaglia, V. Buscaglia, P. Nanni, Hydrothermal synthesis of $SrTiO_3$ mesocrystals: single crystal to mesocrystal transformation induced by topochemical reactions, Cryst. Growth Des. 12 (9) (2012) 4450–4456.

[29] J. Liang, L. Li, M. Luo, Y. Wang, Fabrication of Fe_3O_4 octahedra by a triethanolamine-assisted hydrothermal process, Cryst. Res. Technol. 46 (1) (2011) 95–98.

[30] L. Li, J. Liang, H. Kang, J. Fang, M. Luo, X. Jin, TEA-assisted synthesis of single-crystalline Mn_3O_4 octahedrons and their magnetic properties, Appl. Surf. Sci. 261 (2012) 717–721.

[31] H. Möckel, M. Giersig, F. Willig, Formation of uniform size anatase nanocrystals from bis (ammonium lactato) titanium dihydroxide by thermohydrolysis, J. Mater. Chem. 9 (12) (1999) 3051–3056.

[32] S.-W. Lee, J. Drwiega, C.-Y. Wu, D. Mazyck, W.M. Sigmund, Anatase TiO_2 nanoparticle coating on barium ferrite using titanium bis-ammonium lactato dihydroxide and its use as a magnetic photocatalyst, Chem. Mater. 16 (6) (2004) 1160–1164.

[33] L. Yahui, M. Fancheng, F. Fuqiang, W. Weijing, C. Jinglong, Q. Tao, Preparation of rutile titanium dioxide pigment from low-grade titanium slag pretreated by the NaOH molten salt method, Dyes Pigm. 125 (2016) 384–391.

[34] P. Wang, X. Yi, Y. Lu, H. Yu, J. Yu, In-situ synthesis of amorphous H_2TiO_3-modified TiO_2 and its improved photocatalytic H_2-evolution performance, J. Colloid Interface Sci. 532 (2018) 272–279.

[35] Y. Cong, J. Zhang, F. Chen, M. Anpo, Synthesis and characterization of nitrogen-doped TiO_2 nanophotocatalyst with high visible light activity, J. Phys. Chem. C 111 (19) (2007) 6976–6982.

[36] G. Yang, Z. Jiang, H. Shi, T. Xiao, Z. Yan, Preparation of highly visible-light active N-doped TiO_2 photocatalyst, J. Mater. Chem. 20 (25) (2010) 5301–5309.

[37] J. Zhang, W. Dang, Z. Ao, S.K. Cushing, N. Wu, Band gap narrowing in nitrogen-doped $La_2Ti_2O_7$ predicted by density-functional theory calculations, Phys. Chem. Chem. Phys. 17 (14) (2015) 8994–9000.

[38] Y.-H. Lu, W.-H. Lin, C.-Y. Yang, Y.-H. Chiu, Y.-C. Pu, M.-H. Lee, Y.-C. Tseng, Y.-J. Hsu, A facile green antisolvent approach to Cu^{2+}-doped ZnO nanocrystals with visible-light-responsive photoactivities, Nanoscale 6 (15) (2014) 8796–8803.

[39] Y.-L. Huang, A. Baji, H.-W. Tien, Y.-K. Yang, S.-Y. Yang, C.-C.-M. Ma, H.-Y. Liu, Y.-W. Mai, N.-H. Wang, Self-assembly of graphene onto electrospun polyamide 66 nanofibers as transparent conductive thin films, Nanotechnology 22 (47) (2011), 475603.

[40] J.S. Park, J.M. Lee, S.K. Hwang, S.H. Lee, H.-J. Lee, B.R. Lee, H.I. Park, J.-S. Kim, S. Yoo, M.H. Song, A ZnO/N-doped carbon nanotube nanocomposite charge transport layer for high performance optoelectronics, J. Mater. Chem. 22 (25) (2012) 12695–12700.

[41] Z. Hua, X. Zhang, X. Bai, L. Lv, Z. Ye, X. Huang, Nitrogen-doped perovskite-type $La_2Ti_2O_7$ decorated on graphene composites exhibiting efficient photocatalytic activity toward bisphenol A in water, J. Colloid Interface Sci. 450 (2015) 45–53.

[42] H. Chen, A. Nambu, W. Wen, J. Graciani, Z. Zhong, J.C. Hanson, E. Fujita, J. A. Rodriguez, Reaction of NH_3 with titania: N-doping of the oxide and TiN formation, J. Phys. Chem. C 111 (3) (2007) 1366–1372.

[43] X. Wang, T.-T. Lim, Solvothermal synthesis of C-N codoped TiO_2 and photocatalytic evaluation for bisphenol A degradation using a visible-light irradiated LED photoreactor, Appl. Catal. B 100 (1–2) (2010) 355–364.

[44] G. Liu, L.-C. Yin, J. Wang, P. Niu, C. Zhen, Y. Xie, H.-M. Cheng, A red anatase TiO_2 photocatalyst for solar energy conversion, Energy Environ. Sci. 5 (11) (2012) 9603–9610.

[45] N.C. Saha, H.G. Tompkins, Titanium nitride oxidation chemistry: An x-ray photoelectron spectroscopy study, J. Appl. Phys. 72 (7) (1992) 3072–3079.

[46] S. Sato, R. Nakamura, S. Abe, Visible-light sensitization of TiO_2 photocatalysts by wet-method N doping, Appl. Catal. A 284 (1–2) (2005) 131–137.

[47] T. Herranz, X. Deng, A. Cabot, Z. Liu, M. Salmeron, In situ XPS study of the adsorption and reactions of NO and O_2 on gold nanoparticles deposited on TiO_2 and SiO_2, J. Catal. 283 (2) (2011) 119–123.

[48] J.L. Gole, J.D. Stout, C. Burda, Y. Lou, X. Chen, Highly efficient formation of visible light tunable $TiO_{2-x}N_x$ photocatalysts and their transformation at the nanoscale, J. Phys. Chem. B 108 (4) (2004) 1230–1240.

[49] M. Sathish, B. Viswanathan, R. Viswanath, C.S. Gopinath, Synthesis, characterization, electronic structure, and photocatalytic activity of nitrogen-doped TiO_2 nanocatalyst, Chem. Mater. 17 (25) (2005) 6349–6353.

[50] C. Di Valentin, G. Pacchioni, A. Selloni, S. Livraghi, E. Giamello, Characterization of paramagnetic species in N-doped TiO_2 powders by EPR spectroscopy and DFT calculations, J. Phys. Chem. B 109 (23) (2005) 11414–11419.

[51] G. Pan, W. Jing, L. Qing-Ju, Z. Wen-Fang, First-principles study on anatase TiO_2 codoped with nitrogen and praseodymium, Chin. Phys. B 19 (8) (2010), 087103.

[52] M. Sahoo, A. Yadav, S. Jha, D. Bhattacharyya, T. Mathews, N. Sahoo, S. Dash, A. Tyagi, Nitrogen location and Ti-O bond distances in pristine and N-doped TiO_2 anatase thin films by X-ray absorption studies, J. Phys. Chem. C 119 (31) (2015) 17640–17647.

[53] C. Feng, Y. Wang, J. Zhang, L. Yu, D. Li, J. Yang, Z. Zhang, The effect of infrared light on visible light photocatalytic activity: an intensive contrast between Pt-doped TiO_2 and N-doped TiO_2, Appl. Catal. B 113 (2012) 61–71.

[54] Z. Ma, K. Wu, R. Sa, Q. Li, C. He, Z. Yi, Mechanism of enhanced photocatalytic activities on N-doped $La_2Ti_2O_7$: An insight from density-functional calculations, Int. J. Hydrogen Energy 40 (2) (2015) 980–989.

[55] S. Ardizzone, C.L. Bianchi, G. Cappelletti, S. Gialanella, C. Pirola, V. Ragaini, Tailored anatase/brookite nanocrystalline TiO_2. The optimal particle features for liquid-and gas-phase photocatalytic reactions, J. Phys. Chem. C 111 (35) (2007) 13222–13231.

[56] B. Bharti, S. Kumar, H.-N. Lee, R. Kumar, Formation of oxygen vacancies and Ti^{3+} state in TiO_2 thin film and enhanced optical properties by air plasma treatment, Sci. Rep. 6 (2016) 32355.

[57] J.F. Moulder, Handbook of X-ray photoelectron spectroscopy, Physical electronics (1995) 230–232.

[58] U.S. Shenoy, H. Bantawal, D.K. Bhat, Band engineering of $SrTiO_3$: effect of synthetic technique and site occupancy of doped rhodium, J. Phys. Chem. C 122 (48) (2018) 27567–27574.

[59] A. Kasahara, K. Nukumizu, T. Takata, J.N. Kondo, M. Hara, H. Kobayashi, K. Domen, $LaTiO_2N$ as a visible-light ($\leq$ 600 nm)-driven photocatalyst (2), J. Phys. Chem. B 107 (3) (2003) 791–797.

[60] S. Yin, K. Ihara, Y. Aita, M. Komatsu, T. Sato, Visible-light induced photocatalytic activity of $TiO_{2-x}A_y$ (A= N, S) prepared by precipitation route, J. Photochem. Photobiol. A 179 (1–2) (2006) 105–114.

[61] L. Preethi, R.P. Antony, T. Mathews, L. Walczak, C.S. Gopinath, A study on doped heterojunctions in TiO_2 nanotubes: an efficient photocatalyst for solar water splitting, Sci. Rep. 7 (1) (2017) 1–15.

[62] F. Spadavecchia, G. Cappelletti, S. Ardizzone, C.L. Bianchi, S. Cappelli, C. Oliva, P. Scardi, M. Leoni, P. Fermo, Solar photoactivity of nano-N-TiO_2 from tertiary amine: role of defects and paramagnetic species, Appl. Catal. B 96 (3–4) (2010) 314–322.

[63] Z. Wang, Y. Huang, L. Chen, M. Chen, J. Cao, W. Ho, S.C. Lee, In situ g-C_3N_4 self-sacrificial synthesis of a g-C_3N_4/$LaCO_3OH$ heterostructure with strong interfacial charge transfer and separation for photocatalytic NO removal, J. Mater. Chem. A 6 (3) (2018) 972–981.

[64] S. Yin, B. Liu, P. Zhang, T. Morikawa, K.-I. Yamanaka, T. Sato, Photocatalytic oxidation of NO_x under visible LED light irradiation over nitrogen-doped titania particles with iron or platinum loading, J. Phys. Chem. C 112 (32) (2008) 12425–12431.

[65] Y. Asakura, Y. Inaguma, K. Ueda, Y. Masubuchi, S. Yin, Synthesis of gallium oxynitride nanoparticles through hydrothermal reaction in the presence of acetylene black and their photocatalytic NO_x decomposition, Nanoscale 10 (4) (2018) 1837–1844.

[66] S. Komatsuda, Y. Asakura, J.J.M. Vequizo, A. Yamakata, S. Yin, Enhanced photocatalytic NO_x decomposition of visible-light responsive F-TiO_2/(N, C)-TiO_2 by charge transfer between F-TiO_2 and (N, C)-TiO_2 through their doping levels, Appl. Catal. B 238 (2018) 358–364.

[67] M. Xia, X. Yan, H. Li, N. Wells, G. Yang, Well-designed efficient charge separation in 2D/2D N doped $La_2Ti_2O_7$/$ZnIn_2S_4$ heterojunction through band structure/morphology regulation synergistic effect, Nano Energy 105401 (2020).

[68] P.M. Wood, The potential diagram for oxygen at pH 7, Biochem. J 253 (1) (1988) 287.

[69] H. Gerischer, A. Heller, The role of oxygen in photooxidation of organic molecules on semiconductor particles, J. Phys. Chem. 95 (13) (1991) 5261–5267.

[70] S. Yin, T. Sato, Synthesis and photocatalytic properties of fibrous titania prepared from protonic layered tetratitanate precursor in supercritical alcohols, Ind. Eng. Chem. Res. 39 (12) (2000) 4526–4530.

[71] H. Wu, R. Chen, H. Wang, W. Cui, J. Li, J. Wang, C. Yuan, L. Zhuo, Y. Zhang, F. Dong, An atomic insight into BiOBr/$La_2Ti_2O_7$ p–n heterojunctions: interfacial charge transfer pathway and photocatalysis mechanism, Catal. Sci. Technol. 10 (3) (2020) 826–834.

Nano Research
2022, 15(1): 438–445
ISSN 1998-0124 CN 11-5974/O4
https://doi.org/10.1007/s12274-021-3498-5

Effects of Bi-dopant and co-catalysts upon hole surface trapping on $La_2Ti_2O_7$ nanosheet photocatalysts in overall solar water splitting

Xiaoyan Cai[1,2], Liang Mao[1,2], Mamoru Fujitsuka[3], Tetsuro Majima[3], Sujan Kasani[4], Nianqiang Wu[5], and Junying Zhang[2] (✉)

[1] School of Materials Science and Physics, Jiangsu Province Engineering Laboratory of High Efficient Energy Storage Technology and Equipment, China University of Mining and Technology, Xuzhou 221116, China
[2] School of Physics, Beihang University, Beijing 100191, China
[3] The Institute of Scientific and Industrial Research (SANKEN), Osaka University, Mihogaoka 8-1, Ibaraki, Osaka 567-0047, Japan
[4] Department of Mechanical and Aerospace Engineering, West Virginia University, Morgantown, WV 26506-6106, USA
[5] Department of Chemical Engineering, University of Massachusetts Amherst, Amherst, MA 01003-9303, USA

© Tsinghua University Press and Springer-Verlag GmbH Germany, part of Springer Nature 2021

Received: 8 February 2021 / **Revised:** 5 April 2021 / **Accepted:** 6 April 2021

ABSTRACT

Pristine and Bi-doped lanthanum titanium oxide ($La_2Ti_2O_7$) nanosheets have been synthesized as photocatalysts for overall solar water splitting. The surface hole trap is a critical factor that limits the photocatalytic activity of pristine $La_2Ti_2O_7$. Deposition of cobalt phosphate (Co-Pi) and platinum (Pt) nanoparticles on $La_2Ti_2O_7$ cannot remove the surface traps although they are essential for enabling the oxygen and hydrogen evolution reactions. It is interesting that doping bismuth (Bi) into $La_2Ti_2O_7$ nanosheets has eliminated the surface traps due to surface enrichment of Bi. The Co-Pi/Bi-$La_2Ti_2O_7$/Pt nanosheets exhibit increasing photocatalytic activity toward overall water splitting with increasing the Bi-dopant level up to 5 at.%. Further increasing the Bi-dopant level leads to the formation of localized states above the valence band, leading to the lifetime reduction of photogenerated charge-carriers, and jeopardizing the photocatalytic activity. This work proposes an effective strategy to address the surface trapping and surface catalysis issues in the nanostructured metal oxide photocatalysts.

KEYWORDS

photocatalyst, water splitting, surface trap, co-catalyst, solar fuel

1 Introduction

Photocatalytic water splitting without sacrificial agents on semiconductor photocatalysts is one of the most cost-effective and scalable means for hydrogen generation [1]. Metal oxides, such as TiO_2, $SrTiO_3$, $La_2Ti_2O_7$, $BiVO_4$, and Fe_2O_3, and metal nitrides, such as GaN:ZnO and GaN, are still considered to be major candidates for water-splitting photocatalysts due to their high photo-stability or low-cost [1, 2]. Band structure and surface trap have been identified as the dominant factors governing the photocatalytic performance of metal oxides. For example, because Fe_2O_3 possesses an almost ideal band gap (1.9–2.2 eV) as a single material photocatalyst for solar water splitting, it has been placed on the spotlight of research in the photocatalysis field. TiO_2 has a lot of appealing characteristics as a water-splitting photocatalyst except for its large band gap (3.2 eV for anatase TiO_2). Doping is the most common method for tuning band gap of metal oxides. However, doping, in most cases, does not narrow the band gap, but induces the shallow or deep states in the mid-gap where charge carriers are immobile [1, 3]. Surface traps have been found to prevail on TiO_2, $BiVO_4$, and Fe_2O_3, holding photo-generated charge carriers on the oxide surface. Typically, co-catalysts (such as cobalt phosphate (Co-Pi) and cobalt oxides) [2, 4, 5] or a secondary semiconductor layer [6] is deposited on the metal oxides to eliminate surface traps.

It is well known that it is difficult to narrow the band gap of metal oxides by not inducing mid-gap states. However, the previous studies have shown that doping of nitrogen into $La_2Ti_2O_7$ can broaden the valence band, which resulted in narrowing of band gap instead of introduction of discrete mid-gap states [7, 8]. A high level of nitrogen dopant can transform $La_2Ti_2O_7$ into $LaTiO_2N$ with a band gap of 2.1 eV [9]. This has aroused strong interest in $La_2Ti_2O_7$ because it has a great potential in being engineered into a high-performance visible-light photocatalyst. However, little is known about the surface traps on $La_2Ti_2O_7$ although surface trapping of photo-generated charge carries has been extensively investigated in TiO_2 [10–12], $SrTiO_3$ [13, 14], $BiVO_4$ [15, 16], and Fe_2O_3 [17, 18]. To achieve high performance of photocatalysis toward water splitting, it is essential to examine whether surface trap is a controlling factor, and to develop an effective strategy to suppress the surface trap if there is [19].

In the present work, a photocatalyst based on $La_2Ti_2O_7$ (LTO) nanosheets is designed for overall water splitting. It is verified for the first time that hole traps exhibit on the LTO surface. Deposition of Co-Pi on the LTO surface cannot suppress the surface traps although Co-Pi serves as an efficient co-catalyst in this work. It is interesting that doping is discovered to suppress the surface trapping of holes on LTO, but it does not increase the charge carrier concentration in LTO. To investigate the

Address correspondence to zjy@buaa.edu.cn

effects of Bi doping on the photocatalytic performance, various concentrations of Bi were doped to LTO through a one-step hydrothermal process. Also, Co-Pi and Pt nanoparticles were deposited onto the Bi doped-LTO nanosheets as the co-catalysts toward the oxygen evolution reaction (OER) and the hydrogen evolution reaction (HER), respectively. Photocatalytic water splitting testing was performed on the LTO photocatalyst doped with different concentrations of Bi. The light absorption, band structure, charge concentration and charge dynamic processes were studied by the combination of density functional theory (DFT) simulation with various analytic techniques, including X-ray photoelectron spectroscopy (XPS), surface photovoltage (SPV) spectroscopy, and femtosecond time-resolved diffuse reflectance (TDR) spectroscopy. The results reveal that the Co-Pi/5% Bi doped-LTO/Pt heterojunction exhibits stable photocatalytic activity toward overall water splitting while the Co-Pi/pristine-LTO/Pt heterojunction shows negligible photocatalytic activity. This indicates the critical role of Bi doping in photocatalytic water splitting.

2 Experimental

2.1 Preparation of Bi-LTO

The pristine and Bi-doped LTO photocatalysts were synthesized by a modified hydrothermal method. Typically, 0.2 g CTAB was dissolved into 10 mL deionized water, resulting in a transparent solution. After a certain amount of $Ti(SO_4)_2$ (2 mmol), $La(NO_3)_3{\cdot}6H_2O$ (1.86–1.98 mmol), and $Bi(NO_3)_3{\cdot}9H_2O$ (0.02–0.14 mmol) with different levels of Bi/(Bi + La) (1.0 at.%, 3.0 at.%, 5.0 at.%, and 7.0 at.%) were introduced into the solution successively, the clear solution became opaque, leading to light yellow precipitates. After 10 mL 2 M NaOH solution was dropped into the above solution, white precipitates were formed. After magnetically stirring for 4 h, the mixture was loaded into a Teflon cup (40 mL) in a stainless autoclave, and then heated at 240 °C for 24 h. After the autoclave was cooled down to room temperature, the white precipitates were collected by centrifugation, washed and dried in air at 80 °C for 12 h. The products with the Bi/(La + Bi) ratio ranging from 1 at.%–7 at.% were noted as 0.01Bi-LTO, 0.03Bi-LTO, 0.05Bi-LTO, and 0.07Bi-LTO, respectively.

2.2 Deposition of co-catalysts

For synthesis of Co-Pi/Bi-LTO/Pt, 30 mg Bi-LTO powder was mixed with 30 mL solution containing 0.5 mM $CoCl_2$ in 0.1 M sodium phosphate (Na-Pi) buffer at pH 7.0, forming a homogenous suspension. The suspension was then exposed to light from a Xe lamp (Beijing Bofeilai Co. Ltd., China; 300 mW·cm^{-2}) under magnetically stirring for 1 h. After washing with water several times, the obtained Co-Pi/Bi-LTO was subsequently dispersed in 30 mL H_2PtCl_6 aqueous solution, followed by irradiation under the Xe lamp at room temperature for 30 min. Finally, the products were collected by centrifugation, and then dried in vacuum at 40 °C overnight, resulting in the Co-Pi/Bi-LTO/Pt composite.

2.3 Photocatalytic water splitting

The photocatalyst (30 mg) was firstly suspended in deionized water (30 mL) in a quartz tube (60 mL), which connecting to a circulating cooling system. The tube was then sealed with a rubber septum and purged with Ar gas for 20 min before the irradiation was initiated. The sample was irradiated with a Xe light source (PerfectLight Co. Ltd., China; 500 mW·cm^{-2}) with constant magnetic stirring at room temperature. The gases produced were analyzed with a gas chromatograph (Shimadzu GC-2030, Japan) equipped with an MS-5A column and a thermal conductivity detector (TCD). For the half reaction, methanol (20 vol.%), and $AgNO_3$ (0.1 M) aqueous solution was used for photocatalytic H_2 and O_2 production, respectively. Apparent quantum efficiency (AQE) for overall water splitting was measured using a low-pressure mercury lamp (32 W) with central wavelength of 254 nm, and LED lamp (Beijing Pulinsaisi Co. Ltd., China; 100 W) with central wavelengths of 365 and 400 nm, respectively.

The details of theoretical calculations, SPV measurements, TDR spectroscopy, and other characterizations are summarized in the Electronic Supplementary Material (ESM).

3 Results

3.1 Microstructure of Bi-LTO

The pristine $La_2Ti_2O_7$ and the Bi-doped $La_2Ti_2O_7$ with four different levels of Bi (1.0 at.%, 3.0 at.%, 5.0 at.%, and 7.0 at.%) were prepared by one-step hydrothermal process. Accordingly, the samples were noted as LTO for the pristine sample, 0.01Bi-LTO, 0.03Bi-LTO, 0.05Bi-LTO, and 0.07Bi-LTO for the doped samples. Inductively coupled plasma (ICP) emission spectrometry was used to verify the contents of metal elements in the pristine and doped samples, giving an actual Bi dopant concentration of 4.11 at.% for 0.05Bi-LTO (Table S1 in the ESM). The pristine and doped LTO samples exhibited a morphology of nanosheets with a lateral size in the range of 200–1,000 nm (Fig. 1 and Fig. S1 in the ESM). The nanosheets were around 13 nm thick, which was consistent with the pristine and Bi-doped nanosheets (Fig. S2 in the ESM). The crystal structure of the LTO nanosheets remained the monoclinic phase after Bi incorporation, as confirmed by X-ray diffraction (XRD) patterns (Fig. 1(a)). As superiority of equivalent doping, neither a noticeable peak shift (lattice constant alteration) nor peak intensity reduction (crystallinity reduction) was observed in the Bi-doped samples. The peak ratios for 2θ = 31° and 32° were evolved gradually with Bi doping, which might be ascribed to a change in the periodic symmetry in the LTO crystal structure by Bi doping. The lattice parameter and the crystallite size were calculated based on the XRD patterns (Table S2 in the ESM), which showed a slight change in the lattice parameter as a function of the doping level. Individual LTO nanosheets were single-crystalline, showing

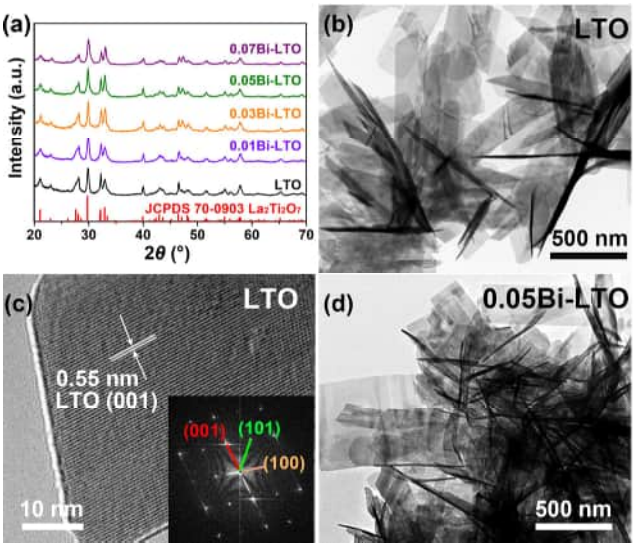

Figure 1 Structure and morphology of nanosheet photocatalysts: (a) XRD patterns, (b) transmission electron microscopy (TEM) image of pristine LTO, (c) high-resolution TEM (HRTEM) image of pristine LTO, and (d) TEM image of 0.05Bi-LTO.

a lattice fringe with a spacing of about 0.55 nm (Fig. 1(c)). And the nanosheets grew along the (001) direction with the dominant facet (010), as explained in ref. [20]. After Bi atoms were introduced, Bi atoms were distributed uniformly along the plane of the nanosheets (Figs. S3 and S4 in the ESM), and the structure of LTO was preserved (Fig. S5 in the ESM). Detailed XPS analysis showed that Bi had a chemical valence of +3, which indicated successful doping (Figs. S6 and S7 in the ESM). In addition, because LTO is a photoactive material which has low surface catalytic activity toward OER and HER, Co-Pi and Pt nanoparticles were deposited on the Bi-LTO nanosheets after hydrothermal processing (Figs. S8–S11 in the ESM), which served as the OER and HER co-catalysts [21]. The Co-Pi and Pt nanoparticles were distributed discretely on the nanosheet surface, approximately 2 and 5 nm in size, respectively (Fig. S8 in the ESM). Apart from metallic Pt, XPS also puts forward the presence of oxidized Pt which would play a role in suppressing the hydrogen oxidation in photocatalytic water splitting [22].

LTO, which is a member of the family of $A_2B_2O_7$-type perovskite, provides a good parent material for chemical substitutions, where both A and B sites in LTO can be substituted by diverse component ions while maintaining the original perovskite structure [23]. As an ionic conductor at room temperature, the ions on the A sites of LTO phase show high ion mobility, which can migrate to the thermodynamic equilibrium locations easily [24]. Hence, the ions doped on the A sites tend to be segregated at locations with lower formation energy. Recently, photocatalysts based on Bi^{3+} cations with the d^{10} configuration have been widely studied because the intrinsic polarization induced by the Bi 6s lone pair electron is beneficial to the separation of photogenerated electrons and holes [25, 26]. Considering the valence and ionic radius, we conducted equivalent doping of Bi^{3+} (with an ionic radius of 1.03 Å) to substitute La^{3+} (1.06 Å) in A sites of LTO because non-equivalent doping usually causes more structural defects.

3.2 Band structure of Bi-LTO

The ultraviolet–visible (UV–vis) absorption spectra show that the pristine and the Bi-doped LTO nanosheets displayed a strong absorption band at UV region with the absorption edge at around 365 nm (Fig. 2(a)). After Bi doped into LTO, the slight absorption tails were observed in the range of 380–600 nm (Fig. 2(b)). The band gap was derived to 3.4 eV for both the pristine and the Bi-doped LTO samples; and Bi doping did not change the band gap (Fig. S12 in the ESM). Further XPS examination of the valence band shows that the valence band maximum of the 0.05Bi-LTO sample exhibited 0.3 eV of shift toward the Fermi level with respect to the pristine LTO (Fig. 2(c)). In addition, M–S measurement was performed on the pristine LTO and 0.05Bi-LTO samples (Fig. 2(d)). The flat-band potential was estimated to be −0.62 and −0.64 V vs. the reversible hydrogen electrode (RHE) for LTO and 0.05Bi-LTO, respectively, which indicated that Bi doping did not change the Fermi level and band bending. Typically, surface catalysis can be evaluated with overpotential. If there is no change in the overpotential, one can say that Bi doping does not change the surface catalysis activity. Also, it can be seen from the M–S plot that the slope of 0.05Bi-LTO was slightly higher than that of LTO, which implied that the charge carrier concentration in the 0.05Bi-LTO sample was slightly lower than that of the pristine LTO sample. The equivalent substitution of La^{3+} (with an ionic radius of 1.06 Å) by Bi^{3+} (1.03 Å) induced very limited variation on LTO lattice, which did not cause a large number of bulk phase defects. Therefore, the charge carrier concentration in bulk phase did not change a lot. However, Bi doping can ultimately reduce the carrier concentration slightly due to elimination of the trap states on LTO surface (surface defects).

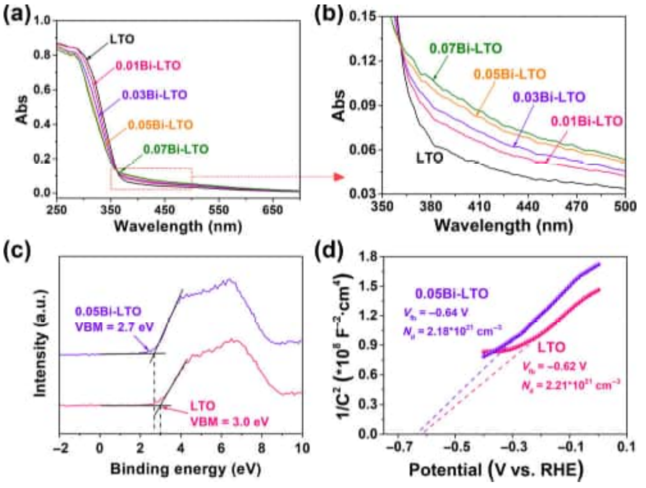

Figure 2 Optical and electronic characterization of photocatalysts: (a) UV–vis spectra, (b) enlarged UV–vis spectra in the range of 350–500 nm, (c) valence-band XPS spectra, and (d) Mott–Schottky (M–S) plot of LTO and 0.05Bi-LTO.

3.3 Photocatalytic water splitting performance

Overall water splitting testing was performed on the catalysts in deionized water in the absence of any sacrificial agent under illumination of a Xe light source (Fig. 3(a)). Typically, the doped sample used for photocatalytic tests was 0.05Bi-LTO (Bi-LTO for clarity) unless otherwise stated. The pristine LTO showed negligible photocatalytic activity toward overall water splitting even though both the Co-Pi and Pt co-catalysts were presented on the LTO surface. In contrast, overall water splitting occurred on all the Bi-doped LTO photocatalysts in the presence of co-catalyst (Figs. 3(a) and 3(b)). The amount of both hydrogen and oxygen generated showed a linear relationship with the irradiation time. After irradiation for 150 min, 66.6 μmol H_2 and 32.1 μmol O_2 were observed, showing a molar ratio of 2.07. This confirmed the overall water splitting occurred in the absence of any sacrificial agent and

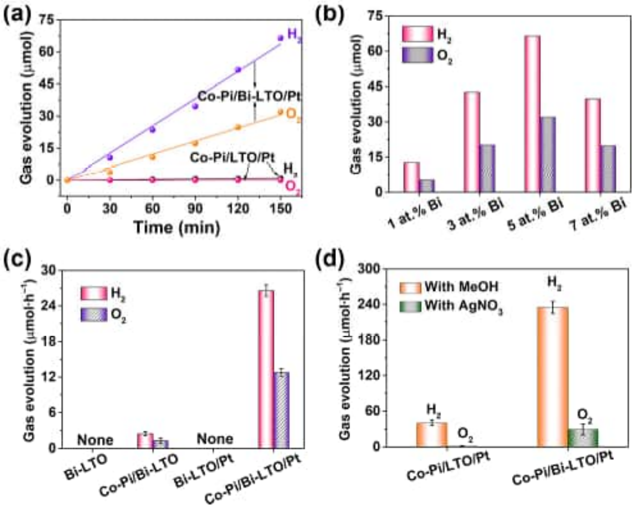

Figure 3 Photocatalytic performance of samples: (a) overall water splitting by Co-Pi/LTO/Pt and Co-Pi/Bi-LTO/Pt, (b) comparison of overall water splitting by Co-Pi/Bi-LTO/Pt with different Bi content, (c) water-splitting gas generation rate by Bi-LTO modified with different co-catalysts, and (d) H_2 or O_2 production from water with sacrificial agents by Co-Pi/LTO/Pt and Co-Pi/0.05Bi-LTO/Pt.

without any external field. In addition, both the hydrogen and oxygen generation rates increased with the Bi content increased up to 5 at.% (Fig. 3(b)). Further increasing the Bi content to 7 at.% in the LTO nanosheet reduced both the hydrogen and oxygen rates by approximately 40%, that is, 39.9 μmol H_2 and 19.8 μmol O_2 under simulated light irradiation for 150 min. The AQE for overall water splitting was evaluated to be 0.58% and 0.11% at 254 and 365 nm monochromatic light, respectively (Fig. S13 in the ESM). On the other hand, the stability of Bi-LTO photocatalyst was assessed by the cyclic water splitting experiments (Fig. S14 in the ESM). There was no obvious deactivation in the release of H_2 and O_2 after three recycles. TEM and XPS results showed that there was not any change in both the morphology and composition for the Bi-LTO photocatalyst after 7.5 h of photocatalytic water splitting testing (Figs. S15 and S16 in the ESM). After electrons and holes are photogenerated in the Bi-LTO, the electrons and holes will migrate to the Bi-LTO surface. The electrons and holes will be injected to water molecules to participate in the redox reaction, which is called "charge-injection". The co-catalyst on the Bi-LTO surface may have an influence on the charge-injection process [27]. As shown in Fig. 3(c), the Bi-doped LTO photocatalyst exhibited negligible photocatalytic activity toward water splitting when the co-catalysts were absent on the Bi-LTO surface; and neither hydrogen nor oxygen was generated when only the Pt co-catalyst was present on the Bi-LTO surface. However, both hydrogen and oxygen were generated slowly when only the Co-Pi co-catalyst was present on the Bi-LTO surface. The hole-injection process and water oxidation reaction were rate-limiting in water splitting. This agreed with the previous conclusion that water oxidation was a sluggish reaction that required a 4-electron transfer with a higher overpotential in water splitting [28]. Without assistance of the Pt co-catalyst, it is difficult for the photogenerated electrons to participate in HER due to the poor surface catalytic activity of Bi-LTO itself. In short, both the Co-Pi and Pt co-catalysts are essential for reducing the energy barrier of charge injection. In addition, the PtO act as efficient H_2 evolution sites while suppressing the undesirable hydrogen back-oxidation [22, 29, 30]. Both the loadings of Co-Pi and Pt on the Bi-LTO nanosheets were optimized, and the experiments demonstrated that 2 wt.% Co-Pi and 2 wt.% Pt were optimal (Fig. S17 in the ESM), which resulted in the H_2 and O_2 production rates of 26.7 and 12.9 μmol·h^{-1}, respectively. Although the water splitting rate of Co-Pi/Bi-LTO system (2.4 μmol·h^{-1}) was only one tenth of that of the system with both Pt and Co-Pi modification (26.7 μmol·h^{-1}), the Co-Pi alone on the Bi-LTO nanosheets facilitated the hole injection significantly.

After the photogenerated charge carriers have migrated to the surface of the photocatalyst, the charge carriers can be mobile, which favours the charge injection. However, the charge carriers can also be immobile due to capture by the surface traps, which may cut off the charge injection process. It is well known that charge-scavengers in the electrolyte solution can harness the photogenerated charge carriers on the photocatalyst surface. For the Co-Pi/LTO/Pt photocatalyst, almost no oxygen was generated when the electron-scavenger ($AgNO_3$) was present in the electrolyte solution. This indicated that the photogenerated holes were captured by the surface traps. When the hole-scavenger (methanol) was present in the electrolyte solution, hydrogen was generated at a low generation rate. This indicated that the hole-scavenger collected some of holes from the surface trap. For the Co-Pi/Bi-LTO/Pt photocatalyst, oxygen was generated when $AgNO_3$ was present in the electrolyte solution. This indicated that Bi doping passivated the photocatalyst surface, which made the photogenerated holes mobile on the Bi-LTO surface. When methanol was present in the electrolyte solution, the hydrogen generation rate increased remarkably. This indicated that many mobile holes were available on the Bi-LTO surface, and the hole-scavenger can collect these mobile holes easily from the passivated surface. The data in Fig. 3(d) also confirm that in the overall water splitting process, the water oxidation reaction was the rate-liming process rather than the water reduction reaction. In addition, the results in Fig. 3(d) also suggest that the Co-Pi co-catalyst cannot suppress the surface hole trapping although it can make the mobile holes participate in the water oxidation reaction.

3.4 SPV analysis

SPV, which was measured as a function of the wavelength of modulated incident light, can derive the photo-induced carrier separation and transfer behaviours in the photocatalyst [6, 31, 32]. As shown in Fig. 4(a), the SPV amplitude of pristine LTO sample showed a maximum value at 330 nm, which indicated that the vast majority of photogenerated charge carriers came from the inter-band transition (the valence band to the conduction band). The surface charge was the net value of the in-phase signal (X) and the out-of-phase signal (Y), which represented the instantaneous surface charges and the delayed surface charges diffusing from the inside to the surface, respectively. The positive X signal indicated the n-type semi-conductor behaviour for the pristine LTO. The positive Y signal implied the relatively slow migration of photogenerated holes from the inside to the surface. The SPV signals in the spectral range of 400–480 nm were attributed to the excitation of the surface states on LTO (Fig. S18 in the ESM). In contrast, there were not any excited SPV signals above 400 nm (Fig. 4(b)), which indicated the surface states were eliminated due to the surface passivation by the Bi-dopant (Fig. S19 in the ESM) [33]. Doping of 5.0 at.% Bi into LTO increased the peak value of total SPV amplitude by 130%, which indicated the significant enhancement of charge separation from the inter-band transition. The Y signal was relatively stronger than the X signal in the 0.05Bi-LTO, which suggested more holes contributed to the out-of-phase surface charge due to the surface passivation. Further increasing the Bi dopant level to 7.0 at.% reduced the peak value of total SPV amplitude (Fig. 4(d)). There were Y signals in the spectral range of 380–460 nm (Fig. 4(c)), ascribed to the excitation from the localized states above the valence band (Fig. S20 in the ESM), as shown in the simulation results in the next section, as well as the absorption tails in Fig. 2(b). It is worth noting that the Y signal became negative, which

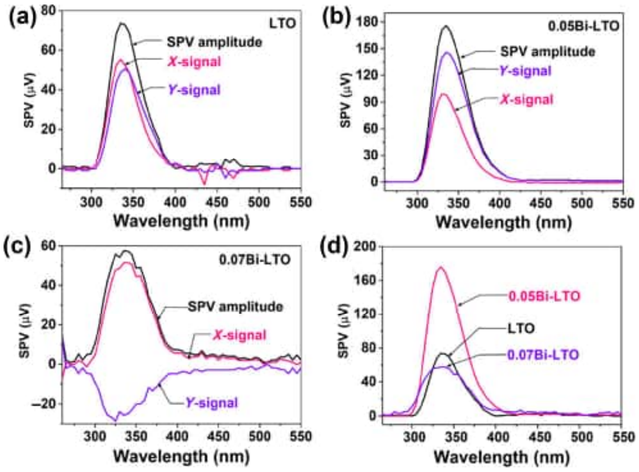

Figure 4 SPV spectra of (a) pristine LTO, (b) 0.05Bi-LTO, and (c) 0.07Bi-LTO. (d) Comparison of SPV amplitude with the pristine LTO, 0.05Bi-LTO, and 0.07Bi-LTO.

indicated that the out-of-phase surface charge was dominated by the photogenerated electrons; and less holes migrated out to the photocatalyst surface.

3.5 TDR measurement

Femtosecond TDR spectra were recorded at different time delays from the pristine, 5 at.% and 7 at.% Bi-doped LTO samples, allowing for direct observation of the time-scale of charge dynamics and carrier population [34–36]. Under excitation of the 380 nm pump pulse laser, electrons transited from the O 2p orbital in the valence band to the Ti 3d orbital in the conduction band, which resulted in the broad transient absorption bands at 800–1,150 nm (Figs. 5(a)–5(c)). The transient absorption of all samples reached the highest immediately after laser flash (1 ps) and then decayed gradually. During 1–1,000 ps after laser flash, the electron population decreased in the multi-exponential decay due to recombination of trapped electrons with holes [37]. Using three-exponential functions, the time profiles of the transient absorption probed at 850 nm were fitted (Fig. 5(d)), giving their lifetime (τ_i) and amplitude (a_i) in Table 1. The electron recovery kinetics was fitted to tri-exponential functions with three-time constants for all the samples, and each time scale represented an electron decay process. The shortest component with lifetime of only several picoseconds (τ_1) was mainly ascribed to direct recombination of electrons and holes, namely, the fast electron relaxation within sub-surface of LTO. In this process, the excited electrons in all the samples were returned to the ground state with an approximate time constant of 4 ps because Bi doping did not significantly change the intrinsic band structure of the photocatalyst. The second component (τ_2) in dozens of picoseconds was attributed to the indirect recombination (also named Shockley–Read–Hall (SRH) recombination) process, in which the trapped electrons recombined with holes in virtue of recombination centres with the deep bound levels, such as impurity and defects in the sub-surface [38]. The duration of this process depended on the number and position of the trap states in the bulk. Being restricted by the bound state levels, 38% of trapped electrons in 0.05Bi-LTO (or 39% of trapped electrons in pristine LTO) rapidly recombined with the holes in the internal recombination centers in 70–80 ps. However, it took only 47 ps for 44% of the trapped electrons in 0.07Bi-LTO to be recovered. For the 7 at.% Bi-doped sample, a large number of photogenerated electrons and holes were consumed in the sub-surface before moving to the surface, because the continued, excessive Bi doping gradually deepened sub-band energy levels into the sub-surface, amplifying the role of localized states as the recombination centres. The long lifetime component (τ_3) was due to the surface charge recombination. These long-lived charge carriers are viewed as the primary contributors to the photochemical reaction on the semiconductor surface [39]. In other words, the larger the τ_3, the lower the probability for electron–hole recombination. Therefore, the τ_3 of 0.05Bi-LTO (1,400 ps), which was much longer than that of pristine LTO (950 ps) and 0.07Bi-LTO (1,000 ps), clearly demonstrated a more efficient charge carrier separation process. Moreover, as compared with LTO and 0.07Bi-LTO, the 5 at.% Bi-doped sample also exhibited an increase in the a_3 value, showing a larger population of charge carriers moving to the surface.

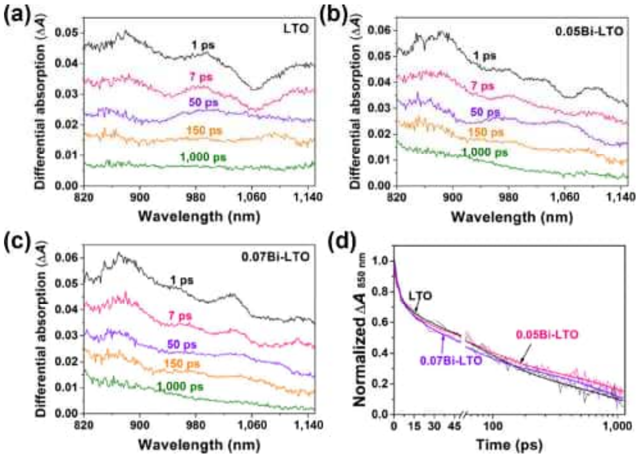

Figure 5 TDR spectra of (a) pristine LTO, (b) 0.05Bi-LTO, and (c) 0.07Bi-LTO under 380 nm laser excitation. (d) Normalized transient absorption traces observed at 850 nm. The solid lines indicate multi-exponential curves fitted to kinetic traces.

Table 1 Lifetime (τ_i) and amplitudes (a_i) of the transient absorption decays of the pristine, 5 at.% and 7 at.% Bi-doped LTO.

Sample	τ_1 (a_1)/ps	τ_2 (a_2)/ps	τ_3 (a_3)/ps
LTO	3.8 ± 0.7 (28%)	74 ± 8 (39%)	950 ± 90 (33%)
0.05Bi-LTO	4.6 ± 0.9 (26%)	70 ± 7 (38%)	1,400 ± 100 (36%)
0.07Bi-LTO	4.0 ± 1 (27%)	47 ± 6 (44%)	1,010 ± 60 (29%)

4 Discussion

As described above, the photocatalysis data in Fig. 3 and the SPV spectra indicate that surface trap states of holes exist on the pristine LTO. Accordingly, the band structure and charge processes (charge separation, migration, trapping, and injection) are proposed in Fig. 6(a). For pristine LTO, the photogenerated holes are captured and immobile on the surface traps, and thus cannot be injected to water molecules in pure water without any assistance of charge scavengers. In this case, the photogenerated electrons could rapidly recombine with holes. To examine the nature of surface trap, we characterized the pristine LTO and the 5 at.% Bi-doped LTO sample with the electron paramagnetic resonance (EPR) and the result are shown in Fig. S21 in the ESM. The g-factor at $g = 2.004$ can be assigned to a hole trap on the surface, which often occurs in metal oxides [40–43]. This value falls into the typical range (2.003 to 2.050) governed by a model of a hole trapped in a non-bonding p orbital on an oxygen ion [40–43]. After Bi doping, the intensity of EPR derivative peak is reduced significantly. This indicated that the hole trap can be stabilized by the La-vacancies on the LTO surface. It is believed that Bi dopant entered the substitutional site of La ions and filled the surface La-vacancies, which removed the surface hole traps.

The production rates of superoxide radicals ($O_2^{\bullet-}$) and hydroxyl radicals (•OH), which come from the photogenerated electrons and holes, respectively, affirm that Bi doping removes hole trap states rather than defects states closed to the conduction band. Compared to pristine LTO, Bi doping increases the production rates of $O_2^{\bullet-}$ and •OH by 1.5 and 5.6 times, respectively (Fig. S22 in the ESM). This indicates that the hole trap on LTO surface reduces the •OH production dramatically. It is further confirmed that the hole trap states increase the charge recombination of the excited holes but keep the conduction band structure and electron kinetics unchanged.

For the Bi-doped LTO samples, Bi doping does not result in any evident change in the band gap, charge carrier concentration, and the flat band potential (Fig. 2). However, Bi doping passivates the nanosheet surface and eliminates the surface traps. This conclusion is supported by several consistent experimental results: (i) The Co-Pi/LTO/Pt sample shows negligible photocatalytic activity while the Co-Pi/Bi-LTO/Pt exhibits strong photocatalytic activity, leading to overall water

splitting in pure water (Fig. 3(a)); (ii) in the presence of electron scavenger in water, water oxidation occurs for Bi-doped LTO but not for pure LTO (Fig. 3(d)); (iii) the SPV charge signal in the 400–480 nm range disappears after Bi doping (Fig. 4); (iv) as shown in Table 1, the τ_3 of 0.05Bi-LTO is much longer than that of pristine LTO, but there is no evident difference in both τ_1 and τ_2 between 0.05Bi-LTO and pristine LTO. In addition, it is observed that Bi is enriched on the nanosheet surface. To prove this, XPS spectra were acquired after the nanosheet samples were etched by argon etching in the XPS instrument chamber. Figure S23 in the ESM shows that the Bi/(Bi + La) content ratio decreases with an increase in the etching time. Hence the surface enrichment of Bi dopant is responsbile for surface passivation of nanosheet photocatalysts.

When the Bi dopant level increases to 7 at.%, the photocatlaytic activity of Bi-doped LTO nanosheet diminishes. This motivates us to examine the electronic band structures of pristine and Bi-doped LTO with the DFT computation (see details in Figs. S24 and S25 in the ESM). Figures S26 and S27 in the ESM show the density of states (DOS) and band structures of Bi-LTO with different Bi/(La + Bi) ratios. The valence band (VB) and conduction band (CB) of pristine LTO are mainly composed of the O 2p and Ti 3d states (Fig. S26(a) in the ESM). The indirect band gap of LTO is calculated to be 2.87 eV (Fig. S27(a) in the ESM), underestimating the experimental value due to the well-known limitations in conventional DFT [44]. A low level of Bi (6.25 at.%) doping has little influence on the DOS and band structure of LTO (Figs. S26(b) and S27(b) in the ESM), while a high level of doping (12.5 at.%) introduces the shallow localized states above the VB maximum (Figs. S26(c) and S27(c) in the ESM). The shallow level, as a filled state under the ground state below the Femi level, is mainly occupied by the O 2p electrons. As pointed in ref. [3], excitation from the localized states can induce light absorption in the visible-light range. However, in the excited localized states, the photogenerated holes in the localized states are immobile, which are barely involved into the photochemical reactions. These localized states serve as the charge recombination centers, which shorten the lifetime of photogenerated charge carriers, as shown by τ_3 in Table 1.

In this work, deposition of Co-Pi cannot remove the surface trap states. It is not surprising that the Co-Pi/LTO/Pt sample does not show evident photocatalytic activity toward water splitting in pure water (Fig. 3(a)), and it does not make water oxidation take place even when the electron scavenger is present in water. However, the co-catalyst is essential for water splitting (Fig. 3(c)). The co-catalyst can reduce the energy barrier of charge injection.

Keeping all the above points in mind, the photocatalysis mechanisms of all the photocatalysts can be understandable (Fig. 6). For the pristine LTO (Fig. 6(a)), the photogenerated holes are captured in the surface traps on the pristine nanosheets. For Co-Pi/LTO/Pt (Fig. 6(b)), the trapped holes are unable to transfer to the Co-Pi co-catalyst. Consequently, Co-Pi/LTO/Pt does not work for water splitting.

For 0.05Bi-LTO (Fig. 6(c)), the surface hole traps have been eliminated by the presence of Bi dopant in the nanosheet. The photogenerated holes are mobile in the valence band on the photocatalyst surface. However, even though holes are present on the surface, they cannot be injected to water molecules due to high energy barrier. For Co-Pi/0.05Bi-LTO/Pt (Fig. 6(d)), the photogenerated holes are transferred to the Co-Pi co-catalyst, and then injected to water molecules. In the meanwhile, photogenerated electrons drive the HER with assistance of the Pt co-catalyst. As a result, Co-Pi/0.05Bi-LTO/Pt exhibits photocatalytic activity toward overall water splitting.

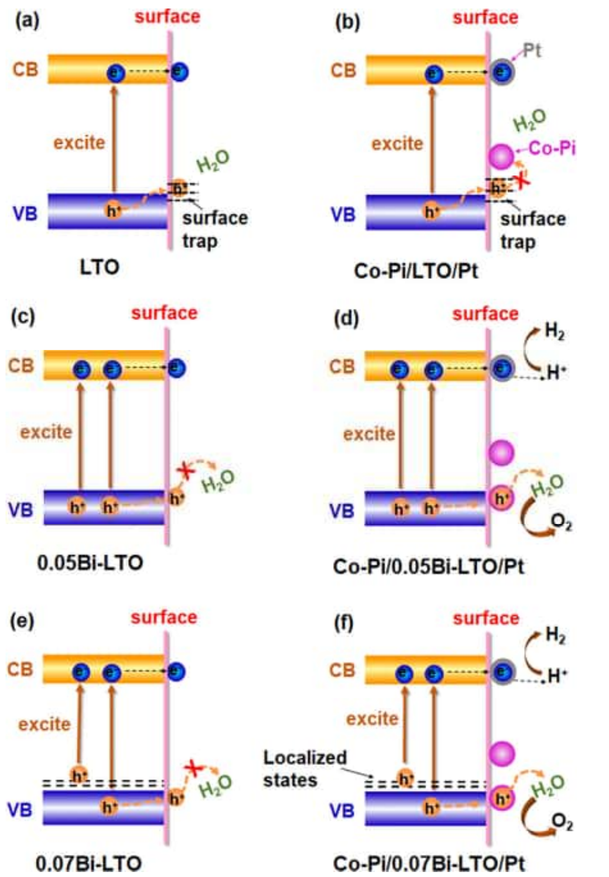

Figure 6 Schematic illustration of the proposed band structure and photocatalysis mechanism for water splitting: (a, b) LTO, (c, d) 0.05Bi-LTO, and (e, f) 0.07Bi-LTO, showing the effects of the Bi and co-catalysts.

For 0.07Bi-LTO (Fig. 6(e)), there is no surface traps. However, there exist the localized states. Some of the electrons are excited from the valence band, and other electrons from the localized states. The photogenerated holes in the valence band are mobile on the photocatalyst surface, but cannot be injected to water molecules without the assistance of Co-Pi co-catalyst. For Co-Pi/0.07Bi-LTO/Pt (Fig. 6(f)), the photogenerated holes in the localized states are immobile and cannot participate in the water oxidation reaction. The localized states serve as the charge recombination centers. Hence the Co-Pi/0.07Bi-LTO/Pt shows photocatalytic activity inferior to Co-Pi/0.05Bi-LTO/Pt.

5 Conclusions

In summary, the pristine LTO and Bi-doped LTO nanosheets were successfully synthesized by hydrothermal processing. The Bi-doped LTO nanosheets exhibited negligible photocatalytic activity in the absence of co-catalysts. Hence, the Co-Pi and Pt nanoparticles were then deposited on the same LTO nanosheets as the co-catalysts for water oxidation reaction and HER, respectively. The Co-Pi/pristine LTO/Pt sample in pure water showed negligible photocatalytic activity. Bi doping into LTO enabled the Co-Pi/Bi-LTO/Pt samples in pure water to exhibit significant photocatalytic activity toward overall water splitting. The optimal photocatalytic activity was achieved at a dopant level of 5 at.%. Too high levels of Bi doping induce the localized states in LTO, which increases the charge recombination, leading to a lower photocatalytic activity.

Surface traps of holes are found on the surface of pristine LTO nanosheets, disabling the photocatalytic activity toward water splitting. Deposition of the co-catalyst on the LTO nanosheets is unable to suppress the surface traps. In contrast,

Bi doping into the LTO nanosheets removes the surface traps from the LTO nanosheet due to the surface enrichment of Bi. In short, surface trapping and surface catalysis are two important factors that need to be considered in design of $La_2Ti_2O_7$ photocatalysts.

Acknowledgements

This project is partially supported by the National Natural Science Foundation of China (Nos. 51972010 and 51472013), the Natural Science Foundation of Jiangsu Province (Youth Fund, Nos. BK20190640 and BK20190641), and the Fundamental Research Funds for the Central Universities (No. 2019XKQYMS11). We thank the Open Sharing Fund for the large-scale instruments and equipment of China University of Mining and Technology (CUMT), and the high-performance computing platform of Beihang University.

Electronic Supplementary Material: Supplementary material (experimental methods, computational details, element content; SEM, AFM, lattice parameter and surface area, STEM-EDS, EDS, TEM, HRTEM, XPS, Tauc plots, AQE, photocatalytic water splitting, band diagram, EPR, reactive oxygen species, crystal structure, total energy, DOS, and band structure) is available in the online version of this article at http://doi.org/10.1007/s12274-021-3498-5.

References

[1] Li, J. T.; Wu, N. Q. Semiconductor-based photocatalysts and photoelectrochemical cells for solar fuel generation: A review. *Catal. Sci. Technol.* **2015**, *5*, 1360–1384.

[2] Tofanello, A.; Shen, S. H.; de Souza, F. L.; Vayssieres, L. Strategies to improve the photoelectrochemical performance of hematite nanorod-based photoanodes. *APL Mater.* **2020**, *8*, 040905.

[3] Wang, J.; Tafen, D. N.; Lewis, J. P.; Hong, Z. L.; Manivannan, A.; Zhi, M. J.; Li, M.; Wu, N. Q. Origin of photocatalytic activity of nitrogen-doped TiO_2 nanobelts. *J. Am. Chem. Soc.* **2009**, *131*, 12290–12297.

[4] Tang, P. Y.; Arbiol, J. Engineering surface states of hematite based photoanodes for boosting photoelectrochemical water splitting. *Nanoscale Horiz.* **2019**, *4*, 1256–1276.

[5] Zachäus, C.; Abdi, F. F.; Peter, L. M.; Van De Krol, R. Photocurrent of $BiVO_4$ is limited by surface recombination, not surface catalysis. *Chem. Sci.* **2017**, *8*, 3712–3719.

[6] Yang, H.; Bright, J.; Kasani, S.; Zheng, P.; Musho, T.; Chen, B. L.; Huang, L.; Wu, N. Q. Metal–organic framework coated titanium dioxide nanorod array p–n heterojunction photoanode for solar water-splitting. *Nano Res.* **2019**, *12*, 643–650.

[7] Meng, F. K.; Hong, Z. L.; Arndt, J.; Li, M.; Zhi, M. J.; Yang, F.; Wu, N. Q. Visible light photocatalytic activity of nitrogen-doped $La_2Ti_2O_7$ nanosheets originating from band gap narrowing. *Nano Res.* **2012**, *5*, 213–221.

[8] Zhang, J. Y.; Dang, W. Q.; Ao, Z. M.; Cushing, S. K.; Wu, N. Q. Band gap narrowing in nitrogen-doped $La_2Ti_2O_7$ predicted by density-functional theory calculations. *Phys. Chem. Chem. Phys.* **2015**, *17*, 8994–9000.

[9] Kasahara, A.; Nukumizu, K.; Hitoki, G.; Takata, T.; Kondo, J. N.; Hara, M.; Kobayashi, H.; Domen, K. Photoreactions on $LaTiO_2N$ under visible light irradiation. *J. Phys. Chem. A* **2002**, *106*, 6750–6753.

[10] Shirai, K.; Sugimoto, T.; Watanabe, K.; Haruta, M.; Kurata, H.; Matsumoto, Y. Effect of water adsorption on carrier trapping dynamics at the surface of anatase TiO_2 nanoparticles. *Nano Lett.* **2016**, *16*, 1323–1327.

[11] Hurum, D. C.; Gray, K. A.; Rajh, T.; Thurnauer, M. C. Recombination pathways in the degussa P25 formulation of TiO_2: Surface versus lattice mechanisms. *J. Phys. Chem. B* **2005**, *109*, 977–980.

[12] Carey, J. J.; McKenna, K. P. Screening doping strategies to mitigate electron trapping at anatase TiO_2 surfaces. *J. Phys. Chem. C* **2019**, *123*, 22358–22367.

[13] Chen, H. D.; Zhang, F.; Zhang, W. F.; Du, Y. G.; Li, G. Q. Negative impact of surface Ti^{3+} defects on the photocatalytic hydrogen evolution activity of $SrTiO_3$. *Appl. Phys. Lett.* **2018**, *112*, 013901.

[14] Ji, Q. Q.; Bi, L.; Zhang, J. T.; Cao, H. J.; Zhao, X. S. The role of oxygen vacancies of ABO_3 perovskite oxides in the oxygen reduction reaction. *Energy Environ. Sci.* **2020**, *13*, 1408–1428.

[15] Trześniewski, B. J.; Digdaya, I. A.; Nagaki, T.; Ravishankar, S.; Herraiz-Cardona, I.; Vermaas, D. A.; Longo, A.; Gimenez, S.; Smith, W. A. Near-complete suppression of surface losses and total internal quantum efficiency in $BiVO_4$ photoanodes. *Energy Environ. Sci.* **2017**, *10*, 1517–1529.

[16] Shi, Q.; Murcia-López, S.; Tang, P. Y.; Flox, C.; Morante, J. R.; Bian, Z. Y.; Wang, H.; Andreu, T. Role of tungsten doping on the surface states in $BiVO_4$ photoanodes for water oxidation: Tuning the electron trapping process. *ACS Catal.* **2018**, *8*, 3331–3342.

[17] Yatom, N.; Neufeld, O.; Caspary Toroker, M. Toward settling the debate on the role of Fe_2O_3 surface states for water splitting. *J. Phys. Chem. C* **2015**, *119*, 24789–24795.

[18] Barroso, M.; Mesa, C. A.; Pendlebury, S. R.; Cowan, A. J.; Hisatomi, T.; Sivula, K.; Grätzel, M.; Klug, D. R.; Durrant, J. R. Dynamics of photogenerated holes in surface modified α-Fe_2O_3 photoanodes for solar water splitting. *Proc. Natl. Acad. Sci. USA* **2012**, *109*, 15640–15645.

[19] Kim, W. D.; Kim, J. H.; Lee, S.; Lee, S.; Woo, J. Y.; Lee, K.; Chae, W. S.; Jeong, S.; Bae, W. K.; McGuire, J. A. Role of surface states in photocatalysis: Study of chlorine-passivated CdSe nanocrystals for photocatalytic hydrogen generation. *Chem. Mater.* **2016**, *28*, 962–968.

[20] Cai, X. Y.; Zhu, M. S.; Elbanna, O. A.; Fujitsuka, M.; Kim, S.; Mao, L.; Zhang, J. Y.; Majima, T. Au nanorod photosensitized $La_2Ti_2O_7$ nanosteps: Successive surface heterojunctions boosting visible to near-infrared photocatalytic H_2 evolution. *ACS Catal.* **2018**, *8*, 122–131.

[21] Tan, H. L.; Wen, X. M.; Amal, R.; Ng, Y. H. $BiVO_4$ {010} and {110} relative exposure extent: Governing factor of surface charge population and photocatalytic activity. *J. Phys. Chem. Lett.* **2016**, *7*, 1400–1405.

[22] Li, Y. H.; Xing, J.; Chen, Z. J.; Li, Z.; Tian, F.; Zheng, L. R.; Wang, H. F.; Hu, P.; Zhao, H. J.; Yang, H. G. Unidirectional suppression of hydrogen oxidation on oxidized platinum clusters. *Nat. Commun.* **2013**, *4*, 2500.

[23] Mao, L.; Cai, X. Y.; Gao, H.; Diao, X. G.; Zhang, J. Y. A newly designed porous oxynitride photoanode with enhanced charge carrier mobility. *Nano Energy* **2017**, *39*, 172–182.

[24] Pussacq, T.; Kabbour, H.; Colis, S.; Vezin, H.; Saitzek, S.; Gardoll, O.; Tassel, C.; Kageyama, H.; Laberty Robert, C.; Mentré, O. Reduction of $Ln_2Ti_2O_7$ layered perovskites: A survey of the anionic lattice, electronic features, and potentials. *Chem. Mater.* **2017**, *29*, 1047–1057.

[25] He, R. A.; Xu, D. F.; Cheng, B.; Yu, J. G.; Ho, W. Review on nanoscale Bi-based photocatalysts. *Nanoscale Horiz.* **2018**, *3*, 464–504.

[26] Li, J.; Cai, L. J.; Shang, J.; Yu, Y.; Zhang, L. Z. Giant enhancement of internal electric field boosting bulk charge separation for photocatalysis. *Adv. Mater.* **2016**, *28*, 4059–4064.

[27] Pan, Z. H.; Yanagi, R.; Wang, Q.; Shen, X.; Zhu, Q. H.; Xue, Y. D.; Röhr, J. A.; Hisatomi, T.; Domen, K.; Hu, S. Mutually-dependent kinetics and energetics of photocatalyst/co-catalyst/two-redox liquid junctions. *Energy Environ. Sci.* **2020**, *13*, 162–173.

[28] Wolff, C. M.; Frischmann, P. D.; Schulze, M.; Bohn, B. J.; Wein, R.; Livadas, P.; Carlson, M. T.; Jäckel, F.; Feldmann, J.; Würthner, F. et al. All-in-one visible-light-driven water splitting by combining nanoparticulate and molecular co-catalysts on CdS nanorods. *Nat. Energy* **2018**, *3*, 862–869.

[29] Ren, X. N.; Hu, Z. Y.; Jin, J.; Wu, L.; Wang, C.; Liu, J.; Liu, F.; Wu, M.; Li, Y.; van Tendeloo, G. et al. Cocatalyzing Pt/PtO phase-junction nanodots on hierarchically porous TiO_2 for highly enhanced photocatalytic hydrogen production. *ACS Appl. Mater. Interfaces* **2017**, *9*, 29687–29698.

[30] Zhang, G. G.; Lan, Z. A.; Lin, L. H.; Lin, S.; Wang, X. C. Overall water splitting by Pt/g-C_3N_4 photocatalysts without using sacrificial agents. *Chem. Sci.* **2016**, *7*, 3062–3066.

[31] Park, H.; Kim, H. I.; Moon, G. H.; Choi, W. Photoinduced charge transfer processes in solar photocatalysis based on modified TiO_2. *Energy Environ. Sci.* **2016**, *9*, 411–433.

[32] Li, Z.; Zhang, L.; Liu, Y.; Shao, C. Y.; Gao, Y. Y.; Fan, F. T.; Wang, J. X.; Li, J. M.; Yan, J. C.; Li, R. G. et al. Surface-polarity-induced spatial charge separation boosts photocatalytic overall water splitting on GaN nanorod arrays. *Angew. Chem., Int. Ed.* **2020**, *132*, 945–952.
[33] Li, S.; Hou, L. B.; Zhang, L. J.; Chen, L. P.; Lin, Y. H.; Wang, D. J.; Xie, T. F. Direct evidence of the efficient hole collection process of the CoO_x cocatalyst for photocatalytic reactions: A surface photovoltage study. *J. Mater. Chem. A* **2015**, *3*, 17820–17826.
[34] Li, J. T.; Cushing, S. K.; Zheng, P.; Senty, T.; Meng, F. K.; Bristow, A. D.; Manivannan, A.; Wu, N. Q. Solar hydrogen generation by a CdS-Au-TiO_2 sandwich nanorod array enhanced with Au nanoparticle as electron relay and plasmonic photosensitizer. *J. Am. Chem. Soc.* **2014**, *136*, 8438–8449.
[35] Yu, H. J.; Li, J. Y.; Zhang, Y. H.; Yang, S. Q.; Han, K. L.; Dong, F.; Ma, T. Y.; Huang, H. W. Three-in-one oxygen vacancies: Whole visible-spectrum absorption, efficient charge separation, and surface site activation for robust CO_2 photoreduction. *Angew. Chem., Int. Ed.* **2019**, *58*, 3880–3884.
[36] Mao, L.; Cai, X. Y.; Yang, S. Q.; Han, K. L.; Zhang, J. Y. Black phosphorus-CdS-$La_2Ti_2O_7$ ternary composite: Effective noble metal-free photocatalyst for full solar spectrum activated H_2 production. *Appl. Catal. B: Environ.* **2019**, *242*, 441–448.
[37] Han, K.; Kreuger, T.; Mei, B.; Mul, G. Transient behavior of Ni@NiO_x functionalized $SrTiO_3$ in overall water splitting. *ACS Catal.* **2017**, *7*, 1610–1614.
[38] Murthy, D. H. K.; Matsuzaki, H.; Wang, Z.; Suzuki, Y.; Hisatomi, T.; Seki, K.; Inoue, Y.; Domen, K.; Furube, A. Origin of the overall water splitting activity of Ta_3N_5 revealed by ultrafast transient absorption spectroscopy. *Chem. Sci.* **2019**, *10*, 5353–5362.
[39] Cai, X. Y.; Mao, L.; Yang, S. Q.; Han, K. L.; Zhang, J. Y. Ultrafast charge separation for full solar spectrum-activated photocatalytic H_2 generation in a black phosphorus-Au-CdS heterostructure. *ACS Energy Lett.* **2018**, *3*, 932–939.
[40] Swinney, M. W.; McClory, J. W.; Petrosky, J. C.; Yang, S.; Brant, A. T.; Adamiv, V. T.; Burak, Y. V.; Dowben, P. A.; Halliburton, L. E. Identification of electron and hole traps in lithium tetraborate ($Li_2B_4O_7$) crystals: Oxygen vacancies and lithium vacancies. *J. Appl. Phys.* **2010**, *107*, 113715.
[41] Walsby, C. J.; Lees, N. S.; Tennant, W. C.; Claridge, R. F. C. 15 K EPR of an oxygen-hole boron centre, $[BO_4]^0$, in *x*-irradiated zircon. *J. Phys.: Condens. Matter* **2000**, *12*, 1441.
[42] Chen, Y.; Abraham, M. M. Trapped-hole centers in alkaline-earth oxides. *J. Phys. Chem. Solids* **1990**, *51*, 747–764.
[43] Nuttall, R. H. D.; Weil, J. A. The magnetic properties of the oxygen-hole aluminum centers in crystalline SiO_2. I. $[AlO]^0$. *Can. J. Phys.* **1981**, *59*, 1696–1708.
[44] Jones, R. O.; Gunnarsson, O. The density functional formalism, its applications and prospects. *Rev. Mod. Phys.* **1989**, *61*, 689–746.

Journal of the European Ceramic Society 37 (2017) 137–143

Contents lists available at www.sciencedirect.com

Journal of the European Ceramic Society

journal homepage: www.elsevier.com/locate/jeurceramsoc

The anisotropic conductivity of ferroelectric $La_2Ti_2O_7$ ceramics

Zhipeng Gao [a,*], Lingfeng Wu [a,b], Chengjia Lu [a], Wei Gu [a], Tao Zhang [a], Gaomin Liu [a], Qinghai Xie [a], Ming Li [c,*]

[a] National Key Laboratory of Shock Wave and Detonation Physics, Institute of Fluid Physics, China Academy of Engineering Physics, Mianyang 621900, China
[b] Electronic Materials Research Laboratory, Xi'an Jiaotong University, Xi'an 710049, China
[c] Advanced Materials Research Group, Department of Mechanical, Materials and Manufacturing Engineering, University of Nottingham, University Park, Nottingham NG7 2RD, United Kingdom

ARTICLE INFO

Article history:
Received 28 May 2016
Received in revised form 6 August 2016
Accepted 16 August 2016
Available online 21 August 2016

Keywords:
Ferroelectrics
Piezoelectric
PLS ceramic
Conductivity
Impedance spectroscopy

ABSTRACT

Ferroelectric ceramics with perovskite-like layered structure (PLS) have good potential for high temperature piezoelectric applications due to their high Curie point (T_c). The electrical conduction behaviour of these materials is a critical parameter to consider for practical applications. In this study, we prepared textured ceramics of the typical PLS ferroelectric $La_2Ti_2O_7$ using spark plasma sintering and investigated the electrical properties using impedance spectroscopy and Seebeck measurements. The results reveal that the bulk resistivity along the parallel direction is much higher than that along the perpendicular direction. The activation energy for conduction (E_a) along the parallel direction is 1.45 eV, which is close to half of the optical band gap and much higher than the 0.67 eV along the perpendicular direction. Electrical conduction along both the directions is dominated by p-type hole conduction. No appreciable contribution of oxide ion conduction to the measured conductivity is observed.

© 2016 Elsevier Ltd. All rights reserved.

1. Introduction

Piezoelectric materials play important roles in the fields of actuators, sensors, and transducers due to the ability to convert between electrical and mechanical energy [1,2]. High temperature piezoelectric sensors are seriously required in many industries such as automotive, electronics, aerospace, material processing, and power generating industries [2,3]. For example, nuclear reactors need to be monitored during operation, and the typical environment temperature can be above 1000 °C [2,3]. Aerospace engines have working temperatures higher than 900 °C [2,3], which requires a high temperature sensor to monitor the operating situation. It is therefore of great significance to develop high temperature piezoelectric sensors to meet stringent requirements for high temperature applications. In the past several decades, researches of high-temperature piezoelectrics or ferroelectrics have been focused on a range of single crystals based on $LiNbO_3$, $GaPO_4$, langasites with general formula, $Ca_3TaAl_3Si_2O_{14}$, and oxyborate $[ReCa_4O(BO_3)]$ (Re is rare earth element) [4–7]. However, the high cost, high electrical conductivity and poor mechanical properties of the single crystals limit their applications. None of the materials mentioned above has the potential to work above 900 °C.

Ferroelectric ceramics with a perovskite-like layered structure (PLS) have caught attentions due to their low cost, high Curie points, and acceptable piezoelectric constant (d_{33}) [8–11]. The PLS structure with the general formula of $A_nB_nO_{3n+2}$ (Fig. 1) [12,13] is characterized by corner-shared BO_6 octahedron and 12-coordinated A cations within the perovskite-like layers separated by oxygen-rich gaps, which are linked by A cations at their boundaries, where n is the number of octahedral layers in the perovskite layers [12,13]. When n is 4, the formula is $RE_2Ti_2O_7$ (RE: La, Ce, Pr, and Nd). Some of these compounds are the best known candidates for high-temperature piezoelectrics because of their super high Curie points and good thermal stability, such as $Sr_2Nb_2O_7$ ($T_c \sim 1330$ °C) [10], $Pr_2Ti_2O_7$ ($T_c \sim 1750$ °C) [9], and $Nd_2Ti_2O_7$ ($T_c \sim 1480$ °C) [11].

Among all these PLS ferroelectrics, $La_2Ti_2O_7$ is a well-known high temperature, layered perovskite, ferroelectric compound ($T_c \sim 1461$ °C) with low crystal symmetry (monoclinic structure with the space group $P2_1$) [11,14]. The piezoelectric properties ($d_{33} \sim 16$ pC/N) and relative permittivity (ε_{11}, ε_{22}, ε_{33} = 42, 52, 62 at 1 kHz) of $La_2Ti_2O_7$ single crystals were reported in the 1970s [15,16]. The phase equilibria in the La_2O_3-TiO_2 system were reported in 1950–1970s [17–19] and were re-examined in 2000 [20]. The dielectric properties of $La_2Ti_2O_7$ ceramics prepared by

* Corresponding authors.
E-mail addresses: z.p.gao@foxmail.com (Z. Gao), ming.li@nottingham.ac.uk (M. Li).

http://dx.doi.org/10.1016/j.jeurceramsoc.2016.08.020
0955-2219/© 2016 Elsevier Ltd. All rights reserved.

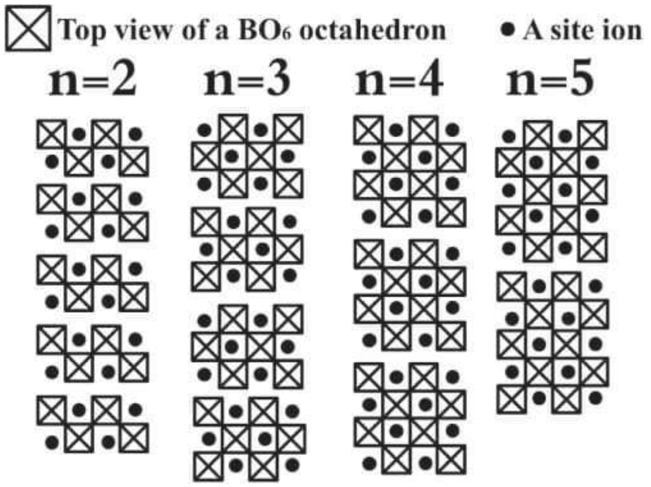

Fig. 1. A general schematic structure of the PLS materials. When n = 4, the formula of the material is $A_2B_2O_7$. [12,13].

different methods were reported in the 1990s [21–23]. The room temperature relative permittivity at 100 kHz is in the range of 43–54, depending on sample preparation methods [23]. However, the ferroelectric and piezoelectric properties of $La_2Ti_2O_7$ ceramics have not been reported until late 2000s, presumably due to that the electrical poling of the ceramics is very difficult. In 2009, the $La_2Ti_2O_7$ textured ceramic with active piezoelectric properties (d_{33} ~ 2.6 pC/N) were fabricated by Yan et al. [11] using a two-step method by spark plasma sintering technique (SPS). Then the band structure and electronic structure were studied [24,25]. In 2012, the $La_2Ti_2O_7$ film was prepared on $SrTiO_3$ substrate by Shao et al. [26] and its ferroelectric properties was studied using piezoresponse force microscopy. In 2015, Gao *et al.* [27] prepared the 3-dimentional textured $La_2Ti_2O_7$ ceramics, by combined magnetic field alignment and templated grain growth. The relative permittivity of the single crystal-like ceramic was measured as ε_{11}, ε_{22}, ε_{33} = 53, 58, 66 at 1 kHz.

Apart from ferroelectric/piezoelectric properties, electrical properties are also of great importance for practical piezoelectric applications. The high operating temperatures for these materials may lead to high leakage current and induce high current loss, which will seriously decrease the signal-to-noise ratio. Understanding the electrical conduction mechanisms in these ultra-high temperature piezoelectric materials will help to design nominal compositions (e.g., choosing suitable chemical dopants) and optimise processing conditions (e.g., sintering temperature, atmosphere) to achieve low leakage current as well as desired piezoelectric properties.

To date there has been limited studies on the electrical properties of the PLS piezoelectric materials. A very recent study reported the temperature dependence of the dc resistivity of Ta-doped $La_2Ti_2O_7$ ($La_2Ti_{2-x}Ta_xO_7$) ceramics [28]. The composition with x = 0.02 was shown to exhibit a high dc resistivity of about 10 MΩ.cm at 700 °C. For undoped $La_2Ti_2O_7$ ceramics, the dc resistivity at 700 °C measured in air using a dense ceramic is in the order of 400 kΩ cm [15]. The conductivity at 700 °C decreases with decreasing pO_2 (p-type behaviour) in the pO_2 range of air and 1 Pa. The conductivity then exhibits little pO_2 dependence (ionic conduction behaviour) in the pO_2 range of ~1 Pa and 10^{-5} Pa. In the lower pO_2 range (<~10^{-5} Pa), the conductivity increase with decreasing pO_2 (n-type behaviour). The porous $La_2Ti_2O_7$ ceramics sintered at 700 °C (relative density ~50%) however shows different behaviours [29]. In the pO_2 range of air and 1 Pa, a conductivity plateau (ionic conduction behaviour) is observed, in contrast to the p-type behaviour observed in the dense sample in this pO_2 range. The associated activation energy for conduction (E_a) is 1.25 eV. It was proposed that the oxygen ion conduction is caused by the Frenkel defects (simultaneous presence of defects of oxygen interstitials and oxygen vacancies) in the samples sintered at low temperature (700 °C) [29]. Such Frenkel defects are considered to be suppressed in the dense ceramic samples sintered at high temperature (1300 °C).

Another study on the ac conductivity of $La_2Ti_2O_7$ at fixed frequencies (1, 10 and 100 kHz) in the 20–880 °C temperature range revealed different E_a values at different temperature ranges [30]. At temperatures below 230 °C, the reported E_a values are surprisingly low (0.1 eV or less). Only at high temperatures (577–880 °C), an E_a value of 1.28 eV is observed. Such a value of E_a and a low frequency arc in the impedance complex plane plot have led the authors to conclude that the electrical conduction is associated with oxygen vacancies [30]. This conclusion is however inconsistent with the p-type behaviour observed in dense $La_2Ti_2O_7$ ceramics when measured in air [29]. Further study is therefore required to probe the origins of such inconsistencies on the electrical properties of $La_2Ti_2O_7$ ceramics reported in the literature.

In this study, we prepared textured $La_2Ti_2O_7$ ceramics using spark plasma sintering and investigated the electrical properties using impedance spectroscopy and Seebeck measurements. We show the substantial differences in electrical properties of the textured $La_2Ti_2O_7$ ceramics along the parallel and perpendicular directions, and clarify the electrical conduction mechanisms.

2. Experimental procedure

The $La_2Ti_2O_7$ single phase powder was obtained by the mixed oxide route. La_2O_3 (99.99%), and TiO_2 (99.9%) were used as the starting materials. The powders were milled using ZrO_2 balls with ethanol as the milling medium (QM-3SP04, Nanjing Nanda Instrument Plant, Nanjing, China). Then the powders were calcined in a normal chamber furnace in air at 1200 °C for 4 h. The powders were re-milled for 4 h to break any agglomerates and reduce the particle size. The ball milling speed was always 350 rpm. The synthesized powders were sintered in a spark plasma sintering (SPS) furnace (HPD 25/1, FCT, Rauenstein, Germany). A heating rate of 100 °C/min was used in all cases. The textured ceramic samples were fabricated using a two-step sintering method. First, the $La_2Ti_2O_7$ powder was pressed in a 20-mm-diameter graphite die and sintered at 1200 °C under a pressure of 80 MPa for 3 min. After this stage, the sample had high relative density (>95%), but the grain size was only slightly larger than that of the starting powder. In the second step, the samples were placed in a larger die of 30 mm diameter and sintered at 1450 °C under a pressure of 50 MPa for 5 min. The sintered disks were then heat treated in air at 1400 °C for 20 h to remove the carbon contamination. For the textured ceramics, the orientation of the surface with a normal line perpendicular to the SPS pressing direction is defined as perpendicular sample (⊥) and parallel to SPS pressing direction as parallel (//) (insert of Fig. 2a).

X-ray diffraction (XRD) patterns for the ceramics were obtained using an X-ray diffractometer (X'pertPro; PANalytical, Almelo, Netherlands) using Cu Kα radiation. The microstructure of the ceramic samples was examined using a scanning electron microscope (SEM; FEI Quanta 250, Hillsboro, Oregon, USA). The samples for SEM were polished, and then heated to a temperature 110 °C below their sintering temperatures for 30 min in air to etch and reveal their grain structure.

High temperature impedance spectroscopy measurements were performed using a Solartron 1255/1287 system and a ModuLab XM Materials Test System (Solartron Analytical, Farnborough, UK). Au paste (fired at 800 °C for 2 h) electrode was used. IS data were corrected for sample geometry (thickness/area of pellet) and

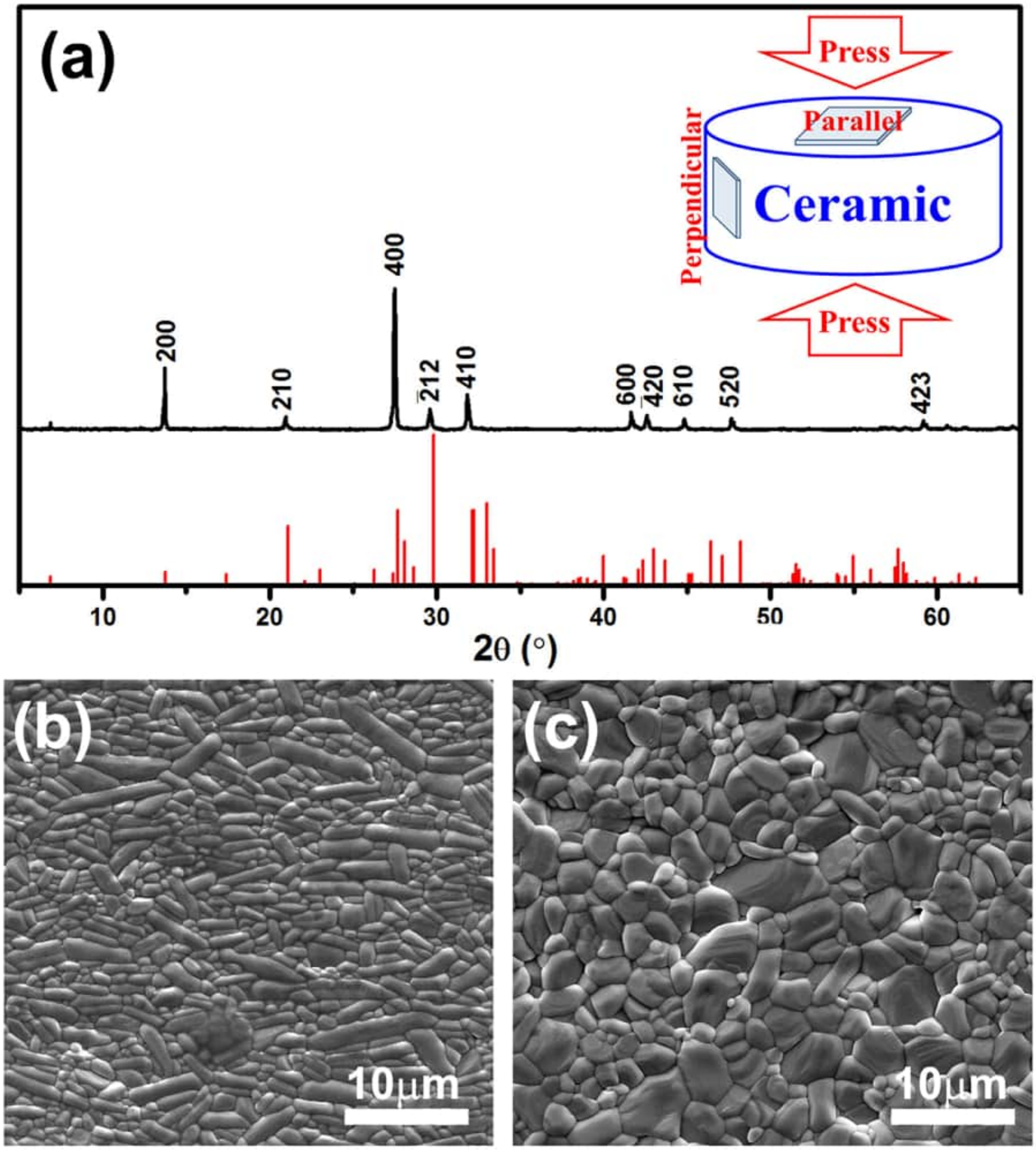

Fig. 2. (a) The XRD diffraction patterns of the textured $La_2Ti_2O_7$ ceramics in parallel direction (//); and the insert is a schema of directions of the samples. (b) The microstructure of the grains viewed in perpendicular direction (⊥). (c) The microstructure of the grains viewed in parallel direction (//).

analysed using ZView (Scribner Associates Inc., USA). Note that after geometry correction, resistance, R, effectively becomes resistivity and capacitance, C, becomes $\varepsilon_r\varepsilon_0$ (ε_0 is the permittivity of free space, 8.854×10^{-14} F cm^{-1}).

3. Results and discussion

Fig. 2a shows XRD patterns of grain-oriented, textured ceramics. The diffraction peaks match the indexed peaks for the $La_2Ti_2O_7$ crystal structure parameters [15,16]. The material is single-phase within the sensitivity of the technique. The crystal lattice parameters of the $La_2Ti_2O_7$ crystals are (a, b, c, β) = (13.0185 Å, 5.5474 Å, 7.8114 Å, 98°43′) [15]. The anisotropic structure leads to plate-like grain growth, which could be orientated using two-step SPS sintering process [11]. The XRD patterns are from the parallel sample, showing strong (h00) reflections, which indicates the grains have preferred orientation. The grain orientation factor, [11,31] f, was 0.81 for the textured $La_2Ti_2O_7$. Fig. 2b-c shows the SEM micrographs of a polished and thermally etched surface of perpendicular and parallel samples. Typically, the grains are plate-like and the morphological features in the two directions are different, which is consistent with the XRD data indicating the grains are well orientated. The thickness direction of the grains corresponds to the long a-axis of the single crystal structure, which is parallel to the pressing direction. The ferroelectric spontaneous polarization, P_s, is along the b-axis, which is perpendicular to the pressing direction [11].

Fig. 3 shows the capacitance (C) and relative permittivity (ε_r) of the $La_2Ti_2O_7$ ceramics as a function of frequency at room temperature (RT) and 500 °C. The capacitance values have been corrected with sample geometry (pellet thickness and area). C effectively becomes $\varepsilon_r\varepsilon_0$ (ε_r is the relative permittivity and ε_0 is the permittiv-

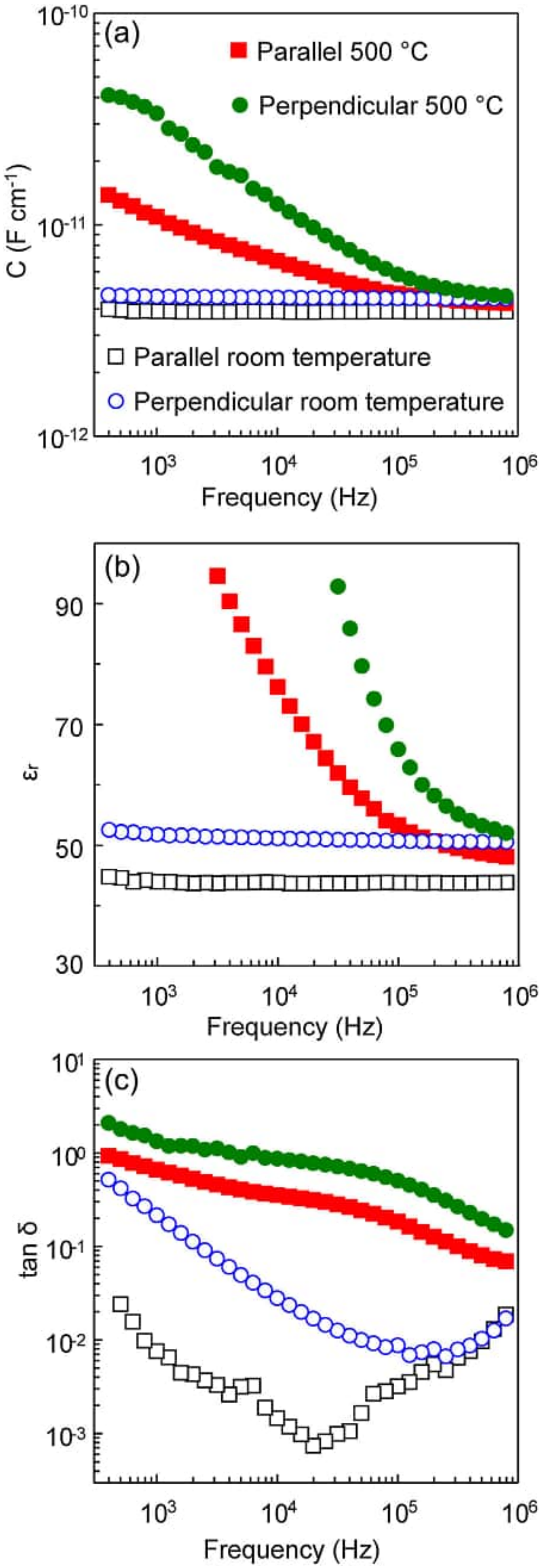

Fig. 3. Frequency dependence of (a) capacitance, C, (b) relative permittivity, ε_r, and (c) tan δ of the $La_2Ti_2O_7$ ceramics at room temperature and 500 °C measured along parallel and perpendicular directions.

ity of free space, 8.854×10^{-14} F cm^{-1}). At RT, the capacitance and ε_r exhibit negligible frequency dependence. Fig. 3b shows expanded view of ε_r at RT. ε_r along the perpendicular direction n (~51) is larger than that along the parallel direction (~44). The ε_r values are within the range (43–54) reported in the literature [8,11].

At 500 °C, the measured capacitance values show strong frequency dependence, which is related to the well-known Maxwell-Wagner effect. In an electrically heterogeneous system with two or more electro-active responses (such as grain core, grain shell, grain boundary responses), the capacitance value at a certain frequency is a composite term determined by the resistance and capacitance of all responses [31]. The low frequency capacitance values are typically dominated by non-bulk high capacitance responses (e.g., a grain boundary effect) [32]. With increasing frequency, the measured capacitance values decrease and become closer to intrinsic values at higher frequency. At 500 °C, capacitance values close to 1 MHz exhibit little frequency dependence and the capacitance at 1 MHz is considered to be close to the intrinsic value. As compared to the data at RT, the capacitance and ε_r values at 1 MHz at 500 °C show very little increase (by ~10%).

The tan δ at RT is low (<0.05) along the parallel direction, Fig. 3c. The tan δ at RT along the perpendicular direction is higher but is still <0.5. With increasing temperature tan δ increases due to increased conductivity. Again, the tan δ at 500 °C is higher along perpendicular direction than the parallel direction, implying higher conductivity along the perpendicular direction.

Impedance data reveal the electrical behaviours along parallel and perpendicular directions are indeed different. Typical impedance complex plane plots at 650 °C are shown in Fig. 4. Heavily suppressed arcs are observed for the data collected along both directions. To check whether oxide ion conduction is present, some impedance measurements were performed down to 0.01 Hz. For an oxide ion conductor, oxygen ion transport in electrode associated with Warburg diffusion will give rise to a spike followed by a semicircle in the low frequency impedance data [33]. No obvious presence of a spike and/or arc is observed for the as-prepared dense $La_2Ti_2O_7$ ceramics, suggesting oxygen ion is not the dominating charge carrier.

Impedance data are often modelled and quantitatively analysed using equivalent circuit consisting of capacitor, C, resistor, R, and constant phase element, CPE. It should be noted that one given set of data can be modelled mathematically using different equivalent circuits and choosing the correct equivalent circuit with appropriate physical meaning for each parameter is not always straightforward [34]. In the current case, both the suppressed arcs along the parallel and perpendicular directions can be modelled using one element consisting of parallel R-C-CPE. They can also be modelled using two parallel R-C-CPE elements connected in series. Fig. 4b shows that the two different circuits give very similar fitting results for the data along the perpendicular direction and it is difficult to identify which model or any other model is more appropriate from the mathematical fittings alone.

Here we take an alternative approach of analysing the impedance data. The raw impedance data contain a set range of frequency points, real and imaginary impedance values. The impedance data are inherently connected to other three basic complex formalisms: the electric modulus M*, the admittance Y* and the permittivity ε* [35–40]. These different formalisms highlight different physical features of the same data. For example, the combined spectroscopic plots of the imaginary components of impedance, Z" and electric modulus, M" of an electrically homogeneous phase with frequency independent permittivity and resistivity can be described by the following equations [37–39]:

$$Z'' = R\left[\frac{\omega RC}{1+(\omega RC)^2}\right] \quad (1)$$

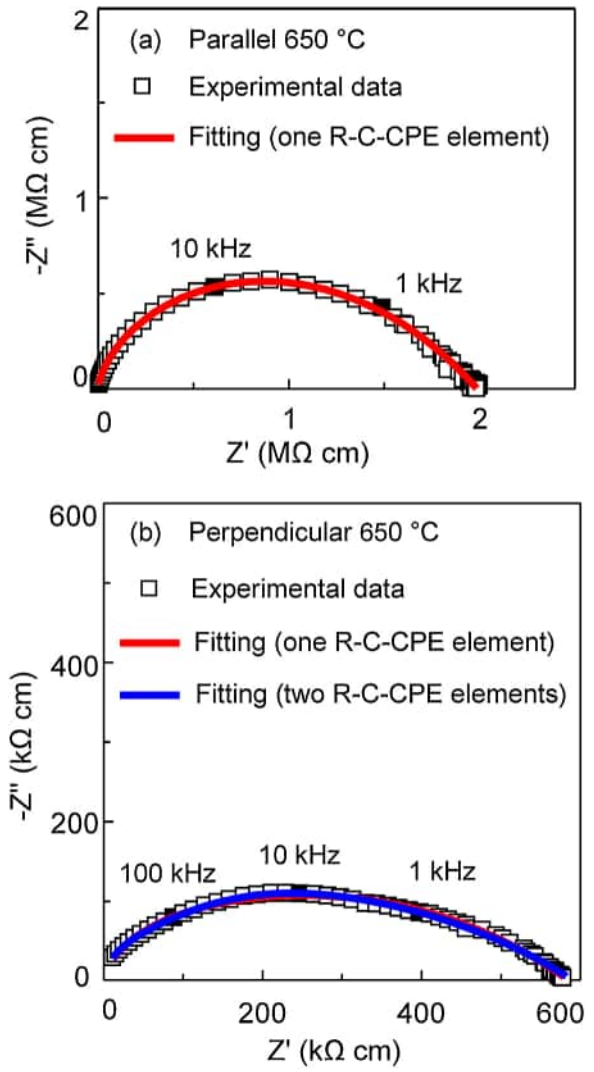

Fig. 4. Impedance complex plane plots of $La_2Ti_2O_7$ ceramics along (a) parallel direction and (b) perpendicular direction measured in air at 650 °C. Filled symbols indicate selected frequencies. The measurement frequency range is 0.1 Hz–1 MHz.

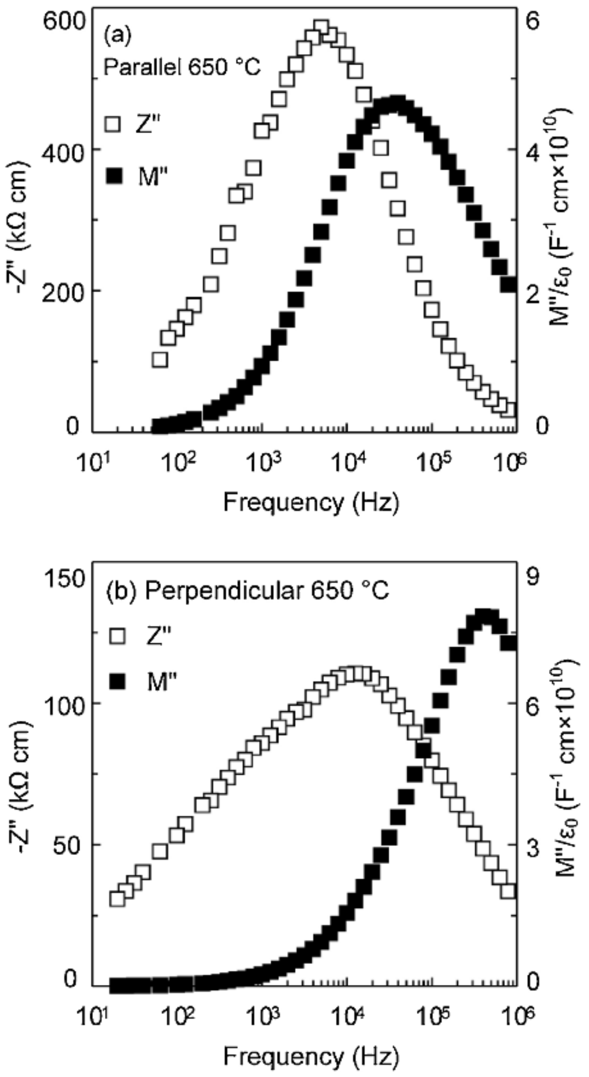

Fig. 5. Combined spectroscopic plots of the imaginary components of impedance, Z″ and electric modulus, M″ of $La_2Ti_2O_7$ ceramics along (a) parallel direction and (b) perpendicular direction measured in air at 650 °C.

$$M'' = \frac{C_0}{C}\left[\frac{\omega RC}{1+(\omega RC)^2}\right] \quad (2)$$

where C_0 is the capacitance of the empty cell ($C_0 = \varepsilon_0 A/d$, A is the sample area and d is the sample thickness), $\omega = 2\pi f$ and f is the applied frequency in Hz. The above equations bear similarity to the well-known Debye equation describing the imaginary part of permittivity [41]. Any component/phase present in different regions (e.g., grain, grain boundary) gives rise to a Debye-type peak in the Z″ and M″ spectroscopic plot. At the peak maxima $\omega_{max}RC = 1$ holds and it follows that:

$$Z'' = \frac{R}{2} \quad (3)$$

$$M'' = \frac{C_0}{2C} \quad (4)$$

Therefore the Z″ spectrum is dominated by the most resistive element, whereas the M″ spectrum is dominated by the element with the smallest capacitance, typically grain (bulk) response. The Z″, M″ Debye peak frequency is determined by:

$$f_{max} = 1/(2\pi RC) = 1/(2\pi\rho\varepsilon_0\varepsilon_r) \quad (5)$$

where ρ is resistivity. The bulk capacitance (and ε_r) can be calculated using the equation 4. Ideal Debye peaks have full-width at half-maximum (FWHM) values of 1.14 decades on a logarithmic frequency scale but they are rarely observed [37,38]. The M″ peaks may be depressed and have a FWHM > 1.14 decades. This could lead to overestimation of capacitance and ε_r values when the equation 4 is used. The bulk ε_r can also be obtained from the plot of ε_r as a function of frequency (Fig. 3), provided that the ε_r in such plots reflects the intrinsic bulk value. The grain (bulk) resistivity (ρ_b) can then be calculated using the equation 5. This method has been employed in previous studies [7,42].

Fig. 5 shows the combined Z″ and M″ plots of $La_2Ti_2O_7$ ceramics at 650 °C. The Z″ and M″ peaks are located at different frequencies. For an electrically homogeneous system, a single set of Z″/M″ peaks are expected at a single frequency (for example, see Fig. 1b in a previous report) [43]. The difference in peak frequency for Z″/M″ peaks is less than two orders of magnitude, suggesting that the inter-grains and intra-grains in $La_2Ti_2O_7$ ceramics exhibit different electrical responses. The difference is not large enough for the arcs in the impedance complex plots to be completely separated (Fig. 4), resulting in suppressed arcs shown in Fig. 4.

The Z″/M″ peaks in Fig. 5 are non-ideal and have a FWHM > 1.14 decades. In this work we calculated ρ_b at a given temperature using the equation 5 with M” Debye peak frequency at the related temperature and the ε_r values at 1 MHz and RT. Fig. 3 shows that the

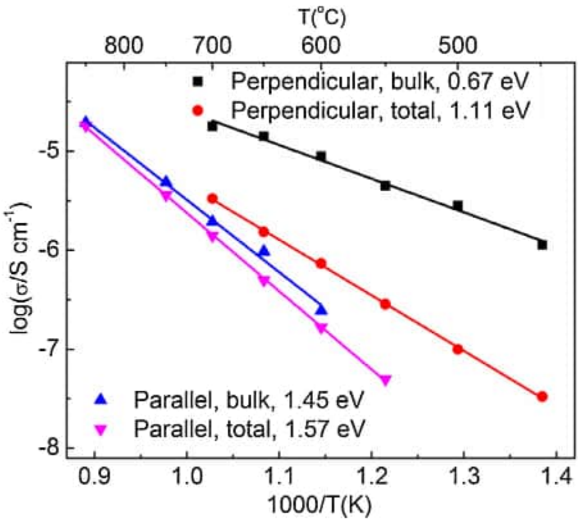

Fig. 6. Arrhenius-type plots of bulk conductivity, σ_b, and total conductivity, σ_t, of $La_2Ti_2O_7$ ceramics along parallel and perpendicular directions.

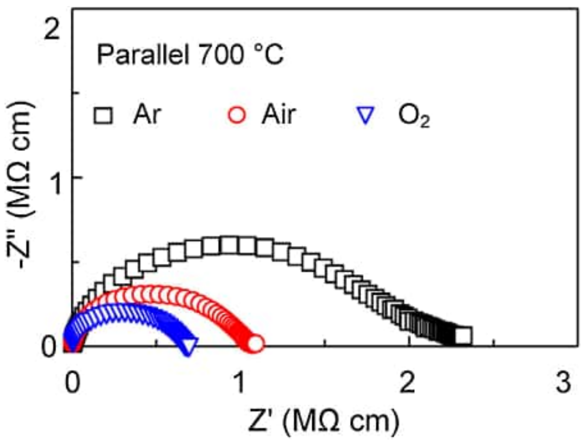

Fig. 7. Impedance complex plane (Z^*) plots under different oxygen partial pressure for $La_2Ti_2O_7$ ceramics along the parallel direction.

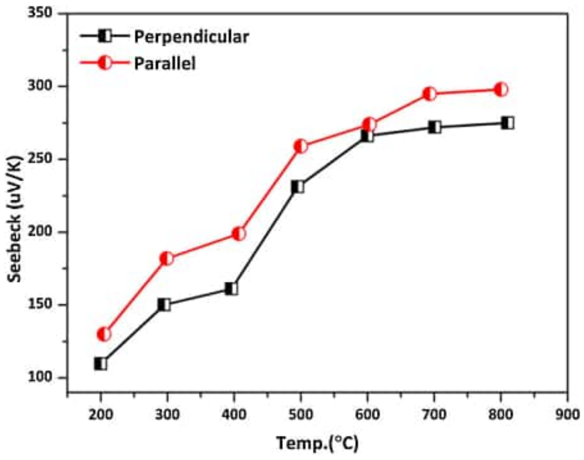

Fig. 8. Seebeck coefficients of the $La_2Ti_2O_7$ ceramics in parallel and perpendicular direction, respectively.

change of ε_r with temperature is very small compared to the change of conductivity with temperature. Thus it is justified to use the ε_r at RT to calculate the ρ_b at different temperatures. The total resistivity (ρ_t) is obtained from the low frequency intercept on the x axis in the impedance complex plane plots (Fig. 4).

The temperature dependence of bulk conductivity, σ_b ($\sigma_b = 1/\rho_b$) and total conductivity, σ_t ($\sigma_t = 1/\rho_t$) are summarised in the Arrhenius-type plot, Fig. 6. Both the σ_b and σ_t along the perpendicular direction are higher than those along the parallel direction. The E_a values for electrical conduction along the parallel direction are about 1.5 eV from both bulk and total conductivity. The reported optical band gap (E_g) of $La_2Ti_2O_7$ in the literature is 3.29 eV [44]. The E_a of 1.5 eV is close to that of intrinsic (band gap) electronic conduction (half of the E_g, 1.65 eV). The electrical conduction along the perpendicular direction exhibits much lower E_a values, 0.67 eV for bulk conductivity and 1.11 eV for total conductivity.

The Z^* plots at 700 °C of $La_2Ti_2O_7$ ceramics along the parallel direction under different atmospheres (Ar, air and O_2) shows the electrical conductivity increases with pO_2, Fig. 7, suggesting a p-type behaviour. Thermopower measurements at 200–800 °C reveal that $La_2Ti_2O_7$ ceramics exhibit positive Seebeck coefficients along both parallel and perpendicular directions, Fig. 8, confirming the dominance of p-type conduction. In general, electrical conductivity is directly proportional to carrier concentration whereas Seebeck coefficient is inversely related to carrier concentration. The Seebeck coefficient in parallel direction is higher than that in perpendicular direction, consistent with the conductivity results that the conductivity in perpendicular direction is higher than that in parallel direction.

The above impedance data and Seebeck coefficients of the $La_2Ti_2O_7$ ceramics indicate the electrical conduction is dominated by hole conduction along both parallel and perpendicular directions. No clear evidence of oxide ion conduction is observed, at least for the frequency range (0.01 Hz–1 MHz), temperature range (RT—850 °C) and pO_2 range (Ar, air and O_2) used in this study. For the electrical conduction along the parallel direction, the E_a is close to half of E_g, suggesting the electrical conduction is close to intrinsic (band gap) electronic conduction. The origin of the lower E_a and higher conductivity for the electrical conduction along the perpendicular direction is unclear at this stage. It is speculated this is related to the crystal structure and/or ceramic microstructure for the textured ferroelectric $La_2Ti_2O_7$ ceramics. Further study is needed to understand the mechanism.

The p-type conduction for our dense, textured $La_2Ti_2O_7$ ceramics measured in pO_2 around atmosphere air is consistent with the behaviour of the dense $La_2Ti_2O_7$ ceramics observed in the previous report [29]. The p-type conduction is also commonly observed in other known titanates such as $SrTiO_3$ and $BaTiO_3$ [45]. One popular model to explain the p-type behaviour is that low levels of unintentional acceptor-type impurities (e.g., Al_2O_3, Fe_2O_3) are present that create oxygen vacancies according to the following reaction:

$$A_2O_3 \leftrightarrow 2A'_{Ti} + 3O_O^{\times} + V_O^{\bullet\bullet} \quad (6)$$

The oxygen vacancies react with molecular oxygen at high oxygen activity, generating holes by the following reaction:

$$\frac{1}{2}O_2 + V_O^{\bullet\bullet} \rightarrow O_O^{\times} + 2h^{\bullet} \quad (7)$$

Another model to explain the p-type behaviour involves formation of Schottky-type defects, i.e., cation vacancies and oxygen vacancies [46]. In the current case, low levels of nonstoichiometry associated with either La or Ti vacancies could generate oxygen vacancies that give holes at high pO_2.

4. Conclusions

In this study, we prepared textured $La_2Ti_2O_7$ ceramics, which is a typical PLS ferroelectric, using spark plasma sintering and investigated the electrical properties using impedance spectroscopy and Seebeck measurements. The textured $La_2Ti_2O_7$ ceramics exhibit high resistivity along the parallel direction. E_a is close to half the optical band gap, suggesting the electrical conduction is dominated by near intrinsic electronic conduction. Along the perpendicular direction the resistivity and E_a are lower. Impedance measurements at different atmospheres and Seebeck measurements indicate the dominating charge carrier is hole for the electrical conduction along both directions when measured in pO_2 around atmosphere air and at RT—850 °C. Impedance data collected at low frequencies down 0.01 Hz reveals no obvious evidence of oxide ion conduction.

Acknowledgements

This work is partly supported by the LSD project with grant No. 2016Z-04 and CSS project with grant No. YK2015-0602006.

References

[1] B. Jaffe, W. Cook, H. Jaffe, Piezoelectric Ceramics, 3, Academic Press, 1971, pp. 317.
[2] R. Turner, P. Fuierer, R. Newnham, T. Shrout, Materials for high − temperature acoustic and vibration sensors—a review, Appl. Acoust. 41 (1994) 299–324.
[3] D. Damjanovic, Materials for high temperature piezoelectric transducers, Curr. Opin. Solid State Mater. Sci. 3 (1998) 469–473.
[4] S.J. Zhang, F.P. Yu, Piezoelectric materials for high temperature sensors, J. Am. Ceram. Soc. 94 (2011) 17–34.
[5] S.J. Zhang, E. Frantz, R. Xia, W. Everson, J. Randi, D. Snyder, T. Shrout, Gadolinium calcium oxyborate piezoelectric single crystals for ultrahigh temperature (>1000 °C) applications, J. Appl. Phys. 104 (2008) 084103–084107.
[6] S.J. Zhang, Y. Fei, E. Frantz, D. Snyder, H. Chai, T. Shrout, High − temperature piezoelectric single crystal $ReCa_4O(BO_3)_3$ for sensor applications, IEEE Trans. Ultrason. Ferroelectr. Freq. Control 55 (2008) 2703–2708.
[7] S. Costa, M. Li, J. Frade, D.C. Sinclair, Modulus spectroscopy of $CaCu_3Ti_4O_{12}$ ceramics: clues to the internal barrier layer capacitance mechanism, RSC. Adv. 3 (2013) 7030–7036.
[8] Z.P. Gao, H.X. Yan, H.P. Ning, R. Wilson, X.Y. Wei, B.G. Shi, H.T. Ye, M.J. Reece, Piezoelectric and dielectric properties of Ce substituted $La_2Ti_2O_7$ ceramics, J. Eur. Ceram. Soc. 33 (2013) 1001–1008.
[9] Z.P. Gao, H.X. Yan, H.P. Ning, M.J. Reece, Ferroelectricity of $Pr_2Ti_2O_7$ ceramics with super-high Curie point, Adv. Appl. Ceram. 112 (2012) 69–74.
[10] H.P. Ning, H.X. Yan, M.J. Reece, Piezoelectric strontium niobate and calcium niobate ceramics with super −high Curie points, J. Am. Ceram. Soc 93 (2010) 1409–1413.
[11] H.X. Yan, H.P. Ning, Y. Kan, P. Wang, M.J. Reece, Piezoelectric ceramics with super-high curie points, J. Am. Ceram. Soc. 92 (2009) 2270–2275.
[12] F. Lichtenberg, A. Herrnberger, K. Wiedenmann, Synthesis, structural, magnetic and transport properties of layered perovskite-related titanates, niobates and tantalates of the type $A_nB_nO_{3n+2}$, $A'A_{k-1}B_kO_{3k+1}$ and $A_mB_{m-1}O_{3m}$, Prog. Solid State Chem. 36 (2008) 253–387.
[13] F. Lichtenberg, A. Herrnberger, K. Wiedenmann, J. Mannhart, Synthesis of perovskite-related layered $A_nB_nO_{3n+2} = ABO_x$ type niobates and titanates and study of their structural, electric and magnetic properties, Prog. Solid State Chem. 29 (2001) 1–70.
[14] N. Ishizawa, F. Marumo, S. Iwai, M. Kimura, T. Kawamura, Compounds with perovskite-type slabs. 5. A high-temperature modification of $La_2Ti_2O_7$, Acta Crystallogr. 38 (1982) 368–372.
[15] S. Nanamats, M. Kimura, K. Doi, S. Matsushi, N. Yamada, New ferroelectric − $La_2Ti_2O_7$, Ferroelectrics 8 (1974) 511–513.
[16] S. Nanamats, M. Kimura, N. Yamada, Ferroelectric properties of $La_2Ti_2O_7$ crystal, NEC Res. Dev. 34 (1974) 39–42.
[17] R.S. Roth, Pyrochlore-type compounds containing double oxides of trivalent and tetravalent ions, J. Res. Nat. Bur. Stand. 56 (1) (1956) 17–25.
[18] J.B. MacChesney, H.A. Sauer, The system La_2O_3-TiO_2; phase equilibria and electrical properties, J. Am. Ceram. Soc. 45 (9) (1962) 416–422.
[19] K. Scheunemann, H.K. Müller-Buschbaum, Zur kristallstruktur von $La_2Ti_2O_7$, J. Inorg. Nucl. Chem. 37 (9) (1975) 1879–1881.
[20] S.D. Škapin, D. Kolar, D. Suvorov, Phase stability and equilibria in the La_2O_3-TiO_2 system, J. Eur. Ceram. Soc. 20 (8) (2000) 1179–1185.
[21] J. Takahashi, K. Kageyama1, T. Hayashi, Dielectric properties of double-oxide ceramics in the system Ln_2O_3-TiO_2 (Ln = La, Nd and Sm), Jpn. J. Appl. Phys. 30 (9B) (1991) 2354–2358.
[22] J. Takahashi, K. Kageyama1, K. Kodaira, Microwave dielectric properties of lanthanide titanate ceramics, Jpn. J. Appl. Phys. 32 (9B) (1993) 4327–4332.
[23] A.F. Paul, E.N. Robert, $La_2Ti_2O_7$ ceramics, J. Am. Ceram. Soc. 74 (1991) 2876–2881.
[24] V. Atuchin, T. Gavrilova, J. Grivel, V. Kesler, Electronic structure of layered ferroelectric high-k titanate $La_2Ti_2O_7$, J. Phys. D: Appl. Phys. 42 (2009) 1404–1406.
[25] E. Bruyer, A. Sayede, Density functional calculations of the structural, electronic, and ferroelectric properties of high-k titanate $Re_2Ti_2O_7$ (Re = La and Nd), J. Appl. Phys. 108 (2010) 053705.
[26] Z.M. Shao, S. Saitzek, P. Roussel, A. Ferri, O. Mentré, R. Desfeux, Investigation of microstructure in ferroelectric lead-free $La_2Ti_2O_7$ thin film grown on (001)-$SrTiO_3$ substrate, CrystEngComm 14 (2012) 6524–6533.
[27] Z.P. Gao, T. Suzuki, S. Grasso, Y. Sakka, M.J. Reece, Highly anisotropic single crystal − like $La_2Ti_2O_7$ ceramic produced by combined magnetic field alignment and templated grain growth, J. Eur. Ceram. Soc. 35 (2015) 1771–1776.
[28] C.C. Li, J. Khaliq, H.P. Ning, X.Y. Wei, H.Y. Yan, M.J. Reece, Study on properties of tantalum-doped $La_2Ti_2O_7$ ferroelectric ceramics, J. Adv. Dielectr. 5 (2015) 1550005.
[29] H. Takamura, K. Enomoto, A. Kamegawa, M. Okada, Electrical conductivity of layered compounds in SrO?La_2O_3?TiO_2 systems prepared by the pechini process, Solid State Ion. 154 (2002) 581–588.
[30] D. Fasquelle, J. Carrua, L. Le Gendreb, C. Le Pavenb, J. Pinelb, F. Cheviréc, F. Tessierc, R. Marchandc, Lanthanum titanate ceramics: electrical characterizations in large temperature and frequency ranges, J. Eur. Ceram. Soc. 25 (2005) 2085–2088.
[31] F. Lotgering, Topotactical reaction with ferromagnetic oxides having hexagonal crystal structures. I, J. Inorg. Nucl. Chem. 9 (1959) 113–123.
[32] M. Li, D.C. Sinclair, A.R. West, Extrinsic origins of the apparent relaxorlike behavior in $CaCu_3Ti_4O_{12}$ ceramics at high temperatures: a cautionary tale, J. Appl. Phys. 109 (2011) 084106.
[33] M. Li, M.J. Pietrowski, R. De Souza, H.R. Zhang, I.M. Reaney, S.N. Cook, J.A. Kilner, D.C. Sinclair, A family of oxide ion conductors based on the ferroelectric perovskite $Na_{0.5}Bi_{0.5}TiO_3$, Nat. Mater. 13 (2014) 31.
[34] A.R. West, D.C. Sinclair, N. Hirose, Characterization of electrical materials, especially ferroelectrics, by impedance spectroscopy, J. Electroceram. 1 (1997) 65–71.
[35] E. Barsoukov, J.R. Macdonald, Impedance Spectroscopy: Theory, Experiment, and Applications, John Wiley & Sons, New Jersey, 2005.
[36] I. Hodge, M. Ingram, A.R. West, Impedance and modulus spectroscopy of polycrystalline solid electrolytes, J. Electroanal. Chem. Interfacial Electrochem. 74 (1976) 125–143.
[37] D.C. Sinclair, A.R. West, Impedance and modulus spectroscopy of semiconducting $BaTiO_3$ showing positive temperature coefficient of resistance, J. Appl. Phys. 66 (1989) 3850–3856.
[38] J. Irvine, D.C. Sinclair, A.R. West, Electroceramics: characterization by impedance spectroscopy, Adv. Mater. 2 (1990) 132–138.
[39] P. Debye, Polar Molecules, Dover Publishers, New York, 1945.
[40] F.D. Morrison, D.C. Sinclair, A.R. West, Characterization of lanthanum-doped barium titanate ceramics using impedance spectroscopy, J. Am. Ceram. Soc. 84 (2001) 531–538.
[41] J.S. Dean, J.H. Harding, D.C. Sinclair, Simulation of impedance spectra for a full three − dimensional ceramic microstructure using a finite element model, J. Am. Ceram. Soc. 97 (2014) 885–891.
[42] L. Enciso-Maldonado, M. Dyer, M. Jones, M. Li, J. Payne, M. Pitcher, M. Omir, J. Claridge, F. Blanc, M. Rosseinsky, Computational identification and experimental realization of lithium vacancy introduction into the olivine $LiMgPO_4$, Chem. Mater. 27 (2015) 2074–2091.
[43] M. Li, L. Li, J. Zang, D.C. Sinclair, Donor-doping and reduced leakage current in Nb-doped $Na_{0.5}Bi_{0.5}TiO_3$, Appl. Phys. Lett. 106 (2015) 102904.
[44] M. Uno, A. Kosuga, M. Okui, K. Horisaka, S. Yamanaka, Photoelectrochemical study of lanthanide titanium oxides, $Ln_2Ti_2O_7$ (Ln = La, Sm, and Gd), J. Alloy Compd. 400 (2005) 270–275.
[45] D.M. Smyth, The Defect Chemistry of Metal Oxides, Oxford University Press, New York, 2000.
[46] P. Erhart, K. Albe, Modeling the electrical conductivity in $BaTiO_3$ on the basis of first-principles calculations, J. Appl. Phys. 104 (2008) 044315.

THE JOURNAL OF PHYSICAL CHEMISTRY C

Article

pubs.acs.org/JPCC

Effect of Dopant–Host Ionic Radii Mismatch on Acceptor-Doped Barium Zirconate Microstructure and Proton Conductivity

Elisa Gilardi,[†] Emiliana Fabbri,[‡] Lei Bi,[§] Jennifer L. M. Rupp,[∥] Thomas Lippert,[†,¶] Daniele Pergolesi,[*,†] and Enrico Traversa[*,⊥,#]

[†]Thin Films and Interfaces, Research with Neutrons and Muons Division, Paul Scherrer Institut, 5232 Villigen, PSI, Switzerland

[‡]Electrochemistry Laboratory, Paul Scherrer Institut, 5232 Villigen, Switzerland

[§]Institute of Materials for Energy and Environment, College of Materials Science and Engineering, Qingdao University, Ningxia Road No. 308, Qingdao 266071, China

[∥]Electrochemical Materials Laboratory, Massachusetts Institute of Technology, MIT, 02139 Cambridge, Massachusetts, United States

[¶]Department of Chemistry and Applied Biosciences, Laboratory of Inorganic Chemistry, Vladimir-Prelog-Weg 1-5/10, ETH Zürich, 8093 Zürich, Switzerland

[⊥]International Research Center for Renewable Energy, Xi'an Jiaotong University, Xi'an, Shaanxi 710049, P. R. China

[#]NAST Center & Department of Chemical Science and Technology, University of Rome Tor Vergata, 00133 Rome, Italy

Downloaded via NANJING TECH UNIV on February 1, 2025 at 06:48:20 (UTC).
See https://pubs.acs.org/sharingguidelines for options on how to legitimately share published articles.

ABSTRACT: In the present study, morphological and electrical properties of $BaZrO_3$ were investigated as a function of the ionic radii mismatch between Zr and the different B-site dopants (Al, Sc, In, Lu, Tm, Y, Gd, Sm, Nd, and La) for the same solute concentration and valence. Our study highlights the critical role of the ionic radius of the acceptor dopant on stability, sinterability, and proton conductivity of barium zirconate. From our study, Gd-doped barium zirconate emerges as a novel promising material for proton conductor electrolytes.

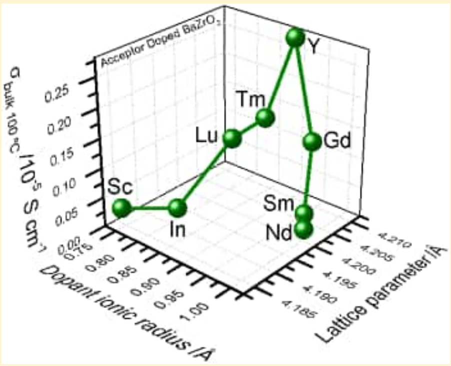

■ INTRODUCTION

High temperature proton conductors (HTPCs) have received growing interest in the last decades as potential electrolyte materials in intermediate temperature (IT, 400–600 °C) solid oxide fuel cells.[1−3] HTPCs, particularly perovskite-type oxides, show high proton conductivity (larger than 10^{-2} S cm^{-1} at 450 °C) when exposed to hydrogen and/or water vapor containing atmospheres.[4−6]

The substitution of the B^{4+} cation with a trivalent element M^{3+} in the ABO_3 perovskite structure leads to the formation of oxygen-ion vacancies; in the presence of water vapor the water molecule dissociates into a hydroxide ion and a proton: the former fills an oxygen vacancy, while the latter can form a covalent bond with lattice oxygen (dissociative absorption of water).[7−10]

Once protonic defects are incorporated inside the lattice, protons migrate by hopping between adjacent oxygen ions via a Grotthuss-type mechanism.[11]

Doped $BaZrO_3$ (BZ) and $BaCeO_3$ (BC) are up to now the most promising perovskite oxides, but despite their high protonic conductivity many factors limit their practical use; such as the poor sinterability, the low grain boundary conductivity, and the inevitable BaO evaporation during sintering at high temperatures for the former, as well as the low chemical stability in water and CO_2 containing environments for the latter.[12−15]

The effect of different trivalent dopants on the sintering and conductive properties of perovskite oxides has been extensively studied in the past years and in particular the close relation between proton mobility and perovskite structural parameters has attracted much attention.[16−28]

Han et al.[29] studied the relation between the structural properties and the bulk proton conductivity in acceptor-doped BZ and suggested a linear relation between the bulk conductivity and the variation of lattice constant upon hydration. They indicated Tm as the best dopant, while for doping with ionic radii larger than Dy the bulk protonic conductivity drastically decreases.

On the other hand, Gorelov et al.[30] suggested that for doped barium zirconate there is no correlation between the total conductivity and the ionic radius of dopants, reporting a more

Received: March 7, 2017
Revised: April 10, 2017
Published: April 10, 2017

ACS Publications © 2017 American Chemical Society

DOI: 10.1021/acs.jpcc.7b02163
J. Phys. Chem. C 2017, 121, 9739−9747

general trend of decreased conductivity in the following order Ho > Sc > Dy, In > Gd.

Additionally, other important factors as the different chemical nature of the cations, as well as the carrier concentration and the trapping effect between protons and dopants have been reported to have a specific influence on proton migration.[7,13,31] In general different correlations between the nature of the dopants and the structural/conducting properties of the perovskite have been suggested.[27,29,30,32−35] In the case of Sc-, In-, Y-, and Gd-doped barium zirconates, the chemical matching in term of basicity and electronegativity for the solute-host was suggested to be the most relevant parameter for the choice of the trivalent dopant since it affects directly the proton mobility. Other parameters as symmetry and lattice constant are instead less critical.[27] In particular, a similar electron density of the oxygen ions coordinated to the Y and to the Zr cations was indicated as one of the main reasons of the unique proton transport properties of Y-doped $BaZrO_3$. The highest proton diffusivity and the lowest activation enthalpy were found for 10 to 20 mol % Y-doped barium zirconate, with tetragonal symmetry. Differently, smaller dopant cations such as Sc lowered the proton mobility, even though the host dopant size mismatch led to smaller cell distortion, allowing the cell to retain the cubic symmetry.

Moreover, for equal dopant content, synthesis method and sintering time, the ionic radius of the dopant has been demonstrated to affect considerably the sintering properties of the samples and thus the microstructure. This in turn affects the protonic transport properties of barium zirconate doped with different trivalent cations.[33] Small cations (Sc and In) led to the formation of relatively large and uniform average grains size; these zirconates showed moderate proton conductivity. In contrast, doping with larger cations (Y, Yb, Tu, Er, Ho) led to the formation of grains with nonuniform and overall smaller average grain sizes showing comparatively higher protonic conductivity. It worth noting that a uniform micromorphology could be achieved increasing the sintering time from 24 to 100 h.[36]

Since structural, chemical and morphological parameters deeply affect the proton conducting properties of doped barium zirconates, this study aims in providing a comprehensive understanding of how these parameters vary as a function of the ionic radii mismatch between Zr and different B-site dopants for equal solute concentration and valence. We contribute to the ongoing debate of the dopant-host lattice mismatch on alteration of protonic conduction and its specific correlation. Despite first studies in the field on microstructural evolution and the implication of dopants acting on grain and grain boundary matrices, still knowledge on sintering and host lattice incorporation of dopants is required. This ultimately affects the ceramic processing approaches to fabricate the next generation proton conducting fuel cells and electrolyzers.

In particular, several trivalent elements in the series Al, Sc, In, Lu, Tm, Y, Gd, Sm, Nd, and La have been substituted in the $BaZrO_3$ lattice and the effects of the dopant on the lattice parameter, sinterability, bulk, specific grain boundary, and total conductivity were investigated.

While in the literature Y is generally identified as the best dopant for $BaZrO_3$, our study unveils that Gd-doped barium zirconate is also a promising protonic conductor.

■ METHODS

Materials Synthesis. For the synthesis of $BaZr_{0.8}M_{0.2}O_{3-\delta}$ (BZ-M, with M = Al, Sc, In, Lu, Tm, Y, Gd, Sm, Nd, La) $Ba(NO_3)_2$ (99.9%), $ZrO(NO_3)_2\cdot 2H_2O$ (97%), $Al(NO_3)_3\cdot 9H_2O$ (99.9%), Sc_2O_3 (99.9%), $In(NO_3)_3\cdot 6H_2O$, Lu_2O_3 (99.9%), $Tm(NO_3)_3\cdot 5H_2O$ (99.9%), $Y(NO_3)_3\cdot 6H_2O$ (99.99%) $Gd(NO_3)_3$ (99.5%), $Sm(NO_3)_3$ (99.5%), $Nd(NO_3)_3$ (99.5%), and La_2O_3 (99.9%) were used as starting materials. Sc_2O_3, Lu_2O_3, and La_2O_3 were first dissolved in a 0.2 M nitric acid solution. $Ba(NO_3)_2$, $ZrO(NO_3)_2$, and $M(NO_3)_3$ were successively dissolved in 200−300 mL of deionized water under continuous stirring at 40 °C to produce transparent metal-nitrate solutions. Citric acid, which is able to bind the metal ions and acts as a fuel in combustion reaction, was then added to the metal-nitrates solution. The molar ratio of citric acid (CA) with respect to the total metal cation (MC) content was set to 1.5 NH_4OH and was added to the solution to adjust the pH value around 8. The transparent aqueous solution containing metal nitrates and citric acid was heated up to 80 °C overnight on a hot plate under stirring. Water vapor gradually evolved with a corresponding increase in viscosity. Finally, the solution was converted to a viscous gel due to thermal dehydration and ignited to a flame, resulting in black ash. The BZ-M powders were calcined under static air at 1100 °C for 6 h to obtain single phase oxides.

Dense BZ-M pellets, 13 mm in diameter and about 1 mm thick, were obtained by uniaxially pressing the powders at 150 MPa for 1 min and subsequently by sintering at 1600 °C for 8 h in air (pellets were covered with powders of the same composition to limit Ba evaporation) with a heating and cooling rate of 5 °C min^{-1}.

For Al, In, Gd, Sm, Nd, and La as dopants the density of the samples was above 94% of the theoretical value. BZY reaches a relative density of 90%, while for M = Sc, Tm, and Lu the relative densities were between 72% and 76%.

Microstructure Characterization. Phase identification of the calcined powders and sintered pellets was carried out using X-ray diffraction (XRD, Rigaku, Ultima III) analysis with Cu K_α radiation using a scanning rate of 2° min^{-1} at room temperature, calibrating the XRD offset using a Si wafer. The pseudocubic lattice parameters were calculated using the MDI Jade program.

The pellet microstructure was observed using a field emission scanning electron microscope (FE-SEM, Hitachi, S-4800). BZ-M compact rectangular bars, with dimensions of 1 mm × 5 mm × 10 mm, were fabricated by uniaxially pressing the ceramic powders at 245 MPa for 1 min. The shrinkage behavior of the green samples was measured from room temperature to 1600 °C with a heating rate of 10 °C min^{-1} using a thermal expansion analyzer (DIL 402C, Netzsch) in flowing air.

Chemical Analysis. The powders and pellet compositions were checked by inductively coupled plasma atomic emission spectroscopy (ICP-AES, Nippon Jarrell-Ash Co., Kyoto, Japan). Experiments were performed at least three times on a sample from the same batch to determine the uncertainty of the concentration of the cations.

Impedance Spectroscopy. For conductivity measurements, two symmetrical porous Pt electrodes were coated onto both sides of polished BZ-M pellets and heated to 1000 °C for 20 min. Impedance spectroscopy (IS) measurements were performed in humidified Ar (ca. 3 vol % H_2O) atmospheres using a multichannel potentiostat VMP3 (Bio-

DOI: 10.1021/acs.jpcc.7b02163
J. Phys. Chem. C 2017, 121, 9739−9747

Logic Co.), in the 0.1 Hz−1 MHz frequency range, with an applied AC voltage amplitude of 100−300 mV. ZView software was used to fit the acquired impedance data to equivalent circuits.

IS measurements, performed in humidified atmosphere between 25 and 700 °C were used to evaluate the conductivity of BZ-M pellets as well as to distinguish the grain interior and the grain boundary contributions. Impedance plot have been fitted with three equivalent circuits connected in series, each equivalent circuit constituted of a resistance and a constant phase element in parallel. The different time constants (τ = RC) of the three circuits enable the separation of the frequency domain in grain-interior (bulk), grain-boundary and electrode contributions. Below 300 °C the complex impedance plane plot of all samples displayed three semicircles, the first one at high-frequencies was assigned to the bulk of BZ-M ceramics. In the case of the most resistive samples (particularly Sm-, Nd-, La-, and Al-doped $BaZrO_3$) the bulk semicircle was already accessible below 400 °C. The second semicircle at intermediate frequencies was assigned to the grain boundary contribution while the third semicircle at low frequencies was instead attributed to the electrode processes. From the analysis of the complex impedance plots bulk, grain boundary and total conductivity were calculated.

Only for BZ-Nd and BZ-Tm the impedance plots were too scattered in the low frequency range to allow a precise estimation of the intercept on the real axis and therefore of the grain boundary resistance, while the bulk semicircle was clearly visible at low temperature in the high frequency range of the plot.

The temperature range used to calculate the activation energy corresponds to the range where the conductivity measurements were performed and it is shown in Figure 7 for all samples.

The specific grain boundary conductivity was calculated according to the brick layer model, under the assumption that the dielectric constants of bulk and grain boundary are very close ($\varepsilon_{bulk} \approx \varepsilon_{g.b.}$), using a single temperature independent $C_{bulk}/C_{g.b.}$ for each sample, as follows:[37]

$$\sigma_{grain\ boundaries,spec} = \sigma_b \frac{R_{bulk}C_{bulk}}{R_{g.b.}C_{g.b.}}$$

■ RESULTS AND DISCUSSIONS

Structural Analysis of BZ-M Powders. Parts a−j of Figure 1 show the X-ray diffraction (XRD) patterns for $BaZr_{0.8}M_{0.2}O_{3-\delta}$ (BZ-M, with M = Al, Sc, In, Lu, Tm, Y, Gd, Sm, Nd, La) powders calcined under static air at 1100 °C for 6 h. Barium zirconate doped with 20 mol % of Al, Sc, In, Lu, Tm, Y, Gd, and Sm shows only the diffraction peaks associated with the cubic perovskite structure (Figure 1a−h), while a clear presence of secondary phases can be observed in the case of doping with the larger cations, namely Nd and La (Figure 1i,j). Figure 1k compares the XRD plots of the different BZ-M compositions in the angular region around the (110) diffraction peak which is the highest intensity peak. The smallest angular position of the (110) diffraction peak, i.e., the largest lattice parameter of the cubic cell, was found for yttrium doping; while for smaller and larger ionic radii of the dopant the angular position of the (110) diffraction peak shifts toward larger 2θ values, indicating a smaller lattice parameter.

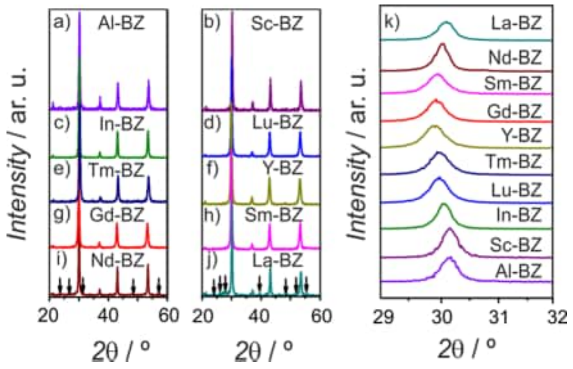

Figure 1. (a−j) XRD patterns of $BaZr_{0.8}M_{0.2}O_{3-\delta}$ (M = Al, Sc, In, Lu, Tu, Y, Gd, Sm, Nd, La) powders. Diffraction peaks belonging to secondary phases are indicated by arrows (i, j). (k) Magnification of the angular region around the (110) diffraction peak of the different compounds.

Figure 2 shows the lattice parameters (pseudocubic symmetry) calculated from the XRD measurements as a function of the dopant ionic radius in octahedral coordination. This highlights the variation of the lattice parameter as a function of the dopant ionic radius, and therefore the structural modifications induced by the dopant to the $BaZrO_3$ cell. Only the BZ-M compositions comprising a cation with an ionic radius ranging between Sc and Y follow Vegard's law. BZ-Al shows a larger lattice parameter than the one expected from the linear relation between lattice parameter of the doped barium zirconate and ionic radius of the dopant. This suggests that Al has been only partially substituted into the barium zirconate lattice. The absence of diffraction peaks from secondary phases indicates that the segregated phase is amorphous.

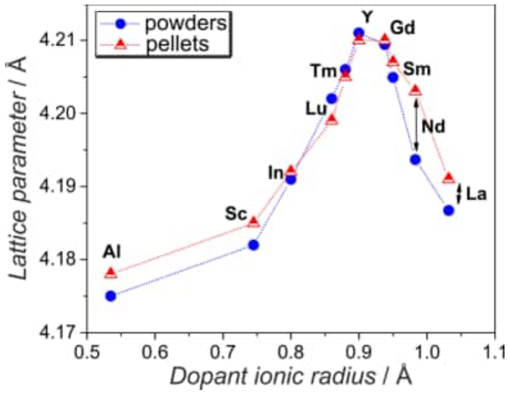

Figure 2. Lattice parameter of BZ-M calculated from the XRD pattern of the powder and pellet plotted as a function of the ionic radius of the dopants.

From Y to La the lattice parameter decreases with increasing the dopant ionic radius. Within the limit of the XRD analysis, no secondary phases were observed for Gd and Sm-doped barium zirconate. The decrease of the lattice parameters may be interpreted as the result of dopant incorporation into the Ba site of the perovskite structure. Since the ionic radius of Ba is 1.61 Å, i.e. larger than the ionic radius of any of the dopants used for this work, the hypothetical substitution of each of the selected dopants into the Ba site would result in a decrease of the lattice parameter. It is reasonable to assume a partial substitution of Gd or Sm cations on the Ba sites, which would

DOI: 10.1021/acs.jpcc.7b02163
J. Phys. Chem. C 2017, 121, 9739−9747

lead to the formation of BaO. As a consequence, oxygen vacancies are consumed instead of being created. This hypothesis is indeed consistent with several experimental and theoretical studies reported in the literature.[7,14,38−42] Haile et al.[39−41] suggested that, particularly for barium cerates, smaller dopant ions are preferentially incorporated into the B site whereas larger ions are incorporated into the A site. In fact, doped barium zirconate remains as a single phase even under 4−10 mol % of Ba deficiency, while undoped perovskites cannot accommodate any alkaline earth deficiencies. Considering the previous observations, the present results for 20 mo % - doped $BaZrO_3$ suggest that for dopant ionic radii larger than Gd a partial substitution of the dopant on the Ba site can occur.

In the specific case of Gd-doped BZ, Gd on Ba site would be in an 8-fold coordination, with an ionic radius of 1.06 Å, thus 34% smaller than barium (here in 12-fold coordination); while in the case of Gd sitting on the Zr site the ionic radius of Gd is 23% larger than that of Zr (both are considered in 6-fold coordination). It is therefore reasonable to assume that a slight substitution of Gd on the Ba site takes place.

It is noteworthy that the difference in lattice parameter of Nd-BZ before and after sintering is significantly larger compared to those measured for all other compositions. This could also be due to the fact that Nd can be stabilized in the oxidation state +4 in $BaZrO_3$, without altering the cubic structure, after heating treatment at high temperature.[43] The lattice parameter here calculated is indeed very close to the value reported on Nd 20% -BZ, where up to the 25% of dopant is oxidized to Nd^{4+}. The smaller ionic radius of Nd^{4+} could favor a redistribution of dopant on the Zr site.[43] In this case, the deriving point defect does not acquire any net charge ($Nd_{Zr}^{\times}$) and no oxygen vacancies are created, thus sensibly reducing the concentration of protonic defects.[44]

Sinterability. Figure 3 reports the XRD patterns of BZ-M pellets sintered at 1600 °C for 8 h. The BZ-Al XRD pattern (Figure 3a) shows that after the high temperature treatment clearly distinguishable diffraction peaks belonging to a secondary phase are visible.

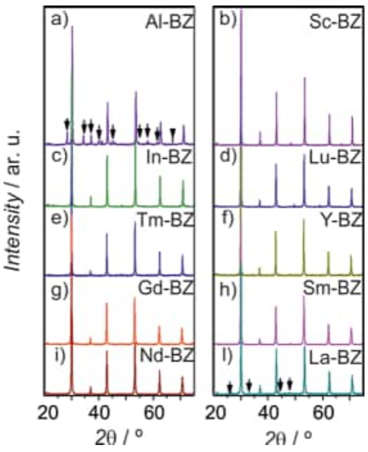

Figure 3. (a−j) XRD patterns of BZ-M pellets sintered at 1600 °C for 8 h. Peaks belonging to secondary phases are indicated by arrows.

The sample is composed by two phases: doped $BaZrO_3$ and $Ba_2Al_2O_4$. The fact that these peaks are visible only after sintering suggests the formation of the secondary phase during the heating treatment; $BaAl_2O_4$ can in fact at high temperature by the reaction of Al_2O_3 and $BaCO_3$.[45]

On the other hand, the XRD pattern of BZ-Nd after sintering indicates a single phase perovskite, while before the heating treatment it showed also second phases. This suggests that the second phase was incorporated into the perovskite structure during the heating treatment.

Also, in the XRD pattern of the BZ-La pellet the relative intensity of the peaks related to secondary phases decreases compared to powders (Figure 3 j). These findings suggest that the high temperature sintering facilitates the incorporation into the $BaZrO_3$ lattice of the cations with large ionic radii such as Nd and La.

As reported in Figure 2, no significant differences were found between the powder and pellet lattice parameters for the same composition with the exceptions of Nd- and La-doped barium zirconate. BZ-Nd and BZ-La pellets showed larger lattice parameters than the respective powders; this suggests larger incorporation of the dopant into the lattice after the high temperature treatment, in agreement with the XRD patterns.

Figure 4 shows the temperature dependence of the normalized linear shrinkage (defined as $\Delta l/l_0$, ratio between the sample length variation on the original length), for BZ-M green (not sintered) pellets. The shrinkage of Sm-, Nd-, and La-doped barium zirconate started at about 1000 °C, which is around 300 °C lower than that of the shrinkage onset for all the other dopants. At 1600 °C, the maximum test temperature, the final shrinkage for BZ-Sm, BZ-Nd, and BZ-La was about 17.5%, 20%, and 32%, respectively, while for all other compositions it ranges between 13% for BZ-In to 9.5% for BZ-Lu, which showed the lowest sinterability. These results indicate that doping at 20 mol % with different cations can strongly influence the sinterability of barium zirconate depending on the selected dopant cation.

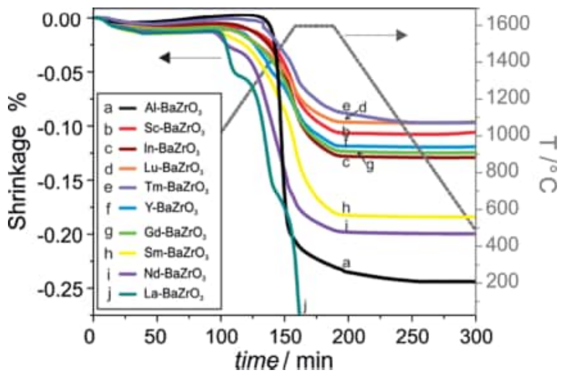

Figure 4. Temperature dependence of the normalized linear shrinkage for not sintered BZ-M pellets.

The influence of the dopant on the sintering properties of barium zirconate was also investigated performing dilatometric measurements and SEM analysis to probe the linear shrinkage upon sintering and the morphology of the sintered pellets.

Scanning electron microscopy (SEM) analysis was used to investigate the microstructure of the BZ-M sintered pellets. Figure 5 shows the SEM images of the BZ-M pellet fractured surfaces after sintering in air at 1600 °C for 8 h. As expected from the dilatometric measurements the good sinterability of Sm-, Nd-, and La-doped barium zirconate compounds was confirmed. The sintered pellets showed dense microstructures, with average grain sizes of 4, 5, and 8 μm, respectively, and well crystallized, polyhedral grains (Figure 5). Among the other compositions, Gd- and In-doped $BaZrO_3$ showed a relatively

DOI: 10.1021/acs.jpcc.7b02163
J. Phys. Chem. C 2017, 121, 9739−9747

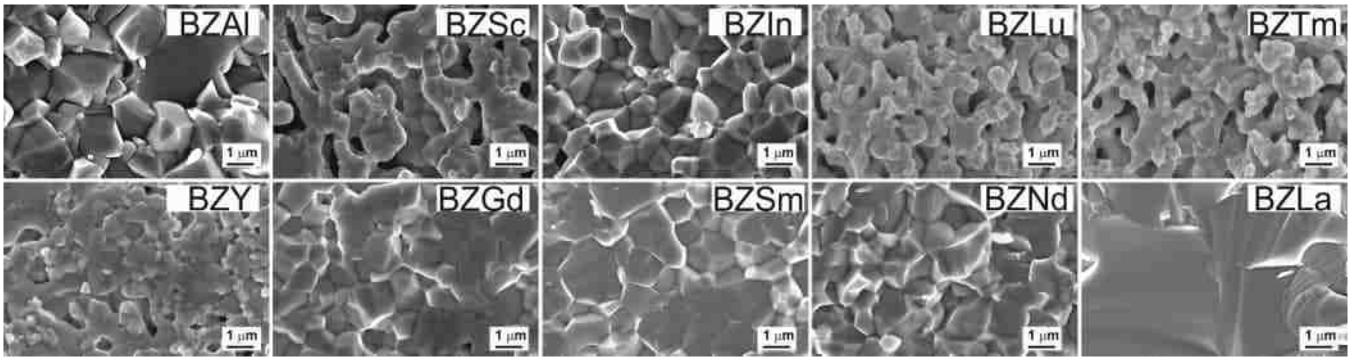

Figure 5. SEM micrographs of BZ-M pellets sintered at 1600 °C for 8 h.

dense microstructure, with an average grain size of 1 μm. BZ-Y showed a more porous microstructure with smaller grain size, and a further decrease of density and grain size was observed for Lu, Sc, and Tm dopants.

The better sinterability of barium zirconate doped with large cations such as Sm, Nd, and La can be explained considering that if the large dopants substitute Ba in the perovskite structure, the segregated barium oxide might form a liquid phase, which would be able to promote the grain growth of barium zirconate during the sintering, as reported for similar compositions.[33] Indeed, as can be seen from Figure 5 in the samples doped with Sm, Nd, and La, no sharp grain boundaries are present, which suggest a different grain boundary composition compared to samples doped with the other cations in the series.

Chemical Composition. The elemental composition for the BZ-M powders and pellets was investigated by inductively coupled plasma analysis. Figure 6 shows the Ba content as a function of the ionic radii of the dopants, for both powders and pellets. The Ba content in the case of the powders was close to the theoretical value for all the dopants with ionic radius smaller than Sm, while for Sm, Nd, and La, the measured Ba content was smaller than the theoretical value, which supports the hypothesis of dopant incorporation into the Ba site, with consequent Ba losses.

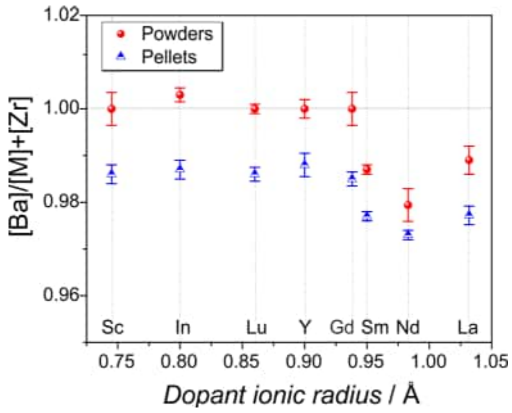

Figure 6. Barium content as a function of the dopant ionic radius in powders (circles) and pellets (triangles) BZ-M.

In the pellets, the measured Ba concentration was lower than in the respective powders, regardless of the ionic radius of the dopant, due to the unavoidable Ba loss that occurs during the high temperature sintering process.

Chemical analysis (not shown here) confirmed also that the dopant content was close to the nominal value for all samples. Only In- and La-doped sintered samples showed a dopant content smaller than the nominal value (0.16 and 0.17, respectively).

Electrical Conductivity. Figure 7a reports the Arrhenius plots of the BZ-M bulk conductivity in humidified atmosphere. The highest conductivity values were observed for Y-, Gd-, Tm-, and Lu-doped barium zirconate pellets. The bulk conductivity values measured for BZ-Y (3×10^{-5} S cm^{-1} at 100 °C) are comparable to literature data.[33,46] For sake of comparison, Figure 7a also reports the bulk conductivity of undoped barium zirconate. Only La- and Al-doped barium zirconate showed conductivity values lower than the undoped barium zirconate, as revealed by the XRD analysis shown in Figure 3, parts a and j; both contain secondary phases. This suggests that the secondary phases hinder the proton migration.

Figure 7b shows the Arrhenius plot of the specific grain boundary conductivities in humidified argon for BZ-M sintered pellets, which as expected are much lower (3 orders of magnitude at 350 °C) than the bulk conductivity for all compositions. The specific grain boundary conductivity of the compositions studied showed a maximum variation of 2 orders of magnitude in the temperature range studied, which indicates that this property is less affected by the choice of the dopant compared to the bulk migration process.

The Arrhenius plot of the total conductivity, which is of critical importance for practical applications, is shown in Figure 7c. This property depends not only on the conductivity of the material but also on morphological properties such as the sinterability and grain dimensions.

To better understand the influence of the dopant on the ionic transport, the bulk conductivity at 110 °C and the activation energy of the bulk conductivity are plotted as a function of the dopant ionic radius in Figure 8a, excluding here La and Al as dopants. It is noteworthy that the choice of the dopant can change the conductivity in a range of 4 orders of magnitude. The highest values of bulk conductivity have been measured for yttrium- and thulium-doped barium zirconate. Similar bulk conductivities were also observed for Gd and Lu as dopants, which indeed have an ionic radius very close to Y and Tm. Bulk conductivities about 1 order of magnitude smaller were observed for the smaller dopants, i.e., Sc and In, while a drastic drop was observed for the larger cations (Sm and Nd). Also the activation energy for the bulk migration process showed a dependence on the dopant ionic radii, achieving the lowest values for dopants with ionic radius between Lu and Gd

DOI: 10.1021/acs.jpcc.7b02163
J. Phys. Chem. C 2017, 121, 9739−9747

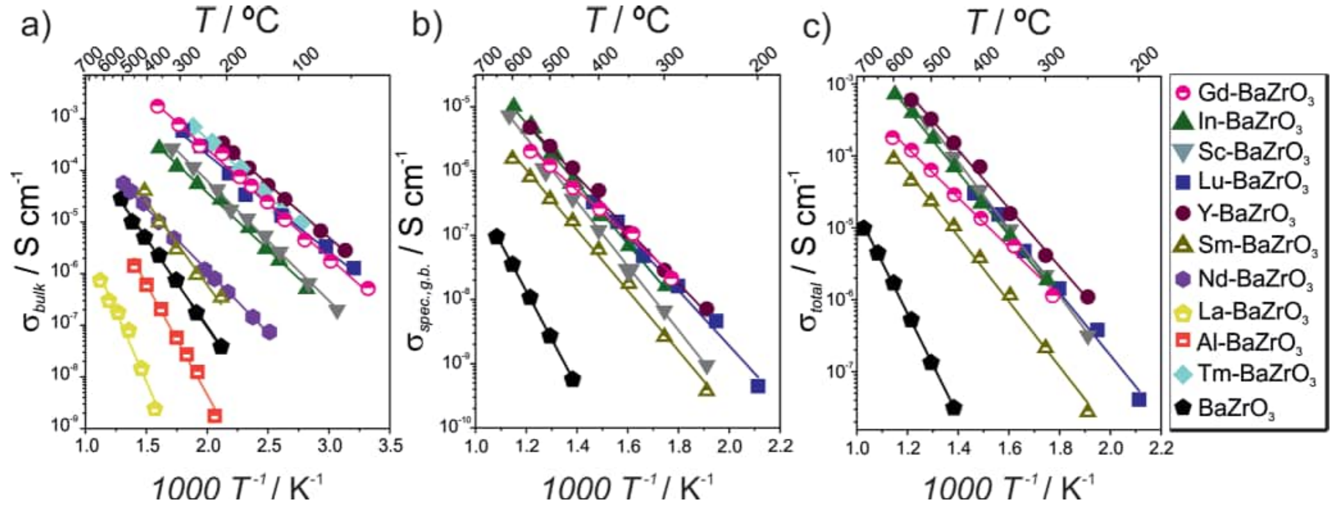

Figure 7. Temperature dependence of the conductivity in humidified (3% H_2O) Ar of BZ-M with different dopants: (a) bulk conductivity, (b) specific grain boundary conductivity, and (c) total conductivity.

(from 0.40 to 0.45 eV), and the highest values of 0.67 eV for Nd.

Our experimental results indeed confirm recent theoretical works reported in the literature where the effect of the dopant ionic size on the mobility and stability of protonic defects in different perovskites (zirconates, stannates, cerates) was studied by first principle density functional theory calculations.[47−49] These studies pointed out that the energy landscape felt by the proton is strongly affected by the ionic radius of the dopant: for small dopant ionic radii the proton is mainly stabilized when bonded to an oxygen first neighbor of the dopant, while for large ionic radii the most stable position is bonded to an oxygen second neighbor of the dopant. Consistently the stability of the proton in the first neighbor position decreases increasing the dopant ionic radii, while the stability in the second neighbor position follows the opposite trend. The total association energy of the proton with the dopant results therefore from the difference between the stabilization energy in the two different positions.

In the aforementioned studies similar dopants, as those used in the present work, were considered: Ga, Sc, In, Y, and Gd, together with Er for the cerates[49] and La for the stannates.[48] The value of the optimal dopant ionic radius depends on the size of the host cation (Zr, Sn and Ce), nevertheless in all cases the trapping energy reaches a minimum for dopants with radius significantly larger than the host cation, generally close to Y and Gd. This means that doping with cations with ionic radius close to these two elements leads to the lowest proton trapping, while for larger or smaller radii the proton trapping increases.

Stokes et al.,[50] using a different computational approach suggested a similar trend of the trapping energy as a function of the ionic radius, reaching a minimum value for Gd, however they considered the proton bonded only to the oxygen first neighbor of the dopant.

The effect of the proton-dopant association on the activation energy has been computed for $BaZrO_3$ by Björketun et al.,[47] the values estimated are in agreement with those reported in the present work considering the experimental uncertainty, especially for dopant ionic radii close to Y. In particular their study suggests that the minimum in the proton-dopant association energy for doped barium zirconates occurs for Y dopant.

In the present work the minimum in the activation energy corresponds to Lu as dopant, while in the results reported by Han et al.[29] corresponds to Tm-doped barium zirconate. This suggests that the minimum in the trapping energy in doped barium zirconates occurs for dopant with ionic radii close to that of Y, but slightly smaller. Trapping energies and activation energies for these values of dopant ionic radii have indeed not been calculated.

Even though the effect on the conductivity has not been computed, it is reasonable to expect a trend opposite to that of the trapping energy: dopants with lower proton-dopant association energy should lead to higher conductivity. The conductivity therefore is expected to increase with increasing the dopant ionic radius toward Y and to decrease for larger dopants, in agreement with our experimental results.

Additionally for Sm and Nd the large ionic radius leads to a partial substitution on the Ba site which decreases the total concentration of oxygen vacancies resulting in a lower incorporation of charge carriers.

As mentioned before, also for BZ-Gd, it is reasonable to expect a certain degree of substitution on the Ba site, which is supported also by the analysis of the lattice parameter from XRD. In this case the enhancement of the conductivity due to the low association between proton and dopant would be reduced by the effect of the Gd substitution on the Ba site, which would decrease the concentration of charge carriers.

As shown in Figure 8b, the grain boundary conductivities are not significantly influenced by the ionic radii of the dopant, and only slightly decrease for dopant ionic radius larger than Y. The activation energies are higher than the respective bulk activation energies (Figure 8a) and in agreement with values reported in literature for comparable doping levels.[6,37,51]

The total conductivity is also not strongly affected by the ionic radius of the dopant as reported in Figure 8c; it slightly decreases for dopants with ionic radii larger than Lu, even though the values do not vary more than 1 order of magnitude. The activation energies show as well a small variation increasing the dopant ionic radius.

From the comparison of the different properties investigated in this study Gd-BZ appears particularly promising for practical application as proton conducting electrolyte in fuel cells. Indeed it shows an high bulk conductivity, originating from a

DOI: 10.1021/acs.jpcc.7b02163
J. Phys. Chem. C 2017, 121, 9739−9747

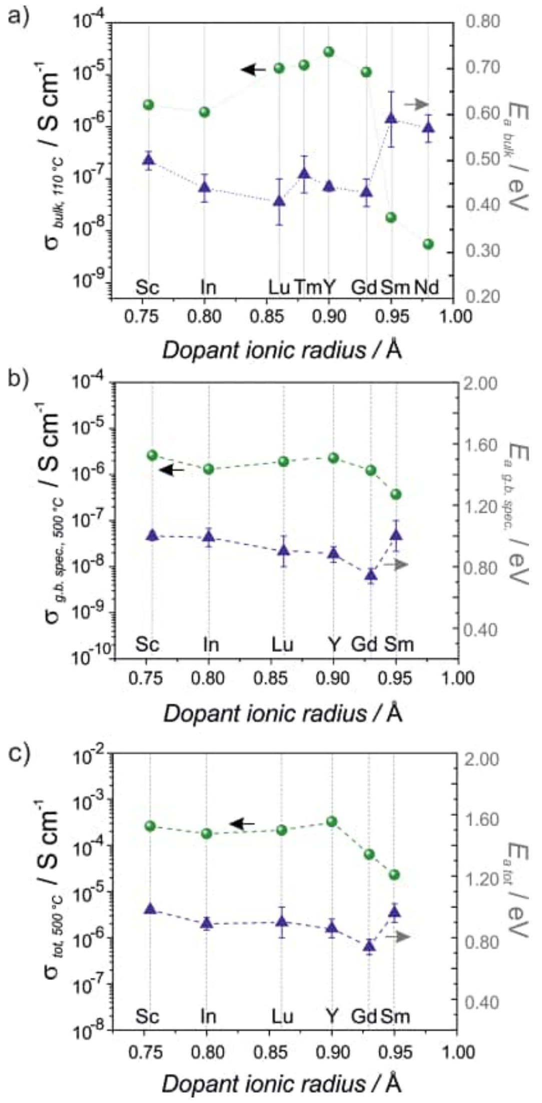

Figure 8. Conductivities and activation energies measured in humidified 3% vol H_2O − Ar of BZ-M doped with different cations. (a) Bulk conductivity at 110 °C and activation energies. (b) Grain boundaries specific conductivity at 500 °C and activation energies. (c) Total conductivity at 500 °C and activation energies. Error bars reported indicate the 95% confidence intervals.

weak trapping effect of Gd on the protons and a grain boundary conductivity which is comparable to the values measured for other dopants. The total conductivity is comparable to the conductivities of barium zirconate doped with other acceptor dopants (In, Sc, Lu), while it has a significantly lower activation energy. Furthermore, Gd-doped barium zirconate reveals better morphological properties since the good sinterability results in the formation of dense samples with large grains. The comprehensive comparison of several materials properties reported in the present study suggests that Gd-BZ may deserve further investigation for the optimization of the doping content and the sintering procedure. The better sinterability of Gd-BZ compared to other doped BZ with similar conductivity, can favor its use as gastight electrolyte in SOFCs. Moreover, computational results indicated Gd as one of the dopants with the smallest trapping effect,[47,49] which further suggests that Gd-BZ could improve its performance upon optimization of the synthesis procedure, sintering and dopant content.

■ CONCLUSIONS

In this study the effect of different dopants, i.e., Al, Sc, In, Lu, Tm, Y, Gd, Sm, Nd, and La on the structural, sintering and electrical properties of 20 mol % doped $BaZrO_3$ was investigated.

Overall we observed that the sinterability improves varying the dopant cationic radii from Sc to Nd and also the shrinkage increases sensibly increasing the ionic radii of the dopants. The highest shrinkages of doped barium zirconate were measured for Sm, Nd, and La as dopants. These form ceramic pellets with dense microstructure and average grain sizes between 4 and 8 μm. A linear relation between the dopant ionic radius and the lattice parameter of the compound was found for the elements between Sc and Y, while dopants with ionic radii larger than Gd showed the tendency to substitute Ba instead of Zr.

Lu-, Tm-, Y-, and Gd-doped barium zirconate exhibit the highest bulk conductivity and the lowest activation energy among the studied compositions, in agreement with computational studies on doped barium zirconate and similar perovskites. Dopants with ionic radii larger than Gd show much lower conductivity, due to their partial substitution on the Ba site.

It is worth noticing that mainly the bulk conductivity is affected by the dopant ionic radii, while only a slight influence was observed on the grain boundary conductivity.

Overall, the present study illustrates the critical role of the dopant element in modifying the stability, sinterability, and proton conductivity of barium zirconate. Our investigations show that high bulk conductivity can be obtained for a variety of dopants, i.e., Tm, Lu, Y, and Gd. When the sinterability and grain boundary density are taken into account, the best dopant appears to be Gd.

Yttrium-doped barium zirconate and thulium-doped barium zirconate showed the highest proton conductivity among the studied composition; in addition Gd also appears as a promising dopant that brings to both high ionic conductivity and good morphological properties.

Indeed, the good sinterability of Gd-BZO allows producing dense samples with relatively large grain size, minimizing the total grain boundary conductivity without hampering the bulk conductivity. The result is a doped BZO with good morphological properties (dense electrolytes are needed in fuel cells to avoid gas crossover) and high total conductivity. Therefore, on the basis of our methodological investigations we proposed Gd-BZO as a novel potential proton conducting electrolyte for low temperature SOFC.

■ AUTHOR INFORMATION

Corresponding Authors

*(D.P.) Telephone: +41 56 310 42 67. E-mail: daniele.pergolesi@psi.ch.
*(E.T.) E-mail: traversa@uniroma2.it. Telephone: +39 06 7259 4492.

ORCID

Elisa Gilardi: 0000-0002-8831-0104

Author Contributions

The manuscript was written through contributions of all authors. All authors have given approval to the final version of the manuscript.

Notes

The authors declare no competing financial interest.

DOI: 10.1021/acs.jpcc.7b02163
J. Phys. Chem. C 2017, 121, 9739−9747

■ ACKNOWLEDGMENTS

This research was supported by the NCCR MARVEL, funded by the Swiss National Science Foundation.

■ REFERENCES

(1) Malavasi, L.; Fisher, C. A. J.; Islam, M. S. Oxide-Ion and Proton Conducting Electrolyte Materials for Clean Energy Applications: Structural and Mechanistic Features. *Chem. Soc. Rev.* **2010**, *39*, 4370−4387.
(2) Shi, Y. U.; Bork, A. H.; Schweiger, S.; Rupp, J. L. M. The Effect of Mechanical Twisting on Oxygen Ionic Transport in Solid-State Energy Conversion Membranes. *Nat. Mater.* **2015**, *14*, 721−728.
(3) Evans, A.; Bieberle-Hutter, A.; Rupp, J. L. M.; Gauckler, L. J. Review on Microfabricated Micro-Solid Oxide Fuel Cell Membranes. *J. Power Sources* **2009**, *194*, 119−129.
(4) Iwahara, H.; Yajima, T.; Hibino, T.; Ushida, H. Performace of Solid Oxide Fuel-Cell Using Proton and Oxide-Ion Mixed Conductors Based on $BaCe_{1-x}Sm_xO_{3-\alpha}$. *J. Electrochem. Soc.* **1993**, *140*, 1687−1691.
(5) Pergolesi, D.; Fabbri, E.; D'Epifanio, A.; Di Bartolomeo, E.; Tebano, A.; Sanna, S.; Licoccia, S.; Balestrino, G.; Traversa, E. High Proton Conduction in Grain-Boundary-Free Yttrium-Doped Barium Zirconate Films Grown by Pulsed Laser Deposition. *Nat. Mater.* **2010**, *9*, 846−852.
(6) Yamazaki, Y.; Hernandez-Sanchez, R.; Haile, S. M. High Total Proton Conductivity in Large-Grained Yttrium-Doped Barium Zirconate. *Chem. Mater.* **2009**, *21*, 2755−2762.
(7) Kreuer, K. D. Proton-Conducting Oxides. *Annu. Rev. Mater. Res.* **2003**, *33*, 333−359.
(8) Fabbri, E.; Pergolesi, D.; Traversa, E. Materials Challenges Toward Proton-Conducting Oxide Fuel Cells: A Critical Review. *Chem. Soc. Rev.* **2010**, *39*, 4355−4369.
(9) Fabbri, E.; Pergolesi, D.; Licoccia, S.; Traversa, E. Does the Increase in Y-Dopant Concentration Improve the Proton Conductivity of $BaZr_{1-x}Y_xO_{3-\delta}$ Fuel Cell Electrolytes? *Solid State Ionics* **2010**, *181*, 1043−1051.
(10) Messerschmitt, F.; Kubicek, M.; Rupp, J. L. M. How Does Moisture Affect the Physical Property of Memristance for Anionic-Electronic Resistive Switching Memories? *Adv. Funct. Mater.* **2015**, *25*, 5117−5125.
(11) Nowick, A. S.; Du, Y. High-Temperature Protonic Conductors with Perovskite-Related Structures. *Solid State Ionics* **1995**, *77*, 137−146.
(12) Fabbri, E.; Bi, L.; Pergolesi, D.; Traversa, E. Towards the Next Generation of Solid Oxide Fuel Cells Operating Below 600 °C with Chemically Stable Proton-Conducting Electrolytes. *Adv. Mater.* **2012**, *24*, 195−208.
(13) Fabbri, E.; Pergolesi, D.; Licoccia, S.; Traversa, E. Does the Increase in Y-Dopant Concentration Improve the Proton Conductivity of $BaZr_{1-x}Y_xO_{3-\delta}$ Fuel Cell Electrolytes? *Solid State Ionics* **2010**, *181*, 1043−1051.
(14) Yamazaki, Y.; Hernandez-Sanchez, R.; Haile, S. M. Cation Non-Stoichiometry in Yttrium-Doped Barium Zirconate: Phase Behavior, Microstructure and Proton Conductivity. *J. Mater. Chem.* **2010**, *20*, 8158−8166.
(15) Yamazaki, Y.; Yang, C. K.; Haile, S. M. Unraveling the Defect Chemistry and Proton Uptake of Yttrium-Doped Barium Zirconate. *Scr. Mater.* **2011**, *65*, 102−107.
(16) Norby, T.; Widerøe, M.; Glockner, R.; Larring, Y. Hydrogen in Oxides. *Dalton Trans.* **2004**, 3012−3018.
(17) Giannici, F.; Longo, A.; Balerna, A.; Kreuer, K. D.; Martorana, A. Proton Dynamics in In:$BaZrO_3$: Insights on the Atomic and Electronic Structure from X-ray Absorption Spectroscopy. *Chem. Mater.* **2009**, *21*, 2641−2649.
(18) Karlsson, M.; Matic, A.; Knee, C. S.; Ahmed, I.; Eriksson, S. G.; Borjesson, L. Short-Range Structure of Proton-Conducting Perovskite $BaIn_xZr_{1-x}O_{3-x/2}$ (x = 0−0.75). *Chem. Mater.* **2008**, *20*, 3480−3486.
(19) Giannici, F.; Longo, A.; Kreuer, K. D.; Balerna, A.; Martorana, A. Dopants and Defects: Local Structure and Dynamics in Barium Cerates and Zirconates. *Solid State Ionics* **2010**, *181*, 122−125.
(20) Chen, Q. L.; Braun, A.; Ovalle, A.; Savaniu, C. D.; Graule, T.; Bagdassarov, N. Hydrostatic Pressure Decreases the Proton Mobility in the Hydrated $BaZr_{0.9}Y_{0.1}O_3$ Proton Conductor. *Appl. Phys. Lett.* **2010**, *97*, 041902.
(21) Chen, Q. L.; Braun, A.; Yoon, S.; Bagdassarov, N.; Graule, T. Effect of Lattice Volume and Compressive Strain on the Conductivity of BaCeY-oxide Ceramic Proton Conductors. *J. Eur. Ceram. Soc.* **2011**, *31*, 2657−2661.
(22) van Duin, A. C. T.; Merinov, B. V.; Han, S. S.; Dorso, C. O.; Goddard, W. A. ReaxFF Reactive Force Field for the Y-Doped $BaZrO_3$ Proton Conductor with Applications to Diffusion Rates for Multi-granular Systems. *J. Phys. Chem. A* **2008**, *112*, 11414−11422.
(23) Merinov, B.; Goddard, W. A. Proton Diffusion Pathways and Rates in Y-doped $BaZrO_3$ Solid Oxide Electrolyte from Quantum Mechanics. *J. Chem. Phys.* **2009**, *130*, 194707.
(24) Gomez, M. A.; Chunduru, M.; Chigweshe, L.; Foster, L.; Fensin, S. J.; Fletcher, K. M.; Fernandez, L. E. The Effect of Yttrium Dopant on the Proton Conduction Pathways of $BaZrO_3$, a Cubic Perovskite. *J. Chem. Phys.* **2010**, *132*, 214709.
(25) Samgin, A. L. Lattice-Assisted Proton Motion in Perovskite Oxides. *Solid State Ionics* **2000**, *136-137*, 291−295.
(26) Islam, M. S.; Davies, R. A.; Gale, J. D. Proton Migration and Defect Interactions in the $CaZrO_3$ Orthorhombic Perovskite: A Quantum Mechanical Study. *Chem. Mater.* **2001**, *13*, 2049−2055.
(27) Kreuer, K. D.; Adams, S.; Munch, W.; Fuchs, A.; Klock, U.; Maier, J. Proton Conducting Alkaline Earth Zirconates and Titanates for High Drain Electrochemical Applications. *Solid State Ionics* **2001**, *145*, 295−306.
(28) Han, D. L.; Hatada, N.; Uda, T. Chemical Expansion of Yttrium-Doped Barium Zirconate and Correlation with Proton Concentration and Conductivity. *J. Am. Ceram. Soc.* **2016**, *99*, 3745−3753.
(29) Han, D. L.; Shinoda, K.; Sato, S.; Majima, M.; Uda, T. Correlation Between Electroconductive and Structural Properties of Proton Conductive Acceptor-Doped Barium Zirconate. *J. Mater. Chem. A* **2015**, *3*, 1243−1250.
(30) Gorelov, V. P.; Balakireva, V. B.; Kleshchev, Y. N.; Brusentsov, V. P. Preparation and Electrical Conductivity of $BaZr_{1-x}R_xO_{3-\alpha}$ (R = Sc, Y, Ho, Dy, Gd, In). *Inorg. Mater.* **2001**, *37*, 535−538.
(31) Yamazaki, Y.; Blanc, F.; Okuyama, Y.; Buannic, L.; Lucio-Vega, J. C.; Grey, C. P.; Haile, S. M. Proton Trapping in Yttrium-Doped Barium Zirconate. *Nat. Mater.* **2013**, *12*, 647−651.
(32) Iwahara, H.; Yajima, T.; Hibino, T.; Ozaki, K.; Suzuki, H. Protonic Conduction in Calcium, Strontium and Barium Zirconates. *Solid State Ionics* **1993**, *61*, 65−69.
(33) Imashuku, S.; Uda, T.; Nose, Y.; Taniguchi, G.; Ito, Y.; Awakura, Y. Dependence of Dopant Cations on Microstructure and Proton Conductivity of Barium Zirconate. *J. Electrochem. Soc.* **2009**, *156*, B1−B8.
(34) Laidoudi, M.; Talib, I. A.; Omar, R. Investigation of the Bulk Conductivity of $BaZr_{0.95}M_{0.05}O_3$ (M = Al, Er, Ho, Tm, Yb and Y) Under Wet N_2. *J. Phys. D: Appl. Phys.* **2002**, *35*, 397−401.
(35) Han, D. L.; Hatada, N.; Uda, T. Microstructure, Proton Concentration and Proton Conductivity of Barium Zirconate Doped with Ho, Er, Tm and Yb. *J. Electrochem. Soc.* **2016**, *163*, F470−F476.
(36) Imashuku, S.; Uda, T.; Nose, Y.; Awakura, Y. Fabrication and Electrical Characterization of 15% Yttrium-Doped Barium Zirconate-Nitrate Freeze Drying Method Combined with Vacuum Heating. *J. Alloys Compd.* **2011**, *509*, 3872−3879.
(37) Chen, C.-T.; Danel, C. E.; Kim, S. On the Origin of the Blocking Effect of Grain-Boundaries on Proton Transport in Yttrium-Doped Barium Zirconates. *J. Mater. Chem.* **2011**, *21*, 5435−5442.
(38) Bonanos, N.; Knight, K. S.; Ellis, B. Perovskite Solid Electrolytes - Structure, Transport-Properties and Fuel-Cell Applications. *Solid State Ionics* **1995**, *79*, 161−170.

DOI: 10.1021/acs.jpcc.7b02163
J. Phys. Chem. C 2017, 121, 9739−9747

(39) Shima, D.; Haile, S. M. The Influence of Cation non-Stoichiometry on the Properties of Undoped and Gadolinia-Doped Barium Cerate. *Solid State Ionics* **1997**, *97*, 443−455.

(40) Haile, S. M.; Staneff, G.; Ryu, K. H. Non-Stoichiometry, Grain Boundary Transport and Chemical Stability of Proton Conducting Perovskites. *J. Mater. Sci.* **2001**, *36*, 1149−1160.

(41) Wu, J.; Li, L. P.; Espinosa, W. T. P.; Haile, S. T. Defect Chemistry and Transport Properties of $Ba_xCe_{0.85}M_{0.15}O_{3-\delta}$. *J. Mater. Res.* **2004**, *19*, 2366−2376.

(42) Islam, M. S.; Slater, P. R.; Tolchard, J. R.; Dinges, T. Doping and Defect Association in $AZrO_3$ (A = Ca, Ba) and $LaMO_3$ (M = Sc, Ga) Perovskite-Type Ionic Conductors. *Dalton Trans.* **2004**, 3061−3066.

(43) Brauer, G.; Kristen, H. Oxygen Perovskites with Tetravalent Neodymium. *Z. Anorg. Allg. Chem.* **1979**, *456*, 41−53.

(44) Oishi, M.; Yashiro, K.; Sato, K.; Mizusaki, J.; Kitamura, N.; Amezawa, K.; Kawada, T.; Uchimoto, Y. Oxygen nonstoichiometry of the perovskite-type oxides $BaCe_{0.9}M_{0.1}O_{3-\delta}$ (M = Y, Yb, Sm, Tb, and Nd). *Solid State Ionics* **2008**, *179*, 529−535.

(45) Periercamby, L.; Thomas, G. Ssolid Way Synthesis of Barium Aluminate. Kinetic Study of the Formation of Intermediate Compounds. *Solid State Ionics* **1993**, *63−65*, 128−135.

(46) Kreuer, K. D. On the Complexity of Proton Conduction Phenomena. *Solid State Ionics* **2000**, *136-137*, 149−160.

(47) Bjorketun, M. E.; Sundell, P. G.; Wahnstrom, G. Effect of Acceptor Dopants on the Proton Mobility in $BaZrO_3$: A Density Functional Investigation. *Phys. Rev. B: Condens. Matter Mater. Phys.* **2007**, *76*, 054307.

(48) Bevillon, E.; Hermet, J.; Dezanneau, G.; Geneste, G. How Dopant Size Influences the Protonic Energy Landscape in $BaSn_{1-x}M_xO_{3-x/2}$ (M = Ga, Sc, In, Y, Gd, La). *J. Mater. Chem. A* **2014**, *2*, 460−471.

(49) Loken, A.; Bjorheim, T. S.; Haugsrud, R. The Pivotal Role of the Dopant Choice on the Thermodynamics of Hydration and Associations in Proton Conducting $BaCe_{0.9}X_{0.1}O_{3-\delta}$ (X = Sc, Ga, Y, In, Gd and Er). *J. Mater. Chem. A* **2015**, *3*, 23289−23298.

(50) Stokes, S. J.; Islam, M. S. Defect Chemistry and Proton-Dopant Association in $BaZrO_3$ and $BaPrO_3$. *J. Mater. Chem.* **2010**, *20*, 6258−6264.

(51) Cervera, R. B.; Oyama, Y.; Miyoshi, S.; Oikawa, I.; Takamura, H.; Yamaguchi, S. Nanograined Sc-Doped $BaZrO_3$ as a Proton Conducting Solid Electrolyte for Intermediate Temperature Solid Oxide Fuel Cells (IT-SOFCs). *Solid State Ionics* **2014**, *264*, 1−6.

DOI: 10.1021/acs.jpcc.7b02163
J. Phys. Chem. C 2017, 121, 9739−9747

Published on 14 November 2014. Downloaded by NANJING TECH UNIVERSITY on 2/1/2025 6:48:47 AM.

Journal of Materials Chemistry A

PAPER

View Article Online
View Journal | View Issue

Cite this: J. Mater. Chem. A, 2015, 3, 1243

Correlation between electroconductive and structural properties of proton conductive acceptor-doped barium zirconate†

Donglin Han,*[a] Kozo Shinoda,[b] Shigeo Sato,[c] Masatoshi Majima[d] and Tetsuya Uda*[a]

Received 24th October 2014
Accepted 14th November 2014

DOI: 10.1039/c4ta05701e

www.rsc.org/MaterialsA

Various dopants were added to $BaZrO_3$ and the conductivities, the proton concentrations, the site occupancy of the dopants and the change in lattice volume as a result of chemical expansion were investigated. Lanthanide group dopants occupied both the Ba and Zr sites, but the amount of these dopants in the Ba site was too limited to significantly influence the conductivity. The samples doped with Yb, Tm, Er, Y and Ho showed both high proton concentrations and high conductivities, together with a relatively large lattice expansion as a result of hydration. We therefore suggest that, in most instances, the proton concentration, proton conductivity and lattice change as a result of chemical expansion were all correlated in proton conductive acceptor-doped $BaZrO_3$. However, Sc-doped $BaZrO_3$ seemed to be different. Its proton concentration was high, but the conductivity and lattice change as a result of chemical expansion were relatively small. This indicates that the conductivity was strongly related to the lattice expansion resulting from hydration rather than simply the proton concentration.

1. Introduction

Proton conductive barium zirconate ($BaZrO_3$), in which the tetravalent Zr host cations are partially substituted by trivalent dopants, is of increasing interest as a result of its promising electrochemical applications, including its use as an electrolyte in protonic ceramic fuel cells.[1,2] In general, Y is regarded as a good dopant because, on doping with 20 mol% Y ($BaZr_{0.8}Y_{0.2}O_{3-\delta}$), a proton conductivity >10^{-2} S cm^{-1} is generated at 600 °C in a humid environment.[3–5] Some lanthanide dopants, such as Ho and Yb, also impart high proton conductivities.[6,7] However, the optimum dopant for proton conductive $BaZrO_3$ has not yet been determined.[8]

Although trivalent dopants have been intentionally introduced to replace the Zr cations, they can also substitute for the divalent Ba cations.[5,9–12] Under such circumstances, oxide ion vacancies ($V_O^{\bullet\bullet}$) are consumed in a charge compensation mechanism. The occupation of Ba sites is therefore detrimental to the incorporation of protons in the form of hydroxide groups ($OH_O^{\bullet}$) through hydration processes (eqn (1)) in which the participation of oxide ion vacancies is necessary:[11–15]

$$H_2O + V_O^{\bullet\bullet} + O_O^{\times} \rightarrow 2OH_O^{\bullet} \quad (1)$$

The hydration process also has significant structural influences, such as a chemical expansion in the volume of the lattice as a result of the uptake of water.[10,14,16–19] As this chemical expansion generates compressional and tensional stresses during hydration and dehydration, respectively, the risk of mechanical failure in the electrolyte is increased.[20] We previously reported the co-existence of two perovskite phases in 20 mol% Y-doped $BaZrO_3$ as a result of differences in the degree of hydration.[9] This sample was stored in a desiccator for 2 years and then the X-ray diffraction (XRD) pattern was determined again. We found that the intensity of the highly hydrated phase increased significantly (Fig. 1), indicating that the hydration process still proceeds at room temperature in a controlled atmosphere with a low partial pressure of water vapor.

In the work reported here, we added various dopants into $BaZrO_3$ and carried out systematic investigations of the conductivity, water content, the site occupancies of the dopants and the chemical expansion due to hydration. The dependence of proton conduction on the dopants was determined and the correlation between these specific behaviors and properties clarified.

[a]Department of Materials Science and Engineering, Kyoto University, Yoshida Honmachi, Sakyo-ku, Kyoto 606-8501, Japan. E-mail: han.donglin.8n@kyoto-u.ac.jp; materials_process@aqua.mtl.kyoto-u.ac.jp; Fax: +81-75-753-5284; Tel: +81-75-753-5445

[b]Institute of Multidisciplinary Research for Advanced Materials, Tohoku University, Katahira 2-1-1, Aoba-ku, Sendai 980-8577, Japan

[c]Graduate School of Science and Engineering, Ibaraki University, 4-12-1 Nakanarusawa, Hitachi 316-8511, Japan

[d]Sumitomo Electric Industries, Ltd., 1-1-1, Koyakita, Itami-shi, Hyogo 664-0016, Japan

† Electronic supplementary information (ESI) available. See DOI: 10.1039/c4ta05701e

Published on 14 November 2014. Downloaded by NANJING TECH UNIVERSITY on 2/1/2025 6:48:47 AM.

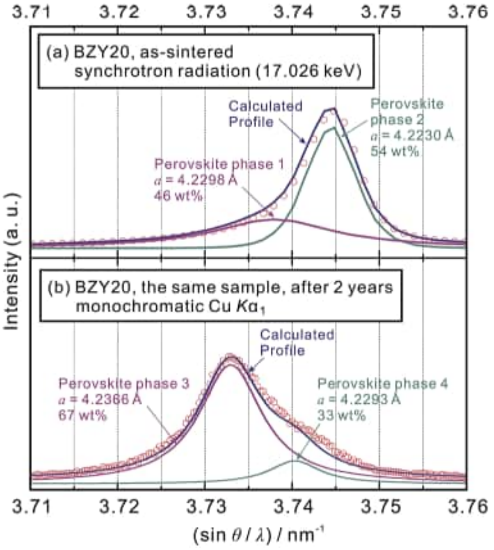

Fig. 1 Plots of 031 diffraction peaks of the $BaZr_{0.8}Y_{0.2}O_{3-\delta}$ (BZY20) samples (a) as-sintered at 1600 °C and subsequently quenched in ambient atmosphere[9] and (b) after keeping for 2 years in a desiccator, in which the partial pressure of water vapor was controlled to around 0.003 atm. As reported previously,[9] the XRD pattern of the as-sintered sample was collected by synchrotron radiation (17.026 eV) and was simulated by Rietveld refinement using a model containing two perovskite phases with lattice constants of 4.2230 Å (dehydrated phase, 54 wt%) and 4.2298 Å (hydrated phase, 46 wt%), respectively.[9] The XRD pattern of the same sample was collected after 2 years using a Cu Kα1 monochromatic X-ray source. Two perovskite phases were simulated to coexist and their lattice constants were 4.2293 Å (33 wt%) and 4.2366 Å (67 wt%).

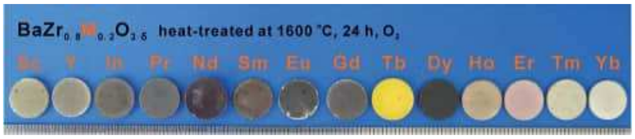

Fig. 2 Photograph of the pellets of $BaZr_{0.8}M_{0.2}O_{3-\delta}$ (M = Sc, Y, In, Pr, Nd, Sm, Eu, Gd, Tb, Dy, Ho, Er, Tm, Yb and In). All the samples were finally heat-treated at 1600 °C in O_2 for 24 h for sintering.

2. Experimental

2.1 Preparation of materials

Undoped $BaZrO_3$ and samples containing 20 mol% of the dopants [$BaZr_{0.8}M_{0.2}O_{3-\delta}$ (BZM20), M = Sc, Y, In, Pr, Nd, Sm, Eu, Gd, Tb, Dy, Ho, Er, Tm and Yb] were prepared by a conventional solid-state reaction method. The starting materials of $BaCO_3$, ZrO_2 and monoxides containing the relevant dopant elements (Sc_2O_3, Y_2O_3, In_2O_3, Pr_6O_{11}, Nd_2O_3, Sm_2O_3, Eu_2O_3, Gd_2O_3, Tb_4O_7, Dy_2O_3, Ho_2O_3, Er_2O_3, Tm_2O_3 and Yb_2O_3) were mixed at the desired ratios and ball-milled for 24 h. The mixtures were then pressed into pellets under 9.8 MPa and heat-treated at 1000 °C for 10 h. After ball-milling for another 10 h, the samples were again pressed into pellets under 9.8 MPa and heated at 1300 °C for 10 h for synthesizing. The samples were then ball-milled for 24 h and subsequently mixed with an organic binder solution consisting of water, polyvinyl alcohol, glycerin and ethanol. The samples were then pressed into pellets at 392 MPa and heat-treated at 600 °C for 8 h to remove the binder solution. Finally, after being buried in sacrificial powders [mixtures of the relevant synthesized BZM20 powders (99 wt%) and $BaCO_3$ (1 wt%)], the pellet-like samples were heated at 1600 °C for 24 h in an oxygen atmosphere for sintering and were finally quenched in an ambient atmosphere. Fig. 2 is a photograph of the as-sintered pellet-like samples.

2.2 Characterization

As the 20 mol% Nd-doped sample is not a single perovskite phase,[7] only the samples doped with Pr, Gd, Tb, Ho, Er, Tm, Yb and In were characterized in detail. The structure of the powder-like samples was examined by XRD under an ambient atmosphere in a Bragg–Brentano (θ–2θ) geometry with a D8 ADVANCE instrument (Bruker AXS GmbH, Karlsruhe, Germany) equipped with a Cu Kα radiation source. The incident radiation was monochromated to the Cu $K\alpha_1$ (0.15406 nm) line with a Johansson-type quartz-101 reflection monochromator. High-temperature XRD (HT-XRD) measurements were performed using Cu Kα radiation with an X'Pert-ProMPD instrument (PANalytical, Almelo, the Netherlands) equipped with an HTK 1200N high-temperature chamber (Anton Paar, Graz, Austria). Dry or wet ($p_{H_2O} = 0.0312$ atm) oxygen gas flowed into the high-temperature chamber. The HT-XRD patterns were collected during cooling from 1000 to 30 °C at an average cooling rate of about 1.06 °C min^{-1}. The detailed procedures for the HT-XRD measurements have been reported previously.[17,18] Rietveld refinement was carried out using the TOPAS commercial software (Bruker AXS GmbH). The chemical compositions of the samples were determined by inductively coupled plasma atomic emission spectrometry (ICP-AES) with an SPS4000 spectrometer (Seiko Instruments, Chiba, Japan).

The water content was determined by the Karl-Fischer titration method to give a direct evaluation of the proton concentration in the samples. The pellet-like samples heat-treated at 1600 °C for sintering were broken into pieces about 2 mm in length and hydrated at 300 °C in wet O_2 ($p_{H_2O} = 0.05$ atm) for 7 days. The detailed procedures have been published previously.[21,22]

All the samples were subjected to conductivity measurements in wet and dry H_2 atmospheres. The partial pressure of water in the wet atmosphere was kept at 0.05 atm. Palladium was electrolessly deposited as the electrodes. The impedance spectra were collected by AC impedance spectrometry in the frequency range 10 Hz to 7 MHz using a frequency response analyzer (Solartron SI 1260, Solartron Analytical, UK) with an applied voltage of 100 mV at temperatures from 600 to 100 °C.

3. Results

3.1 Chemical expansion

The HT-XRD measurements were obtained in both dry and wet O_2 ($p_{H_2O} = 0.0312$ atm) to observe the chemical expansion effect resulting from hydration. A single perovskite ($Pm\bar{3}m$) model was used to evaluate the lattice constant to reflect the average

 This journal is © The Royal Society of Chemistry 2015

Published on 14 November 2014. Downloaded by NANJING TECH UNIVERSITY on 2/1/2025 6:48:47 AM.

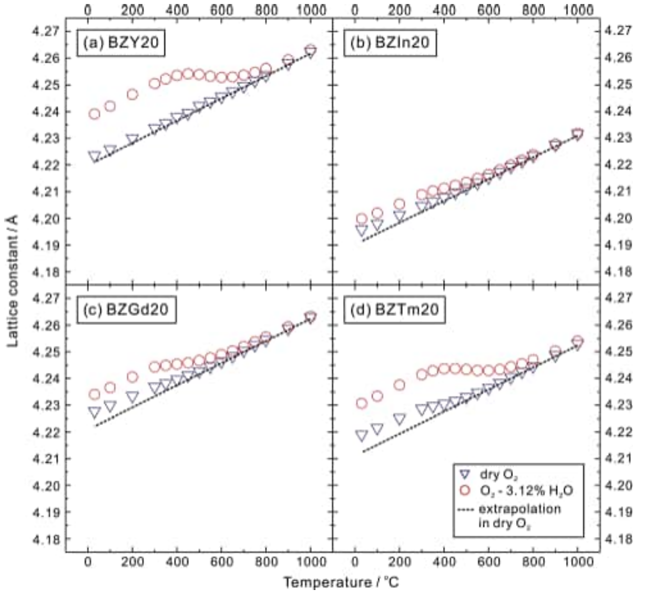

Fig. 3 Variation of lattice constants with temperature for (a) $BaZr_{0.8}Y_{0.2}O_{3-\delta}$ (BZY20), (b) $BaZr_{0.8}In_{0.2}O_{3-\delta}$ (BZIn20), (c) $BaZr_{0.8}Gd_{0.2}O_{3-\delta}$ (BZGd20) and (d) $BaZr_{0.8}Tm_{0.2}O_{3-\delta}$ (BZTm20) in dry O_2 and wet O_2 ($p_{H_2O} = 0.0312$ atm). All the samples were finally heat-treated at 1600 °C in O_2 for 24 h for sintering. The broken line shows the extrapolation of the data in the temperature range 700–1000 °C in dry O_2. Fig. S1† gives the results for the other dopants (Sc, Sm, Eu, Dy, Ho, Er and Yb); the same phenomenon was observed.

influence of the humidity on the crystal structure. As shown in Fig. 3, the lattice constants were obviously larger in the wet O_2 atmosphere than in the dry atmosphere for the samples doped with Y, In, Gd and Tm. This is because the substitution of the tetravalent Zr cations ($Zr_{Zr}^{\times}$) with trivalent dopant cations (M_{Zr}') generates oxide ion vacancies as a result of charge compensation [eqn (2)], which benefits the hydration reaction of eqn (1):

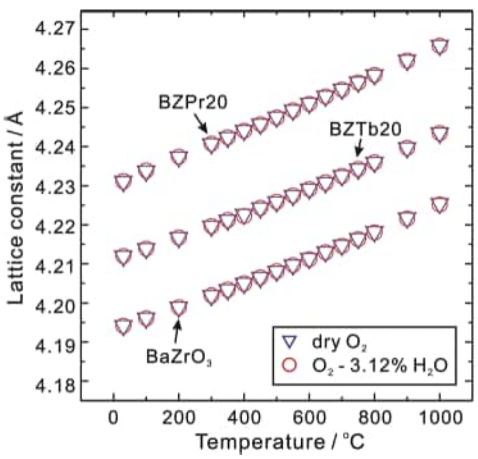

Fig. 4 Variation in lattice constants of undoped $BaZrO_3$, $BaZr_{0.8}Pr_{0.2}O_{3-\delta}$ (BZPr20) and $BaZr_{0.8}Tb_{0.2}O_{3-\delta}$ (BZTb20) in dry O_2 and wet O_2 ($p_{H_2O} = 0.0312$ atm) with temperature. All the samples were finally heat-treated at 1600 °C in O_2 for 24 h for sintering.

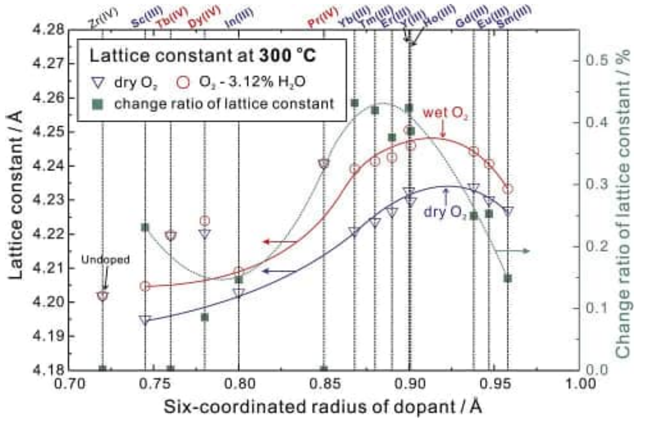

Fig. 5 Plot of the lattice constant of the samples of undoped $BaZrO_3$ and those doped with rare earth elements and In at 300 °C as determined from the HT-XRD measurements. The lattice constants in dry and wet O_2 atmospheres are marked using open triangles and open circles, respectively. The change ratio of the lattice constant is marked using closed square symbols. All the samples were sintered at 1600 °C in O_2 for 24 h before the HT-XRD measurements. In dry O_2, the values determined by extrapolation were used. The solid and broken lines are used to highlight the trend of variation in the lattice constants with increasing dopant radius for the samples doped with trivalent cations (Sc, In, Yb, Tm, Er, Y, Ho, Gd, Eu and Sm) and their change ratio. Fig. S2† shows the results obtained at 30 and 600 °C.

$$2Zr_{Zr}^{\times} + M_2O_3 + O_O^{\times} \rightarrow 2M_{Zr}' + 2ZrO_2 + V_O^{\cdot\cdot} \quad (2)$$

The lattice constants in dry O_2 in the low temperature range (30–400 °C) show a positive deviation from the predicted tendency obtained by extrapolating the data from the high-temperature range (700–1000 °C), shown as the broken line in Fig. 3. This indicates that the samples were slightly hydrated in

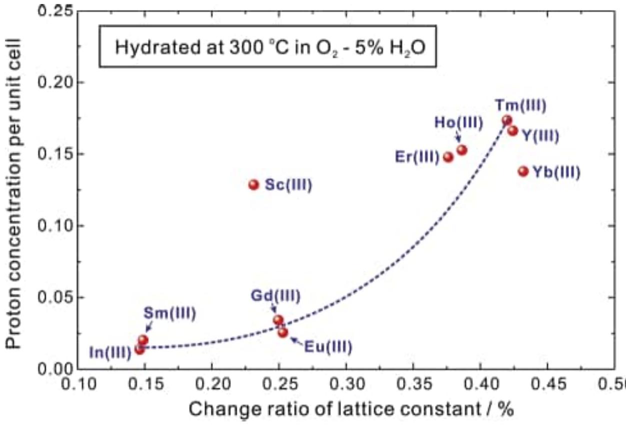

Fig. 6 Proton concentration of $Ba(Zr_{0.8}M_{0.2})O_{3-\delta}$ (M = Sc, Y, In, Sm, Eu, Gd, Ho, Er, Tm, Yb) at 300 °C plotted against the change ratio of the lattice constant. The change ratio of the lattice constant was determined by HT-XRD measurements performed in dry and wet ($p_{H_2O} = 0.0312$ atm) O_2. The proton concentration was determined by Karl-Fischer titration to measure the water content in the samples hydrated at 300 °C in O_2 − 5% H_2O. The data for samples doped with Sc, Y, Sm and Eu are cited from our previous work.[5]

Published on 14 November 2014. Downloaded by NANJING TECH UNIVERSITY on 2/1/2025 6:48:47 AM.

the low temperature range because it is difficult to remove water vapor entirely to produce an absolutely dry environment.

In contrast, Fig. 4 shows that the lattice constants of the Pr- and Tb-doped samples are very similar in both the wet and dry atmospheres. This is analogous to the results for undoped $BaZrO_3$. Pr and Tb are therefore highly likely to be mainly tetravalent in an O_2 atmosphere.

Fig. 5 summarizes the lattice constants at 300 °C in dry and wet O_2 atmospheres. The chemical expansion effect is quantitatively evaluated based on the change ratio of the lattice constant, defined as in eqn (3), in which $a_{\text{wet O}_2}$ and $a_{\text{dry O}_2}$ are the lattice constants in wet and dry O_2, respectively:

$$\text{Change ratio of lattice constant} = \frac{a_{\text{wet O}_2} - a_{\text{dry O}_2}}{a_{\text{dry O}_2}} \times 100\% \quad (3)$$

In Fig. 5, tetravalent radii were adopted for Zr (undoped $BaZrO_3$), Tb, Pr and also Dy (Dy is mainly tetravalent in $BaZrO_3$ in an O_2 atmosphere[10,23]), whereas trivalent radii were adopted for the other dopants.[24,25] For the dopants with radii less than that of Ho(III), the lattice constants for both the hydrated and dehydrated phases tended to increase with increasing radius of the dopant. However, when trivalent Gd, Eu and Sm, which are larger than Ho, were doped, the lattice constant decreased with increasing radius of the dopant. It is also interesting that the change ratio of the lattice constant was obviously higher (>0.37%) for the samples doped with Yb, Tm, Er, Y and Ho, indicating a stronger effect of chemical expansion as a result of the incorporation of water.

3.2 Proton concentration

Fig. 6 shows the proton concentration per unit cell plotted against the change ratio of the lattice constant for the samples doped with trivalent dopants. Apart from Sc, a fairly good correlation was seen between the proton concentration and the change ratio of the lattice constant. The proton concentrations are small for the In-,

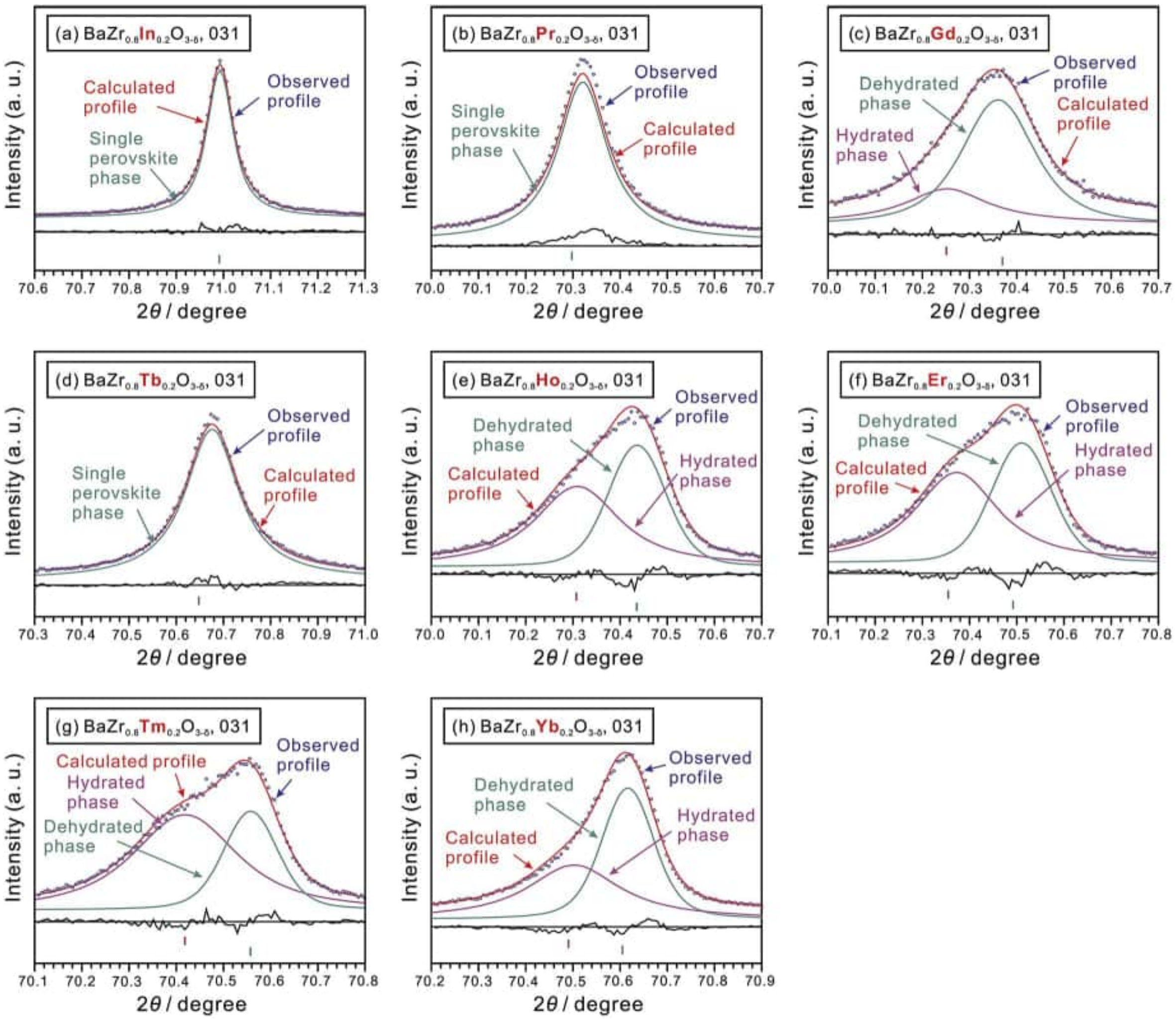

Fig. 7 Rietveld refinement of the 031 diffraction peaks of $BaZr_{0.8}M_{0.2}O_{3-\delta}$ (M = In, Pr, Gd, Tb, Ho, Er, Tm and Yb). The diffraction patterns were collected using a Cu Kα_1 monochromatic X-ray source. Before the XRD measurements, all the samples were sintered at 1600 °C in O_2 for 24 h and quenched in an ambient atmosphere. A cubic perovskite ($Pm\bar{3}m$) structure was used to simulate all the phases. The observed profile (blue circle), calculated profile (red line), difference (black line at bottom of plot) and the Bragg peaks of the candidate phases (vertical lines) are shown.

 This journal is © The Royal Society of Chemistry 2015

Published on 14 November 2014. Downloaded by NANJING TECH UNIVERSITY on 2/1/2025 6:48:47 AM.

Table 1 Actual composition of $BaZr_{0.8}M_{0.2}O_{3-\delta}$ (M = In, Pr, Gd, Tb, Ho, Er, Tm and Yb) determined by ICP-AES

Nominal composition	Actual composition by ICP-AES
$BaZr_{0.8}In_{0.2}O_{3-\delta}$	$Ba_{0.98}Zr_{0.83}In_{0.17}O_{3-\delta}$
$BaZr_{0.8}Pr_{0.2}O_{3-\delta}$	$Ba_{0.97}Zr_{0.82}Pr_{0.18}O_{3-\delta}$
$BaZr_{0.8}Gd_{0.2}O_{3-\delta}$	$Ba_{0.96}Zr_{0.80}Gd_{0.20}O_{3-\delta}$
$BaZr_{0.8}Tb_{0.2}O_{3-\delta}$	$Ba_{0.96}Zr_{0.82}Tb_{0.18}O_{3-\delta}$
$BaZr_{0.8}Ho_{0.2}O_{3-\delta}$	$Ba_{0.97}Zr_{0.81}Ho_{0.19}O_{3-\delta}$
$BaZr_{0.8}Er_{0.2}O_{3-\delta}$	$Ba_{0.97}Zr_{0.81}Er_{0.19}O_{3-\delta}$
$BaZr_{0.8}Tm_{0.2}O_{3-\delta}$	$Ba_{0.96}Zr_{0.81}Tm_{0.19}O_{3-\delta}$
$BaZr_{0.8}Yb_{0.2}O_{3-\delta}$	$Ba_{0.97}Zr_{0.81}Yb_{0.19}O_{3-\delta}$

Sm-, Gd- and Eu-doped samples, the lattice volumes of which only changed slightly on hydration. For the samples doped with Er, Ho, Tm, Y and Yb, both a relatively large change ratio of the lattice constant and a high proton concentration were confirmed. It is reasonable that the proton concentration is a very important factor, but it is probably not the only factor influencing the chemical expansion effect. The sample doped with Sc behaves differently; although its proton concentration was comparable with those of the samples doped with Er, Ho and Yb, the change ratio of the lattice volume was obviously smaller.

3.3 Site occupancy of dopants in $BaZrO_3$

Profiles of the 031 diffraction peaks of the samples doped with In, Pr, Gd, Tb, Ho, Er, Tm and Yb are shown in Fig. 7. The 031 peaks of the In-, Pr- and Tb-doped $BaZrO_3$ showed fairly good symmetry. In contrast, the peaks for the samples doped with Gd, Ho, Er, Tm and Yb were obviously asymmetrical as a result of partial hydration during quenching in the ambient atmosphere after sintering at 1600 °C, similar to the phenomenon seen in the system doped with Y, Sm and Eu.[9,10]

Rietveld refinement was performed to determine the site occupancy of the dopants. To fit the diffraction patterns of the Gd-, Ho-, Er-, Tm- and Yb-doped samples, two perovskite phases ($Pm\bar{3}m$) were used to simulate the hydrated and dehydrated phases, the whole compositions of which were treated to give the same value as determined by ICP-AES (Table 1). As the diffraction peaks show good symmetry for the samples doped with In, Pr and Tb, only one perovskite phase ($Pm\bar{3}m$) was used. For the samples doped with Pr, Gd, Tb, Ho, Er, Tm and Yb, which have a slight Ba deficiency (0.02–0.04), the dopants were determined to partially occupy the Ba site (Table 2). However, for the In-doped sample, which is also slightly deficient in Ba (0.02), all the In cations were determined to occupy the Zr site. By combining the results of our previous study on the site occupancy of Sc, Y, Sm, Eu and Dy in $BaZrO_3$,[9,10] Fig. 8 summarizes the content of the dopants at the Ba site. It is interesting that, in a nearly stoichiometric system with a slight Ba deficiency, the lanthanide elements (Pr, Nd, Sm, Eu, Gd, Tb, Dy, Ho, Er, Tm and Yb) partition over the Ba and Zr sites, whereas the non-lanthanide elements (Sc, Y and In) only occupy the Zr site.

Table 2 Rietveld refinement results of the diffraction patterns of $BaZr_{0.8}M_{0.2}O_{3-\delta}$ (M = In, Pr, Gd, Tb, Ho, Er, Tm and Yb) collected with a Cu Kα_1 monochromatic X-ray source. The structures of all the phases were assigned to be cubic ($Pm\bar{3}m$). All the samples were sintered at 1600 °C in O_2 for 24 h and finally quenched in an ambient atmosphere

						Site occupancy				Equivalent isotropic temperature factor ($Å^2$)	
						A site		B site			
Nominal sample composition	R_{wp} (%)	χ^2	Phase (composition)	Lattice constant (Å)	Fraction (wt%)	Ba	M	Zr	M	A-site	B-site
$BaZr_{0.8}In_{0.2}O_{3-\delta}$	6.11	1.12	$Ba_{0.98}Zr_{0.83}In_{0.17}O_{3-\delta}$	4.1965(2)	100.00	0.980(1)	0.000(1)	0.830(1)	0.170(1)	0.20(2)	0.20(2)
$BaZr_{0.8}Pr_{0.2}O_{3-\delta}$	9.53	2.12	$Ba_{0.97}Zr_{0.82}Pr_{0.18}O_{3-\delta}$	4.2312(3)	100.00	0.985(2)	0.015(2)	0.802(2)	0.198(2)	0.71(2)	0.20(2)
$BaZr_{0.8}Gd_{0.2}O_{3-\delta}$	5.74	1.08	Dehydrated phase ($Ba_{0.96}Zr_{0.80}Gd_{0.20}O_{3-\delta}$)	4.2291(3)	66.99	0.980(1)	0.020(1)	0.816(1)	0.184(1)	0.80(5)	0.20(6)
			Hydrated phase ($Ba_{0.96}Zr_{0.80}Gd_{0.20}O_{3-\delta}$)	4.2348(3)	33.01	0.980(1)	0.020(1)	0.816(1)	0.184(1)	0.80(2)	0.32(2)
$BaZr_{0.8}Tb_{0.2}O_{3-\delta}$	6.44	1.18	$Ba_{0.96}Zr_{0.82}Tb_{0.18}O_{3-\delta}$	4.2129(2)	100.00	0.972(2)	0.013(2)	0.830(2)	0.170(2)	0.40(3)	0.20(3)
$BaZr_{0.8}Ho_{0.2}O_{3-\delta}$	7.21	1.16	Dehydrated phase ($Ba_{0.97}Zr_{0.81}Ho_{0.19}O_{3-\delta}$)	4.2250(2)	43.46	0.979(2)	0.009(2)	0.817(1)	0.183(1)	0.80(5)	0.25(5)
			Hydrated phase ($Ba_{0.97}Zr_{0.81}Ho_{0.19}O_{3-\delta}$)	4.2316(2)	56.54	0.979(2)	0.009(2)	0.817(1)	0.183(1)	0.80(5)	0.42(5)
$BaZr_{0.8}Er_{0.2}O_{3-\delta}$	7.08	1.18	Dehydrated phase ($Ba_{0.97}Zr_{0.81}Er_{0.19}O_{3-\delta}$)	4.2210(3)	41.32	0.984(2)	0.014(2)	0.821(9)	0.179(9)	0.80(5)	0.20(5)
			Hydrated phase ($Ba_{0.97}Zr_{0.81}Er_{0.19}O_{3-\delta}$)	4.2283(3)	58.68	0.984(2)	0.014(2)	0.821(9)	0.179(9)	0.80(4)	0.31(4)
$BaZr_{0.8}Tm_{0.2}O_{3-\delta}$	7.07	1.13	Dehydrated phase ($Ba_{0.96}Zr_{0.81}Tm_{0.19}O_{3-\delta}$)	4.2177(3)	45.30	0.970(2)	0.011(2)	0.819(1)	0.181(1)	0.49(6)	0.20(6)
			Hydrated phase ($Ba_{0.96}Zr_{0.81}Tm_{0.19}O_{3-\delta}$)	4.2249(2)	54.70	0.970(2)	0.011(2)	0.819(1)	0.181(1)	0.800(4)	0.39(4)
$BaZr_{0.8}Yb_{0.2}O_{3-\delta}$	7.52	1.58	Dehydrated phase ($Ba_{0.97}Zr_{0.81}Yb_{0.19}O_{3-\delta}$)	4.2150(2)	49.57	0.975(2)	0.005(2)	0.814(2)	0.186(2)	0.50(4)	0.20(4)
			Hydrated phase ($Ba_{0.97}Zr_{0.81}Yb_{0.19}O_{3-\delta}$)	4.2206(2)	50.43	0.975(2)	0.005(2)	0.814(2)	0.186(2)	0.80(5)	0.80(5)

Published on 14 November 2014. Downloaded by NANJING TECH UNIVERSITY on 2/1/2025 6:48:47 AM.

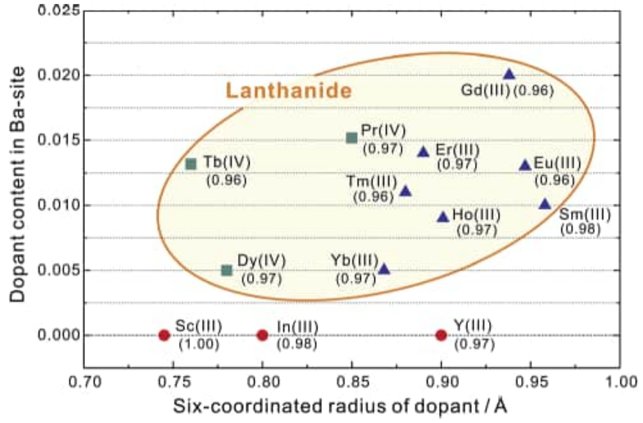

Fig. 8 Dopant content in Ba site determined by Rietveld refinement. The Ba deficiency for all the samples was <0.04. The content of Sc, Y, Sm, Eu and Dy in the Ba site is cited from our previous work.[9,10] The Ba content of the samples are given in parentheses. Although the radius of tetravalent Pr cations is used here, it is possible that Pr in the A-site of the perovskite structure is trivalent.[26]

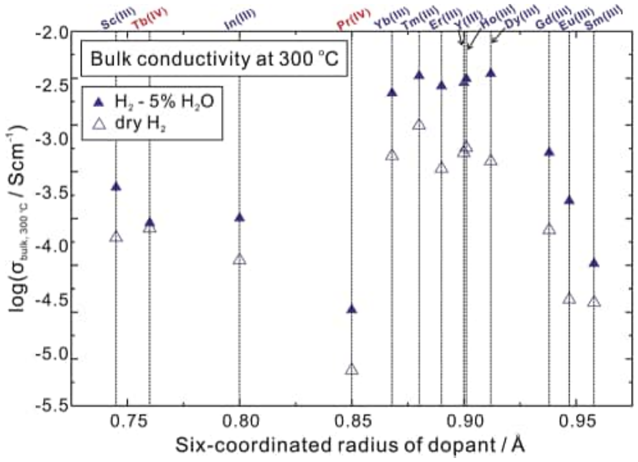

Fig. 9 Bulk conductivity of acceptor-doped $BaZrO_3$ at 300 °C in dry and wet atmospheres of H_2. The partial pressure of water was kept at 0.05 atm. All the samples were sintered at 1600 °C in O_2 for 24 h before the conductivity measurements.

3.4 Conductivity of doped $BaZrO_3$

The bulk (intra-grain) conductivities were determined by separating the contribution of bulk conduction in the AC impedance spectra[5,15] obtained in a relatively low temperature range because, at higher temperatures, only the contributions from grain boundary conduction and the electrode reaction are seen (an example for $BaZr_{0.8}Y_{0.2}O_{3-\delta}$ is given in the ESI†). The bulk conductivities of the samples doped with Sc, Y, In, Pr, Sm, Eu, Gd, Tb, Dy, Ho, Er, Tm and Yb were determined in the available temperature range and are given in the ESI.†

To facilitate the comparison, the bulk conductivities at 300 °C are plotted in Fig. 9. The radii of the tetravalent Tb and Pr cations are used. In addition, Dy is trivalent in $BaZrO_3$ in a hydrogen atmosphere.[23] Higher bulk conductivities were obtained in wet H_2 for the samples doped with trivalent cations, indicating the generation of proton conduction in a humidified environment. It is clear that the samples doped with trivalent Yb, Tm, Er, Y, Ho and Dy show relatively high conductivities. The conductivity decreases when comparably smaller (Sc and In) or larger dopants (Gd, Eu and Sm) are used. This tendency is generally in agreement with our previous study on the system containing 15 mol% dopants with the conductivity measured in a wet Ar atmosphere.[7] In addition, the conductivities of the Tb-doped samples change little between the measurements in dry and wet H_2, suggesting that the charge carriers of protons, and therefore the concentration of oxide ion vacancies for the hydration reaction in eqn (1), is fairly limited. Tb is therefore highly likely to be tetravalent, even in a reducing atmosphere. However, from the results of the HT-XRD measurements performed in an O_2 atmosphere (Fig. 4), Pr is considered to be mainly tetravalent, as no chemical expansion as a result of the incorporation of water was observed, although the conductivity measurements in H_2 clearly indicate that a humid reducing environment resulted in an increase in the bulk conductivity. It has been reported that tetravalent Pr tends to become partially trivalent with a decrease in the partial pressure of oxygen.[27] The concentration of oxide ion vacancies increases for charge compensation, which benefits the proton conduction.

The bulk conductivity was plotted against the proton concentration and the change ratio of the lattice constant (Fig. 10). In most instances the conductivity has an almost linear relationship with the proton concentration. However, as shown in Fig. 10a, the sample doped with Sc behaves very differently; although it has an obviously high proton concentration, its conductivity is very small. However, Fig. 10b shows a clear trend in the bulk conductivity, which generally increased with increasing change ratio of the lattice constant as a result of the incorporation of water.

3.5 Activation energy and pre-exponential factor

The activation energy (E_a) and the pre-exponential term (A) of bulk conduction were evaluated over the temperature range 100–250 °C based on eqn (4), where σ_{bulk} is the bulk conductivity, T is the temperature and k_B is Boltzmann's constant:

$$\sigma_{bulk}T = A\exp\left(\frac{-E_a}{k_BT}\right) \quad (4)$$

As shown in Fig. 11, for the samples doped with trivalent Yb, Tm, Er, Y and Ho, which have radii between 0.868 Å [Yb(III)] (ref. 21) and 0.901 Å [Ho(III)],[21] low activation energies between 0.40 and 0.49 eV were obtained (detailed values are given in the ESI†), which are typical values for proton conduction. The lowest activation energy was 0.40 eV for $BaZr_{0.8}Tm_{0.2}O_{3-\delta}$ in wet H_2. The activation energies increased to over 0.5 eV in wet H_2 when doping with the smaller cations of Sc and In. When the larger cations of Gd, Eu and Sm were doped, the activation energy increased monotonically with increasing radius of the dopant. In addition, the pre-exponential term tended to increase with increasing radius of the dopant.

This journal is © The Royal Society of Chemistry 2015

Published on 14 November 2014. Downloaded by NANJING TECH UNIVERSITY on 2/1/2025 6:48:47 AM.

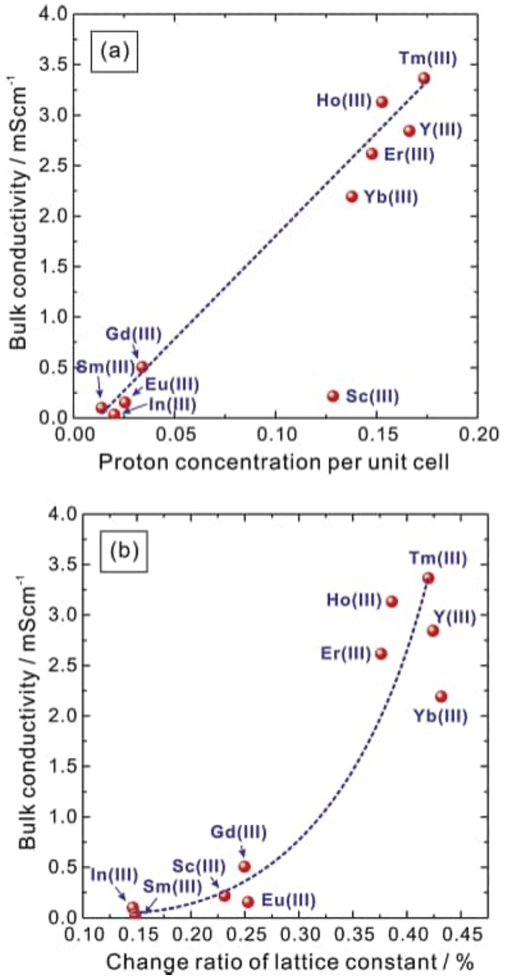

Fig. 10 Bulk conductivity of $Ba(Zr_{0.8}M_{0.2})O_{3-\delta}$ (M = Sc, Y, In, Sm, Eu, Gd, Ho, Er, Tm, Yb) at 300 °C plotted against (a) the proton concentration per unit cell and (b) the change ratio of the lattice constant. The proton conductivity was measured in an H_2 − 5% H_2O atmosphere. The change ratio of the lattice constant was determined by HT-XRD measurements performed in dry and wet O_2 (p_{H_2O} = 0.0312 atm).

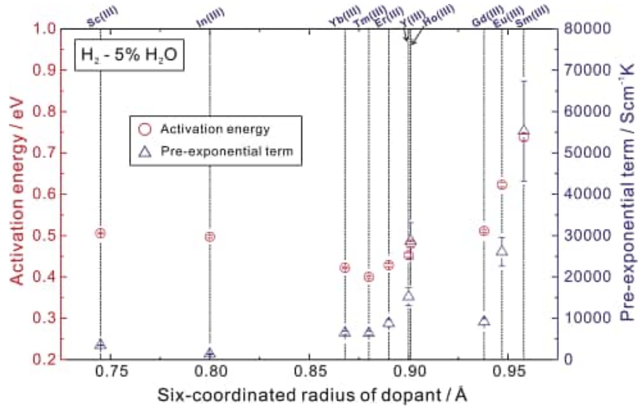

Fig. 11 Activation energy (red open circles) and pre-exponential term (blue open triangles) of the bulk conduction in $BaZrO_3$ doped with trivalent rare earth elements (Sc, Y, Sm, Eu, Gd, Ho, Er, Tm, Yb) in H_2 − 5% H_2O.

4. Discussion

Sc, In and Y were determined to occupy only the Zr site in a nearly stoichiometric sample containing a slight deficiency of Ba, whereas the lanthanide elements occupied both the Ba and Zr sites. Such a difference in site occupation seems to be a special property of the lanthanide elements. From the point of view of defect chemistry, replacing the divalent Ba cations by trivalent dopant cations consumed the oxide ion vacancies required for the hydration reaction and is thought to be a reason for the lowered conductivity when either a Ba deficiency[12] or a large dopant such as Gd[7] was introduced. However, we found that the amount of lanthanide elements at the Ba site was fairly limited. Even for the samples doped with Er and Tm, the amounts of which at the Ba site were determined to be 0.014 and 0.011, respectively, their conductivities were comparable with or even higher than that of the Y-doped sample. The Ba site occupation of the trivalent dopants may decrease the conductivity, but we consider that, in the nearly stoichiometric sample containing a slight Ba deficiency, this negative influence is fairly small and is not the main factor restricting the conductivity.

In most instances the proton concentration, conductivity and degree of lattice expansion as a result of the chemical expansion effect behaved in a generally related way – that is, the sample which showed a high proton conductivity also had a relatively high proton concentration and a strong chemical expansion effect. However, the Sc-doped sample behaved in an obviously different way: although the proton concentration was obviously high, the conductivity and lattice expansion was small. Therefore it is clear that the proton concentration is not the only factor influencing the conductivity and the method of proton trapping is another important factor that should be taken into account.[8,28] It is possible that the protons are trapped in a different way (relative to that in Y-doped $BaZrO_3$)[8] in the Sc-doped system and further investigation is required.

Y is regarded to be a good dopant to impart high proton conductivity in $BaZrO_3$. Dy also shows a high proton conductivity in wet H_2, but becomes tetravalent in an O_2 atmosphere, with a dramatic decrease in proton conductivity, which restricts its application as an electrolyte in fuel cells.[5,23] We found that, by introducing the same amount of 20 mol% of the dopants into $BaZrO_3$, the elements Yb and Er imparted comparatively high proton conductivities. However, on doping with Ho and Tm, even higher proton conductivities were generated in $BaZrO_3$ (Fig. 9). Therefore with respect to conductivity alone, Ho and Tm are better dopants than Y. However, as Tm is much more expensive than Y and Ho, the latter two are more practically appropriate dopants for proton conductive $BaZrO_3$.

5. Conclusion

Various dopants were added to $BaZrO_3$ and the conductivities were measured. The samples doped with Tm and Ho have even higher proton conductivities than the samples doped with Y. A systematic investigation was therefore carried out of the dopant site occupancy and the dependence of the proton concentration and chemical expansion on the dopants to determine the

Published on 14 November 2014. Downloaded by NANJING TECH UNIVERSITY on 2/1/2025 6:48:47 AM.

reason for the difference in conductivity seen with different dopants. In nearly stoichiometric samples with a slight Ba deficiency, Sc, Y and In occupied only the Zr site, whereas the dopants belonging to the lanthanide group occupied both the Ba and Zr sites. However, the amount of dopant in the Ba site is too limited to significantly influence the conductivity. In most instances, the samples with high proton concentrations had high conductivities, although the Sc-doped sample behaved differently. The Sc-doped sample had a high proton concentration, but a low conductivity. The lattice volume change resulting from chemical expansion also behaved in a similar way. The change in lattice volume of the Sc-doped $BaZrO_3$ was comparably small, but the samples doped with Yb, Tm, Er, Y and Ho showed large lattice expansions, suggesting that for the proton conductive acceptor-doped $BaZrO_3$, the proton conductivity and lattice change resulting from chemical expansion are correlated.

References

1 W. Grover Coors, *J. Power Sources*, 2003, **118**, 150.
2 Y. Okumura, Y. Nose, J. Katayama and T. Uda, *J. Electrochem. Soc.*, 2011, **158**, B1067.
3 Y. Yamazaki, R. Hernandez-Sanchez and S. M. Haile, *Chem. Mater.*, 2009, **21**, 2755.
4 D. Pergolesi, E. Fabbri, A. D'Epifanio, E. D. Bartolomeo, A. Tebano, S. Sanna, S. Licoccia, G. Balestrino and E. Traversa, *Nat. Mater.*, 2010, **9**, 846.
5 D. Han, Y. Nose, K. Shinoda and T. Uda, *Solid State Ionics*, 2012, **213**, 2.
6 R. C. T. Slade, S. D. Flint and N. Singh, *Solid State Ionics*, 1995, **82**, 135.
7 S. Imashuku, T. Uda, Y. Nose, G. Taniguchi, Y. Ito and Y. Awakura, *J. Electrochem. Soc.*, 2009, **156**, B1.
8 Y. Yamazaki, F. Blanc, Y. Okuyama, L. Buannic, J. C. Lucio-Vega, C. P. Grey and S. M. Haile, *Nat. Mater.*, 2013, **12**, 647.
9 D. Han, K. Kishida, K. Shinoda, H. Inui and T. Uda, *J. Mater. Chem. A*, 2013, **1**, 3027.
10 D. Han, K. Shinoda and T. Uda, *J. Am. Ceram. Soc.*, 2014, **97**, 643.
11 A. K. Azad, C. Savaniu, S. Tao, S. Duval, P. Holtappels, R. M. Ibberson and J. T. S. Irvine, *J. Mater. Chem.*, 2008, **18**, 3414.
12 Y. Yamazaki, R. Hernandez-Sanchez and S. M. Haile, *J. Mater. Chem.*, 2010, **20**, 8158.
13 T. Norby, M. Widerøe, R. Glöckner and Y. Larring, *Dalton Trans.*, 2004, **19**, 3012.
14 J. Wu, R. A. Davies, M. S. Islam and S. M. Haile, *Chem. Mater.*, 2005, **17**, 846.
15 D. Han, K. Kishida, H. Inui and T. Uda, *RSC Adv.*, 2014, **4**, 31589.
16 S. Yamaguchi and N. Yamada, *Solid State Ionics*, 2003, **162**, 23.
17 C. Hiraiwa, D. Han, A. Kuramitsu, A. Kuwabara, H. Takeuchi, M. Majima and T. Uda, *J. Am. Ceram. Soc.*, 2013, **96**, 879.
18 D. Han, M. Majima and T. Uda, *J. Solid State Chem.*, 2013, **205**, 122.
19 D. Han, K. Kojima, M. Majima and T. Uda, *J. Electrochem. Soc.*, 2014, **161**, F977.
20 A. K. E. Andersson, S. M. Selbach, C. S. Knee and T. Grande, *J. Am. Ceram. Soc.*, 2014, **97**, 2654.
21 D. Han, Y. Okumura, Y. Nose and T. Uda, *Solid State Ionics*, 2010, **181**, 1601.
22 D. Han, K. Shinoda, S. Tsukimoto, H. Takeuchi, C. Hiraiwa, M. Majima and T. Uda, *J. Mater. Chem. A*, 2014, **2**, 12552.
23 D. Han, T. Uda, Y. Nose, T. Okajima, H. Murata, I. Tanaka and K. Shinoda, *Adv. Mater.*, 2012, **24**, 2051.
24 R. D. Shannon, *Acta Crystallogr., Sect. A: Cryst. Phys., Diffr., Theor. Gen. Crystallogr.*, 1976, **32**, 751.
25 O. Knop and J. S. Carlow, *Can. J. Chem.*, 1974, **52**, 2175.
26 M. A. Thundathil, C. Y. Jones, G. J. Snyder and S. M. Haile, *Chem. Mater.*, 2005, **17**, 5146.
27 C. Y. Jones, J. Wu, L. Li and S. M. Haile, *J. Appl. Phys.*, 2005, **97**, 114908.
28 F. Blanc, L. Sperrin, D. Lee, R. Dervisoğlu, Y. Yamazaki, S. M. Haile, G. De Paëpe and C. P. Grey, *J. Phys. Chem. Lett.*, 2014, **5**, 2431.

 This journal is © The Royal Society of Chemistry 2015

crystals

MDPI

Article

Growth and Dielectric Properties of Ta-Doped $La_2Ti_2O_7$ Single Crystals

Hui Wang [1,2], Qin Li [2], Chaoyue Wang [2], Huan He [2], Jianding Yu [2,*] and Jiayue Xu [1,*]

[1] School of Materials Science and Engineering, Shanghai Institute of Technology, Shanghai 201418, China; zhifeiji0613@163.com
[2] Shanghai Institute of Ceramics, Chinese Academy of Science, Shanghai 200050, China; liqin@mail.sic.ac.cn (Q.L.); cywang@mail.sic.ac.cn (C.W.); ch.hh@mail.sic.ac.cn (H.H.)
* Correspondence: yujianding@mail.sic.ac.cn (J.Y.); xujiayue@sit.edu.cn (J.X.); Tel.: +86-021-5241-2503 (J.Y.); +86-021-6087-3581 (J.X.)

Received: 20 November 2017; Accepted: 13 February 2018; Published: 27 February 2018

Abstract: High-quality Ta-doped $La_2Ti_2O_7$ (Ta-LTO) single crystal of about 40 mm in length and 5 mm in diameter was successfully prepared by the optical floating zone method. An X-ray rocking curve reveals that the crystal of LTO has excellent crystalline quality. As-grown crystals were transparent after annealing in air and the transmittance is up to 76% in the visible and near-infrared region. X-ray diffraction showed that this compound possessed a monoclinic structure with $P2_1$ space group. The dielectric properties were investigated as functions of temperature (0~300 °C) and frequency (10^2 Hz~10^5 Hz). Dielectric spectra indicated an increase in the room-temperature dielectric constant accompanied by a drop in the loss tangent as a result of the Ta doping. One relaxation was observed in the spectra of electric modulus, which was ascribed to be related to the oxygen vacancy. The dielectric relaxation with activation energy of 1.16 eV is found to be the polaron hopping caused by the oxygen vacancies.

Keywords: Ta-LTO single crystal; floating zone method; dielectric properties; oxygen vacancy

1. Introduction

With the recent progress and development in chemical and material processing, automotive, aerospace, and power-generating industries, there has been an ever-increasing need for actuators and sensors that can be operated at high temperatures [1–3]. For example, nuclear reactors need to be monitored during operation, and the temperature can be reached above 1000 °C [1,2]. Similarly, aerospace engines have working temperature at above 900 °C [1,2]. They both require a high-temperature sensor to monitor the operation situation. In the past several decades, most studies on high-temperature piezoelectric or ferroelectric have focused on a range of single crystals based on gallium phosphate ($GaPO_3$), lithium niobate ($LiNbO_3$, LN), langasites with general formula, $Ca_3TaAl_3Si_2O_{14}$, and oxyborate [$ReCa_4O(BO_3)$] (Re is rare earth element) [1,2,4–7]. Low Curie point, high cost and poor mechanical properties have limited their practical applications at very high temperature. So it is of great significance to develop piezoelectric materials with high Tc to meet urgent requirements for high-temperature applications.

Ferroelectric materials with a perovskite-like layered structure (PLS) have caught attention due to the highest Curie temperature [8,9]. Generally, the PLS structure with a formula of $A_nB_nO_{3n+2}$ is characterized by coner-shared BO_6 octahedron and 12-coordinated A cations within the perovskite-like layered separated by oxygen-rich gaps [10,11]. Among all these PLS ferroelectrics, $La_2Ti_2O_7$ (LTO) is a promising candidate for actuators and sensors at extremely high temperature (Tc = 1500 °C). Masakazu et.al had reported the LTO single crystal for the first time in 1972 [12]. The piezoelectric coefficient (d_{22}) and relative permittivity (ε) of LTO single crystal had been reported to be 16 pC/N

and 42–62, respectively [9]. The ferroelectric and piezoelectric properties of LTO had been studied in the form of polycrystalline ceramics and thin films [13–20]. In addition, Ni-doped LTO has been found to have good photocatalytic activity in the water-splitting reaction [21] and in the oxidative decomposition of CH_3Cl [22]. Cai et al. [23] found that Graphitic-C_3N_4 hybridized N-doped LTO two-dimensional layered composites as an efficient visible-light-driven photocatalyst. Also, a series of ceramics with a general formula $La_2Ti_{2-x}Ta_xO_7$ (x = 0.05, 0.1, 0.2 and 0.3) were prepared by Spark Plasma Sintering [24].

However, LTO and Ta-doped LTO single crystals have barely been reported. On the one hand, their melting points are very high and volatile; on the other hand, they are easily cleaved due to the special layered structure. Generally speaking, it was difficult to prepare a single crystal, since the piezoelectric properties are closely related to the dielectric properties, and in lead zirconate titanate (PZT), pentavalent donor additions (tantalum) were reported to produce significant effects on the mechanical and electrical properties characterized by enhanced dielectric constant and DC resistivity [25]. Therefore this motivated us to study effects of tantalum substitution on the structure and dielectric properties of $La_2Ti_2O_7$. A thorough understanding of the dielectric properties is of vital importance.

In the present work, we successfully prepared the LTO and Ta-doped LTO single crystals using optical floating zone method (OFZ). The scanning electron microscope (SEM) with energy-dispersive X-ray (EDX) experiment used a field emission scanning electron microscope Hitachi SU8220 manufactured by Hitachi, Japan. The XRD patterns were recorded in the range 10°–70°, with a step of 0.02° and with a speed of 2°/min, using a Rigaku Ultima IV X-ray diffractometer equipped with Cu anticathode (λkα1 = 1.54059 nm), nickel foil filter used to attenuate Cu kβ radiation and Soller slits to limit the divergence of X-ray beam. The transmittance (Varian Cary-5000, Agilent, Varian, Palo Alto, CA, USA) of LTO and Ta-LTO single crystals in visible and near-infrared region were measured. Besides, detailed investigations on the frequency (10^2 Hz–10^5 Hz) dielectric properties of Ta-doped LTO single crystal over a wide temperature range from 0–300 °C were performed. The dielectric properties were measured on a precise impedance analyzer dielectric measuring system (Broadband Dielectric/Impedance Spectrometer, Novocontrol Technologies Gmbh and Co. KG, Germany).

2. Experimental Details

High-purity powders of La_2O_3 (purity 99.99%, Sinopharm Chemical Regent, Shanghai, China), TiO_2 (Purity 99.99%, imported from Japan) and Ta_2O_5 (Purity 99.99%, Aladdin, Shanghai, China) were mixed in a stoichiometry of $La_2Ti_2O_7$ and $La_2Ti_{1.98}Ta_{0.02}O_7$ chemical formula. They were milled in the presence of absolute ethanol for 24 h. After being dried and screened, the mixture was calcined at 1300 °C for 8 h in air with an intermediate grinding to improve its homogeneity. Then, the well-mixed powders were ground and formed into round rods using a cold isostatic pressure of 200 MPa. The rods were then sintered at 1200 °C–1500 °C for 5–8 h in air. The typical dimensions of the sintered rod were 7–9 mm in diameter and 60–80 mm in length. The polycrystalline rods were used as feed and seed rods.

The crystal growth experiments were carried out by the OFZ technique with four ellipsoidal mirrors (FZ-T4000H), using four 4.0 KW halogen lamps as heat sources and the LabVIEW control system. The feed rod was suspended at the upper shaft and the seed was attached to the lower shaft. Both the feed rod and seed rod moved downwards and the melting zone was formed. The feed and seed shafts rotated at 15 rpm in the opposite direction and the crystal was grown from the bottom to top in a vertical direction, and the growth rate was 4–6 mm/h.

In this work, we have grown single crystals in an argon atmosphere and crack-free single crystals were obtained. The as-grown single crystals were annealed in air at 1200 °C for 1 h to eliminate thermal stress and reduce oxygen vacancy, then were naturally cooled down to room temperature. We have randomly selected two pieces of crystal from the big Ta-LTO crystals. One of them was characterized by energy-dispersive X-ray (EDX). The other was ground into powder to measure the

X-ray diffraction. In order to check the crystal perfection, a (100) wafer of LTO crystal was carefully polished for measuring the rocking curve by high resolution X-ray diffraction (HR-XRD). The crystals ware cut into several slices perpendicular to the direction of growth and then polished. The prior growth direction was (622) determined by X-ray orientation.

3. Result and Discussion

The melting point of tantalum oxide is higher than lanthanum oxide, so it is more difficult to grow Ta-doped LTO solution crystals at higher temperature. Considering the melting point (1790 °C) and furnace power (1.94 KW) of LTO crystals, we adopted furnace power of 2.16 KW to grow Ta-LTO crystals in an argon atmosphere. By optimizing growth parameters, we have successfully grown the LTO and Ta-LTO single crystals, and a typical as-grown Ta-LTO single crystal boule is shown in Figure 1a. The Ta-LTO single crystal without annealing is black and non-transparent because of oxygen vacancy. The Figure 1b is the Ta-LTO single crystal after annealing for 1 h at 1200 °C in air. Annealing not only eliminated the thermal stress, but also reduced the oxygen vacancy and made the single crystal transparent. Oh et al. had suggest that the post-annealing effect, which involves a reduction in carrier concentration, originates with the loss of oxygen vacancies in the contact region by absorbing the oxygen present in the annealing atmosphere [26]. One sample was cut directly from the Ta-LTO single crystal boule perpendicular to the growth direction, then polished as shown in Figure 1c. Some cracks can be seen in Figure 1b. After the X-ray diffraction orientation, it is found that these cracks are due to incomplete cleavage along (400) direction consistent with pure LTO as Figure 1d [9]. Figure 2 shows the X-ray rocking curve of the LTO crystal. The profile of the (400) Bragg-reflections has a symmetrical peak with a full width at half maximum (FWHM) of 19.4 arcsec. The nearly perfect shape without any shoulder clarified the absence of any sub-grain, indicating the excellent quality of the LTO crystals.

The schematic diagram of the growth direction specimen of Ta-LTO single crystal wafer and the XRD of the Ta-LTO crystal wafer after annealing are shown in Figure 3, respectively. The Ta-LTO single crystals were grown by spontaneous nucleation. The prior growth direction was (622) determined by X-ray orientation. The energy-dispersive X-ray (EDX) images of Ta-LTO single crystal were shown in Figure 4. The results showed the composition homogeneity.

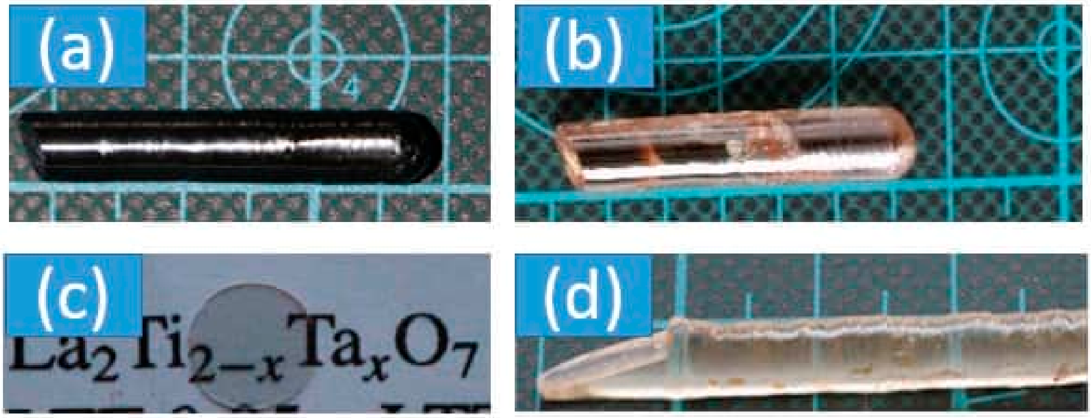

Figure 1. Ta-LTO single crystal grown by spontaneous nucleation with optical floating zone method (**a**) Ta-LTO single crystal was annealed for 1 h at 1200 °C; (**b**) Ta-LTO crystal wafer after annealing; (**c**) LTO single crystal by floating zone method (**d**).

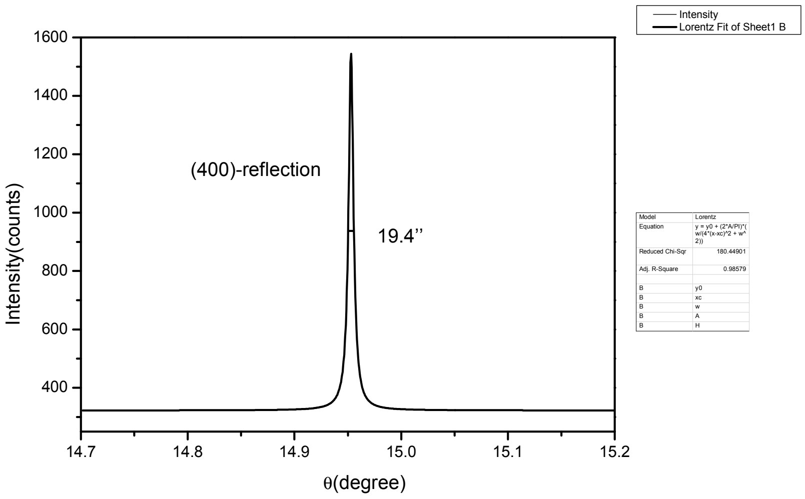

Figure 2. X-ray rocking curve of LTO single crystal.

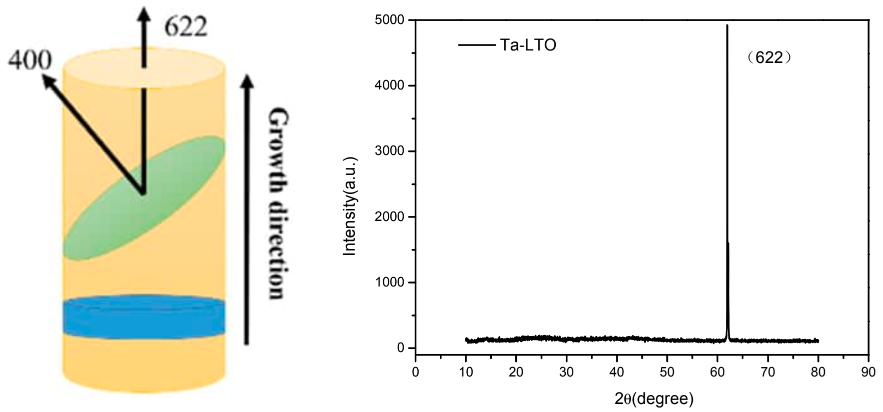

Figure 3. The schematic diagram of the growth direction specimen of Ta-LTO single crystal (**Left**) and the XRD of the Ta-LTO crystal wafer (**Right**).

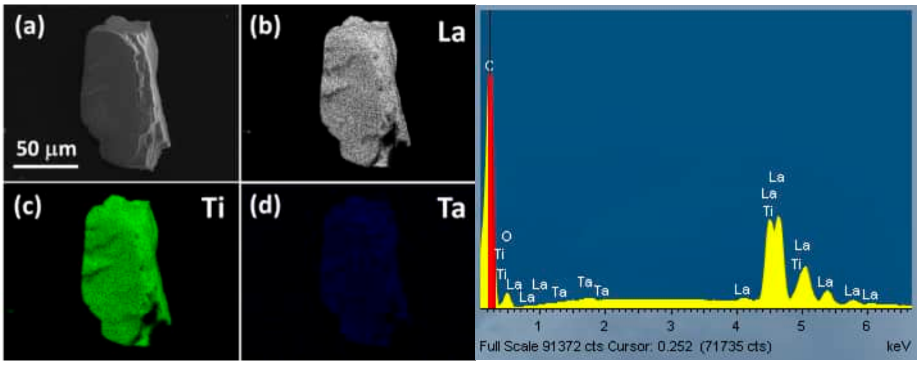

Figure 4. EDX images of Ta-LTO single crystal. (**a**): Ta-LTO (**b**): La (**c**):Ti (**d**):Ta.

The XRD patterns of the LTO and Ta-LTO powder samples at room temperature were shown in Figure 5. It can be noted that the main phase of Ta doped LTO was the same as LTO. All the peaks were successfully indexed based on a monoclinic crystal structure with $P2_1$ space group. As is shown in the inset, we can see that the position of the corresponding peak move to a large diffraction angle compared with LTO. The lattice parameters calculated with Jade 6.0 software are a = 12.94924(3) Å, b = 5.56649(4) Å, and c = 7.81266(3) Å. Compared to LTO [9], it is observed that both b and c increased

slightly with Ta doping, whereas a exhibited an obvious decrease. Generally, Ta ions replace Ti ions and the Ta ion (0.65 Å) radius are larger than Ti (0.604Å) [27], which induce an expansion of the crystal structure. Similar phenomena can be seen in indium-doped barium hexaferrites [28].

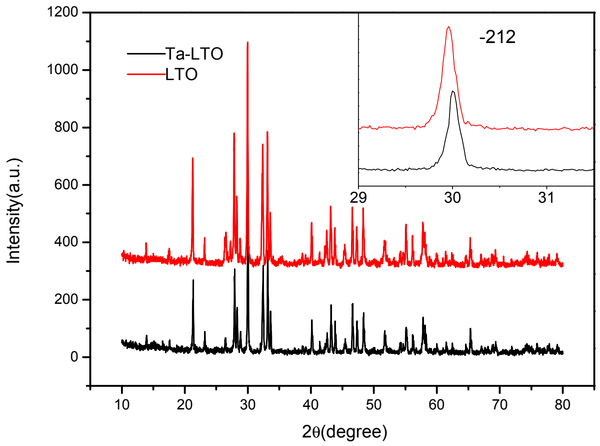

Figure 5. XRD patterns of the Ta-LTO and $La_2Ti_2O_7$ crystalline powder. The inset shows the magnified patterns of main peak.

Figure 6 shows the transmission spectrum of Ta-LTO and LTO crystals. It is transparent in the wavelength range of 350–900 nm and both of the transmittance are about 70%. Compared to the transmittance spectrum of Ta-LTO and LTO single crystals, we can see that the absorption edge shifted to infrared direction due to doping Ta_2O_5 into LTO single crystals.

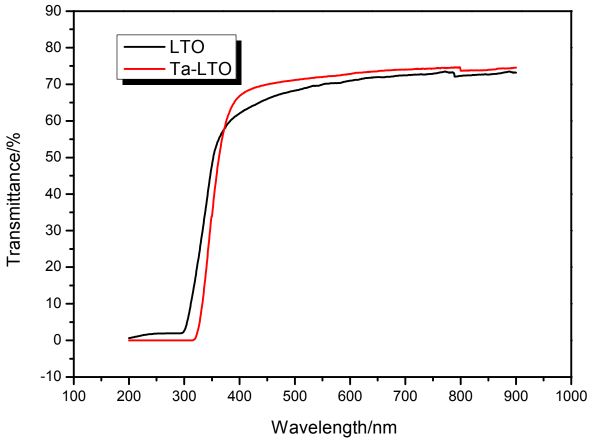

Figure 6. Transmission spectrum of Ta-LTO and LTO single crystals (0.02 mm).

The temperature dependences of the dielectric constant and dielectric loss tangent at various frequencies of LTO and Ta-LTO single crystals are shown in Figure 7a,b, respectively. From Figure 7a, we can see that dielectric constant for LTO crystals are almost independent of frequency and temperature in the low-temperature range from 0~100 °C. However, when the temperature is over 100 °C, the loss tangent in the form of index increased. This may be attributed to the larger conductivity due to oxygen vacancies. Although the Ta-LTO crystal changed from black to transparent after annealing at 1200 °C, it still has some oxygen vacancies. As the temperature increases, the movement speed of the oxygen vacancy increases rapidly, leading to the increase in current. In Figure 7b, it can be seen that the dielectric constant for Ta-LTO crystals are almost independent of frequency and temperature in the low-temperature range from 0–150 °C. This behavior indicates that Ta-LTO shows an intrinsic dielectric response resulting from the electronic and/or ionic polarization in the

low-temperature range [29,30]. The dielectric constant plateau of Ta-LTO shows a value of ~68 as Figure 7b, which is higher than the dielectric constant of LTO as shown in Figure 7a, so Ta doping can improve the dielectric properties of LTO. As shown in the inset of Figure 7a,b, the dielectric loss tangent of Ta-LTO crystal was lower than LTO crystal, which is more suitable for practical applications. It is worth noting that the frequency-dependent dielectric permittivity of Ta-LTO remarkably increases as the temperature increases above 200 °C, and tanδ (T) increases rapidly with increasing temperature, causing pronounced background. The remarkable background in high temperature is usually caused by conductivity [31]. In this case, we applied the electric modulus, which is a powerful function for revealing background-obscured relaxation [30]. We, therefore, resort to the electric modulus.

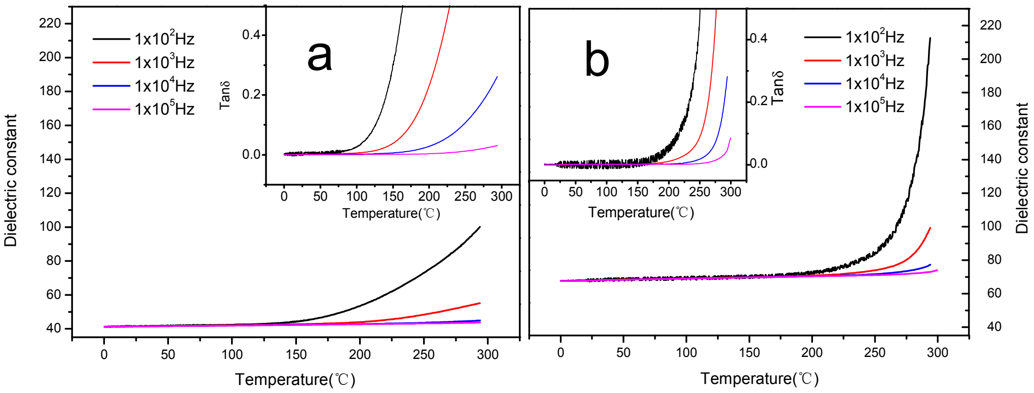

Figure 7. Temperature dependence of dielectric constant and tanδ at various frequencies from 10^2 Hz to 10^5 Hz. (**a**) for LTO single crystal and (**b**) for Ta-LTO single crystal.

Figure 8a shows the imaginary part of the electric modulus M'' as a function of frequency in the temperature range from 398 k to 573 k. We can clearly see that M'' registers a peak whose position shifts to a higher temperature with increasing frequency. Meanwhile, the relaxation cannot be observed in the spectra of dielectric constants, indicating that electric modulus is a "good" dielectric function in revealing dielectric relaxation in the absence of a well-defined peak in tanδ (T) or dielectric constant. It is well known that there are two sources contributing to the dielectric permittivity in the high-temperature range: the dielectric relaxation and the conductivity. When the conductivity contribution surpasses the relaxation contribution, especially in the high-temperature range, the relaxation is merged by the background. Since the electric modulus is defined as $M^* = M' + jM'' = 1/\varepsilon^*$, the higher values of the background and the smaller values of M'' can be obtained. Therefore, the electric modulus can greatly lessen the background and become a "good" dielectric function in revealing the background-merged relaxation. The relaxation parameter can be obtained in terms of Arrhenius law

$$f = f_0 \exp(E_a/k_B T_P) \quad (1)$$

where f_0 is the pre-exponential factor, E_a is the activation energy, k_B is the Boltzman constant, T_P is the peak temperature. Figure 8c,d shows the Arrhenius plot of ln$f$ as a function of $1000/T_p$. The Arrhenius relation are quite good straight lines, the straight line fitting to Equation (1) gives the activation energy for relaxation E_a = 1.16 eV in the Ta-LTO and 1.07 eV in the LTO. Obviously LTO has a much wider frequency span than Ta-LTO in Figure 7a,b. It results in a relatively slight enhancement in activation energy values for Ta-doped LTO. As this may be Ta^{5+} ion to Ti^{4+} site and enhance the energy gap, the original bound electron hole is a more difficult to excite carrier, causing increased activation.

Now we turn our attention to the origin of dielectric anomaly in the Ta-LTO crystal at a temperature of around 250–300 °C in Figure 7b. As can be seen from inset of Figure 7b, the dielectric anomaly appears in the temperature where tanδ nearly exponentially increases with temperature. This fact indeed indicates that the conductivity becomes remarkable in this temperature range that causes pronounced background. So from Figure 8a we can clearly see that a set of M'' (T) peaks

appear in the temperature range where the dielectric anomaly occurs. The peak shifts to higher temperature with increasing frequency indicative of a thermally activated relaxation process. So the dielectric anomaly behavior in the Ta-LTO crystal sample might be related to the oxygen vacancies. An oxygen-related dielectric anomaly has been reported in different material systems [32–34]. Two oxygen-vacancy-related relaxation processes have been reported [35]: a dipolar relaxation related to the hopping motions of oxygen vacancy and a Maxwell-Wagner relaxation as the defects were blocked by the interface on sample contacts. Typically, the dielectric relaxation of Mn-doped $YFeO_3$ ceramics at high temperature was originated from the polaron hopping based on the electron defect [36]. Meanwhile the dielectric relaxation of bismuth-doped $La_2Ti_2O_7$ ceramics in the temperature range of 600–1000 °C originates from the Maxwell-Wagner effect [37]. It is well known that oxygen vacancies can make contributions to polarization in the form of doubly charged state at high temperature ranges. For example, the activation energy for the relaxation associated with doubly ionized oxygen vacancies in $NiTiO_3$ was found to be 1.17 eV [38]. The activation energy value for Ta-LTO crystal samples is 1.16 eV, so the dielectric relaxation in the Ta-LTO crystal stems from the doubly charge oxygen vacancies. If the dielectric relaxation was related to the electron migration of oxygen vacancies, the polarization and conduction processes should show close values of activation energy [39]. The carrier conduction process can be understood through AC conductivity measurements.

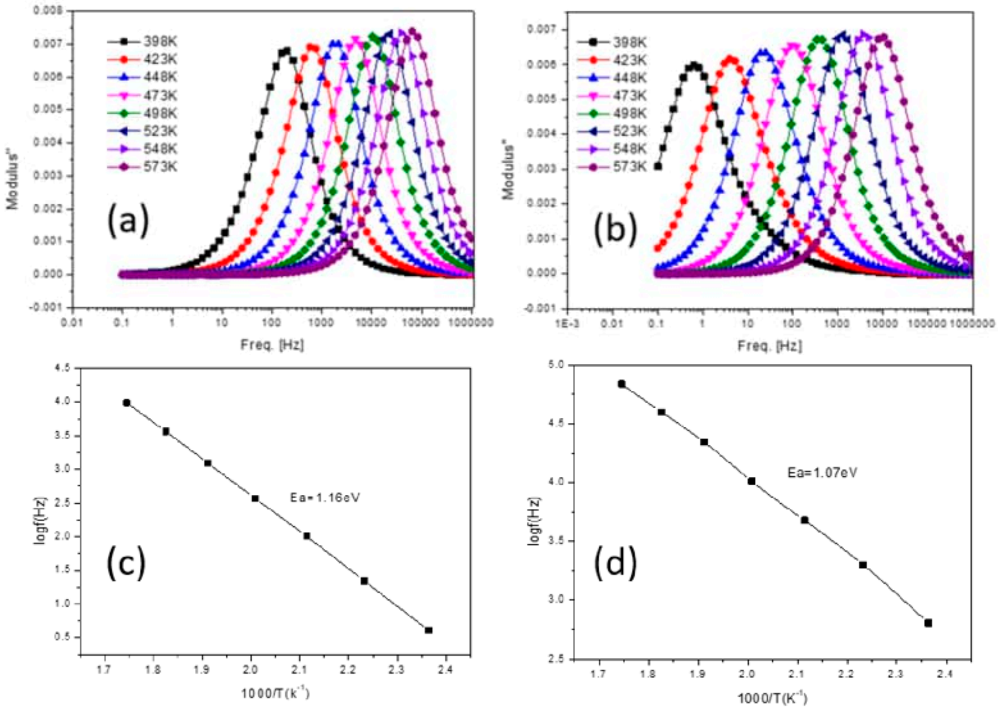

Figure 8. (**a**,**b**) are frequency dependence of the imaginary part of the electric modulus in the temperature range from 398 k to 573 k for Ta-LTO and LTO single crystals, respectively; and (**c**,**d**) are the corresponding Arrhenius plots of Ta-LTO and LTO crystals, respectively.

Figure 9 shows the variation of AC conductivity with frequency at several temperatures. The conductivity was observed to be nearly the frequency independent in the low frequency, and then exponentially increases with frequency. As seen in Figure 9, the AC conductivity is nearly constant at low frequencies and is approximately equal to DC conductivity (σdc). The plateau region extends to higher frequencies with the decrease of temperature, the σdc can be read directly and is plotted as a function of the reciprocal of temperature as shown in the insert of Figure 9. It follows the Arrhenius law:

$$\sigma_{dc} = \sigma_0 exp[-E_{cond}/k_B T] \quad (2)$$

where σ_0 is the pre-exponential factor, and E_{cond} is the activation energy for conduction. The activation energy was deduced to be E_{cond} = 0.96 eV. It was a little smaller than the activation energy for dielectric relaxation (E_a = 1.16 eV), which suggests that the dielectric anomaly at temperature of about 275 °C is truly related to the hopping motions of doubly charged oxygen vacancies.

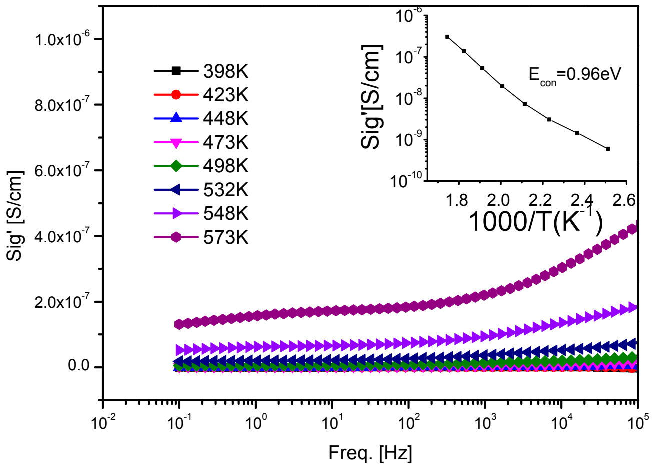

Figure 9. AC conductivity as a function of frequency at selected temperatures for Ta-LTO crystal. The inset shows the date in Arrhenius representation.

4. Conclusions

In this work, a Ta-doped $La_2Ti_2O_7$ single crystal of about 40 mm in length and 5 mm in diameter was successfully grown by the floating zone method in an argon atmosphere. The crystal has a monoclinic structure ($P2_1$) with good composition homogeneity. The dielectric properties of Ta-LTO crystal has been systematically investigated in the frequency of 10^2 Hz to 10^5 Hz and a temperature range of 0–300 °C. An obvious dielectric relaxation with the activation energy of 1.16 eV was observed in the spectra of the electric modulus, which may be associated with the migration of oxygen vacancies.

Acknowledgments: The work is supported by the Nature Science Foundation of China (51572175) and the International Cooperation Program of Chinese Academy of Sciences (Y35YQ1110G).

Author Contributions: Jianding Yu conceived and designed the experiments; Huan He and Chaoyue Wang grew the single crystals; Hui Wang measured the dielectric properties; Hui Wang and Qin Li analyzed the data; Hui Wang wrote the manuscript; Jiayue Xu made the final modification. All authors read and approved the final version of the manuscript to be submitted.

Conflicts of Interest: The authors declare no conflict of interest.

References

1. Turner, R.; Fuierer, P.; Newnham, R.; Shrout, T. Materials for high-temperature acoustic and vibration sensors: A review. *Appl. Acoust.* **1994**, *41*, 299–324. [CrossRef]
2. Damjanovic, D. Materials for high temperature piezoelectric transducers. *Solid State Mater. Sci.* **1998**, *3*, 469–473. [CrossRef]
3. Zhang, S.J.; Xia, R.; Lebrun, L.; Anderson, D.; Shrout, T.R. Piezoelectric materials for high power, high temperature applications. *Mater. Lett.* **2005**, *59*, 3471–3475. [CrossRef]
4. Zhang, S.J.; Yu, F.P. Piezoelectric materials for high temperature sensors. *J. Am. Ceram. Soc.* **2011**, *94*, 17–34. [CrossRef]
5. Zhang, S.J.; Frantz, E.; Xia, R.; Everson, W.; Randi, J.; Snyder, D.; Shrout, T. Gadolinium calcium oxyborate piezoelectric single crystals for ultra-high temperature (>1000 °C) applications. *J. Appl. Phys.* **2008**, *104*, 084103. [CrossRef]

6. Zhang, S.J.; Fei, Y.; Frantz, E.; Snyder, D.; Chai, H.; Shrout, T. High-temperature piezoelectric single crystal $ReCa_4O(BO_3)_3$ for sensor applications. *IEEE Trans. Ultrason. Ferroelectr. Freq. Control* **2008**, *55*, 2703–2708. [CrossRef] [PubMed]
7. Costa, S.; Li, M.; Frade, J.; Sinclair, D.C. Modulus spectroscopy of $CaCu_3Ti_4O_{12}$ ceramics: Clues to the internal barrier layer capacitance mechanism. *RSC Adv.* **2013**, *3*, 7030–7036. [CrossRef]
8. Yamamoto, J.K.; Bhalla, A.S. Piezoelectric properties of layered perovskite $A_2Ti_2O_7$ (A = La and Nd) single-crystal fibers. *J. Appl. Phys.* **1991**, *70*, 4469. [CrossRef]
9. Nanamatsu, S.; Kimura, M.; Doi, K.; Matsushita, S.; Yamada, N. A new ferroelectric: $La_2Ti_2O_7$. *Ferroelectrics* **1974**, *8*, 511–513. [CrossRef]
10. Lichtenberg, F.; Herrnberger, A.; Wiedenmann, K. Synthesis, structural, magnetic and transport properties of layered perovskite-related titanates, niobates and tantalates of the type $A_nB_nO_{3n+2}$, $A'A_{k-1}$ B_kO_{3k+1} and $A_mB_{m-1}O_{3m}$. *Prog. Solid State Chem.* **2008**, *36*, 253–387. [CrossRef]
11. Lichtenberg, F.; Herrnberger, A.; Wiedenmann, K.; Mannhart, J. Synthesis of perovskite-related layered $A_nB_nO_{3n+2}$ = ABO_x type niobates and titanates and study of their structural, electric and magnetic properties. *Prog. Solid State Chem.* **2001**, *29*, 1–70. [CrossRef]
12. Kimura, M.; Nanmatsu, S.; Doi, K.; Matsushita, S.; Takahashi, M. Electrooptic and piezoelectric properties of $La_2Ti_2O_7$ single crystal. *Jpn. J. Appl. Phys.* **1972**, *11*, 904. [CrossRef]
13. Takahashi, J.; Kageyamal, K.; Kodaira, K. Microwave dielectric properties of lanthanide titanate ceramics. *Jpn. J. Appl. Phys.* **1993**, *32*, 4327–4331. [CrossRef]
14. Paul, A.F.; Robert, E.N. $La_2Ti_2O_7$ ceramics. *J. Am. Ceram. Soc.* **1991**, *74*, 2876–2881.
15. Yan, H.X.; Ning, H.P.; Kan, Y.; Wang, P.; Reece, M.J. Piezoelectric ceramics with super-high curie points. *J. Am. Ceram. Soc.* **2009**, *92*, 2270–2275. [CrossRef]
16. Atuchin, V.V.; Gavrilova, T.A.; Grivel, J.C.; Kesler, V.G. Electronic structure of layered ferroelectric high-k titanate $La_2Ti_2O_7$. *J. Phys. D* **2009**, *42*, 035305. [CrossRef]
17. Fasquelle, D.; Carru, J.C.; Le Gendre, L.; Le Paven, C.; Pinel, J.; Cheviré, F.; Tessier, F.; Marchand, R. Lanthanum titanate ceramics: Electrical characterizations in large temperature and frequency ranges. *J. Eur. Ceram. Soc.* **2005**, *25*, 2085–2088. [CrossRef]
18. Gao, Z.; Wu, L.; Lu, C.; Wei, G.; Zhang, T.; Liu, G.; Xie, Q.; Li, M. The anisotropic conductivity of ferroelectric $La_2Ti_2O_7$ ceramics. *J. Eur. Ceram. Soc.* **2017**, *37*, 137–143. [CrossRef]
19. Orum, A.; Takatori, K.; Hori, S.; Ikeda, T.; Yoshimura, M.; Tani, T. Atomic force microscopy surface analysis of layered perovskite $La_2Ti_2O_7$ particles grown by molten flux method. *Jpn. J. Appl. Phys.* **2016**, *55*, 08NB08. [CrossRef]
20. Kim, H.G.; Hwang, D.W.; Bae, S.W.; Jung, J.H.; Lee, J.S. Photocatalytic water splitting over $La_2Ti_2O_7$ synthesized by the polymerizable complex method. *Catal. Lett.* **2003**, *91*, 193–198. [CrossRef]
21. Hwang, D.W.; Kim, H.G.; Kim, J.; Cha, K.Y.; Kim, Y.G.; Lee, J.S. Photocatalytic water splitting over highly donor-doped (110) layered perovskites. *J. Catal.* **2000**, *193*, 40–48. [CrossRef]
22. Hwang, D.W.; Cha, K.; Kim, Y.J.; Kim, H.G.; Bae, S.W.; Lee, J.S. Photocatalytic degradation of CH_3Cl over a nickel-loaded layered perovskite. *Ind. Eng. Chem. Res.* **2003**, *42*, 1184–1189. [CrossRef]
23. Cai, X.; Zhang, J.; Mamoru, F.; Tetsuro, M. Graphitic-C_3N_4 hybridized N-doped $La_2Ti_2O_7$ two-dimensional layered composites as efficient visible-light-driven photocatalyst. *Appl. Catal. B* **2017**, *202*, 191–198. [CrossRef]
24. Li, C.C.; Khaliq, J.; Ning, H.P.; Wei, X.Y.; Yan, H.Y.; Reece, M.J. Study on properties of tantalum-doped $La_2Ti_2O_7$ ferroelectric ceramics. *J. Adv. Dielectr.* **2015**, *5*, 1550005. [CrossRef]
25. Gerson, R. Variation in ferroelectric characteristics of lead zirconate titanate ceramics due to minor chemical modifications. *J. Appl. Phys.* **1960**, *31*, 188. [CrossRef]
26. Oh, Y.W.; Kim, H.; Baek, K.H.; Do, L.M.; Lee, G.W.; Kang, C.M. Effect of post-annealing on low-temperature solution-processed high-performance indium oxide thin film transistors. *Sci. Adv. Mater.* **2018**, *10*, 518–521. [CrossRef]
27. Shannon, R.D. Revised effective ionon radii and systematic studies of interatomic distances in halides and chalcogenides. *Acta Crystallogr.* **1976**, *32*, 751–767. [CrossRef]
28. Trukhanov, S.V.; Trukhanov, A.V.; Turchenko, V.A.; Trukhanov, A.V.; Trukhanova, E.L.; Tishkevich, D.I.; Ivanov, V.M.; Zubar, T.I.; Salem, M.; Kostishyn, V.G.; et al. Polarization origin and iron positions in indium doped barium hexaferrites. *Cream. Int.* **2018**, *44*, 290–300. [CrossRef]

29. Fiedziuszko, S.J.; Holme, S. Dielectric resonators raise your high-Q. *IEEE Microw. Mag.* **2001**, *2*, 50–60. [CrossRef]
30. Wang, C.C.; Wang, J.; Sun, X.H.; Liu, L.N.; Zheng, J.; Cheng, C. Oxygen-vacancy-relaxated dielectric relaxation in $Na_{0.5}K_{0.5}NbO_3$. *Solid State Commun.* **2014**, *179*, 29–33. [CrossRef]
31. Wang, C.C.; Cui, Y.M.; Zhang, L.W. Dielectric properties of $TbMnO_3$ ceramics. *Appl. Phys. Lett.* **2007**, *90*. [CrossRef]
32. Wang, C.C.; Zhang, L.W. Oxygen-vacancy-related dielectric anomaly in $CaCu_3Ti_4O_{12}$: Post-sintering annealing studies. *Phys. Rev. B* **2006**, *74*. [CrossRef]
33. Wang, C.C.; Zhang, M.N.; Xia, W. High-temperature dielectric relaxation in $Pb(Mg_{1/3}Nb_{2/3})O_3$–$PbTiO_3$ single crystals. *J. Am. Ceram. Soc.* **2013**, *96*, 1521–1525. [CrossRef]
34. Liu, L.N.; Wang, C.C.; Lei, C.M.; Li, T.; Wang, G.J.; Sun, X.H.; Wang, G.J.; Huang, S.G.; Li, Y.D.; Wang, H. Relaxor- and phase-transition-like behaviors in ZnO single crystals at high temperatures. *Appl. Phys. Lett.* **2013**, *102*. [CrossRef]
35. Ye, J.L.; Wang, C.C.; Ni, W.; Sun, X.H. Dielectric properties of $ErFeO_3$ ceramics over a broad temperature range. *J. Alloy. Compd.* **2014**, *617*, 850–854. [CrossRef]
36. Zhang, C.; Wang, X.; Wang, Z.; Yan, H.; Li, H.; Li, L. Dielectric relaxation, electric modulus and ac conductivity of Mn-doped $YFeO_3$. *Ceram. Int.* **2016**, *42*, 19461–19465. [CrossRef]
37. Zhang, N.; Li, Q.J.; Huang, S.G.; Yu, Y.; Zheng, J.; Cheng, C.; Wang, C.C. Dielectric relaxations in multiferroic $La_2Ti_2O_7$ ceramics. *J. Alloy. Compd.* **2015**, *652*, 1–8. [CrossRef]
38. Li, T.; Wang, C.C.; Lei, C.M.; Sun, X.H.; Wang, G.J.; Liu, L.N. Conductivity relaxation in $NiTiO_3$ at high temperatures. *Curr. Appl. Phys.* **2013**, *13*, 1728–1731. [CrossRef]
39. Deng, G.C.; Li, G.R.; Ding, A.L.; Yin, Q.R. Evidence for oxygen vacancy inducing spontaneous normal-relaxor transition in complex perovskite ferroelectrics. *Appl. Phys. Lett.* **2005**, *87*. [CrossRef]

© 2018 by the authors. Licensee MDPI, Basel, Switzerland. This article is an open access article distributed under the terms and conditions of the Creative Commons Attribution (CC BY) license (http://creativecommons.org/licenses/by/4.0/).

Applied Catalysis B: Environmental 343 (2024) 123464

Contents lists available at ScienceDirect

Applied Catalysis B: Environmental

journal homepage: www.elsevier.com/locate/apcatb

Oxygen vacancies-modified S-scheme heterojunction of Bi-doped $La_2Ti_2O_7$ and La-doped $Bi_4Ti_3O_{12}$ to improve the NO gas removal avoiding NO_2 product

Li Lv [a,b,c], Lin Lei [a], Qi-Wen Chen [c], Cheng-Li Yin [c], Huiqing Fan [a,*,1], Jian-Ping Zhou [c,*,2,3]

[a] State Key Laboratory of Solidification Processing, School of Materials Science and Engineering, Northwestern Polytechnical University, Xi'an 710072, People's Republic of China

[b] School of General Education, Xi'an Mingde Institute of Technology, Xi'an 710124, People's Republic of China

[c] School of Physics and Information Technology, Shaanxi Normal University, Xi'an 710119, People's Republic of China

A R T I C L E I N F O

Keywords:
Photocatalysis
NO removal
Heterojunction
Oxygen vacancy
Doping

A B S T R A C T

Monoclinic phase $La_2Ti_2O_7$ and orthorhombic phase $Bi_4Ti_3O_{12}$ with layered crystal structure constructed by the perovskite slab were widely used in photocatalysis. Their electronic structures are the most crucial factor in high photocatalytic activity. Bi-doped $La_2Ti_2O_7$ gradually converts to La-doped $Bi_4Ti_3O_{12}$ with the incorporation of bismuth ions, and the two phases build an S-scheme heterojunction. More oxygen vacancies were introduced into the S-scheme heterojunction after heat treatment in a nitrogen atmosphere. The 0.1BTO/LTO-OV sample exhibits the largest NO removal efficiency of 52% and only 5.6 ppb NO_2 intermediate generation. The improved NO removal efficiency was ascribed to the synergistic effect of oxygen vacancies, doping and heterojunction, providing a new insight into the photocatalytic NO removal. The photocatalytic mechanism was eventually proposed on the basis of trapping experiments.

1. Introduction

Modern industry brings about a series of air pollution to severely deteriorate the sustainable development of human world. The various air pollutants include carbon dioxide (CO_2), nitrogen oxide (NO_x), sulfur dioxide (SO_2) and volatile organic compounds (VOCs) [1]. Among them, nitric oxide (NO) is the most dangerous pollutant because it can cause atmospheric environmental problems, endanger human health and destroy the environment ecosystem. But the NO concentration in atmosphere increases continuously in the past decades because of the rapid development of industry and transportation. Therefore, it is urgent to develop green and sustainable strategies for NO removal. Semiconductor photocatalytic technology has stood out in the degradation of ppb concentrations of NO due to its high efficiency, economical cost and environmental protection. However, a certain amount of NO_2 gas is inevitable as the intermediate product during the photocatalytic degradation of NO gas. In fact, the intermediate NO_2 ought to be also removed as far as possible during the NO treatments since NO_2 is even more toxic than NO [2,3].

Heterojunctions combining two photocatalysts are demonstrated to effectively improve photocatalytic performance by relieving the recombination of photogenerated carriers in a single photocatalyst [4,5]. S-scheme heterojunction composed of a reduction semiconductor and an oxidation semiconductor was recently proposed with suitably staggered band configurations. The oxidation photocatalyst owns a more positive valance band position, while the reduction photocatalyst has a more negative conduction band position [6,7]. The S-scheme heterojunctions retain the redox capability of both semiconductors in comparison with conventional type-II heterojunctions and enhance the charge transfer at interface by the built-in electric field to promote photocatalytic efficiency. Oxygen vacancies, with abundant localized electrons, have been demonstrated to serve as electron trap centers that can effectively capture and activate inert gas molecules such as CO_2, N_2 and NO gas [3,8–10]. At the same time, the oxygen vacancy with

* Corresponding authors.
E-mail addresses: hqfan@nwpu.edu.cn (H. Fan), zhoujp@snnu.edu.cn (J.-P. Zhou).
[1] ORCID: 0000–0002-1405–9279
[2] ORCID: 0000–0003-0807–1404
[3] ResearcherID: AGE-2972–2022

https://doi.org/10.1016/j.apcatb.2023.123464
Received 13 July 2023; Received in revised form 11 October 2023; Accepted 2 November 2023
Available online 4 November 2023
0926-3373/© 2023 Elsevier B.V. All rights reserved.

appropriate concentration can also improve light absorption and promote the separation of photogenerated electrons and holes [3].

The perovskite $La_2Ti_2O_7$ is built by a stack of four layers of corner-sharing $[TiO_6]$ octahedron along the *c* axis, linked to a neighboring slab by lanthanum cations lying near the boundary [11,12]. The perovskite $La_2Ti_2O_7$ possesses many unique properties, such as superior thermal stability, good electron mobility, high conductivity and ferroelectric property. Therefore, it has been investigated as a functional material in various applications like photocatalytic hydrogen evolution [13], Lithium-Ion Batteries [14], sensors [15], storage devices [16] and photodegradation [17,18]. However, its large band gap (~ 3.6 eV) prevents photocatalytic efficiency in visible light. As an ionic conductor at room temperature, $La_2Ti_2O_7$ shows a high ion mobility due to the ions on A sites, which can easily migrate to thermodynamic equilibrium locations. Hence, $La_2Ti_2O_7$ provides a good parent material for chemical substitutions maintaining the original perovskite structure. The ions doped on the A sites tend to be segregated at locations with lower formation energy. Bi^{3+} cation is a good candidate to dope $La_2Ti_2O_7$ photocatalyst because the intrinsic polarization induced by the Bi 6s lone pair electron is beneficial to the separation of photogenerated electrons and holes [19]. The ferroelectric bismuth titanate ($Bi_4Ti_3O_{12}$), as a member of the Aurivillius family, is constructed by alternate stacking of $(Bi_2O_2)^{2+}$ layers and perovskite $(Bi_2Ti_3O_{10})^{2-}$ layers along the *c* axis [20–22], which form an internal electric field to promote the separation of photogenerated electrons and holes, thereby enhancing the photocatalytic activity. Nevertheless, the large band gap of $Bi_4Ti_3O_{12}$ (~3 eV) limits its light absorption, reducing the photocatalytic performance as a photocatalyst. Foreign element doping can effectively improve the photocatalytic activity of semiconductors by introducing dopant energy levels in the bandgap. And carrier trapping centers efficiently prevent the recombination of photogenerated electrons and holes [21,23]. Considering the chemical state and ionic radius, we equivalently doped La^{3+} ions (1.032 Å) to substitute Bi^{3+} ions (1.03 Å) in $Bi_4Ti_3O_{12}$.

In this study, we prepared S-scheme heterojunctions of Bi-doped $La_2Ti_2O_7$ and La-doped $Bi_4Ti_3O_{12}$. Then, oxygen vacancies were introduced into the heterojunction to promote photocatalytic performance. The improved NO gas removal avoiding NO_2 secondary pollution was systematically researched. This work provides hopeful insights into designing oxygen vacancies-modified and metal ion-doped S-scheme heterojunction with highly efficient photocatalysis.

2. Experimental details

2.1. Sample preparation

The Bi-doped $La_2Ti_2O_7$, La-doped $Bi_4Ti_3O_{12}$ and their heterojunctions were prepared by a normal hydrothermal method [18]. All the raw reagents were analytically pure. The appropriate molar quantities of $La(NO_3)_3 \cdot 6H_2O$, $Bi(NO_3)_3 \cdot 5H_2O$ and $Ti(SO_4)_2$ were dissolved in deionized water under magnetic stirring until a clear aqueous solution. 1 mol KOH solution was dropped into the above solution to form a white precipitation mixture. The mixture was stirred for 30 min and then sealed in a 30 mL Teflon-lined stainless-steel autoclave to react at 220 °C for 36 h. After reaction, the resulting powders were repeatedly washed several times with deionized water and then dried at 80 °C for 12 h to get white powders. The samples with different Bi/(La+Bi) molar ratios of 0.01, 0.05, 0.1, 0.2, 0.3 and 0.4 are denoted as 0.01BLTO, 0.05BTO/LTO, 0.1BTO/LTO, 0.2BTO/LTO, 0.3BTO/LTO and 0.4LBTO, respectively.

1 g composite 0.1BTO/LTO power and 4 g $NaBH_4$ were ground together for 30 min in an agate mortar. Then, the composite powers were put in an open crucible to heat at 350 °C for 1 h under the 100 mL/min flow of N_2 gas. After heat treatment, the resulting powders were repeatedly washed several times with deionized water and then dried at 80 °C for 10 h to get gray powder, which was denoted as 0.1BTO/LTO-OV.

2.2. Measurements

The crystal structure of the samples was identified by an X-ray diffractometer (XRD, D/Max2550, Rigaku, CuKα radiation, accelerating voltage of 40 kV, applied current of 100 mA and scan rate of 5°/min) and Raman spectroscopy (Bruker Optics Senterra, laser wavelength of 532 nm and power of 2 mW). The powder morphology was observed by a field emission scanning electron microscope (FESEM, Nova Nano SEM 450). The crystal characteristics were further determined by field emission transmission electron microscopy (TEM, Talos F200i) combined with selected area electron diffraction (SAED). X-ray photoelectron spectroscopy (XPS) was measured with an instrument ESCALAB 250Xi (Kratos Analytical Ltd, Japan). All binding energies were calibrated with C 1s peak of adventitious carbon at 284.6 eV. UV–vis absorption spectra were recorded by a Lambda 950 UV–vis-NIR spectrophotometer (Perkin-Elmer, USA) in the wavelength range of 200 – 800 nm. The Mott-Schottky, photocurrent and electrochemical impedance were measured with an electrochemical workstation (CHI660E, Chenhua Instrument Co., Shanghai, China). Specific surface areas of the samples were measured with nitrogen adsorption-desorption isotherms at 77 K by a Micromeritics ASAP 2010 system after being preheated at 120 °C for 12 h in a vacuum. The Fourier transform infrared (FT-IR) spectra were recorded on a Tensor27 spectrophotometer (Bruker, Germany) between 400 and 4000 cm^{-1}. The in situ electron spin resonance (ESR) signals were measured by a Bruker EMXplus spectrometer to detect the free radicals by mixing the as-prepared samples with 25 mM of 5,5′-dimethyl-1-pyrroline n-oxide (DMPO) solution and 2,2,6,6-tetramethylpiperidine (TEMP) trapping agent.

The photocatalytic activity of samples was evaluated by NO removal at ambient temperature in a continuous flowing NO gas with ppb level. 0.1 g sample was evenly dispersed in a glass dish (10 cm in diameter) via deionized water and ultrasonic treatment for 10 min, then dried under 60 °C in the oven for 12 h. The photo-oxidative NO removal was performed in a quartz reactor (4.5 L, 10 × 30 × 15 cm) under irradiation of a 300 W Xenon lamp, which is 20 cm above the glass dish. The concentration of NO gas was recorded by a NO-NO_2-NO_x analyzer (42i, Thermo Scientific) per minute. An entire measurement was conducted at room temperature and relative humidity of 30 ± 5%. After the adsorption-desorption balance was achieved, the sample was illuminated.

The removal rate (η) of NO was calculated with Eq. (1).

$$\eta = 1 - (C/C_0) \times 100\% \quad (1)$$

where C is the NO concentration at a given time and C_0 is the initial concentration of NO with ppb. In the trapping experiments, 10 mg KI, $K_2Cr_2O_7$, β-carotene and PBQ and 10 mL of IPA were separately mixed with 0.1 g photocatalyst powder in 15 mL deionized water under sonication for 15 min and then, deposited onto the glass substrates for measurement.

The processes of adsorption and reaction of NO over 0.1BTO/LTO-OV were investigated using an in situ diffuse reflectance infrared Fourier-transform spectroscopy (DRIFTS) (TENSOR II, Bruker). The sample was sealed and degassed in a specimen chamber with high-purity helium (He) gas for 1 h to remove the air and impurities on catalyst surface. Subsequently, NO and O_2 were admitted into the reaction chamber and then spectra were recorded every two minutes in the dark and under illumination to research the adsorption and reaction processes.

2.3. Computational calculation

The formation energies and electronic structures of the single-phase doped materials were calculated by the Vienna ab initio simulation package program (VASP) [24] embedded in MedeA® software [25].

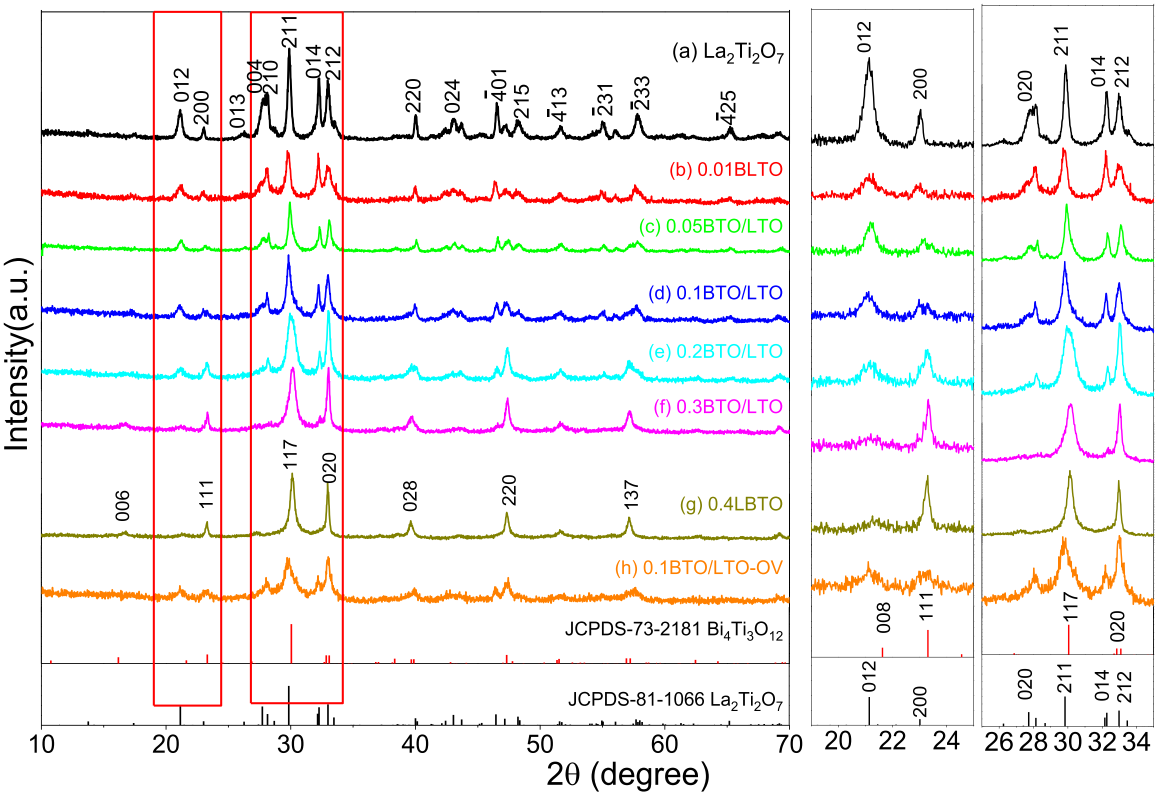

Fig. 1. XRD patterns of (a) pure $La_2Ti_2O_7$, (b) Bi-doped $La_2Ti_2O_7$, (c – f) heterojunctions, (g) La-doped $Bi_4Ti_3O_{12}$ and (h) 0.1BTO/LTO-OV, and their enlarged patterns near 22° and 30°.

Projector augmented wave potentials [26] were employed during calculations based on the density functional theory and generalized gradient approximation (GGA) with the Perdew-Burke-Ernzerhof (PBE) functional [27] to describe the interactions. The default cutoff energy of plane wave is 450 eV, and the electronic iteration convergence criterion is that the energy of each two-step iteration is ultimately less than 1×10^{-4} eV. The required k-spacing in reciprocal space is 0.3/Å, and the optimized structure meets the criterion of 0.02 eV/Å.

3. Results and discussions

3.1. Structural and morphological analysis

The crystal structure of photocatalysts was characterized by XRD as shown in Fig. 1. The pure $La_2Ti_2O_7$ has a monoclinic structure with space group P2$_1$(4), $a = 7.81$ Å, $b = 5.55$ Å, $c = 13.02$ Å and $\beta = 98.43°$ (JCPDS No. 81–1066). The ionic radius of La^{3+} (103.2 pm) is close to that of Bi^{3+} (103 pm), providing a possibility to replace La^{3+} cations with Bi^{3+} cations in the $La_2Ti_2O_7$ lattice. The sample keeps $La_2Ti_2O_7$ single phase at low Bi/(La + Bi) molar ratio of 0.01 to be Bi-doped $La_2Ti_2O_7$ powders. $Bi_4Ti_3O_{12}$ phase appears with the increase in Bi/(La+Bi) molar ratio, and the $La_2Ti_2O_7$ peaks gradually weaken until disappear to become the orthorhombic $Bi_4Ti_3O_{12}$ phase with space group Fmmm, $a = 5.46$ Å, $b = 5.44$ Å and $c = 33.17$ Å (JCPDS No. 73–2181). The samples keep the coexistence of monoclinic and orthorhombic phases at the Bi/(La+Bi) molar ratios of 0.05, 0.1, 0.2 and 0.3. The La-doped $Bi_4Ti_3O_{12}$ single phase is obtained at the molar ratio of 0.4. The phase transition can be attributed to the doping-induced lattice distortion and tilting of octahedral by the incorporation of bismuth ions during the hydrothermal process [14]. The 0.1BTO/LTO-OV sample still keeps the same phases with 0.1BTO/LTO after introducing oxygen vacancies. However, its peak intensity becomes weak, indicating poor crystallinity caused by the oxygen vacancies, which will supply more active sites during the photocatalytic reaction.

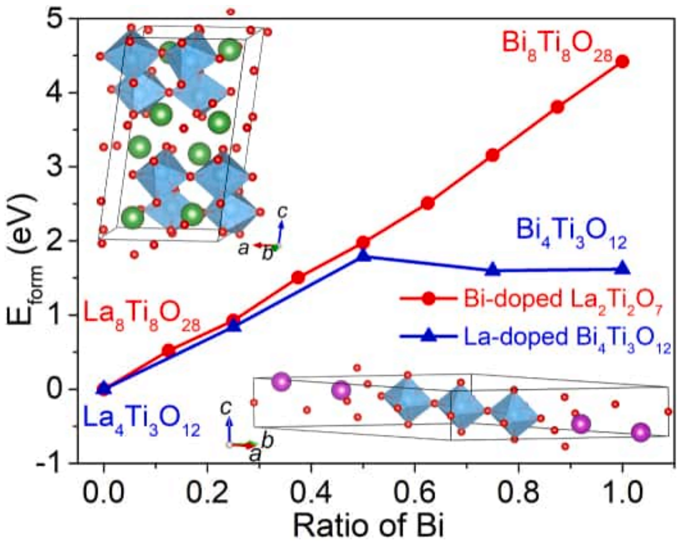

Fig. 2. Formation energy of $La_2Ti_2O_7$ and $Bi_4Ti_3O_{12}$ crystal structures with relative content of Bi in the doping process. The insets show the structural models used for calculation.

We calculated the formation energy of Bi-doped $La_2Ti_2O_7$ and La-doped $Bi_4Ti_3O_{12}$ to understand the phase change by density functional theory. The formation energy of doping materials is expressed as [28].

$$E_{form} = E_{dope} - E_{bulk} - n\mu_M + n\mu_N \tag{2}$$

where E_{dope} and E_{bulk} are the total energy after and before doping an atom, respectively. μ_M is the chemical potential of doping element, and

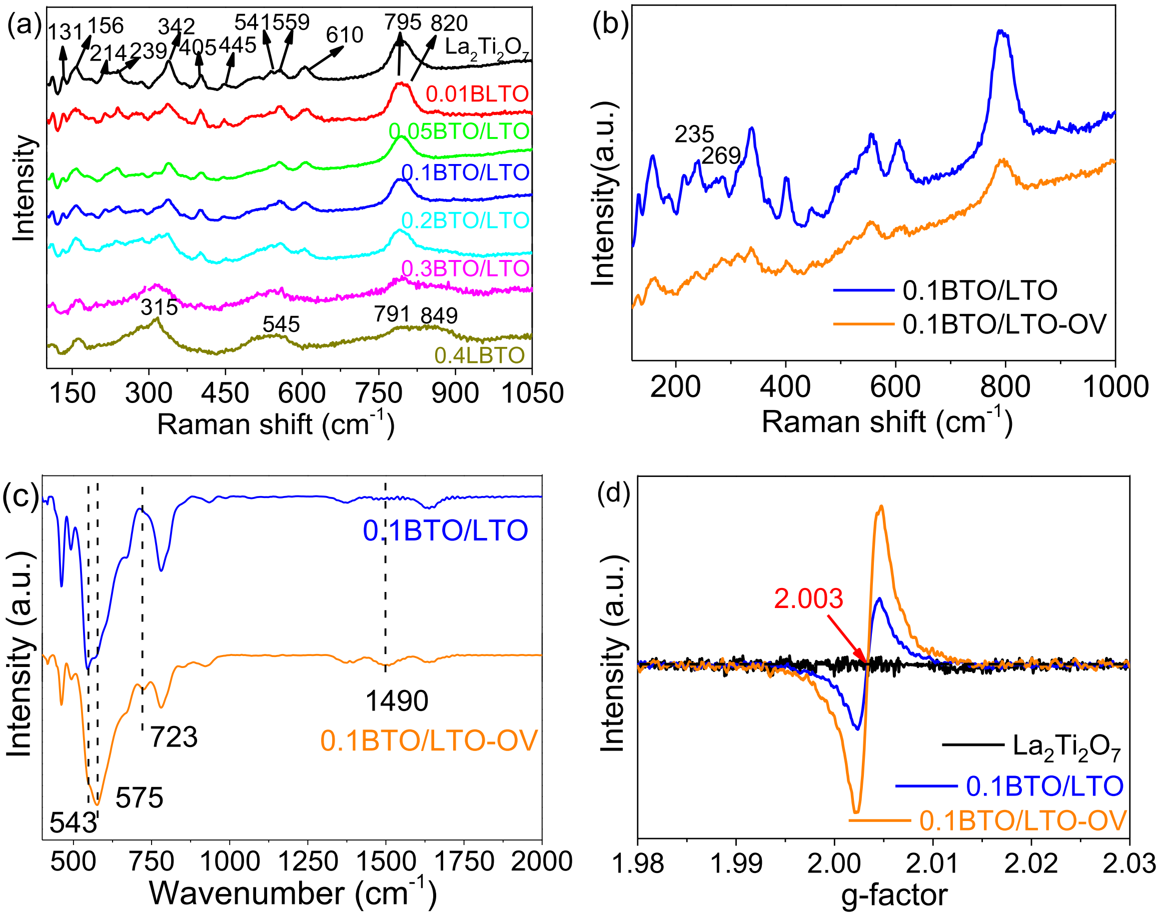

Fig. 3. (a) Raman spectra of pure $La_2Ti_2O_7$, Bi-doped $La_2Ti_2O_7$, La-doped $Bi_4Ti_3O_{12}$ and heterojunctions. (b) Raman spectra and (c) FT-IR spectra of 0.1BTO/LTO and 0.1BTO/LTO-OV. (d) ESR spectra of $La_2Ti_2O_7$, 0.1BTO/LTO and 0.1BTO/LTO-OV for comparison.

μ_N is the chemical potential of substituted atom. n is the number of substitutions. The chemical potential μ_M is obtained by the corresponding metal oxide with the following method

$$\mu_M = \left(\mu_{M_aO_b} - b\mu_O\right)/a \quad (3)$$

where $\mu_{M_aO_b}$ represents the chemical potential of the corresponding metal oxide, and$\mu_O = \mu_{O_2}/2$ is the chemical potential of oxygen. Then, we get $\mu_{Bi} = -6.953$ eV and $\mu_{La} = -13.603$ eV.

Fig. 2 shows the formation energies of Bi-doped $La_2Ti_2O_7$ and La-doped $Bi_4Ti_3O_{12}$. The formation energy of $La_2Ti_2O_7$ increases with the Bi doping content, meaning La ions are difficultly substituted with Bi ions in the $La_2Ti_2O_7$ lattice. The formation energy of $Bi_4Ti_3O_{12}$ is steady at low La doping content and decreases at high La content, suggesting La ions easily enter into the $Bi_4Ti_3O_{12}$ lattice to replace Bi ions. This confirms the XRD results that the $Bi_4Ti_3O_{12}$ phase appears at a low Bi/(La+Bi) molar ratio of 0.05 due to the low Bi doping content in $La_2Ti_2O_7$ phase and the powder retains La-doped $Bi_4Ti_3O_{12}$ phase at a Bi/(La+Bi) molar ratio of 0.4 due to the high La doping content in $Bi_4Ti_3O_{12}$ phase.

Raman spectroscopy is sensitive to the fluctuation in internal crystal structure of samples. Fig. 3(a) shows the Raman peaks at 131, 156, 214, 239, 342, 405, 445, 541, 559, 610, 795 and 820 cm^{-1} correspond to the typical monoclinic layered perovskite crystalline structure [13,29]. The bands at 156, 239, 342 and 405 cm^{-1} are most likely from the La-O bond vibration. The stretching of octahedral O-Ti-O bonds can be found at 445 (E_g mode), 610 and 795 cm^{-1}. The peaks at 131 (B_{1g} mode) and 820 (B_{2g} mode) cm^{-1} are similar to the Ti-O bond stretching vibration for the 6-fold coordinated Ti in rutile TiO_2 [30,31]. The fine Raman peaks confirm the formation of monoclinic layered perovskite phase $La_2Ti_2O_7$ doped with Bi element. The Bi doping could result in crystal distortion in $La_2Ti_2O_7$ lattice and generate crystal defects. The peaks become broader and shift towards high wavenumber with the increase in Bi content.

The Raman peaks of 0.4LBTO at 315, 545 and 849 cm^{-1} correspond to the typical orthorhombic layered perovskite structure [32,33]. $Bi_4Ti_3O_{12}$ with orthorhombic crystal structure has 24 ($6A_g + 2B_{1g} + 8B_{2g} + 8B_{3g}$) Raman active vibration modes. However, less number of peaks are observed in the 0.4LBTO Raman spectrum because the La doping. The high-frequency bands originate from the various stretching and bending vibrations of TiO_6 octahedra in the perovskite $[Bi_2Ti_3O_{10}]^{2-}$ layers. The bands at 849, and 315 cm^{-1} can be ascribed to the A_{1g} mode, reflected from the octahedral stretching mode of O-Ti-O stretching vibration. The bands at 545, 269 and 235 cm^{-1} are from B_{2g} and B_{3g} modes. The B_{3g} mode is generally caused by TiO_6 octahedron lattice strain or partial oxygen vacancy. The peaks below 220 cm^{-1} are from the vibration of Bi and O atoms in the $[Bi_2O_2]^{2+}$ layers [20,34]. The band at 791 is a new peak after doping La ions. The peaks of 0.1BTO/LTO-OV are weaker than that of 0.1BTO/LTO as shown in Fig. 3(b), meaning the deformation of crystal structure and disorder surface damages induced by the oxygen vacancies [35,36].

Fig. 3(c) shows the FT-IR spectra of part samples. The vibration peaks at 575 and 543 cm^{-1} are characterized as Ti-O and La-O stretching vibrations, respectively [13,23,37]. The vibration peak of La-O of 0.1BTO/LTO-OV becomes weak in comparison with 0.1BTO/LTO, indicating the lattice oxygen vacancies mainly appear around La ions after heat treatment in the nitrogen atmosphere. New peaks at 1490 cm^{-1} and 723 cm^{-1} are attributed to the stretching C-O and H-O-H bonds of the species [38,39] absorbed by the surface oxygen vacancies. The solid-state ESR spectra are measured to further confirm the oxygen

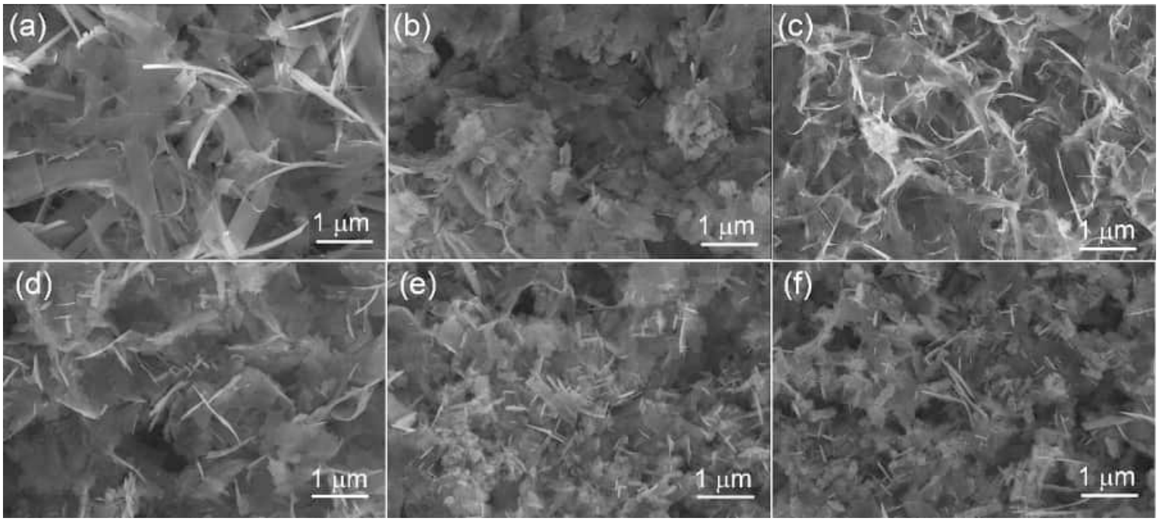

Fig. 4. SEM images of (a) pure $La_2Ti_2O_7$, (b) 0.05BTO/LTO, (c) 0.1BTO/LTO, (d) 0.2BTO/LTO, (e) 0.3BTO/LTO and (f) 0.4LBTO.

defects in samples. Fig. 3(d) shows both 0.1BTO/LTO and 0.1BTO/LTO-OV exhibit a single Lorentz line with a g-value of around 2.003 from oxygen vacancy [3,8,9,36]. However, the pure $La_2Ti_2O_7$ does not exhibit a significant oxygen vacancy characteristic peak, indicating that the oxygen defects in 0.1BTO/LTO can be attributed to the crystal distortion in $La_2Ti_2O_7$ and $Bi_4Ti_3O_{12}$ lattices induced by Bi and La doping, respectively [40,41]. The ESR signal is much enhanced in 0.1BTO/LTO-OV powders, confirming more oxygen vacancies via heat treatment in the nitrogen atmosphere.

Fig. 4 displays typical SEM images of part samples. Pure $La_2Ti_2O_7$ shows regular long ultrathin nanosheets stacked together. With the increase in Bi concentration, the samples transfer to irregular nanosheets with different sizes, which inherit the original layered structure of $La_2Ti_2O_7$. Other ultrathin nanosheets appear in the composite sample, belonging to the orthorhombic $Bi_4Ti_3O_{12}$ phase [42,43]. The uniform cross structure consists of self-assembled nanosheets. Part amorphous nanoparticles assemble into a compact cross before the crystallization. This array configuration could greatly reduce the surface energy of the nanosheets.

Fig. 5 shows TEM images of the as-prepared 0.1BTO/LTO and 0.3BTO/LTO composites. 0.1BTO/LTO powder in Fig. 5(a) has irregular nanosheets similar to the SEM images. The SAED pattern well meets the monoclinic $La_2Ti_2O_7$ with (0 2 0) and (2 0 0) crystal planes and their included angle of 90°, indicating the main $La_2Ti_2O_7$ phase in the 0.1BTO/LTO powder. But the $Bi_4Ti_3O_{12}$ phase was captured by the XRD pattern. The corresponding EDS elemental mappings show the Bi ions are evenly distributed in the sample, suggesting Bi^{3+} ions replace part La^{3+} ions in $La_2Ti_2O_7$ lattice. Fig. 5(b) clearly displays the cross structure of 0.3BTO/LTO composite. The insets show its high-resolution transmission electron microscopy (HRTEM) images and corresponding SAED patterns. There are two different SAED patterns. One is from the orthorhombic $Bi_4Ti_3O_{12}$ phase with (0 0 12) and (0 2 0) crystal planes and their included angle of 90°. Another meets the monoclinic $La_2Ti_2O_7$ structure with ($\bar{4}$ 0 1) crystal plane. The lattice fringes with *d* spacings of 0.277 and 0.212 nm in HRTEM are assigned to the (0 2 0) and ($\bar{2}$ 1 5) planes of $La_2Ti_2O_7$, respectively. The interplane distance of 0.297 nm well matches the (1 1 7) plane of $Bi_4Ti_3O_{12}$. The (0 2 0) plane of $La_2Ti_2O_7$ is close to the (1 1 7) plane of $Bi_4Ti_3O_{12}$, providing a foundation to form a heterojunction between $La_2Ti_2O_7$ and $Bi_4Ti_3O_{12}$. The interfacial connections with lattice crossing through two phases could serve as migration paths to facilitate charge separation and improve the synergistic effects for photocatalytic performance. The corresponding EDS elemental mappings reveal La, Bi, Ti and O are uniformly distributed throughout the nanosheet-assembled hierarchical architectures, suggesting the uniform Bi doping in $La_2Ti_2O_7$ lattice and La doping in $Bi_4Ti_3O_{12}$ lattice.

Fig. 6(a) presents the XPS full spectra of samples. La 3d spectra in Fig. 6(b) show doublet peaks from La $3d_{3/2}$ and La $3d_{5/2}$ states with good symmetry, indicating a La^{3+} oxidation state [44,45]. The La^{3+} characteristic doublets of Bi-doped $La_2Ti_2O_7$, La-doped $Bi_4Ti_3O_{12}$ and heterojunctions positively shift to higher binding energy by ~0.3 eV in comparison with the pure $La_2Ti_2O_7$, meaning a loss of electrons. The symmetric peaks at 463.8 and 458.1 eV of pure $La_2Ti_2O_7$ in Fig. 6(c) are from Ti $2p_{1/2}$ and $2p_{3/2}$ states, assigned to the Ti^{4+} in TiO_6 octahedral slabs [44]. After introducing Bi ions, its binding energies shift to 464.1 and 458.4 eV, also suggesting a decrease in electron density due to the formation of O-Ti-Bi bonds [22]. The O 1 s spectra of samples are fitted to three peaks at around 529.5, 531.0 eV and 532.7 eV as shown in Fig. 6 (d). The peak at 529.5 eV belongs to the oxygen-metal bonds in crystal lattices [46]. The weak peaks at around 532.7 eV refer to OH^- species from the adsorbed hydroxyl groups H_2O and CO_3^{2-} [4,47]. The middle peak can be associated with oxygen in crystal lattices near the oxygen vacancies [35,48]. Clearly, this peak of 0.1BTO/LTO-OV shifts to 530.7 eV and becomes stronger, further confirming more oxygen vacancies after heat treatment in a nitrogen atmosphere. The binding energies of 164.1 and 158.8 eV in Fig. 6(e) are attributed to Bi 4 $f_{5/2}$ and 4 $f_{7/2}$. The binding peaks of heterojunctions negatively shift to low energy in comparison with those of La-doped $Bi_4Ti_3O_{12}$ phase, indicating $Bi_4Ti_3O_{12}$ receives electrons. The change in binding energy means electrons migrate from Bi-doped $La_2Ti_2O_7$ to La-doped $Bi_4Ti_3O_{12}$ to build an electric field from $Bi_4Ti_3O_{12}$ to $La_2Ti_2O_7$ to be an S-scheme heterojunction. The peaks of 0.1BTO/LTO-OV shift to low energy due to more oxygen vacancies in the sample.

3.2. *Photoelectric properties and energy band structure analysis*

The optical properties of samples were characterized by UV–vis absorption spectra as shown in Fig. 7(a). The absorption edge of pure $La_2Ti_2O_7$ is at about 320 nm, indicating that $La_2Ti_2O_7$ is difficult in response to visible light. The Bi-doped $La_2Ti_2O_7$ samples show a redshift. Bi ions into $La_2Ti_2O_7$ lattice introduce metal impurity energy levels at the bottom of conduction band of $La_2Ti_2O_7$, reducing its band gap to promote light absorption [49]. 0.4LBTO exhibits a weak absorption peak in the 500 – 540 nm wavelength. The lattice will distort after part substitution of trivalent cations in the crystal lattice, resulting in surface oxygen vacancies [40]. The donor energy levels are introduced in the $Bi_4Ti_3O_{12}$ band with the oxygen vacancies, reducing its band gap and causing the weak absorption peak [50]. Similarly, there should be a peak

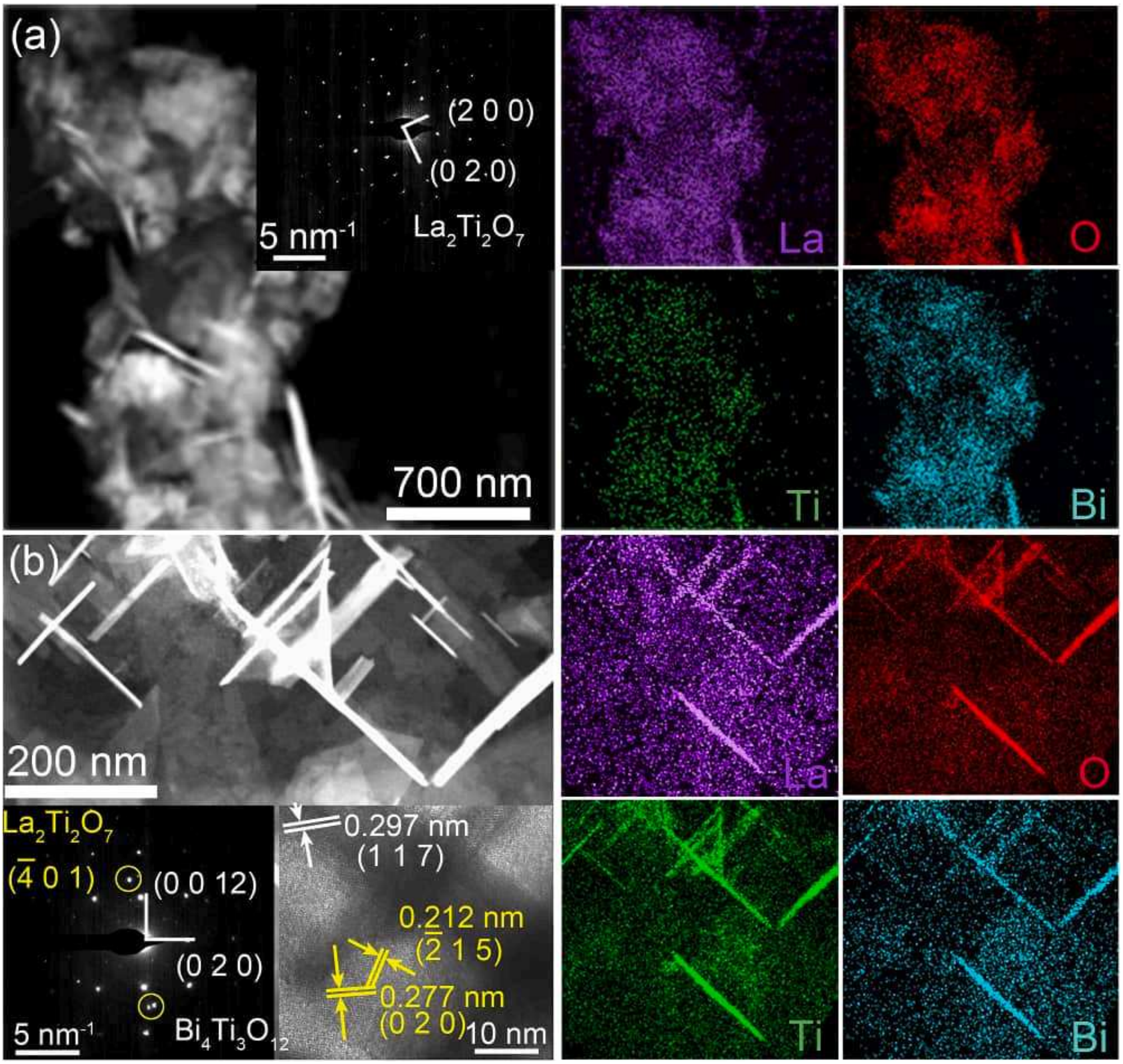

Fig. 5. TEM images of (a) 0.1BTO/LTO and (b) 0.3BTO/LTO and their EDS elemental mapping images. The insets show the corresponding SAED images and HRTEM images.

in the UV–vis absorption spectra of Bi-doped $La_2Ti_2O_7$ sample. But there is no clear peak in the spectra due to the low Bi doping content. The composite photocatalysts combine both characteristics, showing two absorption peaks, especially for 0.3BTO/LTO. 0.1BTO/LTO-OV has a further redshift and higher absorption intensity in the visible range after introducing more oxygen vacancies. The construction of oxygen vacancies into semiconductors can effectively promote their light absorption [2,51]. 0.1BTO/LTO-OV exhibits two clear absorption peaks based on 0.1BTO/LTO sample. They are caused by the oxygen vacancies that produce donor levels in the two semiconductors, which promotes the light-capturing capability [51]. The oxygen vacancies were confirmed by the ESR result as shown in Fig. 3(d).

The energy band gap of semiconductor meets the following formula.

$$\alpha h\nu = A(h\nu - E_g)^{n/2} \quad (4)$$

where α, ν, E_g and A are the absorption coefficient, light frequency, band gap energy and a constant, respectively. n depends on the mode of photon transition in semiconductors. $n = 1$ or 3 is a direct transition semiconductor while $n = 4$ or 6 is an indirect transition semiconductor [52]. n was determined as 1 for the pure phase samples by plotting ln $(\alpha h\nu)$ versus $\ln(h\nu - E_g)$ with the data in high slope region as shown in Fig. 7(b), meaning that both $La_2Ti_2O_7$ and $Bi_4Ti_3O_{12}$ are direct transition semiconductors. Then, the band gap E_g is determined by the intercept on the $h\nu$ axis through linearly fitting $(\alpha h\nu)^2$ versus $h\nu$ as shown in Fig. 7(c). The band gaps of $La_2Ti_2O_7$, 0.01BLTO and 0.4LBTO are 3.82, 3.58 and 3.59 eV, respectively, as shown in Table 1.

The electronic structures were simulated by GGA with PBE embedded in the VASP code for the single-phase samples as shown in Fig. 8. $La_2Ti_2O_7$, $La_7BiTi_8O_{28}$, $Bi_4Ti_3O_{12}$ and $Bi_2La_2Ti_3O_{12}$ are direct transition semiconductors, which are consistent with the experimental results. The bandgap of $La_7BiTi_8O_{28}$ is a little smaller than that of $La_2Ti_2O_7$, which is in agreement with the change between 0.01BLTO and $La_2Ti_2O_7$. But the bandgap of $Bi_2La_2Ti_3O_{12}$ becomes larger than that of $Bi_4Ti_3O_{12}$. This is caused by the difference in atomic electronic structure between La $5d^16s^2$ and Bi $6s^26p^3$. The density of states shows that the valence band maximum is primarily composed of O 2p orbit while the

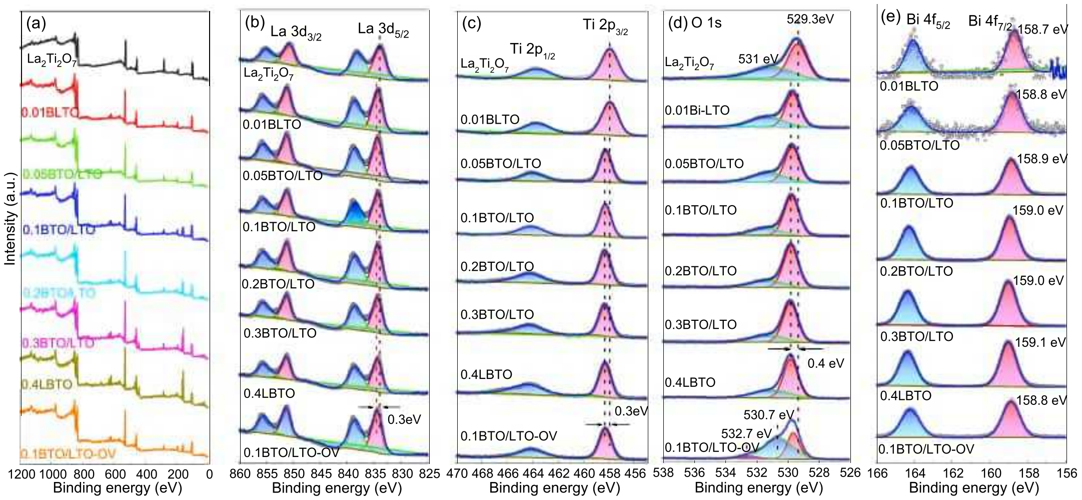

Fig. 6. XPS spectra of pure $La_2Ti_2O_7$, Bi-doped $La_2Ti_2O_7$, La-doped $Bi_4Ti_3O_{12}$ and heterojunctions. (a) Survey spectra, (b) La 3d, (c) Ti 2p, (d) O1s and (e) Bi 4f.

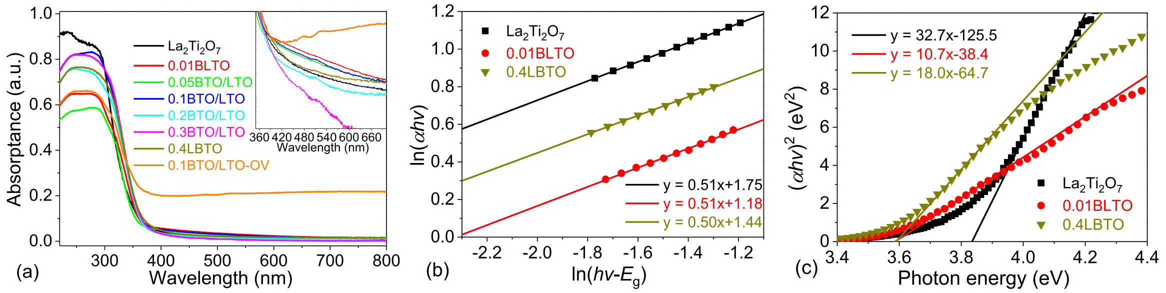

Fig. 7. (a) UV–vis absorption spectra of $La_2Ti_2O_7$, Bi-doped $La_2Ti_2O_7$, La-doped $Bi_4Ti_3O_{12}$ and heterojunctions, where the enlarged spectra show the absorption peaks. (b) Linear relationship between $\ln(hv–E_g)$ and $\ln(\alpha hv)$ of the single-phase samples to determine the semiconductor type, and (c) relationship between $(\alpha hv)^2$ and photon energy to calculate the band gaps.

Table 1
Band parameters and carrier concentration of pure $La_2Ti_2O_7$, Bi-doped $La_2Ti_2O_7$ and La-doped $Bi_4Ti_3O_{12}$.

Samples	Band gap E_g (eV)	Fermi level E_F (eV)	Conduction band E_c (eV)	Valence band E_v (eV)	Carrier concentration N_D (cm^{-3})
$La_2Ti_2O_7$	3.82	− 0.453	− 0.52	3.27	4.31×10^{20}
0.01BLTO	3.58	− 0.578	− 0.66	3.01	6.23×10^{20}
0.4LBTO	3.59	− 0.373	− 0.45	3.14	5.47×10^{20}

conduction band minimum is mainly occupied by Ti 3d orbit.

Fig. 9 shows the Mott-Schottky measurements of single-phase samples to elucidate the flat-band potential (V_{fb}) and carrier concentration (N_D) of semiconductors [53], which satisfy the relationship

$$\frac{1}{C^2} = \frac{2}{\varepsilon\varepsilon_0 e N_D}\left(V - V_{fb} - \frac{k_B T}{e}\right) \quad (5)$$

where C is the semiconductor capacitance per square centimeter, V is the applied voltage, T is the absolute temperature, e is the electronic charge, k_B, ε_0 and ε are the Boltzmann's constant, permittivity in a vacuum and relative permittivity of semiconductor, respectively. Therefore, plots of $1/C^2$ against V should yield a straight line, by which N_D can be conveniently found from the slope. The Fermi energy (E_F) is nearly flat-band potential, which is the intercept of the x-axis. The bottom energy of conduction band E_C and Fermi level E_F in n-type non-degenerate semiconductors obey

$$E_C = E_F + k_B T \ln\frac{N_C}{n_0}, N_C = 2\left(\frac{m_n^* k_B T}{2\pi\hbar^2}\right)^{3/2} \quad (6)$$

where N_C is the effective state density of the conduction band, m_n^* is the effective mass of electrons at the bottom of conduction band (E_c), and $\hbar$ is the reduced Planck constant. Then, we obtain the band parameters as shown in Table 1. The carrier concentration increases after doping the foreign elements, caused by the oxygen vacancies. The measured potentials versus Ag/AgCl electrode can be converted to the normal hydrogen electrode (NHE) potentials using the Nernst equation [40,54]. The conduction band E_c of 0.01BLTO increases to − 0.66 V (vs. NHE) after doping Bi ions, enjoying stronger exchange-reduction ability. Then, the electrons in the conduction band of 0.01BLTO can directly react with $O_2/\bullet O_2^-$ (−0.33 eV vs. NHE). The valence band of 0.4LBTO is + 3.14 eV, which is more positive than both + 1.99 eV vs. NHE for $OH^-/\bullet OH$ and 2.37 eV vs. NHE for $H_2O/\bullet OH$. Then, the holes in the 0.4LBTO valence band can directly react with H_2O to H^+ and active $\bullet OH$ [55].

3.3. Photocatalytic performances

The photocatalytic activities of as-prepared samples were evaluated by photocatalytic NO removal experiments. Fig. 10(a) shows NO can be quickly purified and the removal ratios reach equilibrium in 8 min. The NO removal ratio of pristine $La_2Ti_2O_7$, 0.01BLTO and 0.4LBTO is 23%, 36% and 42% in 8 min, respectively, suggesting photocatalytic performance is improved in the doped samples. The photocatalytic ability of heterojunctions is further enhanced. Among them, 0.1BTO/LTO sample

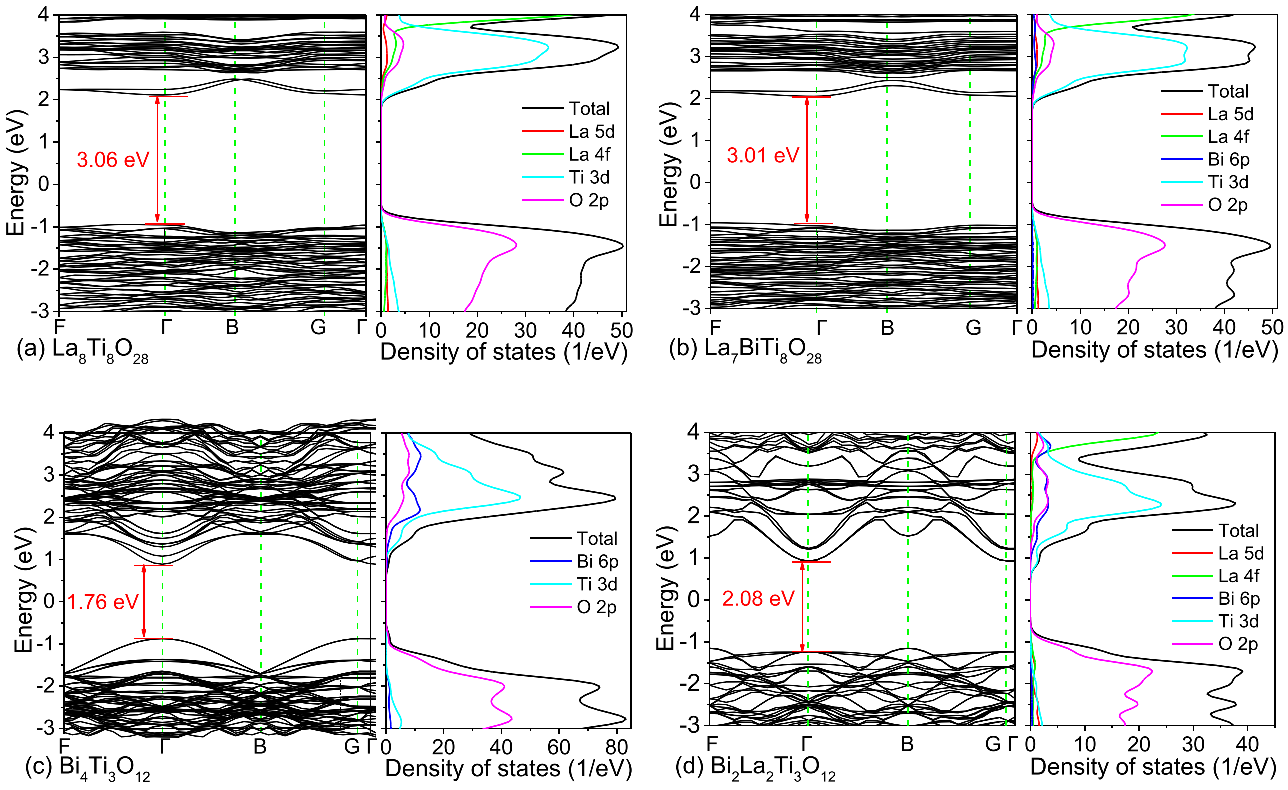

Fig. 8. Calculated band structure and density of states of (a) $La_2Ti_2O_7$, (b) $La_7BiTi_8O_{28}$, (c)$Bi_4Ti_3O_{12}$ and (d) $Bi_2La_2Ti_3O_{12}$, corresponding to the pure phases.

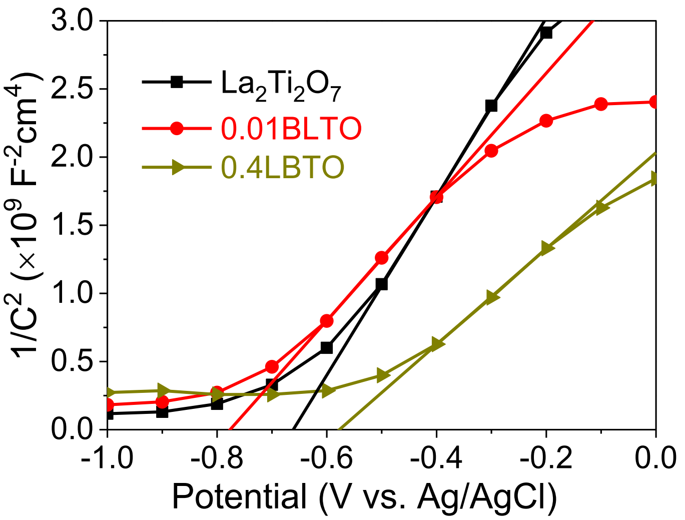

Fig. 9. Mott-Schottky plot of $La_2Ti_2O_7$, Bi-doped $La_2Ti_2O_7$ and La-doped $Bi_4Ti_3O_{12}$.

enjoys the highest NO removal ratio of 48%. This is mainly attributed to the internal electric field at the interface between Bi-doped $La_2Ti_2O_7$ and La-doped $Bi_4Ti_3O_{12}$, which facilitates the separation of photogenerated electron-hole pairs and allows more carriers to migrate to the sample surface to participate in the removal of NO gas [7]. The NO removal ratio is further improved to 55% by 0.1BTO/LTO-OV, ascribed to the more oxygen vacancies in sample after the heat treatment. The introduction of oxygen vacancy can effectively improve the charge transfer rate and electron-hole separation efficiency and provide more active sites to enhance the adsorption and reaction ability of pollutants [56]. Usually, the oxygen vacancies include two types, i.e., Vö′ (trapped one electron) and Vö″ (trapped two electrons). Following the interfacial two-electron charging (Vö″ + O_2 → Vö-O_2^{2-}) and subsequent one-electron back-donation (Vö-O_2^{2-} + NO → Vö-NO_3^- + e^-) process, the back-donated electron could be retrapped by Vö to produce a new single-electron-trapped Vö′, simultaneously triggering a second round of NO oxidation (Vö′-•O_2^- + NO → Vö-NO_3^-) [10]. Therefore, constructing the oxygen vacancies on the 0.1BTO/LTO-OV surface is necessary and has a significant effect on enhancing the photocatalytic performance in photo-oxidative NO removal.

The intermediate product NO_2 during the photo-oxidation is a notorious member in NO_x with even more toxicity and hazardous than

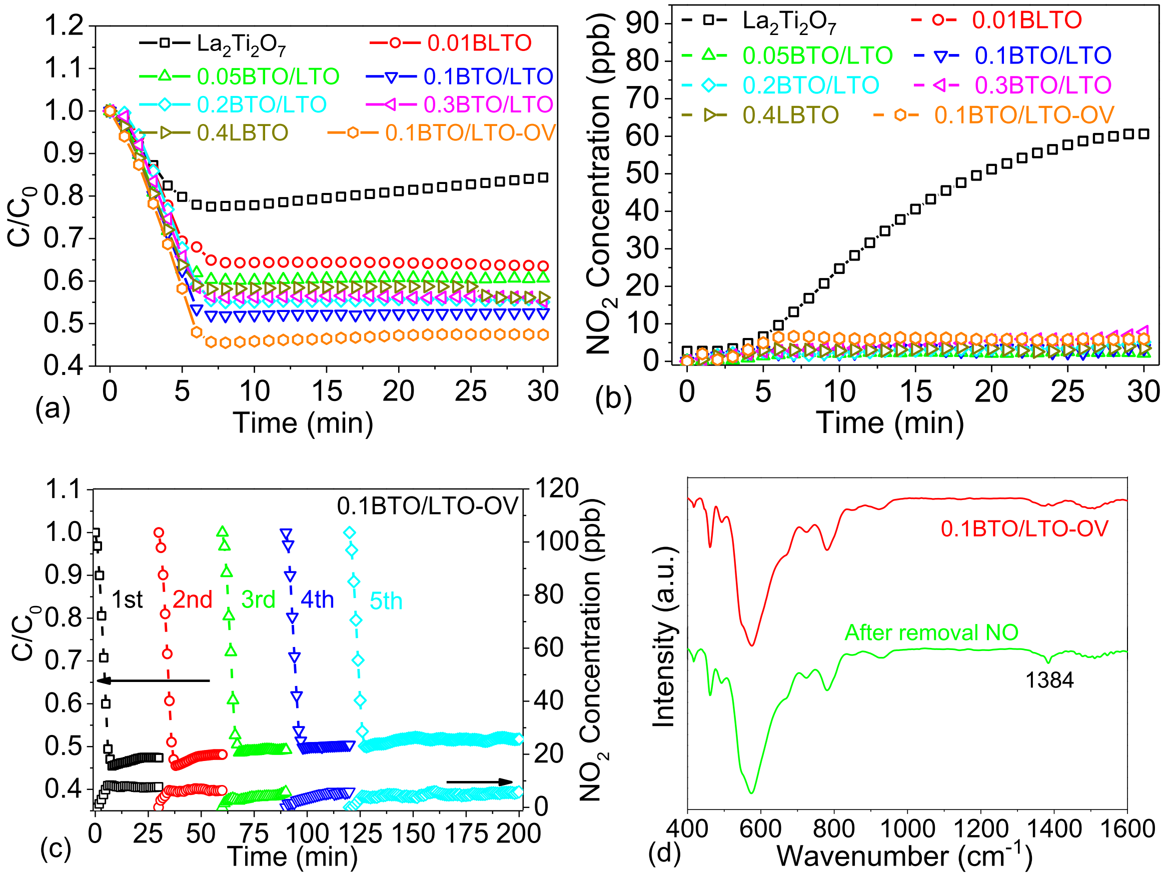

Fig. 10. (a) Photocatalytic NO removal and (b) NO_2 production of pure $La_2Ti_2O_7$, Bi-doped $La_2Ti_2O_7$, La-doped $Bi_4Ti_3O_{12}$ and heterojunctions. (c) Photocatalytic stability of NO removal and NO_2 generation over 0.1BTO/LTO-OV in cyclic tests and (d) FT-IR spectra of 0.1BTO/LTO-OV before and after five photocatalytic NO runs.

Table 2
Comparison of removal performance of NO gas by various photocatalysts.

Photocatalyst	NO source (ppb)	C_0/C	NO_2 generation (ppb)	Light resource	Active species/target products	Year, ref.
$ZnWO_4$	400	0.4	N/A	300 W Xenon lamp	•OH and $•O_2^-/NO_3^-$	2016 [64]
$(BiO)_2CO_3$	430	0.53	15.6	Visible light	$•O_2-$ and •OH/mo-NO_3- and bi-NO_3^-	2021 [65]
Vo-BiOCl	500	0.6	80	Xenon lamp	$•O_2^-/NO_2$ and NO_3^-	2019 [10]
Vo-Bi_2MoO_6 Nanosheets	600	0.47	90	Visible light	e^-, $^1O_2$ and $•O_2^-/NO_2$ and NO_3^-	2022[2]
Bi/BiOBr nanoflowers	800	0.63	29	300 W Xenon lamp	e^-, h^+ and $•O_2^-/NO_2$ and NO_3^-	2023 [60]
$Bi_{12}GeO_{20}/Bi_2S_3$	580	0.46	8	500 W Xenon lamp	e^-, h^+ and $•O_2^-/NO_3^-$	2022[8]
g-C_3N_4	600	0.57	50	Visible light	h^+ and e^-/NO_3^-	2022 [66]
g-C_3N_4 microtubes	400	0.33	25	Visible light	$•O_2^-$ and h^+/NO_3^-	2019 [67]
Pd/g-C_3N_4	600	0.75	60	Visible light	h^+, $•O_2^-$ and $•OH/NO_3^-$	2022 [68]
$CaCO_3$/g-C_3N_4	550	0.51	120	Visible light	$^1O_2$, •OH and $•O_2^-/NO_2$ and NO_3^-	2023 [61]
g-C_3N_4/TiO_2-Ti_3C_2	500	0.61	35	300 W Xenon lamp	e^-, $•O_2^-$ and $•OH/NO_3^-$	2021 [69]
3D–2D $Bi_4O_5Br_2$-GO	550	0.54	20	Visible light	e^- and $•O_2^-/NO_3^-$	2021 [70]
$Bi_2Ti_2O_7/CaTiO_3$	600	0.77	70	Visible light	$•O_2^-$/ NO_3^-	2022[3]
$TiO_{2-\delta}$/Carbon	430	0.55	13	Visible light	$•O_2^-$ and $•OH/NO_3^-$	2021 [71]
BiOBr/BiOI	600	0.57	50	Visible light	h^+, $•O_2^-$ and $^1O_2/NO_3^-$	2019 [72]
Bi-doped $La_2Ti_2O_7$/La-doped $Bi_4Ti_3O_{12}$ S-scheme heterojunction	430	**0.52**	**5.6**	300 W Xenon lamp	e^-,$^1O_2$, h^+, $•O_2^-$ and $•OH/NO_3^-$	This work

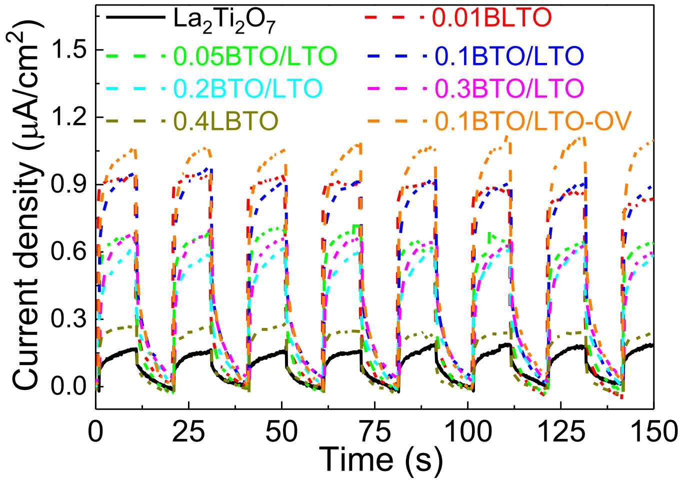

Fig. 11. (a) Photocurrents of the pure $La_2Ti_2O_7$, Bi-doped $La_2Ti_2O_7$, La-doped $Bi_4Ti_3O_{12}$ and heterojunctions.

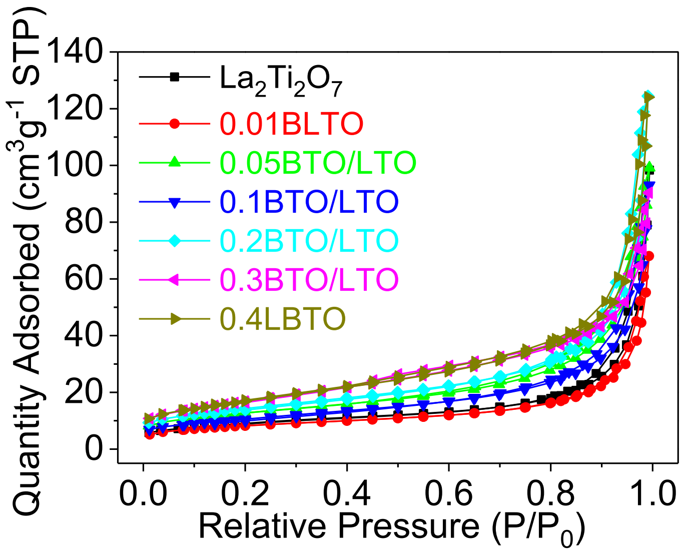

Fig. 12. Nitrogen adsorption-desorption curves for the pure $La_2Ti_2O_7$, Bi-doped $La_2Ti_2O_7$, La-doped $Bi_4Ti_3O_{12}$ and heterojunctions at 77 K.

the NO gas [2,3]. NO_2 production was also monitored during the NO removal reaction as shown in Fig. 10(b). The NO_2 yields of *x*BTO/LTO are much lower than that of pure $La_2Ti_2O_7$ (60 ppb) and the 0.1BTO/LTO enjoys the lowest NO_2 production of only 3.4 ppb. The NO_2 concentration of 0.1BTO/LTO-OV was still kept low level to avoid the second pollutant. The oxygen vacancies-modified S-scheme heterojunction of Bi-doped $La_2Ti_2O_7$ and La-doped $Bi_4Ti_3O_{12}$ shows greatly improved photocatalytic performance, especially in the inhibition of NO_2 secondary pollutant, in comparison with the recent reports as shown in Table 2.

The cycling experiment was performed to further explore the catalyst stability. Five successive runs of catalytic NO removal are conducted under identical conditions. The sample 0.1BTO/LTO-OV exhibits good durability, maintaining 95% of the catalytic activity in the first run after five cycles of testing with less NO_2 production than 10 ppb as shown in Fig. 10(c). The little reduction of catalytic performance is attributed to the accumulation of intermediates covering active sites on the surface. BTO/LTO composite exhibits excellent photocatalytic stability in removing NO. Especially, it is more environmentally friendly without generating dangerous secondary NO_2 pollution.

The FT-IR spectra of 0.1BTO/LTO-OV are tested to confirm the

Table 3
BET surface area, pore volume and average pore diameter of the samples.

Samples	Surface area ($m^2 \cdot g^{-1}$)	Pore volume ($cm^3 \cdot g^{-1}$)	Average pore diameter (nm)
$La_2Ti_2O_7$	31.94	0.152	21.51
0.01BLTO	29.05	0.101	19.76
0.05BTO/LTO	45.19	0.148	13.42
0.1BTO/LTO	47.81	0.141	15.13
0.2BTO/LTO	51.06	0.187	16.04
0.3BTO/LTO	60.695	0.134	10.43
0.4LBTO	60.96	0.187	14.38

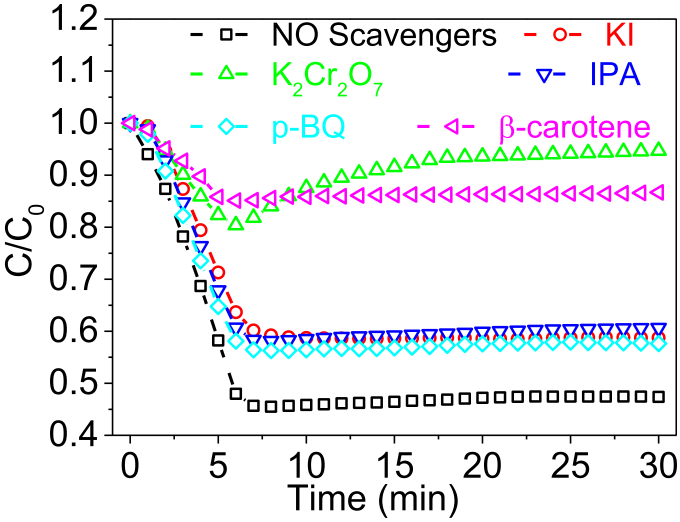

Fig. 13. Photocatalytic performance with different trapping agents over 0.1BTO/LTO-OV.

photocatalytic stability as shown in Fig. 10(d). The 0.1BTO/LTO-OV possesses all bands before and after NO removal, indicating good chemical structure stability. Two curves present the typical structure of perovskite in the range of 500 – 800 cm^{-1}. NO_3^- is evidenced by the little sharp peak at 1384 cm^{-1}, corresponding to NO_3^- on the sample surface after the NO photodegradation reaction [57].

3.4. Photodegradation mechanism

The photocurrent intensity of all samples instantly increases with the light excitation to high points in a short time as shown in Fig. 11. Then, it decreases rapidly to a low current with the light off to form a stable dark current. The Bi-dopped $La_2Ti_2O_7$ shows a much higher photocurrent response than the pure $La_2Ti_2O_7$ sample, implying its more photogenerated carriers. La-doped $Bi_4Ti_3O_{12}$ has a similar photocurrent response with the pure $La_2Ti_2O_7$. The composite photocatalysts also exhibit higher photocurrent response via the formation of heterojunction. Its photocurrent of 0.1BTO/LTO-OV further increases in comparison with the 0.1BTO/LTO. The enhanced photocurrent is related to the oxygen vacancies, which act as electron traps to promote charge separation efficiency [2]. Therefore, the oxygen vacancies effectively suppress the backflow of electrons and holes, to achieve better electron-hole separation efficiency [36].

Fig. 12 shows the N_2 adsorption-desorption isotherm. The catalysts display type IV isotherms with hysteresis loops, indicating the existence of mesopores. 0.01BLTO shares similar specific surface area, pore volume and average pore diameter with $La_2Ti_2O_7$ as shown in Table 3. 0.4LBTO exhibits a higher surface area for its cross structure. Then, the composite photocatalysts enjoy high specific surface areas after constructing the heterojunction. The specific surface areas and porosities of

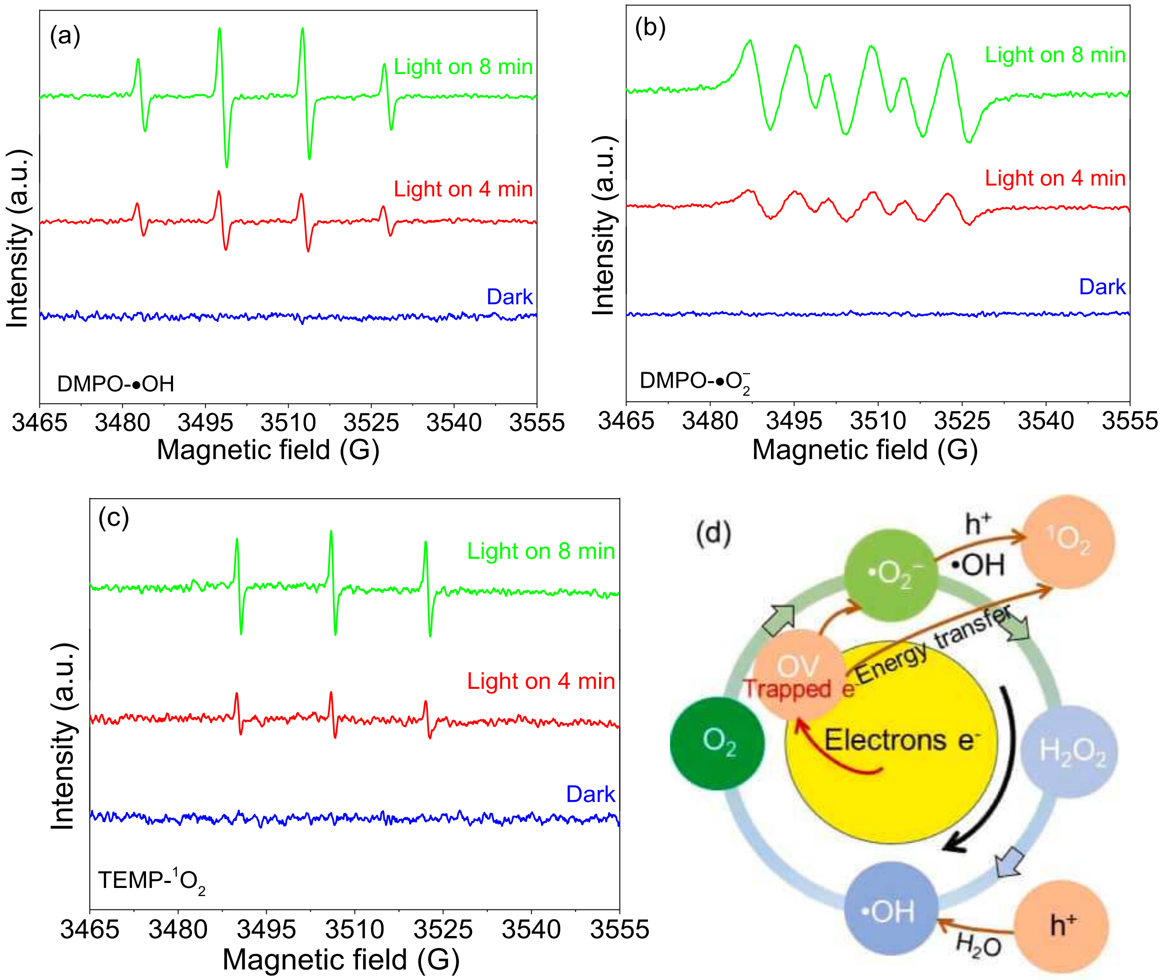

Fig. 14. ESR signals of 0.1BTO/LTO-OV for (a) •OH, (b) $•O_2^-$ and (c) $^1O_2$ in the dark and under light irradiation, and (d) schematic generation of reactive oxygen species.

catalyst particles play important roles in the catalytic activity. Thus, the increments in specific surface area and porosity of the composite catalysts provide more active sites for pollutant degradation.

The trapping experiments were performed with different sacrificial agents to probe the mechanism of NO removal by 0.1BTO/LTO-OV. Potassium iodide (KI), potassium dichromate ($K_2Cr_2O_7$), isopropyl alcohol (IPA), p-benzoquinone (p-BQ) and β-carotene were used to trap the holes (h^+), electrons (e^-), hydroxyl radical (•OH), superoxide radical ($•O_2^-$) and singlet oxygen ($^1O_2$), respectively [2,3]. When the scavengers of e^- and $^1O_2$ are added, the photocatalytic NO removal drastically decreases as shown in Fig. 13, indicating the critical role of electrons and $^1O_2$ in the photocatalytic reactions. When the scavengers of h^+, •OH and $•O_2^-$ are added, the photocatalytic NO removal decreases less in the photocatalytic performance of 0.1BTO/LTO-OV, which means an involvement of h^+, •OH and $•O_2^-$ radicals.

ESR test is the most direct evidence to identify the radicals, such as hydroxyl radical (•OH), superoxide radical ($•O_2^-$) and singlet oxygen ($^1O_2$). The radical signal is almost undetectable in the dark as shown in Fig. 14(a)– 14(c). However, typical quadruplet peaks of DMPO superoxide and hydroxyl adducts, i.e., DMPO-$•O_2^-$ and DMPO-•OH are detected under illumination. In the presence of TEMP, the triplet spectra can be found for TEMP-$^1O_2$, which is consistent with the results of capturing experiment. The radical signals become stronger with the illumination time, indicating more active radicals on the sample surface to promote the photocatalytic reaction.

The generation of reactive oxygen species is schematized in Fig. 14 (d) based on the above results. The abundant electrons on conduction band or trapped by oxygen vacancies react with O_2 to form $•O_2^-$ [Eqs. (7) and (11)], and then part $•O_2^-$ combines with electrons to generate •OH with H_2O_2 as intermediates [Eqs. (8) and (9)]. The •OH can also be formed by hole oxidation of water [Eq.(10)]. Subsequently, part $•O_2^-$ further reacts with h^+ or •OH to form $^1O_2$ [Eq. (12) – (14)]. At the same time, the oxygen vacancies introduce an impurity level below the conduction band, which can accommodate electrons relaxed from the conduction band. Besides, the photosensitivity of catalyst is greatly enhanced and the energy barrier of $^1O_2$ generation reduces [58]. The generation pathways of reactive oxygen species are as follows:

$$e^- + O_2 \rightarrow •O_2^- \quad (7)$$

$$•O_2^- + 2H^+ + e^- \rightarrow 2H_2O_2 \quad (8)$$

$$e^- + 2H_2O_2 \rightarrow •OH + OH^- \quad (9)$$

$$h^+ + H_2O \rightarrow H^+ + •OH \quad (10)$$

$$V\ddot{o} + e^- + O_2 \rightarrow •O_2^- \quad (11)$$

$$O_2 \rightarrow {}^1O_2 \text{ (Energy transfer)} \quad (12)$$

$$•O_2^- + h^+ \rightarrow O_2^1 \quad (13)$$

$$•O_2^- + \cdot OH \rightarrow O_2^1 \quad (14)$$

In situ DRIFTS spectra were measured to provide a further

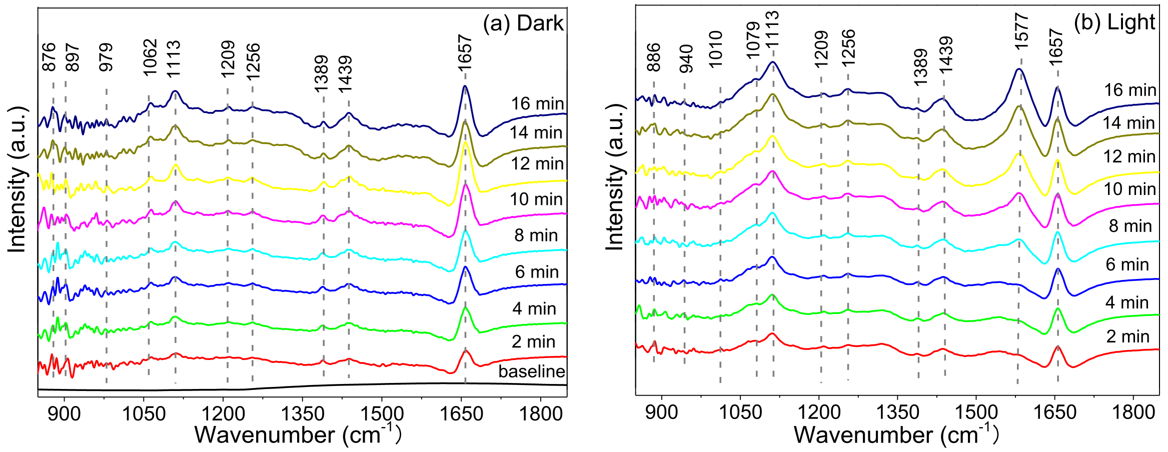

Fig. 15. In situ DRIFT spectra over 0.1BTO/LTO-OV (a) in the dark and (b) under light illumination.

Table 4
Assignments of the infrared bands observed during the photocatalytic NO oxidation processes over 0.1BTO/LTO-OV.

Wavenumbers (cm^{-1})	Assignment	References
886	O-O band stretching vibration	[2]
876, 979, 1062	NO_2	[9,59,61]
897, 940	N_2O_4, $N_2O_4^{2-}$	[9,59]
1113	NO^-	[61]
1079	Chelated nitrites NO_2^-	[73]
1389	Bridge nitrites NO_2^-	[8,59]
1439	Linear nitrites NO_3^-	[9]
1256	Monodentate nitrates NO_3^-	[2,74,75]
1209	Bidentate nitrates NO_3^-	[73]
1010, 1577	Bridging nitrates NO_3^-	[60,76]
1657	NO	[5,60]

mechanistic description of the NO photocatalytic oxidation over 0.1BTO/LTO-OV. Fig. 15(a) exhibits the time-dependent characteristic peaks of NO and intermediates in the adsorption process in the dark. The bands at 1657 cm^{-1} (NO adsorption) were detected upon exposure to NO and O_2 [5,9,59–61]. The generation of NO_2 is associated with direct NO oxidation by O_2 ($2NO + O_2 = 2NO_2$ and $2NO_2 \rightarrow N_2O_4$), which is from a series of infrared peaks assigned to NO_2 (876, 979 and 1062 cm^{-1}) and N_2O_4 (897 cm^{-1}). Furthermore, the appearance of peaks at 1113 cm^{-1} (NO^-), 1389 and 1439 cm^{-1} (NO_2^-) was detected as middle products. Some other weak characteristics at 1209, 1256 and 1439 cm^{-1} are considered as NO_3^- on the surface of 0.1BTO/LTO-OV.

Fig. 15(b) shows the in situ DRIFTS spectra associated with NO photocatalytic removal after light illumination. The peak centered at 886 cm^{-1} is assigned to the O-O band stretching vibration of surface peroxide species, such as $\bullet O_2^-$ and $^1O_2$ [2]. The infrared bands of NO_2 (876, 979, 1062 cm^{-1}) disappear after irradiating several minutes in comparison with the spectra in the dark. At the same time, a considerable number of nitrite species (NO_2^- and $N_2O_4^{2-}$) appear at 1079, 1389 cm^{-1} and 940 cm^{-1}. Particularly, new peaks at 1010 and 1577 cm^{-1} are assigned to the bridging nitrates, which are more stable than the monodentate nitrates [60]. These peaks grow increasingly with the irradiation time, indicating that NO_2 has been further deeply oxidized to nitrate and nitrite species. Table 4 lists the main infrared bands of the adsorbed species as well as their chemical designations.

Based on the trapping experiment and in situ DRIFTS, NO captures

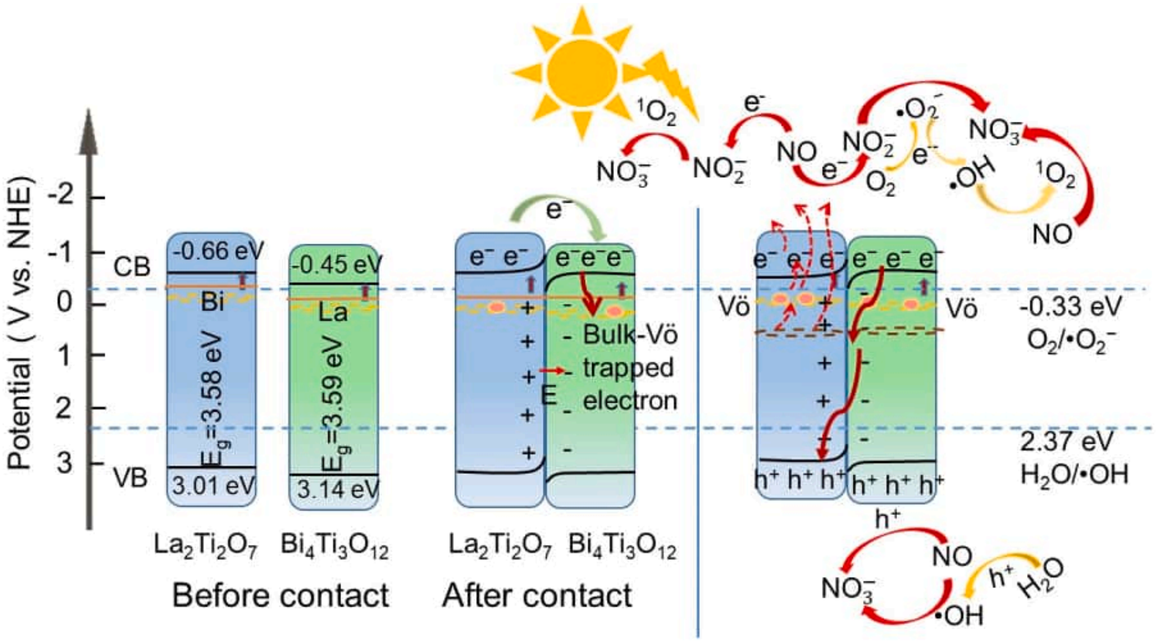

Fig. 16. Schematic illustration of charge transfers and radical generation over 0.1BTO/LTO-OV S-scheme heterojunction for the NO removal by different routes.

an electron and O_2 to trigger the transformation of surface NO_2^- and $N_2O_4^{2-}$, which helps NO further convert into NO_3^- to inhibit the intermediate NO_2 product. On the other hand, the abundant electrons in conduction band and the electrons released by oxygen vacancies can help the catalyst surface produce more $^1O_2$, $\bullet O_2^-$ and $\bullet OH$ to remove the NO gas. Especially, $^1O_2$ owns electrophilicity and tends to oxidate the NO_2^- to NO_3^-. Based on the above analyses, the pathways of photocatalytic NO oxidation are below:

$$e^- + NO_2 \rightarrow NO_2^- \text{ or } 2e^- + 2NO_2 \rightarrow N_2O_4^{2-} \quad (15)$$

$$2NO_2^- + \bullet O_2^- \rightarrow 2NO_3^- \quad (16)$$

$$NO_2^- + \bullet OH \rightarrow NO_3^- \quad (17)$$

$$h^+ + NO + H_2O \rightarrow NO_3^- + 2H^+ \quad (18)$$

$$V\ddot{o} - O_3^{2-} + NO \rightarrow V\ddot{o} - NO_3^- + e^- \quad (19)$$

$$2NO_2^- + {}^1O_2 \rightarrow 2NO_3^- \quad (20)$$

Fig. 16 illustrates the schematic of electron charge transfer and radical generation over the 0.1BTO/LTO-OV S-scheme heterojunction for NO removal by different routes. The Fermi level of Bi-doped $La_2Ti_2O_7$ is higher than that of La-doped $Bi_4Ti_3O_{12}$. After the two phases are in contact, electrons transfer from the $La_2Ti_2O_7$ conduction band to the $Bi_4Ti_3O_{12}$ conduction band until the Fermi levels reach equilibrium to form a built-in electron field from $La_2Ti_2O_7$ to $Bi_4Ti_3O_{12}$.

Under light irradiation, the photogenerated electrons are excited from the valence band of $La_2Ti_2O_7$ and $Bi_4Ti_3O_{12}$ to their conduction bands, leaving holes in their valence bands. The photogenerated electrons in space charge region of $Bi_4Ti_3O_{12}$ transfer to the interface under the built-in electric field to combine with the photogenerated holes from $La_2Ti_2O_7$ driven by the built-in electric field. Then, holes remain in the valence band of $Bi_4Ti_3O_{12}$ to keep a strong oxidation ability while the electrons remain in the conduction band of $La_2Ti_2O_7$ to keep a high reduction ability. This process achieves the spatial separation of photogenerated electrons and holes performing as an S-scheme heterojunction. At the same time, the foreign Bi^{3+}, La^{3+} and oxygen vacancies supply the energy levels to help the separation of holes and electrons.

NO easily reacts with O_2 to NO_2, which captures e^- to be NO_2^- and $N_2O_4^{2-}$ [Reaction (15)]. This is beneficial for the conversion of NO_2 to NO_3^-, thereby inhibiting the NO_2 intermediate. The abundant electrons photogenerated on the conduction band and released from oxygen vacancies can generate more $^1O_2$, $\bullet O_2^-$ and $\bullet OH$ on the catalyst surface to remove NO gas[Reactions (16), (17) and (20)]. Especially, electrophilic $^1O_2$ easily oxidize NO_2^- to NO_3^-. The photogenerated holes on $Bi_4Ti_3O_{12}$ valence band potential in the S-scheme heterojunction have strong oxidability for NO to NO_3^- [Reaction (18)]. The oxygen vacancy on 0.1BTO/LTO-OV surface receives electrons from inside, which has a better adsorption effect on gas and provides more active sites for photocatalytic reaction. At the same time, oxygen vacancies also directly oxidize NO to NO_3^- [Reaction (19)] [62,63].

4. Conclusions

We have successfully fabricated S-scheme heterojunctions of Bi-doped $La_2Ti_2O_7$ and La-doped $Bi_4Ti_3O_{12}$ via a simple hydrothermal method. With the increase in Bi concentration, the content of orthorhombic La-doped $Bi_4Ti_3O_{12}$ increases and the monoclinic Bi-doped $La_2Ti_2O_7$ transfers to the orthorhombic La-doped $Bi_4Ti_3O_{12}$. The 0.1BTO/LTO composite has a higher NO removal ratio (46%) and much lower NO_2 generation (only 3.4 ppb) in comparison with pristine $La_2Ti_2O_7$. The doped electronic states and oxygen defect states narrow the band gap to extend the light absorption range. The S-scheme heterojunction formed between Bi-doped $La_2Ti_2O_7$ and La-doped $Bi_4Ti_3O_{12}$ enhances the separation efficiency of photogenerated charge carriers and improves the transfer rate of electrons across the interface. After heat treatment in N_2 environment, the enriched oxygen vacancies in heterojunction further enhance the light absorption and suppress the carrier recombination. The NO removal ratio of 0.1BTO/LTO-OV reaches 52% with only 5.6 ppb NO_2 generation. The present work provides a synergistic strategy to improve photocatalytic NO removal with doping element, building S-scheme heterojunction and introducing oxygen vacancies.

CRediT authorship contribution statement

Li Lv: Experimental investigation, Formal analysis, Writing – original draft, Visualization. **Lin Lei:** Part experimental measurements. **Qi-Wen Chen:** Part experimental research. **Cheng-Li Yin:** Theoretical research and Visualization. **Huiqing Fan:** Conceptualization, Supervision. **Jian-Ping Zhou:** Project administration, Supervision, Writing – review and editing.

Declaration of Competing Interest

We declare that we have not financial and personal relationships with other people or organizations that can inappropriately influence our work, there is no professional or other personal interest of any nature of kink in any product, service and company that could be construed as influencing the position presented in the manuscript entitled.

Data availability

No data was used for the research described in the article.

Acknowledgments

The SEM and TEM works were finished at the Electron Microscopy Platform of School of Physics and Information Technology, Shaanxi Normal University, Xi'an, China. This work was supported by the National Key Research and Development Project (2020YFC1521900 and 2020YFC1521904) and the National Natural Science Foundation of China (51802140 and 51672168).

Appendix A. Supporting information

Supplementary data associated with this article can be found in the online version at doi:10.1016/j.apcatb.2023.123464.

References

[1] B. Yuan, X. Mao, Z. Wang, R. Hao, Y. Zhao, Radical-induced oxidation removal of multi-air-pollutant: a critical review, J. Hazard. Mater. 383 (2020), 121162.
[2] X. Wen, X. Jiang, T. Jin, H. Chen, X. Zhang, S. Wang, Constructing oxygen vacancies on Bi_2MoO_6 nanosheets by aqueous ammonia etching with enhanced photocatalytic NO oxidation performance, Energ. Fuel. 36 (2022) 11485–11494.
[3] N. Li, M. Shi, Y. Xin, W. Zhang, J. Qin, K. Zhang, H. Lv, M. Yuan, C. Wang, Oxygen vacancies-modified S-scheme $Bi_2Ti_2O_7/CaTiO_3$ heterojunction for highly efficient photocatalytic NO removal under visible light, J. Environ. Chem. Eng. 10 (2022), 107420.
[4] Y. Dong, G. Li, D. Xu, Q. Wang, T. Yang, S. Pang, G. Zhang, L. Lv, Y. Xia, Z. Ren, P. Wang, A novel hierarchical heterostructure of hollow $La_2Ti_2O_7/In_2O_3$ with strong interface interaction for photocatalytic antibiotic degradation, Chem. Eng. J. 446 (2022), 136705.
[5] H. Wu, R. Chen, H. Wang, W. Cui, J. Li, J. Wang, C. Yuan, L. Zhuo, Y. Zhang, F. Dong, An atomic insight into $BiOBr/La_2Ti_2O_7$ p-n heterojunctions: interfacial charge transfer pathway and photocatalysis mechanism, Catal. Sci. Technol. 10 (2020) 826–834.
[6] L. Zhang, J. Zhang, H. Yu, J. Yu, Emerging S-scheme photocatalyst, Adv. Mater. 34 (2022), 2107668.
[7] X. Wang, M. Sayed, O. Ruzimuradov, J. Zhang, Y. Fan, X. Li, X. Bai, J. Low, A review of step-scheme photocatalysts, Appl. Mater. Today 29 (2022), 101609.
[8] F. Chang, X. Wang, C. Yang, S. Li, J. Wang, W. Yang, F. Dong, X. Hu, D.-g. Liu, Y. Kong, Enhanced photocatalytic NO removal with the superior selectivity for NO_2^-/NO_3^- species of $Bi_{12}GeO_{20}$-based composites via a ball-milling treatment: Synergetic effect of surface oxygen vacancies and n-p heterojunctions, Compos, Part B: Eng. 231 (2022), 109600.

[9] T. Cui, Y. Su, X. Fu, Y. Zhu, Y. Zhang, The key role of surface hydroxyls on the activity and selectivity in photocatalytic degradation of organic pollutants and NO, Remov., J. Alloy. Compd. 921 (2022), 165931.

[10] H. Li, H. Shang, Y. Li, X. Cao, Z. Yang, Z. Ai, L. Zhang, Interfacial charging-decharging strategy for efficient and selective aerobic NO oxidation on oxygen vacancy, Environ. Sci. Technol. 53 (2019) 6964–6971.

[11] M. Hojamberdiev, A. Yamaguchi, K. Yubuta, S. Oishi, K. Teshima, Fabrication of $La_2Ti_2O_7$ crystals using an alkali-metal molybdate flux growth method and their nitridability to form $LaTiO_2N$ Crystals under a high-temperature NH_3 atmosphere, Inorg. Chem. 54 (2015) 3237–3244.

[12] V. Tuyikeze, L.H. Omari, M. Bouhbou, A. Boudali, F. Fraija, Study of structural, and optical properties of the layered perovskite $La_2Ti_2O_7$ nanoparticle, Mater. Today.: Proc. 30 (2020) 828–832.

[13] E. Hua, S. Jin, X. Wang, S. Ni, G. Liu, X. Xu, Ultrathin 2D type-II p-n heterojunctions $La_2Ti_2O_7/In_2S_3$ with efficient charge separations and photocatalytic hydrogen evolution under visible light illumination, Appl. Catal. B: Environ. 245 (2019) 733–742.

[14] X. Lin, H. Wang, H. Du, X. Xiong, B. Qu, Z. Guo, D. Chu, Growth of lithium lanthanum titanate nanosheets and their application in lithium-ion batteries, ACS Appl. Mater. Inter. 8 (2016) 1486–1492.

[15] N.I. Ansari, P. Sivagnanapalani, V. Sureshkumar, B.W. Shivaraj, P.K. Panda, Synthesis of lanthanum titanate ($La_2Ti_2O_7$) for high temperature sensor applications, J. Mater. Sci.: Mater. El 32 (2021) 27422–27428.

[16] Y. Cao, P. Tang, Y. Han, W. Qiu, Synthesis of $La_2Ti_2O_7$ flexible self-supporting film and its application in flexible energy storage device, J. Alloy. Compd. 842 (2020), 155581.

[17] S. Leroy, J.-F. Blach, M. Huvé, B. Léger, N. Kania, J.-F. Henninot, A. Ponchel, S. Saitzek, Photocatalytic and sonophotocatalytic degradation of rhodamine B by nano-sized $La_2Ti_2O_7$ oxides synthesized with sol-gel method, J. Photoch. Photobio. A 401 (2020), 112767.

[18] L. Lv, H.-D. Yang, H. Fan, L. Yang, Q.-W. Chen, J.-P. Zhou, $La_2Ti_2O_7$ nanosheets synthesized under magnetic field for ofloxacin ferrophotocatalytic degradation, J. Environ. Chem. Eng. 10 (2022), 108088.

[19] X. Cai, L. Mao, M. Fujitsuka, T. Majima, S. Kasani, N. Wu, J. Zhang, Effects of Bi-dopant and co-catalysts upon hole surface trapping on $La_2Ti_2O_7$ nanosheet photocatalysts in overall solar water splitting, Nano Res 15 (2021) 438–445.

[20] K. Das, D. Majhi, R. Bariki, B.G. Mishra, $SnS_2/Bi_4Ti_3O_{12}$ heterostructure material: A UV–visible light active direct Z-scheme photocatalyst for aqueous phase degradation of diazinon, ChemistrySelect 5 (2020) 1567–1577.

[21] H. Shi, H. Tan, Z. W.-b. Zhu, Y. Sun, E. Ma, Wang, Electrospun Cr-doped $Bi_4Ti_3O_{12}/Bi_2Ti_2O_7$ heterostructure fibers with enhanced visible-light photocatalytic properties, J. Mater. Chem. A 3 (2015) 6586–6591.

[22] Y. Ou, J. Shi, Q. Yan, C. Li, Y. Zheng, Ethanol-assisted molten salt synthesis of $Bi_4Ti_3O_{12}/Bi_2Ti_2O_7$ with enhanced visible light photocatalytic performance, Inorg. Chem. Commun. 133 (2021), 108867.

[23] T. Cheng, Q. Ma, H. Gao, S. Meng, Z. Lu, S. Wang, Z. Yi, X. Wu, G. Liu, X. Wang, H. Yang, Enhanced photocatalytic activity, mechanism and potential application of I_{doped}-$Bi_4Ti_3O_{12}$ photocatalysts, Mater. Today Chem. 23 (2022), 100750.

[24] G. Kresse, J. Furthmüller, Efficient iterative schemes for ab initio total-energy calculations using a plane-wave basis set, Phys. Rev. B 54 (1996) 11169–11186.

[25] MedeA, Materials Design, Inc.: Santa Fe, New Mexico, USA, 2013.

[26] G. Kresse, D. Joubert, From ultrasoft pseudopotentials to the projector augmented-wave method, Phys. Rev. B 59 (1999) 1758–1775.

[27] J.P. Perdew, K. Burke, M. Ernzerhof, Generalized gradient approximation made simple, Phys. Rev. Lett. 77 (1996) 3865–3868.

[28] D.D. Ren, K.T. Gui, S.C. Gu, Y.L. Wei, Mechanism of improving the SCR NO removal activity of Fe_2O_3 catalyst by doping Mn, J. Alloy. Compd. 867 (2021), 158787.

[29] C. Chen, Z. Gao, H. Yan, M.J. Reece, M.P. Paranthama, Crystallographic structure and ferroelectricity of $[A_xLa_{(1-x)}]_2Ti_2O_7$ ($A = $ Sm and Eu) solid solutions with high T_c, J. Am. Ceram. Soc. 99 (2016) 523–530.

[30] J. Xu, Y. Zhang, X. Xu, X. Fang, R. Xi, Y. Liu, R. Zheng, X. Wang, Constructing $La_2B_2O_7$ (B = Ti, Zr, Ce) compounds with three typical crystalline phases for the oxidative coupling of methane: The effect of phase structures, superoxide anions, and alkalinity on the reactivity, ACS Catal. 9 (2019) 4030–4045.

[31] R. Swami, R. Bokolia, K. Sreenivas, Effects of sintering temperature on structural, electrical and ferroelectric properties of $La_2Ti_2O_7$ ceramics, Ceram. Int. 46 (2020) 26790–26799.

[32] S.R. Das, P.S. Dobal, B. Sundarakannan, R.R. Das, R.S. Katiyar, Micro-Raman study of Zr-substituted $Bi_4Ti_3O_{12}$ ceramics, J. Raman. Spectrosc. 38 (2007) 1077–1081.

[33] Z.C. Ling, H.R. Xia, W.L. Liu, H. Han, X.Q. Wang, S.Q. Sun, D.G. Ran, L.L. Yu, Lattice vibration of bismuth titanate nanocrystals prepared by metalorganic decomposition, Mater. Sci. Eng.: B 128 (2006) 156–160.

[34] Y. Cui, P. Dang, F. Wang, P. Yuan, W. Liu, Y. Pu, Effect of oxygen vacancies on photoelectrochemical properties of amphoteric semiconductor $Bi_4Ti_3O_{12}$ photoelectrode, Vacuum 210 (2023), 111899.

[35] X. Hu, J. Wang, J. Wang, Y. Deng, H. Zhang, T. Xu, W. Wang, β particles induced directional inward migration of oxygen vacancies: Surface oxygen vacancies and interface oxygen vacancies synergistically activate PMS, Appl. Catal. B: Environ. 318 (2022), 121879.

[36] J. Qiu, D. Dai, L. Zhang, G. Xia, J. Yao, Oxygen vacancy-rich Bi_2MoO_6 anchored on cuboid metal-organic frameworks for photocatalytic elimination of Cr(VI)/2-nitrophenol mixed pollutants, Sep. Purif. Technol. 301 (2022), 121990.

[37] T. Cheng, H. Gao, S. Wang, Z. Yi, G. Liu, Z. Pu, X. Wang, H. Yang, Surface doping of $Bi_4Ti_3O_{12}$ with S: enhanced photocatalytic activity, mechanism and potential photodegradation application, Mater. Res. Bull. 149 (2022), 111711.

[38] T. Cheng, H. Gao, G. Liu, Z. Pu, S. Wang, Z. Yi, X. Wu, H. Yang, Preparation of core-shell heterojunction photocatalysts by coating CdS nanoparticles onto $Bi_4Ti_3O_{12}$ hierarchical microspheres and their photocatalytic removal of organic pollutants and Cr(VI) ions, Colloids Surf. A: Physicochem. Eng. Asp. 633 (2022), 127918.

[39] S. Niu, R. Zhang, X. Zhang, J. Xiang, C. Guo, Morphology-dependent photocatalytic performance of $Bi_4Ti_3O_{12}$, Ceram. Int. 46 (2020) 6782–6786.

[40] Y. Zhou, Q. Li, J. Zhang, M. Xiang, Y. Zhou, Z. Chen, Y. Chen, T. Yao, Broad spectrum driven Y doped BiO_{2-x} for enhanced degradation of tetracycline: synergy between singlet oxygen and free radicals, Appl. Surf. Sci. 607 (2023), 154957.

[41] M. Arif, M. Zhang, Y. Mao, Q. Bu, A. Ali, Z. Qin, T. Muhmood, X. Shahnoor, B. Liu, S.M. Zhou, Chen, Oxygen vacancy mediated single unit cell Bi_2WO_6 by Ti doping for ameliorated photocatalytic performance, J. Colloid Interf. Sci. 581 (2021) 276–291.

[42] G. Xu, Y. Yang, H. Bai, J. Wang, H. Tian, R. Zhao, X. Wei, X. Yang, G. Han, Hydrothermal synthesis and formation mechanism of the single-crystalline $Bi_4Ti_3O_{12}$ nanosheets with dominant (010) facets, CrystEngComm 18 (2016) 2268–2274.

[43] D. Makovec, N. Križaj, S. Gyergyek, Hydrothermal formation of bismuth-titanate nanoplatelets and nanowires: the role of metastable polymorphs, CrystEngComm 24 (2022) 3972–3981.

[44] Z. Huang, J. Liu, L. Huang, L. Tian, W. Sen, G. Zhang, J. Li, F. Liang, H. Zhang, Q. Jia, S. Zhang, One-step synthesis of dandelion-like lanthanum titanate nanostructures for enhanced photocatalytic performance, NPG Asia Mater. 12 (2020) 11.

[45] X. Cai, M. Zhu, O.A. Elbanna, M. Fujitsuka, S. Kim, L. Mao, J. Zhang, T. Majima, Au nanorod photosensitized $La_2Ti_2O_7$ nanosteps: successive surface heterojunctions boosting visible to near-infrared photocatalytic H_2 evolution, ACS Catal. 8 (2018) 122–131.

[46] S. Wei, Y. Chen, P. Wu, X. Liu, J. Ren, B. Yao, H. Xu, W. Dou, Y. Wang, R. Wu, Z. Fang, Q. Liang, Surface defects engineered $Bi_4Ti_3O_{12}$ nanosheets for photocatalytic degradation of antibiotic levofloxacin, Appl. Catal. A: Gen. 640 (2022), 118675.

[47] G.A.S. Alves, H.A. Centurion, J.R. Sambrano, M.M. Ferrer, R.V. Gonçalves, Band gap narrowing of Bi-doped $NaTaO_3$ for photocatalytic hydrogen evolution under simulated sunlight: A pseudocubic phase induced by doping, ACS Appl. Energ. Mater. 4 (2020) 671–679.

[48] F. Zhang, Y. Xu, H. Yang, S. Guan, W. Shi, Y. Chen, C. Huang, J. Xing, H. Liu, Q. Chen, Effects of cerium on structures and electrical properties of (Nb, Ta) modified $Bi_4Ti_3O_{12}$, piezoelectric Ceramics, J. Am. Ceram. Soc. 105 (2022) 4161–4170.

[49] Z. Ma, Y. Li, Y. Lv, R. Sa, Q. Li, K. Wu, Synergistic effect of doping and compositing on photocatalytic efficiency: a case study of $La_2Ti_2O_7$, ACS Appl. Mater. Inter. 10 (2018) 39327–39335.

[50] Z. Liu, Z. Ma, Promoting the photocatalytic activity of $Bi_4Ti_3O_{12}$ microspheres by incorporating iron, RSC Adv. 10 (2020) 19232–19239.

[51] X. Feng, B. Lv, L. Lu, X. Feng, H. Wang, B. Xu, Y. Yang, F. Zhang, Role of surface oxygen vacancies in zinc oxide/graphitic carbon nitride composite for adjusting energy band structure to promote visible-light-driven photocatalytic activity, Appl. Surf. Sci. 562 (2021), 150106.

[52] R. López, R. Gómez, Band-gap energy estimation from diffuse reflectance measurements on sol–gel and commercial TiO_2: a comparative study, J. Sol. -gel. Sci. Techn. 61 (2011) 1–7.

[53] K. Gelderman, L. Lee, S.W. Donne, Flat-band potential of a semiconductor: using the Mott-Schottky, Equ., J. Chem. Educ. 84 (2007) 685.

[54] Y. Li, R. Li, Y. Zhai, Y. Huang, S. Lee, J. Cao, Improved photocatalytic activity of $BaTiO_3/La_2Ti_2O_7$ heterojunction composites via piezoelectric-enhanced charge transfer, Appl. Surf. Sci. 570 (2021), 151146.

[55] Y. Duan, J. Luo, S. Zhou, X. Mao, M.W. Shah, F. Wang, Z. Chen, C. Wang, TiO_2-supported Ag nanoclusters with enhanced visible light activity for the photocatalytic removal of NO, Appl. Catal. B: Environ. 234 (2018) 206–212.

[56] Y. Lu, Y. Huang, Y. Zhang, J.-j. Cao, H. Li, C. Bian, S. C. Lee, Oxygen vacancy engineering of $Bi_2O_3/Bi_2O_2CO_3$ heterojunctions: Implications of the interfacial charge transfer, NO adsorption and removal, Appl. Catal. B: Environ. 231 (2018) 357–367.

[57] P. Zhao, N. Feng, F. Fang, H. Wan, G. Guan, Surface acid etching for efficient anchoring of potassium on 3DOM $La_{0.8}Sr_{0.2}MnO_3$ catalyst: an integration strategy for boosting soot and NO_x simultaneous elimination, J. Hazard. Mater. 409 (2021), 124916.

[58] Y. Deng, Z. Zhou, H. Zeng, R. Tang, L. Li, J. Wang, C. Feng, D. Gong, L. Tang, Y. Huang, Phosphorus and kalium co-doped g-C_3N_4 with multiple-locus synergies to degrade atrazine: Insights into the depth analysis of the generation and role of singlet oxygen, Appl. Catal. B: Environ. 320 (2023), 121942.

[59] W. Zhang, J. Zhao, A.A. Allam, Y. Xin, J. Lin, T. Gao, J.S. Ajarem, X. Li, C. Wang, D. W. Bahnemann, Palladium nanoparticles embedded nutshell-like Bi_2WO_6 as an efficient and stable visible-light-responsive photocatalysts for NO removal, Energ. Fuel. 36 (2022) 13852–13862.

[60] Y. Xin, Q. Zhu, T. Gao, X. Li, W. Zhang, H. Wang, D. Ji, Y. Huang, M. Padervand, F. Yu, C. Wang, Photocatalytic NO removal over defective Bi/BiOBr nanoflowers: The inhibition of toxic NO_2 intermediate via high humidity, Appl. Catal. B: Environ. 324 (2023), 122238.

[61] K. Li, W. Zhou, X. Li, Q. Li, S.A.C. Carabineiro, S. Zhang, J. Fan, K. Lv, Synergistic effect of cyano defects and $CaCO_3$ in graphitic carbon nitride nanosheets for efficient visible-light-driven photocatalytic NO removal, J. Hazard. Mater. 442 (2023), 130040.

[62] Y. Sun, H. Wang, Q. Xing, W. Cui, J. Li, S. Wu, L. Sun, The pivotal effects of oxygen vacancy on Bi_2MoO_6: promoted visible light photocatalytic activity and reaction mechanism, Chinese, J. Catal. 40 (2019) 647–655.
[63] X. Xie, Q.-U. Hassan, H. Lu, F. Rao, J. Gao, G. Zhu, In situ construction of oxygen-vacancy-rich Bi0@Bi_2WO_{6-x} microspheres with enhanced visible light photocatalytic for NO removal, Chinese, Chem. Lett. 32 (2021) 2038–2042.
[64] Y. Huang, Y. Gao, Q. Zhang, J.-j Cao, R.-j Huang, W. Ho, S.C. Lee, , Hierarchical porous $ZnWO_4$ microspheres synthesized by ultrasonic spray pyrolysis: Characterization, mechanistic and photocatalytic NO removal studies, Appl. Catal. A: Gen. 515 (2016) 170–178.
[65] F. Rao, G. Zhu, W. Zhang, Y. Xu, B. Cao, X. Shi, J. Gao, Y. Huang, Y. Huang, M. Hojamberdiev, Maximizing the formation of reactive oxygen species for deep oxidation of NO via manipulating the oxygen-vacancy defect position on $(BiO)_2CO_3$, ACS Catal. 11 (2021) 7735–7749.
[66] K. Qi, J. Jing, G. Dong, P. Li, Y. Huang, The excellent photocatalytic NO removal performance relates to the synergistic effect between the prepositive NaOH solution and the g-C_3N_4 photocatalysis, Environ. Res. 212 (2022), 113405.
[67] Z. Wang, Y. Huang, M. Chen, X. Shi, Y. Zhang, J. Cao, W. Ho, S.C. Lee, Roles of N-vacancies over porous g-C_3N_4 microtubes during photocatalytic NO_x removal, ACS Appl. Mater. Inter. 11 (2019) 10651–10662.
[68] J. Geng, L. Zhao, M. Wang, G. Dong, W. Ho, The photocatalytic NO-removal activity of g-C_3N_4 significantly enhanced by the synergistic effect of Pd^0 nanoparticles and N vacancies, Environ. Sci.: Nano 9 (2022) 742–750.
[69] X. Hu, Y. Wang, Z. Ling, H. Song, Y. Cai, Z. Li, D. Zu, C. Li, Ternary g-C_3N_4/TiO_2/Ti_3C_2 MXene S-scheme heterojunction photocatalysts for NO_x removal under visible light, Appl. Surf. Sci. 556 (2021), 149817.
[70] F. Chang, C. Yang, J. Wang, B. Lei, S. Li, H. Kim, Enhanced photocatalytic conversion of NO_x with satisfactory selectivity of ^{3}D–^{2}D $Bi_4O_5Br_2$-GO hierarchical structures via a facile microwave-assisted preparation, Sep. Purif. Technol. 266 (2021), 118237.
[71] Y. Ou, G. Zhu, F. Rao, J. Gao, J. Chang, X. Xie, W. Zhang, Y. Huang, M. Hojamberdiev, Coral-shaped $TiO_{2-\delta}$ decorated with carbon quantum dots and carbon nanotubes for NO removal, ACS Appl. Nano Mater. 4 (2021) 7330–7342.
[72] X. Shi, P. Wang, W. Li, Y. Bai, H. Xie, Y. Zhou, L. Ye, Change in photocatalytic NO removal mechanisms of ultrathin BiOBr/BiOI via NO_3^- adsorption, Appl. Catal. B: Environ. 243 (2019) 322–329.
[73] J. Li, M. Ran, P. Chen, W. Cui, J. Li, Y. Sun, G. Jiang, Y. Zhou, F. Dong, Controlling the secondary pollutant on B-doped g-C_3N_4 during photocatalytic NO removal: a combined DRIFTS and DFT investigation, Catal. Sci. Technol. 9 (2019) 4531–4537.
[74] N. Tang, Y. Liu, H. Wang, Z. Wu, Mechanism study of NO catalytic oxidation over MnO_x/TiO_2, Catal., J. Phys. Chem. C. 115 (2011) 8214–8220.
[75] G. Jiang, X. Li, M. Lan, T. Shen, X. Lv, F. Dong, S. Zhang, Monodisperse bismuth nanoparticles decorated graphitic carbon nitride: Enhanced visible-light-response photocatalytic NO removal and reaction pathway, Appl. Catal. B: Environ. 205 (2017) 532–540.
[76] G. Li, B. Wang, Z. Wang, Z. Li, Q. Sun, W.Q. Xu, Y. Li, Reaction mechanism of low-temperature selective catalytic reduction of NO_x over Fe-Mn oxides supported on fly-ash-derived SBA-15 molecular sieves: structure-activity relationships and in situ DRIFT analysis, J. Phys. Chem. C. 122 (2018) 20210–20231.

Ceramics International 49 (2023) 13321–13329

Contents lists available at ScienceDirect

Ceramics International

journal homepage: www.elsevier.com/locate/ceramint

Engineering oxygen vacancy to accelerate proton conduction in Y-doped $BaZrO_3$

Kang Zhu [a], Nai Shi [b], Lijie Zhang [a], Daoming Huan [a], Xinyu Li [a], Xiaoyu Zhang [a], Rui Song [a], Changrong Xia [a], Ranran Peng [a,c,d,*], Yalin Lu [a,c,d,**]

[a] CAS Key Laboratory of Materials for Energy Conversion, Department of Materials Science and Engineering, University of Science and Technology of China, Hefei, 230026, China

[b] Kyushu University Platform of Inter-/Transdisciplinary Energy Research, Kyushu University, 744 Motooka, Fukuoka, 819-0395, Japan

[c] Anhui Laboratory of Advanced Photon Science and Technology, University of Science and Technology of China, Hefei, 230026, China

[d] Hefei National Laboratory of Physical Science at the Micro-scale, University of Science and Technology of China, Hefei, 230026, China

A R T I C L E I N F O

Handling Editor: Dr P. Vincenzini

Keywords:
Proton conducting solid oxide cells
Proton conducting perovskite oxides
Proton conductivity
Oxygen vacancy engineering
Density functional theory
Ab initio molecular dynamics

A B S T R A C T

Proton conducting oxides have drawn great interest as electrolytes for proton-conducting reversible solid oxide cells (P-RSOCs), but suffered from the inferior ionic conductivity. To accelerate proton conduction, oxygen vacancy engineering via calcium-doping is proposed and validated, which generates more oxygen vacancies to increase proton concentration and importantly tailors the position of oxygen vacancy to accelerate proton diffusion. TG and EIS results show that calcium-doped $BaZr_{0.8}Y_{0.2}O_{3-\delta}$ (BZY2), $BaZr_{0.8}Ca_{0.1}Y_{0.2}O_{3-\delta}$ (BZCa1Y2), owns higher proton concentration, and demonstrates ionic conductivity of 0.008 S cm^{-2} at 700 °C, 2.7 times higher than BZY2. The lower activation energy of conductivity in BZCa1Y2 confirms the faster proton conduction behaviour. DFT calculation concludes that oxygen vacancies prefer to cluster with Ca site and proton diffusion barrier is decreased most when vacancy is tailored to generate nearing Ca. When considering the concentration of proton and oxygen vacancy, proton diffusion coefficient obtained from Ab-initio Molecular Dynamics simulation is 1.1×10^{-5} cm^2s^{-1} for BZCa1Y2 in 200 °C, larger than BZY2 (9.0×10^{-6} cm^2s^{-1}) and verifying the accelerated proton diffusion due to the tailored oxygen vacancy. The oxygen vacancy engineering provides a further understanding of proton diffusion in proton conducting oxides and a new promising opportunity to improve conductivity.

1. Introduction

Proton conducting oxides have drawn increasing attention because of their fascinating nature of allowing the traverse of protons [1–5], and have been widely investigated for their potential as electrolyte materials for proton-conducting reversible solid oxide cells (P-RSOCs), an efficient device that can convert chemical energy to electrical energy freely and reversibly [6–10]. As the essential materials for P-RSOCs, proton conducting electrolytes require high proton conductivity and outstanding chemical stability in vapor/CO2-containing atmospheres. Among various proton conducting oxides, $BaZrO_3$ (BZO) based electrolytes, such as Y doped BZO (BZY) [11] and Sc doped BZO (BZSc) [12], have aroused broad concerns recently for their excellent CO_2 resistance and high grain conductivity. Nevertheless, further development of BZO-based electrolytes is obstructed by their poor sinterability [13], the large proton trapping effect rooting in the defect association between positive proton and negative B site dopants (e.g. Y'_{Zr}) [14]and especially the low dehydration temperature (~375 °C, about 100 °C lower than that of doped $BaCeO_3$ [15]), which greatly hindered the proton migration at the operating temperatures of P-RSOCs (400–700 °C). Lots of works, including the addition of sintering aids [16], doping with elements with lower melting point [4,5,17,18], advanced preparation processes [19] and sintering technologies [20] have been devoted to improving the conductivity of BZO based electrolytes, yet the

* Corresponding author. CAS Key Laboratory of Materials for Energy Conversion, Department of Materials Science and Engineering, University of Science and Technology of China, Hefei, 230026, China.

** Corresponding author. CAS Key Laboratory of Materials for Energy Conversion, Department of Materials Science and Engineering, University of Science and Technology of China, Hefei, 230026, China.

E-mail addresses: pengrr@ustc.edu.cn (R. Peng), yllu@ustc.edu.cn (Y. Lu).

https://doi.org/10.1016/j.ceramint.2022.12.206
Received 13 November 2022; Received in revised form 5 December 2022; Accepted 21 December 2022
Available online 28 December 2022
0272-8842/© 2023 Elsevier Ltd and Techna Group S.r.l. All rights reserved.

improvement of proton concentration at elevated temperatures rooting in the enhanced dehydration temperatures [15] and the increase of proton diffusion coming from tailoring oxygen vacancy have been barely noticed.

$$V_O^{\bullet\bullet} + H_2O + O_O^{\times} = 2OH_O^{\bullet} \quad (1)$$

The improvement of proton concentration at elevated temperatures and the accelerated proton diffusion could be realized by adopting suitable dopants. Han et al.'s investigation suggested that Ce dopant could improve the proton concentration at elevated temperatures [15]. For example, $BaCe_{0.2}Zr_{0.6}Y_{0.2}O_{3-\delta}$ possesses proton defects about 0.114 and 0.065 per unit at 600 and 700 °C, respectively, higher than those of BZY (~0.082 at 600 °C, and 0.05 at 700 °C) in O_2 -5%H_2O atmosphere. Unfortunately, the proton conductivities of $BaCe_{0.2}Zr_{0.6}Y_{0.2}O_{3-\delta}$ have not improved accordingly, about 0.019 and 0.018 S cm^{-1} at 600 and 700 °C, respectively, whereas those of BZY are 0.027 and 0.023 S cm^{-1}. These results imply that depressing the trapping of protons is very important to improve proton conduction. Recently, heavily scandium-doped BZO ($BaZr_{0.4}Sc_{0.6}O_{3-\delta}$) demonstrates attractive conductivity for its high proton concentration and fast proton conduction [21], and reveals that high-level doping is a possible way to provide both high proton concentration and low proton migration energies [22]. However, this phenomenon is irreproducible to the typical Y-doped BZO of which high Y doping level results in lower protonic conductivity because of the clustering of dopants [23] as proved by calculation [24]. That is to say, the development of BZO-based electrolytes caught in the conflict between high proton concentration and severe proton trapping. And thus, how to cripple or even extinguish proton trapping along with improving proton concentration seems to be an efficient way to improve proton conductivity, based on the discovery and investigation of the nature of proton trapping [14,25,26].

Proton defects in oxides are universally formed via hydration reaction (Eq. (1)) in humid oxidizing atmosphere at the expense of oxygen vacancies and is an entropy-decreasing reaction essentially, which makes the reaction shift backward at elevated temperatures. This means that at the operating temperatures of P-RSOCs, typically 400–700 °C, oxygen vacancies coexist with proton defects, which makes the oxygen vacancies, generally formed via B site dopants with low valence, a promising factor both to modulate the proton diffusion properties [27, 28] and to adjust the proton concentrations. In this work, we adopted the low-cost dopant, Ca, as dopant for BZY to intensively engineering proton concentration and proton diffusion. It was found that $BaZr_{0.8}Ca_{0.1}Y_{0.2}O_{3-\delta}$ (BZCa1Y2) demonstrated much larger ionic conductivity and higher hydration temperatures than BZY. DFT calculations provide a new insight to evaluate the role of the oxygen vacancy on proton diffusions that inactive oxygen vacancies preferring to cluster with Ca sites could significantly depress the energy barrier for proton diffusion and thus accelerate proton diffusion.

2. Results and discussion

2.1. Structure & defect evolution

A series of oxides, $BaZr_{0.8-x}Y_{0.2}Ca_xO_{3-\delta}$ (x = 0–0.1), were prepared as potential promising proton conductors, and their nominal compositions and abbreviations are summarized in Table 1. ICP-AES measurements ensure that the actual compositions of all the samples are very close to their nominal ones. Fig. 1a shows room temperature XRD patterns of these oxides. All oxides have similar XRD patterns with that of BZY2, and no additional peaks corresponding to any secondary phase or impurity were observed. Notably, the magnified XRD patterns (Fig. 1b) show clearly that the peak locating at 29–32° shifts toward lower angle with increased Ca^{2+} content, suggesting enlarged lattice parameters at high Ca^{2+} content. Such lattice enlargement should be ascribed to the larger ionic radii of Ca^{2+} (1.00 Å for six-coordination) than Zr^{4+} (0.72 Å for six-coordination) and Y^{3+} (0.90 Å for six-coordination), which also confirms that Ca^{2+} has been doped into BZY2 successfully at B site.

Table 1
Nominal and actual compositions of the as-synthesized samples determined by ICP-OES. Abbreviations of these samples are also given.

Abbreviation	Nominal composition	Actually composition
BZY2	$BaZr_{0.8}Y_{0.2}O_{3-\delta}$	$Ba_{0.99}Zr_{0.81}Y_{0.19}O_{3-\delta}$
BZCa05Y2	$BaZr_{0.75}Ca_{0.05}Y_{0.2}O_{3-\delta}$	$Ba_{1.03}Zr_{0.76}Ca_{0.05}Y_{0.18}O_{3-\delta}$
BZCa1Y2	$BaZr_{0.7}Ca_{0.1}Y_{0.2}O_{3-\delta}$	$Ba_{1.01}Zr_{0.69}Ca_{0.11}Y_{0.21}O_{3-\delta}$

Rietveld refinement results (Table S1) confirm that all of the compositions show P m $\overline{3}$ m space group, in spite of the larger radius of Ca^{2+} and more oxygen vacancies introduced by Ca^{2+} dopant (Eq. (2)), both of which might bring lattice distortion. The low values of R_{wp} and χ^2 ensure the reliability of refinements. BZCa1Y2 is intensively analysed and discussed since this work is going to investigate the role of oxygen vacancy, which in principle reaches the maximum in BZCa1Y2 within our $BaZr_{0.8-x}Y_{0.2}Ca_xO_{3-\delta}$ series (Eq. (2)). The actual crystal structure of BZCa1Y2 is obtained on high-angle-annular-dark-field scanning transmission electron microscopy (HAADF-STEM), as illustrated in Fig. 1c. The observed crystal structure and corresponding FFT image demonstrate that BZCa1Y2 remains cubic perovskite structure, and the lattice parameter calculated from HAADF is 4.23 Å, consistent well with the XRD refinement result. Energy dispersive X-ray spectroscopy (EDX) mappings show that all of the elements distribute normally in BZCa1Y2 (Fig. S2), confirming the nonexistence of secondary phase.

$$CaO \xrightarrow{ZrO_2 \text{ in BZY2}} Ca_{Zr}'' + O_O^{\times} + V_O^{\bullet\bullet} \quad (2)$$

The incorporation of Ca^{2+} at B site brings different O 1s spectra shapes between BZCa1Y2 and BZY2. O 1s peak can be aligned into two parts: the peak around 528.3–528.8 eV corresponding to lattice oxygen; and the other peak, centered at 531.5 eV, assigned to the less electron-rich oxygen species generally related to the oxygen vacancies [29–33]. As shown in Fig. 1f, the peak fitted for oxygen vacancy has obviously higher area (relative to that of lattice oxygen peak) in BZCa1Y2 than that in BZY2, indicating that Ca^{2+} doping increases the concentration of oxygen vacancy in the way of charge compensation. This result further demonstrates that Ca^{2+} has actually doped into B-site since there will be no change of oxygen vacancy if Ca^{2+} was doped into A-site.

To primarily evaluate the hydration property of BZCa1Y2, XRD patterns of BZCa1Y2 and BZY2 powders before and after the full-hydration process are shown in Fig. 1d and e. After the full-hydration process, lattice parameters of BZCa1Y2 and BZY2 increase from 4.23 Å and 4.19 Å to 4.25 Å and 4.20 Å, respectively. The expansion of lattices can be attributed to hydration reaction, as illustrated in Eq. (1) [34]. The hydration degree is then evaluated by calculating the lattice expansion ratio. For BZY2, the degree of lattice expansion is about 0.24% after hydration; while for BZCa1Y2, it is 0.47%. The larger hydration expansion of BZCa1Y2 primarily advises that it has a better hydration degree comparing with BZY2. Further discussion of hydration properties will be given in next section.

As one of the basic requirements for electrolytes, stabilities of BZCa1Y2 in various atmospheres are evaluated. As shown in Fig. S3, BZCa1Y2 remains its crystal structure and negligible second phase/impurity after being treated at 500 °C for 50 h in various atmospheres, including pure H_2, 10% H_2O-air, pure and dry CO_2, and even the hash 10%H_2O–CO_2 atmosphere. The chemical stability of BZCa1Y2 in Lewis acids (e.g. H_2O and CO_2) containing atmospheres is comparable with BZY2, indicating that the basicity of Ca^{2+} dopant doesn't impact the Lewis basicity of oxide obviously.

2.2. Proton concentration & conductivity

In proton conductors, proton defects can be formed via Eq. (1) at the expense of oxygen vacancies in humid atmosphere, and then migrate

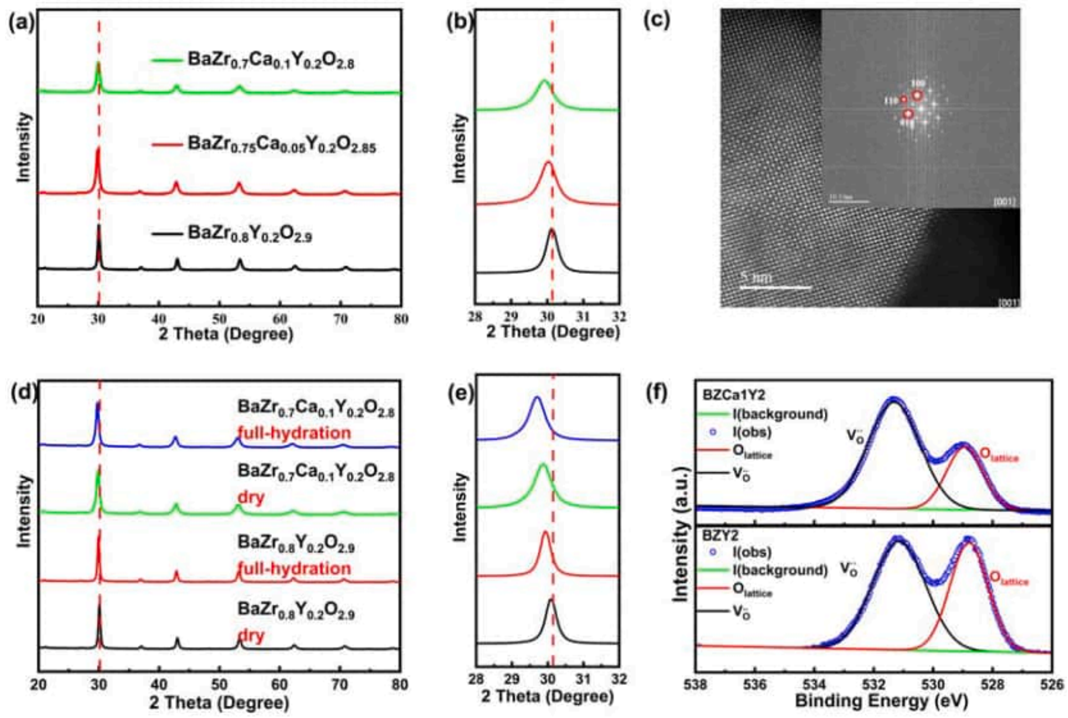

Fig. 1. (a) XRD patterns and (b) the magnified patterns at 29-32° of $BaZr_{0.8-x}Y_{0.2}Ca_xO_{3-\delta}$ powders with different Ca doping levels. (c) HAADF-STEM images along with the selected area electron diffraction (SAED) pattern of BZCa1Y2. (d) XRD patterns for dry and full hydrated powders and (e) the corresponding magnified patterns. (f) XPS results of BZY and BZCa1Y2.

among lattice oxygens via hopping and rotation [25]. To reach a desirable proton conductivity (σ), both proton concentration and proton diffusion coefficient contribute in the form of the Nernst-Einstein equation (Eq. (3)),

$$\sigma = F^2 C_H D_H / RT \tag{3}$$

where F refers to Faraday's constant, R is the gas constant, and T is the temperature, C_H and D_H refer to proton concentration and diffusion coefficient, respectively. Proton concentration, as one of the most important factors governing proton conductivity, can be measured using the thermogravimetry method (TG) by fully relaxing at a fixed temperature in a humid atmosphere (Fig. S4). Here, vapor-containing Ar

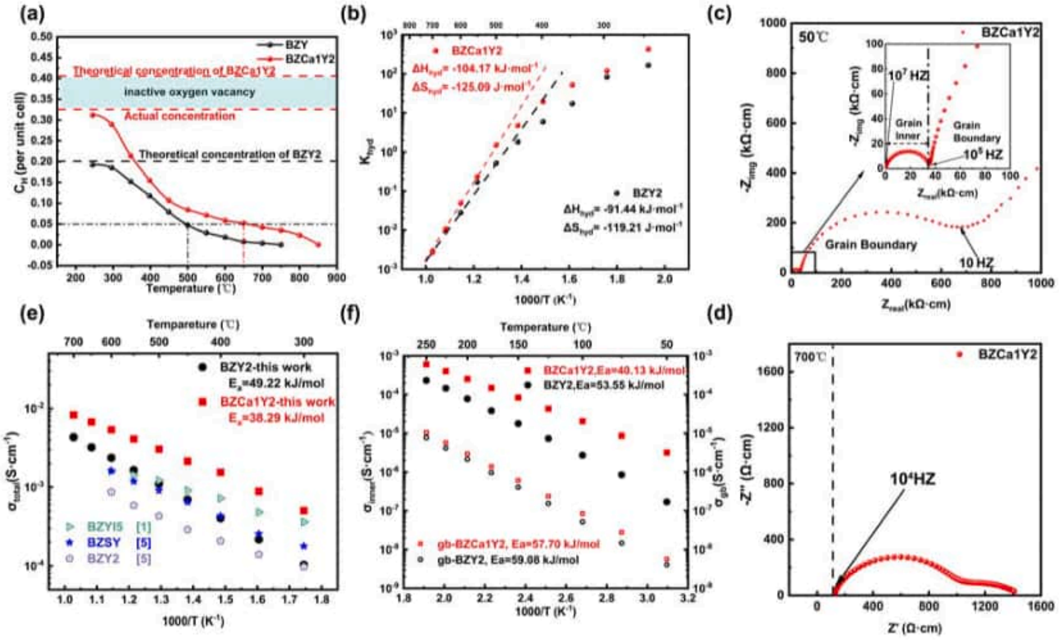

Fig. 2. (a) Proton concentrations of BZCa1Y2 and BZY2 at various temperatures in wet Ar ($P_{H2O} = 0.02$ atm). (b) Hydration thermodynamics in BZY2 and BZCa1Y2. Typical EIS curves of BZCa1Y2 and BZY2 at (c) 50 and (d) 700 °C. (e) Total proton conductivity of various samples measured from 700 to 300 °C in wet N_2. $BaZr_{0.8}Y_{0.15}In_{0.05}O_{3-\delta}$ (BZYI5) [1]; $BaZr_{0.8}Sn_{0.1}Y_{0.2}O_{3-\delta}$ (BZSY) [5]: and $BaZr_{0.8}Y_{0.2}O_{3-\delta}$ (BZY2) [5]: $BaZr_{0.8}Y_{0.2}O_{3-\delta}$. (f) Grain inner and grain boundary conductivities of BZCa1Y2 and BZY2 measured at 50–250 °C in wet N_2.

atmosphere (2% H_2O) is adopted for TG measurements. Then proton concentrations at various temperatures can be calculated as demonstrated in Fig. 2a. When oxygen vacancies are totally hydrated, the proton concentrations should be 0.4 and 0.2 for BZCa1Y2 and BZY2, respectively. As the hydration process is an entropy reduction process, high temperature is adverse to proton formation and even can reverse the hydration process. As shown in Fig. 2a, the observed temperatures to reach 5% proton per unit are approximately 650 °C for BZCa1Y2, around 150 °C higher than that for BZY2, indicating a much-enlarged operating temperature range of BZCa1Y2 as proton conductor. Hydration enthalpy and hydration entropy can be calculated as illustrated in Eq. S1 ~ Eq. S6, and calculation results are shown in Fig. 2b. BZCa1Y2 owns more negative hydration enthalpy ($\Delta H = -99.37$ kJ mol^{-1}) and more positive entropy ($\Delta S = -114.83$ J mol^{-1} K^{-1}) than BZY2 ($\Delta H = -91.44$ kJ mol^{-1}, and $\Delta S = -119.21$ J mol^{-1} K^{-1}, close to previous reports [12]).

Fig. 2a also indicates that BZCa1Y2 has much higher proton concentration than BZY2 within the investigated temperatures (250–850 °C). Measured at 250 °C, proton concentration is 0.312 per unit cell for BZCa1Y2, about 1.6 times higher than that in BZY (~0.191 per unit cell). At the typical operating temperatures (500–700 °C), the proton concentration of BZCa1Y2 is over 2-fold higher than that in BZY2, implying a better potential for proton conduction in BZCa1Y2. It is noteworthy that when measuring at 250 °C, proton concentration in BZY2 is very close to its theory limitation (0.2 per unit cell). This phenomenon agrees well with previous studies [34,35], and proves that almost all oxygen vacancies are expensed to form proton defects at low temperatures. However, in BZCa1Y2 system, only 78% (=0.312/0.4) of oxygen vacancies can be filled even at 250 °C, and there will be 22% of vacancies still unoccupied, named inactive oxygen vacancies. Hydration ratio is calculated as $[OH_O^{\bullet}]/\ 2[V_O^{\bullet\bullet}]$ and demonstrated in Fig. S5. Measured at operating temperatures, the hydration ratio of them are lower than 20% for both oxides, indicating that a large number of oxygen vacancy exist, which might greatly impact the transition property of protons.

Conductivities of BZCa1Y2 are then investigated at different temperatures (50–700 °C) under humid N_2 (~2% H_2O). Typical EIS curves at high (700 °C) and low temperatures (50 °C) are shown in Fig. 2c and d, respectively. When measured at low temperatures (below 250 °C), three arcs with distinct capacitances (~10^{-11} F, ~10^{-9} F and ~10^{-5} F) are observed (Fig. 2c), which correspond to the resistances of grain inner, grain boundary and electrode reaction, respectively [21,36,37]. Then the conductivities of grain inner, and grain boundary are calculated by figuring out their respective ohmic resistances, which are determined from the left intercept of the first arc with the real axis, and the length of the first left arc in the spectra, respectively, as illustrated in Fig. 2c. And the total conductivity, the sum of grain inner conductivity and grain boundary conductivity, can be also obtained. While measured at higher temperature (e.g. at 700 °C in Fig. 2d), the arc corresponding to grain boundary conduction disappear, and the observed two impedance arcs should be both ascribed to the electrode reactions because of their low capacities (~10^{-5} F). In such cases, grain inner conductivity and grain boundary conductivity cannot be distinguished.

The obtained conductivities are shown in Fig. 2e (≥300 °C) and 2f (≤250 °C), respectively, as a function of testing temperatures. Within the investigated temperature range, BZCa1Y2 owns higher ionic conductivities than BZY2. Measured at 700 °C, the total conductivity of BZCa1Y2 is 0.008 S cm^{-1}, about 2.7 times higher than that of BZY2 (0.003 S cm^{-1}). Measured at 300–700 °C, the activation energy for total conductivity, Ea, is about 38.29 kJ mol^{-1} for BZCa1Y2, much lower than that of BZY2 (49.22 kJ mol^{-1}), implying lower energy barrier for ionic conduction. It should be noted that measured at high temperature, both electrolytes contain a large ratio of oxygen vacancies (Fig. 2a) which introduce a staggering proportion of oxygen ion conduction, and especially, such proportion increases with the testing temperatures. And thus, the obtained apparent Ea cannot be deemed as the activation energy for only proton conduction, but a factor integrating both the concentrations and conducting energy barriers of protons and oxygen vacancies.

Measured below 250 °C, grain inner conductivity (σ_{inner}) can be distinguished from grain boundary conductivity (σ_{gb}). For both electrolytes, their σ_{inner} is about one or two orders of magnitude larger than σ_{gb}, indicating that the total ionic conduction is drastically hindered by grain boundaries at low temperatures [16]. Measured at 250 °C, the values of σ_{inner} and σ_{gb} are 6.03×10^{-4} and 1.05×10^{-5} S cm^{-1} for BZCa1Y2 and 2.32×10^{-4} and 7.74×10^{-6} S cm^{-1} for BZY2, respectively. These results indicate that BZCa1Y2 demonstrates a 2.6 times higher grain inner conductivity than that of BZY2, and a 1.35 times higher grain boundary conductivity when measured at 250 °C. The activation energies for grain conduction are simulated as 40.13 kJ mol^{-1} for BZCa1Y2 and 53.55 kJ mol^{-1} for BZY, both of which are higher than those obtained at higher temperatures. As indicated in TG analysis, the ratio of $[OH_O^{\bullet}]/\ 2[V_O^{\bullet\bullet}]$ is very close to 1 below 250 °C, suggesting that the obtained conductivity is mainly from proton conduction. Notably, as discussed above, the proton concentrations at 250 °C are 0.312 per unit cell in BZCa1Y2, about 1.6 times larger than that of BZY2; yet the grain inner conduction of BZCa1Y2 is 2.6 times larger than that of BZY2. Considering the low concentration and high conduction energy barrier for oxygen vacancy, this improved ionic conduction of BZCa1Y2 should be ascribed to its better proton diffusion property rooting in the lower diffusion energy barrier.

It is noteworthy that the activation energy of σ_{gb} is 57.70 kJ mol^{-1} for BZCa1Y2, very close to that in BZY2 (59.08 kJ mol^{-1}), suggesting that the conduction behaviours of grain boundaries are similar in these materials, which agrees well with precious investigations [13]. And when the measurement temperature is higher than 300 °C, the arc of grain inner and grain boundary cannot be distinguished, and the observed arcs at higher temperatures should be assigned as electrode reactions [14].

2.3. Oxygen vacancy engineering

To intensively evaluate the proton conduction properties, diffusion energy barriers in BZCa1Y2 and BZY2 are calculated via CI-NEB method [38]. In the supercell containing only one proton defect, there are two and three different proton diffusion paths in BZCa1Y2 and BZY2 (Fig. 3a and b), respectively. Here, the path I refers to the proton diffusion between ZrO_6 octahedrons, path II refers to that between ZrO_6 and YO_6, and path III refers to that between ZrO_6 and CaO_6 only in BZCa1Y2. Fig. 3d and e shows the calculated diffusion barrier for each path in BZCa1Y2 and BZY2. When the proton is located at the O site in Ca–O–Zr chain, the diffusion barrier from CaO_6 octahedron to ZrO_6 octahedron is quite large, about 0.524 eV; yet that from ZrO_6 to CaO_6 is only 0.411 eV. The 0.113 eV difference in forward and backward energy barriers of path III can be attributed to the interaction between Ca_{Zr}'' defect (negative charge) and proton defect (positive charge), leading to proton trapping [14]. As for the barriers when proton migrates from YO_6 to ZrO_6 in BZCa1Y2, they are 0.432 eV for forward and 0.355 eV for backward diffusion with the energy difference of 0.077 eV. Corresponding energy barriers for proton migration between ZrO_6 octahedrons are 0.391–0.368 eV, depending on the impact from neighbouring octahedrons. The differences for forward and backward migration between ZrO_6 octahedrons are close to zero, indicating no trapping effect nearing Zr–O–Zr. While for BZY2, migration barriers from YO_6 to ZrO_6 and from ZrO_6 to ZrO_6 are 0.463 eV and 0.412 eV, respectively, both slightly higher than those in BZCa1Y2. This can be interpreted by more space for proton migration coming from larger lattice parameters in BZCa1Y2. Moreover, the trapping energy of Y_{Zr}' in BZY2 (0.072 eV) is very close to that in BZCa1Y2, suggesting that the presence of Ca_{Zr}'' has negligible effect on the proton diffusion around Y_{Zr}' in BZCa1Y2. Notably, it can be obviously found that migration from CaO_6 is more hindered, and will obstruct the total diffusion behaviour in BZCa1Y2.

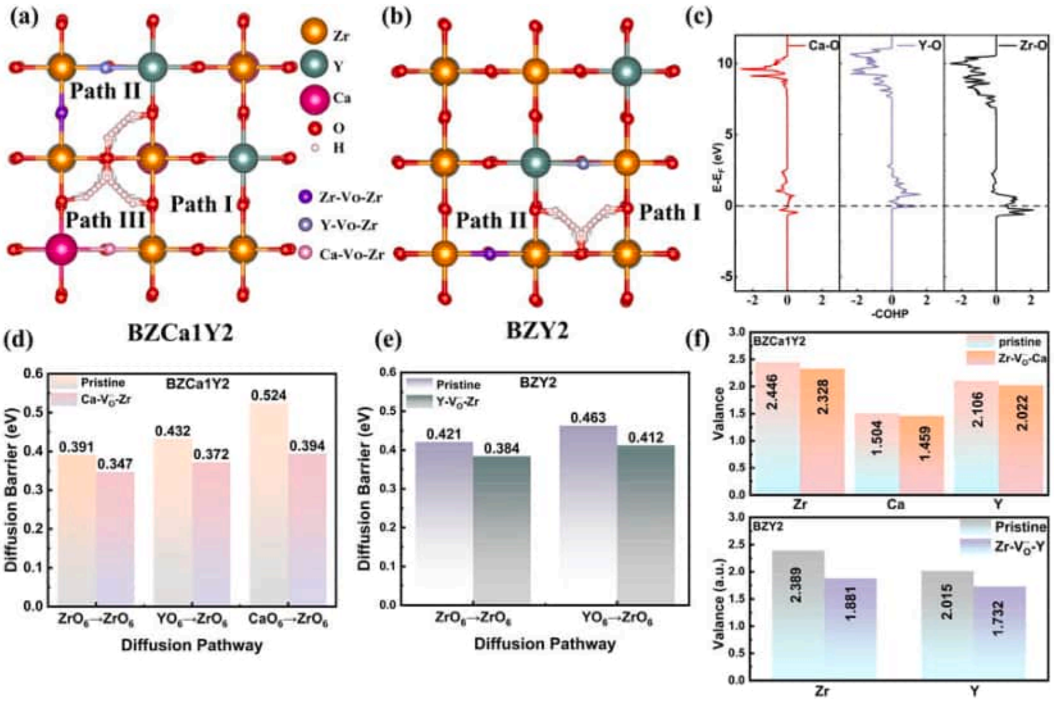

Fig. 3. Schemes of proton diffusion path in BZCa1Y2(a) and BZY2(b). (c) COHP curves of Ca–O, Y–O and Zr–O in BZCa1Y2. Proton diffusion barriers for (d) BZCa1Y2 and (d) BZY2 with and without an oxygen vacancy formed at the most energetically desirable site. (f) Valances of B-sites elements before and after the formation of oxygen vacancy at the most desired sites in BZCa1Y2 and BZY2, respectively.

However, under the practical operation temperature of proton electrolytes, oxygen vacancies largely exist. And thus, oxygen vacancies should be taken into consideration in both BZCa1Y2 and BZY2. Then diffusion energy barriers were intensively calculated with one oxygen vacancy contained in the 3 × 3 × 3 supercell, which coexists with proton defect as inactive host for proton. The formation energies for oxygen vacancies at different oxygen sites are calculated. As shown in Figs. S6a and S6b, there are 4 different oxygen sites in both BZY2 and BZCa1Y2, and oxygen vacancy prefers to form at Ca–O–Zr site (−0.820 eV) in BZCa1Y2 and Y–O–Zr site (−0.632 eV) in BZY2 because of their lowest formation energy in oxide lattice.

Furthermore, it can also be observed that the formation energies differ slightly when neighbouring different B site ions in BZY2 and suggests the propensity of the oxygen vacancy locations is quite energetically small in BZY2, implying that oxygen vacancies in BZY2 system prefer distributing normally to clustering with dopant or host elements. In the case of BZCa1Y2, the difference of formation energy is much larger than that in BZY2, indicating that oxygen vacancies in BZCa1Y2 are more preferable to form neighbouring Ca sites. Crystal orbital Hamilton populations (COHP) [39,40]are shown in Fig. 3c. The negative and positive values of -COHP denote the antibonding and bonding interaction contributions, respectively. There is a distinct sharp peak for antibonding states between Ca and O below fermi energy, which is invisible in Y–O and Zr–O bonds. This result indicates that the Ca–O bond is more disruptive comparing with Y–O and Zr–O bonds, of which only bonding states exist below fermi energy. Besides, analysis of electron localization functional (ELF), an effective criterion for the strength of chemical bonds [41], shows that the degree of electron localization in Zr–O–Ca is obviously lower than those in Zr–O–Zr and Zr–O–Y (Fig. S7). Both COHP and ELF results indicate a weaker bond between Zr–O–Ca than Zr–O–Zr and Zr–O–Y, which makes oxygen vacancies prefer to generate at Zr–O–Ca sites, consistent with the analysis results on oxygen vacancy formation energies.

When $V_O^{\cdot\cdot}$ is generated at the O site of Ca–O–Zr chain, it exerts very positive effects on proton diffusion, as shown in Fig. 3d. It can be clearly seen that energy barriers reduce dominantly from 0.524 to 0.394 eV for the proton diffusing from CaO6 to ZrO6, and from 0.432 to 0.372 eV and 0.391 to 0.347 eV for the proton migrations from YO6 to ZrO6 and from ZrO6 to ZrO6, respectively, of which the reduction ratios are 24.8%, 13.9% and 11.3%, respectively. Importantly, the existence of oxygen vacancy largely weakens the trapping effect induced by Ca_{Zr}'', which is very beneficial to proton diffusion. Whereas in BZY2, when oxygen vacancy is located at Zr–O–Y, the proton diffusion barriers decrease from 0.463 eV to 0.412 eV–0.421 eV and 0.384 eV for the proton diffusing from YO6 to ZrO6 and ZrO6 to ZrO6, respectively, as indicated in Fig. 3e. The presence of oxygen vacancy decreases diffusion energy barriers of BZY2 as it does in BZCa1Y2, but the reduction rate is obviously smaller, suggesting that oxygen vacancy in BZCa1Y2 plays a much more positive effect on proton diffusion. Especially, the existence of oxygen vacancy makes the proton diffusion energy barriers in BZCa1Y2 (0.394 eV) lower than those in BZY2 (0.421 eV), which should account for the lower diffusion energy barrier experimentally obtained in BZCa1Y2 than in BZY2 within the investigated temperature range, demonstrating a tremendous positive impact on proton conduction.

To explore the reason for the more accelerated proton diffusion in BZCa1Y2, Bader charge analysis is conducted on both materials. When $V_0^{\bullet\bullet}$ is generated, the effective valence of cations, especially the B-site cations, will be changed. It is generally accepted that proton trapping is mainly aroused by the negative charge center of low valence dopants at B-site, and reflected in the diffusion barriers [14]. And thus the effect of oxygen vacancy on proton diffusion can be evaluated by comparing valence changes of B site elements before and after oxygen vacancy formation based on Bader charge analysis [42], as shown in Fig. 3f. In BZY2 system, when a vacancy is generated at Zr-$V_0^{\bullet\bullet}$-Y, valances of Zr and Y decrease from 2.389 to 2.015 to 2.505 and 1.753, respectively, both of which decrease about 15%, close to Kim et al.'s reports [28]. This suggests that the proton trapping aroused by the formation of $V_0^{\bullet\bullet}$ is severe in BZY2 and the proton diffusion will be hindered by the trapping. Yet, In BZCa1Y2 system, the formation of oxygen vacancy at Zr-$V_0^{\bullet\bullet}$-Ca site makes valence states of Ca, Y and Zr all decrease less than 5%, so the trapping effect from the valence decrease of B-site is tuned and thus accelerate the proton diffusion.

2.4. Ab initio molecular dynamics simulation

To simulate the proton diffusion in practical conditions, ab initio molecular dynamics (AIMD) calculations are performed at 473 K, at which there will be 6 protons in BZY2 (Fig. S10b) and 10 protons along with one oxygen vacancy in BZCa1Y2 (Fig. S10a) in the 3 × 3 × 3 supercells according to the TG results.

For AIMD calculations, diffusion behaviour can be reflected by mean square displacement (MSD):

$$< [\vec{r}(t)]^2 >= \frac{\sum_{i=1}^{n} < [\vec{r}_i\,(t)]^2 - [\vec{r}_i\,(t_0)]^2 >}{N} \quad (4)$$

where $r_i(t)$ and $r_i(t_0)$ are the instantaneous and initial positions of atom i, respectively, and N is the number of H atoms. And thus, diffusion coefficient (D) can be calculated as:

$$D = \lim_{t\to\infty}\left[\frac{< [\vec{r}(t)]^2 >}{2dt}\right] \quad (5)$$

where d is the dimension of diffusion, which is 3 in our calculation; and t is the time. Corresponding AIMD results are shown in Fig. 4. The AIMD energy and temperature of BZY2 and BZCa1Y2 maintain stable (Figs. S10c and d), indicating the corresponding system reaches thermodynamic equilibrium during the sampling. The MSDs of the two systems show good linear relationships with simulation time (Fig. 4a and b), and diffusion coefficients of proton (D_H) in BZY2 and BZCa1Y2 can be calculated using Eq. (5). D_H of BZCa1Y2 is calculated as 1.1×10^{-5} cm^2s^{-1}, larger than that of BZY2 (9.0×10^{-6} cm^2s^{-1}) at 473 K. Besides, the dynamical properties of proton transport can be intensively characterized using the power-law formulation of the MSD over time (Eq. (6)) [43], as shown in the inset picture in Fig. 3d and e. The exponent α, which is used to distinguish the diffusion behaviour, can then be Figured out. According to the values of α, the proton diffusions in BZY2 and BZCa1Y2 can be divided into three motion behaviours, named superdiffusion ($\alpha > 1$) at short time range (<10 fs), trapped diffusion ($\alpha < 1$) at intermediate time range (10–30 fs), and normal diffusion ($\alpha \approx 1$) and at the long time (>30 fs) [44]. It is noteworthy that the values of α in supperdiffusion and normal diffusion in BZCa1Y2 are larger than those in BZY2, indicating the faster diffusion process in BZCa1Y2; and the smaller α value in trapped diffusion suggests that the proton trapping in BZCa1Y2 is depressed. These results are highly consistent with previous DFT calculations, indicating that the trapping effect in BZCa1Y2 is greatly suppressed.

$$MSD(t) \simeq K_\alpha t^\alpha \quad (6)$$

In order to better understand the proton diffusions in BZCa1Y2 and BZY2, radial distribution function g(r) of BZY2 and BZCa1Y2 are analysed and shown in Fig. 4c and d. In BZY2, the intensity of g(r) of Y–H is higher than that of Zr–H obviously. This result indicates that protons prefer to diffuse surrounding Y atoms, implying that protons will be trapped nearing Y atoms, and that the diffusion process will be hindered. by this trapping effect. On the contrary, In BZCa1Y2 system, the intensity of g(r) of Y–H and Ca–Y is not significantly higher than that of Zr–H when taking oxygen vacancy into consideration, verifying that the presence of oxygen vacancy could minimize the trapping effect and promote proton diffusion.

In general, AIMD results show that BZCa1Y2 demonstrates a higher proton diffusion coefficient, which can be attributed to the slighter trapping effect aroused by the presence of oxygen vacancies.

3. Conclusions

In this work, we proposed a promoted proton diffusion behaviour caused by the presence of oxygen vacancy in Ca doped BZY2. Evidence from experiments suggested that Ca2+ with large radius could be doped

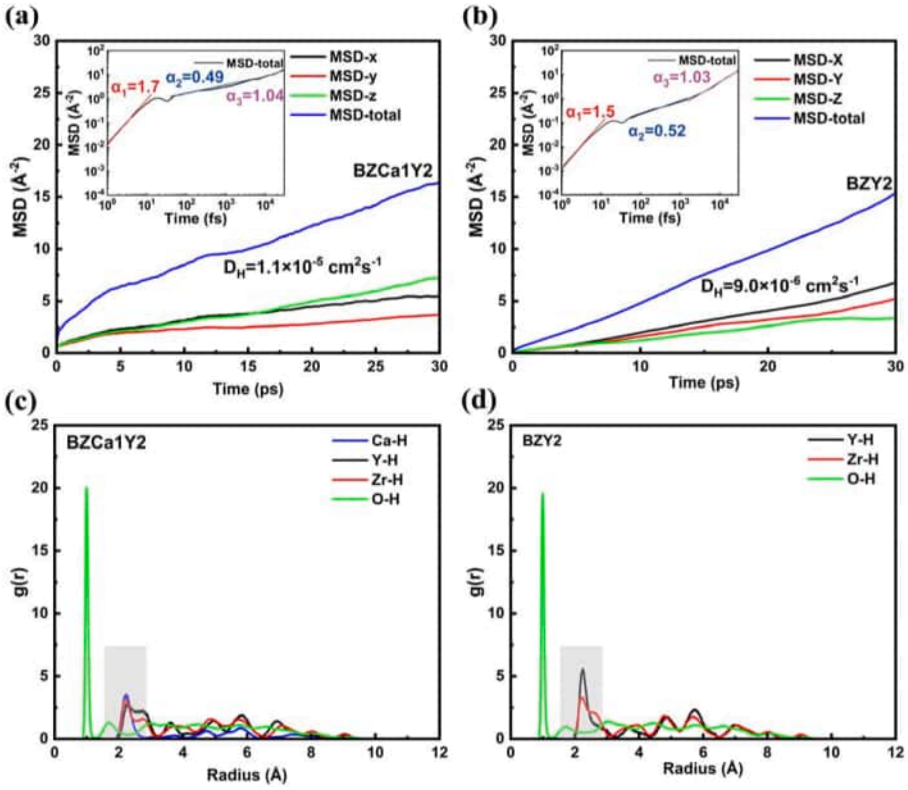

Fig. 4. Time-depended MSD of (a) BZCa1Y2and (b) BZY2, and MSD in logarithmic coordinates (inset). Radial distribution function (RDF) g(r) of BZCa1Y2 (c) and BZY2 (d).

into B site in BZY2 successfully, and the obtained oxide BZCa1Y2 demonstrated outstanding chemical stability and fascinating hydration behaviour. TG measurement indicated Ca doping provided more active oxygen vacancy sites for hydration, which largely increases proton concentration, although a portion of inactive ones remained unhydrated. Conductivity measurements indicated that BZCa1Y2 possessed higher conductivities along with lower activation energies within the investigated temperatures. DFT calculations illustrated that oxygen vacancies prefer to form neighbouring Ca due to the weak bond strength of Ca–O bonds. Especially, the presence of inactive oxygen vacancies could greatly accelerate proton diffusion by largely decreasing their diffusion barriers. Then, AIMD simulation and the radial distribution function further verified that the diffusion coefficient of BZCa1Y2 is higher than BZY2's which root in the much-depressed trapping effect at the presence of oxygen vacancy. Our findings provide a new method to improve proton conduction via oxygen vacancy engineering, which demonstrates enormous potentiality to explore new materials with better protonic conductivity and higher pronation temperatures.

4. Experimental

4.1. Materials preparation

All of the powders were prepared by solid-state reaction (SSR). Stoichiometric amounts of Y_2O_3 (99%), CaO (99%), ZrO_2 (99%) and $BaCO_3$ (99%) were mixed with alcohol. After a 20-h ball milling, powders were dried for 10 h, pressed into pellets under the stress of 56 MPa and sintered at 1200 °C for 20 h. Then the pellets were crushed, ball milled, pressed and sintered twice to get the desired powders. All chemicals were purchased from Sinopharm Chemical Reagent Co. Ltd.

The pellets used for conductivity measurements were fabricated by pressing powders at 300 MPa. The thickness and diameter of green pellets were around 0.5 and 13 mm, respectively, which were then buried in sacrificial powders, a mixture of the as-synthesized powder and 1 wt% $BaCO_3$, and sintered at 1600 °C for 20 h to get the desired pellets. The as-sintered samples showed relative density higher than 97% determined by the Archimedes method.

4.2. Characterization

In order to verify the actual compositions of sintered pellets, samples were polished and crushed into powders, and the compositions were determined by Inductively Coupled Plasma Optic Emission Spectrometer (ICP-OES) with Opmita 7300 DV. Microstructures of as-sintered pellets were investigated by JEOL scanning electron microscope (SEM, JXA-8100). Morphologies of powders were characterized on a transmission electron microscope (TEM, Talos F200X). X-ray photoelectron spectroscopy (XPS, Thermo ESCALAB 250) was applied to investigate the valence states of elements of powders. XPSPEAK software was used to analyze XPS data.

Crystal structures of samples were measured at room temperature (RT) by X-ray diffractometer (Rigaku Smartlab) with Cu K_α radiation ($\lambda = 1.5418$ Å) over the 2θ range of 20–80° with a scanning speed of 3° min^{-1} and the step of 0.02°. In order to qualitatively investigate the degree of hydration, samples were treated in the air with 10% H_2O from 700 °C to room temperature with a very slow cooling speed (about 0.5 °C/min) and kept at 200 °C for 10 h to ensure that powders were full-hydrated. Then RT-XRD characterizations were applied to distinguish the lattice expansion after hydration. The temperature-dependent equilibrated proton concentrations in BZY2 and BZCa1Y2 were evaluated by thermogravimetric analysis (TGA, STA449F3 Jupiter, NETZSCH). The proton concentrations were measured in the temperature range between 200 and 700 °C. The samples were first treated at 900 °C for 2 h in dry Ar to dehydrate, the gas was subsequently switched to a wet Ar ($P_{H2O} = 0.02$). The proton concentration was calculated assuming the weight gains from the dehydrated samples were in accordance with the hydration reaction, as noted in Supplementary File. The measured weight change was corrected by a blank test to eliminate the buoyancy effect.

4.3. Conductivity measurements

Conductivity measurements of a pellet-like BZY2 and BZCa1Y2 samples with sputtered platinum (Pt) electrodes were performed in wet atmospheres of N_2(2% H_2O). Before the preparation of Pt electrodes, pellets were grinded and polished into mirrorlike ones. The N_2 gas was passed through deionized water kept at 17.5 °C to be saturated with water vapor with partial pressures (pH_2O) of 0.02 atm, respectively. Also, the gas lines were wiped with ribbon heaters whose temperature was set to be 50 °C higher than that of the deionized water to avoid the condensation of water vapor. Pt electrodes were used as a current collector and Ag wires were used as the lead. Impedance spectra were collected on a frequency response analyzer (Solartron 1260, Solartron Analytical, Farnborough, UK) in the frequency range of 1 Hz–10 MHz under an applied voltage of 50–200 mV. In addition, Arrhenius plots were obtained by collecting the temperature-balanced impedance spectra during cooling from 700 down to 50 °C after the sample was annealed at 700 °C for about 18 h. ZView software (Scribner Associates, Inc., USA) was used to analyze the impedance spectra.

4.4. Calculation methods

All of the calculations were performed by the Density Functional Theory (DFT) with the projector augmented wave (PAW) [45] method implemented in Vienna ab initio simulation package (VASP) [46,47]. The generalized gradient approximation (GGA) [47] with the Perdew Burke Ernzerhof revised for solid functional (PBEsol) [48] was used to treat the exchanging correlation effects. We applied the PAW potentials with valence configurations of Ba (5s5p6s), Zr (4s4p5s4d), Ca (3s3p4s), Y (3s3p4s), O (2s2p) and H(1s). The plane-wave basis set was set to kinetic cutoff energy of 520 eV. Integration over the Brillouin zone used the Gaussian smearing with the width of the smearing of 0.05 eV. All calculations were carried out in 3 × 3 × 3 supercell utilizing a single gamma point and 2 × 2 × 2 Γ-centered k-mesh for structure optimization and static calculation, respectively. The convergence criteria for SCF calculations and ionic relaxations were set to 10^{-5} eV and 0.02 eV/Å, respectively. Dopant structures were modelled with the compositions as $BaZr_{18/27}Y_{6/27}O_3$ and $BaZr_{15/27}Ca_{3/27}Y_{6/27}O_3$, which are close to the experimental compositions protonated structures were generated by home-made codes based on Pymatgen [49]. Structure search results indicated that Y was located at body diagonal in BZY, and Ca and Y were located at diagonal position, the energy convergence criterion of optimization and statistic calculations were 10^{-5} and 10^{-6} eV, respectively. All structures were relaxed until the force on each atom lower than 0.02 eV $Å^{-1}$. Data analysis was performed with the help of vaspkit [50], pymatgen and qvasp [51]. The convergence of cut-off energy and K-mesh is tested when the change of total energy is smaller than 2 meV/atom.

Defect formation energies were calculated according to following method:

$$E_F(D^q) = E_{sup}(D^q) - E_{sup}(perf) - \sum_i \Delta n_i \mu_i + q(E_{Fermi} + E_{VBT})$$

where $E_{sup}(D^q)$ and $E_{sup}(perf)$ were total energies of the supercell including the defect D^q of charge state q, and of the perfect (defect-free) supercell, respectively; Δn_i was the difference in the number of component atom i between the defective and perfect supercells; μ_i was the chemical potential of atom i; E_{Fermi} and E_{VBT} were the energies of the valence band top (VBT) and the Fermi energy in the band gap measured from the VBT, respectively. In the case of supercells containing charged defects, errors caused by electrostatic interactions from periodic

boundary images were corrected using Kumagai and Oba's extension to the method performed by pycdt code [52]. The bulk proton migration barrier was calculated using climbing-image nudged elastic band (CI-NEB) method in a 3 × 3 × 3 supercell, of which five images were inserted in each migration pathway. Convergence was confirmed when the force was lower than 0.03 eV $Å^{-1}$.

In order to investigate the proton diffusion properties, Ab initio Molecular Dynamics (AIMD) calculation was performed in NVT assemble. The standard Nose–Hoover thermostat [53,54] implemented in VASP was used with the default Nose mass set by VASP, and the time step was set to 0.5 fs. Optimized structures were heated from 0 to 473 K in 3ps, remain at 473 K for 5 Ps for the pre-balance, and then the data of AIMD was collected during 30 ps to get an accurate description of proton diffusion. Crystal orbital Hamilton populations (COHP) was performed and analysed based on LOBSTER [55] and wxdragon software.

Author contributions

Conceptualization, K. Zhu and R. Peng.; formal analysis, K. Zhu, R. Peng; funding acquisition, C. Xia, R. Peng and Y. Lu; investigation, Zhu, N. Shi, L. Zhang, D. Huan, X. Li, X. Zhang and R. Song; methodology, K. Zhu, N. Shi and L. Zhang; supervision, R. Peng and Y. Lu; visualization, K. Zhu and R. Peng; writing-original draft K. Zhu; writing-review editing, K. Zhu and R. Peng; all authors have read and agreed to the published version of the manuscript.

Notes

The authors have no competing interests to declare that are relevant to the content of this article.

Declaration of competing interest

The authors declare that they have no known competing financial interests or personal relationships that could have appeared to influence the work reported in this paper.

Acknowledgment

This work was financially supported by the National Natural Science Foundation of China (52272247, 52102323), the National Key Research and Development Program of China (2017YFA0402800), the Fundamental Research Funds for the Central Universities (WK340000004), and the Key Program of Research and Development of Hefei Science Center CAS (2018HSC-KPRD002). We acknowledge the Supercomputing Center of University of Science and Technology of China (USTC) for providing computational resources.

Appendix A. Supplementary data

Supplementary data to this article can be found online at https://doi.org/10.1016/j.ceramint.2022.12.206.

References

[1] W. Sun, Z. Shi, M. Liu, L. Bi, W. Liu, An easily sintered, chemically stable, barium zirconate-based proton conductor for high-performance proton-conducting solid oxide fuel cells, Adv. Funct. Mater. 24 (2014) 5695–5702.
[2] W. Sun, M. Liu, W. Liu, Chemically stable yttrium and tin Co-doped barium zirconate electrolyte for next generation high performance proton-conducting solid oxide fuel cells, Adv. Energy Mater. 3 (2013) 1041–1050.
[3] H. IwaharaT, EsakaH.UchidaN. Maeda, Proton conduction in sintered oxides and its application to steam electrolysis for hydrogen production, Solid State Ionics 3–4 (1981) 359–363.
[4] Y.D.A.S. Nowick, High-temperature protonic conductors with perovskite-related structures, Solid State Ionics 77 (1995) 137–146.
[5] R. Murphy, Y. Zhou, L. Zhang, L. Soule, W. Zhang, Y. Chen, M. Liu, A new family of proton-conducting electrolytes for reversible solid oxide cells: $BaHf_xCe_{0.8-x}Y_{0.1}Yb_{0.1}O_{3-\delta}$, Adv. Funct. Mater. 30 (2020).
[6] E. Fabbri, L. Bi, H. Tanaka, D. Pergolesi, E. Traversa, Chemically stable Pr and Y Co-doped barium zirconate electrolytes with high proton conductivity for intermediate-temperature solid oxide fuel cells, Adv. Funct. Mater. 21 (2011) 158–166.
[7] C. Duan, J. Tong, M. Shang, S. Nikodemski, M. Sanders, S. Ricote, A. Almansoori, R. O'Hayre, Readily processed protonic ceramic fuel cells with high performance at low temperatures, Science 349 (2015) 1321–1326.
[8] C. Duan, R. Kee, H. Zhu, N. Sullivan, L. Zhu, L. Bian, D. Jennings, R. O'Hayre, Highly efficient reversible protonic ceramic electrochemical cells for power generation and fuel production, Nat. Energy 4 (2019) 230–240.
[9] X. Li, Z. Chen, Y. Yang, D. Huan, H. Su, K. Zhu, N. Shi, Z. Qi, X. Zheng, H. Pan, Z. Zhan, C. Xia, R. Peng, S. Wei, Y. Lu, Highly stable and efficient Pt single-atom catalyst for reversible proton-conducting solid oxide cells, Appl. Catal., B 316 (2022).
[10] Y. Yang, N. Shi, Y. Xie, X. Li, X. Hu, K. Zhu, D. Huan, R. Peng, C. Xia, Y. Lu, K doping as a rational method to enhance the sluggish air-electrode reaction kinetics for proton-conducting solid oxide cells, Electrochim. Acta 389 (2021).
[11] D. Huan, N. Shi, L. Zhang, W. Tan, Y. Xie, W. Wang, C. Xia, R. Peng, Y. Lu, New, efficient, and reliable air electrode material for proton-conducting reversible solid oxide cells, ACS Appl. Mater. Interfaces 10 (2018) 1761–1770.
[12] T.S. Hans, G. Bohn, Electrical conductivity of the high-temperature proton conductor $BaZr_{0.9}Y_{0.1}O_{2.95}$, J. Am. Ceram. Soc. 83 (2000) 768–772.
[13] S.A.K.D. Kreuer, W. Münch, A.FuchsaU. Klock, J. Maiera, Proton conducting alkaline earth zirconates and titanates for high drain electrochemical applications, Solid State Ionics 145 (2001) 295–306.
[14] Z. Sun, E. Fabbri, L. Bi, E. Traversa, Lowering grain boundary resistance of $BaZr_{0.8}Y_{0.2}O_{3-\delta}$ with $LiNO_3$ sintering-aid improves proton conductivity for fuel cell operation, Phys. Chem. Chem. Phys. 13 (2011) 7692–7700.
[15] Y. Yamazaki, F. Blanc, Y. Okuyama, L. Buannic, J.C. Lucio-Vega, C.P. Grey, S. M. Haile, Proton trapping in yttrium-doped barium zirconate, Nat. Mater. 12 (2013) 647–651.
[16] D. Han, X. Liu, T.S. Bjørheim, T. Uda, Yttrium-doped barium zirconate-cerate solid solution as proton conducting electrolyte: why higher cerium concentration leads to better performance for fuel cells and electrolysis cells, Adv. Energy Mater. 11 (2021), 2003149.
[17] P. Babilo, S.M. Haile, Enhanced sintering of yttrium-doped barium zirconate by addition of ZnO, J. Am. Ceram. Soc. 88 (2005) 2362–2368.
[18] C.Y. Regalado Vera, H. Ding, D. Peterson, W.T. Gibbons, M. Zhou, D. Ding, A mini-review on proton conduction of BaZrO3-based perovskite electrolytes, J. Phys.: Energy 3 (2021), 032019.
[19] Y. Yamazaki, R. Hernandez-Sanchez, S.M. Haile, High total proton conductivity in large-grained yttrium-doped barium zirconate, Chem. Mater. 21 (2009) 2755–2762.
[20] C. Madhusudan, V. Kasarapu, M. Chittimadula, Y.S. Reddy, C.V. Reddy, Synthesis and characterization of Y and Dy co-doped ceria solid electrolytes for IT-SOFCs: a microwave sintering, Rare Met. 40 (2018) 3329–3336.
[21] J. Hyodo, K. Kitabayashi, K. Hoshino, Y. Okuyama, Y. Yamazaki, Fast and stable proton conduction in heavily scandium-doped polycrystalline barium zirconate at intermediate temperatures, Adv. Energy Mater. 10 (2020), 2000213.
[22] F.M. Draber, C. Ader, J.P. Arnold, S. Eisele, S. Grieshammer, S. Yamaguchi, M. Martin, Nanoscale percolation in doped $BaZrO_3$ for high proton mobility, Nat. Mater. 19 (2020) 338–346.
[23] E. Fabbri, D. Pergolesi, S. Licoccia, E. Traversa, Does the increase in Y-dopant concentration improve the proton conductivity of $BaZr_{1-x}Y_xO_{3-\delta}$ fuel cell electrolytes? Solid State Ionics 181 (2010) 1043–1051.
[24] O.S. Shusuke Kasamatsu, Takafumi Ogawa, Akihide Kuwabara, Dopant arrangements in Y-doped $BaZrO_3$ under processing conditions and their impact on proton conduction: a large-scale first-principles thermodynamics study, J. Mater. Chem. A 8 (2020) 12674–12686.
[25] U. S, Th. Matzke, Ch. Karmonik, M. Soetratmo, R. Hempelmann, F. Güthoff, Quasielastic thermal neutron scattering experiment on the proton conductor $SrCe_{0.95}Yb_{0.05}H_{0.02}O_{2.985}$, Solid State Ion 86 (1996) 621–628.
[26] P.J. W, J.P. Devlin, Proton trapping and defect energetics in ice from FT-IR monitoring of photoinduced isotopic exchange of isolated D_2O, J. Chem. Phys. 88 (1987) 3086–3091.
[27] Z. Lin, S. Lin, Y. Tian, A. Van Bokkelen, M. Valerio, M.A. Gomez, Oxygen vacancies altering the trapping in the proton conduction landscape of doped barium zirconate, J. Phys. Chem. C 124 (2020) 27954–27964.
[28] H.S. Kim, A. Jang, S.Y. Choi, W. Jung, S.Y. Chung, Vacancy-induced electronic structure variation of acceptors and correlation with proton conduction in perovskite oxides, Angew. Chem. Int. Ed. 55 (2016) 13499–13503.
[29] X. Li, H. Liu, Y. Sun, L. Zhu, X. Yin, S. Sun, Z. Fu, Y. Lu, X. Wang, Z. Cheng, High oxygen evolution activity of tungsten bronze oxides boosted by anchoring of Co^{2+} at Nb^{5+} sites accompanied by substantial oxygen vacancy, Adv. Sci. 7 (2020), 2002242.
[30] X. Ding, Z. Gao, D. Ding, X. Zhao, H. Hou, S. Zhang, G. Yuan, Cation deficiency enabled fast oxygen reduction reaction for a novel SOFC cathode with promoted CO_2 tolerance, Appl. Catal., B 243 (2019) 546–555.
[31] S. Huang, K. Zhu, J. Huang, J. Yang, J. Wang, Z. Fu, R. Peng, Y. Lu, Structure and the enhanced ferromagnetism in single phase $Sr_4Fe_5CoO_{13-\delta}$ ceramic, Ceram. Int. 48 (2022) 19963–19970.
[32] J. Zou, Y. Lin, S. Wu, Y. Zhong, C. Yang, Molybdenum dioxide nanoparticles anchored on nitrogen-doped carbon nanotubes as oxidative desulfurization catalysts: role of electron transfer in activity and reusability, Adv. Funct. Mater. 31 (2021), 2100442.

[33] Y. Zhang, Y. Hu, Z. Wang, T. Lin, X. Zhu, B. Luo, H. Hu, W. Xing, Z. Yan, L. Wang, Lithiation-induced vacancy engineering of Co_3O_4 with improved faradic reactivity for high-performance supercapacitor, Adv. Funct. Mater. 30 (2020), 2004172.
[34] D. Han, K. Toyoura, T. Uda, Protonated $BaZr_{0.8}Y_{0.2}O_{3-\delta}$: impact of hydration on electrochemical conductivity and local crystal structure, ACS Appl. Energy Mater. 4 (2021) 1666–1676.
[35] D. Han, N. Hatada, T. Uda, R. Koc, Chemical expansion of yttrium-doped barium zirconate and correlation with proton concentration and conductivity, J. Am. Ceram. Soc. 99 (2016) 3745–3753.
[36] D.D.C.S. Dr. John, T.S. Irvine, Anthony R. West, Electroceramics: characterization by impedance spectroscopy, Adv. Mater. 2 (1990) 132–138.
[37] R. Pornprasertsuk, O. Kosasang, K. Somroop, M. Horprathum, P. Limnonthakul, P. Chindaudom, S. Jinawath, Proton conductivity of Y-doped $BaZrO_3$: pellets and thin films, Solid State Sci. 13 (2011) 1429–1437.
[38] B.P.U. Graeme Henkelman, Hannes Jónsson, A climbing image nudged elastic band method for finding saddle points and minimum energy paths, J. Chem. Phys. 113 (2000) 9901–9904.
[39] S. Steinberg, R. Dronskowski, The crystal orbital Hamilton population (COHP) method as a tool to visualize and analyze chemical bonding in intermetallic compounds, Crystals 8 (2018) 225.
[40] V.L. Deringer, A.L. Tchougréeff, R. Dronskowski, Crystal orbital Hamilton population (COHP) analysis as projected from plane-wave basis sets, J. Phys. Chem. A 115 (2011) 5461–5466.
[41] B. Silvi, I. Fourré, M.E. Alikhani, The topological analysis of the electron localization function. A Key for a position space representation of chemical bonds, Monatsh. Chem. 136 (2005) 855–879.
[42] G. Henkelman, A. Arnaldsson, H. Jónsson, A fast and robust algorithm for Bader decomposition of charge density, Comput. Mater. Sci. 36 (2006) 354–360.
[43] R. Metzler, J.H. Jeon, A.G. Cherstvy, E. Barkai, Anomalous diffusion models and their properties: non-stationarity, non-ergodicity, and ageing at the centenary of single particle tracking, Phys. Chem. Chem. Phys. 16 (2014) 24128–24164.
[44] H. Niu, Y. Jing, Y. Sun, L. Guo, N.R. Aluru, W. Li, J. Yang, X. Li, On the anomalous diffusion of proton in Y-doped $BaZrO_3$ perovskite oxide, Solid State Ionics 376 (2022), 115859.
[45] P.E. Blochl, Projector augmented-wave method, Phys. Rev. B Condens. Matter 50 (1994) 17953–17979.
[46] G. Kresse, J. Hafner, Ab initio molecular dynamics for open-shell transition metals, Phys. Rev. B Condens. Matter 48 (1993) 13115–13118.
[47] G.F.l. Kresse,J, Efficient iterative schemes for ab initio total-energy calculations using a plane-wave basis set, Phys. Rev. B Condens. Matter 54 (1996) 169–185.
[48] J.P. Perdew, A. Ruzsinszky, G.I. Csonka, O.A. Vydrov, G.E. Scuseria, L. A. Constantin, X. Zhou, K. Burke, Restoring the density-gradient expansion for exchange in solids and surfaces, Phys. Rev. Lett. 100 (2008), 136406.
[49] W.D.R. Shyue Ping Ong, Anubhav Jain, Geoffroy Hautier, Michael Kocher, Shreyas Cholia, Dan Gunter, Vincent L. Chevrier, Kristin A. Persson, Gerbrand Ceder, Python Materials Genomics (pymatgen): a robust, open-source python library for materials analysis, Comput. Mater. Sci. 68 (2013) 314–319.
[50] V. Wang, N. Xu, J.-C. Liu, G. Tang, W.-T. Geng, VASPKIT: a user-friendly interface facilitating high-throughput computing and analysis using VASP code, Comput. Phys. Commun. 267 (2021), 108033.
[51] W. Yi, G. Tang, X. Chen, B. Yang, X. Liu, qvasp: a flexible toolkit for VASP users in materials simulations, Comput. Phys. Commun. 257 (2020), 107535.
[52] D. Broberg, B. Medasani, N.E.R. Zimmermann, G. Yu, A. Canning, M. Haranczyk, M. Asta, G. Hautier, PyCDT: a Python toolkit for modeling point defects in semiconductors and insulators, Comput. Phys. Commun. 226 (2018) 165–179.
[53] C. Braga, K.P. Travis, A configurational temperature Nose-Hoover thermostat, J. Chem. Phys. 123 (2005), 134101.
[54] D.J. Evans, B.L. Holian, The nose–hoover thermostat, J. Chem. Phys. 83 (1985) 4069–4074.
[55] S. Maintz, V.L. Deringer, A.L. Tchougréeff, R. Dronskowski, LOBSTER: a tool to extract chemical bonding from plane-wave based DFT, J. Comput. Chem. 37 (2016) 1030–1035.

IOPscience iopscience.iop.org

Home Search Collections Journals About Contact us My IOPscience

Hydrothermal synthesis and photocatalytic properties of layered $La_2Ti_2O_7$ nanosheets

This article has been downloaded from IOPscience. Please scroll down to see the full text article.

2006 Nanotechnology 17 4863

(http://iopscience.iop.org/0957-4484/17/19/014)

View the table of contents for this issue, or go to the journal homepage for more

Download details:
IP Address: 128.135.12.127
The article was downloaded on 23/02/2013 at 04:58

Please note that terms and conditions apply.

Nanotechnology **17** (2006) 4863–4867 doi:10.1088/0957-4484/17/19/014

Hydrothermal synthesis and photocatalytic properties of layered $La_2Ti_2O_7$ nanosheets

KunWei Li, Yan Wang, Hao Wang[1], Mankang Zhu and Hui Yan

The College of Materials Science and Engineering, Beijing University of Technology, Beijing 100022, People's Republic of China

E-mail: haowang@bjut.edu.cn

Received 17 July 2006, in final form 1 August 2006
Published 11 September 2006
Online at stacks.iop.org/Nano/17/4863

Abstract
Layered $La_2Ti_2O_7$ nanosheets were prepared through a one-step hydrothermal method at low temperature. The concentration of NaOH mineralizer plays an important role in the synthesis. The scanning electron microscopy (SEM) and transmission electron microscopy (TEM) images show that the thickness of every nanosheet is about 5–10 nm, while the planar dimension is more than 1 μm. The photo-catalytic activities of the nanosheets were characterized by the decolourization of methyl orange solution and the evolution rate of H_2. The results demonstrated that the $La_2Ti_2O_7$ nanosheets possess significantly improved photocatalytic properties in water purification and evolution rate of H_2 from water–ethanol solution compared with those of samples prepared by conventional solid-state reaction.

1. Introduction

Lanthanide titanate ($La_2Ti_2O_7$), as one of the layered compounds, has attracted widespread attention [1, 2] in the photocatalytic domain due to its unique layered structure and chemical activity [3]. Hwang *et al* have reported that $La_2Ti_2O_7$ loaded with Ni–NiO has good photocatalytic activity in the water-splitting reaction [4] and the destruction of volatile organic compounds (CH_3Cl) [5]. However, they obtained $La_2Ti_2O_7$ through the conventional solid-state reaction (SSR) or the polymerizable complex method [6]. Both of these methods need high temperatures and produce materials with low specific surface areas, nonuniform particle sizes and low phase purity, which lead to a relatively low photocatalytic activity. Zhang *et al* [7] obtained nanopowders of $La_2Ti_2O_7$ by a metallorganic decomposition method. However, no photocatalytic properties were reported in their study. The desired catalyst should have high specific surface area, uniform particle sizes and pure phase. Therefore, if we control the experimental parameters to synthesize the layered compounds with a few layers, that is to say, nanosheets, the products will better answer our desire for a catalyst.

The use of solution-based soft chemical methods to prepare nanocrystalline materials is expected to result in chemically homogeneous and phase-pure specimens, a narrow particle size distribution, and low crystallization temperatures of the materials. Among many methods, hydrothermal synthesis is particularly promising for low-cost production of advanced catalysts on a large scale. Because crystalline powders are directly produced in a hydrothermal process, the needs for high-temperature calcination and milling procedures are eliminated. Chen and Xu [8] have prepared $La_2Ti_2O_7$ powders through the hydrothermal treatment of $La_2O_3{\cdot}2TiO_2{\cdot}nH_2O$ coprecipitation, but this method needs two steps to get the final product, and they did not get the $La_2Ti_2O_7$ nanosheets.

Here, we report the simple hydrothermal synthesis of $La_2Ti_2O_7$ two-dimensional (2D) nanosheets. The products were used as photocatalysts for the decolourization of methyl orange solution and the evolution of H_2 from water–ethanol solution.

2. Experimental details

2.1. Synthesis

All reagents were of analytical grade and purchased from Beijing Chemical Reagent Ltd without further purification. The exact quantity of La in the lanthanum nitrate hydrate,

[1] Author to whom any correspondence should be addressed.

0957-4484/06/194863+05$30.00 © 2006 IOP Publishing Ltd Printed in the UK

$La(NO_3)_3{\cdot}6H_2O$, was determined by thermo-gravimetric analysis. The equivalent molar quantities of $La(NO_3)_3{\cdot}6H_2O$ and $Ti(SO_4)_2$ were dissolved in deionized water to form the clear aqueous solution. Then some amount of NaOH solution was dropped into the above solution to form white precipitation mixtures with different nominal NaOH concentrations. The mixture was stirred at ambient temperature for 10 min and then sealed in a 50 ml Teflon lined stainless-steel autoclave and allowed to heat at temperatures ranging from 140 to 220 °C for different reaction times. After reaction, the resulting powders were repeatedly washed by centrifugation and decantation with deionized water to remove undesirable anions such as Na^+, SO_4^{2-} and NO_3^-, and then dried at 80 °C for 6 h to get the products.

2.2. Characterization

The structure of the products was examined by an x-ray diffractometer (XRD, Bruker Advance D8) using Cu Kα radiation (λ = 1.5406 Å). Transmission electron microscopy (TEM) and selected area electron diffraction (SAED) were taken on a JEOL-JEM 2010F transmission electron microscope, using an accelerating voltage of 200 kV. The specific surface areas of the powders were determined using a Micromeritics ASAP 2020 specific surface area and porosity analyser in the method of Brunauer–Emmett–Teller (BET) nitrogen adsorption and desorption. Optical absorption studies were carried out using an ultraviolet–visible–near-infrared (UV–vis–NIR) spectrophotometer (Shimadzu UV-3101PC).

The decolourization of methyl orange solution was carried out in our home-made instruments. At first, 0.2 g of product was dispersed into five beakers which were filled with 100 ml of 10 mg l^{-1} methyl orange solution separately, and irradiated under ultraviolet light by using a light resource (400 W high-pressure Hg lamp) for different times, and then characterized by UV–vis spectroscopy.

The evolution of H_2 from water–ethanol solution was carried out at room temperature and atmospheric pressure in a closed gas circulation system containing air using a high-pressure Hg lamp (400 W) placed in an inner irradiation-type quartz reaction cell. The catalyst (1 g) was suspended in 1:5 ethanol/water solution (500 ml). The amounts of H_2 and O_2 were determined by a Shimadzu 14C gas chromatograph (molecular sieve 5 Å column and Ar carrier) equipped with TCD.

3. Results and discussion

In order to obtain suitable NaOH concentrations, samples were synthesized under different NaOH concentrations (a: 0 M; b: 1 M; c: 3 M) at 200 °C for 24 h. As can be seen from the XRD patterns in figure 1, three kinds of materials were formed at different NaOH concentrations. Figure 1(a) was ascribed to TiO_2 with a tetragonal system (JCPDS 78-2486) while figure 1(c) was attributed to lanthanium hydroxide $La(OH)_3$ with the hexagonal system (JCPDS 83-2034). Figure 1(b) shows pure monoclinic phase $La_2Ti_2O_7$ with a perovskite structure conforming to the $P2_1$ space group (JCPDS 81-1066). It could be seen that the concentration of NaOH played a critical role in the formation of phase-pure $La_2Ti_2O_7$ by the hydrothermal method.

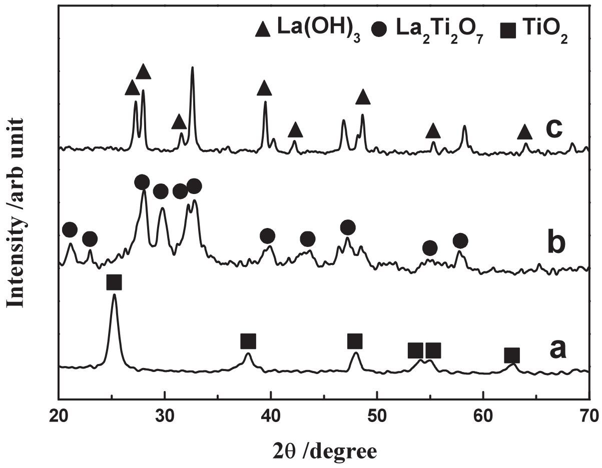

Figure 1. XRD of $La_2Ti_2O_7$ samples synthesized at 200 °C for 24 h in different concentration of NaOH (a: 0, b: 1, c: 3 mol l^{-1}).

According to the above results and the related experiments, we conclude that the following reactions might occur in this process:

$$Ti^{4+} + 3H_2O \rightleftharpoons H_2TiO_3 + 4H^+ \quad (1)$$

$$H_2TiO_3 \xrightleftharpoons{\text{heat}} TiO_2 \downarrow + H_2O. \quad (2)$$

$$La^{3+} + 3OH^- \longrightarrow La(OH)_3 \downarrow \quad (3)$$

$$2La(OH)_3 + 2H_2TiO_3 \longrightarrow La_2Ti_2O_7 \downarrow + 5H_2O \quad (4)$$

$$H_2TiO_3 + 2OH^- + H_2O \longrightarrow Ti(OH)_6^{2-}. \quad (5)$$

It is well known that the mineralizers such as KOH or NaOH play a fundamental part in the hydrothermal process [9, 10]. When there was no NaOH in the solution, the $La(NO_3)_3$ and $Ti(SO_4)_2$ were ionized. Due to the stronger hydrolysis effect of Ti^{4+} ions, equation (1) occurred and led to the formation of colloidal deposition H_2TiO_3. When these reactants were sealed in an autoclave and heated, TiO_2 deposits were formed from the decomposition equation (2). However, La^{3+} ions were still kept in the solution and were washed away after reaction. When the concentration of NaOH was 1 M, the H^+ ions formed from the strong hydrolysis effect of Ti^{4+} ions were neutralized by NaOH. The neutralization of H^+ led to the break of the equilibrium of equation (1) and the formation of colloidal H_2TiO_3. At the same time, $La(OH)_3$ precipitates were formed through equation (3). Then, during the hydrothermal process, $La_2Ti_2O_7$ precipitates were formed from the neutralization equation (4) between $La(OH)_3$ and H_2TiO_3, because the solubility of $La_2Ti_2O_7$ was lower than that of $La(OH)_3$ under this condition. However, when the concentration of NaOH was 3 M, the $La(OH)_3$ (formed by equation (3)) was very stable and could not be dissolved, as was shown in figure 1(c). In the meantime, Ti species were washed away after hydrothermal reaction according to equation (5).

Figure 2 shows the XRD patterns for the samples synthesized using 1 M NaOH for 24 h under different temperatures. From figure 2(a), we can see that the products

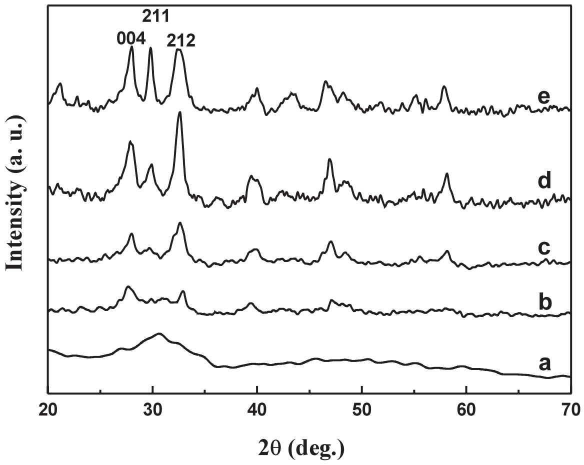

Figure 2. XRD for the samples synthesized using 1 M NaOH under different temperatures (a: 140 °C; b: 160 °C; c: 180 °C; d: 200 °C; e: 220 °C) by the hydrothermal method for 24 h.

were amorphous when the synthesis temperature was 140 °C. With an increase in temperature, the main peaks (004, 211 and 212) of $La_2Ti_2O_7$ gradually became obvious. As can be seen from figures 2(d) and (e), when the temperature was above 200 °C, phase-pure $La_2Ti_2O_7$ crystals were obtained. The following measurements were based on the sample synthesized at 200 °C using 1 M NaOH as the mineralizer.

In figure 3, the SEM and TEM image show the nanosheet morphology of the $La_2Ti_2O_7$ synthesized by the hydrothermal method. The SAED pattern was obtained by aligning the electron beam perpendicular to the face of this plate. The regular spot pattern indicates single-crystalline structure. The clear lattice fringes shown in the high-resolution (HRTEM) image of the selected area marked by the square in figure 3(b) indicates that the nanosheet presented perfect crystal structure without observed defects. The spacing of 0.5091 nm corresponds to the (110) planes of $La_2Ti_2O_7$. Interestingly, although the planar dimension is more than 1 μm, the plate is transparent under the electron beam, indicating that the plate is very thin. It can be estimated from the SEM image that the thickness is about 5–10 nm. The formation of a thin nanosheet may be decided by the structure of $La_2Ti_2O_7$ and the solution environment for crystal growth. In the structure of $La_2Ti_2O_7$ with a monoclinic cell [11], the spacing of layers results in a feeble binding power and it is favourable to grow in a layered structure. In the hydrothermal condition, the ions can freely assemble according to the relative specific surface energies associated with the facets of this crystal [12]. However, in the solid-state reaction, the obvious diffusion of ions occurred only at high temperature. At the same time, the rapid growth of particle size at high temperature made it difficult to form nanoparticles. To the best of our knowledge, there have been no reports on the $La_2Ti_2O_7$ nanosheets to date.

The specific surface area of the $La_2Ti_2O_7$ sample synthesized by the hydrothermal method was approximately 69.8 $m^2\ g^{-1}$. However, that of the $La_2Ti_2O_7$ sample synthesized by the solid-state method at 1050 °C was approximately 1.2 $m^2\ g^{-1}$, which was consistent with that of [13]. This indicates that the hydrothermal method significantly enlarges the specific surface area of the sample.

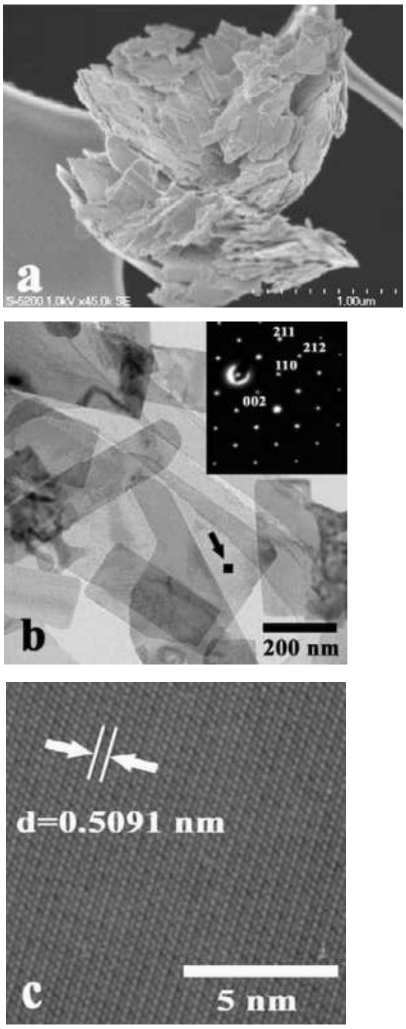

Figure 3. SEM and TEM images of the $La_2Ti_2O_7$ prepared at 200 °C by using the hydrothermal method: (a) SEM; (b) TEM (SAED inserted); and (c) HRTEM images of the selected area marked by the square in figure 2(b).

The higher specific surface area would affect the photocatalytic efficiency.

In figure 4, the steep shape of the spectra indicated that the visible light absorption was not due to the transition from the impurity level but was due to the band-gap transition [14]. Compared to the SSR sample, the absorption of $La_2Ti_2O_7$ nanosheets appeared to red-shift obviously. The band gap of the nanosheets was estimated to be 2.92 eV from the onset of the absorption edge, which was smaller than the SSR sample (3.31 eV). This can be attributed to the nanosize effect. As is well known, the red-shift phenomenon mainly resulted from the surface effect which occurs widely in the absorption of nanoparticle size.

In figure 5, curve (a) shows the decolourization curve of absolute methyl orange solution without any $La_2Ti_2O_7$ sample irradiated under ultraviolet light for 50 min, while other curves denote methyl orange solution with a 2 g l^{-1} $La_2Ti_2O_7$ sample irradiated for different times (b: 10 min, c: 20 min, d: 30 min, e: 40 min, f: 50 min). Seen from the figure, the solutions with the $La_2Ti_2O_7$ sample decoloured gradually

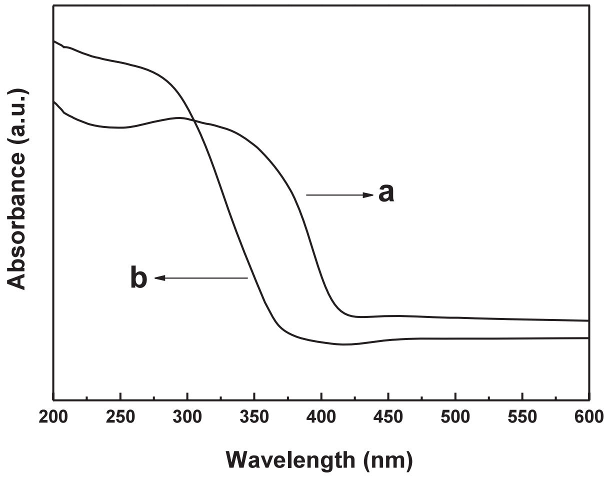

Figure 4. The UV–vis spectra of $La_2Ti_2O_7$ sample synthesized (a: by the hydrothermal method at 200 °C; b: by the solid-state method at 1050 °C).

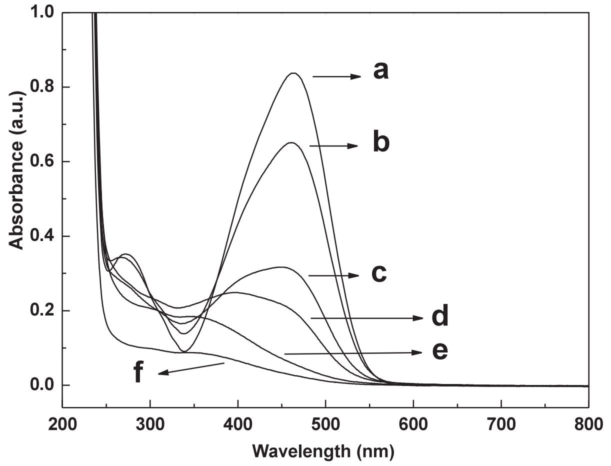

Figure 5. Decolourization curves of methyl orange solutions irritated under ultraviolet light (a) without $La_2Ti_2O_7$ for 50 min, (b) with $La_2Ti_2O_7$ for 10 min, (c) with $La_2Ti_2O_7$ for 20 min, (d) with $La_2Ti_2O_7$ for 30 min, (e) with $La_2Ti_2O_7$ for 40 min, and (f) with $La_2Ti_2O_7$ for 50 min.

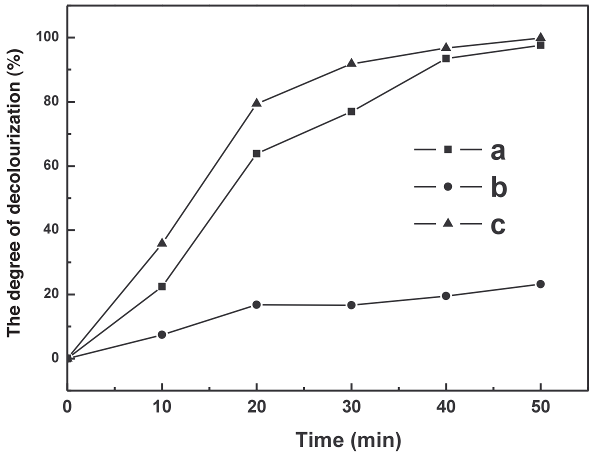

Figure 6. The degree of decolourization of methyl orange solution with the role of $La_2Ti_2O_7$ samples and Degussa P25 (a: hydrothermal; b: SSR; c: P25).

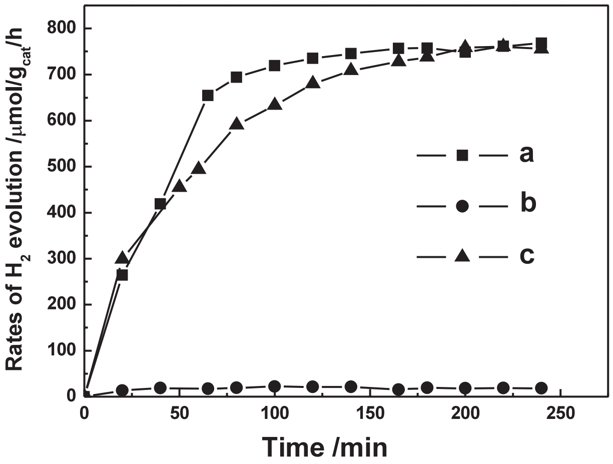

Figure 7. The rates of H_2 evolution with the role of $La_2Ti_2O_7$ samples synthesized by (a) hydrothermal method; (b) solid state method; and (c) P25 under ultraviolet light for different times.

and were almost decoloured totally when the irradiation time was up to 50 min. Obviously, the $La_2Ti_2O_7$ sample played the role of a photocatalyst. We have compared the XRD patterns of the $La_2Ti_2O_7$ sample before and after photocatalytic measurements. It confirmed that the photocatalytic reaction did not change the crystal structure of the catalyst. So the catalysts can be recycled and used again.

As can be seen from figure 6, the solutions decoloured gradually with prolonged irradiation. After 50 min, the decolourization degree of methyl orange solution with $La_2Ti_2O_7$ sample synthesized by the hydrothermal method reached 98%, while it is still lower than that with TiO_2, whose rate is up to nearly 100%. In spite of this, the $La_2Ti_2O_7$ sample synthesized by the hydrothermal method may find potential application due to its chemical and structural stability in solution [15]. Comparatively, the photocatalytic abilities of the $La_2Ti_2O_7$ sample synthesized by the solid-state method are weaker; its decolourization degree is just 20% after 50 min. This may result from the reduction in the sample specific surface area and the decrease in the active sites on the surface of the samples.

From figure 7, the rates of H_2 evolution from water–ethanol solution with P25 and hydrothermal $La_2Ti_2O_7$ were about 750 μmol g^{-1} h^{-1} at 160 min. However, the rate with the sample synthesized by SSR was only about 20 μmol g^{-1} h^{-1}. All the samples were not loaded with nickel or other metals. The rate of H_2 evolution on hydrothermal $La_2Ti_2O_7$ was about 37.5 times larger than that of the SSR sample. As was shown by BET measurements, the specific surface areas of the hydrothermal samples is about 60 times larger than that of the SSR sample. It was noted that the photocatalytic rate is not proportional to the surface area. The reasons for this phenomenon could be explained by the following: the specific surface areas of the powders in this study were determined by nitrogen adsorption and desorption. In this vapour atmosphere during the measurement, the surface of the samples could be adequately exposed and detected. However,

in the case of photocatalytic measurement, the samples were dispersed in the solution with magnetic stirring; even so, only the surface that the light irradiated could produce electrons and holes. Due to the agglomeration and stacking of some powders, one could not ensure that the light could irradiate the entire surface of the sample. As a result, parts of surface were 'lost' during the photocatalytic measurements, and the quantity of photocatalytic results is not proportional to the results of surface areas. Similar results have also been reported in the literature [16–18]. However, after all, it could be seen that the photocatalytic properties of hydrothermal-synthesized $La_2Ti_2O_7$ samples have been significantly improved compared with the SSR samples.

The reason for the high H_2 evolution with hydrothermal $La_2Ti_2O_7$ may lie in the following two aspects. Firstly, the nanosheet thickness is about 5 nm, which is equivalent to that of a few layers of TiO_6 octahedron. Photo-produced electrons and holes will rapidly diffuse to the surface of a nanosheet, which decreases the combination of electrons and holes. Secondly, the larger specific surface area will supply more chance for contact between photo-produced carriers and water or organics.

4. Conclusions

In summary, layered $La_2Ti_2O_7$ nanosheets have been prepared through the one-step hydrothermal method at 200 °C for 24 h when the NaOH concentration equals 1 M. The XRD and the SAED patterns confirm that the products are well-crystallized single crystals. The TEM images show samples with a nanosheet shape whose width is about 150 nm and length is near to 300 nm. The specific surface area of the $La_2Ti_2O_7$ sample synthesized by the hydrothermal method was approximately 69.8 $m^2\ g^{-1}$, which was about 60 times that of the $La_2Ti_2O_7$ sample synthesized by the solid-state method. Photocatalytic measurements through the photo-decolourization of methyl orange show that the $La_2Ti_2O_7$ nanosheets possess superior photocatalytic properties in water purification and may find potential application in related fields. The rate of H_2 evolution on the hydrothermal $La_2Ti_2O_7$ without loading of nickel was about 750 μmol $g^{-1}\ h^{-1}$ from water–ethanol solution, which was remarkably improved compared with that of the SSR sample. The high H_2 evolution rate may result from the unique nanosheet structure and higher specific surface area. A change of material synthesis method may be an effective route to improve the material properties.

Acknowledgment

The authors are grateful to the Project of New Star of Science and Technology of Beijing for financial support.

References

[1] Shangguan W and Yoshida A 2002 *J. Phys. Chem.* B **106** 12227
[2] Yoshimura J, Ebina Y, Kondo J, Domen K and Tanaka A 1993 *J. Phys. Chem.* **97** 1970
[3] Yeong K I, Samer S, Munir J H and Thomas E M 1991 *J. Am. Chem. Soc.* **113** 9561
[4] Kim H, Hwang D W, Kim J, Kim Y and Lee J S 1999 *Chem. Commun.* **12** 1077
[5] Hwang D W, Cha K Y, Kim J, Kim H G, Bae S W and Lee J S 2003 *Ind. Eng. Chem. Res.* **42** 1184
[6] Kim H G, Hwang D W, Bae S W, Jung J H and Lee J S 2003 *Catal. Lett.* **91** 193
[7] Zhang Z T, Zhang Y M, Yang J, Li H, Song W and Zhao X Q 2005 *J. Ceram. Soc. Japan* **113** 67
[8] Chen D and Xu R 1998 *Mater. Res. Bull.* **33** 409
[9] Lencka M M and Riman R E 1995 *Chem. Mater.* **7** 18
[10] Wang H, Liu J B, Zhu M K, Wang B and Yan H 2003 *Mater. Lett.* **57** 2371
[11] Abe R, Higashi M, Sayama K, Abe Y and Sugihara H 2006 *J. Phys. Chem.* B **110** 2219
[12] Li W J, Shi E W, Zhong W Z and Yin Z W 1999 *J. Cryst. Growth* **203** 186
[13] Kim H G, Hwang D W, Bae S W, Jung J H and Lee J S 2003 *Catal. Lett.* **91** 193
[14] Kudo A, Tsuji I and Kato H 2002 *Chem. Commun.* **17** 1958
[15] Fuierer P A and Newnham R E 1991 *J. Am. Ceram. Soc.* **74** 2876
[16] Yoshino M, Kakihara M, Cho W S, Kato H and Kudo A 2002 *Chem. Mater.* **14** 3369
[17] Fei D Q, Hudaya T and Adesina A A 2005 *Catal. Commun.* **6** 253
[18] Ikeda S, Hara M, Kondo J N, Domen K, Takahashi H, Okubo T and Kakihara M 1998 *J. Mater. Res.* **13** 852

ACS Catalysis

Research Article

Cite This: ACS Catal. 2018, 8, 122−131

pubs.acs.org/acscatalysis

Au Nanorod Photosensitized $La_2Ti_2O_7$ Nanosteps: Successive Surface Heterojunctions Boosting Visible to Near-Infrared Photocatalytic H_2 Evolution

Xiaoyan Cai,[†,‡] Mingshan Zhu,[‡] Ossama A. Elbanna,[‡] Mamoru Fujitsuka,[‡] Sooyeon Kim,[‡] Liang Mao,[†] Junying Zhang,[*,†] and Tetsuro Majima[*,‡]

[†]Key Laboratory of Micro-nano Measurement, Manipulation and Physics (Ministry of Education), Department of Physics, Beihang University, Beijing 100191, P. R. China

[‡]The Institute of Scientific and Industrial Research (SANKEN), Osaka University, Mihogaoka 8-1, Ibaraki, Osaka 567-0047, Japan

Supporting Information

ABSTRACT: Visible and near-infrared (NIR) light utilization is a high-priority target for solar-to-chemical energy conversion. In this work, a promising surface heterojunction-based plasmonic photocatalyst was developed by integrating Au nanorods (NRs) with $La_2Ti_2O_7$ nanosteps (Au-LTO NSP) for photocatalytic H_2 evolution in visible and near-infrared (NIR) regions. At wavelengths longer than 420 nm, Au-LTO NSP displayed H_2 production rate that was separately 2.4 and 4.7 times that of Au-LTO nanosheets (NS) and Au−P25 composites, using methanol as the sacrificial agent. At wavelengths longer than 780 nm, the enhancement was 2.3 and 5.8 times, respectively. The high apparent quantum efficiency (AEQ) of 1.4% at 920 nm irradiation makes the Au-LTO NSP photocatalyst especially efficient for the NIR light utilization. The broadband photocatalytic activity of Au-LTO NSP was mainly caused by longitudinal surface plasmon resonance of Au NRs, generating and injecting hot electrons into LTO NSP. Substantial electrons transferred from Au NRs to the (010) facets and then directionally migrated to the (012) facets of LTO NSP, as consequence of the successive (010) and (012) surface heterojunctions within a LTO NSP single particle. The unique step structure of LTO retarded the recombination of the photoinduced electrons and holes in Au NRs, showing the powerful role of the semiconductor surface heterojunction in favoring the plasmon-induced interfacial hot electron transfer.

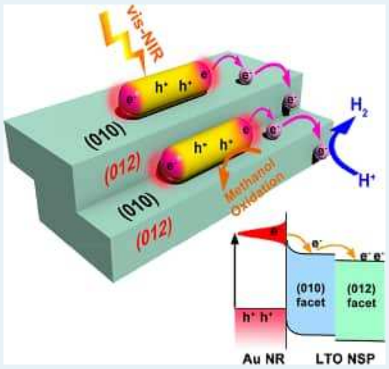

KEYWORDS: *plasmonic photocatalysis, vis−NIR light, surface heterojunction, hot electron transfer*

INTRODUCTION

Hydrogen (H_2) has been known as an ideal source of energy due to its environmental friendliness and high energy capacity.[1] Since the pioneering work on photoelectrocatalytic splitting of water by TiO_2 under UV light irradiation,[2] H_2 evolution via photocatalytic water splitting has been extensively studied and shown to be a promising solution for alleviating worldwide energy issues.[3−5] TiO_2 is the most common semiconductor photocatalyst used for solar H_2 generation.[6] However, because of the large band gap of TiO_2, its light harvesting is limited in UV light region which only accounts for ca. 4% of the solar spectrum.[5] Fully harvesting solar light, from the visible to near-infrared (NIR) region, is always the goal and challenge for photocatalysis.[7] Unfortunately, only a few semiconductors reveal NIR activity. Because of the low photon energy or strong thermal effect, narrow bandgap semiconductors with NIR light absorption usually either lack photocatalytic activity or convert NIR light energy into heat directly.[8] For example, black hydrogenated TiO_2 revealed significantly broadened absorption in the vis−NIR region, but only ca. 1% enhancement of the photocatalytic activity has been achieved.[9−11] Up-conversion materials were used by many researchers to convert NIR light into visible and UV light.[12−14] For instance, NIR photocatalytic degradation of organic dyes was achieved by Yb^{3+} and Tm^{3+} codoped mesoporous TiO_2, but the overall conversion efficiency was significantly low since a 980 nm laser had to be used.[12] Recently, the surface plasmon resonance (SPR) effect has been exploited to realize photocatalytic splitting of water in the NIR region.[15−20] Depending sensitively on the nanostructure of the metal and the dielectric properties of the surrounding environment, the SPR bands of the plasmonic metal are tunable from UV to NIR.[15] When a plasmonic metal is coupled to a semiconductor, the plasmon energy can transfer from the metal to the semiconductor, enhancing charge generation via hot electron/hole transfer, photonic enhancement, and local electromagnetic field enhancement.[16,21,22] In addition, by forming Schottky barriers, the recombination of photoexcited electron−holes can be significantly reduced. For example, Au nanorods (NRs)/CdS composite photocatalysts were fabricated to generate H_2 under

Received: August 31, 2017
Revised: November 2, 2017
Published: November 21, 2017

Downloaded via NANJING TECH UNIV on February 1, 2025 at 06:54:04 (UTC). See https://pubs.acs.org/sharingguidelines for options on how to legitimately share published articles.

ACS Publications © 2017 American Chemical Society

DOI: 10.1021/acscatal.7b02972
ACS Catal. 2018, 8, 122−131

vis−NIR light irradiation.[23] By tuning the ratio of length and width, the optical resonance of Au NRs covers most of the solar spectrum, which can be used to design panchromatic absorbers. Accordingly, Au NRs decorated semiconductors have offered a new opportunity to achieve more efficient utilization of the solar spectrum.

The geometry and location of the plasmonic metallic nanostructures relative to the neighboring semiconductors, as one of the most crucial factors for efficient hot electron transfer from plasmonic nanostructures to semiconductor materials, have been intensively studied.[24−28] Recently, Majima's group found that a large part of electrons could transfer from Au nanospheres to TiO_2 with coexposed {001} and {101} facets while only a few electrons to TiO_2 nanoparticles through SPR excitation using the time-resolved diffuse reflectance spectroscopy.[29] The electron transport dynamics from plasmonic metal to the neighboring semiconductor are also greatly influenced by the geometry (morphology, structure, and exposed crystal surface) of the semiconductor.[30−32] Particularly, plasmon-induced chemical reactions of Au nanostructures loaded on semiconductor substrates with coexposed different crystal surfaces have attracted growing attention in photocatalysis, because the directional migration of hot electrons in the semiconductor can be facilitated by taking advantage of the Schottky barrier between the Au nanostructure and the crystal surface of the semiconductor.[29,33] However, to the best of our knowledge, few papers deal with optimizing the exposed crystal surface of the semiconductor to efficiently achieve Au NR induced broad spectrum photocatalytic H_2 evolution.

Herein, we propose a new design for the efficient utilization of a broad spectrum from visible to NIR light toward enhanced plasmonic photocatalytic activity in H_2 evolution. Au NRs with visible and NIR plasmonic absorption were decorated on the surface of $La_2Ti_2O_7$ nanosteps (Au-LTO NSP), which have been demonstrated to exhibit higher electron flow and remarkably long-lived charges compared to nanoparticles (NP) and nanosheets (NS) because of the successive surface heterojunctions.[34] The photocatalytic H_2 generation performance of the Au-LTO NSP composite was significantly higher than that of Au-LTO NS and Au−P25, under wavelengths longer than 420 and 780 nm light irradiation. Especially, the Au-LTO NSP composite performs well in the NIR region, promising the more efficient utilization of the long-wavelength solar light in comparison with similar materials. Single-particle photoluminescence (PL) spectroscopy and femtosecond time-resolved transient absorption measurements provided direct evidence that the plasmon-induced interfacial hot electrons transfer from Au NRs to LTO nanostructures. The effect of the successive surface heterojunctions of LTO NSP on the interfacial hot electron transfer dynamics was fully investigated.

■ RESULTS AND DISCUSSION

Morphology, Structure, and Optical Properties. The synthesis procedures of Au NRs decorated LTO nanosteps (Au-LTO NSP) composites by a facile self-assembly approach are schematically shown in Figure 1a. Typically, Au NRs samples with a longitudinal surface plasmon resonance (LSPR) absorption peak located at 780, 860, and 940 nm (Figure S1a) were prepared through seed-mediated strategy and then attached on the mercaptopropionic acid (MPA)-functionalized LTO nanostructures by ligand exchange for the construction of Au-LTO composites. The intermediate ligand of MPA can replace the original capping agent CTAB on Au NRs,

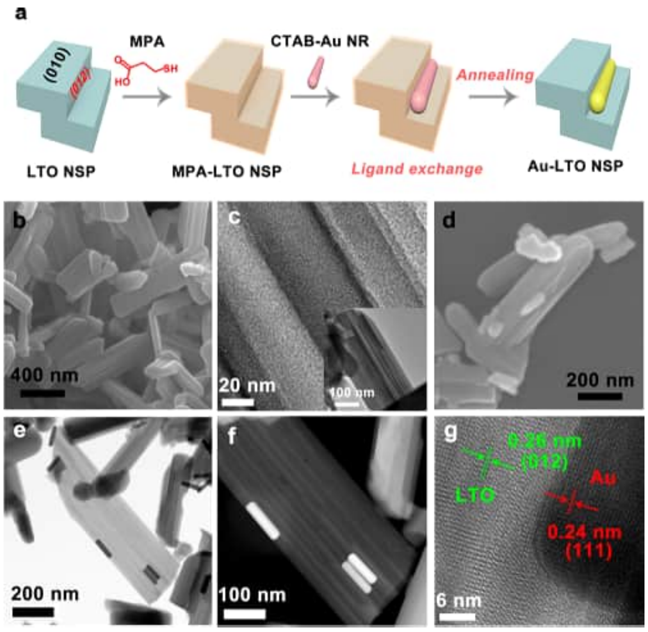

Figure 1. Schematic illustration of the preparation for Au-LTO NSP composites (a). SEM (b) and TEM (c) images of LTO NSP. SEM (d), TEM (e, f), and HRTEM (g) images of Au-LTO NSP.

facilitating a strong interaction between Au and LTO through its thiol and carboxylic groups, respectively.[35] The samples are denoted as 780-, 860-, and 940-Au-LTO NSP, respectively, based on the absorption maximum. Scanning electron microscopy (SEM) and transmission electron microscopy (TEM) images were taken to analyze the morphology of the samples. As seen from Figure S1b-d and Table S1, Au NRs in three samples exhibit different aspect ratios, leading to the different light harvesting ability (Figure S1a). Figure 1b shows a SEM image of three-dimensional (3D) LTO crystals in a polygonal cross profile with about 100−500 nm size obtained through a molten-salt growth process. The uniform strip-like structures on the surface of LTO particles are the edge/boundary of steps. The TEM image demonstrates that the nanostep is enclosed by several well-defined terraces through internal and external stairways (Figure 1c). The LTO NSP are single-crystalline and grow by stacking well-organized multi-layers along the direction of [010], with periodically coexposed high-energy (012) facets and low-energy (010) facets as explained in our previous work.[34] Under high temperature (1100 °C) condition, the formation energy of (010) surfaces is increased while that of the (012) surface decreased because the molten salt ions (Na^+ or Cl^-) enriched in (012) surfaces to prevent the crystal growth along the [012] direction, inducing the exposure of (012) facets and thus the formation of the step structure. With regard to 860-Au-LTO NSP composites, it can be observed from Figure 1d−f that the Au NRs are well loaded on the step-structured LTO surfaces by the effective 1D/3D interfacial interaction. The high resolution TEM (HRTEM) image of the interface of LTO and Au regions shows the identified lattice spacing of 0.26 and 0.24 nm, corresponding to the (012) facet of the monoclinic phase LTO and the (111) crystal plane of the cubic phase Au, respectively (Figure 1g). Scanning transmission electron microscopy (STEM) and energy dispersive X-ray spectroscopy (EDS) elemental mapping in Figure S2 further confirm the decoration of Au NRs on LTO NSP. The EDS analysis of the whole area of

DOI: 10.1021/acscatal.7b02972
ACS Catal. 2018, 8, 122−131

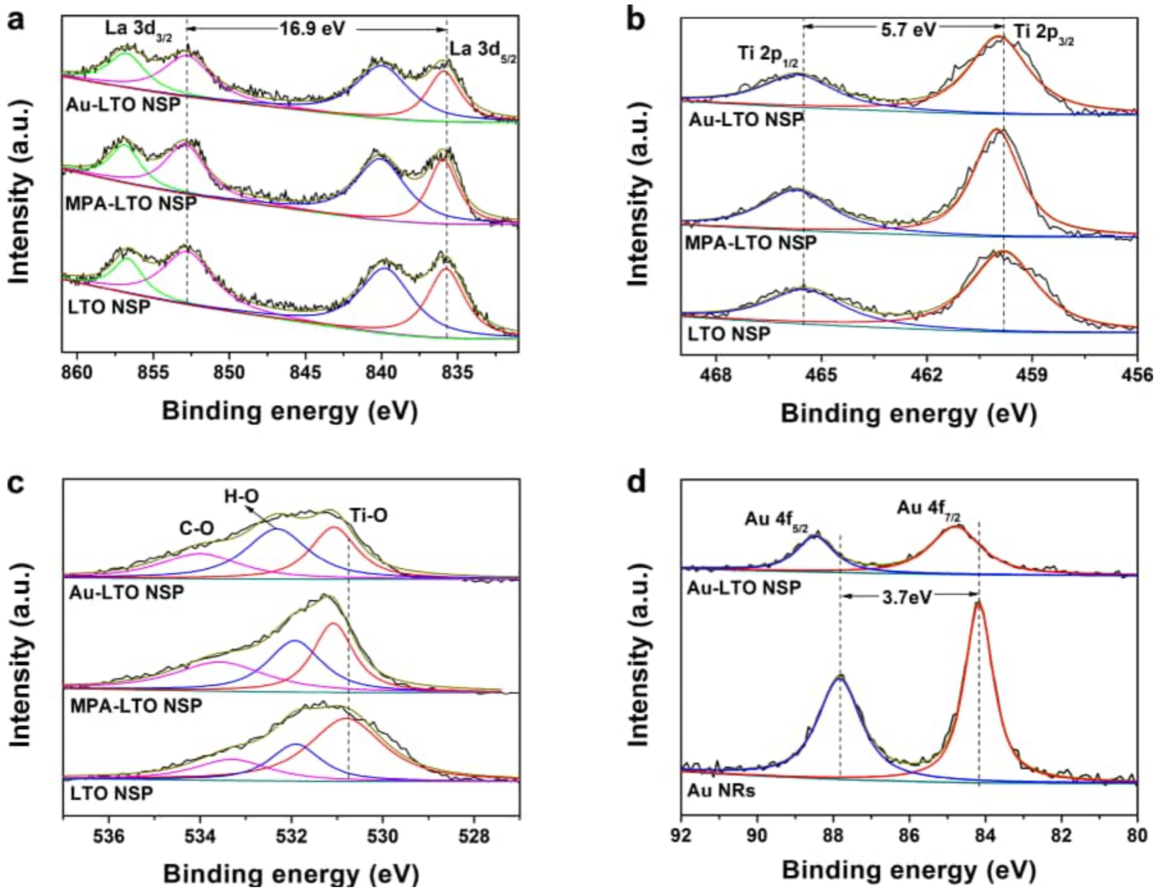

Figure 2. High-resolution XPS of La 3d (a), Ti 2p (b), O 1s (c), and Au 4f (d) in LTO NSP, MPA-LTO NSP, Au-LTO NSP, and Au NRs samples.

Figure 1e showed that weight ratio of La, Ti, O, and Au is 2.5:1.0:0.70:0.13 (Figure S3), indicating that the Au-LTO NSP sample contains about 3 wt % of Au.

The crystal and chemical structures of as-synthesized Au-LTO composites were investigated by X-ray diffraction (XRD). Figure S4 shows the XRD patterns of LTO NSP and 860-Au-LTO NSP decorated with 3 wt % Au NRs. It is clearly seen that both samples exhibit good crystallinity and distinct diffraction peaks corresponding to perovskite structured LTO with the monoclinic crystalline phase (JCPDS no. 70-0903). The LTO phase in Au-LTO NSP composites displays similar diffraction peaks to those of LTO NSP, indicating negligible structural modifications of LTO NSP after its adsorption of Au NRs by the surface functionalized process. The XRD patterns of Au-LTO NSP composites indicate two additional peaks at 38.4° and 44.6°, corresponding to the (111) and (200) crystal planes of cubic Au (JCPDS no. 04-0784), respectively.

To learn in-depth fundamental information on the interaction of Au NRs with LTO NSP, X-ray photoelectron spectroscopy (XPS) of La 3d, Ti 2p, O 1s, and Au 4f of the samples was measured as shown in Figure 2, and their survey spectra are presented in Figure S5. First, La 3d spectra of LTO NSP show doublet peaks at 835.8 and 852.7 eV with good symmetry (Figure 2a), indicating that the La ions are in the oxidation state La^{3+}.[36] Second, the bare LTO sample exhibits symmetric Ti $2p_{3/2}$ and $2p_{1/2}$ peaks at 459.8 and 465.5 eV, respectively (Figure 2b), with a peak separation of 5.7 eV, being in good agreement with the energy splitting reported for TiO_2.[36,37] Third, the O 1s region (Figure 2c) was fitted by three peaks in LTO, in which the Ti−O appeared at 530.8 eV, the physisorption of (OH) and chemisorbed H_2O appeared at 531.7 eV, and the C−O bond appeared at 533.1 eV.[38] As for MPA-LTO NSP and Au-LTO NSP sample, the above assigned peaks (La 3d, Ti 2p, and Ti−O) are positively shifted by 0.2−0.4 eV, as compared to those for LTO NSP. This phenomenon can be explained by the surficial interaction of the MPA ligand with LTO NSP. For Au 4f spectra, as shown in Figure 2d, around 3.7 eV of peak separation between Au $4f_{7/2}$ (84.1 eV) and Au $4f_{5/2}$ (87.8 eV) was observed, indicating the formation of metallic Au.[36] Similarly, when Au was attached on LTO, the signals of Au 4f shift to higher binding energies (ca. 0.7 eV), suggesting a strong interaction between Au and LTO in the Au-LTO system.[38]

The diffuse reflectance spectra (DRS) of LTO NSP and Au-LTO NSP were recorded in the range of 200−1200 nm, as shown in Figure 3. The bare LTO NSP can absorb only UV light with wavelengths $\lambda < 383$ nm due to the bandgap energy (3.23 eV). After loading Au NRs, the optical absorption thresholds of 780-, 860-, and 940-Au-LTO NSP are all similar to those of bare LTO NSP. However, two additional absorption bands centered at visible and NIR regions appear, as a consequence of the transverse surface plasmon resonance (TSPR) and LSPR photoabsorption of Au NRs, respectively. As we know, the SPR absorption band is sensitive to the surrounding environment of the metal nanostructure.[15] Compared to bare Au NRs colloids (Figure S1a), both TSPR and LSPR absorptions of the corresponding Au-LTO NSP samples are red-shifted due to the large refractive index of the semiconductor substrate.[39] By comparing the spectra of Au-LTO NSP samples, it is found that the aspect ratio of Au NRs

DOI: 10.1021/acscatal.7b02972
ACS Catal. 2018, 8, 122−131

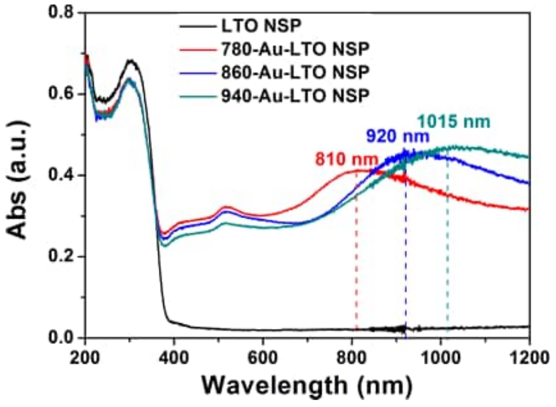

Figure 3. UV−vis−NIR DRS of LTO NSP and Au-LTO NSP samples.

has a great influence on the plasmon absorption of the composites. The maximum LSPR absorption of Au-LTO NSP shifts to low resonance energy with the increase of the aspect ratio of Au NRs. These results show that the light harvesting properties of Au-LTO NSP composites can be flexibly adjusted by changing the aspect ratio of Au NRs. Furthermore, the board absorption peak in the NIR region offers a new opportunity for the utilization of a long-wavelength solar spectrum.

Photocatalytic H_2 Evolution Activities. To understand the plasmonic effect of Au NRs on promoting photocatalysis, the testing of Au-LTO systems in reductive generation of H_2 was performed under vis−NIR light (λ > 420 nm) or NIR light (λ > 780 nm) irradiation, by putting a 420 or 780 nm long-pass filter between the reactor and light source (a Xe lamp with output wavelength, 350−1800 nm, 350 mW cm^{-2}). Under such conditions, the bandgap photoexcitation of LTO can be prevented so that the SPR-mediated local electromagnetic field enhancement and photonic enhancement mechanism cannot work in our systems, and thus the SPR excitation effects of Au NRs in the composites are exclusively studied.[40] The H_2 evolution amount obtained in our experiments corresponded to three times of independent tests using three samples prepared independently, wherein methanol was used as a sacrificial electron donor. Figure 4a shows the H_2 evolved over 780-, 860-, and 940-Au-LTO NSP samples with equivalent Au NRs loadings (3 wt %) under the λ > 420 nm light irradiation. The amount of H_2 generated from the 780-, 860-, and 940-Au-LTO NSP composites increased with time and reached 0.83 ± 0.08, 1.07 ± 0.09, and 0.92 ± 0.04 mmol g^{-1} under vis−NIR light irradiation for 3 h, respectively. Among the tested samples, 860-Au-LTO NSP exhibits the highest photocatalytic activity, which may be induced by the higher light intensity of Xe lamp in the NIR region ranging from 800 to 900 nm (Figure S6). As the irradiated wavelength was longer than 780 nm, similar phenomena were observed (Figure S7), demonstrating that Au NRs are suitable photosensitizers under both visible and NIR light irradiation. As a reference experiment, LTO NSP exhibited no obvious photocatalytic activity because the low energy photons coming from a filtered Xe lamp cannot cause

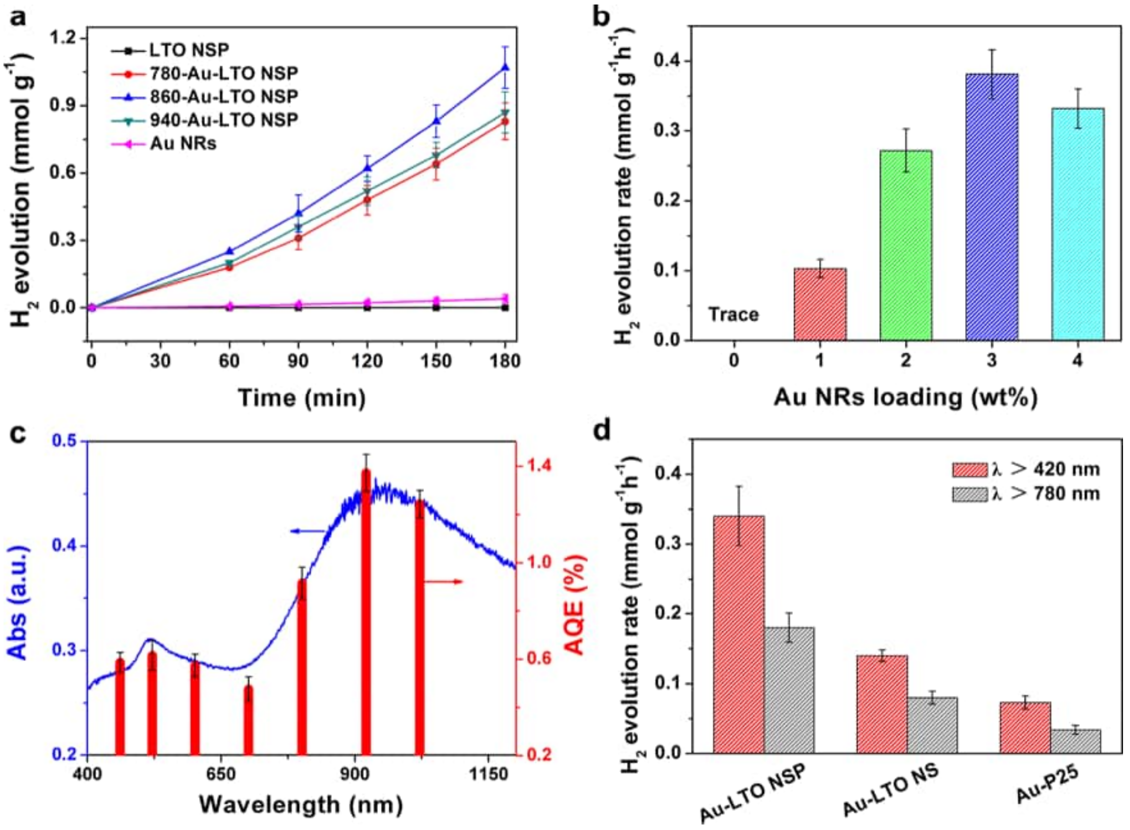

Figure 4. Photocatalytic H_2 evolution over different samples with 3 wt % Au loading (a) and effect of Au loading amounts in Au-LTO NSP on photocatalytic H_2 evolution rate (b) under vis−NIR (λ > 420 nm) light irradiation. DRS (left axis, blue line) and AQE (right axis, red column) of Au-LTO NSP (c). Photocatalytic H_2 evolution rate over different samples with 3 wt % Au loading under vis−NIR (λ > 420 nm) and NIR (λ > 780 nm) light irradiation (d).

DOI: 10.1021/acscatal.7b02972
ACS Catal. 2018, 8, 122−131

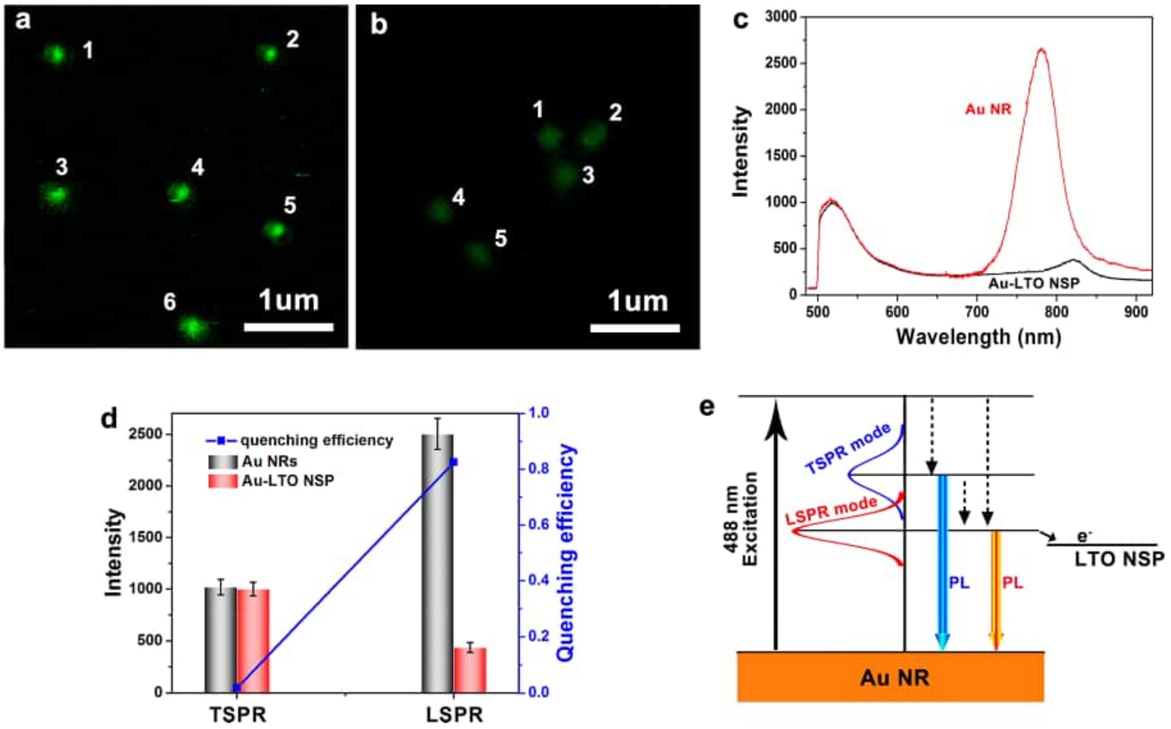

Figure 5. Single-particle PL images of Au NRs before (a) and after (b) loading on LTO NSP. Typical single-particle PL spectra of Au NRs before and after loading on LTO NSP (c). Averaged intensity (left axis, column) and quenching efficiency (right axis, line+symbol) of single-particle PL spectra of Au NRs loaded on LTO NSP (d). SPR radiative decay of hot electron on Au NR loaded on LTO NSP (e).

the bandgap photoexcitation of LTO. Moreover, negligible H_2 generation was observed with Au NRs alone since the energetically hot electrons cannot be effectively extracted and migrated to participate in photocatalytic processes although Au NRs have significant SPR absorption.[23] These results indicate that the interaction between Au and LTO plays a crucial role in the photocatalytic H_2 evolution. Figure 4b summarizes the effects of the loading amount of Au NRs on the photocatalytic activity of 860-Au-LTO NSP nanocomposites under light illumination with wavelengths longer than 420 nm. The photocatalytic H_2 generation performance initially increases and eventually decreases when the loading amount of the Au NRs increases. The optimal amount of Au NRs in the present photocatalytic system is demonstrated to be 3 wt %, which led to a H_2 evolution rate of 0.34 ± 0.03 mmol g^{-1} h^{-1}. The decrease in the photocatalytic activity can be attributed to the increased recombination events of the photoelectrons and holes as a result of the high number density of the Au NRs, since Au particles can dually serve as recombination centers for photoinduced electrons and holes.[41] The photocatalytic stability of Au-LTO NSP was investigated by cyclic photocatalytic H_2 evolution experiments. As shown in Figure S8, continuous H_2 evolution was detected without noticeable degradation in the subsequent runs. No obvious change could be observed in the XRD pattern and SEM and TEM images of Au-LTO NSP after the photocatalytic reaction (Figure S9), indicating that the Au NR decorated LTO NSPs act as stable photocatalysts.

To verify whether the vis−NIR light responsive activity came from the SPR excitation of Au NRs, we measured the H_2 evolved over 860-Au-LTO NSP at a series of monochromatic light (width: ±5 nm; intensity: ~10 mW cm^{-2}). The calculated apparent quantum efficiency (AQE) at each wavelength was averaged by three repeated tests. From Figure 4c, one can see that the AQE action spectrum basically agrees with the SPR spectrum of the 860-Au-LTO NSP composite. Especially, the AEQ of 1.4 ± 0.1% at 920 nm indicates that the Au-LTO NSP composites perform well in the NIR region. The result implied that the observed H_2 evolution was originated from the LSPR rather than TSPR excitation of Au NRs, which will be further confirmed by single-particle PL measurements below.

In order to investigate the impact of the LTO structure on the SPR-mediated system, we prepared two reference samples, Au-LTO NS and Au-P25, containing the same amount of 860-Au NRs as Au-LTO NSP. Their XRD, TEM, and DRS analyses are displayed in Figures S10 and S11, respectively. Sheet-like LTO exposed with (010) surfaces was obtained through hydrothermal processing (300−800 nm lateral size, 10 nm thickness, and 44 m^2 g^{-1} surface area).[34] Due to the unbounded oxygen atoms, the perovskite LTO is readily formed into a two-dimensional (2D) plate and exposes the O-terminated inactive (010) surfaces. Commercial P25 TiO_2 powder was often used as a benchmark in photocatalysis (particle size: 19 nm; surface area: 55 m^2 g^{-1}).[29] Both LTO NS and P25 TiO_2 have no absorption for visible light and possess a much larger surface area than LTO NSP (5.7 m^2 g^{-1}).[34] After loading Au NRs onto LTO NS, two new absorption bands appear at around 520 and 920 nm (Figure S10c), ascribing to the TSPR and LSPR excitation of Au NRs, respectively. The Au-P25 sample shows the SPR absorption peaks at about 520 and 900 nm, respectively (Figure S11c). The optimal Au NRs loading amounts on LTO NS and P25 were determined to be 3 and 1 wt %, respectively (Figure S12). As summarized in Figure 4d, with 3 wt % Au NRs loading, these two composites exhibited lower activity than Au-LTO NSP, in which the H_2 generation rate (0.34 ± 0.03 mmol g^{-1} h^{-1}) was 2.4 and 4.7 times that of Au-LTO NS (0.14 ± 0.008 mmol g^{-1} h^{-1}) and Au−P25 (0.073 ± 0.009 mmol g^{-1} h^{-1}) under λ > 420 nm light irradiation,

DOI: 10.1021/acscatal.7b02972
ACS Catal. 2018, 8, 122−131

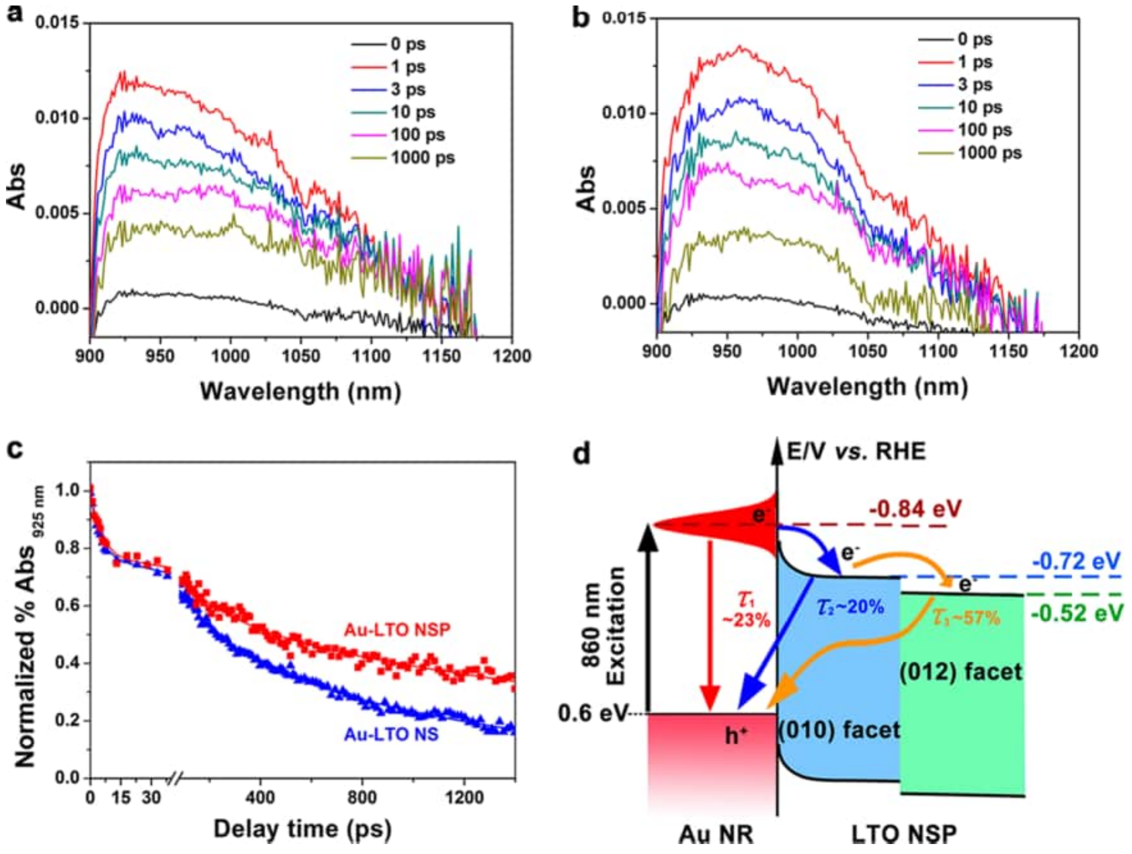

Figure 6. TDR spectra observed after 860 nm laser flash photolysis of Au-LTO NSP (a) and Au-LTO NS (b). Normalized transient absorption traces observed at 925 nm for Au-LTO NSP and Au-LTO NS systems (c). Bold lines indicate multiexponential curves fitted to kinetic traces. Schematic illustration of the three different electron decay processes in the Au-LTO NSP system (d).

respectively. When it comes to NIR light illumination (λ > 780 nm), the photocatalytic H_2 evolution rates of Au-LTO NSP, Au-LTO NS, and Au-P25 were measured to be 0.18 ± 0.02, 0.080 ± 0.009, and 0.031 ± 0.006 mmol g^{-1} h^{-1}, respectively (Figure 4d). The poor activity of the Au-P25 composite may be induced by the insufficient interfacial contact between Au NRs and P25 nanoparticles. The above results indicated that the step-like LTO nanostructure is a superior electron acceptor when SPR Au NR is used as a hot electron donor. Photocurrent and electrochemical impedance spectra (EIS) of the three samples were measured as shown in Figure S13, which further demonstrated the higher charge separation in Au-LTO NSP.

Interfacial Hot Electron Transfer Dynamics. Single-particle PL microscopy was used to clarify the spatial electron transfer from single Au NR to LTO NSP. Using a 488 nm CW laser as the excitation light source, PL images and the corresponding spectra were detected by the electron-multiplying charge-coupled device (EMCCD) camera. It is essential to achieve the monodispersibility of nanorods for the statistical PL evolution of Au NRs. A typical SEM image of monodispersed Au NRs on a glass substrate is shown in Figure S14a, confirming the monodispersibility of the sample. Figure 5a shows single-particle PL images of Au NRs, while single-particle PL spectra of the Au NR particles identified by the number in Figure 5a are shown in Figure S15. A strong PL peak at longer wavelength (~800 nm) which can be assigned to PL from LSPR was observed for each particle. Because of the different aspect ratio of individual Au NR particles, the position and intensity of LSPR PL bands have some differences. The presence of a long pass filter (498 nm) installed to remove the excitation light obstructed the full display of the PL at the TSPR region (~520 nm). When Au NRs were loaded on the surface of LTO NSP, the dispersed sample on a glass substrate is also observed by a SEM image in Figure S14b. Compared with bare Au NRs, the brightness of single-particle PL images of Au NRs loaded on LTO NSP became weak because of the PL quenching by LTO (Figure 5b). In the same manner, single-particle PL spectra of the Au NR particles on LTO NSP indexed by the number in Figure 5b are shown in Figure S16. The PL quenching was clearly observed in the spectra in Figure 5c. After analyzing the PL spectra of 30 individual Au NRs with and without LTO adsorption, we can compare the absolute values of the LSPR and TSPR PL intensities (Figure S17). Upon composing with LTO, the PL intensities at the LSPR region substantially decreased while that at the TSPR region remained nearly unvaried. Moreover, the averaged intensity and quenching efficiency of SPR PL bands of the 30 Au NR particles were calculated (Figure 5d). The quenching efficiencies of PL from TSPR and LSPR for Au-LTO NSP were 2% and 82%, respectively, indicating that the hot electrons transfer from Au NRs to LTO was mainly caused by the LSPR of Au NRs.

Under 488 nm laser light irradiation, electron–hole pairs was generated in Au NRs via the interband transition. The short-wavelength PL peak is caused by a fast interconversion occurring between the electron–hole pairs and TSPR which subsequently decay radiatively.[42] The LSPR PL is induced by the directly excited hot electrons which may lose the energy nonradiatively and interconvert to the LSPR mode, emitting a photon subsequently.[42] In the meantime, promoting by

DOI: 10.1021/acscatal.7b02972
ACS Catal. 2018, 8, 122–131

electron−hole pairs, an energy transfer occurs from the TSPR to LSPR mode (Figure 5e).[19] As reported, the PL lifetime from the TSPR mode is much shorter than that from the LSPR mode,[43] implying that some other decay routes are easier to influence the LSPR PL, while the TSPR PL is invariant and more related to its intrinsic features. In this work, PL damping occurred when Au NRs were loaded on LTO, as a result of hot electrons transfer from Au NRs to LTO, competing with the LSPR emission. Under vis−NIR light irradiation, the hot electrons are generated by TSPR/LSPR excitation and then transfer to LTO, where H^+ reduces to H_2. In the visible light region, the AQE of Au-LTO NSP for H_2 evolution was lower than 1% (see Figure 4c). Because TSPR excitation was the dominant hot electrons generation path, the interband energy transition from TSPR to the LSPR mode could only lead to a low quenching efficiency of 2% for TSPR PL upon composing with LTO. In the NIR light region, the dominant LSPR excitation caused abundant hot electron injection (82%) from Au NRs into LTO, giving rise to a very high AQE for H_2 evolution. The electron-transfer process causes charge separation spatially.[19] The spatial separation of plasmon-induced hot electron−hole pairs in Au NRs decorated LTO NSP results in an efficient charge separation and, hence, an improved NIR light utilization for H_2 production.

In our previous publication, successive surface heterojunctions constituted by two periodically arranged facets in nanostep structured LTO were demonstrated to efficiently enhance space-charge separation.[34] Due to different band structures and band edge positions, the periodically coexposed high-energy (012) facets and low-energy (010) facets of LTO NSP can form successive surface heterojunctions within a single LTO particle, which is beneficial for the transfer of photogenerated electrons and holes to (012) and (010) facets, respectively. In the present work, we can conclude that photogenerated hot electrons from Au NRs could migrate to LTO NSP more easily and efficiently than in LTO NS when Au NRs is irradiated under visible and NIR light. However, the electron transfer behavior from Au NRs to LTO with different geometry (morphology, structure, and exposed crystal surface) is still unclear, which is a significant factor influencing the photocatalytic H_2 production activity. Therefore, femtosecond time-resolved diffuse reflectance (TDR) spectroscopy, a powerful analytical tool in understanding fundamental photophysical processes, was carried out here to help us directly observe the dynamics of electron transfer time-scale and carrier populations after the SPR excitation. The TDR spectra of Au-LTO NSP and Au-LTO NS were recorded at different time delays after exciting Au NRs with 860 nm pump pulse laser. The spectra of all samples revealed a broad absorption band from 900 to 1200 nm (Figure 6a,b), which could be assigned to trapped and free (or shallowly trapped) hot electrons generated from the LSPR mode of Au NRs according to the previous studies.[44,45] The transient absorption of all samples reached highest immediately after the laser flash (1 ps) and then decayed gradually, showing a subpicosecond time scale of the electron injection process from the excited Au NRs to LTO. At 1−1000 ps after the laser flash, the concentration of electrons in the Au-LTO composite reduced in a multiexponential decay owing to the recombination of these electrons with the holes in Au NRs.[29] At 1000 ps, approximately 40% of the electrons survived in the Au-LTO NSP system, while only ∼23% survived in the Au-LTO NS system. Furthermore, the concentration of the trapped electrons (900−1000 nm) reduced faster than that of the free electrons (1000−1200 nm). A similar phenomenon has been observed in the Au/TiO_2 system,[29] which implied a fast charge recombination between the trapped electrons in semiconductors and the holes in Au before diffusing away from the vicinity of the Au particles. To study the decay kinetics of trapped electrons, the time profiles of absorption probed at 925 nm were fitted using a multiexponential function as shown in Figure 6c. The lifetimes (τ_i) and amplitudes (a_i) are presented in Table 1. The transient absorption trace on the Au-LTO NSP was fitted to a three-exponential function with time constants of 4.8 (23%), 227 (20%), and 2800 ps (57%). Three decay lifetimes for Au-LTO NS were also obtained: 4.2 (22%), 250 (29%), and 1100 ps (49%). As illustrated in Figure 6d, the three time scales represent three different electron decay processes in the Au-LTO systems. The shortest component (τ_1) corresponded to the recombination of LSPR modes of Au NRs, namely, the hot electron relaxation process within Au. It has been reported that the apparent lifetime of the hot electron−hole recombination in bare Au NRs at the LSPR band was several picoseconds.[24] Hot electrons transfer from Au to LTO, competitive with SPR modes for recombination, resulted in the quenching of SPR PL.[27] The proportions of the transferred hot electrons (injection efficiency, $1 - a_1$) in Au-LTO NSP (77%) and Au-LTO NS (78%) systems were very similar, indicating that the geometry of the LTO substrate had little effect on the formation of Schottky barriers between Au and LTO. The second component with a lifetime of several hundreds of picoseconds (τ_2) was due to the electrons injected from Au into the neighboring (010) and/or (012) facets of LTO NSP (or the neighboring (010) facets of LTO NS) within a short distance. Lacking effective diffusion, 20% of the trapped electrons in LTO NSP (or 29% of the trapped electrons in LTO NS) rapidly recombined with the holes in Au in 200−300 ps. The relatively long-lived component, τ_3, could be attributed to the recombination between the long-distance diffused electrons in LTO and the holes in Au. The larger the τ_3 is, the lower probability for electron−hole pairs recombination, while in turn the redox reaction can take place more easily. Therefore, the τ_3 of Au-LTO NSP (2800 ps), which was much longer than that of Au-LTO NS (1100 ps), clearly demonstrated the more efficient charge carrier separation process in the Au-LTO NSP system. Note that, comparing with Au-LTO NS, Au-LTO NSP also revealed an increased a_3 value. Because the exposed (010) facets had more negative CBM potential (−0.72 eV vs NHE) than (012) facets (−0.52 eV vs NHE) within a LTO NSP particle,[34] the match of work functions of Au with (010) facets was higher than those of Au with (012) facets. A large proportion of photogenerated electrons preferentially injected from Au into (010) facets, while a small part of electrons injected from Au into (012) facets nearby. However, because of the built-in field established by successive surface heterojunctions, the injected electrons in (010) surfaces readily transferred to adjacent (012) surfaces

Table 1. Lifetimes (τ_i) and Amplitudes (a_i) of the Transient Absorption Decays for Au-LTO Samples

sample	τ_1 (a_1)	τ_2 (a_2)	τ_3 (a_3)
Au-LTO NSP	4.8 ± 0.7 ps (23%)	230 ± 50 ps (20%)	2800 ± 900 ps (57%)
Au-LTO NS	4.2 ± 0.4 ps (22%)	250 ± 30 ps (29%)	1100 ± 400 ps (49%)

DOI: 10.1021/acscatal.7b02972
ACS Catal. 2018, 8, 122−131

which were far away from Au NRs. The formed anisotropic electron flows from (010) to (012) surfaces thus increased the population of long-lived electrons and retarded the charge recombination process. As a result, the photocatalytic activity of the Au-LTO NSP system in the NIR region could be intensively improved.

CONCLUSIONS

In this work, Au NRs photosensitized LTO NSP hybrid structure was constructed and used as an efficient visible and NIR photocatalyst for H_2 evolution. The optimal H_2 evolution rate of Au-LTO NSP was 0.34 mmol g^{-1} h^{-1} under vis−NIR (λ > 420 nm) light irradiation, which was 2.4 and 4.7 times that of Au-LTO NS and Au-P25, respectively. The high AEQ of 1.4% at 920 nm irradiation indicated that the Au-LTO NSP composites could utilize NIR light efficiently. Single-particle fluorescence measurements showed that only the LSPR mode of Au NR realizes hot electron transfer to LTO NSP. Charge carrier lifetimes of Au-LTO NSP and Au-LTO NS measured by femtosecond TDR spectroscopy indicated that the redox reaction could take place more easily and the population of long-lived electrons was also greatly increased in the Au-LTO NSP system than in the Au-LTO NS system. Compared to LTO NS with only (010) facets exposure, a great part of injected hot electrons from Au NRs into the (010) facets of LTO NSP could efficiently migrate to the adjacent (012) facets and then be temporally stored there for further reactions, driven by successive surface heterojunctions. This is the first example to show the powerful role of a semiconductor surface heterojunction in favoring the plasmon-induced interfacial hot electron transfer.

EXPERIMENTAL SECTION

Preparation of $La_2Ti_2O_7$ Nanostructures. LTO nanosteps and nanosheets were respectively synthesized by the molten-salt method and the hydrothermal method, following our previous publications.[34,37]

Preparation of Au Nanorods. A seed-mediated method was employed to synthesize Au NRs.[46] First, a freshly prepared, ice-cold $NaBH_4$ (Fluka, 0.6 mL, 0.01 M) aqueous solution was instantly added into the CTAB (TCI, 5 mL, 0.2 M) and $HAuCl_4{\cdot}3H_2O$ (Sigma-Aldrich, 5 mL, 0.5 mM) mixture solution. A seed solution was obtained by vigorously stirring the above solution for 2 min and then aging at 30 °C for 2 h. Second, 3.6 g of CTAB and 0.493 g of sodium oleate (Tokyo Chemical Industry Co.) were dissolved in 100 mL of warm water (Millipore, 50 °C). Then 4 mL of a 4 mM $AgNO_3$ solution (Sigma-Aldrich) was added when the solution temperature was decreased to 30 °C. After gentle stirring (100 rpm) for 15 min, 100 mL of a $HAuCl_4{\cdot}3H_2O$ solution (1 mM) was added. Upon 90 min of stirring at 700 rpm, 0.6 mL of HCl (37 wt % in water, Wako) was then introduced, followed by another 15 min of slow stirring (400 rpm). By adding 0.32 mL of 0.064 M ascorbic acid (Sigma-Aldrich) and vigorous stirring for 30 s, the growth solution was prepared. Finally, 0.04 mL of seed solution was injected to the entire growth solution. After stirring for 30 s, the mixture was left undisturbed at 30 °C for 12 h. The final product of 860-Au NRs was isolated by centrifugation at 7000 rpm for 30 min to remove the supernatant.

Preparation of Au-LTO Composites. A ligand-exchange method was used to deposit Au NRs on LTO nanostructures to obtain the Au-LTO hybrid structures.[35] A total of 100 mg of LTO was dispersed in 100 mL of aqueous solution containing 100 μL of $NH_3{\cdot}H_2O$ (28%, Nacalai Tesque) and 100 μL of 3-mercaptopropionic acid (MPA, Sigma-Aldrich) for 12 h. Then, a calculated volume of Au NRs solution (concentration determined by ICP measurements) was introduced. After sufficient stirring for 12 h, the mixture was centrifuged, washed with water and ethanol, dried at 60 °C for 12 h, and annealed at 200 °C for 2 h to obtain the final product.

Characterization of Materials. XRD (Rigaku, Smartlab; operated at 40 kV and 200 mA, Cu Kα source) was employed to examine the structures of the samples. SEM (JEOL, JSM-6330FT) and TEM (JEOL, JEM3000F operated at 300 kV or Hitachi, H-800 operated at 200 kV) equipped with an EDS analyzer were used to investigate the morphologies, microstructures, and compositions. The XPS measurements were carried out on a PHI X-tool 8ULVAC-PHI instrument. The steady-state absorption and diffuse reflectance spectra were conducted on a UV−vis−NIR spectrophotometer (Jasco, V-770) at room temperature.

Photocatalytic H_2 Evolution. A total of 10 mg of photocatalyst was dispersed in 10 mL of aqueous solution containing 20 vol % methanol (Nacalai Tesque) in a 30 mL glass tube. Prior to the irradiation, the tube was sealed with a rubber septum and then bubbled with Ar gas for 20 min to completely remove the oxygen. The sample was irradiated using a Xe lamp (Asahi Spectra, HAL-320; 350 mW cm^{-2}) with constant stirring at room temperature. The volume of the generated H_2 was measured using a gas chromatograph (Shimadzu GC-8A). The AQE was calculated as AQE = (2 × number of evolved H_2 molecules/number of incident photons) × 100%.

Photoelectrochemical Measurements. Photoelectrochemical (PEC) measurements were conducted in three-electrode quartz cells connected with an electrochemical analyzer (ALS, model 660A). A platinum wire and Ag/AgCl electrode were used as the counter electrode and reference electrode, respectively. Au-LTO NSP, Au-LTO NS, and Au-P25 film electrode on an ITO substrate (BAS Inc., ITO11) served as the working electrode. A 0.1 M Na_2SO_4 aqueous solution was used as the electrolyte. The photocurrent and EIS plots were obtained under a bias of 1.23 V vs reversible hydrogen electrode (RHE).

Single-Particle Photoluminescence Measurements. For the single-particle PL measurement, we use an objective scanning confocal fluorescence microscope (PicoQuant, Micro-Time 200) to record the images and spectra. It was coupled with an oil-immersion objective lens (Olympus, UplanSApochromat, 100×, 1.4 NA). The circular-polarized 488 nm pulse wave laser source was controlled using a PDL-800B driver (PicoQuant). To excite Au NRs, the excitation power was 300 μW. A single-photon avalanche photodiode (Micro Photon Devices, PDM 50CT) was tuned using the dichroic beam splitter (Chroma, 405/488rdc) together with a long pass filter (Chroma, HQ513CP). The emission from the sample was collected and detected using the above objective and the photodiode, respectively. The imaging spectrograph (Acton Research, SP-2356) only receives the emission passing through a slit that entered on it. The spectrograph was equipped with EMCCD camera (Princeton Instruments, ProEM). Every spectrum was integrated for 20 s.

Time-Resolved Diffuse Reflectance Spectral Measurements. The femtosecond TDR spectra were measured by the

DOI: 10.1021/acscatal.7b02972
ACS Catal. 2018, 8, 122−131

pump and probe method. The source is a regeneratively amplified titanium sapphire laser (Spectra-Physics, Spitfire Pro F, 1 kHz), which is pumped using the Nd:YLF laser (Spectra-Physics, Empower 15). A titanium sapphire laser (Spectra-Physics, Mai Tai VFSJW; fwhm 80 fs) was used to generate the seed pulse. The excitation pulse (320 or 420 nm, 3 mJ pulse^{-1}) was from the optical parametric amplifier (Spectra-Physics, OPA-800CF-1). A white light continuum pulse was divided into two parts to serve as probe and reference lights. Laser fluctuation was compensated using the reference light. The sample powder was coated on a glass sheet. Both the probe and the reference lights were directed to the samples. A linear InGaAs array detector which was equipped with a polychromator (Solar, MS3504) was used to detect the reflected lights. The mechanical chopper which was synchronized to the laser repetition rate was used to chop the pump pulse. A pair of spectra were achieved with and without the pump. The absorption change (% absorption) induced by the pump pulse was estimated based on these two spectra.

■ ASSOCIATED CONTENT

Supporting Information

The Supporting Information is available free of charge on the ACS Publications website at DOI: 10.1021/acscatal.7b02972.

> vis−NIR absorption spectra and TEM images, Figure S1; structural parameters, Table S1; STEM-EDS images, Figure S2; EDS, Figure S3; XRD patterns, Figure S4; XPS, Figure S5; irradiance spectrum, Figure S6; photocatalytic H_2 evolution, Figures S7, S8, and S12; XRD patterns, SEM image, TEM image, and UV−vis−NIR DRS, Figures S9−S11; photocurrents and EIS plots, Figure S13; SEM images, Figure S14; single-particle PL spectra, Figures S15 and S16; statistical graph of PL intensity, Figure S17. (PDF)

■ AUTHOR INFORMATION

Corresponding Authors

*E-mail: zjy@buaa.edu.cn.

*E-mail: majima@sanken.osaka-u.ac.jp.

ORCID

Mingshan Zhu: 0000-0002-5926-5383

Mamoru Fujitsuka: 0000-0002-2336-4355

Junying Zhang: 0000-0002-4860-8774

Tetsuro Majima: 0000-0003-1805-1677

Notes

The authors declare no competing financial interest.

■ ACKNOWLEDGMENTS

This project is financially supported by National Natural Science Foundation of China (Nos. 51472013 and 51672106) and the Excellence Foundation of BUAA for PhD Students (No. 2017061). We thank the help from the Comprehensive Analysis Center of SANKEN, Osaka University. This work is partly supported by a Grant-in-Aid for Scientific Research (Project 25220806 and others) from the Ministry of Education, Culture, Sports, Science and Technology (MEXT) of the Japanese Government.

■ REFERENCES

(1) Chen, X.; Shen, S.; Guo, L.; Mao, S. S. *Chem. Rev.* **2010**, *110*, 6503−6570.

(2) Fujishima, A.; Honda, K. *Nature* **1972**, *238*, 37−38.

(3) Hisatomi, T.; Kubota, J.; Domen, K. *Chem. Soc. Rev.* **2014**, *43*, 7520−7535.

(4) Ismail, A. A.; Bahnemann, D. W. *Sol. Energy Mater. Sol. Cells* **2014**, *128*, 85−101.

(5) Wang, X.; Wang, F.; Sang, Y.; Liu, H. *Adv. Energy Mater.* **2017**, 1700473.

(6) Kou, J.; Lu, C.; Wang, J.; Chen, Y.; Xu, Z.; Varma, R. S. *Chem. Rev.* **2017**, *117*, 1445−1514.

(7) Li, Y.; Wang, Z.; Xia, T.; Ju, H.; Zhang, K.; Long, R.; Xu, Q.; Wang, C.; Song, L.; Zhu, J.; Jiang, J.; Xiong, Y. *Adv. Mater.* **2016**, *28*, 6959−6965.

(8) Sang, Y.; Zhao, Z.; Zhao, M.; Hao, P.; Leng, Y.; Liu, H. *Adv. Mater.* **2015**, *27*, 363−369.

(9) Chen, X.; Liu, L.; Yu, P. Y.; Mao, S. S. *Science* **2011**, *331*, 746−750.

(10) Hu, Y. H. *Angew. Chem., Int. Ed.* **2012**, *51*, 12410−12412.

(11) Chen, X.; Liu, L.; Huang, F. *Chem. Soc. Rev.* **2015**, *44*, 1861−1885.

(12) Li, Z. X.; Shi, F. B.; Zhang, T.; Wu, H. S.; Sun, L. D.; Yan, C. H. *Chem. Commun.* **2011**, *47*, 8109−8111.

(13) Guo, X.; Song, W.; Chen, C.; Di, W.; Qin, W. *Phys. Chem. Chem. Phys.* **2013**, *15*, 14681−14688.

(14) Tang, Y.; Di, W.; Zhai, X.; Yang, R.; Qin, W. *ACS Catal.* **2013**, *3*, 405−412.

(15) Meng, X.; Liu, L.; Ouyang, S.; Xu, H.; Wang, D.; Zhao, N.; Ye, J. *Adv. Mater.* **2016**, *28*, 6781−6803.

(16) Cushing, S. K.; Wu, N. *J. Phys. Chem. Lett.* **2016**, *7*, 666−675.

(17) Linic, S.; Christopher, P.; Ingram, D. B. *Nat. Mater.* **2011**, *10*, 911−921.

(18) Liu, L.; Ouyang, S.; Ye, J. *Angew. Chem., Int. Ed.* **2013**, *52*, 6689−6693.

(19) Zheng, Z.; Tachikawa, T.; Majima, T. *J. Am. Chem. Soc.* **2014**, *136*, 6870−6873.

(20) Elbanna, O.; Kim, S.; Fujitsuka, M.; Majima, T. *Nano Energy* **2017**, *35*, 1−8.

(21) Ng, C.; Cadusch, J. J.; Dligatch, S.; Roberts, A.; Davis, T. J.; Mulvaney, P.; Gomez, D. E. *ACS Nano* **2016**, *10*, 4704−4711.

(22) Su, R.; Tiruvalam, R.; Logsdail, A. J.; He, Q.; Downing, C. A.; Jensen, M. T.; Dimitratos, N.; Kesavan, L.; Wells, P. P.; Bechstein, R.; et al. *ACS Nano* **2014**, *8*, 3490−3497.

(23) Han, C.; Quan, Q.; Chen, H. M.; Sun, Y.; Xu, Y. J. *Small* **2017**, *13*, 1602947.

(24) Zhang, P.; Fujitsuka, M.; Majima, T. *Nanoscale* **2017**, *9*, 1520−1526.

(25) Pu, Y. C.; Wang, G.; Chang, K. D.; Ling, Y.; Lin, Y. K.; Fitzmorris, B. C.; Liu, C. M.; Lu, X.; Tong, Y.; Zhang, J. Z.; Hsu, Y. J.; Li, Y. *Nano Lett.* **2013**, *13*, 3817−3823.

(26) Jiang, W.; Bai, S.; Wang, L.; Wang, X.; Yang, L.; Li, Y.; Liu, D.; Wang, X.; Li, Z.; Jiang, J.; Xiong, Y. *Small* **2016**, *12*, 1640−1648.

(27) Lou, Z.; Fujitsuka, M.; Majima, T. *J. Phys. Chem. Lett.* **2017**, *8*, 844−849.

(28) Gołąbiewska, A.; Malankowska, A.; Jarek, M.; Lisowski, W.; Nowaczyk, G.; Jurga, S.; Zaleska-Medynska, A. *Appl. Catal., B* **2016**, *196*, 27−40.

(29) Bian, Z.; Tachikawa, T.; Zhang, P.; Fujitsuka, M.; Majima, T. *J. Am. Chem. Soc.* **2014**, *136*, 458−465.

(30) Liu, X.; Iocozzia, J.; Wang, Y.; Cui, X.; Chen, Y.; Zhao, S.; Li, Z.; Lin, Z. *Energy Environ. Sci.* **2017**, *10*, 402−434.

(31) Wu, B.; Liu, D.; Mubeen, S.; Chuong, T. T.; Moskovits, M.; Stucky, G. D. *J. Am. Chem. Soc.* **2016**, *138*, 1114−1117.

(32) Hong, J. W.; Wi, D. H.; Lee, S. U.; Han, S. W. *J. Am. Chem. Soc.* **2016**, *138*, 15766−15773.

(33) Wang, L.; Ge, J.; Wang, A.; Deng, M.; Wang, X.; Bai, S.; Li, R.; Jiang, J.; Zhang, Q.; Luo, Y.; Xiong, Y. *Angew. Chem.* **2014**, *126*, 5207−5211.

(34) Cai, X.; Mao, L.; Zhang, J.; Zhu, M.; Fujitsuka, M.; Majima, T. *J. Mater. Chem. A* **2017**, *5*, 10442−10449.

(35) Ding, D.; Liu, K.; He, S.; Gao, C.; Yin, Y. *Nano Lett.* **2014**, *14*, 6731−6736.

(36) Chastain, J.; King, R. C.; Moulder, J. *Handbook of X-ray photoelectron spectroscopy: a reference book of standard spectra for identification and interpretation of XPS data*; Physical Electronics Division, Perkin-Elmer Corporation: Eden Prairie, MN, 1992.

(37) Cai, X.; Zhang, J.; Fujitsuka, M.; Majima, T. *Appl. Catal., B* **2017**, *202*, 191−198.

(38) Zhu, M.; Cai, X.; Fujitsuka, M.; Zhang, J.; Majima, T. *Angew. Chem., Int. Ed.* **2017**, *56*, 2064−2068.

(39) Ma, L.; Liang, S.; Liu, X.-L.; Yang, D.-J.; Zhou, L.; Wang, Q.-Q. *Adv. Funct. Mater.* **2015**, *25*, 898−904.

(40) Primo, A.; Corma, A.; García, H. *Phys. Chem. Chem. Phys.* **2011**, *13*, 886−910.

(41) Gomes Silva, C.; Juárez, R.; Marino, T.; Molinari, R.; García, H. *J. Am. Chem. Soc.* **2011**, *133*, 595−602.

(42) Zheng, Z.; Tachikawa, T.; Majima, T. *Chem. Commun.* **2015**, *51*, 14373−14376.

(43) Wackenhut, F.; Failla, A. V.; Meixner, A. J. *J. Phys. Chem. C* **2013**, *117*, 17870−17877.

(44) Bian, Z.; Tachikawa, T.; Kim, W.; Choi, W.; Majima, T. *J. Phys. Chem. C* **2012**, *116*, 25444−25453.

(45) Yoshihara, T.; Katoh, R.; Furube, A.; Tamaki, Y.; Murai, M.; Hara, K.; Murata, S.; Arakawa, H.; Tachiya, M. *J. Phys. Chem. B* **2004**, *108*, 3817−3823.

(46) Ye, X.; Zheng, C.; Chen, J.; Gao, Y.; Murray, C. B. *Nano Lett.* **2013**, *13*, 765−771.

DOI: 10.1021/acscatal.7b02972
ACS Catal. 2018, 8, 122−131

This is an open access article published under an ACS AuthorChoice License, which permits copying and redistribution of the article or any adaptations for non-commercial purposes.

ACS OMEGA

http://pubs.acs.org/journal/acsodf

Article

Preparation of Y-Doped $La_2Ti_2O_7$ Flexible Self-Supporting Films and Their Application in High-Performance Flexible All-Solid-State Supercapacitor Devices

Yi Cao, Peiyuan Tang, Wenfeng Qiu,* and Tong Zhao*

Cite This: *ACS Omega* 2020, 5, 29722−29732

Read Online

ACCESS | Metrics & More | Article Recommendations | Supporting Information

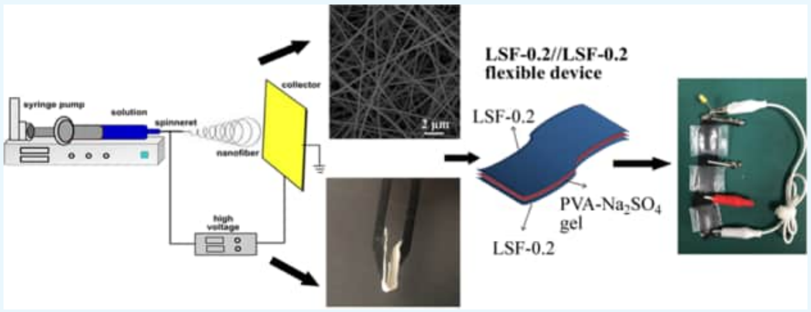

ABSTRACT: Flexible all-solid-state supercapacitors have drawn more attention owing to the rapid growth of wearable electronic equipments. Herein, we have succeeded in synthesizing a series of Y-doped lanthanum titanate flexible self-supporting films (LSF-x, $0.1 \leq x \leq 0.5$) and investigating the change of microstructures, morphological characteristics, and lattice structures of these films affected by different Y-doping contents. To further determine the optimum Y-doping content, we have explored the electrochemical properties of working electrodes prepared by LSF-x ($0.1 \leq x \leq 0.5$) samples as the main active material. As the LSF-0.2 electrode has the best areal capacitance of 1.3 F·cm^{-2} at 2 mA·cm^{-2}, we use the LSF-0.2 electrodes and PVA−Na_2SO_4 gel to fabricate a flexible all-solid-state supercapacitor device. This device has a high areal capacitance of 255.9 mF·cm^{-2} at a current density of 2 mA·cm^{-2} with a high cell voltage of 2.1 V, while the corresponding energy density is 156.8 μWh·cm^{-2} with a power density of 2.1 mW·cm^{-2}. Moreover, it also shows a long cycling life and outstanding flexibility. Therefore, the LSF-0.2 sample can be used as an excellent energy-storage material for a wearable electronic device.

1. INTRODUCTION

Over the last few decades, as the smart bracelet, foldable mobile phone, flexible electronic display, and other wearable electronic equipment have been broadly applied in all areas of our daily lives, the development of flexible electronic device has drawn much attention of scientific and technological researchers.[1−3] To meet the growing demand of lightweight design, portability, and low manufacturing cost for flexible electronic equipment, high-performance flexible power sources have become one of the research focuses.[4−6] Among them, the flexible supercapacitors have been considered as the appropriate device because of their higher power density and longer cycling performance than those of lithium-ion batteries.[7−9] Although the flexible supercapacitors exhibit superior advantages such as small size, high flexibility, and lightweight, these devices still have some challenges like low energy density and unsatisfactory reliability.[10−12] According to the equation $E = 1/2CV^2$, the energy density can be improved by enlarging the capacitance and operating voltage of the supercapacitor. Hence, we are able to synthesize a flexible supercapacitor electrode material with ultrahigh energy-storage ability to enhance the capacitance and potential windows first, and then we can improve the security of the device through fabricating the all-solid-state supercapacitor.[13−15]

To satisfy the needs of an all-solid-state supercapacitor, the development of novel flexible electrode materials for them has become the current intense pursuit. In recent years, it has been reported that some electrode materials such as carbon materials, transition-metal oxides (TMO)/compound oxides, and electrically conductive polymers have been widely used for flexible all-solid-state supercapacitors.[16−18] In particular, compared with carbon and electrically conductive polymers materials, TMO/compound oxides have attracted tremendous interests owing to their higher capacitance, better energy density, and environmental friendliness. Although a wide

Received: July 15, 2020
Accepted: November 2, 2020
Published: November 12, 2020

Downloaded via NANJING TECH UNIV on February 1, 2025 at 06:54:40 (UTC).
See https://pubs.acs.org/sharingguidelines for options on how to legitimately share published articles.

variety of TMO/compound oxides have been applied broadly for all-solid-state supercapacitors, these composite flexible electrodes often limit the utilization of the active electrode materials, for example, Wang et al. have prepared a typical flexible all-solid-state supercapacitor (FSCs) based on mesoporous Bi_2MoO_6 quasi-nanospheres (MBiN) on an activated carbon cloth (ACC) hybrid electrode for improving the energy-storage density and cyclic stability, and the MBiN/ACC flexible electrodes displays an ultrahigh mass capacitance;[19] Lei's research group has reported the successful growth of α-Fe_2O_3 nanosheets on the CS electrode position followed by low-temperature thermal annealing, and α-Fe_2O_3 on CS displays high specificity that is 1.7-times higher than that of the CS substrate;[20] Li et al. have synthesized $NiCo_2O_4$/C core−shell nanoneedles grown on a Ni foam by a simple route and applied in supercapacitors, and the electrode exhibits excellent rate capability and remarkable cycling stability;[21] Haiqun Chen's research group has prepared a composite consisting of $Mn_{0.1}Ni_{0.9}MoO_4$ mesoporous nanorods and reduced graphene oxide ($Mn_{0.1}Ni_{0.9}MoO_4$/rGO), which is assembled into a symmetrical all-solid-state device as electrode material, with alkaline poly(vinyl alcohol) as a solid-state electrolyte.[22] These research studies show that because of the rigid nature of oxides, the TMO/compound oxides that can reduce the obtainable energy and power density have to be decorated on conductive soft carriers for the application in flexible supercapacitors.[23] As a result, synthesizing a new self-supporting oxide film with excellent flexibility, large capacity, and outstanding energy density would be an effective solution to meet the demands of the development of flexible all-solid-state supercapacitors.

In the past decades, various kinds of oxides have been widely used for the supercapacitors as active electrode materials.[24−28] Among them, the pyrochlore compounds show a variety of interesting physical and chemical properties such as high electrical conductivity, good elemental flexibility, and oxygen-vacancy structure.[29−32] Because the previous study indicates that oxygen vacancies have been regarded as the charge-storage sites of compound oxides, $A_2B_2O_7$-type lanthanum titanate (LTO) can be used as outstanding electrochemical energy-storage material.[33] To apply LTO as the main active material to flexible all-solid-state supercapacitors, we can prepare a flexible self-supporting film of LTO, which can also significantly increase the electrochemistry performance and the mechanical property of the flexible device. Moreover, according to the mechanism of oxygen intercalation, the number of oxygen vacancies of LTO can be obviously improved by doping a tertiary component into A or B site of LTO.[34,35] As the increase of oxygen vacancies may enhance the capacitance of LTO, we can modify the energy- storage ability of LTO flexible self-supporting films by doping the transition-metal element Y.[36−38] In addition, the electrospinning combined with the low-temperature sintering technology is practiced to synthesize these Y-modified self-supporting materials.

In this research, we report the synthesis of a series of Y-doped LTO flexible self-supporting films with rich-oxygen-vacancy structure by a sol−gel-assisted electrospinning method followed with low-temperature calcination. The $La_2Y_xTi_{2-x}O_3$ flexible self-supporting films (when x = 0.1, 0.3, 0.3, 0.4, 0.5) were denoted as LSF-0.1, LSF-0.2, LSF-0.3, LSF-0.4, and LSF-0.5, respectively. Besides, the change of the microstructure, morphological characteristics, element composition, and electrochemical property of LSF-x influenced by Y doping was further explored using XRD, SEM, TEM, BET, XPS, and an electrochemical workstation. Because the LSF-0.2 sample showed the highest areal capacitance (1307 mF·cm^{-2} at 2 mA·cm^{-2}) compared with the others, we fabricated a symmetrical LSF-0.2//LSF-0.2 flexible all-solid-state supercapacitor device using a soft-matter substrate and an LSF-0.2 flexible self-supporting film. In addition, this device exhibited high areal capacitance (255.9 mF·cm^{-2} at 2 mA·cm^{-2}), large energy density (156.8 μWh·cm^{-2} at 2.1 mW·cm^{-2}), long cycling stability, and excellent flexibility.

2. EXPERIMENTAL SECTION

2.1. Preparation of the Precursor Films. In this study, we prepared the precursor solution using poly-(vinylpyrolidone) (PVP, M_w = 1 300 000), $La(NO_3)_3$·$6H_2O$ (≥99.0%), titanium tetraisopropanolate (TTIP), and $Y(NO_3)_3$ (≥99.5%), and all of these materials were purchased from Aladdin, China. First, $La(NO_3)_3$·$6H_2O$ (≥99.0%), $Y(NO_3)_3$, and TTIP were weighed based on the molar ratio of 2:x:2−x, respectively, and these drugs were dissolved into a mixture of absolute ethanol, deionized water, and acetic acid (weight ratio, 5:4:1) in a weight ratio of 1:1. We marked this mixture solution as solution A. In addition, we dissolved 15 wt % PVP in a mix solution of deionized water and absolute ethanol in a weight ratio of 1:1, and stirred for 4 h to form a homogeneous solution at room temperature. This mix solution was marked as solution B. To form the precursor solution for electrospinning, we added the solution A into solution B (weight ratio of 1:2) and continuously stirred for about 2 h until clear. After that, as shown in Figure 1, we applied this precursor solution to prepare the precursor films by the electrospinning method. All of the processing parameters of the electrospinning process were determined as follows: the flow rate was 0.3 mL·h^{-1}, the positive potential was 15 kV (HD90, high voltage power supply, China), and the distance between the needle tip and the collector was 15 cm.

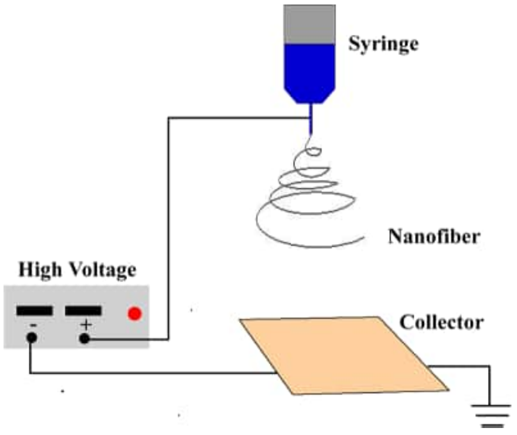

Figure 1. Schematic illustration of the fabrication processes of LSF-x precursors using the electrospinning method.

2.2. Preparation and Characterization of LTO Films. To synthesize the Y-doped LTO flexible self-supporting films, we calcinated these precursor films in a muffle furnace at 750 °C for 2 h under an air atmosphere without a conducting foil. In this calcination process, the heating rate of all of the samples was 3 °C/min. To determine the optimum Y-doping ratio, we

https://dx.doi.org/10.1021/acsomega.0c03402
ACS Omega 2020, 5, 29722−29732

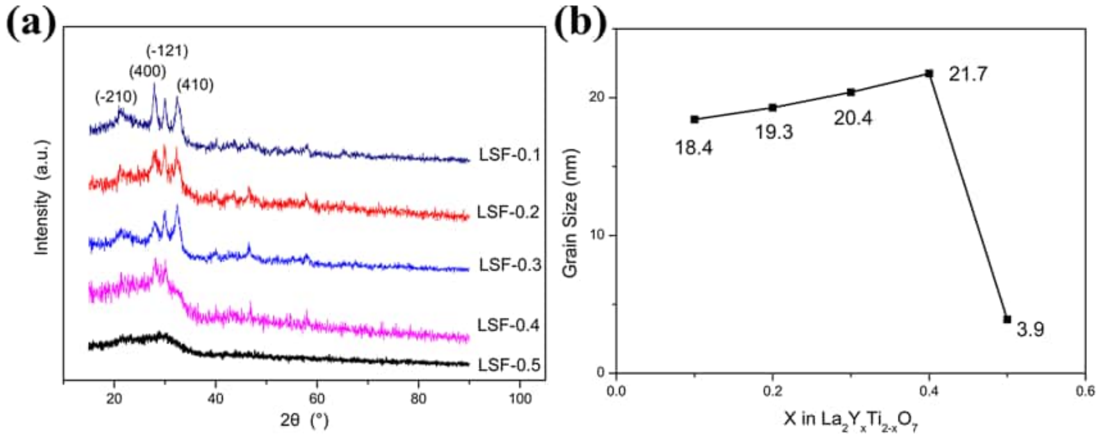

Figure 2. (a) XRD spectra of LSF-0.1, LSF-0.2, LNF-0.3, LSF-0.4, and LSF-0.5 samples; (b) comparison of the grain sizes of LSF-0.1, LSF-0.2, LNF-0.3, LSF-0.4, and LSF-0.5 samples.

carefully studied the change of morphologies and the structure of LSF-x ($0.1 \leq x \leq 0.5$) films with different proportions of Y doping using transmission electron microscopy (TEM, FEI Tecnai G20), scanning electron microscopy (SEM, Cam Scan2600 FE), X-ray photoelectron spectroscopy (XPS, PHI-5000 Versaprobe), and the powder X-ray diffraction (XRD, Rigaku-D/max, Cu Kα), respectively.

2.3. Electrochemical Measurements. The working electrodes were prepared by attaching a series of LSF-x ($0.1 \leq x \leq 0.5$) films on the carbon fabric. Poly(vinylidene fluoride) (PVDF), which was dissolved in *n*-methyl pyrrolidone (NMP) at 5 wt %, served as a binder for electrodes in this process. In addition, we studied the electrochemical measurements of these electrodes using a CHI760E (Shanghai Chenhua, China) workstation with a three-electrode system in a 1 M Na_2SO_4 electrolyte solution. In this test system, the platinum wire was the counter electrode and the saturated calomel electrode (SCE) was the reference electrode. Moreover, the flexible all-solid-state supercapacitor device was assembled by two symmetrical electrodes. Then, the PVA–Na_2SO_4 gel was used as a solid electrolyte in this device, and the weight ratio between PVA and Na_2SO_4 was 5:7. The flexible all-solid-state supercapacitor device was tested by a two-electrode system.

We calculated the areal capacitance (C, F·cm^{-2}) of the working electrode using the following equation based on the GCD curves

$$C = \int I_{(t)} dt/(a\Delta V) \tag{1}$$

In this equation, $I_{(t)}$ is the impressed current, a (cm^2) is the area of all working electrodes, dt is the cycle time, and ΔV is the voltage range of each scan.

The energy density (E) and power density (P) of the device can be calculated using the following two equations

$$E = \frac{1}{2}CV^2 \tag{2}$$

$$P = \frac{3600 \times E}{t} \tag{3}$$

As mentioned above, C is the areal specific capacitance of the device (F·cm^{-2}), V is the voltage (V), and t is the discharge time (s).

3. RESULTS AND DISCUSSION

3.1. Investigation of the Effect of Y Doping. To further confirm the best content of Y in LSF-x ($0.1 \leq x \leq 0.5$), we have thoroughly investigated the effects of Y doping with different proportions on the microstructure, morphological characteristics, and element composition of these LSF-x samples. In this study, these tests have been carried out using XRD, SEM, TEM, and XPS, and the results are shown as follows.

3.1.1. Crystal Structure. The crystal structures of LSF-x ($0.1 \leq x \leq 0.5$) samples with different contents of Y element are characterized by X-ray diffraction (XRD) patterns in a 2θ range between 15 and 90°, as illustrated in Figure 2a. From these patterns, we can observe four clearly main characteristic peaks of LSF-0.1, LSF-0.2, and LNF-0.3 samples, which correspond to the orientations of (−210), (400), (−212), and (410) planes, respectively. These peaks are in agreement with the standard card (JCPDS No. 28-0517) of a pyrochlore-type structure $La_2Ti_2O_7$ with a cubic lattice system. Besides, this image also shows that intensity of these characteristic peaks is decreased with the increase of Y doping. When $x = 0.4$, although these characteristic peaks still remain, their intensities become weaker and the boundary between them is not clear. While the content of x is increased to 0.5, the LSF-0.5 presents no clear characteristic peaks, which can be explained by the changes from a crystalline to amorphous structure of the sample. These results indicate that the crystallinity of the LSF-x ($0.1 \leq x \leq 0.5$) samples is inversely proportional to the Y-doping proportion, and the trend of crystallization reduces with the increasing Y-doping content until the formation of an amorphous structure. The great changes of the crystal system and the lattice distortion in this doping progress can be attributed to the Jahn–Teller effect. In general, the cationic radius between A sites and B sites in LTO becomes mismatched when Y replaces Ti at B sites, and this mismatch may cause the dramatic distortion in the crystal structure. What is more, excess of Y doping may change crystallization conditions of pyrochlore-type $La_2Y_xTi_{2-x}O_3$ compounds and influence the precipitation of the main crystal phase. Due to the positive relationship of the number of oxygen vacancies with the crystallinity, the low crystallization degree of the $La_2Y_xTi_{2-x}O_3$ sample may reduce its energy-storage ability. Hence, we cannot dope too much Y element into $A_2B_2O_7$-type lanthanum titanate (LTO).

https://dx.doi.org/10.1021/acsomega.0c03402
ACS Omega 2020, 5, 29722−29732

Moreover, we calculate the grain sizes of LSF-x ($0.1 \leq x \leq 0.5$) samples by Scherrer's formula to explore the connection between grain growth and the Y-doping ratio. As shown in Figure 2b, the grain size is proportional to the content of the Y element when $0.1 \leq x \leq 0.4$. Due to the even distribution of Y element in the precursor in the form of ions, the crystal lattice may grow with increasing Y doping, which can lead to the increase of grain size. However, the smaller grain size of the LSF-0.5 sample than the others suggests that nanofibers just begin to form crystal nuclei. This result agrees with the previous analysis.

3.1.2. Surface Morphology. The morphologies of the LSF-x ($0.1 \leq x \leq 0.5$) samples are further investigated by SEM measurement and are exhibited in Figure 3a−e. These images show that LSF-x ($0.1 \leq x \leq 0.5$) samples can still maintain good flexibility and fiber structure after being sintered at 750 °C, and these samples with different Y proportions all have rough surfaces and an average diameter of 200−300 nm. As shown in Figure 3a,b, the nanofibers of LSF-0.1 and LSF-0.2 samples presenting a scaly rough surface consist of closely connected particles and the size distribution of these particles is uniform. From Figure 3c,d, the nanofibers of LSF-0.3 and LSF-0.4 samples show a granular and scabrous surface. Compared with the LSF-0.1 and LSF-0.2 samples, we can observe that the surfaces of LSF-0.3 and LSF-0.4 samples tend to be much rougher with increased Y doping and the sizes of particles also become inconformity. However, because of the formation of the amorphous structure, the LSF-0.5 sample has a smooth surface. These results suggest that the surface roughness and particle size of LSF-x samples are according to their degree of crystallinity. Owing to the lattice distortion, the amorphous structure grows in quantity with the increase of Y doping until the LSF-x sample is unable to form a complete pyrochlore structure.

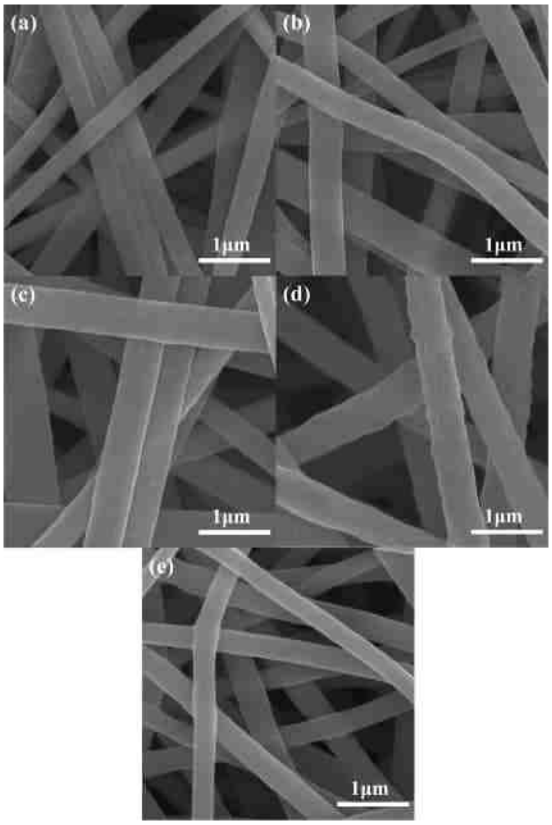

Figure 3. SEM images of (a) LSF-0.1, (b) LSF-0.2, (c) LSF-0.3, (d) LSF-0.4, and (e) LSF-0.5.

To further investigate the LSF-x ($0.1 \leq x \leq 0.5$) samples, we have characterized their nanostructure and crystalline characteristics using HRTEM, and these results are exhibited in Figure 4. The graphs in Figure 4a−e correspond to the TEM photos of LSF-0.1, LSF-0.2, LSF-0.3, LSF-0.4, and LSF-0.5, respectively. These images show that all samples are made of particles or crystallites with a close connection, and their sizes are influenced by the variation of Y proportion. As $0.1 \leq x \leq 0.4$, the grain sizes of LSF-x samples increase with the growth of Y content, and their homogeneous particles also change to inhomogeneous. For the LSF-0.5 sample, the particle sizes sharply decline, owing to the formation of the amorphous structure in excessive levels of Y doping. Besides, Figure 4f−j exhibits the lattice fringes of LSF-x ($0.1 \leq x \leq 0.5$) samples. As shown in Figure 4f,g, the LSF-0.1 and LSF-0.2 samples both display clear lattice fringes, and their interplanar spacing is 0.172 and 0.196 nm, respectively. However, excessive Y doping can damage the lattice structure of LSF-x. The little change of LSF-0.3 sample lattice can be proved by the minor bending of its lattice fringes in Figure 4h, and its interplanar spacing grows up to 0.216 nm at the same time. Moreover, an interplanar spacing of the LSF-0.4 sample observed from Figure 4i is 0.241 nm, while the bend, crack, and malposition of its lattice fringes can well determine the occurrence of lattice distortion. In addition, no complete lattice fringe shown in Figure 4j indicates that the LSF-0.5 sample has an amorphous structure, which is in good agreement with the above-mentioned results. In conclusion, although Y doping can improve the number of oxygen vacancies, we should maintain the Y content at an appropriate level.

3.1.3. XPS Analyses. To determine the change regularity of the composition and the valence state of LSF-x ($0.1 \leq x \leq 0.5$) samples with different Y-doping proportions, we investigate these samples by performing the XPS analyses. As shown in Figure 5a, these XPS spectra of LSF-x ($0.1 \leq x \leq 0.5$) samples prove the presence of La, Ti, and Y elements. From Figure 5b, we can observe two spin−orbit characteristic peaks of all LSF-x ($0.1 \leq x \leq 0.5$) samples, which can be attributed to Y $3d_{5/2}$ and Y $3d_{3/2}$, respectively. Moreover, the intensity of these characteristic peaks has been increased obviously with the gradual extending of the Y-doping ratio. Furthermore, Figure 5c displays the Ti $2p_{1/2}$ and Ti $2p_{3/2}$ spin−orbit peaks of all LSF-x ($0.1 \leq x \leq 0.5$) samples, which both can be divided into two satellite peaks and corresponded to Ti^{3+} and Ti^{4+}, respectively. In these XPS spectra, the binding energies of Ti^{3+} were located at 457.8 eV (Ti $2p_{3/2}$) and 463.5 eV (Ti $2p_{1/2}$) and Ti^{4+} were located at 458.1 eV (Ti $2p_{3/2}$) and 464.0 eV (Ti $2p_{1/2}$). As the content of Ti^{3+} and Ti^{4+} is related to the peak area, the change of the peak intensity suggests that the proportion of Ti^{3+} is developed with the growth of Y content. Since the concentration of O vacancies is directly proportional to the ratio of Ti^{3+}, the LSF-x ($0.1 \leq x \leq 0.5$) samples can supply more O vacancies with the increase of the Y-doping ratio. Besides, the two main peaks at 529.5 and 531.3 eV in O 1s spin−orbit spectra of LSF-x samples can be ascribed to the lattice oxygen species (O^{2-}) and chemisorbed oxygen species (hydroxyl groups, O^-), respectively (Figure 5d). In these spectra images, the peaks of lattice oxygen species (O^{2-}) are divided into La−O, Y−O, and Ti−O peaks, and the peaks of

https://dx.doi.org/10.1021/acsomega.0c03402
ACS Omega 2020, 5, 29722−29732

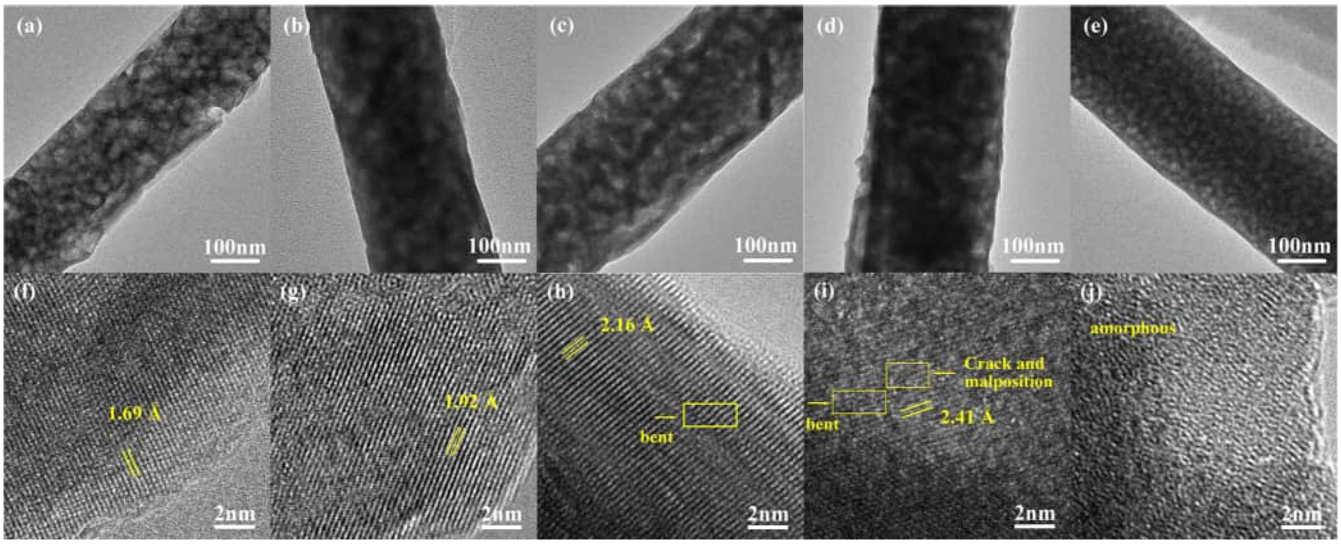

Figure 4. TEM images of (a) LSF-0.1, (b) LSF-0.2, (c) LSF-0.3, (d) LSF-0.4, and (e) LSF-0.5. HRTEM images of (f) LSF-0.1, (g) LSF-0.2, (h) LSF-0.3, (i) LSF-0.4, and (j) LSF-0.5.

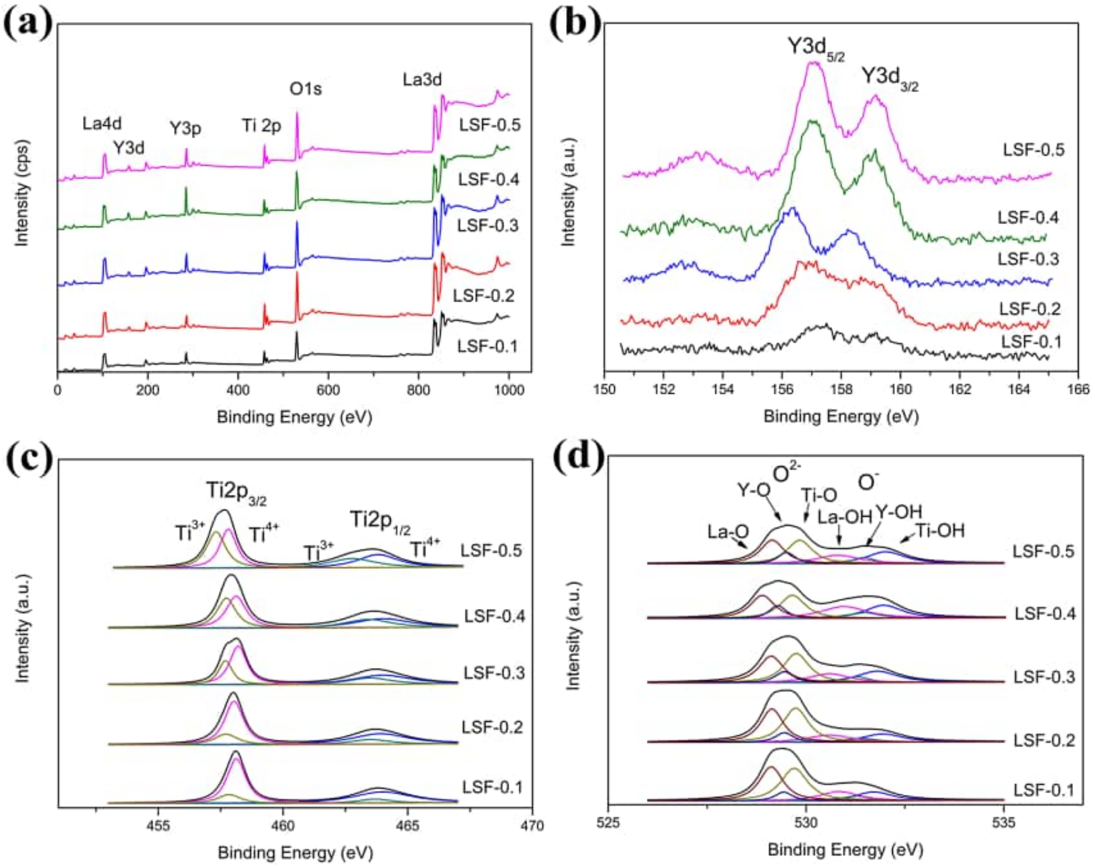

Figure 5. (a) XPS pattern of LSF-x; (b) Y 3d XPS spectra of LSF-x; (c) Zr 3d XPS spectra of LSF-x; and (d) O 1s XPS spectra of LSF-x.

chemisorbed oxygen species (hydroxyl groups, O^-) consist of La−OH, Y−OH, and Ti−OH peaks. Generally, because the hydroxyl groups adsorbed by LSF-x ($0.1 \leq x \leq 0.5$) samples can be regarded as evidence of the existence of oxygen vacancies, the increase of the O^- peak area can further prove the growth of oxygen vacancies after doping. These results are well accorded with a previous study.

3.2. Electrochemical Properties. *3.2.1. Effects of Y Content.* In this study, we have prepared the LSF-x ($0.1 \leq x \leq 0.5$) electrodes as working electrodes using the corresponding LSF-x ($0.1 \leq x \leq 0.5$) samples. To investigate the effects of Y-doping proportion on the electrochemical performance of LSF-x ($0.1 \leq x \leq 0.5$) samples, the electrochemical workstation has been used to explore the electrochemical behavior of working electrodes by cyclic voltammetry (CV), galvanostatic charge−discharge (GCD) measurements, and the AC impedance method.

https://dx.doi.org/10.1021/acsomega.0c03402
ACS Omega 2020, 5, 29722−29732

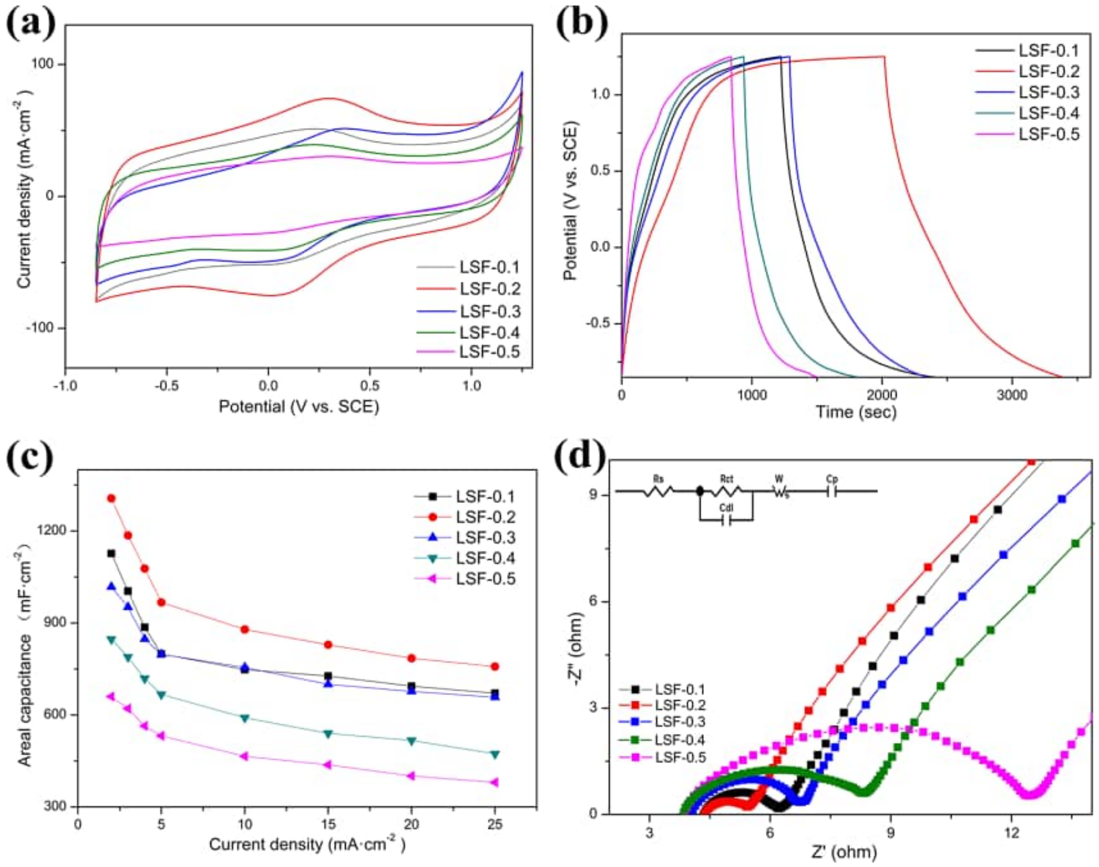

Figure 6. (a) Cyclic voltammograms (CVs) of LSF-x electrodes in a 1 M Na_2SO_4 solution at a sweep rate of 50 mV·s^{-1}. (b) Galvanostatic charge–discharge (GCD) curves of LSF-x electrodes in a 1 M Na_2SO_4 solution at 2 mA·cm^{-2}. (c) Comparison of the areal capacities as a function of the current density for LSF-x electrodes. (d) Nyquist plots of the LSF-x electrodes in a 1 M Na_2SO_4 solution. The inset is the equivalent circuit diagram from the EIS analysis.

The CV curves of LSF-x ($0.1 \leq x \leq 0.5$) electrodes measured in a 1 M Na_2SO_4 aqueous solution at a scan rate of 50 mV·s^{-1} with a three-electrode system are manifested in Figure 6a. Because the larger CV curve integral area represents the higher specific capacitance, the LSF-0.2 electrode may possess the best electrochemical property compared with the other LSF-x ($0.1 \leq x \leq 0.5$) electrodes. Moreover, Figure S1 exhibits the CV curves of all LSF-x ($0.1 \leq x \leq 0.5$) electrodes tested in a 1 M Na_2SO_4 aqueous solution at various scan rates ranging from 5 to 100 mV·s^{-1}. Generally, these curves all display a deformed rectangle shape with a pair of remarkable redox peaks, and the potential range is −0.85 to 1.25 V versus the reference electrode (vs SCE). Based on the oxygen intercalation mechanism, these peaks can correspond to the conversion of Ti^{3+}/Ti^{4+}. The reversible redox reaction can be described as following

$$Ti^{3+} + H_2O \underset{\text{discharge}}{\overset{\text{charge}}{\rightleftharpoons}} Ti(OH)^{3+} + H^+ + e^- \quad (4)$$

For definitely stating this energy-storage mechanism, the schematic diagrams of oxygen intercalation of LSF-x ($0.1 \leq x \leq 0.5$) samples are shown in Figure S2. According to this image, it can be observed that oxygen ions at the surface can be absorbed and migrated to oxygen vacancies in the bulk structure through the lattice. Although the reversible redox reactions of Ti^{3+}/Ti^{4+} occurred in the lattice, the composition of main elements and crystal lattice still remains the same in this process. Hence, these oxygen vacancies in LSF-x ($0.1 \leq x \leq 0.5$) samples are considered as the main charge-storage sites for the pseudocapacitive process, and the redox reaction can be known as a topotactic reaction. As the previous analyses indicate that the amount of oxygen vacancies in LSF-x ($0.1 \leq x \leq 0.5$) samples is multiplied along with the increase of Y doping; the Y doping can improve the electrochemical performance of samples. However, a large proportion of Y doping can cause the lattice distortion of LSF-x ($0.1 \leq x \leq 0.5$) samples. As a result, to obtain the best energy-storage property, consideration must be given to both the degree of crystallinity and the number of oxygen vacancies.

Figure 6b shows the GCD curves of all LSF-x ($0.1 \leq x \leq 0.5$) samples tested at a current density of 2 mA·cm^{-2} with the potential window of −0.85 to 1.25 V (vs SCE) in a 1 M Na_2SO_4 aqueous solution. Due to the reversible redox process of pseudocapacitance, all of the curves do not have a mirror symmetry, which is in good agreement with the CV consequences. Since the energy-storage performance of all LSF-x ($0.1 \leq x \leq 0.5$) electrodes can be estimated by comparing the relevant discharging time, the LSF-0.2 electrode with the longest discharge time in these GCD curves suggests the best. Furthermore, the GCD curves of LSF-0.1, LSF-0.2, LSF-0.3, LSF-0.4, and LSF-0.5 electrodes have been measured at various current densities to further investigate their electrochemical behaviors (Figure S3a–e). These GCD curves of the same sample display an approximate shape under different current densities, showing that all electrodes can have the good electrochemical ability at a high power density. To

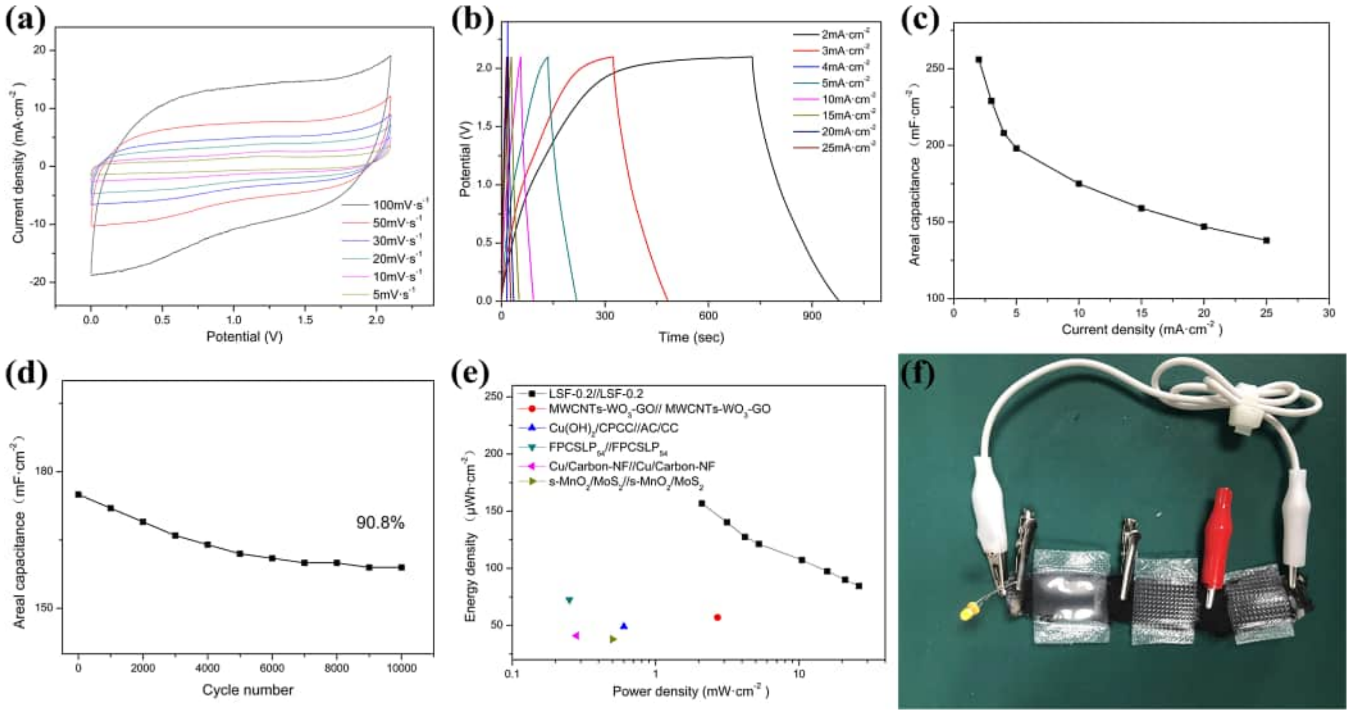

Figure 7. (a) Cyclic voltammograms (CVs) of the LSF-0.2//LSF-0.2 device at various sweep rates of 5−100 mV·s^{-1}. (b) Galvanostatic charge−discharge (GCD) curves of the LSF-0.2//LSF-0.2 device at different current densities of 2−25 mA·cm^{-2}. (c) Comparison of the areal capacities as a function of the current density for the LSF-0.2//LSF-0.2 device. (d) Cycling performance of the LSF-0.2//LSF-0.2 device at a current density of 25 mA·cm^{-2}. (e) Power density vs energy density of the LSF-0.2//LSF-0.2 device in a Ragone plot. (f) Yellow LED illuminated by two LSF-0.2//LSF-0.2 devices connected in series.

determine the above results, we have calculated the corresponding areal capacitance of LNF-x electrodes by these GCD curves of all of the LSF-x ($0.1 \leq x \leq 0.5$) electrodes at different current densities, ranging from 2 to 25 mA·cm^{-2} according to eq 1, and these results are exhibited in Figure 6c. In this image, the LSF-0.2 electrode shows the best capacitance with the highest value of 1307 mF·cm^{-2} (324 F·g^{-1}), and this result can further confirm this oxygen intercalation mechanism.

To further explore the electrochemical properties of LSF-x ($0.1 \leq x \leq 0.5$) electrodes affected by the change of the Y content, the frequency response of electrodes has been studied by carrying out the electrochemical impedance spectroscopy technique (EIS) test at open-circuit potential. As shown in Figure 6d, the Nyquist plots of all LSF-x ($0.1 \leq x \leq 0.5$) electrodes are both consisted of two regions. In the high-frequency range, there are five Warburg semicircles with different radii, while the five corresponding straight lines with a slope of 60° are exhibited in the low-frequency range. For the purpose of analyzing the properties of energy storage and dissipation, we have fitted the equivalent circuit according to the EIS data using the Z-View computer software. The equivalent circuit is presented in the inset of Figure 6d, and the values of solution resistance (R_s), double-layer capacitance between the solution and the electrode (C_{dl}), charge-transfer resistance between the solution and the electrode (R_{ct}), pseudocapacitance of the electrode (C_p), and Warburg impedance (W_s) are calculated and listed in Table S1. From this table, we can observe that R_s values of various electrodes are close, about 4.2 Ω. Besides, since the low R_{ct} value suggests the fast charge and discharge rates, these data can also be applied to judge the oxygen intercalation capability. In conclusion, these results obtained from the table are in very good agreement with the changing trend of electrochemical properties of LSF-x ($0.1 \leq x \leq 0.5$) electrodes influenced by Y doping.

3.2.2. Electrochemical Performance of the All-Solid-State Device. As the LSF-0.2 electrode shows better areal capacitance values than other working electrodes, the symmetric flexible all-solid-state supercapacitor devices are prepared by two LSF-0.2 electrodes with the PVA−Na_2SO_4 gel as the solid electrolyte. As exhibited in Figure S4, the thickness of the LSF-0.2 sample used in this device is around 23.7 μm, while the mass density of the film is 3.2 mg·cm^{-2}.

To further investigate the electrochemical performance of this all-solid-state device, the CV curves of the device are measured by cyclic voltammetry at various scan rates and are shown in Figure 7a. These CV curves obtained at different scan rates both have nearly rectangular shapes and maintain almost the same shape under different integral areas. This result indicates that the all-solid-state device has a safe, stable, and fast charge−discharge performance. Furthermore, this LSF-0.2//LSF-0.2 flexible all-solid-state supercapacitor device shows a high stable voltage window of 2.1 V, which is much higher than the previous studies as follows: 1.8 V for $NiFe_2O_4$@rGO//$NiFe_2O_4$@rGO (PVA−KNO_3 gel),[39] 1.0 V for rGO/CNT-c//rGO/CNT-c (PVA−H_2SO_4 gel),[40] 2.0 V for MnO_2/CNTs/cellophane//MnO_2/CNTs/cellophane (PVA−H_2SO_4 gel),[41] 1.8 V for MnSe@CT//MnSe@CT (PVA/LiCl hydrogel),[42] 1.8 V for Ni−Co/CP//AC (PBI−KOH gel),[43] and 1.2 V for AC//AC (PGPE).[44]

To study the energy-storage ability of the LSF-0.2//LSF-0.2 flexible all-solid-state device, we need to calculate the areal specific capacitances through eq 1. Consequently, we have

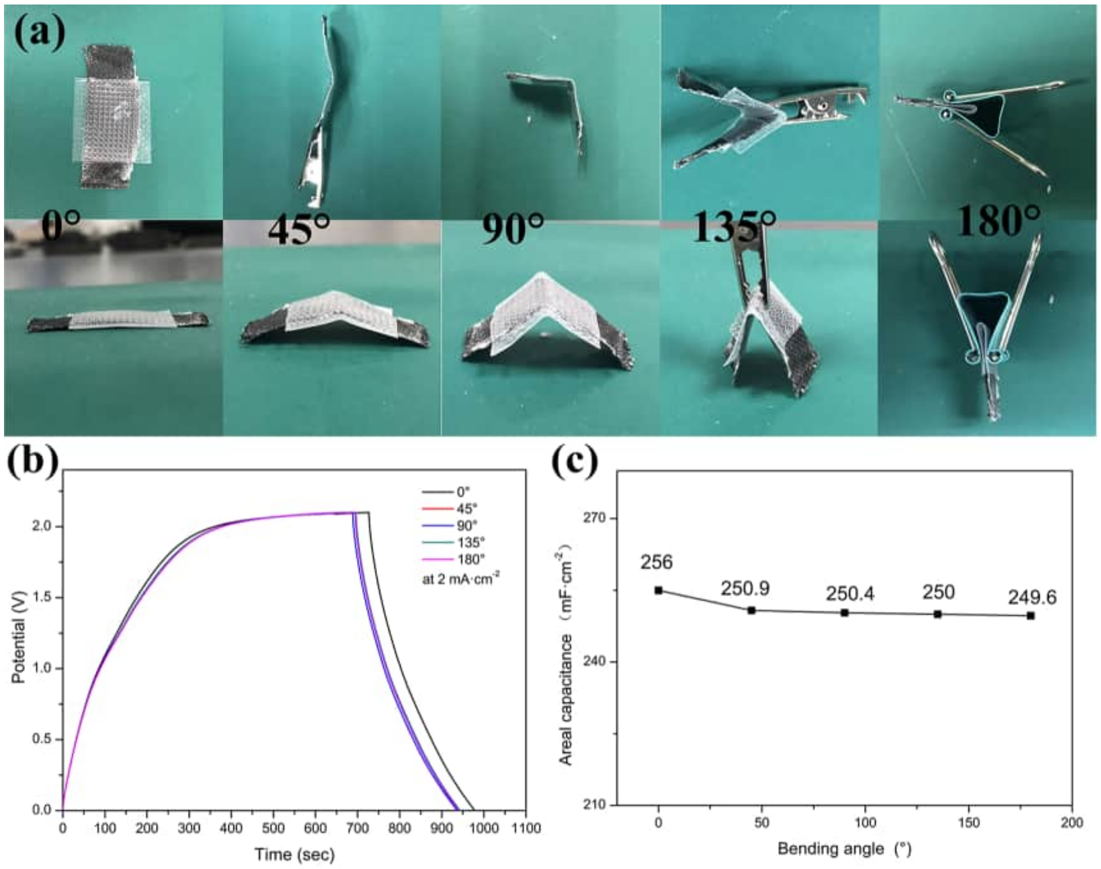

Figure 8. (a) Digital photographs of the LSF-0.2//LSF-0.2 device bent at different angles (0, 45, 90, 135, and 180°). (b) Galvanostatic charge–discharge (GCD) curves of the LSF-0.2//LSF-0.2 device bent at different angles (0, 45, 90, 135, and 180°). (c) Areal capacitance of the LSF-0.2//LSF-0.2 device obtained at each angle.

tested the LSF-0.2//LSF-0.2 flexible all-solid-state device by the galvanostatic charge–discharge method at different current densities and the GCD curves are exhibited in Figure 7b. These GCD curves measured at different current densities ranging from 2 to 25 mA·cm^{-2} show similar shapes, indicating a high Coulombic efficiency of the all-solid-state device. Then, the specific capacity of the device referred by mass is shown in Figure S5. Moreover, Figure 7c shows the corresponding specific capacitances of this device, calculated according to various GCD curves. From this image, we can observe that the maximum areal capacitance value of this device is 255.9 mF·cm^{-2} at a current density of 2 mA·cm^{-2}. Meanwhile, as the current density increases up to 25 mA·cm^{-2}, the value of capacitance is 138.1 mF·cm^{-2}, and the retention is about 53.9%. To examine the cycle stability of the LSF-0.2//LSF-0.2 flexible all-solid-state device, we have carried out the successive charge–discharge cycles at a high current density of 10 mA·cm^{-2} for 10 000 times. As shown in Figure 7d, the value of capacitance of this device is still 90.8%, retained after 10 000 cycles. Furthermore, we display the GCD curves with the 1st, 1000th, and 10 000th charge–discharge cycle test in Figure S6, and these curves all have obvious similar shapes. These results can demonstrate that the LSF-0.2//LSF-0.2 flexible all-solid-state device owns an outstanding cycling performance to be ready for application.

To better determine the energy-storage properties of this flexible all-solid-state device, we have calculated the value of the areal energy density (E_A) and areal power density (P_A) based on eqs 2 and 3. As shown in Figure 7e, the maximum value of E_A is 156.8 μWh·cm^{-2} at a low P_A of 2.1 mW·cm^{-2}, and E_A still remains at 84.5 μWh·cm^{-2} while P_A increased to 26.3 mW·cm^{-2}. However, since the energy densities and power densities of most all-solid-state supercapacitors reported before have been calculated according to the volume and the mass, we also present the maximum and minimum energy densities (E_V in Wh·m^{-3} and E_m in Wh·kg^{-1}) with the corresponding power densities (P_V in W·m^{-3} and P_m in W·kg^{-1}). The maximum volumetric energy densities (E_V) and mass-energy densities (E_m) are 66.1 Wh·cm^{-3} and 49 Wh·kg^{-1} when the corresponding volumetric power densities (P_V) and mass power densities (P_m) are 0.89 W·cm^{-3} and 656.3 W·kg^{-1}, respectively. Moreover, corresponding to previous data, the maximum values of P_V and P_m are 11.1 W·cm^{-3} and 8.2 kW·kg^{-1}, while the minimum E_V and E_m are 35.7 Wh·cm^{-3} and 26.4 Wh·kg^{-1}, respectively. In addition, to compare with the devices studied before, we give the examples as follows: MWCNTs-WO_3-GO//MWCNTs-WO_3-GO (57 μWh·cm^{-2} at 2.70 mW·cm^{-2}),[45] $Cu(OH)_2$/CPCC//AC/CC (49 μWh·cm^{-2} at 0.6 mW·cm^{-2}),[46] $FPCSLP_{54}$//$FPCSLP_{54}$ (72.5 μWh·cm^{-2} at 250 μW·cm^{-2}),[47] Cu/Carbon-NF//Cu/Carbon-NF (41 μWh·cm^{-2} at 280 μW·cm^{-2}),[48] and s-MnO_2/MoS_2//s-MnO_2/MoS_2 (38 μWh·cm^{-2} at 500 μW·cm^{-2}).[49]

Besides, the energy-storage performance of the state-of-the-art supercapacitors, which are calculated based on the mass, are listed as follows: 80$MnFe_2O_4$–20rGO//80$MnFe_2O_4$–20rGO (27.7 Wh·kg^{-1} at 750 W·kg^{-1}),[50] Mn_3O_4/rGO//AC (36.76 Wh·kg^{-1} at 500 W·kg^{-1}),[51] $NiCo_2S_4$@MF/PPY‖Fe_2O_3@MF/PPY (20.1 Wh·kg^{-1} at 159.4 W·kg^{-1}),[52] MS-CMTBs//MS-

https://dx.doi.org/10.1021/acsomega.0c03402
ACS Omega 2020, 5, 29722–29732

CMTBs (15.1 Wh·kg^{-1} at 250 W·kg^{-1}),[53] Ni−Co−S//AC (48.9 Wh·kg^{-1} at 390 W·kg^{-1}),[54] rGO-$FeMoO_4$@CC//rGO-MnO_2@CC (38.8 Wh·kg^{-1} at 1.34 kW·kg^{-1}),[55] $NiCo_2O_4$//$NiCo_2O_4$ (30.5 Wh·kg^{-1} at 750 W·kg^{-1}),[56] and NCHrGO/CF//NQrGO/CF (50.7 Wh·kg^{-1} at 204 W·kg^{-1}).[57] In addition, the Ragone plots of these devices based on mass are exhibited in Figure S7. These results can further prove that the LSF-0.2//LSF-0.2 flexible all-solid-state device shows excellent energy-storage performance. For the practical application, we have lighted a light-emitting diode (LED, ~4.2 V) using three LSF-0.2//LSF-0.2 flexible all-solid-state devices in series, and the digital photo is presented in Figure 7f.

3.2.3. Flexibility of the Device. To apply the LSF-0.2//LSF-0.2 device in a more wide field, this all-solid-state supercapacitor should show excellent mechanical flexibility. For determining the flexibility, we have tested the electrochemical properties of the LSF-0.2//LSF-0.2 device bent at different angles (0, 45, 90, 135, and 180°), and the corresponding photos are displayed in Figure 8a. The change of electrochemical performance of the device after bending can be analyzed according to the GCD curves (at 2 mA·cm^{-2}) in Figure 8b. From this image, although these curves show a negligible change of shapes at different bending angles, the charge−discharge cycle times gradually become little narrow with the increase of the bending angle. While this bent device can still work normally, the energy-storage ability of the device may decrease a lot. To further confirm the stability of the LSF-0.2//LSF-0.2 device after bending, the areal capacitances of the device corresponding to each bending angles are calculated and presented in Figure 8c. It can be noticed that the values of capacitance decrease obviously with the growth of the bending angle, and the minimum is 249.6 mF·cm^{-2} (97.5% left) at 180°. This result demonstrates excellent flexibility of the LSF-0.2//LSF-0.2 device.

4. CONCLUSIONS

In summary, the $La_2Y_xTi_{2-x}O_3$ flexible self-supporting films ($0.1 \leq x \leq 0.5$) have been successfully synthesized by electrospinning combined with the low-temperature sintering technology in this research. To investigate the variety of crystal structures of LSF-x influenced by different proportions of Y doping, we have applied the XRD, SEM, TEM, and XPS measurement on these samples. These results suggest that the oxygen-vacancy numbers increase with the growth of Y-doping content but the degree of crystallinity decreases with the increasing Y doping. To balance their relationship, we should not dope too much Y elements. In addition, to determine the optimum Y-doping content, we have explored the electrochemical properties of working electrodes prepared by LSF-x ($0.1 \leq x \leq 0.5$) samples. As the LSF-0.2 electrode shows a higher areal capacitance of 1.3 F·cm^{-2} in a 1 M Na_2SO_4 electrolyte than the other electrodes, $x = 0.2$ can be considered as the best doping ratio. As a result, we have fabricated the symmetric flexible all-solid-state supercapacitor device using two LSF-0.2 electrodes as working electrodes and PVA−Na_2SO_4 gel as the solid electrolyte, and this device has a high areal capacitance of 255.9 mF·cm^{-2} at a current density of 2 mA·cm^{-2}. Moreover, the maximum value of the energy density of this device is 156.8 μWh·cm^{-2} with a low corresponding power density of 2.1 mW·cm^{-2}. In addition, this all-solid-state device also shows a long cycle life and outstanding flexibility, which can be conducive to extend the application range of the device. Thus, we have prepared a new energy-storage material with excellent flexibility for a wearable energy-storage device in this study.

■ ASSOCIATED CONTENT

Supporting Information

The Supporting Information is available free of charge at https://pubs.acs.org/doi/10.1021/acsomega.0c03402.

Table S1: the values of the equivalent circuit fitted for EIS spectra of LSF-x electrodes; Figure S1: the cyclic voltammograms (CVs) of (a) LSF-0.1, (b) LSF-0.2, (c) LSF-0.3, (d) LSF-0.4, and (e) LSF-0.5 electrodes at various sweep rates of 5−100 mV·s^{-1}; Figure S2: the mechanism of oxygen intercalation into LSF-x; Figure S3: the galvanostatic charge−discharge (GCD) curves of (a) LSF-0.1, (b) LSF-0.2, (c) LSF-0.3, (d) LSF-0.4, and (e) LSF-0.5 electrodes at different current densities of 2−25 mA·cm^{-2}; Figure S4: the cross-sectional SEM images of (a) LSF-0.2 and (b) piece of the LSF-0.2 flexible thin film; Figure S5: the specific capacity of the LSF-0.2//LSF-0.2 device referred by mass; Figure S6: the galvanostatic charge−discharge (GCD) curves of the LSF-0.2//LSF-0.2 device at the 1th, 1000th, and 10 000th cycles; and Figure S7: the power density vs energy density of the LSF-0.2//LSF-0.2 device in a Ragone plot (PDF)

■ AUTHOR INFORMATION

Corresponding Authors

Wenfeng Qiu − *South China Advanced Institute for Soft Matter Science and Technology, South China University of Technology, Guangzhou 510641, P. R. China;* orcid.org/0000-0002-4935-3267; Email: wfqiu@scut.edu.cn

Tong Zhao − *South China Advanced Institute for Soft Matter Science and Technology, South China University of Technology, Guangzhou 510641, P. R. China; Laboratory of Advanced Polymeric Materials, Institute of Chemistry, Chinese Academy of Sciences, Beijing 100190, P. R. China;* orcid.org/0000-0002-9517-3240; Email: tzhao@iccas.ac.cn

Authors

Yi Cao − *South China Advanced Institute for Soft Matter Science and Technology, South China University of Technology, Guangzhou 510641, P. R. China*

Peiyuan Tang − *South China Advanced Institute for Soft Matter Science and Technology, South China University of Technology, Guangzhou 510641, P. R. China*

Complete contact information is available at: https://pubs.acs.org/10.1021/acsomega.0c03402

Notes

The authors declare no competing financial interest.

■ ACKNOWLEDGMENTS

This work was financially supported by the Recruitment Program of Guangdong (No. 2016ZT06C322) and the Guangdong Provincial Key Laboratory of Functional and Intelligent Hybrid Materials and Devices (No. 2019B121203003).

REFERENCES

(1) Gao, W.; Ota, H.; Kiriya, D.; Takei, K.; Javey, A. Flexible Electronics toward Wearable Sensing. *Acc. Chem. Res.* **2019**, *52*, 523−533.

(2) Xu, K.; Lu, Y.; Takei, K. Multifunctional Skin-Inspired Flexible Sensor Systems for Wearable Electronics. *Adv. Mater. Technol.* **2019**, *4*, No. 1800628.

(3) Jayathilaka, W. A. D. M.; Qi, K.; Qin, Y.; Chinnappan, A.; Serrano-Garcia, W.; Baskar, C.; Wang, H.; He, J.; Cui, S.; Thomas, S. W.; Ramakrishna, S. Significance of Nanomaterials in Wearables: A Review on Wearable Actuators and Sensors. *Adv. Mater.* **2019**, *31*, No. 1805921.

(4) Zamarayeva, A. M.; Ostfeld, A. E.; Wang, M.; Duey, J. K.; Deckman, I.; Lechene, B. P.; Davies, G.; Steingart, D. A.; Arias, A. C. Flexible and stretchable power sources for wearable electronics. *Sci. Adv.* **2017**, *3*, No. e1602051.

(5) Zhang, W.; Feng, P.; Chen, J.; Sun, Z.; Zhao, B. Electrically conductive hydrogels for flexible energy storage systems. *Prog. Polym. Sci.* **2019**, *88*, 220−240.

(6) Sumboja, A.; Liu, J.; Zheng, W. G.; Zong, Y.; Zhang, H.; Liu, Z. Electrochemical energy storage devices for wearable technology: a rationale for materials selection and cell design. *Chem. Soc. Rev.* **2018**, *47*, 5919−5945.

(7) Xing, J.; Tao, P.; Wu, Z.; Xing, C.; Liao, X.; Nie, S. Nanocellulose-graphene composites: A promising nanomaterial for flexible supercapacitors. *Carbohydr. Polym.* **2019**, *207*, 447−459.

(8) Dubal, D. P.; Chodankar, N. R.; Kim, D.-H.; Gomez-Romero, P. Towards flexible solid-state supercapacitors for smart and wearable electronics. *Chem. Soc. Rev.* **2018**, *47*, 2065−2129.

(9) Zhu, Y.-H.; Yang, X.; Bao, D.; Bie, X.-F.; Sun, T.; Wang, S.; Jiang, Y.-S.; Zhang, X.-B.; Yan, J.-M.; Jiang, Q. High-Energy-Density Flexible Potassium-Ion Battery Based on Patterned Electrodes. *Joule* **2018**, *2*, 736−746.

(10) Wang, Q.; Ma, Y.; Liang, X.; Zhang, D.; Miao, M. Flexible supercapacitors based on carbon nanotube-MnO2 nanocomposite film electrode. *Chem. Eng. J.* **2019**, *371*, 145−153.

(11) Eftekhari, A. The mechanism of ultrafast supercapacitors. *J. Mater. Chem. A* **2018**, *6*, 2866−2876.

(12) Li, Q.; Zheng, S.; Xu, Y.; Xue, H.; Pang, H. Ruthenium based materials as electrode materials for supercapacitors. *Chem. Eng. J.* **2018**, *333*, 505−518.

(13) Jha, M. K.; Ball, R.; Seelaboyina, R.; Subramaniam, C. All Solid-State Coaxial Supercapacitor with Ultrahigh Scan Rate Operability of 250 000 mV/s by Thermal Engineering of the Electrode-Electrolyte Interface. *ACS Appl. Energy Mater.* **2020**, *3*, 3454−3464.

(14) Lv, T.; Liu, M.; Zhu, D.; Gan, L.; Chen, T. Nanocarbon-Based Materials for Flexible All-Solid-State Supercapacitors. *Adv. Mater.* **2018**, *30*, No. 1705489.

(15) Xue, Q.; Sun, J.; Huang, Y.; Zhu, M.; Pei, Z.; Li, H.; Wang, Y.; Li, N.; Zhang, H.; Zhi, C. Recent Progress on Flexible and Wearable Supercapacitors. *Small* **2017**, *13*, No. 1701827.

(16) Zhao, X.; Mao, L.; Cheng, Q.; Li, J.; Liao, F.; Yang, G.; Xie, L.; Zhao, C.; Chen, L. Two-dimensional Spinel Structured Co-based Materials for High Performance Supercapacitors: A Critical Review. *Chem. Eng. J.* **2020**, *387*, No. 124081.

(17) Hillier, N.; Yong, S.; Beeby, S. The good, the bad and the porous: A review of carbonaceous materials for flexible supercapacitor applications. *Energy Rep.* **2020**, *6*, 148−156.

(18) Hu, C.; Zhang, X.; Liu, B.; Chen, S.; Liu, X.; Liu, Y.; Liu, J.; Chen, J. Orderly and highly dense polyaniline nanorod arrays fenced on carbon nanofibers for all-solid-state flexible electrochemical energy storage. *Electrochim. Acta* **2020**, *338*, No. 135846.

(19) Wang, L.; Peng, Q.; Shen, X.; Song, Z.; Chen, W.; Liu, X. Mesoporous Bi2MoO6 quasi-nanospheres anchored on activated carbon cloth for flexible all-solid-state supercapacitors with enhanced energy density. *J. Power Sources* **2020**, *463*, No. 228202.

(20) Liang, X.; Jia, Y.; Liu, Z.; Lei, Z. Growing Iron Oxide Nanosheets on Highly Compressible Carbon Sponge for Enhanced Capacitive Performance. *Acta Phys. Chim. Sin.* **2020**, *36*, No. 1903034.

(21) Li, Y.; Wang, Q.; Shao, J.; Li, K.; Zhao, W. NiCo2O4/C Core-Shell Nanoneedles on Ni Foam for All-Solid-State Asymmetric Supercapacitors. *ChemistrySelect* **2020**, *5*, 5501−5506.

(22) Yuan, J.; Yao, D.; Jiang, L.; Tao, Y.; Che, J.; He, G.; Chen, H. Mn-Doped NiMoO4 Mesoporous Nanorods/Reduced Graphene Oxide Composite for High-Performance All-Solid-State Supercapacitor. *ACS Appl. Energy Mater.* **2020**, *3*, 1794−1803.

(23) Gao, W.; Zhu, Y.; Wang, Y.; Yuan, G.; Liu, J.-M. A review of flexible perovskite oxide ferroelectric films and their application. *J. Materiomics* **2020**, *6*, 1−16.

(24) Song, Y.-L.; Wang, Z.-C.; Yan, Y.-D.; Zhang, M.-L.; Wang, G.-L.; Yin, T.-Q.; Xue, Y.; Gao, F.; Qiu, M. Molten salt synthesis and supercapacitor properties of oxygen-vacancy LaMnO3-delta. *J. Energy Chem.* **2020**, *43*, 173−181.

(25) Nan, H.-s.; Hu, X.-y.; Tian, H.-w. Recent advances in perovskite oxides for anion-intercalation supercapacitor: A review. *Mater. Sci. Semicond. Process.* **2019**, *94*, 35−50.

(26) Liu, P.; Liu, J.; Cheng, S.; Cai, W.; Yu, F.; Zhang, Y.; Wu, P.; Liu, M. A high-performance electrode for supercapacitors: Silver nanoparticles grown on a porous perovskite-type material La0.7Sr0.3-CoO3-delta substrate. *Chem. Eng. J.* **2017**, *328*, 1−10.

(27) Wang, N.; Zhang, Q.; Zhao, P.; Yao, M.; Hu, W.; Komarneni, S. Highly mesoporous LaNiO3/NiO composite with high specific surfacMater. Sci. Semicond. Process.e area as a battery-type electrode. *Ceram. Int.* **2017**, *43*, 5687−5692.

(28) Xu, K.; Ma, S.; Shen, Y.; Ren, Q.; Yang, J.; Chen, X.; Hu, J. CuCo2O4 nanowire arrays wrapped in metal oxide nanosheets as hierarchical multicomponent electrodes for supercapacitors. *Chem. Eng. J.* **2019**, *369*, 363−369.

(29) Shanmugavani, A.; Murugeswari, R.; Sanjeeviraja, C.; Selvan, R. K. Nanocrystalline Pyrochlore La2Sn1.6Zr0.4O7 as a New Candidate for Supercapacitor Electrodes. *J. Nanosci. Nanotechnol.* **2015**, *15*, 2790−2797.

(30) Jing, M.-x.; Yang, H.; Han, C.; Chen, F.; Yuan, W.-y.; Ju, B.-w.; Tu, F.-y.; Shen, X.-q.; Qin, S.-b. Improving room-temperature electrochemical performance of solid-state lithium battery by using electrospun La2Zr2O7 fibers-filled composite solid electrolyte. *Ceram. Int.* **2019**, *45*, 18614−18622.

(31) Gul, S. R.; Khan, M.; Zeng, Y.; Wu, B. Theoretical investigations of electronic and thermodynamic properties of Ce doped La2Zr2O7 pyrochlore. *Mater. Res. Express* **2019**, *6*, No. 085210.

(32) Meng, T.; Li, B.; Hu, L.; Yang, H.; Fan, W.; Zhang, S.; Liu, P.; Li, M.; Gu, F. L.; Tong, Y. Engineering of Oxygen Vacancy and Electric-Field Effect by Encapsulating Lithium Titanate in Reduced Graphene Oxide for Superior Lithium Ion Storage. *Small Methods* **2019**, *3*, No. 1900185.

(33) Cao, Y.; Tang, P.; Han, Y.; Qiu, W. Synthesis of La2Ti2O7 flexible self-supporting film and its application in flexible energy storage device. *J. Alloys Compd.* **2020**, *842*, No. 155581.

(34) Wang, L.; Xie, X.; Khang Ngoc, D.; Yan, Q.; Ma, J. Synthesis, characterizations, and utilization of oxygen-deficient metal oxides for lithium/sodium-ion batteries and supercapacitors. *Coord. Chem. Rev.* **2019**, *397*, 138−167.

(35) Mefford, J. T.; Hardin, W. G.; Dai, S.; Johnston, K. P.; Stevenson, K. J. Anion charge storage through oxygen intercalation in LaMnO3 perovskite pseudocapacitor electrodes. *Nat. Mater.* **2014**, *13*, 726−732.

(36) Zhou, R.; Lin, P.; Pun, E. Y. B.; Lin, H.; Yuan, J.; Zhao, X. Hybrid excitation mechanism of upconversion fluorescence in hollow La2Ti2O7: Tm3+/Yb3+ submicron fibers. *J. Mater. Sci.* **2020**, *55*, 4633−4645.

(37) Wang, H.; Li, Q.; Wang, C.; He, H.; Yu, J.; Xu, J. Growth and Dielectric Properties of Ta-Doped La2Ti2O7 Single Crystals. *Crystals* **2018**, *8*, 113.

(38) Edmondson, B. I.; Liu, S.; Lu, S.; Wu, H.; Posadas, A.; Smith, D. J.; Gao, X. P. A.; Demkov, A. A.; Ekerdt, J. G. Effect of SrTiO3 oxygen vacancies on the conductivity of LaTiO3/SrTiO3 heterostructures. *J. Appl. Phys.* **2018**, *124*, No. 185303.

https://dx.doi.org/10.1021/acsomega.0c03402
ACS Omega 2020, 5, 29722−29732

(39) Zhang, X.; Zhu, M.; Ouyang, T.; Chen, Y.; Yan, J.; Zhu, K.; Ye, K.; Wang, G.; Cheng, K.; Cao, D. NiFe2O4 nanocubes anchored on reduced graphene oxide cryogel to achieve a 1.8 V flexible solid-state symmetric supercapacitor. *Chem. Eng. J.* **2019**, *360*, 171−179.

(40) Wu, D.-Y.; Zhou, W.-H.; He, L.-Y.; Tang, H.-Y.; Xu, X.-H.; Ouyang, Q.-S.; Shao, J.-J. Micro-corrugated graphene sheet enabled high-performance all-solid-state film supercapacitor. *Carbon* **2020**, *160*, 156−163.

(41) Rostami, R.; Faraji, M. Porous MnO2-CNTs-Cellophane Nanocomposite for High-Voltage Flexible Supercapacitors. *J. Inorg. Organomet. Polym. Mater.* **2020**, 3438−3447.

(42) Javed, M. S.; Shah, S. S. A.; Hussain, S.; Tan, S.; Mai, W. Mesoporous manganese-selenide microflowers with enhanced electrochemical performance as a flexible symmetric 1.8 V supercapacitor. *Chem. Eng. J.* **2020**, *382*, No. 122814.

(43) Chen, L.; Ou, D.; Zhang, G.; Yan, J.; Liu, J.; Wang, Z.; Wang, Y.; Cui, J.; Zhang, Q.; Zhang, Y.; Hu, X.; Wu, Y. Ni-Co coordination hollow spheres for high performance flexible all-solid-state supercapacitor. *Electrochim. Acta* **2020**, *337*, No. 135828.

(44) Yan, C.; Jin, M.; Pan, X.; Ma, L.; Ma, X. A flexible polyelectrolyte-based gel polymer electrolyte for high-performance all-solid-state supercapacitor application. *RSC Adv.* **2020**, *10*, 9299−9308.

(45) Faraji, M.; Soltanahmadi, R. K.; Aydisheh, H. M.; Bavani, B. M. 2.0-V flexible all-solid-state symmetric supercapacitor device with high electrochemical performance composed of MWCNTs-WO3-graphite sheet. *Ionics* **2020**, *26*, 3003−3013.

(46) Zhu, D.; Yan, M.; Chen, R.; Liu, Q.; Liu, J.; Yu, J.; Zhang, H.; Zhang, M.; Liu, P.; Li, J.; Wang, J. 3D Cu(OH)(2) nanowires/carbon cloth for flexible supercapacitors with outstanding cycle stability. *Chem. Eng. J.* **2019**, 348−355.

(47) Peng, Z.; Wang, C.; Zhang, Z.; Zhong, W. Synthesis and Enhancement of Electroactive Biomass/Polypyrrole Hydrogels for High Performance Flexible All-Solid-State Supercapacitors. *Adv. Mater. Interfaces* **2019**, No. 1901393.

(48) Zhang, Y.; Zhang, W.; Hui, S.; Fan, C.; Sun, J.; Ying, Z. Preparation of a Super Flexible Cu/Carbon Composite Nanofiber Film Electrode for High-Performance Flexible All-Solid-State Supercapacitors. *J. Electron. Mater.* **2020**, *49*, 3165−3173.

(49) Zhang, H. H.; Wei, J.; Yan, Y.; Guo, Q. J.; Xie, L. Q.; Yang, Z. C.; He, J.; Qi, W.; Cao, Z. S.; Zhao, X. H.; Pan, P.; Li, H. Y.; Zhang, K. L.; Zhao, J. S.; Li, X.; Zhang, P.; Shah, K. W. Facile and scalable fabrication of MnO2 nanocrystallines and enhanced electrochemical performance of MnO2/MoS2 inner heterojunction structure for supercapacitor application. *J. Power Sources* **2020**, *450*, No. 227616.

(50) Makkar, P.; Ghosh, N. N. Facile Synthesis of MnFe2O4 Hollow Sphere-Reduced Graphene Oxide Nanocomposites as Electrode Materials for All-Solid-State Flexible High-Performance Asymmetric Supercapacitors. *ACS Appl. Energy Mater.* **2020**, *3*, 2653−2664.

(51) Huang, Z.; Li, S.; Li, Z.; Li, J.; Zhang, G.; Cao, L.; Liu, H. Mn3O4 nanoflakes/rGO composites with moderate pore size and (O=) C-O-Mn bond for enhanced supercapacitor performance. *J. Alloys Compd.* **2020**, *830*, No. 154637.

(52) Yuan, Z.; Zhang, A.; Jiang, D.; Mao, N.; Tian, J.; Huang, W.; Liu, R.; Liu, J. Hollow 3D Frame Structure Modified with NiCo2S4 Nanosheets and Spinous Fe2O3 Nanowires as Electrode Materials for High-Performance All-Solid-State Asymmetric Supercapacitors. *Chem. - Eur. J.* **2020**, *26*, 4790−4797.

(53) Wang, Y.; Liu, R.; Tian, Y.; Sun, Z.; Huang, Z.; Wu, X.; Li, B. Heteroatoms-doped hierarchical porous carbon derived from chitin for flexible all-solid-state symmetric supercapacitors. *Chem. Eng. J.* **2020**, *384*, No. 123263.

(54) Wang, Z.; Wang, H.; Ji, S.; Wang, H.; Brett, D. J. L.; Wang, R. Design and synthesis of tremella-like Ni-Co-S flakes on co-coated cotton textile as high-performance electrode for flexible supercapacitor. *J. Alloys Compd.* **2020**, *814*, No. 151789.

(55) Ranjith, K. S.; Raju, G. S. R.; Chodankar, N. R.; Ghoreishian, S. M.; Kwak, C. H.; Huh, Y. S.; Han, Y. K. Electroactive Ultra-Thin rGO-Enriched FeMoO4 Nanotubes and MnO2 Nanorods as Electrodes for High-Performance All-Solid-State Asymmetric Supercapacitors. *Nanomaterials* **2020**, *10*, 289.

(56) Cao, Z. Z.; Liu, C. H.; Huang, Y. X.; Gao, Y.; Wang, Y.; Li, Z. M.; Yan, Y. X.; Zhang, M. L. Oxygen-vacancy-rich NiCo2O4 nanoneedles electrode with poor crystallinity for high energy density all-solid-state symmetric supercapacitors. *J. Power Sources* **2020**, *449*, No. 227571.

(57) Zhou, C.; Gao, T.; Liu, Q.; Wang, Y.; Xiao, D. Preparation of quinone modified graphene-based fiber electrodes and its application in flexible asymmetrical supercapacitor. *Electrochim. Acta* **2020**, *336*, No. 135628.

AIP Journal of Applied Physics

Defectenhanced electron field emission from chemical vapor deposited diamond

W. Zhu, G. P. Kochanski, S. Jin, and L. Seibles

Citation: Journal of Applied Physics **78**, 2707 (1995); doi: 10.1063/1.360066
View online: http://dx.doi.org/10.1063/1.360066
View Table of Contents: http://scitation.aip.org/content/aip/journal/jap/78/4?ver=pdfcov
Published by the AIP Publishing

Articles you may be interested in

Field emission from chemical vapor deposition diamond surface with graphitic patches
J. Vac. Sci. Technol. B **20**, 238 (2002); 10.1116/1.1434972

Field emission mechanism from undoped chemical vapor deposition diamond films
J. Vac. Sci. Technol. B **18**, 1031 (2000); 10.1116/1.591321

Field emission spectroscopy from discharge activated chemical vapor deposition diamond
J. Vac. Sci. Technol. B **17**, 1064 (1999); 10.1116/1.590694

Electron field emission from chemical vapor deposited diamond
J. Vac. Sci. Technol. B **14**, 2011 (1996); 10.1116/1.588976

Electron emission from chemical vapor deposited diamond and dielectric breakdown
Appl. Phys. Lett. **65**, 863 (1994); 10.1063/1.112184

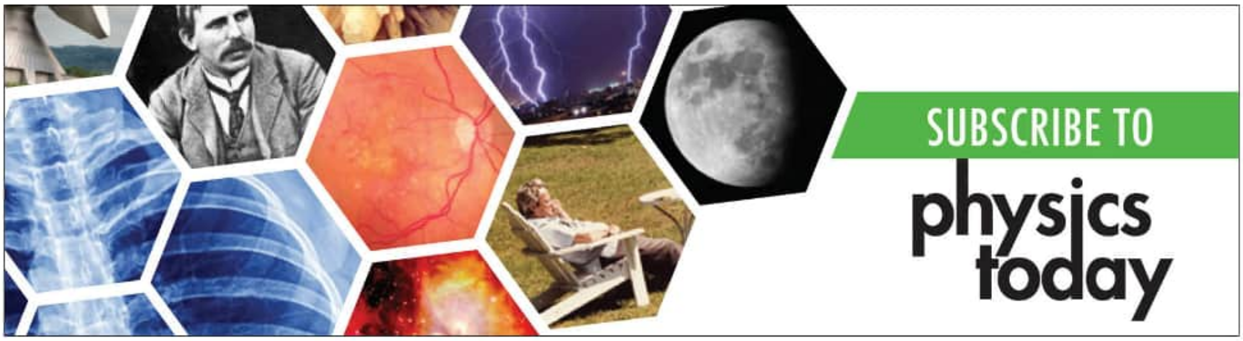

[This article is copyrighted as indicated in the article. Reuse of AIP content is subject to the terms at: http://scitation.aip.org/termsconditions. Downloaded to] IP: 129.22.67.107 On: Mon, 24 Nov 2014 20:31:38

Defect-enhanced electron field emission from chemical vapor deposited diamond

W. Zhu,[a] G. P. Kochanski, S. Jin, and L. Seibles
AT&T Bell Laboratories, 600 Mountain Avenue, Murray Hill, New Jersey 07974

(Received 15 December 1994; accepted for publication 18 April 1995)

Diamond samples with varying defect densities have been synthesized by chemical vapor deposition, and their field emission characteristics have been investigated. Vacuum electron field emission measurements indicate that the threshold electric field required to generate sufficient emission current densities for flat panel display applications (>10 mA/cm^2) can be significantly reduced when the diamond is grown so as to contain a substantial number of structural defects. The defective diamond has a Raman spectrum with a broadened peak at 1332 cm^{-1} with a full width at half maximum (FWHM) of 7–11 cm^{-1}. We establish a strong correlation between the field required for emission and the FWHM of the diamond peak. The threshold fields are typically less than 50 V/μm and can reach as low as 30 V/μm for diamond with a FWHM greater than 8.5 cm^{-1}. It is believed that the defects create additional energy bands within the band gap of diamond and thus contribute electrons for emission at low electric fields. © *1995 American Institute of Physics.*

I. INTRODUCTION

Diamond has attracted much attention as a cold cathode for field emission displays due to its negative electron affinity[1] and robust mechanical and chemical properties. Observations of electron emission from chemical vapor deposited (CVD) diamond under relatively low electric fields (3–40 V/μm) have been reported.[2–4] Fabrication of diamond field emitter arrays has also been attempted,[5] and a diode-structured prototype field emission display based on a diamond-like carbon cathode has been demonstrated.[6] However, it is not entirely clear how the electron emission from these seemingly undoped or p-type-doped CVD diamonds can occur at such low fields.

Usually, for a semiconductor field emitter, the emitted electrons can originate from either the conduction band, the valence band, and/or surface states. Diamond has a wide band gap with $E_g=5.5$ eV. In order to induce stable electron field emission from diamond, either the bulk or the surface must first be made conductive. Additionally, to optimally take advantage of diamond's negative electron affinity to realize low-voltage electron emission, the Fermi level must be as high in the gap as possible. Unfortunately, efforts so far in search of effective and reliable n-type dopants have not been successful.[7,8] Common n-type dopants in silicon such as nitrogen, phosphorus, and arsenic are found to either occupy too deep a level in diamond to be activated or have very low solubilities in diamond. Although p-type semiconducting diamond is readily available by doping with boron, it is not helpful for achieving low-field electron emission because the energy levels filled by electrons are approximately 5 eV below the vacuum level. Surface states do exist in the band gap on reconstructed diamond surfaces,[9,10] but it is not known how the electrons are transported to these surface states to sustain the emission from the undoped or p-type-doped diamonds.

This study identifies structural properties which govern the electron field emission process from undoped and p-type-doped diamonds. Low-voltage diamond field emitters with such properties have been synthesized by controlling CVD process parameters. A strong correlation was established between the field required for emission and the width of the diamond Raman peak. It is believed that the defects create additional energy bands within the band gap of diamond and thus contribute electrons for emission at low electric fields.

II. EXPERIMENTS AND DATA ANALYSIS

The diamond samples were prepared by microwave plasma enhanced CVD using a mixture of methane and hydrogen gases. The methane concentration and growth time was systematically manipulated to produce a variety of diamond samples with varying defect densities as listed in Table I. For the undoped samples, the methane was turned off first in the system shutdown procedure, to allow the hydrogen plasma to etch away any graphite that might exist on the surface. p-type semiconducting diamond was prepared by bubbling hydrogen through a solution of boron oxide (B_2O_3) in ethyl alcohol during the growth process. The dopant levels and distribution were measured by secondary ion mass spectroscopy (SIMS). The introduction of both structural defects and boron dopants increases the conductivity and alters the work function of diamond, thus directly affecting the field emission properties of diamond.

Raman spectroscopy was used to evaluate the structural quality of the diamond samples. As shown in Fig. 1, the FWHM of the diamond peak at 1332 cm^{-1} is a measure of the perfection of the diamond structure (e.g., point defects and stacking faults), and the intensity ratio of the graphitic peak to the diamond peak (I_{g1}/I_{d1}) indicates the amount of graphitic phases present in diamond.[11] We correlated both the peak width and the intensity ratio with the emission properties and found no clear trend between the electric field necessary for emission and the intensity ratio (I_{g1}/I_{d1}). This indicates that the graphitic defects alone do not account for

[a]Electronic mail: zhu@clockwise.att.com

[This article is copyrighted as indicated in the article. Reuse of AIP content is subject to the terms at: http://scitation.aip.org/termsconditions. Downloaded to] IP: 129.22.67.107 On: Mon, 24 Nov 2014 20:31:38

TABLE I. Field emission properties of CVD diamond.

Sample[a]	Growth conditions	FWHM of diamond Raman peak (cm^{-1})	Turn-on field for a current density of 0.01 mA/cm^2 (V/μm)	Threshold field for a current density of 10 mA/cm^2 (V/μm)	Comments
#1	0.4%CH_4/H_2, 7 h	3.8	...	...	no emission
#2	0.8%CH_4/H_2, 45 h	4.2	...	...	no emission
#3	1%CH_4/H_2, 12 h	7.4	72	120	...
#4	0.5%CH_4/H_2, 7 h	7.8	51	84	...
#5	2%CH_4/H_2, 4 h	10.9	22	31	...
#6	1%CH_4/H_2, 7 h	9.4	32	46	...
#7	1%CH_4/H_2, 7 h, and overcoated at 8%CH_4/H_2 for 15 m	10.2	24	40	...
#8	0.3%CH_4/H_2, 30 h, and B doped	3.7	111	164	boron conc. 10^{20} cm^{-3}
#9	5%C_2H_5OH/H_2, 14 h, and B doped	8.1	58	107	boron conc. 10^{20} cm^{-3}
#10	0.6%C_2H_5OH/H_2, 45 h, and B doped	9.1	16	30	boron conc. 10^{19} cm^{-3}
#11	2.5%C_2H_5OH/H_2, 18 h, and B doped	9.2	21	37	boron conc. $>10^{20}$ cm^{-3}
#12	1.5%C_2H_5OH/H_2, 60 h, and B doped	10.5	21	38	boron conc. $>10^{20}$ cm^{-3}

[a]Samples #1, 6, and 7 have an island-type morphology, while all the other samples are continuous films.

the emission characteristics of diamond. We saw a strong correlation of the emission field with the peak width, indicating that other types of defects are at least as important. Scanning electron microscopy (SEM) was used to measure the crystallite size or emitting tip density; we assume that there was one emitter site per crystallite, on average.

The experimental apparatus used for the field emission measurements is shown in Fig. 2. A voltage cycled from zero up to 2 kV was applied to a tungsten anode (1 mm in diameter) to collect electrons emitted from the diamond surface. A precision step controller (3.3 μm step size) was used to control the movement of the probe toward the sample surface,

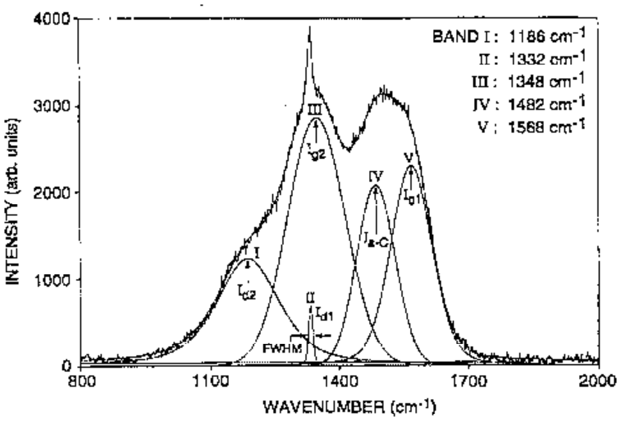

FIG. 1. A representative Raman spectrum with deconvolved components from defective diamond showing the width of the diamond peak at 1332 cm^{-1} and the intensity ratio of the graphitic peak to the diamond peak (I_{g1}/I_{d1}). Peaks I and III are reported to be associated with the small crystal sizes and/or disordered structures of diamond and graphite, respectively (See Ref. 17). Peak IV is due to the presence of amorphous carbon.

and the emission current-voltage (I-V) characteristics was repeatedly measured as a function of the distance between the probe and the diamond surface as shown in Fig. 3. To eliminate the effects of surface contamination on the electron emission from diamond, a hydrogen ion gun (400 V, 0.2 mA/cm^2) was used to gently sputter-clean the sample prior to each measurement. The I-V data were then analyzed using a Fowler–Nordheim equation. The emission current is taken to be an appropriate integral over geometry and a distribution of material parameters:

$$I=2\pi\int R\int N(\zeta)I_t[\zeta,E_m(R)]d\zeta\, dR. \tag{1}$$

This is necessary because the emitting surface is nonuniform, with electronic properties, curvature, and applied field varying from tip to tip. In Eq. (1), the outer integral is over the surface of the emitter, accounting for the decreasing electrical field as one moves a distance R away from the point directly underneath the spherical anode; $E_m(R)$ is the mac-

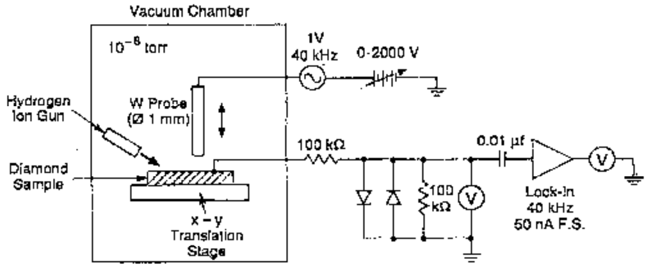

FIG. 2. A schematic diagram of the experimental setup for the field emission measurement.

[This article is copyrighted as indicated in the article. Reuse of AIP content is subject to the terms at: http://scitation.aip.org/termsconditions. Downloaded to] IP: 129.22.67.107 On: Mon, 24 Nov 2014 20:31:38

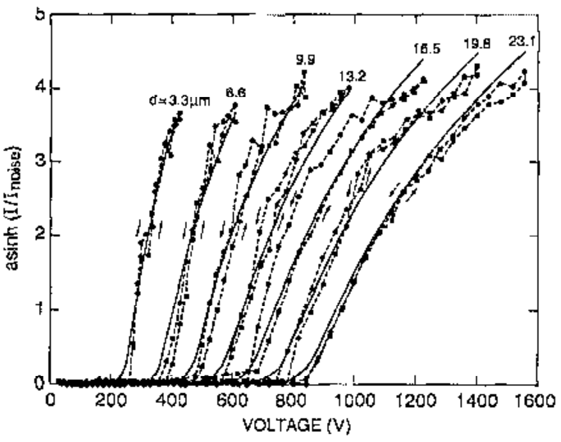

FIG. 3. Representative I-V emission curves from defective CVD diamond obtained at various spacings (d) between the anode and the cathode. The dots and dashed lines are actual data, and the solid lines are fitted curves based on the modified Fowler–Nordheim equation. I_{noise} is 0.1 nA.

roscopic electric field without local field enhancement.[12] The inner integral is over the probability distribution of tip parameters (e.g., some combination of sharpness and work function); we take ζ to be the most important combination of sharpness and work function. $N(\zeta)$ is the density of tips per unit area that have parameter ζ, and I_t is the current emitted by each such tip. The shape of $N(\zeta)$ is generally unknown, though it is clearly broad (when we place a phosphor screen over the emitter, we find that the emission is dominated by small areas; only 1%–10% of the emitter surface contributes nearly all the current). We have taken $N(\zeta)=N_0 \exp(-|\zeta|)/2$, where N_0 is the tip density obtained from the SEM measurement; a Gaussian distribution function gives similar results.

Because of the nonuniformity, it is likely that the best emission region (which may dominate the emission current) is not directly under the end of the probe. We have introduced a parameter α that specifies how far laterally the hot spot is from the probe; this should vary randomly from location to location. We calculate the effective radius R_{eff} of an emitting region that would be required to produce I if the current per tip were constant at its central (e.g., $R=0$) value; we then mathematically move the tip up by $\alpha^2 R_{eff}^2/(2R_{probe})$ (the amount the spherical probe has curved away from the surface at a radius αR_{eff}). We find that fitted values of α are typically less than one, as would be expected.

The current per tip, $I_t=2\pi r^2 \int_0^{\pi/2} \sin\phi \cdot J[\zeta,E_m(R)\cdot f(\phi)]d\phi$ is similarly an angular integral of the local current density over the end of each emitting tip. We take r to be an average tip radius (simply to make units consistent—the value of r collapses into a_0, below); deviations from the average are collapsed into a_1 (below) or ζ. Likewise, the part of the field enhancement factor that depends on tip radius is collapsed into $J(\ldots)$ via other parameters (i.e., a_0 and b_0, below). What is left is the angular dependence of the field enhancement, $f(\phi)$, which is taken from a sphere-on-cone model.[12]

Finally, $J(\ldots)$ is parameterized with the Fowler–Nordheim form: $J=E^2 \exp[(a_0+a_1\zeta)-(b_0+b_1\zeta)/E]$,[13] with a_0 the log of the typical F-N offset, and b_0 the typical F-N slope. As typical, these parameters contain a combination of information on the tip radius, work function, geometry, and surface physics. The parameters a_1 and b_1 then measure the width of the probability distribution of emitters and are proportional to the nonuniformity of the emitters. If $a_1\approx 1$ or $b_1\approx 1$, then one begins to get strong nonuniformity, with tip-to-tip current variations on the order of 100%.

Therefore, in addition to the two parameters a_0 and b_0 which are used to describe the classic Fowler–Nordheim equation, three new parameters (a_1, b_1, α) were found necessary to achieve a good fit of the data. The fitting of data to calculations was carried out by means of a weighted least-square fit between $\operatorname{asinh}(I_{data}/I_{noise})$ and $\operatorname{asinh}(I_{calculated}/I_{noise})$, where I_{noise} was the observed measurement noise near zero current (10^{-10} A). The hyperbolic arc-sine function was used as a substitute for log function that has good behavior at zero current. The appropriately fitted parameters are then used to extrapolate the I-V characteristics of emitters to a standard display pixel. Both the threshold electric field required to obtain a current of 1 μA over an area of 100 μm^2 (that is, a current density of 10 mA/cm^2 for sufficient phosphor activation) and the turn-on field (for a current density of 0.01 mA/cm^2) were subsequently calculated.

III. RESULTS AND DISCUSSION

As indicated in Table I, high quality diamond (e.g., samples #1 and 2), that has low defect densities and a narrow Raman peak (FWHM <5 cm^{-1}), generally does not emit electrons. When the applied electric field increases, electrical breakdown or arcing eventually occurred on the surface due to the highly insulating nature of the high quality diamond. However, the defective diamond (e.g., samples #3–7) with the Raman peak width broadened to beyond approximately 7 cm^{-1} exhibited characteristic Fowler–Nordheim emission behavior. Smooth and consistent I-V curves were measured from these samples in a history independent, reproducible manner as shown in Fig. 3. In the figure, the voltage was raised from zero to the maximum (+2 kV) and then decreased to zero. As the probe moved one step closer (3.3 μm) to the sample surface, the above voltage cycle was repeated. Curve fitting analysis gives threshold field values in the range of 30–120 V per micron distance between the anode-cathode gap and turn-on field in the range of 22–72 V per micron for these defective diamond samples. This compares favorably with the high quality, p-type-doped diamond which requires a turn-on field of more than 110 V/μm and a threshold field of greater than 160 V/μm. A strong correlation between the threshold emission field and the FWHM of the diamond Raman peak is evident as shown in Fig. 4. In the figure, data from ion-implanted diamond samples are also included.[14]

It can be seen that for the field emission to occur, undoped diamond must exhibit a Raman peak width in a narrow range of about 7–11 cm^{-1}. Undoped diamond with the Raman peak width less than 7 cm^{-1} are generally too insulating to sustain a stable emission. On the other hand, when the peak width exceeds 11 cm^{-1}, the diamond Raman signature becomes so broad and weak compared to peaks originating from graphitic and amorphous carbon components

[This article is copyrighted as indicated in the article. Reuse of AIP content is subject to the terms at: http://scitation.aip.org/termsconditions. Downloaded to] IP: 129.22.67.107 On: Mon, 24 Nov 2014 20:31:38

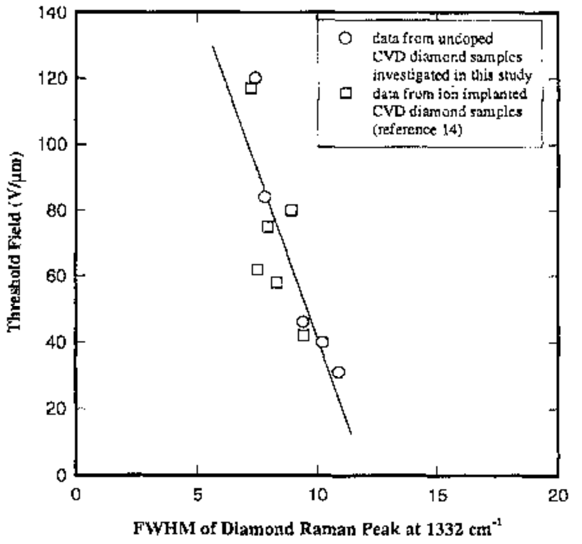

FIG. 4. A diagram showing the correlation between the diamond Raman peak width with the threshold field required to yield an emission current density of 10 mA/cm^2 for the undoped diamond.

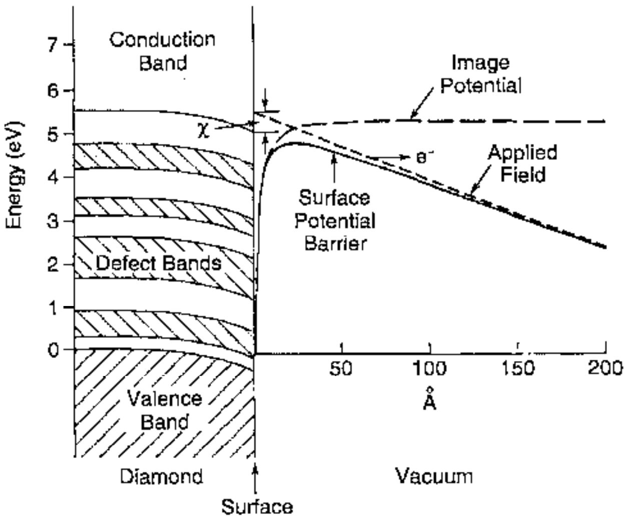

FIG. 6. A schematic energy band diagram showing the presence of defect-induced energy bands within the band gap of diamond. A positive electron affinity (χ) of 0.5 eV is assumed. The applied field is 150 V/μm which causes the band bending, and no surface states are shown. The position and width of the defect-induced bands are arbitrary for illustration purpose only.

that the deposits are usually considered as diamond-like or amorphous carbon films rather than genuine diamond. These films are reported to be capable of emitting electrons at relatively low fields,[6] but their emission stability and reliability have not be established, and they are not the subject of the present study. For the undoped diamond, the required threshold field for emission falls rapidly as the Raman peak width increases, from about 120 V/μm at the peak width of 7.4 cm^{-1} to about 30 V/μm at 10.9 cm^{-1}.

Such a strong correlation between the emission field and the width of the diamond Raman peak is also observed from the boron-doped, p-type semiconducting diamond as shown in Fig. 5. Samples with larger peak width or higher defect densities require lower fields for emission. The samples with the peak width in the range of 9.1–10.5 cm^{-1} (samples #10–12) require a threshold field in the range of 30–40 V/μm. This is almost five times lower than that required for a high quality p-type sample (sample #8) which has a peak width of 3.7 cm^{-1}. It is interesting to note that the p-type semiconductivity allows stable emission to be obtained from diamond exhibiting a Raman peak width as low as 3.7 cm^{-1}. If undoped, such high quality diamond would show arcing or breakdown behavior similar to samples #1 and 2.

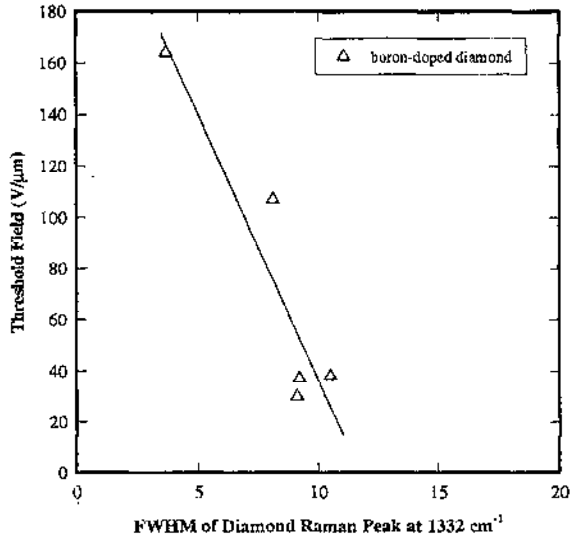

FIG. 5. A diagram showing the correlation between the diamond Raman peak width with the threshold field required to yield an emission current density of 10 mA/cm^2 for the boron-doped, p-type diamond.

There have been a number of reasons proposed that would relate defect density to field emission characteristics. For instance, emission from graphitic inclusions or enhanced conductivity along grain boundaries have been suggested.[3,4] However, both our sample growth technique and Raman measurements argue against any contribution from graphite. We believe it is more likely that the enhanced field emission originates from the defect-induced energy band(s) which are formed within the band gap of bulk diamond.[15,16] As schematically shown in Fig. 6, a series of defect-induced bands are assumed to form throughout the band gap because of the presence of a wide variety of structural defects. If these bands are wide enough or closely spaced, the electron hopping within the band(s) or excitation from the valence band could easily provide a steady flow of electrons to the surface (or surface states which are not identified in the diagram) to sustain stable emission of electrons into vacuum. The formation of these defect bands raises the Fermi level into the upper part of the band gap, and thus reduces the energy barrier that the electrons must tunnel through. However, the exact positions of these defect-induced energy bands can not be determined from the field emission measurements because of uncertainties in the local field enhancement and the emission area, both of which are impossible to quantify at present. Both theoretical calculations and experimental studies such as photoemission are needed to determine how the

[This article is copyrighted as indicated in the article. Reuse of AIP content is subject to the terms at: http://scitation.aip.org/termsconditions. Downloaded to] IP: 129.22.67.107 On: Mon, 24 Nov 2014 20:31:38

defects couple together to form conducting bands and to identify the exact energy levels from which the electrons originate.

IV. CONCLUSIONS

In summary, vacuum field emission measurements on CVD diamond samples with varying defect densities indicate that the electric field required for electron emission can be significantly reduced when the CVD diamond is properly grown so as to contain a substantial number of structural defects. A strong correlation is established between the required threshold field to generate sufficient emission current densities for display applications (>10 mA/cm^2) and the Raman peak width which is a measure of the defect densities in diamond. It is likely that the broad types of defects present in the diamond structure create additional energy bands within the band gap of diamond and thus contribute to the electron emission at low electric fields.

[1] F. J. Himpsel, J. A. Knapp, J. A. van Vechten, and D. E. Eastman, Phys. Rev. B **20**, 624 (1979).

[2] M. W. Geis, N. N. Efremow, J. D. Woodhouse, M. D. McAleese, M. Marchywka, D. G. Socker, and J. F. Hochedez, IEEE Electron Device Lett. **12**, 456 (1991).

[3] C. Wang, A. Garcia, D. C. Ingram, M. Lake, and M. E. Kordesch, Electronics Lett. **27**, 1459 (1991).

[4] N. S. Xu, Y. Tzeng, and R. V. Latham, J. Phys. D **26**, 1776 (1993).

[5] K. Okano, K. Hoshina, M. Iida, S. Koizumi, and T. Inuzuka, Appl. Phys. Lett. **64**, 2742 (1994).

[6] H. K. Schmidt, M. H. Clark, I. Yee, and N. Kumar, Abstract for the SID Manuf. Conference, 1994, p. 21.

[7] G. S. Gildenblat, S. A. Grot, and A. R. Badzian, Proc. IEEE **79**, 647 (1991).

[8] K. K. Das, in *Diamond Films and Coatings*, edited by R. F. Davis (Noyes, Park Ridge, NJ, 1993), p. 381.

[9] B. B. Pate, M. Oshima, J. A. Silberman, G. Rossi, I. Lindau, and W. E. Spicer, J. Vac. Sci. Technol. A **2**, 957 (1984).

[10] B. B. Pate, Surf. Sci. **165**, 83 (1986).

[11] W. A. Yarbrough and R. Messier, Sci. **247**, 688 (1990).

[12] J. He, P. H. Cutler, N. M. Miskovsky, T. E. Feuchtwang, T. E. Sullivan, and M. Chung, Surf. Sci. **246**, 348 (1991). Note that equation 63 in this reference makes the approximation that the tip radius is much smaller than the tip-to-anode spacing. This must be generalized if diamond particle sizes are similar to the spacing.

[13] R. H. Fowler and L. W. Nordheim, Proc. R. Soc. London A **119**, 173 (1928).

[14] W. Zhu, G. P. Kochanski, S. Jin, L. Seible, D. Jacobson, M. McCormack, and A. White (unpublished).

[15] G. B. Bachelet, G. A. Baraff, and M. Schluter, Phys. Rev. B **24**, 4736 (1981).

[16] W. V. M. Machado, J. Kintop, M. L. De Siqueira, and L. G. Ferreira, Phys. Rev. B **47**, 13219 (1993).

[17] R. J. Nemanich, J. T. Glass, G. Lucovsky, and R. E. Shroder, J. Vac. Sci. Technol. A **6**, 1783 (1988).

[This article is copyrighted as indicated in the article. Reuse of AIP content is subject to the terms at: http://scitation.aip.org/termsconditions. Downloaded to] IP: 129.22.67.107 On: Mon, 24 Nov 2014 20:31:38

IOPscience iopscience.iop.org

Home Search Collections Journals About Contact us My IOPscience

Defect emissions in ZnO nanostructures

This article has been downloaded from IOPscience. Please scroll down to see the full text article.

2007 Nanotechnology 18 095702

(http://iopscience.iop.org/0957-4484/18/9/095702)

View the table of contents for this issue, or go to the journal homepage for more

Download details:
IP Address: 65.39.15.37
The article was downloaded on 05/03/2013 at 07:32

Please note that terms and conditions apply.

IOP Publishing — Nanotechnology
Nanotechnology **18** (2007) 095702 (8pp) doi:10.1088/0957-4484/18/9/095702

Defect emissions in ZnO nanostructures

A B Djurišić[1], Y H Leung[2], K H Tam[1], Y F Hsu[1], L Ding[3], W K Ge[3], Y C Zhong[3], K S Wong[3], W K Chan[2], H L Tam[4], K W Cheah[4], W M Kwok[2] and D L Phillips[2]

[1] Department of Physics, The University of Hong Kong, Pokfulam Road, Hong Kong
[2] Department of Chemistry, The University of Hong Kong, Pokfulam Road, Hong Kong
[3] Department of Physics, The Hong Kong University of Science and Technology, Clear Water Bay, Hong Kong
[4] Department of Physics and Center of Advanced Luminescent Materials, The Hong Kong Baptist University, Kowloon Tong, Hong Kong

E-mail: dalek@hkusua.hku.hk (A B Djurišić)

Received 23 October 2006, in final form 28 November 2006
Published 24 January 2007
Online at stacks.iop.org/Nano/18/095702

Abstract
Defects in three different types of ZnO nanostructures before and after annealing under different conditions were studied. The annealing atmosphere and temperature were found to strongly affect the yellow and orange-red defect emissions, while green emission was not significantly affected by annealing. The defect emissions exhibited a strong dependence on the temperature and excitation wavelength, with some defect emissions observable only at low temperatures and for certain excitation wavelengths. The yellow emission in samples prepared by a hydrothermal method is likely due to the presence of OH groups, instead of the commonly assumed interstitial oxygen defect. The green and orange-red emissions are likely due to donor acceptor transitions involving defect complexes, which likely include zinc vacancy complexes in the case of orange-red emissions.

(Some figures in this article are in colour only in the electronic version)

1. Introduction

ZnO is a wide bandgap semiconductor which is of interest for a great variety of practical applications, including short-wavelength photonic devices [1]. Therefore, the optical properties of ZnO have been extensively studied [2–46]. At room temperature, ZnO typically exhibits one emission peak in the UV region due to the recombination of free excitons, and possibly one or more peaks in the visible spectral range which are attributed to defect emissions, but the origin of defect emissions is still not fully clear [1, 2]. In addition to experimental studies of the defect emissions, energy levels of different defects [18, 47, 48] and their formation energies [49–53] have been calculated. However, the origins of different defect emissions are still not fully understood, and controversial hypotheses have been proposed to explain the different defect emissions (violet, blue, green, yellow and orange-red).

It is known that different types of defects are responsible for green, yellow and orange-red emissions [21], but the chemical natures of the defects responsible for these emissions have not been conclusively established. The most commonly observed green defect emission [2, 8, 10–16, 18] is also the most controversial one, for which various hypotheses have been proposed. There is convincing evidence that the defects involved are located at the surface [2, 29, 30] based on the suppression of this emission by a surface coating [2] and results from polarized luminescence measurements [29], but their chemical nature requires further study.

Yellow and orange-red emissions have been less controversial. It has been proposed that these two emissions may involve similar deep levels but different initial states (conduction band and shallow donors) [22], and they were also found to exhibit different dependences on the excitation wavelength [9]. The yellow emission is commonly attributed to oxygen interstitial defects [3, 7], although some impurities such as Li may also play a role [7]. In addition to this common hypothesis, the possible presence of $Zn(OH)_2$ at the surface was identified as a possible reason for the weak UV and the strong visible (broad yellow and green) emission [31]. The orange-red emission centred at ~640–650 nm is also commonly attributed to the presence of excess oxygen in the

 © 2007 IOP Publishing Ltd Printed in the UK

samples [6, 19, 20], such as oxygen interstitial defects [35]. Other hypotheses include surface dislocations [34] and zinc interstitials [23].

The proposed explanations for the different visible emissions in ZnO are often contradictory, with different defect types proposed to explain the same emission or the same defect types proposed to explain emission in different spectral ranges. In order to study the origins of the different defect emissions in ZnO, we investigated in detail three types of nanostructures, which emit green, yellow and orange-red defect emissions. For all three morphologies (shells, needles and rods), the size is sufficiently large so that no significant size effects are expected, other than small variations in the UV to visible emission ratios. The nanostructures have been chosen in such a manner to be able to separate the three different defect emissions (green, yellow and orange-red).

2. Experimental details

The needles [6], rods [7] and hollow shells [8] were prepared according to previously reported procedures. The rods have been synthesized by a hydrothermal method [7], while the nanoneedles and hollow shells have been prepared by thermal evaporation in a tube furnace [6, 8]. Their morphology was investigated by field emission scanning electron microscopy (SEM) using a LEO 1530 field emission SEM. Annealing was performed for 30 min in a tube furnace at temperatures 200, 400 and 600 °C in air (atmospheric pressure, relative humidity ∼55–60%), argon, nitrogen and forming gas (90% N_2/10% H_2). For annealing in a gas flow, the gas flow rate was 0.1 Lpm, and the pressure was ∼1 Torr. For variable temperature photoluminescence measurements, samples were mounted at the cold finger and placed in a liquid He closed-cycle cryostat (APD Cryogenics, Inc. model HC-2). The excitation source was a HeCd laser (325 nm) and Ti:sapphire laser (390 nm). The spectra were dispersed by a spectrometer SPEX 500M and recorded by a photomultiplier tube R943. A frequency doubled femtosecond Ti:sapphire laser tuned to 355 nm was used as an excitation source for PL and time resolved PL measurements in the UV range. A Hamamatsu streak camera (model C4334) coupled to a spectrometer was used for the time resolved PL measurements in the picosecond range. For obtaining better time resolution for samples with fast decay, measurements using the Kerr gated fluorescence technique were performed, which has been described in detail elsewhere [54]. For the time resolved PL measurements of defect emission, a HP 54522A oscilloscope was used to capture the decay curve following an excitation pulse from a nitrogen laser (337 nm). It should be noted that the ratio of the UV and defect emission measured for different excitation sources may vary due to differences in excitation intensity, as reported previously [2], but the positions of the defect emissions exhibit no dependence on the excitation power. XPS spectra were obtained using PAL 102 XPS and the position of the carbon C 1s peak (285.0 eV) was taken as a reference. Sputtering to remove surface contamination was not performed since it typically leads to considerable broadening of the peaks due to sputter induced damage, which would not be meaningful for the study of the defect levels.

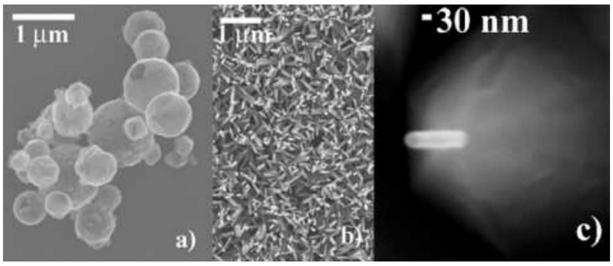

Figure 1. Representative SEM images of the ZnO nanorods: (a) shells, (b) rods, (c) needles.

3. Results and discussion

Figure 1 shows the representative SEM images of the investigated nanostructures. It can be observed that the shells (figure 1(a)) have different sizes with diameters in the range 300 nm–2 μm, and there are typically several hundreds of shells per laser spot area. Nanorods are ∼250 nm long and have diameters in the range 30–50 nm, as shown in figure 1(b). The nanorods are densely packed, with several thousands of nanorods per laser spot area. The needles have a density similar to the shells and consist of a thin (20–60 nm) needle on top of a wide micron-sized base, as shown in figure 1(c). The annealing does not significantly affect the morphology of the nanostructures, except in the case of forming gas annealing at 600 °C for nanorods, where some surface damage of the nanorods occurs, in agreement with previous reports on forming gas annealing of hydrothermally grown nanorods [3].

The low temperature PL spectra of the three different types of nanostructures are shown in figure 2. It can be observed that the emission is most intense in the case of needles, followed by shells, and then rods. In addition to the lowest emission intensity, the rods also exhibit the highest FWHM (full width half-maximum) of the peak, indicating large concentration of defects in the samples. In the case of shells, the dominant peak is located at 3.364 eV (full width half-maximum FWHM = 10 meV) and two shoulders at 3.368 and 3.377 eV can also be observed. The highest energy shoulder is due to the free exciton emission [1, 4, 5], while the features observed at 3.364 and 3.368 eV correspond to donor bound excitons [1, 4, 5]. Two other peaks can be observed at ∼3.300 and 3.320 eV, which likely correspond to an LO phonon replica of the exciton peaks (∼70 meV below the exciton peak) and excitons bound to structural defects [1, 4]. In the case of ZnO nanorods, the PL spectrum at 12 K is dominated by a broad peak at 3.368 eV (FWHM = 30 meV) which can be attributed to donor bound excitons [1, 4]. No free exciton emission can be observed and there is another shoulder at ∼3.333 eV which can likely be attributed to excitons bound to structural defects [4]. Considering the short decay times of UV luminescence in both shells and nanorods [55], indicating large defect concentrations, the presence of excitons bound to structural defects is not surprising. The low temperature PL spectra of needles, on the other hand, exhibit a very strong and narrow (FWHM ∼ 3 meV) bound exciton peak centred at 3.357 eV. The origin of this line is somewhat controversial, since it has been assigned both to donor bound excitons (indium donor) [4] and acceptor bound excitons [1, 5]. In

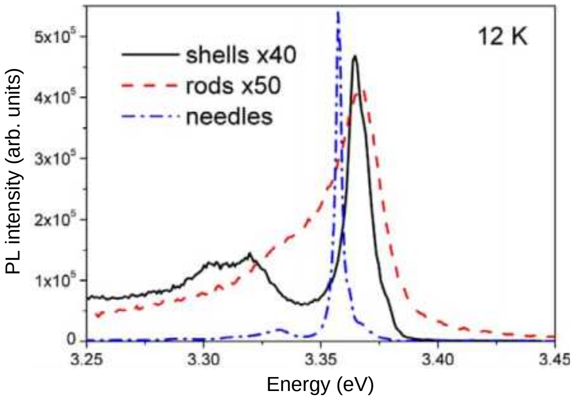

Figure 2. Excitonic emission spectra of different ZnO nanostructures at 12 K.

addition to this line, small shoulders can be observed at 3.362 and 3.367 eV, which likely correspond to donor bound exciton emissions [1, 4, 5]. In addition, a feature can be observed around ∼3.332 eV, which can be either due to a two-electron satellite of the neutral donor bound exciton [1] or an exciton bound to structural defects [1, 4, 33].

The temperature dependences of defect emissions for two different excitation wavelengths are shown in figure 3. The use of different excitation wavelengths is useful in examining the luminescence spectra of materials containing multiple defect levels, since different defect centres would exhibit different behaviour for different excitation wavelengths. Excitation below the bandgap of ZnO allows us to directly probe radiative relaxation of defect levels. In the case of shells (figures 3(a) and (b)), no significant shift of the emission peak can be observed with temperature, which possibly indicates that this emission originates from a donor–acceptor transition. Common characteristics of donor–acceptor transitions include nearly Gaussian broad peaks, small decrease in half-widths with decrease in temperature, and moderate increase in intensity (by a factor of ten or less) with decrease of temperature [56]. In addition, the donor–acceptor pair emission is expected to show a blue shift with increasing excitation energy, which has been observed both for shallow donor–acceptor pair emissions [45, 46], as well as deep level donor–acceptor emissions [56]. However, no significant peak shift for different excitation intensities is observed for the green emission from the shells, as shown in the inset of figure 4(a). However, it should be noted the peak shifts for powers in the range 1–100 W cm^{-2} are small for defect concentrations below $\sim 5 \times 10^{18}$ cm^{-3} [56]. Therefore, although no blue shift with increasing excitation intensity is observed, based on the temperature dependence of this emission it is likely a donor–acceptor emission but the deep level concentration is not very high.

In the case of rods (figures 3(c) and (d)), for both excitation wavelengths we can observe blue shifts with increasing temperature. The peak position for 325 nm is red-shifted compared to that obtained for 390 nm excitation, but in both cases similar dependence of emission intensity on temperature is obtained, with emission intensity remaining the same at low temperatures, and then decreasing after ∼150–180 K. On the other hand, needles exhibit entirely

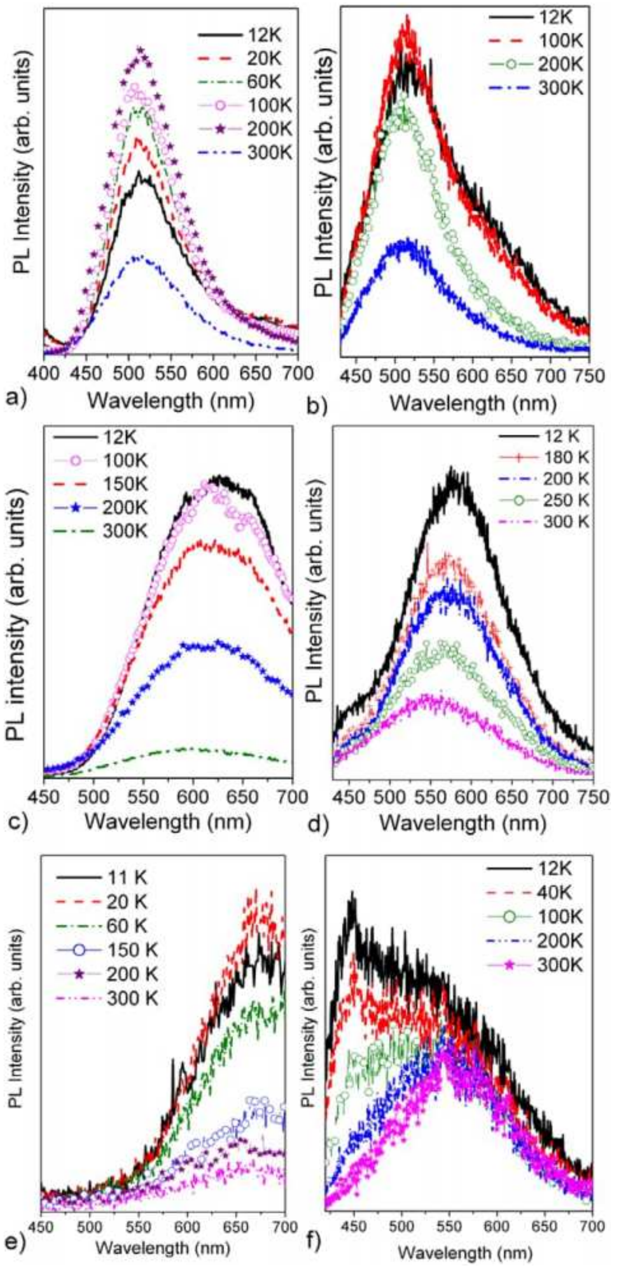

Figure 3. Variable temperature PL spectra (defect emission) of different ZnO nanostructures excited at 325 and 390 nm: (a), (b) shells, ((c), (d)) rods and ((e), (f)) needles.

different behaviour, as shown in figures 3(e) and (f). Under 325 nm excitation, orange-red emission, whose intensity decreases with temperature, is observed. Under 390 nm excitation, two emission peaks, one blue and one green, are found at low temperatures. The emission of the blue peak decreases with increasing temperature and disappears at ∼200 K, while that of the green peak remains the same. It should be noted that similar behaviour has been previously reported for the blue and yellow defect luminescence in GaN [57–59], where quenching of the blue band has been observed at ∼200 K, while the intensity of the yellow band remained nearly unchanged. The different PL bands were attributed to multiple negatively charged defects, but

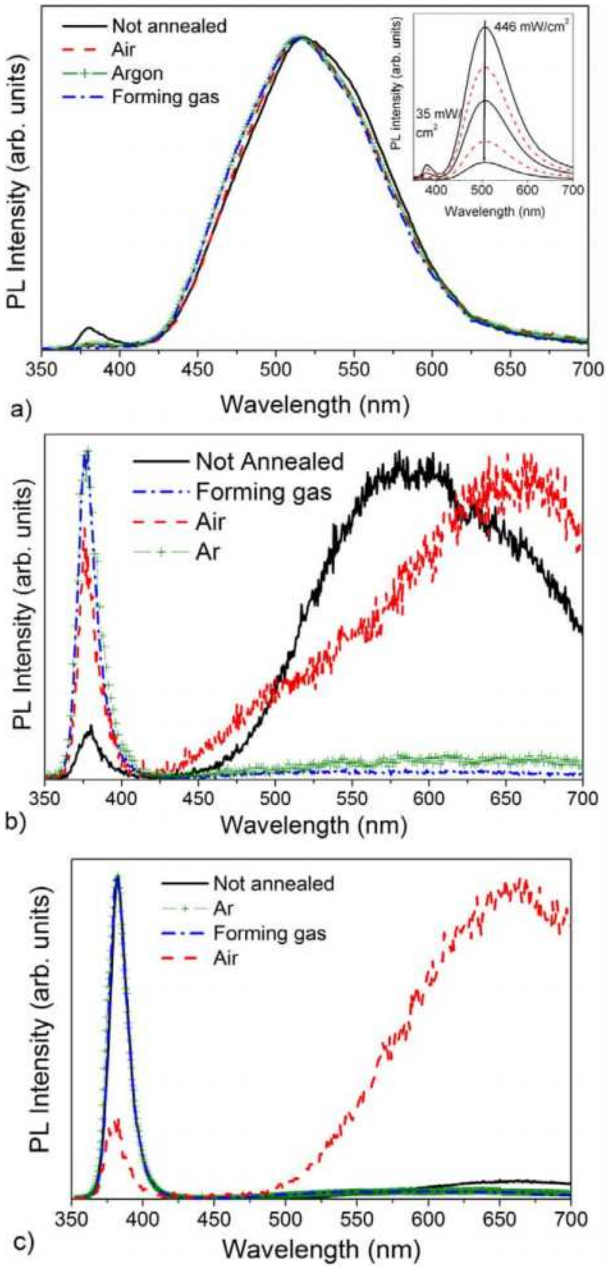

Figure 4. Effect of different annealing atmospheres on the room temperature photoluminescence (normalized spectra) of ZnO nanostructures annealed at 600 °C: (a) shells, (b) rods and (c) needles. Excitation wavelength was 325 nm. The inset in (a) shows the PL spectra of as-grown shells for different excitation powers.

the exact nature of the acceptors responsible has not been established [59].

In order to obtain more information on the behaviour of the different defects causing the visible emission, annealing of the samples under different conditions was performed. The normalized PL spectra before and after annealing in different environments at 600 °C are shown in figure 4. It can be observed that the green defect emission from the shells (figure 4(a)) did not exhibit significant changes with annealing in different environments, while reduction in UV emission intensity is observed after annealing. Green emission has been

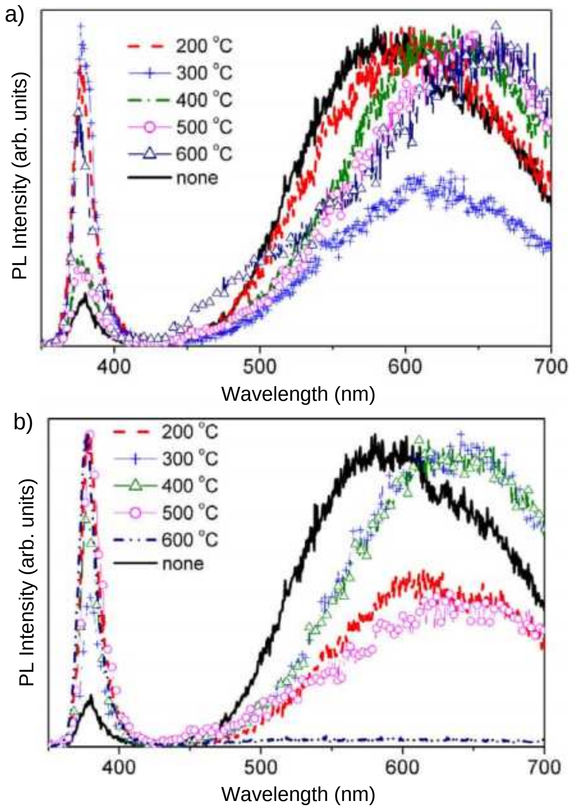

Figure 5. Effect of annealing temperature on the room temperature PL (normalized spectra) from ZnO nanorods annealed in (a) air or (b) forming gas. Excitation wavelength was 325 nm.

previously attributed to surface defects [2, 29, 30], as well as the defects just below the crystallite surface [20], and the importance of band bending at grain boundaries has also been recognized [16]. Band bending will result in creation of a depletion region at the grain boundaries, which will affect the ionization state of the defects within the depletion region [16]. Since the shells have a large surface area and lots of grain boundaries, the green emission likely originates from donor–acceptor transitions, where donor and/or acceptor defects are located at surfaces or grain boundaries. The exact chemical nature of those defects, however, is difficult to establish since no significant changes with annealing are observed.

On the other hand, the defect emission from the rods (figure 4(b)) exhibited a strong dependence on the annealing atmosphere. The defect emission was significantly reduced after annealing in argon and forming gas, while a strong orange-red instead of a yellow emission was observed after annealing in air. In the case of the needles (figure 4(c)), the weak orange-red emission was reduced after annealing in argon and forming gas, and enhanced by annealing in air. Since the rods exhibited the most dramatic change of the defect emission for different annealing conditions, a detailed study of the PL spectra dependence on annealing temperature was performed. The results obtained for annealing in air and forming gas are shown in figure 5. The UV emission increases with annealing at 200 °C, regardless of the annealing atmosphere. For annealing in air (figure 5(a)), the emission maximum red-shifts with increasing annealing temperature.

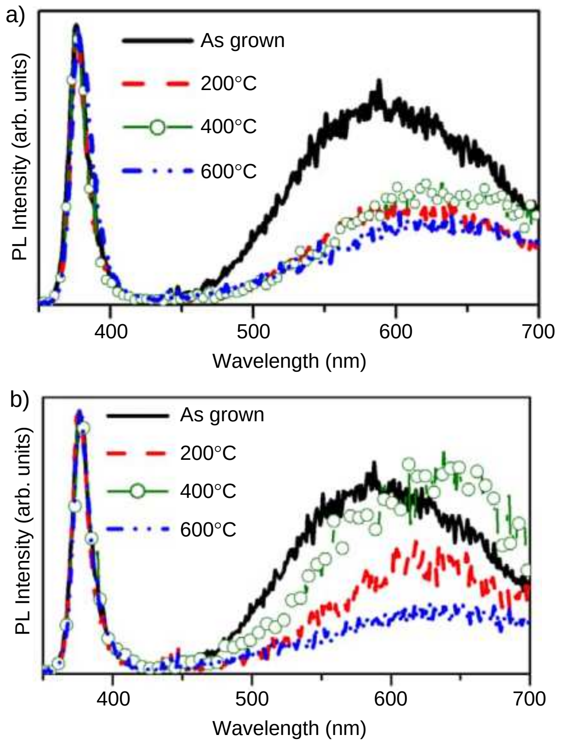

Figure 6. Normalized room temperature PL spectra of ZnO nanorods annealed in oxygen at (a) 1 Torr and (b) 100 Torr. Excitation wavelength was 325 nm.

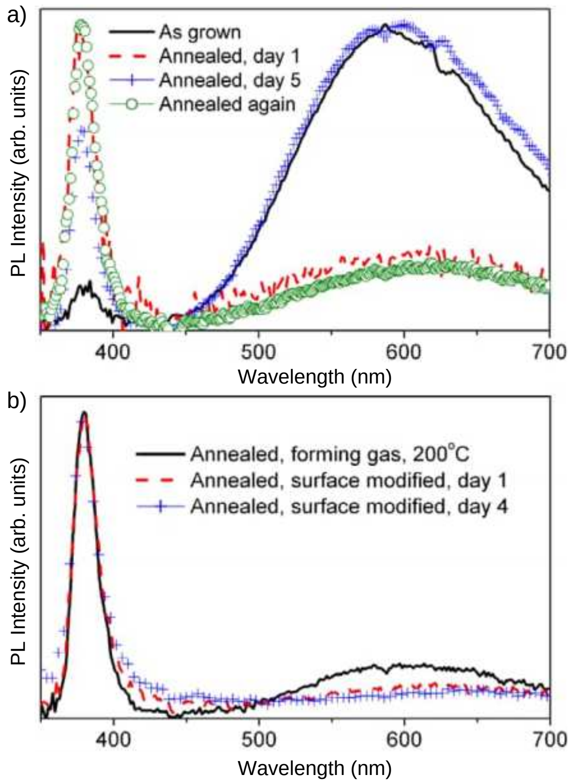

Figure 7. Normalized room temperature PL spectra of ZnO nanorods for different annealing conditions and different air exposure times: (a) without silane surfactant, (b) with silane surfactant. Excitation wavelength was 325 nm.

The UV to visible emission ratio is strongly dependent on the annealing temperature for both air and forming gas annealing. The enhancement obtained for the UV emission after annealing at 200 °C is probably related to the desorption of hydroxyl groups, since the desorption rate of the hydroxyl groups peaks at ∼150 °C, while hydrogen predominantly desorbs at ∼420 °C [32]. Since hydrogen can passivate the defects and consequently enhance the UV emission and reduce the visible emission [32], the worsening of the UV to visible emission ratio for annealing at ∼400 °C is likely related to the release of residual hydrogen. It should also be noted that the yellow emission is present only in as-grown nanorods, and it disappears with annealing. The yellow emission (centred at ∼2.15 eV or ∼576 nm) was previously attributed to surface capping by zinc hydroxide [31]. Since water and hydroxyl groups can be desorbed by annealing at 200 °C, the yellow emission is likely due to the presence of $Zn(OH)_2$ or hydroxyl groups, instead of the commonly assumed interstitial oxygen. After annealing at 200 °C only the red component of the emission can be observed in all atmospheres (forming gas, argon, nitrogen) except for air where the red shift occurs more slowly. This is likely due to a slower desorption at atmospheric pressure and in the presence of ambient humidity. To verify this hypothesis, the nanorods were annealed in oxygen gas flow at pressures of 1 and 100 Torr. The results obtained are shown in figure 6. It can be observed that the yellow emission red-shifts with increasing annealing temperature, confirming the assignment of the yellow emission to the presence of hydroxyl groups or $Zn(OH)_2$ instead of commonly assumed interstitial oxygen. Since the orange-red emission is obviously enhanced after annealing under oxygen-rich conditions, it is likely due to defects related to excess oxygen. It should be noted that this explanation for yellow defect emission is plausible for the samples fabricated by the hydrothermal method, and it does not necessarily apply to the ZnO samples fabricated by other methods where the presence of the hydroxyl groups or water at the surface is less likely.

To confirm the influence of the OH group's adsorption on the defect emission from ZnO nanorods, we have monitored the emission after annealing in forming gas at 200 °C for different times of storage in air, as shown in figure 7(a). The defect emission increases with time of air exposure and reaches the level of that in as-grown nanorods in several days. Repeated annealing at 200 °C in forming gas results in repeated reduction of this emission. On the other hand, annealed nanorods with surface coated with silane surfactant show no significant change of the emission spectra with time of air exposure, as shown in figure 7(b). Coating of the surface with silane surfactant was performed by dipping the annealed substrates into 3-aminopropyltrimethoxysilane solution, followed by rinsing in toluene to remove excess silane and drying. It can be observed that silane coating further reduces the weak orange emission and the yellow emission does not appear since OH group surface adsorption is prevented by the presence of the surfactant. If surfactant is added to as-grown nanorods which already contain OH groups on the surface, only red shift of the emission occurs but there is no reduction in its intensity, in agreement with our previous

Table 1. XPS results for different nanostructures before and after annealing. The areas of XPS peaks have been adjusted by the sensitivity factors, 0.733 for oxygen and 2.768 for Zn.

Nanostructure	$O_1/O_1 + O_2$	O_1/Zn
Shells, as-grown	0.42	0.43
Shells, forming gas, 600 °C	0.33	0.42
Shells, air, 600 °C	0.33	0.43
Rods, as-grown	0.22	0.48
Rods, forming gas, 600 °C	0.36	0.58
Rods, air, 600 °C	0.02	—
Needles, as-grown	0.39	0.73
Needles, forming gas, 600 °C	0.20	0.40
Needles, air, 600 °C	0.21	0.53

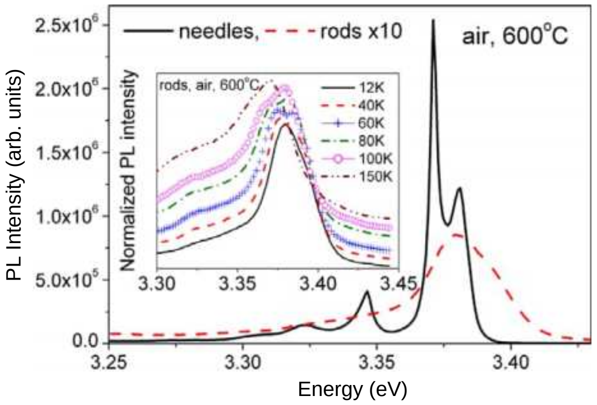

Figure 8. Excitonic emission spectra at 12 K of ZnO needles and rods after annealing in air at 600 °C. Excitation wavelength was 325 nm. The inset shows the temperature dependence of the excitonic emission spectra of the nanorods.

result [7]. Thus, we can unambiguously assign the yellow emission in hydrothermally grown nanorods to the presence of OH groups on the surface. OH groups can also occur after prolonged storage of nanorod samples in air. However, other investigated morphologies do not show pronounced changes in the emission spectra when annealed at 200 °C, indicating no major role of the presence of OH groups on the surface. This is likely because of different water adsorption properties of different surfaces of the ZnO crystal [60, 61].

To further study the effects of annealing on PL, time resolved PL measurements have been performed. In the UV range, all the samples exhibit biexponential emission, with decay times of 20 ps and 87 ps for needles [6], 10 ps and 37 ps for shells, and 7 ps and 44 ps for rods [55]. Annealing in all cases was found to affect only the slow component of the decay, while the faster one remains unchanged. Annealing was also found to affect the decay times of the defect emissions, but not as significantly as in the case of excitonic emission. The decay times of the green, yellow and orange-red emissions measured for different nanostructures were similar, with one dominant component in the 6–10 ns range and one low magnitude contribution with a slower decay, typically in the 30–100 ns range. This is different from a previous report on the very different decay times observed for the yellow and red emissions in ZnO [22], which is likely due to the different origin of the defect responsible for yellow emission in our samples. The effects of annealing on the sample composition were also examined, and obtained results from XPS measurements are summarized in table 1. The O 1s spectra in all cases could be fitted with two peaks [62], labeled O1 (lower energy) and O2 (higher energy), while the peak at ~533 eV attributed to water [63, 64] was not observed. The O1 peak, located at 529–530.7 eV [62–64], can be attributed to oxygen ions in a wurtzite ZnO structure. The O2 peak, at 531–532 eV [62–64], is typically assigned to loosely bound oxygen on the surface, such as OH groups [63, 64] or O^{-2} ions in oxygen-deficient regions [62]. The needles have the highest O_1/Zn ratio, in agreement with excellent PL properties. Also, the annealing does not have a significant effect on the O_1/Zn ratio in shells, in agreement with the lack of change in defect emission. However, there is no simple relationship between the sample composition and the defect emission. For example, needles annealed in forming gas have both lower defect emission intensity and lower O_1/Zn ratio than as-grown needles.

To obtain more information about orange-red emission, we have done a comparative study of two types of nanostructures with similar room temperature PL spectra with prominent orange-red emission, namely rods and needles annealed in air at 600 °C. The comparison between excitonic emissions at 12 K for these two samples is shown in figure 8. For needles, the low temperature spectrum is dominated by the donor bound exciton peak at 3.371 eV [4], with another strong peak found at 3.381 eV (spectral range where free exciton and polariton peaks are found [1]) and a weak peak at 3.346 eV, which was previously associated with Zn vacancies [33]. For the rods, the excitonic peak is now centred at 3.379 eV, which is also in the spectral region where free exciton emission is expected to occur [1]. However, the peak remains very broad (FWHM = 30 meV) indicating that rods have inferior crystal quality compared to the needles. From the temperature dependence of this emission (shown in the inset of figure 8), it can be observed that another, higher energy peak appears at ~40 K, which likely corresponds to free exciton emission based on its temperature dependence. Due to the large emission peak width, it is difficult to assign an exact origin to different components of the UV emission since individual transitions cannot be resolved. In both needles and rods blue, green and red components of visible emission can be clearly observed for excitation at 325 nm for both types of nanostructures, as shown in figure 9. However, clear differences are observed in temperature dependences of the defect emissions when the excitation wavelength is changed to 390 nm. This indicates that, in spite of similarities of the defect emissions at room temperature between these two samples, they do not contain the same types and concentrations of defects.

As for the origin of the orange-red emission, its enhancement upon annealing in air apparently indicates defects associated with excess oxygen. However, XPS results for needles annealed in air indicate reduction in the O_1/Zn ratio. The samples exhibiting prominent orange-red defect emission commonly also exhibit a bound exciton peak at 3.346 eV, which was previously attributed to zinc vacancy [33]. However, from the energy level calculations of the defects no obvious candidate for orange-red emission can be identified [18, 47]. In addition, since common defects,

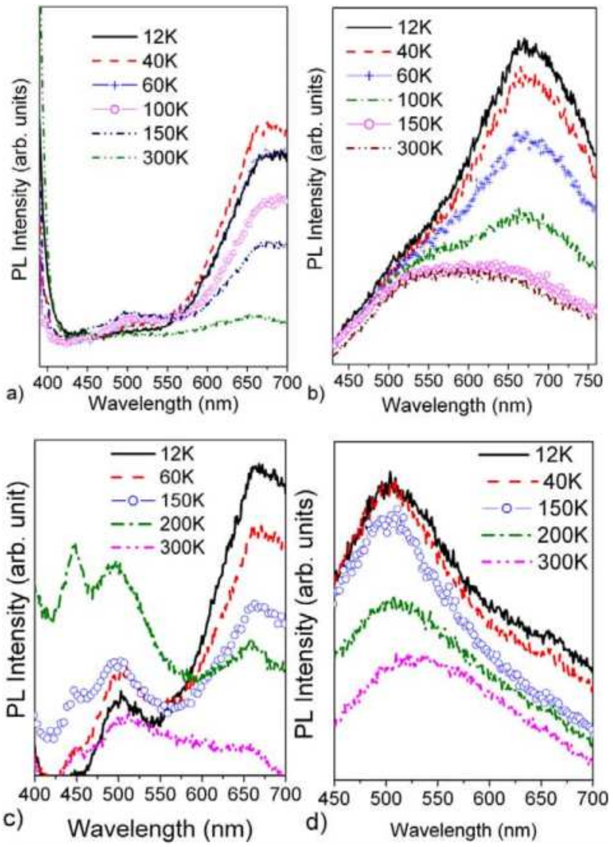

Figure 9. Variable temperature PL spectra (defect emission) of samples annealed in air at 600 °C: (a) needles, excited at 325 nm, (b) needles, excited at 390 nm, (c) rods, excited at 325 nm, (d) rods, excited at 390 nm.

such as V_{Zn} [65] and Zn_i [49] are expected to be mobile at relatively low temperatures, the persistence of the defect emissions after annealing at 600 °C supports the hypothesis that defect complexes are responsible for the visible emission in ZnO.

4. Conclusions

ZnO nanostructures exhibiting different types of defect emissions were studied before and after annealing in different environments and at different temperatures. It was found that the yellow emission in nanorods prepared by a hydrothermal method can be attributed to $Zn(OH)_2$ or OH groups, instead of the commonly assumed interstitial oxygen defect. The green emission likely originated from surface or grain-boundary defects. The red emission originated from defects related to excess oxygen, possibly involving zinc vacancy complexes.

Acknowledgments

The work reported in this paper has been supported by the Research Grants Council of The Hong Kong Special Administrative Region, China (Project Numbers HKU 7008/04P, 7010/05P, 7019/04P, and HKU 1/01C). Financial support from the Strategic Research Theme, University Development Fund, and Seed Funding Grant (administrated by The University of Hong Kong) are also acknowledged.

References

[1] Özgür U, Alivov Ya I, Liu C, Teke A, Reshchikov M A, Doğan S, Avrutin V, Cho S-J and Morkoç H 2005 *J. Appl. Phys.* **98** 041301
[2] Djurišić A B and Leung Y H 2006 *Small* **2** 944
[3] Greene L E, Law M, Goldberger J, Kim F, Johnson J C, Zhang Y, Saykally R J and Yang P 2003 *Angew. Chem. Int. Edn* **42** 3031
[4] Meyer B K *et al* 2004 *Phys. Status Solidi* b **241** 231
[5] Teke A, Özgür U, Doğan S, Gu X, Morkoç H, Nemeth B, Nause J and Everitt H O 2004 *Phys. Rev.* B **70** 195207
[6] Kwok W M, Leung Y H, Djurišić A B, Chan W K and Phillips D L 2005 *Appl. Phys. Lett.* **87** 093108
[7] Li D, Leung Y H, Djurišić A B, Liu Z T, Xie M H, Shi S L, Xu S J and Chan W K 2004 *Appl. Phys. Lett.* **85** 1601
[8] Leung Y H, Tam K H, Djurišić A B, Xie M H, Chan W K, Ding L and Ge W K 2005 *J. Cryst. Growth* **283** 134
[9] Djurišić A B, Leung Y H, Tam K H, Ding L, Ge W K, Chen H Y and Gwo S 2006 *Appl. Phys. Lett.* **88** 103107
[10] Garces N Y, Wang L, Bai L, Giles N C, Halliburton L E and Cantwell G 2002 *Appl. Phys. Lett.* **81** 622
[11] Reynolds D C, Look D C and Jogai B 2001 *J. Appl. Phys.* **89** 6189
[12] van Dijken A, Meulenkamp E, Vanmaekelbergh D and Meijerink A 2000 *J. Phys. Chem.* B **104** 1715
[13] van Dijken A, Meulenkamp E, Vanmaekelbergh D and Meijerink A 2000 *J. Lumin.* **90** 123
[14] Heo Y W, Norton D P and Pearton S J 2005 *J. Appl. Phys.* **98** 073502
[15] Zhao Q X, Klason P, Willander M, Zhong H M, Lu W and Yang J H 2005 *Appl. Phys. Lett.* **87** 211912
[16] Vanheusden K, Seager C H, Warren W L, Tallant D R and Voigt J A 1996 *Appl. Phys. Lett.* **68** 403
[17] Wang Y G, Lau S P, Zhang X H, Lee H W, Yu S F, Tay B K and Hng H H 2003 *Chem. Phys. Lett.* **375** 113
[18] Lin B, Fu Z and Jia Y 2001 *Appl. Phys. Lett.* **79** 943
[19] Cross R B M, De Souza M M and Sankara Narayanan E M 2005 *Nanotechnology* **16** 2188
[20] Studenikin S A, Golego N and Cocivera M 1998 *J. Appl. Phys.* **84** 2287
[21] Ong H C and Du G T 2004 *J. Cryst. Growth* **265** 471
[22] Lauer R B 1973 *J. Phys. Chem. Solids* **34** 249
[23] Gomi M, Oohira N, Ozaki K and Koyano M 2003 *Japan. J. Appl. Phys.* **42** 481
[24] Lin C C, Chen H P, Liao H C and Chen S Y 2005 *Appl. Phys. Lett.* **86** 183103
[25] Liu M, Kitai A H and Mascher P 1992 *J. Lumin.* **54** 35
[26] Huang M H, Mao S, Feick H, Yan H, Wu Y, Kind H, Weber E, Russo R and Yang P 2001 *Science* **292** 1897
[27] Meng X Q, Shen D Z, Zhang J Y, Zhao D X, Lu Y M, Dong L, Zhang Z Z, Liu Y C and Fan X M 2005 *Solid State Commun.* **135** 179
[28] Wang Y G, Lau S P, Zhang X H, Lee H W, Yu S F, Tay B K and Hng H H 2003 *Chem. Phys. Lett.* **375** 113
[29] Hsu N E, Hung W K and Chen Y F 2004 *J. Appl. Phys.* **96** 4671
[30] Shalish I, Temkin H and Narayanamurti V 2004 *Phys. Rev.* B **69** 245401
[31] Zhou H, Alves H, Hofmann D M, Kriegseis W, Meyer B K, Kaczmarczyk G and Hoffmann A 2002 *Appl. Phys. Lett.* **80** 210
[32] Xie R, Sekiguchi T, Ishigaki T, Ohashi N, Li D, Yang D, Liu B and Bando Y 2006 *Appl. Phys. Lett.* **88** 134103
[33] Zubiaga A, Garcia J A, Plazola F, Tuomisto F, Saarinen K, Zuñiga Pérez J and Muñoz-Sanjosé V 2006 *J. Appl. Phys.* **99** 053516

[34] Fan H J, Scholz R, Kolb F M, Zacharias M, Gösele U, Heyroth F, Eisenschmidt C, Hempel T and Christen J 2004 *Appl. Phys.* A **79** 1895
[35] Liu X, Wu X, Cao H and Chang R P H 2004 *J. Appl. Phys.* **95** 3141
[36] Zhao D, Andreazza C, Andreazza P, Ma J, Liu Y and Shen D 2004 *Chem. Phys. Lett.* **399** 522
[37] Yang Q, Tang K, Zuo J and Qian Y 2004 *Appl. Phys.* A **79** 1847
[38] Yu W, Li X and Gao X 2005 *Cryst. Growth Design* **5** 151
[39] Wu R, Yang Y, Cong S, Wu Z, Xie C, Usui H, Kawaguchi K and Koshizaki N 2005 *Chem. Phys. Lett.* **406** 457
[40] Weng L, Zhang X, Zhao S, Zhou G, Zhou Y and Qi J 2005 *Appl. Phys. Lett.* **86** 024108
[41] Chen Z, Wu N, Shan Z, Zhao M, Li S, Jiang C B, Chyu M K and Mao S X 2005 *Scr. Mater.* **52** 63
[42] Lin B J, Im S and Lee S Y 2000 *Thin Solid Films* **366** 107
[43] Reynolds D C, Look D C, Jogai B and Morkoç H 1997 *Solid State Commun.* **101** 643
[44] Zhong H, Chen X, Sun L Z, Lu W, Zhao Q X and Willander M 2006 *Chem. Phys. Lett.* **421** 309
[45] Zhang B P, Binh N T, Segawa Y, Wakatsuki K and Usami N 2003 *Appl. Phys. Lett.* **83** 1635
[46] Zhang B P, Binh N T, Wakatsuki K, Segawa Y, Kashiwaba Y and Haga K 2004 *Nanotechnology* **15** S382
[47] Xu P S, Sun Y M, Shi C S, Xu F Q and Pan H B 2003 *Nucl. Instrum. Methods* B **199** 286
[48] Lima S A M, Sigoli F A, Jafelicci M Jr and Davolos M R 2001 *Int. J. Inorg. Mater.* **3** 749
[49] Janotti A and Van de Walle C G 2006 *J. Cryst. Growth* **287** 58
[50] Zhao J L, Zhang W, Li X M, Feng J W and Shi X 2006 *J. Phys.: Condens. Matter* **18** 1495
[51] Kohan A F, Ceder G, Morgan D and van de Walle C G 2000 *Phys. Rev.* B **61** 15109
[52] Zhang S B, Wei S H and Zunger A 2001 *Phys. Rev.* B **63** 075205
[53] Oba F, Nishitani S R, Isotani S, Adachi H and Tanaka I 2001 *J. Appl. Phys.* **90** 824
[54] Ma C, Kwok W M, Chan W S, Zuo P, Kan J T W, Toy P H and Phillips D L 2005 *J. Am. Chem. Soc.* **127** 1463
[55] Kwok W M, Djurišić A B, Leung Y H, Chan W K and Phillips D L 2005 *Appl. Phys. Lett.* **87** 223111
[56] Kaufmann U, Kunzer M, Obloh H, Maier M, Manz Ch, Ramakrishnan A and Santic B 1999 *Phys. Rev.* B **59** 5561
[57] Reshchikov M A and Morkoç H 2005 *J. Appl. Phys.* **97** 061301
[58] Reshchikov M A, Moon T, Gu X, Nemeth B, Nause J and Morkoç H 2006 *Physica* B **377** 715
[59] Reshchikov M A and Korotkov R Y 2001 *Phys. Rev.* B **64** 115205
[60] Fryar J, McGlynn E, Henry M O, Cafolla A A and Hanson C J 2004 *Nanotechnology* **15** 1797
[61] Meyer B, Rabaa H and Marx D 2006 *Phys. Chem. Chem. Phys.* **8** 1513
[62] Ye J D *et al* 2005 *Appl. Phys.* A **81** 809
[63] Ogata K, Komuro T, Hama K, Koike K, Sasa S, Inoue M and Yano M 2004 *Phys. Status Solidi* b **241** 616
[64] Coppa B, Davis R F and Nemanich R J 2003 *Appl. Phys. Lett.* **82** 400
[65] Tuomisto F, Saarinen K, Look D C and Farlow G C 2005 *Phys. Rev.* B **72** 085206

Check for updates

ChemSusChem

Research Article
doi.org/10.1002/cssc.202201879

Chemistry Europe
European Chemical Societies Publishing

www.chemsuschem.org

$(La_{1-x}M_x)_2(Nb_{0.45}Yb_{0.55})_2O_{7-\delta}$ (M=Ca, Sr, Ba) Ionic Conductors Promoted by Foreign/Domestic Dual Acceptor-Doping Strategies

Xiaorong Zhang,[a] Lulu Jiang,[a] Shihang Guo,[a] and Donglin Han*[a, b, c, d]

In this work, a class of ionic conductor $(La_{1-x}M_x)_2(Nb_{0.45}Yb_{0.55})_2O_{7-\delta}$ (M=Ca, Sr, and Ba) with a cubic pyrochlore structure was reported. Two strategies were adopted to increase the concentration of oxygen vacancies favoring the hydration reaction to introduce protons. One was increasing the cation ratio between Yb and Nb over unity, the other was doping divalent alkaline earth elements to replace trivalent La. Proton conduction was evidenced by confirming the proton incorporation and H/D isotope effect in electrical conductivity. Doping Ca, Sr, and Ba further promoted the proton conduction. The results of crystal structure refinement indicated that the extrinsically introduced oxygen vacancies by the two strategies were accommodated in the tetrahedra (48*f*) containing two La and two Yb/Nb cations, while the tetrahedra containing four La cations (8*a*) were fully occupied by oxide ions. A discussion was thereby performed, leading to the suggestion that not all the tetrahedra in the cubic pyrochlore structure of $(La_{1-x}M_x)_2(Nb_{0.45}Yb_{0.55})_2O_{7-\delta}$ helped in incorporating and conducting protons, and only the oxygen vacancies surrounded by four Y cations (48*f* site) or two La and two Y cations (8*b* site) were hydratable. It is thereby suggested that to enhance the proton conduction in pyrochlore oxides, an effective strategy might be tuning the ability of hydration or protonation of the tetrahedra to increase the proton concentration and expand the route for proton conduction.

Introduction

Stemming from the discovery of proton conduction in acceptor-doped $SrCeO_3$ in 1980s by Iwahara et al.,[1,2] proton conducting oxide ceramics have been developed for more than forty years and are attracting increasing attention due to their promising application as electrolytes in various intermediate temperature (450–700 °C) solid-state electrochemical devices, including fuel cells,[3–8] electrolysis cells,[9–12] hydrogen pumps,[13,14] and membrane reactors.[15–17] The conduction of protons in the oxide ceramics may follow a vehicle mechanism, asking for protons moving along with oxide ions (e.g., as hydroxide ions), such as the case reported recently in the rock-salt layers in oxygen-excess $La_2NiO_{4+\delta}$ with a Ruddlesden–Popper structure.[18] However, the vehicle mechanism is rarely observed in oxide ceramics, and most of the proton conductors show a Grotthuss conduction mechanism, where protons are introduced by dissociative hydration of oxygen vacancies following Equation (1):

$$H_2O + V_O^{\bullet\bullet} + O_O^{\times} \rightarrow 2OH_O^{\bullet} \quad (1)$$

and rotate around and hop among the lattice oxides. Here, $V_O^{\cdot\cdot}$ and $O_O^{\times}$ are Krönig–Vink notations for oxygen vacancies and oxide ions, respectively. Since protons associate with oxide ions, they are expressed as $OH_O^{\cdot}$.

At present, perovskite (ABO_3) oxides (particularly those with Ba in the A-site and Zr, Ce, and acceptor dopants in the B-site) are the most attractive proton conductors due to their high proton conductivity (e.g., 0.01 $S\,cm^{-1}$ for $BaZr_{0.8}Y_{0.2}O_{3-\delta}$ at 500 °C[19–21]). However, these compositions with high Ba concentration easily react with and decompose in an atmosphere containing CO_2 and H_2O. Even the doped $BaZrO_3$, which was regarded to be chemically stable, was found to react with CO_2, giving rise to the formation of a resistive Ba-deficient surface.[22] Although these Ba-containing perovskite proton conductors receive great success at present, it is still necessary and interesting to expand our understanding by continuing searching new and stable proton conductors.

We recently reported proton conductors of $La_2(Nb_{1-y}Y_y)_2O_{7-\delta}$ (LNY) with a cubic pyrochlore ($A_2B_2O_7$) structure,[23,24] showing excellent chemical stability benefitted from the absence of stable carbonates and hydroxides of La, Nb and Y at the intermediate temperature. The idea of designing such a composition was inspired by using a mixture of pentavalent Nb and trivalent Y to replace tetravalent Zr in $La_2Zr_2O_7$. $La_2Zr_2O_7$ is

[a] *X. Zhang, L. Jiang, S. Guo, Prof. D. Han*
College of Energy
Soochow University
No 1 Shizi Street, Gusu District, Suzhou 215006 (P. R. China)
E-mail: dlhan@suda.edu.cn
[b] *Prof. D. Han*
Provincial Key Laboratory for Advanced Carbon Materials and Wearable Energy Technologies
Soochow University
No 1 Shizi Street, Gusu District, Suzhou 215006 (P. R. China)
[c] *Prof. D. Han*
Light Industry Institute of Electrochemical Power Sources
Shahu Science & Technology Innovation Park
Suzhou 215638 (P. R. China)
[d] *Prof. D. Han*
Key Laboratory of Core Technology of High Specific Energy Battery and Key Materials for Petroleum and Chemical Industry
Soochow University
Suzhou 215006 (P. R. China)

Supporting information for this article is available on the WWW under https://doi.org/10.1002/cssc.202201879

© 2022 Wiley-VCH GmbH

Chemistry Europe
European Chemical Societies Publishing

known to exhibit proton conduction by doping the La-site with divalent dopants (M) to form oxygen vacancies following Equation (2):

$$2MO + 2La_{La}^{\times} + O_O^{\times} \rightarrow 2M_{La}^{/} + La_2O_3 + V_O^{\bullet\bullet} \tag{2}$$

A total conductivity of $6.8\times10^{-4}\,S\,cm^{-1}$ at 600 °C was reported by Omata et al. for $(La_{0.975}Ca_{0.025})_2Zr_2O_{7-\delta}$.[25] But interestingly, in the case of LNY, the concentration of oxygen vacancies can be increased by increasing the cation ratio between Y and Nb over unity, following Equation (3):

$$2Nb_B^{\bullet} + Y_2O_3 + 2O_O^{\times} \rightarrow 2Y_B^{/} + Nb_2O_5 + 2V_O^{\bullet\bullet} \tag{3}$$

where $Nb_B^{\cdot}$ and $Y_B^{/}$ represent the Nb and Y cations occupying the B-site of the pyrochlore structure. In fact, such regulation of the composition also makes Y the acceptor dopant. However, different from the strategy to introduce foreign acceptor dopants (we name it as "foreign acceptor-doping" hereafter), the acceptor-doping can also be realized by simply changing the ratio between Y and Nb over unity in the B-site. We therefore name it as "domestic acceptor-doping" hereafter.

The discovery of the proton conduction in LNY offers potential to explore new proton conductors, since some other rare earth elements, like Yb, have similar properties with Y.[26] In this work, we report a study on preparing a new pyrochlore proton conductor $La_2(Nb_{1-y}Yb_y)_2O_{7-\delta}$ (LNYb), and both the domestic acceptor-doping (by increasing the Yb content to $x=0.55$) and foreign acceptor-doping (by doping Ca, Sr, or Ba into the La-site) strategies were applied to regulate its electrochemical properties. Furthermore, the relatively large difference in the scattering factors between Nb^{IV} and Yb^{III} enables a precise evaluation of the crystal structure of LNYb by simulating the X-ray diffraction (XRD) patterns of lab Cu K_α radiation, which was not feasible in the LNY system.

Results

Phase analysis

Firstly, $La_2(Nb_{1-y}Yb_y)_2O_{7-\delta}$ ($y=0.50$, 0.55, and 0.60) was prepared, and as shown in Figure 1, only $La_2(Nb_{0.45}Yb_{0.55})_2O_{7-\delta}$ (LNYb55) is identified to be of a single cubic pyrochlore phase. The inset in Figure 1 shows the peak belonging to the (331) reflection of the cubic pyrochlore structure, which does not appear in a fully disordered fluorite structure. A second phase (possibly $LaYbO_3$) with the feature of an orthorhombic structure can be confirmed in the XRD pattern of $La_2(Nb_{0.40}Yb_{0.60})_2O_{7-\delta}$ (LNYb60), which contains higher Yb content. For $La_2(Nb_{0.50}Yb_{0.50})_2O_{7-\delta}$ (LNYb50) with the Yb content lower than that of LNYb55, the 2θ positions where peaks appear in general agree with the cubic pyrochlore structure reference (00-009-032), but several adjacent peaks coexist at most of the 2θ positions. At the present stage, we cannot determine whether the LNYb50 sample contains multiple phases or is a single phase of distorted pyrochlore structure, but at least it is not a single cubic pyrochlore phase.

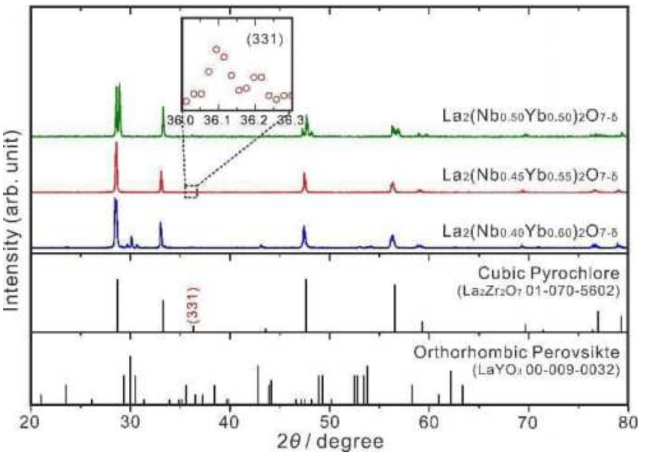

Figure 1. Powder XRD patterns of $La_2(Nb_{1-y}Yb_y)_2O_{7-\delta}$ ($y=0.50$, 0.55, 0.60), which were finally heated at 1600 °C for 24 h for sintering. References of $La_2Zr_2O_7$ (01-070-5602) and $LaYO_3$ (00-009-032) are used to show the features of cubic pyrochlore and orthorhombic perovskite structures. The inset shows the (331) reflection of $La_2(Nb_{0.45}Yb_{0.55})_2O_{7-\delta}$, which splits due to the Cu $K_{\alpha1}$ and $K_{\alpha2}$ radiations in the X-ray source.

In this work, we focus on the cubic pyrochlore system, and select LNYb55 for further doping with Ca, Sr, and Ba. As shown in Figure 2a, when the Ca doping level (x) in $(La_{1-x}Ca_x)_2(Nb_{0.45}Yb_{0.55})_2O_{7-\delta}$ is 0.05, the peaks belonging to $LaYbO_3$ appear. The samples were named as "x dopant-LNYb55" hereafter [e.g., 0.05 Ca-LNYb55 represents $(La_{0.95}Ca_{0.05})_2(Nb_{0.45}Yb_{0.55})_2O_{7-\delta}$]. With the Ca doping level decreasing to 0.03, there is still trace of the $LaYbO_3$ peaks, but the sample is almost a single cubic pyrochlore phase. When the Ca doping level was 0.01, only the diffraction peaks belonging to the cubic pyrochlore phase were observed, indicating that the solubility of Ca is within 0.01 and 0.03. For the Sr and Ba-doped samples, as shown in Figure 2b,c, when the doping level is as low as 0.01, trace of second phases can already be observed, but the peak intensity is negligibly weak, meaning that the solubility of Sr and Ba is lower than but close to 0.01. With the doping level of Sr and Ba increasing to 0.03, the peaks belonging to $LaYbO_3$ and $Ba_4Yb_2O_9$ can be seen clearly. The different solubility of these alkaline earth elements may be resulted from their different cation radius. The eight coordinated radius of Ca^{II} is 1.12 Å, close to that of La^{III} (1.160 Å). However, the radii of Sr^{II} (1.26 Å) and Ba^{II} (1.42 Å) are larger than that of La^{III}.[27]

Microstructure and composition

Figure 3 shows the microstructure of the selected as-sintered samples, which are (or nearly) single cubic pyrochlore phases. The samples are dense with the relative density higher than 94% measured by the Archimedes method (Table 1). The grain size is estimated to be around 10 μm from the scanning electron microscopy (SEM) images. The actual doping level, which was measured by the SEM energy-dispersive X-ray

© 2022 Wiley-VCH GmbH
1864564x, 2022, 24, Downloaded from https://chemistry-europe.onlinelibrary.wiley.com/doi/10.1002/cssc.202201879 by Nanjing Tech University, Wiley Online Library on [31/01/2025]. See the Terms and Conditions (https://onlinelibrary.wiley.com/terms-and-conditions) on Wiley Online Library for rules of use; OA articles are governed by the applicable Creative Commons License

doi.org/10.1002/cssc.202201879

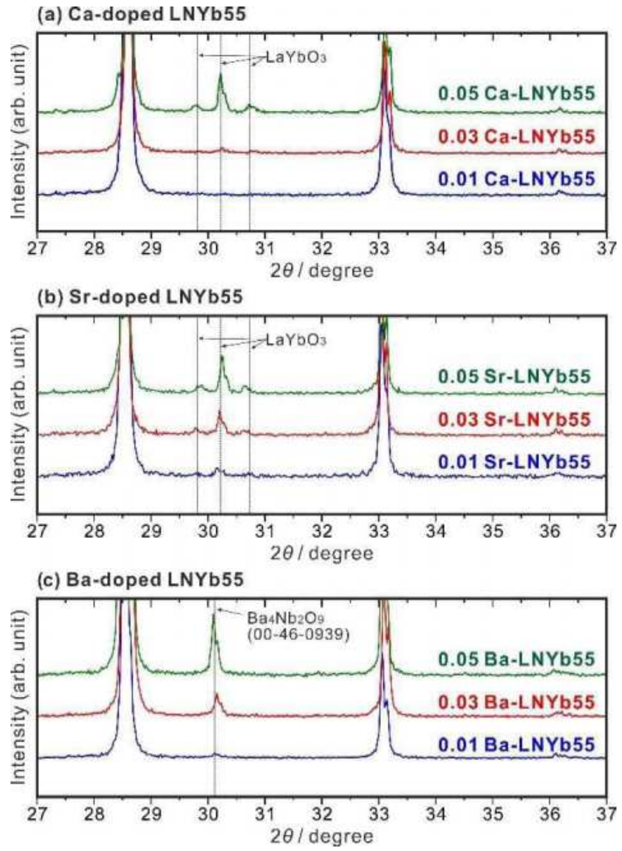

Figure 2. Powder XRD patterns in the range of 27–37° of (a) $(La_{1-x}Ca_x)_2(Nb_{0.45}Yb_{0.55})_2O_{7-\delta}$, (b) $(La_{1-x}Sr_x)_2(Nb_{0.45}Yb_{0.55})_2O_{7-\delta}$, and (c) $(La_{1-x}Ba_x)_2(Nb_{0.45}Yb_{0.55})_2O_{7-\delta}$ ($x=0.01$, 0.03, 0.05). All the samples were finally heated at 1600 °C for 24 h for sintering.

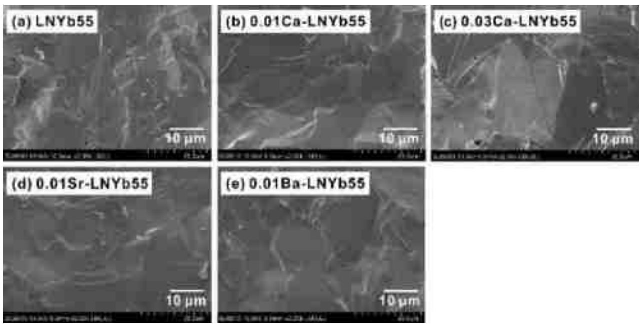

Figure 3. SEM images of the fractured cross-section of as-sintered (a) pristine LNYb55, (b) 0.01 Ca-LNYb55, (c) 0.03 Ca-LNYb55, (d) 0.01 Sr-LNYb55, and (e) 0.01 Ba-LNYb55.

spectroscopy (EDS) analysis, is close to the nominal value, as shown in Table 1 (a SEM-EDS spectrum of 0.03 Ca-LNYb55 is given in Figure S1).

Crystal structure analysis

Due to the very close scattering factors of Nb^V and Y^{III} (Figure S2) resulted from their almost same configuration of the valence electrons, we were unable to determine the site occupancy of Nb and Y in LNY by simulating the powder XRD patterns collected by the Cu K_α radiation in our previous works.[23,24] However, since the scattering factor of Yb^{III} is larger sufficiently than that of Nb^V, more precise structural analysis can be performed on LNYb. The best fitting of the XRD pattern of LNYb55 (Rwp ≈ 8.9 %), as shown in Figure 4, was obtained by assuming Nb and Yb to be distributed randomly in the B-site. Interestingly, as shown in Table 2, the four La cation-coordinated O1-site was found to be fully occupied. The additional oxygen vacancies, which formed due to the higher Yb/Nb ratio than unity, are accommodated in the O2-site. Similar results were also obtained by simulating the XRD patterns of the Ca, Sr, and Ba-doped samples; that is, only the O2-site surrounded by two La and two Nb/Y cations is available to accommodate oxygen vacancies generated by acceptor-doping. Further discussion will be given in the Discussion section.

Hydration behavior

Proton concentration of $(La_{1-x}M_x)_2(Nb_{0.45}Yb_{0.55})_2O_{7-\delta}$ (M=Ca, Sr, and Ba) measured by the Karl-Fischer titration is shown in Figure 5. The proton concentration per formula unit in LNYb55, which has the oxygen vacancy concentration of 0.1 in the O2-site and 1 in the intrinsic oxygen vacancy-site, are found to be 0.0229, 0.0169, and 0.0132 in wet Ar ($p_{H_2O}=0.05$ atm) at 500, 600, and 700 °C, respectively. By doping 0.01 Ca, the oxygen vacancy concentration in the O2-site increases by 10 % to 0.11, but interestingly, the proton concentration is almost doubled from 0.0229 in LNYb55 to 0.0454 in 0.01 Ca-LNYb55 at 500 °C. Since there is no change in the B-site, the coordination environment and therefore the hydration ability of the intrinsic oxygen vacancies surrounded by only the B-site cations are expected to be invariable. So, the enhancement of the hydration ability is induced from the slight increase in the concentration of oxygen vacancies in the O2-site and the

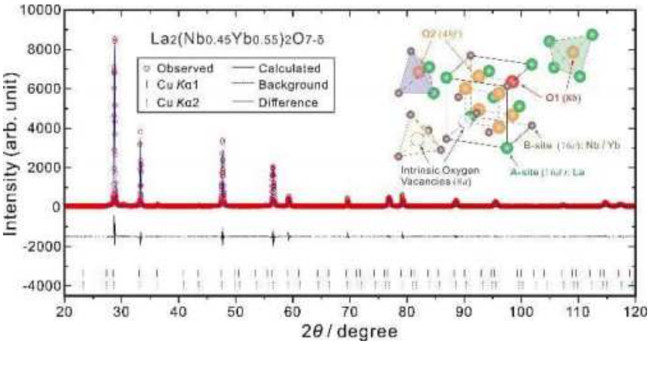

Figure 4. Fitting of the powder XRD pattern of $La_2(Nb_{0.45}Yb_{0.55})_2O_{7-\delta}$ by the Rietveld refinement.

© 2022 Wiley-VCH GmbH

1864564x, 2022, 24, Downloaded from https://chemistry-europe.onlinelibrary.wiley.com/doi/10.1002/cssc.202201879 by Nanjing Tech University, Wiley Online Library on [31/01/2025]. See the Terms and Conditions (https://onlinelibrary.wiley.com/terms-and-conditions) on Wiley Online Library for rules of use; OA articles are governed by the applicable Creative Commons License

1864564x, 2022, 24, Downloaded from https://chemistry-europe.onlinelibrary.wiley.com/doi/10.1002/cssc.202201879 by Nanjing Tech University, Wiley Online Library on [31/01/2025]. See the Terms and Conditions (https://onlinelibrary.wiley.com/terms-and-conditions) on Wiley Online Library for rules of use; OA articles are governed by the applicable Creative Commons License

Table 1. Dopant (M) concentration, relative density, transport number of ionic conduction in wet oxygen (p_{H_2O} = 0.05 atm) of pristine $La_2(Nb_{0.45}Yb_{0.55})_2O_{7-\delta}$ and those doped with Ca, Sr, or Ba.

Composition	Abbreviation	Relative density [%]	Dopant ratio [M/(M+La)] nominal	actual	Ionic transport number 500 °C	600 °C	700 °C
$La_2(Nb_{0.45}Yb_{0.55})_2O_{7-\delta}$	LNYb55	94.1	–	–	0.97	0.92	0.78
$(La_{0.99}Ca_{0.01})_2(Nb_{0.45}Yb_{0.55})_2O_{7-\delta}$	0.01 Ca-LNYb55	98.2	0.010	0.009	0.98	0.95	0.90
$(La_{0.97}Ca_{0.03})_2(Nb_{0.45}Yb_{0.55})_2O_{7-\delta}$	0.03 Ca-LNYb55	97.9	0.030	0.040	0.98	0.91	0.81
$(La_{0.99}Sr_{0.01})_2(Nb_{0.45}Yb_{0.55})_2O_{7-\delta}$	0.01 Sr-LNYb55	97.6	0.010	0.010	0.99	0.91	0.82
$(La_{0.99}Ba_{0.01})_2(Nb_{0.45}Yb_{0.55})_2O_{7-\delta}$	0.01 Ba-LNYb55	97.9	0.010	0.014	0.99	0.93	0.80

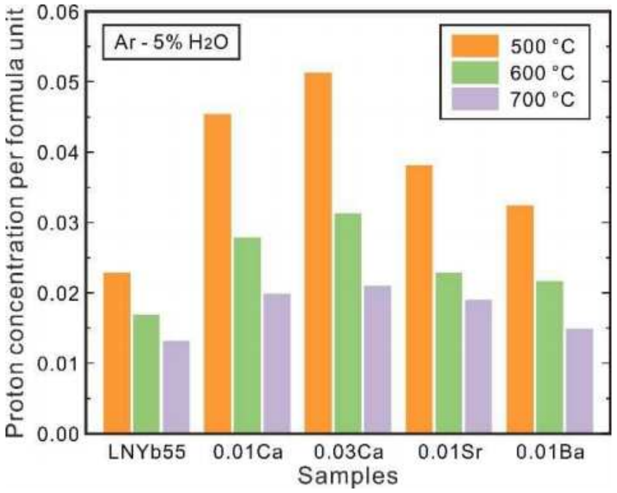

Figure 5. Proton concentration per formula unit of pristine LNYb55, 0.01 Ca-LNYb55, 0.03 Ca-LNYb55, 0.01 Sr-LNYb55, and 0.01 Ba-LNYb55, which were annealed in wet argon (p_{H_2O} = 0.05 atm) at 500, 600, or 700 °C. The proton concentration was calculated from the water content determined by Karl-Fischer titration.

change in its coordination environment due to the Ca-doping. With the Ca doping level increasing from 0.01 to 0.03, the proton concentration further increases. Doping Sr and Ba also enhances the hydration ability of LNYb55. At the same doping level of 0.01, Ca appears to favor the hydration of LNYb55 the most, and the proton concentration decreases in the sequence of Ca > Sr > Ba.

Conduction behavior

As show in Figure 6, the impedance spectrum of LNYb55 shifted to larger resistance side by altering the atmosphere from Ar/3% H_2O to Ar/3% D_2O at 700 °C. Each spectrum is composed of two semicircles with the high- and low-frequency ones belonging to the contributions from the grain boundary ($\approx 10^{-9}$ F) and electrode ($\approx 10^{-6}$ F), respectively, judged from their capacitances. The value at the valley between the two semicircles is read as the resistance of the sample (R_{sample}). Then, the total conductivity (σ_{total}), which reflects the sum of the intra-grain and inter-grain contributions, is calculated following Equation (4), where L is the thickness of the sample, and S is the area of the Ag electrodes at one side:

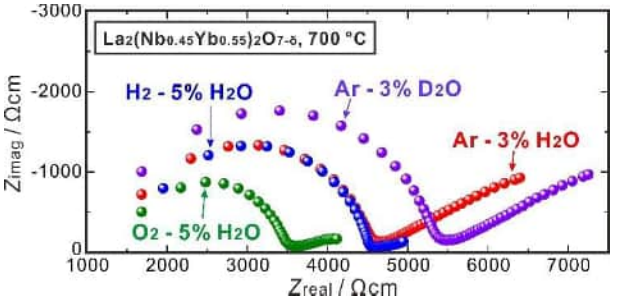

Figure 6. AC impedance spectra of LNYb55 collected at 700 °C in Ar/3% H_2O, Ar/3% D_2O, H_2/5% H_2O, and O_2/5% H_2O.

$$\sigma_{total} = \frac{L}{R_{sample}\ S} \tag{4}$$

The ratio between the conductivities in H_2O- and D_2O-humidified argon is about 1.2, indicating the generation of proton conduction. However, such ratio is lower than the typical value about 1.4 for the H/D isotope effect in high-temperature proton conducting oxides,[28] indicating that protons are probably not the only ionic charge carriers, and this material may conduct oxide ions as well.

Figure 7a shows the Arrhenius plots of total conductivities in wet hydrogen ($\sigma_{wet\ H_2}$) and wet oxygen ($\sigma_{wet\ O_2}$). The difference between $\sigma_{wet\ H_2}$ and $\sigma_{wet\ O_2}$ is negligibly small at relatively low temperature (e.g., 400 °C). With the increasing temperature, $\sigma_{wet\ O_2}$ turns to be larger than $\sigma_{wet\ H_2}$ (a comparison between the impedance spectra in wet hydrogen and wet oxygen at 700 °C is given in Figure 6), due to the formation of electron holes ($h^\bullet$) in an oxidizing atmosphere following Equation (5):

$$O_2 + 2V_O^{\bullet\bullet} \rightarrow 2O_O^{\times} + 4h^{\cdot} \tag{5}$$

Interestingly, the slope of Arrhenius plots changes with the temperature. By fitting with Equation (6):

$$\sigma_{total} = Aexp\left(\frac{-E_a}{k_B T}\right) \tag{6}$$

where A is pre-exponential factor, E_a is activation energy, k_B is Boltzmann's constant, T is temperature, E_a at the temperature ranges of 400–500 and 550–700 °C in H_2/5% H_2O was determined to be 0.92 and 0.59 eV, respectively. The reduced E_a at high temperature may be due to the weakened trapping effect of the dopants to protons,[29–32] or decreased ordering of

© 2022 Wiley-VCH GmbH

Table 2. Results of Rietveld refinement. The position and multiplicity are (0, 0, 0) and 16*d* for the A-site, (0.5, 0.5, 0.5) and 16*c* for the B-site, (0.125, 0.125, 0.125) and 8*b* for the O1-site, and (0.419, 0.419, 0.419) and 48*f* for the O2-site.

Composition	Lattice constant [Å]	A-site Cation	Occupancy	Isotropic temperature factor [Å³]	B-site Cation	Occupancy	Isotropic temperature factor [Å³]	O1-site (oxide ions) Occupancy	O1-site (oxide ions) Isotropic temperature factor [Å³]	O2-site (oxide ions) Occupancy	O2-site (oxide ions) Isotropic temperature factor [Å³]
$La_2(Nb_{0.45}Yb_{0.55})_2O_{7-\delta}$	10.8417	La	1.000	0.018	Nb	0.450	0.024	1.000	0.018	0.983	0.018
					Yb	0.550	0.024				
$(La_{0.99}Ca_{0.01})_2(Nb_{0.45}Yb_{0.55})_2O_{7-\delta}$	10.8379	La	0.990	0.022	Nb	0.450	0.024	1.000	0.031	0.982	0.031
		Ca	0.010	0.022	Yb	0.550	0.024				
$(La_{0.97}Ca_{0.03})_2(Nb_{0.45}Yb_{0.55})_2O_{7-\delta}$	10.8358	La	0.970	0.027	Nb	0.450	0.021	1.000	0.033	0.978	0.033
		Ca	0.030	0.027	Yb	0.550	0.021				
$(La_{0.99}Sr_{0.01})_2(Nb_{0.45}Yb_{0.55})_2O_{7-\delta}$	10.8397	La	0.990	0.023	Nb	0.450	0.025	1.000	0.031	0.982	0.031
		Sr	0.010	0.023	Yb	0.550	0.025				
$(La_{0.99}Ba_{0.01})_2(Nb_{0.45}Yb_{0.55})_2O_{7-\delta}$	10.8411	La	0.990	0.018	Nb	0.450	0.019	1.000	0.023	0.982	0.023
		Ba	0.010	0.018	Yb	0.550	0.019				

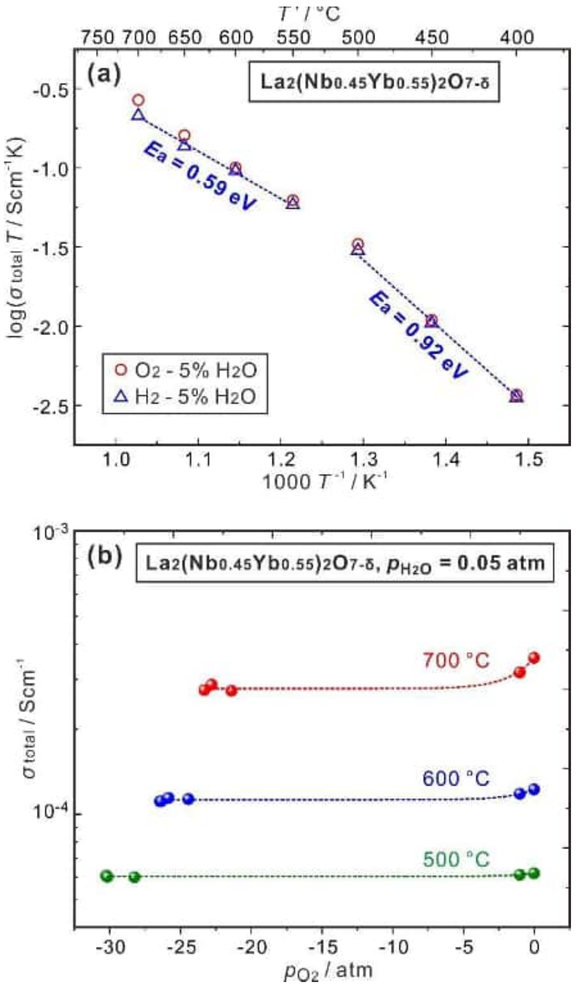

Figure 7. (a) Arrhenius plots of total conductivities of LNYb55 in wet oxygen and hydrogen atmospheres. (b) Dependence of total conductivities of LNYb55 at 500, 600, and 700 °C on partial pressure of oxygen. p_{H_2O} was kept at 0.05 atm.

the lattice oxide ions and oxygen vacancies excited by the high temperature, which may facilitate the proton conduction. But, in the LNYb55-based materials studied in this work, loss of protons with increasing temperature[19,20] is more likely to be the reason, which will be explained later.

Transport number

By plotting σ_{total} against p_{O_2}, as shown in Figure 7b, σ_{total} changes little at low p_{O_2} region and increases with the increasing p_{O_2} at high p_{O_2} region. Based on the reactions to introduce protons [Eq. (1)] and holes [Eq. (5)], σ_{total} can be treated as a sum of the p_{O_2}-independent ionic conductivity (σ_{ion}) and hole conductivity (σ_h), which is proportional to $p_{O_2}^{1/4}$, as given in Equation (7), where $\sigma_{h,\,p_{O_2}=1\ atm}$ is the hole conductivity under $p_{O_2} = 1$ atm.

© 2022 Wiley-VCH GmbH

1864564x, 2022, 24, Downloaded from https://chemistry-europe.onlinelibrary.wiley.com/doi/10.1002/cssc.202201879 by Nanjing Tech University, Wiley Online Library on [31/01/2025]. See the Terms and Conditions (https://onlinelibrary.wiley.com/terms-and-conditions) on Wiley Online Library for rules of use; OA articles are governed by the applicable Creative Commons License

$$\sigma_{\text{total}} = \sigma_{\text{ion}} + \sigma_{\text{h},\, p_{O_2}=1\text{ atm}} \left(p_{O_2}\right)^{\frac{1}{4}} \tag{7}$$

By fitting the dependence of σ_{ion} on p_{O_2}, the ionic and hole conductivities can be separated. As shown in Figure 8, for all the five samples studied, σ_{ion} increases with the increasing temperature. σ_{ion} of the pristine LNYb55 is 2.78×10^{-4} S cm^{-1} at 700 °C, and increases to 4.24 and 4.51×10^{-4} S cm^{-1} by doping 0.01 and 0.03 Ca, respectively, indicating that σ_{ion} can be improved by doping alkaline earth elements to substitute La. The results of $\sigma_{\text{h},\, p_{O_2}=1\text{ atm}}$ are shown in Figure 9. It is difficult to figure out a clear tendency of the effect on the hole conduction by doping alkaline earth elements. For example, the hole conductivities of 0.01 Sr and Ba-doped samples are lower than that of LNYb55 at 500 °C, but are slightly higher at 700 °C. However, in general, the hole conductivity appears to not change greatly by doping with Ca, Sr, and Ba.

Based on the information of σ_{ion} and $\sigma_{\text{h},\, p_{O_2}=1\text{ atm}}$, the transport number of ionic conduction (t_{ion}) can be calculated following Equation (8):

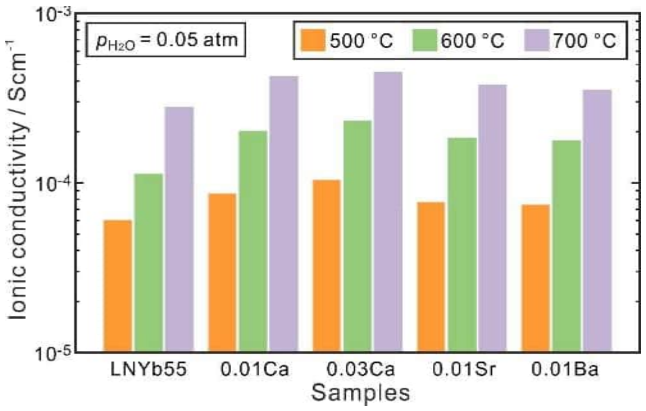

Figure 8. Ionic conductivity of pristine LNYb55, 0.01 Ca-LNYb55, 0.03 Ca-LNYb55, 0.01 Sr-LNYb55, and 0.01 Ba-LNYb55 in wet atmosphere (p_{H_2O} = 0.05 atm) at 500, 600, or 700 °C.

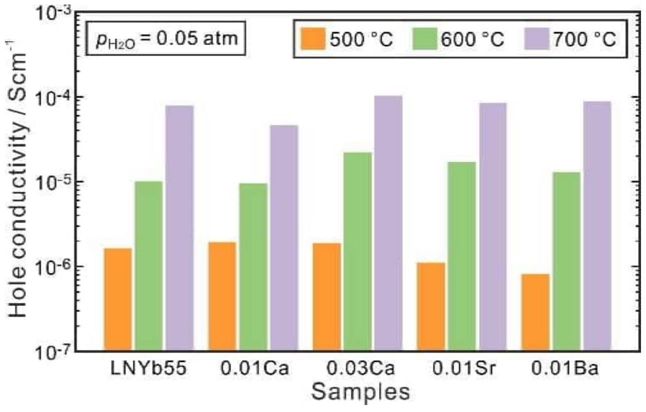

Figure 9. Hole conductivity of pristine LNYb55, 0.01 Ca-LNYb55, 0.03 Ca-LNYb55, 0.01 Sr-LNYb55, and 0.01 Ba-LNYb55 in wet oxygen (p_{H_2O} = 0.05 atm) at 500, 600, or 700 °C.

$$t_{\text{ion}} = \frac{\sigma_{\text{ion}}}{\sigma_{\text{ion}} + \sigma_{\text{h, 1 atm}} \left(p_{O_2}\right)^{\frac{1}{4}}} \tag{8}$$

As shown in Figure 10, all the samples show $t_{\text{ion}} \geq 0.97$ at 500 °C. With the temperature increasing to 600 and 700 °C, t_{ion} falls into the range of 0.91–0.95 and 0.78–0.90, respectively. For comparison, the state-of-the-art proton conductor $BaZr_{0.8}Y_{0.2}O_{3-\delta}$ (BZY20) shows t_{ion} of 0.94, 0.79, and 0.43 in wet O_2 (p_{H_2O} = 0.05 atm) at 500, 600, and 700 °C, respectively.[33] At 500 °C, t_{ion} of BZY20 is close to that of the LNYb55-based samples. However, with the increasing temperature, the LNYb55- based samples show much higher t_{ion}. In this work, the highest t_{ion} at 600 and 700 °C is 0.95 and 0.90, respectively, obtained in 0.01-Ca-LNYb55. As far as we know, such high t_{ion} is among the top class of the proton conducting oxides, higher than t_{ion} at 600 °C of $SrZr_{0.9}Y_{0.1}O_{3-\delta}$ (0.90),[34] $BaCe_{0.95}Y_{0.05}O_{3-\delta}$ (0.79),[35] $Ba_3Ca_{1.18}Nb_{1.82}O_{3-\delta}$ (0.75),[36] and $Ba_3Sr_{1.4}Ti_{1.6}O_{8.4}$ (0.90).[37] However, since the ionic conductivity of the LNYb55-based materials is still not high enough, it is difficult to apply them directly as the electrolyte in solid-state electrochemical devices. It is more reasonable to apply them as a barrier layer on some other electrolyte materials with high conductivity (such as BZY20) to improve the chemical stability against water vapor and improve the total t_{ion} of the electrolyte.

Proton diffusivity

The proton conductivity can be expressed as Equation (9):

$$\sigma_{H^+} = nqc_{H^+}\mu_{H^+} \tag{9}$$

where σ_{H^+}, c_{H^+}, and μ_{H^+} are conductivity, concentration, and mobility of protons, respectively. q is the quantity of electric charge of a single proton (the same as that of an electron), and n is the number of charge of a proton (namely, 1). The diffusivity (D_{H^+}) of protons can be further calculated by Equation (10):

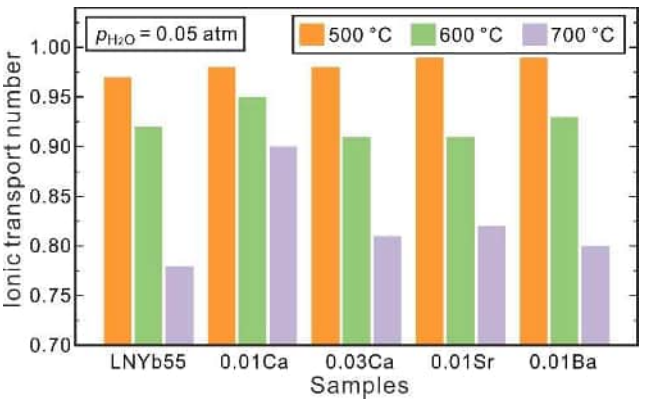

Figure 10. Transport numbers of ionic conduction in pristine LNYb55, 0.01 Ca-LNYb55, 0.03 Ca-LNYb55, 0.01 Sr-LNYb55, and 0.01 Ba-LNYb55 in wet oxygen (p_{H_2O} = 0.05 atm) at 500, 600, or 700 °C.

© 2022 Wiley-VCH GmbH

1864564x, 2022, 24, Downloaded from https://chemistry-europe.onlinelibrary.wiley.com/doi/10.1002/cssc.202201879 by Nanjing Tech University, Wiley Online Library on [31/01/2025]. See the Terms and Conditions (https://onlinelibrary.wiley.com/terms-and-conditions) on Wiley Online Library for rules of use; OA articles are governed by the applicable Creative Commons License

doi.org/10.1002/cssc.202201879
Chemistry Europe
European Chemical Societies Publishing

$$D_{H^+} = \left(\frac{k_B T}{q}\right) \mu_{H^+} \quad (10)$$

The thereby determined diffusivities of protons are plotted in Figure 11. In the temperature studied in this work, D_{H^+} of LNYb55-based materials is higher than that of $SrZr_{0.9}Y_{0.1}O_{3-\delta}$ (SZY10) and $SrCe_{0.9}Y_{0.1}O_{3-\delta}$ (SCY10), but lower that of $BaCe_{0.9}Y_{0.1}O_{3-\delta}$ (BCY10). Although the temperature range (500–700 °C) studied in this work is relatively limited, we roughly estimated the activation energy for the proton diffusion in LNYb55 (0.82 eV), 0.01 Ca-LNYb55 (0.93 eV), 0.03 Ca-LNYb55 (0.91 eV), 0.01 Sr-LNYb55 (0.89 eV), and 0.01 Ba-LNYb55 (0.90 eV), respectively. Such value is higher than that obtained by fitting the conductivity data at the same temperature range, but close to that at the low temperature range (400–500 °C), indicating that the true E_a changes little within the temperature range between 400 and 700 °C. Therefore, the bend-off of the Arrhenius plots as shown in Figure 7a is more likely due to the loss of proton by dehydration at the high temperature.

Discussion

From the results of Rietveld refinement in this work, the oxygen vacancies formed due to the internal acceptor-doping (by increasing the cation ratio of Y/Nb over unity) and external acceptor-doping (by using divalent Ca, Sr, and Ba to replace trivalent La) are accommodated in the O2-site. The O1-site was found to be fully occupied by oxide ions. One possible reason is that the open space in the center of O1 tetrahedron surrounded by four La cations is larger than that of the O2 tetrahedron surrounded by two La and two Y/Nb cations, since the cation radius decreases in the sequence of La^{III} (1.160 Å) > Yb^{III} (0.985 Å) > Nb^{V} (0.74 Å).[27] Furthermore, in our previous work on using extended X-ray absorption fine structure (EXAFS) analysis to probe the local crystal structure of LNY,[24] there is negligible small difference in the coordination number of the oxide ions nearest to Nb between the hydrated and dehydrated LNY samples, implying that the oxygen vacancies or oxide ions in the vicinity of Nb contribute little to the hydration reaction to introduce protons, and the Nb-containing tetrahedra are possibly inactive for the hydration reaction. In contrary, the coordination number of the nearest oxide ions to Y increases obviously when the LNY samples were hydrated.[24] Since the radii of Yb^{III} and Y^{III} are very close,[27] and similar doping behavior was reported in other proton conductors,[39] we assume that Y and Yb may exhibit similar properties in LNY and LNYb. In consequence, not all the O2 tetrahedra are favorable for the hydration reaction, except the one having two La and two Y cations. Furthermore, oxygen vacancies may also prefer to locate in such specific tetrahedra, since Yb in the lattice of LNYb takes a negative charge ($Yb_B^{/}$) favoring the association with positively charged oxygen vacancies ($V_O^{\cdot\cdot}$), leading to the stabilization of oxygen vacancies in the tetrahedra with two Y and two La cations.

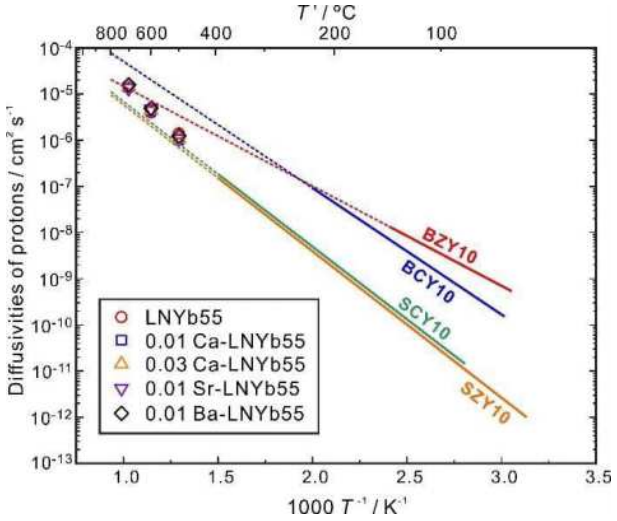

Figure 11. Proton diffusivities in LNYb55, 0.01 Ca-LNYb55, 0.03 Ca-LNYb55, 0.01 Sr-LNYb55, and 0.01 Ba-LNYb55. The data (solid lines) for $SrZr_{0.9}Y_{0.1}O_{3-\delta}$ (SZY10), $SrCe_{0.9}Y_{0.1}O_{3-\delta}$ (SCY10), $BaZr_{0.9}Y_{0.1}O_{3-\delta}$ (BZY10), and $BaCe_{0.9}Y_{0.1}O_{3-\delta}$ (BCY10)[38] are plotted for comparison. The dash lines are extrapolated from the solid lines.

The concentration of oxygen vacancies in the intrinsic oxygen vacancy-site (8*a*) is 1 per LNYb55 formula unit, much larger than that in the O2-site (0.1 for LNYb45). Since these oxygen vacancies are only surrounded with four B-site cations (Nb or Yb), and the oxygen vacancies close to Nb has been ruled out for contributing to the hydration reaction, only the one surrounded with four Yb cations may favor the hydration reaction. At present stage, it is difficult to give a quantitative analysis. However, it deserves noting that Kalland et al. reported that only the tetrahedra containing four La cations (namely, without Ce) in a $La_2Ce_2O_7$ fluorite oxide participated in the hydration reaction,[40] corresponding to the proton concentration of 0.099 per 1 tetrahedron containing four La at 600 °C in Ar/5 % H_2O.[41,42] Besides, it is known that Y-stabilized ZrO_2 (YSZ) is difficult to be hydrated, and the proton/deuteron concentration per $Zr_{0.85}Y_{0.15}O_{2-\delta}$ (8YSZ) formula unit is about 1.88×10^{-5} at 600 °C in Ar/1 % D_2/2 % D_2O.[43] Then, assuming that only the tetrahedra without Zr can be protonated, the proton concentration per 1 tetrahedron composed of four Y is estimated to be 0.037. Since Y and Yb act similarly as dopants in proton conductors,[34] the tetrahedron containing four Yb is supposed to have similar properties. These results indicate that the tetrahedra in a fluorite-based structure is easier to be protonated when it contains La than Y/Yb. If assuming that only the tetrahedra in LNYb55 containing four Yb (8*a* site) or two Yb and two La (48*f* site) can be protonated, the average proton concentration per 1 tetrahedron is therefore 0.088 at 600 °C in Ar/5 % H_2O, between those of the tetrahedron with only Y/Yb and only La. The above analysis gives a rough estimation showing the potential difference in the ability to be protonated depending on the coordination of the tetrahedra in fluorite-based crystal structures, but further experimental or computational investigation is essential to give a more comprehensive understanding.

© 2022 Wiley-VCH GmbH

1864564x, 2022, 24, Downloaded from https://chemistry-europe.onlinelibrary.wiley.com/doi/10.1002/cssc.202201879 by Nanjing Tech University, Wiley Online Library on [31/01/2025]. See the Terms and Conditions (https://onlinelibrary.wiley.com/terms-and-conditions) on Wiley Online Library for rules of use; OA articles are governed by the applicable Creative Commons License

1864564x, 2022, 24, Downloaded from https://chemistry-europe.onlinelibrary.wiley.com/doi/10.1002/cssc.202201879 by Nanjing Tech University, Wiley Online Library on [31/01/2025]. See the Terms and Conditions (https://onlinelibrary.wiley.com/terms-and-conditions) on Wiley Online Library for rules of use; OA articles are governed by the applicable Creative Commons License

Compared with some state-of-the-art proton conductors (such as BZY20), LNYb55 shows lower proton concentration and proton conductivity. The low proton concentration is correlated with the limited active oxygen vacancies and lattice oxide ions for hydration or protonation and is also one of the reasons leading to low proton conductivity. As aforementioned, since not all the tetrahedra in the crystal structure of LNYb55 can be protonated, the route for proton to migrate is restricted. For further developing the proton conductors with the pyrochlore structure, regulating the coordination of the tetrahedra to enhance the ability for hydration or protonation, and expanding the available route for proton migration are necessary.

Conclusion

In this work, a class of ionic conductor $La_2(Nb_{0.45}Yb_{0.55})_2O_{7-\delta}$ with the cubic pyrochlore structure was prepared. Incorporation of protons by dissociative hydration and H/D isotope effect in electrical conductivity were confirmed, indicating that this material is a proton conductor. By doping alkaline earth elements into the La-site, increase in proton concentration and conductivity was observed. For example, the ionic conductivity of LNYb55 is 2.78×10^{-4} S cm^{-1} at 700 °C and increases to 4.24×10^{-4} S cm^{-1} for 0.01 Ca-doped LNYb55. Based on the refinement of the crystal structure, it was found that the extrinsically introduced oxygen vacancies by doping aliovalent cations or increasing the cation ratio between Yb and Nb over unity are accommodated in the tetrahedra (48*f*) containing two La and two Yb/Nb cations, leaving the tetrahedra containing four La cations (8*a*) fully occupied by oxide ions. Then, a discussion based on literature data and our previous results on $La_2(Nb_{1-x}Y_x)_2O_{7-\delta}$ was performed, suggesting that not all the tetrahedra in the cubic pyrochlore structure of LNYb help in incorporating and conducting protons. Only the oxygen vacancies surrounded by four Y cations (48*f* site) or two La and two Y cations (8*b* site) are hydratable. Tuning the ability of hydration or protonation of the tetrahedra in the cubic pyrochlore structure to increase the proton concentration and expand the route for proton conduction are thereby potential strategies for increasing the proton conductivity.

Experimental Section

Material preparation

Samples with the nominal compositions of $La_2(Nb_{1-y}Yb_y)_2O_{7-\delta}$ ($y=$ 0.50, 0.55, 0.60) and $(La_{1-x}M_x)_2(Nb_{0.45}Yb_{0.55})_2O_{7-\delta}$ (M=Ca, Sr, Ba; $x=$ 0.01, 0.03, 0.05) were prepared by a conventional solid-state reaction method. The raw materials of La_2O_3 (99.9%), Nb_2O_5 (99.9%), Yb_2O_3 (99.99%), $CaCO_3$ (99.9%), $SrCO_3$ (99%), and $BaCO_3$ (99.95%) were mixed in the desired ratio and ball-milled for 24 h. The mixtures were then heat treated at 1000 °C for 10 h. After crushing and ball-milling for 10 h, the samples were pressed into pellets under 76 MPa and annealed at 1300 °C for 10 h. Then, the samples were pulverized by ball-milling for 50 h and pressed into pellets at 371 MPa. Finally, the pellets were sintered at 1600 °C for 24 h in ambient atmosphere. The samples doped with Ca, Sr, or Ba were covered with corresponding sacrificial powder (namely, the powder obtained after annealing at 1300 °C) to suppress the evaporation of CaO, SrO, or BaO during sintering.

Characterization

Powder XRD analysis was performed with a Bruker D8 Advance diffractometer (Laguna Hills, CA, USA). Rietveld refinement was performed by utilizing GSAS-II.[39] SEM (Hitachi SU 8010 Boerne, Tokyo, Japan) was performed to observe the microstructure of the as-sintered samples. Composition was measured by SEM-EDS (SU 8010, USA). The relative density of the as-sintered samples was measured by Archimedes method. Proton concentration was determined from the water content which was measured by Karl-Fischer titration method. The samples sintered at 1600 °C were crashed into small pieces about 2 mm in length and annealed in a wet Ar atmosphere at 500, 600, or 700 °C. The partial pressure of water vapor (p_{H_2O}) of 0.05 atm was obtained by bubbling Ar gas through de-ionized water kept at 33 °C.

Conductivity measurements were performed at 500, 600, and 700 °C on the as-sintered pellets, which were painted with silver electrodes, in wet atmospheres of hydrogen, oxygen, or those diluted with argon. p_{H_2O} was kept at 0.05 atm. The AC impedance spectra were collected with a frequency response analyzer (SI 1260, Solartron Analytical, Farnborough, UK) in the frequency range of 10 Hz–7 MHz under the applied voltage of 100 mV. The impedance spectra were analyzed with a Zview software (Scribner Associates, Inc. NC, USA).

Acknowledgements

This work was supported by the Natural Science Foundation of Jiangsu Province (Grant No. BK20211071), the Key Technology Initiative of Suzhou Municipal Science and Technology Bureau (Grant No. SYG202011), and the Project of Innovation and Entrepreneurship of Jiangsu Province (Grant No. JSSCRC2021526).

Conflict of Interest

The authors declare no conflict of interest.

Data Availability Statement

The data that support the findings of this study are available from the corresponding author upon reasonable request.

Keywords: conducting materials · hydration · oxygen vacancies · proton conductor · pyrochlore structure

[1] H. Iwahara, T. Esaka, H. Uchida, N. Maeda, *Solid State Ionics* **1981**, *3–4*, 359–363.
[2] H. Iwahara, H. Uchida, S. Tanaka, *Solid State Ionics* **1983**, *9–10*, 1021–1026.
[3] C. Duan, J. Tong, M. Shang, S. Nikodemski, M. Sanders, S. Ricote, A. Almansoori, R. O'Hayre, *Science* **2015**, *349*, 1321–1326.
[4] K. Bae, D. Y. Jang, H. J. Choi, D. Kim, J. Hong, B. Kim, J. Lee, J. Son, J. H. Shim, *Nat. Commun.* **2017**, *8*, 14553.

© 2022 Wiley-VCH GmbH

[5] K. Bae, D. H. Kim, H. J. Choi, J. Son, J. H. Shim, *Adv. Energy Mater.* **2018**, *8*, 1801315.
[6] S. Choi, C. J. Kucharczyk, Y. Liang, X. Zhang, I. Takeuchi, H. Ji, S. M. Haile, *Nat. Energy* **2018**, *3*, 202–210.
[7] H. An, H. Lee, B. Kim, J. W. Son, K. J. Yoon, H. Kim, D. Shin, H. I. Ji, J. H. Lee, *Nat. Energy* **2018**, *3*, 870–875.
[8] W. Bian, W. Wu, B. Wang, W. Tang, M. Zhou, C. Jin, H. Ding, W. Fan, Y. Dong, J. Li, D. Ding, *Nature* **2022**, *604*, 479–485.
[9] L. Lei, Z. Tao, X. Wang, J. P. Lemmon, F. Chen, *J. Mater. Chem. A* **2017**, *5*, 22945–22951.
[10] K. Leonard, Y. Okuyama, Y. Takamura, Y. Lee, K. Miyazaki, M. E. Ivanova, W. A. Meulenberg, H. Matsumoto, *J. Mater. Chem. A* **2018**, *6*, 19113–19124.
[11] C. Duan, R. Kee, H. Zhu, N. Sullivan, L. Zhu, L. Bian, D. Jennings, R. O'Hayre, *Nat. Energy* **2019**, *4*, 230–240.
[12] E. Vøllestad, R. Strandbakke, M. Tarach, D. Catalán-Martínez, M. Fontaine, D. Beeaff, D. R. Clark, J. M. Serra, T. Norby, *Nat. Mater.* **2019**, *18*, 752–759.
[13] S. Fang, S. Wang, K. S. Brinkman, F. Chen, *J. Mater. Chem. A* **2014**, *2*, 5825–5833.
[14] S. Fang, K. Brinkman, F. Chen, *ACS Appl. Mater. Interfaces* **2014**, *6*, 725–730.
[15] S. H. Morejudo, R. Zanón, S. Escolástico, I. Yuste-Tirados, H. Malerød-Fjeld, P. K. Vestre, W. G. Coors, A. Martínez, T. Norby, J. M. Serra, C. Kjølseth, *Science* **2016**, *353*, 563–566.
[16] H. Malerød-Fjeld, D. Clark, I. Yuste-Tirados, R. Zanón, D. Catalán-Martinez, D. Beeaff, S. H. Morejudo, P. K. Vestre, T. Norby, R. Haugsrud, J. M. Serra, C. Kjølseth, *Nat. Energy* **2017**, *2*, 923–931.
[17] D. Clark, H. Malerød-Fjeld, M. Budd, I. Yuste-Tirados, D. Beeaff, S. Aamodt, K. Nguyen, L. Ansaloni, T. Peters, P. K. Vestre, D. K. Pappas, M. I. Valls, S. Remiro-Buenamañana, T. Norby, T. S. Bjørheim, J. M. Serra, C. Kjølseth, *Science* **2022**, *376*, 390–393.
[18] P. Zhong, K. Toyoura, L. Jiang, L. Chen, S. A. Ismail, N. Hatada, T. Norby, D. Han, *Adv. Energy Mater.* **2022**, *12*, 2200392.
[19] Y. Yamazaki, R. Hernandez-Sanchez, S. M. Haile, *Chem. Mater.* **2009**, *21*, 2755–2762.
[20] D. Han, T. Uda, *J. Mater. Chem. A* **2018**, *6*, 18571–18582.
[21] D. Han, X. Liu, T. S. Bjørheim, T. Uda, *Adv. Energy Mater.* **2021**, *11*, 2003149.
[22] R. Sazinas, C. Bernuy-López, M. Einarsrud, T. Grande, *J. Am. Ceram. Soc.* **2016**, *99*, 3685–3695.
[23] D. Han, K. Kato, T. Uda, *Chem. Commun.* **2017**, *53*, 12684–12687.
[24] D. Han, P. Zhong, X. Zhang, L. Jiang, *J. Mater. Chem. A* **2022**, *10*, 8887–8897.
[25] T. Omata, S. Otsuka-Yao-Matsuo, *J. Electrochem. Soc.* **2001**, *148*, E252-E261.
[26] D. Han, K. Shinoda, S. Sato, M. Majima, T. Uda, *J. Mater. Chem. A* **2015**, *3*, 1243–1250.
[27] R. D. Shannon, *Acta Crystallogr.* **1976**, *A32*, 751–767.
[28] A. S. Nowick, A. V. Vaysleyb, *Solid State Ionics* **1997**, *97*, 17–26.
[29] Y. Yamazaki, F. Blanc, Y. Okuyama, L. Buannic, J. C. Lucio-Vega, C. P. Grey, S. M. Haile, *Nat. Mater.* **2013**, *12*, 647–651.
[30] F. M. Draber, C. Ader, J. P. Arnold, S. Eisele, S. Grieshammer, S. Yamaguchi, M. Martin, *Nat. Mater.* **2020**, *19*, 338–346.
[31] K. Toyoura, W. Meng, D, Han, T, Uda, *J. Mater. Chem. A* **2018**, *6*, 22721–22730.
[32] K. Toyoura, T. Fujii, N. Hatada, D. Han, T, Uda, *J. Phys. Chem. C* **2019**, *123*, 26823–26830.
[33] D. Han, K. Toyoura, T. Uda, *ACS Appl. Energ. Mater.* **2021**, *4*, 1666–1676.
[34] K. Kato, D. Han, T. Uda, *J. Am. Ceram. Soc.* **2019**, *102*, 1201–1210.
[35] J. Guan, S. E. D. U. Balachardran, M. Liu, *Solid State Ionics* **1997**, *100*, 45–52.
[36] N. V. Sharova, V. P. Gorelov, *Russ. J. Electrochem.* **2005**, *41*, 1001–1007.
[37] W. Huang, Y. Ding, Y. Li, Z. Wang, *Ceram. Int.* **2020**, *47*, 2517–2524.
[38] K. D. Kreuer, *Solid State Ionics* **1999**, *125*, 285–302.
[39] D. Han, K. Shinoda, S. Sato, M. Majima, T. Uda, *J. Mater. Chem. A* **2015**, *3*,1243–1250.
[40] L.-E. Kalland, A. Løken, T. S. Bjørheim, R. Haugsrud, T. Norby, *Solid State Ionics* **2020**, *354*, 115401.
[41] S. A. Ismail, L. Jiang, P. Zhong, T, Norby, D. Han, *J. Alloys Compd.* **2022**, *899*, 163306.
[42] S. A. Ismail, D. Han, *J. Am. Ceram. Soc.* **2022**, *105*, 7548.
[43] N. Sakai, K. Yamaji, T. Horita, H. Kishimoto, Y. P. Xiong, H. Yokokawa, *Solid State Ionics* **2004**, *175*, 387–391.
[44] B. H. Toby, R. B. Von Dreele, *J. Appl. Crystallogr.* **2013**, *46*, 544–549.

Manuscript received: October 8, 2022
Revised manuscript received: October 18, 2022
Accepted manuscript online: October 18, 2022
Version of record online: November 11, 2022

© 2022 Wiley-VCH GmbH

1864564x, 2022, 24, Downloaded from https://chemistry-europe.onlinelibrary.wiley.com/doi/10.1002/cssc.202201879 by Nanjing Tech University, Wiley Online Library on [31/01/2025]. See the Terms and Conditions (https://onlinelibrary.wiley.com/terms-and-conditions) on Wiley Online Library for rules of use; OA articles are governed by the applicable Creative Commons License

ELSEVIER

13 October 1995

CHEMICAL PHYSICS LETTERS

Chemical Physics Letters 244 (1995) 456-462

The Grotthuss mechanism

Noam Agmon

Department of Physical Chemistry and the Fritz Haber Research Center, The Hebrew University, Jerusalem 91904, Israel

Received 13 April 1995; in final form 31 July 1995

Abstract

Suggested mechanisms for proton mobility are confronted with experimental findings and quantum mechanical calculations, indicating that no model is consistent with the existing data. It is suggested that the molecular mechanism behind prototropic mobility involves a periodic series of isomerizations between $H_9O_4^+$ and $H_5O_2^+$, the first triggered by hydrogen-bond cleavage of a second-shell water molecule and the second by the reverse, hydrogen-bond formation process.

1. Introduction

Proton mobility in water is abnormally high [1]. At room temperature its limiting ionic conductance is about seven times that of a sodium cation, or approximately five times that of K^+. The usual explanation is in terms of a sequence of proton-transfer reactions (proton hops) between water molecules ('prototropic mobility', 'Grotthuss mechanism' [2]). In spite of many years of hard research and heated discussions, the exact details of this fundamental process remain obscure [3-12].

The models suggested for prototropic conduction no doubt contain the right ingredients for its explanation. Unfortunately, none has the full mechanism correctly assembled. In analyzing these models, it will become obvious that they all contradict known experimental facts (some, it seems, were suggested *in spite* of experimental reality). By carefully considering a wide variety of literature data, improbable mechanisms are eliminated and a likely microscopic picture of the Grotthuss mechanism emerges. This discussion is timely, because with the rapid advance in quantum chemistry calculations of proton transfer in water [13-18], and of Monte Carlo and molecular dynamics simulations [8,17,19], it may soon become possible to perform a sufficiently large calculation to check the proposed mechanism [18]. Reciprocally, insight from the following discussion may provide important guidelines for choosing relevant reaction coordinates in ab initio calculations.

2. Mechanisms of prototropic mobility

Two opposing limits of solvent involvement in prototropic mobility may be considered. Bernal and Fowler [4] pictured a proton hopping from a H_3O^+ moiety to a freely rotating nearest-neighbor water molecule. This 'complete disorder' limit presents one example for an incoherent transport mechanism. Conway and co-workers [5] suggested a 'field-induced' water rotation: in the $H_2OH^+ \ldots HOH$ complex, the water is forced to rotate its lone-pair towards the hydronium.

The opposite extreme is that of a large, static water cluster within which an ordered hydrogen-bonded network exists. In a familiar picture ('the relay mech-

0009-2614/95/$09.50 © 1995 Elsevier Science B.V. All rights reserved
SSDI 0009-2614(95)00905-1

anism'), the proton hops in a concerted fashion across a whole array of water molecules

$$\begin{array}{l}\mathrm{H}\quad\ \ \curvearrowright\mathrm{H}\quad\curvearrowright\mathrm{H}\quad\curvearrowright\mathrm{H}\\ \mathrm{HOH}'\ \ldots\ \mathrm{OH}\ \ldots\ \mathrm{OH}\ \ldots\ \mathrm{OH}\ \rightarrow\end{array} \tag{1}$$

The rate-limiting step could then be either the proton motion itself or a 'structural diffusion' process of adding water molecules to the periphery of the cluster [6]. Eigen claimed that proton motion is rate determining in ice, explaining their observation that its mobility is one or two orders of magnitude faster than in water [6]. Consequently, 'structural diffusion' is rate limiting in water [6].

Such pictures of proton mobility dominate physical chemistry textbooks. For example, Fig. 26.4 in Atkins shows a fast relay mechanism coupled to a rate-limiting 'structural diffusion' step involving a Conway-like forced water rotation [20]. More recent discussions of proton mobility suggest proton delocalization across large water clusters [10,11]. There is ample evidence suggesting that all the above mechanisms are wrong. Such evidence is surveyed below.

2.1. *Free rotors and water structure*

Water is not a free rotor, but rather tightly involved in hydrogen bonding. The local structure around any given water molecule tends to be tetrahedral. There are roughly four water molecules in the first solvation shell, two donating and two accepting hydrogen bonds from the central water. X-ray diffraction yields a clear description of the oxygen–oxygen radial distribution function [21]. Its peaks correspond to nearest-neighbors and next-nearest-neighbors in tetrahedral symmetry. These peaks diminish in amplitude with increasing temperature, indicating a decreasing hydrogen-bonding content. There is evidence for the persistence of even larger ice-structure motifs in liquid water [22]. Likewise, Raman experiments [23] and molecular-dynamics simulations [19] show that in liquid water most molecules are involved in either three or four hydrogen bonds, with the enhancement of tetrahedral symmetry at lower temperatures.

On the other hand, water molecule rotation does correlate with proton mobility. Current interpretations of Raman-induced Kerr effect [23], Rayleigh light scattering [24] and inelastic neutron scattering [25] suggest that water molecule reorientation takes 1–2 ps at room temperature. Proton hopping times as obtained by NMR and ^{17}O resonance [26] are of similar magnitude, $\tau_p \approx 1.5$ ps. These times can explain the abnormal proton mobility, see below. Thus while water molecule reorientation may be an important ingredient in proton mobility, the mechanism is not likely to involve free water rotors as suggested by Bernal and Fowler [4].

2.2. *The relay mechanism and incoherent proton mobility*

The persistence of structural motifs in liquid water does not mean that the relay mechanism is correct and that protons hop in a concerted fashion across large water clusters, Eq. (1). Such a mechanism contradicts so many experimental data that one wonders how it survived that long.

2.2.1. *Proton mobility is incoherent*

The proton does not delocalize over large water clusters.

(i) The relay mechanism predicts that proton mobility increases with increasing size of hydrogen-bonded clusters. Thus mobility should be highest in ice [6], then in water at low temperatures and pressures. Although the issue of proton conductivity in ice is still unsettled, it appears now to be about a factor of two *slower* than in super-cooled water of equal temperature [27,28]. The evidence for proton mobility in water is unequivocal, it *increases* with increasing temperature and pressure [29]. That the fraction of hydrogen-bonded water decreases with increasing temperature is clear intuitively as well as from simulations [19] and experiment [23]. Near atmospheric pressures the hydrogen-bond content decreases with increasing pressure [23], apparently because of transitions from an open water structure to a closed form with fewer interlayer hydrogen bonds [22]. Thus proton mobility increases as the hydrogen bonds weaken.

(ii) The NMR proton hopping times, τ_p, account for the abnormal proton mobility if one assumes that hopping is across a *single* water molecule at a time. Using the Einstein relation for mobility in three dimensions

$$D = l^2/6\tau_p. \tag{2}$$

Meiboom was able to estimate a reasonable proton diffusion coefficient [26]. Let us slightly modify this estimate by taking the hopping length as $l = 2.5$ Å, the hydrogen-bond length between water and H_3O^+ [8], rather than the water–water distance of 2.8 Å. Using $\tau_p = 1.5$ ps gives $D = 7 \times 10^{-5}$ cm^2/s, a very reasonable estimate for the abnormal proton mobility at room temperature (subtract from the proton diffusion coefficient, 9.3×10^{-5} cm^2/s, the water self-diffusion coefficient, 2.3×10^{-5} cm^2/s [1]). Even the most modest coherent effect, with proton hopping across just two water molecules, already leads to a factor of 4 in the predicted mobility. Thus proton mobility is best described as an incoherent, Markovian hopping process.

(iii) Additional evidence for the Markovian nature of the solvated proton comes from excited-state proton transfer to solvent [30]. These transient fluorescence measurements, having time resolution of about 50 ps, can be explained quantitatively by the time-dependent Smoluchowski equation describing proton diffusion in a field of force. The diffusive behavior persists under a wide variety of conditions: solvent composition, salt concentrations and pH values [30]. On even shorter timescales, femtosecond geminate recombination of proton and solvated electron in pure liquid water is interpreted [31] by diffusive proton motion on timescales of just a few ps.

2.2.2. *Proton motion is not rate limiting*

The possibility that proton motion within clusters is rate-limiting may be ruled-out by several considerations:

(i) The deuterium isotope effect on prototropic mobility is rather small, 1.4 at room temperature [1]. Conway and co-workers [5] estimated that proton tunneling should lead to a minimal isotope effect of 2. Westheimer and Melander have suggested that kinetic isotope effects (KIEs) are *maximal* for the symmetric, $\Delta G = 0$, reaction [32]. Proton hopping between two water molecules is expected to correspond to this symmetric limit. In this case, when a light particle is transferred between two heavy particles, the KIE is particularly large. As seen in Fig. 7 of Ref. [33], the maximal KIE for reaction series involving proton transfer is 6–10. The small isotope effect on proton mobility suggests that the reaction coordinate does not involve proton motion.

(ii) While the activation energy of proton mobility is 2–3 kcal/mol [1], proton motion within symmetric entities such as $H_5O_2^+$ may be activationless. Quantum mechanical calculations show that the potential along the proton coordinate in $H_2O \ldots H^+ \ldots OH_2$ has a barrier only if the O–O distance exceeds its equilibrium distance of 2.39 Å [16]. At the equilibrium distance, there is no barrier for proton transfer at all [13]. The proton resides in a flat minimum inbetween the oxygens.

(iii) Intramolecular proton transfer, which *may* be limited by proton tunneling, can be much faster than the 1–2 ps timescale for proton hopping in water. Consider just one model system, 3-hydroxyflavone [34]. Direct proton transfer, observed in non-hydrogen-bonded solvents, or proton hopping across a single solvent bridge (e.g., in methanol) may be faster than 100 fs. Interestingly, in hydrogen-bonded solvents (under conditions that the donor–acceptor are unbridged) intramolecular proton transfer in 3-hydroxyflavone slows down considerably [34]. When solvent conformation is right for proton transfer, its rate can be an order of magnitude *faster* than observed in mobility measurements.

2.2.3. *Cluster-growth is not rate-limiting*

To the contrary, cluster degradation may be the rate-determining step: Eigen has suggested a relay mechanism with 'structural diffusion' as the rate-limiting step [6]. However, the hydrogen-bond content of water is so high, every molecule participating in 3–4 hydrogen bonds [19,23], that the whole body of water may be considered as one huge cluster. Since hydrogen-bond connectivity in liquid water is well above the percolation threshold, it is not clear why the formation of new hydrogen bonds at the periphery of water clusters should be rate-limiting.

2.2.4. *The rate-limiting step is hydrogen-bond cleavage*

Proton mobility correlates with hydrogen-bond cleavage rather than with its formation, as suggested by the fact that it *increases* with decreasing hydrogen-bond content of water, i.e. at higher temperatures and pressures [29]. The NMR hopping times [26] agree with single molecule reorientation times [23–25], a

process requiring hydrogen-bond cleavage.

The temperature dependence of the NMR [10] and proton mobility [1] data is similar, with an activation energy of 2–3 kcal/mol. This agrees with the in situ hydrogen-bond strength of 2.6 kcal/mol obtained from Raman experiments [23]. It represents the *directional* component of the hydrogen-bond enthalpy, associated with water rotation. An additional 2.6 kcal/mol is associated with isotropic electrostatic interactions, leading to a total enthalpy of about 5.2 kcal/mol per hydrogen bond, in agreement with the 10.5 kcal/mol enthalpy of vaporization of water.

The 1.4 D/H isotope effect on proton mobility [1] can also be interpreted as arising from water reorientation that leads to hydrogen-bond cleavage. Rotation around the heavy oxygen is a nearly pure hydrogen motion, so that the isotope effect should be given by the mass ratio, $\sqrt{m_D/m_H} = \sqrt{2}$. The OD...O versus OH...O enthalpy difference is thus expected to be $\Delta\Delta H = \frac{1}{2}RT\ln 2 = 0.2$ kcal/mol at room temperature. This prediction has recently been verified experimentally [23]: the temperature-dependence of the Raman OD and OH stretch bands gives $\Delta H = 2.8$ kcal/mol and 2.6 kcal/mol for deuterium- and hydrogen-bond enthalpies, respectively. While conservative error estimates on ΔH are themselves ± 0.2 kcal/mol, the difference reflected in $\Delta\Delta H$ is real.

2.3. *Proton solvation and proton mobility*

All models are incompatible with elementary considerations of proton solvation. Unlike water which is predominantly tetrahedral the solvated H_3O^+ cation is a nearly plane-trigonal complex (unsolvated, gas-phase H_3O^+ is pyramidal [8]). Unlike water, which is involved in 4 hydrogen bonds as both a donor and an acceptor, the positive H_3O^+ disallows hydrogen-bonding to its oxygen. This cation is best described as donating three hydrogen bonds to 3 water molecules in its first solvation shell. This forms the 'Eigen cation' [6], $H_9O_4^+$, as depicted in Fig. 1.

How does the extra stability of this cation arise [13]? Gas-phase mass-spectrometric data [35] show that water ligands in the first solvation-shell are more tightly bound than those in the second and subsequent shells. The enthalpies for sequential removal of water molecules from gas-phase clusters are summarized in Fig. 1. Although these numbers cannot be

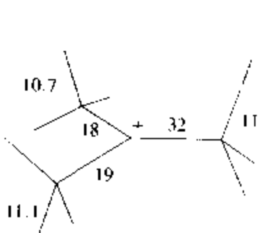

Fig. 1. The structure of $H_9O_4^+$, stressing the different coordination states of central and peripheral oxygens. Each vertex represents an oxygen, and each line a hydrogen bond. Hydrogen atoms are not shown. Assuming a cluster with three second shell waters, the numbers (in increasing magnitude) are the sequential bond-dissociation enthalpies (in kcal/mol) from mass-spectrometric data [35].

directly transferred to bulk solution, it is clear that hydrogen bonding to H_3O^+ is considerably stronger than in bulk water. The extra-strong hydrogen bonding correlates with shorter OH...O distances, 2.55 Å compared with 2.8 Å in bulk water. A proton mobility mechanism that involves the rupture of even one of these three bonds will lead to an activation energy exceeding the observed 2–3 kcal/mol value. The first solvation shell of H_3O^+ must remain intact during the proton transfer act [9].

Although the above trends are anything but surprising, they have been largely ignored. Hückel, for example, has suggested the rotation of H_3O^+ as the rate-limiting step [3]. This requires the rupture of all three hydrogen bonds in the first solvation shell. In addition to the huge activation energy, such a rotation is quite unnecessary, as the three hydrogens in H_3O^+ are equivalent (electronic reorganization being much faster than nuclear motion) and any one of them may serve as the mobile proton [5]. The Bernal–Fowler [4] and Conway models [5], assume that a water in the first hydration shell rotates either freely [4] or through an angle of 110° [5]. This requires not only the rupture of one of the strong hydrogen bonds to H_3O^+, but one or two additional 'ordinary' hydrogen bonds to allow rotation through such large angles. The activation energy for this process may easily exceed 10 kcal/mol.

The proton-relay mechanism is incompatible with solvation requirements for either a gas-phase cluster or a solvated $H_9O_4^+$. In the first case the proton cannot transfer from the center to one of the three peripheral water molecules because it will become solvated by one instead of three hydrogen bonds. Ab initio calcu-

lations indeed report [13] that such a process is uphill by over 13 kcal/mol. The proton is 80% localized at the central H_3O^+ unit [13]. When the $H_9O_4^+$ is solvated, the tetrahedral coordination of the first-shell water ligands (Fig. 1) is unfavorable for proton solvation. Cartoons of hydrogen-bonded networks, which omit the three-dimensional structure as well as some of the hydrogen bonds, may at first look convincing. If, however, one adds the missing hydrogen bonds to the relay scheme of Eq. (1),

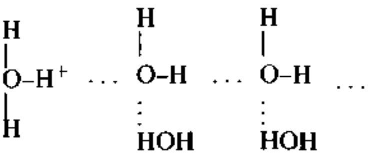
(3)

it becomes clear that the oxygen sites are inequivalent: all are 4-coordinated except the hydronium which is 3-coordinated, so that no relay is possible.

Zundel and Fritsch [7] stressed the importance of the $H_5O_2^+$ cation over that of $H_9O_4^+$. The proton in-between the two water molecules shows extremely high polarizability, leading to the observation of an IR continuum [7]. This 'Zundel polarization' has been reproduced in ab initio calculations [15]. Driven by fluctuations in the water environment which momentarily makes one or the other oxygen more stable, the structure seems to fluctuate between $H_9O_4^+$ and $H_5O_2^+$ [18]. While proton polarization undoubtedly plays an important role in proton mobility, it can only be *part* of the mechanism. First, the triply coordinated $H_9O_4^+$ is probably the more stable protonated cation [16]. Thus, $H_5O_2^+$ is an appropriate symmetric structure for the intrinsic transfer step [16], but it can only be an intermediate along the reaction coordinate for prototropic motion. Second, the above picture misses the pivotal role played by hydrogen-bond cleavage in the rate-determining step.

3. Towards an acceptable prototropic mechanism

The above discussion suggests the following criteria for an acceptable molecular mechanism of prototropic mobility:

– Proton mobility is an incoherent proton hopping process.

– The actual proton motion is much faster than solvent reorganization.

– The rate-limiting step involves cleavage of a single, ordinary hydrogen bond with an activation energy of about 2.6 kcal/mol and a deuterium isotope effect of 1.4.

– The three hydrogen bonds in the first solvation shell of H_3O^+ cannot participate in this step.

– During the elementary transfer step itself, the donor and acceptor water molecules with their solvation shells present equivalent structures.

With these restrictions in mind, it is almost self-evident what the Grotthuss mechanism might be.

Consider a triply coordinated H_3O^+, i.e. the $H_9O_4^+$ cation, as the reactant state. Since the rate-limiting step involves cleavage of a hydrogen bond, but not one in the first solvation shell, it is natural to consider hydrogen-bond cleavage in the *second* solvation shell as the rate-limiting step. This leads to isomerization of the $H_9O_4^+$ cation into $H_5O_2^+$, namely

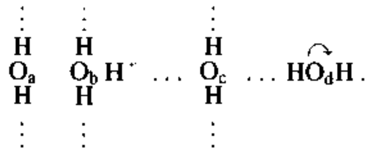
(4)

This is depicted in Fig. 2. For clarity, four of the oxygen atoms are identified by corresponding letters.

Initially, the proton is localized on O_b and H_2O_c is one of the three water ligands in the 1st solvation shell, Fig. 2a. Thus $O_bH \ldots O_c$ is an extra-strong bond which cannot easily break. H_2O_c has at most one hydrogen bond donating ligand, H_2O_d, which is second-shell with respect to O_b and thus of ordinary strength. The cleavage of the $O_c \ldots HO_d$ bond is postulated to be the rate-limiting step, Fig. 2b. The reorientation of H_2O_d may be quite small (much smaller than the 110° rotation required by the Conway mechanism [5]), so that just a single hydrogen bond is broken. This may cost 2–3 kcal/mol and take around 1 ps at room temperature.

Following bond cleavage, one expects ultrafast (fs) readjustment of bond angles and bond lengths to form $H_2O_b \ldots H^+ \ldots O_cH_2$. For example, the $O_b \ldots O_c$ distance should shrink by some 0.15 Å, from 2.55 Å in $H_9O_4^+$ to the 2.4 Å distance of $H_5O_2^+$ (these are gas-phase values). At the same time, the distances between

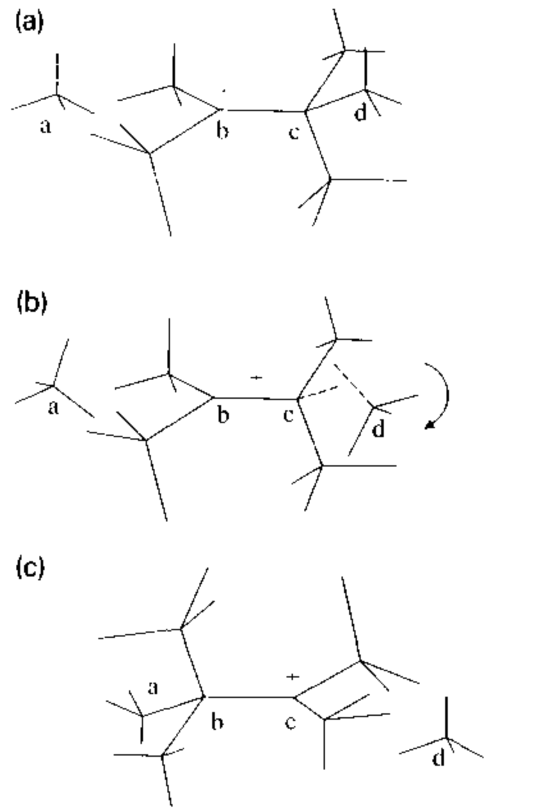

Fig. 2. A molecular mechanism for prototropic mobility. See text for detail.

O_b and the remaining two oxygens are expected to increase from 2.55 Å to below 2.8 Å. Subsequently, fast fluctuations of surrounding water dipoles momentarily stabilize O_b or O_c, leading to large proton polarizabilities within this complex [7,15]. Eventually, one of these fluctuations couples to reorientation of the H_2O_a water, which donates the fourth hydrogen bond to O_b. The proton is now localized on O_c, Fig. 2c. The $H_5O_2^+$ has re-isomerized to a $H_9O_4^+$ cation, centered on a neighboring water molecule. The proton has effectively hopped incoherently across the O–O distance in $H_9O_4^+$.

Proofs of the above mechanism are more likely to come from computational chemistry than from any single experiment. Thus, in their simulation of proton transfer from HCl to water, Ando and Hynes [17] have observed the cleavage of a second-shell hydrogen bond following proton transfer. Tuckerman et al. [18] conducted ab initio molecular dynamics simulations on a 32-water molecule cluster using the Car–Parrinello density-functional technique. The animated trajectories are qualitatively consistent with the above mechanism. It would be interesting to map-out the complete reaction-coordinate and verify that the dominant barrier is in breaking the second-shell hydrogen bond.

Concluding, proton migration may be envisioned as a process propelled by hydrogen-bond cleavage, taking place in front of the moving proton, and hydrogen-bond formation in its back. If an allegoric description is required, it may be found in Moses crossing the sea, with waves parting before him and reclosing behind his track. It would be amusing if this understanding could be put to practical use. Could proton migration be speeded-up by a moving, hydrogen-bond-cleaving IR wave? The 180 cm^{-1} 'intermolecular' band, thought to involve hydrogen-bond stretch [23], might be a useful frequency to try.

Acknowledgement

I am grateful to Victoria Buch-Elber, J. Paul Devlin, Robert S. Eisenberg, Menachem Gutman, James T. Hynes, Gunnar Karlström, Maurice M. Kreevoy, Klaus-Dieter Kreuer, Berta Perlmutter-Hayman, Steve Scheiner, Mark A. Ratner, Michiel Sprik, George E. Walrafen and Georg Zundel for discussions, correspondence and remarks. Work supported in part by The Israel Science Foundation, administered by The Israel Academy of Sciences and Humanities. The Fritz Haber Research Center is supported by the Minerva Gesellschaft für die Forschung, Munich, Germany.

References

[1] R.A. Robinson and R.H. Stokes, Electrolyte solutions, 2nd Ed. (Butterworths, London, 1959);
T. Erdey-Grúz, Transport phenomena in aqueous solutions (Adam Hilger, London, 1974).
[2] C.J.D. von Grotthuss, Ann. Chim. LVIII (1806) 54.
[3] E. Hückel, Z. Electrochem. 34 (1928) 546.
[4] J.D. Bernal and R.H. Fowler, J. Chem. Phys. 1 (1933) 515.
[5] B.E. Conway, in: Modern aspects of electrochemistry, Vol. 3, eds. J.O'M. Bockris and B.E. Conway (Butterworths, London, 1964) p. 43.
[6] M. Eigen, Ang. Chem. Intern. Ed. 3 (1964) 1.

[7] G. Zundel and J. Fritsch, in: The chemical physics of solvation, Vol. 2, eds. R.R. Dogonadze, E. Kálmán, A.A. Kornyshev and J. Ulstrup (Elsevier, Amsterdam 1986) ch. 2.
[8] F.H. Stillinger, in: Theoretical chemistry advances and perspectives, Vol. 3, eds. H. Eyring and D. Henderson (Academic Press, New York, 1978) p. 177.
[9] B. Halle and G. Karlström, J. Chem. Soc. Faraday Trans. II 79 (1983) 1047.
[10] R. Pfeifer and H.G. Hertz, Ber. Bunsenges. Physik. Chem. 94 (1990) 1349.
[11] C.A. Chatzidimitriou-Dreismann and E.J. Brändas, Ber. Bunsenges. Physik. Chem. 95 (1991) 263.
[12] K.D. Kreuer, Th. Dippel, N.G. Hainovsky and J. Maier, Ber. Bunsenges. Physik. Chem. 96 (1992) 1736.
[13] M.D. Newton and S. Ehrenson, J. Am. Chem. Soc. 93 (1971) 4971.
[14] S. Scheiner, Accounts Chem. Res. 18 (1985) 174.
[15] D. Borgis, G. Tarjus and H. Azzouz, J. Chem. Phys. 97 (1992) 1390.
[16] T. Komatsuzaki and I. Ohmine, Chem. Phys. 180 (1994) 239.
G. Corongiu, R. Kelterbaum and E. Kochanski, J. Phys. Chem. 99 (1995) 8038.
[17] K. Ando and J.T. Hynes, in: Structure, energetics and reactivity in aqueous solution, eds. C. J. Cramer and D. G. Truhlar (ACS Books, Washington, 1994);
A. Warshel, J. Am. Chem. Soc. 86 (1982) 2218.
[18] M. Tuckerman, K. Laasonen, M. Sprik and M. Parrinello, J. Phys. Chem. 99 (1995) 5749.
[19] W.L. Jorgensen, J. Chem. Phys. 77 (1982) 4156.
[20] P.W. Atkins, Physical chemistry, 2nd Ed. (Oxford Univ. Press, Oxford, 1982).
[21] A.H. Narten and H.A. Levy, J. Chem. Phys. 55 (1971) 2263.
[22] C.M. Davis Jr. and T.A. Litovitz, J. Chem. Phys. 42 (1965) 2563.
[23] G.E. Walrafen, M.R. Fisher, M.S. Hokmabadi and W.-H. Yang, J. Chem. Phys. 85 (1986) 6970;
E.W. Castner Jr., Y.J. Chang, Y.C. Chu and G.E. Walrafen, J. Chem. Phys. 102 (1995) 653;
G.E. Walrafen, W.-H. Yang, Y.C. Chu and M.S. Hokmabadi, J. Phys. Chem., in press.
[24] C.J. Montrose, J.A. Bucaro, J. Marshall-Coakley and T.A. Litovitz, J. Chem. Phys. 60 (1974) 5025;
O. Conde and J. Teixeira, J. Phys. (Paris) 44 (1983) 525.
[25] M.-C. Bellissent-Funel and J. Teixeira, J. Mol. Struct. 250 (1991) 213.
[26] S. Meiboom, J. Chem. Phys. 34 (1961) 375.
[27] L. Onsager, in: Physics and chemistry of ice, eds. E. Whalley, S.J. Jones and L.W. Gold (Royal Society of Canada, Ottawa, 1973) p. 10.
[28] M. Kunst and J.M. Warman, J. Phys. Chem. 87 (1983) 4093.
[29] E.U. Franck, D. Hartmann and F. Hensel, Discussion Faraday Soc. 39 (1965) 200;
R.A. Horne, J. Electrochem. Soc. 112 (1965) 857.
[30] E. Pines, D. Huppert and N. Agmon, J. Chem. Phys. 88 (1988) 5620;
N. Agmon, D. Huppert, A. Masad and E. Pines, J. Phys. Chem. 95 (1991) 10407; 96 (1992) 2020 (E);
D. Huppert, S.Y. Goldberg, A. Masad and N. Agmon, Phys. Rev. Letters 68 (1992) 3932.
[31] Y. Gauduel, S. Pommeret, A. Migus and A. Antonetti, J. Phys. Chem. 93 (1989) 3880.
[32] L. Melander, Isotope effects on reaction rates (Ronald Press, New York, 1960);
F.H. Westheimer, Chem. Rev. 61 (1960) 265.
[33] N. Agmon, Intern. J. Chem. Kinetics 13 (1981) 333.
[34] P.F. Barbara, P.K. Walsh and L.E. Brus, J. Phys. Chem. 93 (1989) 29;
B.J. Schwartz, L.A. Peteanu and C.B. Harris, J. Phys. Chem. 96 (1992) 3591.
[35] Y.K. Lau, S. Ikuta and P. Kebarle, J. Am. Chem. Soc. 104 (1982) 1462;
M. Meot-Ner, J. Am. Chem. Soc. 108 (1986) 6189;
Z. Shi, J.V. Ford, S. Wei and A.W. Castleman Jr., J. Chem. Phys. 99 (1993) 8009.

Uptake of Nitrogen Dioxide and Nitrous Acid on Aqueous Surfaces

Stephan Mertes* and Andreas Wahner

Institut für Atmosphärische Chemie, Forschungszentrum Jülich GmbH, Postfach 1913, D-52425 Jülich, Germany

Received: November 10, 1994; In Final Form: June 21, 1995[®]

The uptake of gaseous nitrogen dioxide (NO_2) and nitrous acid (HNO_2) on aqueous surfaces is determined using a liquid jet technique. Both the loss of the two species from the gas phase and the formation of nitrite and nitrate in the liquid phase are measured. The experimental results are compared with two-dimensional model calculations simulating transport and chemical processes in the gas and liquid phase. The measured uptake of NO_2 is much larger than that calculated from the model and literature values for the solubility of NO_2 in water indicating, as all other experimental evidence, a reaction of NO_2 on the aqueous surface. The measurements also indicate a partial release of HNO_2 from the surface to the gas phase. The comparison of experiment and model yields a lower limit of the mass accommodation coefficient of NO_2 of $\alpha \geq 2 \times 10^{-4}$ at 278 K surface temperature. For the mass accommodation coefficient of HNO_2 a range from 4×10^{-3} to 4×10^{-2} at 278 K surface temperature is obtained.

Downloaded via NANJING TECH UNIV on February 1, 2025 at 06:58:55 (UTC).
See https://pubs.acs.org/sharingguidelines for options on how to legitimately share published articles.

Introduction

Uptake on aqueous surfaces and subsequent chemical reaction in the bulk liquid or on the surface can play an important role in the removal and conversion of soluble trace gases. One example of such heterogeneous reactions is the conversion of nitrogen dioxide (NO_2) to nitrous acid (HNO_2) on wet aerosol surfaces observed at night in the polluted troposphere[1-6] and attributed to reactions 1 and 2, since the analogous reactions in the gas phase are too slow to explain the observations:[7,8]

$$2NO_2(g) + H_2O(a) \rightarrow HNO_2(g/a) + HNO_3(a) \quad (1)$$

$$NO_2(g) + NO(g) + H_2O(a) \rightarrow 2HNO_2(g/a) \quad (2)$$

$$HNO_3(a) \leftrightarrow H^+ + NO_3^- \text{ and } HNO_2(a) \leftrightarrow H^+ + NO_2^-$$

(a) aqueous phase; (g) gas phase;
(g/a) gas phase or aqueous phase

Investigations of these heterogeneous reactions in smog chamber studies show that they proceed fast on chamber surfaces.[6,9-11] To quantify some possibly rate limiting steps, we investigate in this paper the uptake rate of gaseous NO_2 and HNO_2 into liquid water using a liquid jet technique. At the same time we observe the release of HNO_2 induced by the uptake of NO_2.

The uptake can be described as a series of steps (Figure 1). Molecules of type A are transported from the gas phase to the surface by diffusion. Once within a distance of one mean free path they can collide gas kinetically with the surface. A fraction of the collisions result in sticking of molecule A to the surface. The sticking probability is called the mass accommodation coefficient α. The molecules on the surface can be reemitted to the gas phase, react to form another species, B, or enter the liquid phase. Inside the liquid phase diffusive transport and chemical reaction occur. The parameters which control the transfer rate are the diffusion coefficient in the gas phase, the mass accommodation coefficient, the diffusion coefficient in the aqueous phase, aqueous reaction rate, and solubility of the gas in the liquid phase. Schwartz[12] defined characteristic times for each of these steps for drops of spherical geometry. They can be used to estimate the rate-limiting step. Under tropospheric conditions and in the absence of any saturation effect, the uptake of trace gases into cloud droplets depends on the mass accommodation coefficient α as long as $\alpha \leq 10^{-2}$. For $\alpha > 10^{-2}$ the phase transfer is controlled by diffusion in the gas phase and is independent of α (see Schwartz[12] for details).

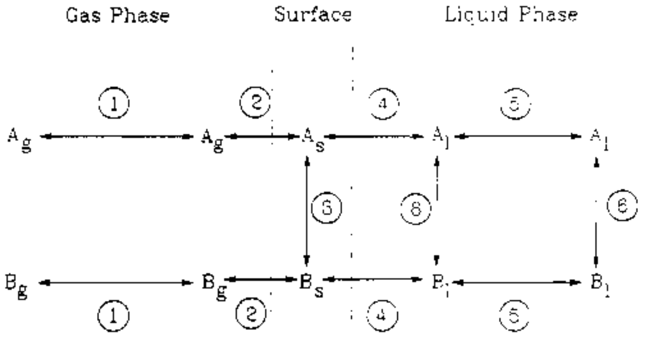

Figure 1. Illustration of the mass transport between gas and liquid phase as a series of in principal reversible steps: (1) gas-phase diffusion, (2) collisions on the surface, (3) surface reaction, (4) hydration into the liquid phase, (5) liquid-phase diffusion, and (6) chemical decomposition in the liquid phase.

Experimental Setup and Measurements

The uptake and reactions of NO_2 and HNO_2 on aqueous surfaces were investigated in a microjet experiment (Figure 2), adapted from the one developed by Kirchner et al.[13] It allows the measurement of the loss of NO_2 and HNO_2 from the gas phase as well as the increase of nitrate (NO_3^-) and nitrite (NO_2^-) in the liquid phase (reactions 1 and 2).

The jet emerges from a capillary (i.d. = 100 μm) at the lower end of a temperature controlled glass tube and is caught by a second capillary (i.d. = 150 μm) which is connected to a movable tube. The liquid is collected in an attached glass cell. The flow rate through the collecting capillary is precisely regulated by controlling the gas-pressure difference between

* Present address: Institut für Troposphärenforschung e.V., Permoserstrasse 15, D-04304 Leipzig, Germany

[®] Abstract published in *Advance ACS Abstracts,* August 1, 1995.

0022-3654/95/2099-14000$09.00/0 © 1995 American Chemical Society

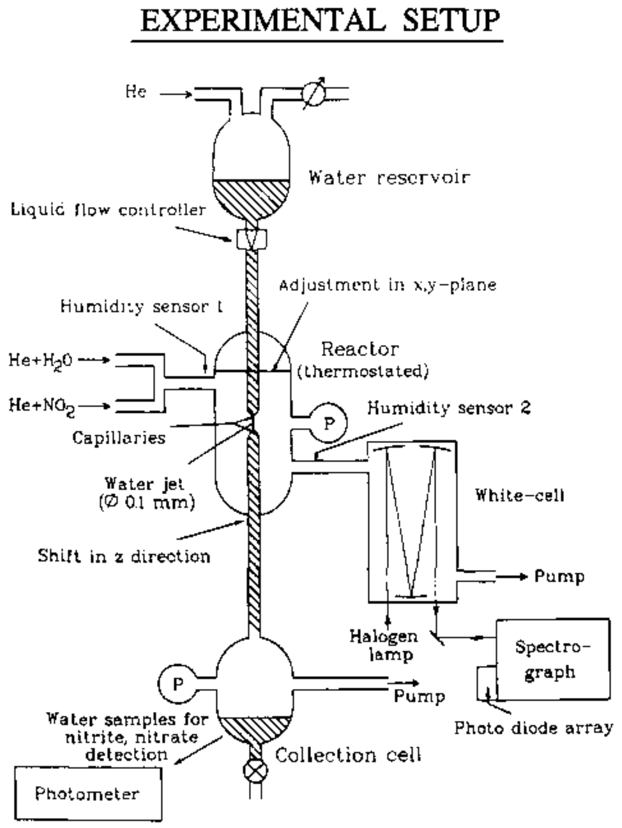

Figure 2. Experimental setup. Generation of the water jet and the interaction with the gas-phase molecules occurs in the reactor. The gas-phase concentrations are measured by DOAS at the exit of the reactor. Water samples for liquid-phase analysis are taken from the collection cell below the reactor. For further details see text.

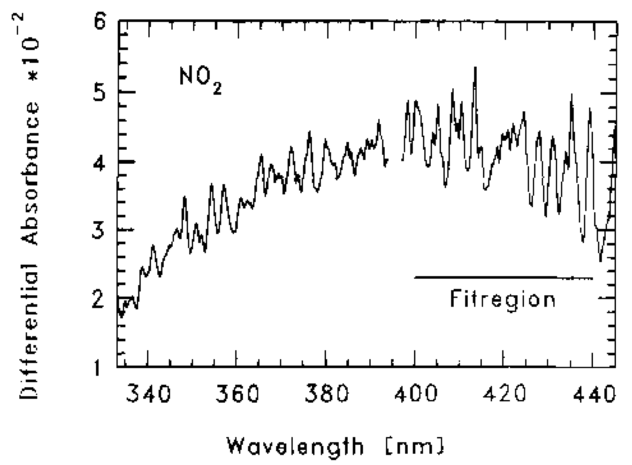

Figure 3. Experimental absorption spectrum dominated by NO_2 in the wavelength range of 335–445 nm. The horizontal line between 400 and 440 nm indicates the undisturbed fit region of the NO_2 reference spectra.

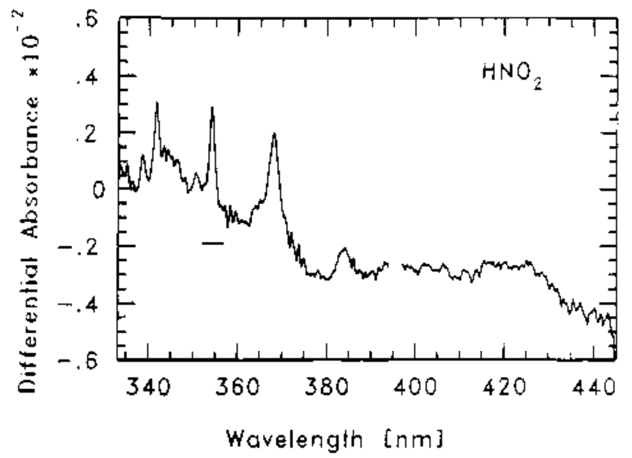

Figure 4. Experimental absorption spectrum of HNO_2 after subtracting the absorption of NO_2 from the measured spectrum. The analyzed absorption line at 354.2 nm is indicated by the horizontal line.

reactor and collection cell in order to avoid any water spills or sucking of gas into the capillary. The capillaries are aligned by sliding the reactor dome in $x-y$ directions on a flat joint. The jet length can be varied by moving the collecting tube in the z direction. This allows to change the contact time of the jet surface with the gas phase. The fast flow of the jet (424 cm/s) continuously renews the aqueous surface. The experiments are carried out at reduced pressure (310–360 hP). The gas entering the reactor above the jet is a mixture of humidified helium (Messer Griesheim, 99.995%) as carrier gas and the trace gas NO_2 (Messer Griesheim, 1.0% NO_2 in He). HNO_2 is formed by reaction of nitrogen dioxide with adsorbed water on the glass surfaces of the reactor and is present in the reactor at a gas phase concentration of 4–6% of the NO_2 concentration. By adjusting the water vapor partial pressure of the gas flow to 100% relative humidity (RH) at jet temperature, net condensation or evaporation of water vapor is avoided. By simultaneous measurements of the gaseous H_2O concentration at the inlet and outlet of the reactor, the effect of small deviations of the relative humidity ($\Delta < 2\%$ RH) on the jet temperature is accounted for by calculating the corresponding change of the jet surface temperature.[14]

The gas outlet of the reactor is located below the jet and connected to a White cell with glass walls. The gas is analyzed for gaseous NO_2 and HNO_2 by differential optical absorption spectroscopy (DOAS). The main components of the optical detection system are a halogen lamp, a White cell (light path of 6 m) and a spectrograph with a photodiode array in the focal plane as detector (Figure 2). Absorption spectra are recorded in the wavelength range 335–445 nm with a resolution of 0.36 nm. The noise level of about 1×10^{-4} absorbance results in a detection limit of approximately 2×10^{12} cm^{-3} for both NO_2 and HNO_2. The determination of the NO_2 concentration is carried out by fitting reference spectra[15] to the experimental absorption spectra (Figure 3) in the range 400–440 nm. Subtraction of the NO_2 component in the spectra reveals the HNO_2 absorption features between 335 and 390 nm (Figure 4). The strong line at 354 nm is used to determine the concentration of nitrous acid by means of the differential UV absorption cross section given by Bongartz et al.[16] The detection limit (3σ) for NO_2 is 4×10^{12} cm^{-3} and for HNO_2 2×10^{12} cm^{-3} using a light path of 106 ± 2 cm. Samples of collected jet water are analyzed for nitrite and nitrate concentrations. The nitrite concentration is measured after a chemical conversion[17] with sulfanilamide and naphthylamine to an azotype dye, which is photometrically detected at 544 nm. The detection limit is about 0.07 μmol/L and therefore a factor of approximately 10 lower than typical concentrations measured. The nitrate is first reduced to nitrite by a Cu/Cd column and then analyzed as described above.

To discriminate the uptake on the jet surface against uptake by the internal surfaces of the reactor, a differential measurement method is used by switching the jet on and off. The difference of the gas phase concentrations between the on and off phase is attributed to the uptake by the jet, since all other experimental parameters stay unchanged. The periods are repeated 10–15 times and each period lasts for about 5–20 min. Only those absorption spectra are used for the determination of the concentration where the difference of the relative humidity at

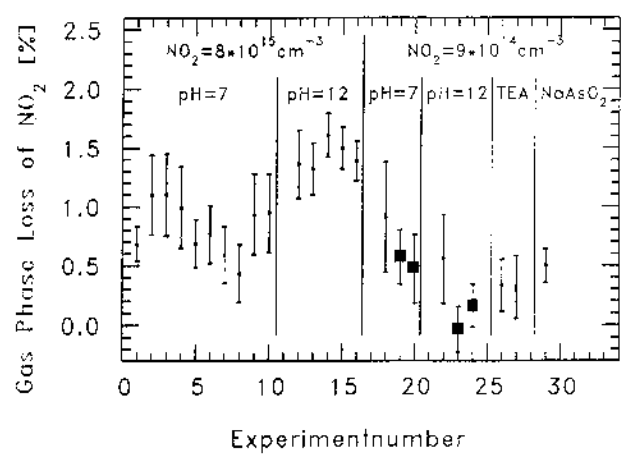

Figure 5. Relative experimental loss of NO_2 from the gas phase plotted for two different NO_2 concentrations and different jet water compositions (pH 7, milli-Q; pH 12, 0.01 N NaOH solution; TEA, 33.4 g/L of triethanolamine solution; $NaAsO_2$, 1 g/L of $NaAsO_2$ solution). Dots, 3 mm jet length; squares, 6 mm jet length.

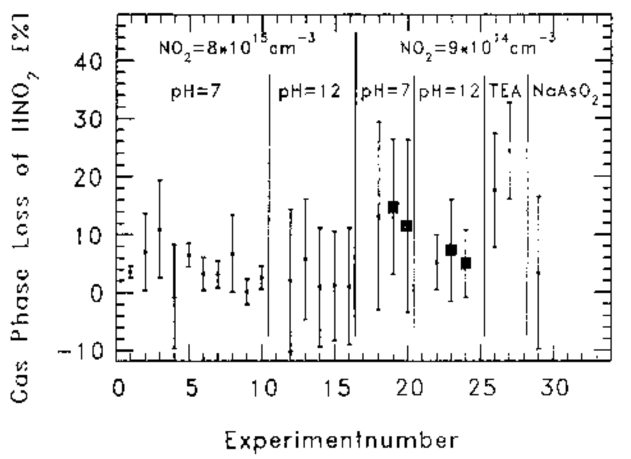

Figure 6. Relative experimental loss of HNO_2 from the gas phase plotted for the different NO_2 concentrations and different jet water compositions (pH 7, milli-Q; pH 12, 0.01 N NaOH solution; TEA, 33.4 g/L of triethanolamine solution; $NaAsO_2$ 1 g/L of $NaAsO_2$ solution). Dots, 3 mm jet length; squares, 6 mm jet length.

TABLE 1: Typical Experimental Conditions

reactor diameter	24 mm
reactor pressure	320–360 hP
reactor temperature	298 K
mean gas flow rate	8.5 cm^3/s
mean gas velocity in reactor	2 cm/s
mean gas contact time with jet	0.16 and 0.32 s
partial pressure of H_2O	7.0–8.6 hP
reynolds number *Re* (gas phase)	15
mean NO_2 concentrations	8×10^{15} cm^{-3}; 9×10^{14} cm^{-3}

TABLE 2: Typical Jet Parameter

jet radius	50 μm
jet length	3 and 6 mm
mean jet velocity	424 cm/sec
jet temperature	276–282 K
jet flow rate	2 mL/min
mean jet contact time with gas phase	0.7 and 1.4 ms
Reynolds number *Re*	320
pH	7 and 12
used solutions milli-Q	
NaOH	0.01 N
triethanolamine (TEA)	33.4 g/L
$NaAsO_2$	1 g/L

the inlet and outlet of the reactor is less than 2% relative humidity. Great care was taken to avoid any condensation of water on the walls of the reactor or tubing by thermostating these parts. The change of the gas phase concentration of HNO_2 introduced by the jet surface is also attributable to an uptake by the jet surface, because the gas phase concentration of HNO_2 is reproducible and shows only slow variations of less than 15% during 1 day whenever the water jet is turned off. The analysis of the liquid phase concentrations enables the calculation of the mass balance of the uptake. Typical experimental conditions and jet parameters are listed in Tables 1 and 2.

A total of 150 differential measurements were evaluated. To improve the scatter, successive measurements were averaged. In Figures 5 and 6 the averaged data of the measured loss of NO_2 and HNO_2 from the gas phase is plotted for different experimental conditions such as NO_2 concentration, jet water pH, and jet length (6 mm in the experiment number 19, 20, 23, and 24, otherwise 3 mm). The error bars indicate the 3σ statistical variation. These statistical variations are larger than the detection limit of NO_2 and HNO_2 due to the reproducibility of the jet during on and off periods. This is mainly due to the small fluctuations of the relative humidity (less than 2% relative humidity). Larger effects on the NO_2 gas-phase concentration

TABLE 3: Mean Relative Losses of NO_2 and HNO_2 from the Gas Phase together with the NO_2 and HNO_2 Concentrations

$[NO_2]$ (cm^{-3})	$[HNO_2]$(cm^{-3})	ΔNO_2 (%)	ΔHNO_2 (%)
8×10^{15}	4×10^{14}	1.03 ± 0.34	3.9 ± 3.0
9×10^{14}	2×10^{13}	0.42 ± 0.26	11.3 ± 6.5

TABLE 4: Mean Nitrite and Nitrate Concentrations of the Jet Water Samples for the Two pH Values and a NO_2 Concentration of 9×10^{14} cm^{-3}

pH	$[NO_2^-]$ (μmol/L)	$[NO_3^-]$ (μmol/L)
7	0.84 ± 0.28	0.89 ± 0.42
12	1.09 ± 0.57	1.22 ± 0.57

can be observed if water is spilled in the reactor. These measurements are discarded. Experiments 26 and 27 were carried out with added triethanolamine (TEA), experiment 29 was carried out with added $NaAsO_2$ to the water of the jet. These chemicals are used in order to speed up the liquid phase conversion of dissolved NO_2 to nitrate.[18,19]

Experimental Results

The measured uptake of gaseous NO_2 shown in Figure 5 is significant under all experimental conditions and leads to a decrease up to 1.6% of the gaseous NO_2 concentration. Within the experimental uncertainty (3σ) the observed decrease is independent of the jet water composition and the jet length. On average, the relative loss of NO_2 from the gas phase is correlated to the NO_2 concentration as can be seen from Table 3: taking the average of all measurements at pH 12 and 7 the decrease in the gas phase rises from 0.42% to 1.03% when the NO_2 concentration is increased from 9×10^{14} cm^{-3} to 8×10^{15} cm^{-3}.

Figure 6 shows the measured loss of HNO_2 from the gas phase. It is significant and amounts up to 25%. Within the large scatter of the HNO_2 gas-phase concentration, no correlation with the jet water composition and the jet length can be detected. However, the averaged loss of HNO_2 from the gas phase is clearly anticorrelated to the NO_2 concentration within 2σ errors, i.e., the loss of HNO_2 is smaller for higher NO_2 concentrations (Table 3).

The amount of nitrite and nitrate in the liquid phase is found to be equal within the experimental errors (Table 4). This is expected in case of the disproportionation of NO_2 via the formation of the intermediate dimer N_2O_4 to nitrite and nitrate. The uncertainty of the experimental values does not exclude a

TABLE 5: Diffusion Coefficients and Henry Constants of NO_2 and HNO_2 Used in the Model ($H(T)$ as a Function of the Temperature Taken from Chameides[84])

	NO_2	HNO_2
D_g (cm^2/s)	1.8	1.8
D_a (cm^2/s)	1.85×10^{-5}	1.85×10^{-5}
H (mol/(L/atm))	2.2×10^{-2}	155
$H(T)$ (mol/(L/atm))	1.2×10^{-2} exp[2500(1/T − 1/298)]	4.9×10^{1} exp[4781(1/T − 1/298)]

surplus of nitrite in the jet water samples possibly caused by the uptake of nitrous acid and its fast dissociation to nitrite. Because the gas-phase concentration of HNO_2 is more than a factor of 10 lower than the NO_2 concentration, the contribution to the liquid-phase concentration of nitrite is expected to be small compared to our error limits.

The uptake of gaseous N_2O_4 is not significant. Due to the equilibrium constant[20] of N_2O_4 formation ($K = 6.84$ atm^{-1}) the gas phase concentration of N_2O_4 amounts to 0.025% of the NO_2 concentration. Though the Henry constant[21] of N_2O_4 ($H(N_2O_4)$ = 1.4 mol/(L atm)) is a factor 100 larger than that of NO_2 ($H(NO_2) = 1.2 \times 10^{-2}$ mol/(L atm)), the resulting maximum N_2O_4 interference in the form of nitrate and nitrite formation is less than 4%. The contribution of gaseous HNO_3 uptake to the nitrate concentration can be assumed to be small. According to laboratory experiments, which investigated the kinetics of the H_2O/NO_2 system using similar kinds of reactors,[6,9-11] no gas-phase HNO_3 has been detected. The detection limit for HNO_3 is reported to be a factor 100 lower than the measured HNO_2 concentrations.

The nitrogen mass balance is used as a further test of the consistency of the experimental results. The loss rates of NO_2 and HNO_2 and the formation rate of NO_2^- and NO_3^- in molecules per second are given by $R_1 = F_g\Delta[NO_2]$, $R_2 = F_g\Delta[HNO_2]$, $R_3 = F_a[NO_2^-]$ and $R_4 = F_a[NO_3^-]$, where F_g and F_a are the gas and liquid flow rates. The condition of mass conservation, i.e., $R_1 + R_2 = R_3 + R_4$, is fulfilled in 80% of the measurements within the error limits of each measurement. The average deviation (3σ) of ($R_1 + R_2$) − ($R_3 + R_4$) from zero of all measurements is $(0.35 \pm 1.8) \times 10^{13}$ molecules s^{-1}. This corresponds to approximately (0.05 ± 0.25)% of the NO_2 gas-phase concentration and approximately to (5 ± 25)% of the NO_2 gas-phase loss. Although the errors of the NO_2, HNO_2, NO_2^-, and NO_3^- individual concentration measurements are rather large (up to 50%), this result indicates that the major loss processes of NO_2 are accounted for.

Model Calculations

The rate of uptake from the gas phase into the liquid jet is controlled by several processes (e.g., diffusion, mass accommodation, chemical reaction). To determine the mass accommodation coefficient from the measured uptake a numerical simulation of the chemistry in the aqueous phase and of the transport and diffusion processes in the gas and aqueous phase is carried out and compared with the experimental data. Other than the mass accommodation, no specific chemical reaction on the surface is included in the model. The numerical model is programmed within FACSIMILE, a 3-dimensional transport and chemistry solver using Gear routines for differential equations. The model treats the water jet as an ideal cylinder with a surface velocity equal to the mean jet velocity and uses the radial grid sizes of 0.0032 mm in the liquid phase and 0.42 mm in the gas phase and the axial grid size of 0.21 mm in both phases. The transport within each of the homogeneous phases is described by diffusion and convection as given by solutions of the generalized diffusion equation (eq 3) in cylindrical geometry with convection along the jet in the z direction only:

$$\frac{\partial C}{\partial t} = D\left[\frac{1}{r}\frac{\partial}{\partial r}\left(r\frac{\partial C}{\partial r}\right) + \frac{\partial^2 C}{\partial z^2}\right] - v_z\frac{\partial C}{\partial z} \quad (3)$$

where C is the concentration, D the diffusion coefficient either in the gas phase (D_g) or in the liquid phase (D_a), r the radius, and v_z the vertical velocity in the gas phase or liquid phase.

The gas-phase diffusion coefficient D_g of NO_2 is determined by eq 4, where P_{H_2O}, P_{He}, P_r, and T_r denote the partial pressure

$$\frac{1}{D_g} = \frac{1}{P_r}\left(\frac{P_{H_2O}}{D_{NO_2-H_2O}(P_r,T_r)} + \frac{P_{He}}{D_{NO_2-He}(P_r,T_r)}\right) \quad (4)$$

of H_2O and He, the total pressure in the reactor and the reactor temperature. $D_{NO_2-H_2O}$ and D_{NO_2-He} are the binary gas-phase diffusion coefficients of NO_2 in H_2O and He, respectively, which are derived from calculations[22] of $D_{SO_2-H_2O}$ and D_{SO_2-He}. For the liquid-phase diffusion coefficient D_a a linear temperature dependence is assumed.[23] Values of D_g and D_a which are used in the model for NO_2 and HNO_2 are given in Table 5. Reduced pressure and the use of He as the carrier gas enhances the diffusive transport by one order of magnitude compared to N_2 and 1 atm pressure.

Relative slow flow velocities are used in the gas phase in order to observe a NO_2 gas-phase loss. Due to the different flow velocities in the gas phase and liquid phase, two different flow profiles of the gas phase, $v_f(r)$ and $v_p(r)$, are used to account for two extreme assumptions (Figure 7). The flow profile v_f assumes that the gas at the aqueous surface adopts the jet flow velocity as a result of the friction between the gas and the aqueous surface. It drops linearly to zero with increasing radius r. The flow profile v_p assumes no friction at the interfaces jet surface/gas and reactor wall/gas and is therefore independent of r. Both flow profiles are normalized to the mean experimental gas flow rate F_g; therefore, v_f drops to zero at a distance of 1.4 mm from the surface (Figure 7). The flow profile v_f shows larger velocities close to the jet than v_p. If the gas-phase transport is the rate-limiting step of the uptake, the calculations using flow profile v_f will result in a larger uptake. The real flow profile is very difficult to determine, but it will be shown that the influence of the flow profile in this investigation is small.

The rate of exchange between the gas phase through the heterogeneous interface and the liquid phase, $F_{g\rightarrow a}$, is implemented by taking the number of gas kinetic collisions on the surface multiplied by the mass accommodation coefficient α. Equation 5 describes the net flow across the jet surface:

$$F_{g\rightarrow a} = \alpha\frac{v_m}{4}\left(C^s_g - \frac{C^s_a}{4HRT}\right) \quad (5)$$

where v_m is the mean speed of molecules, H the Henry constant, C^s_g the gas-phase concentration at the surface, and C^s_a the liquid-phase concentration at the surface.

The Henry constants of NO_2 and HNO_2 are calculated using the temperature dependent expressions[24] given in Table 5. The so obtained Henry constant of NO_2 at room temperature agrees within a factor of 2 with the results of other authors, who

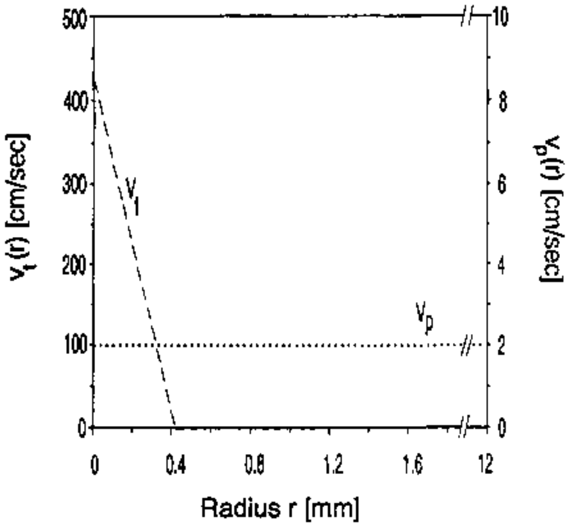

Figure 7. Gas flow profiles v_p (dotted line, right scale) and v_t (dashed line, left scale) used in the model calculations as a function of the reactor radius r (jet surface at $r = 0$ mm). Both profiles are normalized to the mean experimental flow rate.

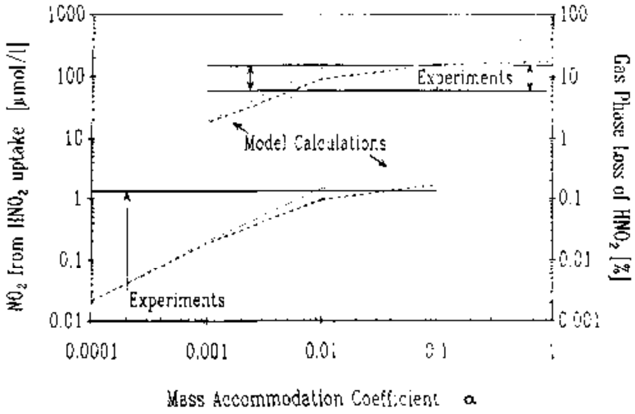

Figure 8. Calculated nitrite formation from HNO_2 uptake (left scale, lower curves) and loss of HNO_2 from the gas phase (right scale, upper curves) as a function of the mass accommodation coefficient using the flow profiles v_p (dashed line) and v_t (dotted line) at 3 mm jet length. These calculations are compared to the maximum surplus of NO_2 estimated from the uncertainty of the liquid-phase analysis (right side) and to the observed range of HNO_2 loss from the gas phase (left side).

determined H experimentally[23,25] or by thermodynamical calculations.[21,26,27]

No gas-phase or wall reactions are used in the model, because the measurement of the uptake are differential concentration measurements during jet off and on periods and the effect of such reactions would essentially be canceled. The transformation of dissolved NO_2 and HNO_2 to NO_2^- and NO_3^- in the liquid phase is calculated according to the following reactions:[28]

$$2NO_2 \leftrightarrow N_2O_4 \qquad K_6 = 6.5 \times 10^4\ M^{-1} \tag{6}$$

$$N_2O_4 + H_2O \rightarrow HNO_2 + HNO_3 \qquad k_7 = 1 \times 10^3\ s^{-1} \tag{7}$$

$$HNO_2 \leftrightarrow H^+ + NO_2^- \qquad K_8 = 4.4 \times 10^{-4}\ M \tag{8}$$

$$HNO_3 \leftrightarrow H^+ + NO_3^- \qquad K_9 = 15.4\ M \tag{9}$$

Comparison of Experimental and Model Results

Figure 8 shows the calculated loss of HNO_2 from the gas phase and the nitrite concentration due to HNO_2 uptake as a

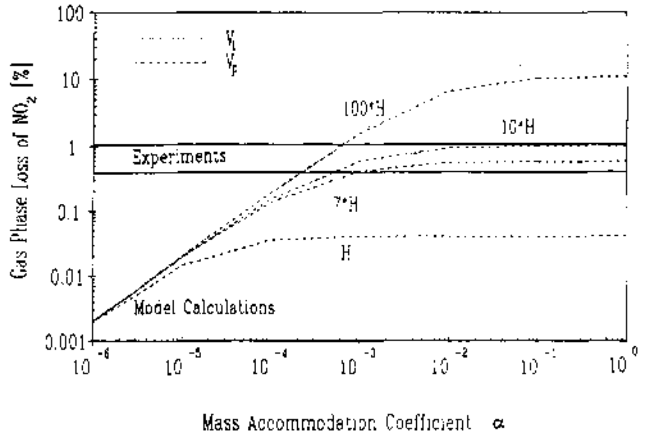

Figure 9. Calculated loss of NO_2 from the gas phase at 3 mm jet length as a function of the mass accommodation coefficient a using the flow profiles v_p (dashed line) and v_t (dotted line) for different modified Henry constants H_m given as a multiple of the physical Henry constant. These calculations are compared to the experimentally observed range of NO_2 loss from the gas phase.

function of the mass accommodation coefficient α for the two different flow profiles v_t (dotted lines) and v_p (dashed lines) and a jet length of 3 mm. For $\alpha \geq 10^{-3}$ the slopes of the curves decrease and begin to separate for the different gas flow profiles, reflecting the increasing influence of gas-phase transport (diffusion and convection) on the uptake rate of HNO_2. Under the current experimental conditions the liquid-phase concentration of the jet does not reach solution equilibrium with the gas-phase concentration even for $\alpha = 1$. The surface concentration of HNO_2 is about 5 orders of magnitude lower than solution equilibrium due to the Henry constant at 278 K and because at the used HNO_2 concentrations HNO_2 is dissociated by K_8. The calculated loss of HNO_2 from the gas phase is sensitive to a mass accommodation coefficient for $\alpha \leq 0.1$. Also shown in Figure 8 are the experimental results: the loss of HNO_2 from the gas phase as a range between the upper two horizontal lines; the liquid phase nitrite concentration from HNO_2 uptake as a horizontal line, indicating the maximum surplus of NO_2^- estimated from the uncertainty of liquid-phase analysis. Comparison of the model calculation and the experimental results for the liquid phase shows that only for a mass accommodation coefficient $\alpha \leq 4 \times 10^{-2}$ the calculated nitrite from HNO_2 uptake agrees with the measured range. The same comparison for the loss of HNO_2 from the gas phase results in a lower limit of $\alpha \geq 4 \times 10^{-3}$, taking the differences due to the two flow profiles into account. The obtained range of the mass accommodation coefficient of HNO_2 on aqueous surfaces at 278 K is consistent with the lower limit of $\alpha \geq 5 \times 10^{-3}$ determined by Kirchner et al.[13] and with the range measured by Bongartz et al.[29] of $0.04 < \alpha < 0.09$ at 245 K and $0.03 < \alpha < 0.15$ at 297 K.

Model calculation of the loss of nitrogen dioxide from the gas phase and of the sum of nitrite and nitrate formation caused by the uptake of NO_2 are plotted as a function of the mass accommodation coefficient α in Figure 9 and 10. The calculations are carried out varying the Henry constant H_m (given in multiples of the physical Henry constant H (cf. Table 6)), and using the two different gas flow profiles v_t (dotted line) and v_p (dashed lines). For small values of α the loss from the gas phase is most sensitive to the rate of interfacial mass transfer. For larger values of α the slope of the curves decreases due to the saturation of the surface. The low physical solubility of NO_2 and the resulting saturation limits the transfer from the gas phase and the uptake becomes independent of α. Using a larger Henry constant, H_m, the influence of saturation is apparent

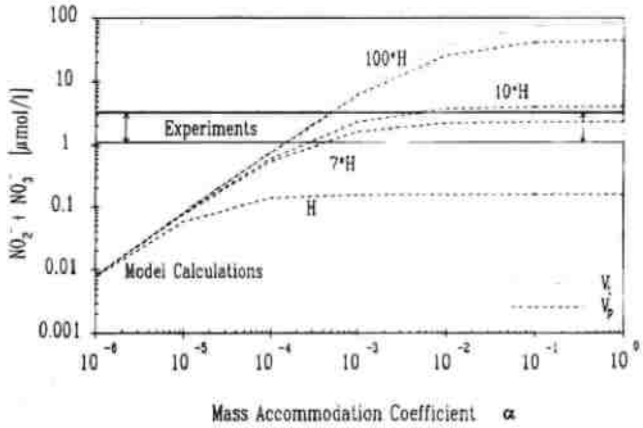

Figure 10. Calculated sum of nitrite and nitrate formation in the liquid phase due to NO_2 uptake as a function of the mass accommodation coefficient a using the flow profiles v_p (dashed line) and v_t (dotted line) for different modified Henry constants H_m given as a multiple of the physical Henry constant. These calculations are compared to the range of the sum of nitrite and nitrate measured in the analyzed jet water samples.

only at higher values of α and therefore the loss from the gas phase and the ion concentration in the liquid phase is larger. As long as the disproportionation reaction of NO_2 in the liquid phase is the rate-determining step, the uptake is independent of the gas-phase transport. Therefore the results of the model calculations using v_t and v_p are identical unless saturation is not reached due to high solubility, as shown for $H_m = 100 \times H$. Comparing the model calculations with the experimental results in the gas and liquid phase (the range is indicated in Figures 9 and 10 by horizontal lines), it becomes obvious that the calculation using the physical Henry constant underestimate the experimental data for all possible α by at least 1 order of magnitude. Calculations using $H_m \geq 7 \times H$ and a mass accommodation coefficient $\alpha \geq 2 \times 10^{-4}$ account for the mean values of the experimental loss of NO_2 from the gas phase and for the sum of the measured liquid-phase concentration of nitrite and nitrate.

Discussion

There are several possibilities on how to interpret the high value of the Henry constant, which is needed to explain the observations:

(1) The physical Henry constant is indeed as large as H_m. This is unlikely, because the literature values of the physical Henry constant either determined experimentally or by thermodynamical calculations[21,23-27] agree within a factor of 2. Therefore a 7 times larger H_m is outside the uncertainty of the literature values of the physical Henry constant for NO_2.

(2) The liquid-phase rate constants of reactions 6 and 7 (i.e., the conversion of dissolved NO_2 to NO_3^- and NO_2^-) are much faster than that used in the model. This would increase the NO_2 uptake. This is unlikely, because the highest in the literature reported rate constant[23] of 1×10^8 M^{-1} s^{-1} for the combination of reactions 6 and 7 is only 54% faster than the value of $K_6k_7 = 6.5 \times 10^7$ M^{-1} s^{-1} used in the model. To reach agreement between the model and the experimental results, an at-least 7 times larger rate constant is needed. Furthermore, the experimental results show within the error limits (approximately a factor of 2) independence of the NO_2 uptake from oxidizing substances (TEA, $NaAsO_2$) or pH in the jet water. This indicates that the rate of liquid-phase reactions have no or only a small influence on the uptake of NO_2. Thus, the interpretation of Schwartz and White,[27] who assumed unknown reactions of unknown impurities in the liquid phase, can be excluded for our experimental conditions.

(3) The discrepancy between the calculated uptake of NO_2 using the physical solubility (H) and the measured uptake is due to properties of the surface, which are not accounted for in the calculations. Using the model results obtained with $H_m = 7H$ and $\alpha = 2 \times 10^{-4}$ the amount of NO_2 molecules taken up by the jet per unit time in excess of the physical solubility can be calculated. Assuming a cross section for each of these NO_2 molecules of 6×10^{-16} cm^2 a coverage of the jet surface of approximately 0.001 is obtained. If the reaction of these molecules on the surface is the rate-limiting process, a reaction order of 2 is expected, compared to the uptake process for physical solvation, which is first order in NO_2. The observed

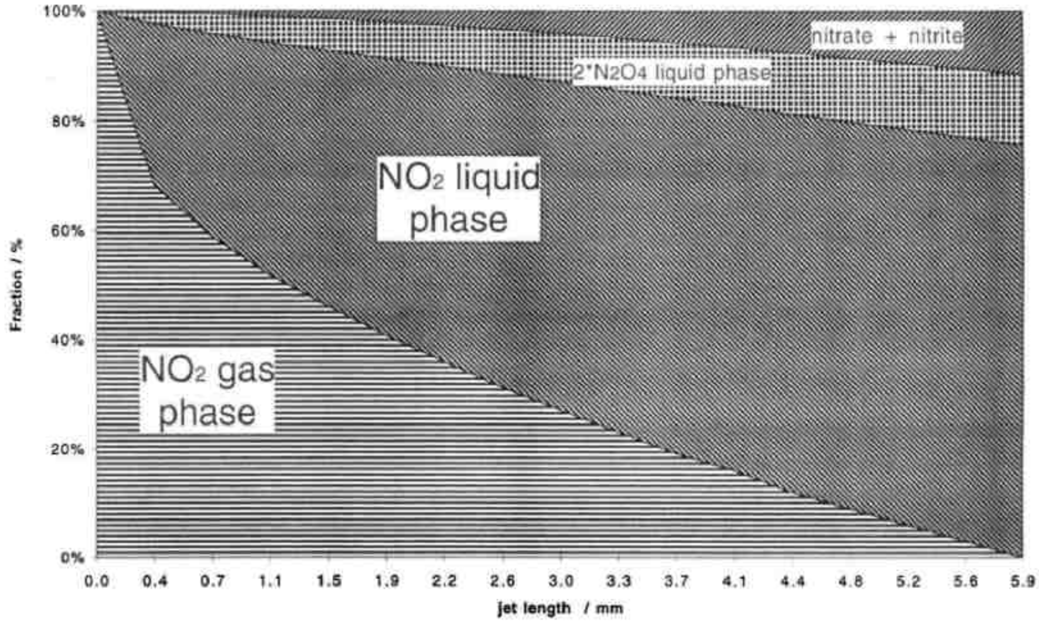

Figure 11. Calculated dependence of the distribution of the nitrogen species in gas (NO_2(g)) and liquid (NO_2(a), N_2O_4(a), and NO_2^-(a) + NO_3^-(a)) phase on exposure time normalized to the gas phase loss of NO_2(g) (=3.05×10^{13} molecules s^{-1}) within 1.4 ms (6 mm jet length). The N_2O_4(g) parameters for the model calculation: $H_m = 7H$, $[NO_2]_g = 9 \times 10^{14}$ cm^{-3}.

dependence of the loss of NO_2 from the gas phase on the NO_2 concentration indicates an apparent reaction order of 1.4 ± 0.3 (Table 3). Since equal amounts of nitrite and nitrate are measured in the liquid phase the net heterogeneous reaction 10 analogous to reaction 1 is conceivably taking place.

$$2NO_2(\text{surface}) + H_2O(\text{surface}) \rightarrow HNO_2(\text{surface}) + HNO_3(\text{surface}) \quad (10)$$

The proposed mechanism produces nitric and nitrous acid on an aqueous surface. Both are most likely taken up by the bulk liquid because of their solubility and dissociation into equal amounts of nitrite and nitrate. However, because of the lower dissociation constant and solubility of HNO_2 compared to HNO_3, HNO_2 can escape to the gas phase. An indication of this is given by the observed anticorrelation of the relative loss of HNO_2 from the gas phase with changing NO_2 concentration (cf. Table 3). The heterogeneous uptake of HNO_2 if undisturbed by saturation effects is a first order process; therefore, the relative loss of HNO_2 from the gas phase should be independent of the concentration of HNO_2 and the same in all experiments. The lower relative loss of HNO_2 from the gas phase observed in the experiments with higher NO_2 concentration can be attributed to the formation and release of HNO_2 on the aqueous surface, partially compensating the loss from the gas phase due to the liquid-phase uptake. This process must be related to the jet surface and cannot be a gas-phase or wall reaction, because of the differential measurements with the jet turned on and off. Assuming a surface reaction such as reaction 10 the observed difference of the relative loss of HNO_2 from the gas phase comparing the experiments at the NO_2 concentration of 8 × 10^{15} and 9 × 10^{14} cm^{-3} is equal to an emission of 1.8 × 10^{13} cm^{-2} s^{-1} of nitrous acid from the surface in the experiments at the NO_2 concentration of 8 × 10^{15} cm^{-3} , which is about 35% of the lost NO_2.

Due to the small Henry constant of NO_2 the equilibrium between gas and the surface layer of the liquid phase is reached within very short exposure times ($\ll$0.7 ms, $\alpha > 10^{-4}$). If no surface reaction is considered, gas-phase loss of NO_2 after that time depends on the bulk liquid-phase reaction of N_2O_4 to nitrate and nitrite and diffusion in the liquid of the jet. Figure 11 shows the dependence of the calculated distribution of the nitrogen species in gas and liquid phase on exposure time or jet length. Establishing the equilibrium between gas and liquid phase within the first 0.4 mm amounts to approximately 30% of the gas phase loss of NO_2 within 1.4 ms (6 mm jet length). The net gas-phase loss between the exposure times of 0.7 ms (3 mm jet length) and 1.4 ms (6 mm jet length) amounts to approximately 27% of the gas-phase loss of NO_2 within 1.4 ms (6 mm jet length). The gas-phase loss of NO_2 is nearly linear, dependent on the square root of the exposure time due to diffusion in the liquid as the gas-phase loss-limiting process. Taking the surface reaction 10 as the gas-phase loss-limiting process into account the gas-phase loss of NO_2 loss is expected to be proportional to the exposure time. No conclusive distinction concerning the exposure time dependence of the NO_2 gas-phase loss can be based on the measurements presented in this paper.

Other authors[31-33] also reported a significant higher uptake of NO_2 in water which is inconsistent with the physical Henry constant. The most recent investigation by Ponche et al.[31] uses a monodisperse droplet technique at a NO_2 gas phase concentration of 6.1 × 10^{15} cm^{-3} and a pressure of 50 Torr. In their analysis they show that the measured NO_2 uptake is significantly larger than calculated by their gas/liquid phase model (see eq 28 in Ponche et al.[31]) with uptake coefficients $\beta_{exp} = (1.5 \pm 0.8) \times 10^{-3}$ independent of the contact time (3.6−15 ms). Using the same equation we obtain $\beta_{exp} = (1.2 \pm 0.4) \times 10^{-3}$ (NO_2 gas-phase concentration: 8 × 10^{15} cm^{-3}) and $\beta_{exp} = (2.4 \pm 1.4) \times 10^{-4}$ (NO_2 gas-phase concentration: 9 × 10^{14} cm^{-3}) at an interaction time of 0.7 ms. As stated by Ponche et al.[31] these values have to be considered carefully, because of unknown chemical reactions. The large change of the uptake coefficient calculated according to Ponche et al.[31] with different gas-phase concentrations of NO_2 may be an indication of such reactions. Our experimental results support a fast reaction on or very close to the aqueous surface. Further work is in progress to quantify this surface enhanced uptake of NO_2 and of other nitrogen oxides.

Acknowledgment. The authors thank D. H. Ehhalt for many helpful discussions. The research is supported by the Deutsche Forschungsgemeinschaft (Wa789/1-1 and1-2).

References and Notes

(1) Platt, U.; Perner, D.; Harris, G. W.; Winer, A. M.; Pitts Jr., J. N. *Nature* **1980**, *285*, 312.
(2) Harris, G. W.; Carter, W. P. L.; Winer, A. M.; Pitts jr., J. N.; Platt, U.; Perner, D. *Environ. Sci. Technol.* **1982**, *16*, 414.
(3) Lammel, G.; Perner, D.; Warneck, P. *Physico-Chemical Behaviour of Atmospheric Pollutants*; Restelli, G., Angeletti, G., Eds.; Kluwer Academic Publishers: Dordrecht, 1989; p 469.
(4) Notholt, J.; Hjorth, J.; Raes, F. *Atmos. Environ.* **1992**, *26A*, 211.
(5) Platt, U. *Chemistry of Multiphase Atmospheric Systems*; Jaeschke, W., Ed.; Springer-Verlag: Berlin, 1986; p 299.
(6) Jenkin, M. E.; Cox, R. A.; Williams, D. J. *Atmos. Environ.* **1988**, *22*, 487.
(7) Kaiser, E. W.; Wu, C. H. *J. Phys. Chem.* **1977**, *81*, 1701.
(8) England, C.; Corcoran, W. H. *Ind. Eng. Chem. Fundam.* **1974**, *13*, 373.
(9) Sakamaki, F.; Hatakeyama, S.; Akimoto, H. *Int. J. Chem. Kinet.* **1983**, *15*, 1013.
(10) Pitts Jr., J. N.; Sanhueza, E.; Atkinson, R., Carter, W. P. L.; Winer, A. M.; Harris, G. W.; Plum, C. N. *Int. J. Chem. Kinet.* **1984**, *16*, 919.
(11) Svensson, R.; Ljungstrim, E.; Lindqvist, O. *Atmos. Environ.* **1987**, 21, 1529.
(12) Schwartz, S. E. *Chemistry of Multiphase Atmospheric Systems*; Jaeschke, W., Ed.; Springer-Verlag: Berlin, 1986; p 415.
(13) Kirchner, W.; Welter, F.; Bongartz, A.; Kames, J.; Schweighoefer, S.; Schurath, U. *J. Atmos. Chem.* **1990**, *10*, 427.
(14) Worsnop, D. R.; Zahniser, M. S.; Kolb, C. E.; Gardner, J. A.; Watson, L. R.; Van Doren, J. M.; Jayne, J. T.; Davidovits, P. *J. Phys. Chem.* **1989**, *93*, 1159.
(15) Schneider, W.; Moorgat, G. K.; Tyndall, G. S.; Burrows, J. P. *J. Photochem. Photobiol. A: Chem.* **1987**, *40*, 195.
(16) Bongartz, A.; Kames, J.; Schurath, U.; George, Ch.; Mirabel, Ph.; Ponche, J. L. *J. Atmos. Chem.* **1994**, *18*, 149.
(17) Saltzman, B. E. *Anal. Chem.* **1954**, *26*, 1949.
(18) Gold, A. *Anal. Chem.* **1977**, *49*, 1448.
(19) Merryman, E. L., C. W. Spicer, A. Levy, *Environ. Sci. Technol.* **1973**, *7*, 1056.
(20) Stull, D. R.; Prophet, A. *Natl. Stand. Ref. Data*; Nat. Bur. of Stand.: Washington, DC, 1971.
(21) Schwartz, S. E.; White, W. H. *Adv. Environ. Sci. Eng.* **1981**, *4*, 1.
(22) Van Doren, J. M.; Watson, L. R.; Davidovits, P.; Worsnop, D. R.; Zahniser, M. S.; Kolb, C. E. *J. Phys. Chem.* **1990**, *93*, 3265.
(23) Lee, Y. N.; Schwartz, S. E. *J. Phys. Chem.* **1981**, *85*, 840.
(24) Chameides, W. L. *J. Geophys. Res.* **1984**, *89*, 4739.
(25) Komiyama, H.; Inoue, H. *Chem. Eng. Sci.* **1980**, *35*, 154.
(26) Epstein, I. R.; Kustin, K.; Warsaw, L. J. *J. Am. Chem. Soc.* **1980**, *102*, 3751.
(27) Schwartz, S. E.; White, W. H. *Advances in Environmental Science and Technology*; Schwartz, S. E., Ed.; John Wiley & Sons: New York, 1983; p 1.
(28) Warneck, P. *Chemistry of the Natural Atmosphere*; Dmowska, R., Holton, J. R., Eds.; Academic Press: San Diego, 1988; Chapter 8.
(29) Bongartz, A.; Kames, J.; Schurath, U.; George, Ch.; Mirabel, Ph.; Ponche, J. L. *J. Atmos. Chem.* **1994**, *18*, 149.
(30) Lee, J. H.; Tang, I. N. *Atmos. Environ.* **1988**, *22*, 1147.
(31) Ponche, J. L.; George, C.; Mirabel, P. *J. Atmos. Chem.* **1993**, *16*, 1.
(32) Borok, M. T. *J. Appl. Chem. USSR* **1960**, *33*, 1761.
(33) Beilke, S. *USAEC Symp. Ser.* **1970**, *22*, 261.

JP942719X
