# Supplementary material for: Yttrium Doping of Perovskite Oxide La2Ti2O7 Nanosheets for Enhanced Proton Conduction and Gas Sensing Under HighHumidity Levels
Source: Sensors (Basel). 2025 Feb 2;25(3):901. doi: 10.3390/s25030901 (PMC11819912; doi:10.3390/s25030901)
Supplement: Supplementary file 1 [file sensors-25-00901-s001.zip › SI.pdf]

sensors

MDPI

Supplementary Materials

*Communication*

# Yttrium Doping of Perovskite Oxide $La_2Ti_2O_7$ Nanosheets for Enhanced Proton Conduction and Gas Sensing Under High Humidity Levels

**Jian Wang, Caicai Sun, Jusheng Bao, Zhiwei Yang, Jian Zhang* and Xiao Huang***

Institute of Advanced Materials (IAM), School of Flexible Electronics (SoFE), Nanjing Tech University (NanjingTech), 30 South Puzhu Road, Nanjing 211816, China; 202262122006@njtech.edu.cn (J.W.); suncaicai202261122082@njtech.edu.cn (C.S.); baojsh@ahstu.edu.cn (J.B.); 202362221243@njtech.edu.cn (Z.Y.)

* Correspondence: iamjzhang@njtech.edu.cn (J.Z.); iamxhuang@njtech.edu.cn (X.H.)

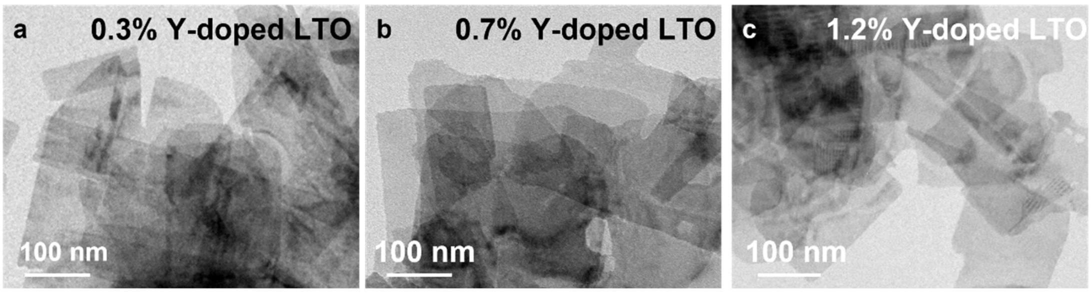


**Figure S1.** TEM images of (**a**) 0.3%, (**b**) 0.7% and (**c**) 1.2% Y-doped LTO nanosheets.

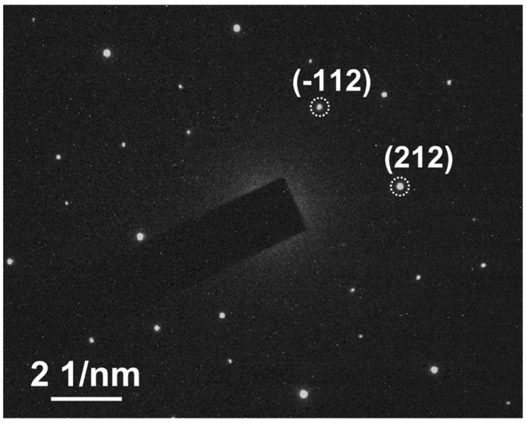


**Figure S2.** SAED pattern of 0.7% Y doped LTO nanosheet.

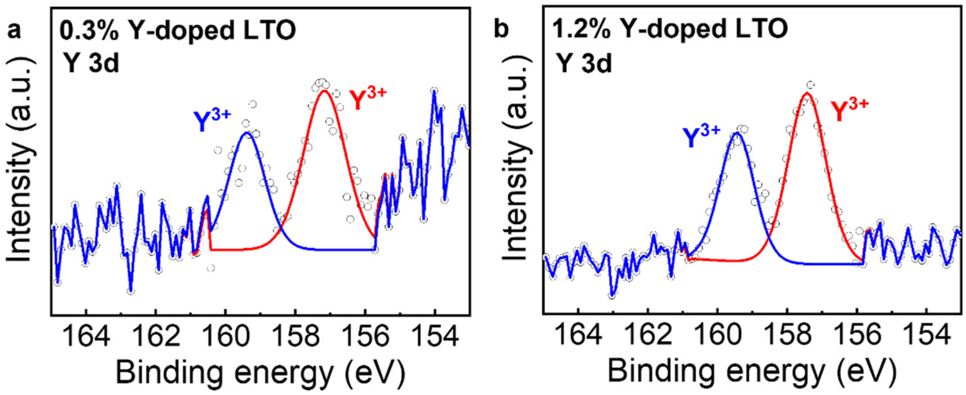

**Figure S3.** High-resolution Y 3d spectra of (**a**) 0.3% and (**b**) 1.2% Y-doped LTO nanosheets.

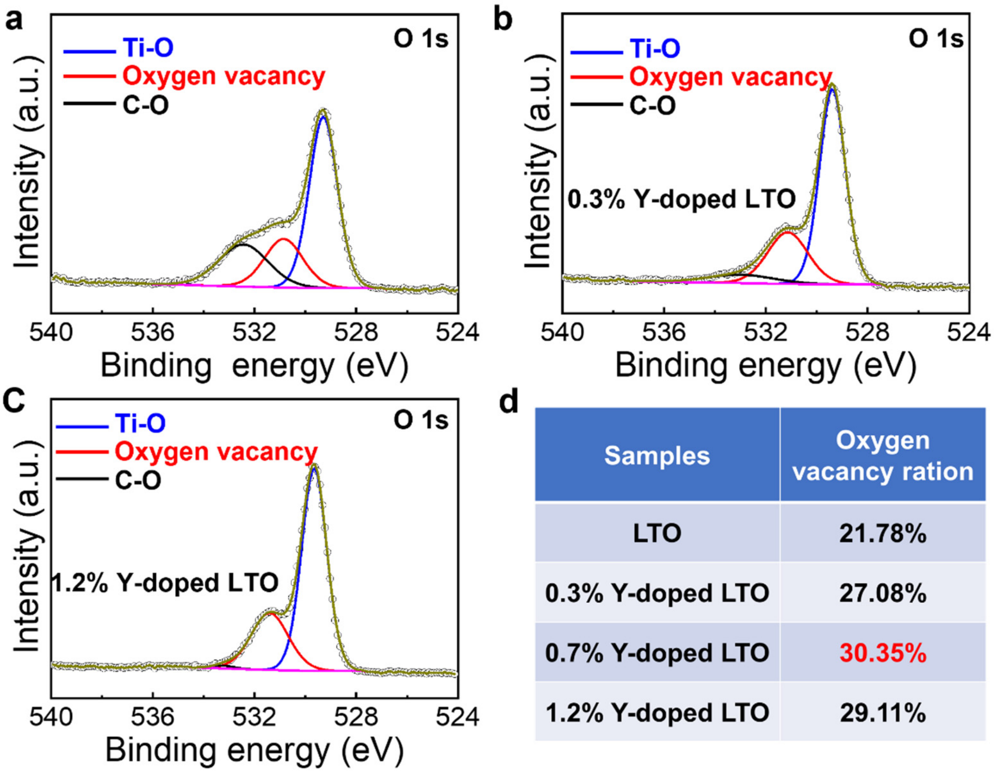


| Samples | Oxygen vacancy ration |
|---|---|
| LTO | 21.78% |
| 0.3% Y-doped LTO | 27.08% |
| 0.7% Y-doped LTO | 30.35% |
| 1.2% Y-doped LTO | 29.11% |

**Figure S4.** High-resolution O 1s spectra of (**a**) pure LTO, (**b**) 0.3% and (**c**) 1.2% Y-doped LTO nanosheets. (**d**) Oxygen vacancy ratio table of YLTO nanosheets.

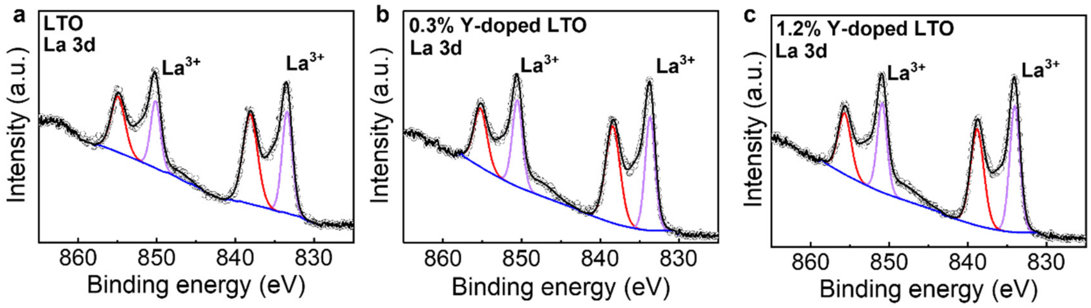


**Figure S5.** High-resolution La 3d spectra of (**a**) pure LTO, (**b**) 0.3% and (**c**) 1.2% Y-doped LTO nanosheets.

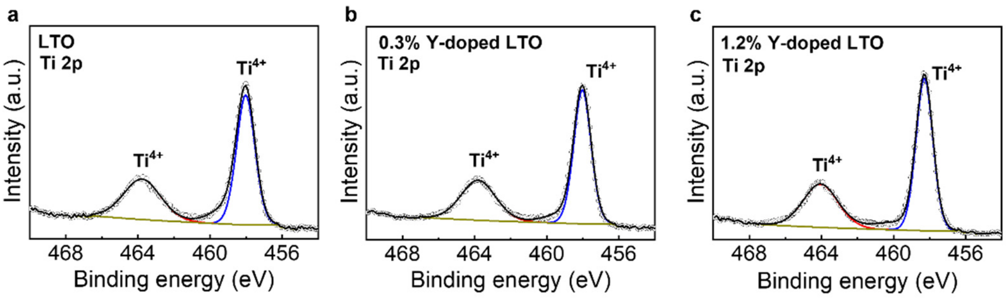


**Figure S6.** High-resolution Ti 2p spectra of (**a**) pure LTO, (**b**) 0.3% and (**c**) 1.2% Y-doped LTO nanosheets.

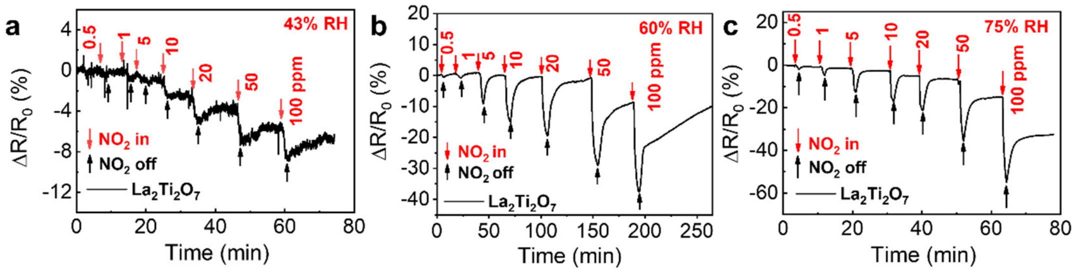


**Figure S7.** Sensing responses of LTO nanosheets to $NO_2$ under (**a**) 43% RH, (**b**) 60% RH and (**c**) 75% RH.

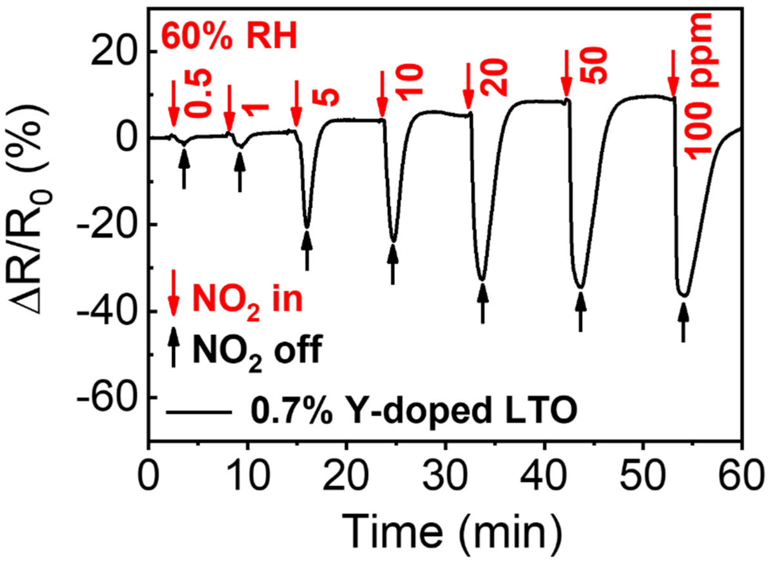


**Figure S8.** Sensing responses of 0.7% Y-doped LTO nanosheets to $NO_2$ under 60% RH.

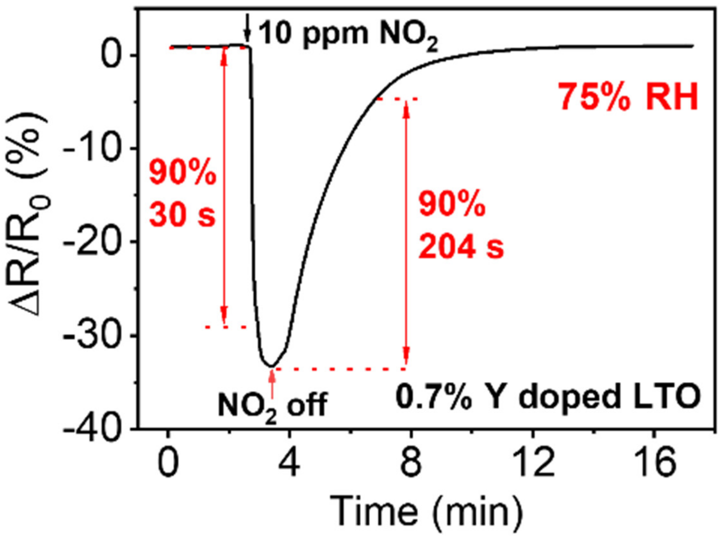


**Figure S9.** Dynamic response-recovery curves of 0.7% Y doped LTO to 10 ppm $NO_2$ at 75% RH.

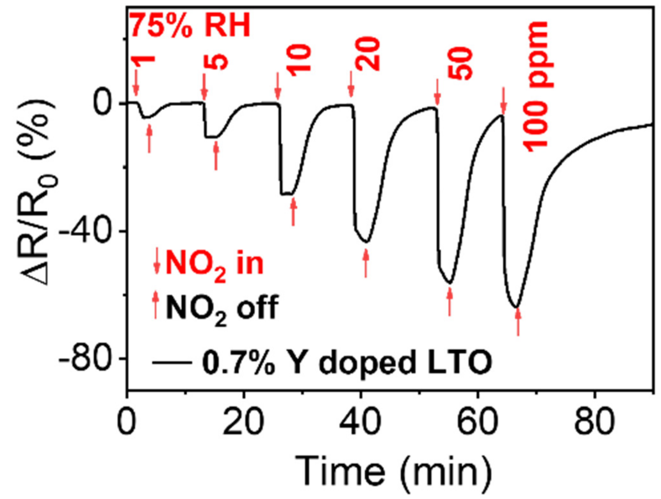


**Figure S10.** Dynamic response-recovery curves of 0.7% Y doped LTO after one week standing.

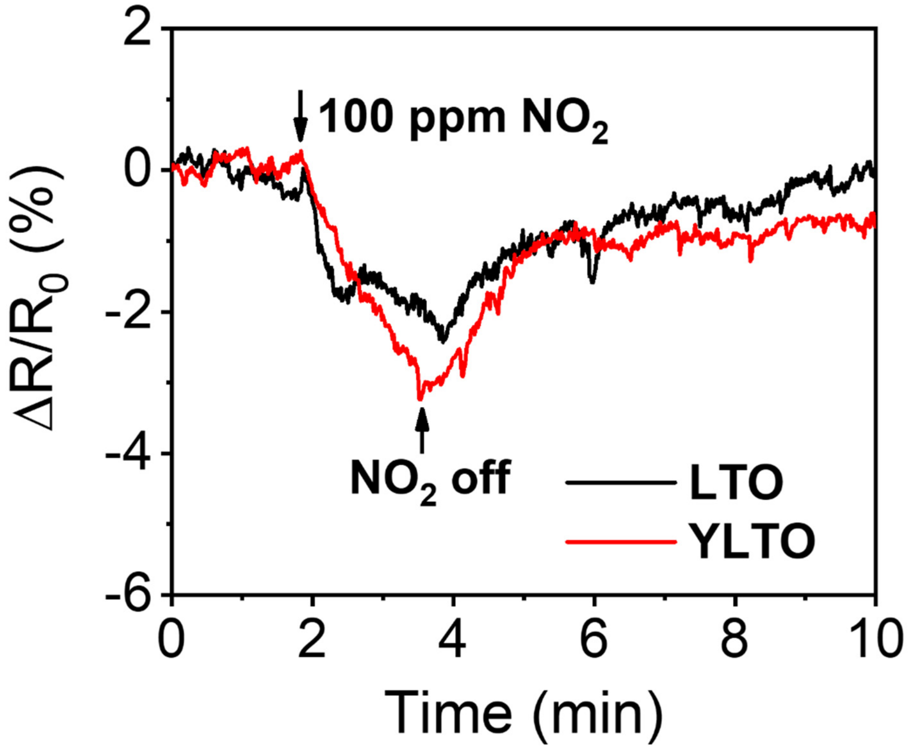


**Figure S11.** Sensing responses of LTO and 0.7% Y-doped LTO nanosheets to 100 ppm $NO_2$ under 23 % RH.

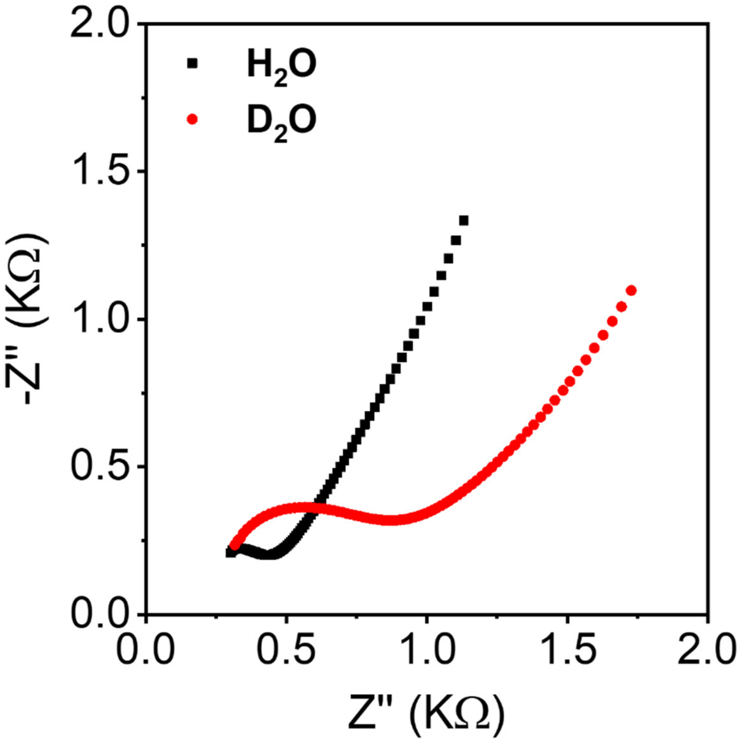


**Figure S12.** Nyquist plot of LTO nanosheet in $H_2O$ and $D_2O$ environments under 100% RH.

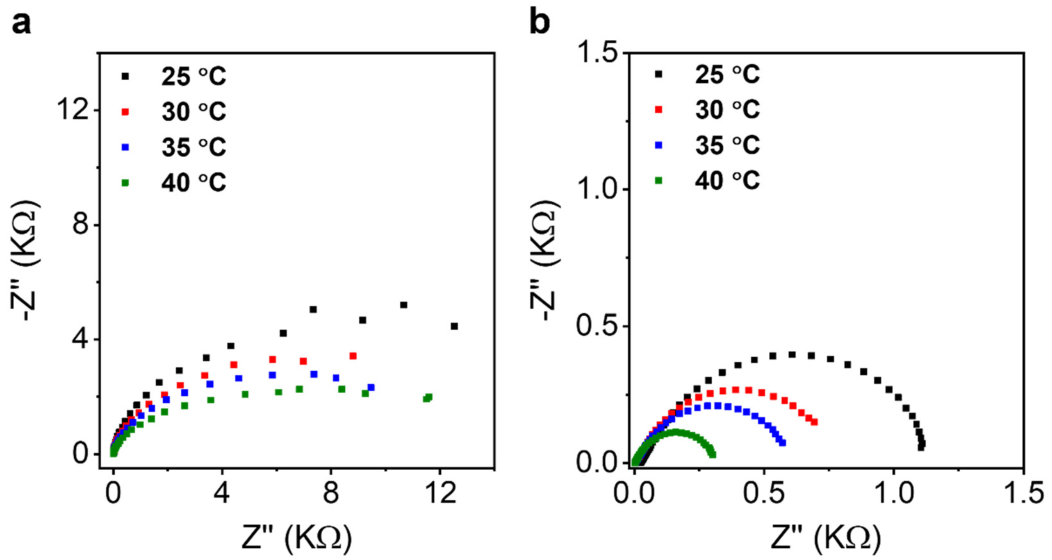


**Figure S13.** Nyquist plots of 0.7% Y-doped LTO nanosheets under (**a**) 43% RH and (**b**) 75% RH.

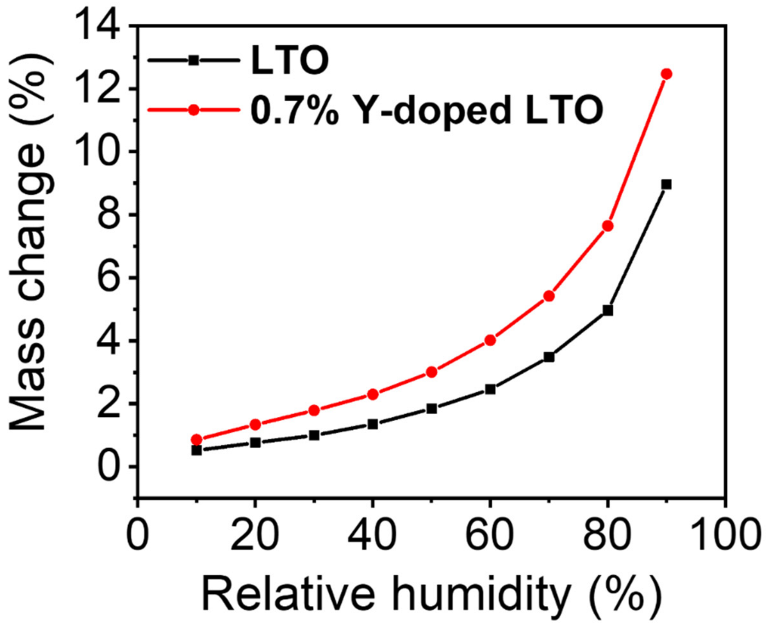


**Figure S14.** Water adsorption curves of LTO and 0.7% Y-doped LTO nanosheets.

**Table S1.** Comparison of the sensing performance of the recently reported $NO_2$ sensors based single-component metal oxides.

| Material | Humidity Tolerance | Response | Response/Recovery Time | Detection Limit (ppm) | Ref. |
|---|---|---|---|---|---|
| YLTO | 43% -75% RH | 36% @ 10 ppm | 30 s/204 s @ 10 ppm | 0.5 | This work |
| CuO | 30% - 70% RH | 33.3% @ 20 ppm | 8 s/176 s @ 10 ppm | 1 | [1] |
| $Ce_2Sn_2O_7$ | 70% - 85% RH | 53% @ 50 ppm | 4 s/52 s @ 10 ppm | 0.5 | [2] |
| $WO_3$ | Decreased response under high RH | 45% @ 50 ppm, 250 °C | 75 s/33 s @ 50 ppm | 1 | [3] |
| $WO_3$ | Unstable response under high RH | 20% @ 3 ppm | 50 s/300 s @ 3 ppm | 3 | [4] |
| $In_2O_3$ | ~ 40% RH | 35% @ 5 ppm, 400 °C | 5 min/30 min @ 5 ppm | 0.2 | [5] |
| ZnO | Decreased response under high RH | 43.7% @ 10 ppm, 300 °C | 80 s/65 s @ 10 ppm | 2 | [6] |
| ZnO | / | 36.4% @ 100 ppm, 200 °C | 25 s/320 s @ 100 ppm | 4 | [7] |

**References:**


1. Chen, X.; Zhao, S.; Zhou, P.; Cui, B.; Liu, W.; Wei, D.; Shen, Y. Room-temperature $NO_2$ sensing properties and mechanism of CuO nanorods with Au functionalization. *Sens. Actuators, B* **2021**, *328*, 129070, doi:10.1016/j.snb.2020.129070.
2. Ganesan, M.; Jayaraman, V.; Selvaraj, P.; Mani, K.M.; Kim, D.-H. Pyrochlore cerium stannate ($Ce_2Sn_2O_7$) for highly sensitive $NO_2$ gas sensing at room temperature. *Appl. Surf. Sci.* **2023**, *624*, 157135, doi:10.1016/j.apsusc.2023.157135.
3. Dwivedi, C.; Srivastava, S.; Singh, P. Highly selective dual gas (NO & $NO_2$) sensing depended on the operating temperature of $WO_3$ thin films sputtered at room temperature. *Curr. Appl. Phy.* **2025**, *69*, 70-80, doi:10.1016/j.cap.2024.11.009.
4. Ghosh, S.; Ilango, M.S.; Prajapati, C.S.; Bhat, N. Reduction of Humidity Effect in $WO_3$ Thin Film-Based $NO_2$

Sensor Using Physiochemical Optimization. *Cryst. Res. Technol.* **2020**, *56*, 2000155, doi:10.1002/crat.202000155.
5. D'Olimpio, G.; Boukhvalov, D.W.; Galstyan, V.; Occhiuzzi, J.; Vorochta, M.; Amati, M.; Milosz, Z.; Gregoratti, L.; Istrate, M.C.; Kuo, C.-N.; et al. Unlocking superior $NO_2$ sensitivity and selectivity: the role of sulfur abstraction in indium sulfide (InS) nanosheet-based sensors. *J. Mater. Chem. A* **2024**, *12*, 10329-10340, doi:10.1039/D4TA01287A.
6. Bang, J.H.; Kwon, Y.J.; Lee, J.H.; Mirzaei, A.; Lee, H.Y.; Choi, H.; Kim, S.S.; Jeong, Y.K.; Kim, H.W. Proton-beam engineered surface-point defects for highly sensitive and reliable $NO_2$ sensing under humid environments. *J. Hazard. Mater.* **2021**, *416*, 125841, doi:10.1016/j.jhazmat.2021.125841.
7. Ambi, R.R.; Mulla, M.G.; Pittala, R.K. $NO_2$ sensing properties of chemically deposited vertically aligned flowerlike hexagonal ZnO nanorods. *Sens. Actuators, A* **2024**, *376*, 115621, doi:10.1016/j.sna.2024.115621.
